# Quantifying the Impact and Extent of Undocumented Biomedical Synonymy

**DOI:** 10.1371/journal.pcbi.1003799

**Published:** 2014-09-25

**Authors:** David R. Blair, Kanix Wang, Svetlozar Nestorov, James A. Evans, Andrey Rzhetsky

**Affiliations:** 1 Institute for Genomics and Systems Biology, University of Chicago, Chicago, Illinois, United States of America; 2 Committee on Genetics, Genomics, and Systems Biology, University of Chicago, Chicago, Illinois, United States of America; 3 Computation Institute, University of Chicago, Chicago, Illinois, United States of America; 4 Department of Sociology, University of Chicago, Chicago, Illinois, United States of America; 5 Departments of Medicine and Human Genetics, University of Chicago, Chicago, Illinois, United States of America; University of Colorado School of Medicine, United States of America

## Abstract

Synonymous relationships among biomedical terms are extensively annotated within specialized terminologies, implying that synonymy is important for practical computational applications within this field. It remains unclear, however, whether text mining actually benefits from documented synonymy and whether existing biomedical thesauri provide adequate coverage of these linguistic relationships. In this study, we examine the impact and extent of undocumented synonymy within a very large compendium of biomedical thesauri. First, we demonstrate that missing synonymy has a significant negative impact on named entity normalization, an important problem within the field of biomedical text mining. To estimate the amount synonymy currently missing from thesauri, we develop a probabilistic model for the construction of synonym terminologies that is capable of handling a wide range of potential biases, and we evaluate its performance using the broader domain of near-synonymy among general English words. Our model predicts that over 90% of these relationships are currently undocumented, a result that we support experimentally through “crowd-sourcing.” Finally, we apply our model to biomedical terminologies and predict that they are missing the vast majority (>90%) of the synonymous relationships they intend to document. Overall, our results expose the dramatic incompleteness of current biomedical thesauri and suggest the need for “next-generation,” high-coverage lexical terminologies.

## Author Summary

Automated systems that extract and integrate information from the research literature have become common in biomedicine. As the same meaning can be expressed in many distinct but synonymous ways, access to comprehensive thesauri may enable such systems to maximize their performance. Here, we establish the importance of synonymy for a specific text-mining task (named-entity normalization), and we suggest that current thesauri may be woefully inadequate in their documentation of this linguistic phenomenon. To test this claim, we develop a model for estimating the amount of missing synonymy. We apply our model to both biomedical terminologies and general-English thesauri, predicting massive amounts of missing synonymy for both lexicons. Furthermore, we verify some of our predictions for the latter domain through “crowd-sourcing.” Overall, our work highlights the dramatic incompleteness of current biomedical thesauri, and to mitigate this issue, we propose the creation of “living” terminologies, which would automatically harvest undocumented synonymy and help smart machines enrich biomedicine.

## Introduction

Most words and phrases in English possess synonyms—expressions that share close or identical meaning within a restricted cultural context [1]. For example, *trait* and *phenotype* are often used interchangeably within the genetics literature [2], but not within general English, while the converse is true of adjectives *blue* and *sad*. Synonymous relationships play a variety of roles within natural language. For example, an entire sentence may be rendered incomprehensible upon encountering a rare, previously unseen word. Knowing a single synonym for such a word, however, even if distantly related, enables at least partial comprehension of the sentence's meaning. Thus, synonyms can be seen as a simple, concise way of encoding the semantics of individual words [3], which makes them useful for artificial intelligence applications. Much like their human counterparts, computer programs that parse natural language must rely on a finite set of “known” synonymous relationships, so deficiencies in their thesauri could have a profound impact on their ability to process human communication. While intuitively important, synonymy has enjoyed relatively little attention from the text mining and natural language processing communities. Synonymy is extensively documented within many large computational lexicons [4], [5], but it is not immediately obvious whether inclusion of synonyms sufficiently improves natural language processing results to justify the computational overhead.

Within the field of biomedical text mining, several studies have demonstrated that manually curated terminologies, and thus thesauri, are dispensable for particular tasks [6], specifically named-entity recognition (NER) [7], [8]. This is not terribly surprising, as the goal of NER is to simply find mentions of diseases, drugs, genes, etc. in free text, but not to determine which disease, drug, or gene to which the mention refers. In our personal experience, however, identification of the precise object being mentioned, or named-entity normalization (NEN), crucially depends on thorough documentation of synonymy, an observation supported, for example, by its extensive use within the BioCreative gene name normalization challenge [9]. Named-entity normalization is absolutely critical for integrating text-mined knowledge with other biomedical datasets. For example, tasks that utilize clinical records to uncover off-label drug usages [10] or to find novel genetic associations [11], [12] inherently depend on the identification of specific named entities. Nevertheless, relatively little effort has been devoted to the general problem of biomedical named entity normalization [13], [14]. Furthermore, although gene name normalization has been extensively studied within the literature [9], [15]–[20], the systematic evaluation of other specific biomedical NEN tasks remains in its infancy [21]–[23]. Finally, given its widespread use within NEN algorithms, it is surprising that the impact of synonymy on this task and text mining in general remains under examined.

In this study, we quantify the importance of synonymy for named-entity normalization within the field of biomedicine. More specifically, we evaluated the performance of several general-purpose NEN algorithms, both with and without synonymy, on two gold-standard disease name normalization corpora. We found that every algorithm, even one that explicitly attempts to learn synonymy during training [21], is detrimentally affected by missing synonymous relationships. To quantify the extent of the missing synonymy problem within biomedicine, we developed a statistical model capable of inferring the number of synonymous relationships missing from a set of manually annotated thesauri while simultaneously accounting for a wide range of potential biases. To investigate the validity of our approach, we applied the model to the much broader domain of general-English near-synonymy, and we demonstrated that the vast majority of these relationships are currently undocumented. To verify this result experimentally, we developed a “crowd-sourcing” pipeline to uncover novel examples of high-quality near-synonyms. Finally, we applied our statistical model to two biomedical sub-domains (*Diseases and Syndromes* and *Pharmacological Substances*) and estimated that the vast majority of their synonymous relationships (>90%) are likely undocumented. Overall, this work quantitatively measures the impact and extent of synonymy within biomedicine and highlights the need for more sophisticated approaches towards detecting, cataloging, and utilizing synonymous relationships.

## Results

### Documented Synonymy Significantly Improves Biomedical Named-Entity Normalization

Although synonymy is not necessarily important for every biomedical text-mining task [6]–[8], we believe that it is absolutely critical for some, especially named entity normalization. It does not appear, however, that biomedical thesauri have been constructed according to any systematic standards or consistency, suggesting that a considerable fraction of documented synonymy may be of low utility. For example, it is possible that only a few common synonyms are ever used in biomedical text, and while these select few may be very useful, the remaining relationships are either irrelevant (never used in natural language) or redundant (such as an obvious spelling variant). To evaluate the utility of documented synonymy, we first examined its impact on the normalization of disease names. We constructed a large terminology of *Diseases and Syndromes* using the UMLS Metathesaurus [5] (see Materials and Methods), asking whether removing synonyms from this terminology significantly impacted the performance of four of normalization algorithms [21], [24] (see Table 1 and Supporting Information Text S1 for details). We evaluated this procedure using two gold standard corpora generated independently of our study: the NCBI and Arizona Disease Corpora, abbreviated NCBI and AZDC, respectively [25], [26]. To ensure that our analyses were not biased by a few commonly occurring diseases, we restricted our analysis to unique mentions only.

**Table 1:** The effects of missing synonymy on disease name normalization.

Algorithm	Corpus	% Recall Due to Syn.	% Recalled Concepts with Red. Syn.	Red. Syn. Fraction
*Boolean Search*	AZDC	29%	46%	12.2%
				(8.4%, 16.5%)
*Boolean Search*	NCBI	30%	37%	7.0%
				(4.7%, 9.5%)
*MetaMap*	AZDC	35%	54%	21.1%
				(15.6%, 27.0%)
*MetaMap*	NCBI	33%	46%	12.2%
				(8.7%, 16.2%)
*Cosine Similarity*	AZDC	37%	20%	5.6%
				(3.6%, 7.8%)
*Cosine Similarity*	NCBI	38%	37%	12.9%
				(9.5%, 16.6%)
*pLTR*	AZDC	31% (Avg.)	≈0%	≈0%
				(0%, 0%)
*pLTR*	NCBI	23% (Avg.)	39%	3.2%
				(2.1%, 4.4%)

This table indicates the total fraction of recall attributable to synonymy (third column) for four different normalization algorithms (first column) and two different gold-standard corpora (second column). The fourth column indicates the fraction of concepts in the third column whose recall depended on redundant synonyms, and the fifth column provides the fraction of the total number of synonyms predicted to be redundant for the recalled concepts (mean plus 95% confidence interval).

Not surprisingly, we observed that synonymy was broadly useful for disease name normalization, accounting for 20–40% of task recall (see Table 1) while having only a slight, positive impact on precision (see Figure S1). Even algorithms that explicitly account for synonymy during use, like MetaMap [24] and pairwise-Learning-to-Rank (pLTR) [21], benefited substantially from thorough synonym annotation. To our knowledge, gold-standard corpora for general biomedical terminologies do not exist, so it is difficult to extend these results to other domains within biomedicine. To further evaluate the importance of synonymy for named-entity normalization, we constructed a terminology for *Pharmacological Substances* (see Materials and Methods), and we repeated our normalization experiment on a random sample of 35,000 unique noun phrases isolated from MEDLINE (see Materials and Methods). We used MetaMap (due to high precision on the previous task) to map noun phrases to this terminology with and without synonymy. Once again, we observed that synonymy was responsible for retrieving a significant fraction of the identified concepts (approximately 30%, see Figure S2). Although the lack of a gold standard renders true assessment of the increase in recall impossible, we note that precision remained constant (or even increased, see Figure S1) in our previous experiment as synonyms were added back to the *Diseases and Syndromes* terminology. Assuming that this trend applies to *Pharmacological Substances*, the increase in recall due to synonymy should have a strictly positive impact on normalization performance, suggesting that our results obtained using gold-standard corpora apply to other and possibly all sublanguages of biomedicine.

Although synonymy as a whole appears to be useful for biomedical named-entity normalization, it is still possible that a large fraction of synonymous relationships are redundant and/or unimportant. If this were true, current terminologies could be made much leaner by removing useless and/or redundant synonyms. It is very difficult to broadly assess the importance of synonyms, as the measurement is highly task and context dependent. Therefore, we will address this issue more extensively in the Discussion. Synonym redundancy, on the other hand, can be directly estimated from the normalization results described in the previous paragraph, at least with respect to the corpora and algorithms considered here. We computed the extent of redundancy in the biomedical terminologies by removing random fractions of synonyms and subsequently re-computing concept recall. If each synonym encodes unique information, recall for a particular corpus and algorithm should increase linearly with the fraction of included synonymy. Alternatively, if redundant synonyms are present, then the recall rate should increase sub-linearly (see Supporting Information Text S1 for details). We did in fact observe sub-linear increases in concept recall (see Figures S1 and S2), indicative of redundancy in documented synonymy. We directly estimated the fraction of redundant synonymous relationships with respect to each corpus and algorithm, as effective redundancy depends on the method employed (some methods are able to internally generate more extensive spelling and lexical variation than others [13], [21], see Supporting Information Text S1 for details). We found that most concepts whose recall depended on synonymy were not paired with redundant synonyms (see Table 1, Column 4). Furthermore, only a minor fraction of the synonymous relationships associated with these concepts were predicted to be redundant (see Table 1, Column 5 and Supporting Information Text S1 for more details). Overall, these results suggest that synonymy is useful for biomedical named-entity normalization and that current terminologies are not saturated with redundant information, highlighting the potential for additional high-value synonyms to further improve performance.

### A Probabilistic Model for Inferring the Extent of Undocumented Synonymy

Based on the analysis we described in the preceding section, we are convinced that synonymy is important for named-entity normalization. Furthermore, assuming that our conclusions are correct, there is much room to improve current biomedical thesauri. The analysis just described, however, does not prove that high-utility synonyms are missing from the terminologies, nor does it indicate how many terms are missing. With respect to the former question, we manually curated the normalization errors made by MetaMap in our prior analysis of disease names, and, consistent with previous observations [21], found that a substantial fraction of its errors (14% for the AZDC corpora and 34% for NCBI) could be traced back to missing synonyms. Moreover, approximately half of these same normalization errors were committed by all of the other algorithms (examples are listed in Table S1). These rates were generally comparable to the magnitude of errors caused by ambiguous terms (26% and 38% for AZDC and NCBI respectively), and they likely represent a lower bound on the true error rates due to missing synonymy. This is because such errors require annotators to recognize a synonym not contained within a large, complex terminology, a task that is likely difficult even for domain experts.

If undocumented synonyms of high utility exist, the question arises, “How many?” This is difficult to answer, as current biomedical terminologies provide no indication of synonym quality. Our analysis from the previous section suggests that a non-negligible fraction of documented synonyms are useful and thus, one approach to quantifying the extent of the problem is to estimate the total number of synonyms missing from terminologies, a considerable fraction of which should be useful. To estimate the extent of undocumented synonymy, we examined the overlap between several distinct biomedical terminologies, which we isolated from the UMLS Metathesaurus [5]. Assuming that the terminologies were constructed approximately independently from one another (detailed assumptions and justifications provided below), the overlap in concepts and synonyms across thesauri should be informative of the missing portion.

**Figure 1:**
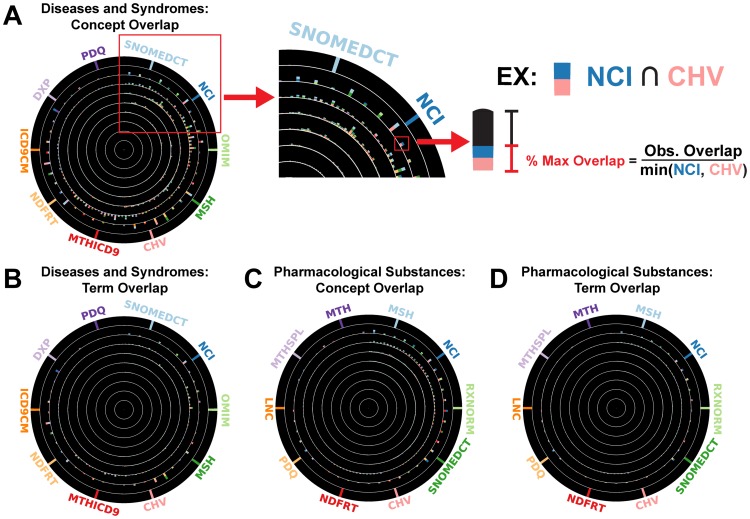
Very little information is shared across multiple biomedical terminologies. (A) The panel on the left illustrates the overlap among the concepts annotated by the terminologies documenting *Diseases and Syndromes*. The figure itself is composed of ten concentric rings, with the outermost ring (*k* = 1) indicating the colors assigned to each dataset. The next ring (*k* = 2) displays the overlap in concepts among all pairwise comparisons, arranged in clockwise order starting with the intersection (MSH, NCI). The extent in overlap was computed by dividing the number of co-occurring annotations by the maximum possible number given the sizes of the terminologies being intersected (percent maximum overlap). This information is displayed within the concentric ring using bi-colored bars, whose heights depict the percent maximum overlap for the terminologies indicated by the colors. The panels on the right illustrate this idea by enlarging a section of the original figure, highlighting a particular intersection (NCI, CHV), and explaining how the colored bar translates into the percent maximum overlap. The remaining concentric rings (*k* = 3…10) display the overlap extent for all higher order intersections (3-way, 4-way, etc.), with each ring containing 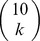 colored bars. (B) This figure illustrates the overlap among terms annotated to each concept for the same ten datasets depicted in (A). (C, D) These panels show the overlap in concepts (C) and terms (D) for the *Pharmacological Substances* terminologies. Note that only the ten largest datasets were included in each panel for the sake of clarity.

In Figure 1A, we depict the concept overlap for ten terminologies [5], [27]–[35] annotating Diseases and Syndromes. The concentric rings in the figure illustrate all of the possible *N*-way intersections among vocabularies (*N* = 2,3,..,10), with the outermost ring indicating the vocabularies themselves, the next ring depicting all possible two-way intersections, the third all three-way intersections and so on, until we reach the center of the plot, which depicts the overlap among all ten vocabularies. Colored bars within each ring indicate the identity of intersecting vocabularies (colors) and the extent of their overlapping information. Precisely, the height of the bars corresponds to the observed overlap among the terminologies, divided by their maximum possible overlap (for example, see Figure 1A, right panel). Therefore, if a colored bar extends through the full width of its concentric ring, then the smallest of the *N* intersected terminologies is perfectly nested within all of the others. Most of the intersections illustrated in Figure 1A are tiny, and this becomes more evident as the number of intersected dictionaries increases (Figure 1A, left panel). This suggests that the pool of concepts used to create these terminologies is much larger than the set currently documented, as there is little repetition in annotated information. The situation appears even more dramatic for synonyms associated with these concepts, as the overlap among annotated terms is far less (Figure 1B). Although terms technically represent a superset of synonyms (synonymy only exists whenever two or more terms are paired with the same concept), large numbers of missing terms directly imply large numbers of missing synonyms. Furthermore, the same trends are readily apparent for the set of terminologies documenting *Pharmacological Substances* [5], [27], [28], [30]–[32], [35]–[37] (Figure 1C and 1D, respectively). Overall, these results imply that biomedical thesauri are missing a vast amount of synonymy, although the true magnitude of the problem remains uncertain.

To estimate the amount of synonymy missing from these terminologies, we extended a statistical framework originally developed for estimating the number of unobserved species from samples of randomly captured animals [38]–[40]. In its simplest form, our approach assumes that each of the terminologies mentioned in Figure 1 was constructed by independently sampling concept-to-term (or synonymous) relationships from some large, unobserved population. In the parlance of the “missing species” problem, these concept-to-term relationships represent the “species,” and their occurrence within biomedical language represent the “population.” The model assumes that these species were “captured” by the annotators who constructed the biomedical terminologies, and once a concept-to-term relationship was captured once or more, it was included in the resource. Given that the total population of relationships is very large and rate of “capture” for any particular relationship is necessarily very small, this process of annotating concept-to-term relationships can be effectively modeled using a Poisson process [40]. By modeling all concept-to-term annotations in each thesaurus, we were able to compute the expected number of synonyms missing from all them (see Materials and Methods for mathematical details). Furthermore, because a concept annotated with zero concept-to-term relationships is effectively missing from the combination of terminologies, this approach enabled us to estimate the number of undocumented concepts.

In the previous description, we made a subtle but critical assumption: each thesaurus was constructed by independently sampling concept-to-term relationships. It is well known that thesauri annotate lexical relationships according to potentially unique goals and biases [3], and such practices could potentially explain a fraction of the reduced overlap observed in Figure 1. Furthermore, some concepts and synonyms may have been easier to notice and annotate than others. For example, it could have been more difficult to find synonyms for very rare concepts. As a result, one might expect these entries to be infrequently replicated across terminologies. This and other examples call into question our independent sampling assumption, as they suggest widespread correlation in the annotation rates of concept-to-term relationships. To account for a range of potential biases, we extended our statistical model by assuming that concepts and terms belong to distinct classes, which are in turn associated with unique annotation rates (see Supporting Information Text S1 for details). By design, this mixture modeling approach enabled us to capture correlation structure that exists among annotations, both within and across terminologies.

To illustrate, imagine that we have two terminologies (denoted *T*_1_ and *T*_2_), each annotating two distinct concepts (*C*_1_ and *C*_2_). Imagine that *C*_1_ is rare while *C*_2_ is common. Furthermore, assume that both terminologies have sparsely annotated synonyms for *C*_1_ but many shared synonyms for *C*_2_. Our hierarchical mixture model could account for this bias by assigning these two concepts to distinct classes, one of which was easier to annotate (the common concept class) while the other was very difficult (the rare concept class). The resulting difference in annotation rates would explain the observed differences in overlap patterns for the two concepts while simultaneously explaining annotation correlations observed across both terminologies. Now, assume that there is a third concept (*C*_3_), which is also quite common and thus shares many annotations across *T*_1_ and *T*_2_. It could also be assigned to the common concept class, and thus, the annotation patterns for these two concepts would correlate as well. In this example, we used concept frequency as the explanation for why some concepts are easier to annotate than others, but biases could be caused by a multitude of factors, such as semantic granularity or hyper/hyponymous relationships. For this reason, we found that the mixture modeling approach accounted for annotation biases much better than explicitly including word frequency or any other particular property as a confounder in the analysis. The mixture model is simply more flexible at capturing all observed variability. In the following section, we illustrate the effectiveness of our approach for capturing widespread annotation variability by applying it to general-English near-synonymy. Because this linguistic domain is widely accessible to non-experts, it enabled us to verify some of our modeling predictions experimentally.

### Undocumented Near-Synonymy within General English

To evaluate our statistical approach for inferring undocumented synonymy, we applied it to a compendium of near-synonymous relationships among general-English words. Although biomedical and general-English synonymy are not equivalent, their documentation and storage patterns are quite similar. Both are contained in key-value structures consisting of manually curated concepts (or headwords with respect to general-English synonymy) and terms (synonyms in general-English). Therefore, we hypothesize that some aspects of our statistical method's performance, including its ability to detect and account for annotation variability, should translate across domains. Additionally, general-purpose thesauri necessarily include fragments of more specialized vocabularies (e.g., genetics, molecular biology, physics, astronomy and so on), so their coverage has practical ramifications for many domains, including biomedicine. We acknowledge, however, that there are differences between general-English and biomedical synonymy. Importantly, such differences enabled us to perform a more thorough analysis of missing synonymy in general-English. First, the dataset consists only of individual words and not phrases, so it is much easier to measure the linguistic properties of various annotations (ex: word length, frequency, etc.) in order to determine whether our method captures specific biases. Second, knowledge of general-English synonymy is collectively held by millions of people and documents, allowing us to experimentally verify some of our method's predictions.

**Figure 2:**
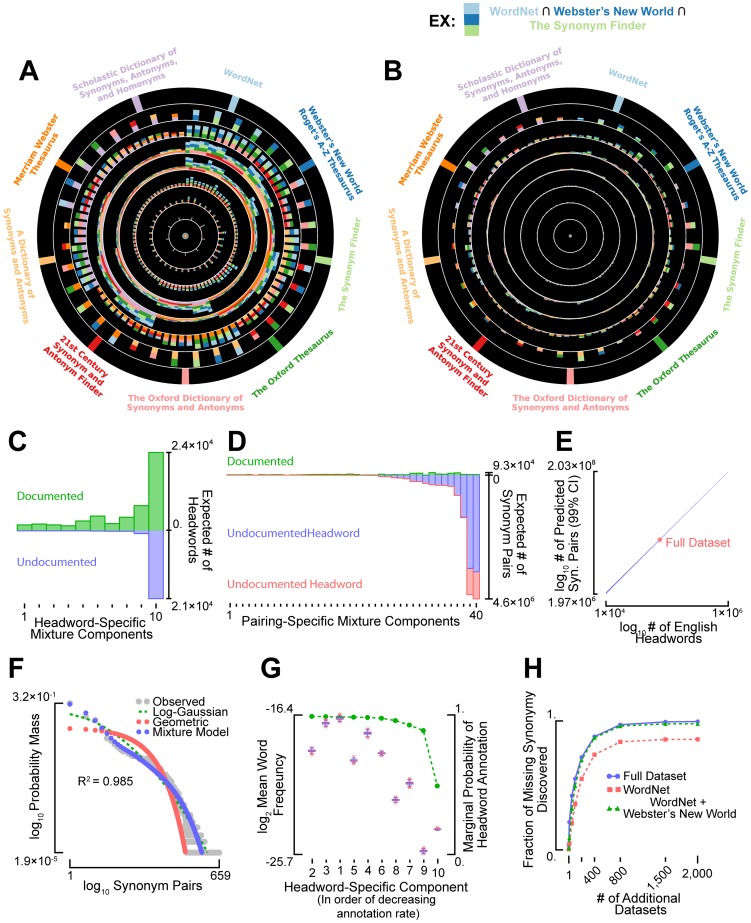
Most near-synonymous relationships among general English words are undocumented. The overlap among the (A) headwords and (B) synonymous relationships annotated within nine general-English thesauri. (C) The number of known (above x-axis) and undocumented (below x-axis) headwords belonging to each of the ten, headword-specific mixture model components (see Supporting Information Text S1). (D) The number of known (above x-axis) and undocumented (below x-axis) synonymous relationships belonging to each mixture component. The blue bars indicate undocumented relationships paired to known headwords while the red bars indicate undocumented relationships paired to latent headwords. (E) The number of synonymous relationships is shown as a function of the total number of headwords in the English language. The width of the line indicates the 99% confidence interval for the estimate (see Supporting Information Text S1). (F) The distribution over the number of synonyms annotated per headword (gray) is compared to the theoretical distribution obtained using best-fitting statistical annotation model (blue). The *R* ^2^-value indicates the fraction of variance in synonym number explained by the model. For reference, log-Gaussian and geometric models were fit to the data as well (red and green, respectively), although their quality of fit was several thousand orders of magnitude worse than the best fitting annotation model (according to marginal likelihood). (G) Box-whisker plots depicting the mean relative word frequencies (1,000 bootstrapped re-samples) for each of the ten headword-specific mixture components. For reference, the probability of headword annotation, marginalized over all possible synonym pairs, is plotted in green. (H) The three curves indicate the expected fraction of undocumented synonymy that would be discovered upon repeatedly and independently constructing additional lexical resources (x-axis) identical to the complete dataset (blue), WordNet only (red), and WordNet plus Webster's New World (green).

We carried out our analysis by combining the annotations provided by eight typeset dictionaries and one digital thesaurus. The typeset dictionaries [41]–[48] represent some of the most widely used synonym references, while WordNet [4] is a digital thesaurus popular within the artificial intelligence community. In Figures 2A and 2B, we depict the overlap among headwords (i.e. concepts) and synonym pairs (i.e. terms) that were annotated by the nine dictionaries in this study. The overlap of annotated headwords appears fairly high for these thesauri (Figure 2A), while the overlap among their synonyms is substantially lower (Figure 2B). After fitting our annotation model to the nine thesauri, we predicted that only 30% of headwords are missing from the combined dataset (see Figure 2C), although we note that the annotation of headwords for general-purpose thesauri appears to be heavily biased towards words of higher frequency (see Figure S3). By contrast, our method predicted that 93% of near-synonymous relationships are currently undocumented, with the majority belonging to previously documented headwords (86%, see Figure 2D, blue vs. red bars).

Our analysis also predicted that general-English near-synonymy is extremely pervasive: each headword, on average, was predicted to have about 200 near-synonyms, although the variance in number of synonyms was predicted to be large (≈4×10^4^). Because “missing species” estimators systematically underestimate richness [39] and the English language is currently undergoing exponential growth [49], the extent of undocumented synonymy predicted by our model is likely a severe underestimate of reality. If we extrapolate our results to more realistic estimates for the number of English headwords [49] (see Supporting Information Text S1 for details), the extent of near-synonymy in the language is likely to be at least an order of magnitude larger than the prediction generated by our model (see Figure 2E).

Beyond providing insight into the extent of near-synonymy among simple English words, this analysis allowed for a more critical scrutiny of our statistical approach to latent synonym inference. For example, we observed that the number of synonyms annotated per general English headword was highly variable and its distribution was multimodal (Figure 2F, in gray). Our mixture modeling approach captured this variation quite well (Figure 2F, in blue), especially in comparison to more standard approaches, like unimodal Geometric or Log-Gaussian models (Figure 2F, in red and green). Furthermore, as noted in the previous section, the terminologies analyzed in Figure 2 were likely constructed according to their authors' own unique preferences and biases. We predicted, for example, that general-English thesauri would be constructed with a bias for writing over reading, following from the observation that thesauri are typically used to add richness and variety while composing text. In support of this hypothesis, we found that headwords in our dictionaries tended to be shorter and more frequent than non-headwords (see Figure S3A and S3B, respectively). Although we did not specifically encode this bias into our statistical framework, our mixture-modeling approach captured it well (see Figure 2G). Our method also captured other types of bias and variability present within the thesauri (e.g., a preference for certain parts-of-speech, see Figure S4A), as the annotation rates for different mixture components varied considerably across terminologies (Figure S4A and S4B). Finally, we note that the continual production and conglomeration of manually curated thesauri is unlikely to be a fruitful strategy for collecting undocumented general-English near-synonymy. It would require approximately 2000 independently collected, WordNet-sized dictionaries to unearth 90% of the undocumented relationships (Figure 2H). Thus, alternative strategies will be necessary to uncover a considerable fraction of undocumented English near-synonymy. In the following section, we utilize one such approach to uncover previously undocumented English near-synonyms.

### Experimental Validation of Undocumented English Near-Synonymy

Our statistical analyses predicted ubiquitous undocumented synonymy among common English words. But do such missing relationships truly exist, and if so, are they of sufficient semantic similarity to necessitate inclusion in English thesauri? We sought to answer these questions and validate some of our predictions concerning undocumented near-synonymy by uncovering relationships not annotated in our combined dataset. Because we predicted so much missing synonymy, we reasoned that it should be relatively straightforward to uncover examples. The analysis performed in the previous section, however, suggested that examining more manually curated thesauri was unlikely to be the most productive approach. Instead, we developed a targeted, “crowd-sourcing” system for near-synonym discovery and validation, and we used this method to test whether such relationships were ubiquitous and potentially of sufficient quality to justify inclusion into lexical resources.

To perform this experiment, we first generated a random list of 300 undocumented, provisional headwords, sampled from Wikipedia, including “phenotype”, “unhealthily”, and “instinctual” (see Materials and Methods for details). We then presented this list to workers on the Amazon Mechanical Turk service (Turkers) and asked them to suggest novel synonyms. The notion of near-synonymy within English is complex [1], so rather than attempting to provide a precise definition of the relationship, we instead relied on Turkers' preconceived notions of “synonymy.” To ensure that their definitions aligned with those used by existing thesauri, we performed a crowd-sourced validation experiment that mixed the harvested relationships with known, high-quality pairings (see Materials and Methods) obtained from the thesauri in our dataset (positive controls) and randomly generated null examples (negative controls). We then asked another round of Turkers to validate these proposed synonyms along with positive and negative controls. Finally, we applied a simple, probabilistic model of agreement in the validation process [50]–[52] to the Turker-generated validation data and computed a posterior probability of accuracy for each proposed synonym. This process enabled the identification of novel synonyms that were most like the positive and least like the negative controls. In other words, it calibrated the near-synonymy obtained from the Turkers so that it closely matched that from existing thesauri. On average, Turker evaluators correctly identified true negatives 93% of the time and true positives 67% of the time.

**Figure 3:**
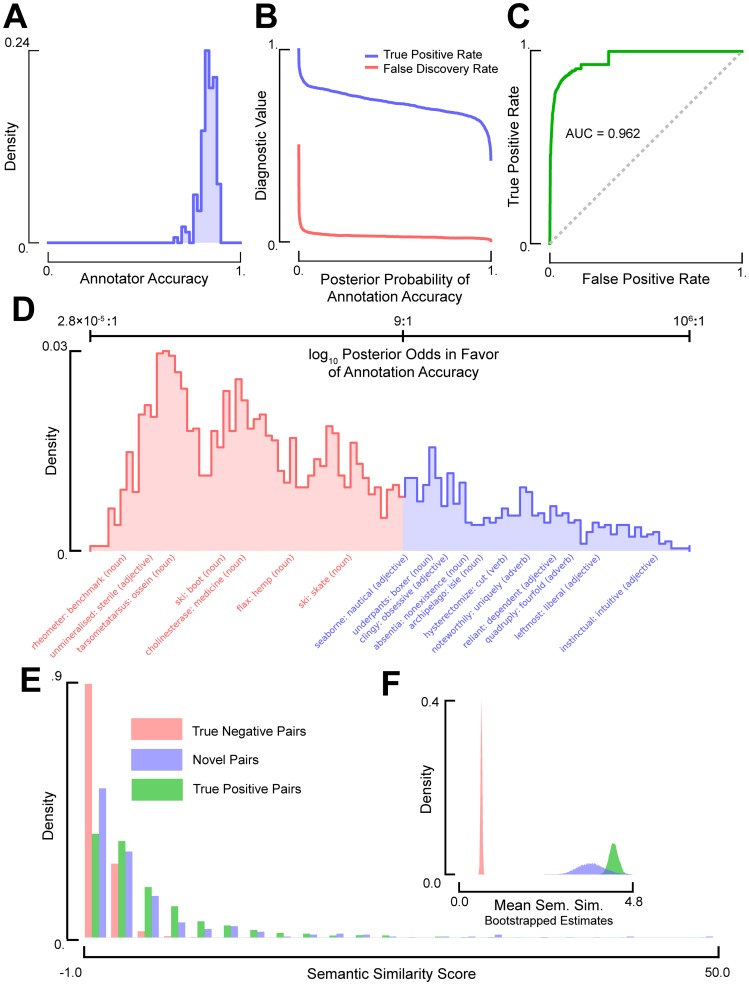
Undocumented, general-English headwords and near-synonyms can be acquired experimentally. (A) The distribution over the inferred accuracies of the annotators validating harvested synonyms. (B) The true positive rate (blue) and false discovery rate (red) of the validation process as a function of the posterior probability of annotation accuracy. Diagnostic statistics were computed using known and random pairings. (C) The Receiver-Operator-Characteristic curve for the statistical model of the validation process, computed using known and random pairings. (D) The distribution over the posterior log-odds in favor of annotation accuracy for the novel synonym-headword pairings, annotated with exemplar pairings (rejected in red and accepted in blue). (E) The distributions over semantic similarity scores for the true negative (red), true positive (green), and novel synonym pairs (blue). (F) Bootstrapped (10,000 re-samples) distributions over the average semantic similarity scores for each group of pairings, computed using the data depicted in (E).

The harvesting experiments proved very successful, generating thousands of potentially novel near-synonym pairs in only a few hours and for a total cost of less than $500. Our amateur linguists proved proficient at identifying previously documented examples of near-synonymy, achieving a mean accuracy of ≈83% (Figure 3A). The combined performance of the crowd-sourcing system was even more impressive: a simple classifier constructed using a model of the validation process [52] was able to distinguish correct synonymous relationships from incorrect ones with an area under the receiver operating characteristic curve (AUC) of 0.962 (Figure 3B and 3C). After selecting a conservative classification threshold (posterior probability (PP)>0.9, false positive rate <2%, true positive rate ≈65%, see Figure 3C and 3D), we generated a list of 707 high-quality, near-synonymous relationships mapping to a total of 214 previously undocumented headwords (provided in Dataset S4). For example, the noun *phenotype* was discovered to be a near-synonym of *trait* (PP>0.99), and the adverb *unhealthily* was paired to *destructively* (PP>0.99), *hazardously* (PP>0.96), and *badly* (PP>0.90). Not all likely candidates of near-synonymy survived this conservative filtering, although the apparent quality of the relationships strongly correlated with their inferred posterior probabilities of accuracy (see Figure 3D). For example, many proposals suggested by more than one Turker were not ultimately accepted (66%), but these were typically strong examples of hypo- or hypernymy (e.g., tribromide: anion). Correspondingly, 44% of the synonyms recommended by multiple Turkers made the final cut, more than double the 21% acceptance rate for those proposed a single time.

To assess the quality our discovered synonymous relationships, we examined their semantic similarity within a corpus of nearly 5 million English Wikipedia articles. Specifically, we measured the semantic similarity among novel, true positive, and true negative synonym pairs by comparing the normalized information content of their shared linguistic contexts to those obtained from a null background (see Supporting Information Text S1) [53]. We found that random synonym pairs (true negatives) had an average semantic similarity of .62, while previously documented synonyms (true positives) had an average similarity score of 4.62 (Figure 3E and 3F). Importantly, the novel synonym pairs validated by our pipeline had an average semantic similarity score of 3.65, and many pairs had scores that were in the top 1% of those obtained by true positive relationships (Figure 3E). This result strongly suggests that at least a fraction of undocumented but easily discoverable relationships are potentially of very high quality.

### The Vast Majority of Biomedical Synonymy Is Undocumented

**Figure 4:**
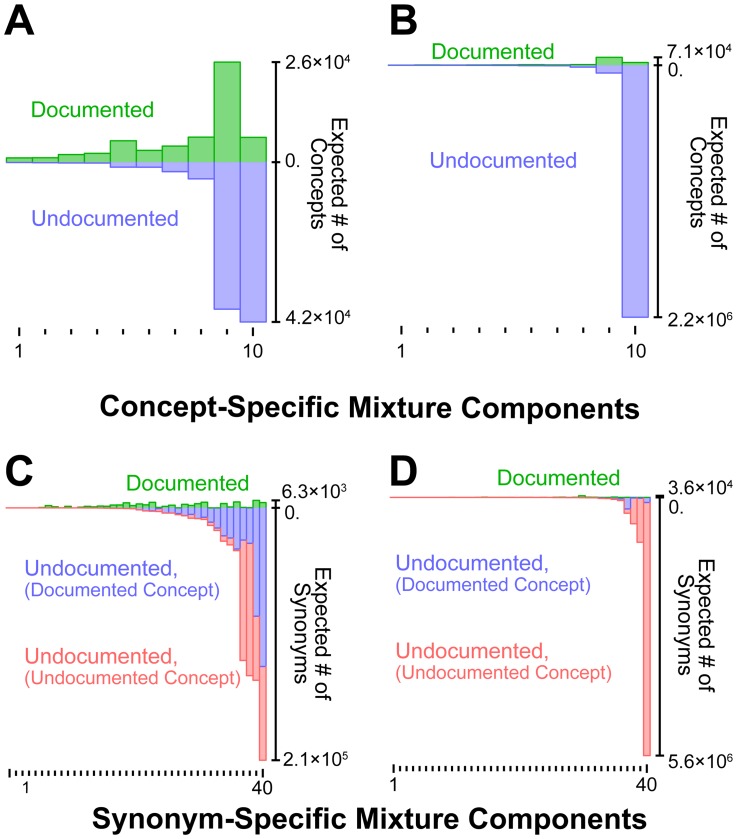
Biomedical terminologies are likely missing the vast majority of domain-specific, synonymous relationships. The numbers of undocumented concepts and synonyms specific to each biomedical sub-domain were estimated using a hierarchical mixture model in order to capture annotation variability that occurred within and across terminologies (10 concept components, each with 4 synonym components, see Materials and Methods and Supporting Information Text S1). In panels (A) and (B), the number of documented concepts per component (green, above x-axis) is compared to the estimated number of undocumented concepts per component (blue, below x-axis): (A) *Diseases and Syndromes* and (B) *Pharmacological Substances*. In panels (C) and (D), the number of documented synonyms per mixture component (green, above x-axis) is compared to the estimated number of undocumented synonyms, which come in two flavors, undocumented synonyms paired to documented concepts (blue, below x-axis) and undocumented synonyms paired to undocumented concepts (red, below x-axis).

Having evaluated and validated the performance of our statistical methodology on the general-English dataset, we applied it to the biomedical terminologies described in the previous sections. The resulting estimates of undocumented synonymy were very high (see Table S5 for a summary of our statistical inference results). Our model predicted that approximately 60% of the concepts and 90% of the synonyms specific to *Diseases and Syndromes* are presently missing from the combined dataset (see Figure 4A and 4C). Furthermore, nearly half of the presently undocumented synonyms belong to concepts currently absent from any terminology (Figure 4C, in red). Finally, we predicted that, on average, each concept in the domain of *Diseases and Syndromes* maps to about 5.85 synonyms, indicating that synonymy is far more prevalent than present vocabularies suggest (each concept currently possesses only 1.15 documented synonyms on average). With respect to the domain of *Pharmacological Substances*, the results are similar but far more extreme: 95% of concepts and 99% of synonyms are presently missing from the combined data set (Figures 4B and 4D, respectively). In contrast with *Diseases and Syndromes*, the vast majority of *Pharmacological Substances* synonyms are associated with undocumented concepts (Figure 4D, red and blue bars), with each concept predicted to have only 3.18 synonyms on average. Thus, it appears that synonymy is more pervasive with respect to *Diseases and Syndromes*.

The amount of synonymy we predict in the biomedical domain pales in comparison to its pervasiveness in general-English, where the average word possesses nearly 200 synonyms. This should not be particularly surprising, as aspects of languages common to more domains of human life should have richer synonymy (i.e. a higher expected number of synonyms per concept). Individuals from numerous cultural backgrounds speak English, and the meanings they assign to common words can be subtle and highly variable. As a whole, this causes such words to become semantically imprecise and increases the odds that their meanings overlap those of other terms, generating a web of enriched synonymy. Moreover, it is important to note that most general-English words are much older than biomedical terms, providing more opportunity for their semantics to evolve and overlap. As the biomedical lexicon becomes used by more sub-cultures, however, it is likely that its terms will acquire new shades of meaning and become less semantically precise. Overall, this suggests that the gulf in synonym richness currently observed between biomedical and general-English terms may attenuate over time.

## Discussion

Terminologies and ontologies have become critical for the analysis and cross-linking of various types of biomedical data [5], [54], [55], especially with respect to information harvested from free text. Due to the enormous amount of lexical and syntactic variation within natural language, most terminologies have made extensive efforts to document synonymy. This work has not been made in vain. In our experiments, synonymy contributed substantially to the successful normalization of disease names, increasing overall recall by 20–40%, depending on the algorithm and corpus (see Table 1). The lack of gold standard corpora makes this normalization experiment difficult to replicate in other biomedical sub-domains, but we showed that the total number of recalled concepts associated with *Pharmacological Substances* also depended strongly on available synonyms (see Supplemental Figure S2). This anecdotal evidence suggests that similar trends are likely to characterize biomedical terminologies in general.

These results, of course, do not automatically imply that all synonyms documented within these terminologies are useful: some fraction of them could be redundant or simply unimportant. Our analyses indicate that, at least for the corpora and algorithms examined, synonymous redundancy is minimal (see Table 1 and Supporting Information Text S1). The notion of “importance,” however, merits a special discussion. At first glance, one might think that the best measurement for a synonym's importance would be its marginal frequency within some large text corpus, but we doubt that this is the case. First, synonyms are context-specific, and therefore, the overall frequency of a synonym without consideration of its various contexts can be misleading. With respected to named-entity normalization, the more relevant metric is likely the estimated frequency with which a synonym maps to a particular concept in natural language, conditional on the occurrence of the concept. Performing such a measurement for every relationship annotated within some thesaurus would clearly require a very large corpus, possibly even the entirety of some linguistic domain. Second, the information content carried by a synonym is inversely related to its frequency. By focusing only on commonly occurring terms, one would invariably miss the rare events that may provide the most insight. Furthermore, determining the “importance” of any particular synonymous relationship using small corpora and a single text-mining task is an ill-posed problem. The utility of synonymy is highly task dependent, so it would be ill advised to deem a relationship “unimportant” after such a limited evaluation. Thus, it is unwise to make universal claims regarding the overall of utility of documented synonymy given the current study. Nevertheless, based on the normalization experiments, we have little reason to believe that our results will not generalize to larger corpora and more nuanced tasks.

Beyond named-entity normalization, synonymy has much broader implications for natural language processing in general [56]. For example, we have proposed that one mechanism for the genesis of synonymy is that it arises from the fusion of diverse “functional linguistic niches,” each drawing on a shared lexicon. In its extreme form, this “narrow field—poor synonymy, broad field—rich synonymy” theory predicts that within narrow subcultures, such as a community of closely interacting biomedical researchers, specialized terms may be used precisely. As more people from different subcultures enter the conversation, however, discourse becomes more ambiguous and synonymy more commonplace. At the same time, disjoint communities may use concepts and phrases that appear dissimilar but are actually very close in meaning. For example, the Black–Scholes equations used in quantitative finance [57], [58] and approximations to the Wright-Fisher process from population genetics [59] are intimately connected to physical models of diffusion, but this may not be evident to a physicist listening to an economics or genetics lecture. Uncovering such deep isomorphisms between concepts and ideas from distinct domains is one of the “Holy Grails” of text mining, but at present, such powers are only available to the most broadly educated human researchers. We believe more thorough documentation of synonymy represents a first step toward the automated discovery of deep semantic relationships that link disparate realms of knowledge.

Given its potential positive impact on named-entity normalization and text mining in general, we believe that documentation of lexical and syntactic variation within biomedical terminologies is a critical problem within the field. Although other types of lexical relationships may be equally or even more important for various text-mining tasks (e.g., hypo/hypernymy, meronymy), we have demonstrated that deficiencies in synonymy levy a clear and quantifiable toll on normalization recall. The question then becomes “How much synonymy is missing, and how should we go about collecting and storing it?” We used statistical modeling to predict that the vast majority (>90%) of synonymous relationships are currently missing from the biomedical terminologies that we investigated. With respect to collection and storage, it seems unlikely that manual annotation and documentation of concept-synonym pairs with no indication of quality will be able to face the enormity of the challenge. For perspective, our statistical model predicts that the “true” *Pharmacological Substances* terminology should contain close to 2.5 million concepts and nearly 8 million synonyms.

Thus, we believe that current biomedical terminologies have substantial room for improvement with respect to the acquisition, storage, and utilization of synonymy. Most importantly, these lexical resources must move well beyond fixed dictionaries of manually curated annotations. Instead, they should become “living” databases, constantly evolving and expanding like search engines that index the enormity of the changing web. Such databases could initially integrate well-established core terminologies, like the Metathesaurus [5], but should ultimately be much broader in scope. Indeed, a distributed lexical database should contain multiple linguistic relationships, and each of the proposed associations should be assigned a unique and consistent measurement of its quality or evidentiary support. This value, computed using a combination of expert evaluations and automated analyses conducted over an ever-expanding corpus of natural language, should be updated in real time. By assigning such measurements to relationships, the terminology should never appear bloated to individuals interested in only the highest quality associations. This weighted, networked approach to lexical terminologies is similar in principle to the functional gene networks globally curated, annotated, and used within the genomics community [60]–[63]. Instead of modeling relationships among genes, however, the nodes of these lexical networks would represent terms or concepts and the weighted (hyper)-edges would encode linguistic relationships. Because of the sheer magnitude of linguistic knowledge that these resources must entail, they should take advantage of memory efficient representations, such as storing some fraction of synonyms using statistically weighted rules or patterns [64].

To some extent, lexical resources with similar goals are already being actively developed. For example, the UMLS Metathesaurus [5], NCBO BioPortal [54], and BioLexicon [55] all the combine numerous independent terminologies and store multiple linguistic relationships. Consistent with our vision of automatic knowledge acquisition from free text, developers of the BioLexicon used computational methods to uncover novel term variants for gene and protein named entities [55]. In fact, automatic acquisition of synonymous relationships from natural language is not a new idea [65]–[67], and numerous researchers have developed general-purpose, automated synonym extraction algorithms for the biomedical domain [56], [64]. These efforts are steps in the right direction, but we feel that they fall short of our vision for “next generation” terminologies in several ways. First, although relatively thorough, these databases do not systematically annotate the quality of their documented linguistic relationships. In our opinion, this greatly decreases their potential utility, both from an efficiency (i.e., very long search times) and efficacy (i.e., results obtained may be of dubious quality) standpoint. Second, current resources are largely static and do not adapt to newly acquired knowledge or the expanding linguistic environment. Thus, they remain distinct from our envisioned “living” terminologies.

For a terminology to be “living” requires a number of essential attributes. First, it needs a large, dedicated community of users and experts heavily invested in maintaining its quality and relevance. Second, the terminology must be able to evolve by identifying and repairing its deficiencies. Many of these deficiencies, such as gaps in coverage or inconsistencies in logical structure, could be identified automatically using statistical methods similar to those utilized in the present work. To be most effective, however, “next generation” terminologies should be designed with computational tools and corpora that extend and repair them in real time. For example, a named entity recognition and normalization tool like MetaMap [24] could encounter an unknown term, store various similarity measurements it inherently computes from corpora, and then provide this data back to the terminology in a structured format. The automated terminology could then integrate this term into its knowledge base. That way, when the term is subsequently encountered in another context, perhaps even by a different computational tool, more and more knowledge concerning its linguistic relationships and contexts would accumulate and become available to all within the community. Ultimately, this would ensure that the terminology evolves with the linguistic domain it was intended to document.

Perhaps most importantly, “next generation” lexical terminologies should be readily accessible to a wide range of computational tools and researchers, as their growth and performance will be inextricably tied to ease of use. This can be partially accomplished by developing a suite of software tools tuned to a specific database, similar to the MetaMap [24] and MetamorphoSys [5] software programs that accompany the UMLS Metathesaurus. We believe that a truly successful “living” terminology, however, must be simple and transparent enough to transcend the use of specialty software. This may prove the most difficult challenge faced in the development of these resources, and we imagine that its solution will require new crowd-sourcing, natural language modeling, and distributed computing technologies that facilitate the integration of diverse information into a networked whole. The development of this technology is not unlike the sequencing of the human genome in scale and importance. A vast library of linguistic relationships among an ever expanding collection of words and phrases would allow a quantum leap in machine reading, understanding and intelligence, with applications relevant not only to biomedicine but all fields of science and scholarship.

## Materials and Methods

### Constructing the Biomedical Terminologies

The two biomedical terminologies used in this study, *Diseases and Syndromes* and *Pharmacological Substances*, were extracted from the UMLS Metathesaurus [5]. The Metathesaurus is a large collection of over 100 vocabularies documenting a variety of linguistic relationships among biomedical concepts, which in turn link into a single, semantic network. Similar to the key-value structure of traditional dictionaries, the Metathesaurus is organized around a set of concepts (keys), each of which is associated with one or more linguistic terms (values). When multiple terms are assigned to a single concept, such variants represent distinct encodings of the same linguistic entity. Therefore, whenever a Metathesaurus key is annotated with two or more phrases (values), those phrases are synonymous with each other. Consistent with previous work [13], [68], [69], we identified a set of technical phrases in the Metathesaurus that were representative of artificial machine-readable sublanguages (such as database-specific encodings) rather than natural language. Previous studies found that removing these specialized terms improved information extraction from natural text [22], [68]. Therefore, prior to isolating the terminologies, we subjected the Metathesaurus to the rule-based filtering outlined in [68]; see the Supporting Information Text S1 for details. To perform the annotation overlap analysis described in the main text, we used the metadata provided by the Metathesaurus in order to determine the vocabularies of origin for each concept-term pair. After processing, the *Diseases and Syndromes* dataset (Dataset S1) incorporated 59,265 concepts paired with 127,431 terms derived from 14 vocabularies (see Table S2). The *Pharmacological Substances* dataset (Dataset S2) contained 122,266 concepts aligned with 198,270 terms harvested from 11 vocabularies (see Table S3). To recast the datasets as concepts and synonyms, one term from each annotated set was assigned as the preferred term (in accordance with the UMLS designations), also known as the headword (consistent with the general-English thesauri), while the remaining were treated as synonyms.

### The General English Headword-Synonym Dataset

We constructed the general English synonym dataset by digitizing 8 hard-copy thesauri [41]–[48] and combined them with the digital WordNet [4], as described previously [70]. Similar to the biomedical terminologies, the general English dataset follows a key-value structure, but instead of annotating concepts with terms, this dataset explicitly assigns lists of synonyms to specific English words, which we call headwords. This direct enumeration of synonymy among specific words implies that these relationships possess an inherent directionality, which in turn suggests that this phenomenon is not bidirectional, at least according to all of the print dictionaries included in our analysis. WordNet is the only exception, as it explicitly assumes synonymy is bidirectional [4]. Implied directionality in most thesauri reflects the subtle nature of near-synonymy in natural language [1], [71] and the complicated notions that underlie thesaurus construction [3]. To account for synonym-headword directionality in our downstream analyses, we treated each headword-synonym pair and its potential inverse as distinct entities. After parsing each dictionary and joining the resulting annotations by headword, the subsequent synonym compendium was subjected to thorough post-processing in order to remove word phrases and linguistic variation caused by differences in conjugation [72]. The full compendium, with words replaced by numerical keys (in accordance with copyright law), is provided as Dataset S3. In total, this file contains over one million unique synonym pairs mapping to just over 53,000 headwords (see Table S4).

### Wikipedia Corpus Generation and Analysis

To examine the properties of general English headwords and their synonyms in free text, we constructed a large corpus using Wikipedia (downloaded in October 2011). Part-of-speech information was assigned to this corpus using the Stanford Tagger (model: left3words-wsj-0-18.tagger) [73], and headword/synonym word frequencies were estimated using a Dirichlet-Multinomial smoothing model [74], [75]. To estimate semantic similarity among synonym pairs, we computed the normalized information content of their shared contexts [53] and compared this value to a null background distribution. Details concerning this procedure are provided in the Supporting Information Text S1.

### Assessing the Effects of Synonymy on Biomedical Concept Normalization

To assess the effects of synonymy on disease name normalization, we used two expertly-annotated gold-standard corpora [25], [26]. The AZDC corpus [26] was constructed using nearly 3,000 sentences isolated from 793 biomedical abstracts, and its disease name mentions were mapped to the UMLS Metathesaurus. The NCBI corpus [25] builds upon the previous dataset by performing a more thorough annotation of these same 793 abstracts, although the version we obtained was annotated using the MEDIC terminology [76] rather than the UMLS. We replaced the MEDIC annotations with UMLS concepts by aligning the database identifiers included within both terminologies. Consistent with previous studies [21], we expanded the abbreviations in each corpus using the tool developed in [77], as we wished to mitigate errors due to abbreviations resolvable using current technology. After abbreviation resolution, we isolated all disease mention-concept pairs from each corpus, as we were focusing on disease name normalization. Finally, in order to prevent a few common disease names from dominating our results, we restricted our analyses to the set of all unique concept-mention pairs.

Following pre-processing, each corpus was evenly split into testing and training sets, although only one of the algorithms included in this study required training [21]. We evaluated the effects of synonymy on named-entity normalization by comparing recall, precision, and the F1-measure (harmonic mean of precision and recall) for four algorithms before and after removing synonymy, using the testing set only. The four normalization algorithms implemented in this study were: Boolean search, MetaMap [13], cosine similarity, and pairwise Learning-to-Rank (pLTR, as described in [21]). Implementation and training (if applicable) of these algorithms is described in more detail in the Supporting Information Text S1. Note that some concepts annotated within the corpora were not included in the *Diseases and Syndromes* terminology—the annotators used a more expansive definition of the sub-domain—so recall and precision were evaluated only with respect to those mentions whose concepts were included in this terminology. To assess the effects of synonym coverage on concept recall for *Pharmacological Substances*, we compared the total number of concepts recovered by MetaMap [13] from a large corpus of free text before and after removing all synonyms from the terminology. Our corpus of free text was constructed by randomly sampling 35,000 unique noun phrases from the abstracts contained within the MEDLINE database [78]. Noun phrases were isolated from free text using the OpenNLP software suite [79].

To determine the fraction of redundant synonyms for a particular algorithm and corpus, we randomly removed fractions of synonyms from the terminology of interest and re-computed the number recalled terms (see Figure S1). Assuming that each disease name mention maps to only one, non-redundant concept-to-term relationship, then the number of recalled concepts should decrease linearly with the fraction of removed synonyms. If such mentions actually map to multiple concept-to-term annotations, however, then the number of recalled concepts will actually decrease at a non-linear rate. In fact, the fraction of redundant concept-to-term annotations (and thus synonyms) can be estimated from changes in concept recall that occur as different fractions of synonyms are randomly removed from the terminology. These estimates are provided in Table 1, but details concerning the estimation (including assumptions and limitations) are described in the Supporting Information Text S1.

### Estimating the Extent of Undocumented Synonymy

As discussed in the main text, we extended a parametric, model-based solution to the “missing species” problem in order to compute estimates for the true numbers of concepts and synonyms belonging to particular biomedical sublanguages. Essentially, solutions to the “missing species” problem attempt to predict the true number of species in some environment of interest given an incomplete sub-sample [38]–[40]. Below, we outline the mathematical details concerning our model and how it can be used to estimate the quantities of interest. The following description can be seen as a sequence of three interconnected parts. First, we describe how the process of annotating synonyms for a single concept can be modeled using a Poisson process. Second, we describe how Bayes' Theorem can be used in conjunction with this Poisson model to generate a prediction for the number of synonyms missing with respect to this concept. Third, we briefly outline how this approach can be extended to infer the total number of synonyms missing from an entire thesaurus. Further details concerning the approach, including its extension to multiple dictionaries and the inclusion of mixture components, are relegated to the Supporting Information Text S1.

To begin, imagine that a lexicographer annotated the terms associated with some concept of interest by “sampling” them from the “environment.” The precise definition of “sampling” is irrelevant, but one can imagine that the complex process of detecting concept-to-term relationships from linguistic experience depends on a series of probabilistic events (e.g., coming across a particular article; having a conversation with a certain scientist, etc), not unlike the capturing of biological species. Thus, according to this analogy, the corpus of natural language specific to a lexicographer's domain of interest represents the “environment.” Let 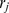 denote the number of times that relationship *j* was sampled by some lexicographer, and let 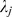 indicate the Poisson process-sampling rate for this relationship. The sampling probability associated with the *j*th concept-to-term relationship is:
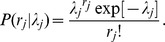
As discussed in the main text, this equation implicitly assumes that the total number of occurrences of relationship *j* in the language of interest (i.e. the “species population”) is infinite. Obviously, this is an approximation, but given that these “populations” are very large and the sampling probabilities are very small, this should be a reasonable assumption.

To extend this model to the full set of synonymous relationships associated with some concept, let 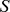 denote the total number of terms that map to this concept (indicating 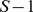 synonyms), including those that were not annotated by the lexicographer. In the parlance of the “missing species problem,” 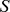 denotes the unobserved total number of species in the environment, and ultimately, we will describe how to infer this value given an incomplete sample derived from the annotations. To do so, we first define the vector 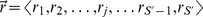, which indicates the number of times each relationship 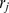 was sampled, given that they were each sampled at least once. This distinction is important, as a relationship that is never sampled (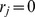) cannot appear in the terminology. Thus, the length of the vector 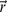, denoted 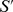, indicates the number relationships annotated by the lexicographer, and thus, 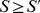.

To simplify the model, let 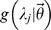 denote the generating (prior) distribution for the Poisson process sampling rates. By marginalizing each 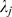 over 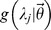, we can reduce the dimensionality of our model by expressing it only in terms of the parameters defining the prior distribution 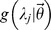:
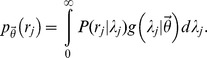
With this notation in place, the full probability model for the relationship-sampling vector 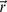 can be decomposed into:
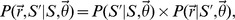
where 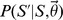 indicates the probability of annotating 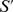 terms at least once and 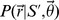 represents the probability of sampling 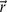, conditional on 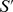. Based on the descriptions of 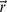 and 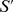 given above, the first term on the right-hand-side is simply the probability that 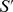 concept-to-term relationships are sampled at least once, which corresponds to the following binomial model [80]:
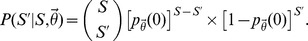
The second term on the right-hand-side corresponds to a multinomial distribution (with an infinite number of categories) [80]. To see this, note that, after marginalizing the Poisson sampling rates out of the model, the probabilities assigned to elements of 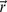 with the same value (denoted *k*) are equivalent:
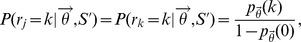
where the denominator in the previous equation re-normalizes the sampling probability to account for the fact that each element in 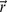 must be greater than 0. Let 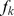 denote the number of relationships that were sampled k times:
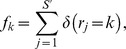
where 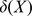 is 1 if 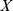 is true and 0 otherwise. Then,
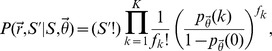
where *K* denotes the largest sampling count in 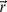 and the factorials correspond to the multinomial coefficient (i.e. accounting for the fact that the elements of 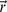 with the same value are statistically indistinguishable).

With our probability model for the annotation data fully specified, we can now describe our approach for estimating the total number of synonymous relationships missing from some annotated set, denoted 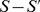. This value can be estimated from the probability distribution over the total number of terms associated with the concept of interest, conditional on the observed data and the model parameters (denoted 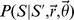). To derive this distribution, we apply Bayes Theorem and note that:
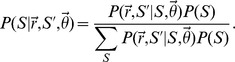
Thus, to estimate 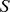, we must specify both the data likelihood (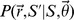, defined in the preceding paragraph) and a prior distribution for the total number of terms paired to the concept of interest (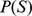). The data likelihood defined above and in the preceding paragraph depends on the sampling count vector 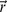, but in practice, we never actually observe the precise number of times each relationship was sampled, only whether it was sampled at least once (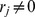). To account for this fact, we can simply marginalize the likelihood 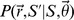 over all possible values of 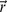. Based on the factorization outlined in the previous paragraph, this is trivial, yielding the following likelihood for observing 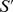 annotated concept-to-term relationships:
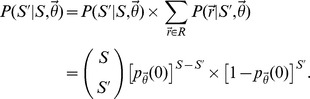
By coupling this likelihood with a simple prior distribution, we can easily specify the desired posterior distribution over the true number concept-to-term relationships. For example, assuming that the true number of terms paired to each concept is geometrically distributed (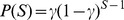), the posterior distribution for 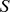 is:
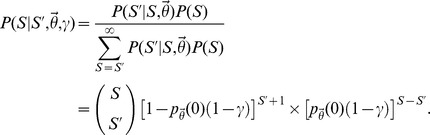
At this point, the expectation of the posterior distribution can used to estimate the total number terms that were not annotated for some concept of interest, given by:
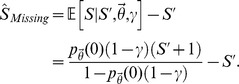
In practice, the previous posterior is not very useful unless 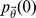 and 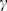 are known. Otherwise, there are three unknown parameters in a model with only one observation, rendering joint inference intractable. To overcome this difficulty, we 1) assumed that the sampling probabilities 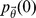 were correlated across concepts annotated by the same dictionary and 2) jointly modeled the annotations provided by multiple, independent dictionaries. Given these assumptions, we were able to construct a global likelihood for all of the synonymous relationships documented by a set of terminologies. This in turn enabled us to estimate the total number of undocumented relationships specific to the linguistic domain of interest while simultaneously providing enough information to estimate the unknown parameters 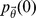 and 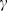.

To derive this likelihood with respect to a single dictionary, let 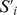 denote the number of concept-to-term relationships that were annotated (observed in the terminology) with respect to the *i*th concept, and similarly, let 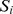 denote the true number of terms for this concept. Assume that a total of 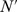 concepts were annotated in the terminology, such that 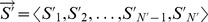 denotes the full vector of observed relationships. To correctly specify a probability model for the vector 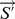, we must also consider those concepts whose terms were not annotated within the terminology (i.e. 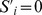). Let 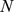 denote the true number of concepts in the linguistic domain, and let 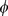 denote the total number of concept-to-term relationships associated with the 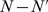 undocumented concepts. The likelihood for the observed data 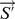 and 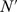
_,_ conditional on fixed 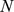, 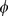 and 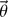, is:
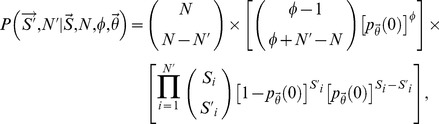
where the first multiplication factor (a binomial coefficient) accounts for the number of ways to select 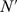 annotated concepts from a total pool of 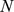, the second factor in square brackets accounts for the probability of failing to annotate 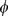 synonymous relationships (marginalized over all possible assignments to the 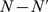 undocumented concepts), and the third factor provides the probability of annotating the 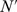 observed concepts. Extending the previous equation to multiple independent dictionaries is straightforward, illustrated in the Supporting Information Text S1.

By coupling the previous likelihood with a joint prior distribution for the unknown quantities of interest (denoted 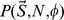, see Supporting Information Text S1 for details), the model outlined above can be used to derive a posterior distribution for the true number of terms paired with the documented concepts (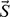), the total number of concepts specific to the linguistic domain of interest (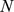), and the number of concept-to-term relationships associated with the undocumented concepts (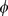). In practice, however, this requires knowledge of the relationship sampling rates (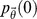) and the parameters defining the prior distribution over the number of undocumented concepts and terms. To circumvent this issue, we jointly inferred the numbers of undocumented concepts and terms along with the unknown parameters using an approximate, Bayesian approach [81], [82]. Details concerning this procedure are provided in the Supporting Information Text S1.

Finally, as discussed in the main text, the sampling model outlined above assumes that the concept-to-term relationships, both within and across concepts, were sampled from the linguistic domain at equivalent rates. This assumption is somewhat artificial and restrictive, as the terminologies included in this study were likely constructed according to their own unique preferences, biases, and perhaps even definitions of synonymy. To formally account for such variability, we extended the above model by allowing the sampling rates 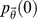 to vary across terminologies, concepts, and terms. Consistent with previous applications [83], we found that the mixture modeling approach successfully captured the variation we observed both within and across different terminologies. Details concerning the mixture model, its inference, and resulting estimates of undocumented synonymy are provided in the Supporting Information Text S1. A summary of the modeling results is provided in Table S4.

### Crowd Sourcing Undocumented General English Synonymy

To find potentially undocumented headwords of high-quality, we passed all of the words contained within our Wikipedia corpus through several filters (including a large English dictionary [84]), removing proper nouns, misspelled words and those words annotated as headwords in our general English dataset. We then randomly sampled 300 of these putative undocumented headwords for downstream analysis. In order to harvest previously undocumented synonymous relationships, we turned to the Amazon Turk web service and hired a work force of general-English speakers. To ensure that our work force included only highly-qualified “Turkers,” we only allowed individuals with an IP address in the United States to complete our task, and we further required that each Turker have an approval rating of at least 85%.

We conducted our harvesting experiment by posting a set of 100 Human Intelligence Tasks (HITs), where each task consisted of a random group of three candidate headwords. Within each HIT, we asked the Turkers to provide at least five novel synonyms for the group of three headwords. In our instructions to Turkers, we specifically emphasized that only single-word answers were allowed and the parts-of-speech of the headword and synonym must match. Because the notion of synonymy is somewhat vague and open to interpretation [1], [71], we did not explicitly provide Turkers with a precise definition of the relationship, relying instead on their individual intuitions and self-imposed definitions. To ensure that Turkers' definitions of synonymy were consistent with those used by established dictionaries, we incorporated positive and negative controls into our subsequent validation stage (see below for details). After each HIT was completed three times, we automatically filtered the candidate synonym pairs for mismatched parts-of-speech and misspellings using the SPECIALIST lexicon [84] and the iSpell word list, respectively. After filtering, we obtained a total of 2,871 entirely novel, candidate synonymous relationships.

We validated the harvested synonymous relationships by combining them with positive and negative controls and subjecting them to an additional crowdsourcing experiment. For a positive control, we selected the top 5,000 synonym pairs from our dataset previously determined to be highly interchangeable in written English text [70]. For a negative control, we generated a set of 5,000 synonymous relationships by randomly shuffling lists of headwords and synonyms with identical parts-of-speech. We conducted the validation experiment by assigning each HIT a random group of ten known pairings, ten random pairings, and ten harvested pairings. For each of the 30 synonym pairs, we asked Turkers a simple true-false question: “Do you think that *A* is a synonym of *B* (given *B*'s part of speech *C*)?” With respect to the harvesting experiment, we set higher criteria for our validation Turkers. In addition to being located within the US, they had to have completed more than 100 HITs with an approval rating of 95% or higher. To prevent poorly performing Turkers from biasing our results, we removed all responses in which the corresponding Turker did not annotate the known and random pairings at a performance level significantly better than random (T-test, *p*>0.05 after correcting for multiple testing); all rejected HITs were re-posted for another round of validation by a different Turker.

After conducting the experiment, we evaluated each candidate synonym pair by computing the posterior probability that it represented a true relationship. We computed these probabilities by applying a statistical model of the validation process [50]–[52] to the Turker-generated synonym data. We fit the model using the PyAnno software package [85], which provided the posterior probability that each synonym pair represented a true relationship, conditional on the data and underlying model parameters. Ultimately, the positive and negative controls allowed us to evaluate the quality of modeling predictions. As described in the main text, a simple binary classifier constructed from the posterior validation probabilities identified synonymous relationships harvested from the dictionaries with an area under of the receiver-operating-characteristic curve of 0.962. Using the known and random pairings as a guide, we estimated the posterior probability threshold values for various true and false positive rates. In the end, we reported our results with respect to a false discovery rate of 2%. The full table of candidate synonym pairs and validation results is provided in Dataset S4.

## Supporting Information

Dataset S1 **The general-English near-synonymy dataset.** Each line in the file provides a headword, its annotated synonyms, and a binary array that indicates the annotating dictionaries for each pair. The dictionaries are listed according to their order in the binary array (column-wise) on the first line of the file. Note, headwords and synonyms have been replaced by integers in accordance with copyright law.
(ZIP)

Dataset S2 **The *Diseases and Syndromes* synonym dataset.** The format of this file is identical to that of Dataset S1. See Supporting Information Text S1 for the processing procedures that resulted in this dataset.
(ZIP)

Dataset S3 **The** ***Pharmacological Substances*** **synonym dataset.** The format of this file is identical to that of Dataset S1. See Supporting Information Text S1 for the processing procedures that resulted in this dataset.
(ZIP)

Dataset S4 **The headwords and harvested synonym pairs obtained from the crowd-sourcing experiment.** Each line in the file contains a provisional a headword, its part-of-speech, its harvested synonyms, and their associated posterior probabilities computed from the validation experiment.
(ZIP)

Figure S1 **Missing synonymy negatively affects disease name normalization.** To test the importance of synonymy for named entity normalization, we removed random subsets of synonyms from the *Diseases and Syndromes* terminology (x-axes indicate the fraction remaining) and computed recall (blue), precision (red), and their harmonic average (F1-measure, green) (y-axis) for four normalization algorithms (bottom) applied to two disease name normalization gold-standard corpora (left). Error bars represent twice the standard error of the estimates, computed from five replicates. Numerical results are presented in Table 1, and a description of the methodology is provided in the Materials and Methods and the Supporting Information Text S1.
(TIF)

Figure S2 **Recall of normalized** ***Pharmacological Substances*** **depends on synonymy.** The fraction of the total number of recalled concepts returned by MetaMap (y-axis) upon removing a subset of the synonyms contained within the *Pharmacological Substances* terminology (x-axis indicates fraction remaining). The evaluation corpus consisted of 35,000 unique noun phrases isolated from MEDLINE (see Materials and Methods for details).
(TIF)

Figure S3 **Headword selection bias in general-English thesauri.** (A) The empirical distribution over stemmed word length shown for headwords (blue) and non-headwords (synonyms only, red). The inset panel depicts bootstrapped estimates (1000 re-samples) for the mean values of these two distributions. (B): Relative word frequency of headwords (blue) and non-headwords (synonyms only, red). In both cases, a Student's T-test for a difference in means produced a *p*-value <2.2×10^−16^.
(TIF)

Figure S4 **Bias and variability captured by the annotation mixture model.** (A) The distributions over parts-of-speech across the ten headword components specified within the best-fitting mixture model. (B): The probability of headword annotation, marginalized over all possible numbers and classes of synonyms, for the complete set of nine, general-English thesauri.
(TIF)

Table S1 **Examples of missing synonyms annotated within the gold-standard disease name normalization corpora.** The first column indicates the term mentioned in the text, while the second column provides the annotated concept. The third column indicates the corpus of origin. Algorithms considered in this study did not properly normalize any examples provided here presumably because the synonym was not provided in the complete disease name terminology.
(PDF)

Table S2 **The sources for the *Diseases and Syndromes* dataset.** Summary statistics for the thirteen thesauri used to construct the Diseases and Syndromes terminology.
(PDF)

Table S3 **The sources for the** ***Pharmacological Substances*** **dataset.** Summary statistics for the ten thesauri used to construct the *Pharmacological Substances* terminology.
(PDF)

Table S4 **The sources for the general-English dataset.** Summary statistics for nine thesauri used to construct the general-English near-synonym terminology.
(PDF)

Table S5 **Estimates for the extent of undocumented synonymy for the three terminologies included in this study.** This table provides the lower bound on the log-evidence for the best fitting annotation mixture models specific to each lexical domain. Moreover, it provides the fraction of headwords/concepts and synonym pairs/terms predicted to be undocumented within each dataset. Values in parenthesis indicate the 99% credible intervals for the estimates.
(PDF)

Text S1 **Supplemental materials and methods.**
(PDF)
