# Supplementary material for: Quantifying the Impact and Extent of Undocumented Biomedical Synonymy
Source: PLoS Comput Biol. 2014 Sep 25;10(9):e1003799. doi: 10.1371/journal.pcbi.1003799 (PMC4177665; doi:10.1371/journal.pcbi.1003799)
Supplement: Dataset S1 — The general-English near-synonymy dataset. Each line in the file provides a headword, its annotated synonyms, and a binary array that indicates the annotating dictionaries for each pair. The dictionaries are listed according to their order in the binary array (column-wise) on the first line of the file. Note, headwords and synonyms have been replaced by integers in accordance with copyright law. (ZIP) [file pcbi.1003799.s001.zip › SupportingDataset_S1.rtf]

Dictionaries: Merriam Webster Thesaurus; The Oxford Thesaurus; The Synonym Finder; Webster's New World Roget's A-Z Thesaurus; WordNet; Scholastic Dictionary of Synonyms, Antonyms, and Homonyms; The Oxford Dictionary of Synonyms and Antonyms; 21st Century Synonym and Antonym Finder; A Dictionary of Synonyms and AntonymsHeadword Key	Synonym Key(s)	Annotations0	1	[[0,0,0,0,1,0,0,0,0]]2	3	[[0,0,0,0,1,0,0,0,0]]4	5,6,7,8,9,10,11,12,13,14,15,16,17,18,19,20,21,22,23,24,25,26,27	[[0,0,1,1,0,0,0,0,0],[0,1,1,1,0,0,0,0,0],[0,1,1,1,0,0,0,0,0],[0,0,1,0,0,0,0,0,0],[0,1,0,0,0,0,0,0,0],[0,1,1,0,0,0,0,0,0],[0,1,1,0,0,0,0,0,0],[0,1,1,0,0,0,0,0,0],[0,0,0,0,1,0,0,0,0],[0,1,1,0,0,0,0,0,0],[0,0,1,0,0,0,0,0,0],[0,0,1,0,0,0,0,0,0],[0,0,1,0,0,0,0,0,0],[0,0,1,0,0,0,0,0,0],[0,0,1,0,0,0,0,0,0],[0,0,1,0,0,0,0,0,0],[0,0,1,0,0,0,0,0,0],[0,1,0,0,0,0,0,0,0],[0,1,1,1,0,0,0,0,0],[0,1,1,1,0,0,0,0,0],[0,1,1,0,0,0,0,0,0],[0,0,1,0,0,0,0,0,0],[0,0,1,0,0,0,0,0,0]]28	29	[[0,0,0,0,1,0,0,0,0]]30	31,32,33,34,35,36,37,38,39,40,41,42,43,44,45,46,47,48,49,50,51,52,53	[[0,0,0,1,0,0,0,0,0],[0,0,0,1,0,0,0,0,0],[0,0,0,1,0,0,0,0,0],[0,0,0,1,0,0,0,0,0],[0,0,0,1,0,0,0,0,0],[0,0,0,1,0,0,0,0,0],[0,0,0,1,0,0,0,0,0],[0,0,0,1,0,0,0,0,0],[0,0,0,1,0,0,0,0,0],[0,0,0,1,0,0,0,0,0],[0,0,0,1,0,0,0,0,0],[0,0,0,1,0,0,0,0,0],[0,0,0,1,0,0,0,0,0],[0,0,0,1,0,0,0,0,0],[0,0,0,1,0,0,0,0,0],[0,0,0,1,0,0,0,0,0],[0,0,0,1,0,0,0,0,0],[0,0,0,1,0,0,0,0,0],[0,0,0,1,0,0,0,0,0],[0,0,0,1,0,0,0,0,0],[0,0,0,1,0,0,0,0,0],[0,0,0,1,0,0,0,0,0],[0,0,0,1,0,0,0,0,0]]54	55,56,57,58	[[0,0,0,1,0,0,0,0,0],[0,0,0,1,0,0,0,0,0],[0,0,0,1,0,0,0,0,0],[0,0,0,1,0,0,0,0,0]]59	60	[[0,0,0,0,1,0,0,0,0]]61	62	[[0,0,0,0,1,0,0,0,0]]63	64,65,66,67,68,69,70,71,72,73,74,75,76,77,78,79,80,81,82,83,84,85,86,87,88,89,90,91,92,93,94,95,96,97,98,99,100,101,102,103,104,105,106,107,108,109,110,111,112,113,114,115,116,117,118,119,120,121,122,123	[[0,0,1,0,0,0,0,0,0],[0,0,1,0,0,0,0,0,0],[0,0,1,0,0,0,0,0,0],[0,0,1,0,0,0,0,0,0],[0,0,1,0,0,0,0,0,0],[0,0,1,0,0,0,0,0,0],[0,0,1,0,0,0,0,0,0],[0,0,1,0,0,0,0,0,0],[0,0,1,0,0,0,0,0,0],[0,0,1,0,0,0,0,0,0],[0,0,1,1,0,0,0,0,0],[0,0,1,0,0,0,0,0,0],[0,0,1,0,0,0,0,0,0],[0,0,0,0,1,0,0,0,0],[0,0,1,0,0,0,0,0,0],[0,0,1,0,0,0,0,0,0],[0,0,1,0,0,0,0,0,0],[0,0,0,1,0,0,0,0,0],[0,0,1,0,0,0,0,0,0],[0,0,1,0,0,0,0,0,0],[0,0,1,0,0,0,0,0,0],[0,0,1,0,1,0,0,0,0],[0,0,1,0,0,0,0,0,0],[0,0,1,1,0,0,0,0,0],[0,0,1,0,0,0,0,0,0],[0,0,0,0,1,0,0,0,0],[0,0,1,0,0,0,0,0,0],[0,0,1,0,0,0,0,0,0],[0,0,1,0,0,0,0,0,0],[0,0,1,0,0,0,0,0,0],[0,0,0,0,1,0,0,0,0],[0,0,1,0,0,0,0,0,0],[0,0,1,0,0,0,0,0,0],[0,0,1,0,0,0,0,0,0],[0,0,1,0,0,0,0,0,0],[0,0,1,0,0,0,0,0,0],[0,0,1,0,0,0,0,0,0],[0,0,1,0,0,0,0,0,0],[0,0,1,0,0,0,0,0,0],[0,0,1,0,0,0,0,0,0],[0,0,1,0,0,0,0,0,0],[0,0,1,0,0,0,0,0,0],[0,0,0,0,1,0,0,0,0],[0,0,1,0,0,0,0,0,0],[0,0,1,0,0,0,0,0,0],[0,0,1,0,0,0,0,0,0],[0,0,1,0,0,0,0,0,0],[0,0,1,0,0,0,0,0,0],[0,0,1,0,0,0,0,0,0],[0,0,1,0,0,0,0,0,0],[0,0,1,0,0,0,0,0,0],[0,0,1,0,1,0,0,0,0],[0,0,1,0,0,0,0,0,0],[0,0,1,0,0,0,0,0,0],[0,0,1,0,1,0,0,0,0],[0,0,1,0,0,0,0,0,0],[0,0,0,0,1,0,0,0,0],[0,0,1,0,0,0,0,0,0],[0,0,1,0,0,0,0,0,0],[0,0,1,0,0,0,0,0,0]]124	125,126,127	[[0,0,0,1,0,0,0,0,0],[0,0,0,1,0,0,0,0,0],[0,0,0,1,0,0,0,0,0]]128	129,130,131,132,133,134,135,136,137,138,139,140,141,142,143,144,145,146,147,148,149,150,151,152,153,154,155,156,157,158,159,160,161,162,163,164,165,166,167,168,169,170,171,172,173,174,175,176,177,178	[[0,0,1,1,0,0,0,0,0],[0,1,0,1,1,0,1,0,0],[0,1,1,1,1,0,1,0,0],[0,0,0,1,0,0,0,0,0],[0,0,0,1,0,0,0,0,0],[0,1,0,0,0,0,0,0,0],[0,0,1,0,0,0,0,0,0],[0,0,0,1,0,0,0,0,0],[0,0,0,1,0,0,0,0,0],[0,1,0,0,0,0,1,0,0],[0,0,1,1,0,0,0,0,0],[0,1,1,1,0,0,1,0,0],[0,0,1,0,0,0,0,0,0],[0,0,1,0,0,0,0,0,0],[0,0,1,1,0,0,0,0,0],[0,1,0,1,1,0,1,0,0],[0,1,1,0,0,0,0,0,0],[0,0,0,1,0,0,0,0,0],[0,1,1,0,0,0,0,0,0],[0,0,1,0,0,0,0,0,0],[0,1,1,0,0,0,1,0,0],[0,0,1,1,0,0,0,0,0],[0,1,0,1,0,0,1,0,0],[0,0,1,1,0,0,0,0,0],[0,0,1,0,0,0,0,0,0],[0,0,1,1,0,0,0,0,0],[0,1,1,0,0,0,1,0,0],[0,1,0,0,0,0,0,0,0],[0,1,0,0,0,0,0,0,0],[0,0,1,1,0,0,0,0,0],[0,0,1,0,0,0,0,0,0],[0,0,1,0,0,0,0,0,0],[0,0,0,1,0,0,0,0,0],[0,0,0,1,0,0,0,0,0],[0,0,0,1,0,0,0,0,0],[0,0,0,0,1,0,0,0,0],[0,0,0,0,1,0,0,0,0],[0,0,0,1,0,0,0,0,0],[0,0,1,0,0,0,0,0,0],[0,1,0,0,0,0,0,0,0],[0,1,0,1,0,0,0,0,0],[0,0,0,1,0,0,0,0,0],[0,0,0,1,0,0,0,0,0],[0,0,0,1,0,0,0,0,0],[0,0,0,1,0,0,0,0,0],[0,0,0,0,1,0,0,0,0],[0,0,1,0,1,0,0,0,0],[0,0,0,1,0,0,0,0,0],[0,0,1,0,0,0,0,0,0],[0,0,0,1,0,0,0,0,0]]179	180,181	[[0,0,0,0,1,0,0,0,0],[0,0,0,0,1,0,0,0,0]]182	41,53,49,50,40,44,33,35,31,39,38,36,34,32,51,48,37,47,46,45,43,52,42	[[0,0,0,1,0,0,0,0,0],[0,0,0,1,0,0,0,0,0],[0,0,0,1,0,0,0,0,0],[0,0,0,1,0,0,0,0,0],[0,0,0,1,1,0,0,0,0],[0,0,0,1,0,0,0,0,0],[0,0,0,1,0,0,0,0,0],[0,0,0,1,0,0,0,0,0],[0,0,0,1,0,0,0,0,0],[0,0,0,1,0,0,0,0,0],[0,0,0,1,0,0,0,0,0],[0,0,0,1,0,0,0,0,0],[0,0,0,1,1,0,0,0,0],[0,0,0,1,0,0,0,0,0],[0,0,0,1,0,0,0,0,0],[0,0,0,1,1,0,0,0,0],[0,0,0,1,0,0,0,0,0],[0,0,0,1,0,0,0,0,0],[0,0,0,1,0,0,0,0,0],[0,0,0,1,0,0,0,0,0],[0,0,0,1,0,0,0,0,0],[0,0,0,1,0,0,0,0,0],[0,0,0,1,0,0,0,0,0]]183	55,58,56,57	[[0,0,0,1,0,0,0,0,0],[0,0,0,1,0,0,0,0,0],[0,0,0,1,0,0,0,0,0],[0,0,0,1,0,0,0,0,0]]184	185,186,187,188,189,190,191,192,193,194,195,196,197,198,199,200,201,202,203,204,205,206,207,208,209,210,211,212,213	[[0,0,1,0,1,0,0,0,0],[0,0,1,0,0,0,0,0,0],[0,0,1,0,0,0,0,0,0],[0,0,1,0,0,0,0,0,0],[0,0,1,0,0,0,0,0,0],[0,0,1,0,0,0,0,0,0],[0,0,0,0,1,0,0,0,0],[0,0,0,0,1,0,0,0,0],[0,0,1,0,1,0,0,0,0],[0,0,1,0,0,0,0,0,0],[0,0,1,0,1,0,0,0,0],[0,0,1,0,0,0,0,0,0],[0,0,1,0,0,0,0,0,0],[0,0,1,0,1,0,0,0,0],[0,0,1,0,0,0,0,0,0],[0,0,1,0,0,0,0,0,0],[0,0,1,0,1,0,0,0,0],[0,0,1,0,0,0,0,0,0],[0,0,0,0,1,0,0,0,0],[0,0,1,0,1,0,0,0,0],[0,0,1,0,0,0,0,0,0],[0,0,1,0,0,0,0,0,0],[0,0,1,0,0,0,0,0,0],[0,0,1,0,0,0,0,0,0],[0,0,1,0,0,0,0,0,0],[0,0,1,0,1,0,0,0,0],[0,0,0,0,1,0,0,0,0],[0,0,0,0,1,0,0,0,0],[0,0,1,0,0,0,0,0,0]]214	215,216,217,218,219	[[0,0,0,0,1,0,0,0,0],[0,0,0,0,1,0,0,0,0],[0,0,0,0,1,0,0,0,0],[0,0,0,0,1,0,0,0,0],[0,0,0,0,1,0,0,0,0]]220	221,222	[[0,0,0,0,1,0,0,0,0],[0,0,0,0,1,0,0,0,0]]223	224,225,226,227	[[0,0,0,0,1,0,0,0,0],[0,0,0,0,1,0,0,0,0],[0,0,0,0,1,0,0,0,0],[0,0,0,0,1,0,0,0,0]]228	229,230,231,232	[[0,0,0,1,0,0,0,0,0],[0,0,0,1,0,0,0,0,0],[0,0,0,1,0,0,0,0,0],[0,0,0,1,0,0,0,0,0]]233	234	[[0,0,0,0,1,0,0,0,0]]235	236,237	[[0,0,0,0,1,0,0,0,0],[0,0,0,0,1,0,0,0,0]]238	239,240	[[0,0,0,0,1,0,0,0,0],[0,0,0,0,1,0,0,0,0]]241	242	[[0,0,0,0,1,0,0,0,0]]243	244	[[0,0,0,0,1,0,0,0,0]]245	246,247,248	[[0,0,1,0,0,0,0,0,0],[0,0,1,0,0,0,0,0,0],[0,0,1,0,0,0,0,0,0]]249	250,251	[[0,0,0,0,1,0,0,0,0],[0,0,0,0,1,0,0,0,0]]252	231,253,254,255,256,257,258,230,259,260,261,262,232,229,263,264,265,266,267	[[0,1,0,1,0,0,0,1,0],[0,1,0,0,0,0,0,1,0],[0,1,0,0,0,0,0,1,0],[0,1,0,0,0,0,0,1,0],[0,1,0,0,0,0,0,0,0],[0,1,0,0,0,0,0,0,0],[0,1,0,0,0,0,0,0,0],[0,1,0,1,0,0,0,1,0],[0,1,0,0,0,0,0,1,0],[0,1,0,0,0,0,0,0,0],[0,0,0,0,0,0,0,1,0],[0,1,0,0,0,0,0,0,0],[0,0,0,1,0,0,0,0,0],[0,1,0,1,0,0,0,1,0],[0,1,0,0,0,0,0,0,0],[0,1,0,0,0,0,0,0,0],[0,1,0,0,0,0,0,0,0],[0,1,0,0,0,0,0,0,0],[0,1,0,0,0,0,0,0,0]]268	269,270,271	[[0,1,0,0,0,0,0,0,0],[0,1,0,0,0,0,0,0,0],[0,1,0,0,0,0,0,0,0]]272	273	[[0,0,0,0,1,0,0,0,0]]274	275	[[0,0,0,0,1,0,0,0,0]]276	277,278,279,280,281,282,283,284,285,286,287,288,289,290,291,292	[[0,1,1,0,1,0,1,1,0],[0,1,0,0,1,0,0,1,0],[0,1,0,0,0,0,1,1,0],[0,1,0,0,0,0,0,0,0],[0,1,0,0,1,0,0,1,0],[0,0,1,0,0,0,0,0,0],[0,0,0,0,0,0,0,1,0],[0,0,1,0,0,0,0,0,0],[0,0,0,1,0,0,0,1,0],[0,1,1,1,0,0,1,1,0],[0,1,0,0,0,0,1,0,0],[0,1,0,0,0,0,1,0,0],[0,0,0,0,1,0,1,0,0],[0,0,1,0,0,0,0,0,0],[0,0,1,0,0,0,0,0,0],[0,0,0,0,0,0,0,1,0]]293	294,295,296	[[0,0,0,0,1,0,0,0,0],[0,0,0,0,1,0,0,0,0],[0,0,0,0,1,0,0,0,0]]297	298	[[0,0,0,0,1,0,0,0,0]]299	300,301,302	[[0,0,0,0,1,0,0,0,0],[0,0,0,0,1,0,0,0,0],[0,0,0,0,1,0,0,0,0]]303	304,305,306,307,308,309,310,311,312,313,314,315,316,317,318,319,320,321,322,323,324,325,326,327,328,329,330,331,332,333,334	[[0,0,1,0,0,0,0,0,0],[0,1,0,0,0,0,0,0,0],[0,1,0,0,0,0,0,0,0],[0,1,0,0,0,0,0,0,0],[0,1,1,0,1,0,0,0,0],[0,1,1,0,0,0,0,0,0],[0,0,1,0,0,0,0,0,0],[0,0,1,0,0,0,0,0,0],[0,0,1,0,0,0,0,0,0],[0,1,0,0,0,0,0,0,0],[0,0,1,0,0,0,0,0,0],[0,0,1,0,0,0,0,0,0],[0,0,1,0,0,0,0,0,0],[0,0,1,0,1,0,0,0,0],[0,0,1,0,0,0,0,0,0],[0,0,1,0,1,0,0,0,0],[0,0,1,0,0,0,0,0,0],[0,0,1,0,0,0,0,0,0],[0,0,0,0,1,0,0,0,0],[0,0,1,0,0,0,0,0,0],[0,0,1,0,0,0,0,0,0],[0,0,1,0,0,0,0,0,0],[0,0,1,0,1,0,0,0,0],[0,0,1,0,0,0,0,0,0],[0,0,1,0,0,0,0,0,0],[0,0,1,0,0,0,0,0,0],[0,0,1,0,0,0,0,0,0],[0,1,0,0,0,0,0,0,0],[0,0,1,0,1,0,0,0,0],[0,0,1,0,0,0,0,0,0],[0,0,1,0,0,0,0,0,0]]335	336,337,338	[[0,0,0,1,0,0,0,0,0],[0,0,0,1,0,0,0,0,0],[0,0,0,1,0,0,0,0,0]]339	340	[[0,0,0,0,1,0,1,0,0]]341	342	[[0,0,0,0,1,0,0,0,0]]343	344,345	[[0,0,0,0,1,0,0,0,0],[0,0,0,0,1,0,0,0,0]]346	347,348,349,350,351,352,353,354,355,356,357	[[0,1,0,0,0,0,0,0,0],[0,1,0,0,0,0,0,0,0],[0,1,0,0,0,0,0,0,0],[0,1,0,0,0,0,0,0,0],[0,1,0,0,0,0,0,0,0],[0,1,0,0,0,0,0,0,0],[0,1,0,1,0,0,0,0,0],[0,1,0,0,0,0,0,0,0],[0,1,0,0,0,0,0,0,0],[0,1,0,0,0,0,0,0,0],[0,0,0,1,0,0,0,0,0]]358	359	[[0,0,0,0,1,0,0,0,0]]360	361,362,363,364	[[0,0,0,0,1,0,0,0,0],[0,0,0,0,1,0,0,0,0],[0,0,0,0,1,0,0,0,0],[0,0,0,0,1,0,0,0,0]]365	366,367,368,369,370,371,372,373,374,375,376,377,378,379,380,381,382,383,384,385,386,387,388,389,390,391,392,393,394,395,396,397,398,399,400,401,402,403,404,405,406,407,408,409,410,411,77,412,413,414,415,416	[[0,0,1,0,0,0,0,0,0],[0,0,1,0,0,0,0,0,0],[0,0,1,0,0,0,0,0,0],[0,0,1,0,0,0,0,0,0],[0,1,1,0,1,0,0,0,0],[0,0,1,0,0,0,0,0,0],[0,1,1,1,1,0,0,0,0],[0,0,1,0,0,0,0,0,0],[0,0,1,0,0,0,0,0,0],[0,0,1,0,0,0,0,0,0],[0,0,1,0,0,0,0,0,0],[0,0,1,0,0,0,0,0,0],[0,0,1,0,0,0,0,0,0],[0,0,1,0,0,0,0,0,0],[0,0,1,0,0,0,0,0,0],[0,0,1,0,0,0,0,0,0],[0,0,1,0,0,0,0,0,0],[0,1,1,0,0,0,0,0,0],[0,0,1,0,0,0,0,0,0],[0,0,1,0,0,0,0,0,0],[0,0,1,0,0,0,0,0,0],[0,0,1,0,0,0,0,0,0],[0,0,1,0,0,0,0,0,0],[0,0,1,0,0,0,0,0,0],[0,0,1,0,0,0,0,0,0],[0,1,1,0,0,0,0,0,0],[0,0,1,0,0,0,0,0,0],[0,0,1,0,0,0,0,0,0],[0,0,1,0,0,0,0,0,0],[0,0,1,0,0,0,0,0,0],[0,0,1,0,0,0,0,0,0],[0,0,1,0,0,0,0,0,0],[0,0,1,0,0,0,0,0,0],[0,0,1,0,0,0,0,0,0],[0,0,1,0,0,0,0,0,0],[0,1,0,0,0,0,0,0,0],[0,1,1,0,0,0,0,0,0],[0,0,1,0,0,0,0,0,0],[0,0,1,0,0,0,0,0,0],[0,0,1,0,0,0,0,0,0],[0,0,1,0,0,0,0,0,0],[0,0,1,0,0,0,0,0,0],[0,0,1,0,0,0,0,0,0],[0,1,1,1,0,0,0,0,0],[0,1,0,0,0,0,0,0,0],[0,0,1,0,0,0,0,0,0],[0,0,1,0,0,0,0,0,0],[0,0,1,0,0,0,0,0,0],[0,0,1,0,0,0,0,0,0],[0,0,1,1,0,0,0,0,0],[0,1,1,0,1,0,0,0,0],[0,0,1,0,0,0,0,0,0]]417	418	[[0,0,0,1,0,0,0,0,0]]419	420	[[0,0,0,0,1,0,0,0,0]]421	422,423,424,425,426,427,428,429,430,431,432,433,434,435,436,437,438,439,440,441	[[0,0,0,1,0,0,0,0,0],[0,0,0,1,0,0,0,0,0],[0,0,0,1,0,0,0,0,0],[0,0,0,1,0,0,0,0,0],[0,0,0,1,0,0,0,0,0],[0,0,0,1,0,0,0,0,0],[0,0,0,1,0,0,0,0,0],[0,0,0,1,0,0,0,0,0],[0,0,0,1,0,0,0,0,0],[0,0,0,1,0,0,0,0,0],[0,0,0,1,0,0,0,0,0],[0,0,0,1,0,0,0,0,0],[0,0,0,1,0,0,0,0,0],[0,0,0,1,0,0,0,0,0],[0,0,0,1,0,0,0,0,0],[0,0,0,1,0,0,0,0,0],[0,0,0,1,0,0,0,0,0],[0,0,0,1,0,0,0,0,0],[0,0,0,1,0,0,0,0,0],[0,0,0,1,0,0,0,0,0]]442	443,444,445,446,337,336,447,448,449,450,451,452,453,454,455,456,457,458,459,460,461,462,463,464,465,466,467,338,468,469,470,471	[[0,0,1,0,0,0,0,0,0],[0,0,1,0,1,0,0,0,0],[0,0,1,0,0,0,0,0,0],[0,0,1,0,0,0,0,0,0],[0,0,1,1,0,0,0,0,0],[0,0,0,1,0,0,0,0,0],[0,0,1,0,0,0,0,0,0],[0,0,1,0,0,0,0,0,0],[0,0,1,0,0,0,0,0,0],[0,0,1,0,0,0,0,0,0],[0,0,0,0,1,0,0,0,0],[0,0,1,0,1,0,0,0,0],[0,0,1,0,0,0,0,0,0],[0,0,0,0,1,0,0,0,0],[0,0,1,0,0,0,0,0,0],[0,0,1,0,0,0,0,0,0],[0,0,1,0,0,0,0,0,0],[0,0,1,0,0,0,0,0,0],[0,0,1,0,0,0,0,0,0],[0,0,1,0,0,0,0,0,0],[0,0,1,0,0,0,0,0,0],[0,0,1,0,0,0,0,0,0],[0,0,1,0,0,0,0,0,0],[0,0,1,0,0,0,0,0,0],[0,0,1,0,0,0,0,0,0],[0,0,1,0,0,0,0,0,0],[0,0,1,0,0,0,0,0,0],[0,0,1,1,0,0,0,0,0],[0,0,1,0,0,0,0,0,0],[0,0,1,0,0,0,0,0,0],[0,0,1,0,0,0,0,0,0],[0,0,1,0,0,0,0,0,0]]472	473,474	[[0,0,0,0,1,0,0,0,0],[0,0,0,0,1,0,0,0,0]]475	476,477,478,479,480,481,482,483,484,485,486,487,488,489,490,491,492,493,494,495,496,497,498,499,500,501,502	[[0,0,1,0,0,0,0,0,0],[0,0,1,0,0,0,0,0,0],[0,0,1,0,0,0,0,1,0],[0,1,1,1,0,0,0,1,0],[0,1,1,0,1,0,0,0,0],[0,1,1,0,0,0,0,1,0],[0,1,1,1,0,0,0,1,0],[0,0,1,0,0,0,0,0,0],[0,0,1,0,0,0,0,0,0],[0,1,1,1,1,0,0,1,0],[0,0,1,0,0,0,0,0,0],[0,0,0,0,0,0,0,1,0],[0,0,1,0,0,0,0,0,0],[0,1,1,0,0,0,0,0,0],[0,1,1,1,0,0,0,1,0],[0,0,1,0,1,0,0,1,0],[0,1,1,0,0,0,0,0,0],[0,0,1,0,0,0,0,0,0],[0,0,1,0,0,0,0,0,0],[0,0,1,0,0,0,0,1,0],[0,1,0,0,0,0,0,0,0],[0,0,1,0,0,0,0,0,0],[0,0,1,0,1,0,0,1,0],[0,0,1,0,0,0,0,0,0],[0,0,1,0,0,0,0,0,0],[0,0,0,0,0,0,0,1,0],[0,0,1,0,0,0,0,0,0]]503	504	[[0,0,0,0,1,0,0,0,0]]505	506,507,508,509,510,511,512,513,514,515,516	[[1,1,0,0,0,0,0,0,0],[0,0,0,1,0,0,0,0,0],[0,0,0,1,1,0,0,0,0],[0,0,0,0,1,0,0,0,0],[0,1,0,0,0,0,0,0,0],[1,1,0,1,0,0,0,0,0],[0,1,0,0,0,0,0,0,0],[0,1,0,0,0,0,0,0,0],[0,1,0,0,0,0,0,0,0],[1,0,0,0,0,0,0,0,0],[1,0,0,0,0,0,0,0,0]]517	518,519,520,521,522	[[0,0,0,0,1,0,0,0,0],[0,0,0,0,1,0,0,0,0],[0,0,0,0,1,0,0,0,0],[0,0,0,0,1,0,0,0,0],[0,0,0,0,1,0,0,0,0]]523	524	[[0,0,0,0,1,0,0,0,0]]525	526,527	[[0,0,0,0,1,0,0,0,0],[0,0,0,0,1,0,0,0,0]]528	529,530,531,532,533,534	[[0,0,0,0,1,0,0,0,0],[0,0,0,0,1,0,0,0,0],[0,0,0,0,1,0,0,0,0],[0,0,0,0,1,0,0,0,0],[0,0,0,0,1,0,0,0,0],[0,0,0,0,1,0,0,0,0]]535	536,537,538,539	[[0,0,0,0,1,0,0,0,0],[0,0,0,0,1,0,0,0,0],[0,1,0,0,0,0,0,0,0],[0,1,0,0,0,0,0,0,0]]540	541	[[0,0,0,0,1,0,0,0,0]]542	543,544	[[0,0,0,0,1,0,0,0,0],[0,0,0,0,1,0,0,0,0]]545	546,547,548	[[0,0,0,0,1,0,0,0,0],[0,0,0,0,1,0,0,0,0],[0,0,0,0,1,0,0,0,0]]549	550,551,552,553,554	[[0,0,0,1,0,0,0,0,0],[0,0,0,1,0,0,0,0,0],[0,0,0,1,0,0,0,0,0],[0,0,0,1,0,0,0,0,0],[0,0,0,1,0,0,0,0,0]]555	556,557	[[0,0,0,0,1,0,0,0,0],[0,0,0,0,1,0,0,0,0]]558	559	[[0,0,0,0,1,0,0,0,0]]560	561,562,563,564,565,566,567,568,569,570,571,572,573,574,575,576,577,578,579,580,581,582,583,584,585,586,587,588,589,590,591,592,593,594,595,596,597,598,599,600,601,602,603,604,605,606,607,608,609,610,611,612,613,614	[[0,0,1,1,0,0,0,0,0],[0,0,1,0,0,0,0,0,0],[0,0,1,0,0,1,0,0,0],[0,0,1,0,0,1,0,0,0],[0,0,1,0,0,0,0,0,0],[0,0,1,0,1,1,0,0,0],[0,0,0,0,1,1,0,0,0],[0,0,1,1,0,0,0,0,0],[0,0,1,0,0,0,0,0,0],[0,0,0,0,1,0,0,0,0],[0,0,1,0,0,0,0,0,0],[0,0,1,0,0,0,0,0,0],[0,0,0,0,1,1,0,0,0],[0,0,1,0,0,0,0,0,0],[0,0,1,0,0,0,0,0,0],[0,0,1,0,1,0,0,0,0],[0,0,1,0,0,1,0,0,0],[0,0,1,0,0,0,0,0,0],[0,0,1,0,0,0,0,0,0],[0,0,0,0,1,0,0,0,0],[0,0,1,0,0,0,0,0,0],[0,0,1,0,0,0,0,0,0],[0,0,1,0,0,0,0,0,0],[0,0,1,0,0,0,0,0,0],[0,0,1,0,0,0,0,0,0],[0,0,0,0,1,0,0,0,0],[0,0,1,0,0,0,0,0,0],[0,0,0,0,0,1,0,0,0],[0,0,1,0,0,0,0,0,0],[0,0,1,0,0,0,0,0,0],[0,0,1,0,0,0,0,0,0],[0,0,1,0,1,0,0,0,0],[0,0,1,0,0,0,0,0,0],[0,0,0,1,0,0,0,0,0],[0,0,1,0,0,0,0,0,0],[0,0,1,0,0,1,0,0,0],[0,0,0,0,1,0,0,0,0],[0,0,1,0,0,0,0,0,0],[0,0,1,0,0,0,0,0,0],[0,0,1,0,0,0,0,0,0],[0,0,1,0,0,0,0,0,0],[0,0,0,0,1,0,0,0,0],[0,0,1,0,0,0,0,0,0],[0,0,1,0,0,0,0,0,0],[0,0,1,0,1,0,0,0,0],[0,0,1,0,0,0,0,0,0],[0,0,1,0,0,0,0,0,0],[0,0,1,0,0,0,0,0,0],[0,0,1,0,0,0,0,0,0],[0,0,1,0,0,1,0,0,0],[0,0,0,0,0,1,0,0,0],[0,0,1,0,1,1,0,0,0],[0,0,1,0,0,0,0,0,0],[0,0,1,0,0,0,0,0,0]]615	616	[[0,0,0,0,1,0,0,0,0]]617	618,619,620	[[0,0,0,1,0,0,0,0,0],[0,0,0,1,0,0,0,0,0],[0,0,0,1,0,0,0,0,0]]621	622,623,537,624,625	[[0,0,0,0,1,0,0,0,0],[0,0,0,0,1,0,0,0,0],[0,0,0,0,1,0,0,0,0],[0,0,0,0,1,0,0,0,0],[0,0,0,0,1,0,0,0,0]]626	627,628	[[0,0,0,0,1,0,0,0,0],[0,0,0,0,1,0,0,0,0]]629	630,631,632,633,634,635,636,637,638,639,640,641,642,643,644,645,646,647,648,649,650,651,652,653,654,655,656,657,658,659,660,661,662,663,664,665,666,667,668,669,670	[[0,1,1,0,0,0,0,0,0],[0,1,0,0,0,0,0,1,0],[0,1,1,0,0,0,1,1,0],[0,1,1,1,1,0,1,1,0],[0,1,1,1,1,0,1,1,0],[0,1,1,1,1,0,1,1,0],[0,1,1,0,1,0,1,1,0],[0,0,1,0,0,0,0,1,0],[0,0,1,0,0,0,0,0,0],[0,1,1,0,0,0,0,0,0],[0,0,1,0,0,0,0,0,0],[0,0,0,0,0,0,0,1,0],[0,1,1,0,0,0,1,1,0],[0,0,1,0,0,0,0,0,0],[0,1,0,0,0,0,0,0,0],[0,0,0,0,1,0,0,1,0],[0,0,1,0,0,0,0,0,0],[0,0,1,0,0,0,0,0,0],[0,0,1,0,0,0,0,0,0],[0,0,1,0,0,0,0,1,0],[0,1,0,0,0,0,1,1,0],[0,0,1,0,0,0,0,0,0],[0,0,0,0,0,0,0,1,0],[0,0,1,0,0,0,0,0,0],[0,0,1,0,0,0,0,0,0],[0,0,1,0,0,0,0,0,0],[0,0,1,0,0,0,0,0,0],[0,0,1,0,0,0,0,0,0],[0,0,1,0,0,0,0,0,0],[0,0,1,0,0,0,0,0,0],[0,0,0,1,0,0,0,1,0],[0,0,1,0,0,0,0,0,0],[0,0,1,0,0,0,0,0,0],[0,0,1,0,0,0,0,0,0],[0,0,1,0,0,0,0,1,0],[0,0,0,0,1,0,0,0,0],[0,0,1,0,0,0,0,0,0],[0,0,1,0,0,0,0,0,0],[0,0,1,0,0,0,0,1,0],[0,0,1,0,0,0,0,0,0],[0,0,1,0,0,0,0,0,0]]671	672,673,18,674	[[0,0,0,0,1,0,0,0,0],[0,0,0,0,1,0,0,0,0],[0,0,0,0,1,0,0,0,0],[0,0,0,0,1,0,0,0,0]]675	676,677,678,679	[[0,0,0,0,1,0,0,0,0],[0,0,0,1,0,0,0,0,0],[0,0,0,1,0,0,0,0,0],[0,0,0,1,0,0,0,0,0]]680	681,682,683	[[0,0,0,0,1,0,0,0,0],[0,0,0,0,1,0,0,0,0],[0,0,0,0,1,0,0,0,0]]684	685,686,687,688,689,690,691,692,693	[[0,1,1,0,0,0,0,0,0],[0,1,1,0,0,0,0,0,0],[0,1,1,0,0,0,0,0,0],[0,1,1,0,1,0,0,0,0],[0,0,0,1,0,0,0,0,0],[0,1,1,0,0,0,0,0,0],[0,0,1,0,0,0,0,0,0],[0,0,1,0,0,0,0,0,0],[0,0,1,1,0,0,0,0,0]]694	695,696,697,698,699,700,701,702,703,704,705,706,707,708,709,710,711,712,713,714,715,716,717,718,719,720,721,722,723,724	[[0,0,1,1,0,0,0,0,0],[0,0,1,0,0,0,0,0,0],[0,0,0,1,0,0,0,0,0],[0,0,1,0,0,0,0,0,0],[0,0,1,0,0,0,0,0,0],[0,0,0,1,0,0,0,0,0],[0,0,1,0,0,0,0,0,0],[0,0,0,1,0,0,0,0,0],[0,0,0,1,0,0,0,0,0],[0,0,1,0,0,0,0,0,0],[0,0,1,0,0,0,0,0,0],[0,0,1,0,0,0,0,0,0],[0,0,0,1,0,0,0,0,0],[0,0,1,0,0,0,0,0,0],[0,0,1,0,0,0,0,0,0],[0,0,1,0,0,0,0,0,0],[0,0,1,1,0,0,0,0,0],[0,0,1,0,0,0,0,0,0],[0,0,1,0,0,0,0,0,0],[0,0,0,1,0,0,0,0,0],[0,0,1,1,0,0,0,0,0],[0,0,1,0,0,0,0,0,0],[0,0,1,0,0,0,0,0,0],[0,0,0,1,0,0,0,0,0],[0,0,1,0,0,0,0,0,0],[0,0,1,0,0,0,0,0,0],[0,0,0,1,0,0,0,0,0],[0,0,1,0,0,0,0,0,0],[0,0,1,0,0,0,0,0,0],[0,0,0,1,0,0,0,0,0]]725	726	[[0,0,0,0,1,0,0,0,0]]727	728,729,730,731,732	[[0,0,0,0,1,0,0,0,0],[0,0,0,0,1,0,0,0,0],[0,0,0,0,1,0,0,0,0],[0,0,0,0,1,0,0,0,0],[0,0,0,0,1,0,0,0,0]]733	734,735,736,737,738,739,740,741,742,743,744,745,413,746,747,748,749,750,751,752,753,754,755,414,756,695,396,757,758,711,759,382,760,761,762,763,764,765,766,767,768,769,770,771,772,773,774,775,707,709,723,776	[[0,1,1,1,1,0,0,0,0],[0,0,1,1,0,0,0,0,0],[0,0,0,1,0,0,0,0,0],[0,0,1,1,0,0,0,0,0],[0,0,1,0,0,0,0,0,0],[0,1,0,0,0,0,0,0,0],[0,1,0,0,0,0,0,0,0],[0,1,1,1,0,0,0,0,0],[0,1,1,0,0,0,0,0,0],[0,1,0,0,0,0,0,0,0],[0,0,0,1,0,0,0,0,0],[0,1,0,0,1,0,0,0,0],[0,1,0,1,0,0,0,0,0],[0,1,0,0,0,0,0,0,0],[0,0,0,1,0,0,0,0,0],[0,1,1,1,0,0,0,0,0],[0,1,1,1,0,0,0,0,0],[0,1,1,1,0,0,0,0,0],[0,1,1,1,1,0,0,0,0],[0,1,1,1,1,0,0,0,0],[0,0,1,0,0,0,0,0,0],[0,0,1,0,0,0,0,0,0],[0,0,0,1,0,0,0,0,0],[0,0,0,1,0,0,0,0,0],[0,0,0,1,0,0,0,0,0],[0,0,1,0,0,0,0,0,0],[0,0,0,1,0,0,0,0,0],[0,1,0,0,0,0,0,0,0],[0,0,0,1,0,0,0,0,0],[0,0,1,0,0,0,0,0,0],[0,0,1,0,0,0,0,0,0],[0,0,0,1,0,0,0,0,0],[0,1,0,0,0,0,0,0,0],[0,0,1,0,0,0,0,0,0],[0,0,1,0,0,0,0,0,0],[0,1,1,0,0,0,0,0,0],[0,1,0,1,0,0,0,0,0],[0,1,1,0,0,0,0,0,0],[0,1,1,1,0,0,0,0,0],[0,0,0,1,0,0,0,0,0],[0,0,1,0,0,0,0,0,0],[0,0,0,1,0,0,0,0,0],[0,0,1,0,0,0,0,0,0],[0,1,0,0,0,0,0,0,0],[0,0,1,1,0,0,0,0,0],[0,0,0,1,0,0,0,0,0],[0,0,1,0,0,0,0,0,0],[0,0,0,1,0,0,0,0,0],[0,0,1,0,0,0,0,0,0],[0,0,1,0,0,0,0,0,0],[0,0,1,0,0,0,0,0,0],[0,0,0,1,0,0,0,0,0]]777	778,779,780,781,782	[[0,0,0,0,1,0,0,0,0],[0,0,0,0,1,0,0,0,0],[0,0,0,0,1,0,0,0,0],[0,0,0,0,1,0,0,0,0],[0,0,0,0,1,0,0,0,0]]783	784	[[0,0,0,0,1,0,0,0,0]]785	634,633,660,635	[[0,0,0,1,0,0,0,0,0],[0,0,0,1,0,0,0,0,0],[0,0,0,1,0,0,0,0,0],[0,0,0,1,0,0,0,0,0]]786	787,788,789,790,791,792,793,794,795,796,797,798,799,800,801,802,803,804,805,806,807,808,418,809,810,811,812,813,814,815,816,817,818,819,820,821,822,823,824,825,826,827,828	[[0,0,1,0,0,0,0,0,0],[0,0,1,1,1,0,1,1,0],[0,0,0,0,1,0,0,0,0],[0,0,0,0,0,0,1,0,0],[0,0,1,0,0,0,0,0,0],[0,0,1,0,0,0,0,0,0],[0,0,1,0,0,0,0,1,0],[0,0,0,0,0,0,0,1,0],[0,0,1,0,0,0,0,0,0],[0,0,1,1,0,0,1,1,0],[0,0,1,0,0,0,1,1,0],[0,0,1,0,0,0,0,0,0],[0,0,1,0,0,0,0,0,0],[0,0,1,0,0,0,0,0,0],[0,0,1,0,0,0,0,0,0],[0,0,1,1,0,0,1,1,0],[0,0,1,0,0,0,0,0,0],[0,0,1,0,0,0,0,0,0],[0,0,0,0,1,0,0,0,0],[0,0,1,0,0,0,0,0,0],[0,0,0,0,0,0,0,1,0],[0,0,1,0,0,0,0,0,0],[0,0,1,0,0,0,0,0,0],[0,0,0,0,0,0,0,1,0],[0,0,1,0,0,0,0,0,0],[0,0,1,0,0,0,0,1,0],[0,0,1,0,0,0,0,0,0],[0,0,1,0,0,0,0,0,0],[0,0,1,0,0,0,0,0,0],[0,0,1,0,0,0,0,0,0],[0,0,1,0,0,0,0,0,0],[0,0,1,1,1,0,1,1,0],[0,0,1,0,0,0,0,0,0],[0,0,0,0,0,0,0,1,0],[0,0,1,0,0,0,0,0,0],[0,0,1,0,0,0,0,0,0],[0,0,1,0,0,0,0,0,0],[0,0,1,0,0,0,0,0,0],[0,0,0,0,0,0,1,0,0],[0,0,1,0,0,0,1,1,0],[0,0,1,0,0,0,0,0,0],[0,0,1,0,0,0,0,1,0],[0,0,1,0,0,0,0,0,0]]829	830,831	[[0,0,0,0,1,0,0,0,0],[0,0,0,0,1,0,0,0,0]]832	833,834,835	[[0,0,0,0,1,0,0,0,0],[0,0,0,0,1,0,0,0,0],[0,0,0,0,1,0,0,0,0]]836	837,838,839,840,841,842,843,844,845,846,847,848,849,850,851,852,853,854,855,856,857,858,859,860,861,862,863,864,865,866,867,868,869,870,871,872,873,874,875,876,877,878,879,880,881,882,883,884,885,886,887,888,889,890,891,892,893,894,895,896,897,898,899,900,901,902,903,904,905,906,907,908,909,910,911,912,913,914,915,916,917,918,919,920,921,922,923,924,925,926,927,928,929,930,931,932,933,934,935,936,937,938,939,940,941,942,943,944,945,946	[[0,0,1,0,0,0,0,0,0],[0,0,1,0,0,0,0,0,0],[0,0,1,0,0,0,0,0,0],[0,0,1,0,0,0,0,0,0],[0,0,1,0,0,0,0,0,0],[0,0,1,0,0,0,0,0,0],[0,0,1,0,0,0,0,0,0],[0,1,1,1,1,0,0,0,1],[0,0,1,0,0,0,0,0,0],[0,0,1,0,0,0,0,0,0],[0,1,1,0,0,0,0,0,0],[0,1,1,0,0,0,0,0,0],[0,1,1,0,0,0,0,0,0],[0,1,1,0,0,0,0,0,0],[0,0,1,0,0,0,0,0,0],[0,0,1,0,0,0,0,0,0],[0,0,1,0,0,0,0,0,0],[0,1,1,0,0,1,0,0,0],[0,0,1,0,0,0,0,0,0],[0,0,1,0,0,0,0,0,0],[0,0,1,0,0,0,0,0,1],[0,0,1,0,0,0,0,0,1],[0,0,1,0,0,0,0,0,0],[0,1,1,0,1,1,0,0,1],[0,0,1,0,0,0,0,0,0],[0,0,1,0,0,0,0,0,0],[0,0,1,0,0,0,0,0,0],[0,0,1,0,0,0,0,0,0],[0,0,1,0,0,0,0,0,0],[0,1,1,0,0,0,0,0,0],[0,1,1,0,1,1,0,0,1],[0,0,1,0,0,0,0,0,0],[0,0,1,0,0,0,0,0,0],[0,0,1,0,0,0,0,0,0],[0,0,1,0,0,0,0,0,0],[0,0,1,0,0,0,0,0,0],[0,0,1,0,0,0,0,0,0],[0,0,1,0,0,0,0,0,0],[0,0,1,0,0,0,0,0,0],[0,0,1,0,0,0,0,0,0],[0,0,1,0,0,0,0,0,0],[0,1,1,0,0,1,0,0,1],[0,0,1,0,0,0,0,0,0],[0,0,1,0,0,0,0,0,0],[0,0,1,0,0,0,0,0,0],[0,0,1,0,0,0,0,0,0],[0,0,1,0,0,0,0,0,0],[0,0,1,0,0,0,0,0,0],[0,0,1,0,0,0,0,0,0],[0,0,1,0,0,0,0,0,0],[0,0,1,0,0,1,0,0,0],[0,0,1,0,0,0,0,0,0],[0,0,1,0,0,0,0,0,0],[0,0,1,0,0,0,0,0,0],[0,0,1,0,0,0,0,0,0],[0,0,1,0,0,0,0,0,0],[0,0,1,0,0,0,0,0,0],[0,0,1,0,0,0,0,0,0],[0,1,0,1,0,0,0,0,1],[0,0,1,0,0,0,0,0,0],[0,0,1,0,0,0,0,0,0],[0,0,1,0,0,0,0,0,0],[0,0,1,0,0,0,0,0,0],[0,0,1,0,0,0,0,0,0],[0,0,1,0,0,0,0,0,0],[0,0,1,0,0,0,0,0,0],[0,0,1,0,0,0,0,0,0],[0,0,1,0,0,0,0,0,0],[0,0,1,0,0,0,0,0,0],[0,0,1,0,0,0,0,0,0],[0,0,1,0,0,0,0,0,0],[0,1,1,0,1,1,0,0,1],[0,0,1,0,0,0,0,0,0],[0,0,1,0,0,0,0,0,1],[0,0,1,0,0,0,0,0,0],[0,1,1,1,1,1,0,0,1],[0,0,1,0,0,0,0,0,0],[0,0,1,0,0,0,0,0,0],[0,0,1,0,0,0,0,0,1],[0,0,1,0,0,0,0,0,0],[0,0,1,0,0,0,0,0,0],[0,0,1,0,0,0,0,0,0],[0,0,1,0,0,0,0,0,0],[0,0,1,0,0,0,0,0,0],[0,0,0,0,0,1,0,0,0],[0,0,1,0,0,0,0,0,0],[0,0,1,0,0,0,0,0,0],[0,0,1,0,0,0,0,0,0],[0,0,1,0,0,1,0,0,0],[0,0,1,0,0,0,0,0,0],[0,0,1,0,0,0,0,0,0],[0,0,1,0,0,0,0,0,0],[0,0,1,0,0,0,0,0,0],[0,0,1,0,0,0,0,0,0],[0,0,1,0,0,0,0,0,0],[0,0,1,0,0,0,0,0,0],[0,0,1,0,0,0,0,0,0],[0,0,1,0,0,0,0,0,0],[0,0,1,0,0,0,0,0,0],[0,0,1,0,0,0,0,0,0],[0,0,1,0,0,0,0,0,0],[0,0,1,0,0,0,0,0,0],[0,0,1,0,0,0,0,0,0],[0,0,1,0,0,0,0,0,0],[0,0,1,0,0,0,0,0,0],[0,0,1,0,0,0,0,0,0],[0,0,1,0,0,0,0,0,0],[0,0,1,0,0,0,0,0,0],[0,0,1,0,0,0,0,0,0],[0,0,1,0,0,0,0,0,0]]947	831,830	[[0,0,0,0,1,0,0,0,0],[0,0,0,0,1,0,0,0,0]]948	949,950,951	[[0,0,0,1,0,0,0,0,0],[0,0,0,1,0,0,0,0,0],[0,0,0,1,0,0,0,0,0]]952	953,954,955,956,957,958,959,960,961,962,963,964,965,966,967,968,969	[[0,0,0,1,0,0,0,0,0],[0,0,1,1,0,0,0,0,0],[0,0,1,0,0,0,0,0,0],[0,1,0,1,0,0,0,0,0],[0,0,0,0,1,0,0,0,0],[0,1,1,0,0,0,0,0,0],[0,1,1,0,0,0,0,0,0],[0,0,0,0,1,0,0,0,0],[0,1,1,0,1,0,0,0,0],[0,0,1,0,0,0,0,0,0],[0,0,1,0,0,0,0,0,0],[0,0,0,0,1,0,0,0,0],[0,0,1,0,0,0,0,0,0],[0,0,1,0,0,0,0,0,0],[0,0,0,1,0,0,0,0,0],[0,0,1,0,0,0,0,0,0],[0,0,1,0,0,0,0,0,0]]970	971,972,973,974	[[0,0,0,1,0,0,0,0,0],[0,0,0,1,0,0,0,0,0],[0,0,0,1,0,0,0,0,0],[0,0,0,1,0,0,0,0,0]]975	976,977,978,979,980,981,982,983,944,984,985,986,987,988,989,990,991,992,993,994,995,996,997,998	[[0,0,1,0,0,0,0,0,1],[0,0,0,0,0,0,0,0,1],[0,0,1,0,0,0,0,0,0],[0,0,1,0,0,0,0,0,1],[1,1,1,0,1,0,0,0,0],[0,1,1,0,0,0,0,0,1],[0,0,1,0,0,0,0,0,0],[0,0,0,0,0,0,0,0,1],[0,0,0,0,0,0,0,0,1],[0,0,1,0,0,0,0,0,0],[0,0,1,0,0,0,0,0,1],[0,1,1,0,0,0,0,0,0],[0,0,0,0,0,0,0,0,1],[1,1,0,0,0,0,0,0,0],[1,1,0,0,0,0,0,0,0],[0,1,0,0,0,0,0,0,0],[0,1,1,0,0,0,0,0,0],[1,0,0,0,0,0,0,0,0],[1,0,0,0,0,0,0,0,0],[0,0,0,0,0,0,0,0,1],[1,0,0,0,0,0,0,0,0],[0,0,0,0,0,0,0,0,1],[1,0,0,0,0,0,0,0,0],[1,0,0,0,0,0,0,0,0]]999	1000,1001,1002,1003,1004,1005,1006,1007,1008,1009,1010	[[0,0,0,1,0,0,0,1,0],[0,0,0,1,0,0,0,1,0],[0,0,0,0,1,0,0,1,0],[0,0,0,0,0,0,0,1,0],[0,0,0,1,1,0,0,1,0],[0,0,0,1,0,0,0,1,0],[0,0,0,0,0,0,0,1,0],[0,0,0,0,0,0,0,1,0],[0,0,0,0,0,0,0,1,0],[0,0,0,0,0,0,0,1,0],[0,0,0,0,0,0,0,1,0]]1011	1012	[[0,0,0,0,1,0,0,0,0]]1013	1014	[[0,0,0,0,1,0,0,0,0]]1015	1016,1017,1018,1019,1020,1021,1022,1023,1024	[[0,0,1,0,0,0,0,0,0],[0,0,1,0,0,0,0,0,0],[0,0,1,0,0,0,0,0,0],[0,0,1,0,0,0,0,0,0],[0,0,1,0,0,0,0,0,0],[0,0,1,0,0,0,0,0,0],[0,0,1,0,0,0,0,0,0],[0,0,1,0,0,0,0,0,0],[0,0,1,0,0,0,0,0,0]]1025	1026	[[0,0,0,0,1,0,0,0,0]]1027	1028,1029,1030,1031,647,1032	[[0,0,0,1,0,0,0,1,0],[0,0,0,0,0,0,0,1,0],[0,0,0,1,0,0,0,1,0],[0,0,0,0,0,0,0,1,0],[0,0,0,1,0,0,0,1,0],[0,0,0,0,0,0,0,1,0]]1033	972,973,971,974	[[0,0,0,1,0,0,0,0,0],[0,0,0,1,0,0,0,0,0],[0,0,0,1,0,0,0,0,0],[0,0,0,1,0,0,0,0,0]]1034	1035	[[0,0,0,0,1,0,0,0,0]]1036	1004,1000,1001,1005	[[0,0,0,1,0,0,0,0,0],[0,0,0,1,0,0,0,0,0],[0,0,0,1,0,0,0,0,0],[0,0,0,1,0,0,0,0,0]]1037	1038,909	[[0,0,0,0,1,0,0,0,0],[0,0,0,0,1,0,0,0,0]]1039	1040,1041,1042	[[0,0,0,0,1,0,0,0,0],[0,0,0,0,1,0,0,0,0],[0,0,0,0,1,0,0,0,0]]1043	1044,1045,1046,1047,1048,1049,1050,1051,1052,1053,1054,1055,1056,1057,1058,1059,1060,1061,1062,1063,1064,1065,1066,1067,1068,1069,1070,1071,1072,1073,1074,1075,1076,1077,1078,1079,1080,1081,1082,1083,1084,1085,1086,1087,1088,1089,1090,1091,1092,1093,1094,1095,1096,1097,1098,1099,1100,1101,1102,1103,1104,1105,1106,1107,1108,1109,1110	[[0,0,1,0,0,0,0,0,0],[0,0,1,0,0,0,0,0,0],[0,1,1,1,1,0,0,0,0],[0,1,0,0,0,0,0,0,0],[0,0,1,0,0,0,0,0,0],[0,1,1,0,0,0,0,0,0],[0,0,1,0,0,0,0,0,0],[0,0,1,0,0,0,0,0,0],[0,0,1,0,0,0,0,0,0],[0,0,1,0,0,0,0,0,0],[0,0,1,0,0,0,0,0,0],[0,0,0,0,1,0,0,0,0],[0,0,1,0,0,0,0,0,0],[0,0,1,0,0,0,0,0,0],[0,0,1,0,0,0,0,0,0],[0,1,1,0,1,0,0,0,0],[0,1,1,0,0,0,0,0,0],[0,1,0,0,0,0,0,0,0],[0,1,0,0,0,0,0,0,0],[0,1,1,0,0,0,0,0,0],[0,1,1,0,1,0,0,0,0],[0,0,1,0,0,0,0,0,0],[0,1,0,0,0,0,0,0,0],[0,0,1,0,0,0,0,0,0],[0,0,1,1,0,0,0,0,0],[0,1,1,0,0,0,0,0,0],[0,0,1,0,0,0,0,0,0],[0,0,1,0,0,0,0,0,0],[0,0,1,0,0,0,0,0,0],[0,0,1,0,0,0,0,0,0],[0,0,1,0,0,0,0,0,0],[0,0,1,0,1,0,0,0,0],[0,0,1,0,0,0,0,0,0],[0,0,0,0,1,0,0,0,0],[0,0,1,0,0,0,0,0,0],[0,0,1,0,0,0,0,0,0],[0,0,1,0,0,0,0,0,0],[0,0,1,0,0,0,0,0,0],[0,0,1,0,0,0,0,0,0],[0,0,1,0,0,0,0,0,0],[0,0,1,0,0,0,0,0,0],[0,0,1,0,0,0,0,0,0],[0,0,1,0,0,0,0,0,0],[0,0,1,0,0,0,0,0,0],[0,0,1,1,0,0,0,0,0],[0,0,1,0,0,0,0,0,0],[0,0,1,0,0,0,0,0,0],[0,0,1,0,0,0,0,0,0],[0,0,1,0,0,0,0,0,0],[0,0,1,0,0,0,0,0,0],[0,0,0,1,0,0,0,0,0],[0,0,1,0,0,0,0,0,0],[0,0,0,1,0,0,0,0,0],[0,0,0,0,1,0,0,0,0],[0,0,1,0,0,0,0,0,0],[0,0,1,0,0,0,0,0,0],[0,0,1,0,0,0,0,0,0],[0,0,1,0,0,0,0,0,0],[0,0,1,1,0,0,0,0,0],[0,1,0,0,0,0,0,0,0],[0,0,1,0,1,0,0,0,0],[0,1,0,0,0,0,0,0,0],[0,1,0,0,0,0,0,0,0],[0,0,1,0,0,0,0,0,0],[0,0,1,0,0,0,0,0,0],[0,0,1,0,0,0,0,0,0],[0,0,1,0,0,0,0,0,0]]1111	1112,1113,1114,1115,1116,1117	[[0,0,0,0,1,0,0,0,0],[0,0,0,0,1,0,0,0,0],[0,0,0,0,1,0,0,0,0],[0,0,0,0,1,0,0,0,0],[0,0,0,0,1,0,0,0,0],[0,0,0,0,1,0,0,0,0]]1118	1119,1120,1121,1122	[[0,0,0,0,1,0,0,0,0],[0,0,0,0,1,0,0,0,0],[0,0,0,0,1,0,0,0,0],[0,0,0,0,1,0,0,0,0]]1123	1124	[[0,0,0,0,1,0,0,0,0]]1125	983,977,976,982,978,981,1126,985,1127,979,988,980,1128,1129,996,1130,984,1131,1132	[[0,0,0,1,0,0,0,0,0],[0,0,0,1,0,0,0,0,0],[0,0,0,1,0,0,0,0,0],[0,0,0,1,0,0,0,0,0],[0,0,0,1,0,0,0,0,0],[0,0,0,1,0,0,0,0,0],[0,0,0,1,0,0,0,0,0],[0,0,0,1,0,0,0,0,0],[0,0,0,1,0,0,0,0,0],[0,0,0,1,0,0,0,0,0],[0,0,0,1,0,0,0,0,0],[0,0,0,1,0,0,0,0,0],[0,0,0,1,0,0,0,0,0],[0,0,0,1,0,0,0,0,0],[0,0,0,1,0,0,0,0,0],[0,0,0,1,0,0,0,0,0],[0,0,0,1,0,0,0,0,0],[0,0,0,1,0,0,0,0,0],[0,0,0,1,0,0,0,0,0]]1133	1134,1135,1136,1137,1138,1139,1140,1141,794,1142,1143,1144,1145,1146,1147,1148,1149,1150,1151,1152,1153,1154,1155,1156,1157,1158,1159,1160,1161,1162,1163,828,1164,1165,1166,1167,821,807,1168,825,1169,814,1170,1171,1172,1173,1174,1175,1176,1177,1178,1179,1180,1181,1182,1183,1184,1185,1186,1187,1188,1189,1190,1191,1192,1193,1194	[[0,1,1,1,1,0,0,0,0],[0,0,1,0,0,0,0,0,0],[0,0,1,0,0,0,0,0,0],[0,1,1,0,0,0,0,0,0],[0,0,0,1,0,0,0,0,0],[0,1,1,0,0,0,0,0,0],[0,1,0,0,0,0,0,0,0],[0,1,1,0,0,0,0,0,0],[0,1,1,0,0,0,0,0,0],[0,1,0,0,0,0,0,0,0],[0,1,0,0,0,0,0,0,0],[0,1,1,1,1,0,0,0,0],[0,1,1,0,0,0,0,0,0],[0,0,1,0,0,0,0,0,0],[0,1,1,1,0,0,0,0,0],[0,0,1,0,0,0,0,0,0],[0,0,1,0,0,0,0,0,0],[0,0,1,0,0,0,0,0,0],[0,1,0,0,0,0,0,0,0],[0,0,0,1,0,0,0,0,0],[0,1,0,1,0,0,0,0,0],[0,0,0,1,0,0,0,0,0],[0,0,1,0,0,0,0,0,0],[0,0,1,0,0,0,0,0,0],[0,0,1,0,0,0,0,0,0],[0,0,1,0,0,0,0,0,0],[0,0,1,0,0,0,0,0,0],[0,0,1,0,0,0,0,0,0],[0,0,1,0,0,0,0,0,0],[0,0,1,1,1,0,0,0,0],[0,0,1,0,0,0,0,0,0],[0,0,1,0,0,0,0,0,0],[0,1,0,0,0,0,0,0,0],[0,0,1,0,0,0,0,0,0],[0,0,1,0,0,0,0,0,0],[0,0,1,0,0,0,0,0,0],[0,0,1,0,0,0,0,0,0],[0,0,1,0,0,0,0,0,0],[0,0,1,0,0,0,0,0,0],[0,0,1,0,0,0,0,0,0],[0,0,0,1,0,0,0,0,0],[0,0,1,0,0,0,0,0,0],[0,0,1,0,0,0,0,0,0],[0,0,1,0,0,0,0,0,0],[0,0,1,0,0,0,0,0,0],[0,0,1,0,0,0,0,0,0],[0,0,1,0,0,0,0,0,0],[0,0,0,1,0,0,0,0,0],[0,0,1,0,0,0,0,0,0],[0,0,1,0,0,0,0,0,0],[0,0,1,0,0,0,0,0,0],[0,0,1,0,0,0,0,0,0],[0,0,1,1,0,0,0,0,0],[0,0,1,0,0,0,0,0,0],[0,0,1,0,0,0,0,0,0],[0,0,0,1,0,0,0,0,0],[0,0,0,1,1,0,0,0,0],[0,0,0,1,0,0,0,0,0],[0,0,1,0,0,0,0,0,0],[0,0,0,1,0,0,0,0,0],[0,0,1,0,0,0,0,0,0],[0,0,1,0,0,0,0,0,0],[0,0,1,0,0,0,0,0,0],[0,0,1,1,0,0,0,0,0],[0,0,1,1,0,0,0,0,0],[0,0,1,0,0,0,0,0,0],[0,0,1,0,0,0,0,0,0]]1195	1196,1197	[[0,0,0,1,0,0,0,0,0],[0,0,0,1,0,0,0,0,0]]1198	1199,1200,1201	[[0,0,0,1,0,0,0,0,0],[0,0,0,1,0,0,0,0,0],[0,0,0,1,0,0,0,0,0]]1202	1203,1204,1205,1206,1207	[[0,0,0,1,0,0,0,0,0],[0,0,0,0,1,0,0,0,0],[0,0,0,1,0,0,0,0,0],[0,0,0,1,0,0,0,0,0],[0,0,0,1,0,0,0,0,0]]1208	1209,1210,1211,1212,1213,1214,1215,1216,1217,1218,1219,1220,1221,1222,1097,1223,1224,1225,1226,1227,1228,1229,1230,1231,1232,1233,1234,1235,1236,1237,1238,1239,1240,1241,1242,1243,1244,1245	[[0,1,0,0,0,0,0,0,0],[0,1,0,0,0,0,0,0,0],[0,0,0,1,0,0,0,0,0],[0,0,0,1,0,0,0,0,0],[0,1,0,0,0,0,0,0,0],[0,1,0,1,0,0,0,0,0],[0,1,0,1,0,0,0,0,0],[0,1,0,1,0,0,0,0,0],[0,1,0,0,0,0,0,0,0],[0,1,0,0,0,0,0,0,0],[0,1,0,0,0,0,0,0,0],[0,1,0,0,0,0,0,0,0],[0,1,0,0,0,0,0,0,0],[0,1,0,0,0,0,0,0,0],[0,1,0,0,0,0,0,0,0],[0,1,0,0,1,0,0,0,0],[0,1,0,0,0,0,0,0,0],[0,1,0,0,0,0,0,0,0],[0,1,0,0,0,0,0,0,0],[0,1,0,1,0,0,0,0,0],[0,1,0,1,0,0,0,0,0],[0,1,0,0,0,0,0,0,0],[0,1,0,0,0,0,0,0,0],[0,0,0,1,0,0,0,0,0],[0,0,0,1,0,0,0,0,0],[0,0,0,1,0,0,0,0,0],[0,0,0,1,0,0,0,0,0],[0,0,0,1,0,0,0,0,0],[0,0,0,1,0,0,0,0,0],[0,0,0,1,0,0,0,0,0],[0,0,0,1,0,0,0,0,0],[0,0,0,1,0,0,0,0,0],[0,0,0,1,1,0,0,0,0],[0,0,0,1,0,0,0,0,0],[0,0,0,1,0,0,0,0,0],[0,0,0,1,0,0,0,0,0],[0,0,0,1,0,0,0,0,0],[0,0,0,1,0,0,0,0,0]]1246	1247,1248,1249,1250,1251,1252,1253,1254,1255,1256,1257,1258,1259	[[0,0,1,1,0,0,0,0,0],[0,0,1,0,0,0,0,0,0],[0,0,1,1,0,0,0,0,0],[0,0,1,0,0,0,0,0,0],[0,0,1,0,0,0,0,0,0],[0,0,1,0,0,0,0,0,0],[0,0,1,0,0,0,0,0,0],[0,0,1,0,0,0,0,0,0],[0,0,1,0,0,0,0,0,0],[0,0,1,0,1,0,0,0,0],[0,0,1,0,0,0,0,0,0],[0,0,1,0,0,0,0,0,0],[0,0,1,1,0,0,0,0,0]]1260	1261,1262,1263,1264,1265	[[0,0,0,1,0,0,0,0,0],[0,0,0,1,0,0,0,0,0],[0,0,0,1,0,0,0,0,0],[0,0,0,1,0,0,0,0,0],[0,0,0,1,0,0,0,0,0]]1266	1267	[[0,0,0,0,1,0,0,0,0]]1268	1269	[[0,0,0,0,1,0,0,0,0]]1270	1271,1201,1272,1200,1199	[[0,0,0,0,1,0,0,0,0],[0,0,0,1,0,0,0,0,0],[0,0,0,0,1,0,0,0,0],[0,0,0,1,0,0,0,0,0],[0,0,0,1,0,0,0,0,0]]1273	1196,1197	[[0,0,0,1,0,0,0,0,0],[0,0,0,1,0,0,0,0,0]]1274	1275,1276,1277,1278,668,1279,1280,1281,1282,1283,1284,1285,1286,1287	[[0,0,0,0,0,0,0,1,0],[0,0,0,0,0,0,0,1,0],[0,0,0,0,0,0,0,1,0],[0,0,0,0,1,0,0,0,0],[0,0,0,0,0,0,0,1,0],[0,0,0,0,0,0,0,1,0],[0,0,0,0,0,0,0,1,0],[0,0,0,0,0,0,0,1,0],[0,0,0,0,1,0,0,0,0],[0,0,0,0,1,0,0,1,0],[0,0,0,0,1,0,0,1,0],[0,0,0,0,1,0,0,1,0],[0,0,0,0,0,0,0,1,0],[0,0,0,0,1,0,0,0,0]]1288	1215,1243,1211,1236,1233,1231,1242,1234,1212,1235,1244,1228,1214,1241,1232,1216,1237,1245,1239,1227,1240,1238	[[0,0,0,1,0,0,0,0,0],[0,0,0,1,0,0,0,0,0],[0,0,0,1,0,0,0,0,0],[0,0,0,1,0,0,0,0,0],[0,0,0,1,0,0,0,0,0],[0,0,0,1,0,0,0,0,0],[0,0,0,1,0,0,0,0,0],[0,0,0,1,0,0,0,0,0],[0,0,0,1,0,0,0,0,0],[0,0,0,1,0,0,0,0,0],[0,0,0,1,0,0,0,0,0],[0,0,0,1,0,0,0,0,0],[0,0,0,1,0,0,0,0,0],[0,0,0,1,0,0,0,0,0],[0,0,0,1,0,0,0,0,0],[0,0,0,1,0,0,0,0,0],[0,0,0,1,0,0,0,0,0],[0,0,0,1,0,0,0,0,0],[0,0,0,1,0,0,0,0,0],[0,0,0,1,0,0,0,0,0],[0,0,0,1,0,0,0,0,0],[0,0,0,1,0,0,0,0,0]]1289	1290,1291,1292,1293,1294,1295	[[0,0,0,0,1,0,0,0,0],[0,0,0,0,1,0,0,0,0],[0,0,0,0,1,0,0,0,0],[0,0,0,0,1,0,0,0,0],[0,0,0,0,1,0,0,0,0],[0,0,0,0,1,0,0,0,0]]1296	1297,1298,1299,1300,1301,1302,1303,1304,1305,1306,1307,1308,1309,130,1310,1311,1312,1313,132,1314,1315,1316,1317,26,1318,1319	[[0,1,1,1,1,0,0,0,0],[0,1,0,0,0,0,0,0,0],[0,1,0,0,0,0,0,0,0],[0,1,1,0,0,0,0,0,0],[0,1,1,0,0,0,0,0,0],[0,0,1,0,0,0,0,0,0],[0,0,1,0,0,0,0,0,0],[0,1,1,0,0,0,0,0,0],[0,0,1,0,0,0,0,0,0],[0,0,1,0,0,0,0,0,0],[0,0,1,0,0,0,0,0,0],[0,0,1,0,0,0,0,0,0],[0,0,0,1,0,0,0,0,0],[0,1,0,0,0,0,0,0,0],[0,1,0,0,0,0,0,0,0],[0,0,1,0,0,0,0,0,0],[0,1,1,1,0,0,0,0,0],[0,1,1,0,0,0,0,0,0],[0,1,1,1,0,0,0,0,0],[0,1,1,0,0,0,0,0,0],[0,1,1,0,0,0,0,0,0],[0,0,1,0,0,0,0,0,0],[0,1,1,0,0,0,0,0,0],[0,1,1,0,0,0,0,0,0],[0,0,1,0,0,0,0,0,0],[0,0,1,0,0,0,0,0,0]]1320	1321,1322	[[0,0,0,0,1,0,0,0,0],[0,0,0,0,1,0,0,0,0]]1323	1324	[[0,0,0,0,1,0,0,0,0]]1325	1326,1327	[[0,0,0,1,0,0,0,0,0],[0,0,0,1,0,0,0,0,0]]1328	1196,1329,1330,1331,1332,1333,1334,1335,1336,1337,1338,1339,1060,1340,1341,1342,1343,1344,1345,1346,1347,1348,1349,1350,1351,1352,1353,1354,1355,1356,1357,1358,1359,1360,1361,285,1362,1363,1364,1365,1366,1367,1368,1057,1369,1370,1371,1372,1373,732,1374,1375,1376,1377,1378,1379,1380,1381,1382,1383,1384,1385,1386,1073,1387,1388,1389,1390,1391,1392,1393,1394,1395,1396,1397,1398,1399,1400,1401,1402,1403,1404,1405,1406,1407,1408,1409,433,1410	[[0,1,1,1,0,0,1,1,0],[0,1,0,0,0,0,1,0,0],[0,0,1,0,0,0,0,0,0],[1,1,1,1,0,0,0,1,0],[0,1,1,0,0,0,1,1,0],[1,1,1,1,0,0,1,1,0],[1,1,1,1,0,0,1,1,0],[1,0,0,0,0,0,0,0,0],[0,0,1,0,0,0,0,1,0],[0,0,1,0,0,0,0,1,0],[1,0,1,0,0,0,0,0,0],[0,0,0,0,0,0,0,1,0],[0,0,0,1,0,0,0,1,0],[0,0,1,0,0,0,0,0,0],[0,1,1,0,0,0,1,0,0],[0,0,0,1,0,0,0,0,0],[0,0,1,0,0,0,0,0,0],[0,0,1,0,0,0,0,0,0],[0,0,0,1,0,0,0,0,0],[0,0,0,1,0,0,0,1,0],[0,1,0,1,0,0,0,0,0],[0,0,0,1,0,0,0,0,0],[0,0,1,0,0,0,0,0,0],[0,1,0,1,0,0,0,0,0],[0,0,1,0,0,0,0,0,0],[1,0,0,0,0,0,0,0,0],[1,0,0,0,0,0,0,0,0],[1,0,0,0,0,0,0,0,0],[1,0,0,0,0,0,0,0,0],[1,0,0,0,0,0,0,0,0],[1,0,0,0,0,0,0,0,0],[1,0,0,0,0,0,0,0,0],[1,0,0,0,0,0,0,0,0],[1,0,0,0,0,0,0,0,0],[1,0,0,0,0,0,0,0,0],[0,0,1,0,0,0,0,0,0],[0,0,0,1,0,0,0,0,0],[1,0,0,0,0,0,0,0,0],[1,0,0,0,0,0,0,0,0],[1,0,0,0,0,0,0,0,0],[1,0,0,0,0,0,0,0,0],[0,0,1,0,0,0,0,0,0],[1,0,0,0,0,0,0,0,0],[1,0,0,0,0,0,0,0,0],[1,0,0,0,0,0,0,0,0],[1,1,1,0,0,0,1,1,0],[0,0,0,0,0,0,0,1,0],[1,0,0,0,0,0,0,0,0],[1,0,0,0,0,0,0,0,0],[1,0,1,0,0,0,0,0,0],[1,0,0,0,0,0,0,0,0],[1,1,1,1,0,0,1,1,0],[0,0,1,0,0,0,0,0,0],[0,0,1,0,0,0,0,0,0],[0,1,1,0,0,0,1,1,0],[0,1,1,0,0,0,1,0,0],[0,1,0,0,0,0,1,0,0],[0,1,0,0,0,0,1,0,0],[0,1,1,0,0,0,1,0,0],[0,0,1,0,0,0,0,0,0],[0,1,1,0,0,0,1,0,0],[0,0,1,0,0,0,0,0,0],[0,0,1,0,0,0,0,0,0],[0,1,0,0,0,0,0,0,0],[0,1,1,1,0,0,0,1,0],[0,0,1,0,0,0,0,0,0],[0,0,1,0,0,0,0,0,0],[0,1,1,0,0,0,0,0,0],[0,0,1,0,0,0,0,0,0],[0,0,0,1,0,0,0,1,0],[0,0,1,0,0,0,0,0,0],[0,0,1,0,0,0,0,0,0],[0,0,0,0,0,0,0,1,0],[0,0,1,0,0,0,0,0,0],[0,0,0,0,0,0,0,1,0],[0,0,0,1,0,0,0,0,0],[0,0,1,0,0,0,0,0,0],[0,0,1,0,0,0,0,0,0],[0,0,1,0,0,0,0,0,0],[0,0,1,0,0,0,0,0,0],[0,0,0,1,0,0,0,0,0],[0,0,1,0,0,0,0,0,0],[0,0,0,1,0,0,0,0,0],[0,0,1,0,0,0,0,0,0],[0,0,1,0,0,0,0,0,0],[0,0,1,0,0,0,0,0,0],[0,0,1,0,0,0,0,0,0],[0,0,0,0,0,0,1,0,0],[0,0,0,1,0,0,0,0,0]]1411	1412	[[0,0,0,0,1,0,0,0,0]]1413	1414,1415,1416,1417,1418,1419,1420,1421,1422,1423,1424,1425,1426,1427,1428	[[0,0,1,1,0,0,0,0,0],[0,0,1,0,0,0,0,0,0],[0,0,1,0,0,0,0,0,0],[0,0,1,0,0,0,0,0,0],[0,0,1,1,0,0,0,0,0],[0,0,1,1,0,0,0,0,0],[0,0,1,0,0,0,0,0,0],[0,0,0,1,0,0,0,0,0],[0,0,1,0,0,0,0,0,0],[0,0,1,0,0,0,0,0,0],[0,0,1,0,0,0,0,0,0],[0,0,0,1,0,0,0,0,0],[0,0,0,0,1,0,0,0,0],[0,0,1,0,0,0,0,0,0],[0,0,1,0,0,0,0,0,0]]1429	1430,1431,1432,1433,1434,1435,1436,1437,1438,1439,1440,1441,1442,1443,1444,1445,1446,1447,1448,1449	[[0,0,0,1,0,0,0,0,0],[0,0,0,1,0,0,0,0,0],[0,0,0,1,0,0,0,0,0],[0,0,0,1,0,0,0,0,0],[0,0,0,1,0,0,0,0,0],[0,0,0,1,0,0,0,0,0],[0,0,0,1,0,0,0,0,0],[0,0,0,1,0,0,0,0,0],[0,0,0,1,0,0,0,0,0],[0,0,0,1,0,0,0,0,0],[0,0,0,1,0,0,0,0,0],[0,0,0,1,0,0,0,0,0],[0,0,0,1,0,0,0,0,0],[0,0,0,1,0,0,0,0,0],[0,0,0,1,0,0,0,0,0],[0,0,0,1,0,0,0,0,0],[0,0,0,1,0,0,0,0,0],[0,0,0,1,0,0,0,0,0],[0,0,0,1,0,0,0,0,0],[0,0,0,1,0,0,0,0,0]]1450	1451,1452,1453,1454	[[0,0,0,1,0,0,0,0,0],[0,0,0,1,0,0,0,0,0],[0,0,0,1,0,0,0,0,0],[0,0,0,1,0,0,0,0,0]]1455	1456	[[0,0,0,0,1,0,0,0,0]]1457	1458	[[0,0,0,0,1,0,0,0,0]]1459	1460,1461,1462,1463,1464,1465,1466,1467,1468,1469,1470,1471,1472,1473	[[0,1,1,0,0,0,0,0,0],[0,1,0,0,0,0,0,0,0],[0,1,0,0,0,0,0,0,0],[0,1,0,0,0,0,0,0,0],[0,1,1,0,0,0,0,0,0],[0,0,1,0,0,0,0,0,0],[0,0,1,0,0,0,0,0,0],[0,0,1,0,0,0,0,0,0],[0,0,1,0,0,0,0,0,0],[0,0,1,0,0,0,0,0,0],[0,0,1,0,0,0,0,0,0],[0,0,1,0,0,0,0,0,0],[0,0,1,0,0,0,0,0,0],[0,0,1,0,0,0,0,0,0]]1474	1196,1387,1362,1331,1342,1334,1333,1410,1350,1345,1347,1346,1375,1405,1398,1348,1392,1403,1060	[[0,0,0,1,0,0,0,0,0],[0,0,0,1,0,0,0,0,0],[0,0,0,1,0,0,0,0,0],[0,0,0,1,0,0,0,0,0],[0,0,0,1,0,0,0,0,0],[0,0,0,1,0,0,0,0,0],[0,0,0,1,0,0,0,0,0],[0,0,0,1,0,0,0,0,0],[0,0,0,1,0,0,0,0,0],[0,0,0,1,0,0,0,0,0],[0,0,0,1,0,0,0,0,0],[0,0,0,1,0,0,0,0,0],[0,0,0,1,0,0,0,0,0],[0,0,0,1,0,0,0,0,0],[0,0,0,1,0,0,0,0,0],[0,0,0,1,0,0,0,0,0],[0,0,0,1,0,0,0,0,0],[0,0,0,1,0,0,0,0,0],[0,0,0,1,0,0,0,0,0]]1475	1476,1477,1478,1479,1480,1481,1482,1483,1484,1485,1326,1486,1327,1487,1488,1489,1490,1491,1492	[[0,1,1,0,0,0,0,0,0],[0,1,1,0,0,0,0,1,0],[0,1,0,0,0,0,0,1,0],[0,1,1,0,1,0,0,1,0],[0,0,0,0,0,0,0,1,0],[0,0,1,0,0,0,0,0,0],[0,0,1,0,0,0,0,0,0],[0,0,1,0,0,0,0,1,0],[0,1,1,0,0,0,0,1,0],[0,1,1,0,0,0,0,1,0],[0,0,1,1,0,0,0,0,0],[0,0,0,0,0,0,0,1,0],[0,1,1,1,0,0,0,1,0],[0,1,1,0,1,0,0,1,0],[0,0,1,0,0,0,0,0,0],[0,1,1,0,0,0,0,1,0],[0,1,1,0,0,0,0,1,0],[0,0,0,0,1,0,0,0,0],[0,0,1,0,0,0,0,0,0]]1493	1494,1495,1496,1497,1498,1499,1500,1501,1502,1503,1504,1505,1506,1470,1507,1508,1509,1510,1511,1512,1513,1514,1515,1516,1517,1518,1519,1520,1521,1522,1523,1524,1525,1526	[[0,0,1,1,0,0,0,0,0],[0,0,1,0,0,0,0,0,0],[0,1,1,0,0,0,0,0,0],[0,0,1,1,0,0,0,0,0],[0,0,1,0,0,0,0,0,0],[0,0,1,0,0,0,0,0,0],[0,0,1,0,0,0,0,0,0],[0,1,1,1,0,0,0,0,0],[0,0,1,1,1,0,0,0,0],[0,0,1,0,0,0,0,0,0],[0,1,1,0,0,0,0,0,0],[0,0,1,0,0,0,0,0,0],[0,1,1,1,0,0,0,0,0],[0,0,1,0,0,0,0,0,0],[0,0,1,0,0,0,0,0,0],[0,0,1,0,0,0,0,0,0],[0,0,1,0,0,0,0,0,0],[0,0,1,0,0,0,0,0,0],[0,0,1,0,0,0,0,0,0],[0,0,1,0,0,0,0,0,0],[0,1,1,0,0,0,0,0,0],[0,1,1,1,0,0,0,0,0],[0,0,1,0,0,0,0,0,0],[0,0,1,0,0,0,0,0,0],[0,0,1,0,0,0,0,0,0],[0,1,1,0,0,0,0,0,0],[0,1,1,0,0,0,0,0,0],[0,1,1,0,0,0,0,0,0],[0,0,1,1,0,0,0,0,0],[0,1,1,0,0,0,0,0,0],[0,0,1,0,0,0,0,0,0],[0,0,1,0,0,0,0,0,0],[0,0,1,0,0,0,0,0,0],[0,0,1,0,0,0,0,0,0]]1527	1528,1529,1530,1531,1532,1533,1479,1534,1535,1536,1537,1538,1539,1540	[[0,0,0,1,0,0,0,0,0],[0,0,0,1,0,0,0,0,0],[0,0,0,1,0,0,0,0,0],[0,0,0,1,0,0,0,0,0],[0,0,0,1,0,0,0,0,0],[0,1,0,0,0,0,0,0,0],[0,1,0,0,0,0,0,0,0],[0,0,0,1,0,0,0,0,0],[0,1,1,1,0,0,0,0,0],[0,1,1,1,0,0,0,0,0],[0,0,0,1,0,0,0,0,0],[0,0,0,1,0,0,0,0,0],[0,0,1,0,0,0,0,0,0],[0,0,0,1,0,0,0,0,0]]1541	1542,1543,1544,1545,668,1546,1547,1548,1549,1550,1551,1552,1553,1554,1555,1556,1557,1558,1559,1560,1561,1562,757,1563,1564,1565,633,1566,1567,1568,1569,1570,944,1571,383,1572,1573,1574,1575,1576,1577,1578,1579,750,987,1580,1581,1582,1583,1584,1585,1586,1587,372,1588,1589,1590,1591,1592,1593,1594,1595,1596,1597,370,1598,1599,1600,1601,1602,1603,1604,1605,1606,1607,1608,1609,1610,1611,1612,1613,561,1614,1615,1616,1617,1618,1619,1620,1621,1622,1623,1624,1625,1626,1627,1628,1629,1630,1631,994,399,1632,1633,1634,1635,1636,1637,1638,1639,1640	[[0,0,1,0,0,0,0,0,0],[0,1,0,0,0,0,0,1,0],[1,0,1,1,0,0,0,1,0],[0,0,1,1,0,0,0,0,0],[0,0,1,0,0,0,0,0,0],[0,0,1,0,0,0,0,0,0],[0,1,1,0,0,0,1,0,0],[0,0,1,0,0,0,0,0,0],[0,0,1,0,0,0,0,0,0],[1,1,1,0,0,0,1,1,0],[0,1,0,0,0,0,1,0,0],[0,1,1,0,0,0,1,1,0],[0,1,1,1,1,0,1,1,0],[0,0,1,0,0,0,0,0,0],[0,0,1,0,0,0,0,0,0],[0,0,1,0,0,0,0,0,0],[0,0,1,0,0,0,0,0,0],[0,0,1,0,0,0,0,0,0],[0,0,1,0,0,0,0,1,0],[1,0,1,0,0,0,0,0,0],[0,0,1,0,0,0,0,0,0],[0,0,0,1,0,0,0,1,0],[1,1,0,0,0,0,1,1,0],[0,1,1,0,0,0,1,1,0],[0,0,1,0,0,0,0,0,0],[0,1,1,0,0,0,1,0,0],[0,0,1,0,0,0,0,0,0],[0,0,1,0,0,0,0,0,0],[0,0,1,0,0,0,0,0,0],[0,0,1,0,0,0,0,1,0],[0,0,1,0,0,0,0,0,0],[0,0,1,0,0,0,0,0,0],[0,0,1,0,0,0,0,0,0],[0,0,0,0,1,0,0,1,0],[0,0,1,0,0,0,0,1,0],[0,0,1,0,0,0,0,0,0],[0,0,1,0,0,0,0,0,0],[1,1,1,1,1,0,1,1,0],[0,0,1,0,0,0,0,0,0],[0,0,0,1,0,0,0,0,0],[0,0,1,0,0,0,0,1,0],[0,0,1,0,0,0,0,0,0],[0,1,1,0,0,0,1,0,0],[1,0,0,0,0,0,0,0,0],[1,1,1,0,0,0,1,1,0],[1,1,1,1,1,0,1,1,0],[1,1,0,1,1,0,1,1,0],[0,0,1,0,0,0,0,0,0],[0,0,1,0,0,0,0,0,0],[0,0,0,0,0,0,0,1,0],[0,0,1,0,0,0,0,0,0],[0,0,1,0,0,0,0,0,0],[0,1,1,0,0,0,1,1,0],[0,1,1,0,0,0,1,0,0],[0,0,1,0,0,0,0,0,0],[0,0,1,0,0,0,0,0,0],[0,0,1,0,0,0,0,0,0],[0,0,0,0,0,0,0,1,0],[0,0,1,0,0,0,0,1,0],[0,0,1,0,0,0,0,0,0],[0,0,1,0,0,0,0,0,0],[0,0,1,0,0,0,0,0,0],[1,0,0,0,0,0,0,1,0],[0,0,1,0,0,0,0,0,0],[0,0,1,0,0,0,0,0,0],[0,0,1,0,0,0,0,0,0],[0,0,1,0,0,0,0,1,0],[0,1,0,0,0,0,1,0,0],[1,0,0,0,0,0,0,0,0],[1,0,1,0,0,0,0,0,0],[1,0,0,0,0,0,0,0,0],[1,1,0,0,0,0,0,0,0],[0,0,1,0,0,0,0,0,0],[0,0,1,0,0,0,0,0,0],[0,0,1,0,0,0,0,0,0],[0,0,1,0,0,0,0,0,0],[0,0,1,0,0,0,0,0,0],[0,0,1,0,0,0,0,0,0],[0,1,0,0,0,0,0,0,0],[0,1,0,0,0,0,0,0,0],[0,0,0,1,1,0,0,0,0],[0,0,1,0,0,0,0,0,0],[0,0,1,0,0,0,0,0,0],[0,0,1,0,0,0,0,0,0],[0,0,1,0,0,0,0,0,0],[0,0,1,0,0,0,0,0,0],[0,0,1,0,0,0,0,0,0],[0,0,1,0,0,0,0,0,0],[0,0,1,0,0,0,0,1,0],[0,1,0,0,0,0,0,0,0],[0,1,1,0,0,0,0,0,0],[0,1,1,0,0,0,1,0,0],[0,0,1,0,0,0,0,1,0],[0,0,0,0,0,0,0,1,0],[0,1,1,0,1,0,0,1,0],[0,0,1,0,0,0,0,0,0],[0,0,0,0,1,0,0,1,0],[0,0,0,0,1,0,0,0,0],[0,0,1,0,0,0,0,0,0],[0,0,1,0,0,0,0,0,0],[0,0,1,0,0,0,0,0,0],[0,0,1,0,0,0,0,0,0],[0,0,1,0,0,0,0,0,0],[0,0,1,0,0,0,0,0,0],[0,0,1,0,0,0,0,0,0],[0,0,1,0,0,0,0,0,0],[0,0,1,0,0,0,0,0,0],[0,0,1,0,0,0,0,0,0],[0,0,1,0,0,0,0,0,0],[0,0,1,0,0,0,0,0,0],[0,0,1,0,0,0,0,0,0]]1641	1453,1642,1643,1644,1645,1646,1647,1648,1649,1650,1651,1652,1653,1654,1595,1655,1656,1386,1657,1658,650,1659,1660,1452,1661,1662,1663,1664,1665,1666,1667,1668,1669,1670,1671,1672,1673,1674,1675,1676,1677,1678,1679,1680,1681,1682,1683,1684,1685,1686,1687,1688,1689,1690,1691,1692,1693,1694,1454,1695,1696,1697,1698,1699,1700,1701,1702,655,1703,1704,1705,1706,1451,1707,1708,1709,1710	[[0,0,1,1,0,0,0,0,0],[0,0,1,0,0,0,0,0,0],[0,0,1,0,0,0,0,1,0],[0,0,1,0,0,0,0,0,0],[0,0,1,0,0,0,0,0,0],[0,0,1,0,0,0,0,0,0],[0,0,1,0,0,0,0,0,0],[0,0,1,0,0,0,0,0,0],[0,0,1,0,0,0,0,0,0],[0,0,1,0,0,0,0,1,0],[0,0,1,0,0,0,0,0,0],[0,0,1,0,0,0,0,0,0],[0,0,1,0,0,0,0,0,0],[0,0,1,0,0,0,0,0,0],[0,0,0,0,0,0,0,1,0],[0,0,1,0,0,0,0,0,0],[0,0,1,0,0,0,0,0,0],[0,0,1,0,0,0,0,0,0],[0,0,1,0,0,0,0,0,0],[0,0,1,0,0,0,0,0,0],[0,0,1,0,0,0,0,0,0],[0,0,1,0,0,0,0,0,0],[0,0,1,0,0,0,0,1,0],[0,1,1,1,0,0,0,1,0],[0,0,1,0,0,0,0,0,0],[0,0,1,0,0,0,0,0,0],[0,0,1,0,0,0,0,0,0],[0,0,0,0,0,0,0,1,0],[0,0,1,0,0,0,0,0,0],[0,0,1,0,0,0,0,0,0],[0,1,1,0,0,0,0,0,0],[0,0,1,0,0,0,0,0,0],[0,0,1,0,0,0,0,0,0],[0,0,1,0,0,0,0,1,0],[0,0,1,0,0,0,0,0,0],[0,1,1,0,0,0,0,0,0],[0,0,1,0,0,0,0,0,0],[0,0,1,0,0,0,0,0,0],[0,0,1,0,0,0,0,0,0],[0,0,1,0,0,0,0,0,0],[0,1,1,0,0,0,0,0,0],[0,0,1,0,0,0,0,0,0],[0,0,1,0,0,0,0,0,0],[0,0,1,0,0,0,0,0,0],[0,0,1,0,0,0,0,0,0],[0,0,1,0,0,0,0,0,0],[0,0,1,0,0,0,0,0,0],[0,0,1,0,0,0,0,0,0],[0,0,1,0,0,0,0,0,0],[0,0,1,0,0,0,0,0,0],[0,0,1,0,0,0,0,1,0],[0,0,1,0,0,0,0,0,0],[0,0,1,0,0,0,0,0,0],[0,0,1,0,0,0,0,0,0],[0,0,1,0,0,0,0,0,0],[0,0,1,0,0,0,0,0,0],[0,0,1,0,0,0,0,1,0],[0,0,1,0,0,0,0,0,0],[0,1,1,1,0,0,0,0,0],[0,0,1,0,0,0,0,0,0],[0,0,0,0,0,0,0,1,0],[0,1,0,0,0,0,0,0,0],[0,0,1,0,0,0,0,0,0],[0,0,1,0,0,0,0,0,0],[0,1,1,0,0,0,0,1,0],[0,0,1,0,0,0,0,0,0],[0,1,0,0,0,0,0,0,0],[0,0,1,0,0,0,0,0,0],[0,0,1,0,0,0,0,0,0],[0,0,1,0,0,0,0,0,0],[0,0,1,0,0,0,0,1,0],[0,0,1,0,0,0,0,0,0],[0,0,1,1,0,0,0,1,0],[0,0,1,0,0,0,0,0,0],[0,0,1,0,0,0,0,0,0],[0,0,1,0,0,0,0,0,0],[0,0,1,0,0,0,0,0,0]]1711	1712,1431,1449,1432,1430,1440,1448,1713,1042,1447,1714,1715,1716,1717,1718,1719,1720,1721,1436,1438,1439,1437,631,401,1434,1435,1444,1445,1433,1442,1722,1443,1723,1446,1441,1724	[[0,0,1,0,0,0,0,0,0],[1,0,0,1,1,0,0,1,0],[1,1,1,1,0,0,1,1,0],[1,0,1,1,0,0,0,1,0],[1,0,1,1,0,0,0,1,0],[0,0,0,1,0,0,0,0,0],[0,0,0,1,0,0,0,1,0],[0,0,0,0,0,0,0,1,0],[1,1,1,0,0,0,1,1,0],[1,1,1,1,1,0,1,0,0],[0,0,0,0,0,0,0,1,0],[0,0,0,0,1,0,0,0,0],[1,0,0,0,0,0,0,0,0],[1,0,0,0,0,0,0,0,0],[1,1,1,0,0,0,0,1,0],[1,1,1,0,0,0,1,0,0],[1,0,0,0,0,0,0,0,0],[1,0,0,0,0,0,0,0,0],[1,1,1,1,0,0,0,1,0],[1,1,1,1,0,0,0,1,0],[1,1,1,1,0,0,0,1,0],[0,0,0,1,0,0,0,1,0],[0,0,1,0,0,0,0,0,0],[0,0,1,0,0,0,0,0,0],[0,0,0,1,0,0,0,0,0],[0,0,0,1,0,0,0,1,0],[0,0,0,1,0,0,0,0,0],[0,0,0,1,0,0,0,1,0],[0,0,0,1,0,0,0,0,0],[0,0,0,1,0,0,0,0,0],[0,0,1,0,0,0,0,0,0],[0,0,1,1,0,0,0,1,0],[0,0,1,0,0,0,0,0,0],[0,0,1,1,0,0,0,0,0],[0,0,0,1,0,0,0,1,0],[0,0,1,0,0,0,0,0,0]]1725	1726	[[0,0,0,0,1,0,0,0,0]]1727	1728,1729,1730,1731	[[0,0,0,0,1,0,0,0,0],[0,0,0,0,1,0,0,0,0],[0,0,0,0,1,0,0,0,0],[0,0,0,0,1,0,0,0,0]]1732	1733,1734,1735,1736,1737,1738,1739,1740,1741,1742,1743,1744,1745	[[0,0,0,0,1,0,0,0,0],[0,1,0,0,0,0,0,0,0],[0,1,0,0,1,0,0,0,0],[0,1,0,0,0,0,0,0,0],[0,0,0,0,1,0,0,0,0],[0,1,1,1,0,0,0,0,0],[0,1,1,0,0,0,0,0,0],[0,1,0,0,0,0,0,0,0],[0,1,0,0,1,0,0,0,0],[0,0,0,1,1,0,0,0,0],[0,1,0,0,0,0,0,0,0],[0,0,0,1,1,0,0,0,0],[0,0,0,0,1,0,0,0,0]]1746	65,1747,1748,1749,1750,1751,1752,1753,1754,1755,1756,1757,1758,1759,1760	[[0,0,0,1,0,0,0,0,0],[0,0,0,1,0,0,0,0,0],[0,0,0,0,1,0,0,0,0],[0,0,0,0,1,0,0,0,0],[0,0,0,0,1,0,0,0,0],[0,0,0,1,1,0,0,0,0],[0,0,0,0,1,0,0,0,0],[0,0,0,0,1,0,0,0,0],[0,0,0,1,0,0,0,0,0],[0,0,0,0,1,0,0,0,0],[0,0,0,0,1,0,0,0,0],[0,0,0,0,1,0,0,0,0],[0,0,0,0,1,0,0,0,0],[0,0,0,0,1,0,0,0,0],[0,0,0,1,0,0,0,0,0]]1761	1762,1763,1764,1765,1766,1767,1768,539,1769,1770,1771,1772,1773,1774,1775,1776,1777,1778,1779,750,1780,1781,1782,1783,1784,1785,1786,1787,1788,1789,1790,1791,1792,1793,1794,1795,1796,1797,1798,1799,1800,1801,1802,1803,1804,1805,1806,1807,1808,67,1809,1810,1811,1812,1813,1814,1815,1816,1817,1818,1819,1820,1821,1822,1823,1824,1825,1826,1827,1828,1829,1830,1831,1832,1833,1834,1835,1836,1837,1838,1839,1840,1841,1842,1843,1844,1845,1846,1847,1848,1849,1850,1851,1852,1853,1854,1855,1856,1857,1858,1859	[[0,1,1,0,0,0,0,0,0],[0,0,1,0,0,0,0,0,0],[0,1,1,0,0,0,0,0,0],[0,0,1,0,0,0,0,0,0],[0,0,1,0,0,0,0,0,0],[0,0,1,0,0,0,0,0,0],[0,0,1,0,0,0,0,0,0],[0,1,1,0,0,0,1,0,0],[0,1,0,0,0,0,0,0,0],[0,1,1,1,0,0,0,0,0],[0,1,1,0,0,0,0,0,0],[0,1,1,0,0,0,0,0,0],[0,0,1,0,0,0,0,0,0],[0,1,1,1,1,0,0,0,0],[0,1,1,0,0,0,0,0,0],[0,1,1,0,0,0,0,0,0],[0,1,1,0,0,0,0,0,0],[0,1,1,1,0,0,1,0,0],[0,0,1,0,0,0,0,0,0],[0,0,1,0,0,0,0,0,0],[0,1,1,0,0,0,0,0,0],[0,1,0,0,0,0,0,0,0],[0,1,1,0,0,0,1,0,0],[0,0,1,0,0,0,0,0,0],[0,0,1,0,0,0,0,0,0],[0,1,1,0,0,0,0,0,0],[0,0,1,0,0,0,0,0,0],[0,1,0,0,0,0,0,0,0],[0,1,1,0,0,0,0,0,0],[0,1,1,0,0,0,0,0,0],[0,1,1,0,0,0,0,0,0],[0,0,1,0,0,0,0,0,0],[0,0,1,0,0,0,0,0,0],[0,0,1,0,0,0,0,0,0],[0,1,1,0,0,0,0,0,0],[0,0,1,0,0,0,0,0,0],[0,0,1,0,0,0,0,0,0],[0,0,1,0,0,0,0,0,0],[0,0,1,0,0,0,0,0,0],[0,0,1,1,0,0,0,0,0],[0,0,1,0,0,0,0,0,0],[0,0,1,0,0,0,0,0,0],[0,1,1,0,0,0,0,0,0],[0,1,1,0,0,0,0,0,0],[0,1,1,0,0,0,0,0,0],[0,0,1,0,0,0,0,0,0],[0,0,1,0,0,0,0,0,0],[0,0,1,0,0,0,0,0,0],[0,0,1,0,0,0,0,0,0],[0,1,1,0,0,0,0,0,0],[0,0,1,0,0,0,0,0,0],[0,1,1,0,0,0,0,0,0],[0,0,1,0,0,0,0,0,0],[0,0,1,0,0,0,0,0,0],[0,0,1,0,0,0,0,0,0],[0,0,1,0,0,0,0,0,0],[0,0,1,0,0,0,0,0,0],[0,0,1,0,0,0,0,0,0],[0,0,1,0,0,0,0,0,0],[0,0,1,0,0,0,0,0,0],[0,0,1,0,0,0,0,0,0],[0,0,1,0,0,0,0,0,0],[0,0,1,0,0,0,0,0,0],[0,0,1,0,0,0,0,0,0],[0,0,1,0,0,0,0,0,0],[0,0,1,0,0,0,0,0,0],[0,0,1,0,0,0,0,0,0],[0,1,1,0,0,0,0,0,0],[0,0,1,0,1,0,0,0,0],[0,0,1,0,0,0,0,0,0],[0,0,1,0,0,0,1,0,0],[0,0,1,0,0,0,0,0,0],[0,0,1,0,0,0,0,0,0],[0,0,1,0,0,0,0,0,0],[0,0,1,0,0,0,0,0,0],[0,0,1,0,0,0,0,0,0],[0,0,1,0,0,0,0,0,0],[0,0,1,0,0,0,0,0,0],[0,0,1,0,0,0,0,0,0],[0,0,1,0,0,0,0,0,0],[0,0,1,0,0,0,0,0,0],[0,0,1,0,0,0,0,0,0],[0,1,1,0,0,0,0,0,0],[0,1,0,0,0,0,0,0,0],[0,1,0,0,0,0,0,0,0],[0,1,0,0,0,0,0,0,0],[0,1,0,0,0,0,0,0,0],[0,1,0,0,0,0,0,0,0],[0,0,1,0,0,0,0,0,0],[0,0,1,0,0,0,0,0,0],[0,0,1,0,0,0,0,0,0],[0,0,1,0,0,0,0,0,0],[0,0,1,0,0,0,0,0,0],[0,0,1,0,0,0,0,0,0],[0,0,1,0,0,0,0,0,0],[0,0,1,0,0,0,0,0,0],[0,0,1,0,0,0,1,0,0],[0,0,1,0,0,0,0,0,0],[0,0,1,0,0,0,0,0,0],[0,0,1,0,0,0,1,0,0],[0,0,1,0,0,0,0,0,0]]1860	1861,1862,1863,1864,1865,1866,1867,1868,1869,1525,1870,1871,1872,1873,1874,1875,1876,1877,1878,1879,1880,1881,1882,1883,1884,1885,1886,1887,1888,1889,1890,1891,1892,1893,1894,1895,1896,1897,1898,1899,1900	[[0,0,1,1,0,0,0,0,0],[0,0,1,0,0,0,0,0,0],[0,1,0,0,1,0,0,0,0],[0,1,1,0,1,0,0,0,0],[0,1,1,0,1,0,0,0,0],[0,0,1,0,0,0,0,0,0],[0,1,1,1,0,0,0,0,0],[0,1,1,0,0,0,0,0,0],[0,0,1,0,0,0,0,0,0],[0,1,1,0,0,0,0,0,0],[0,0,1,0,0,0,0,0,0],[0,0,1,0,0,0,0,0,0],[0,0,1,0,0,0,0,0,0],[0,0,1,0,0,0,0,0,0],[0,0,1,0,0,0,0,0,0],[0,0,1,0,0,0,0,0,0],[0,1,1,0,0,0,0,0,0],[0,0,1,0,0,0,0,0,0],[0,1,1,0,0,0,0,0,0],[0,0,1,0,0,0,0,0,0],[0,0,0,1,1,0,0,0,0],[0,0,1,1,0,0,0,0,0],[0,0,1,0,0,0,0,0,0],[0,0,1,0,0,0,0,0,0],[0,1,0,1,0,0,0,0,0],[0,0,1,0,1,0,0,0,0],[0,1,1,0,0,0,0,0,0],[0,0,1,0,0,0,0,0,0],[0,0,1,0,0,0,0,0,0],[0,0,1,0,0,0,0,0,0],[0,1,1,1,0,0,0,0,0],[0,0,0,0,1,0,0,0,0],[0,0,0,1,0,0,0,0,0],[0,1,0,0,0,0,0,0,0],[0,1,0,0,0,0,0,0,0],[0,1,0,0,0,0,0,0,0],[0,1,1,1,0,0,0,0,0],[0,0,1,0,0,0,0,0,0],[0,0,0,1,0,0,0,0,0],[0,1,0,0,0,0,0,0,0],[0,1,0,0,0,0,0,0,0]]1901	1902,1903,1904,1905,1906,1907,1908	[[0,1,0,0,0,0,0,0,0],[0,1,0,0,0,0,0,0,0],[0,1,0,0,0,0,0,0,0],[0,1,0,0,0,0,0,0,0],[0,1,0,0,0,0,0,0,0],[0,0,0,0,1,0,0,0,0],[0,1,0,0,0,0,0,0,0]]1909	1910	[[0,0,0,0,1,0,0,0,0]]1911	1912,1913,1914,1915,1916,1917,1918,1919,1920,1921,1922,1923,1924,1925,1926,1927,1928,1929,1930,1931	[[0,0,1,0,0,0,0,0,0],[0,0,1,0,0,0,0,0,0],[0,1,0,1,0,0,0,0,0],[0,0,1,0,0,0,0,0,0],[0,1,0,0,0,0,0,0,0],[0,1,1,0,0,0,0,0,0],[0,0,0,0,1,0,0,0,0],[0,1,0,0,0,0,0,0,0],[0,0,1,0,0,0,0,0,0],[0,1,1,1,0,0,0,0,0],[0,0,0,0,1,0,0,0,0],[0,0,1,0,0,0,0,0,0],[0,0,1,0,0,0,0,0,0],[0,0,1,0,0,0,0,0,0],[0,0,0,1,0,0,0,0,0],[0,0,1,0,0,0,0,0,0],[0,0,1,0,0,0,0,0,0],[0,0,1,0,0,0,0,0,0],[0,0,0,0,1,0,0,0,0],[0,0,0,0,1,0,0,0,0]]1932	1742,1738,1744	[[0,0,0,1,0,0,0,0,0],[0,0,0,1,0,0,0,0,0],[0,0,0,1,0,0,0,0,0]]1933	1665,1934,1935,1936,1937,1938,1939,1940,1941,1942,1943,1944,1945,1946,1685,1497,156,1947,1948,1949	[[0,0,0,1,0,0,0,0,0],[0,0,0,1,0,0,0,0,0],[0,0,0,1,0,0,0,0,0],[0,0,0,1,0,0,0,0,0],[0,0,0,1,0,0,0,0,0],[0,0,0,1,0,0,0,0,0],[0,0,0,1,0,0,0,0,0],[0,0,0,1,0,0,0,0,0],[0,0,0,1,0,0,0,0,0],[0,0,0,1,0,0,0,0,0],[0,0,0,1,0,0,0,0,0],[0,0,0,1,0,0,0,0,0],[0,0,0,1,0,0,0,0,0],[0,0,0,1,0,0,0,0,0],[0,0,0,1,0,0,0,0,0],[0,0,0,1,0,0,0,0,0],[0,0,0,1,0,0,0,0,0],[0,0,0,1,0,0,0,0,0],[0,0,0,1,0,0,0,0,0],[0,0,0,1,0,0,0,0,0]]1950	1951	[[0,0,0,0,1,0,0,0,0]]1952	1953,1954,1955,1956	[[0,0,0,0,1,0,0,0,0],[0,0,0,0,1,0,0,0,0],[0,0,0,0,1,0,0,0,0],[0,0,0,0,1,0,0,0,0]]1957	1924,1923,1925,1915,1928,1929,1927,1912,1913,1920,1921,1914	[[0,0,1,0,0,0,0,0,0],[0,0,1,0,0,0,0,0,0],[0,0,1,0,0,0,0,0,0],[0,0,1,0,0,0,0,0,0],[0,0,1,0,0,0,0,0,0],[0,0,1,0,0,0,0,0,0],[0,0,1,0,0,0,0,0,0],[0,0,1,0,0,0,0,0,0],[0,0,1,0,0,0,0,0,0],[0,0,1,0,0,0,0,0,0],[0,0,1,0,0,0,0,0,0],[0,0,1,0,0,0,0,0,0]]1958	1959	[[0,0,0,0,1,0,0,0,0]]1960	1961,1962	[[0,0,0,1,0,0,0,0,0],[0,0,0,1,0,0,0,0,0]]1963	1964,1965	[[0,0,0,0,1,0,0,0,0],[0,0,0,0,1,0,0,0,0]]1966	1967,1968	[[0,0,0,0,1,0,0,0,0],[0,0,0,0,1,0,0,0,0]]1969	1970,1971,1972,1973	[[0,0,0,0,1,0,0,0,0],[0,0,0,0,1,0,0,0,0],[0,0,0,0,1,0,0,0,0],[0,0,0,0,1,0,0,0,0]]1974	1975,1976,1977,551,1978,1979,1980,1981,1982,1983,1984,1985,1986,1987,1988,1989,1990,1991,1992,1993,1994,1995,1996,1997,1998,1999,2000,2001,2002,2003,2004,2005,2006,2007,2008,2009,2010,2011,2012,2013,2014,1099,1101,2015,2016,2017,2018,2019,2020,2021,2022,2023,2024,2025,2026,2027,2028,2029,2030,2031,2032,2033,2034,2035,2036,2037,2038,2039,2040,2041,2042,2043,2044,2045,2046,1079,2047,2048,1052,2049,2050,2051,2052,2053,2054,2055,1056,2056,2057,2058,2059,2060,2061,2062,1050,2063,2064	[[0,1,1,1,0,0,1,0,0],[0,0,1,0,0,0,0,0,0],[0,0,1,1,0,0,0,0,0],[0,1,0,0,0,0,0,0,0],[0,0,1,1,0,0,0,1,0],[0,0,1,1,0,0,0,1,0],[0,1,1,1,0,0,1,0,0],[0,0,1,0,1,0,0,0,0],[0,0,1,0,0,0,0,0,0],[0,0,1,1,0,0,0,1,0],[0,1,1,1,0,0,0,1,0],[0,1,1,1,0,0,0,1,0],[0,1,1,1,0,0,0,0,0],[0,1,0,0,0,0,0,0,0],[0,1,1,1,1,0,0,0,0],[0,0,0,1,0,0,0,0,0],[0,1,1,1,0,0,1,0,0],[0,0,0,0,1,0,0,0,0],[0,0,1,1,0,0,0,0,0],[0,0,1,1,0,0,0,0,0],[0,1,1,1,0,0,1,0,0],[0,1,1,1,0,0,1,0,0],[0,1,0,0,0,0,1,0,0],[0,1,0,0,0,0,1,0,0],[0,1,1,1,0,0,0,0,0],[0,1,1,0,0,0,0,0,0],[0,1,0,0,0,0,0,0,0],[0,0,0,1,0,0,0,0,0],[0,0,1,0,0,0,0,0,0],[0,0,0,1,0,0,0,0,0],[0,0,1,1,0,0,0,0,0],[0,0,1,0,0,0,0,0,0],[0,0,1,0,1,0,0,0,0],[0,0,1,0,0,0,0,0,0],[0,0,0,1,0,0,0,0,0],[0,0,0,1,0,0,0,0,0],[0,0,0,1,0,0,0,0,0],[0,0,1,1,0,0,0,0,0],[0,1,0,0,0,0,0,0,0],[0,0,1,1,0,0,0,1,0],[0,0,0,1,0,0,0,0,0],[0,0,1,0,0,0,0,0,0],[0,0,1,0,0,0,0,0,0],[0,0,1,0,0,0,0,0,0],[0,0,1,0,0,0,0,0,0],[0,0,1,0,0,0,0,0,0],[0,0,1,1,0,0,0,0,0],[0,0,1,1,0,0,0,0,0],[0,0,1,1,0,0,0,1,0],[0,0,0,1,0,0,0,1,0],[0,0,0,1,0,0,0,1,0],[0,0,1,1,0,0,0,0,0],[0,0,0,1,0,0,0,0,0],[0,0,1,1,0,0,0,0,0],[0,0,1,0,0,0,0,0,0],[0,0,1,0,0,0,0,0,0],[0,0,0,1,0,0,0,0,0],[0,0,0,1,0,0,0,0,0],[0,0,1,0,0,0,0,0,0],[0,0,0,1,0,0,0,0,0],[0,0,1,0,0,0,0,0,0],[0,0,0,1,0,0,0,1,0],[0,0,1,1,0,0,0,0,0],[0,0,0,1,0,0,0,0,0],[0,0,1,1,0,0,0,0,0],[0,0,1,1,0,0,0,1,0],[0,0,1,0,0,0,0,0,0],[0,0,0,1,0,0,0,0,0],[0,0,0,1,0,0,0,0,0],[0,0,0,1,0,0,0,0,0],[0,0,1,1,0,0,0,0,0],[0,0,0,1,0,0,0,0,0],[0,0,1,0,0,0,0,0,0],[0,0,1,1,0,0,0,0,0],[0,0,1,0,0,0,0,0,0],[0,0,0,1,1,0,0,1,0],[0,0,0,0,0,0,0,1,0],[0,0,1,1,0,0,0,0,0],[0,0,1,0,0,0,0,0,0],[0,0,1,0,0,0,0,0,0],[0,0,1,0,0,0,0,0,0],[0,0,1,1,0,0,0,0,0],[0,0,0,1,0,0,0,0,0],[0,0,0,1,0,0,0,1,0],[0,0,1,1,0,0,0,0,0],[0,0,1,0,0,0,0,0,0],[0,0,1,0,0,0,0,0,0],[0,0,1,0,0,0,0,0,0],[0,0,1,0,0,0,0,0,0],[0,0,1,1,0,0,0,0,0],[0,0,0,1,0,0,0,0,0],[0,0,0,0,1,0,0,0,0],[0,0,0,1,0,0,0,0,0],[0,0,1,1,0,0,0,0,0],[0,0,1,1,0,0,0,0,0],[0,0,1,0,0,0,0,0,0],[0,0,1,0,0,0,0,0,0]]2065	2066,2067	[[0,0,0,0,1,0,0,0,0],[0,0,0,0,1,0,0,0,0]]2068	2069,2070,2071	[[0,0,0,0,1,0,0,0,0],[0,0,0,0,1,0,0,0,0],[0,0,0,0,1,0,0,0,0]]2072	2073,2074,2075,2076,2077,2078,2079,2080	[[0,0,1,1,0,0,0,0,0],[0,0,1,1,0,0,0,0,0],[0,0,1,0,0,0,0,0,0],[0,0,1,1,0,0,0,0,0],[0,0,1,1,1,0,0,0,0],[0,0,0,1,0,0,0,0,0],[0,0,0,1,0,0,0,0,0],[0,0,1,1,0,0,0,0,0]]2081	2082,2083,2084,2085,2086,1709,2087,2088,2089,2090,2091,2092,2093,2094,2095,2096,2097,2098,2099,2100,2101,2102,2103,2104,2105,2106,2107,2108,2109,2110,2111,2112,2113,2114,2115,2116,2117,2118,2119,2120,2121,2122,2123,2124,2125,2126	[[0,0,1,0,0,0,0,1,0],[0,0,0,0,0,0,0,1,0],[0,1,1,0,0,0,0,1,0],[0,1,1,0,0,0,0,1,1],[0,1,1,1,0,0,0,1,1],[0,1,0,0,0,0,0,0,0],[0,1,1,0,1,0,0,1,0],[0,0,1,0,0,0,0,0,0],[0,1,1,0,0,0,0,1,1],[0,1,0,0,0,0,0,0,0],[0,1,0,0,0,0,0,0,0],[0,1,0,0,0,0,0,0,0],[0,0,1,0,0,0,0,1,1],[0,1,0,0,0,0,0,1,0],[0,1,1,0,1,0,0,0,0],[0,1,1,0,0,0,0,0,0],[0,0,0,0,0,0,0,0,1],[0,1,1,0,0,0,0,1,1],[0,1,1,0,0,0,0,0,0],[0,1,1,0,0,0,0,0,0],[0,0,1,0,0,0,0,1,0],[0,1,1,1,1,0,0,1,1],[0,1,0,0,0,0,0,0,0],[0,1,1,0,0,0,0,0,0],[0,1,1,0,0,0,0,0,0],[0,1,1,1,1,0,0,1,1],[0,0,1,0,0,0,0,0,0],[0,0,0,0,0,0,0,0,1],[0,0,1,0,0,0,0,0,0],[0,0,1,0,0,0,0,0,0],[0,0,1,0,0,0,0,0,1],[0,0,1,0,0,0,0,1,1],[0,0,1,0,0,0,0,0,0],[0,0,1,0,0,0,0,0,0],[0,0,0,0,1,0,0,0,0],[0,0,1,0,0,0,0,0,0],[0,1,0,0,0,0,0,0,0],[0,1,0,0,0,0,0,0,0],[0,1,0,0,0,0,0,0,0],[0,1,0,0,0,0,0,0,0],[0,1,0,0,0,0,0,0,0],[0,1,0,0,0,0,0,0,0],[0,1,0,0,0,0,0,0,0],[0,0,1,0,0,0,0,0,0],[0,0,1,0,0,0,0,0,0],[0,1,0,0,0,0,0,0,0]]2127	2128,2129,2130,2131,2132,2133,2134,2135,2136,2137,2138,2139,2140,2141,2142,2143,2144,2145,2146,2147,2148,2149,2150,2151,2152,2153,2154,2155,2156,2157,2158,2159,1639,2160,2161,2162,2163,2164,2165,2166,2167,2168,2169,2170,2171,712,2172,2173,2174,2175,2176,2177,2178,2179,2180	[[0,1,1,0,0,0,0,0,0],[0,0,1,0,0,0,0,0,0],[0,1,1,0,0,0,0,0,0],[0,0,1,0,0,0,0,0,0],[0,0,1,0,1,0,0,0,0],[0,1,0,0,0,0,0,0,0],[0,1,0,0,0,0,0,0,0],[0,1,0,0,0,0,0,0,0],[0,1,1,0,0,0,0,0,0],[0,0,1,0,0,0,0,0,0],[0,1,1,0,0,0,0,0,0],[0,1,1,0,0,0,0,0,0],[0,1,1,0,1,0,0,0,0],[0,0,1,0,0,0,0,0,0],[0,0,1,0,0,0,0,0,0],[0,0,1,0,0,0,0,0,0],[0,0,1,0,0,0,0,0,0],[0,0,1,0,0,0,0,0,0],[0,1,1,0,0,0,0,0,0],[0,0,1,0,0,0,0,0,0],[0,0,1,0,0,0,0,0,0],[0,0,1,0,0,0,0,0,0],[0,0,1,0,0,0,0,0,0],[0,0,1,0,0,0,0,0,0],[0,0,1,0,0,0,0,0,0],[0,0,1,0,0,0,0,0,0],[0,1,0,0,1,0,0,0,0],[0,1,1,0,0,0,0,0,0],[0,0,1,0,0,0,0,0,0],[0,0,1,0,0,0,0,0,0],[0,1,1,0,0,0,0,0,0],[0,0,1,0,0,0,0,0,0],[0,0,1,0,0,0,0,0,0],[0,0,1,0,0,0,0,0,0],[0,0,1,0,0,0,0,0,0],[0,0,1,0,0,0,0,0,0],[0,0,1,0,0,0,0,0,0],[0,0,1,0,0,0,0,0,0],[0,0,1,0,0,0,0,0,0],[0,0,1,0,0,0,0,0,0],[0,0,1,0,0,0,0,0,0],[0,0,1,0,0,0,0,0,0],[0,0,1,0,0,0,0,0,0],[0,0,1,0,0,0,0,0,0],[0,0,1,0,0,0,0,0,0],[0,0,1,0,0,0,0,0,0],[0,1,1,0,0,0,0,0,0],[0,0,1,0,0,0,0,0,0],[0,0,1,0,0,0,0,0,0],[0,0,1,0,0,0,0,0,0],[0,0,1,0,0,0,0,0,0],[0,0,1,0,0,0,0,0,0],[0,0,1,0,0,0,0,0,0],[0,0,1,0,0,0,0,0,0],[0,0,1,0,0,0,0,0,0]]2181	2182,2183,2184,2185,2186,2187,2188,2189,2190,2191,2192,2193,2194,2125,2195	[[0,0,0,1,0,0,0,0,0],[0,0,0,1,0,0,0,0,0],[0,0,0,1,0,0,0,0,0],[0,0,0,1,0,0,0,0,0],[0,0,0,1,0,0,0,0,0],[0,0,0,1,0,0,0,0,0],[0,0,0,1,0,0,0,0,0],[0,0,0,1,0,0,0,0,0],[0,0,0,1,0,0,0,0,0],[0,0,0,1,0,0,0,0,0],[0,0,0,1,0,0,0,0,0],[0,0,0,1,0,0,0,0,0],[0,0,0,1,0,0,0,0,0],[0,0,0,1,0,0,0,0,0],[0,0,0,1,0,0,0,0,0]]2196	2197,2198,2199,2200,2201,2202,2203,2204,2205,2206,2207,2208,2209,2210,2211,2212,404,2213,2214,2215,2216,2217,2218,2219,1128,2220,2221,2222,1258,2223,2224,2225,368,2226,2227,2228,2229,2230,2231,391,2232,2233,2234,2235	[[0,0,1,1,0,0,0,0,0],[0,0,1,1,0,0,0,0,0],[0,0,1,0,0,0,0,0,0],[0,0,0,0,1,0,0,0,0],[0,0,1,0,0,0,0,0,0],[0,0,1,1,1,0,0,0,0],[0,0,1,0,0,0,0,0,0],[0,0,1,1,1,0,0,0,0],[0,0,1,0,0,0,0,0,0],[0,0,1,1,0,0,0,0,0],[0,0,1,0,0,0,0,0,0],[0,0,1,0,0,0,0,0,0],[0,0,1,1,0,0,0,0,0],[0,0,1,0,0,0,0,0,0],[0,0,1,0,0,0,0,0,0],[0,0,1,0,0,0,0,0,0],[0,0,1,0,0,0,0,0,0],[0,0,1,0,0,0,0,0,0],[0,0,1,0,0,0,0,0,0],[0,0,0,1,0,0,0,0,0],[0,0,1,1,0,0,0,0,0],[0,0,1,0,0,0,0,0,0],[0,0,1,0,0,0,0,0,0],[0,0,1,1,0,0,0,0,0],[0,0,1,0,0,0,0,0,0],[0,0,1,0,0,0,0,0,0],[0,0,1,0,0,0,0,0,0],[0,0,0,0,1,0,0,0,0],[0,0,1,0,0,0,0,0,0],[0,0,1,0,0,0,0,0,0],[0,0,1,0,0,0,0,0,0],[0,0,1,0,0,0,0,0,0],[0,0,1,1,0,0,0,0,0],[0,0,1,1,0,0,0,0,0],[0,0,1,1,0,0,0,0,0],[0,0,1,1,0,0,0,0,0],[0,0,1,1,0,0,0,0,0],[0,0,1,0,0,0,0,0,0],[0,0,1,0,0,0,0,0,0],[0,0,1,0,0,0,0,0,0],[0,0,1,1,0,0,0,0,0],[0,0,1,0,0,0,0,0,0],[0,0,1,1,0,0,0,0,0],[0,0,1,0,0,0,0,0,0]]2236	2237,2238,2239,2240,2241,2242,2243,2244,2245,2246,2247,2248,2249,2250,2251,312,2252,2253,2254,2255,2256,2257,2258,2259,2260,2261,2262,2263,2264,2265,2266,796,2267,2268,2269,2270,2271,2178	[[0,1,0,0,0,0,1,0,0],[0,1,0,0,0,0,1,1,0],[0,1,0,0,0,0,1,0,0],[0,1,1,0,0,0,1,0,0],[0,1,1,0,0,0,1,0,0],[0,0,1,0,1,0,0,1,0],[0,0,1,0,0,0,0,0,1],[0,1,0,0,0,0,1,0,0],[0,1,1,1,0,0,1,0,1],[0,0,0,0,0,0,0,1,0],[0,0,0,0,0,1,0,1,0],[0,0,0,0,0,1,0,1,1],[0,0,1,0,0,0,0,1,0],[0,0,1,0,0,0,0,1,1],[0,1,0,0,0,0,0,0,0],[0,0,0,0,1,0,0,0,0],[0,1,0,0,0,0,0,0,0],[0,0,0,0,0,1,0,1,1],[0,1,1,0,0,0,1,0,1],[0,0,1,0,0,0,0,1,0],[0,0,1,0,0,0,0,1,1],[0,0,1,0,0,0,0,0,0],[0,1,0,0,0,0,0,1,0],[0,1,0,0,0,0,0,0,0],[0,1,1,0,1,0,0,1,1],[0,1,1,1,0,1,1,0,1],[0,1,0,0,0,0,0,0,0],[0,1,0,0,0,0,0,0,0],[0,1,0,0,0,0,0,1,0],[0,0,0,1,1,0,0,1,0],[0,0,1,0,0,0,1,1,0],[0,0,0,0,0,1,0,1,0],[0,0,1,0,0,0,0,0,0],[0,0,1,0,0,0,0,0,0],[0,0,1,0,0,0,0,0,0],[0,0,1,0,0,0,0,0,0],[0,0,0,0,0,0,0,1,0],[0,0,0,0,1,0,0,0,0]]2272	2185,2187	[[0,0,0,0,1,0,0,0,0],[0,0,0,0,1,0,0,0,0]]2273	2274,2275,2276,933,2277,2278,2279,2280,2281,2282,2283,2284,2285,2286,2287,2288,2289,2290,2291,2292,2293,2294,1180,96,2295,2296,1192,2297,2298,2299,2300,2301,2302,927,2303,90,2304,2305,2306,2307,2308,313,2309,2310,2311,2312,2313,2314,2315,2316,2317,2318,2319,2320,2321,2322,2323,2324,2325,2326,2327,2328,2329,2330,2331,2332,2333,2334,2335,2336,2337,2338,2339,940,2340,2341,2342,2343,2344,1183,2345,2346,2347,2348,2349,2350,2351,2352,2353,2354,2355,2356,2357,2358,2359	[[0,0,1,0,0,0,0,0,0],[0,0,0,1,0,0,0,0,0],[0,0,1,0,0,0,0,0,0],[0,0,1,1,0,0,0,0,0],[0,0,1,1,0,0,0,0,0],[0,0,1,0,0,0,0,0,0],[0,1,1,1,0,0,0,0,0],[0,1,1,1,0,0,0,0,1],[0,0,0,1,0,0,0,0,0],[0,0,1,0,0,0,0,0,0],[0,1,1,1,0,0,0,0,0],[0,0,0,1,0,0,0,0,0],[0,1,1,1,0,0,0,0,0],[0,0,1,1,0,0,0,0,0],[0,0,1,0,0,0,0,0,0],[0,0,1,0,0,0,0,0,0],[0,0,1,0,0,0,0,0,0],[0,0,0,1,0,0,0,0,0],[0,0,1,0,0,0,0,0,0],[0,0,1,0,0,0,0,0,0],[0,0,0,1,0,0,0,0,0],[0,1,1,1,0,0,0,0,0],[0,0,1,0,0,0,0,0,0],[0,0,1,0,0,0,0,0,0],[0,0,0,1,0,0,0,0,1],[0,0,1,0,0,0,0,0,0],[0,0,1,0,0,0,0,0,0],[0,0,1,1,0,0,0,0,0],[0,0,1,0,0,0,0,0,0],[0,1,1,1,0,0,0,0,1],[0,0,1,0,0,0,0,0,0],[0,0,1,0,0,0,0,0,0],[0,0,0,0,1,0,0,0,0],[0,0,1,0,0,0,0,0,0],[0,0,0,0,0,0,0,0,1],[0,0,0,1,0,0,0,0,0],[0,0,1,0,0,0,0,0,0],[0,0,1,0,0,0,0,0,0],[0,0,1,0,0,0,0,0,0],[0,0,1,0,0,0,0,0,0],[0,0,1,0,0,0,0,0,0],[0,0,1,0,0,0,0,0,0],[0,0,1,0,0,0,0,0,0],[0,1,0,1,0,0,0,0,0],[0,0,0,1,0,0,0,0,0],[0,0,0,1,0,0,0,0,0],[0,0,1,0,0,0,0,0,0],[0,0,1,1,0,0,0,0,0],[0,0,0,1,0,0,0,0,0],[0,0,0,1,0,0,0,0,0],[0,0,1,0,0,0,0,0,0],[0,0,0,1,0,0,0,0,1],[0,0,1,0,0,0,0,0,0],[0,0,0,1,0,0,0,0,0],[0,0,0,1,0,0,0,0,0],[0,0,1,0,0,0,0,0,0],[0,0,1,0,0,0,0,0,0],[0,0,0,1,0,0,0,0,0],[0,0,1,0,0,0,0,0,0],[0,0,1,0,0,0,0,0,0],[0,0,1,1,0,0,0,0,1],[0,0,0,1,0,0,0,0,1],[0,0,0,0,0,0,0,0,1],[0,0,1,1,0,0,0,0,0],[0,0,1,1,0,0,0,0,0],[0,0,0,1,0,0,0,0,1],[0,1,1,0,0,0,0,0,0],[0,0,1,0,0,0,0,0,0],[0,0,0,0,0,0,0,0,1],[0,1,1,1,0,0,0,0,0],[0,0,1,0,0,0,0,0,0],[0,1,1,1,0,0,0,0,0],[0,0,1,0,0,0,0,0,0],[0,0,0,1,0,0,0,0,1],[0,0,1,0,0,0,0,0,1],[0,0,0,1,0,0,0,0,1],[0,1,1,1,0,0,0,0,0],[0,0,1,0,0,0,0,0,0],[0,0,1,0,0,0,0,0,0],[0,0,1,1,0,0,0,0,0],[0,0,1,0,0,0,0,0,0],[0,0,1,0,0,0,0,0,0],[0,0,1,0,0,0,0,0,0],[0,0,1,0,0,0,0,0,0],[0,0,0,1,0,0,0,0,0],[0,0,0,0,0,0,0,0,1],[0,0,0,0,0,0,0,0,1],[0,0,1,0,0,0,0,0,0],[0,0,0,1,0,0,0,0,0],[0,0,1,0,0,0,0,0,0],[0,0,1,0,0,0,0,0,0],[0,0,1,1,0,0,0,0,1],[0,0,0,0,0,0,0,0,1],[0,0,1,1,0,0,0,0,0],[0,0,1,1,0,0,0,0,1]]2360	2361	[[0,0,0,0,1,0,0,0,0]]2362	2102,2086,2106	[[0,0,0,1,0,0,0,0,0],[0,0,0,1,0,0,0,0,0],[0,0,0,1,0,0,0,0,0]]2363	2364,2365,2366,2367,2368,2369,2143,2370,896,2371,2372,2373,2374,2375,2376,2377,2378,1772,2379,2380,2381,2382,2383,2384,2385,2386,2387,2388,2389,2390,2391,2392,2393,2394,2395,2396,2397,2398,2399,2400,2401,2402,2403,2404,2405,2406,2407,21,2408,2409,2410,2411,2412,2413,2414,2415,2416,2417,2418,2419,2420,2421,2422,2423,2424,2159,2425,2426	[[0,1,0,0,0,0,0,0,0],[0,1,0,0,0,0,1,0,0],[0,0,1,1,0,0,0,0,0],[0,0,0,1,0,0,0,0,0],[0,1,0,0,0,0,0,0,0],[0,0,0,1,0,0,0,0,1],[0,0,1,0,0,0,0,0,0],[0,1,1,1,0,0,0,0,0],[0,0,0,1,0,0,0,0,1],[0,1,1,1,0,0,1,0,1],[0,1,1,0,1,0,1,0,1],[0,1,1,1,0,0,1,0,0],[0,1,1,1,0,0,0,0,1],[0,1,1,0,0,0,0,0,1],[0,1,0,0,0,0,0,0,0],[0,1,0,0,0,0,0,0,0],[0,1,0,0,0,0,0,0,0],[0,1,0,0,0,0,0,0,0],[0,1,0,0,0,0,0,0,0],[0,0,0,1,0,0,0,0,0],[0,0,0,1,0,0,0,0,0],[0,0,0,1,0,0,0,0,0],[0,0,0,1,0,0,0,0,1],[0,0,1,0,0,0,0,0,0],[0,0,1,0,0,0,0,0,0],[0,1,1,1,0,0,1,0,0],[0,0,1,1,1,0,0,0,1],[0,0,0,1,0,0,0,0,0],[0,0,0,1,0,0,0,0,0],[0,0,1,0,0,0,0,0,0],[0,0,0,1,0,0,0,0,0],[0,0,0,1,0,0,0,0,0],[0,0,0,1,0,0,0,0,0],[0,0,1,0,0,0,0,0,0],[0,0,0,1,0,0,0,0,0],[0,0,0,1,0,0,0,0,0],[0,1,1,1,0,0,1,0,0],[0,0,0,1,0,0,0,0,0],[0,0,1,0,0,0,0,0,0],[0,0,0,0,0,0,1,0,0],[0,0,0,0,0,0,1,0,0],[0,1,1,1,0,0,1,0,1],[0,0,1,0,0,0,0,0,0],[0,0,1,0,0,0,0,0,0],[0,1,1,1,0,0,1,0,1],[0,1,0,1,0,0,1,0,1],[0,0,0,0,1,0,0,0,1],[0,1,1,1,1,0,1,0,0],[0,1,1,1,0,0,1,0,1],[0,0,0,1,0,0,0,0,0],[0,0,1,1,0,0,0,0,0],[0,0,1,0,0,0,0,0,0],[0,0,1,1,0,0,0,0,0],[0,0,1,1,0,0,0,0,0],[0,0,0,1,0,0,0,0,0],[0,0,1,0,0,0,0,0,0],[0,0,0,1,0,0,0,0,0],[0,0,0,1,0,0,0,0,0],[0,0,1,0,0,0,0,0,0],[0,0,1,0,0,0,0,0,0],[0,0,1,0,0,0,0,0,0],[0,0,1,0,0,0,0,0,0],[0,0,0,1,0,0,0,0,0],[0,0,1,1,0,0,0,0,0],[0,0,0,1,0,0,0,0,0],[0,0,1,0,0,0,0,0,0],[0,0,1,0,0,0,0,0,0],[0,0,1,0,0,0,0,0,1]]2427	2428,2429,2430,2431,2432	[[0,0,1,0,0,0,0,0,0],[0,0,1,0,0,0,0,0,0],[0,0,1,0,0,0,0,0,0],[0,0,1,0,0,0,0,0,0],[0,0,1,0,0,0,0,0,0]]2433	2434,2435,2436,2356,2437,2438,2439,2440,898,2441,2442,2443,2444,2445,2446,927,2447,2278,2448,2449,2450,2451,2452	[[0,0,1,0,0,0,0,0,0],[0,1,1,0,0,0,0,0,0],[0,1,1,0,1,0,1,0,0],[0,0,1,0,0,0,0,0,0],[0,1,1,0,0,0,1,0,0],[0,1,1,0,0,0,0,0,0],[0,1,1,0,1,0,1,0,0],[0,1,0,0,0,0,0,0,0],[0,0,1,0,0,0,0,0,0],[0,0,1,0,0,0,0,0,0],[0,0,1,0,0,0,0,0,0],[0,1,1,0,0,0,0,0,0],[0,0,1,0,0,0,0,0,0],[0,0,1,0,0,0,0,0,0],[0,0,1,0,0,0,0,0,0],[0,0,1,0,0,0,0,0,0],[0,0,1,0,0,0,0,0,0],[0,0,1,0,0,0,0,0,0],[0,1,1,0,1,0,1,0,0],[0,0,1,0,0,0,0,0,0],[0,0,1,0,0,0,0,0,0],[0,0,1,0,0,0,0,0,0],[0,0,1,0,0,0,0,0,0]]2453	2454,2455,2456,2457	[[0,0,0,0,1,0,0,0,0],[0,0,0,0,1,0,0,0,0],[0,0,0,0,1,0,0,0,0],[0,0,0,0,1,0,0,0,0]]2458	2459,2460,2461,2462,2463,2464,2465,2189,2186,2184,2125,2193,2192,2185,2188,2183,2187,2190,2191,2195,2182,2194	[[0,1,0,0,0,0,0,0,0],[0,1,0,0,0,0,0,0,0],[0,1,0,0,0,0,0,0,0],[0,1,0,0,0,0,0,0,0],[0,1,0,0,0,0,0,0,0],[0,1,0,0,0,0,0,0,0],[0,1,0,0,0,0,0,0,0],[0,1,0,1,0,0,0,0,0],[0,0,0,1,0,0,0,0,0],[0,1,0,1,0,0,0,0,0],[0,1,0,1,0,0,0,0,0],[0,0,0,1,0,0,0,0,0],[0,0,0,1,0,0,0,0,0],[0,0,0,1,0,0,0,0,0],[0,0,0,1,0,0,0,0,0],[0,0,0,1,0,0,0,0,0],[0,0,0,1,0,0,0,0,0],[0,0,0,1,0,0,0,0,0],[0,0,0,1,0,0,0,0,0],[0,0,0,1,0,0,0,0,0],[0,0,0,1,0,0,0,0,0],[0,0,0,1,0,0,0,0,0]]2466	2467	[[0,0,0,0,1,0,0,0,0]]2468	2469,2470,2471,2472,2473,2474,2475,2476,2477,2478,2479,2480,2481,2482,2483,474,2484,2485,2486,1078,2428,2487,2488,2489,2490,2491,2492,2493,2494,2495,2496,2497,2498,2499,2500,2501,2424,2502,2503,2504,2505,2506,2507,2508,2509,2510,2511,2512,2513,2514,2515,201,2516,2517,2518,2519,2520,2521,2522,2523,2524,2525,2526,2527,2528,2529,2530,2531,2532,2533,2534,1305,2535,2536,2537,2538,2539,2540,2541,2542,2543,2544,2545,2546,2547,2548,2549,2550,2551,2552,2553,2554,2555,2556,2557,2558,2559,2560,2561,2562,2563,1300,2564,2565,2566,2567,2568,2569,2570,2571,2572,2573,2574,2575,2576,2577,719,1775,1954,2578,1953,2579,2580,2581,2582,2583,2584,2585,2586,2587,2588,2589,2590,2591,2592,2593,2594,2595,2596,2597,2598,2599,2600	[[0,1,1,0,0,0,1,0,0],[0,1,0,0,0,0,1,0,0],[0,1,1,0,0,0,0,0,0],[0,1,1,0,0,1,0,0,0],[0,1,1,0,0,1,1,0,0],[0,1,0,0,0,0,1,0,0],[0,1,0,0,0,0,1,0,0],[0,1,0,0,0,0,0,0,0],[0,0,1,1,1,1,0,0,0],[0,0,0,0,0,0,1,0,0],[0,1,0,0,0,0,1,0,0],[1,1,0,1,0,0,1,0,0],[0,1,1,0,0,0,1,0,0],[0,1,1,0,0,0,1,0,0],[0,1,1,0,0,0,1,0,0],[0,1,1,0,0,0,1,0,0],[1,1,1,1,0,1,0,0,0],[0,0,1,0,0,0,0,0,0],[1,1,1,1,0,1,0,0,0],[0,1,1,0,0,0,1,0,0],[0,1,1,0,0,0,1,0,0],[0,0,1,1,0,0,0,0,0],[0,0,0,1,0,0,0,0,0],[0,1,1,0,0,0,0,0,0],[0,1,0,0,0,1,0,0,0],[0,0,1,0,0,0,0,0,0],[0,0,0,1,0,0,0,0,0],[0,1,1,0,0,0,1,0,0],[0,0,0,1,0,0,0,0,0],[0,0,1,0,0,0,0,0,0],[0,0,1,0,0,0,0,0,0],[0,0,1,0,0,0,0,0,0],[0,1,1,0,1,1,0,0,0],[0,1,1,1,0,0,1,0,0],[0,0,0,1,0,0,0,0,0],[0,0,0,1,0,0,0,0,0],[0,0,1,0,0,0,0,0,0],[0,0,0,0,0,0,1,0,0],[0,0,1,0,0,0,0,0,0],[0,0,1,0,0,0,0,0,0],[0,0,1,0,0,0,0,0,0],[0,0,0,0,0,1,0,0,0],[0,0,1,0,0,0,0,0,0],[0,1,1,0,1,0,1,0,0],[0,1,1,0,1,0,1,0,0],[0,1,0,0,0,0,1,0,0],[0,0,1,0,0,0,0,0,0],[0,0,1,0,0,0,0,0,0],[0,0,1,0,0,0,0,0,0],[0,0,0,1,0,0,0,0,0],[0,0,1,0,0,0,0,0,0],[0,0,0,1,0,0,0,0,0],[0,0,1,0,0,0,0,0,0],[0,1,1,1,0,1,1,0,0],[0,0,1,0,0,0,0,0,0],[0,1,1,0,0,0,1,0,0],[0,0,0,1,0,0,0,0,0],[1,1,1,1,0,0,1,0,0],[0,0,1,0,0,0,0,0,0],[0,0,1,0,0,0,0,0,0],[0,0,1,0,0,0,1,0,0],[0,0,1,1,0,0,0,0,0],[0,1,1,0,1,0,0,0,0],[0,0,1,0,0,1,0,0,0],[0,0,1,0,0,0,0,0,0],[0,1,1,0,0,0,1,0,0],[0,0,0,1,0,0,0,0,0],[0,0,0,1,0,0,0,0,0],[0,0,0,0,0,1,0,0,0],[0,1,1,0,0,0,0,0,0],[0,0,1,0,0,0,0,0,0],[0,1,1,0,0,0,1,0,0],[0,1,1,0,0,0,1,0,0],[0,1,1,0,0,0,0,0,0],[0,1,0,0,0,0,1,0,0],[0,1,1,1,0,0,1,0,0],[0,1,0,0,0,0,0,0,0],[0,1,0,0,0,0,0,0,0],[0,1,1,0,0,0,1,0,0],[0,1,0,0,0,0,0,0,0],[0,1,1,0,0,0,1,0,0],[0,1,1,1,1,1,1,0,0],[1,1,1,0,0,1,1,0,0],[0,0,1,0,0,0,0,0,0],[0,0,1,0,0,0,0,0,0],[0,0,0,0,1,0,0,0,0],[0,1,0,0,0,0,1,0,0],[0,1,1,0,0,0,0,0,0],[0,1,1,0,0,0,0,0,0],[0,0,1,0,0,0,0,0,0],[0,0,0,1,0,0,0,0,0],[0,0,1,0,0,1,0,0,0],[0,0,1,0,0,0,0,0,0],[0,0,1,0,0,0,0,0,0],[0,0,0,1,0,0,0,0,0],[0,0,1,0,0,0,0,0,0],[0,0,0,1,0,0,0,0,0],[1,0,1,1,0,0,0,0,0],[0,0,0,1,0,0,0,0,0],[0,0,1,0,0,0,0,0,0],[0,0,1,1,0,0,0,0,0],[0,0,0,1,0,0,0,0,0],[0,0,1,0,0,0,0,0,0],[0,0,1,0,0,0,0,0,0],[0,0,1,0,0,0,0,0,0],[0,0,1,0,0,0,0,0,0],[0,1,0,0,0,0,1,0,0],[0,0,0,0,0,0,1,0,0],[0,0,0,0,0,0,1,0,0],[0,0,1,0,0,0,0,0,0],[0,0,1,0,0,0,0,0,0],[0,0,0,0,0,0,1,0,0],[0,0,0,1,0,0,0,0,0],[0,0,1,0,0,0,0,0,0],[0,0,0,1,0,0,0,0,0],[1,0,0,1,0,0,0,0,0],[0,0,0,1,1,0,0,0,0],[0,0,1,0,0,0,0,0,0],[0,0,0,0,1,0,0,0,0],[0,0,1,1,0,1,0,0,0],[0,0,1,0,0,0,0,0,0],[0,0,1,0,0,0,0,0,0],[0,0,0,1,0,0,0,0,0],[0,0,0,1,0,0,0,0,0],[0,0,1,0,0,0,0,0,0],[0,0,1,0,0,0,0,0,0],[0,0,1,0,0,0,0,0,0],[0,0,0,0,0,1,0,0,0],[0,0,1,0,0,0,0,0,0],[0,0,1,0,0,0,0,0,0],[0,0,0,1,0,0,0,0,0],[0,0,1,0,0,0,0,0,0],[0,0,1,0,0,0,0,0,0],[0,0,0,0,0,1,0,0,0],[0,0,1,0,0,0,0,0,0],[0,0,1,0,0,0,0,0,0],[0,0,1,0,0,0,0,0,0],[0,0,1,0,0,0,0,0,0],[0,0,0,1,0,0,0,0,0],[0,0,1,0,0,0,0,0,0],[1,0,0,0,0,0,0,0,0],[0,0,1,1,0,0,0,0,0],[0,0,1,0,0,0,0,0,0]]2601	2602,2603,2604,2605,2606,2607,2608,2609,2610,2611,1931,2612,2613,2614,2615,2616,2617,2618,2619,2620,2621,2622,2623,2624,2625,2626,2627,2628	[[0,1,1,1,0,0,0,0,0],[0,0,1,1,0,0,0,0,0],[0,0,0,1,0,0,0,0,0],[0,0,1,1,0,0,0,0,0],[0,0,0,1,0,0,0,0,0],[0,0,1,1,1,0,0,0,0],[0,0,0,1,0,0,0,0,0],[0,0,0,0,1,0,0,0,0],[0,0,1,1,0,0,0,0,0],[0,1,0,1,0,0,0,0,0],[0,0,1,1,0,0,0,0,0],[0,0,1,0,0,0,0,0,0],[0,0,1,0,0,0,0,0,0],[0,0,0,1,0,0,0,0,0],[0,0,0,0,1,0,0,0,0],[0,0,1,0,0,0,0,0,0],[0,0,1,0,0,0,0,0,0],[0,0,1,0,0,0,0,0,0],[0,0,0,0,1,0,0,0,0],[0,0,1,1,0,0,0,0,0],[0,1,0,0,0,0,0,0,0],[0,1,0,0,0,0,0,0,0],[0,1,0,0,0,0,0,0,0],[0,1,0,0,0,0,0,0,0],[0,0,1,0,0,0,0,0,0],[0,1,0,0,0,0,0,0,0],[0,1,0,0,0,0,0,0,0],[0,1,0,0,0,0,0,0,0]]2629	2630,2631,2632,2633,2634,2635,422,2636,2637,1962,2638,2639,2640,2641,2642,2643,2644,2645,2646,2647,2648,2649,2031,2650,2651,2652,2653,2654,2655,2656,2657,2658,2659,2660,2661,2662,2663,2664,2665,2666,2667,2668,2669,2670,2671,1476,2672,2673,2674,2675,2676,2677	[[0,0,0,1,0,0,0,0,0],[0,0,0,1,0,0,0,0,0],[0,0,0,1,0,0,0,0,0],[0,0,0,1,0,0,0,0,0],[0,0,0,1,0,0,0,0,0],[0,0,0,1,0,0,0,0,0],[0,0,0,1,0,0,0,0,0],[0,0,0,1,0,0,0,0,0],[0,0,0,1,0,0,0,0,0],[0,0,0,1,0,0,0,0,0],[0,0,0,1,0,0,0,0,0],[0,0,0,1,0,0,0,0,0],[0,0,0,1,0,0,0,0,0],[0,0,0,1,0,0,0,0,0],[0,0,0,1,0,0,0,0,0],[0,0,0,1,0,0,0,0,0],[0,0,0,1,0,0,0,0,0],[0,0,0,1,0,0,0,0,0],[0,0,0,1,0,0,0,0,0],[0,0,0,1,0,0,0,0,0],[0,0,0,1,0,0,0,0,0],[0,0,0,1,0,0,0,0,0],[0,0,0,1,0,0,0,0,0],[0,0,0,1,0,0,0,0,0],[0,0,0,1,0,0,0,0,0],[0,0,0,1,0,0,0,0,0],[0,0,0,1,0,0,0,0,0],[0,0,0,1,0,0,0,0,0],[0,0,0,1,0,0,0,0,0],[0,0,0,1,0,0,0,0,0],[0,0,0,1,0,0,0,0,0],[0,0,0,1,0,0,0,0,0],[0,0,0,1,0,0,0,0,0],[0,0,0,1,0,0,0,0,0],[0,0,0,1,0,0,0,0,0],[0,0,0,1,0,0,0,0,0],[0,0,0,1,0,0,0,0,0],[0,0,0,1,0,0,0,0,0],[0,0,0,1,0,0,0,0,0],[0,0,0,1,0,0,0,0,0],[0,0,0,1,0,0,0,0,0],[0,0,0,1,0,0,0,0,0],[0,0,0,1,0,0,0,0,0],[0,0,0,1,0,0,0,0,0],[0,0,0,1,0,0,0,0,0],[0,0,0,1,0,0,0,0,0],[0,0,0,1,0,0,0,0,0],[0,0,0,1,0,0,0,0,0],[0,0,0,1,0,0,0,0,0],[0,0,0,1,0,0,0,0,0],[0,0,0,1,0,0,0,0,0],[0,0,0,1,0,0,0,0,0]]2678	2679,2680,2681,2682,2683,2684,2685,2686,2687,2688,2689,2690,2691,2692,2693,2694,2695,2696,2697,2698	[[0,0,1,0,0,0,0,0,0],[0,0,1,0,0,0,0,0,0],[0,0,1,0,0,0,0,0,0],[0,0,1,0,0,0,0,0,0],[0,0,1,0,0,0,0,0,0],[0,0,1,0,0,0,0,0,0],[0,0,1,0,0,0,0,0,0],[0,0,1,0,0,0,0,0,0],[0,0,1,0,0,0,0,0,0],[0,0,1,0,0,0,0,0,0],[0,0,1,0,0,0,0,0,0],[0,0,1,0,0,0,0,0,0],[0,0,0,0,1,0,0,0,0],[0,0,1,0,0,0,0,0,0],[0,0,1,0,0,0,0,0,0],[0,0,1,0,0,0,0,0,0],[0,0,1,0,0,0,0,0,0],[0,0,1,0,0,0,0,0,0],[0,0,1,0,0,0,0,0,0],[0,0,1,0,0,0,0,0,0]]2699	2700,2701,2702,2703,2704,2705,2706,2707,2593,2708,2709,2710,2495,2711,2552,2712,2713,2714,2715,2716,2717,2718,2719,2720,2590,2721,2722,2723,2724,2725,2726,2727,2728,2567,2729,2730,2731,2732,2733,2734,2735,2736,2737,2738,2739,2534	[[0,1,1,0,0,0,1,0,0],[0,0,1,0,0,0,0,0,0],[0,0,0,1,0,0,0,0,0],[0,1,1,0,0,0,1,0,0],[0,1,1,0,0,0,1,0,0],[0,1,0,0,0,0,1,0,0],[0,0,0,1,0,0,0,0,0],[1,1,1,1,0,0,0,0,0],[1,1,1,0,1,0,1,0,0],[0,1,0,1,0,0,1,0,0],[0,0,0,1,0,0,0,0,0],[1,1,1,0,0,0,1,0,0],[0,0,1,0,0,0,0,0,0],[0,0,1,0,0,0,0,0,0],[0,0,1,0,0,0,0,0,0],[0,0,1,0,0,0,0,0,0],[1,1,0,1,0,0,0,0,0],[0,0,1,0,0,0,0,0,0],[0,1,1,0,0,0,1,0,0],[0,1,1,0,0,0,1,0,0],[0,1,1,0,0,0,1,0,0],[0,0,1,0,0,0,0,0,0],[0,0,1,0,0,0,0,0,0],[0,0,1,0,0,0,0,0,0],[1,0,1,0,0,0,0,0,0],[0,0,0,1,0,0,0,0,0],[0,0,0,1,0,0,0,0,0],[0,0,0,1,0,0,0,0,0],[0,0,1,0,0,0,0,0,0],[1,0,0,0,0,0,0,0,0],[1,0,0,0,0,0,0,0,0],[1,0,0,0,0,0,0,0,0],[0,0,0,1,0,0,0,0,0],[1,0,0,0,0,0,0,0,0],[1,0,0,0,0,0,0,0,0],[1,0,0,0,0,0,0,0,0],[1,1,1,1,0,0,0,0,0],[0,0,1,0,0,0,0,0,0],[1,0,0,0,0,0,0,0,0],[1,0,0,0,0,0,0,0,0],[0,0,1,0,0,0,0,0,0],[1,0,0,0,0,0,0,0,0],[0,0,1,0,0,0,0,0,0],[0,0,1,0,0,0,0,0,0],[1,0,0,0,0,0,0,0,0],[1,0,0,0,0,0,0,0,0]]2740	2741,2742,2743,2744,2745,2746,2747,2748,2749,2750	[[0,0,1,1,0,0,0,0,0],[0,0,1,1,0,0,0,0,0],[0,0,1,0,0,0,0,0,0],[0,0,1,1,0,0,0,0,0],[0,0,1,0,0,0,0,0,0],[0,0,1,1,0,0,0,0,0],[0,0,1,0,0,0,0,0,0],[0,0,1,0,0,0,0,0,0],[0,0,1,0,0,0,0,0,0],[0,0,1,0,0,0,0,0,0]]2751	2752,2753	[[0,0,0,0,1,0,0,0,0],[0,0,0,0,1,0,0,0,0]]2754	2755,2756,2757,2758,2759,2760,2761,2762,2763,2764,2765,2766,2767,2768,2769,2770,2771,2772,2773	[[0,0,1,0,0,0,0,0,0],[0,0,1,0,0,0,0,1,0],[0,0,1,0,0,0,0,1,0],[0,0,1,0,0,0,0,0,0],[0,0,1,1,0,0,0,1,0],[0,0,1,0,0,0,0,0,0],[0,0,0,1,0,0,0,0,0],[0,0,0,1,1,0,0,0,0],[0,0,1,0,0,0,0,0,0],[0,0,1,1,0,0,0,1,0],[0,0,1,0,0,0,0,0,0],[0,0,1,0,0,0,0,0,0],[0,0,1,0,0,0,0,0,0],[0,0,1,0,0,0,0,0,0],[0,0,0,1,0,0,0,0,0],[0,0,1,0,0,0,0,1,0],[0,0,0,0,0,0,0,1,0],[0,0,1,0,0,0,0,0,0],[0,0,1,0,0,0,0,0,0]]2774	2775	[[0,0,0,0,1,0,0,0,0]]2776	2777,2778	[[0,0,0,0,1,0,0,0,0],[0,0,0,0,1,0,0,0,0]]2779	1403,2645,2661,2677,2651,2780,2781,2633,2652,2782,2656,2783,2654,2784,2785,2630,2786,257,2631,2637,2664,2787,2788,2669,2789,2790,2791,2668,2639,2660,2674,1814,2636,2657,1962,2662,2792,2644,2793,2794,2795,2796,2663,2797,2798,2799,2648,2800,2655,2801,2802,2803,2804,2031,2805,425,2806,2807,2808,2809,261,2810,2646,422,2811,2650,2812,2813,2814,2815,2816,2817,2818,2819,1136,2643,2667,2641,2653,2676,2673,2820,2632,2634,2675,2821,2649,2822,2672,2659,2642,2658,2638,2823,2635,2670,2647,2665,2666,2671,1476,2824,2640	[[1,0,0,0,0,0,0,0,0],[1,1,1,1,0,0,1,1,1],[0,0,0,1,0,0,0,0,0],[1,1,0,1,0,0,1,1,1],[1,1,1,1,1,0,1,0,1],[1,0,0,0,0,0,0,0,0],[0,0,1,0,0,0,0,0,0],[0,0,0,1,0,0,0,0,0],[1,0,1,1,0,0,0,0,1],[0,1,0,0,0,0,1,0,0],[1,1,1,1,0,0,1,0,1],[1,0,0,0,0,0,0,0,0],[1,0,0,1,0,0,0,0,0],[1,0,0,0,1,0,0,0,0],[0,1,0,0,0,0,0,0,0],[0,0,0,1,0,0,0,0,0],[1,0,0,0,0,0,0,0,0],[1,0,0,0,0,0,0,0,0],[1,1,1,1,0,0,1,1,1],[1,1,1,1,0,0,1,0,1],[1,0,0,1,0,0,0,1,0],[0,0,1,0,0,0,0,0,0],[1,1,0,0,0,0,0,0,0],[1,0,0,1,0,0,0,0,0],[0,0,1,0,0,0,0,0,0],[1,0,1,0,0,0,0,0,0],[0,0,1,0,0,0,0,0,0],[1,0,0,1,0,0,0,0,0],[0,0,1,1,0,0,0,0,0],[1,1,1,1,0,0,1,0,1],[1,1,1,1,0,0,1,1,1],[1,0,0,0,0,0,0,0,0],[1,0,0,1,0,0,0,1,0],[0,0,0,1,0,0,0,1,0],[1,0,0,1,0,0,0,0,0],[0,0,1,1,0,0,0,1,0],[0,1,0,0,0,0,0,0,0],[1,1,1,1,0,0,1,1,1],[1,0,0,0,0,0,0,0,0],[1,0,0,0,0,0,0,0,0],[1,0,0,0,0,0,0,0,0],[1,0,0,0,0,0,0,0,0],[0,1,0,1,0,0,1,0,0],[1,0,0,0,0,0,0,0,0],[1,0,0,0,0,0,0,0,0],[1,0,0,0,0,0,0,0,0],[1,0,0,1,0,0,0,1,0],[1,0,0,0,0,0,0,0,0],[0,0,1,1,0,0,0,1,0],[1,0,0,0,0,0,0,0,0],[1,0,0,0,0,0,0,0,0],[0,1,0,0,0,0,0,0,0],[1,0,0,0,0,0,0,0,0],[1,0,0,1,0,0,0,1,0],[1,0,0,0,0,0,0,0,0],[1,0,0,0,1,0,0,0,0],[1,0,0,0,0,0,0,0,0],[0,1,0,0,0,0,0,0,0],[1,0,0,0,0,0,0,0,0],[1,0,0,0,0,0,0,0,0],[1,0,0,0,0,0,0,0,0],[1,0,0,0,0,0,0,1,0],[0,1,1,1,0,0,0,1,0],[1,0,0,1,1,0,0,1,0],[1,0,1,0,0,0,0,0,0],[0,1,1,1,0,0,1,0,0],[1,0,0,0,0,0,0,0,0],[1,0,0,0,0,0,0,0,0],[1,0,0,0,0,0,0,0,0],[1,0,0,0,0,0,0,0,0],[1,0,0,0,0,0,0,0,0],[1,0,0,0,0,0,0,0,0],[1,0,0,0,0,0,0,0,0],[1,0,0,0,0,0,0,0,0],[1,0,0,0,0,0,0,0,0],[0,0,1,1,0,0,0,1,0],[0,0,0,1,0,0,0,0,0],[0,0,0,1,0,0,0,0,0],[0,0,1,1,0,0,0,1,0],[0,0,0,1,0,0,0,0,0],[0,0,0,1,0,0,0,0,0],[0,0,0,0,1,0,0,0,0],[0,0,1,1,0,0,0,0,0],[0,0,0,1,0,0,0,0,0],[0,0,0,1,0,0,0,0,0],[0,0,1,0,0,0,0,0,0],[0,0,0,1,0,0,0,0,0],[0,0,0,0,0,0,0,1,0],[0,0,0,1,0,0,0,1,0],[0,0,1,1,0,0,0,0,0],[0,0,0,1,0,0,0,0,0],[0,0,1,1,0,0,0,1,0],[0,0,1,1,0,0,0,0,0],[0,0,0,0,0,0,1,0,0],[0,0,0,1,0,0,0,0,0],[0,0,1,1,0,0,0,1,0],[0,0,0,1,0,0,0,1,0],[0,0,0,1,0,0,0,1,0],[0,0,0,1,0,0,0,0,0],[0,0,0,1,0,0,0,0,0],[0,0,0,1,0,0,0,0,0],[0,0,1,0,0,0,0,0,0],[0,0,0,1,0,0,0,0,0]]2825	2826,2827,350,2828,1525,2829,2830,280,2831,355,2832,1874,1873,2833,2834,2006,2835,2836,2837,349,348,353,351,356,354,2838,138,1868,2839,2840,2841,1867,2842,1886,1885,2843,1865,2844,2845,2846,2847,2848,2849,1878,2850,352,2851,2852,2853,2854,1880,1881,1891,2855,900,2856,2857,2858,2859,1515,347	[[0,1,0,0,0,0,0,0,0],[0,1,0,0,0,0,0,0,0],[0,1,1,0,0,0,0,0,0],[0,1,1,1,0,0,0,0,0],[0,1,1,0,0,0,0,1,0],[0,0,0,1,0,0,0,1,0],[0,0,1,0,0,0,0,0,0],[0,0,1,0,0,0,0,0,0],[0,0,0,0,0,0,0,1,0],[0,1,0,0,0,0,0,0,0],[0,1,1,0,0,0,0,1,0],[0,1,1,1,0,0,0,1,0],[0,0,0,1,0,0,0,0,0],[0,0,1,0,0,0,0,0,0],[0,0,1,0,0,0,0,0,0],[0,1,0,0,0,0,0,0,0],[0,1,0,0,0,0,0,1,0],[0,1,0,0,0,0,0,0,0],[0,0,0,0,0,0,0,1,0],[0,1,0,0,0,0,0,0,0],[0,1,1,0,0,0,0,0,0],[0,1,0,0,0,0,0,0,0],[0,1,0,0,0,0,0,0,0],[0,1,1,0,0,0,0,0,0],[0,1,1,1,0,0,0,0,0],[0,1,1,0,0,0,0,0,0],[0,1,1,0,0,0,0,0,0],[0,1,0,0,0,0,0,0,0],[0,1,1,0,0,0,0,0,0],[0,1,0,0,0,0,0,0,0],[0,1,0,0,0,0,0,0,0],[0,1,1,0,0,0,0,1,0],[0,1,0,0,0,0,0,0,0],[0,0,1,1,0,0,0,0,0],[0,0,1,0,0,0,0,0,0],[0,0,1,0,0,0,0,0,0],[0,0,1,0,0,0,0,1,0],[0,0,1,0,0,0,0,0,0],[0,0,1,0,0,0,0,0,0],[0,0,1,0,0,0,0,0,0],[0,0,1,0,0,0,0,0,0],[0,0,1,0,0,0,0,0,0],[0,0,1,1,0,0,0,0,0],[0,0,1,0,0,0,0,1,0],[0,0,1,0,0,0,0,0,0],[0,0,1,0,0,0,0,0,0],[0,0,0,0,0,0,0,1,0],[0,0,1,0,0,0,0,0,0],[0,0,1,0,0,0,0,0,0],[0,0,1,0,0,0,0,0,0],[0,0,1,0,0,0,0,0,0],[0,0,1,0,0,0,0,0,0],[0,0,1,0,0,0,0,1,0],[0,0,1,0,0,0,0,0,0],[0,0,1,0,0,0,0,0,0],[0,0,1,0,0,0,0,0,0],[0,0,1,0,0,0,0,0,0],[0,0,0,0,0,0,0,1,0],[0,0,0,0,0,0,0,1,0],[0,0,0,0,0,0,0,1,0],[0,0,1,0,0,0,0,0,0]]2860	2861,2862,2484,2493,2863,2864,2865,2866,2867,2424,2868,2869,2870,2871,2573,1309,2872,2873,2874,2570,2875,2876,2877,2878,2879,2880,2480,2541,2881,2545,2882,2883,2884,2511,2885,2886,2887	[[0,1,0,0,0,0,1,1,0],[0,1,1,1,1,0,1,1,0],[0,1,1,1,1,0,0,1,0],[0,1,1,1,0,0,0,1,0],[0,1,0,0,0,0,0,0,0],[0,1,1,1,0,0,1,1,0],[0,1,1,0,0,0,1,1,0],[0,0,0,0,0,0,0,1,0],[0,1,0,1,0,0,0,0,0],[0,1,0,1,1,0,1,1,0],[0,1,0,0,0,0,0,0,0],[0,1,0,0,0,0,0,0,0],[0,1,0,0,0,0,0,0,0],[0,1,0,0,0,0,0,0,0],[0,1,1,0,0,0,0,1,0],[0,1,1,0,0,0,0,1,0],[0,1,1,0,0,0,0,1,0],[0,1,0,1,0,0,0,1,0],[0,1,1,1,0,0,1,1,0],[0,0,0,0,1,0,1,0,0],[0,0,1,0,0,0,0,0,0],[0,0,1,0,0,0,0,0,0],[0,0,0,0,0,0,0,1,0],[0,0,0,1,0,0,0,0,0],[0,0,0,1,0,0,0,0,0],[0,0,0,1,0,0,0,1,0],[0,0,1,0,1,0,1,0,0],[0,0,0,1,0,0,0,0,0],[0,0,0,1,0,0,0,0,0],[0,0,1,1,0,0,0,1,0],[0,0,0,1,0,0,0,0,0],[0,0,1,0,0,0,0,1,0],[0,0,1,0,0,0,0,0,0],[0,0,0,1,0,0,0,0,0],[0,0,0,1,0,0,0,1,0],[0,0,1,0,0,0,0,1,0],[0,0,1,0,0,0,0,0,0]]2888	2889,2890,2891,2892,306,2602,2893,2894,1931,2617,2895,2604,2896,2897,2898,2899,2900	[[0,0,0,0,1,0,0,1,0],[0,0,0,0,0,0,0,1,0],[0,0,0,0,0,0,0,1,0],[0,0,0,0,0,0,0,1,0],[0,0,1,0,0,0,0,0,0],[0,0,1,0,0,0,0,1,0],[0,0,1,0,0,0,0,1,0],[0,0,1,0,1,0,0,1,0],[0,0,1,0,1,0,0,0,0],[0,0,1,0,1,0,0,0,0],[0,0,0,0,1,0,0,1,0],[0,0,1,0,0,0,0,0,0],[0,0,1,0,0,0,0,0,0],[0,0,0,0,0,0,0,1,0],[0,0,0,0,1,0,0,0,0],[0,0,1,0,1,0,0,0,0],[0,0,1,0,1,0,0,1,0]]2901	2902	[[0,0,0,0,1,0,0,0,0]]2903	2904	[[0,0,0,0,1,0,0,0,0]]2905	2906	[[0,0,0,0,1,0,0,0,0]]2907	2908,2909,2910,2911,2912,2913,2914,746,2915,2916,2917,7,2918,2919,1930,2920,2921,2922,2923,2924,91,2925,2926,898,2927,2928,2929,2930,2931,2932,2933,2934,2935,2936,2937,2938,2939,2940,2941,2942,2943,2944,2945,2946,2947,2948,2949,2950	[[0,0,1,0,0,0,0,0,0],[0,0,1,0,0,0,0,0,0],[0,1,1,0,0,0,0,0,0],[0,0,1,0,0,0,0,0,0],[0,1,1,0,0,0,0,0,0],[0,1,1,0,0,0,0,0,0],[0,0,1,0,0,0,0,0,0],[0,0,1,0,0,0,0,0,0],[0,0,1,0,0,0,0,0,0],[0,0,1,0,0,0,0,0,0],[0,0,1,0,0,0,0,0,0],[0,0,1,0,1,0,0,0,0],[0,0,1,0,0,0,0,0,0],[0,0,1,0,0,0,0,0,0],[0,0,1,0,0,0,0,0,0],[0,0,1,0,1,0,0,0,0],[0,0,1,0,0,0,0,0,0],[0,0,1,0,0,0,0,0,0],[0,0,1,1,1,0,0,0,0],[0,0,1,0,0,0,0,0,0],[0,0,1,0,0,0,0,0,0],[0,0,1,0,0,0,0,0,0],[0,0,1,0,0,0,0,0,0],[0,0,1,0,0,0,0,0,0],[0,0,1,0,0,0,0,0,0],[0,0,1,0,0,0,0,0,0],[0,0,1,0,0,0,0,0,0],[0,0,1,0,0,0,0,0,0],[0,0,1,0,0,0,0,0,0],[0,1,1,0,0,0,0,0,0],[0,1,1,0,1,0,0,0,0],[0,0,1,0,0,0,0,0,0],[0,0,1,0,0,0,0,0,0],[0,0,1,0,0,0,0,0,0],[0,0,1,0,0,0,0,0,0],[0,0,1,0,0,0,0,0,0],[0,0,0,0,1,0,0,0,0],[0,0,1,0,0,0,0,0,0],[0,0,1,0,0,0,0,0,0],[0,0,1,0,0,0,0,0,0],[0,0,1,0,0,0,0,0,0],[0,0,1,0,0,0,0,0,0],[0,0,1,0,0,0,0,0,0],[0,1,0,0,0,0,0,0,0],[0,0,1,0,0,0,0,0,0],[0,0,1,0,0,0,0,0,0],[0,0,1,0,1,0,0,0,0],[0,0,1,0,0,0,0,0,0]]2951	2952,2953	[[0,0,0,0,1,0,0,0,0],[0,0,0,0,1,0,0,0,0]]2954	2955,2956,2957	[[0,0,0,0,1,0,0,0,0],[0,0,0,0,1,0,0,0,0],[0,0,0,0,1,0,0,0,0]]2958	2959	[[0,0,0,0,1,0,0,0,0]]2960	2961	[[0,0,0,0,1,0,0,0,0]]2962	2963,2964,2965,2966,2967,2968,2969,2970,2030,2971,2972	[[0,0,0,0,1,0,0,0,0],[0,1,0,0,0,0,0,0,0],[0,1,0,0,0,0,0,0,0],[0,1,0,0,0,0,0,0,0],[0,1,0,0,0,0,0,0,0],[0,1,0,0,0,0,0,0,0],[0,1,0,0,0,0,0,0,0],[0,1,0,0,0,0,0,0,0],[0,1,0,0,1,0,0,0,0],[0,1,0,0,0,0,0,0,0],[0,1,0,0,0,0,0,0,0]]2973	2974,2975,1900,2976,2977,2978,2979,2980,2981,2982,2983,2984,2985,2986,2987,2988,2989,2990,2991,2992,2993,2994,2995	[[0,1,1,1,0,0,0,1,0],[0,1,0,1,0,0,0,1,0],[0,1,0,1,0,0,0,0,0],[0,0,1,0,0,0,0,0,0],[0,0,0,1,0,0,0,1,0],[0,0,1,0,0,0,0,0,0],[0,0,0,1,0,0,0,0,0],[0,1,1,1,0,0,0,1,0],[0,0,0,1,0,0,0,0,0],[0,1,0,0,0,0,0,1,0],[0,0,1,0,0,0,0,0,0],[0,0,1,0,0,0,0,0,0],[0,0,1,1,0,0,0,1,0],[0,0,1,0,0,0,0,0,0],[0,1,0,0,0,0,0,0,0],[0,0,0,1,0,0,0,1,0],[0,0,0,1,0,0,0,1,0],[0,0,0,1,0,0,0,0,0],[0,0,0,1,0,0,0,1,0],[0,0,0,1,0,0,0,1,0],[0,0,0,1,0,0,0,0,0],[0,0,1,0,0,0,0,0,0],[0,0,1,1,0,0,0,0,0]]2996	2997,2998,2999,3000,3001,3002,3003,3004,1826,3005,3006,3007,3008,3009,3010,3011,3012,3013,3014,3015,1798,3016,3017,3018,3019,3020,3021,3022,3023,3024,3025,3026,2595,3027,3028,2490,3029,3030,3031,3032,2722	[[0,1,0,1,0,0,1,0,0],[0,0,1,1,0,0,0,0,1],[0,0,1,1,0,0,0,0,1],[0,0,1,0,0,0,0,0,0],[0,0,1,1,0,0,0,0,1],[0,1,1,1,0,0,1,0,0],[0,0,0,1,0,0,0,0,0],[0,0,1,0,0,0,0,0,0],[0,0,1,1,0,0,0,0,1],[0,1,1,1,0,0,1,0,0],[0,0,1,1,0,0,0,0,0],[0,0,1,1,0,0,0,0,0],[0,0,1,0,0,0,0,0,0],[0,0,0,1,0,0,0,0,0],[0,0,1,0,0,0,0,0,0],[0,0,0,1,0,0,0,0,0],[0,1,0,0,1,0,0,0,0],[0,1,1,1,0,0,1,0,0],[0,0,1,0,0,0,0,0,0],[0,1,1,1,0,0,1,0,0],[0,0,1,1,0,0,0,0,1],[0,0,1,0,0,0,0,0,0],[0,0,1,0,0,0,0,0,0],[0,1,0,0,0,0,0,0,0],[0,1,1,0,0,0,1,0,0],[0,1,1,0,0,0,0,0,0],[0,0,1,0,0,0,0,0,0],[0,0,0,0,0,0,1,0,0],[0,0,1,0,0,0,0,0,0],[0,0,1,1,0,0,0,0,0],[0,0,1,0,0,0,0,0,0],[0,0,0,1,0,0,0,0,1],[0,0,1,0,0,0,0,0,1],[0,0,1,0,0,0,0,0,1],[0,0,0,0,0,0,0,0,1],[0,0,1,1,0,0,0,0,1],[0,0,1,0,0,0,0,0,0],[0,0,0,0,0,0,0,0,1],[0,0,1,0,0,0,0,0,0],[0,0,1,0,0,0,0,0,0],[0,0,0,1,0,0,0,0,0]]3033	3034,3035,3036,3037,3038,3039,3040,3041	[[0,0,0,0,1,0,0,0,0],[0,0,0,0,1,0,0,0,0],[0,0,0,0,1,0,0,0,0],[0,0,0,0,1,0,0,0,0],[0,0,0,0,1,0,0,0,0],[0,0,0,0,1,0,0,0,0],[0,0,0,0,1,0,0,0,0],[0,0,0,0,1,0,0,0,0]]3042	3043	[[0,0,0,0,1,0,0,0,0]]3044	3045	[[0,0,0,0,1,0,0,0,0]]3046	3047,3048,2353,3049,3050,3051,3052,3053,3054,2911,3055,3056,3057,3058,3059,3060,3061,3062,3063,3064,3065,3066,3067,3068,3069	[[0,0,0,1,0,0,0,0,0],[0,0,1,0,0,0,0,0,0],[0,0,0,1,0,0,0,0,0],[0,0,0,1,0,0,0,0,0],[0,0,1,1,0,0,0,0,0],[0,0,1,0,0,0,0,0,0],[0,0,1,0,0,0,0,0,0],[0,1,0,0,1,0,0,0,0],[0,0,1,0,0,0,0,0,0],[0,1,1,0,0,0,0,0,0],[0,0,0,1,0,0,0,0,0],[0,0,0,1,0,0,0,0,0],[0,0,1,1,0,0,0,0,0],[0,0,0,0,1,0,0,0,0],[0,0,1,0,0,0,0,0,0],[0,0,1,0,0,0,0,0,0],[0,0,1,1,0,0,0,0,0],[0,0,0,0,1,0,0,0,0],[0,1,1,0,0,0,0,0,0],[0,0,1,0,0,0,0,0,0],[0,0,1,0,0,0,0,0,0],[0,0,1,0,0,0,0,0,0],[0,1,0,0,0,0,0,0,0],[0,0,0,1,0,0,0,0,0],[0,0,1,0,0,0,0,0,0]]3070	3071	[[0,0,0,0,1,0,0,0,0]]3072	3027,3073,3074,3075,3076,3077,3078,3079,2998,3080,3081,3008,3082,3083,3084	[[0,1,1,0,1,0,1,0,0],[0,0,1,0,0,0,0,0,0],[0,1,1,0,1,0,1,0,0],[0,0,1,0,0,0,0,0,0],[0,1,1,0,1,0,1,0,0],[0,1,1,0,0,0,1,0,0],[0,0,1,0,0,0,0,0,0],[0,1,1,0,0,0,1,0,0],[0,1,1,0,1,0,1,0,0],[0,0,1,0,0,0,0,0,0],[0,0,1,0,0,0,0,0,0],[0,1,1,0,0,0,0,0,0],[0,1,0,0,0,0,1,0,0],[0,0,1,0,0,0,0,0,0],[0,0,0,0,1,0,0,0,0]]3085	3086,3087	[[0,0,0,0,1,0,0,0,0],[0,0,0,0,1,0,0,0,0]]3088	3089	[[0,0,0,0,1,0,0,0,0]]3090	3091,3092,3093,3094,3095,3096,3097,3098,3099,815,3100,3101,3102,3103,3104,3105,3106,3107,3108,3109,3110,3111,3112,3113,3114,3115,3116,3117,3067,3118,3119,3120,3121,3122,3123,797,3124,3125,3126,3127,1143,3128,3129,3130,3131,3132,3133,795,3134,771,3135,3136,3137,3138,3139,3140,3141,3142,3143,3144,1187,3145,3146,3147,3148,3149,3150,3151,3152,3153,3154,3155,3156,3157,3158,3159	[[0,0,1,0,0,0,0,0,1],[0,0,1,0,0,0,0,0,0],[0,0,1,0,0,0,0,0,0],[0,0,1,0,0,0,0,0,0],[0,0,0,0,0,0,0,1,1],[0,0,1,1,0,0,0,1,1],[0,0,1,0,0,0,0,0,0],[0,1,1,1,0,0,1,1,0],[0,0,1,0,0,0,0,1,0],[0,0,1,0,0,0,0,1,0],[1,1,1,1,0,0,1,1,0],[0,0,1,0,0,0,0,0,0],[0,0,0,0,0,0,0,1,0],[0,0,1,0,0,0,0,0,0],[0,0,1,0,0,0,0,0,0],[0,0,1,0,0,0,0,1,0],[0,0,1,0,0,0,0,0,0],[0,1,1,0,0,0,1,1,0],[0,0,0,0,0,0,0,1,0],[0,0,1,0,0,0,0,1,0],[0,0,1,0,0,0,0,0,1],[0,0,1,0,0,0,0,0,0],[0,0,1,1,0,0,0,1,0],[0,0,1,0,0,0,0,0,1],[0,0,1,0,0,0,0,0,0],[0,0,1,0,1,0,0,0,1],[0,0,0,0,1,0,0,0,0],[0,0,1,0,0,0,0,0,0],[0,0,1,0,0,0,0,0,0],[0,0,1,0,0,0,0,0,1],[0,0,0,0,1,0,0,0,0],[1,0,1,1,0,0,0,1,0],[1,0,0,0,0,0,0,0,0],[0,0,1,0,0,0,0,0,0],[0,0,1,0,0,0,0,0,0],[0,0,1,0,0,0,0,0,0],[0,0,1,1,0,0,0,1,0],[0,0,1,0,0,0,0,0,0],[0,0,1,0,0,0,0,0,0],[1,0,1,1,0,0,0,1,0],[0,0,1,0,1,0,0,0,0],[0,0,0,0,0,0,0,0,1],[0,0,1,1,0,0,0,0,0],[0,0,1,0,0,0,0,0,1],[0,0,0,0,1,0,0,0,0],[0,0,1,1,1,0,0,0,0],[0,0,1,0,0,0,0,0,0],[0,0,1,0,0,0,0,0,0],[0,0,1,0,0,0,0,0,0],[0,0,1,0,0,0,0,0,0],[0,0,1,0,0,0,0,1,1],[0,0,1,1,0,0,0,1,1],[0,0,1,0,0,0,0,1,1],[0,0,1,0,0,0,0,0,0],[0,0,1,0,0,0,0,0,0],[0,0,1,0,0,0,0,0,0],[1,0,0,0,0,0,0,0,0],[1,0,0,0,0,0,0,0,0],[0,0,1,1,0,0,0,1,1],[0,0,1,0,0,0,0,0,0],[0,0,1,0,0,0,0,0,0],[0,0,1,0,0,0,0,0,0],[0,0,1,0,0,0,0,0,0],[0,0,1,0,0,0,0,0,0],[0,0,0,0,0,0,0,1,0],[0,0,1,0,0,0,0,0,1],[0,0,1,0,0,0,0,0,0],[0,0,1,0,0,0,0,1,0],[0,0,1,0,0,0,0,0,0],[0,0,1,0,0,0,0,0,0],[0,0,1,0,0,0,0,0,0],[0,0,1,0,0,0,0,0,0],[0,0,1,0,0,0,0,0,0],[0,0,0,0,0,0,0,1,0],[0,0,1,0,0,0,0,0,0],[0,0,0,0,1,0,0,0,0]]3160	3161	[[0,0,0,0,1,0,0,0,0]]3162	2992,2995,2990,2977,2975,2993,2985,2988,2989,2980,2991,2974,2979,1900,2981	[[0,0,0,1,0,0,0,0,0],[0,0,0,1,0,0,0,0,0],[0,0,0,1,0,0,0,0,0],[0,0,0,1,0,0,0,0,0],[0,0,0,1,0,0,0,0,0],[0,0,0,1,0,0,0,0,0],[0,0,0,1,0,0,0,0,0],[0,0,0,1,0,0,0,0,0],[0,0,0,1,0,0,0,0,0],[0,0,0,1,0,0,0,0,0],[0,0,0,1,0,0,0,0,0],[0,0,0,1,0,0,0,0,0],[0,0,0,1,0,0,0,0,0],[0,0,0,1,0,0,0,0,0],[0,0,0,1,0,0,0,0,0]]3163	3164	[[0,0,0,0,1,0,0,0,0]]3165	3166,3167,3168,3169,3170,3171,1733	[[0,0,0,0,1,0,0,0,0],[0,0,0,0,1,0,0,0,0],[0,0,0,0,1,0,0,0,0],[0,0,0,0,1,0,0,0,0],[0,0,0,0,1,0,0,0,0],[0,0,0,0,1,0,0,0,0],[0,0,0,0,1,0,0,0,0]]3172	3173	[[0,0,0,0,1,0,0,0,0]]3174	3175,3176,3177	[[0,0,0,1,0,0,0,0,0],[0,0,0,1,0,0,0,0,0],[0,0,0,1,0,0,0,0,0]]3178	3179,3180,3181,3182,431,3183,3184,3185,3186,3187,3188,3189,3190,3191,3192,3193,3194,3195,3196,3197,3198,3199,3200,3201,3202,3203,3204,3205,3206,3207,3208	[[0,1,0,0,0,0,1,0,0],[0,1,1,0,0,0,1,0,0],[0,1,1,0,0,0,1,0,0],[0,1,1,0,0,0,1,0,0],[0,1,1,1,0,0,1,0,0],[0,1,1,0,0,0,1,0,0],[0,0,1,0,0,0,0,0,0],[0,0,1,0,0,0,0,0,0],[0,0,1,0,0,0,0,0,0],[0,1,0,0,0,0,1,0,0],[0,0,1,0,0,0,0,0,0],[0,1,1,1,0,0,0,0,0],[0,1,0,0,1,0,1,0,0],[0,1,0,0,0,0,0,0,0],[0,0,1,1,0,0,0,0,0],[0,0,1,0,0,0,0,0,0],[0,0,1,0,0,0,0,0,0],[0,1,0,0,0,0,0,0,0],[0,1,0,0,0,0,0,0,0],[0,1,0,0,0,0,1,0,0],[0,0,1,0,0,0,0,0,0],[0,0,1,0,0,0,0,0,0],[0,0,1,0,0,0,0,0,0],[0,0,1,0,0,0,0,0,0],[0,0,1,0,0,0,0,0,0],[0,0,1,0,0,0,0,0,0],[0,0,1,0,0,0,0,0,0],[0,0,1,0,0,0,0,0,0],[0,0,1,0,0,0,0,0,0],[0,0,1,0,0,0,0,0,0],[0,0,1,0,0,0,0,0,0]]3209	3210	[[0,0,0,0,1,0,0,0,0]]3211	3212,2372,3213,3214,3215,3216,3217,3218,2152,3219,3220,632,3221,3222,3223,3224,3225,3226,3227,3228,3229,3230,3231,3232,3233,2129,3234,3235,2711,3236,3237,3238,3239,3240	[[1,0,0,0,0,0,0,0,0],[1,0,0,0,0,0,0,0,0],[1,0,0,0,0,0,0,0,0],[1,0,0,0,0,0,0,0,0],[1,0,0,0,0,0,0,0,0],[1,0,0,0,0,0,0,0,0],[1,0,0,0,0,0,0,0,0],[1,0,1,0,0,0,0,1,0],[1,0,1,0,0,0,0,1,0],[1,0,0,0,0,0,0,1,0],[0,0,0,0,0,0,0,1,0],[1,0,1,0,0,0,0,1,0],[0,1,0,0,0,0,1,0,0],[0,1,0,0,0,0,0,0,0],[0,1,0,0,0,0,0,0,0],[0,1,1,0,0,0,0,0,0],[0,1,1,0,0,0,0,0,0],[0,1,0,0,0,0,1,0,0],[0,1,0,0,0,0,1,0,0],[1,0,1,0,0,0,0,1,0],[0,0,0,0,0,0,0,1,0],[0,0,0,0,0,0,0,1,0],[1,0,1,0,0,0,0,0,0],[1,0,1,0,0,0,0,1,0],[0,0,0,0,1,0,0,0,0],[1,0,1,0,0,0,0,1,0],[0,0,0,0,1,0,0,0,0],[0,0,1,0,0,0,0,1,0],[1,0,0,0,0,0,0,0,0],[1,0,0,0,0,0,0,0,0],[1,0,0,0,0,0,0,0,0],[1,0,0,0,0,0,0,0,0],[1,0,0,0,0,0,0,0,0],[1,0,0,0,0,0,0,0,0]]3241	1961,3242,3243,3244,1962,3245	[[0,0,0,1,0,0,0,0,0],[0,0,0,0,1,0,0,0,0],[0,0,0,0,1,0,0,0,0],[0,0,0,0,1,0,0,0,0],[0,0,0,1,0,0,0,0,0],[0,0,0,0,1,0,0,0,0]]3246	2664,3247,3248	[[0,0,0,1,0,0,0,0,0],[0,0,0,1,0,0,0,0,0],[0,0,0,1,0,0,0,0,0]]3249	3250,3251	[[0,0,0,0,1,0,0,0,0],[0,0,0,0,1,0,0,0,0]]3252	3253	[[0,0,0,0,1,0,0,0,0]]3254	3255,3256,3257,3258,3259,3260,3261,3262,3185,3263,3264,3265,3266,3267,3268,3269,3270,3271,3272,3273,3274,3275,3276,3277,3278,3236,3279,3280,3281,3282,3237,3283,3284,3285,69,3286,3287,3288,3289,3290,3291,3292,3293,3294,3295,3296,3297,3298,3299,3300,3301,3302,3303	[[1,0,1,0,0,0,0,0,0],[1,1,1,1,0,0,1,0,0],[0,1,0,0,0,0,1,0,0],[1,1,1,0,0,0,1,0,0],[1,1,1,0,1,0,1,0,0],[0,0,1,0,0,0,1,0,0],[1,1,1,1,0,0,1,0,0],[1,0,1,0,0,0,0,0,0],[1,0,1,0,0,0,0,0,0],[1,1,0,0,0,0,1,0,0],[1,0,0,0,0,0,0,0,0],[0,1,0,0,0,0,1,0,0],[1,1,1,0,1,0,1,0,0],[1,0,0,0,0,0,0,0,0],[1,0,0,0,0,0,0,0,0],[1,1,1,0,0,0,1,0,0],[1,1,0,0,0,0,1,0,0],[0,1,1,0,0,0,1,0,0],[0,0,1,0,0,0,0,0,0],[1,0,1,0,0,0,0,0,0],[1,0,0,0,0,0,0,0,0],[1,0,0,0,0,0,0,0,0],[1,0,0,0,0,0,0,0,0],[0,0,0,0,1,0,0,0,0],[1,0,0,0,0,0,0,0,0],[1,0,0,0,0,0,0,0,0],[1,0,1,0,0,0,0,0,0],[0,1,0,0,0,0,0,0,0],[1,0,0,0,0,0,0,0,0],[1,0,0,0,0,0,0,0,0],[1,1,1,0,0,0,1,0,0],[0,1,0,0,0,0,1,0,0],[0,0,1,0,1,0,0,0,0],[0,0,1,0,0,0,0,0,0],[0,0,0,0,1,0,0,0,0],[0,0,0,0,1,0,1,0,0],[1,0,0,0,0,0,0,0,0],[1,0,0,0,0,0,0,0,0],[1,0,0,0,0,0,0,0,0],[1,0,0,0,0,0,0,0,0],[1,0,0,0,0,0,0,0,0],[1,1,0,0,0,0,0,0,0],[1,0,0,0,0,0,0,0,0],[1,0,0,0,0,0,0,0,0],[1,0,0,0,0,0,0,0,0],[0,1,0,0,0,0,1,0,0],[1,0,0,0,0,0,0,0,0],[1,0,0,0,0,0,0,0,0],[1,0,0,0,0,0,0,0,0],[1,0,0,0,0,0,0,0,0],[1,0,0,0,0,0,0,0,0],[1,0,0,0,0,0,0,0,0],[1,0,0,0,0,0,0,0,0]]3304	3305,3306	[[0,0,0,0,1,0,0,0,0],[0,0,0,0,1,0,0,0,0]]3307	3308,3309,3310,3311,3312,1880,3313,3314,3315,3316,3317,3318,3319,3320,3321,1863,3322,3323,1878,3324,3325,3326,3327,3328,3185,3329,3330,546,3331,3332,3333,3334,3335,3336,3337,3338,3339,3340,3341,3342,3343,3344,3345	[[0,0,0,1,0,0,0,0,0],[0,0,1,0,0,0,0,0,0],[0,0,1,0,0,0,0,0,0],[0,0,1,1,0,0,0,0,0],[0,0,1,0,0,0,0,0,0],[0,0,1,0,0,0,0,0,0],[0,0,1,0,0,0,0,0,0],[0,0,1,0,0,0,0,0,0],[0,0,1,0,0,0,0,0,0],[0,0,1,0,0,0,0,0,0],[0,0,1,0,0,0,0,0,0],[0,0,1,0,0,0,0,0,0],[0,0,1,0,0,0,0,0,0],[0,0,1,1,0,0,0,0,0],[0,0,1,0,0,0,0,0,0],[0,0,1,0,0,0,0,0,0],[0,0,1,0,0,0,0,0,0],[0,0,1,0,0,0,0,0,0],[0,0,1,0,0,0,0,0,0],[0,0,1,1,0,0,0,0,0],[0,0,1,0,0,0,0,0,0],[0,0,1,0,0,0,0,0,0],[0,0,1,0,0,0,0,0,0],[0,0,1,1,0,0,0,0,0],[0,0,1,0,0,0,0,0,0],[0,0,0,1,0,0,0,0,0],[0,0,1,1,0,0,0,0,0],[0,0,1,0,0,0,0,0,0],[0,0,1,0,0,0,0,0,0],[0,0,1,0,0,0,0,0,0],[0,0,0,1,0,0,0,0,0],[0,0,1,0,0,0,0,0,0],[0,0,1,0,0,0,0,0,0],[0,0,1,1,1,0,0,0,0],[0,0,1,0,0,0,0,0,0],[0,0,1,1,0,0,0,0,0],[0,0,1,0,0,0,0,0,0],[0,0,1,0,0,0,0,0,0],[0,0,1,0,0,0,0,0,0],[0,0,0,1,0,0,0,0,0],[0,0,1,0,0,0,0,0,0],[0,0,1,1,0,0,0,0,0],[0,0,1,0,0,0,0,0,0]]3346	3347,3348,3177,3349,3350,3351,3352,3353,1625,3354,1591,3355,3356,3357,3175,3358,3359,3360,3361,3362,3363,3364,3176,3365,3366,3367,3368,3369,3370,3371,3372,3373,3374,3375,3376,3377,3378,3379,3380,3381,3382,3383,3384,3385,3386,3387,3388,1571,3389,3390,3391,3392,3393,3394,3395,3396,3397	[[0,0,1,0,0,0,0,0,1],[0,0,0,0,1,0,0,0,0],[0,1,1,1,0,0,0,1,0],[0,0,1,0,0,0,0,0,0],[0,0,1,0,1,0,0,1,0],[0,0,0,0,0,0,0,0,1],[0,0,1,0,0,0,0,0,1],[0,0,1,0,0,0,0,0,1],[0,0,0,0,0,0,0,1,0],[0,0,0,0,0,0,0,1,0],[0,1,1,0,0,0,0,0,0],[0,0,1,0,0,0,0,0,1],[0,0,0,0,0,0,0,0,1],[0,0,0,0,0,0,0,1,0],[0,1,1,1,0,0,0,1,1],[0,0,1,0,0,0,0,0,0],[0,1,0,0,0,0,0,0,0],[0,1,1,0,0,0,0,1,0],[0,1,1,0,0,0,0,0,0],[0,1,0,0,0,0,0,0,0],[0,1,0,0,0,0,0,0,0],[0,1,1,0,0,0,0,1,0],[0,1,1,1,0,0,0,1,1],[0,0,1,0,0,0,0,0,0],[0,0,1,0,0,0,0,1,0],[0,0,1,0,0,0,0,0,0],[0,1,1,0,0,0,0,0,0],[0,0,0,0,0,0,0,1,0],[0,1,1,0,0,0,0,0,0],[0,0,1,0,0,0,0,1,0],[0,0,1,0,0,0,0,0,0],[0,0,0,0,0,0,0,0,1],[0,0,1,0,0,0,0,1,0],[0,0,1,0,0,0,0,0,0],[0,0,1,0,0,0,0,0,0],[0,0,1,0,0,0,0,0,0],[0,0,1,0,0,0,0,0,0],[0,0,1,0,0,0,0,0,0],[0,0,1,0,0,0,0,0,0],[0,0,0,0,0,0,0,1,0],[0,0,1,0,0,0,0,0,0],[0,0,1,0,0,0,0,0,0],[0,0,0,0,1,0,0,0,0],[0,0,1,0,0,0,0,0,0],[0,0,1,0,0,0,0,0,0],[0,0,0,0,0,0,0,1,0],[0,0,0,0,0,0,0,1,0],[0,0,0,0,0,0,0,1,0],[0,0,1,0,0,0,0,0,0],[0,0,1,0,0,0,0,0,0],[0,0,1,0,0,0,0,0,0],[0,0,1,0,0,0,0,0,0],[0,0,1,0,0,0,0,0,0],[0,0,1,0,0,0,0,0,0],[0,0,1,0,0,0,0,0,0],[0,0,1,0,0,0,0,0,0],[0,0,1,0,0,0,0,0,0]]3398	3399	[[0,0,0,0,1,0,0,0,0]]3400	3401,3402	[[0,0,0,1,0,0,0,0,0],[0,0,0,1,0,0,0,0,0]]3403	3404	[[0,0,0,0,1,0,0,0,0]]3405	3406	[[0,0,0,0,1,0,0,0,0]]3407	3408,1363,1361,370,369,3409,3410,3411,2428,3412,1356,3413,3414,3415,3416,391,3417,3418,3419,3420,3421,2549	[[0,0,1,0,0,0,0,0,0],[0,0,1,0,0,0,0,0,0],[0,0,1,0,0,0,0,0,0],[0,0,1,1,0,0,0,0,0],[0,0,1,0,0,0,0,0,0],[0,0,1,0,0,0,0,0,0],[0,0,1,1,0,0,0,0,0],[0,0,1,0,0,0,0,0,0],[0,0,1,0,0,0,0,0,0],[0,0,1,0,0,0,0,0,0],[0,0,1,0,0,0,0,0,0],[0,0,1,0,0,0,0,0,0],[0,0,1,1,0,0,0,0,0],[0,0,1,0,0,0,0,0,0],[0,0,1,0,0,0,0,0,0],[0,0,1,0,0,0,0,0,0],[0,0,1,0,0,0,0,0,0],[0,0,1,0,0,0,0,0,0],[0,0,1,0,0,0,0,0,0],[0,0,1,0,0,0,0,0,0],[0,0,1,0,0,0,0,0,0],[0,0,1,0,0,0,0,0,0]]3422	3423,3424,3425,3426,3427,3428,3429,3430,620,3431,3432,619,3433,3434,310,3435,3436,1674,3437,3438,3439,3440,262,3441,3442,3443,3444,3445,3446,3447,3448,3449,3450,1042,668,3451	[[1,0,0,0,0,0,0,0,0],[1,0,0,0,0,0,0,1,0],[0,1,0,0,0,0,0,0,0],[1,1,1,0,0,1,0,0,1],[0,0,1,0,0,0,0,0,0],[0,1,1,1,0,0,0,1,0],[1,0,1,0,1,1,0,1,1],[0,0,1,0,0,0,0,1,0],[1,0,0,0,0,0,0,0,0],[0,0,1,0,0,0,0,0,0],[1,1,1,1,0,0,0,1,1],[1,0,0,0,0,0,0,0,0],[1,0,0,0,0,0,0,0,0],[1,0,0,0,0,0,0,0,0],[1,0,1,1,0,1,0,1,1],[1,0,1,0,0,0,0,1,1],[1,0,1,0,1,0,0,1,0],[1,0,0,0,0,0,0,0,0],[1,0,1,0,0,0,0,0,1],[1,0,0,0,0,0,0,0,0],[1,0,0,0,0,0,0,0,0],[1,0,1,0,0,0,0,1,0],[1,0,0,0,0,0,0,0,0],[1,0,0,0,0,0,0,0,0],[1,0,0,0,0,0,0,0,0],[1,0,0,0,0,0,0,0,0],[1,0,0,0,0,0,0,1,0],[1,0,0,0,0,0,0,0,0],[0,0,0,0,1,0,0,0,0],[0,0,0,0,0,1,0,0,1],[0,0,1,0,0,0,0,0,0],[0,0,1,0,0,0,0,1,0],[0,0,0,0,0,1,0,0,1],[0,0,1,0,0,0,0,0,0],[0,0,0,0,1,0,0,0,0],[0,0,0,0,0,0,0,1,0]]3452	3453,3454,3455,3456,3457,3458,3459,3460,3461,3462	[[0,0,1,0,0,0,0,0,0],[0,0,1,0,0,0,0,0,0],[0,0,1,0,0,0,0,0,0],[0,0,1,0,0,0,0,0,0],[0,0,1,0,0,0,0,0,0],[0,0,0,1,0,0,0,0,0],[0,0,0,1,0,0,0,0,0],[0,0,0,0,1,0,0,0,0],[0,0,0,1,0,0,0,0,0],[0,0,0,1,0,0,0,0,0]]3463	3464,3465,3466,3467	[[0,0,0,0,1,0,0,0,0],[0,0,0,0,1,0,0,0,0],[0,0,0,0,1,0,0,0,0],[0,0,0,0,1,0,0,0,0]]3468	3469,3470,3471	[[0,0,0,1,0,0,0,0,0],[0,0,0,1,0,0,0,0,0],[0,0,0,1,0,0,0,0,0]]3472	3473	[[0,0,0,0,1,0,0,0,0]]3474	3475,3476,3477	[[0,0,0,1,0,0,0,0,0],[0,0,0,1,0,0,0,0,0],[0,0,0,1,0,0,0,0,0]]3478	3479	[[0,0,0,0,1,0,0,0,0]]3480	3481,3482,3483,3484,3485,3486,458,3487,3488,3489,3490,3491,3492,3493	[[0,1,0,0,1,0,0,0,0],[0,1,0,0,0,0,0,0,0],[0,0,0,0,1,0,0,0,0],[0,1,0,0,1,0,0,0,0],[0,1,0,0,0,0,0,0,0],[0,0,0,0,1,0,0,0,0],[0,1,0,0,0,0,0,0,0],[0,1,0,0,0,0,0,0,0],[0,0,0,0,1,0,0,0,0],[0,1,0,0,0,0,0,0,0],[0,1,0,0,0,0,0,0,0],[0,1,0,0,0,0,0,0,0],[0,1,0,0,0,0,0,0,0],[0,0,0,0,1,0,0,0,0]]3494	3495,3496,3497	[[0,0,0,0,1,0,0,0,0],[0,0,0,0,1,0,0,0,0],[0,0,0,0,1,0,0,0,0]]3498	3432,310,3428	[[0,0,0,1,0,0,0,0,0],[0,0,0,1,0,0,0,0,0],[0,0,0,1,0,0,0,0,0]]3499	3500,3501,3502,3503,3504	[[0,0,0,0,1,0,0,0,0],[0,0,0,0,1,0,0,0,0],[0,0,0,0,1,0,0,0,0],[0,0,0,0,1,0,0,0,0],[0,0,0,0,1,0,0,0,0]]3505	3506,3507,3508,3509,3510,3511,3512,3513,3514,3515,3516,3517,3471,3518,3519,3520,3470,3521,3522,3523,3524,3525,3526,633,3527,3528,3529,3530,3531,3532,3533,639,3534,3535,3536,3537,3538,3539,3469	[[0,0,0,0,0,0,0,1,0],[0,1,0,0,0,0,0,0,0],[0,0,0,0,0,0,0,1,0],[0,1,0,0,0,0,0,0,0],[0,1,0,0,0,0,0,0,0],[0,0,0,0,0,0,0,1,0],[0,1,1,0,1,0,0,0,0],[0,1,0,0,0,0,0,0,0],[0,1,0,0,0,0,0,0,0],[0,1,0,0,0,0,0,1,0],[0,1,0,0,0,0,0,0,0],[0,1,0,0,0,0,0,1,0],[0,1,0,1,1,0,0,0,0],[0,1,0,0,0,0,0,0,0],[0,1,0,0,0,0,0,0,0],[0,1,0,0,0,0,0,1,0],[0,1,0,1,0,0,0,1,0],[0,1,0,0,0,0,0,0,0],[0,1,0,0,0,0,0,0,0],[0,1,0,0,1,0,0,0,0],[0,1,0,0,0,0,0,0,0],[0,1,0,0,1,0,0,0,0],[0,1,0,0,0,0,0,1,0],[0,0,0,0,0,0,0,1,0],[0,0,0,0,1,0,0,0,0],[0,0,0,0,0,0,0,1,0],[0,1,1,0,0,0,0,0,0],[0,0,0,0,0,0,0,1,0],[0,0,0,0,0,0,0,1,0],[0,0,0,0,0,0,0,1,0],[0,0,0,0,0,0,0,1,0],[0,0,0,0,0,0,0,1,0],[0,0,0,0,0,0,0,1,0],[0,0,0,0,0,0,0,1,0],[0,0,0,0,0,0,0,1,0],[0,0,0,0,0,0,0,1,0],[0,0,0,0,0,0,0,1,0],[0,0,0,0,0,0,0,1,0],[0,0,0,1,0,0,0,0,0]]3540	3541	[[0,0,0,0,1,0,0,0,0]]3542	3543	[[0,0,0,0,1,0,0,0,0]]3544	3475,3477,3476	[[0,0,0,1,0,0,0,0,0],[0,0,0,1,0,0,0,0,0],[0,0,0,1,0,0,0,0,0]]3545	307,3546,3547,3548,494,3549,3550,2519,3551,3552,3553	[[0,1,0,0,0,0,0,0,0],[0,1,0,1,0,0,0,0,0],[0,1,0,1,0,0,0,0,0],[0,1,0,0,0,0,0,0,0],[0,0,0,1,0,0,0,0,0],[0,1,0,0,0,0,0,0,0],[0,0,0,1,0,0,0,0,0],[0,0,0,1,0,0,0,0,0],[0,1,0,0,0,0,0,0,0],[0,0,0,1,0,0,0,0,0],[0,0,0,1,0,0,0,0,0]]3554	3555,3556	[[0,0,0,0,1,0,0,0,0],[0,0,0,0,1,0,0,0,0]]3557	3558,3559,3560,3561,3562,3563,3564,3565,3566,3567,3568,3569,3570,3571,3572,3573,3574,3575,3576,3577,3578	[[0,1,1,0,0,0,0,0,0],[0,1,1,0,0,0,0,0,0],[0,1,1,0,0,0,0,0,0],[0,1,1,0,0,0,0,0,0],[0,0,1,0,0,0,0,0,0],[0,0,1,0,0,0,0,0,0],[0,1,0,0,1,0,0,0,0],[0,0,1,0,0,0,0,0,0],[0,0,1,0,0,0,0,0,0],[0,0,1,0,0,0,0,0,0],[0,1,1,0,0,0,0,0,0],[0,1,1,0,0,0,0,0,0],[0,0,1,0,0,0,0,0,0],[0,1,1,0,0,0,0,0,0],[0,0,1,0,0,0,0,0,0],[0,0,1,0,0,0,0,0,0],[0,0,1,0,0,0,0,0,0],[0,0,1,0,1,0,0,0,0],[0,0,1,0,0,0,0,0,0],[0,0,1,0,0,0,0,0,0],[0,0,1,0,0,0,0,0,0]]3579	3580,3581,3582	[[0,0,0,1,0,0,0,0,0],[0,0,0,1,0,0,0,0,0],[0,0,0,1,0,0,0,0,0]]3583	3584,3585,3586,3587,3588,3589,3590,3591,370,1364,383,3592,3593,3594,3595,3419,3596,3597,3598,3599,3600,3601,3602,3603,3604,3605,3606,3607,3608,3609	[[0,0,1,0,0,0,0,0,0],[0,1,1,0,0,0,1,1,0],[0,1,1,1,1,0,0,1,0],[0,1,1,1,1,0,1,1,0],[0,1,1,0,0,0,0,0,0],[0,0,1,0,0,0,0,0,0],[0,1,0,0,0,0,0,0,0],[0,1,0,0,0,0,1,0,0],[0,1,0,0,0,0,0,0,0],[0,1,0,0,0,0,0,0,0],[0,1,0,0,0,0,0,0,0],[0,1,0,0,0,0,0,0,0],[0,0,0,1,0,0,0,0,0],[0,0,1,0,0,0,0,0,0],[0,0,0,0,0,0,0,1,0],[0,0,0,0,1,0,0,0,0],[0,0,1,0,1,0,0,0,0],[0,1,1,1,1,0,1,1,0],[0,1,1,0,0,0,1,1,0],[0,0,1,0,0,0,0,0,0],[0,0,0,0,0,0,0,1,0],[0,1,1,1,0,0,1,1,0],[0,0,0,1,0,0,0,0,0],[0,0,0,0,1,0,0,0,0],[0,0,1,0,1,0,0,0,0],[0,0,0,0,1,0,0,0,0],[0,0,1,0,0,0,0,0,0],[0,0,1,0,0,0,0,0,0],[0,0,0,0,0,0,0,1,0],[0,0,1,0,0,0,0,0,0]]3610	3611,3612,3613,3614,3615	[[0,0,0,0,1,0,0,0,0],[0,0,0,0,1,0,0,0,0],[0,0,0,0,1,0,0,0,0],[0,0,0,0,1,0,0,0,0],[0,0,0,0,1,0,0,0,0]]3616	3617,3618,3619,3620	[[0,0,0,0,1,0,0,0,0],[0,1,0,0,1,0,0,0,0],[0,1,0,0,0,0,0,0,0],[0,1,0,0,0,0,0,0,0]]3621	3622,3623	[[0,0,0,0,1,0,0,0,0],[0,0,0,0,1,0,0,0,0]]3624	3625,3626	[[0,0,0,0,1,0,0,0,0],[0,0,0,0,1,0,0,0,0]]3627	3628	[[0,0,0,0,1,0,0,0,0]]3629	3630,3631,3632,3633	[[0,0,0,1,0,0,0,0,0],[0,0,0,1,0,0,0,0,0],[0,0,0,1,0,0,0,0,0],[0,0,0,1,0,0,0,0,0]]3634	3635,3636,3637,3638,3639	[[0,0,0,0,1,0,0,0,0],[0,0,0,0,1,0,0,0,0],[0,0,0,0,1,0,0,0,0],[0,0,0,0,1,0,0,0,0],[0,0,0,0,1,0,0,0,0]]3640	3641	[[0,0,0,0,1,0,0,0,0]]3642	3643	[[0,0,0,0,1,0,0,0,0]]3644	3645,3646	[[0,0,0,0,1,0,0,0,0],[0,0,0,0,1,0,0,0,0]]3647	3648,3649	[[0,0,0,0,1,0,0,0,0],[0,0,0,0,1,0,0,0,0]]3650	3651,3652,3653,3654,3655,730,3656,2842,2408,3657,3658,3659,3660,3661,3662,3663,3664,3665,2724,2712,3666,3667,3668,3669,3670,3671,3672,3673,3674,3675,3676,3677,2700,3678,3679,3680,3681,3682,3683,3684,1796,3685,3686,3687,2715,3688,3689,3690,3691,3692,3693,3694,3695,3696,3697,3698,3699,3700,3701,2703,3231,2916,3702,3703,3704,2006,3705,3706,3707,3708,3709,3710,3711,3712,3713,1310,3714,3715,3716,3717,3718,3719,3720,3721,3722,3723,3724,3725,3726,3727,2590,3728,3729,3730,2946,3731,3732,3733,3734,3735,3736,3737,3738,3739,3219,2913,3740,3741,3742,3743,3744,3745,3746,958,3747,3748,2567,3749,3750,3751,3752,3753,3754,3755,3756,1631,3295,3757,3758,3759,3760,3761,3762,3763,3764,3765,2932,3412,3766,3767,3768,3769,1449,2915,3770,3771,3772,3773,3774,1965,423,649,3775,3776,2397,3777,3778,3779,3780,3781,3782,3783,2600,3784	[[0,1,1,0,0,0,0,0,0],[0,1,1,0,0,0,0,0,0],[0,1,1,1,0,0,0,0,0],[0,0,1,0,0,0,0,0,0],[0,1,1,1,0,1,0,0,0],[0,1,1,0,0,0,0,0,0],[0,0,1,0,0,0,0,0,0],[1,1,1,1,1,0,0,0,0],[0,0,1,0,0,0,0,0,0],[0,0,0,1,0,0,0,0,0],[0,1,1,1,1,0,1,0,0],[0,1,0,0,0,0,1,0,0],[0,0,0,0,0,0,1,0,0],[0,0,1,0,0,0,0,0,0],[0,1,1,0,0,0,1,0,0],[1,1,1,0,0,0,0,0,0],[1,1,1,0,1,1,1,0,0],[1,0,1,0,0,0,0,0,0],[1,1,1,0,0,0,1,0,0],[1,1,1,0,0,0,1,0,0],[1,1,1,1,0,0,0,0,0],[0,0,0,1,0,0,0,0,0],[1,0,0,0,0,0,0,0,0],[0,0,0,0,0,1,0,0,0],[0,1,1,0,0,0,1,0,0],[0,1,0,0,0,0,1,0,0],[1,0,0,0,0,0,0,0,0],[0,0,1,0,0,0,0,0,0],[0,0,1,0,0,0,0,0,0],[0,0,1,0,0,1,0,0,0],[1,1,1,0,0,0,0,0,0],[0,0,1,0,0,0,0,0,0],[0,1,1,0,0,0,1,0,0],[0,1,0,0,0,0,0,0,0],[0,0,1,0,0,1,0,0,0],[0,1,1,0,0,1,0,0,0],[0,1,0,0,0,0,0,0,0],[0,0,1,0,0,0,0,0,0],[1,1,0,0,0,0,1,0,0],[1,1,1,0,0,0,1,0,0],[0,1,0,0,0,0,0,0,0],[0,1,0,0,0,0,1,0,0],[0,1,1,0,0,0,0,0,0],[0,1,0,0,0,0,0,0,0],[0,1,1,0,0,0,0,0,0],[0,1,0,1,0,0,1,0,0],[0,0,1,0,0,0,0,0,0],[1,0,0,0,0,0,0,0,0],[0,0,1,0,0,0,0,0,0],[1,0,1,1,0,0,0,0,0],[0,1,1,0,0,0,0,0,0],[0,1,1,0,0,0,0,0,0],[0,0,1,0,0,0,0,0,0],[0,1,1,1,0,1,1,0,0],[0,0,0,1,0,0,0,0,0],[0,0,1,0,0,0,0,0,0],[1,1,1,1,0,0,1,0,0],[0,0,1,0,0,0,0,0,0],[0,0,0,0,0,0,1,0,0],[0,1,1,1,0,1,1,0,0],[0,1,1,0,0,1,0,0,0],[0,0,1,0,0,0,0,0,0],[0,1,1,0,0,1,0,0,0],[1,0,0,1,0,0,0,0,0],[0,0,1,0,0,0,0,0,0],[0,0,1,0,0,0,0,0,0],[0,0,1,0,0,0,0,0,0],[1,0,0,0,0,0,0,0,0],[0,0,1,0,0,0,0,0,0],[0,1,0,1,0,0,1,0,0],[0,0,1,0,0,0,0,0,0],[1,0,0,0,0,0,0,0,0],[1,1,1,1,0,1,0,0,0],[1,1,1,1,1,0,1,0,0],[1,0,0,0,0,0,0,0,0],[0,0,1,0,0,0,0,0,0],[0,0,0,1,0,0,0,0,0],[1,0,0,0,0,0,0,0,0],[0,0,0,1,0,0,0,0,0],[0,0,1,0,0,0,0,0,0],[0,0,1,0,0,0,0,0,0],[0,0,1,0,0,0,0,0,0],[0,0,1,0,0,0,0,0,0],[0,0,1,0,0,0,0,0,0],[0,0,0,1,0,0,0,0,0],[0,0,1,0,0,0,0,0,0],[0,0,1,0,0,0,0,0,0],[0,0,1,0,0,0,0,0,0],[0,0,0,1,0,0,0,0,0],[0,0,1,0,0,0,0,0,0],[0,0,0,0,1,0,0,0,0],[0,0,1,0,0,0,0,0,0],[0,0,1,0,0,0,0,0,0],[0,0,1,0,0,0,0,0,0],[0,0,1,0,0,0,0,0,0],[0,0,1,0,0,0,0,0,0],[0,0,1,0,0,0,0,0,0],[0,0,1,0,0,0,0,0,0],[0,0,1,0,0,0,0,0,0],[0,0,1,0,0,0,0,0,0],[0,0,1,0,0,0,0,0,0],[0,0,1,0,0,1,0,0,0],[0,0,1,0,0,0,0,0,0],[0,1,0,1,0,0,1,0,0],[0,0,1,0,0,0,0,0,0],[0,0,1,0,0,0,0,0,0],[0,0,1,0,0,0,0,0,0],[0,0,1,0,0,0,0,0,0],[0,0,1,0,0,0,0,0,0],[0,1,0,1,0,0,1,0,0],[0,1,1,0,0,1,1,0,0],[0,0,1,0,0,0,0,0,0],[0,0,1,0,0,0,0,0,0],[0,0,1,0,0,0,0,0,0],[0,0,1,0,0,0,0,0,0],[0,0,1,0,0,0,0,0,0],[0,0,1,0,0,1,0,0,0],[0,0,1,0,0,0,0,0,0],[0,1,0,0,0,0,1,0,0],[0,0,1,0,0,1,0,0,0],[0,0,1,0,0,0,0,0,0],[0,0,1,0,0,1,0,0,0],[0,0,1,0,0,0,0,0,0],[0,0,1,0,0,0,0,0,0],[0,1,1,0,0,0,1,0,0],[0,0,1,0,0,0,0,0,0],[0,0,0,1,0,0,0,0,0],[0,1,1,0,0,1,1,0,0],[0,0,1,0,0,0,0,0,0],[0,0,1,0,0,0,0,0,0],[0,0,1,0,0,0,0,0,0],[0,0,1,0,0,0,0,0,0],[0,0,1,0,0,0,0,0,0],[0,0,1,0,0,0,0,0,0],[0,1,1,1,0,0,1,0,0],[0,0,0,0,0,0,1,0,0],[0,0,1,0,0,0,0,0,0],[0,0,0,0,1,0,0,0,0],[0,0,1,0,0,0,0,0,0],[0,1,1,0,0,0,0,0,0],[0,1,0,0,0,0,0,0,0],[0,0,1,0,0,0,0,0,0],[0,1,1,0,0,0,0,0,0],[0,0,1,0,0,0,0,0,0],[0,0,0,0,1,0,0,0,0],[0,1,1,0,0,0,1,0,0],[0,0,1,0,0,0,0,0,0],[0,0,1,0,0,0,0,0,0],[0,0,1,0,0,0,0,0,0],[0,0,1,0,0,0,0,0,0],[0,0,1,0,0,0,0,0,0],[0,0,0,0,0,1,0,0,0],[0,0,1,0,0,0,0,0,0],[0,0,0,1,0,0,0,0,0],[0,0,1,0,0,0,0,0,0],[1,0,0,0,0,0,0,0,0],[0,0,1,0,1,0,0,0,0],[0,0,1,0,0,0,0,0,0],[0,0,0,1,0,0,0,0,0],[0,0,1,0,0,0,0,0,0],[0,0,1,0,0,1,0,0,0],[0,0,1,0,0,0,0,0,0],[0,0,1,0,0,1,0,0,0],[0,0,0,1,0,0,0,0,0]]3785	3786,3787,3788,3789,3790,3791,3792,3793,3794,3795,3796,3797,3798,3799,3800,3801,3802,3803,3804,3805,3806,3807,3808,3809,3810,3811,3812,3813,3814,3815,3816,3817,3818,3819,3820,3821,3822,3823,3824,3825,3826,3827,3828,3829,3830,3831,3832,3833,3834,3835,3836,3837,3838,3839,3840,3841,3842	[[0,0,1,1,0,0,0,1,0],[0,0,1,0,0,0,0,0,0],[0,0,1,1,0,0,0,1,0],[0,0,0,1,0,0,0,1,0],[0,0,1,1,0,0,0,1,0],[0,0,0,0,1,0,0,0,0],[0,0,1,0,0,0,0,0,0],[0,0,1,1,1,0,0,1,0],[0,0,1,1,1,0,0,1,0],[0,0,1,1,0,0,0,1,0],[0,1,1,0,0,0,0,0,0],[0,0,1,1,0,0,0,0,0],[0,0,1,0,0,0,0,1,0],[0,0,1,0,0,0,0,0,0],[0,0,1,0,0,0,0,0,0],[0,0,1,0,0,0,0,0,0],[0,0,1,0,0,0,0,0,0],[0,0,1,0,0,0,0,0,0],[0,0,1,0,0,0,0,0,0],[0,0,1,0,0,0,0,0,0],[0,0,0,1,0,0,0,1,0],[0,0,1,0,0,0,0,0,0],[0,1,1,0,0,0,0,0,0],[0,0,1,0,0,0,0,0,0],[0,0,1,0,0,0,0,0,0],[0,0,0,0,1,0,0,0,0],[0,0,1,0,0,0,0,0,0],[0,0,1,0,0,0,0,0,0],[0,0,1,1,1,0,0,1,0],[0,0,1,1,0,0,0,0,0],[0,0,1,0,0,0,0,0,0],[0,0,1,0,0,0,0,0,0],[0,0,0,0,1,0,0,0,0],[0,0,1,0,0,0,0,0,0],[0,0,1,0,0,0,0,0,0],[0,0,1,0,0,0,0,0,0],[0,0,1,1,0,0,0,1,0],[0,0,0,0,1,0,0,1,0],[0,0,1,1,1,0,0,0,0],[0,0,0,0,1,0,0,0,0],[0,1,1,1,0,0,0,1,0],[0,1,0,0,0,0,0,0,0],[0,1,1,0,1,0,0,0,0],[0,1,0,0,0,0,0,0,0],[0,1,1,1,0,0,0,0,0],[0,0,0,1,0,0,0,1,0],[0,0,1,1,0,0,0,1,0],[0,0,0,0,1,0,0,0,0],[0,0,1,0,0,0,0,0,0],[0,0,1,1,0,0,0,1,0],[0,0,1,0,0,0,0,0,0],[0,0,1,0,0,0,0,0,0],[0,0,1,0,0,0,0,0,0],[0,0,0,0,1,0,0,0,0],[0,0,1,0,0,0,0,0,0],[0,0,1,0,0,0,0,0,0],[0,0,1,0,0,0,0,0,0]]3843	3844,3845,3846,3847,3848	[[0,0,0,0,1,0,0,0,0],[0,0,0,0,1,0,0,0,0],[0,0,0,0,1,0,0,0,0],[0,0,0,0,1,0,0,0,0],[0,0,0,0,1,0,0,0,0]]3849	3850,3851,3852,3853,3854,3855,3856,423,3633,3857,3858,3859,3860,3861,3862,1094,3863,3864,3865,3866,3867,3868,3869,3870,3871,3872,3873	[[0,1,0,0,0,0,0,0,0],[0,1,0,0,0,0,0,0,0],[0,1,0,0,0,0,0,0,0],[0,1,0,0,0,0,0,0,0],[0,1,0,0,0,0,0,0,0],[0,1,0,0,0,0,0,0,0],[0,1,0,0,0,0,0,0,0],[0,1,0,0,0,0,0,0,0],[0,1,0,0,0,0,0,0,0],[0,1,0,0,0,0,0,0,0],[0,1,0,0,0,0,0,0,0],[0,1,0,0,0,0,0,0,0],[0,1,0,0,0,0,0,0,0],[0,1,0,0,0,0,0,0,0],[0,1,0,0,0,0,0,0,0],[0,1,0,0,0,0,0,0,0],[0,1,0,0,0,0,0,0,0],[0,1,0,0,0,0,0,0,0],[0,1,0,0,0,0,0,0,0],[0,1,0,0,0,0,0,0,0],[0,1,0,0,0,0,0,0,0],[0,1,0,0,0,0,0,0,0],[0,1,0,0,0,0,0,0,0],[0,1,0,0,0,0,0,0,0],[0,1,0,0,0,0,0,0,0],[0,1,0,0,0,0,0,0,0],[0,1,0,0,0,0,0,0,0]]3874	3875,3876,3877,1640,3878,3879,3880,3881,3882,3883,3884,3885,2868,3886,3887,3652,3888,3889,3890,3891,3892,3893,3894,3895,3896,3897,3898,3899,3900,3128,3901,3902,3903,3904,3905,3906,3907,3908,3909,3910,3911,3912,3913	[[0,1,1,0,0,0,0,1,0],[0,0,1,0,0,0,0,1,0],[0,0,1,0,0,0,0,1,0],[0,0,0,0,1,0,0,0,0],[0,1,1,1,0,0,0,1,0],[0,1,0,0,0,0,0,0,0],[0,0,1,0,0,0,0,0,0],[0,0,1,0,0,0,0,1,0],[0,1,1,1,0,0,0,0,0],[0,0,1,0,0,0,0,0,0],[0,0,0,0,1,0,0,0,0],[0,0,1,0,0,0,0,0,0],[0,1,0,0,0,0,0,0,0],[0,0,1,0,0,0,0,0,0],[0,0,1,0,0,0,0,0,0],[0,1,0,0,0,0,0,1,0],[0,0,1,0,0,0,0,0,0],[0,0,1,0,1,0,0,0,0],[0,1,1,0,0,0,0,1,0],[0,1,1,1,1,0,0,1,0],[0,0,0,0,1,0,0,0,0],[0,0,0,0,0,0,0,1,0],[0,0,0,0,1,0,0,0,0],[0,0,1,0,0,0,0,0,0],[0,0,1,0,0,0,0,1,0],[0,0,1,0,0,0,0,0,0],[0,0,0,0,0,0,0,1,0],[0,0,1,1,0,0,0,1,0],[0,0,1,0,0,0,0,0,0],[0,0,0,0,1,0,0,0,0],[0,0,1,0,0,0,0,0,0],[0,0,1,0,0,0,0,0,0],[0,0,1,0,0,0,0,0,0],[0,0,0,0,1,0,0,0,0],[0,0,0,0,1,0,0,0,0],[0,0,1,0,0,0,0,0,0],[0,0,1,0,0,0,0,0,0],[0,0,0,0,0,0,0,1,0],[0,0,1,0,0,0,0,0,0],[0,0,1,0,0,0,0,0,0],[0,0,1,0,0,0,0,0,0],[0,0,1,0,0,0,0,0,0],[0,0,1,0,0,0,0,0,0]]3914	3915,3916,3917,3918,3919,1319,3920,3219,3921,3922,3923,3924,3669,3925,132,3926,3927,3928,3929,1786,3930,3931,288,3932,3933,3934,3686,3935,3936,3937,3938,3939,3940,3941,3525,3942,3943,3944,3945,3946,1305,3658,3764,2434	[[0,0,1,0,0,0,0,0,0],[0,0,1,0,0,0,0,0,0],[0,0,1,0,0,0,0,0,0],[0,0,1,0,0,0,0,0,0],[0,1,0,0,0,0,1,0,0],[0,1,1,0,0,0,1,0,0],[0,0,1,0,0,0,0,0,0],[0,1,1,0,1,0,1,0,0],[0,0,1,0,0,0,0,0,0],[0,0,1,0,0,0,0,0,0],[0,0,1,0,0,0,0,0,0],[0,0,1,0,0,0,0,0,0],[0,0,1,0,0,0,0,0,0],[0,1,0,0,0,0,1,0,0],[0,0,1,0,0,0,0,0,0],[0,0,1,0,0,0,0,0,0],[0,0,1,0,0,0,0,0,0],[0,0,1,0,0,0,0,0,0],[0,1,1,0,0,0,1,0,0],[0,1,0,0,0,0,1,0,0],[0,0,1,0,0,0,0,0,0],[0,0,1,0,0,0,0,0,0],[0,1,0,0,0,0,0,0,0],[0,1,0,0,0,0,1,0,0],[0,0,1,0,0,0,0,0,0],[0,0,1,0,0,0,0,0,0],[0,1,0,0,1,0,1,0,0],[0,1,0,0,0,0,0,0,0],[0,1,0,0,0,0,0,0,0],[0,0,1,0,0,0,0,0,0],[0,1,0,0,0,0,0,0,0],[0,0,1,0,0,0,0,0,0],[0,1,0,0,0,0,0,0,0],[0,0,1,0,0,0,0,0,0],[0,0,1,0,0,0,0,0,0],[0,0,1,0,0,0,0,0,0],[0,0,0,0,1,0,0,0,0],[0,0,1,0,0,0,0,0,0],[0,0,1,0,0,0,0,0,0],[0,1,0,0,0,0,1,0,0],[0,0,1,0,0,0,0,0,0],[0,0,1,0,0,0,0,0,0],[0,0,1,0,0,0,0,0,0],[0,0,1,0,0,0,0,0,0]]3947	3935	[[0,0,0,0,1,0,0,0,0]]3948	3949,1920,3950,3951,3952,1927,3953,1926,3954,3955,3956,3957,3958,3959,3960,3961,3857,3962,3963,3964,3965,3966	[[0,1,1,0,0,0,1,0,0],[0,1,1,0,1,0,1,0,0],[0,0,1,0,0,0,0,0,0],[0,1,1,0,0,0,0,0,0],[0,1,1,0,0,0,1,0,0],[0,1,1,0,0,0,1,0,0],[0,0,0,0,0,0,1,0,0],[0,1,1,0,0,0,1,0,0],[0,0,1,0,0,0,0,0,0],[0,0,1,0,0,0,1,0,0],[0,0,1,0,0,0,0,0,0],[0,0,1,0,0,0,0,0,0],[0,1,1,0,0,0,0,0,0],[0,0,1,0,0,0,0,0,0],[0,1,1,0,0,0,1,0,0],[0,0,1,0,0,0,0,0,0],[0,0,1,0,0,0,1,0,0],[0,0,1,0,0,0,0,0,0],[0,0,1,0,0,0,0,0,0],[0,0,0,0,0,0,1,0,0],[0,0,1,0,0,0,0,0,0],[0,0,1,0,0,0,0,0,0]]3967	3968,3969,3970,3971,3972,3973,3974,3894,3975,3976,3891,3977,3978,3979,3980,3981,3982,3877,3900,3983,3984,3985,3986,3987,3988,2711,3989,3990,3813,3784,3991,3992,3993,3994,3995,3768,3996	[[0,0,1,0,0,0,0,0,0],[0,0,1,0,0,0,0,0,0],[0,0,1,0,0,0,0,0,0],[0,0,1,0,0,0,0,0,0],[0,0,1,0,0,0,0,0,0],[0,0,1,0,0,0,0,0,0],[0,0,1,0,0,0,0,0,0],[0,0,1,0,0,0,0,0,0],[0,0,1,0,0,0,0,0,0],[0,0,1,0,0,0,0,0,0],[0,0,0,0,1,0,0,0,0],[0,0,1,0,0,0,0,0,0],[0,0,1,0,0,0,0,0,0],[0,0,1,0,0,0,0,0,0],[0,0,1,0,0,0,0,0,0],[0,0,1,0,0,0,0,0,0],[0,0,1,0,0,0,0,0,0],[0,0,1,0,0,0,0,0,0],[0,0,1,0,0,0,0,0,0],[0,0,1,0,0,0,0,0,0],[0,0,1,0,0,0,0,0,0],[0,0,1,0,0,0,0,0,0],[0,0,1,0,0,0,0,0,0],[0,0,1,0,0,0,0,0,0],[0,0,1,0,0,0,0,0,0],[0,0,1,0,0,0,0,0,0],[0,0,1,0,0,0,0,0,0],[0,0,1,0,0,0,0,0,0],[0,0,1,0,0,0,0,0,0],[0,0,1,0,0,0,0,0,0],[0,0,1,0,0,0,0,0,0],[0,0,1,0,0,0,0,0,0],[0,0,1,0,0,0,0,0,0],[0,0,1,0,0,0,0,0,0],[0,0,1,0,0,0,0,0,0],[0,0,1,0,0,0,0,0,0],[0,0,1,0,0,0,0,0,0]]3997	3998,3999,4000,4001,4002,4003,4004,4005,4006,4007,4008,4009,4010,4011,4012,4013,4014,4015,4016,2084,4017,4018,4019,4020,4021,2512,4022,4023,4024,4025,4026,4027,4028,4029,4030,4031,4032,4033,4034,4035,4036,4037,4038,622,4039,4040,4041,4042,3857,2562,4043,4044,4045,3442,4046,4047,3715,1476,4048,2082,4049,4050,1645,4051,4052,4053,4054,4055,4056,4057,4058,1686,2183,4059,4060,1065,4061,4062,4063,4064,1044,4065,4066,4067,4068,4069,1067,4070	[[0,0,0,1,0,0,0,0,0],[0,0,0,1,0,0,0,0,0],[0,0,0,1,0,0,0,0,0],[0,0,0,1,0,0,0,0,0],[0,0,0,1,0,0,0,0,0],[0,0,0,1,0,0,0,0,0],[0,0,0,1,0,0,0,0,0],[0,0,0,1,0,0,0,0,0],[0,0,0,1,0,0,0,0,0],[0,0,0,1,0,0,0,0,0],[0,0,0,1,0,0,0,0,0],[0,0,0,1,0,0,0,0,0],[0,0,0,1,0,0,0,0,0],[0,0,0,1,0,0,0,0,0],[0,0,0,1,0,0,0,0,0],[0,0,0,1,0,0,0,0,0],[0,0,0,1,0,0,0,0,0],[0,0,0,1,0,0,0,0,0],[0,0,0,1,0,0,0,0,0],[0,0,0,1,0,0,0,0,0],[0,0,0,1,0,0,0,0,0],[0,0,0,1,0,0,0,0,0],[0,0,0,1,0,0,0,0,0],[0,0,0,1,0,0,0,0,0],[0,0,0,1,0,0,0,0,0],[0,0,0,1,0,0,0,0,0],[0,0,0,1,0,0,0,0,0],[0,0,0,1,0,0,0,0,0],[0,0,0,1,0,0,0,0,0],[0,0,0,1,0,0,0,0,0],[0,0,0,1,0,0,0,0,0],[0,0,0,1,0,0,0,0,0],[0,0,0,1,0,0,0,0,0],[0,0,0,1,0,0,0,0,0],[0,0,0,1,0,0,0,0,0],[0,0,0,1,0,0,0,0,0],[0,0,0,1,0,0,0,0,0],[0,0,0,1,0,0,0,0,0],[0,0,0,1,0,0,0,0,0],[0,0,0,1,0,0,0,0,0],[0,0,0,1,0,0,0,0,0],[0,0,0,1,0,0,0,0,0],[0,0,0,1,0,0,0,0,0],[0,0,0,1,0,0,0,0,0],[0,0,0,1,0,0,0,0,0],[0,0,0,1,0,0,0,0,0],[0,0,0,1,0,0,0,0,0],[0,0,0,1,0,0,0,0,0],[0,0,0,1,0,0,0,0,0],[0,0,0,1,0,0,0,0,0],[0,0,0,1,0,0,0,0,0],[0,0,0,1,0,0,0,0,0],[0,0,0,1,0,0,0,0,0],[0,0,0,1,0,0,0,0,0],[0,0,0,1,0,0,0,0,0],[0,0,0,1,0,0,0,0,0],[0,0,0,1,0,0,0,0,0],[0,0,0,1,0,0,0,0,0],[0,0,0,1,0,0,0,0,0],[0,0,0,1,0,0,0,0,0],[0,0,0,1,0,0,0,0,0],[0,0,0,1,0,0,0,0,0],[0,0,0,1,0,0,0,0,0],[0,0,0,1,0,0,0,0,0],[0,0,0,1,0,0,0,0,0],[0,0,0,1,0,0,0,0,0],[0,0,0,1,0,0,0,0,0],[0,0,0,1,0,0,0,0,0],[0,0,0,1,0,0,0,0,0],[0,0,0,1,0,0,0,0,0],[0,0,0,1,0,0,0,0,0],[0,0,0,1,0,0,0,0,0],[0,0,0,1,0,0,0,0,0],[0,0,0,1,0,0,0,0,0],[0,0,0,1,0,0,0,0,0],[0,0,0,1,0,0,0,0,0],[0,0,0,1,0,0,0,0,0],[0,0,0,1,0,0,0,0,0],[0,0,0,1,0,0,0,0,0],[0,0,0,1,0,0,0,0,0],[0,0,0,1,0,0,0,0,0],[0,0,0,1,0,0,0,0,0],[0,0,0,1,0,0,0,0,0],[0,0,0,1,0,0,0,0,0],[0,0,0,1,0,0,0,0,0],[0,0,0,1,0,0,0,0,0],[0,0,0,1,0,0,0,0,0],[0,0,0,1,0,0,0,0,0]]4071	4072,4073,4074	[[0,0,0,0,1,0,0,0,0],[0,0,0,0,1,0,0,0,0],[0,0,0,0,1,0,0,0,0]]4075	4076,4077,4078,1667,3281,4079,2656,4080,4081,3365,4082,4083,4084,4085,4086,4087,4088,4089,4090,4091,4092,4093,1068,4094,1699,4095,4096,4097,2810,4098,4099,4100,1626,4101,4102,4103,4104,2641,4105,4106,4107,1280,4108,4109,261,4110,4111,4112,4113,4114,4115,4116,4117,4118,4119,4120,4121,4122,4123,1925,4124,4125,4126,2061,2646,4127,4128,4129,4130,1284,4131,4132,3177,4133,4134,4135,4136,4137,1677,4138,4139,4140,3354,4141,4142,1684,4143,4144,4145,4146,4147,2803,4148,3408,4149,4150,4151,3387,2009,4152,4153,3388,4154,4155,4156,4157,4158,4159,2642,4160,4161,4162,4163,4164,2821,4165,4166,4167,4168,4169,4170,1946,4171,4172,4173,4174,4175,4176,4177,4178,4179,4180,1462,4181,1708,1595,4182,4183,3358,2024,1571,4184,1681,4185,4186,4187,4188,4189,4190,4191,4192,1999,4193,4194,4195,1587,4196,4197,4198,949	[[0,0,1,0,0,0,0,0,1],[0,0,1,0,0,0,0,0,0],[0,0,1,0,0,0,0,0,0],[0,1,1,0,0,0,0,0,0],[0,0,1,0,0,0,0,0,0],[0,0,1,0,0,0,0,0,0],[0,0,1,0,0,0,0,0,0],[0,0,1,0,0,0,0,0,0],[0,0,1,0,0,0,0,0,0],[0,0,1,0,0,0,0,0,0],[0,0,1,0,0,0,0,0,0],[0,0,1,0,0,0,0,0,0],[0,0,1,0,0,0,0,0,0],[0,1,1,0,0,0,0,1,1],[0,0,1,0,0,0,0,0,0],[0,0,1,0,0,0,0,0,0],[0,0,1,0,0,0,0,0,0],[0,1,0,0,0,0,0,1,0],[0,0,1,0,0,0,0,0,0],[0,0,1,0,0,0,0,0,0],[0,0,0,0,0,0,0,0,1],[0,0,1,0,0,0,0,0,0],[0,0,1,0,0,0,0,0,0],[0,0,1,0,0,0,0,0,0],[0,0,1,0,0,0,0,0,0],[0,0,1,0,0,0,0,0,0],[0,0,1,1,0,0,0,0,1],[0,0,1,0,0,0,0,0,0],[0,0,1,0,0,0,0,0,0],[0,1,1,0,0,0,0,1,0],[0,0,1,0,0,0,0,0,0],[0,0,1,0,0,0,0,0,0],[0,0,1,0,1,0,0,0,1],[0,0,1,0,0,0,0,0,0],[0,0,1,0,0,0,0,0,0],[0,0,1,0,0,0,0,0,0],[0,0,0,0,0,0,0,0,1],[0,0,1,0,0,0,0,0,0],[0,0,1,0,0,0,0,0,0],[0,0,1,0,0,0,0,0,0],[0,0,1,0,0,0,0,0,0],[0,1,1,0,0,0,0,0,0],[0,0,1,0,0,0,0,0,0],[0,1,1,0,0,0,0,0,0],[0,0,1,0,0,0,0,0,0],[0,0,1,0,0,0,0,0,0],[0,0,1,0,0,0,0,0,0],[0,0,1,0,0,0,0,0,0],[0,0,1,0,0,0,0,0,0],[0,0,1,0,0,0,0,0,0],[0,0,1,0,0,0,0,0,0],[0,0,1,0,0,0,0,0,0],[0,0,1,0,0,0,0,0,0],[0,0,1,0,0,0,0,0,0],[0,0,1,0,0,0,0,0,0],[0,0,1,0,0,0,0,0,0],[0,0,1,0,0,0,0,0,0],[0,0,1,0,0,0,0,0,0],[0,0,1,0,0,0,0,0,0],[0,0,1,0,0,0,0,0,0],[0,0,1,0,0,0,0,0,0],[0,0,1,0,0,0,0,0,0],[0,0,1,0,0,0,0,0,0],[0,0,1,0,0,0,0,0,0],[0,0,1,0,0,0,0,0,0],[0,0,1,0,0,0,0,0,0],[0,0,1,0,0,0,0,0,0],[0,0,1,0,0,0,0,0,0],[0,0,1,0,0,0,0,0,0],[0,1,0,0,0,0,0,0,0],[0,1,0,0,0,0,0,0,0],[0,0,1,0,0,0,0,0,0],[0,0,1,0,0,0,0,0,0],[0,0,1,0,0,0,0,0,0],[0,0,1,0,0,0,0,0,0],[0,0,1,0,0,0,0,0,0],[0,0,1,0,0,0,0,1,0],[0,0,1,0,0,0,0,0,0],[0,0,1,0,0,0,0,0,0],[0,0,1,0,0,0,0,0,0],[0,0,1,0,0,0,0,0,0],[0,0,1,0,0,0,0,0,0],[0,0,1,0,0,0,0,1,0],[0,0,1,0,0,0,0,0,0],[0,0,0,0,0,0,0,1,0],[0,0,1,0,0,0,0,0,0],[0,0,1,0,0,0,0,0,0],[0,0,1,0,0,0,0,0,0],[0,0,0,0,0,0,0,1,0],[0,0,1,0,0,0,0,0,1],[0,0,0,0,0,0,0,0,1],[0,0,1,0,0,0,0,0,0],[0,0,1,0,0,0,0,0,0],[0,0,1,0,0,0,0,0,0],[0,1,1,0,0,0,0,0,0],[0,0,1,0,0,0,0,0,0],[0,0,1,0,0,0,0,0,0],[0,1,1,0,0,0,0,1,0],[0,0,1,0,0,0,0,0,1],[0,0,1,0,0,0,0,0,0],[0,0,0,0,0,0,0,0,1],[0,1,1,1,0,0,0,0,1],[0,0,1,0,0,0,0,0,0],[0,0,1,0,0,0,0,0,0],[0,0,1,0,0,0,0,0,0],[0,0,1,0,0,0,0,0,0],[0,0,1,0,0,0,0,0,0],[0,1,1,0,0,0,0,0,0],[0,0,1,0,0,0,0,0,0],[0,0,1,0,0,0,0,0,0],[0,0,1,0,0,0,0,0,0],[0,0,1,0,0,0,0,0,0],[0,0,1,0,0,0,0,0,0],[0,1,1,0,0,0,0,1,0],[0,0,1,0,0,0,0,0,0],[0,0,1,0,0,0,0,0,1],[0,0,1,0,0,0,0,0,0],[0,0,1,0,0,0,0,0,1],[0,1,1,0,0,0,0,1,0],[0,0,1,0,0,0,0,0,0],[0,1,1,0,0,0,0,0,0],[0,1,0,0,0,0,0,0,0],[0,0,1,0,0,0,0,0,0],[0,0,1,0,0,0,0,1,0],[0,0,1,0,0,0,0,0,0],[0,0,1,0,0,0,0,0,0],[0,0,1,0,0,0,0,0,1],[0,0,1,0,0,0,0,0,0],[0,0,1,0,0,0,0,0,1],[0,0,1,0,0,0,0,0,0],[0,0,1,0,0,0,0,0,0],[0,0,1,0,0,0,0,0,0],[0,0,0,0,0,0,0,1,0],[0,0,1,0,0,0,0,0,0],[0,0,0,0,0,0,0,1,0],[0,0,1,0,0,0,0,0,0],[0,0,0,0,0,0,0,1,0],[0,0,1,0,0,0,0,0,0],[0,0,1,0,0,0,0,0,0],[0,0,1,0,0,0,0,0,0],[0,1,1,1,1,0,0,0,1],[0,0,1,0,0,0,0,0,0],[0,0,1,0,0,0,0,0,0],[0,0,1,0,0,0,0,0,0],[0,0,1,0,0,0,0,0,0],[0,0,1,0,0,0,0,0,0],[0,0,1,0,0,0,0,0,0],[0,0,1,0,0,0,0,0,0],[0,0,1,0,0,0,0,0,0],[0,0,1,0,0,0,0,0,0],[0,0,1,0,0,0,0,0,0],[0,0,1,0,0,0,0,0,0],[0,0,1,0,0,0,0,0,0],[0,0,1,0,0,0,0,0,0],[0,0,1,0,0,0,0,0,0],[0,0,1,0,0,0,0,0,0],[0,0,1,0,0,0,0,0,0],[0,0,1,0,0,0,0,0,0],[0,0,1,0,0,0,0,0,0],[0,0,1,0,0,0,0,0,0]]4199	4200,4201,4202,4203	[[0,0,0,0,1,0,0,0,0],[0,0,0,0,1,0,0,0,0],[0,0,0,0,1,0,0,0,0],[0,0,0,0,1,0,0,0,0]]4204	4205	[[0,0,0,0,1,0,0,0,0]]4206	3832,3794,3790,3814	[[0,0,0,1,0,0,0,0,0],[0,0,0,1,0,0,0,0,0],[0,0,0,1,0,0,0,0,0],[0,0,0,1,0,0,0,0,0]]4207	4208,4209,4210,4211,4212,4213,4214,4215,4216,4217,3680,4218,4219,4220,4221,4222,4223,4224,4225,4226,4227,4228,3941,3652,4229,2426,4230,4231,4232,482,4233,4234,1329,1563,4235,792,4236,4237,3185,4238,4239,4240,4241,4242,2430,4243,4244,3890,4245,4246,4247,4248,4249,4250,4251,3218,1631,4252,4253,4254,4255,4256,4257,4258,2343,1197,4259,4260,4261,4262,4263,4264,329	[[0,0,0,1,0,0,0,1,1],[0,0,0,1,0,0,0,0,0],[0,0,0,1,0,0,0,0,1],[1,0,1,1,0,0,0,1,1],[0,0,0,1,0,0,0,1,1],[0,0,1,0,0,0,0,0,0],[0,0,0,1,0,0,0,0,0],[0,0,0,1,0,0,0,0,1],[0,0,0,1,0,0,0,1,1],[0,0,0,1,0,0,0,1,1],[0,0,1,0,0,0,0,0,0],[0,0,0,1,0,0,0,1,1],[0,0,0,0,0,0,0,0,1],[1,1,1,1,0,0,1,1,1],[0,0,1,1,0,0,0,1,0],[1,0,0,1,0,0,0,0,0],[0,0,1,1,0,0,0,1,1],[0,0,0,1,0,0,0,1,0],[0,0,1,0,0,0,0,0,0],[0,0,0,1,0,0,0,0,1],[0,0,1,1,0,0,0,0,0],[0,0,0,1,0,0,0,1,1],[0,1,1,0,1,0,1,1,0],[0,0,1,0,0,0,0,0,0],[0,0,1,0,0,0,0,0,0],[1,1,1,1,0,0,1,1,1],[0,1,1,0,0,0,0,1,0],[0,0,1,1,0,0,0,1,1],[0,0,0,0,0,0,0,0,1],[0,0,0,1,0,0,0,1,0],[0,0,1,1,0,0,0,0,0],[0,0,0,1,0,0,0,0,0],[0,0,0,1,0,0,0,0,0],[0,0,0,1,0,0,0,0,0],[0,0,1,1,0,0,0,0,0],[0,0,0,1,0,0,0,1,1],[0,0,0,1,0,0,0,1,0],[0,0,0,1,0,0,0,1,1],[0,0,0,1,0,0,0,1,0],[0,0,1,1,0,0,0,1,0],[0,0,1,1,0,0,0,1,1],[0,0,0,1,0,0,0,1,0],[1,0,1,1,0,0,0,1,1],[0,1,1,1,0,0,1,1,0],[1,0,0,0,0,0,0,0,0],[1,0,1,1,1,0,0,1,1],[1,1,1,1,0,0,1,0,1],[0,1,1,0,0,0,0,1,0],[0,0,1,0,0,0,0,0,0],[0,1,0,1,0,0,0,0,1],[0,0,0,1,0,0,0,1,1],[0,1,1,1,0,0,1,1,0],[0,0,0,0,0,0,0,1,0],[0,0,0,1,0,0,0,0,0],[0,0,1,1,0,0,0,1,0],[0,0,1,0,0,0,0,0,0],[0,0,1,0,0,0,0,0,0],[0,0,0,1,0,0,0,0,0],[0,0,0,1,0,0,0,0,0],[0,1,1,1,0,0,0,0,1],[0,0,0,1,0,0,0,0,1],[0,0,1,0,0,0,0,0,0],[0,0,0,1,0,0,0,1,0],[0,0,1,0,0,0,0,0,0],[0,0,0,1,0,0,0,0,0],[0,0,0,1,0,0,0,1,0],[0,0,0,0,0,0,0,0,1],[0,0,1,1,0,0,0,1,1],[0,0,1,0,0,0,0,0,0],[0,0,0,1,0,0,0,0,0],[0,0,0,1,0,0,0,0,0],[0,0,0,1,0,0,0,1,0],[0,0,0,1,0,0,0,0,0]]4265	1738,1743,4266,4267,1741,3259,4268,4269,4270,4271,4272,4273,4274,4275,4276,4277,4278,1742,1734,1744,4279,3268,4280,1397,1740,1737,4281,1736,4282,4283,4284,4285,4286,4287,4288,1735,4289	[[0,1,1,1,1,0,0,0,0],[0,1,1,0,0,0,0,1,0],[0,0,1,0,0,0,0,0,0],[0,0,0,0,0,0,0,1,0],[0,0,1,1,0,0,0,1,0],[0,0,1,0,0,0,0,0,0],[0,1,1,0,1,0,0,1,0],[0,0,1,0,0,0,0,0,0],[0,0,1,0,0,0,0,0,0],[0,1,0,1,0,0,0,1,0],[0,0,1,0,0,0,0,0,0],[0,0,1,0,0,0,0,1,0],[0,1,1,0,0,0,0,1,0],[0,0,1,0,0,0,0,0,0],[0,1,1,0,0,0,0,1,0],[0,0,0,0,1,0,0,0,0],[0,1,1,0,0,0,0,0,0],[0,1,1,0,0,0,0,1,0],[0,0,1,0,0,0,0,0,0],[0,1,1,0,0,0,0,1,0],[0,0,1,0,0,0,0,0,0],[0,0,1,0,0,0,0,0,0],[0,1,1,0,0,0,0,0,0],[0,0,1,0,0,0,0,1,0],[0,1,1,0,0,0,0,0,0],[0,0,1,0,0,0,0,0,0],[0,1,1,0,0,0,0,0,0],[0,1,1,0,0,0,0,1,0],[0,0,1,0,0,0,0,0,0],[0,0,1,0,0,0,0,0,0],[0,0,1,0,0,0,0,1,0],[0,0,0,0,1,0,0,1,0],[0,0,1,0,0,0,0,0,0],[0,0,0,0,0,0,0,1,0],[0,0,1,0,0,0,0,0,0],[0,1,0,0,0,0,0,0,0],[0,0,1,0,0,0,0,0,0]]4290	4291,4292,1498,4293,4294,4295,735,3813,4296,772,4297,4298,4299,4300,4301,4302,4303,4304,2906,4305,4306,4307,4308,767,4309,4310,758,748,755,4311,4312,4313,756,4314,4315,4316,696,4317,4318,4319,766,751,1672,4320,4321,4322,4323,4324,4325,4326,753,4327,752,4328,4329,4330,4331,4332,4333,4334,4335,4336,4337,4338,4339,4340,4341,4342,4343,4344,4345,4346,1601,1640,4347,4348	[[1,0,1,1,0,0,0,1,0],[0,0,0,0,1,0,0,0,0],[0,0,1,0,0,0,0,0,0],[0,0,0,0,0,0,0,1,0],[0,0,0,1,0,0,0,0,0],[0,0,0,1,0,0,0,1,0],[1,1,0,1,0,0,1,1,1],[1,0,1,1,0,0,0,0,0],[1,0,0,1,0,0,0,0,1],[1,1,0,1,0,0,1,1,1],[1,1,1,1,0,0,1,1,1],[1,1,1,1,1,0,1,0,0],[1,1,1,0,0,0,0,1,0],[1,1,1,1,0,0,0,0,0],[0,0,0,1,0,0,0,1,0],[0,1,1,1,0,0,0,1,0],[0,0,0,1,0,0,0,0,0],[0,0,1,0,0,0,0,0,0],[0,0,0,1,0,0,0,0,0],[0,0,1,0,0,0,0,0,0],[0,0,0,1,0,0,0,0,1],[1,0,1,1,0,0,0,1,0],[0,0,1,1,0,0,0,0,0],[1,1,1,1,0,0,1,1,0],[0,0,0,1,0,0,0,0,0],[1,0,0,0,0,0,0,0,0],[1,0,1,1,0,0,0,1,0],[1,0,0,1,0,0,0,1,0],[0,0,1,0,0,0,0,0,0],[0,0,0,1,0,0,0,0,0],[0,0,1,1,0,0,0,0,0],[1,0,1,1,0,0,0,1,0],[1,0,0,0,0,0,0,0,0],[0,0,0,1,0,0,0,0,0],[0,0,1,0,0,0,0,0,0],[0,0,0,0,0,0,0,1,0],[0,0,0,1,0,0,0,1,0],[1,0,0,1,0,0,0,1,0],[0,0,1,0,0,0,0,0,0],[1,0,1,1,0,0,0,0,0],[1,0,0,0,0,0,0,1,0],[1,0,0,1,0,0,0,1,0],[1,0,0,0,0,0,0,0,0],[0,0,0,1,0,0,0,0,0],[0,0,1,0,0,0,0,0,0],[1,0,1,1,0,0,0,1,0],[0,0,1,0,0,0,0,1,0],[0,1,0,0,0,0,0,0,0],[1,0,0,0,0,0,0,1,0],[1,0,0,0,0,0,0,1,0],[0,1,0,0,0,0,1,0,0],[0,0,0,1,0,0,0,0,0],[1,0,0,1,0,0,0,1,0],[0,0,0,1,0,0,0,1,0],[0,0,1,0,0,0,0,0,0],[0,1,1,1,1,0,0,0,1],[0,1,0,0,0,0,1,0,1],[0,1,0,1,0,0,1,0,0],[0,0,1,0,0,0,0,0,0],[0,0,1,0,0,0,0,0,0],[1,0,0,0,0,0,0,0,0],[0,0,0,1,0,0,0,0,0],[0,0,1,0,0,0,0,1,0],[0,0,0,1,0,0,0,0,0],[0,0,1,0,0,0,0,0,0],[0,0,1,0,0,0,0,0,0],[0,0,0,1,0,0,0,0,0],[0,0,1,0,0,0,0,0,0],[0,0,1,0,0,0,0,0,0],[1,0,0,0,0,0,0,0,0],[0,0,1,0,0,0,0,0,0],[0,0,0,1,0,0,0,0,0],[0,0,1,0,0,0,0,0,0],[0,0,1,0,0,0,0,0,0],[0,0,1,0,0,0,0,0,0],[0,0,0,1,0,0,0,0,0]]4349	4350,4351,4352,4353,4354,4355,2195,4356,4357,3513,4358,4359,4360,4361,4362,4363,4364,2190,4365,4366,4367,2192,817,4368,4369,3533,4034,4370,4371,4372,344,4373,4374,4046,4375,4376,4377,4378,4379,4380,4381,3532,4382,4383,3515,3525,3529,4384,4385,4386,4036,4041,2676,3539,4052,4387,3526,2667,3520,4388,4389,2803,4390,3470,4391,4054,2188,4392,4393,4394,4395,4396,3536,653,4397,4048,4398,4399,4400,4401,4402,1714,4403,4404,4405	[[0,1,1,0,0,0,0,0,0],[0,1,0,0,0,0,0,0,0],[0,1,1,0,0,0,0,0,1],[0,1,1,0,0,0,0,0,0],[0,1,1,1,0,0,0,1,1],[0,1,1,0,0,0,0,0,0],[0,1,1,0,0,0,0,1,0],[0,1,1,0,0,0,0,1,0],[0,1,0,0,0,0,0,0,0],[0,0,1,0,0,0,0,0,0],[0,1,1,0,0,0,0,0,0],[0,0,1,0,0,0,0,0,0],[0,1,0,0,0,0,0,0,0],[0,1,1,0,0,0,0,1,1],[0,1,1,0,0,0,0,0,0],[0,0,1,0,0,0,0,0,0],[0,0,1,0,0,0,0,0,0],[0,1,1,0,0,0,0,0,1],[0,0,1,0,0,0,0,1,0],[0,1,0,0,0,0,0,1,1],[0,1,1,1,0,0,0,1,0],[0,1,1,0,0,0,0,1,1],[0,0,1,0,0,0,0,0,0],[0,1,1,0,0,0,0,1,0],[0,0,1,0,0,0,0,0,0],[0,1,1,0,0,0,0,0,0],[0,0,1,0,0,0,0,0,0],[0,1,1,1,0,0,0,1,1],[0,0,1,0,0,0,0,0,0],[0,0,1,0,0,0,0,0,0],[0,0,1,0,0,0,0,0,0],[0,0,1,0,0,0,0,0,0],[0,0,1,0,0,0,0,0,0],[0,0,1,0,0,0,0,0,0],[0,0,1,0,0,0,0,0,0],[0,0,0,0,0,0,0,1,0],[0,0,1,0,0,0,0,0,0],[0,0,1,0,0,0,0,0,1],[0,0,1,0,0,0,0,0,0],[0,0,1,0,1,0,0,0,0],[0,0,1,0,0,0,0,0,0],[0,0,1,0,0,0,0,0,0],[0,0,1,0,0,0,0,0,0],[0,0,1,0,0,0,0,0,0],[0,0,1,0,0,0,0,0,0],[0,1,1,0,0,0,0,0,0],[0,0,1,0,0,0,0,0,0],[0,1,0,0,0,0,0,1,0],[0,0,1,0,0,0,0,0,0],[0,0,1,0,0,0,0,0,0],[0,0,1,0,0,0,0,0,0],[0,0,1,0,0,0,0,0,0],[0,0,1,0,0,0,0,0,0],[0,0,1,0,0,0,0,0,0],[0,0,1,0,0,0,0,0,0],[0,0,1,0,0,0,0,0,0],[0,0,1,0,0,0,0,0,0],[0,0,1,0,0,0,0,0,0],[0,0,1,0,0,0,0,0,0],[0,0,1,0,0,0,0,0,0],[0,0,1,0,0,0,0,0,0],[0,0,1,0,0,0,0,0,0],[0,0,1,0,0,0,0,0,0],[0,0,1,0,0,0,0,0,0],[0,0,1,0,0,0,0,0,0],[0,0,1,0,0,0,0,0,0],[0,1,0,0,0,0,0,0,0],[0,0,1,0,0,0,0,0,0],[0,0,1,0,0,0,0,0,0],[0,0,1,0,0,0,0,0,0],[0,1,1,0,0,0,0,1,1],[0,0,1,0,0,0,0,0,0],[0,0,1,0,0,0,0,0,0],[0,0,1,0,0,0,0,0,0],[0,0,1,0,0,0,0,0,0],[0,0,1,0,0,0,0,0,0],[0,0,1,0,0,0,0,0,0],[0,0,1,0,0,0,0,0,0],[0,0,1,0,0,0,0,0,0],[0,0,1,0,0,0,0,0,0],[0,0,1,0,0,0,0,0,0],[0,0,1,0,0,0,0,0,0],[0,0,1,0,0,0,0,0,0],[0,0,1,0,0,0,0,0,0],[0,0,1,0,0,0,0,0,0]]4406	4407,4408,4409	[[0,0,0,1,0,0,0,0,0],[0,0,0,1,0,0,0,0,0],[0,0,0,1,0,0,0,0,0]]4410	4411,4412,4413,4414,4415,4416,4417,4418,747,4419,4420,4421,4422,4423,4424,4425,4426,4427,4428,4429,3988,4430,4431,251,4432,4433,4434,4435,250	[[0,1,1,0,0,0,0,0,1],[0,1,1,0,0,0,0,0,0],[0,1,0,1,0,0,0,0,0],[0,0,0,0,1,0,0,0,1],[0,0,0,0,0,0,0,0,1],[0,0,0,0,0,0,0,0,1],[0,1,1,0,1,0,0,0,1],[0,0,0,0,0,0,0,0,1],[0,1,0,0,0,0,0,0,0],[0,1,0,0,0,0,0,0,1],[0,0,0,0,0,0,0,0,1],[0,1,0,0,0,0,0,0,0],[0,0,1,0,0,0,0,0,0],[0,1,0,0,0,0,0,0,0],[0,0,1,0,0,0,0,0,0],[0,1,0,0,0,0,0,0,0],[0,1,1,0,0,0,0,0,0],[0,0,1,0,0,0,0,0,1],[0,1,1,0,1,0,0,0,1],[0,0,0,0,0,0,0,0,1],[0,0,0,1,0,0,0,0,0],[0,1,0,0,0,0,0,0,0],[0,1,0,0,0,0,0,0,0],[0,1,0,0,0,0,0,0,0],[0,1,0,0,0,0,0,0,0],[0,1,0,0,0,0,0,0,0],[0,0,0,0,0,0,0,0,1],[0,0,1,0,0,0,0,0,0],[0,1,0,0,0,0,0,0,0]]4436	4437	[[0,0,0,0,1,0,0,0,0]]4438	4439,4440,4441	[[0,0,0,0,1,0,0,0,0],[0,0,0,0,1,0,0,0,0],[0,0,0,0,1,0,0,0,0]]4442	4271,1738,1741	[[0,0,0,1,0,0,0,0,0],[0,0,0,1,0,0,0,0,0],[0,0,0,1,0,0,0,0,0]]4443	4444	[[0,0,0,0,1,0,0,0,0]]4445	4446,2118,2112	[[0,1,0,0,0,0,0,0,0],[0,1,0,0,0,0,0,0,0],[0,1,0,0,0,0,0,0,0]]4447	4448,4449,4450	[[0,0,0,0,1,0,0,0,0],[0,0,0,0,1,0,0,0,0],[0,0,0,0,1,0,0,0,0]]4451	4452,4453,463,4454,4455,4456,4457,4458,4459,2005,4460,4461,4462,4463,4464,4465,4466,4467,4468,4469,4470,4471,4472,3082,4473,3489,4474,3633,4147,4195,4092,4475,4476,4175,4477,4478,4479,3859,4480,4481,4482,4483,4484,4485,4486,4487,4488,4489,4490,4146,4491,2677,4492,4493,4494,2009,4495,4496,2018,1962,4497,4498,4499,4500,4501,4502,4503,2823,4504,4505,4506,4507,4508	[[0,0,1,0,1,0,0,0,0],[0,0,1,0,0,0,0,0,0],[0,0,1,0,0,0,0,0,0],[0,0,1,0,1,0,0,0,0],[0,0,1,0,0,0,0,0,0],[0,0,1,0,0,0,0,0,0],[0,0,1,0,0,0,0,0,0],[0,0,1,0,0,0,0,0,0],[0,0,1,0,0,0,0,0,0],[0,0,1,0,0,0,0,0,0],[0,0,1,0,1,0,0,0,0],[0,0,0,1,0,0,0,0,0],[0,0,1,0,0,0,0,0,0],[0,0,1,0,0,0,0,0,0],[0,0,1,0,1,0,0,0,0],[0,0,0,0,1,0,0,0,0],[0,0,0,0,1,0,0,0,0],[0,0,1,0,0,0,0,0,0],[0,0,1,0,0,0,0,0,0],[0,0,1,0,0,0,0,0,0],[0,0,1,0,0,0,0,0,0],[0,0,1,0,0,0,0,0,0],[0,0,1,0,0,0,0,0,0],[0,0,1,0,0,0,0,0,0],[0,0,1,0,1,0,0,0,0],[0,0,0,0,1,0,0,0,0],[0,0,1,0,0,0,0,0,0],[0,0,1,0,0,0,0,0,0],[0,0,1,0,0,0,0,0,0],[0,0,1,0,0,0,0,0,0],[0,0,1,0,0,0,0,0,0],[0,0,1,0,0,0,0,0,0],[0,0,1,0,0,0,0,0,0],[0,0,1,0,0,0,0,0,0],[0,0,1,0,1,0,0,0,0],[0,0,1,0,0,0,0,0,0],[0,0,0,1,0,0,0,0,0],[0,0,1,0,0,0,0,0,0],[0,0,1,0,0,0,0,0,0],[0,0,1,0,0,0,0,0,0],[0,0,1,0,0,0,0,0,0],[0,0,1,0,0,0,0,0,0],[0,0,1,0,0,0,0,0,0],[0,0,1,0,0,0,0,0,0],[0,0,1,0,0,0,0,0,0],[0,0,1,0,0,0,0,0,0],[0,0,1,1,0,0,0,0,0],[0,0,1,0,0,0,0,0,0],[0,0,1,0,0,0,0,0,0],[0,0,1,0,0,0,0,0,0],[0,0,1,0,0,0,0,0,0],[0,0,1,0,0,0,0,0,0],[0,0,1,0,0,0,0,0,0],[0,0,1,0,0,0,0,0,0],[0,0,1,0,0,0,0,0,0],[0,0,1,0,0,0,0,0,0],[0,0,1,0,0,0,0,0,0],[0,0,1,0,0,0,0,0,0],[0,0,1,0,0,0,0,0,0],[0,0,1,0,0,0,0,0,0],[0,0,1,0,0,0,0,0,0],[0,0,1,0,0,0,0,0,0],[0,0,1,0,0,0,0,0,0],[0,0,1,0,0,0,0,0,0],[0,0,1,0,0,0,0,0,0],[0,0,0,1,0,0,0,0,0],[0,0,1,0,0,0,0,0,0],[0,0,0,0,1,0,0,0,0],[0,0,1,0,0,0,0,0,0],[0,0,1,0,0,0,0,0,0],[0,0,1,0,0,0,0,0,0],[0,0,1,0,0,0,0,0,0],[0,0,1,0,0,0,0,0,0]]4509	4510,4511,4512,4513	[[0,0,0,0,1,0,0,0,0],[0,0,0,0,1,0,0,0,0],[0,0,0,0,1,0,0,0,0],[0,0,0,0,1,0,0,0,0]]4514	4515	[[0,0,0,0,1,0,0,0,0]]4516	4517,4518,4519,4520,1040,4521,4522,4523,4524,4525,651,43,4526,4527,643,4528,4529,4530	[[0,1,0,1,0,1,0,1,0],[0,1,0,1,0,1,0,1,0],[0,1,0,0,0,1,0,1,0],[0,1,0,0,0,1,0,1,0],[0,1,0,0,1,0,0,0,0],[0,1,0,0,0,1,0,1,0],[0,1,0,0,1,0,0,1,0],[0,1,0,1,0,0,0,1,0],[0,1,0,0,0,0,0,1,0],[0,1,0,0,0,1,0,1,0],[0,1,0,0,0,0,0,0,0],[0,1,0,0,1,0,0,1,0],[0,1,0,0,0,0,0,0,0],[0,1,0,0,0,0,0,0,0],[0,1,0,0,0,0,0,0,0],[0,0,0,0,0,0,0,1,0],[0,0,0,0,0,0,0,1,0],[0,0,0,0,0,0,0,1,0]]4531	1622,1602,4532,4533,1048,1567	[[0,0,1,0,0,0,0,0,0],[0,1,1,0,0,0,0,0,0],[0,0,1,0,0,0,0,0,0],[0,0,1,0,0,0,0,0,0],[0,1,0,0,0,0,0,0,0],[0,1,1,0,0,0,0,0,0]]4534	4213,4535,3694,4536,4537,1635,4538,4539,4540	[[0,1,1,0,0,0,0,0,0],[0,1,1,0,1,0,0,0,0],[0,1,1,0,0,0,0,0,0],[0,0,1,0,0,0,0,0,0],[0,1,1,0,0,0,0,0,0],[0,1,1,0,0,0,0,0,0],[0,1,1,0,0,0,0,0,0],[0,0,1,0,0,0,0,0,0],[0,0,1,0,0,0,0,0,0]]4541	422,4542,4543,4544,1693,4545,1550,4546,3619,4033,425,4547,1048,4548,4549	[[0,0,0,1,0,0,0,0,0],[0,0,0,1,0,0,0,0,0],[0,0,0,1,0,0,0,0,0],[0,0,0,1,0,0,0,0,0],[0,0,0,1,0,0,0,0,0],[0,0,0,1,0,0,0,0,0],[0,0,0,1,0,0,0,0,0],[0,0,0,1,0,0,0,0,0],[0,0,0,1,0,0,0,0,0],[0,0,0,1,0,0,0,0,0],[0,0,0,1,0,0,0,0,0],[0,0,0,1,0,0,0,0,0],[0,0,0,1,0,0,0,0,0],[0,0,0,1,0,0,0,0,0],[0,0,0,1,0,0,0,0,0]]4550	4488,4479,4502,4461	[[0,0,0,1,0,0,0,0,0],[0,0,0,1,0,0,0,0,0],[0,0,0,1,0,0,0,0,0],[0,0,0,1,0,0,0,0,0]]4551	704,4552,4553	[[0,0,0,0,1,0,0,0,0],[0,0,0,0,1,0,0,0,0],[0,0,0,0,1,0,0,0,0]]4554	4555,4556,1549,4557,2617,4558,4559,4560,4561,4562,4563,4564,4565,4566,4567,4568,4569,4570,4571,4572,4573,4574,4575,4576,4577,1143,4578,4579,4580,4581,4582	[[0,0,1,0,0,0,0,0,0],[0,0,1,0,0,0,0,0,0],[0,0,1,0,0,0,0,0,0],[0,0,1,0,0,0,0,0,0],[0,0,1,0,0,0,0,0,0],[0,0,1,0,0,0,0,0,0],[0,0,1,0,0,0,0,0,0],[0,0,1,0,0,0,0,0,0],[0,0,1,0,0,0,0,0,0],[0,0,1,0,0,0,0,0,0],[0,0,1,0,0,0,0,0,0],[0,0,1,0,0,0,0,0,0],[0,0,1,0,0,0,0,0,0],[0,0,1,0,0,0,0,0,0],[0,0,1,0,0,0,0,0,0],[0,0,1,0,0,0,0,0,0],[0,0,1,0,0,0,0,0,0],[0,0,1,0,0,0,0,0,0],[0,0,1,0,0,0,0,0,0],[0,0,1,1,0,0,0,0,0],[0,0,0,1,0,0,0,0,0],[0,0,1,0,0,0,0,0,0],[0,0,1,0,0,0,0,0,0],[0,0,1,0,0,0,0,0,0],[0,0,1,0,0,0,0,0,0],[0,0,0,1,0,0,0,0,0],[0,0,1,0,0,0,0,0,0],[0,0,1,0,0,0,0,0,0],[0,0,1,0,0,0,0,0,0],[0,0,1,0,0,0,0,0,0],[0,0,1,0,0,0,0,0,0]]4583	4584,4585,4586,4587,4588,4589,4590	[[0,0,0,0,1,0,0,0,0],[0,0,0,0,1,0,0,0,0],[0,0,0,0,1,0,0,0,0],[0,0,0,0,1,0,0,0,0],[0,0,0,0,1,0,0,0,0],[0,0,0,0,1,0,0,0,0],[0,0,0,0,1,0,0,0,0]]4591	4592	[[0,0,0,0,1,0,0,0,0]]4593	4594,4595	[[0,0,0,0,1,0,0,0,0],[0,0,0,0,1,0,0,0,0]]4596	4597	[[0,0,0,0,1,0,0,0,0]]4598	4599,595,4600,4601,2881,4602,1930,4603,4604,2867,4605,4606,1563,4607,1918,4608,4609,1931,1568,2511,4610	[[0,1,1,0,0,0,0,0,0],[0,0,1,0,0,0,0,0,0],[0,0,0,1,0,0,0,0,0],[0,1,1,0,0,0,0,0,0],[0,1,1,0,0,0,0,0,0],[0,1,1,0,0,0,0,0,0],[0,1,1,1,1,0,0,0,0],[0,1,1,0,0,0,0,0,0],[0,1,0,0,0,0,0,0,0],[0,1,1,0,0,0,0,0,0],[0,1,0,1,0,0,0,0,0],[0,0,0,1,0,0,0,0,0],[0,0,0,1,0,0,0,0,0],[0,0,1,0,0,0,0,0,0],[0,0,0,1,0,0,0,0,0],[0,0,1,0,0,0,0,0,0],[0,0,0,1,0,0,0,0,0],[0,0,0,1,0,0,0,0,0],[0,0,0,1,0,0,0,0,0],[0,0,1,1,0,0,0,0,0],[0,0,0,1,0,0,0,0,0]]4611	4612,4613,1602,4614,649,1603,4615,1214,4616,4617,2820,4618,4619,4033,4620,4621,4622,4623,4624,1099,4625,4626,4627,4628,4629,4630,4542,3619,4631,4632,4633,4548,4634,4635,4636,4637,4638,3561,1229,4639,4640,4641,4642,4643,4644,4645,4646,4647,4648,4649,4650,1585,4651,4652,4096,4653,4654,4655,4656,4657,4658,4659,4660,3560,3534,4661,4662,429,4663,4664,4665,4529,4666,4667,4668,4669,4670,4671,1218,1587,4672,4057,2062,4673,4674,4675,3873,4676,4677,4678,4679,4680,2051,4681,4682,4683,1217,4684,1048,4685,3870,4686,1222,4546,4549,4687,4688,3562,4689,425,1550,4545,4690,4691,4692,4693,4547,422,4694,2398,4695,3856,4009,4696,1595,4697,4306,4698,1400,4699,4700,4701,4702,4703,4704,4705,4706,1948,4707,4708,4709,4710,4544,4711,4712,4713,4714,4715,4716,1396,4543,1693,4717,4094,3867,4718,4719,4720,642,4721,4722,4723,4724,4725,4726,4727,4728	[[0,1,0,0,0,0,0,0,0],[0,0,1,0,0,0,0,1,1],[0,1,1,0,0,0,1,1,1],[0,1,1,0,1,0,1,1,0],[0,1,1,0,0,0,1,0,0],[0,0,1,0,0,1,0,0,1],[0,0,1,0,0,1,0,0,1],[0,0,1,0,0,0,0,0,0],[0,0,1,0,0,0,0,0,0],[0,1,0,0,0,0,0,0,0],[0,1,1,0,0,0,1,0,0],[0,0,1,0,0,0,0,0,0],[0,0,1,0,0,0,0,0,0],[0,0,1,1,0,0,0,1,0],[0,0,1,0,0,0,0,0,0],[0,0,1,0,0,0,0,0,0],[0,0,1,0,0,0,0,0,0],[0,0,1,0,0,0,0,0,0],[0,1,1,0,0,0,1,1,0],[0,0,1,0,0,0,0,0,0],[0,0,1,0,0,0,0,0,0],[0,0,1,0,0,0,0,0,0],[0,0,1,0,0,0,0,0,0],[0,0,1,0,0,0,0,0,0],[0,0,1,0,0,0,0,0,0],[0,1,1,0,0,0,0,0,0],[0,0,1,1,1,1,0,1,1],[1,1,1,1,0,0,1,1,0],[0,0,0,0,0,0,0,1,0],[0,0,1,0,0,0,0,0,0],[0,0,1,0,0,0,0,0,0],[1,1,1,1,0,1,0,1,0],[0,0,1,0,0,0,0,0,0],[0,0,1,0,0,1,0,0,0],[0,1,1,0,1,0,1,0,1],[0,0,1,0,0,0,0,0,0],[0,0,1,0,0,0,0,0,0],[0,0,1,0,0,0,0,0,0],[0,0,1,0,0,0,0,0,0],[0,0,1,0,0,0,0,0,0],[0,0,1,0,0,0,0,0,0],[0,0,1,0,0,0,0,0,0],[0,0,1,0,0,0,0,0,0],[0,0,1,0,0,0,0,0,0],[0,0,1,0,0,0,0,0,0],[0,0,1,0,0,0,0,1,0],[0,1,0,0,0,1,0,1,0],[0,0,1,0,0,0,0,0,0],[0,0,1,0,0,0,0,0,0],[1,0,1,0,0,1,0,1,1],[0,0,1,0,0,0,0,0,0],[0,0,1,0,0,0,0,0,0],[0,1,1,0,0,0,0,0,0],[0,0,1,0,0,0,0,0,0],[0,0,1,0,0,0,0,0,0],[0,0,1,0,0,0,0,0,0],[0,0,1,0,0,0,0,0,0],[0,0,1,0,0,0,0,0,0],[0,0,1,0,0,0,0,0,0],[0,0,1,0,0,0,0,0,0],[0,0,1,0,0,0,0,0,0],[0,0,1,0,0,0,0,0,0],[0,0,0,0,1,0,1,0,0],[0,0,1,0,0,0,0,0,0],[0,0,1,0,0,0,0,0,0],[0,0,1,0,0,0,0,0,0],[0,0,1,0,0,0,0,0,0],[0,0,1,0,0,0,0,0,0],[0,0,1,0,0,0,0,0,0],[0,1,1,0,0,0,0,1,0],[0,0,1,0,0,0,0,0,0],[0,0,1,0,0,0,0,0,0],[0,0,1,0,0,0,0,0,0],[0,0,1,0,0,0,0,0,0],[0,0,1,0,0,0,0,1,0],[0,0,1,0,0,0,0,0,0],[0,0,1,0,0,0,0,0,0],[0,0,1,0,0,0,0,0,0],[0,0,1,0,0,0,0,0,0],[0,0,1,0,0,1,0,0,1],[0,1,1,0,0,0,0,0,0],[0,0,1,0,0,0,0,0,0],[0,0,1,0,0,0,0,0,0],[0,0,1,0,0,0,0,0,0],[0,0,1,0,0,0,0,0,0],[0,0,1,0,0,0,0,0,0],[0,0,1,0,0,0,0,0,0],[0,0,1,0,0,0,0,0,0],[0,0,1,0,0,0,0,0,0],[0,0,1,0,0,0,0,0,0],[0,0,1,0,0,0,0,0,0],[0,0,1,0,0,0,0,0,0],[0,0,1,0,0,0,0,0,0],[0,0,1,0,0,0,0,0,0],[0,0,1,0,0,0,0,0,0],[0,0,1,0,0,0,0,0,0],[0,0,1,0,0,0,0,0,0],[0,0,1,0,0,0,0,0,0],[0,0,0,1,0,1,0,1,1],[0,1,0,0,0,0,0,0,0],[0,1,0,0,0,0,0,0,0],[0,1,0,0,0,0,1,0,0],[0,0,1,0,0,0,0,0,0],[1,1,0,1,0,0,0,0,0],[1,1,0,1,0,0,1,1,0],[0,0,1,0,0,0,0,0,0],[0,1,0,0,0,0,0,1,0],[0,1,1,0,1,0,0,1,0],[0,1,1,0,0,0,1,0,0],[0,1,1,1,1,1,1,0,0],[0,0,1,1,0,1,0,0,1],[0,0,1,1,0,0,0,1,0],[0,0,1,0,0,0,0,0,0],[0,0,0,0,1,0,0,0,0],[0,0,0,0,0,1,0,0,0],[0,1,0,0,0,0,0,0,0],[0,0,0,1,0,0,0,0,0],[0,1,1,1,1,1,1,1,1],[0,1,0,0,0,0,0,0,0],[0,0,1,0,0,0,0,0,0],[0,0,1,0,0,0,0,0,0],[0,0,1,0,0,0,0,0,0],[0,0,1,0,0,0,0,0,0],[0,0,1,0,0,0,0,0,0],[0,0,1,0,0,0,0,0,0],[0,0,1,0,0,0,0,0,0],[0,0,1,0,0,0,0,0,0],[0,0,1,0,0,0,0,1,0],[0,0,1,0,0,0,0,0,0],[0,0,1,0,0,0,0,0,0],[0,0,1,0,0,0,0,0,0],[0,1,1,0,0,0,1,0,0],[0,0,1,0,0,0,0,0,0],[0,0,1,0,0,0,0,0,0],[0,0,1,0,0,0,0,0,0],[0,1,0,0,0,0,0,0,0],[1,0,0,0,0,0,0,0,0],[0,0,1,0,0,0,0,0,0],[1,0,0,0,0,0,0,0,0],[0,0,1,0,0,0,0,0,0],[0,0,1,0,0,0,0,0,0],[0,0,1,0,0,0,0,0,0],[0,0,1,1,0,0,0,1,0],[0,0,1,0,0,0,0,1,0],[0,0,0,0,0,0,0,1,0],[0,0,1,0,0,0,0,0,0],[0,0,1,0,0,0,0,0,0],[0,0,1,0,0,0,0,0,0],[0,0,1,0,0,0,0,0,0],[0,0,1,0,0,0,0,0,0],[0,0,0,1,0,0,0,1,0],[0,0,0,1,0,0,0,1,0],[0,0,1,0,0,0,0,0,0],[0,0,1,0,0,0,0,0,0],[0,0,1,0,0,0,0,0,0],[0,0,1,0,0,0,0,0,0],[0,0,1,0,0,0,0,0,0],[0,0,0,0,0,1,0,0,0],[0,1,0,0,0,0,0,0,0],[0,0,1,0,0,0,0,0,0],[0,0,1,0,0,0,0,0,0],[0,0,1,0,0,0,0,0,0],[0,0,1,0,0,0,0,0,0],[0,0,1,0,0,0,0,0,0],[0,0,1,0,0,0,0,0,0],[0,0,1,0,0,0,0,0,0],[0,0,1,0,0,0,0,0,0]]4729	4730	[[0,0,0,0,1,0,0,0,0]]4731	4732,4733,4734,4735,4736,4737,4738,4739,4740,4741,4742,4743,4744,4745,4746,4747,4748,4749,4750	[[0,0,0,1,0,0,0,0,0],[0,0,0,1,0,0,0,0,0],[0,0,0,1,0,0,0,0,0],[0,0,0,1,0,0,0,0,0],[0,0,0,1,0,0,0,0,0],[0,0,0,1,0,0,0,0,0],[0,0,0,1,0,0,0,0,0],[0,0,0,1,0,0,0,0,0],[0,0,0,1,0,0,0,0,0],[0,0,0,1,0,0,0,0,0],[0,0,0,1,0,0,0,0,0],[0,0,0,1,0,0,0,0,0],[0,0,1,0,0,0,0,0,0],[0,0,1,1,0,0,0,0,0],[0,0,0,1,0,0,0,0,0],[0,0,0,1,0,0,0,0,0],[0,0,0,1,0,0,0,0,0],[0,0,0,1,0,0,0,0,0],[0,0,0,1,0,0,0,0,0]]4751	4752,4753	[[0,0,0,0,1,0,0,0,0],[0,0,0,0,1,0,0,0,0]]4754	4755,4756,4757,4758,4759,4760,4761,4762,4763,4764,4765,4766	[[0,0,0,0,1,0,0,0,0],[0,0,0,0,1,0,0,0,0],[0,0,0,0,1,0,0,0,0],[0,0,0,1,0,0,0,0,0],[0,0,0,0,1,0,0,0,0],[0,0,0,0,1,0,0,0,0],[0,0,0,0,1,0,0,0,0],[0,0,0,0,1,0,0,0,0],[0,0,0,1,0,0,0,0,0],[0,0,0,0,1,0,0,0,0],[0,0,0,0,1,0,0,0,0],[0,0,0,0,1,0,0,0,0]]4767	4768,4769,4770,4771,4772,4773,4774,4775,4776,4777,4778,4779	[[0,0,1,1,0,0,0,1,0],[0,0,1,0,0,0,0,0,0],[0,0,1,1,0,0,0,1,0],[0,0,1,0,0,0,0,1,0],[0,0,1,1,0,0,0,1,0],[0,0,1,0,0,0,0,1,0],[0,0,1,0,0,0,0,0,0],[0,0,1,0,0,0,0,0,0],[0,0,1,1,0,0,0,1,0],[0,0,1,0,0,0,0,0,0],[0,0,1,0,0,0,0,0,0],[0,0,0,1,0,0,0,0,0]]4780	4781,4782,4783,4784,4785	[[0,0,0,0,1,0,0,0,0],[0,0,0,0,1,0,0,0,0],[0,0,0,0,1,0,0,0,0],[0,0,0,0,1,0,0,0,0],[0,0,0,0,1,0,0,0,0]]4786	4787,1451,4788	[[0,0,0,1,0,0,0,0,0],[0,0,0,1,0,0,0,0,0],[0,0,0,1,0,0,0,0,0]]4789	4790,4791,4792,4793,4794,3753,4795,4796,3657,4797,4798,4799,4800,2343,4801,4802	[[0,1,0,0,0,0,0,0,0],[0,1,0,0,0,0,0,0,0],[0,1,0,0,0,0,0,0,0],[0,1,0,0,0,0,0,0,0],[0,1,0,0,0,0,0,0,0],[0,1,0,0,0,0,0,0,0],[0,1,0,0,0,0,0,0,0],[0,1,0,0,0,0,0,0,0],[0,1,0,0,0,0,0,0,0],[0,1,0,0,0,0,0,0,0],[0,1,0,0,0,0,0,0,0],[0,1,0,0,0,0,0,0,0],[0,1,0,0,0,0,0,0,0],[0,1,0,0,0,0,0,0,0],[0,1,0,0,0,0,0,0,0],[0,1,0,0,0,0,0,0,0]]4803	4804,1919,3813,4805,4806,3323,4807,4808,4809,4810,4811,4812,4813,731,730,3652,1797,1928,4814,4815,1319,3694,4816,1369,4817,1840,1370,4818,4819,2110,4820,4821,4822,4823,4824,4825,4826,4827,1163,4828,1925,4829,4830,4831,4832,4833,485,4247,4834,2520,4835,4836,4837,502,4838,4839,4840,4841,2344,4842,2354,4843,4844,4845,4846	[[0,0,1,0,0,0,0,0,0],[0,1,0,1,1,0,1,0,0],[0,0,1,0,0,0,0,0,0],[0,0,1,0,0,0,0,0,0],[0,0,1,0,0,0,0,0,0],[0,0,1,0,0,0,0,0,0],[0,0,1,0,0,0,0,0,0],[0,1,0,0,0,0,1,0,0],[0,0,1,0,0,0,0,0,0],[0,0,1,0,0,0,0,0,0],[0,1,0,0,0,0,1,1,0],[0,1,0,0,1,0,1,0,0],[0,1,0,0,0,0,1,1,0],[0,1,0,1,0,0,1,1,0],[0,0,0,0,0,0,0,1,0],[0,1,0,0,0,0,1,1,0],[0,0,0,0,0,0,0,1,0],[0,0,0,1,0,0,0,0,0],[0,0,1,0,0,0,0,0,0],[0,0,0,1,0,0,0,1,0],[0,0,0,1,0,0,0,0,0],[0,0,1,0,0,0,0,0,0],[0,1,0,0,0,0,0,1,0],[0,0,1,0,1,0,0,0,0],[0,1,0,0,0,0,1,1,0],[0,0,0,0,1,0,0,0,0],[0,0,1,0,0,0,0,0,0],[0,1,0,0,0,0,1,1,0],[0,0,0,0,1,0,0,0,0],[0,0,1,0,0,0,0,0,0],[0,1,0,0,0,0,1,0,0],[0,0,1,0,0,0,0,0,0],[0,0,1,0,0,0,0,0,0],[0,1,0,0,0,0,1,0,0],[0,0,1,0,0,0,0,0,0],[0,0,1,0,0,0,0,0,0],[0,0,1,0,0,0,0,0,0],[0,1,0,0,0,0,1,0,0],[0,1,1,0,0,0,1,1,0],[0,1,1,0,0,0,0,0,0],[0,0,0,1,0,0,0,0,0],[0,1,1,0,0,0,1,0,0],[0,0,1,0,0,0,0,0,0],[0,0,0,0,1,0,0,0,0],[0,0,1,0,0,0,0,0,0],[0,0,0,0,1,0,0,0,0],[0,1,1,0,0,0,1,0,0],[0,0,1,0,0,0,0,1,0],[0,1,0,0,0,0,1,0,0],[0,0,1,0,0,0,0,0,0],[0,0,1,0,0,0,0,0,0],[0,0,1,0,0,0,0,0,0],[0,0,1,0,0,0,0,0,0],[0,0,1,0,0,0,0,0,0],[0,0,1,0,0,0,0,0,0],[0,1,0,0,0,0,1,0,0],[0,0,0,0,0,0,0,1,0],[0,0,1,0,0,0,0,0,0],[0,0,1,0,0,0,0,0,0],[0,0,0,0,1,0,0,0,0],[0,0,1,0,0,0,0,0,0],[0,0,1,0,0,0,0,0,0],[0,0,1,0,0,0,0,0,0],[0,1,0,0,0,0,0,0,0],[0,0,1,0,0,0,0,0,0]]4847	4848,4849,4850,4851,4852,4853,4854,4855,4856,4470,4857,4508	[[0,1,0,0,0,0,0,0,0],[0,1,0,0,0,0,0,0,0],[0,1,0,0,0,0,0,0,0],[0,1,0,0,0,0,0,0,0],[0,1,0,0,0,0,0,0,0],[0,1,0,0,0,0,0,0,0],[0,1,0,0,0,0,0,0,0],[0,1,0,0,0,0,0,0,0],[0,1,0,0,0,0,0,0,0],[0,1,0,0,0,0,0,0,0],[0,1,0,0,0,0,0,0,0],[0,1,0,0,0,0,0,0,0]]4858	4859,4860,4861,4862,4863,4864,4865,4866,4867,4868,3327	[[0,1,0,0,0,0,0,0,0],[0,1,0,0,0,0,0,0,0],[0,1,0,0,0,0,0,0,0],[0,1,0,0,1,0,0,0,0],[0,1,0,0,0,0,0,0,0],[0,1,0,0,0,0,0,0,0],[0,1,0,0,0,0,0,0,0],[0,1,0,0,0,0,0,0,0],[0,1,0,0,0,0,0,0,0],[0,1,0,0,0,0,0,0,0],[0,1,0,0,0,0,0,0,0]]4869	4870,4871	[[0,0,0,0,1,0,0,0,0],[0,0,0,0,1,0,0,0,0]]4872	4873,4874	[[0,0,0,0,1,0,0,0,0],[0,0,0,0,1,0,0,0,0]]4875	4876,4877,1919,4878,4879,1928,4880,64,4832,4881,484,4244,2520,1916,4812,4882,1926,2426,4883,4211,4884,4885,4886,4841,4887,2866,4888,4889,4890,3932,2941,4891,3958,4247,4892,4893,4894,4895,4258,4896,2896,502,4897,3694,4898,3680,4899,1927,4609,4900,4901,4902,1920,4903,4904,4905,4906,4827,4907,4908,4909,4910,4911,4912,4818,4913,4914,1163,4915,1925,3323,4916,4917,3652,4918,1917,4919,4920,4921,4922,4923	[[0,1,0,0,0,0,0,0,0],[0,1,1,0,0,0,0,0,0],[0,1,1,1,0,0,1,0,0],[0,0,1,0,0,0,0,0,0],[0,0,0,0,0,0,0,1,0],[0,1,1,0,0,0,1,0,0],[0,0,1,0,0,0,0,0,0],[0,0,0,1,0,0,0,1,0],[0,1,1,0,0,0,1,0,0],[0,0,1,0,0,0,0,0,0],[0,1,1,0,0,0,1,0,0],[0,1,1,0,1,0,1,1,0],[0,1,1,0,0,0,1,0,0],[0,1,1,0,0,0,1,0,0],[0,1,1,1,1,0,1,0,0],[0,1,0,0,0,0,1,0,0],[0,1,0,0,0,0,1,0,0],[0,1,1,1,1,0,1,1,0],[0,1,1,0,0,0,1,0,0],[0,1,1,1,0,0,1,1,0],[0,1,0,0,0,0,1,0,0],[0,1,0,1,1,0,1,1,0],[0,1,0,0,0,0,1,0,0],[0,0,1,0,0,0,0,0,0],[0,0,1,0,0,0,0,0,0],[0,0,0,0,0,0,0,1,0],[0,0,0,0,0,0,0,1,0],[0,0,1,0,0,0,0,0,0],[0,0,1,0,0,0,0,0,0],[0,1,0,0,0,0,1,0,0],[0,0,1,0,0,0,0,0,0],[0,0,1,0,0,0,0,0,0],[0,0,1,0,0,0,0,0,0],[0,0,1,0,0,0,0,0,0],[0,1,1,0,0,0,1,0,0],[0,0,1,0,0,0,0,0,0],[0,0,1,0,0,0,0,0,0],[0,0,1,0,0,0,0,0,0],[0,0,1,0,0,0,0,1,0],[0,0,1,0,0,0,0,0,0],[0,0,1,0,0,0,0,0,0],[0,0,1,0,0,0,0,0,0],[0,0,0,0,1,0,0,0,0],[0,0,1,0,0,0,0,0,0],[0,0,1,0,0,0,0,0,0],[0,1,1,0,0,0,1,0,0],[0,0,1,0,0,0,0,0,0],[0,0,1,0,0,0,0,0,0],[0,0,0,0,1,0,0,0,0],[0,1,0,0,0,0,1,0,0],[0,0,1,0,0,0,0,0,0],[0,0,1,0,0,0,0,0,0],[0,0,1,0,0,0,0,0,0],[0,0,1,0,0,0,0,0,0],[0,0,0,0,1,0,0,0,0],[0,0,0,0,0,0,1,0,0],[0,0,1,0,0,0,0,0,0],[0,1,1,0,0,0,1,0,0],[0,1,1,0,0,0,1,0,0],[0,1,1,1,0,0,1,0,0],[0,0,1,0,0,0,0,0,0],[0,0,0,0,0,0,0,1,0],[0,0,0,0,0,0,0,1,0],[0,1,1,0,0,0,1,0,0],[0,0,1,0,0,0,0,0,0],[0,0,1,0,0,0,0,0,0],[0,0,1,0,0,0,0,0,0],[0,0,1,0,0,0,0,1,0],[0,0,1,0,0,0,0,0,0],[0,0,1,0,1,0,0,0,0],[0,0,1,0,0,0,0,0,0],[0,0,1,0,0,0,0,0,0],[0,0,1,0,0,0,0,0,0],[0,0,1,0,0,0,0,0,0],[0,0,0,0,0,0,0,1,0],[0,0,1,0,0,0,0,0,0],[0,0,1,0,0,0,0,0,0],[0,0,1,0,0,0,0,0,0],[0,0,0,0,0,0,0,1,0],[0,0,1,0,0,0,0,0,0],[0,0,1,0,0,0,0,0,0]]4924	4096,3388,1571	[[0,0,0,1,0,0,0,0,0],[0,0,0,1,0,0,0,0,0],[0,0,0,1,0,0,0,0,0]]4925	4926,4927	[[0,0,0,0,1,0,0,0,0],[0,0,0,0,1,0,0,0,0]]4928	4929,2132,4930	[[0,0,0,0,1,0,0,0,0],[0,0,0,0,1,0,0,0,0],[0,0,0,0,1,0,0,0,0]]4931	4932,4933,4934	[[0,0,0,0,1,0,0,0,0],[0,0,0,0,1,0,0,0,0],[0,0,0,0,1,0,0,0,0]]4935	4936,4937,4938	[[0,0,0,0,1,0,0,0,0],[0,0,0,0,1,0,0,0,0],[0,0,0,0,1,0,0,0,0]]4939	4940	[[0,0,0,0,1,0,0,0,0]]4941	4942,4943	[[0,0,0,0,1,0,0,0,0],[0,0,0,0,1,0,0,0,0]]4944	4945,4946,4947	[[0,0,0,0,1,0,0,0,0],[0,0,0,1,1,0,0,0,0],[0,0,0,0,1,0,0,0,0]]4948	4949,4950,4951,4952,232,230,4953,4954,4955,4956,4957,4958,4959,4960,4961,4962,4963,4964,4965,4966,4967,4968,4969,4970,4971,4972,4973,4974	[[0,0,1,0,0,0,0,0,0],[0,1,0,0,0,0,0,0,0],[0,1,0,0,0,0,0,0,0],[0,1,0,0,0,0,0,0,0],[0,1,1,1,0,0,0,0,0],[0,0,0,0,1,0,0,0,0],[0,1,1,0,0,0,0,0,0],[0,0,1,0,0,0,0,0,0],[0,0,1,0,0,0,0,0,0],[0,1,0,0,0,0,0,0,0],[0,1,0,0,0,0,0,0,0],[0,1,0,0,0,0,0,0,0],[0,0,1,0,0,0,0,0,0],[0,1,0,0,0,0,0,0,0],[0,1,1,1,0,0,0,0,0],[0,1,0,0,0,0,0,0,0],[0,0,1,1,0,0,0,0,0],[0,0,1,0,0,0,0,0,0],[0,0,1,0,0,0,0,0,0],[0,1,1,0,0,0,0,0,0],[0,0,1,0,0,0,0,0,0],[0,0,1,0,0,0,0,0,0],[0,0,1,0,0,0,0,0,0],[0,0,1,0,0,0,0,0,0],[0,0,1,0,0,0,0,0,0],[0,0,1,0,0,0,0,0,0],[0,0,1,0,0,0,0,0,0],[0,0,1,0,0,0,0,0,0]]4975	4976	[[0,0,0,0,1,0,0,0,0]]4977	4978,1222,4979,4980,4981,4982	[[0,0,0,0,1,0,0,0,0],[0,0,0,1,0,0,0,0,0],[0,0,0,0,1,0,0,0,0],[0,0,0,0,1,0,0,0,0],[0,0,0,1,0,0,0,0,0],[0,0,0,1,0,0,0,0,0]]4983	4984,3684,4985,4986,4987,2260,4988,849,944,4989,4990,4991,132,1912,2868,4992,1575,26,4993,2249,4994,2266,4995,4996,4997,4998,1304,2887,4999,2261,1309,3226,5000,5001,3525,3092,5002,5003,5004,5005,5006,826,5007,5008,4218,5009,3879,5010,3964,3921,2255,5011,1143,5012,5013,5014,5015,3883,795,794,2137	[[0,1,1,0,0,0,0,0,0],[0,1,1,1,0,0,0,0,1],[0,1,1,1,0,0,0,1,0],[0,1,1,1,0,0,0,0,0],[0,0,1,0,0,0,0,1,1],[0,0,0,0,0,0,0,1,0],[0,1,0,0,0,0,0,0,0],[0,1,1,1,1,0,0,1,1],[0,1,0,0,0,0,0,0,0],[0,0,1,0,0,0,0,0,0],[0,0,0,1,0,0,0,1,0],[0,0,1,0,0,0,0,0,0],[0,0,1,0,0,0,0,0,0],[0,0,0,0,0,0,0,1,0],[0,0,1,0,0,0,0,0,0],[0,0,0,0,0,0,0,1,0],[0,0,0,0,0,0,0,1,0],[0,0,1,0,0,0,0,0,0],[0,0,1,0,0,0,0,0,0],[0,0,1,0,0,0,0,0,0],[0,0,1,0,0,0,0,0,0],[0,1,0,1,0,0,0,1,0],[0,0,1,0,0,0,0,0,0],[0,1,0,0,0,0,0,0,0],[0,0,1,0,0,0,0,0,0],[0,0,1,0,0,0,0,0,0],[0,0,1,0,0,0,0,0,0],[0,0,1,0,0,0,0,0,0],[0,0,1,0,0,0,0,0,0],[0,0,0,0,0,0,0,1,0],[0,0,1,0,0,0,0,0,0],[0,0,1,0,0,0,0,0,0],[0,0,1,0,0,0,0,0,0],[0,0,1,0,0,0,0,0,0],[0,0,0,0,0,0,0,1,0],[0,0,1,0,0,0,0,0,0],[0,0,1,0,0,0,0,0,0],[0,0,0,0,1,0,0,0,0],[0,0,0,0,0,0,0,0,1],[0,0,1,0,0,0,0,0,0],[0,0,1,0,0,0,0,0,1],[0,0,1,0,0,0,0,0,0],[0,0,1,0,0,0,0,0,0],[0,0,1,0,0,0,0,0,0],[0,0,1,0,0,0,0,0,0],[0,0,1,0,0,0,0,0,0],[0,0,1,0,0,0,0,0,0],[0,0,1,0,0,0,0,0,0],[0,0,1,0,0,0,0,1,1],[0,0,1,0,0,0,0,0,0],[0,0,1,0,0,0,0,1,0],[0,0,1,0,0,0,0,0,0],[0,0,1,0,0,0,0,0,0],[0,0,1,0,0,0,0,1,0],[0,0,1,0,0,0,0,0,0],[0,0,1,0,0,0,0,0,0],[0,0,1,0,0,0,0,0,0],[0,0,1,0,1,0,0,0,0],[0,0,1,0,0,0,0,1,0],[0,0,1,0,0,0,0,1,0],[0,0,1,0,0,0,0,0,0]]5016	3774,5017,5018,4756,5019,5020,898,5021,5022,5023,415,5024,5025,5026,5027,3417,2131,5028,5029,5030,5031,5032,4580,5033,5034,202,5035,2947,5036,2502,5037,5038,5039,5040,5041,5042,5043	[[0,1,1,1,0,0,0,0,0],[0,0,0,0,1,0,1,0,0],[0,0,1,1,0,0,0,0,0],[0,0,0,0,1,0,0,0,0],[0,1,1,1,0,0,1,0,0],[0,0,1,1,0,0,0,0,0],[0,0,1,0,0,0,0,0,0],[0,1,1,1,0,0,0,0,0],[0,1,0,0,0,0,0,0,0],[0,1,0,0,0,0,0,0,0],[0,1,0,0,0,0,0,0,0],[0,1,1,1,0,0,1,0,0],[0,1,1,0,0,0,1,0,0],[0,1,1,1,0,0,1,0,0],[0,1,1,1,0,0,1,0,0],[0,1,1,1,0,0,0,0,0],[0,0,1,0,0,0,0,0,0],[0,0,0,1,0,0,0,0,0],[0,1,1,1,0,0,0,0,0],[0,0,1,0,0,0,0,0,0],[0,0,1,0,0,0,0,0,0],[0,0,0,1,0,0,0,0,0],[0,0,0,0,1,0,0,0,0],[0,0,1,1,0,0,0,0,0],[0,0,1,0,0,0,0,0,0],[0,0,1,1,1,0,0,0,0],[0,0,0,1,0,0,0,0,0],[0,0,1,0,0,0,0,0,0],[0,0,1,1,0,0,1,0,0],[0,0,0,1,0,0,0,0,0],[0,0,1,1,1,0,0,0,0],[0,0,1,0,0,0,0,0,0],[0,0,1,0,0,0,0,0,0],[0,0,1,1,0,0,0,0,0],[0,0,0,1,1,0,0,0,0],[0,0,0,0,0,0,1,0,0],[0,0,0,0,1,0,0,0,0]]5044	5045,5046	[[0,0,0,0,1,0,0,0,0],[0,0,0,0,1,0,0,0,0]]5047	5048,5049	[[0,0,0,0,1,0,0,0,0],[0,0,0,0,1,0,0,0,0]]5050	232,4961,4963	[[0,0,0,1,0,0,0,0,0],[0,0,0,1,0,0,0,0,0],[0,0,0,1,0,0,0,0,0]]5051	2023	[[0,0,0,0,1,0,0,0,0]]5052	5053	[[0,0,0,0,1,0,0,0,0]]5054	5055,5056,5057,5058,5059,2130,5060,5061,5062,5063,5064,5065,5066,5067,5068,3392,3712,3744,5069,3764,5070,5071,5072,2703,5073,5001,5074,5075,5076,5077,3658	[[0,1,1,0,0,0,0,0,0],[0,0,0,1,0,0,0,0,0],[0,1,1,0,0,0,0,0,0],[0,1,1,0,0,0,0,0,0],[0,1,1,0,0,0,0,0,0],[0,1,0,0,0,0,0,0,0],[0,0,0,1,0,0,0,0,0],[0,0,1,0,0,0,0,0,0],[0,0,0,1,0,0,0,0,0],[0,0,0,1,0,0,0,0,0],[0,0,0,1,0,0,0,0,0],[0,1,1,1,0,0,0,0,0],[0,1,1,1,1,0,0,0,0],[0,1,1,0,0,0,0,0,0],[0,1,1,0,0,0,0,0,0],[0,1,0,0,0,0,0,0,0],[0,0,0,1,0,0,0,0,0],[0,0,0,1,0,0,0,0,0],[0,0,0,1,0,0,0,0,0],[0,0,0,1,0,0,0,0,0],[0,0,0,1,0,0,0,0,0],[0,0,0,1,0,0,0,0,0],[0,0,0,1,0,0,0,0,0],[0,0,0,1,0,0,0,0,0],[0,0,1,0,0,0,0,0,0],[0,0,0,1,0,0,0,0,0],[0,0,0,1,0,0,0,0,0],[0,0,1,1,0,0,0,0,0],[0,0,1,1,0,0,0,0,0],[0,0,1,0,0,0,0,0,0],[0,0,0,1,0,0,0,0,0]]5078	4981,1222,4982	[[0,0,0,1,0,0,0,0,0],[0,0,0,1,0,0,0,0,0],[0,0,0,1,0,0,0,0,0]]5079	5080,5070,5081,5082,5083,5084,668,5085,2239,5086,5087,5088,5089,5090,5091,5092,5093,5094,5095,5096,5097,5098	[[0,0,0,1,0,0,0,0,0],[0,0,0,1,0,0,0,0,0],[0,0,0,1,1,0,0,0,0],[0,0,0,1,0,0,0,0,0],[0,0,0,1,0,0,0,0,0],[0,0,0,0,1,0,0,0,0],[0,0,0,1,0,0,0,0,0],[0,0,0,1,1,0,0,0,0],[0,0,0,0,1,0,0,0,0],[0,0,0,1,0,0,0,0,0],[0,0,0,1,1,0,0,0,0],[0,0,0,1,0,0,0,0,0],[0,0,0,1,0,0,0,0,0],[0,0,0,1,0,0,0,0,0],[0,0,0,1,0,0,0,0,0],[0,0,0,1,0,0,0,0,0],[0,0,0,1,0,0,0,0,0],[0,0,0,1,0,0,0,0,0],[0,0,0,0,1,0,0,0,0],[0,0,0,0,1,0,0,0,0],[0,0,0,1,0,0,0,0,0],[0,0,0,1,0,0,0,0,0]]5099	5100	[[0,0,0,0,1,0,0,0,0]]5101	5102,5103,5104,5105	[[0,0,0,0,1,0,0,0,0],[0,0,0,0,1,0,0,0,0],[0,0,0,0,1,0,0,0,0],[0,0,0,0,1,0,0,0,0]]5106	5107	[[0,0,0,0,1,0,0,0,0]]5108	5109	[[0,0,0,0,1,0,0,0,0]]5110	5111,5112,5113,5114	[[0,0,0,0,1,0,0,0,0],[0,0,0,0,1,0,0,0,0],[0,0,0,0,1,0,0,0,0],[0,0,0,0,1,0,0,0,0]]5115	5116	[[0,0,0,0,1,0,0,0,0]]5117	5118,5119,5120	[[0,0,0,0,1,0,0,0,0],[0,0,0,0,1,0,0,0,0],[0,0,0,0,1,0,0,0,0]]5121	5122,5123,5124,3217,2711,3214,3232,5125,5126,5127,2912,5128,632,3192,3213,5129,5130,3218,5131,3236,3219,5132,5133,5134,5135,3224,5136,5137,2129,5138,3675,5139,2152,5140,5141,3239,5142,3228,3225,3215,1300,5143,3229,5144,5145,3284,2132,5146,3240,5147	[[0,0,1,0,0,0,0,0,0],[0,0,1,0,0,0,0,0,0],[0,0,1,0,0,0,0,0,0],[0,1,1,0,0,0,0,0,0],[0,0,1,0,0,0,0,0,0],[0,0,1,0,0,0,0,0,0],[0,1,1,1,1,0,0,1,0],[0,1,0,0,0,0,0,0,0],[0,0,1,0,0,0,0,0,0],[0,0,1,0,0,0,0,0,0],[0,0,1,0,0,0,0,0,0],[0,0,1,0,0,0,0,0,0],[0,1,1,0,0,0,0,0,0],[0,0,1,0,0,0,0,0,0],[0,0,1,0,0,0,0,0,0],[0,0,1,0,0,0,0,0,0],[0,0,1,0,0,0,0,1,0],[0,1,1,0,1,0,0,0,0],[0,0,0,0,0,0,0,1,0],[0,0,0,0,0,0,0,1,0],[0,0,1,0,0,0,0,0,0],[0,1,0,0,0,0,0,0,0],[0,0,1,0,0,0,0,0,0],[0,1,0,0,0,0,0,0,0],[0,0,1,0,0,0,0,0,0],[0,0,1,0,0,0,0,0,0],[0,0,0,0,0,0,0,1,0],[0,0,1,0,0,0,0,0,0],[0,0,1,0,0,0,0,0,0],[0,0,1,0,0,0,0,0,0],[0,0,1,0,0,0,0,0,0],[0,0,0,0,0,0,0,1,0],[0,0,1,0,0,0,0,1,0],[0,0,1,0,0,0,0,0,0],[0,0,0,0,0,0,0,1,0],[0,0,1,0,0,0,0,0,0],[0,0,1,0,0,0,0,0,0],[0,1,1,1,1,0,0,0,0],[0,0,1,0,0,0,0,0,0],[0,0,1,0,0,0,0,0,0],[0,0,1,0,0,0,0,0,0],[0,0,1,0,0,0,0,0,0],[0,0,0,0,0,0,0,1,0],[0,0,1,0,0,0,0,0,0],[0,0,1,0,0,0,0,0,0],[0,0,1,0,0,0,0,0,0],[0,0,1,0,0,0,0,0,0],[0,0,1,0,0,0,0,0,0],[0,0,0,0,0,0,0,1,0],[0,0,1,0,0,0,0,0,0]]5148	5149,5150	[[0,0,0,0,1,0,0,0,0],[0,0,0,0,1,0,0,0,0]]5151	5152,4587,3652,5153,5154,5155,1520,5156,3401,2353,5157,5158,4014,5159,5160,5161,5162,5163,5164,5165,5166,4880,5167,5168,5169,1742,1743,5170,1734,3050,1739,1748,3047,5171,4268,4270,1735,5172,1736,1744,5173,1738,4289,4279,5174,4273,4278,4281,5175,5176,5177,4818,5178	[[0,0,1,0,0,0,0,0,0],[0,0,1,0,0,0,0,0,0],[0,0,1,0,0,0,0,0,0],[0,0,1,0,0,0,0,1,0],[0,0,1,0,0,0,0,1,0],[0,1,0,0,0,0,1,1,0],[0,0,1,0,0,0,0,0,0],[0,0,1,0,0,0,0,0,0],[0,0,1,0,0,0,0,1,0],[0,0,1,0,0,0,1,0,0],[0,0,1,0,0,0,0,0,0],[0,0,1,0,0,0,0,0,0],[0,0,1,0,0,0,0,1,0],[0,1,0,0,0,0,0,0,0],[0,1,0,0,0,0,0,0,0],[0,1,0,0,0,0,0,0,0],[0,1,0,0,0,0,0,0,0],[0,1,0,0,0,0,0,0,0],[0,1,0,0,0,0,0,0,0],[0,1,0,0,0,0,0,0,0],[0,1,0,0,0,0,0,0,0],[0,0,1,0,0,0,0,1,0],[0,0,1,0,0,0,0,0,0],[0,0,1,0,0,0,0,0,0],[0,1,1,0,1,0,1,1,0],[0,1,0,0,0,0,0,0,0],[0,1,0,0,0,0,0,0,0],[0,1,1,0,1,0,1,1,0],[0,1,0,0,0,0,0,0,0],[0,0,1,0,0,0,0,0,0],[0,1,0,0,0,0,0,0,0],[0,0,1,0,0,0,0,0,0],[0,0,1,0,0,0,0,1,0],[0,0,0,0,1,0,0,0,0],[0,1,0,0,0,0,0,0,0],[0,1,0,0,0,0,0,0,0],[0,1,0,0,0,0,0,0,0],[0,1,0,0,0,0,0,0,0],[0,1,0,0,0,0,0,0,0],[0,1,0,0,0,0,0,0,0],[0,0,1,0,0,0,0,0,0],[0,1,0,0,0,0,0,0,0],[0,1,0,0,0,0,0,0,0],[0,1,0,0,0,0,0,0,0],[0,1,0,0,0,0,0,0,0],[0,1,0,0,0,0,0,0,0],[0,1,0,0,0,0,0,0,0],[0,1,0,0,0,0,0,0,0],[0,0,1,0,0,0,0,0,0],[0,1,0,0,0,0,0,0,0],[0,0,1,0,0,0,0,0,0],[0,0,1,0,0,0,0,0,0],[0,0,0,0,0,0,0,1,0]]5179	5180,5181,5182,5183	[[0,0,0,1,0,0,0,0,0],[0,0,0,1,0,0,0,0,0],[0,0,0,1,0,0,0,0,0],[0,0,0,0,1,0,0,0,0]]5184	5185,954,5186,5187,953,5157,5188,5189,5190,5191,5192,5193,5194,962,5195,5196,5197,5198,5199,957,3899,5200,5201,959,5202,5203,5204,960,966,4600,5205,968,964,958,5206	[[0,0,1,0,0,0,0,0,0],[0,0,1,0,0,0,0,0,0],[0,0,1,0,0,0,0,0,0],[0,0,1,0,0,0,0,0,0],[0,0,1,0,0,0,0,0,0],[0,0,1,0,0,0,0,0,0],[0,0,1,0,0,0,0,0,0],[0,0,1,0,0,0,0,0,0],[0,0,1,0,1,0,0,0,0],[0,0,1,0,0,0,0,0,0],[0,0,1,0,0,0,0,0,0],[0,0,1,0,0,0,0,0,0],[0,0,1,0,0,0,0,0,0],[0,0,1,0,0,0,0,0,0],[0,0,1,0,0,0,0,0,0],[0,0,1,0,0,0,0,0,0],[0,0,1,0,0,0,0,0,0],[0,0,1,0,0,0,0,0,0],[0,0,1,0,0,0,0,0,0],[0,0,0,0,1,0,0,0,0],[0,0,1,0,0,0,0,0,0],[0,0,1,0,0,0,0,0,0],[0,0,1,0,0,0,0,0,0],[0,0,1,0,0,0,0,0,0],[0,0,1,0,0,0,0,0,0],[0,0,1,0,0,0,0,0,0],[0,0,1,0,0,0,0,0,0],[0,0,1,0,1,0,0,0,0],[0,0,1,0,0,0,0,0,0],[0,0,1,0,0,0,0,0,0],[0,0,1,0,0,0,0,0,0],[0,0,1,0,0,0,0,0,0],[0,0,0,0,1,0,0,0,0],[0,0,1,0,0,0,0,0,0],[0,0,1,0,0,0,0,0,0]]5207	5208,5209	[[0,0,0,0,1,0,0,0,0],[0,0,0,0,1,0,0,0,0]]5210	5211,5212,5213	[[0,0,0,0,1,0,0,0,0],[0,0,0,0,1,0,0,0,0],[0,0,0,0,1,0,0,0,0]]5214	5215,2920,5216,5217,5218,5219,5220,5221,91,5222,2940,5223,5224,5225,5226,2938,5227,2488,5228,2896,94,2919,546,74,5229,5230	[[0,0,1,0,0,0,0,0,0],[0,0,1,0,0,0,0,0,0],[0,0,0,0,1,0,0,0,0],[0,0,0,1,0,0,0,0,0],[0,0,1,0,0,0,0,0,0],[0,0,1,0,0,0,0,0,0],[0,0,1,0,0,0,0,0,0],[0,0,1,0,0,0,0,0,0],[0,0,1,0,0,0,0,0,0],[0,0,1,0,0,0,0,0,0],[0,0,1,0,0,0,0,0,0],[0,0,1,0,0,0,0,0,0],[0,0,1,0,0,0,0,0,0],[0,0,1,0,0,0,0,0,0],[0,0,1,0,0,0,0,0,0],[0,0,1,0,0,0,0,0,0],[0,0,1,1,0,0,0,0,0],[0,0,0,1,0,0,0,0,0],[0,0,1,0,0,0,0,0,0],[0,0,0,1,0,0,0,0,0],[0,0,1,0,0,0,0,0,0],[0,0,1,0,0,0,0,0,0],[0,0,0,1,0,0,0,0,0],[0,0,0,1,0,0,0,0,0],[0,0,1,0,0,0,0,0,0],[0,0,1,0,0,0,0,0,0]]5231	5232,5233,5234,5235,5236,5237,5238,5239,5240,5241,3569,5242,3155,5243,181,5244,5245,5246,5247,5248,5249,5250,5251,2024,5252,5253,5254,5255,4285,5256,5257,5258,5259,5260,5261,5262,3561,3411,5263,5264,5265,4699,5266,5267,5268,5269,5270,5271,5272,5273,5274,5275,3166,3436,1602,5276,5277,5278,5279,3424,5280,5281,5282,5283,5284,5285,5286,1899,5287,5288,5289,5290,5291,5292,5293,5294,5295,5296,3437,5297,5298,5299	[[0,1,1,1,1,0,0,1,0],[0,1,1,1,0,0,0,1,0],[0,0,1,0,0,0,0,0,0],[0,0,1,1,0,0,0,0,0],[0,0,1,0,0,0,0,0,0],[0,0,1,0,0,0,0,0,0],[0,0,1,0,0,0,0,0,0],[0,1,1,1,0,0,1,1,0],[0,1,1,1,0,0,1,0,0],[0,1,1,1,0,0,1,1,0],[0,0,1,0,0,0,0,1,0],[0,1,0,0,0,0,1,0,0],[0,0,1,0,0,0,0,0,0],[0,1,1,1,0,0,1,1,0],[0,1,1,1,1,0,1,1,0],[0,1,1,1,0,0,0,1,0],[0,0,1,0,0,0,0,0,0],[0,0,1,0,0,0,0,0,0],[0,0,1,0,0,0,0,0,0],[0,0,1,0,0,0,0,0,0],[0,0,0,0,0,0,0,1,0],[0,0,1,0,0,0,0,0,0],[0,1,0,0,0,0,0,0,0],[0,1,1,1,0,0,1,1,0],[0,1,1,1,0,0,0,1,0],[0,1,1,1,0,0,1,0,0],[0,1,1,1,1,0,1,1,0],[0,1,1,0,0,0,1,1,0],[0,1,1,1,1,0,1,0,0],[0,1,0,0,0,0,0,0,0],[0,1,1,1,1,0,0,1,0],[0,1,1,1,0,0,1,1,0],[0,1,1,0,0,0,0,0,0],[0,1,1,1,0,0,0,0,0],[0,1,1,1,0,0,1,0,0],[0,1,1,1,0,0,1,1,0],[0,1,0,0,0,0,0,0,0],[0,1,0,0,0,0,0,0,0],[0,1,0,0,0,0,1,1,0],[0,1,0,0,0,0,0,0,0],[0,1,1,1,0,0,0,0,0],[0,1,0,0,0,0,0,0,0],[0,0,1,1,0,0,0,0,0],[0,0,1,0,0,0,0,0,0],[0,0,1,0,0,0,0,0,0],[0,0,1,1,0,0,0,0,0],[0,0,1,0,0,0,0,0,0],[0,0,1,0,0,0,0,0,0],[0,0,1,0,0,0,0,1,0],[0,0,1,1,0,0,0,0,0],[0,0,1,1,0,0,0,1,0],[0,0,1,1,0,0,0,1,0],[0,0,1,1,0,0,0,0,0],[0,0,1,0,0,0,0,0,0],[0,0,0,0,0,0,0,1,0],[0,0,1,0,0,0,0,0,0],[0,0,1,0,0,0,0,0,0],[0,0,1,0,0,0,0,0,0],[0,0,1,0,0,0,0,0,0],[0,0,1,1,0,0,0,0,0],[0,0,1,0,0,0,0,0,0],[0,0,1,0,0,0,0,1,0],[0,0,1,0,0,0,0,0,0],[0,0,0,1,0,0,0,0,0],[0,0,0,1,0,0,0,0,0],[0,0,1,0,0,0,0,0,0],[0,0,1,0,0,0,0,1,0],[0,0,1,0,0,0,0,0,0],[0,0,1,0,0,0,0,0,0],[0,0,0,1,0,0,0,1,0],[0,0,1,0,0,0,0,0,0],[0,0,0,1,0,0,0,0,0],[0,0,1,0,0,0,0,0,0],[0,0,0,1,0,0,0,0,0],[0,0,1,0,0,0,0,0,0],[0,0,1,0,0,0,0,1,0],[0,0,1,1,0,0,0,0,0],[0,0,1,1,0,0,0,0,0],[0,0,0,1,0,0,0,0,0],[0,0,1,0,0,0,0,0,0],[0,0,0,1,0,0,0,0,0],[0,0,1,0,0,0,0,0,0]]5300	5301,5302	[[0,0,0,0,1,0,0,0,0],[0,0,0,0,1,0,0,0,0]]5303	5304,5305,5306,5307,5308,5309	[[0,0,0,0,1,0,0,0,0],[0,0,0,0,1,0,0,0,0],[0,0,0,0,1,0,0,0,0],[0,0,0,0,1,0,0,0,0],[0,0,0,0,1,0,0,0,0],[0,0,0,0,1,0,0,0,0]]5310	1352,4972,5311,5312,5313,5314,5315,5316,5317,5318,5319,5320,4971,5321,5322,2967,5323,5324,5325,5326,5327,5328,5329,5330,5331,5332	[[0,0,0,1,0,0,0,0,0],[0,0,0,1,0,0,0,0,0],[0,0,0,1,0,0,0,0,0],[0,0,0,1,0,0,0,0,0],[0,0,0,1,0,0,0,0,0],[0,0,0,1,0,0,0,0,0],[0,0,0,0,1,0,0,0,0],[0,0,0,1,0,0,0,0,0],[0,0,0,1,0,0,0,0,0],[0,0,0,1,0,0,0,0,0],[0,0,0,1,0,0,0,0,0],[0,0,0,1,0,0,0,0,0],[0,0,0,1,0,0,0,0,0],[0,0,0,1,0,0,0,0,0],[0,0,0,1,0,0,0,0,0],[0,0,0,1,0,0,0,0,0],[0,0,0,1,0,0,0,0,0],[0,0,0,1,0,0,0,0,0],[0,0,0,1,0,0,0,0,0],[0,0,0,1,0,0,0,0,0],[0,0,0,1,0,0,0,0,0],[0,0,0,1,0,0,0,0,0],[0,0,0,1,0,0,0,0,0],[0,0,0,1,0,0,0,0,0],[0,0,0,1,0,0,0,0,0],[0,0,0,1,0,0,0,0,0]]5333	5334	[[0,0,0,0,1,0,0,0,0]]5335	1556,5336,5337,1545,5142,1586,5338,5339,5340,5341,5342,5343,5344,5345,1609,5346,5347,5348,5349,5350,561,5351,5352,5353,5145,1767,5354,5355,5356,5357,5358,5359,5360,5361,5362,5363,5364,5365,69,5366,5367,5368,5369,5370,5371,5372	[[1,0,0,0,0,0,0,0,0],[0,1,1,0,0,0,0,0,0],[1,0,1,0,0,0,0,0,0],[1,0,0,0,0,0,0,0,0],[0,1,0,0,0,0,1,0,0],[1,0,1,0,0,0,0,0,0],[0,1,1,0,0,0,1,0,0],[1,1,1,0,1,0,1,0,0],[1,0,0,0,0,0,0,0,0],[1,0,0,0,0,0,0,0,0],[1,0,1,0,0,0,0,0,0],[1,0,0,0,0,0,0,0,0],[1,0,0,0,0,0,0,0,0],[0,1,0,0,0,0,1,0,0],[1,0,0,0,0,0,0,0,0],[0,1,0,0,0,0,1,0,0],[1,0,0,0,0,0,0,0,0],[0,0,0,0,1,0,1,0,0],[0,1,0,0,0,0,1,0,0],[0,1,1,0,1,0,1,0,0],[1,0,0,0,0,0,0,0,0],[0,0,1,0,1,0,0,0,0],[0,1,0,0,0,0,1,0,0],[0,0,1,0,0,0,0,0,0],[0,1,0,0,0,0,1,0,0],[1,0,0,0,0,0,0,0,0],[1,0,0,0,1,0,0,0,0],[0,1,0,0,0,0,1,0,0],[1,0,0,0,0,0,0,0,0],[1,1,0,0,1,0,1,0,0],[1,0,0,0,0,0,0,0,0],[0,0,0,0,1,0,0,0,0],[0,0,1,0,0,0,0,0,0],[0,1,0,0,0,0,1,0,0],[0,1,0,0,0,0,1,0,0],[0,1,0,0,0,0,0,0,0],[0,1,0,0,0,0,1,0,0],[0,0,0,0,0,0,1,0,0],[0,0,1,0,0,0,0,0,0],[0,1,1,0,0,0,1,0,0],[0,1,0,0,0,0,0,0,0],[0,1,0,0,0,0,0,0,0],[0,0,0,0,1,0,0,0,0],[0,1,0,0,0,0,1,0,0],[0,1,0,0,0,0,0,0,0],[0,1,0,0,0,0,1,0,0]]5373	5374,5375	[[0,0,0,0,1,0,0,0,0],[0,0,0,0,1,0,0,0,0]]5376	5377,5378,5379,5380,5381,5382,5383,5384,5385,5386,5387,5176,5388,5389,5390,4653,4225,3446,5391,5392,4687,5393,5394,5395,5396,5397,5398,5399,4678,5400,5401,5402,4633,5403,5404,5405,5406,4626,5407	[[0,1,1,0,0,0,0,0,0],[0,1,1,1,0,0,1,1,1],[0,0,1,0,0,0,0,0,0],[0,1,1,0,0,0,1,1,0],[0,1,1,0,0,0,0,0,0],[0,1,1,0,0,0,0,0,0],[0,1,1,0,0,0,1,0,1],[0,1,0,0,0,0,0,0,0],[0,1,1,0,1,0,0,1,1],[0,0,1,0,0,0,0,1,0],[0,1,1,1,0,0,1,1,0],[0,1,1,0,0,0,1,1,1],[0,1,1,0,0,0,1,0,0],[0,1,1,0,0,0,1,0,0],[0,0,1,0,0,0,0,0,0],[0,0,1,0,0,0,0,0,0],[0,0,1,0,0,0,0,0,0],[0,0,1,0,0,0,0,0,0],[0,1,1,1,1,0,1,1,1],[0,0,0,0,0,0,0,1,1],[0,0,1,1,0,0,0,1,0],[0,0,0,0,0,0,0,1,0],[0,0,0,0,0,0,0,1,1],[0,0,1,0,0,0,0,0,0],[0,0,1,0,0,0,0,0,0],[0,0,1,0,0,0,0,0,0],[0,0,1,0,0,0,0,0,0],[0,0,1,0,0,0,0,0,0],[0,0,1,0,0,0,0,0,0],[0,0,1,0,0,0,0,0,0],[0,0,1,0,0,0,0,0,0],[0,0,1,0,0,0,0,0,0],[0,0,1,0,0,0,0,1,0],[0,0,0,0,0,0,0,1,0],[0,0,1,0,0,0,0,1,0],[0,0,1,0,0,0,0,0,0],[0,0,1,0,0,0,0,0,0],[0,0,0,0,0,0,0,1,0],[0,0,0,0,0,0,0,1,0]]5408	5409,5410,1333,4669,5411,5412	[[0,0,0,1,0,0,0,0,0],[0,0,0,1,0,0,0,0,0],[0,0,0,1,0,0,0,0,0],[0,0,0,1,0,0,0,0,0],[0,0,0,1,0,0,0,0,0],[0,0,0,0,1,0,0,0,0]]5413	5414,5415,45,5416	[[0,0,0,1,0,0,0,0,0],[0,0,0,1,0,0,0,0,0],[0,0,0,1,0,0,0,0,0],[0,0,0,1,0,0,0,0,0]]5417	5418,5419,5420	[[0,0,0,1,0,0,0,0,0],[0,0,0,1,0,0,0,0,0],[0,0,0,1,0,0,0,0,0]]5421	5422	[[0,0,0,0,1,0,0,0,0]]5423	5424,5425	[[0,0,0,0,1,0,0,0,0],[0,0,0,0,1,0,0,0,0]]5426	5427	[[0,0,0,0,1,0,0,0,0]]5428	5411,5409,5410,4669,1333	[[0,0,0,1,0,0,0,0,0],[0,0,0,1,0,0,0,0,0],[0,0,0,1,0,0,0,0,0],[0,0,0,1,0,0,0,0,0],[0,0,0,1,0,0,0,0,0]]5429	5378,5391,4687,5387	[[0,0,0,1,0,0,0,0,0],[0,0,0,1,0,0,0,0,0],[0,0,0,1,0,0,0,0,0],[0,0,0,1,0,0,0,0,0]]5430	5431,5432,985,5433,994,5434,1721,5435,5436,5437,2428,3410,5438,5439,2225,1259,1361,3409,370,5440,2999,5441,2239,5442,1590,5443,5444,5039,996,1356,2997,3415,5445,5446,5447,5448,5449,5450,5451,5452,1132,925,5453,5454,5152,5455,2244,5456,391,5457,392,5458,3419,5459,377,5460,5461,5462,979,1153,5463,5464,5316,5465,5466,5467,2213,5468,5469,5470,5471,5472,407,5473,5474,976,3655,5475,5476,5477,5478,5479,5480,5481,369,5482	[[0,0,1,0,0,0,0,0,0],[0,0,1,0,0,0,0,0,0],[0,0,1,0,0,0,0,0,0],[0,1,1,0,0,0,1,0,0],[0,1,1,0,0,0,1,0,0],[0,1,1,0,0,0,1,0,0],[0,0,1,0,0,0,0,0,0],[0,0,1,0,0,0,0,0,0],[0,1,0,0,1,0,1,0,0],[0,1,1,1,0,0,1,0,1],[0,1,1,0,0,0,0,0,0],[0,1,1,0,0,0,0,0,0],[0,1,0,0,0,0,0,0,0],[0,1,1,0,0,0,1,0,0],[0,1,1,0,0,0,1,0,0],[0,1,1,0,0,0,1,0,0],[0,1,1,0,0,0,1,0,0],[0,1,1,0,0,0,1,0,0],[0,1,1,0,0,0,1,0,0],[0,1,1,0,0,0,1,0,1],[0,0,1,0,0,0,0,0,0],[0,0,1,0,1,0,0,0,1],[0,1,1,0,0,0,1,0,0],[0,1,1,0,0,0,1,0,0],[0,1,1,0,0,0,1,0,0],[0,1,1,0,0,0,1,0,0],[0,0,1,0,0,0,0,0,0],[0,0,1,0,0,0,0,0,0],[0,0,1,0,0,0,0,0,0],[0,1,1,0,0,0,0,0,0],[0,0,1,0,0,0,0,0,0],[0,1,1,1,0,0,1,0,1],[0,1,1,1,0,0,1,0,0],[0,1,0,0,0,0,0,0,0],[0,1,0,0,0,0,1,0,1],[0,0,1,0,0,0,0,0,0],[0,0,0,0,1,0,0,0,0],[0,0,0,0,1,0,0,0,0],[0,1,0,0,0,0,1,0,0],[0,1,1,0,0,0,1,0,1],[0,0,1,0,0,0,0,0,0],[0,0,1,0,0,0,0,0,0],[0,0,1,0,0,0,0,0,0],[0,0,1,0,0,0,0,0,0],[0,0,1,0,0,0,0,0,0],[0,0,1,0,0,0,0,0,0],[0,0,1,0,0,0,0,0,0],[0,0,1,0,0,0,0,0,0],[0,0,1,0,0,0,0,0,0],[0,0,1,0,0,0,0,0,0],[0,0,1,0,0,0,0,0,0],[0,0,1,0,0,0,0,0,0],[0,1,1,0,0,0,1,0,0],[0,0,1,0,0,0,0,0,0],[0,0,1,0,0,0,0,0,0],[0,0,1,0,0,0,0,0,0],[0,0,1,0,0,0,0,0,0],[0,0,1,0,0,0,0,0,0],[0,0,1,0,0,0,0,0,0],[0,0,1,0,0,0,0,0,0],[0,0,1,0,0,0,0,0,0],[0,0,1,0,0,0,0,0,1],[0,0,1,0,0,0,0,0,0],[0,0,0,0,1,0,0,0,0],[0,0,1,0,0,0,0,0,0],[0,0,0,0,0,0,0,0,1],[0,0,1,0,0,0,0,0,0],[0,0,1,0,0,0,0,0,0],[0,0,1,0,0,0,0,0,0],[0,0,1,0,0,0,0,0,0],[0,0,1,0,0,0,0,0,0],[0,0,1,0,0,0,0,0,0],[0,0,1,0,0,0,0,0,0],[0,0,1,0,1,0,0,0,0],[0,0,1,0,0,0,0,0,0],[0,0,1,0,0,0,0,0,0],[0,0,1,0,0,0,0,0,0],[0,0,1,0,0,0,0,0,0],[0,0,0,0,0,0,0,0,1],[0,0,0,0,0,0,0,0,1],[0,0,1,0,0,0,0,0,0],[0,0,1,0,0,0,0,0,0],[0,0,1,0,1,0,0,0,0],[0,1,0,0,0,0,0,0,0],[0,1,0,0,0,0,0,0,0],[0,0,1,0,0,0,0,0,0]]5483	5484,4635,5485,5486,1057,5487,5488,5489	[[0,0,1,0,0,0,0,0,0],[0,0,1,0,0,0,0,0,0],[0,0,1,0,0,0,0,0,0],[0,1,1,0,0,0,0,0,0],[0,0,1,0,0,0,0,0,0],[0,0,1,0,0,0,0,0,0],[0,1,1,1,1,0,0,0,0],[0,0,1,0,0,0,0,0,0]]5490	507,511,508	[[0,0,0,1,0,0,0,0,0],[0,0,0,1,0,0,0,0,0],[0,0,0,1,0,0,0,0,0]]5491	5492	[[0,0,0,0,1,0,0,0,0]]5493	5494,5495,4827	[[0,0,0,0,1,0,0,0,0],[0,0,0,0,1,0,0,0,0],[0,0,0,0,1,0,0,0,0]]5496	5497,4934,4933	[[0,0,0,0,1,0,0,0,0],[0,0,0,0,1,0,0,0,0],[0,0,0,0,1,0,0,0,0]]5498	5499,5500,2365,5501,3673,4587,2255,4590,3745,4585,5502,5503,3738,2380,2074,5504,5505,4586,4584,3748,5506,5507	[[0,0,1,0,0,0,0,0,0],[0,0,1,0,0,0,0,0,0],[0,1,0,0,0,0,0,0,0],[0,1,1,0,0,0,0,0,0],[0,0,1,0,0,0,0,0,0],[0,1,1,1,1,0,0,0,0],[0,1,1,1,0,0,0,0,0],[0,0,0,0,1,0,0,0,0],[0,0,1,0,0,0,0,0,0],[0,0,1,1,1,0,0,0,0],[0,1,0,0,0,0,0,0,0],[0,0,1,0,1,0,0,0,0],[0,0,1,0,0,0,0,0,0],[0,0,1,0,0,0,0,0,0],[0,0,1,0,0,0,0,0,0],[0,0,1,0,0,0,0,0,0],[0,0,1,0,0,0,0,0,0],[0,0,1,0,1,0,0,0,0],[0,0,1,0,1,0,0,0,0],[0,0,1,0,0,0,0,0,0],[0,0,1,0,0,0,0,0,0],[0,0,1,0,0,0,0,0,0]]5508	5509,5510,4643,2398,4650,5511,5512,5513,5514,5515,5516,5517,5518,5519,5520	[[0,0,0,1,0,0,0,0,0],[0,0,0,1,0,0,0,0,0],[0,0,0,1,0,0,0,0,0],[0,0,0,1,0,0,0,0,0],[0,0,0,1,0,0,0,0,0],[0,0,0,1,0,0,0,0,0],[0,0,0,1,0,0,0,0,0],[0,0,0,1,0,0,0,0,0],[0,0,0,1,0,0,0,0,0],[0,0,0,1,0,0,0,0,0],[0,0,0,1,0,0,0,0,0],[0,0,0,1,0,0,0,0,0],[0,0,0,1,0,0,0,0,0],[0,0,0,1,0,0,0,0,0],[0,0,0,1,0,0,0,0,0]]5521	5522,5523,2612,2611,5524,5525,5526,5527,3338,5528,5529,2624,5530,5531,5532,5533,5534,5535,5536	[[0,0,1,0,0,0,0,0,0],[0,0,1,0,0,0,0,0,0],[0,1,1,0,0,0,0,0,0],[0,0,1,0,0,0,0,0,0],[0,0,1,0,0,0,0,0,0],[0,0,1,0,0,0,0,0,0],[0,0,1,0,0,0,0,0,0],[0,0,1,0,0,0,0,0,0],[0,1,1,0,1,0,0,0,0],[0,1,0,0,0,0,0,0,0],[0,1,1,0,0,0,0,0,0],[0,1,1,0,0,0,0,0,0],[0,1,1,1,0,0,0,0,0],[0,0,1,0,0,0,0,0,0],[0,0,1,0,0,0,0,0,0],[0,0,1,0,0,0,0,0,0],[0,0,1,0,0,0,0,0,0],[0,0,0,1,0,0,0,0,0],[0,0,0,0,1,0,0,0,0]]5537	5538,5539,5540,5541,5542,5543,5544,264,5545,5546,3264,5547,4462,5548,5549,5550,5551,5552,5553,5554,5555,5556,265,3082,5557,5558,5559,5560,5561	[[0,1,0,0,1,0,0,1,0],[0,0,0,0,0,0,0,1,0],[0,0,0,0,0,0,0,1,0],[0,1,0,0,0,0,0,1,0],[0,0,0,0,0,0,0,1,0],[0,0,0,0,0,0,0,1,0],[0,1,0,0,0,0,0,1,0],[0,1,0,0,0,0,0,0,0],[0,0,0,0,0,0,0,1,0],[0,1,0,0,0,0,0,0,0],[0,1,0,0,0,0,0,0,0],[0,0,0,0,0,0,0,1,0],[0,1,0,0,0,0,0,1,0],[0,0,0,0,0,0,0,1,0],[0,1,0,0,0,0,0,1,0],[0,1,0,0,0,0,0,0,0],[0,0,0,0,0,0,0,1,0],[0,1,0,0,0,0,0,1,0],[0,0,0,0,0,0,0,1,0],[0,1,0,0,1,0,0,1,0],[0,1,0,0,0,0,0,1,0],[0,1,0,0,0,0,0,0,0],[0,1,0,0,0,0,0,0,0],[0,0,0,0,0,0,0,1,0],[0,0,0,0,0,0,0,1,0],[0,0,0,0,0,0,0,1,0],[0,0,0,0,0,0,0,1,0],[0,0,0,0,0,0,0,1,0],[0,0,0,0,0,0,0,1,0]]5562	5563,5564,5565,5566,5567,5568,5569,5570,3663,5571,5572,5345,3665,5573,5574,5575,5576,5577,5578,5579,5580,5581,5582,2939,5127	[[0,1,1,0,1,0,0,0,0],[0,1,1,1,1,0,0,0,0],[0,1,0,0,0,0,0,0,0],[0,1,1,0,0,0,0,0,0],[0,1,1,1,0,0,1,0,0],[0,1,1,1,0,0,1,0,0],[0,1,1,1,0,0,1,0,0],[0,1,1,0,0,0,1,0,0],[0,1,0,0,0,0,1,0,0],[0,0,1,1,0,0,0,0,0],[0,0,0,0,0,0,1,0,0],[0,0,1,0,0,0,0,0,0],[0,1,1,1,0,0,1,0,0],[0,0,1,0,0,0,0,0,0],[0,0,1,0,0,0,0,0,0],[0,0,0,1,0,0,0,0,0],[0,0,1,0,0,0,0,0,0],[0,0,1,0,1,0,0,0,0],[0,0,1,0,1,0,0,0,0],[0,0,0,0,1,0,0,0,0],[0,0,1,0,0,0,0,0,0],[0,0,1,0,0,0,0,0,0],[0,0,1,0,0,0,0,0,0],[0,0,1,1,0,0,0,0,0],[0,0,1,0,0,0,0,0,0]]5583	1812,1847,1764,5584,1768,1766,1846,5585,1835,1810,1820,5586,1787,1831	[[0,0,0,0,1,0,0,0,0],[0,0,0,0,1,0,0,0,0],[0,0,0,0,1,0,0,0,0],[0,0,0,0,1,0,0,0,0],[0,0,0,0,1,0,0,0,0],[0,0,0,0,1,0,0,0,0],[0,0,0,0,1,0,0,0,0],[0,0,0,0,1,0,0,0,0],[0,0,0,0,1,0,0,0,0],[0,0,0,0,1,0,0,0,0],[0,0,0,0,1,0,0,0,0],[0,0,0,0,1,0,0,0,0],[0,0,0,0,1,0,0,0,0],[0,0,0,0,1,0,0,0,0]]5587	5588,5589,5590,5591,5592,5593,5594,5595,5596,5597,5598	[[1,1,1,0,1,0,0,0,0],[1,1,1,0,1,0,0,0,0],[1,0,1,0,0,0,0,0,0],[1,0,1,0,0,0,0,0,0],[1,1,1,0,0,0,0,0,0],[0,0,1,0,0,0,0,0,0],[0,0,1,0,0,0,0,0,0],[0,1,0,0,0,0,0,0,0],[0,0,1,0,0,0,0,0,0],[0,0,1,0,0,0,0,0,0],[0,0,1,0,0,0,0,0,0]]5599	5600	[[0,0,0,0,1,0,0,0,0]]5601	5602	[[0,0,0,0,1,0,0,0,0]]5603	5604,5605,5606,5607	[[0,0,0,0,1,0,0,0,0],[0,0,0,0,1,0,0,0,0],[0,0,0,0,1,0,0,0,0],[0,0,0,0,1,0,0,0,0]]5608	2710,14,5609,5610,5611,5612,5613,5614,5615,5616,5617,5618,5619,5620,5621,2408,5622,5623,5624,5625,5626,5627,5628,5629,5630,5631,5632,5633,5634,5635,166,5636,5637,5638,5639,5640,2722,5641,5642,5643,5644,5645,5646,5647,5648,5649,5650,5651,5652,140,3011,5653,5654,5655,5165,5656,5657,137,5658,1670,4790	[[0,0,1,0,0,0,0,0,0],[0,0,1,0,0,0,0,0,0],[0,1,1,0,0,0,1,0,0],[0,1,0,0,0,0,1,0,0],[0,0,1,0,0,0,0,0,0],[0,0,1,0,0,0,0,0,0],[0,0,1,0,0,0,0,0,0],[0,0,1,0,0,0,0,0,0],[0,1,0,1,0,0,1,0,0],[0,0,1,0,0,0,0,0,0],[0,1,0,0,0,0,0,0,0],[0,1,0,0,0,0,1,0,0],[0,0,0,1,0,0,0,0,0],[0,0,0,1,0,0,0,0,0],[0,1,1,0,0,0,0,0,0],[0,1,1,0,0,0,1,0,0],[0,0,0,1,0,0,0,0,0],[0,0,1,0,0,0,0,0,0],[0,0,1,1,0,0,0,0,0],[0,0,1,0,0,0,0,0,0],[0,1,0,0,0,0,1,0,0],[0,1,0,0,1,0,1,0,0],[0,0,1,0,0,0,0,0,0],[0,1,1,0,0,0,1,0,0],[0,0,1,0,0,0,0,0,0],[0,1,1,0,0,0,0,0,0],[0,0,1,0,0,0,0,0,0],[0,0,1,0,0,0,0,0,0],[0,0,1,0,0,0,0,0,0],[0,0,1,0,0,0,0,0,0],[0,0,1,0,0,0,0,0,0],[0,0,1,0,0,0,0,0,0],[0,0,1,0,0,0,0,0,0],[0,0,1,0,0,0,0,0,0],[0,0,1,0,0,0,0,0,0],[0,0,1,0,0,0,0,0,0],[0,1,0,0,0,0,1,0,0],[0,1,1,0,0,0,1,0,0],[0,1,0,0,1,0,1,0,0],[0,0,1,0,0,0,0,0,0],[0,0,1,0,0,0,0,0,0],[0,0,1,0,0,0,0,0,0],[0,0,1,0,0,0,0,0,0],[0,0,1,0,0,0,0,0,0],[0,0,1,0,0,0,0,0,0],[0,0,1,0,0,0,0,0,0],[0,1,1,1,0,0,0,0,0],[0,0,1,0,0,0,0,0,0],[0,0,1,0,0,0,0,0,0],[0,0,1,0,0,0,0,0,0],[0,0,1,0,0,0,0,0,0],[0,1,0,0,0,0,0,0,0],[0,0,1,0,0,0,0,0,0],[0,0,1,0,0,0,0,0,0],[0,0,1,0,0,0,0,0,0],[0,0,1,0,0,0,0,0,0],[0,0,1,0,0,0,0,0,0],[0,0,1,0,0,0,0,0,0],[0,0,1,0,0,0,0,0,0],[0,0,1,0,0,0,0,0,0],[0,0,1,0,0,0,0,0,0]]5659	5660,5661,5662,5663,5664,5665,5666,5667,5668,5669,5670,5671,5672	[[0,1,1,1,0,0,0,0,0],[0,0,1,1,1,0,0,0,0],[0,1,1,1,0,0,0,0,0],[0,0,1,0,0,0,0,0,0],[0,0,1,0,0,0,0,0,0],[0,1,1,0,1,0,0,0,0],[0,0,1,0,0,0,0,0,0],[0,0,1,0,0,0,0,0,0],[0,0,1,0,0,0,0,0,0],[0,1,1,0,0,0,0,0,0],[0,0,1,0,0,0,0,0,0],[0,0,1,0,0,0,0,0,0],[0,0,1,0,0,0,0,0,0]]5673	5674,5675,5676,5677,5678,5679,5680,5681,5682,5683,1543,5684,5685,4633,5686,5687,5688,5689,5690,1904,5691,5692,5363,735,5693,5694,5695	[[1,1,1,1,0,0,1,1,0],[1,1,1,1,0,0,0,0,0],[0,0,0,0,0,0,0,1,0],[0,0,0,1,0,0,0,0,0],[0,0,0,1,0,0,0,0,0],[1,1,0,1,0,0,0,1,0],[1,1,0,1,0,0,1,0,0],[1,0,0,1,0,0,0,0,0],[0,0,0,1,0,0,0,0,0],[1,0,1,1,0,0,0,1,0],[0,1,1,0,0,0,0,0,0],[0,0,0,1,0,0,0,0,0],[1,1,1,1,0,0,1,0,0],[1,0,1,1,0,0,0,0,0],[1,0,0,0,0,0,0,0,0],[0,0,1,1,0,0,0,0,0],[0,0,1,1,0,0,0,0,0],[1,0,0,0,0,0,0,0,0],[1,1,0,0,0,0,1,1,0],[0,1,0,0,0,0,0,0,0],[1,1,0,0,0,0,0,0,0],[1,0,0,0,0,0,0,0,0],[0,1,0,0,0,0,0,0,0],[1,0,0,0,0,0,0,0,0],[1,0,0,1,0,0,0,0,0],[1,0,0,0,0,0,0,0,0],[1,0,0,0,0,0,0,0,0]]5696	5697	[[0,0,0,0,1,0,0,0,0]]5698	5699,4976,3672,5700,5701,4308,5702,5703,5704,5705,3663,5575,5706,5707,5708,5709,5710,5711,5712,5713,5714,5715,5563,5716,5717,1604,5718,5719,5569,3231,2495,2590,5132,5581,3218,5720,5721,4879,5084,5722,3665,5573	[[0,1,1,0,0,0,0,0,0],[0,0,0,0,1,0,0,0,0],[0,1,1,0,0,0,1,0,0],[0,0,1,0,0,0,0,0,0],[0,1,1,0,0,0,0,0,0],[0,0,1,0,0,0,0,0,0],[0,1,1,0,0,0,1,0,0],[0,0,1,0,0,0,0,0,0],[0,0,1,0,0,0,0,0,0],[0,1,1,0,1,0,1,0,0],[0,0,1,0,0,1,0,0,0],[0,1,1,0,1,1,1,0,0],[0,1,1,0,0,0,1,0,0],[0,1,1,0,0,0,1,0,0],[0,1,0,0,0,0,1,0,0],[0,0,1,0,0,0,0,0,0],[0,1,1,0,1,0,1,0,0],[0,0,1,0,0,0,0,0,0],[0,0,0,0,0,1,0,0,0],[0,1,1,0,0,0,0,0,0],[0,1,1,0,0,0,0,0,0],[0,0,1,0,0,0,0,0,0],[0,0,0,0,1,0,0,0,0],[0,0,1,0,0,0,0,0,0],[0,0,1,0,1,0,0,0,0],[0,1,1,0,0,0,0,0,0],[0,0,0,0,1,0,0,0,0],[0,0,1,0,0,0,0,0,0],[0,0,1,0,0,0,0,0,0],[0,0,1,0,0,0,0,0,0],[0,0,1,0,0,0,0,0,0],[0,0,1,0,0,0,0,0,0],[0,0,0,0,1,0,0,0,0],[0,0,1,0,0,0,0,0,0],[0,0,0,0,0,1,0,0,0],[0,0,1,0,0,0,0,0,0],[0,0,0,0,1,0,0,0,0],[0,1,0,0,0,0,0,0,0],[0,1,0,0,0,0,0,0,0],[0,1,0,0,0,0,0,0,0],[0,0,1,0,0,0,0,0,0],[0,0,0,0,0,1,0,0,0]]5723	5724	[[0,0,0,0,1,0,0,0,0]]5725	5726,5727	[[0,0,0,0,1,0,0,0,0],[0,0,0,0,1,0,0,0,0]]5728	5729,2939,5730,5567,5731,746,5732,5733,5734,5735,5736,5737,2480,5738,5739,5740,5741,5742,5743,5744,2887,5745,5746,5747,2541,5748,5749	[[0,0,1,0,0,0,0,0,0],[0,0,1,0,0,0,0,0,0],[0,0,1,0,0,0,0,0,0],[0,0,1,0,0,0,0,0,0],[0,0,1,1,1,0,0,0,0],[0,0,1,0,0,0,0,0,0],[0,0,1,0,0,0,0,0,0],[0,0,0,0,1,0,0,0,0],[0,0,1,0,0,0,0,0,0],[0,0,1,0,0,0,0,0,0],[0,0,1,1,1,0,0,0,0],[0,0,1,0,0,0,0,0,0],[0,0,1,1,0,0,0,0,0],[0,0,1,0,0,0,0,0,0],[0,0,1,0,0,0,0,0,0],[0,0,1,0,0,0,0,0,0],[0,0,1,0,0,0,0,0,0],[0,0,1,0,0,0,0,0,0],[0,0,1,0,0,0,0,0,0],[0,0,1,0,0,0,0,0,0],[0,0,1,0,0,0,0,0,0],[0,0,1,0,0,0,0,0,0],[0,0,1,0,0,0,0,0,0],[0,0,1,0,0,0,0,0,0],[0,0,1,0,0,0,0,0,0],[0,0,1,0,0,0,0,0,0],[0,0,1,0,0,0,0,0,0]]5750	5751,5752,5753,5754,5755,5756,5757,5758,5759,5760	[[0,0,0,1,1,1,0,0,0],[0,0,0,0,1,0,0,0,0],[0,0,0,0,0,1,0,0,0],[0,0,0,0,0,1,0,0,0],[0,0,0,0,0,1,0,0,0],[0,0,0,0,0,1,0,0,0],[0,0,0,1,1,0,0,0,0],[0,0,0,0,0,1,0,0,0],[0,0,0,0,1,0,0,0,0],[0,0,0,1,0,0,0,0,0]]5761	5762,5763,814,5764,5765,5766,5112,5767	[[0,0,0,0,1,0,0,0,0],[0,0,0,0,1,0,0,0,0],[0,0,0,0,1,0,0,0,0],[0,0,0,0,1,0,0,0,0],[0,0,0,0,1,0,0,0,0],[0,0,0,0,1,0,0,0,0],[0,0,0,0,1,0,0,0,0],[0,0,0,0,1,0,0,0,0]]5768	5769	[[0,0,0,0,1,0,0,0,0]]5770	5771	[[0,0,0,0,1,0,0,0,0]]5772	5773,5774,5775,5776,5777,5778,5779,5780	[[0,1,0,0,0,0,0,0,0],[0,1,0,0,0,0,0,0,0],[0,1,0,0,0,0,0,0,0],[0,1,0,0,0,0,0,0,0],[0,1,0,0,0,0,0,0,0],[0,1,0,0,0,0,0,0,0],[0,0,0,0,1,0,0,0,0],[0,1,0,0,0,0,0,0,0]]5781	5165,5782,5783,2702,5784,5785,5786	[[0,0,1,0,0,0,0,0,0],[0,0,1,0,0,0,0,0,0],[0,0,1,0,0,0,0,0,0],[0,0,1,0,0,0,0,0,0],[0,0,1,0,0,0,0,0,0],[0,0,1,0,0,0,0,0,0],[0,0,1,0,0,0,0,0,0]]5787	5788	[[0,0,0,0,1,0,0,0,0]]5789	5790	[[0,0,0,0,1,0,0,0,0]]5791	5792,5793,5794,5795,5796,5797,5798,5799,3894,5800,5801,5802,5803,5804,2511,5023,5805,5806,5807,5808,5809,5810,5811,5812,5813,5814,5815,5816,3987,2914,5817,5818,1797,5819,5820,5821,5822,1930,5823,5824,5825,5826,5827,5828,2944,5829,5830,5831,5832,5833,5834,5835,5836,5837,2550,5838,5839,5840,5841,5842,5843,1356,5844,1140,5845,5846,5847,5848,5849,5850,5851,5852,5853,5854,2234,5855,5856,5857	[[0,0,1,1,0,0,0,0,0],[1,1,1,1,0,0,0,0,1],[1,1,1,1,0,1,1,0,1],[1,1,1,1,0,0,1,0,1],[0,1,1,1,0,0,1,0,1],[1,1,1,1,1,0,1,0,0],[1,1,1,1,0,0,1,0,0],[0,0,1,1,0,0,0,0,1],[1,1,0,1,0,1,0,0,1],[1,1,1,1,0,0,0,0,1],[0,1,0,0,0,0,0,0,0],[0,1,1,1,0,0,1,0,1],[0,1,1,1,0,0,1,0,0],[0,1,0,1,0,0,1,0,0],[0,0,0,1,0,0,0,0,0],[0,0,1,1,0,0,0,0,0],[0,0,1,0,0,0,0,0,0],[0,0,1,1,0,0,0,0,0],[1,1,1,1,0,1,0,0,1],[0,0,0,1,0,0,0,0,0],[0,0,1,0,0,0,0,0,0],[0,1,0,0,0,0,1,0,0],[0,0,1,0,0,0,0,0,0],[0,1,1,0,0,0,1,0,0],[1,1,1,1,0,0,1,0,0],[1,0,1,1,0,0,0,0,1],[1,0,0,0,0,0,0,0,0],[1,0,0,0,0,0,0,0,0],[1,0,0,0,0,0,0,0,0],[1,0,0,1,0,0,0,0,1],[1,0,0,0,0,0,0,0,0],[1,0,0,0,0,0,0,0,0],[1,0,0,0,0,0,0,0,0],[1,0,0,0,0,0,0,0,0],[1,1,0,1,1,0,0,0,0],[1,0,1,1,0,1,0,0,1],[0,0,1,0,0,0,0,0,0],[0,0,0,1,0,0,0,0,0],[1,0,0,0,0,0,0,0,0],[1,0,0,0,0,0,0,0,0],[1,0,0,0,0,0,0,0,0],[1,0,0,1,0,0,0,0,0],[1,0,0,0,0,0,0,0,0],[0,1,0,1,0,0,1,0,1],[0,0,0,1,0,0,0,0,0],[0,1,1,1,0,0,0,0,1],[0,1,1,0,0,0,1,0,0],[0,1,0,0,0,0,1,0,0],[0,1,0,0,0,0,1,0,0],[0,1,1,1,0,0,1,0,1],[0,1,1,0,0,0,1,0,0],[1,0,0,0,0,0,0,0,0],[0,0,1,0,0,0,0,0,0],[0,0,1,0,0,0,0,0,0],[0,0,1,1,0,0,0,0,0],[0,0,0,1,0,0,0,0,0],[0,0,0,1,0,0,0,0,0],[0,0,1,0,0,0,0,0,0],[0,0,1,0,0,0,0,0,0],[0,0,0,1,0,0,0,0,1],[0,0,1,0,0,0,0,0,0],[0,0,0,1,0,0,0,0,0],[0,0,1,1,0,0,0,0,0],[0,0,1,0,0,0,0,0,1],[0,0,1,0,0,0,0,0,0],[0,0,1,0,0,0,0,0,0],[0,0,0,1,0,0,0,0,0],[0,0,1,1,0,0,0,0,0],[0,0,1,1,0,0,0,0,0],[0,0,0,1,0,0,0,0,0],[0,0,1,0,0,0,0,0,0],[0,0,1,0,0,0,0,0,0],[0,0,1,0,0,0,0,0,0],[0,0,1,0,0,0,0,0,0],[0,0,1,1,0,0,0,0,1],[0,0,0,1,0,0,0,0,1],[0,0,0,1,0,0,0,0,0],[0,0,1,0,0,0,0,0,0]]5858	5859,5860	[[0,0,1,0,0,0,0,0,0],[0,0,1,0,0,0,0,0,0]]5861	5862,5863,5864,5865,5866,1187,5867,5868,5869,5870,5871,5872,5873,5874,5875,2412,5876,5877,5878,5879,5880,5881,5882,5883,5884,5885,5886,5887,5888,5889,5890,5891,5892,5893,5894,5895,5896,5897,5898,5899,5900,5901,5902,5903,5904,5905,5906,5907,5908,2576,5909,5910,5911,5912,5913	[[0,0,1,0,0,0,0,0,0],[0,0,1,0,0,0,0,0,0],[0,0,1,0,0,0,0,0,0],[0,0,0,1,0,0,0,0,0],[0,0,1,0,0,0,0,0,0],[0,1,0,0,0,0,1,0,0],[0,0,1,0,0,0,0,0,0],[0,1,1,0,0,0,0,0,0],[0,1,1,0,0,0,0,0,0],[0,0,1,0,0,0,0,0,0],[0,0,1,0,0,0,0,0,0],[0,0,1,0,0,0,0,0,0],[0,1,1,1,0,0,0,0,0],[0,0,1,0,0,0,0,0,0],[0,0,1,1,0,0,0,0,0],[0,0,1,0,0,0,0,0,0],[0,0,1,0,0,0,0,0,0],[0,0,1,0,0,0,0,0,0],[0,0,1,0,0,0,0,0,0],[0,0,1,0,0,0,0,0,0],[0,0,1,0,0,0,0,0,0],[0,0,1,0,0,0,0,0,0],[0,0,1,0,0,0,0,0,0],[0,0,1,0,0,0,0,0,0],[0,0,1,0,0,0,0,0,0],[0,0,1,0,0,0,0,0,0],[0,1,1,0,0,0,1,0,0],[0,0,1,1,0,0,0,0,0],[0,1,1,0,0,0,0,0,0],[0,1,1,0,0,0,1,0,0],[0,0,1,0,0,0,0,0,0],[0,0,1,0,0,0,0,0,0],[0,1,1,0,0,0,0,0,0],[0,0,1,0,0,0,0,0,0],[0,0,0,1,0,0,0,0,0],[0,1,0,0,0,0,1,0,0],[0,1,1,0,0,0,1,0,0],[0,1,0,0,0,0,0,0,0],[0,1,0,0,0,0,0,0,0],[0,1,1,0,0,0,0,0,0],[0,1,1,1,0,0,0,0,0],[0,1,0,0,0,0,1,0,0],[0,1,0,0,0,0,0,0,0],[0,1,0,0,0,0,0,0,0],[0,1,0,0,0,0,0,0,0],[0,0,1,0,0,0,0,0,0],[0,0,1,1,0,0,1,0,0],[0,0,1,0,0,0,0,0,0],[0,0,1,0,0,0,0,0,0],[0,0,1,0,0,0,0,0,0],[0,0,1,0,0,0,0,0,0],[0,0,1,0,0,0,0,0,0],[0,0,1,0,0,0,0,0,0],[0,0,1,0,0,0,0,0,0],[0,0,1,0,0,0,0,0,0]]5914	5915,5627	[[0,0,0,0,1,0,0,0,0],[0,0,0,0,1,0,0,0,0]]5916	5917,5918,5919,5920,5921,5922,5923,5924,5925	[[0,0,0,1,0,0,0,0,0],[0,0,0,1,0,0,0,0,0],[0,0,0,1,0,0,0,0,0],[0,0,0,1,0,0,0,0,0],[0,0,0,1,0,0,0,0,0],[0,0,0,1,0,0,0,0,0],[0,0,0,1,0,0,0,0,0],[0,0,0,1,0,0,0,0,0],[0,0,0,1,0,0,0,0,0]]5926	5927,5928,1554,5929,5930,5931,2131,5932,5933,5934,5935,5936,5937,5938,3908,5939,5940,2827,5941,5942,5529,5943,5944,3234,5945,5946	[[0,0,1,0,0,0,0,0,0],[0,0,1,1,1,0,0,0,0],[0,0,1,0,0,0,0,0,0],[0,0,1,0,0,0,0,0,0],[0,0,1,0,1,0,0,0,0],[0,0,1,1,1,0,0,0,0],[0,0,1,0,0,0,0,0,0],[0,0,1,0,0,0,0,0,0],[0,0,1,0,0,0,0,0,0],[0,0,1,0,0,0,0,0,0],[0,0,1,0,0,0,0,0,0],[0,0,1,0,0,0,0,0,0],[0,0,1,0,0,0,0,0,0],[0,0,1,0,0,0,0,0,0],[0,0,1,0,0,0,0,0,0],[0,0,1,0,0,0,0,0,0],[0,0,1,0,0,0,0,0,0],[0,0,1,0,0,0,0,0,0],[0,0,1,0,0,0,0,0,0],[0,0,1,0,0,0,0,0,0],[0,0,1,0,0,0,0,0,0],[0,0,1,0,0,0,0,0,0],[0,0,0,1,0,0,0,0,0],[0,0,1,0,0,0,0,0,0],[0,0,1,0,0,0,0,0,0],[0,0,1,0,0,0,0,0,0]]5947	2035,5948	[[0,0,0,0,1,0,0,0,0],[0,0,0,0,1,0,0,0,0]]5949	5950	[[0,0,0,0,1,0,0,0,0]]5951	5952,5953,5954	[[0,0,0,0,1,0,0,0,0],[0,0,0,0,1,0,0,0,0],[0,0,0,0,1,0,0,0,0]]5955	5956,5957,5958	[[0,0,0,0,1,0,0,0,0],[0,0,0,0,1,0,0,0,0],[0,0,0,0,1,0,0,0,0]]5959	5960	[[0,0,0,0,1,0,0,0,0]]5961	5962	[[0,0,0,0,1,0,0,0,0]]5963	5964,5547,5551	[[0,0,0,1,0,0,0,0,0],[0,0,0,1,0,0,0,0,0],[0,0,0,1,0,0,0,0,0]]5965	5966,5967,5968,5969,5970	[[0,0,0,0,1,0,0,0,0],[0,0,0,0,1,0,0,0,0],[0,0,0,0,1,0,0,0,0],[0,0,0,0,1,0,0,0,0],[0,0,0,0,1,0,0,0,0]]5971	1713,5972,5973,4089,5974,4143,3365,1280,4123,5975,4508,4079,3387,5976,5977,1441,3859,5978,4190,4099,4109,5979,5980,5981,5982,5983,5984,4145,4076,4306,4182,4183,3358,5985,4144,5986,5987,5988,5989,1625,5990,1030,5991,5992,5993,4098,4193,4137,4194,5994,4135,4107,5995,5996,5997,1284,4105,4077,4104,4103,1626,4102,5998,125,5999,667,4133,4084,3354,4108,6000,4179,4196,6001,6002,6003,4185,4669,6004,6005,6006,1571,6007,3388,6008,6009,4181,4187,6010,4049,4168,6011,660,1031,4176,2651,640,4095,6012,6013,3870,6014,4174,6015,6016,6017,4093,4091,6018,4131,1993,4150,6019,4121,4151	[[0,1,0,1,0,0,1,0,0],[0,0,0,1,0,0,0,0,1],[0,0,1,0,0,0,0,0,0],[0,1,1,1,0,0,1,1,0],[0,0,1,0,0,0,0,0,0],[0,0,1,1,0,0,0,0,0],[0,0,1,1,0,0,0,0,0],[0,0,1,1,0,0,0,1,0],[0,0,1,0,0,0,0,0,0],[0,0,0,1,0,0,0,1,0],[1,0,0,0,0,0,0,0,0],[0,0,1,0,0,0,0,1,0],[1,1,1,1,0,0,1,1,1],[0,1,0,0,0,0,1,0,0],[0,0,1,0,0,0,0,0,0],[0,0,0,1,0,0,0,0,0],[1,0,0,0,0,0,0,0,0],[1,0,0,1,0,0,0,0,0],[0,0,1,1,0,0,0,0,0],[0,1,1,1,0,0,1,0,0],[1,1,1,1,0,0,1,1,1],[1,0,0,0,0,0,0,0,0],[1,0,0,0,0,0,0,0,0],[1,0,0,0,0,0,0,0,0],[1,0,0,0,0,0,0,0,0],[1,0,0,0,0,0,0,0,0],[1,0,0,0,0,0,0,0,0],[1,0,1,1,0,0,0,1,0],[0,0,1,1,0,0,0,0,0],[0,0,0,1,0,0,0,1,0],[0,0,0,1,0,0,0,1,0],[0,0,1,1,0,0,0,0,0],[0,0,0,1,0,0,0,0,0],[0,0,1,0,0,0,0,0,0],[0,0,1,0,0,0,0,0,0],[1,1,1,0,0,0,0,0,0],[0,0,1,0,0,0,0,0,0],[0,1,0,0,0,0,1,0,0],[0,0,0,1,0,0,0,0,0],[1,1,1,1,0,0,1,0,0],[0,0,0,0,0,0,0,1,0],[1,1,1,1,0,0,1,1,0],[0,0,1,0,0,0,0,0,0],[0,0,0,1,0,0,0,1,0],[0,0,1,0,0,0,0,0,0],[1,0,1,1,0,0,0,0,1],[0,0,1,0,0,0,0,0,0],[1,1,1,1,0,0,1,1,1],[0,0,1,1,0,0,0,0,0],[0,0,1,0,0,0,0,0,0],[1,1,1,1,0,0,1,0,0],[0,0,1,1,0,0,0,0,0],[1,0,1,1,0,0,0,0,0],[1,0,0,0,0,0,0,0,0],[0,0,1,0,0,0,0,0,0],[0,0,1,1,0,0,0,1,0],[0,0,1,1,0,0,0,0,0],[0,0,1,0,0,0,0,0,0],[1,0,0,1,0,0,0,0,0],[0,0,1,1,0,0,0,0,0],[0,0,1,1,0,0,0,1,0],[1,1,1,1,0,0,1,1,1],[0,0,1,1,0,0,0,1,0],[0,0,1,0,0,0,0,0,0],[0,0,0,1,0,0,0,1,0],[0,0,1,0,0,0,0,0,0],[1,1,1,1,0,0,1,0,0],[0,0,0,1,0,0,0,1,1],[1,0,1,1,0,0,0,0,0],[1,0,1,1,0,0,0,0,0],[0,0,0,1,0,0,0,0,0],[1,0,1,1,0,0,0,1,1],[1,0,1,1,0,0,0,1,0],[0,0,1,0,0,0,0,0,0],[0,1,0,0,0,0,0,0,0],[0,1,0,0,0,0,0,0,0],[0,0,1,1,0,0,0,0,1],[1,0,0,0,0,0,0,0,0],[1,0,0,0,0,0,0,0,0],[1,0,0,0,0,0,0,0,0],[1,0,0,0,0,0,0,0,0],[0,1,1,1,0,0,1,0,0],[0,1,0,0,0,0,0,0,0],[1,1,1,1,0,0,0,1,0],[0,0,1,0,0,0,0,0,0],[0,1,0,0,0,0,1,0,0],[1,1,1,0,0,0,1,0,1],[0,0,1,1,0,0,0,0,0],[0,1,0,0,0,0,0,0,0],[1,1,0,0,0,0,1,0,0],[0,1,1,1,0,0,1,0,0],[0,1,0,1,0,0,1,1,0],[0,0,0,0,0,0,0,1,0],[0,0,0,0,0,0,0,1,0],[1,1,1,1,0,0,1,0,0],[1,0,0,0,0,0,0,0,0],[1,0,0,0,0,0,0,0,0],[0,0,1,0,0,0,0,0,1],[0,0,0,1,0,0,0,0,0],[0,1,0,0,0,0,1,0,0],[1,0,0,0,0,0,0,0,0],[1,0,0,0,0,0,0,0,0],[0,0,1,0,0,0,0,0,0],[0,0,0,1,1,0,0,0,0],[1,0,0,0,0,0,0,0,0],[1,0,1,0,0,0,0,0,0],[0,0,1,0,0,0,0,0,0],[0,0,1,0,0,0,0,0,0],[0,0,0,0,0,0,1,0,0],[0,1,1,1,0,0,1,0,0],[0,0,1,0,0,0,0,0,0],[0,0,1,1,0,0,0,0,0],[0,0,0,1,0,0,0,0,0],[0,0,1,0,0,0,0,0,0],[0,0,1,0,0,0,0,0,0]]6020	5255,6021,6022,5275,6023,5266,6024,6025,5258,6026	[[0,0,0,1,0,0,0,0,0],[0,0,0,1,0,0,0,0,0],[0,0,0,1,0,0,0,0,0],[0,0,0,1,0,0,0,0,0],[0,0,0,1,0,0,0,0,0],[0,0,0,1,0,0,0,0,0],[0,0,0,1,0,0,0,0,0],[0,0,0,1,0,0,0,0,0],[0,0,0,1,0,0,0,0,0],[0,0,0,1,0,0,0,0,0]]6027	6028	[[0,0,0,0,1,0,0,0,0]]6029	6030	[[0,0,0,0,1,0,0,0,0]]6031	6019,5972,4150,4185,3387,4076,4183,1625,3365,6011,4196,5992,4143,4133,3358,5998,4168,4182,4131,4137,1571,4306,3388,4089,3354,1030,4098,4104,5975,4187,4108,5999,4105,4176,5989,4084,4179,4107,4102,5995,4103,6000,1280,1441,4190,4145,1713,4109,4135,5978,4194,4099,1284,6015,1626,6012	[[0,0,0,1,0,0,0,0,0],[0,0,0,1,0,0,0,0,0],[0,0,0,1,0,0,0,0,0],[0,0,0,1,0,0,0,0,0],[0,0,0,1,0,0,0,0,0],[0,0,0,1,0,0,0,0,0],[0,0,0,1,0,0,0,0,0],[0,0,0,1,0,0,0,0,0],[0,0,0,1,0,0,0,0,0],[0,0,0,1,0,0,0,0,0],[0,0,0,1,0,0,0,0,0],[0,0,0,1,0,0,0,0,0],[0,0,0,1,0,0,0,0,0],[0,0,0,1,0,0,0,0,0],[0,0,0,1,0,0,0,0,0],[0,0,0,1,0,0,0,0,0],[0,0,0,1,0,0,0,0,0],[0,0,0,1,0,0,0,0,0],[0,0,0,1,0,0,0,0,0],[0,0,0,1,0,0,0,0,0],[0,0,0,1,0,0,0,0,0],[0,0,0,1,0,0,0,0,0],[0,0,0,1,0,0,0,0,0],[0,0,0,1,0,0,0,0,0],[0,0,0,1,0,0,0,0,0],[0,0,0,1,0,0,0,0,0],[0,0,0,1,0,0,0,0,0],[0,0,0,1,0,0,0,0,0],[0,0,0,1,0,0,0,0,0],[0,0,0,1,0,0,0,0,0],[0,0,0,1,0,0,0,0,0],[0,0,0,1,0,0,0,0,0],[0,0,0,1,0,0,0,0,0],[0,0,0,1,0,0,0,0,0],[0,0,0,1,0,0,0,0,0],[0,0,0,1,0,0,0,0,0],[0,0,0,1,0,0,0,0,0],[0,0,0,1,0,0,0,0,0],[0,0,0,1,0,0,0,0,0],[0,0,0,1,0,0,0,0,0],[0,0,0,1,0,0,0,0,0],[0,0,0,1,0,0,0,0,0],[0,0,0,1,0,0,0,0,0],[0,0,0,1,0,0,0,0,0],[0,0,0,1,0,0,0,0,0],[0,0,0,1,0,0,0,0,0],[0,0,0,1,0,0,0,0,0],[0,0,0,1,0,0,0,0,0],[0,0,0,1,0,0,0,0,0],[0,0,0,1,0,0,0,0,0],[0,0,0,1,0,0,0,0,0],[0,0,0,1,0,0,0,0,0],[0,0,0,1,0,0,0,0,0],[0,0,0,1,0,0,0,0,0],[0,0,0,1,0,0,0,0,0],[0,0,0,1,0,0,0,0,0]]6032	6033,6034,6035,6036,6037	[[0,0,0,1,0,0,0,0,0],[0,0,0,1,0,0,0,0,0],[0,0,0,1,0,0,0,0,0],[0,0,0,1,0,0,0,0,0],[0,0,0,1,0,0,0,0,0]]6038	6039,6040,6041,6042,6043	[[0,0,0,0,1,0,0,0,0],[0,0,0,0,1,0,0,0,0],[0,0,0,0,1,0,0,0,0],[0,0,0,0,1,0,0,0,0],[0,0,0,0,1,0,0,0,0]]6044	6045,6046	[[0,0,0,0,1,0,0,0,0],[0,0,0,0,1,0,0,0,0]]6047	6048,6049	[[0,0,0,0,1,0,0,0,0],[0,0,0,0,1,0,0,0,0]]6050	6024,5258,3446,6023,6026,6022,5255,6025,5266,6021,5275	[[0,0,0,1,0,0,0,0,0],[0,0,0,1,0,0,0,0,0],[0,0,0,0,1,0,0,0,0],[0,0,0,1,0,0,0,0,0],[0,0,0,1,0,0,0,0,0],[0,0,0,1,0,0,0,0,0],[0,0,0,1,0,0,0,0,0],[0,0,0,1,0,0,0,0,0],[0,0,0,1,0,0,0,0,0],[0,0,0,1,0,0,0,0,0],[0,0,0,1,0,0,0,0,0]]6051	6052	[[0,0,0,0,1,0,0,0,0]]6053	6054,2393,6055,6056,6057,6058,6059,6060,6061,5945,6062,6063,6064,6065,6066,6067,6068,4433,2367,6069,6070,6071,6072,6073,6074,6075,6076,6077,6078,6079,6080,6081,6082,6083,6084,6085,6086,6087,6088,6089,6090,6091,6092,6093,6094,6095,6096,6097,6098	[[0,1,1,1,0,0,0,0,1],[0,0,1,0,0,0,0,0,0],[0,0,1,0,0,0,0,1,1],[0,0,0,0,0,0,0,1,0],[0,0,1,0,0,0,0,0,0],[0,1,1,1,0,0,0,1,0],[0,0,1,0,0,0,0,0,0],[0,0,1,0,0,0,0,0,0],[0,0,1,0,0,0,0,0,0],[0,1,0,0,0,0,0,1,0],[0,0,0,0,0,0,0,0,1],[0,1,1,0,0,0,0,1,1],[0,1,1,1,0,0,0,1,1],[0,0,1,0,0,0,0,0,1],[0,0,1,0,0,0,0,0,0],[0,0,0,0,0,0,0,1,0],[0,0,0,0,0,0,0,0,1],[0,0,0,0,0,0,0,0,1],[0,0,1,0,0,0,0,0,0],[0,0,1,0,0,0,0,0,0],[0,0,0,0,0,0,0,0,1],[0,0,1,0,0,0,0,0,1],[0,0,0,0,0,0,0,0,1],[0,0,1,0,0,0,0,0,0],[0,0,0,0,0,0,0,1,0],[0,0,1,0,0,0,0,0,0],[0,0,1,0,0,0,0,0,1],[0,0,0,0,0,0,0,0,1],[0,0,0,0,0,0,0,1,0],[0,0,1,0,0,0,0,0,0],[0,0,0,0,0,0,0,1,0],[0,1,1,1,1,0,0,0,0],[0,0,1,0,0,0,0,1,0],[0,1,1,0,0,0,0,0,0],[0,1,1,0,0,0,0,1,0],[0,1,0,0,0,0,0,1,0],[0,0,1,0,0,0,0,0,0],[0,0,1,0,0,0,0,0,0],[0,0,0,0,0,0,0,1,0],[0,0,1,0,0,0,0,1,0],[0,0,0,0,1,0,0,0,0],[0,0,0,0,0,0,0,1,0],[0,1,1,0,0,0,0,1,0],[0,0,0,0,0,0,0,1,0],[0,1,0,0,0,0,0,0,0],[0,1,0,0,0,0,0,0,0],[0,1,0,0,0,0,0,0,0],[0,1,1,0,0,0,0,0,0],[0,0,1,0,0,0,0,0,0]]6099	6100,6101,6102	[[0,0,0,0,1,0,0,0,0],[0,0,0,0,1,0,0,0,0],[0,0,0,0,1,0,0,0,0]]6103	6104	[[0,0,0,0,1,0,0,0,0]]6105	6106,6107	[[0,0,0,0,1,0,0,0,0],[0,0,0,0,1,0,0,0,0]]6108	5629,6109,6110,1670,6111,6112,367,408,6113,6114,6115,6116,6117,6118,6119,6120,6121,5620,6122,6123,5617,6124,414,6125,6126,6127,5650,2408,2575,6128,6129,6130,6131,6132,3462,6133,170,6134,5631,6135,720,6136,6137,6138,6139,6140,410,6141,3066,6142,5624,6143,6144,5636,6145,6146,6147,6148,6149,6150,6151,6152,6153	[[0,1,1,1,0,0,0,0,0],[0,1,0,0,0,0,0,0,0],[0,1,0,0,0,0,0,0,0],[0,1,1,0,0,0,0,0,0],[0,1,0,0,0,0,0,0,0],[0,0,1,0,0,0,0,0,0],[0,1,1,0,0,0,0,0,0],[0,0,1,0,0,0,0,0,0],[0,1,0,0,0,0,0,0,0],[0,0,1,0,0,0,0,0,0],[0,0,1,0,0,0,0,0,0],[0,1,1,0,0,0,0,0,0],[0,0,1,0,0,0,0,0,0],[0,1,1,0,0,0,0,0,0],[0,0,1,0,0,0,0,0,0],[0,0,1,0,0,0,0,0,0],[0,1,1,0,0,0,0,0,0],[0,0,1,0,0,0,0,0,0],[0,1,0,0,1,0,0,0,0],[0,0,1,0,0,0,0,0,0],[0,1,1,1,0,0,0,0,0],[0,0,1,0,0,0,0,0,0],[0,0,1,0,0,0,0,0,0],[0,0,1,0,0,0,0,0,0],[0,0,1,0,0,0,0,0,0],[0,0,1,0,0,0,0,0,0],[0,0,1,0,0,0,0,0,0],[0,0,1,0,0,0,0,0,0],[0,0,1,0,0,0,0,0,0],[0,0,1,0,0,0,0,0,0],[0,0,1,0,0,0,0,0,0],[0,1,0,0,0,0,0,0,0],[0,1,0,0,0,0,0,0,0],[0,1,1,0,0,0,0,0,0],[0,1,1,0,0,0,0,0,0],[0,1,1,1,1,0,0,0,0],[0,0,1,0,0,0,0,0,0],[0,0,1,0,0,0,0,0,0],[0,0,1,0,0,0,0,0,0],[0,0,1,0,0,0,0,0,0],[0,0,1,0,0,0,0,0,0],[0,0,1,0,0,0,0,0,0],[0,0,1,0,0,0,0,0,0],[0,0,1,0,0,0,0,0,0],[0,0,1,0,0,0,0,0,0],[0,0,1,0,0,0,0,0,0],[0,0,1,0,0,0,0,0,0],[0,0,1,0,0,0,0,0,0],[0,0,1,0,0,0,0,0,0],[0,0,1,0,0,0,0,0,0],[0,1,1,0,0,0,0,0,0],[0,0,1,0,0,0,0,0,0],[0,1,1,0,0,0,0,0,0],[0,1,1,0,0,0,0,0,0],[0,0,1,0,0,0,0,0,0],[0,1,0,0,0,0,0,0,0],[0,1,0,0,0,0,0,0,0],[0,0,1,0,0,0,0,0,0],[0,0,1,0,0,0,0,0,0],[0,0,1,0,0,0,0,0,0],[0,0,1,0,0,0,0,0,0],[0,1,0,0,0,0,0,0,0],[0,1,0,0,0,0,0,0,0]]6154	4467,6155,337,6156,6157,6158,4454	[[0,0,0,1,0,0,0,0,0],[0,0,0,1,0,0,0,0,0],[0,0,0,1,0,0,0,0,0],[0,0,0,1,0,0,0,0,0],[0,0,0,1,0,0,0,0,0],[0,0,0,1,0,0,0,0,0],[0,0,0,1,0,0,0,0,0]]6159	3129,3109,3159,6160,6161,6162,815,6163,4562,3156,6164,1143,3107,6165,4391,794,3119,808,6166,6167,2432,3137,6168,6169,3116,3113,3115,6170,6171,6172,6173,6174,6175,6176,6177,6178,3123,2501,6179,4236,3093,494,6180,6181,78,6182,6183,6184	[[0,1,1,0,0,0,1,1,0],[0,1,1,0,0,0,1,1,0],[0,1,0,0,0,0,1,0,0],[0,0,0,0,0,0,0,1,0],[0,0,0,0,0,0,0,1,0],[0,0,1,0,0,0,0,0,0],[0,1,1,0,0,0,1,1,0],[0,1,0,0,0,0,1,0,0],[0,0,0,0,1,0,0,0,0],[0,0,1,0,0,0,0,0,0],[0,0,1,1,0,0,0,0,0],[0,1,1,0,0,0,1,1,0],[0,1,1,0,0,0,1,1,0],[0,1,1,0,1,0,1,1,0],[0,1,1,0,0,0,1,0,0],[0,1,1,0,0,0,1,0,0],[0,1,1,0,0,0,1,1,0],[0,1,1,0,0,0,1,1,0],[0,0,1,0,0,0,0,0,0],[0,1,0,0,0,0,1,0,0],[0,1,1,0,0,0,1,1,0],[0,0,1,0,0,0,0,1,0],[0,0,1,0,0,0,0,0,0],[0,0,1,0,0,0,0,0,0],[0,0,1,0,0,0,0,0,0],[0,0,1,0,0,0,0,1,0],[0,0,1,0,0,0,0,0,0],[0,0,1,0,0,0,0,0,0],[0,0,1,0,0,0,0,0,0],[0,0,1,0,0,0,0,0,0],[0,1,0,0,1,0,1,0,0],[0,0,0,1,0,0,0,0,0],[0,0,1,0,0,0,0,0,0],[0,0,1,0,0,0,0,0,0],[0,0,0,0,0,0,0,1,0],[0,0,1,0,0,0,0,0,0],[0,0,1,0,0,0,0,0,0],[0,0,1,0,0,0,0,0,0],[0,0,1,0,0,0,0,0,0],[0,0,0,1,0,0,0,0,0],[0,1,1,0,0,0,1,1,0],[0,0,1,0,0,0,0,0,0],[0,0,0,0,0,0,0,1,0],[0,0,1,0,0,0,0,1,0],[0,0,1,0,0,0,0,0,0],[0,1,1,0,0,0,0,0,0],[0,1,0,0,0,0,0,0,0],[0,1,0,0,0,0,0,0,0]]6185	6186,6187,6188,6189,6190,6191,6192,6193,6194,6195,6196,6197,6198	[[0,1,1,0,0,0,1,0,0],[0,1,1,1,1,0,1,0,0],[0,0,1,0,0,0,1,0,0],[0,1,0,0,0,0,1,0,0],[0,0,1,0,0,0,1,0,0],[0,1,1,1,0,0,0,0,0],[0,0,1,0,0,0,0,0,0],[0,0,1,0,0,0,0,0,0],[0,0,1,0,0,0,0,0,0],[0,0,1,0,0,0,0,0,0],[0,0,1,0,0,0,0,0,0],[0,1,1,0,0,0,1,0,0],[0,0,1,0,0,0,0,0,0]]6199	6200	[[0,0,0,0,1,0,0,0,0]]6201	4440	[[0,0,0,0,1,0,0,0,0]]6202	488,6203,6204,6205,6206,6207,6208,6209,6210	[[0,0,0,1,0,0,0,0,0],[0,0,1,1,1,0,0,0,0],[0,0,0,1,1,0,0,0,0],[0,0,0,1,0,0,0,0,0],[0,0,0,1,0,0,0,0,0],[0,0,1,1,1,0,0,0,0],[0,0,0,1,1,0,0,0,0],[0,0,1,1,0,0,0,0,0],[0,0,1,1,0,0,0,0,0]]6211	6212	[[0,0,0,0,1,0,0,0,0]]6213	6157,4492,6214,6215,6216,4454,6217,6218,337,6155,4502,6219,4476,4473,6220,6221,6222,4494,2122,6158,6156,6223,6224,6225,6226,2635,6227,336,3558,6228,4466,6229,6230,6231,6232,6233,6234,6235,6236,6237,6238,468,6239,6240,470,6241,6242,6243,6244,6245,4457,6246,6247,6248,6249,2117,6250,6251,2100,6252,4499,3633,4793,4467,6253,6254,6255	[[0,1,1,1,0,0,0,1,0],[0,0,0,0,0,0,0,1,0],[0,1,1,0,0,0,0,1,0],[0,1,1,0,0,0,0,1,0],[0,0,1,0,0,0,0,1,0],[0,0,1,1,0,0,0,1,0],[0,0,1,0,0,0,0,0,0],[0,0,1,0,0,0,0,1,0],[0,1,1,1,0,0,0,0,0],[0,0,1,1,0,0,0,1,0],[0,0,1,0,0,0,0,0,0],[0,0,1,0,0,0,0,0,0],[0,0,0,0,0,0,0,1,0],[0,0,0,0,0,0,0,1,0],[0,0,1,0,0,0,0,0,0],[0,1,1,0,0,0,0,1,0],[0,1,0,0,1,0,0,0,0],[0,0,1,0,0,0,0,0,0],[0,1,1,0,0,0,0,1,0],[0,1,1,1,1,0,0,1,0],[0,0,0,1,0,0,0,1,0],[0,0,1,0,0,0,0,0,0],[0,0,1,0,0,0,0,0,0],[0,0,1,0,0,0,0,0,0],[0,0,1,0,0,0,0,0,0],[0,0,1,0,0,0,0,0,0],[0,0,0,0,1,0,0,0,0],[0,1,1,0,1,0,0,1,0],[0,0,1,0,0,0,0,0,0],[0,1,1,0,0,0,0,1,0],[0,0,1,0,0,0,0,0,0],[0,0,1,0,0,0,0,0,0],[0,0,1,0,0,0,0,0,0],[0,0,1,0,0,0,0,0,0],[0,0,1,0,0,0,0,0,0],[0,0,1,0,0,0,0,0,0],[0,0,1,0,0,0,0,0,0],[0,0,1,0,0,0,0,0,0],[0,0,1,0,0,0,0,0,0],[0,0,1,0,0,0,0,0,0],[0,0,1,0,0,0,0,0,0],[0,0,1,0,0,0,0,1,0],[0,0,0,0,0,0,0,1,0],[0,0,1,0,0,0,0,0,0],[0,1,1,0,1,0,0,1,0],[0,1,1,0,0,0,0,1,0],[0,1,0,0,0,0,0,0,0],[0,1,1,0,1,0,0,1,0],[0,0,0,0,1,0,0,0,0],[0,0,1,0,0,0,0,0,0],[0,0,1,0,0,0,0,0,0],[0,1,0,0,1,0,0,0,0],[0,1,0,0,0,0,0,0,0],[0,1,0,0,0,0,0,0,0],[0,1,0,0,0,0,0,0,0],[0,1,0,0,0,0,0,0,0],[0,1,0,0,0,0,0,0,0],[0,0,1,0,0,0,0,1,0],[0,1,0,0,0,0,0,0,0],[0,0,0,0,1,0,0,0,0],[0,0,1,0,0,0,0,0,0],[0,0,1,0,0,0,0,0,0],[0,0,1,0,0,0,0,1,0],[0,0,1,1,0,0,0,0,0],[0,0,1,0,0,0,0,0,0],[0,0,1,0,0,0,0,0,0],[0,0,1,0,0,0,0,0,0]]6256	6257,6258,6259,6260,5738,6261,6262,6160,6263,6264,6265,6266,6267,6268,6269,6270,6271	[[0,1,0,0,0,0,0,0,0],[0,1,0,0,0,0,0,0,0],[0,1,0,0,0,0,0,0,0],[0,1,0,0,0,0,0,0,0],[0,1,0,0,0,0,0,0,0],[0,1,0,0,0,0,0,0,0],[0,1,0,0,0,0,0,0,0],[0,1,0,0,0,0,0,0,0],[0,1,0,0,0,0,0,0,0],[0,1,0,0,0,0,0,0,0],[0,1,0,0,0,0,0,0,0],[0,1,0,0,0,0,0,0,0],[0,1,0,0,0,0,0,0,0],[0,1,0,0,0,0,0,0,0],[0,1,0,0,0,0,0,0,0],[0,1,0,0,0,0,0,0,0],[0,1,0,0,0,0,0,0,0]]6272	6273,6274,4529	[[0,0,0,1,0,0,0,0,0],[0,0,0,1,0,0,0,0,0],[0,0,0,1,0,0,0,0,0]]6275	1570,1578,1574,6276,6277,1581,1553,1626,6278,1613,1608,5689,6279,3106,6280,5366,668,6281,4626,2006,6282,6283,6284,4633	[[0,0,1,0,0,0,0,0,0],[0,0,1,0,0,0,0,0,0],[0,0,1,0,1,0,0,0,0],[0,0,1,0,0,0,0,0,0],[0,0,1,0,0,0,0,0,0],[0,0,1,0,0,0,0,0,0],[0,0,1,0,0,0,0,0,0],[0,0,1,0,0,0,0,0,0],[0,0,1,0,0,0,0,0,0],[0,0,0,0,1,0,0,0,0],[0,0,1,0,0,0,0,0,0],[0,0,1,0,0,0,0,0,0],[0,0,1,0,0,0,0,0,0],[0,0,1,1,1,0,0,0,0],[0,0,1,0,0,0,0,0,0],[0,0,1,0,0,0,0,0,0],[0,0,1,0,0,0,0,0,0],[0,0,1,0,1,0,0,0,0],[0,0,1,0,0,0,0,0,0],[0,0,1,0,0,0,0,0,0],[0,0,1,0,0,0,0,0,0],[0,0,1,0,0,0,0,0,0],[0,0,1,0,0,0,0,0,0],[0,0,1,0,0,0,0,0,0]]6285	6286	[[0,0,0,0,1,0,0,0,0]]6287	6288,6289,6290,4563,6291,6292,5088,6293,6294,6295,6296,6297,6298,5070,5098,6299,6300,3435,5003,6301,6302,4582,6303,5351,6304,4565,4567,2930,6305,6306,6307,6308,6309,6310,6311,5367,561,6312,4574,6313,6314,4580,4252,6315,4555,4560,6316,6317,6318,6319,1640,6320,6321,6322,6323,6324	[[1,0,1,0,0,0,0,0,0],[1,0,0,0,0,0,0,0,0],[1,0,1,0,0,0,0,0,0],[1,0,1,0,1,0,0,0,0],[1,0,1,0,0,0,0,0,0],[1,0,0,0,0,0,0,0,0],[0,0,1,0,0,0,0,0,0],[0,0,1,0,0,0,0,0,0],[0,0,1,0,0,0,0,0,0],[1,0,1,0,0,0,0,0,0],[1,0,0,0,0,0,0,0,0],[1,0,1,0,0,0,0,0,0],[0,0,0,0,1,0,0,0,0],[0,0,1,0,0,0,0,0,0],[0,0,1,0,0,0,0,0,0],[1,0,0,0,0,0,0,0,0],[1,0,1,0,0,0,0,0,0],[0,0,1,0,0,0,0,0,0],[0,0,0,0,1,0,0,0,0],[1,0,0,0,0,0,0,0,0],[0,0,1,0,0,0,0,0,0],[0,0,0,0,1,0,0,0,0],[0,0,1,0,1,0,0,0,0],[0,0,1,0,0,0,0,0,0],[1,0,1,0,0,0,0,0,0],[0,0,1,0,0,0,0,0,0],[1,0,0,0,0,0,0,0,0],[1,0,0,0,0,0,0,0,0],[1,0,0,0,0,0,0,0,0],[0,0,1,0,0,0,0,0,0],[0,0,1,0,1,0,0,0,0],[1,0,0,0,0,0,0,0,0],[1,0,0,0,0,0,0,0,0],[1,0,0,0,0,0,0,0,0],[1,0,0,0,0,0,0,0,0],[0,0,1,0,0,0,0,0,0],[1,0,0,0,0,0,0,0,0],[0,0,0,0,1,0,0,0,0],[0,0,0,0,1,0,0,0,0],[0,0,1,0,0,0,0,0,0],[0,0,1,0,0,0,0,0,0],[1,0,0,0,0,0,0,0,0],[1,0,0,0,0,0,0,0,0],[1,0,0,0,0,0,0,0,0],[1,0,1,0,1,0,0,0,0],[1,0,0,0,0,0,0,0,0],[1,0,0,0,0,0,0,0,0],[1,0,0,0,0,0,0,0,0],[1,0,0,0,0,0,0,0,0],[1,0,0,0,0,0,0,0,0],[0,0,1,0,0,0,0,0,0],[1,0,0,0,0,0,0,0,0],[0,0,1,0,0,0,0,0,0],[0,0,1,0,0,0,0,0,0],[0,0,1,0,0,0,0,0,0],[0,0,1,0,0,0,0,0,0]]6325	6326,4529,6327,4267,6328,6274,6329,6330,6331,6332,6273,6333,6334	[[0,0,1,0,0,0,0,0,0],[0,1,1,1,1,0,0,1,0],[0,1,1,0,1,0,1,1,0],[0,0,1,0,0,0,0,0,0],[0,0,1,0,0,0,0,0,0],[0,1,1,1,1,0,1,1,0],[0,0,0,0,1,0,0,1,0],[0,1,0,0,0,0,1,0,0],[0,0,1,0,0,0,0,0,0],[0,0,0,0,1,0,0,0,0],[0,0,0,1,0,0,0,1,0],[0,0,1,0,0,0,0,0,0],[0,0,1,0,0,0,0,0,0]]6335	2301,6336,6337,6338,6339,6340,6341,6342,6343,6344,6345,6346,6347,6348,6349,6350,6351,6352,6353,6354,6355,2334	[[1,1,1,1,0,0,0,0,0],[1,0,1,0,0,0,0,0,0],[1,0,0,0,0,0,0,0,0],[1,0,1,0,0,0,0,0,0],[1,0,0,0,0,0,0,0,0],[1,0,1,0,0,0,0,0,0],[1,1,1,1,0,0,0,0,0],[1,1,1,1,0,0,0,0,0],[0,0,0,0,1,0,0,0,0],[0,0,0,1,0,0,0,0,0],[0,0,0,0,1,0,0,0,0],[0,0,1,0,0,0,0,0,0],[0,1,0,0,0,0,0,0,0],[0,0,1,0,0,0,0,0,0],[0,0,0,0,1,0,0,0,0],[0,0,1,0,0,0,0,0,0],[0,0,0,1,0,0,0,0,0],[0,0,1,0,0,0,0,0,0],[0,0,1,1,0,0,0,0,0],[0,0,0,1,0,0,0,0,0],[0,0,0,0,1,0,0,0,0],[0,0,1,0,0,0,0,0,0]]6356	6357	[[0,0,0,0,1,0,0,0,0]]6358	6359,6360,6361,6362,6363,5479,6364,6365,6366,6367,6368,6369,6370,5454,6371,6372,6373,6374,6375,2331,6376,1570,6377,6345,6378,2412,6379,2576,6380,6381,5900,6382,6383,6384,2340,2299,6385,6386,6387,6388,413	[[1,0,0,0,0,0,0,0,0],[1,0,0,0,0,0,0,0,0],[1,0,1,0,0,0,0,0,0],[0,1,0,0,0,0,0,1,0],[1,0,0,0,0,0,0,1,0],[0,0,1,0,0,0,0,0,0],[0,1,1,0,0,0,0,0,0],[0,1,1,0,0,0,0,0,0],[0,1,1,0,0,0,0,0,0],[1,1,0,1,0,0,0,1,0],[1,1,1,0,0,0,0,1,0],[0,1,0,1,0,0,0,1,0],[0,0,1,0,0,0,0,0,0],[0,0,1,0,0,0,0,0,0],[1,0,1,1,0,0,0,1,0],[0,1,0,0,0,0,0,0,0],[0,1,1,0,0,0,0,0,0],[0,0,1,0,0,0,0,1,0],[0,1,0,0,0,0,0,0,0],[1,0,0,0,0,0,0,0,0],[0,1,0,0,0,0,0,0,0],[0,0,1,0,0,0,0,0,0],[1,0,0,0,0,0,0,0,0],[1,0,0,0,0,0,0,0,0],[1,0,0,0,0,0,0,0,0],[1,0,0,0,0,0,0,0,0],[1,0,0,0,0,0,0,0,0],[1,0,0,0,0,0,0,0,0],[0,1,0,0,0,0,0,0,0],[0,0,1,0,0,0,0,1,0],[1,0,0,0,0,0,0,0,0],[0,0,1,0,0,0,0,0,0],[1,0,0,0,0,0,0,0,0],[1,0,0,0,0,0,0,0,0],[1,0,0,0,0,0,0,0,0],[1,0,0,0,0,0,0,0,0],[1,0,0,0,0,0,0,0,0],[1,0,0,0,0,0,0,0,0],[0,0,0,0,0,0,0,1,0],[0,0,0,0,0,0,0,1,0],[0,0,1,0,0,0,0,0,0]]6389	6390,6391,6392,6393	[[0,0,0,0,1,0,0,0,0],[0,0,0,0,1,0,0,0,0],[0,0,0,0,1,0,0,0,0],[0,0,0,0,1,0,0,0,0]]6394	6395,6396,6397,6398,6399,6400,4560,6401,6402,1534,6403,6404,6405,6406,6407,4818,6408,6409,6410,6411,6412,6413,6414,6415,6416,6417,6418,2795,6419,1313,6296,6420,6421,6422,6423,6424,6425,6426,4582,6427,6428,6429,6430,6431,6432,6433,6434,6435,1312,6436,6437,5464,5069,6438,6439,6440,1794,6441,5070,2434,5264	[[0,0,1,0,0,0,0,0,0],[0,0,1,0,0,0,0,0,0],[0,0,1,0,0,0,0,0,0],[0,0,1,0,0,0,0,0,0],[0,0,1,0,0,0,0,0,0],[0,0,1,0,0,0,0,0,0],[0,0,1,0,0,0,0,0,0],[0,0,1,0,0,0,0,0,0],[0,0,1,0,0,0,0,0,0],[0,0,1,0,0,0,0,0,0],[0,0,1,0,0,0,0,0,0],[0,0,1,0,0,0,0,0,0],[0,0,0,0,1,0,0,0,0],[0,0,1,0,0,0,0,0,0],[0,0,1,0,0,0,0,0,0],[0,0,1,0,0,0,0,0,0],[0,0,1,0,0,0,0,0,0],[0,0,1,0,0,0,0,0,0],[0,0,1,0,0,0,0,0,0],[0,0,1,0,0,0,0,0,0],[0,0,1,0,0,0,0,0,0],[0,0,1,0,0,0,0,0,0],[0,0,1,0,0,0,0,0,0],[0,0,1,0,0,0,0,0,0],[0,0,1,0,0,0,0,0,0],[0,0,1,0,0,0,0,0,0],[0,0,1,0,0,0,0,0,0],[0,0,0,1,0,0,0,0,0],[0,0,1,0,0,0,0,0,0],[0,0,1,0,0,0,0,0,0],[0,0,1,0,0,0,0,0,0],[0,0,1,0,0,0,0,0,0],[0,0,1,0,0,0,0,0,0],[0,1,1,0,0,0,0,0,0],[0,1,1,1,1,0,0,0,0],[0,0,1,0,0,0,0,0,0],[0,0,1,0,0,0,0,0,0],[0,0,1,0,0,0,0,0,0],[0,0,1,0,0,0,0,0,0],[0,0,1,0,0,0,0,0,0],[0,0,1,0,0,0,0,0,0],[0,0,1,0,0,0,0,0,0],[0,0,1,0,0,0,0,0,0],[0,0,1,0,0,0,0,0,0],[0,0,1,1,0,0,0,0,0],[0,0,1,0,0,0,0,0,0],[0,0,1,0,0,0,0,0,0],[0,0,1,0,0,0,0,0,0],[0,0,1,0,0,0,0,0,0],[0,0,1,0,0,0,0,0,0],[0,0,1,0,0,0,0,0,0],[0,0,1,0,0,0,0,0,0],[0,0,1,0,0,0,0,0,0],[0,0,1,0,0,0,0,0,0],[0,0,1,0,0,0,0,0,0],[0,0,1,0,0,0,0,0,0],[0,0,1,0,0,0,0,0,0],[0,0,0,1,0,0,0,0,0],[0,0,1,0,0,0,0,0,0],[0,0,1,0,0,0,0,0,0],[0,0,1,0,0,0,0,0,0]]6442	6443,6444,6445,6446,6447,6448,6449,6450,6451,127,6452,6453,6454,6455	[[0,0,0,0,1,0,0,0,0],[0,1,0,0,0,0,0,0,0],[0,1,0,0,1,0,0,0,0],[0,1,0,0,0,0,0,0,0],[0,1,0,0,0,0,0,0,0],[0,1,0,0,0,0,0,0,0],[0,1,0,0,0,0,0,0,0],[0,1,0,0,0,0,0,0,0],[0,1,0,0,0,0,0,0,0],[0,1,0,0,0,0,0,0,0],[0,1,0,0,0,0,0,0,0],[0,1,0,0,0,0,0,0,0],[0,0,0,0,1,0,0,0,0],[0,1,0,0,0,0,0,0,0]]6456	6457,6458,6459,6460	[[0,0,0,0,1,0,0,0,0],[0,0,0,0,1,0,0,0,0],[0,0,0,0,1,0,0,0,0],[0,0,0,0,1,0,0,0,0]]6461	6462,6463,6464,4407,6465,6466,1648,6467,1678,1679	[[0,0,0,1,0,0,0,0,0],[0,0,0,1,0,0,0,0,0],[0,0,0,1,0,0,0,0,0],[0,0,0,1,0,0,0,0,0],[0,0,0,1,0,0,0,0,0],[0,0,0,1,0,0,0,0,0],[0,0,0,1,0,0,0,0,0],[0,0,0,1,0,0,0,0,0],[0,0,0,1,0,0,0,0,0],[0,0,0,1,0,0,0,0,0]]6468	6469,6470,6471	[[0,0,0,1,0,0,0,0,0],[0,0,0,1,0,0,0,0,0],[0,0,0,1,0,0,0,0,0]]6472	6473	[[0,0,0,0,1,0,0,0,0]]6474	5011	[[0,0,0,0,1,0,0,0,0]]6475	6476,6477,6478,6479,6480,6481,21,6482,6483,6403,6484,6485,6486,6487,6488,6489,6490	[[0,0,0,1,0,0,0,0,1],[0,0,0,0,0,0,0,0,1],[0,0,0,0,0,0,0,0,1],[0,0,0,1,0,0,0,0,0],[0,0,0,0,0,0,0,0,1],[0,0,0,0,0,0,0,0,1],[0,0,0,0,0,0,0,0,1],[0,0,0,1,0,0,0,0,0],[0,0,0,0,0,0,0,0,1],[0,0,0,1,0,0,0,0,0],[0,0,0,0,0,0,0,0,1],[0,0,0,0,0,0,0,0,1],[0,0,0,1,0,0,0,0,1],[0,0,0,1,0,0,0,0,0],[0,0,0,0,0,0,0,0,1],[0,0,0,1,0,0,0,0,0],[0,0,0,0,0,0,0,0,1]]6491	5072	[[0,0,0,0,1,0,0,0,0]]6492	6493	[[0,0,0,0,1,0,0,0,0]]6494	6470,6471,6469	[[0,0,0,1,0,0,0,0,0],[0,0,0,1,0,0,0,0,0],[0,0,0,1,0,0,0,0,0]]6495	1668,2398,1654,6462,6451,6496,6497,6498,4452,6499,6500,1476,6501,2794,6502,6503,6504,6505,6506,6463,1648,6466,1701,6507,1652,6508,5403,6509,6510,6464,6511,4407,6512,6513,6514,6515,6516,6517,1656,1679,6518,6519,6520,6521,2685,6522,1971,1232,6523,1698,2982,1386,1657,6524,6525,6526,6467,5485,6527,6528,6529,6530,6531,6532,6465,1691,6533,6534,1687,2649,6535,6536,6537,1678,6538,6539,6540,6541,6542,6543,6448	[[0,1,1,0,0,1,1,1,0],[0,1,1,0,0,0,0,1,0],[0,0,1,0,0,0,0,0,0],[0,1,0,1,0,0,1,1,0],[0,1,1,0,0,0,0,0,0],[0,1,0,0,0,0,1,0,0],[0,1,0,0,0,0,1,0,0],[0,1,0,0,0,0,1,0,0],[0,1,0,0,0,0,1,0,0],[0,1,1,0,0,0,0,1,1],[0,1,1,0,0,0,0,1,0],[0,1,0,0,0,0,0,0,0],[0,1,0,0,0,0,1,0,0],[0,1,0,0,0,0,0,0,0],[0,0,1,0,0,0,0,0,0],[0,0,1,0,0,0,0,0,0],[0,1,0,0,0,0,1,0,0],[0,1,0,0,1,0,1,0,0],[0,1,1,0,0,0,1,0,0],[0,1,1,1,0,0,1,1,0],[0,1,1,1,0,0,0,0,0],[0,1,1,1,0,1,1,0,0],[0,1,1,0,0,1,0,0,1],[0,1,1,0,0,0,0,0,0],[0,1,1,0,0,0,0,1,0],[0,0,1,0,0,0,0,0,0],[0,1,0,0,0,0,0,1,1],[0,1,1,0,0,0,1,0,1],[0,1,0,0,0,0,0,0,0],[0,1,1,1,0,0,1,1,1],[0,0,1,0,0,0,0,0,0],[0,1,1,1,0,1,1,0,1],[0,0,1,0,0,0,0,0,1],[0,0,1,0,0,0,0,0,0],[0,1,1,0,0,0,1,0,0],[0,1,0,0,0,0,1,0,0],[0,1,0,0,0,0,0,1,0],[0,1,0,0,0,0,1,1,0],[0,0,1,0,0,0,0,0,0],[0,1,1,1,0,1,1,0,1],[0,1,0,0,0,0,0,0,0],[0,1,0,0,0,0,1,0,0],[0,1,0,0,0,0,1,0,0],[0,1,0,0,0,0,1,0,0],[0,1,0,0,0,0,0,0,0],[0,1,0,0,0,0,1,0,0],[0,1,0,0,0,0,1,0,0],[0,1,0,0,0,0,1,0,0],[0,0,1,0,0,0,0,0,0],[0,1,1,0,0,0,0,0,1],[0,1,1,0,0,0,1,0,0],[0,0,1,0,0,0,0,1,0],[0,0,1,0,0,0,0,0,0],[0,0,1,0,0,0,0,0,0],[0,0,0,0,0,0,0,0,1],[0,1,1,0,0,0,0,0,0],[0,0,1,1,0,0,0,0,1],[0,0,0,0,0,0,1,0,0],[0,0,1,0,0,0,0,0,0],[0,0,1,0,0,0,0,0,0],[0,0,0,0,0,0,0,1,0],[0,1,1,0,0,0,1,1,0],[0,0,0,0,0,0,0,1,0],[0,0,1,0,0,0,0,1,0],[0,0,1,1,0,0,0,1,1],[0,0,0,0,0,1,0,0,1],[0,1,0,0,0,0,1,0,0],[0,0,1,0,0,0,0,0,0],[0,0,0,0,0,1,0,0,0],[0,0,1,0,0,0,0,0,0],[0,0,1,0,0,0,1,0,0],[0,0,1,0,0,0,0,0,0],[0,0,0,0,0,0,0,0,1],[0,0,0,1,0,0,0,1,1],[0,0,0,0,0,0,0,0,1],[0,0,0,0,1,0,0,0,0],[0,1,0,0,0,0,1,0,0],[0,0,1,0,0,0,0,0,0],[0,0,0,0,0,0,1,0,0],[0,0,0,0,0,0,0,1,0],[0,0,1,0,0,0,0,0,1]]6544	4879,6545,6546,5708,6547,5189,6548,6549,6550,6551,6552,5699,6280,5705,6553,6554,6555,5707,6556,5205,6557,6558,6559,6560,6561	[[0,0,1,0,1,0,0,1,0],[0,0,1,0,0,0,0,0,0],[0,0,1,0,0,0,0,0,0],[0,0,1,0,0,0,0,0,0],[0,0,1,0,0,0,0,0,0],[0,0,0,0,0,0,0,1,0],[0,0,1,0,0,0,0,0,0],[0,0,0,0,0,0,0,1,0],[0,1,1,1,0,0,0,0,0],[0,1,1,1,0,0,0,1,0],[0,1,1,0,1,0,0,1,0],[0,0,1,0,0,0,0,0,0],[0,1,1,0,0,0,0,1,0],[0,0,0,0,1,0,0,0,0],[0,0,1,0,0,0,0,1,0],[0,0,0,1,0,0,0,1,0],[0,1,1,0,0,0,0,1,0],[0,0,1,0,0,0,0,0,0],[0,0,1,0,0,0,0,0,0],[0,1,1,0,0,0,0,0,0],[0,1,0,0,0,0,0,0,0],[0,0,0,0,0,0,0,1,0],[0,0,0,0,0,0,0,1,0],[0,0,1,0,0,0,0,0,0],[0,1,0,0,0,0,0,0,0]]6562	6563,6564	[[0,0,0,0,1,0,0,0,0],[0,0,0,0,1,0,0,0,0]]6565	6566	[[0,0,0,0,1,0,0,0,0]]6567	6568,6569,6570,6571,3031,6572,6573,6574	[[0,0,0,1,0,0,0,0,0],[0,0,0,1,0,0,0,0,0],[0,0,0,1,0,0,0,0,0],[0,0,0,1,0,0,0,0,0],[0,0,0,0,1,0,0,0,0],[0,0,0,1,0,0,0,0,0],[0,0,0,1,0,0,0,0,0],[0,0,0,1,0,0,0,0,0]]6575	6576,6577,6578,6579,6580,6581,6582,6583,6584,6585,6586,2423,6587,6588,6589,6590,6591,6592,6593,6594,6595,6596,6597,6598,6599,3550,6600,6601,4985,6602,723,6603,6604,6605,6606,6607,6608,6609,6610,6611,6612,6613,6614,6615,6616,6617,6618,6619,6620	[[0,1,1,0,0,0,0,0,0],[0,1,1,0,1,0,1,0,0],[0,1,1,0,0,0,1,0,0],[0,1,1,0,0,0,0,0,0],[0,0,1,0,0,0,0,0,0],[0,0,0,0,1,0,0,0,0],[0,0,1,0,0,0,0,0,0],[0,1,1,0,0,0,0,0,0],[0,1,1,1,0,0,1,0,0],[0,1,0,0,0,0,1,0,0],[0,0,1,0,1,0,0,0,0],[0,0,1,1,0,0,0,0,0],[0,0,1,0,0,0,0,0,0],[0,1,1,1,0,0,0,0,0],[0,1,0,0,1,0,0,0,0],[0,0,0,0,1,0,0,0,0],[0,0,1,0,0,0,0,0,0],[0,0,1,0,0,0,0,0,0],[0,1,1,0,0,0,0,0,0],[0,1,1,0,0,0,1,0,0],[0,0,1,0,0,0,0,0,0],[0,0,1,0,0,0,0,0,0],[0,0,0,0,1,0,0,0,0],[0,0,1,0,0,0,0,0,0],[0,1,1,0,0,0,0,0,0],[0,0,1,0,0,0,0,0,0],[0,0,1,0,0,0,0,0,0],[0,1,1,0,0,0,1,0,0],[0,0,1,0,0,0,0,0,0],[0,0,1,0,0,0,0,0,0],[0,1,1,0,0,0,0,0,0],[0,1,1,0,0,0,1,0,0],[0,1,1,0,0,0,1,0,0],[0,0,1,0,0,0,0,0,0],[0,0,1,0,0,0,0,0,0],[0,0,0,0,0,0,1,0,0],[0,1,0,0,0,0,0,0,0],[0,1,1,0,0,0,1,0,0],[0,1,1,0,0,0,1,0,0],[0,1,1,0,0,0,0,0,0],[0,0,1,0,0,0,0,0,0],[0,0,1,0,0,0,0,0,0],[0,1,1,0,0,0,1,0,0],[0,0,1,0,0,0,0,0,0],[0,1,1,0,0,0,0,0,0],[0,0,1,0,0,0,0,0,0],[0,0,1,0,0,0,0,0,0],[0,0,1,0,0,0,0,0,0],[0,0,1,0,0,0,0,0,0]]6621	6622	[[0,0,0,0,1,0,0,0,0]]6623	6624,6625,6626,4268,4267,3155,5282,6627,6628,5257,513,6629,4956,6630,4276,6631,6632,6633,6634,5267,6635,6636,181,6637,6638,6639,6640,6641,6642,6643,4275,6644,3840,6645,6646,6647,6648,6649,6650,6083,6651,6060,6652,6064,6094,6054,6082,6242,6058,6653,6654,5945,6655,2832,6656,6084	[[1,0,0,0,0,0,0,0,0],[1,0,0,0,0,0,0,0,0],[1,0,0,0,0,0,0,0,0],[1,0,0,0,0,0,0,0,0],[1,0,0,0,0,0,0,0,0],[1,0,0,0,0,0,0,0,0],[1,0,0,0,0,0,0,0,0],[1,0,0,0,0,0,0,0,0],[1,0,0,0,0,0,0,0,0],[1,0,0,0,0,0,0,0,0],[1,0,0,0,0,0,0,0,0],[1,0,0,0,0,0,0,0,0],[1,1,0,0,0,0,0,1,0],[1,0,0,0,0,0,0,0,0],[1,0,0,0,0,0,0,0,0],[0,0,1,0,0,0,0,1,0],[1,1,1,0,0,0,0,1,0],[0,1,1,0,0,0,0,0,1],[1,1,1,0,0,0,0,0,0],[1,0,0,0,0,0,0,0,0],[1,1,1,0,0,0,0,1,0],[1,0,0,0,0,0,0,0,0],[1,0,0,0,0,0,0,0,0],[0,1,0,0,0,0,0,1,0],[0,1,1,1,0,0,0,1,1],[1,0,1,0,0,0,0,1,1],[0,0,0,0,0,0,0,1,0],[0,1,1,0,0,0,0,0,1],[1,1,1,1,0,0,0,1,1],[1,1,0,0,0,0,0,0,0],[1,0,0,0,0,0,0,0,0],[1,1,1,1,0,0,0,1,1],[1,1,1,0,0,0,0,1,0],[0,0,1,0,0,0,0,0,0],[1,1,1,0,0,0,0,0,0],[1,0,0,0,0,0,0,0,0],[1,0,0,0,0,0,0,0,0],[1,1,1,0,0,0,0,1,1],[0,0,1,0,0,0,0,1,0],[0,0,1,0,0,0,0,0,0],[1,0,1,0,1,0,0,0,0],[0,0,1,0,0,0,0,0,0],[0,0,1,0,0,0,0,0,0],[0,0,1,0,0,0,0,0,0],[0,0,1,0,0,0,0,0,0],[0,0,1,0,0,0,0,0,0],[0,0,1,0,0,0,0,0,0],[0,0,1,0,0,0,0,1,0],[0,0,1,0,0,0,0,0,0],[1,0,0,0,0,0,0,0,0],[0,0,1,0,0,0,0,0,0],[0,0,1,0,0,0,0,0,0],[1,0,0,0,0,0,0,0,0],[0,1,0,0,0,0,0,0,0],[0,0,0,0,0,0,0,1,0],[0,0,1,0,0,0,0,0,0]]6657	6658	[[0,0,0,0,1,0,0,0,0]]6659	4678	[[0,0,0,0,1,0,0,0,0]]6660	6661,1839,2929,6662,6663,6664,6351,6665,6666,6667,6668,6669,1796,6440,6670,6671,1791,6672	[[0,0,1,1,0,0,0,0,0],[0,0,1,0,1,0,0,0,0],[0,0,1,0,0,0,0,0,0],[0,0,1,0,1,0,0,0,0],[0,0,1,1,1,0,0,0,0],[0,0,1,0,0,0,0,0,0],[0,0,1,0,0,0,0,0,0],[0,0,1,0,0,0,0,0,0],[0,0,1,0,0,0,0,0,0],[0,0,1,0,0,0,0,0,0],[0,0,0,0,1,0,0,0,0],[0,0,1,0,1,0,0,0,0],[0,0,1,0,0,0,0,0,0],[0,0,1,0,0,0,0,0,0],[0,0,1,0,0,0,0,0,0],[0,0,1,0,0,0,0,0,0],[0,0,1,0,0,0,0,0,0],[0,0,0,0,1,0,0,0,0]]6673	87,6674,6675,6676,6677,6678	[[0,0,0,1,0,0,0,0,0],[0,0,0,1,0,0,0,0,0],[0,0,0,1,0,0,0,0,0],[0,0,0,1,0,0,0,0,0],[0,0,0,1,0,0,0,0,0],[0,0,0,1,0,0,0,0,0]]6679	6680	[[0,0,0,0,1,0,0,0,0]]6681	6682,6683,6684,6685	[[0,0,0,0,1,0,0,0,0],[0,0,0,0,1,0,0,0,0],[0,0,0,0,1,0,0,0,0],[0,0,0,0,1,0,0,0,0]]6686	6687,6688,6689,6690,6691	[[0,0,0,0,1,0,0,0,0],[0,0,0,0,1,0,0,0,0],[0,0,0,0,1,0,0,0,0],[0,0,0,0,1,0,0,0,0],[0,0,0,0,1,0,0,0,0]]6692	6693,6694,6695,6696,6697,6698,6699,6700,6701,6702,6703,6704,1898,6705,6706,6707,6708,6709,6710,6711,6712,6713,6714,6715,6716,6717,6718,6719,6720,6721,6722,6723,6724	[[0,0,1,0,0,0,0,0,0],[0,0,1,0,0,0,0,0,0],[0,0,1,0,0,0,0,0,0],[0,0,1,0,0,0,0,0,0],[0,0,1,0,0,0,0,0,0],[0,0,1,0,0,0,0,0,0],[0,0,1,1,0,0,0,0,0],[0,0,1,0,0,0,0,0,0],[0,0,1,0,0,0,0,0,0],[0,0,1,0,0,0,0,0,0],[0,0,1,0,0,0,0,0,0],[0,0,1,1,0,0,0,0,0],[0,0,1,0,0,0,0,0,0],[0,0,1,0,0,0,0,0,0],[0,0,1,0,0,0,0,0,0],[0,0,1,0,0,0,0,0,0],[0,0,1,0,0,0,0,0,0],[0,0,1,0,0,0,0,0,0],[0,0,1,0,0,0,0,0,0],[0,0,1,0,1,0,0,0,0],[0,0,1,0,1,0,0,0,0],[0,0,1,0,0,0,0,0,0],[0,0,1,0,0,0,0,0,0],[0,0,1,1,1,0,0,0,0],[0,0,1,0,0,0,0,0,0],[0,0,1,0,0,0,0,0,0],[0,0,1,0,0,0,0,0,0],[0,0,1,0,0,0,0,0,0],[0,0,0,0,1,0,0,0,0],[0,0,1,0,0,0,0,0,0],[0,0,1,0,0,0,0,0,0],[0,0,1,0,0,0,0,0,0],[0,0,1,0,0,0,0,0,0]]6725	6726,3928	[[0,0,0,0,1,0,0,0,0],[0,0,0,0,1,0,0,0,0]]6727	6728,6729	[[0,0,0,0,1,0,0,0,0],[0,0,0,0,1,0,0,0,0]]6730	6731,6732,6733	[[0,0,0,0,1,0,0,0,0],[0,0,0,0,1,0,0,0,0],[0,0,0,0,1,0,0,0,0]]6734	6735,6736	[[0,0,0,0,1,0,0,0,0],[0,0,0,0,1,0,0,0,0]]6737	6506,6738,6739,6740,6741,1534,6742,4010,6743,6744,6745,6746,6747,6748,1528,6749,6412,148,6420,6750,6751,6752,1257,6753,373,6754,6755,6756,6757,5818,3020,6758,1313,6759,6760,6761,6762,6763,2997,6764,6765,6766,6767,6768,3370,6769,6770,6771,6772,6773,6774,6775,6776,6777,6778,6779,6780,6781,6782,5432,6783,6395,3005,1312,6784,6785,6786,6787,3013,6788,6789,6790,1258,6791,824,6792,6793,6794,6795,6796	[[0,1,0,0,0,0,0,0,0],[0,1,0,0,0,0,0,0,0],[0,1,1,0,0,0,0,0,0],[0,0,1,0,0,0,0,0,0],[0,1,1,0,0,0,0,0,0],[0,1,1,1,0,0,0,0,0],[0,1,1,0,0,0,0,0,0],[0,0,1,0,0,0,0,0,0],[0,1,1,0,0,0,0,0,0],[0,1,1,0,0,0,0,0,0],[0,0,1,1,0,0,0,0,0],[0,0,1,0,0,0,0,0,0],[0,0,1,0,0,0,0,0,0],[0,0,1,0,0,0,0,0,0],[0,0,1,1,0,0,0,0,0],[0,0,1,0,0,0,0,0,0],[0,0,1,1,0,0,0,0,0],[0,0,1,0,0,0,0,0,0],[0,0,1,0,0,0,0,0,0],[0,1,1,0,0,0,0,0,0],[0,0,1,0,0,0,0,0,0],[0,0,1,0,0,0,0,0,0],[0,0,1,0,0,0,0,0,0],[0,0,0,0,1,0,0,0,0],[0,0,1,0,0,0,0,0,0],[0,1,1,0,0,0,0,0,0],[0,0,1,0,0,0,0,0,0],[0,0,0,0,1,0,0,0,0],[0,0,1,0,0,0,0,0,0],[0,0,1,0,0,0,0,0,0],[0,1,0,0,0,0,0,0,0],[0,0,1,0,0,0,0,0,0],[0,0,1,0,0,0,0,0,0],[0,0,1,0,0,0,0,0,0],[0,0,1,0,0,0,0,0,0],[0,0,1,0,0,0,0,0,0],[0,0,1,0,0,0,0,0,0],[0,0,1,0,0,0,0,0,0],[0,0,1,0,0,0,0,0,0],[0,0,1,0,0,0,0,0,0],[0,0,1,0,1,0,0,0,0],[0,0,1,0,0,0,0,0,0],[0,0,1,0,0,0,0,0,0],[0,0,1,0,0,0,0,0,0],[0,0,1,0,0,0,0,0,0],[0,0,1,0,0,0,0,0,0],[0,0,1,0,0,0,0,0,0],[0,0,1,0,0,0,0,0,0],[0,0,1,0,0,0,0,0,0],[0,0,1,0,0,0,0,0,0],[0,0,1,0,0,0,0,0,0],[0,0,1,0,1,0,0,0,0],[0,0,0,0,1,0,0,0,0],[0,0,1,0,0,0,0,0,0],[0,0,1,0,0,0,0,0,0],[0,0,1,0,0,0,0,0,0],[0,0,1,0,0,0,0,0,0],[0,0,1,0,0,0,0,0,0],[0,0,1,0,0,0,0,0,0],[0,0,1,0,0,0,0,0,0],[0,0,1,0,0,0,0,0,0],[0,0,1,0,0,0,0,0,0],[0,0,1,0,0,0,0,0,0],[0,0,1,0,0,0,0,0,0],[0,0,1,0,0,0,0,0,0],[0,0,1,0,0,0,0,0,0],[0,0,1,0,0,0,0,0,0],[0,0,1,0,0,0,0,0,0],[0,0,1,0,0,0,0,0,0],[0,0,1,0,0,0,0,0,0],[0,0,1,0,0,0,0,0,0],[0,0,1,0,0,0,0,0,0],[0,0,1,0,0,0,0,0,0],[0,0,1,0,0,0,0,0,0],[0,0,1,0,0,0,0,0,0],[0,0,1,0,0,0,0,0,0],[0,0,1,0,0,0,0,0,0],[0,0,1,0,0,0,0,0,0],[0,0,1,0,0,0,0,0,0],[0,0,1,0,0,0,0,0,0]]6797	6798	[[0,0,0,0,1,0,0,0,0]]6799	6800,6801	[[0,0,0,0,1,0,0,0,0],[0,0,0,0,1,0,0,0,0]]6802	6803,6804,6805,6806,6807,5901,6808,6809,6810,6811,6812,5027,6813,6814,6815,6816,6817,6818,6819,6820,6821,6822,1187,6823,6824,1141,2224,6825,6826,6827,6828,6829,5864,6830,6831,6832,5896,6833,3159,6834,1140	[[0,1,0,0,0,0,0,1,0],[0,1,0,0,0,0,0,0,0],[0,1,0,0,0,0,0,0,0],[0,1,0,0,0,0,0,0,0],[0,1,1,0,0,0,0,1,0],[0,1,0,0,1,0,0,0,0],[0,0,1,0,0,0,0,0,0],[0,1,1,1,0,0,0,1,0],[0,1,0,0,0,0,0,1,0],[0,1,0,0,0,0,0,0,0],[0,1,0,0,0,0,0,0,0],[0,1,1,0,0,0,0,0,0],[0,1,0,0,0,0,0,1,0],[0,1,1,1,1,0,0,0,0],[0,0,0,1,0,0,0,0,0],[0,1,1,0,0,0,0,0,0],[0,1,0,0,0,0,0,0,0],[0,1,1,0,0,0,0,1,0],[0,1,1,0,0,0,0,0,0],[0,1,0,0,0,0,0,0,0],[0,1,1,0,0,0,0,0,0],[0,1,0,0,0,0,0,0,0],[0,0,1,0,0,0,0,0,0],[0,0,0,0,0,0,0,1,0],[0,0,1,0,0,0,0,0,0],[0,0,1,0,0,0,0,1,0],[0,0,0,0,0,0,0,1,0],[0,0,0,0,0,0,0,1,0],[0,0,0,0,0,0,0,1,0],[0,0,0,0,0,0,0,1,0],[0,0,0,0,0,0,0,1,0],[0,0,1,0,0,0,0,0,0],[0,0,1,0,0,0,0,1,0],[0,0,1,0,0,0,0,0,0],[0,0,1,0,0,0,0,0,0],[0,0,0,1,0,0,0,0,0],[0,0,1,0,0,0,0,0,0],[0,0,1,0,0,0,0,1,0],[0,0,1,0,0,0,0,1,0],[0,0,1,0,0,0,0,0,0],[0,0,0,0,0,0,0,1,0]]6835	6836,6837,6838	[[0,0,0,0,1,0,0,0,0],[0,0,0,0,1,0,0,0,0],[0,0,0,0,1,0,0,0,0]]6839	4219,6840,6841,6842,5895,6843,6844,1140,6845,6846,6847,6848,6849,6850,6851,6852,5881,6809,6853,6814,5901,6854,5913,6855,2264,6856,6857,6858,5445,6859,6860,6861,6862,6863,6864,6865,6866,6867,6868,6869,3020,6870	[[0,0,1,0,0,0,0,0,0],[0,0,1,0,1,0,0,0,0],[0,1,1,0,1,0,0,0,0],[0,1,1,1,0,0,0,0,0],[0,1,0,0,0,0,0,0,0],[0,0,1,1,0,0,0,0,0],[0,1,0,0,0,0,0,0,0],[0,1,0,0,0,0,0,0,0],[0,1,1,1,0,0,0,0,0],[0,0,1,0,0,0,0,0,0],[0,1,0,0,0,0,0,0,0],[0,1,1,0,0,0,0,0,0],[0,1,0,0,0,0,0,0,0],[0,1,1,1,0,0,0,0,0],[0,1,0,0,0,0,0,0,0],[0,0,1,0,0,0,0,0,0],[0,1,1,0,0,0,0,0,0],[0,1,0,0,0,0,0,0,0],[0,1,0,0,0,0,0,0,0],[0,1,0,0,0,0,0,0,0],[0,1,0,0,0,0,0,0,0],[0,0,1,0,0,0,0,0,0],[0,1,1,0,0,0,0,0,0],[0,1,0,0,0,0,0,0,0],[0,0,1,0,0,0,0,0,0],[0,0,0,0,1,0,0,0,0],[0,0,1,0,0,0,0,0,0],[0,0,1,1,0,0,0,0,0],[0,0,1,0,0,0,0,0,0],[0,0,1,0,0,0,0,0,0],[0,0,1,0,0,0,0,0,0],[0,0,1,1,0,0,0,0,0],[0,0,1,0,0,0,0,0,0],[0,0,1,0,0,0,0,0,0],[0,0,1,0,0,0,0,0,0],[0,0,1,0,0,0,0,0,0],[0,0,1,0,0,0,0,0,0],[0,0,1,0,0,0,0,0,0],[0,0,1,0,0,0,0,0,0],[0,0,1,0,0,0,0,0,0],[0,0,1,0,0,0,0,0,0],[0,0,1,0,0,0,0,0,0]]6871	6872	[[0,0,0,0,1,0,0,0,0]]6873	5833,5798	[[0,0,0,0,1,0,0,0,0],[0,0,0,0,1,0,0,0,0]]6874	6875	[[0,0,0,0,1,0,0,0,0]]6876	6877,6878,6879	[[0,0,0,0,1,0,0,0,0],[0,0,0,0,1,0,0,0,0],[0,0,0,0,1,0,0,0,0]]6880	6881	[[0,0,0,0,1,0,0,0,0]]6882	2814,2819,6883,2805,6884,6885,2815,6886,2818,2817,2797,2031,3248,6887,5327,6888,2654,321,6889,2802,2801,6890	[[0,1,0,0,0,0,1,1,0],[0,1,0,0,0,0,1,0,0],[0,1,0,0,0,0,0,0,0],[0,1,0,0,0,0,0,0,0],[0,1,0,0,0,0,0,0,0],[0,1,0,0,1,0,0,1,0],[0,1,0,0,1,0,1,1,0],[0,0,0,0,0,0,0,1,0],[0,1,0,0,0,0,1,1,0],[0,0,0,0,0,0,0,1,0],[0,1,0,0,0,0,1,0,0],[0,1,0,0,0,0,0,1,0],[0,1,0,0,0,0,1,1,0],[0,0,0,0,1,0,0,0,0],[0,1,0,0,0,0,0,0,0],[0,0,0,0,0,0,0,1,0],[0,1,0,0,0,0,0,0,0],[0,0,0,0,1,0,0,0,0],[0,1,0,0,0,0,0,0,0],[0,1,0,0,0,0,0,0,0],[0,1,0,0,0,0,1,1,0],[0,1,0,0,0,0,0,0,0]]6891	6892	[[0,0,0,0,1,0,0,0,0]]6893	6894,6296,6895	[[0,0,0,0,1,0,0,0,0],[0,0,0,0,1,0,0,0,0],[0,0,0,0,1,0,0,0,0]]6896	6897	[[0,0,0,0,1,0,0,0,0]]6898	3786,3790,3797,3830,3826,3835,3793,3788,3789,3815,3832,3822,3795,3806,3824,3794,3814,3831	[[0,0,0,1,0,0,0,0,0],[0,0,0,1,0,0,0,0,0],[0,0,0,1,0,0,0,0,0],[0,0,0,1,0,0,0,0,0],[0,0,0,1,0,0,0,0,0],[0,0,0,1,0,0,0,0,0],[0,0,0,1,0,0,0,0,0],[0,0,0,1,0,0,0,0,0],[0,0,0,1,0,0,0,0,0],[0,0,0,1,0,0,0,0,0],[0,0,0,1,0,0,0,0,0],[0,0,0,1,0,0,0,0,0],[0,0,0,1,0,0,0,0,0],[0,0,0,1,0,0,0,0,0],[0,0,0,1,0,0,0,0,0],[0,0,0,1,0,0,0,0,0],[0,0,0,1,0,0,0,0,0],[0,0,0,1,0,0,0,0,0]]6899	6900,6901,4615,6902,6903,6904,3766,6905,6906,6907,6908,3691,6909,6910,3936,6911,6912,3698,6913,6914	[[0,1,1,0,0,0,1,1,0],[0,0,0,0,1,0,0,1,0],[0,0,0,1,0,0,0,0,0],[0,0,0,0,0,0,0,1,0],[0,0,1,0,0,0,0,1,0],[0,0,1,1,0,0,0,0,0],[0,1,1,0,0,0,0,0,0],[0,0,1,0,0,0,0,1,0],[0,0,0,1,0,0,0,1,0],[0,0,1,0,0,0,0,0,0],[0,0,1,0,0,0,0,0,0],[0,0,1,0,0,0,0,1,0],[0,0,1,0,0,0,0,1,0],[0,0,1,0,0,0,1,1,0],[0,0,1,0,0,0,0,0,0],[0,1,1,0,0,0,1,1,0],[0,0,0,0,0,0,0,1,0],[0,0,1,0,0,0,0,0,0],[0,0,0,0,0,0,0,1,0],[0,0,1,0,0,0,0,0,0]]6915	6916,6917,6918	[[0,0,0,1,0,0,0,0,0],[0,0,0,1,0,0,0,0,0],[0,0,0,1,0,0,0,0,0]]6919	6920	[[0,0,0,0,1,0,0,0,0]]6921	6922,6923,6924,860,925,6925,6926,5200,6927,1601,6928,5189,6929,6930,6931,929,6704,4900,1606,944,5205,6932,6933,6934,6935,6936,865,6937,6938,6939,6940,6941,6942,6943,6944,994,907,6945,6946,6947,6948,6949,6950,6951,1593	[[0,1,1,1,0,0,0,0,1],[0,0,1,1,1,0,0,0,1],[0,0,1,1,0,0,0,0,1],[0,0,1,0,0,0,0,0,0],[0,1,1,1,0,0,0,0,1],[0,0,1,0,0,0,0,0,0],[0,0,0,0,1,0,0,0,0],[0,0,1,0,0,0,0,0,0],[0,0,1,0,0,0,0,0,0],[0,0,1,1,0,0,0,0,0],[0,1,1,1,0,0,0,0,1],[0,0,1,0,0,0,0,0,0],[0,0,0,0,1,0,0,0,0],[0,0,1,1,0,0,0,0,0],[0,0,1,0,0,0,0,0,0],[0,0,1,0,0,0,0,0,0],[0,0,1,1,0,0,0,0,0],[0,0,1,1,0,0,0,0,0],[0,0,1,1,0,0,0,0,1],[0,0,0,1,0,0,0,0,1],[0,0,1,0,0,0,0,0,0],[0,0,0,0,1,0,0,0,0],[0,0,1,0,0,0,0,0,0],[0,0,1,0,0,0,0,0,0],[0,0,0,0,0,0,0,0,1],[0,0,1,0,0,0,0,0,0],[0,0,1,1,0,0,0,0,1],[0,1,1,1,1,0,0,0,1],[0,0,1,0,1,0,0,0,0],[0,0,1,1,0,0,0,0,0],[0,1,1,1,0,0,0,0,1],[0,0,0,1,0,0,0,0,0],[0,0,1,0,0,0,0,0,0],[0,0,1,0,0,0,0,0,0],[0,0,1,1,0,0,0,0,1],[0,0,1,0,0,0,0,0,0],[0,0,1,0,0,0,0,0,0],[0,0,1,1,0,0,0,0,0],[0,0,1,0,0,0,0,0,0],[0,0,1,0,0,0,0,0,0],[0,0,0,1,0,0,0,0,0],[0,0,0,1,0,0,0,0,0],[0,0,1,0,0,0,0,0,0],[0,0,0,0,1,0,0,0,0],[0,0,1,0,0,0,0,0,1]]6952	949	[[0,0,0,0,1,0,0,0,0]]6953	4615,6906,6904	[[0,0,0,1,0,0,0,0,0],[0,0,0,1,0,0,0,0,0],[0,0,0,1,0,0,0,0,0]]6954	6955,6956	[[0,0,0,0,1,0,0,0,0],[0,0,0,0,1,0,0,0,0]]6957	6958	[[0,0,0,0,1,0,0,0,0]]6959	6960,6961,6962	[[0,0,0,0,1,0,0,0,0],[0,0,0,0,1,0,0,0,0],[0,0,0,0,1,0,0,0,0]]6963	3023,5462	[[0,0,0,0,1,0,0,0,0],[0,0,0,0,1,0,0,0,0]]6964	6965,6966,6967,6968,5810,6969,6970,6971,6972,6973,6974,6975,6976,6977,6978,6979,6980,6981,6982,6983	[[0,0,0,1,0,0,0,0,0],[0,0,0,1,0,0,0,0,0],[0,0,0,1,0,0,0,0,0],[0,0,0,1,0,0,0,0,0],[0,0,0,1,0,0,0,0,0],[0,0,0,1,0,0,0,0,0],[0,0,0,1,0,0,0,0,0],[0,0,0,1,0,0,0,0,0],[0,0,0,1,0,0,0,0,0],[0,0,0,1,0,0,0,0,0],[0,0,0,1,0,0,0,0,0],[0,0,0,1,0,0,0,0,0],[0,0,0,1,0,0,0,0,0],[0,0,0,1,0,0,0,0,0],[0,0,0,1,0,0,0,0,0],[0,0,0,1,0,0,0,0,0],[0,0,0,1,0,0,0,0,0],[0,0,0,1,0,0,0,0,0],[0,0,0,1,0,0,0,0,0],[0,0,0,1,0,0,0,0,0]]6984	6985	[[0,0,0,0,1,0,0,0,0]]6986	2206,6987,6988,2220	[[0,0,0,0,1,0,0,0,0],[0,0,0,0,1,0,0,0,0],[0,0,0,0,1,0,0,0,0],[0,0,0,0,1,0,0,0,0]]6989	6990,6991,6992,6993	[[0,0,0,0,1,0,0,0,0],[0,0,0,0,1,0,0,0,0],[0,0,0,0,1,0,0,0,0],[0,0,0,0,1,0,0,0,0]]6994	6995	[[0,0,0,0,1,0,0,0,0]]6996	6997,6998,3593,5713,4213,6999,7000,7001,7002,4238,3694,7003,482,7004,7005,2429,7006,7007,7008,3686,7009,5352,7010,7011,7012,7013,7014,7015,3941,1631,1329,7016,7017,4879,7018,4258,2786,7019,4261,7020,7021,2268,7022,4538,650,7023,7024,4536,7025,7026,798,7027,7028,7029,7030,7031,7032,7033,686	[[0,1,1,1,0,0,1,1,0],[1,1,0,0,0,0,1,0,0],[0,0,1,0,0,0,0,0,0],[0,1,1,1,1,0,1,1,0],[0,0,1,0,0,0,0,0,0],[0,0,1,0,0,0,0,1,0],[1,0,0,0,1,0,0,0,0],[0,0,1,1,0,0,0,1,0],[0,0,1,1,0,0,0,1,0],[0,1,1,1,0,0,1,0,0],[1,1,1,1,1,0,0,1,0],[0,0,1,1,0,0,0,1,0],[0,1,0,1,0,0,1,1,0],[0,0,0,0,0,0,0,1,0],[0,0,0,0,1,0,0,0,0],[1,0,0,1,0,0,0,1,0],[0,0,1,1,0,0,0,1,0],[0,0,1,1,0,0,0,1,0],[1,1,0,1,0,0,1,1,0],[0,0,1,1,0,0,0,1,0],[1,0,0,0,0,0,0,0,0],[1,0,0,0,0,0,0,0,0],[1,0,0,0,0,0,0,0,0],[1,0,0,0,0,0,0,0,0],[1,0,0,0,0,0,0,0,0],[1,1,0,1,0,0,1,1,0],[1,1,0,1,0,0,1,1,0],[0,1,0,0,0,0,0,0,0],[0,1,1,0,0,0,1,1,0],[1,0,0,0,0,0,0,0,0],[0,0,1,0,0,0,0,0,0],[0,1,1,1,0,0,0,0,0],[0,1,0,1,0,0,1,1,0],[0,0,0,0,1,0,0,0,0],[0,0,1,0,0,0,0,1,0],[0,0,0,0,1,0,0,0,0],[1,0,0,0,0,0,0,0,0],[1,0,0,0,0,0,0,0,0],[1,0,0,0,0,0,0,0,0],[1,0,0,0,0,0,0,0,0],[1,0,0,0,0,0,1,1,0],[0,0,0,1,0,0,0,1,0],[1,0,1,1,0,0,0,0,0],[0,0,1,0,0,0,0,0,0],[0,0,1,0,0,0,0,0,0],[0,0,1,0,0,0,0,1,0],[0,0,1,1,0,0,0,1,0],[0,0,0,0,1,0,0,0,0],[1,0,1,0,0,0,0,0,0],[0,0,1,0,0,0,0,0,0],[1,0,0,0,0,0,0,0,0],[1,0,0,0,0,0,0,0,0],[1,0,0,0,0,0,0,0,0],[1,0,0,0,0,0,0,0,0],[1,0,0,0,0,0,0,0,0],[1,0,0,0,0,0,0,0,0],[1,0,0,0,0,0,0,0,0],[1,0,0,0,0,0,0,0,0],[0,0,1,0,0,0,0,1,0]]7034	7035,7036,7037,7038	[[0,0,0,1,0,0,0,0,0],[0,0,0,1,0,0,0,0,0],[0,0,0,1,0,0,0,0,0],[0,0,0,0,1,0,0,0,0]]7039	7040	[[0,0,0,0,1,0,0,0,0]]7041	7042,2554,7043,7044,7045,7046,7047,7048,7049,7050,7051,7052,87,7053,7054,7055,7056,7057,7058,7059,7060,7061,1305,7062,7063,7064,7065,7066,7067,7068,7069,7070,7071,7072,5367,7073,7074,7075	[[0,0,1,0,0,0,0,0,0],[0,0,1,0,0,0,0,0,0],[0,0,1,0,0,0,0,0,0],[0,0,1,0,0,0,0,0,0],[0,0,1,1,0,0,0,0,0],[0,0,1,0,0,0,0,0,0],[0,0,0,0,1,0,0,0,0],[0,0,1,0,0,0,0,0,0],[0,0,1,0,0,0,0,0,0],[0,0,1,1,0,0,0,0,0],[0,0,1,0,0,0,0,0,0],[0,0,1,0,0,0,0,0,0],[0,0,0,1,0,0,0,0,0],[0,0,1,0,0,0,0,0,0],[0,0,1,0,0,0,0,0,0],[0,0,1,0,0,0,0,0,0],[0,0,1,0,0,0,0,0,0],[0,0,0,0,1,0,0,0,0],[0,0,0,0,1,0,0,0,0],[0,0,1,1,0,0,0,0,0],[0,0,1,0,0,0,0,0,0],[0,0,1,1,0,0,0,0,0],[0,0,1,0,0,0,0,0,0],[0,0,1,1,0,0,0,0,0],[0,0,0,1,0,0,0,0,0],[0,0,1,1,0,0,0,0,0],[0,0,1,0,0,0,0,0,0],[0,0,1,0,0,0,0,0,0],[0,0,1,0,0,0,0,0,0],[0,0,1,0,0,0,0,0,0],[0,0,1,0,0,0,0,0,0],[0,0,1,0,0,0,0,0,0],[0,0,1,0,0,0,0,0,0],[0,0,1,1,0,0,0,0,0],[0,0,1,0,0,0,0,0,0],[0,0,1,0,0,0,0,0,0],[0,0,1,1,0,0,0,0,0],[0,0,1,0,1,0,0,0,0]]7076	7077	[[0,0,0,0,1,0,0,0,0]]7078	7079,7080	[[0,0,0,0,1,0,0,0,0],[0,0,0,0,1,0,0,0,0]]7081	7082	[[0,0,0,0,1,0,0,0,0]]7083	4539,7084,6603,7085,7086,7087,7088,7089	[[0,1,0,0,1,0,0,0,0],[0,1,0,1,0,0,0,0,0],[0,1,0,0,0,0,0,0,0],[0,1,0,0,0,0,0,0,0],[0,1,0,0,0,0,0,0,0],[0,1,0,1,0,0,0,0,0],[0,0,0,1,1,0,0,0,0],[0,0,0,1,0,0,0,0,0]]7090	3143,7091,7092,7093,3416,7094,7095,7096,7097,2137,7098,4916,3093,7099,7100,7101,815,7102,7103,7104,1566,5474,614,7105,6168,7106,7107,7108,3932,7109,494,3129,6165,7110,7111,7112,7113,5452,7061,5164,7114,7115,7116,7117,2501,6175,1140,7118,7119,4391,1143,7120,7121,6170,7122,2432,7123,2827,7124,7125,7126,7127,3119,7128,7129,3107,7130,7131,3118,7132,7133,7134,7135,3137	[[0,0,1,0,0,0,0,0,0],[1,1,1,1,0,0,0,1,0],[0,0,1,0,0,0,0,0,0],[0,0,1,0,0,0,0,0,0],[1,1,0,1,0,0,1,1,0],[0,1,0,1,0,0,0,0,0],[1,0,0,1,0,0,0,1,0],[1,1,1,1,0,0,0,1,0],[1,1,0,1,0,0,0,1,0],[1,1,1,1,1,0,1,1,0],[0,0,1,0,0,0,0,0,0],[0,1,1,1,0,0,0,1,0],[0,1,0,0,0,0,1,0,0],[0,0,0,1,0,0,0,0,0],[0,0,1,0,0,0,0,0,0],[1,0,0,0,0,0,0,0,0],[0,0,0,1,0,0,0,1,0],[1,0,0,0,0,0,0,0,0],[1,0,0,0,0,0,0,0,0],[1,0,0,0,0,0,0,0,0],[1,0,0,0,0,0,0,0,0],[1,0,0,0,0,0,0,0,0],[1,0,0,0,0,0,0,0,0],[1,1,0,0,0,0,1,1,0],[0,1,1,1,1,0,1,1,0],[0,0,0,0,0,0,0,1,0],[0,0,0,1,0,0,0,1,0],[1,1,1,0,0,0,1,0,0],[1,1,1,1,0,0,1,1,0],[0,1,0,0,0,0,1,0,0],[0,1,1,0,0,0,1,0,0],[0,0,1,0,0,0,0,0,0],[0,1,0,0,0,0,1,0,0],[0,0,0,1,0,0,0,1,0],[0,0,1,0,0,0,0,0,0],[0,0,0,1,0,0,0,0,0],[0,1,0,0,0,0,1,0,0],[1,0,0,0,0,0,0,0,0],[0,0,1,1,0,0,0,0,0],[0,0,0,1,0,0,0,0,0],[0,1,0,0,0,0,1,0,0],[0,0,1,0,0,0,0,0,0],[0,0,1,0,0,0,0,0,0],[0,1,1,0,0,0,1,0,0],[0,0,0,1,0,0,0,1,0],[0,1,1,0,0,0,1,0,0],[0,0,1,0,0,0,0,0,0],[1,1,1,0,0,0,1,0,0],[0,0,0,0,0,0,0,1,0],[0,0,1,0,0,0,0,0,0],[0,0,1,0,0,0,0,0,0],[1,0,0,0,0,0,0,0,0],[1,0,1,1,0,0,0,1,0],[0,0,1,0,0,0,0,0,0],[0,1,0,1,0,0,0,1,0],[0,1,1,0,0,0,1,0,0],[0,0,1,0,0,0,0,0,0],[0,1,0,0,0,0,0,0,0],[1,1,0,0,0,0,1,1,0],[1,0,0,0,0,0,0,0,0],[1,0,0,0,0,0,0,0,0],[1,0,0,0,0,0,0,0,0],[0,0,1,0,0,0,0,0,0],[1,0,0,0,0,0,0,0,0],[0,0,1,1,0,0,0,1,0],[0,0,1,0,0,0,0,0,0],[0,0,0,0,0,0,0,1,0],[1,0,0,0,0,0,0,0,0],[0,0,1,0,0,0,0,0,0],[0,0,1,0,0,0,0,0,0],[1,0,0,0,0,0,0,0,0],[1,0,0,0,0,0,0,0,0],[1,0,0,0,0,0,0,1,0],[0,0,1,0,0,0,0,0,0]]7136	7137,7138,7139,7140	[[0,0,0,0,1,0,0,0,0],[0,0,0,0,1,0,0,0,0],[0,0,0,0,1,0,0,0,0],[0,0,0,0,1,0,0,0,0]]7141	4291,6670,7142,7143,7144,7145,6661,1839,7146,7147,7148,7149,7150,7151,7152,7153,6798	[[0,0,1,0,0,0,0,0,0],[0,0,1,0,0,0,0,0,0],[0,0,1,0,0,0,0,0,0],[0,0,1,0,0,0,0,0,0],[0,0,1,0,0,0,0,0,0],[0,0,1,0,0,0,0,0,0],[0,0,1,0,0,0,0,0,0],[0,0,1,0,0,0,0,0,0],[0,0,1,0,0,0,0,0,0],[0,0,1,0,0,0,0,0,0],[0,0,1,0,0,0,0,0,0],[0,0,0,0,1,0,0,0,0],[0,0,1,0,0,0,0,0,0],[0,0,1,0,0,0,0,0,0],[0,0,1,0,0,0,0,0,0],[0,0,1,0,0,0,0,0,0],[0,0,1,0,0,0,0,0,0]]7154	2943,7155,2935,2928,7156,7157,2942,7158,6160,6842,7159,7160,7161,5913,5909,7162,2941,2944,6268,5372,7163,7164,7165,7166,1164,1277,898,3136,7167,7168,7169,97,7170,1566,1767,7171,7172,7173,7174,7175,7176,2930,2947,1143,7177,7101,2926,5889	[[1,1,1,1,1,1,1,1,1],[0,0,1,0,0,0,0,0,0],[1,1,1,1,0,0,1,1,1],[0,0,1,0,0,0,0,1,0],[1,0,1,0,0,0,0,0,1],[0,0,1,0,0,0,0,0,0],[1,1,1,0,0,0,1,0,1],[0,1,1,1,0,0,0,1,1],[0,0,1,0,0,0,0,0,0],[0,1,1,0,0,0,0,0,0],[0,0,1,0,0,0,0,0,0],[0,0,1,0,0,0,0,0,0],[0,0,1,0,0,0,0,0,0],[0,0,0,0,0,0,0,1,0],[0,0,0,0,0,1,0,0,0],[1,0,1,0,0,0,0,0,0],[0,0,1,0,0,0,0,0,0],[1,0,1,0,0,0,0,0,0],[0,0,1,0,0,0,0,0,0],[1,1,0,0,0,1,1,1,1],[1,0,0,0,0,0,0,1,0],[1,0,0,0,0,0,0,0,0],[1,0,0,0,0,0,0,0,0],[1,0,0,0,0,0,0,1,0],[1,1,1,0,0,1,0,1,1],[1,0,0,0,0,0,0,0,0],[1,0,0,0,0,0,0,0,0],[0,0,1,0,0,0,0,0,0],[0,1,1,0,0,0,0,0,0],[1,0,1,0,0,0,0,0,0],[0,0,1,0,0,0,0,1,0],[1,0,1,0,0,0,0,0,0],[1,1,0,0,0,0,0,0,0],[1,0,0,0,0,0,0,0,0],[0,1,1,0,0,0,0,1,0],[0,0,1,0,0,0,0,0,0],[0,0,1,0,0,0,0,0,0],[1,0,1,0,0,0,0,1,0],[0,0,1,0,0,0,0,0,0],[1,0,0,0,0,0,0,0,0],[1,0,0,0,0,0,0,0,0],[0,0,1,0,0,0,0,0,0],[0,0,1,0,0,1,0,1,0],[0,1,1,0,0,0,0,0,0],[0,1,0,0,0,0,1,0,0],[0,0,0,0,0,1,0,0,0],[0,0,0,0,0,0,0,1,0],[0,0,1,0,0,0,0,0,0]]7178	1925,7179,7180,7181,7182,7183,7184,4989,7185,7186,7187,7188,7189,7190,7191,7192,7193,7194,7195	[[0,0,1,0,0,0,0,0,0],[0,0,1,0,0,0,0,0,0],[0,0,1,0,0,0,0,0,0],[0,0,1,0,0,0,0,0,0],[0,0,1,0,0,0,0,0,0],[0,0,1,1,1,0,1,0,0],[0,0,1,1,0,0,1,0,0],[0,0,1,0,0,0,1,0,0],[0,0,1,0,0,0,0,0,0],[0,0,1,1,0,0,1,0,0],[0,0,1,0,0,0,0,0,0],[0,0,1,0,0,0,0,0,0],[0,0,0,0,1,0,0,0,0],[0,0,1,0,0,0,0,0,0],[0,0,1,1,0,0,1,0,0],[0,0,0,1,0,0,0,0,0],[0,0,1,0,0,0,0,0,0],[0,0,1,1,0,0,1,0,0],[0,0,1,0,0,0,0,0,0]]7196	1562,1544	[[0,0,0,0,1,0,0,0,0],[0,0,0,0,1,0,0,0,0]]7197	6850,7198,7199,7200,7201,7202,7203,2247,3573,6781,7204,7205,7206,7207,7208,7209,7210,376,7211,7212,7213,7214,7215,824,7216,2257,7217,7218,7219,7220,7221,7222,5206,1250,7223,7224,4510,368,7225,7226,7227,7228,409,7229,7230,3024,826,7231,5196,7232,7233,7234,7235,7236,7237,7238,7239,7240,1255,7241,6847,1160,415,7242,7243,800,7244,7245,7246,7247,7248,1256,7249,7250,7251,7252,7253,7254,7255,379,7256,26,7257,7258,372,7259,396,7260,7261,7262,7263,6848,7264,7265,1172,6863,7266,1252,7267,7268,2250,7269,412,7270,7271,7272,5191,7273,7274,7275,7276,7277,7278,7279,7280,7281,7282	[[0,0,1,0,0,0,0,0,0],[0,0,1,0,0,0,0,0,0],[0,0,1,0,0,0,0,0,0],[0,0,1,0,0,0,0,0,0],[0,0,1,0,0,0,0,0,0],[0,0,1,0,0,0,0,0,0],[0,0,1,0,0,0,0,0,0],[0,0,1,0,0,0,0,0,0],[0,0,1,0,0,0,0,0,0],[0,0,1,0,0,0,0,0,0],[0,0,1,0,0,0,0,0,0],[0,0,1,0,0,0,0,0,0],[0,0,1,0,0,0,0,0,0],[0,0,1,0,0,0,0,0,0],[0,0,1,0,0,0,0,0,0],[0,0,1,0,0,0,0,0,0],[0,0,1,0,1,0,0,0,0],[0,0,1,0,0,0,0,0,0],[0,0,1,0,0,0,0,0,0],[0,0,1,0,0,0,0,0,0],[0,0,1,0,0,0,0,0,0],[0,0,1,0,0,0,0,0,0],[0,0,1,0,0,0,0,0,0],[0,0,1,0,0,0,0,0,0],[0,0,1,0,0,0,0,0,0],[0,0,1,0,0,0,0,0,0],[0,0,1,0,0,0,0,0,0],[0,0,1,0,0,0,0,0,0],[0,0,1,0,0,0,0,0,0],[0,0,1,0,0,0,0,0,0],[0,0,1,0,0,0,0,0,0],[0,0,1,0,0,0,0,0,0],[0,0,1,0,0,0,0,0,0],[0,0,1,0,0,0,0,0,0],[0,0,1,0,1,0,0,0,0],[0,0,1,0,0,0,0,0,0],[0,0,1,0,0,0,0,0,0],[0,0,1,0,0,0,0,0,0],[0,0,1,0,0,0,0,0,0],[0,0,1,0,0,0,0,0,0],[0,0,1,0,0,0,0,0,0],[0,0,1,0,0,0,0,0,0],[0,0,1,0,0,0,0,0,0],[0,0,1,0,0,0,0,0,0],[0,0,1,0,0,0,0,0,0],[0,0,1,0,0,0,0,0,0],[0,0,1,0,0,0,0,0,0],[0,0,1,0,0,0,0,0,0],[0,0,1,1,0,0,0,0,0],[0,0,1,0,0,0,0,0,0],[0,0,1,0,0,0,0,0,0],[0,0,1,0,0,0,0,0,0],[0,0,1,1,0,0,0,0,0],[0,0,1,0,0,0,0,0,0],[0,0,1,0,0,0,0,0,0],[0,0,1,0,0,0,0,0,0],[0,0,1,0,0,0,0,0,0],[0,0,1,0,0,0,0,0,0],[0,0,1,0,0,0,0,0,0],[0,0,1,0,0,0,0,0,0],[0,0,1,0,0,0,0,0,0],[0,0,1,0,0,0,0,0,0],[0,0,1,0,0,0,0,0,0],[0,0,1,0,0,0,0,0,0],[0,0,1,1,0,0,0,0,0],[0,0,1,0,0,0,0,0,0],[0,0,1,0,0,0,0,0,0],[0,0,1,0,0,0,0,0,0],[0,0,1,0,0,0,0,0,0],[0,0,1,0,0,0,0,0,0],[0,0,1,1,0,0,0,0,0],[0,0,1,0,0,0,0,0,0],[0,0,1,0,0,0,0,0,0],[0,0,1,1,0,0,0,0,0],[0,0,1,0,0,0,0,0,0],[0,0,1,0,0,0,0,0,0],[0,0,1,0,0,0,0,0,0],[0,0,1,0,0,0,0,0,0],[0,0,1,0,0,0,0,0,0],[0,0,1,0,0,0,0,0,0],[0,0,1,0,0,0,0,0,0],[0,0,1,0,0,0,0,0,0],[0,0,1,0,0,0,0,0,0],[0,0,1,0,0,0,0,0,0],[0,0,1,0,0,0,0,0,0],[0,0,1,0,0,0,0,0,0],[0,0,1,0,0,0,0,0,0],[0,0,1,0,0,0,0,0,0],[0,0,1,0,0,0,0,0,0],[0,0,1,0,0,0,0,0,0],[0,0,1,0,0,0,0,0,0],[0,0,1,0,0,0,0,0,0],[0,0,1,0,0,0,0,0,0],[0,0,1,0,0,0,0,0,0],[0,0,1,0,0,0,0,0,0],[0,0,1,0,0,0,0,0,0],[0,0,1,0,0,0,0,0,0],[0,0,1,1,1,0,0,0,0],[0,0,1,0,0,0,0,0,0],[0,0,1,0,0,0,0,0,0],[0,0,1,0,0,0,0,0,0],[0,0,1,0,0,0,0,0,0],[0,0,1,0,0,0,0,0,0],[0,0,1,0,0,0,0,0,0],[0,0,1,0,0,0,0,0,0],[0,0,1,0,0,0,0,0,0],[0,0,1,0,0,0,0,0,0],[0,0,1,0,0,0,0,0,0],[0,0,1,0,0,0,0,0,0],[0,0,1,0,0,0,0,0,0],[0,0,1,0,0,0,0,0,0],[0,0,1,0,0,0,0,0,0],[0,0,1,0,0,0,0,0,0],[0,0,1,0,0,0,0,0,0],[0,0,1,0,0,0,0,0,0],[0,0,1,0,0,0,0,0,0],[0,0,1,0,0,0,0,0,0]]7283	7284,7285	[[0,0,0,0,1,0,0,0,0],[0,0,0,0,1,0,0,0,0]]7286	7287,5790,5912,7288,7289,7290,7291,7292,7293,7294,7295,7296,7297,7298,7299,2723,7300,7301,7302,7303,7304,7305,7306,7307,7308,7309,2706,7310,7311,7312,7313,7314,3122,7315,7316,2721,7317,7318,7319	[[0,0,1,0,0,0,0,0,0],[0,0,0,1,0,0,0,0,0],[0,0,0,1,0,0,0,0,0],[0,1,1,0,0,0,0,0,0],[0,1,1,0,0,0,0,0,0],[0,0,1,0,0,0,0,0,0],[0,0,1,0,0,0,0,0,0],[0,0,1,0,0,0,0,0,0],[0,0,1,0,0,0,0,0,0],[0,1,1,0,0,0,0,0,0],[0,0,1,0,0,0,0,0,0],[0,0,1,0,0,0,0,0,0],[0,1,1,0,0,0,0,0,0],[0,0,1,0,0,0,0,0,0],[0,0,1,0,0,0,0,0,0],[0,0,1,0,0,0,0,0,0],[0,0,1,0,0,0,0,0,0],[0,0,1,0,0,0,0,0,0],[0,0,1,0,0,0,0,0,0],[0,0,1,0,0,0,0,0,0],[0,0,1,0,0,0,0,0,0],[0,1,0,0,0,0,0,0,0],[0,0,1,0,0,0,0,0,0],[0,1,0,0,0,0,0,0,0],[0,0,1,0,0,0,0,0,0],[0,0,1,0,0,0,0,0,0],[0,1,1,0,0,0,0,0,0],[0,0,1,0,0,0,0,0,0],[0,0,1,0,0,0,0,0,0],[0,0,1,0,0,0,0,0,0],[0,0,1,0,0,0,0,0,0],[0,1,0,0,0,0,0,0,0],[0,0,1,0,0,0,0,0,0],[0,0,1,0,0,0,0,0,0],[0,1,0,0,0,0,0,0,0],[0,1,0,0,0,0,0,0,0],[0,1,0,0,0,0,0,0,0],[0,1,0,0,0,0,0,0,0],[0,0,0,1,0,0,0,0,0]]7320	7321,7322,7323,7324,7325,7326,7327,7328,7329,7330,7331,7332,7333,7334,7335,6317,7336,7337,7338,7339,2428,7340,7341,944,1563,7342,7343,7344,3774,1607,704,1633,7345,1632,7346	[[1,1,1,1,0,0,1,1,0],[0,0,1,1,0,0,0,1,0],[1,0,1,1,0,0,0,1,0],[1,0,0,1,0,0,0,0,0],[1,0,0,1,0,0,0,0,0],[1,0,0,1,0,0,0,0,0],[1,0,0,0,0,0,0,1,0],[1,1,1,1,0,0,0,0,0],[0,1,0,1,0,0,1,1,0],[0,1,1,1,0,0,1,0,0],[1,0,0,0,1,0,0,0,0],[0,1,1,0,0,0,1,0,0],[0,1,1,0,0,0,0,0,0],[0,0,1,0,0,0,0,0,0],[0,1,0,0,0,0,0,0,0],[0,1,0,0,0,0,1,0,0],[0,1,1,1,0,0,1,1,0],[0,0,0,1,0,0,0,0,0],[0,1,1,0,0,0,1,0,0],[0,0,0,0,0,0,0,1,0],[0,0,0,0,0,0,0,1,0],[0,1,1,1,0,0,0,1,0],[0,0,1,0,0,0,0,1,0],[0,0,1,0,0,0,0,0,0],[0,0,0,1,0,0,0,0,0],[0,0,1,0,0,0,0,0,0],[0,0,0,0,0,0,0,1,0],[0,0,0,1,0,0,0,0,0],[0,0,0,1,0,0,0,0,0],[0,0,1,0,0,0,0,0,0],[0,0,0,1,0,0,0,0,0],[0,0,1,0,0,0,0,0,0],[0,0,1,0,0,0,0,0,0],[0,0,1,0,0,0,0,0,0],[0,0,1,0,0,0,0,0,0]]7347	7348	[[0,0,0,0,1,0,0,0,0]]7349	7350,7351	[[0,0,0,0,1,0,0,0,0],[0,0,0,0,1,0,0,0,0]]7352	5076,7353,7354,7355,7356,7357,65,2310,2329,7358,7359,7360,7361,7362,3984,7363,7364,7365,3323,3047,7366,7367,7368,3932,2353,7369,2341,6934,2340,6363,2299,7108,2295,7370,7371,7372,1372,7135,7373,7374,7375,7061,7376,2283,5034,7377,7378,7379,1512,7380,2296,7381,7382,2137,7383,5066,7384,7385,7386,7387	[[0,0,1,1,0,0,0,0,0],[0,0,1,0,0,0,0,0,0],[0,0,0,0,1,0,0,0,0],[0,0,1,0,0,0,0,0,0],[0,0,0,1,0,0,0,0,0],[0,1,1,1,0,0,1,0,0],[0,1,1,1,1,0,1,0,0],[0,1,1,0,0,0,0,0,0],[0,0,0,1,0,0,0,0,0],[0,0,1,0,0,0,0,0,0],[0,1,1,1,0,0,1,0,0],[0,1,0,1,0,0,0,0,0],[0,0,1,0,0,0,0,0,0],[0,0,0,1,0,0,0,0,0],[0,1,1,0,0,0,0,0,0],[0,0,1,0,0,0,0,0,0],[0,0,1,0,0,0,0,0,0],[0,0,0,1,0,0,0,0,0],[0,0,0,1,0,0,0,0,0],[0,0,1,0,0,0,0,0,0],[0,0,1,0,0,0,0,0,0],[0,1,1,0,0,0,0,0,0],[0,0,1,0,0,0,0,0,0],[0,0,1,0,0,0,0,0,0],[0,1,1,1,0,0,0,0,0],[0,0,0,1,0,0,0,0,0],[0,0,1,0,0,0,0,0,0],[0,1,1,0,0,0,0,0,0],[0,0,1,0,0,0,0,0,0],[0,0,1,0,0,0,0,0,0],[0,0,0,1,0,0,0,0,0],[0,0,1,0,0,0,0,0,0],[0,0,1,0,0,0,0,0,0],[0,1,0,0,0,0,0,0,0],[0,1,0,0,0,0,0,0,0],[0,0,1,0,0,0,0,0,0],[0,1,0,0,0,0,0,0,0],[0,0,0,0,1,0,0,0,0],[0,1,0,0,0,0,0,0,0],[0,0,1,0,0,0,0,0,0],[0,0,1,0,0,0,0,0,0],[0,0,1,0,0,0,0,0,0],[0,0,0,1,0,0,0,0,0],[0,0,1,0,0,0,0,0,0],[0,0,0,1,0,0,0,0,0],[0,1,1,1,0,0,0,0,0],[0,0,0,1,0,0,0,0,0],[0,0,1,0,0,0,0,0,0],[0,1,1,0,0,0,0,0,0],[0,0,0,1,0,0,0,0,0],[0,0,0,1,0,0,0,0,0],[0,0,1,0,0,0,0,0,0],[0,0,0,1,0,0,0,0,0],[0,0,1,0,1,0,0,0,0],[0,0,1,0,0,0,0,0,0],[0,0,1,0,0,0,0,0,0],[0,1,1,0,0,0,0,0,0],[0,0,0,1,0,0,0,0,0],[0,1,0,0,0,0,0,0,0],[0,0,0,1,0,0,0,0,0]]7388	876,875,874,852,883,884,881,864,853,928,851,837,838,841,892,891,945,889,847,7389,908,929,873,839,840,879,888,890,936,916,880,855,882,901,902,7390,866,903,904,905,907,938,937,895,933,913,900,899,878,914,915,863,935,917,918,919,856,939,927,920,857,924,911,894,886,844,859,854,861,912,940,922,906,869,898,897,909,910,867,942,870,845,887,877,872,868,849,871,943,858,941,946,896,934,931,930,923,848,850,932,7391	[[0,0,1,0,0,0,0,0,0],[0,0,1,0,0,0,0,0,0],[0,0,1,0,0,0,0,0,0],[0,0,1,0,0,0,0,0,0],[0,0,1,0,0,0,0,0,0],[0,0,1,0,0,0,0,0,0],[0,0,1,0,0,0,0,0,0],[0,1,1,0,0,0,0,0,0],[0,0,1,0,0,0,0,0,0],[0,0,1,0,0,0,0,0,0],[0,0,1,0,0,0,0,0,0],[0,0,1,0,0,0,0,0,0],[0,0,1,0,0,0,0,0,0],[0,0,1,0,0,0,0,0,0],[0,0,1,0,0,0,0,0,0],[0,0,1,0,0,0,0,0,0],[0,0,1,0,0,0,0,0,0],[0,0,1,0,0,0,0,0,0],[0,1,1,0,0,0,0,0,1],[0,1,1,0,1,0,0,0,1],[0,1,1,1,1,0,0,0,1],[0,0,1,0,0,0,0,0,0],[0,0,1,0,0,0,0,0,0],[0,0,1,0,0,0,0,0,0],[0,0,1,0,0,0,0,0,0],[0,0,1,0,0,0,0,0,0],[0,0,1,0,0,0,0,0,0],[0,0,1,0,0,0,0,0,0],[0,0,1,0,0,0,0,0,0],[0,0,1,0,0,0,0,0,0],[0,0,1,0,0,0,0,0,0],[0,0,1,0,0,0,0,0,0],[0,0,1,0,0,0,0,0,0],[0,0,1,0,0,0,0,0,0],[0,0,1,0,0,0,0,0,0],[0,0,1,0,0,0,0,0,0],[0,1,1,0,0,0,0,0,0],[0,0,1,0,0,0,0,0,0],[0,0,1,0,0,0,0,0,0],[0,0,1,0,0,0,0,0,0],[0,0,1,0,0,0,0,0,0],[0,0,1,0,0,0,0,0,0],[0,0,1,0,0,0,0,0,0],[0,1,0,1,0,0,0,0,0],[0,0,1,0,0,0,0,0,0],[0,0,1,0,0,0,0,0,0],[0,0,1,0,0,0,0,0,0],[0,0,1,0,0,0,0,0,0],[0,0,1,0,0,0,0,0,0],[0,0,1,0,0,0,0,0,0],[0,0,1,0,0,0,0,0,0],[0,0,1,0,0,0,0,0,0],[0,0,1,0,0,0,0,0,0],[0,0,1,0,0,0,0,0,0],[0,0,1,0,0,0,0,0,0],[0,0,1,0,0,0,0,0,0],[0,0,1,0,0,0,0,0,0],[0,0,1,0,0,0,0,0,0],[0,0,1,0,0,0,0,0,0],[0,0,1,0,0,0,0,0,0],[0,1,1,0,0,0,0,0,0],[0,0,1,0,0,0,0,0,0],[0,0,1,0,0,0,0,0,0],[0,0,1,0,0,0,0,0,0],[0,0,1,0,0,0,0,0,0],[0,0,1,0,1,0,0,0,1],[0,0,1,0,0,0,0,0,0],[0,0,1,0,0,0,0,0,0],[0,0,1,0,0,0,0,0,0],[0,1,1,1,0,0,0,0,1],[0,0,1,0,0,0,0,0,0],[0,0,1,0,0,0,0,0,0],[0,0,1,0,0,0,0,0,0],[0,0,1,0,0,0,0,0,1],[0,0,1,0,0,0,0,0,0],[0,0,1,0,0,0,0,0,0],[0,0,1,0,0,0,0,0,0],[0,0,1,0,0,0,0,0,0],[0,1,1,0,1,0,0,0,1],[0,0,1,0,0,0,0,0,0],[0,0,1,0,0,0,0,0,0],[0,0,1,0,0,0,0,0,0],[0,0,1,0,0,0,0,0,0],[0,0,1,0,0,0,0,0,0],[0,0,1,0,0,0,0,0,0],[0,0,1,0,0,0,0,0,0],[0,1,1,0,0,0,0,0,0],[0,0,1,0,0,0,0,0,0],[0,0,1,0,0,0,0,0,0],[0,0,1,0,0,0,0,0,0],[0,0,1,0,0,0,0,0,0],[0,0,1,0,0,0,0,0,0],[0,0,1,0,0,0,0,0,0],[0,0,1,0,0,0,0,0,0],[0,0,1,0,0,0,0,0,0],[0,0,1,0,0,0,0,0,0],[0,0,1,0,0,0,0,0,0],[0,0,1,0,0,0,0,0,0],[0,0,1,0,0,0,0,0,0],[0,0,1,0,0,0,0,0,0],[0,0,1,0,0,0,0,0,0]]7392	7393,7394,7395,7396,7397,7398,7399,7400,7401,7402,7403	[[0,0,0,1,0,0,0,0,0],[0,0,0,1,0,0,0,0,0],[0,0,0,0,1,0,0,0,0],[0,0,0,1,0,0,0,0,0],[0,0,0,1,0,0,0,0,0],[0,0,0,1,0,0,0,0,0],[0,0,0,1,0,0,0,0,0],[0,0,0,1,0,0,0,0,0],[0,0,0,1,0,0,0,0,0],[0,0,0,1,0,0,0,0,0],[0,0,0,1,0,0,0,0,0]]7404	7405	[[0,0,0,0,1,0,0,0,0]]7406	7407,7408,7409,7410,7411,7412,7413,7414,7415,7416,7417	[[0,1,1,1,0,0,0,0,0],[0,0,1,1,0,0,0,0,0],[0,1,0,1,0,0,0,0,0],[0,0,0,1,0,0,0,0,0],[0,0,1,1,0,0,0,0,0],[0,0,0,1,0,0,0,0,0],[0,1,1,1,0,0,0,0,0],[0,0,0,1,0,0,0,0,0],[0,0,1,1,0,0,0,0,0],[0,0,0,1,0,0,0,0,0],[0,1,0,0,0,0,0,0,0]]7418	513,3155,7419,7420,4956,7421,7422,7423	[[0,0,0,1,0,0,0,0,0],[0,0,0,1,0,0,0,0,0],[0,0,0,1,0,0,0,0,0],[0,0,0,1,0,0,0,0,0],[0,0,0,1,0,0,0,0,0],[0,0,0,1,0,0,0,0,0],[0,0,0,1,0,0,0,0,0],[0,0,0,1,0,0,0,0,0]]7424	139,7425,7426,7427,7428,7429,154,144,4626,7430,7431,135,7432,6387,3199,7433,7434,7435,7436,7437,7438,7439,7440,150,6362,6135,722,7441,7442,6388,6271	[[0,0,1,0,0,0,0,0,0],[0,0,1,1,0,0,0,0,0],[0,0,1,0,0,0,0,0,0],[0,0,0,1,0,0,0,0,0],[0,0,1,0,0,0,0,0,0],[0,0,1,1,0,0,0,0,0],[0,0,1,0,0,0,0,0,0],[0,0,1,0,0,0,0,0,0],[0,0,1,0,0,0,0,0,0],[0,0,1,0,0,0,0,0,0],[0,0,1,0,0,0,0,0,0],[0,0,1,0,0,0,0,0,0],[0,0,1,0,0,0,0,0,0],[0,0,1,0,0,0,0,0,0],[0,0,1,0,0,0,0,0,0],[0,0,1,0,0,0,0,0,0],[0,0,1,0,0,0,0,0,0],[0,0,1,0,0,0,0,0,0],[0,0,1,0,0,0,0,0,0],[0,0,1,0,0,0,0,0,0],[0,0,1,0,0,0,0,0,0],[0,0,1,0,0,0,0,0,0],[0,0,1,0,0,0,0,0,0],[0,0,1,0,0,0,0,0,0],[0,0,1,0,0,0,0,0,0],[0,0,1,0,0,0,0,0,0],[0,0,1,0,0,0,0,0,0],[0,0,1,0,0,0,0,0,0],[0,0,1,0,0,0,0,0,0],[0,0,1,0,0,0,0,0,0],[0,0,1,0,0,0,0,0,0]]7443	7444	[[0,0,0,0,1,0,0,0,0]]7445	4495,6502,6530,6464,6510,1701,7446,6525,6526,7447	[[0,1,0,0,1,0,0,0,0],[0,1,0,0,1,0,0,0,0],[0,1,0,0,1,0,0,0,0],[0,1,0,0,0,0,0,0,0],[0,1,0,0,0,0,0,0,0],[0,1,0,0,0,0,0,0,0],[0,1,0,0,0,0,0,0,0],[0,1,0,0,0,0,0,0,0],[0,1,0,0,0,0,0,0,0],[0,1,0,0,0,0,0,0,0]]7448	7449	[[0,0,0,0,1,0,0,0,0]]7450	7451,7452,7453	[[0,0,0,0,1,0,0,0,0],[0,0,0,0,1,0,0,0,0],[0,0,0,0,1,0,0,0,0]]7454	7455,7456,7457,7458,7459,7460	[[0,0,0,1,0,0,0,0,0],[0,0,0,1,0,0,0,0,0],[0,0,0,1,0,0,0,0,0],[0,0,0,1,0,0,0,0,0],[0,0,0,1,0,0,0,0,0],[0,0,0,1,0,0,0,0,0]]7461	4962,4956,5331,7462,7463,7464,7465,7420,7466,4951,513,3155,4958,7467,3401,7422,7468,7469,7470,7471,7472,7473,7421,441,7474,7475,7476,4957,1261,7477,7478,7337,3993,7479,7480,4967,7481,7482,7483,7484,7485,7486,7487,6647,4952,7488,7489,6644,4961,7419,5329,6642,4595,7490,7491,7492,4960,7493,7494,7495,4953,7496,7497,7423,7498,7499,7500	[[0,1,1,0,0,0,0,1,0],[0,1,1,1,0,0,1,1,0],[0,0,0,0,1,0,0,0,0],[0,0,1,0,0,0,0,0,0],[0,0,1,0,0,0,0,0,0],[0,0,1,0,0,0,0,0,0],[0,0,1,0,0,0,0,0,0],[0,0,0,1,0,0,0,0,0],[0,0,1,0,0,0,0,0,0],[0,1,1,0,0,0,0,1,0],[0,1,1,1,0,0,1,1,0],[0,1,1,1,0,0,1,1,0],[0,1,0,0,0,0,0,1,0],[0,1,0,0,0,0,1,1,0],[0,1,1,0,0,0,1,0,0],[0,1,1,1,0,0,1,0,0],[0,1,1,0,0,0,1,1,0],[0,1,1,0,0,0,1,0,0],[0,1,1,0,0,0,1,1,0],[0,0,0,0,0,0,0,1,0],[0,0,1,0,0,0,0,0,0],[0,0,1,0,0,0,0,0,0],[0,0,0,1,0,0,0,0,0],[0,0,1,0,0,0,0,0,0],[0,0,1,0,0,0,0,0,0],[0,0,1,0,0,0,0,0,0],[0,0,1,0,0,0,0,0,0],[0,0,0,0,0,0,0,1,0],[0,0,1,0,0,0,0,0,0],[0,0,1,0,0,0,0,0,0],[0,0,1,0,0,0,0,0,0],[0,0,1,0,0,0,0,1,0],[0,0,1,0,0,0,0,0,0],[0,0,1,0,0,0,0,0,0],[0,0,1,0,0,0,0,0,0],[0,0,1,0,0,0,0,0,0],[0,0,0,0,0,0,0,1,0],[0,0,0,0,0,0,1,0,0],[0,0,1,0,0,0,0,0,0],[0,0,1,0,0,0,0,0,0],[0,0,1,0,0,0,0,0,0],[0,0,1,0,0,0,0,0,0],[0,0,0,0,0,0,0,1,0],[0,0,1,0,0,0,0,1,0],[0,0,0,0,0,0,0,1,0],[0,0,1,0,0,0,0,0,0],[0,0,1,0,0,0,0,1,0],[0,0,0,0,0,0,1,0,0],[0,0,1,0,0,0,0,0,0],[0,0,0,1,0,0,0,1,0],[0,0,1,0,0,0,0,0,0],[0,0,1,0,0,0,0,1,0],[0,0,1,0,0,0,0,0,0],[0,0,1,0,0,0,0,0,0],[0,0,0,0,0,0,0,1,0],[0,0,1,0,1,0,0,0,0],[0,0,1,0,0,0,0,1,0],[0,0,0,0,0,0,1,0,0],[0,0,1,0,0,0,0,0,0],[0,0,1,0,0,0,0,0,0],[0,0,1,0,0,0,0,0,0],[0,0,1,0,0,0,0,0,0],[0,1,0,0,0,0,1,1,0],[0,0,0,1,0,0,0,1,0],[0,0,1,0,0,0,0,0,0],[0,0,1,0,0,0,0,0,0],[0,0,1,0,0,0,0,0,0]]7501	7502,7503,7504,7505,7506,7507,7508,944	[[0,0,1,0,0,0,0,0,0],[0,0,1,0,0,0,0,0,0],[0,0,1,0,0,0,0,0,0],[0,0,1,0,0,0,0,0,0],[0,0,1,0,0,0,0,0,0],[0,0,1,0,0,0,0,0,0],[0,0,1,0,0,0,0,0,0],[0,0,1,0,0,0,0,0,0]]7509	1696,7510,7511,7512,7513,7514,7515,7516,7517,7518,7519,7520,7521,7522,7523,1700,7524,7525,7526,7527,7528,7529,7530,7531,1660,1656,7532,7533,7534,7535,7536,7537,7538,7539,7540,7541,7542,7543,7544,7545,7546,7547,7548,6624,7549,1657,1651,7550,7551,7552,7553	[[0,0,1,0,1,0,0,1,0],[0,0,1,0,1,0,0,0,0],[0,0,1,0,0,0,0,0,0],[0,0,1,0,0,0,0,0,0],[0,0,0,0,0,0,0,1,0],[0,0,1,0,0,0,0,0,0],[0,0,0,0,0,0,0,1,0],[0,0,0,0,0,0,0,1,0],[0,0,1,0,0,0,0,0,0],[0,0,0,0,1,0,0,0,0],[0,0,0,0,0,0,0,1,0],[0,0,1,0,0,0,0,0,0],[0,0,0,0,0,0,0,1,0],[0,0,0,0,0,0,0,1,0],[0,0,1,0,0,0,0,0,0],[0,0,1,0,0,0,0,1,0],[0,0,1,0,0,0,0,0,0],[0,0,0,0,0,0,0,1,0],[0,0,1,0,0,0,0,0,0],[0,0,0,0,0,0,0,1,0],[0,0,1,0,0,0,0,0,0],[0,0,1,0,0,0,0,0,0],[0,0,1,0,0,0,0,0,0],[0,0,1,0,0,0,0,0,0],[0,0,1,0,1,0,0,1,0],[0,0,0,0,0,0,0,1,0],[0,0,0,0,1,0,0,0,0],[0,0,1,0,0,0,0,1,0],[0,0,0,0,1,0,0,0,0],[0,0,1,0,0,0,0,0,0],[0,0,1,0,0,0,0,0,0],[0,0,0,0,1,0,0,0,0],[0,0,1,0,0,0,0,0,0],[0,0,1,0,0,0,0,0,0],[0,0,1,0,0,0,0,0,0],[0,0,0,0,0,0,0,1,0],[0,0,1,0,0,0,0,0,0],[0,0,1,0,0,0,0,0,0],[0,0,1,0,0,0,0,0,0],[0,0,1,0,0,0,0,0,0],[0,0,1,0,0,0,0,0,0],[0,0,1,0,0,0,0,0,0],[0,0,1,0,0,0,0,0,0],[0,0,0,0,0,0,0,1,0],[0,0,0,0,0,0,0,1,0],[0,0,1,0,1,0,0,0,0],[0,0,1,0,0,0,0,0,0],[0,0,1,0,0,0,0,1,0],[0,0,1,0,0,0,0,0,0],[0,0,1,0,0,0,0,0,0],[0,0,0,0,0,0,0,1,0]]7554	7555,7556,7557,7558,7559,7560,7561	[[0,0,0,0,1,0,0,0,0],[0,0,0,0,1,0,0,0,0],[0,0,0,0,1,0,0,0,0],[0,0,0,0,1,0,0,0,0],[0,0,0,0,1,0,0,0,0],[0,0,0,0,1,0,0,0,0],[0,0,0,0,1,0,0,0,0]]7562	7563	[[0,0,0,0,1,0,0,0,0]]7564	7565,7566,7460,7567,7457,7568,7458,2035,7569,7570,7571,7572,7573,7574,7575,7576,7459,7577,2757,7578,7579,7580,7581,5809,7582,7583,7455,7584,7585,7586,7456,138,7587	[[0,0,1,0,0,0,0,0,0],[0,0,1,0,0,0,0,0,0],[0,0,0,1,0,0,0,0,0],[0,0,1,0,0,0,0,0,0],[0,0,1,1,0,0,0,0,0],[0,0,1,0,0,0,0,0,0],[0,0,0,1,0,0,0,0,0],[0,0,1,0,0,0,0,0,0],[0,0,1,0,0,0,0,0,0],[0,0,1,0,0,0,0,0,0],[0,0,1,0,0,0,0,0,0],[0,0,1,0,0,0,0,0,0],[0,0,1,0,0,0,0,0,0],[0,0,1,0,0,0,0,0,0],[0,0,1,0,0,0,0,0,0],[0,0,1,0,0,0,0,0,0],[0,0,0,1,0,0,0,0,0],[0,0,1,0,0,0,0,0,0],[0,0,1,0,0,0,0,0,0],[0,0,1,0,0,0,0,0,0],[0,0,1,0,0,0,0,0,0],[0,0,1,0,0,0,0,0,0],[0,0,1,0,0,0,0,0,0],[0,0,1,0,0,0,0,0,0],[0,0,1,0,0,0,0,0,0],[0,0,1,0,0,0,0,0,0],[0,0,1,1,0,0,0,0,0],[0,0,1,0,0,0,0,0,0],[0,0,1,0,0,0,0,0,0],[0,0,1,0,0,0,0,0,0],[0,0,0,1,0,0,0,0,0],[0,0,1,0,0,0,0,0,0],[0,0,1,0,0,0,0,0,0]]7588	7589,7590,7591	[[0,0,0,1,0,0,0,0,0],[0,0,0,1,0,0,0,0,0],[0,0,0,1,0,0,0,0,0]]7592	7593	[[0,0,0,0,1,0,0,0,0]]7594	7595,7596,7597,1294,7598,7599,7600,7601,7602,7603,1295,7604,7605,7606,7607,1293,1292,7608,7609,7610,7611,7612,7613,7614,7615,7616,7617,7618	[[0,0,0,1,0,0,0,0,0],[0,0,0,1,0,0,0,0,0],[0,0,0,1,0,0,0,0,0],[0,0,0,1,0,0,0,0,0],[0,0,0,1,0,0,0,0,1],[0,0,0,0,0,0,0,0,1],[0,0,0,0,0,0,0,0,1],[0,0,0,1,0,0,0,0,1],[0,0,0,0,0,0,0,0,1],[0,0,0,1,0,0,0,0,0],[0,0,0,0,1,0,0,0,0],[0,0,0,1,0,0,0,0,0],[0,0,0,0,0,0,0,0,1],[0,0,0,1,0,0,0,0,0],[0,0,0,1,0,0,0,0,0],[0,0,0,1,0,0,0,0,0],[0,0,0,0,1,0,0,0,0],[0,0,0,0,0,0,0,0,1],[0,0,0,0,0,0,0,0,1],[0,0,0,1,0,0,0,0,1],[0,0,0,1,0,0,0,0,1],[0,0,0,1,0,0,0,0,1],[0,0,0,1,0,0,0,0,0],[0,0,0,0,1,0,0,0,0],[0,0,0,1,0,0,0,0,0],[0,0,0,1,0,0,0,0,0],[0,0,0,1,0,0,0,0,0],[0,0,0,1,0,0,0,0,1]]7619	7620,4189,3861,4164,7621,7622,1948,1673,3869,7623,7624,7625,7626,7627,7628,1664,2492,1665,7629,1643,7630,7631,6014,1938,7632,4100,3870,7633,3871,7634,4094,7635,3797,7636,7637,3850,1705,7638,7639,3862,3201,7640	[[0,1,0,0,0,0,0,0,0],[0,1,1,0,0,0,0,0,0],[0,1,0,0,0,0,0,1,0],[0,1,1,0,0,0,1,0,0],[0,1,1,0,0,0,0,0,0],[0,1,1,0,0,0,1,1,0],[0,1,0,0,0,0,0,0,0],[0,1,0,0,0,0,0,0,0],[0,1,0,0,0,0,1,1,0],[0,1,1,0,0,0,1,1,0],[0,0,1,0,0,0,0,0,0],[0,0,1,0,0,0,0,0,0],[0,0,1,0,0,0,0,0,0],[0,0,1,0,0,0,0,0,0],[0,0,0,1,0,0,0,1,0],[0,0,1,1,0,0,0,0,0],[0,0,1,0,0,0,0,0,0],[0,0,0,1,0,0,0,1,0],[0,0,1,0,0,0,0,0,0],[0,1,1,0,0,0,1,1,0],[0,0,1,0,0,0,0,0,0],[0,1,1,0,0,0,1,1,0],[0,1,1,0,0,0,1,1,0],[0,0,0,1,0,0,0,1,0],[0,0,1,0,0,0,0,0,0],[0,1,1,0,0,0,1,0,0],[0,1,1,0,0,0,1,0,0],[0,0,1,0,0,0,0,0,0],[0,0,1,0,0,0,0,0,0],[0,0,1,0,0,0,0,0,0],[0,1,1,0,0,0,1,1,0],[0,0,1,0,0,0,0,0,0],[0,0,1,0,0,0,0,0,0],[0,0,1,0,0,0,0,0,0],[0,0,1,0,0,0,0,0,0],[0,0,0,0,0,0,0,1,0],[0,1,1,0,0,0,1,1,0],[0,0,1,0,0,0,0,0,0],[0,0,0,0,0,0,0,1,0],[0,0,1,0,0,0,0,0,0],[0,0,1,0,0,0,0,0,0],[0,0,1,0,0,0,0,0,0]]7641	2600,2719,2716,2735,2703,2717,2700,2710,7642,2715,2738,2711,2704,2593	[[0,0,0,1,0,0,0,0,0],[0,0,1,1,0,0,0,0,0],[0,1,1,1,1,0,0,0,0],[0,0,1,0,0,0,0,0,0],[0,1,1,0,0,0,0,0,0],[0,0,1,0,0,0,0,0,0],[0,1,1,1,0,0,0,0,0],[0,1,1,0,0,0,0,0,0],[0,0,1,0,0,0,0,0,0],[0,0,1,0,0,0,0,0,0],[0,0,1,0,0,0,0,0,0],[0,1,0,0,0,0,0,0,0],[0,0,1,0,0,0,0,0,0],[0,1,1,0,0,0,0,0,0]]7643	7644,2170,719,7645,7646,7647,7648,7649,7650,7651,5908,7652,5645,2514,7653,7654,559,7655,5624,7656,7657,7658,7659,7660,7661,7426,5636,7662,7663,7664,2502,7665,7666,7667,7668,7669,5372,5611,7670,5142,7671,7672,3864,2474,7673,7674,2563,5634,7675,7676,7677,7678,7679,7680,7681,7682,7316,5146,7683,5623,2527,5628,3968,7684,5133,7685,7686,2885,2544,5629,7687,7688,6141,7689,7690,7691,7692,849,3183,5221,7693,7694,7695,7696,7697,7698,3187,7699,3066,3190,7700,2178,5656,761,2408,7701,7702,7703,7704,7705,2709,7706,7707,7708,5613,2492,3684,7709,3196,5617,7710,2722,7711,2505,7712,7713,7714,7715,7716,7717,3982,3973,7718,3237,5223,7719,2524,7720,7721,7722	[[0,1,1,0,0,0,1,0,0],[0,1,1,0,0,0,1,0,0],[0,1,0,0,0,0,1,0,0],[0,1,0,0,0,0,0,0,0],[0,1,1,1,0,1,1,0,1],[0,0,1,0,0,0,0,0,0],[0,1,1,0,0,1,0,0,0],[0,0,1,0,0,0,1,0,0],[0,0,1,0,0,0,0,0,0],[0,0,1,0,0,1,0,0,1],[0,1,1,0,1,0,0,0,0],[0,0,1,0,0,0,0,0,0],[0,0,1,0,0,0,0,0,0],[0,0,1,0,0,0,0,0,1],[0,0,1,0,0,0,1,0,0],[0,1,1,0,0,0,1,0,0],[0,0,0,0,0,0,0,0,1],[0,0,1,0,0,0,0,0,0],[0,0,0,1,0,0,0,0,0],[0,1,1,1,0,0,1,0,1],[0,1,0,0,0,0,1,0,0],[0,0,1,0,0,0,0,0,0],[0,0,1,0,0,0,0,0,0],[0,1,0,0,0,0,1,0,0],[0,1,0,0,0,0,1,0,0],[0,0,0,1,0,0,0,0,0],[0,1,1,0,1,1,1,0,0],[0,0,1,0,0,0,0,0,0],[0,0,1,0,0,0,0,0,1],[0,1,1,0,0,0,1,0,0],[0,1,0,0,0,0,1,0,0],[0,0,1,0,0,0,0,0,0],[0,1,0,0,0,0,1,0,0],[0,0,1,0,0,0,0,0,0],[0,1,0,0,0,0,1,0,0],[0,0,1,0,0,0,0,0,0],[0,0,1,0,0,0,0,0,0],[0,1,1,0,0,0,0,0,0],[0,0,0,1,0,0,0,0,0],[0,0,1,0,0,0,0,0,0],[0,1,0,0,0,0,1,0,0],[0,1,1,0,0,0,1,0,1],[0,0,1,0,1,0,0,0,0],[0,1,0,0,0,0,1,0,0],[0,0,1,0,0,0,0,0,0],[0,0,1,0,0,0,0,0,0],[0,0,1,0,0,0,1,0,0],[0,1,1,0,0,0,1,0,0],[0,0,1,0,0,0,0,0,0],[0,1,0,0,0,0,0,0,0],[0,0,1,0,0,0,0,0,0],[0,0,0,1,0,0,0,0,0],[0,0,1,0,0,0,0,0,0],[0,1,1,0,0,0,1,0,0],[0,1,1,0,0,0,1,0,0],[0,1,1,0,0,0,0,0,0],[0,1,0,0,0,0,1,0,0],[0,1,1,0,0,0,1,0,0],[0,1,0,0,0,0,1,0,0],[0,1,1,0,0,0,0,0,0],[0,1,1,1,0,0,1,0,0],[0,1,0,0,0,0,1,0,0],[0,1,0,0,0,0,0,0,0],[0,0,1,0,1,1,0,0,1],[0,0,1,0,0,0,0,0,0],[0,0,0,0,0,0,0,0,1],[0,0,1,0,0,0,1,0,1],[0,0,1,0,0,1,0,0,0],[0,0,1,0,1,1,0,0,1],[0,1,1,0,0,0,1,0,0],[0,0,0,0,0,0,0,0,1],[0,0,1,0,0,0,1,0,0],[0,0,0,1,0,0,0,0,0],[0,0,1,0,0,0,0,0,0],[0,0,1,0,0,0,0,0,0],[0,1,0,0,0,0,1,0,0],[0,0,1,0,0,0,0,0,0],[0,0,1,0,0,0,0,0,0],[0,0,1,1,0,0,0,0,0],[0,0,1,0,0,0,0,0,0],[0,0,1,0,0,0,0,0,1],[0,1,0,0,0,0,0,0,0],[0,1,0,0,0,0,0,0,0],[0,1,0,0,0,0,0,0,0],[0,1,0,0,0,0,1,0,0],[0,0,1,0,0,0,0,0,0],[0,0,1,0,1,1,1,0,1],[0,1,0,0,0,0,0,0,0],[0,0,1,0,0,0,0,0,0],[0,0,1,0,0,1,0,0,1],[0,0,0,0,0,0,0,0,1],[0,0,1,0,0,0,0,0,0],[0,1,1,0,0,0,0,0,0],[0,1,0,0,1,0,1,0,0],[0,1,1,0,0,0,1,0,0],[0,1,0,0,0,0,1,0,0],[0,0,1,0,0,0,0,0,0],[0,0,1,0,0,0,0,0,1],[0,0,1,0,0,1,0,0,1],[0,0,0,0,0,0,0,0,1],[0,0,1,0,0,0,0,0,0],[0,0,1,0,0,0,0,0,0],[0,0,1,1,0,0,0,0,1],[0,0,1,0,0,0,0,0,0],[0,1,0,0,0,0,0,0,0],[0,1,0,0,0,0,1,0,0],[0,0,1,0,0,0,0,0,0],[0,0,1,0,0,0,0,0,0],[0,0,0,0,0,0,0,0,1],[0,0,1,0,0,0,0,0,0],[0,0,1,1,0,1,1,0,1],[0,0,0,1,0,0,0,0,0],[0,0,1,0,0,0,0,0,0],[0,0,1,0,0,0,0,0,0],[0,0,1,0,0,0,0,0,0],[0,0,1,0,0,0,0,0,0],[0,1,0,0,0,0,1,0,0],[0,1,1,0,0,0,1,0,0],[0,0,1,0,0,0,0,0,0],[0,0,1,0,0,0,0,0,0],[0,1,0,0,0,0,0,0,0],[0,1,0,0,0,0,0,0,0],[0,0,1,0,0,0,0,0,0],[0,0,0,1,0,0,0,0,0],[0,0,1,0,0,0,0,0,0],[0,0,1,0,0,0,0,0,0],[0,0,0,0,0,0,1,0,0],[0,0,1,0,0,0,0,0,0],[0,0,0,0,0,0,0,0,1],[0,0,0,0,0,1,0,0,0]]7723	313,7724,5024,7189,7725,7726,3655,7727,7728,5451,7729,7730,7731,7732,898,7733,7734,7735,7736,7737,7738,7739,5026,7740,1631,3774,5018	[[0,0,0,1,0,0,0,0,0],[0,0,1,0,0,0,0,0,0],[0,0,1,0,0,0,0,0,0],[0,0,1,0,0,0,0,0,0],[0,0,1,0,0,0,0,0,0],[0,0,1,0,0,0,0,0,0],[0,0,1,0,0,0,0,0,0],[0,0,0,0,1,0,0,0,0],[0,0,1,0,0,0,0,0,0],[0,0,1,0,0,0,0,0,0],[0,0,1,0,0,0,0,0,0],[0,0,1,1,0,0,0,0,0],[0,0,1,0,0,0,0,0,0],[0,0,1,0,0,0,0,0,0],[0,0,1,0,0,0,0,0,0],[0,0,1,0,0,0,0,0,0],[0,0,1,0,0,0,0,0,0],[0,0,1,0,0,0,0,0,0],[0,0,1,0,0,0,0,0,0],[0,0,1,1,0,0,0,0,0],[0,0,1,0,0,0,0,0,0],[0,0,1,0,0,0,0,0,0],[0,0,1,0,0,0,0,0,0],[0,0,1,0,0,0,0,0,0],[0,0,1,0,0,0,0,0,0],[0,0,1,0,0,0,0,0,0],[0,0,1,0,0,0,0,0,0]]7741	7742,2144,2177,7743,7744,7745	[[0,0,0,0,1,0,0,0,0],[0,0,0,0,1,0,0,0,0],[0,0,0,0,1,0,0,0,0],[0,0,0,0,1,0,0,0,0],[0,0,0,0,1,0,0,0,0],[0,0,0,0,1,0,0,0,0]]7746	7747	[[0,0,0,0,1,0,0,0,0]]7748	7749,7750,1496,7751,1504,7752,7753,1494,1515,2859,1509,4865,1514,7754,7755,1397,7756,7757,6962,5157,1501,7758,1518,1886,3208,6960,6961,7759,7760,7761,7762,7763,7764,7765,1525,1499,1505,7766,7767	[[0,0,0,1,0,0,0,0,0],[0,1,0,0,0,0,0,0,1],[1,1,0,1,0,0,1,0,1],[0,0,1,0,0,0,0,0,0],[1,1,1,1,1,0,1,0,1],[0,0,1,0,0,0,0,0,0],[0,1,0,1,0,0,0,0,0],[0,0,1,0,0,0,0,0,0],[1,1,1,1,1,0,1,0,0],[0,0,1,0,0,0,0,0,0],[0,1,1,1,0,0,0,0,1],[0,0,1,0,0,0,0,0,0],[0,1,0,0,0,0,1,0,0],[0,0,1,0,0,0,0,0,0],[0,0,1,0,0,0,0,0,1],[1,0,1,1,0,0,0,0,0],[0,0,0,0,1,0,0,0,0],[0,0,1,0,0,0,0,0,0],[0,0,1,0,0,0,0,0,0],[1,0,1,1,0,0,0,0,0],[1,1,0,0,0,0,1,0,0],[0,1,1,0,0,0,1,0,0],[0,1,0,0,0,0,0,0,0],[0,1,0,0,0,0,0,0,0],[1,0,0,0,0,0,0,0,0],[1,1,1,1,0,0,0,0,0],[1,0,0,0,0,0,0,0,0],[1,0,0,0,0,0,0,0,0],[1,0,0,0,0,0,0,0,0],[1,0,0,0,0,0,0,0,0],[0,0,1,0,0,0,0,0,0],[0,0,1,0,0,0,0,0,0],[0,0,1,0,0,0,0,0,0],[0,0,1,0,0,0,0,0,0],[0,1,0,0,0,0,0,0,0],[0,0,0,1,0,0,0,0,0],[0,0,1,0,0,0,0,0,0],[0,0,1,0,0,0,0,0,0],[0,0,1,0,0,0,0,0,0]]7768	7769	[[0,0,0,0,1,0,0,0,0]]7770	7771	[[0,0,0,0,1,0,0,0,0]]7772	7773,4555,6578,7774,7775,2104,7776,7777,7778,3929,7779,7780,6584,7781,7782,7783,7784,2423,6614,7785,6598,6587,6616,6603,2579,7786,6607,4894,4845,7787,5199,1305	[[0,0,0,0,0,0,1,0,0],[0,0,0,0,0,0,1,0,0],[0,1,1,1,0,0,1,0,0],[0,0,0,0,0,0,1,0,0],[0,0,0,0,0,0,0,1,0],[0,1,0,1,0,0,0,0,0],[0,0,1,0,0,0,0,0,0],[0,0,0,0,0,0,1,0,0],[0,0,1,0,0,0,0,0,0],[0,1,1,0,0,0,1,0,0],[0,1,1,0,0,0,0,0,0],[0,1,1,1,0,0,0,1,0],[0,1,1,0,0,0,0,1,0],[0,1,0,0,0,0,0,0,0],[0,0,1,0,0,0,0,0,0],[0,0,0,0,0,0,0,1,0],[0,1,1,0,0,0,1,0,0],[0,0,1,0,0,0,0,1,0],[0,1,0,0,0,0,1,0,0],[0,1,0,0,0,0,0,0,0],[0,1,1,0,0,0,0,1,0],[0,0,1,0,0,0,0,0,0],[0,0,1,0,0,0,1,1,0],[0,1,1,0,0,0,1,0,0],[0,0,0,0,0,0,1,0,0],[0,0,0,0,0,0,0,1,0],[0,1,0,0,0,0,1,1,0],[0,1,0,0,0,0,1,1,0],[0,1,0,0,0,0,1,0,0],[0,0,1,0,0,0,0,0,0],[0,0,1,0,0,0,0,0,0],[0,0,1,0,0,0,0,0,0]]7788	7789	[[0,0,0,0,1,0,0,0,0]]7790	7791,5730,7792	[[0,0,0,0,1,0,0,0,0],[0,0,0,0,1,0,0,0,0],[0,0,0,0,1,0,0,0,0]]7793	7794	[[0,0,0,0,1,0,0,0,0]]7795	213,7299,7796	[[0,0,0,1,0,0,0,0,0],[0,0,0,1,0,0,0,0,0],[0,0,0,1,0,0,0,0,0]]7797	5339,7798,5025,7799,7800,3661,2720,6939,2916,7801,2244,7705,7802,987,4235,7803,7804,7805,5443,898,1762,5944,7806,7807,2915,7808,896,7809,5458,7810,3742,4224,7811,1235,5451,7812,7171,3762,7813,2148,7814,5433,994,5434,6391,4840,7815,3779,7816,887,7817,7818,310,3723,7819,3726,7820,5479,7821,5026,921,7166,7822,7823,2913,7824,7825,5442,7732,7826,1859,5033,2932,7739,7827,996,7828,7829,3722,7830,1674,7831,7832,7189,7728,7833,7834,7835,7836,2301,7837,3732,7838,7839,7840,3741,7841,7842,7843,7844,7845,7729,7725,7846,3733,7847,7848,1132,7849,2239,7850,2172,7851,7061,919,7852	[[0,1,1,0,0,0,1,0,0],[0,0,1,0,0,0,0,0,0],[0,0,1,0,0,0,0,0,0],[0,0,1,0,0,0,0,0,0],[0,0,1,0,0,0,0,0,0],[0,1,1,0,0,0,1,0,0],[0,1,0,0,0,0,1,0,0],[0,1,1,0,0,0,1,0,0],[0,0,1,0,0,0,0,0,0],[0,0,1,0,0,0,0,0,0],[0,0,1,0,0,0,0,0,0],[0,1,1,0,0,0,1,0,0],[0,0,1,0,0,0,0,0,0],[0,0,1,0,0,0,0,0,0],[0,0,1,0,0,0,0,0,0],[0,0,1,0,0,0,0,0,0],[0,0,1,0,0,0,0,0,0],[0,0,1,0,0,0,0,0,0],[0,0,1,0,0,0,0,0,0],[0,0,1,0,0,0,0,0,0],[0,0,1,0,0,0,0,0,0],[0,0,1,0,0,0,0,0,0],[0,1,1,0,0,0,0,0,0],[0,0,1,0,0,0,0,0,0],[0,0,1,1,0,0,0,0,0],[0,0,1,0,0,0,0,0,0],[0,0,1,0,0,0,0,0,0],[0,0,1,0,0,0,0,0,0],[0,0,1,0,0,0,0,0,0],[0,1,1,0,0,0,1,0,0],[0,0,1,0,0,0,0,0,0],[0,0,0,0,1,0,0,0,0],[0,0,1,0,0,0,0,0,0],[0,0,0,0,1,0,0,0,0],[0,1,1,0,0,0,1,0,0],[0,0,1,0,0,0,0,0,0],[0,1,0,0,0,0,1,0,0],[0,0,0,1,0,0,0,0,0],[0,0,1,0,0,0,0,0,0],[0,0,1,0,0,0,0,0,0],[0,1,1,0,0,0,0,0,0],[0,1,1,0,0,0,1,0,0],[0,0,1,0,0,0,0,0,0],[0,0,1,0,0,0,0,0,0],[0,0,1,0,0,0,0,0,0],[0,0,1,0,0,0,0,0,0],[0,0,1,0,0,0,0,0,0],[0,0,1,0,0,0,0,0,0],[0,1,1,0,0,0,1,0,0],[0,1,1,0,1,0,0,0,0],[0,0,1,0,0,0,0,0,0],[0,0,1,0,0,0,0,0,0],[0,0,1,0,0,0,0,0,0],[0,1,0,0,0,0,1,0,0],[0,0,0,1,0,0,0,0,0],[0,1,1,1,0,0,1,0,0],[0,1,1,0,0,0,1,0,0],[0,0,1,0,0,0,0,0,0],[0,1,1,1,0,0,0,0,0],[0,0,1,0,0,0,0,0,0],[0,0,1,0,0,0,0,0,0],[0,0,1,0,0,0,0,0,0],[0,0,0,0,0,0,1,0,0],[0,0,1,0,0,0,0,0,0],[0,1,0,0,0,0,1,0,0],[0,1,0,0,0,0,1,0,0],[0,0,1,0,0,0,0,0,0],[0,0,1,0,0,0,0,0,0],[0,0,1,0,0,0,0,0,0],[0,1,1,1,0,0,0,0,0],[0,1,1,0,0,0,0,0,0],[0,1,1,0,0,0,1,0,0],[0,1,1,1,0,0,1,0,0],[0,0,1,0,0,0,0,0,0],[0,0,1,0,0,0,0,0,0],[0,0,1,0,0,0,0,0,0],[0,0,1,0,0,0,0,0,0],[0,1,0,0,0,0,1,0,0],[0,0,0,0,0,0,1,0,0],[0,1,1,1,0,0,1,0,0],[0,1,0,0,1,0,1,0,0],[0,1,1,0,0,0,0,0,0],[0,0,1,0,0,0,0,0,0],[0,1,0,0,0,0,0,0,0],[0,1,0,0,0,0,0,0,0],[0,0,1,0,0,0,0,0,0],[0,0,1,0,0,0,0,0,0],[0,0,1,0,0,0,0,0,0],[0,0,0,0,1,0,0,0,0],[0,0,1,0,0,0,0,0,0],[0,0,1,0,0,0,0,0,0],[0,1,1,0,0,0,1,0,0],[0,0,1,0,0,0,0,0,0],[0,0,1,0,0,0,0,0,0],[0,1,0,0,0,0,1,0,0],[0,1,1,0,0,0,1,0,0],[0,1,0,0,0,0,0,0,0],[0,1,1,0,0,0,1,0,0],[0,0,1,0,0,0,0,0,0],[0,0,1,1,0,0,0,0,0],[0,0,1,0,0,0,0,0,0],[0,0,1,0,0,0,0,0,0],[0,0,1,0,0,0,0,0,0],[0,0,1,0,0,0,0,0,0],[0,0,1,0,0,0,0,0,0],[0,0,1,0,0,0,0,0,0],[0,0,1,0,0,0,0,0,0],[0,0,1,0,0,0,0,0,0],[0,0,1,0,0,0,0,0,0],[0,0,1,0,0,0,0,0,0],[0,0,1,0,0,0,0,0,0],[0,0,1,0,0,0,0,0,0],[0,0,1,0,0,0,0,0,0],[0,0,1,0,0,0,0,0,0],[0,0,0,1,0,0,0,0,0],[0,0,1,0,0,0,0,0,0]]7853	7854,7855	[[0,0,0,0,1,0,0,0,0],[0,0,0,0,1,0,0,0,0]]7856	7857,7858,7859	[[0,1,0,0,0,0,0,0,0],[0,1,0,0,0,0,0,0,0],[0,1,0,0,0,0,0,0,0]]7860	1211,7861,7862,7863,7864,7865	[[0,0,0,1,0,0,0,0,0],[0,0,0,1,0,0,0,0,0],[0,0,0,1,0,0,0,0,0],[0,0,0,1,0,0,0,0,0],[0,0,0,1,0,0,0,0,0],[0,0,0,1,0,0,0,0,0]]7866	7867,7868,7869,7870,7871,7872,7873,7874,7875,7876,7877,7878,7879,7880,7881,7882,7481,7883,7884,1378,4175,5552,3082,4490,7885,7886,7887,4506,5560,7888,4195,951,7889,7890,7891,7892	[[0,1,1,0,0,0,0,1,1],[0,1,1,0,0,0,0,1,0],[0,0,0,0,1,0,0,0,0],[0,0,1,0,0,0,0,0,0],[0,1,1,0,0,0,0,0,1],[0,0,1,0,0,0,0,0,0],[0,1,1,0,0,0,0,0,0],[0,1,0,0,0,0,0,0,0],[0,0,1,0,0,0,0,0,0],[0,1,0,0,0,0,0,0,0],[0,1,1,0,0,0,0,0,0],[0,1,1,0,0,0,0,0,0],[0,1,0,0,1,0,0,0,0],[0,1,1,0,0,0,0,1,1],[0,1,1,0,0,0,0,1,0],[0,1,1,1,0,0,0,1,0],[0,0,1,0,0,0,0,0,0],[0,0,1,1,0,0,0,1,1],[0,0,1,0,0,0,0,0,0],[0,0,0,0,1,0,0,0,0],[0,0,0,0,0,0,0,1,0],[0,0,0,0,0,0,0,1,0],[0,0,1,0,0,0,0,0,0],[0,0,0,0,0,0,0,1,0],[0,0,0,0,1,0,0,0,0],[0,0,1,0,0,0,0,0,0],[0,0,1,0,0,0,0,0,0],[0,0,0,0,0,0,0,1,0],[0,0,0,1,0,0,0,0,0],[0,0,1,0,0,0,0,0,0],[0,0,0,0,0,0,0,1,0],[0,0,1,0,0,0,0,0,0],[0,0,1,0,0,0,0,0,0],[0,0,1,0,0,0,0,0,0],[0,0,1,0,0,0,0,1,1],[0,0,1,0,0,0,0,0,0]]7893	2852,7894,7895,955,7896,2915,7897,7898,7899,7900,965,963,7901,959,3417,487,4326,7902,7903,962,7904,7905,7906,7907,7908,7909,7910,7911,7912,7913,2486,7914,4555,7915,1016,132,7916,7917,5941,7918,7919,7920,7921,7922,3126,7923,3239,7924,2587,7925,7926,7927,7928,7929,7930,7931,7932,4678,7092,7933,1786,7934,3729,5091,5070,7935,7936,7937,7025,2513,827,7938,3768,7939,7940,7941,798,7942,3437,7943,5204,7944,7945,7777	[[0,0,1,0,0,0,0,0,0],[1,1,1,0,1,0,0,0,1],[1,0,1,0,0,0,0,0,1],[0,0,1,0,0,0,0,0,0],[1,0,1,0,1,0,0,0,0],[0,0,1,0,0,0,0,0,0],[0,0,1,0,0,0,0,0,0],[0,0,1,0,0,0,0,0,0],[0,0,1,0,0,0,0,0,0],[0,1,1,0,0,0,0,0,0],[0,0,1,0,0,0,0,0,0],[0,0,1,0,0,0,0,0,0],[1,1,1,1,1,0,0,0,1],[0,0,1,0,0,0,0,0,0],[0,0,1,0,0,0,0,0,0],[0,0,1,0,0,0,0,0,0],[0,0,1,0,1,0,0,0,0],[1,0,0,0,0,0,0,0,1],[1,1,1,1,0,0,0,0,1],[1,1,1,0,1,0,0,0,0],[1,0,1,0,0,0,0,0,0],[1,0,0,0,0,0,0,0,0],[0,0,1,0,0,0,0,0,0],[0,1,1,1,0,0,0,0,1],[0,1,0,0,0,0,0,0,0],[1,1,1,0,0,0,0,0,0],[1,1,0,0,0,0,0,0,0],[1,1,1,0,0,0,0,0,0],[1,0,1,1,0,0,0,0,1],[0,0,1,0,0,0,0,0,0],[0,0,1,0,0,0,0,0,0],[1,0,1,0,0,0,0,0,0],[0,1,1,0,0,0,0,0,0],[1,0,1,0,0,0,0,0,0],[0,1,0,0,0,0,0,0,0],[0,1,1,0,0,0,0,0,0],[0,1,0,1,0,0,0,0,0],[1,1,1,0,0,0,0,0,1],[0,1,0,0,0,0,0,0,0],[0,1,0,1,0,0,0,0,0],[0,1,0,0,0,0,0,0,0],[0,1,0,0,0,0,0,0,0],[0,1,0,0,0,0,0,0,0],[1,0,1,1,0,0,0,0,0],[1,0,0,0,0,0,0,0,0],[0,0,1,0,0,0,0,0,0],[0,0,1,0,0,0,0,0,0],[0,0,1,0,0,0,0,0,0],[0,0,1,0,0,0,0,0,0],[0,0,1,0,0,0,0,0,0],[0,0,1,0,0,0,0,0,0],[0,0,1,0,0,0,0,0,0],[0,0,1,0,0,0,0,0,0],[0,0,1,0,0,0,0,0,0],[0,0,1,0,0,0,0,0,0],[1,0,0,0,0,0,0,0,0],[1,0,1,0,0,0,0,0,0],[1,0,0,0,0,0,0,0,0],[1,0,0,0,0,0,0,0,0],[1,0,0,0,0,0,0,0,0],[1,0,0,0,0,0,0,0,0],[0,0,1,0,0,0,0,0,0],[0,0,1,0,0,0,0,0,0],[1,0,0,0,0,0,0,0,0],[1,0,0,0,0,0,0,0,0],[1,0,0,0,0,0,0,0,0],[1,0,0,0,0,0,0,0,0],[0,0,1,0,0,0,0,0,0],[0,0,1,0,0,0,0,0,0],[0,0,1,0,0,0,0,0,0],[0,0,1,0,0,0,0,0,0],[0,0,1,0,0,0,0,0,0],[0,0,1,0,0,0,0,0,0],[0,0,1,0,0,0,0,0,0],[0,0,1,0,0,0,0,0,0],[0,0,1,0,0,0,0,0,0],[0,0,1,0,0,0,0,0,0],[1,0,0,0,0,0,0,0,0],[1,0,0,0,0,0,0,0,0],[1,0,0,0,0,0,0,0,0],[0,0,1,0,0,0,0,0,0],[0,0,1,0,0,0,0,0,0],[0,0,1,0,0,0,0,0,0],[0,0,1,0,0,0,0,0,0]]7946	2257,7947,7948,7949,7950,7951,7952,2256,2243,2267,2247,7953,7954,7339	[[0,0,1,0,0,0,0,1,0],[0,1,1,1,0,1,1,1,1],[0,1,1,0,0,1,1,1,1],[0,1,1,1,1,1,1,1,1],[0,0,1,0,0,1,0,0,1],[0,0,1,0,0,0,0,0,1],[0,1,1,1,1,1,1,1,1],[0,0,1,0,0,0,0,0,0],[0,0,1,0,0,0,0,0,0],[0,1,1,0,0,1,1,1,1],[0,0,1,0,0,0,0,1,1],[0,0,0,0,0,1,0,0,0],[0,1,1,0,1,0,0,0,0],[0,0,0,0,0,1,0,0,0]]7955	7956,7957,2006,896,4491,2397,5144,5720,7958,7959,2268,7844,887,7960,7802,3231,5433	[[0,1,0,0,0,0,0,0,0],[0,0,0,1,0,0,0,0,0],[0,1,0,0,0,0,1,0,0],[0,0,0,0,0,0,1,0,0],[0,0,1,0,0,0,0,0,0],[0,1,1,0,0,0,1,0,0],[0,1,0,0,0,0,0,0,0],[0,1,1,0,0,0,1,0,0],[0,1,0,0,0,0,1,0,0],[0,1,0,0,0,0,0,0,0],[0,1,0,0,0,0,0,0,0],[0,1,1,1,0,0,1,0,0],[0,0,1,0,0,0,0,0,0],[0,0,1,1,0,0,0,0,0],[0,0,1,0,0,0,0,0,0],[0,0,1,0,0,0,0,0,0],[0,0,0,1,0,0,0,0,0]]7961	7962,7963,5502,2249,7964,6066,7965,2372,7966,630,7967,7968,6073,2255	[[0,0,0,0,0,0,0,1,0],[0,0,0,0,0,0,0,1,0],[0,0,0,0,0,0,0,1,0],[0,0,0,0,0,0,0,1,0],[0,0,0,0,0,0,0,1,0],[0,0,0,0,0,0,0,1,0],[0,0,0,0,0,0,0,1,0],[0,0,0,0,0,0,0,1,0],[0,0,0,0,0,0,0,1,0],[0,0,0,0,0,0,0,1,0],[0,0,0,0,0,0,0,1,0],[0,0,0,0,0,0,0,1,0],[0,0,0,0,0,0,0,1,0],[0,0,0,0,0,0,0,1,0]]7969	5560,7883,7882	[[0,0,0,1,0,0,0,0,0],[0,0,0,1,0,0,0,0,0],[0,0,0,1,0,0,0,0,0]]7970	7863,7865,7861,7862,7864,1211	[[0,0,0,1,0,0,0,0,0],[0,0,0,1,0,0,0,0,0],[0,0,0,1,0,0,0,0,0],[0,0,0,1,0,0,0,0,0],[0,0,0,1,0,0,0,0,0],[0,0,0,1,0,0,0,0,0]]7971	7972,7973	[[0,0,0,1,0,0,0,0,0],[0,0,0,1,0,0,0,0,0]]7974	7975,7976,7977	[[0,0,0,0,1,0,0,0,0],[0,0,0,0,1,0,0,0,0],[0,0,0,0,1,0,0,0,0]]7978	1197,7979,2485,7980,2442,7981,1561,7982,7027,7983,5175,7984,6928,7028,1397,7985,895,6386,7986,6360,7987,7988,6377,7989,1640,6383,2434,7990,7991,7992,6439,6930,6430,7993,887,860,6945,7994,3920,6432,7995,3047,7996,5087,3050,921	[[0,0,1,1,0,0,0,0,0],[0,0,1,0,0,0,0,0,0],[1,1,0,0,0,0,1,0,0],[0,0,1,0,0,0,0,0,0],[0,0,1,0,0,0,0,0,0],[1,0,1,1,0,0,0,1,0],[0,1,0,0,0,0,1,0,0],[1,0,0,1,0,0,0,1,0],[0,0,1,0,0,0,0,0,0],[1,1,1,1,0,0,1,1,0],[1,1,1,1,0,0,1,1,0],[0,0,1,0,0,0,0,0,0],[0,0,1,1,0,0,0,1,0],[0,0,1,0,0,0,0,0,0],[0,0,1,0,0,0,0,0,0],[0,0,1,0,0,0,0,0,0],[1,0,1,1,0,0,0,1,0],[1,1,1,1,0,0,0,1,0],[0,1,0,1,0,0,0,0,0],[0,1,1,1,0,0,1,1,0],[0,0,1,0,0,0,0,0,0],[0,0,0,0,0,0,0,1,0],[1,1,1,1,0,0,1,1,0],[0,0,0,1,0,0,0,0,0],[0,0,1,0,0,0,0,0,0],[1,1,1,1,0,0,1,0,0],[1,1,1,1,0,0,1,0,0],[0,1,0,0,0,0,0,0,0],[0,1,0,0,0,0,1,0,0],[0,0,1,0,0,0,0,0,0],[0,0,1,0,0,0,0,0,0],[0,0,1,0,0,0,0,0,0],[0,0,1,0,0,0,0,0,0],[0,0,1,1,0,0,0,1,0],[1,0,0,0,0,0,0,0,0],[1,0,0,0,0,0,0,0,0],[0,0,1,0,0,0,0,0,0],[0,0,1,0,0,0,0,0,0],[1,0,0,0,0,0,0,0,0],[0,0,1,1,0,0,0,0,0],[0,0,1,0,0,0,0,0,0],[0,0,1,0,0,0,0,0,0],[0,0,0,1,0,0,0,1,0],[1,0,0,0,0,0,0,0,0],[0,0,1,0,0,0,0,0,0],[0,0,1,0,0,0,0,0,0]]7997	7998,7999,5136,8000,3906,8001,8002,8003,2864,2243,8004,7684,5135,3921,8005,8006,5674,8007,3680,8008,8009,8010,8011,8012,2255,8013,2249,1640,8014,4606,8015,8016,6935,5930,1554,5933,8017,8018,2178,6429,4247,8019,8020,8021,4552,8022,8023,8024,8025,3639,760,2131,8026,8027,4553,4635,8028,8029,8030,5141,3724,8031,8032,8033,8034,8035,8036,8037,7008,8038,5139,8039,5146,2141,5125,8040,8041,8042,5137,8043,8044,7949,8045,8046,8047,8048,8049,8050,8051,8052,705,2267,8053,8054,8055,5937,8056,8057,2874,8058,8059,4884,3741,3141,8060,5128,3229,2247,8061,8062,8063,8064,8065,4256,8066,6927,2865,1897,5126,8067,3902,8068,8069,8070,5004,8071,8072,704,3190,3876,8073,3216,8074,8075,8076,7206,8077,6929	[[0,1,1,0,0,0,1,0,0],[0,1,0,0,0,0,1,0,0],[0,1,1,1,0,0,1,1,1],[0,0,1,0,1,0,0,1,1],[0,0,1,0,0,0,0,0,0],[0,0,1,0,0,0,0,0,0],[0,1,1,1,0,0,1,0,0],[0,0,1,0,0,0,0,0,0],[0,0,0,0,0,0,0,0,1],[0,0,1,0,0,0,0,0,0],[0,0,1,0,0,0,0,0,0],[0,0,1,0,0,0,0,0,0],[0,0,0,1,1,0,0,1,0],[0,0,1,0,0,0,0,0,0],[0,0,0,0,0,0,0,1,0],[0,0,1,0,0,0,0,0,0],[0,0,1,0,0,0,0,0,0],[0,0,1,0,0,0,0,0,0],[0,0,1,0,0,0,0,0,0],[0,0,0,0,0,0,1,0,0],[0,0,1,0,0,0,0,0,0],[0,0,1,0,0,0,0,0,0],[0,0,1,0,0,0,0,0,0],[0,0,1,0,0,0,0,0,0],[0,1,1,0,1,0,0,0,0],[0,0,1,0,0,0,0,0,0],[0,1,1,0,1,0,0,0,0],[0,0,1,0,0,0,0,0,0],[0,0,1,0,0,0,0,0,0],[0,0,1,0,0,0,0,0,0],[0,0,1,0,0,0,0,0,0],[0,0,1,0,0,0,0,0,0],[0,0,1,0,1,0,0,0,0],[0,0,0,0,1,0,0,0,0],[0,0,1,0,0,0,0,0,0],[0,0,1,0,1,0,0,0,0],[0,0,0,0,1,0,0,0,0],[0,0,1,0,0,0,0,0,0],[0,0,1,0,0,0,0,0,0],[0,0,1,0,0,0,0,0,0],[0,0,1,0,0,0,0,0,0],[0,0,1,0,0,0,0,0,0],[0,0,1,0,0,0,0,0,0],[0,0,1,0,0,0,0,0,0],[0,0,1,0,0,0,0,0,0],[0,1,1,1,0,0,1,1,0],[0,0,1,0,0,0,0,0,0],[0,0,1,1,0,0,1,1,0],[0,0,1,0,0,0,0,0,0],[0,0,1,0,0,0,0,0,0],[0,0,1,0,0,0,0,0,0],[0,0,1,1,0,0,0,1,0],[0,0,1,0,0,0,0,0,0],[0,0,1,0,0,0,0,0,0],[0,0,1,0,0,0,0,0,0],[0,0,1,0,0,0,0,0,0],[0,0,1,0,0,0,0,0,0],[0,1,1,1,0,0,0,1,1],[0,1,1,1,0,0,1,1,0],[0,1,1,1,0,0,1,0,1],[0,1,1,1,0,0,1,0,0],[0,1,1,1,0,0,1,1,1],[0,1,1,0,0,0,0,1,0],[0,1,0,0,0,0,1,0,0],[0,1,1,0,0,0,0,0,0],[0,1,1,1,0,0,1,1,1],[0,1,1,0,0,0,1,1,0],[0,0,1,0,0,0,0,1,0],[0,1,1,0,1,0,0,0,0],[0,1,1,0,1,0,0,0,0],[0,1,1,0,0,0,0,0,0],[0,1,1,1,0,0,1,1,1],[0,1,1,1,0,0,1,1,1],[0,1,1,1,0,0,1,1,1],[0,1,1,1,0,0,1,1,1],[0,0,1,0,0,0,0,0,0],[0,0,1,0,0,0,0,0,0],[0,0,1,0,0,0,0,1,0],[0,0,1,0,0,0,0,0,0],[0,0,1,0,0,0,0,0,0],[0,0,1,0,0,0,0,0,0],[0,0,1,0,0,0,0,0,0],[0,0,1,1,0,0,1,0,1],[0,0,1,0,0,0,0,0,0],[0,0,1,0,0,0,0,0,0],[0,0,1,0,0,0,0,0,0],[0,0,1,0,0,0,0,0,0],[0,0,1,0,0,0,0,0,0],[0,0,1,1,0,0,1,1,1],[0,0,1,0,1,0,0,0,0],[0,0,1,0,0,0,0,0,0],[0,0,1,0,0,0,0,0,0],[0,0,1,1,0,0,0,0,0],[0,0,1,0,0,0,0,0,0],[0,0,1,0,0,0,0,0,0],[0,0,0,0,1,0,0,0,0],[0,0,0,0,1,0,0,0,0],[0,0,1,0,0,0,0,0,0],[0,0,0,0,0,0,0,0,1],[0,0,1,0,1,0,0,0,0],[0,0,1,0,0,0,0,0,0],[0,0,1,0,0,0,0,0,0],[0,0,1,0,0,0,0,0,0],[0,0,1,0,0,0,0,1,0],[0,0,1,0,0,0,0,0,0],[0,0,1,0,0,0,0,0,1],[0,0,1,0,0,0,0,0,0],[0,0,1,0,0,0,0,0,0],[0,0,1,0,0,0,0,0,0],[0,0,0,0,0,0,0,1,0],[0,0,0,0,1,0,0,0,0],[0,0,1,0,0,0,0,0,0],[0,0,1,0,0,0,0,0,0],[0,0,1,0,0,0,0,0,0],[0,0,1,0,0,0,0,0,0],[0,0,1,0,0,0,0,0,0],[0,0,0,1,0,0,0,0,1],[0,0,1,1,0,0,1,0,0],[0,0,1,0,0,0,0,1,0],[0,0,1,0,0,0,0,0,0],[0,0,1,0,0,0,0,0,0],[0,0,1,1,0,0,1,1,0],[0,0,1,0,0,0,0,0,0],[0,0,1,1,0,0,0,1,1],[0,0,0,0,0,0,0,0,1],[0,0,1,1,0,0,1,0,1],[0,0,1,0,0,0,0,0,0],[0,0,1,0,0,0,0,0,0],[0,0,1,0,0,0,0,1,0],[0,0,1,0,0,0,0,0,1],[0,0,1,0,0,0,0,0,0],[0,0,1,0,0,0,0,0,0],[0,0,0,0,0,0,0,0,1],[0,0,1,0,0,0,0,0,0],[0,0,1,0,0,0,0,0,0],[0,0,1,0,0,0,0,0,0],[0,0,1,0,0,0,0,0,0],[0,0,1,0,0,0,0,0,0]]8078	8079	[[0,0,0,0,1,0,0,0,0]]8080	8081,310,8082,7047	[[0,0,1,0,0,0,0,0,0],[0,0,0,0,1,0,0,0,0],[0,0,0,0,1,0,0,0,0],[0,0,1,0,1,0,0,0,0]]8083	7982,7984,6387,2442,912,844,7822,7981,872,3050,8084,857,2434,6383,7389,880,860,867,8085,2328,7985,843,8086,852,8087,132,8088,2436,6363,6365,2437,2445,847,7169,2340,8089,2448,8090,879,7996,686,8091,8092,908,848,923,7983,922,915,2438,6369,8093,8094,8095,2356,2439,875,8096,2485,6345,7994,859,4626,6374,6386,8097,2452,6941,927,2451,78,939,895,8098,2446,6364,2295,5175,6377,8099,8100,3920,2299,6371,8101,910,8102,3047,2353,6366,850,7841,6367,5458,2440,906,6381,2341,898,2441,6432,6368,8103,6373,8104,876,8105,8106,2278,6360,2338,931,858,8107,2443,8108,7986,866,6136,2450,7993,8109,2449,2447,903,7989,881,5410,8110,1197,2332,8111,8112,874,878,987,8113,8114,2351,8115,864,2444,887,8116,8117,2333	[[0,0,1,0,0,0,0,0,1],[1,0,0,1,0,0,0,0,0],[0,0,1,0,0,0,0,0,0],[1,1,1,1,0,0,1,0,0],[1,0,1,0,0,0,0,0,0],[1,0,1,0,0,0,0,0,0],[0,0,1,0,0,0,0,0,0],[1,1,1,1,0,0,0,0,1],[1,0,0,1,0,0,0,0,0],[0,0,1,0,0,0,0,0,0],[0,0,1,0,0,0,0,0,0],[1,0,0,0,0,0,0,0,0],[1,1,1,0,0,0,1,0,1],[1,0,0,1,0,0,0,0,1],[1,0,1,0,0,0,0,0,0],[1,0,1,0,0,0,0,0,0],[1,0,1,0,0,0,0,0,0],[0,1,1,0,0,0,1,0,0],[1,0,1,0,0,0,0,0,0],[0,0,1,0,0,0,0,0,0],[0,0,1,0,0,0,0,0,0],[0,0,1,0,0,0,0,0,0],[0,0,1,0,0,0,0,0,0],[0,0,1,0,0,0,0,0,0],[0,0,1,0,0,0,0,0,0],[0,0,1,0,0,0,0,0,0],[0,0,1,0,0,0,0,0,0],[1,0,1,0,0,0,0,0,0],[0,1,1,1,0,0,1,0,0],[0,0,1,0,0,0,0,0,0],[1,1,1,1,0,0,1,0,0],[1,1,1,1,0,0,0,0,0],[0,0,1,0,0,0,0,0,0],[0,0,1,0,0,0,0,0,0],[0,1,1,0,1,0,1,0,0],[0,0,1,0,0,0,0,0,0],[1,0,1,0,0,0,0,0,0],[1,0,1,0,0,0,0,0,0],[0,0,1,0,0,0,0,0,0],[0,0,0,0,0,0,0,0,1],[0,0,1,0,0,0,0,0,0],[0,0,1,0,0,0,0,0,0],[0,0,1,0,0,0,0,0,0],[1,0,1,0,0,0,0,0,0],[1,0,1,0,0,0,0,0,0],[1,0,1,0,0,0,0,0,0],[0,0,0,0,0,0,0,0,1],[0,0,1,0,0,0,0,0,0],[0,0,1,0,0,0,0,0,0],[1,1,1,1,0,0,1,0,0],[0,0,1,0,0,0,0,0,0],[0,0,0,1,0,0,0,0,0],[0,0,1,0,0,0,0,0,0],[0,0,1,0,0,0,0,0,0],[0,0,1,0,0,0,0,0,0],[1,0,1,1,0,0,0,0,0],[0,0,1,0,0,0,0,0,0],[0,1,0,0,0,0,0,0,0],[0,1,1,1,0,0,1,0,0],[0,1,1,1,0,0,1,0,0],[0,0,1,0,0,0,0,0,0],[0,0,0,1,0,0,0,0,0],[0,0,1,0,0,0,0,0,0],[0,0,1,0,0,0,0,0,0],[0,0,0,0,0,0,0,0,1],[0,0,0,1,0,0,0,0,0],[0,0,1,1,0,0,0,0,0],[0,0,0,1,0,0,0,0,0],[0,0,1,0,0,0,0,0,0],[0,0,1,0,0,0,0,0,0],[0,0,1,0,0,0,0,0,0],[0,0,1,0,0,0,0,0,0],[1,0,0,1,0,0,0,0,1],[1,0,0,0,0,0,0,0,0],[0,0,1,0,0,0,0,0,0],[0,0,1,0,0,0,0,0,0],[1,0,1,0,0,0,0,0,0],[0,0,0,0,0,0,0,0,1],[0,0,0,1,0,0,0,0,1],[0,0,1,0,0,0,0,0,0],[0,0,1,0,0,0,0,0,0],[0,0,0,1,0,0,0,0,0],[0,0,1,1,0,0,0,0,0],[0,0,1,0,0,0,0,0,0],[0,0,1,0,0,0,0,0,0],[0,0,1,0,0,0,0,0,0],[0,0,1,0,0,0,0,0,0],[0,0,1,0,0,0,0,0,0],[0,0,1,0,0,0,0,0,0],[0,0,1,0,0,0,0,0,0],[0,0,1,0,0,0,0,0,0],[0,0,1,0,0,0,0,0,0],[0,0,1,0,0,0,0,0,0],[0,0,1,0,0,0,0,0,0],[1,0,1,0,0,0,0,0,0],[0,0,1,0,0,0,0,0,0],[0,0,1,0,0,0,0,0,0],[1,0,1,0,0,0,0,0,0],[0,0,1,0,0,0,0,0,0],[0,0,1,0,0,0,0,0,0],[0,0,0,0,0,0,0,0,1],[0,0,1,0,0,0,0,0,0],[0,0,1,0,0,0,0,0,0],[0,0,1,0,0,0,0,0,0],[0,0,1,0,0,0,0,0,0],[0,0,1,0,0,0,0,0,0],[0,0,1,0,0,0,0,0,0],[0,0,1,0,0,0,0,0,0],[0,0,1,0,0,0,0,0,0],[0,0,1,0,0,0,0,0,1],[0,0,1,0,0,0,0,0,0],[0,0,1,0,0,0,0,0,0],[0,0,1,0,0,0,0,0,0],[0,0,1,0,0,0,0,0,0],[1,0,1,0,0,0,0,0,0],[0,0,1,0,0,0,0,0,0],[0,0,0,0,0,0,0,0,1],[0,0,1,0,0,0,0,0,0],[0,0,1,0,0,0,0,0,0],[0,0,1,0,0,0,0,0,0],[0,0,0,0,0,0,0,0,1],[0,0,1,0,0,0,0,0,0],[0,0,1,0,0,0,0,0,0],[0,0,1,0,0,0,0,0,0],[0,0,1,0,0,0,0,0,0],[0,0,0,0,0,0,0,0,1],[0,0,0,1,0,0,0,0,0],[0,0,1,0,0,0,0,0,0],[1,0,0,0,0,0,0,0,0],[0,0,0,0,0,0,0,0,1],[1,0,0,0,0,0,0,0,0],[0,0,1,0,0,0,0,0,0],[1,0,0,0,0,0,0,0,0],[0,0,1,0,0,0,0,0,0],[1,0,0,0,0,0,0,0,0],[1,0,0,0,0,0,0,0,0],[0,0,1,0,0,0,0,0,0],[1,0,0,0,0,0,0,0,0],[1,0,0,0,0,0,0,0,0],[0,0,1,0,0,0,0,0,0],[0,0,1,0,0,0,0,0,0],[1,0,0,0,0,0,0,0,0],[1,0,0,0,0,0,0,0,0],[1,0,0,0,0,0,0,0,0],[1,0,0,0,0,0,0,0,0],[1,0,0,0,0,0,0,0,0]]8118	8119,8120,5532,1495,8121,895,8122,8123,8124,3256,8125,2334	[[0,0,0,1,0,0,0,0,0],[0,0,0,1,0,0,0,0,0],[0,0,0,1,0,0,0,0,0],[0,0,0,1,0,0,0,0,0],[0,0,0,1,0,0,0,0,0],[0,0,0,1,0,0,0,0,0],[0,0,0,1,0,0,0,0,0],[0,0,0,1,0,0,0,0,0],[0,0,0,1,0,0,0,0,0],[0,0,0,1,0,0,0,0,0],[0,0,0,1,0,0,0,0,0],[0,0,0,1,0,0,0,0,0]]8126	7973,7972	[[0,0,0,1,0,0,0,0,0],[0,0,0,1,0,0,0,0,0]]8127	4906,8128,4917,4880,2941	[[0,0,0,0,1,0,0,0,0],[0,0,0,0,1,0,0,0,0],[0,0,0,0,1,0,0,0,0],[0,0,0,0,1,0,0,0,0],[0,0,0,0,1,0,0,0,0]]8129	8130,8131,8132	[[0,0,0,1,0,0,0,0,0],[0,0,0,1,0,0,0,0,0],[0,0,0,1,0,0,0,0,0]]8133	8134,8135,8136,5266,8137,8138,8139,8140	[[0,0,0,0,1,0,0,0,0],[0,0,0,1,0,0,0,0,0],[0,0,0,0,1,0,0,0,0],[0,0,0,1,0,0,0,0,0],[0,0,0,1,1,0,0,0,0],[0,0,0,1,0,0,0,0,0],[0,0,0,1,1,0,0,0,0],[0,0,0,0,1,0,0,0,0]]8141	8142,2257,8143,826,8144,824,8145,8146,7211,7228,7216,5763,7213,7227,5767,8147,4510,7243,8148,7192,8149,2250,7267,2895,7266,7250,7278,8150,7256,7254,8151,6848,7271,8152,8153,7212,8154,7202,8155,7201,7219,7222,2245,7272,7238,7248,8156,5765,8157,8158,7231,1184,8159,7236,7189,3101,8160,7275,6781,7252,8161,7270,7215,7244,1172,8162,7261,8163,8164,8165,8166,8167,8168,701,8169,8170,5935,8171,8172,980,6850,1145,1144,6847,8173,8174,1255,8175,1157,1155,7258,7251,8176,7259,1156,8177,8178,814,8179,1171,7276,8180,8181,8182,6863,8183,8184,7214,3144,3774,1915,8185,7253,8186,1165,26,2247	[[0,0,1,0,0,0,0,0,0],[0,0,1,0,0,0,0,0,1],[0,0,1,0,0,0,0,0,0],[1,1,1,1,1,0,1,0,1],[0,0,1,0,0,0,0,0,0],[1,0,1,1,0,0,0,0,1],[1,0,1,1,0,0,0,0,0],[0,0,0,1,0,0,0,0,0],[0,0,1,0,0,0,0,0,0],[1,0,1,0,0,0,0,0,0],[0,0,1,1,0,0,0,0,1],[0,0,1,1,0,0,0,0,1],[0,0,1,0,0,0,0,0,0],[0,1,1,0,0,0,0,0,0],[0,0,1,1,0,0,0,0,1],[0,0,1,0,0,0,0,0,0],[0,0,1,0,0,0,0,0,1],[0,0,1,0,0,0,0,0,0],[0,0,1,0,0,0,0,0,0],[0,0,1,0,0,0,0,0,0],[1,0,1,1,0,0,0,0,0],[1,1,1,1,0,0,1,0,0],[0,0,1,1,0,0,0,0,1],[1,1,1,1,0,0,1,0,0],[1,1,1,0,0,0,1,0,0],[0,0,1,1,0,0,0,0,1],[0,0,1,1,0,0,0,0,0],[0,0,1,0,0,0,0,0,0],[1,0,1,1,0,0,0,0,0],[1,0,0,1,0,0,0,0,0],[0,0,1,1,0,0,0,0,0],[1,0,1,0,0,0,0,0,0],[0,1,1,1,0,0,0,0,0],[0,0,1,0,0,0,0,0,0],[0,0,1,0,0,0,0,0,0],[0,0,0,0,1,0,0,0,0],[0,0,0,0,0,0,0,0,1],[0,0,1,1,0,0,0,0,1],[0,0,1,0,0,0,0,0,0],[0,0,1,0,0,0,0,0,0],[0,0,1,1,1,0,0,0,0],[1,1,1,1,1,0,1,0,1],[0,0,1,0,0,0,0,0,0],[0,0,1,1,0,0,0,0,1],[0,0,1,0,0,0,0,0,0],[0,0,1,0,0,0,0,0,0],[0,0,0,0,0,0,0,0,1],[0,0,1,1,0,0,0,0,1],[0,0,1,0,0,0,0,0,0],[0,0,1,1,0,0,0,0,0],[1,0,1,0,0,0,0,0,0],[0,0,1,0,0,0,0,0,0],[0,1,0,0,0,0,1,0,0],[0,0,1,0,0,0,0,0,0],[0,1,0,0,0,0,0,0,0],[0,1,0,0,0,0,1,0,0],[0,1,1,0,0,0,0,0,0],[1,1,1,1,0,0,1,0,1],[0,1,1,0,0,0,0,0,0],[0,1,1,1,0,0,0,0,0],[1,1,1,0,0,0,1,0,0],[1,0,1,0,0,0,0,0,0],[1,0,0,0,0,0,0,0,0],[1,0,0,0,0,0,0,0,0],[1,0,0,0,0,0,0,0,0],[1,0,0,1,0,0,0,0,0],[1,0,1,0,0,0,0,0,0],[1,0,0,0,0,0,0,0,0],[1,0,0,0,0,0,0,0,0],[1,0,0,0,0,0,0,0,0],[1,0,0,0,0,0,0,0,0],[1,0,0,0,0,0,0,0,0],[1,0,0,0,0,0,0,0,0],[1,0,0,0,0,0,0,0,0],[1,0,0,0,0,0,0,0,0],[1,0,0,0,0,0,0,0,0],[1,0,0,0,0,0,0,0,0],[1,0,0,0,0,0,0,0,0],[1,0,0,0,0,0,0,0,0],[0,0,1,0,0,0,0,0,0],[0,0,1,0,0,0,0,0,0],[0,0,1,0,0,0,0,0,0],[0,0,1,0,0,0,0,0,0],[0,0,1,0,0,0,0,0,0],[0,0,1,0,0,0,0,0,0],[0,0,1,0,0,0,0,0,1],[0,0,1,0,0,0,0,0,0],[0,0,1,0,0,0,0,0,0],[0,0,1,0,0,0,0,0,1],[0,0,1,0,0,0,0,0,0],[0,0,1,0,0,0,0,0,0],[0,0,1,1,0,0,0,0,1],[0,0,1,0,0,0,0,0,0],[0,0,1,0,0,0,0,0,1],[0,0,0,0,0,0,0,0,1],[0,0,1,0,0,0,0,0,1],[0,0,1,0,0,0,0,0,0],[0,0,0,0,0,0,0,0,1],[0,0,1,0,0,0,0,0,0],[0,0,0,0,0,0,0,0,1],[0,0,1,0,0,0,0,0,0],[0,0,1,0,0,0,0,0,0],[0,0,1,0,0,0,0,0,0],[0,0,1,0,0,0,0,0,0],[0,0,1,0,0,0,0,0,0],[0,0,1,0,0,0,0,0,0],[1,0,0,0,0,0,0,0,0],[1,0,0,0,0,0,0,0,0],[1,0,0,0,0,0,0,0,0],[0,0,1,0,0,0,0,0,0],[0,0,1,0,0,0,0,0,0],[0,0,1,0,0,0,0,0,0],[0,0,1,1,1,0,0,0,1],[0,0,1,0,0,0,0,0,0],[0,0,1,0,0,0,0,0,0],[1,0,0,0,0,0,0,0,0],[0,0,1,0,0,0,0,0,0]]8187	3124,8188,8189,8190,8191,8192,8193,3122,8194,4234,8195,8196	[[0,0,0,0,1,0,0,0,0],[0,0,0,0,1,0,0,0,0],[0,0,0,0,1,0,0,0,0],[0,0,0,0,1,0,0,0,0],[0,0,0,0,1,0,0,0,0],[0,0,0,0,1,0,0,0,0],[0,0,0,0,1,0,0,0,0],[0,0,0,0,1,0,0,0,0],[0,0,0,0,1,0,0,0,0],[0,0,0,0,1,0,0,0,0],[0,0,0,0,1,0,0,0,0],[0,0,0,0,1,0,0,0,0]]8197	8198	[[0,0,0,0,1,0,0,0,0]]8199	8200,8201	[[0,0,0,1,0,0,0,0,0],[0,0,0,1,0,0,0,0,0]]8202	8203,8204,8205	[[0,1,0,0,0,0,0,0,0],[0,1,0,0,0,0,0,0,0],[0,1,0,0,0,0,0,0,0]]8206	8204,8207,8208,8205,8209,8203,8210,8211,8212,8213,8214	[[0,1,1,0,0,0,1,1,0],[0,0,1,0,0,0,0,0,0],[0,0,1,0,0,0,0,0,0],[0,0,1,0,0,0,0,0,0],[0,0,1,0,0,0,0,0,0],[0,1,1,0,1,0,1,1,0],[0,0,1,0,0,0,0,0,0],[0,0,1,0,0,0,0,0,0],[0,0,1,0,0,0,0,0,0],[0,0,1,0,0,0,0,0,0],[0,0,1,0,0,0,0,0,0]]8215	8216	[[0,0,0,0,1,0,0,0,0]]8217	8218	[[0,0,0,0,1,0,0,0,0]]8219	8220	[[0,0,0,0,1,0,0,0,0]]8221	8222,8223,640,8224	[[0,0,0,1,0,0,0,0,0],[0,0,0,1,0,0,0,0,0],[0,0,0,1,0,0,0,0,0],[0,0,0,1,0,0,0,0,0]]8225	1575,8226,8227	[[0,0,0,1,1,0,0,0,0],[0,0,0,1,0,0,0,0,0],[0,0,0,1,0,0,0,0,0]]8228	8229,8230,8231,8232,8233,8234,8235,8236,6273,286	[[0,0,1,1,0,0,0,0,0],[0,0,1,0,0,0,0,0,0],[0,0,1,0,0,0,0,0,0],[0,0,0,1,0,0,0,0,0],[0,1,1,0,0,0,0,0,0],[0,0,0,0,1,0,0,0,0],[0,1,1,0,0,0,0,0,0],[0,0,1,0,0,0,0,0,0],[0,0,1,0,0,0,0,0,0],[0,0,0,1,0,0,0,0,0]]8237	3302,8238,3297,8239,8240,8241,2605,2615	[[0,1,0,1,0,0,1,0,0],[0,0,0,0,1,0,0,0,0],[0,1,0,1,0,0,1,0,0],[0,1,0,1,0,0,1,0,0],[0,0,0,1,0,0,0,0,0],[0,1,0,0,0,0,1,0,0],[0,1,0,0,0,0,0,0,0],[0,1,0,0,0,0,0,0,0]]8242	8243,8244,8245,8246,8247,8248	[[0,0,0,0,1,0,0,0,0],[0,0,0,0,1,0,0,0,0],[0,0,0,0,1,0,0,0,0],[0,0,0,0,1,0,0,0,0],[0,0,0,0,1,0,0,0,0],[0,0,0,0,1,0,0,0,0]]8249	4523,8250,4522	[[0,0,0,1,0,0,0,0,0],[0,0,0,1,0,0,0,0,0],[0,0,0,1,0,0,0,0,0]]8251	927,7983,6377,2434,8252,8253,1546,7992,8254,8255,8256,2439,7991,2435,6383,1561,8257,2447,987,2252,8258,6432	[[0,0,0,0,0,0,0,1,0],[0,1,0,0,0,0,0,0,0],[0,1,0,0,0,0,0,0,0],[0,1,0,0,0,0,0,0,0],[0,1,0,0,0,0,0,0,0],[0,1,0,0,0,0,0,0,0],[0,1,0,0,0,0,0,0,0],[0,1,0,0,0,0,0,0,0],[0,1,0,0,0,0,0,0,0],[0,1,0,0,0,0,0,0,0],[0,1,0,0,0,0,0,0,0],[0,0,0,0,0,0,0,1,0],[0,1,0,0,0,0,0,1,0],[0,1,0,0,0,0,0,0,0],[0,1,0,0,0,0,0,0,0],[0,1,0,0,0,0,0,0,0],[0,1,0,0,0,0,0,0,0],[0,1,0,0,0,0,0,0,0],[0,0,0,0,0,0,0,1,0],[0,0,0,0,0,0,0,1,0],[0,1,0,0,0,0,0,0,0],[0,0,0,0,0,0,0,1,0]]8259	8260,848,8261,8262,8263,8264,8265,8266,8267,8268,8269,5501,8270,8271,8272,7911,8273	[[0,0,0,1,0,0,0,0,0],[0,0,0,1,0,0,0,0,0],[0,0,0,1,0,0,0,0,0],[0,0,0,0,1,0,0,0,0],[0,0,0,1,0,0,0,0,0],[0,0,0,1,0,0,0,0,0],[0,0,0,1,0,0,0,0,0],[0,0,0,1,0,0,0,0,0],[0,0,0,1,0,0,0,0,0],[0,0,0,1,0,0,0,0,0],[0,0,0,1,0,0,0,0,0],[0,0,0,1,0,0,0,0,0],[0,0,0,1,0,0,0,0,0],[0,0,0,1,0,0,0,0,0],[0,0,0,1,0,0,0,0,0],[0,0,0,1,0,0,0,0,0],[0,0,0,1,0,0,0,0,0]]8274	8275	[[0,0,0,0,1,0,0,0,0]]8276	8277,8278,8279,8280,8281,8282,4069,2253,8283,1086,8284,8285,8286,8287,8288,8289,8290,658,640,8291,8292,8222,8223,8293,8294,8295,8296,8224,8297,8298,8299,1044,8300,8301,8302,8303,1090,1069,2783,1070,8304,8305,8306	[[0,0,1,0,1,0,0,0,0],[0,0,1,0,0,0,0,0,0],[0,0,1,0,0,0,0,0,0],[0,1,0,0,0,0,0,0,0],[0,1,1,0,1,0,0,1,0],[0,1,1,0,0,0,0,0,0],[0,1,0,0,0,0,0,0,0],[0,1,1,0,1,0,0,1,0],[0,1,1,0,1,0,0,0,0],[0,1,1,0,0,0,0,1,0],[0,0,1,0,0,0,0,0,0],[0,0,0,0,0,0,0,1,0],[0,0,1,0,0,0,0,0,0],[0,1,1,0,0,0,0,0,0],[0,0,1,0,0,0,0,1,0],[0,0,1,0,0,0,0,0,0],[0,1,1,0,0,0,0,0,0],[0,1,1,0,0,0,0,0,0],[0,1,1,1,0,0,0,0,0],[0,0,1,0,0,0,0,1,0],[0,1,1,0,0,0,0,1,0],[0,1,1,1,1,0,0,1,0],[0,1,1,1,0,0,0,1,0],[0,1,0,0,0,0,0,0,0],[0,1,1,0,0,0,0,0,0],[0,1,0,0,0,0,0,0,0],[0,0,1,0,0,0,0,0,0],[0,0,1,1,0,0,0,1,0],[0,1,1,0,0,0,0,0,0],[0,1,1,0,0,0,0,0,0],[0,0,1,0,0,0,0,1,0],[0,1,1,0,0,0,0,1,0],[0,0,1,0,0,0,0,0,0],[0,0,0,0,1,0,0,0,0],[0,0,0,0,0,0,0,1,0],[0,0,1,0,0,0,0,0,0],[0,1,0,0,0,0,0,0,0],[0,0,1,0,0,0,0,0,0],[0,0,1,0,0,0,0,0,0],[0,1,1,0,0,0,0,0,0],[0,0,1,0,0,0,0,0,0],[0,0,1,0,0,0,0,0,0],[0,0,1,0,0,0,0,0,0]]8307	8308,8309,8310,8311,8312,8313,8314,8033,8315,8316,8317,8318,8319	[[0,0,1,0,0,0,0,0,0],[0,0,0,1,0,0,0,0,0],[0,1,0,0,0,0,0,0,0],[0,1,1,1,0,0,0,0,0],[0,0,1,1,0,0,0,0,0],[0,0,1,0,0,0,0,0,0],[0,0,1,0,0,0,0,0,0],[0,0,1,0,1,0,0,0,0],[0,0,1,0,0,0,0,0,0],[0,0,1,0,1,0,0,0,0],[0,0,0,1,0,0,0,0,0],[0,0,1,0,0,0,0,0,0],[0,0,0,0,1,0,0,0,0]]8320	8321	[[0,0,0,0,1,0,0,0,0]]8322	8323,8324,4841,2652,8325,8326,8327,8328,8329,8330,8331,8332,3774,8333,8334,8335,8336,8337,5043,8338,8339,8340,8341,8342,8343,8344,8345,8346,8347,6948,3618,8348,8349,8350	[[0,1,0,1,0,0,0,1,0],[0,1,0,0,0,0,1,1,0],[0,0,0,1,0,0,0,0,0],[0,1,0,0,0,0,1,1,0],[0,0,0,1,0,0,0,1,0],[0,0,0,1,0,0,0,0,0],[0,0,0,1,0,0,0,1,0],[0,1,0,0,0,0,1,1,0],[0,0,0,1,1,0,0,1,0],[0,1,0,0,0,0,1,1,0],[0,0,0,1,0,0,0,1,0],[0,1,0,0,0,0,1,0,0],[0,1,0,0,0,0,1,0,0],[0,0,0,1,0,0,0,0,0],[0,1,0,0,0,0,1,1,0],[0,0,0,1,0,0,0,0,0],[0,0,0,1,0,0,0,0,0],[0,1,0,0,0,0,1,1,0],[0,1,0,1,0,0,1,1,0],[0,0,0,1,0,0,0,1,0],[0,0,0,1,0,0,0,1,0],[0,0,0,1,0,0,0,1,0],[0,0,0,1,0,0,0,0,0],[0,1,0,0,0,0,1,1,0],[0,1,0,1,0,0,1,1,0],[0,0,0,1,0,0,0,0,0],[0,0,0,0,1,0,0,0,0],[0,1,0,0,0,0,1,0,0],[0,0,0,1,0,0,0,1,0],[0,1,0,1,0,0,1,1,0],[0,0,0,1,0,0,0,0,0],[0,0,0,1,0,0,0,1,0],[0,0,0,0,1,0,0,0,0],[0,0,0,1,0,0,0,0,0]]8351	8352,3237,3659,8353,8354	[[0,0,0,0,1,0,0,0,0],[0,0,0,0,1,0,0,0,0],[0,0,0,0,1,0,0,0,0],[0,0,0,0,1,0,0,0,0],[0,0,0,0,1,0,0,0,0]]8355	8356,8357	[[0,0,0,0,1,0,0,0,0],[0,0,0,0,1,0,0,0,0]]8358	8359,2827,8360,5144,8361,8362,3664,887,5930,6100,8363,5943,8364,7844,8365,5720,8366,8367,3231,3412,8368,2828,8369,8370,8371,8372,5705,8373,5931,8374,8375,8376,8377,2842,5132,6102,8378,8379,8380,8381,8382,8383,4491	[[0,0,0,1,0,0,0,1,0],[0,1,1,0,0,0,0,1,0],[0,0,1,0,0,0,0,0,0],[0,0,1,0,0,0,0,0,0],[0,1,1,1,0,0,0,1,0],[0,1,1,0,0,0,0,0,0],[0,0,1,0,0,0,0,0,0],[0,0,1,0,0,0,0,0,0],[0,0,0,1,0,0,0,0,0],[0,1,1,1,0,0,0,1,0],[0,1,1,1,0,0,0,1,0],[0,0,0,1,0,0,0,0,0],[0,0,1,0,0,0,0,0,0],[0,0,1,0,0,0,0,1,0],[0,0,1,1,0,0,0,1,0],[0,1,1,1,0,0,0,1,0],[0,0,1,0,0,0,0,0,0],[0,0,0,1,0,0,0,0,0],[0,1,1,0,0,0,0,1,0],[0,1,1,1,0,0,0,1,0],[0,0,1,0,0,0,0,0,0],[0,1,1,0,0,0,0,0,0],[0,1,1,0,0,0,0,1,0],[0,0,1,1,0,0,0,1,0],[0,0,1,0,1,0,0,0,0],[0,0,1,0,0,0,0,0,0],[0,0,0,1,0,0,0,0,0],[0,1,1,0,0,0,0,0,0],[0,0,0,1,0,0,0,1,0],[0,1,0,0,0,0,0,0,0],[0,0,0,1,0,0,0,1,0],[0,0,0,1,0,0,0,1,0],[0,1,0,0,0,0,0,0,0],[0,1,1,0,0,0,0,0,0],[0,1,1,0,0,0,0,0,0],[0,1,1,0,0,0,0,0,0],[0,0,0,0,1,0,0,0,0],[0,0,0,0,1,0,0,0,0],[0,0,1,0,0,0,0,0,0],[0,0,1,0,0,0,0,0,0],[0,1,1,1,0,0,0,0,0],[0,0,1,0,0,0,0,0,0],[0,0,1,0,0,0,0,0,0]]8384	8385,8386,8387	[[0,0,1,0,0,0,0,0,0],[0,0,1,0,0,0,0,0,0],[0,0,1,0,0,0,0,0,0]]8388	8389,5338	[[0,0,0,0,1,0,0,0,0],[0,0,0,0,1,0,0,0,0]]8390	8391,8392	[[0,0,0,0,1,0,0,0,0],[0,0,0,0,1,0,0,0,0]]8393	8394	[[0,0,0,0,1,0,0,0,0]]8395	8396,8397,8398	[[0,0,0,0,1,0,0,0,0],[0,0,0,0,1,0,0,0,0],[0,0,0,0,1,0,0,0,0]]8399	462,8400	[[0,0,0,0,1,0,0,0,0],[0,0,0,0,1,0,0,0,0]]8401	8402,8403,8404	[[0,0,0,0,1,0,0,0,0],[0,0,0,0,1,0,0,0,0],[0,0,0,0,1,0,0,0,0]]8405	4707	[[0,0,0,0,1,0,0,0,0]]8406	8407	[[0,0,0,0,1,0,0,0,0]]8408	8409,8410,8411,8412,8413	[[0,0,0,0,1,0,0,0,0],[0,0,0,0,1,0,0,0,0],[0,0,0,0,1,0,0,0,0],[0,0,0,0,1,0,0,0,0],[0,0,0,0,1,0,0,0,0]]8414	4708,8415,8416,8417,4969,8418,7482,8419,4687,8420,4077,2020,8421,4618,5404,8422,5383,8423,8424,8425,5380,8426,5384,8427,8428,5405,8429,4103,8430,8431,1993,8432,8433,8434,8435,8436,8437,5396,8438,5711,8439,8440,8441,8442,5176,4697,8443,8444,8445,8446,8447,8448,8449,8450,8451,8452,8453,8454,8455,4635,8456,4684,5992,8457,8458,7493,8459,8460,8461,5387,8462,8463,5388,4705,5277,8464,8465,8466,8467,8468,1814,1197,8469,5402,8470,2045,8471,5385,8472,5391,8473,8474,4185,8475,8476,8477,4678,8478,8479,6009,6023	[[0,0,1,0,0,0,0,1,0],[0,0,1,0,0,0,0,0,0],[0,0,1,0,0,0,0,0,0],[0,1,1,1,0,0,0,0,0],[0,0,1,0,0,0,0,0,0],[0,0,1,0,0,0,0,0,0],[0,0,1,0,0,0,0,0,0],[0,0,1,1,0,0,0,1,0],[0,0,1,1,0,0,0,1,0],[0,0,1,0,0,0,0,0,0],[0,0,1,0,0,0,0,0,0],[0,0,1,0,0,0,0,0,0],[0,0,1,0,1,0,0,0,0],[0,0,1,0,0,0,0,0,0],[0,0,1,0,0,0,0,0,0],[0,0,1,1,0,0,0,0,0],[0,0,1,1,0,0,0,1,0],[0,1,1,0,0,0,0,0,0],[0,1,1,0,0,0,0,0,0],[0,1,1,0,0,0,0,0,0],[0,0,1,0,0,0,0,0,0],[0,1,1,0,1,0,0,0,0],[0,1,1,0,0,0,0,0,0],[0,1,1,0,0,0,0,0,0],[0,1,1,0,0,0,0,0,0],[0,0,1,0,0,0,0,0,0],[0,0,1,0,0,0,0,0,0],[0,0,1,0,0,0,0,0,0],[0,0,1,0,0,0,0,0,0],[0,0,1,1,0,0,0,0,0],[0,0,1,0,0,0,0,0,0],[0,0,0,1,0,0,0,0,0],[0,0,1,0,0,0,0,0,0],[0,0,1,0,0,0,0,0,0],[0,0,0,1,0,0,0,0,0],[0,0,1,0,0,0,0,0,0],[0,0,1,0,0,0,0,0,0],[0,0,0,1,0,0,0,1,0],[0,0,1,0,0,0,0,0,0],[0,0,1,0,0,0,0,0,0],[0,0,1,0,0,0,0,0,0],[0,0,0,0,1,0,0,0,0],[0,0,1,0,0,0,0,0,0],[0,0,1,0,0,0,0,0,0],[0,0,1,0,0,0,0,1,0],[0,1,1,0,1,0,0,0,0],[0,1,1,0,0,0,0,0,0],[0,0,1,0,0,0,0,0,0],[0,0,1,0,0,0,0,0,0],[0,0,1,0,0,0,0,0,0],[0,0,1,0,0,0,0,0,0],[0,0,1,0,0,0,0,0,0],[0,0,1,0,0,0,0,0,0],[0,0,1,0,0,0,0,0,0],[0,0,1,0,0,0,0,0,0],[0,0,1,0,0,0,0,0,0],[0,1,0,0,0,0,0,0,0],[0,1,1,0,0,0,0,0,0],[0,0,1,0,0,0,0,0,0],[0,0,0,0,0,0,0,1,0],[0,0,0,1,0,0,0,0,0],[0,1,1,1,0,0,0,1,0],[0,1,1,0,0,0,0,0,0],[0,0,0,1,0,0,0,0,0],[0,0,1,1,0,0,0,0,0],[0,0,1,1,0,0,0,0,0],[0,0,1,0,0,0,0,0,0],[0,0,1,0,0,0,0,0,0],[0,0,1,0,0,0,0,0,0],[0,0,1,0,0,0,0,1,0],[0,0,1,0,0,0,0,0,0],[0,0,1,0,0,0,0,0,0],[0,1,1,1,0,0,0,0,0],[0,0,1,0,0,0,0,0,0],[0,0,1,0,0,0,0,0,0],[0,1,0,0,0,0,0,0,0],[0,0,0,0,0,0,0,1,0],[0,0,1,0,0,0,0,0,0],[0,0,1,0,0,0,0,0,0],[0,0,1,0,0,0,0,0,0],[0,0,0,0,0,0,0,1,0],[0,0,0,1,0,0,0,1,0],[0,0,1,0,0,0,0,0,0],[0,1,1,0,0,0,0,0,0],[0,0,1,0,0,0,0,0,0],[0,0,1,0,0,0,0,0,0],[0,0,0,0,1,0,0,0,0],[0,1,0,1,0,0,0,1,0],[0,0,1,0,0,0,0,0,0],[0,0,1,0,0,0,0,0,0],[0,0,1,0,0,0,0,0,0],[0,0,1,1,0,0,0,1,0],[0,0,1,0,0,0,0,0,0],[0,1,1,1,0,0,0,0,0],[0,0,1,0,0,0,0,0,0],[0,0,1,0,0,0,0,0,0],[0,0,0,1,0,0,0,1,0],[0,0,1,0,0,0,0,0,0],[0,0,1,0,0,0,0,0,0],[0,0,1,0,0,0,0,0,0],[0,0,1,0,0,0,0,0,0]]8480	8481,8482,8483,8484,8485,8486,8487,8488,8489,8490,8491,5684,8492,8493,8494,8495,8496,4884	[[0,0,0,1,0,0,0,0,0],[0,0,0,1,0,0,0,0,0],[0,0,0,1,0,0,0,0,0],[0,0,0,1,0,0,0,0,0],[0,0,0,1,1,0,0,0,0],[0,0,0,1,0,0,0,0,0],[0,0,0,1,0,0,0,0,0],[0,0,0,1,1,0,0,0,0],[0,0,0,1,0,0,0,0,0],[0,0,0,1,0,0,0,0,0],[0,0,0,1,0,0,0,0,0],[0,0,0,1,0,0,0,0,0],[0,0,0,1,1,0,0,0,0],[0,0,0,1,0,0,0,0,0],[0,0,0,1,0,0,0,0,0],[0,0,0,1,0,0,0,0,0],[0,0,0,0,1,0,0,0,0],[0,0,0,1,0,0,0,0,0]]8497	8498,4644,8499,8500	[[0,0,0,1,0,0,0,0,0],[0,0,0,1,0,0,0,0,0],[0,0,0,1,0,0,0,0,0],[0,0,0,1,0,0,0,0,0]]8501	8502,8503,8504,8505,8506,8507,8508,8509,3619,8510,8511,4545,4549	[[0,0,1,0,0,0,0,0,0],[0,0,1,0,0,0,0,0,0],[0,0,1,0,0,0,0,0,0],[0,0,1,0,0,0,0,0,0],[0,0,1,0,0,0,0,0,0],[0,0,0,1,0,0,0,0,0],[0,0,1,0,0,0,0,0,0],[0,0,1,0,0,0,0,0,0],[0,0,1,0,1,0,0,0,0],[0,0,1,0,0,0,0,0,0],[0,0,1,1,0,0,0,0,0],[0,0,1,0,0,0,0,0,0],[0,0,0,1,0,0,0,0,0]]8512	8513,8514,8515,8516	[[0,0,0,1,0,0,0,0,0],[0,0,0,1,0,0,0,0,0],[0,0,0,1,0,0,0,0,0],[0,0,0,1,0,0,0,0,0]]8517	8518,8519,8520,8521,8522,8523	[[0,0,0,0,1,0,0,0,0],[0,0,0,0,1,0,0,0,0],[0,0,0,0,1,0,0,0,0],[0,0,0,0,1,0,0,0,0],[0,0,0,0,1,0,0,0,0],[0,0,0,0,1,0,0,0,0]]8524	8525	[[0,0,0,0,1,0,0,0,0]]8526	8527	[[0,0,0,0,1,0,0,0,0]]8528	7900,4555,8529,7777,8530,7773,8531,8532,8533,8534,8535,8536,3417,8537,8538,8539,8540,8541,306,8542,1023,8543,6584	[[0,0,0,0,0,0,0,1,0],[0,0,0,0,0,0,0,1,0],[0,0,0,0,0,0,0,1,0],[0,0,0,0,0,0,0,1,0],[0,0,0,0,0,0,0,1,0],[0,0,0,0,0,0,0,1,0],[0,0,0,0,0,0,0,1,0],[0,0,0,0,0,0,0,1,0],[0,0,0,0,0,0,0,1,0],[0,0,0,0,0,0,0,1,0],[0,0,0,0,0,0,0,1,0],[0,0,0,0,0,0,0,1,0],[0,0,0,0,0,0,0,1,0],[0,0,0,0,0,0,0,1,0],[0,0,0,0,0,0,0,1,0],[0,0,0,0,0,0,0,1,0],[0,0,0,0,0,0,0,1,0],[0,0,0,0,0,0,0,1,0],[0,0,0,0,0,0,0,1,0],[0,0,0,0,0,0,0,1,0],[0,0,0,0,0,0,0,1,0],[0,0,0,0,0,0,0,1,0],[0,0,0,0,0,0,0,1,0]]8544	8545	[[0,0,0,0,1,0,0,0,0]]8546	8547,8548,8549,8550,8551,2602,8552,8553,8554,5529,8555,8556,2624,8557,8558,8559,8560,4901,2621,8561,8562,8563,8564,2611,2833,8565,8566,8567,1509,8568,8569,2746,8570,8571,8572,8573,8574,8575,3922,8576,8577,8578,5929,8579,8580,8581,2627,8582,8583,8584,8585	[[1,1,1,1,1,0,1,1,0],[0,0,0,1,0,0,0,0,0],[0,0,1,0,0,0,0,0,0],[1,0,0,0,0,0,0,1,0],[0,0,0,1,0,0,0,1,0],[0,0,1,0,0,0,0,0,0],[1,1,0,0,0,0,1,1,0],[0,0,1,0,0,0,0,0,0],[1,0,1,1,0,0,0,1,0],[1,0,1,0,0,0,0,0,0],[0,0,0,1,0,0,0,1,0],[0,0,1,0,0,0,0,0,0],[0,0,1,0,0,0,0,0,0],[1,0,0,0,0,0,0,1,0],[1,0,0,0,0,0,0,1,0],[0,0,0,1,0,0,0,1,0],[1,1,1,1,0,0,1,1,0],[1,1,0,1,0,0,0,1,0],[1,1,1,1,0,0,1,1,0],[1,1,1,1,0,0,1,1,0],[0,0,1,0,0,0,0,0,0],[1,0,0,1,0,0,0,1,0],[0,0,1,1,0,0,0,1,0],[0,0,1,0,0,0,0,0,0],[0,0,0,0,0,0,0,1,0],[1,0,1,1,0,0,0,1,0],[0,0,1,0,0,0,0,0,0],[0,0,1,0,0,0,0,0,0],[0,0,0,1,0,0,0,0,0],[0,0,1,0,0,0,0,0,0],[0,0,1,0,0,0,0,0,0],[0,0,0,0,0,0,0,1,0],[0,0,1,0,0,0,0,0,0],[0,1,1,0,0,0,1,0,0],[0,0,0,1,0,0,0,1,0],[0,0,0,1,0,0,0,1,0],[1,1,1,0,0,0,0,0,0],[1,0,0,0,0,0,0,0,0],[0,0,0,0,0,0,0,1,0],[1,0,0,0,0,0,0,0,0],[1,0,0,0,0,0,0,0,0],[0,1,1,0,0,0,0,0,0],[0,0,0,0,0,0,0,1,0],[0,0,1,0,0,0,0,0,0],[0,0,1,0,0,0,0,0,0],[0,0,1,0,0,0,0,0,0],[0,0,0,1,0,0,0,0,0],[0,0,1,0,0,0,0,0,0],[0,0,0,1,0,0,0,1,0],[0,0,1,0,0,0,0,0,0],[0,0,1,0,0,0,0,0,0]]8586	8587	[[0,0,0,0,1,0,0,0,0]]8588	8589	[[0,0,0,0,1,0,0,0,0]]8590	8591,8592,8593,3016,8594,3525,8595,1140,8596,6351,1164,8597,8598,8599,1137,8600,1554,8601,8602,8603,8604,8605,2031,2861,8606,8607,6831,8608,8609,8610,7192,8611,3062,8612,2400,1147,8613,8614,1167,8615,494,8616,8617,3813,8618,6758,1154,8619,8620,8621,8622,8623,8624,8625,8626,5056,7170,5804,5896,1143,1150,8627,8628,1170,8629,8630,1191,1142,8631,8632,1187,8633,1141,579,1139,1161,8634,8635,8636,8637,8157,3029,8638,8639,8640,8641,8642,2883,794,8643,8644,8645,2862,3974,3724,8646,8647,8648,8649,8650,8651,5591,8652,8653,1050,8654,8655,5674,8656,6809,1145,8657,8658,2261,5824,8659,8660,5814,8661,1309,8662,8663,8664,8665,2878,4261,1788,7189,8666,8667,8668,8669,5147,2887,2406,8670,1155,3219,8671,8672,1298,7207,8673,8674	[[0,0,1,0,0,1,0,0,1],[0,0,1,0,0,0,0,0,0],[0,0,1,0,0,0,0,0,0],[0,0,1,0,0,0,0,0,0],[1,1,1,1,1,1,1,0,1],[1,0,1,0,0,0,0,0,0],[0,1,0,0,0,0,1,0,0],[0,0,1,0,0,0,0,0,0],[0,0,1,0,0,0,0,0,0],[0,1,0,1,0,0,1,0,0],[1,1,0,0,0,0,1,0,0],[1,0,0,1,0,0,0,0,0],[0,1,1,0,1,0,0,0,0],[0,1,1,0,0,1,1,0,1],[0,1,1,0,0,0,0,0,0],[0,1,1,1,0,0,1,0,1],[0,1,1,0,0,0,1,0,0],[0,1,1,1,0,0,1,0,0],[0,1,0,1,0,0,0,0,0],[0,1,0,1,0,0,1,0,0],[0,1,0,0,0,0,0,0,0],[0,1,0,0,0,0,1,0,0],[0,0,1,0,0,0,0,0,0],[0,1,1,1,1,1,1,0,1],[0,0,1,0,0,0,0,0,0],[0,0,1,0,0,0,0,0,0],[0,0,1,1,0,1,0,0,0],[0,0,1,0,0,0,0,0,0],[1,1,1,0,0,0,0,0,0],[1,1,1,1,0,0,1,0,0],[0,0,1,0,0,0,0,0,0],[1,0,1,0,0,1,0,0,0],[0,0,1,0,0,0,0,0,0],[1,1,0,0,0,0,1,0,0],[0,0,1,0,0,1,0,0,1],[1,1,1,1,0,0,0,0,0],[0,0,0,1,0,0,0,0,0],[0,0,1,0,0,0,0,0,0],[0,1,1,0,0,0,0,0,0],[0,0,0,1,0,0,0,0,0],[0,0,1,0,0,0,0,0,0],[0,0,1,0,0,0,0,0,0],[0,0,1,0,0,1,0,0,0],[0,0,1,0,0,0,0,0,0],[0,0,1,0,0,0,0,0,0],[0,0,0,1,0,0,0,0,0],[1,1,1,1,0,1,1,0,0],[1,1,1,1,0,1,1,0,1],[1,0,0,0,0,0,0,0,0],[0,1,0,0,0,0,0,0,0],[0,1,0,0,0,0,0,0,0],[0,0,1,1,0,0,0,0,0],[0,1,0,1,0,0,1,0,0],[1,1,0,0,0,0,0,0,0],[0,1,1,0,0,0,0,0,0],[0,1,1,0,0,0,0,0,0],[0,1,0,0,0,0,1,0,0],[1,1,1,0,0,0,0,0,0],[0,1,0,0,0,0,0,0,0],[1,1,1,0,0,1,1,0,0],[0,1,0,0,0,0,0,0,0],[0,0,0,1,0,0,0,0,0],[0,0,1,0,0,0,0,0,0],[0,0,1,0,0,0,0,0,0],[0,1,0,0,0,0,1,0,0],[0,0,1,0,0,0,0,0,0],[0,1,1,1,0,0,0,0,0],[1,1,1,0,0,0,0,0,0],[0,1,0,0,0,0,1,0,0],[0,1,0,0,0,0,0,0,0],[0,1,0,0,0,0,0,0,0],[0,1,1,0,0,1,0,0,0],[0,1,1,1,0,0,0,0,0],[0,1,0,0,0,0,0,0,0],[0,1,1,1,0,0,0,0,0],[0,1,0,0,0,0,0,0,0],[0,0,1,0,0,0,0,0,0],[0,0,0,1,0,0,0,0,0],[0,0,1,0,0,1,0,0,0],[0,0,1,1,0,0,0,0,0],[0,0,1,0,0,0,0,0,0],[0,0,0,1,0,0,0,0,0],[0,0,1,0,0,0,0,0,0],[0,0,0,1,0,0,0,0,0],[0,1,1,0,0,0,0,0,0],[0,1,0,0,0,0,1,0,0],[0,1,0,0,0,0,1,0,0],[0,0,0,0,0,0,0,0,1],[0,1,1,1,0,0,0,0,0],[0,1,0,0,0,0,0,0,0],[0,1,0,0,0,0,0,0,0],[0,0,0,1,0,0,0,0,0],[0,0,1,1,0,0,0,0,0],[0,0,1,0,0,0,0,0,0],[0,0,1,0,0,0,0,0,0],[0,0,1,0,1,0,0,0,0],[0,0,0,1,0,0,0,0,0],[0,0,1,0,0,0,0,0,0],[0,0,1,0,0,0,0,0,0],[0,0,1,0,0,0,0,0,1],[0,0,1,0,0,0,1,0,0],[0,0,1,0,0,0,0,0,0],[0,1,0,0,0,0,0,0,0],[0,0,1,0,0,0,0,0,0],[0,0,1,0,0,0,0,0,0],[0,0,1,0,0,0,0,0,0],[0,0,1,0,0,0,0,0,0],[0,0,1,0,0,0,0,0,0],[0,0,1,0,0,0,0,0,1],[1,0,0,0,0,0,0,0,0],[1,0,1,1,0,0,0,0,0],[1,0,0,0,0,0,0,0,0],[1,0,0,0,0,0,0,0,0],[1,0,0,0,0,0,0,0,0],[1,0,1,0,0,0,0,0,0],[1,0,0,0,0,0,0,0,0],[0,0,1,0,0,0,0,0,0],[1,0,0,0,0,0,0,0,0],[0,0,1,0,0,0,0,0,0],[0,0,1,0,0,0,0,0,0],[0,0,1,0,0,0,0,0,0],[0,0,1,0,0,1,0,0,1],[0,0,0,1,0,0,0,0,0],[0,0,1,0,0,0,0,0,0],[0,0,1,0,0,0,0,0,0],[0,0,1,0,0,0,0,0,0],[0,0,1,0,0,0,0,0,0],[0,0,1,0,0,0,0,0,0],[0,0,1,0,0,0,0,0,0],[1,0,0,0,0,0,0,0,0],[0,0,1,0,0,0,0,0,0],[0,0,0,1,0,0,0,0,0],[0,0,1,0,0,0,0,0,0],[0,0,1,0,0,0,0,0,0],[0,0,0,0,0,0,0,0,1],[0,0,0,0,0,1,0,0,0],[0,0,1,0,0,0,0,0,0],[0,0,0,0,1,0,0,0,0],[0,0,0,0,0,1,0,0,0],[0,0,0,0,0,0,0,0,1],[0,0,1,0,0,0,0,0,0],[0,0,1,0,0,0,0,0,0],[0,0,0,0,0,1,0,0,0],[0,0,0,1,0,0,0,0,0]]8675	8676,1585,1587,8677,8678,1588,1602,8679,329,1568,8680,7964,1604,944,1563,1554,1555,8681,633,1573,8682,1622,1620,8683,1619,8684,8685,8686,8687,1615,8688,327,8689,8690,8691,8692,925,1553,8693,1623,8694,757,7342,8695,1548,8696,1579,1550,8697,8698,1564,1565,8699,1559,8700,1546,1582,1601,1542,815,1598,1590,8701,1578,1572,8702,8703,8704,1596,1577,8705,372,8706,333,1547,8707,1552,1567,1627,1560,8708,7025	[[1,0,1,1,1,0,0,1,0],[0,0,1,0,0,0,0,1,0],[0,0,1,0,0,0,0,0,0],[0,0,1,0,0,0,0,0,0],[0,0,1,0,0,0,0,0,0],[0,0,1,0,0,0,0,0,0],[0,1,1,0,0,0,1,1,0],[1,1,1,0,0,0,0,1,0],[0,1,1,0,0,0,0,1,0],[0,1,1,0,0,0,0,0,0],[0,0,1,0,0,0,0,0,0],[0,1,0,0,0,0,0,0,0],[0,0,1,0,0,0,0,0,0],[0,1,1,0,0,0,0,0,0],[0,1,0,0,0,0,0,0,0],[0,0,1,0,0,0,0,0,0],[0,0,1,0,0,0,0,0,0],[0,0,0,1,0,0,0,1,0],[0,0,1,0,0,0,0,0,0],[0,0,1,0,0,0,0,0,0],[0,0,1,0,0,0,0,0,0],[0,0,1,0,0,0,0,0,0],[0,0,1,0,0,0,0,0,0],[0,0,1,0,0,0,0,0,0],[0,1,1,0,0,0,0,1,0],[0,0,0,0,0,0,0,1,0],[0,0,1,0,1,0,1,1,0],[1,1,1,0,0,0,0,0,0],[0,0,1,0,0,0,0,0,0],[0,0,1,0,0,0,0,0,0],[0,0,1,0,0,0,0,0,0],[0,0,1,0,0,0,0,1,0],[0,1,1,0,0,0,0,1,0],[0,0,1,0,0,0,0,0,0],[0,0,1,0,0,0,0,0,0],[0,0,1,0,0,0,0,0,0],[0,0,1,0,0,0,0,0,0],[0,0,1,0,0,0,0,0,0],[0,0,1,0,0,0,0,0,0],[0,0,1,0,0,0,0,0,0],[0,0,1,0,0,0,0,0,0],[0,0,1,0,0,0,0,0,0],[0,0,1,0,0,0,0,0,0],[0,1,0,0,0,0,1,0,0],[0,0,1,0,0,0,0,0,0],[0,0,1,0,0,0,0,1,0],[0,0,1,0,0,0,0,0,0],[0,0,1,0,0,0,0,0,0],[0,0,0,0,0,0,0,1,0],[0,0,1,0,0,0,0,0,0],[0,0,1,0,0,0,0,0,0],[0,0,1,0,0,0,0,0,0],[0,0,1,0,0,0,0,0,0],[0,0,1,0,0,0,0,0,0],[0,0,1,0,0,0,0,0,0],[0,1,1,0,0,0,0,1,0],[1,0,1,1,0,0,0,1,0],[1,0,1,0,0,0,0,0,0],[1,1,1,0,1,0,1,1,0],[1,0,0,0,0,0,0,0,0],[0,0,1,0,0,0,0,0,0],[0,0,1,0,0,0,0,1,0],[0,0,1,0,0,0,0,0,0],[0,0,1,0,0,0,0,0,0],[0,0,1,0,0,0,0,0,0],[0,1,1,0,0,0,1,1,0],[0,1,1,0,0,0,1,1,0],[0,0,1,0,0,0,0,0,0],[0,0,1,0,0,0,0,0,0],[0,0,1,0,0,0,0,0,0],[0,0,1,0,0,0,0,0,0],[0,0,1,0,0,0,0,0,0],[0,1,1,0,0,0,0,0,0],[0,0,1,0,0,0,0,1,0],[0,0,1,0,0,0,0,0,0],[0,0,1,0,0,0,0,0,0],[0,0,1,0,0,0,0,0,0],[0,0,1,0,0,0,0,1,0],[0,0,1,0,0,0,0,0,0],[0,0,1,0,0,0,0,0,0],[0,0,1,0,0,0,0,0,0],[1,0,0,0,0,0,0,0,0]]8709	8710,8711	[[0,0,0,0,1,0,0,0,0],[0,0,0,0,1,0,0,0,0]]8712	8713	[[0,0,0,0,1,0,0,0,0]]8714	8715	[[0,0,0,0,1,0,0,0,0]]8716	8717	[[0,1,0,0,0,0,0,0,0]]8718	8719,8720,8676,4698,2032,8721,8722	[[0,0,0,1,0,0,0,0,0],[0,0,0,1,0,0,0,0,0],[0,0,0,1,0,0,0,0,0],[0,0,0,1,0,0,0,0,0],[0,0,0,1,0,0,0,0,0],[0,0,0,1,0,0,0,0,0],[0,0,0,1,0,0,0,0,0]]8723	8724	[[0,0,0,0,1,0,0,0,0]]8725	8726,7583,8727,8728,5147,8729,2861,8730,8731,1432,8732,8733,8734,8735	[[0,0,1,0,0,0,0,0,0],[0,1,1,0,0,0,0,1,0],[0,1,1,1,0,0,0,1,0],[0,1,1,0,0,0,0,1,0],[0,1,1,0,0,0,0,1,0],[0,1,1,0,0,0,0,1,0],[0,1,0,1,0,0,0,1,0],[0,0,0,0,0,0,0,1,0],[0,0,1,1,0,0,0,1,0],[0,0,0,1,0,0,0,0,0],[0,0,0,1,0,0,0,1,0],[0,0,1,0,0,0,0,0,0],[0,0,0,0,0,0,0,1,0],[0,0,1,0,0,0,0,0,0]]8736	8737,8738,8739,8740,8741,8742	[[0,1,0,0,0,0,0,0,0],[0,1,0,0,0,0,0,0,0],[0,1,0,0,0,0,0,0,0],[0,0,0,0,1,0,0,0,0],[0,0,0,0,1,0,0,0,0],[0,1,0,0,0,0,0,0,0]]8743	8744,8745,8746	[[0,0,0,1,0,0,0,0,0],[0,0,0,1,0,0,0,0,0],[0,0,0,1,0,0,0,0,0]]8747	8748,8749	[[0,0,0,0,1,0,0,0,0],[0,0,0,0,1,0,0,0,0]]8750	8751,8752,1989,8753,7632,8754,8755,8756,7189,7638,8757,8758,8759,8760,7634,3169,7633,7637,8761,8762,3201,8763,8764,1814,3082,3489,7636,7635,427	[[0,0,0,0,0,0,0,0,1],[0,0,0,0,0,0,1,0,0],[0,0,0,0,0,0,0,0,1],[0,0,0,0,0,0,0,0,1],[0,0,0,0,1,0,0,0,0],[0,0,0,0,0,0,0,0,1],[0,0,0,0,0,0,1,0,0],[0,0,0,0,0,0,1,0,1],[0,0,0,0,0,0,1,0,0],[0,0,0,0,0,0,0,0,1],[0,0,0,0,1,0,0,0,1],[0,0,0,0,0,0,1,0,1],[0,0,0,0,0,0,1,0,1],[0,0,0,0,0,0,1,0,1],[0,0,0,0,0,0,1,0,1],[0,0,0,0,0,0,1,0,0],[0,0,0,0,1,0,0,0,1],[0,0,0,0,0,0,0,0,1],[0,0,0,0,0,0,0,0,1],[0,0,0,0,0,0,1,0,1],[0,0,0,0,1,0,1,0,1],[0,0,0,0,0,0,0,0,1],[0,0,0,0,0,0,0,0,1],[0,0,0,0,1,0,0,0,0],[0,0,0,0,0,0,1,0,0],[0,0,0,0,0,0,0,0,1],[0,0,0,0,0,0,0,0,1],[0,0,0,0,1,0,0,0,1],[0,0,0,0,0,0,0,0,1]]8765	8766,8767	[[0,0,0,0,1,0,0,0,0],[0,0,0,0,1,0,0,0,0]]8768	8769,8770	[[0,0,0,0,1,0,0,0,0],[0,0,0,0,1,0,0,0,0]]8771	8772,6158	[[0,0,0,1,0,0,0,0,0],[0,0,0,1,0,0,0,0,0]]8773	7189,7942,668,8774	[[0,0,0,1,0,0,0,0,0],[0,0,0,1,0,0,0,0,0],[0,0,0,1,0,0,0,0,0],[0,0,0,1,0,0,0,0,0]]8775	2017,8722,8776,8777,8473,2032,8721,1072,1058,8778,8779,8780,8719,8781,8782,8783,1099,2035,6881,8784,8676,8785,8786,8787,4698,8788,1067,8720,8789,2055,8790,8791,8792,8793,8794,8795	[[0,1,1,0,0,0,0,1,0],[0,1,1,1,0,0,0,1,0],[0,0,1,0,0,0,0,1,0],[0,0,1,0,0,0,0,1,0],[0,0,0,0,0,0,0,1,0],[0,0,1,1,0,0,0,0,0],[0,1,0,1,0,0,0,0,0],[0,0,1,0,0,0,0,0,0],[0,0,1,0,0,0,0,0,0],[0,0,1,0,0,0,0,0,0],[0,0,1,0,0,0,0,0,0],[0,1,0,0,0,0,0,0,0],[0,0,0,1,0,0,0,0,0],[0,1,0,0,0,0,0,0,0],[0,1,0,0,0,0,0,0,0],[0,1,1,0,0,0,0,0,0],[0,0,1,0,1,0,0,0,0],[0,0,0,0,0,0,0,1,0],[0,1,0,0,0,0,0,1,0],[0,1,0,0,0,0,0,1,0],[0,1,0,1,0,0,0,0,0],[0,0,1,0,0,0,0,0,0],[0,0,0,0,0,0,0,1,0],[0,0,1,0,0,0,0,1,0],[0,0,0,1,0,0,0,0,0],[0,0,0,0,1,0,0,0,0],[0,0,1,0,0,0,0,1,0],[0,0,0,1,0,0,0,0,0],[0,0,0,0,0,0,0,1,0],[0,0,1,0,0,0,0,0,0],[0,0,0,0,0,0,0,1,0],[0,0,0,0,0,0,0,1,0],[0,0,1,0,0,0,0,0,0],[0,0,0,0,0,0,0,1,0],[0,0,1,0,0,0,0,1,0],[0,0,1,0,0,0,0,0,0]]8796	8797,8798,8799,8800,8801,8802,8803,8804,987,1544,8805,8806,8807,1550,2485,8808,8809,8810,8811,8812,8813,8676,8814,1542,8815,8816,8817,8818,5902,8819,5905,8820,8821,8822,1602,8823,8824,5893,8825,8826,8827,6144,8828,8829,8830,8831,8832,8833,8834,8835,8836,5886,7819,5862,5867,5892,8837,8089,8838,8839,8840,8088,8841,8842,8843,8844,8845,8846,8847,8848,8849,8850,8851,8852,8853,8854,8855,8856,8857,8858,8859,8860,1590,8861,8862,8863,8864,4896,4897	[[0,1,1,0,0,0,0,0,0],[1,1,1,0,0,0,0,0,0],[0,0,0,1,0,0,0,0,0],[0,1,1,0,0,0,0,0,0],[0,0,1,0,0,0,0,0,0],[0,1,1,0,1,0,0,0,0],[1,0,0,0,0,0,1,0,0],[1,1,1,1,0,1,1,0,0],[0,0,1,0,0,0,0,0,0],[0,0,1,0,0,0,0,0,0],[1,1,1,0,0,0,1,0,0],[1,1,1,1,0,1,1,0,0],[1,1,1,0,0,0,0,0,0],[1,0,1,1,0,1,0,0,0],[0,0,1,0,0,0,0,0,0],[0,0,1,0,0,0,0,0,0],[0,0,1,0,0,0,0,0,0],[0,1,1,0,0,0,0,0,0],[1,1,1,1,0,0,1,0,0],[0,1,1,1,1,1,0,0,0],[1,1,1,1,1,1,0,0,0],[1,1,1,0,0,0,1,0,0],[1,0,1,1,0,0,0,0,0],[1,1,1,1,0,1,1,0,0],[0,1,1,1,0,0,0,0,0],[0,0,1,0,0,0,0,0,0],[0,0,1,0,0,0,0,0,0],[0,0,1,0,0,0,0,0,0],[0,0,1,0,0,0,0,0,0],[0,0,1,0,0,0,0,0,0],[0,0,1,0,0,0,0,0,0],[0,0,1,0,0,0,0,0,0],[1,0,1,0,0,0,0,0,0],[0,1,1,1,0,0,0,0,0],[1,1,1,1,1,0,1,0,0],[1,1,1,1,0,0,0,0,0],[1,1,1,1,0,0,1,0,0],[0,0,1,0,0,0,0,0,0],[0,0,1,0,0,0,0,0,0],[0,0,1,0,0,1,0,0,0],[0,0,1,0,0,0,0,0,0],[0,0,1,0,0,1,1,0,0],[0,0,1,0,0,0,0,0,0],[0,0,1,0,0,0,0,0,0],[0,0,1,0,0,0,0,0,0],[1,0,0,1,0,0,0,0,0],[0,0,1,0,0,0,0,0,0],[0,0,1,0,0,0,0,0,0],[0,0,1,0,0,0,0,0,0],[0,0,1,0,0,0,0,0,0],[0,0,1,0,0,0,0,0,0],[0,0,1,0,0,0,0,0,0],[0,0,1,0,0,0,0,0,0],[0,0,1,0,0,0,0,0,0],[0,0,1,0,0,0,0,0,0],[0,0,1,0,0,0,0,0,0],[0,0,1,0,0,0,0,0,0],[0,0,1,0,0,0,0,0,0],[0,0,1,0,0,0,0,0,0],[0,0,1,0,0,0,0,0,0],[0,0,1,0,0,0,0,0,0],[0,0,1,0,0,0,0,0,0],[0,0,1,0,0,0,0,0,0],[0,0,1,0,0,0,0,0,0],[0,0,1,0,0,0,0,0,0],[0,0,1,0,0,0,0,0,0],[1,0,0,0,0,0,0,0,0],[1,0,0,0,0,0,0,0,0],[1,0,0,0,0,0,0,0,0],[1,0,0,0,0,0,0,0,0],[1,0,0,0,0,0,0,0,0],[1,0,0,0,0,0,0,0,0],[1,0,0,0,0,0,0,0,0],[1,0,0,0,0,0,0,0,0],[1,0,0,0,0,0,0,0,0],[1,0,0,0,0,0,0,0,0],[1,0,0,0,0,0,0,0,0],[1,0,0,0,0,0,0,0,0],[1,0,0,0,0,0,0,0,0],[1,0,0,0,0,0,0,0,0],[1,0,0,0,0,0,0,0,0],[1,0,0,0,0,0,0,0,0],[1,0,0,0,0,0,0,0,0],[1,0,0,0,0,0,0,0,0],[1,0,0,0,0,0,0,0,0],[1,0,0,0,0,0,0,0,0],[1,0,0,0,0,0,0,0,0],[1,0,0,0,0,0,0,0,0],[1,0,0,0,0,0,0,0,0]]8865	2861,8731,1432,8732,8727	[[0,0,0,1,0,0,0,0,0],[0,0,0,1,0,0,0,0,0],[0,0,0,1,0,0,0,0,0],[0,0,0,1,0,0,0,0,0],[0,0,0,1,0,0,0,0,0]]8866	1978,8867,2064	[[0,0,0,0,1,0,0,0,0],[0,0,0,0,1,0,0,0,0],[0,0,0,0,1,0,0,0,0]]8868	8869,2096	[[0,0,0,0,1,0,0,0,0],[0,0,0,0,1,0,0,0,0]]8870	3341,6955,3323,8871,8872,8873	[[0,0,0,0,1,0,0,0,0],[0,0,0,0,1,0,0,0,0],[0,0,0,0,1,0,0,0,0],[0,0,0,0,1,0,0,0,0],[0,0,0,0,1,0,0,0,0],[0,0,0,0,1,0,0,0,0]]8874	8875,8876,8877,8878	[[0,0,0,0,1,0,0,0,0],[0,0,0,0,1,0,0,0,0],[0,0,0,0,1,0,0,0,0],[0,0,0,0,1,0,0,0,0]]8879	8880	[[0,0,0,0,1,0,0,0,0]]8881	8882,2653,8883,8884,8885,8886,4275,513,8887,8888,8889,657,637,8890,649,4678,8891,8892,8893,8894,8895,8896,4491,8897,8898,8899,8900,8901,8902,659,8903,651,8904,4225,8905,4644,8906,8907,8908,8909,8910,8911,8912,8913,8914,1339,643,5406,8915,8916,8055,4648,650,8917,8918,8919,5407,8920,3431,3439,8921,8922,8923,8924,8925,8926,8927,5934,5394,8928,8929,4388,8930,4296	[[0,1,1,1,1,0,0,1,0],[0,0,1,0,0,0,0,0,0],[0,1,1,0,0,0,0,1,0],[0,0,1,0,0,0,0,0,0],[0,0,1,0,0,0,0,0,0],[0,0,1,0,0,0,0,0,0],[0,1,0,0,0,0,0,0,0],[0,1,1,1,0,0,0,1,0],[0,1,0,0,0,0,0,0,0],[0,1,1,0,0,0,0,1,0],[0,1,1,0,0,0,0,0,0],[0,1,1,0,0,0,0,0,0],[0,1,1,0,0,0,0,0,0],[0,0,1,0,0,0,0,0,0],[0,1,1,0,1,0,0,0,0],[0,1,1,0,0,0,0,1,0],[0,1,0,0,0,0,0,1,0],[0,1,1,0,0,0,0,1,0],[0,1,0,0,0,0,0,0,0],[0,1,1,0,1,0,0,1,0],[0,1,1,1,1,0,0,0,0],[0,1,1,0,0,0,0,0,0],[0,0,1,0,0,0,0,0,0],[0,0,1,0,0,0,0,0,0],[0,0,1,0,0,0,0,0,0],[0,0,1,0,0,0,0,0,0],[0,0,1,0,0,0,0,0,0],[0,0,1,1,0,0,0,1,0],[0,0,1,0,0,0,0,0,0],[0,0,1,0,0,0,0,0,0],[0,0,0,0,0,0,0,1,0],[0,0,1,0,0,0,0,0,0],[0,0,0,0,0,0,0,1,0],[0,0,1,0,0,0,0,0,0],[0,0,1,0,0,0,0,0,0],[0,0,1,0,0,0,0,0,0],[0,0,1,0,0,0,0,0,0],[0,0,1,0,0,0,0,0,0],[0,0,1,0,0,0,0,0,0],[0,0,1,0,0,0,0,0,0],[0,0,1,0,0,0,0,0,0],[0,0,1,0,0,0,0,0,0],[0,0,1,0,0,0,0,1,0],[0,0,1,0,0,0,0,0,0],[0,0,1,0,0,0,0,0,0],[0,0,1,0,0,0,0,0,0],[0,0,1,0,0,0,0,0,0],[0,0,1,0,0,0,0,0,0],[0,0,1,0,0,0,0,1,0],[0,0,1,0,0,0,0,0,0],[0,0,1,0,0,0,0,0,0],[0,0,1,0,0,0,0,0,0],[0,0,1,0,0,0,0,0,0],[0,0,1,0,0,0,0,0,0],[0,0,1,0,0,0,0,0,0],[0,0,1,0,1,0,0,1,0],[0,0,1,0,0,0,0,0,0],[0,0,1,0,0,0,0,0,0],[0,0,1,0,0,0,0,0,0],[0,0,1,0,0,0,0,0,0],[0,0,1,0,0,0,0,0,0],[0,0,1,0,0,0,0,0,0],[0,0,1,0,0,0,0,0,0],[0,0,1,0,0,0,0,0,0],[0,0,1,0,0,0,0,0,0],[0,0,1,0,0,0,0,0,0],[0,0,1,0,0,0,0,1,0],[0,0,1,0,0,0,0,0,0],[0,0,1,0,0,0,0,0,0],[0,0,0,0,0,0,0,1,0],[0,0,1,0,0,0,0,0,0],[0,0,1,0,0,0,0,0,0],[0,0,1,0,0,0,0,0,0],[0,0,1,0,0,0,0,0,0]]8931	2005,8932,8933	[[0,0,0,1,0,0,0,0,0],[0,0,0,1,0,0,0,0,0],[0,0,0,1,0,0,0,0,0]]8934	1555,8935,8936,8937,8938,8939,1554,8940,8941,3664,944,8942,2528,8943,2129,5862,8944,8945,8946	[[0,1,1,0,0,0,0,0,0],[0,0,1,0,0,0,0,0,0],[0,0,1,0,0,0,0,0,0],[0,0,1,0,0,0,0,0,0],[0,0,1,0,0,0,0,0,0],[0,0,1,0,0,0,0,0,0],[0,1,1,0,0,0,0,0,0],[0,0,1,0,0,0,0,0,0],[0,0,1,0,0,0,0,0,0],[0,0,0,1,0,0,0,0,0],[0,0,1,0,0,0,0,0,0],[0,0,1,0,0,0,0,0,0],[0,1,1,0,0,0,0,0,0],[0,1,1,0,0,0,0,0,0],[0,1,1,1,0,0,0,0,0],[0,1,1,0,0,0,0,0,0],[0,0,1,0,0,0,0,0,0],[0,1,1,0,0,0,0,0,0],[0,1,0,0,0,0,0,0,0]]8947	861,8948,8949,6342,6353,8950,6351,7146,8951,8952,7192,8269,8953,8954,8366,8955,6340,8956,942,8957,8958,6344,5042,7169,8959,8960,2297,4440,8961,8962,8963,2530,8964,8965,859,8966,8967,8665,8968,8969,8970,8971,8972,8717,8973,8974,8975,8976,8977,8978,8979	[[0,0,0,0,0,0,1,0,0],[0,1,1,0,0,0,0,0,0],[0,1,1,0,0,0,1,0,0],[0,1,1,0,0,0,1,0,0],[0,1,1,0,0,0,1,0,0],[0,1,1,1,0,0,1,0,0],[0,1,1,1,0,0,1,0,0],[0,1,1,1,0,0,0,0,0],[0,1,1,1,0,0,1,0,0],[0,0,1,0,0,0,0,0,0],[0,0,1,0,0,0,0,0,0],[0,1,1,0,0,0,1,0,0],[0,1,0,0,0,0,1,0,0],[0,0,0,0,0,0,1,0,0],[0,0,1,0,0,0,0,0,0],[0,0,1,0,0,0,0,0,0],[0,0,1,0,0,0,0,0,0],[0,0,1,0,0,0,0,0,0],[0,0,1,0,0,0,0,0,0],[0,0,1,0,0,0,0,0,0],[0,0,1,0,0,0,0,0,0],[0,0,1,0,0,0,0,0,0],[0,0,1,0,0,0,0,0,0],[0,0,0,0,1,0,0,0,0],[0,0,1,0,0,0,0,0,0],[0,0,1,0,0,0,0,0,0],[0,0,1,0,0,0,0,0,0],[0,0,1,0,0,0,0,0,0],[0,0,1,0,0,0,0,0,0],[0,0,1,0,0,0,0,0,0],[0,0,1,0,0,0,0,0,0],[0,0,1,0,0,0,0,0,0],[0,0,1,0,0,0,0,0,0],[0,0,1,0,0,0,0,0,0],[0,0,0,0,0,0,1,0,0],[0,0,0,0,1,0,0,0,0],[0,0,1,0,0,0,1,0,0],[0,0,1,0,0,0,0,0,0],[0,0,1,0,0,0,0,0,0],[0,0,0,0,1,0,0,0,0],[0,0,0,0,1,0,0,0,0],[0,0,1,0,0,0,1,0,0],[0,0,1,0,0,0,0,0,0],[0,0,1,0,0,0,0,0,0],[0,0,1,0,0,0,0,0,0],[0,0,1,0,0,0,0,0,0],[0,0,1,0,0,0,0,0,0],[0,0,1,0,0,0,0,0,0],[0,0,0,0,0,0,1,0,0],[0,0,1,0,0,0,0,0,0],[0,0,1,0,0,0,0,0,0]]8980	8981,8982,8983,8984,3275,8985,8986,8987,5535,8988,1463,8989,8990,8991,1460,8992,8993,8994,8995,8996,8997,2612,8998,1170,8999,9000,9001,9002,2611,8241,9003,1471,9004,9005,9006,9007,2858,9008,9009,9010,9011,8541,9012,9013,9014,9015,9016,9017,9018,9019,1468,9020	[[0,0,1,0,0,0,0,0,0],[0,0,1,0,0,0,0,0,0],[0,0,1,0,0,0,0,0,0],[0,0,1,0,0,0,0,0,0],[0,0,0,1,0,0,0,0,0],[0,0,1,0,0,0,0,0,0],[0,0,1,0,0,0,0,0,0],[0,0,1,0,0,0,0,0,0],[0,0,1,0,0,0,0,0,0],[0,0,1,0,0,0,0,0,0],[0,1,1,0,0,0,0,0,0],[0,0,1,0,0,0,0,0,0],[0,0,1,0,0,0,0,0,0],[0,0,1,0,0,0,0,0,0],[0,1,1,0,0,0,0,0,0],[0,1,1,0,0,0,0,0,0],[0,0,1,0,0,0,0,0,0],[0,0,1,0,0,0,0,0,0],[0,0,1,0,0,0,0,0,0],[0,0,1,0,0,0,0,0,0],[0,1,1,1,0,0,0,0,0],[0,0,1,0,0,0,0,0,0],[0,0,1,0,0,0,0,0,0],[0,0,1,0,0,0,0,0,0],[0,0,1,0,0,0,0,0,0],[0,0,1,0,0,0,0,0,0],[0,0,1,0,0,0,0,0,0],[0,0,1,0,0,0,0,0,0],[0,0,1,0,0,0,0,0,0],[0,0,1,0,0,0,0,0,0],[0,0,1,0,0,0,0,0,0],[0,1,1,0,0,0,0,0,0],[0,0,1,0,0,0,0,0,0],[0,1,1,1,1,0,0,0,0],[0,1,1,0,0,0,0,0,0],[0,0,1,0,0,0,0,0,0],[0,0,1,0,0,0,0,0,0],[0,0,1,0,0,0,0,0,0],[0,0,1,0,0,0,0,0,0],[0,0,1,0,0,0,0,0,0],[0,1,0,0,0,0,0,0,0],[0,0,1,1,1,0,0,0,0],[0,0,1,0,0,0,0,0,0],[0,1,1,0,1,0,0,0,0],[0,1,0,0,0,0,0,0,0],[0,1,0,0,0,0,0,0,0],[0,0,1,0,0,0,0,0,0],[0,0,1,0,0,0,0,0,0],[0,0,1,0,0,0,0,0,0],[0,0,1,0,0,0,0,0,0],[0,0,1,0,0,0,0,0,0],[0,0,1,0,0,0,0,0,0]]9021	9022,9023,9024,9025,9026,9027,9028,9029,9030,6463,9031,6908,9032,9033,9034,9035	[[0,0,1,0,1,0,0,0,0],[0,0,1,0,1,0,0,0,0],[0,0,1,0,1,0,0,0,0],[0,0,1,0,0,0,0,0,0],[0,0,1,0,0,0,0,0,0],[0,0,1,0,0,0,0,0,0],[0,0,1,0,1,0,0,0,0],[0,0,1,0,0,0,0,0,0],[0,0,0,1,0,0,0,0,0],[0,0,1,0,1,0,0,0,0],[0,0,1,0,0,0,0,0,0],[0,0,0,0,1,0,0,0,0],[0,0,0,0,1,0,0,0,0],[0,0,1,0,0,0,0,0,0],[0,0,0,1,0,0,0,0,0],[0,0,0,1,0,0,0,0,0]]9036	9037,9038,9039	[[0,0,0,1,0,0,0,0,0],[0,0,0,1,0,0,0,0,0],[0,0,0,1,0,0,0,0,0]]9040	9041,3682,8968,8269,9042,9043,9044,2480,8960,307,9045,9046,7996,9047,9048,7062,8965,9049,4440,9050,9051,8958,8950,5745,8253,9052,7989,8717,9053,9054,9055,8637,9056,2530,6087,9057,8973,9058,8665,9059,9060,9061,9062,9063,9064,8979,3892,9065,8184,5042,9066,9067,9068,9069,9070,9071,9072,9073,9074,9075,9076,2581,8964,9077,9078,9079,9080,9081,9082,2352,8962,9083,9084,9085	[[0,1,0,0,0,0,1,0,0],[0,1,0,0,0,0,0,0,0],[0,1,1,1,1,0,1,0,0],[0,1,1,1,0,0,1,0,0],[0,1,0,0,0,0,1,0,0],[0,1,0,0,0,0,0,0,0],[0,1,1,1,0,0,1,0,0],[0,1,0,0,0,0,0,0,0],[0,1,1,0,0,0,0,0,0],[0,1,0,1,0,0,1,0,0],[0,1,0,0,0,0,1,0,0],[0,1,1,0,0,0,0,0,0],[0,1,1,0,0,0,0,0,0],[0,1,1,0,0,0,0,0,0],[0,1,0,0,0,0,0,0,0],[0,1,0,0,0,0,0,0,0],[0,1,1,0,0,0,0,0,0],[0,1,1,0,0,0,0,0,0],[0,1,1,1,0,0,0,0,0],[0,1,1,1,0,0,0,0,0],[0,1,0,0,0,0,0,0,0],[0,1,0,0,0,0,0,0,0],[0,1,0,1,0,0,1,0,0],[0,1,1,0,0,0,1,0,0],[0,1,1,0,0,0,0,0,0],[0,1,1,1,0,0,1,0,0],[0,1,0,0,0,0,0,0,0],[0,1,1,1,0,0,0,0,0],[0,1,1,1,0,0,0,0,0],[0,1,0,0,0,0,0,0,0],[0,0,1,0,0,0,0,0,0],[0,0,1,0,0,0,0,0,0],[0,0,1,0,0,0,0,0,0],[0,1,0,1,0,0,0,0,0],[0,0,1,0,0,0,0,0,0],[0,0,1,0,0,0,1,0,0],[0,0,1,0,0,0,0,0,0],[0,0,1,0,0,0,0,0,0],[0,1,1,0,0,0,0,0,0],[0,0,1,0,0,0,0,0,0],[0,0,0,1,0,0,0,0,0],[0,1,0,0,0,0,0,0,0],[0,0,1,0,0,0,0,0,0],[0,0,1,0,0,0,0,0,0],[0,1,0,0,0,0,0,0,0],[0,1,1,0,0,0,0,0,0],[0,0,1,0,0,0,0,0,0],[0,0,1,0,0,0,0,0,0],[0,0,1,0,0,0,0,0,0],[0,0,1,0,0,0,0,0,0],[0,0,1,0,0,0,0,0,0],[0,0,1,0,0,0,0,0,0],[0,1,1,0,0,0,0,0,0],[0,0,1,0,0,0,0,0,0],[0,0,1,0,0,0,1,0,0],[0,0,1,0,0,0,0,0,0],[0,0,1,0,0,0,0,0,0],[0,0,1,0,0,0,0,0,0],[0,0,1,0,0,0,0,0,0],[0,0,1,0,0,0,0,0,0],[0,0,1,0,0,0,0,0,0],[0,1,0,0,0,0,0,0,0],[0,1,0,0,0,0,0,0,0],[0,0,1,0,0,0,0,0,0],[0,0,1,0,0,0,0,0,0],[0,0,1,0,0,0,0,0,0],[0,0,1,0,0,0,0,0,0],[0,0,1,0,0,0,0,0,0],[0,0,1,0,0,0,0,0,0],[0,0,1,0,0,0,0,0,0],[0,0,1,0,0,0,0,0,0],[0,1,0,0,0,0,0,0,0],[0,0,1,0,0,0,0,0,0],[0,0,1,0,0,0,0,0,0]]9086	9087,9088	[[0,0,0,0,1,0,0,0,0],[0,0,0,0,1,0,0,0,0]]9089	8763,4639,4673,4454,4466,4460,9090,4508,3853,4701,2635,4452,9091,4475,9092,4473,4457,9093,3857,3871,3797,9094,9095,9096,9097,6022,4464,9098,7635,9099,9100,9101,9102,4619,423,3489,1094,9103,9104,3369,4494,8764,8761,4480,9105,6006,3856,3860,2677,9106,5981,9107,3858,3864,9108,8754,9109,1603,9110,7633,6219,2790,9111,3861,6155,1028,6215,4506,5996,9112,4488,4487,9113,3633,9114,9115,9116,7678,9117,9118,3851,1443,9119,4306,9120,9121,9122,3850,9123,4465,9124,9125,9126,9127,427,5977,9128,431,9129,4499,1286,9130,9131,9132,4467,9133,9134,9135,9136,9137,9138,9139,2027,1585,9140,9141,3446,1031,9142,9143,8684,9144,1591,660	[[1,1,1,0,0,0,1,0,1],[0,0,0,0,0,0,0,0,1],[0,0,0,0,0,0,0,1,1],[0,0,1,0,0,0,0,1,0],[1,0,1,0,0,0,0,0,0],[1,0,1,0,0,0,0,1,0],[1,0,1,0,0,0,0,0,0],[0,0,1,0,1,0,0,0,0],[1,1,1,1,1,1,1,1,0],[0,0,0,0,0,0,0,1,0],[0,0,1,0,0,0,0,0,0],[1,0,1,0,0,0,0,0,0],[0,0,1,0,0,0,0,0,0],[1,0,1,0,0,0,0,0,0],[0,0,1,0,0,0,0,0,0],[1,0,1,0,0,0,0,1,1],[0,0,1,0,0,0,0,0,0],[1,0,0,0,0,0,0,0,0],[1,0,1,1,0,0,0,1,0],[1,1,1,0,0,1,1,0,0],[1,0,0,0,0,0,0,0,0],[1,0,1,0,0,0,0,0,0],[1,0,0,0,0,0,0,0,0],[1,0,0,0,0,0,0,0,0],[0,0,1,0,0,0,0,0,0],[1,0,0,0,0,0,0,0,0],[1,0,1,0,0,0,0,0,0],[0,0,0,0,0,0,0,0,1],[0,0,1,0,0,0,0,0,0],[0,0,0,0,0,0,0,1,0],[0,0,1,0,0,0,0,0,0],[0,0,1,0,0,0,0,0,0],[0,0,0,0,0,0,0,0,1],[0,0,0,0,0,0,0,0,1],[1,1,1,0,0,0,0,1,0],[1,0,1,0,0,0,0,0,0],[0,1,1,1,0,1,0,1,0],[0,0,1,0,0,0,0,0,0],[0,0,1,0,0,0,0,0,0],[0,0,0,0,0,0,0,0,1],[0,0,1,0,0,0,0,0,0],[1,0,1,0,0,0,0,0,0],[1,0,1,1,0,0,0,0,0],[1,0,1,0,0,0,0,1,0],[0,0,0,0,0,0,0,0,1],[0,0,0,0,0,0,0,0,1],[0,0,0,0,0,0,0,1,0],[1,1,1,1,0,1,0,0,0],[0,0,1,0,0,0,0,0,0],[1,1,1,1,0,0,1,0,1],[0,0,0,0,0,0,0,0,1],[0,0,1,0,0,0,0,0,0],[1,1,1,1,0,0,0,1,0],[0,1,0,0,0,0,0,1,0],[0,0,0,0,0,0,0,1,0],[1,1,1,0,0,0,1,1,0],[0,0,1,0,0,0,0,0,0],[0,0,0,0,0,0,0,0,1],[0,0,1,0,0,0,0,0,0],[0,0,1,0,0,0,0,0,0],[0,0,1,0,0,0,0,0,0],[0,0,1,0,0,0,0,0,0],[1,0,0,0,0,0,0,0,0],[1,1,1,1,0,0,1,1,0],[1,0,1,0,0,0,0,0,0],[1,0,0,0,0,0,0,0,0],[0,0,1,0,0,0,0,0,0],[0,0,1,0,0,0,0,0,0],[0,0,0,1,1,0,0,1,1],[0,0,0,0,1,0,0,0,0],[0,0,0,0,1,0,0,0,0],[0,0,1,0,0,0,0,0,0],[1,0,1,0,0,1,0,0,0],[1,1,1,1,1,0,1,0,0],[1,1,0,0,0,0,0,0,0],[1,0,0,0,0,0,0,0,0],[0,0,0,0,0,0,0,0,1],[1,0,0,1,0,0,0,1,1],[0,0,1,0,0,0,0,0,0],[0,0,0,0,0,0,0,0,1],[0,0,0,0,0,1,0,0,0],[1,0,1,1,0,0,0,0,0],[1,0,0,0,0,0,0,0,0],[1,0,0,0,0,0,0,0,0],[1,0,0,0,0,0,0,0,0],[1,0,0,0,0,0,0,0,0],[1,0,0,0,0,0,0,0,0],[1,0,1,0,0,1,0,1,1],[1,0,0,0,0,0,0,0,0],[1,0,1,1,0,0,0,0,0],[1,0,0,0,0,0,0,0,0],[1,0,0,0,0,0,0,0,0],[1,0,0,0,0,0,0,0,0],[1,0,1,1,0,0,0,0,0],[1,0,0,0,0,0,0,0,0],[1,0,0,0,0,0,0,0,0],[1,0,0,0,0,0,0,0,0],[1,0,0,0,0,0,0,0,0],[1,0,1,0,0,0,0,0,0],[0,0,1,0,0,0,0,0,0],[0,0,1,0,0,0,0,0,0],[0,0,0,0,0,0,0,0,1],[1,0,0,0,0,0,0,0,0],[0,0,0,0,0,0,0,0,1],[0,0,1,0,0,0,0,0,0],[1,0,0,0,0,0,0,0,0],[1,0,0,0,0,0,0,0,0],[1,0,0,0,0,0,0,0,0],[1,0,0,0,0,0,0,0,0],[1,0,0,0,0,0,0,0,0],[1,0,0,0,0,0,0,0,0],[1,0,0,0,0,0,0,0,0],[1,0,0,0,0,0,0,0,0],[1,0,0,0,0,0,0,0,0],[0,0,0,0,0,0,0,0,1],[1,0,0,0,0,0,0,0,0],[1,0,0,0,0,0,0,0,0],[1,0,0,0,0,0,0,0,0],[1,0,0,0,0,0,0,0,0],[1,0,0,0,0,0,0,0,0],[1,0,0,0,0,0,0,0,0],[1,0,0,0,0,0,0,0,0],[1,0,0,0,0,0,0,0,0],[1,0,0,0,0,0,0,0,0]]9145	9146,9147,9148	[[0,0,0,0,1,0,0,0,0],[0,0,0,0,1,0,0,0,0],[0,0,0,0,1,0,0,0,0]]9149	2870,2863,9150,9151,2543,8543,2542,9152,2484,2877,2424,9153,9154	[[0,0,0,0,0,0,1,0,0],[0,0,1,1,0,0,0,1,0],[0,0,1,0,0,0,0,0,0],[0,0,1,1,0,0,1,1,0],[0,0,0,0,0,0,1,1,0],[0,0,0,0,0,0,0,1,0],[0,0,0,0,0,0,0,1,0],[0,0,0,0,0,0,0,1,0],[0,0,0,0,0,0,0,1,0],[0,0,0,0,0,0,0,1,0],[0,0,1,1,0,0,1,1,0],[0,0,0,0,0,0,0,1,0],[0,0,0,0,0,0,0,1,0]]9155	9156	[[0,0,0,0,1,0,0,0,0]]9157	9158,9159,5407,5394,9160,9161,8930,9162,9163,5392,8924,9164,5992,2922,9165,9166,9167,9168,9169,9170,8576,4623,9171	[[0,0,1,0,0,0,0,0,0],[0,1,1,0,0,0,1,0,0],[0,1,1,1,0,0,1,0,0],[0,1,1,0,0,0,1,0,0],[0,0,1,0,0,0,0,0,0],[0,0,1,0,0,0,0,0,0],[0,1,1,0,0,0,1,0,0],[0,0,1,0,0,0,0,0,0],[0,0,1,0,0,0,0,0,0],[0,1,0,1,0,0,1,0,0],[0,1,0,0,0,0,1,0,0],[0,0,1,0,0,0,0,0,0],[0,0,1,0,0,0,0,0,0],[0,0,1,0,0,0,0,0,0],[0,0,1,0,0,0,0,0,0],[0,0,1,0,0,0,0,0,0],[0,1,0,0,0,0,0,0,0],[0,1,0,0,0,0,0,0,0],[0,1,1,1,0,0,1,0,0],[0,0,1,0,0,0,0,0,0],[0,0,1,0,0,0,0,0,0],[0,0,1,0,0,0,0,0,0],[0,0,1,0,0,0,0,0,0]]9172	9173,9174,1587,9175,4617,9176,2398,9177,9178,9179,5990,9180,9181,9182,9183,9184,9035,9030,9185,9034,9186,9187,9188,9189,9190,9191,3082,4103,9192,4612	[[0,1,1,0,0,0,0,1,0],[0,0,0,0,0,0,0,1,0],[0,1,1,0,0,0,0,1,0],[0,0,1,0,0,0,0,0,0],[0,0,0,0,0,0,0,1,0],[0,1,1,0,0,0,0,0,0],[0,1,1,0,1,0,0,1,0],[0,1,1,0,0,0,0,1,0],[0,1,1,0,0,0,0,0,0],[0,1,1,0,0,0,0,0,0],[0,0,1,0,0,0,0,1,0],[0,0,0,0,0,0,0,1,0],[0,1,0,0,0,0,0,1,0],[0,0,1,0,0,0,0,0,0],[0,1,1,0,0,0,0,1,0],[0,1,0,0,0,0,0,0,0],[0,1,1,1,1,0,0,1,0],[0,1,1,1,0,1,0,1,0],[0,1,0,0,0,0,0,0,0],[0,1,1,1,1,1,0,1,0],[0,1,0,0,0,0,0,0,0],[0,0,1,0,0,0,0,1,0],[0,0,1,0,0,0,0,0,0],[0,0,0,0,0,0,0,1,0],[0,0,1,0,0,0,0,0,0],[0,0,0,0,0,0,0,1,0],[0,0,1,0,0,0,0,0,0],[0,0,1,0,0,0,0,0,0],[0,0,1,0,0,0,0,0,0],[0,0,0,0,0,0,0,1,0]]9193	9194,9195,9196,9197,9198,9199,9200,9201,6516,9202,9203,9204	[[0,0,0,0,1,0,0,0,0],[0,0,0,0,1,0,0,0,0],[0,0,0,0,1,0,0,0,0],[0,0,0,0,1,0,0,0,0],[0,0,0,0,1,0,0,0,0],[0,0,0,0,1,0,0,0,0],[0,0,0,0,1,0,0,0,0],[0,0,0,0,1,0,0,0,0],[0,0,0,0,1,0,0,0,0],[0,0,0,0,1,0,0,0,0],[0,0,0,0,1,0,0,0,0],[0,0,0,0,1,0,0,0,0]]9205	9206,9207,9208,9209,9210,9211,9212,9213,9214,9215,9216,9217,9218,9219,9220,9221	[[0,0,0,0,1,0,0,0,0],[0,0,0,0,1,0,0,0,0],[0,0,0,0,1,0,0,0,0],[0,0,0,0,1,0,0,0,0],[0,0,0,0,1,0,0,0,0],[0,0,0,0,1,0,0,0,0],[0,0,0,0,1,0,0,0,0],[0,0,0,0,1,0,0,0,0],[0,0,0,0,1,0,0,0,0],[0,0,0,0,1,0,0,0,0],[0,0,0,0,1,0,0,0,0],[0,0,0,0,1,0,0,0,0],[0,0,0,0,1,0,0,0,0],[0,0,0,0,1,0,0,0,0],[0,0,0,0,1,0,0,0,0],[0,0,0,0,1,0,0,0,0]]9222	329,8680,8704,9223,4988,1597,3239,1566,90,9224,2835,3776,5123,9225,9226,1604,9227,1554,8692,9228,9229,925,3215,9230,5075,2711,1603,2129,9231,6362,9232,1555,9233,632,9234,9235,8131,7820,7842,6381,6387	[[0,0,0,0,0,0,0,1,0],[0,0,1,0,0,0,0,0,0],[0,1,1,0,0,0,1,0,0],[0,0,1,0,0,0,0,1,0],[0,0,1,0,0,0,0,0,0],[0,0,1,0,0,0,0,0,0],[0,0,1,0,0,0,0,0,0],[0,0,0,0,0,0,0,1,0],[0,0,1,0,0,0,0,0,0],[0,0,1,0,0,0,0,0,0],[0,1,0,0,0,0,0,0,0],[0,1,0,0,0,0,0,0,0],[0,1,0,0,0,0,1,0,0],[0,1,0,0,0,0,1,0,0],[0,0,1,0,0,0,0,0,0],[0,0,0,0,0,0,0,1,0],[0,1,1,0,0,0,1,0,0],[0,1,1,0,0,0,1,1,0],[0,0,0,0,0,0,0,1,0],[0,0,1,0,0,0,0,0,0],[0,1,0,0,0,0,1,1,0],[0,0,1,0,0,0,0,0,0],[0,0,1,0,0,0,0,0,0],[0,0,0,0,1,0,0,0,0],[0,0,1,0,0,0,0,0,0],[0,0,0,0,1,0,0,0,0],[0,0,0,0,0,0,0,1,0],[0,1,1,0,0,0,1,0,0],[0,0,1,0,0,0,0,0,0],[0,1,0,0,0,0,0,0,0],[0,1,0,0,0,0,0,0,0],[0,1,1,0,0,0,1,1,0],[0,0,1,0,0,0,0,0,0],[0,0,1,0,0,0,0,0,0],[0,1,0,0,0,0,1,1,0],[0,0,1,0,0,0,0,0,0],[0,1,0,0,0,0,0,0,0],[0,1,0,0,0,0,0,0,0],[0,1,0,0,0,0,0,0,0],[0,1,0,0,0,0,0,0,0],[0,1,0,0,0,0,0,0,0]]9236	9237,9238,9239	[[0,0,0,0,1,0,0,0,0],[0,0,0,0,1,0,0,0,0],[0,0,0,0,1,0,0,0,0]]9240	9241,9242,9243,9244,9071,9245,2530,9246,9247,9068,4440,8973,7171,9248,9075,2581,8963,2520,9249,9062,9250,3729,9046	[[0,0,1,0,0,0,0,0,0],[0,0,1,0,0,0,0,0,0],[0,0,1,0,0,0,0,0,0],[0,0,1,0,0,0,0,0,0],[0,0,1,0,1,0,0,0,0],[0,0,1,0,0,0,0,0,0],[0,0,1,0,0,0,0,0,0],[0,0,1,0,0,0,0,0,0],[0,0,1,0,0,0,0,0,0],[0,0,1,0,0,0,0,0,0],[0,0,1,0,0,0,0,0,0],[0,0,1,0,0,0,0,0,0],[0,0,1,0,0,0,0,0,0],[0,0,1,0,0,0,0,0,0],[0,0,1,0,0,0,0,0,0],[0,0,1,0,0,0,0,0,0],[0,0,1,0,0,0,0,0,0],[0,0,1,0,0,0,0,0,0],[0,0,0,0,1,0,0,0,0],[0,0,1,0,0,0,0,0,0],[0,0,1,0,0,0,0,0,0],[0,0,1,0,0,0,0,0,0],[0,0,1,0,0,0,0,0,0]]9251	9252,9253,9254,9255,5540,9256,9257,9258,6872,9259,9260,9261,9262,9263,3343	[[0,0,0,1,0,0,0,0,0],[0,0,0,0,1,0,0,0,0],[0,0,0,1,1,0,0,0,0],[0,0,0,1,1,0,0,0,0],[0,0,0,1,0,0,0,0,0],[0,0,0,1,1,0,0,0,0],[0,0,0,1,0,0,0,0,0],[0,0,0,1,0,0,0,0,0],[0,0,0,1,0,0,0,0,0],[0,0,0,1,0,0,0,0,0],[0,0,0,1,0,0,0,0,0],[0,0,0,1,1,0,0,0,0],[0,0,0,1,0,0,0,0,0],[0,0,0,1,0,0,0,0,0],[0,0,0,1,0,0,0,0,0]]9264	9265,9266	[[0,0,0,0,1,0,0,0,0],[0,0,0,0,1,0,0,0,0]]9267	9268	[[0,0,0,0,1,0,0,0,0]]9269	9270,9271	[[0,0,0,0,1,0,0,0,0],[0,0,0,0,1,0,0,0,0]]9272	9265	[[0,0,0,0,1,0,0,0,0]]9273	9274,6608,7007,9275,8239,4903,9276,9277,9278,9279,4894,3415,9280,9281,9282,9283,6929,9284,6617,6611,7017,9285	[[0,1,1,1,0,0,0,1,0],[0,1,1,0,0,0,0,1,0],[0,1,1,0,0,0,0,1,0],[0,0,1,0,0,0,0,0,0],[0,0,0,0,0,0,0,1,0],[0,0,1,0,0,0,0,1,0],[0,1,1,0,0,0,0,0,0],[0,1,0,1,0,0,0,0,0],[0,1,1,0,0,0,0,1,0],[0,1,1,0,0,0,0,1,0],[0,1,1,0,0,0,0,1,0],[0,1,0,0,0,0,0,0,0],[0,1,1,0,0,0,0,1,0],[0,1,1,0,0,0,0,0,0],[0,1,1,0,0,0,0,0,0],[0,0,0,0,0,0,0,1,0],[0,0,0,0,0,0,0,1,0],[0,0,1,1,0,0,0,1,0],[0,0,1,0,0,0,0,1,0],[0,0,1,0,0,0,0,0,0],[0,0,1,0,0,0,0,0,0],[0,0,1,0,0,0,0,0,0]]9286	9287,1272	[[0,0,0,0,1,0,0,0,0],[0,0,0,0,1,0,0,0,0]]9288	9289	[[0,0,0,0,1,0,0,0,0]]9290	9291,3411,3573	[[0,0,0,1,0,0,0,0,0],[0,0,0,1,0,0,0,0,0],[0,0,0,1,0,0,0,0,0]]9292	9293	[[0,0,0,0,1,0,0,0,0]]9294	9295	[[0,0,0,0,1,0,0,0,0]]9296	9297	[[0,0,0,0,1,0,0,0,0]]9298	9299	[[0,0,0,0,1,0,0,0,0]]9300	9301,6842,9302,9303,9304,9305,9306	[[0,0,0,0,1,0,0,0,0],[0,0,0,0,1,0,0,0,0],[0,0,0,0,1,0,0,0,0],[0,0,0,0,1,0,0,0,0],[0,0,0,0,1,0,0,0,0],[0,0,0,0,1,0,0,0,0],[0,0,0,0,1,0,0,0,0]]9307	9308,9309	[[0,0,0,0,1,0,0,0,0],[0,0,0,0,1,0,0,0,0]]9310	9311	[[0,0,0,0,1,0,0,0,0]]9312	9313	[[0,0,0,0,1,0,0,0,0]]9314	9315	[[0,0,0,0,1,0,0,0,0]]9316	9317,9318,9319,8196	[[0,0,0,0,1,0,0,0,0],[0,0,0,0,1,0,0,0,0],[0,0,0,0,1,0,0,0,0],[0,0,0,0,1,0,0,0,0]]9320	9321,9322,9323,9324,9325,1720	[[0,0,0,0,1,0,0,0,0],[0,0,0,1,0,0,0,0,0],[0,0,0,0,1,0,0,0,0],[0,0,0,1,0,0,0,0,0],[0,0,0,1,0,0,0,0,0],[0,0,0,0,1,0,0,0,0]]9326	9170,9327,9328,7624,9329,9330,5509,9331,9332,9333,9334,9335,550,9336,9337,9338,9339,9340,9341,9342,9343,9344,9345,4641,9346,9347,4712,8138,9348,4688,9349,9350,9351,3995,2795,644,9352,1073,5400,4664,9353,9354,9355,1400,1099,9356,4701,9357,9358,9359,9091,4635,3295,1048,9360,9361,9362,7636,1692,9363,9364,9365,9366,1067,9367,3279,1046,6706,9368,8753,8790,5510,9369,4615,9370,9371	[[0,0,1,1,0,0,0,1,0],[0,0,0,1,0,0,0,0,0],[0,0,0,0,0,0,0,1,0],[0,0,1,1,0,0,0,0,0],[0,1,1,1,0,0,1,0,0],[0,0,0,0,0,0,0,0,1],[0,0,1,0,0,0,0,0,0],[1,1,1,1,0,0,1,0,1],[0,0,0,1,0,0,0,1,0],[0,0,0,1,0,0,0,1,0],[0,0,0,0,0,0,0,1,0],[1,1,1,1,0,0,1,1,1],[0,1,1,1,0,0,1,1,0],[1,0,1,1,0,0,0,1,0],[0,0,0,1,0,0,0,1,0],[0,0,1,1,0,0,0,1,0],[1,0,1,1,0,0,0,1,0],[1,0,0,0,0,0,0,0,0],[1,0,1,0,0,0,0,0,0],[1,0,1,1,0,0,0,1,1],[1,0,0,0,0,0,0,0,0],[0,1,1,0,0,0,1,0,0],[0,1,1,1,0,0,1,1,0],[0,0,1,0,0,0,0,0,0],[0,0,1,1,0,0,0,1,0],[0,0,0,1,0,0,0,1,0],[0,0,0,1,0,0,0,1,0],[0,0,0,1,0,0,0,1,0],[0,0,0,0,1,0,0,0,0],[0,0,0,0,0,0,0,1,0],[0,0,0,0,0,0,0,1,0],[0,0,0,0,0,0,0,0,1],[0,0,0,1,0,0,0,1,0],[0,0,0,0,0,0,0,0,1],[0,0,0,0,0,0,0,1,0],[0,0,1,1,0,0,0,0,0],[0,1,1,1,0,0,1,0,0],[0,0,0,1,0,0,0,1,0],[0,0,1,1,0,0,0,0,0],[0,0,1,1,0,0,0,0,0],[0,0,0,1,0,0,0,1,0],[0,0,1,0,0,0,0,0,0],[0,1,0,1,0,0,1,0,0],[0,1,0,0,0,0,1,1,0],[0,0,0,1,0,0,0,1,0],[0,0,0,1,0,0,0,0,0],[0,1,1,0,0,0,1,1,0],[0,0,0,1,0,0,0,1,0],[0,0,1,1,0,0,0,1,0],[0,0,0,1,0,0,0,0,0],[0,0,0,1,0,0,0,1,0],[0,0,1,0,0,0,0,1,0],[0,0,1,1,0,0,0,0,1],[0,0,0,1,0,0,0,0,0],[0,0,0,1,0,0,0,0,0],[0,0,1,0,0,0,0,0,0],[0,0,1,0,0,0,0,0,0],[0,0,0,1,0,0,0,1,0],[0,0,1,1,0,0,0,0,1],[0,0,1,0,0,0,0,0,0],[0,0,1,1,0,0,0,1,0],[0,0,1,0,0,0,0,0,0],[0,0,1,1,0,0,0,0,0],[0,0,0,1,0,0,0,1,0],[0,1,1,1,0,0,1,0,0],[0,1,1,0,0,0,1,0,0],[0,1,0,0,0,0,1,0,0],[0,0,0,0,0,0,0,1,0],[1,0,0,0,0,0,0,0,0],[0,0,0,1,0,0,0,1,0],[0,0,1,1,0,0,0,1,0],[0,0,1,0,0,0,0,1,0],[0,0,0,0,0,0,0,0,1],[0,0,1,0,0,0,0,0,0],[0,0,1,0,0,0,0,0,0],[0,0,1,0,0,0,0,0,0]]9372	9373,9374,9375,9376,9377	[[0,0,0,0,1,0,0,0,0],[0,0,0,0,1,0,0,0,0],[0,0,0,0,1,0,0,0,0],[0,0,0,0,1,0,0,0,0],[0,0,0,0,1,0,0,0,0]]9378	9379,9380,7172,9381,9382,9068,9383	[[0,0,1,0,0,0,0,0,0],[0,0,0,0,1,0,0,0,0],[0,0,1,0,0,0,0,0,0],[0,0,1,0,0,0,0,0,0],[0,0,1,0,0,0,0,0,0],[0,0,1,0,0,0,0,0,0],[0,0,1,0,0,0,0,0,0]]9384	712,8069,4985,9385,9386,9387,9388,4251,8039,1631,479,9389,9390,7747,3941,8166,9391,7985,5004,4987,8163,8037,704,9392,8004,9393,9394,9395,9396,9397,4209,9398,9399,9400,9401,8165,9402,4552,7344,9403,9404,8061,8042,9405,9406,2874,6613,706,5155,9407,9408,771,2866,9409,4553,9410,9411,9412,9413,5196,9414,7252,6883,7271,9415,1915,7270,3074,3083,5203,2018,9416,7213,2250,8148,8021,8015,9417,9418,9419,4678,9420,9421,9422,5932,9423,9424	[[0,1,1,0,1,0,0,1,0],[0,1,1,0,0,0,0,1,0],[1,1,1,0,0,0,0,0,0],[1,1,1,0,0,0,0,1,0],[1,1,0,0,0,0,0,1,0],[1,1,1,0,0,0,0,1,0],[1,1,1,0,0,0,0,1,0],[0,1,0,0,0,0,0,0,0],[1,1,0,0,1,0,0,1,0],[0,0,0,1,0,0,0,0,0],[0,1,0,0,0,0,0,0,0],[0,1,0,0,0,0,0,0,0],[0,1,0,0,0,0,0,0,0],[0,1,0,0,0,0,0,0,0],[0,1,0,0,0,0,0,0,0],[1,0,1,0,0,0,0,1,0],[0,1,0,0,0,0,0,0,0],[0,1,0,0,0,0,0,1,0],[1,1,0,0,0,0,0,0,0],[1,1,1,0,0,0,0,0,0],[1,1,1,0,0,0,0,1,0],[0,1,1,0,0,0,0,1,0],[0,1,1,1,0,0,0,1,0],[0,1,1,0,0,0,0,0,0],[0,1,1,0,0,0,0,1,0],[0,0,1,0,0,0,0,1,0],[1,0,1,0,0,0,0,1,0],[0,0,1,0,0,0,0,0,0],[0,0,1,0,0,0,0,0,0],[0,0,0,1,0,0,0,1,0],[0,0,1,0,0,0,0,0,0],[1,1,1,0,0,0,0,0,0],[0,0,1,0,0,0,0,0,0],[0,0,1,0,0,0,0,1,0],[0,0,1,0,0,0,0,1,0],[1,1,1,0,0,0,0,1,0],[0,0,1,0,0,0,0,0,0],[0,1,1,0,0,0,0,1,0],[0,0,0,0,0,0,0,1,0],[0,1,1,1,0,0,0,1,0],[0,0,0,0,1,0,0,0,0],[0,1,1,0,0,0,0,1,0],[0,1,0,0,0,0,0,0,0],[0,0,1,0,0,0,0,1,0],[0,0,1,0,0,0,0,0,0],[0,0,0,0,0,0,0,1,0],[0,0,1,0,0,0,0,0,0],[0,1,1,0,0,0,0,0,0],[0,0,1,0,0,0,0,0,0],[0,0,1,0,0,0,0,0,0],[0,0,0,0,0,0,0,1,0],[1,0,1,0,0,0,0,0,0],[0,1,1,0,0,0,0,1,0],[0,0,1,0,0,0,0,1,0],[0,0,1,0,0,0,0,0,0],[0,1,1,1,0,0,0,1,0],[0,0,1,0,1,0,0,0,0],[0,0,0,1,0,0,0,0,0],[0,0,0,0,0,0,0,1,0],[1,0,0,0,0,0,0,0,0],[1,0,0,0,0,0,0,0,0],[1,0,0,0,0,0,0,0,0],[1,0,0,0,0,0,0,0,0],[1,0,0,0,0,0,0,0,0],[1,0,0,0,0,0,0,0,0],[1,0,0,0,0,0,0,0,0],[1,0,0,0,0,0,0,0,0],[1,0,0,0,0,0,0,0,0],[1,0,0,0,0,0,0,0,0],[1,0,0,0,0,0,0,0,0],[1,0,0,0,0,0,0,0,0],[1,0,0,0,0,0,0,0,0],[1,0,0,0,0,0,0,0,0],[1,0,0,0,0,0,0,0,0],[1,0,0,0,0,0,0,0,0],[0,1,1,1,0,0,0,1,0],[1,0,1,0,0,0,0,0,0],[0,1,0,0,0,0,0,0,0],[1,0,0,0,0,0,0,0,0],[1,0,0,0,0,0,0,0,0],[0,0,0,1,0,0,0,0,0],[1,0,0,0,0,0,0,0,0],[1,0,0,0,0,0,0,0,0],[1,0,0,0,0,0,0,0,0],[1,0,0,0,0,0,0,0,0],[1,0,0,0,0,0,0,0,0],[1,0,0,0,0,0,0,0,0]]9425	9426,5850,9427,5164,7942,9428,9429,9430,7336,309,9431,314,5162,9432,9433,9434,7345,9435,9436,7338	[[0,0,1,1,0,0,0,0,0],[0,1,0,0,0,0,0,0,0],[0,1,1,1,0,0,1,1,1],[0,0,1,0,0,0,0,0,1],[0,0,0,0,0,0,0,1,0],[0,0,1,0,0,0,0,0,0],[0,0,1,0,0,0,0,0,1],[0,0,1,0,0,0,0,0,0],[0,0,1,0,0,0,0,0,0],[0,0,1,0,0,0,0,0,0],[0,1,1,0,0,0,1,0,0],[0,0,1,0,0,0,0,0,0],[0,1,1,0,0,0,1,0,0],[0,0,0,0,0,0,0,1,0],[0,0,1,0,0,0,0,0,0],[0,0,1,0,0,0,0,1,1],[0,0,1,1,0,0,0,1,1],[0,0,1,0,0,0,0,0,0],[0,0,1,0,0,0,0,0,0],[0,0,1,0,0,0,0,0,1]]9437	9438,9439,9440,9441,9442,9443,9444,9445,9446,9447,9448	[[0,1,0,0,0,0,1,0,0],[0,1,0,0,0,0,1,0,0],[0,1,0,0,0,0,1,0,0],[0,1,0,0,0,0,1,0,0],[0,1,0,0,0,0,1,0,0],[0,1,0,0,0,0,1,0,0],[0,1,0,0,0,0,1,0,0],[0,1,0,0,0,0,1,0,0],[0,1,0,0,0,0,1,0,0],[0,1,0,0,0,0,0,0,0],[0,0,0,0,1,0,0,0,0]]9449	9450,9451,9452,2209,5854,5846,2218,2210,9453,9454,5820,9455,2208,2214,5806,9456,9457,2204,9458,3894,9459,5811,5851,9460,5799,5844,5837,5812,5841,5830,9461,9462,5829,2202,5856,9463,2220,5848,9464,9465,5802,9466,5821,9467,9468,9469,9470,5857	[[0,0,1,0,0,0,0,0,0],[0,0,1,0,0,0,0,0,0],[0,0,1,0,0,0,0,0,0],[0,0,1,0,0,0,0,0,0],[0,0,1,0,1,0,0,0,0],[0,0,1,0,0,0,0,0,0],[0,0,1,0,0,0,0,0,0],[0,0,1,0,0,0,0,0,0],[0,0,1,0,0,0,0,0,0],[0,0,1,0,0,0,0,0,0],[0,0,1,0,1,0,0,0,0],[0,0,1,0,0,0,0,0,0],[0,0,1,0,0,0,0,0,0],[0,0,1,0,0,0,0,0,0],[0,0,1,0,1,0,0,0,0],[0,0,1,0,0,0,0,0,0],[0,0,1,0,0,0,0,0,0],[0,0,1,0,0,0,0,0,0],[0,0,1,0,0,0,0,0,0],[0,0,1,1,0,0,0,0,0],[0,0,1,0,0,0,0,0,0],[0,0,1,0,0,0,0,0,0],[0,0,1,0,0,0,0,0,0],[0,0,1,0,0,0,0,0,0],[0,0,1,0,0,0,0,0,0],[0,0,1,0,0,0,0,0,0],[0,0,1,0,0,0,0,0,0],[0,0,1,0,0,0,0,0,0],[0,0,1,0,1,0,0,0,0],[0,0,1,0,0,0,0,0,0],[0,0,0,0,1,0,0,0,0],[0,0,1,0,0,0,0,0,0],[0,0,1,0,0,0,0,0,0],[0,0,1,0,0,0,0,0,0],[0,0,0,1,0,0,0,0,0],[0,0,1,0,0,0,0,0,0],[0,0,1,0,1,0,0,0,0],[0,0,1,0,0,0,0,0,0],[0,0,0,1,0,0,0,0,0],[0,0,1,0,0,0,0,0,0],[0,0,1,1,0,0,0,0,0],[0,0,0,1,0,0,0,0,0],[0,0,1,0,0,0,0,0,0],[0,0,1,0,0,0,0,0,0],[0,0,0,1,0,0,0,0,0],[0,0,1,0,0,0,0,0,0],[0,0,1,0,0,0,0,0,0],[0,0,1,0,0,0,0,0,0]]9471	9472,9473,9474	[[0,0,0,1,0,0,0,0,0],[0,0,0,1,0,0,0,0,0],[0,0,0,1,0,0,0,0,0]]9475	9476,9477,9478	[[0,0,0,0,1,0,0,0,0],[0,0,0,0,1,0,0,0,0],[0,0,0,0,1,0,0,0,0]]9479	7247,7219,7201,7252,7271,7263,6847,7236,7237,7238,7239,7240,5206,7249,9480,7274,9481,9482,7212,7211,7244,6781,9483,7207,7241,4474,6850,7218,9484,9485,7246,7235,7222,7227,9486,9487,7608,7775,7602,8160,7280,7273,6926,7248,7243,9488,7253,7268,7266,1252,7166,7223,9489,5196,9490,9491,9492,9493,8162,7255,7254,7257,7604	[[0,0,1,0,0,0,0,0,0],[0,1,1,0,0,0,0,0,0],[0,1,1,0,1,1,0,0,0],[0,1,1,0,0,1,0,0,1],[0,1,1,1,1,1,0,0,1],[0,0,1,0,0,0,0,0,0],[0,0,1,0,0,0,0,0,0],[0,0,1,0,1,0,0,0,0],[0,0,1,0,0,0,0,0,0],[0,0,1,0,1,0,0,0,0],[0,0,1,1,0,1,0,0,1],[0,0,1,0,0,0,0,0,0],[0,0,1,0,0,0,0,0,0],[0,0,1,0,0,0,0,0,0],[0,0,1,0,0,0,0,0,0],[0,0,1,0,0,0,0,0,0],[0,0,1,0,0,0,0,0,1],[0,0,0,0,0,0,0,0,1],[0,0,1,0,0,0,0,0,0],[0,1,1,0,1,0,0,0,0],[0,0,1,0,0,0,0,0,0],[0,0,1,0,0,0,0,0,0],[0,0,1,0,0,0,0,0,0],[0,0,1,0,0,0,0,0,0],[0,0,1,0,0,0,0,0,0],[0,0,0,0,0,0,0,0,1],[0,0,1,0,0,0,0,0,0],[0,0,1,0,0,0,0,0,1],[0,0,1,0,0,0,0,0,0],[0,0,1,0,0,0,0,0,0],[0,0,1,0,0,0,0,0,0],[0,0,1,0,0,0,0,0,0],[0,0,1,0,0,0,0,0,0],[0,0,1,1,1,1,0,0,1],[0,0,1,0,0,0,0,0,0],[0,0,1,0,0,0,0,0,0],[0,0,0,0,0,0,0,0,1],[0,0,1,0,0,0,0,0,0],[0,0,1,0,0,0,0,0,0],[0,0,1,0,0,0,0,0,0],[0,0,1,0,0,0,0,0,0],[0,0,1,0,0,0,0,0,0],[0,0,1,0,0,0,0,0,0],[0,0,1,0,0,1,0,0,1],[0,0,1,0,1,1,0,0,1],[0,1,0,0,0,0,0,0,0],[0,1,0,0,0,0,0,0,0],[0,0,1,0,0,0,0,0,0],[0,0,1,0,0,0,0,0,0],[0,0,1,0,0,0,0,0,0],[0,0,1,0,0,0,0,0,0],[0,0,1,0,0,0,0,0,0],[0,0,1,0,0,0,0,0,0],[0,0,1,0,0,0,0,0,0],[0,0,1,0,0,0,0,0,0],[0,0,1,0,0,0,0,0,0],[0,0,1,0,0,0,0,0,0],[0,0,1,0,0,0,0,0,0],[0,0,0,0,0,1,0,0,0],[0,0,1,0,0,0,0,0,0],[0,0,1,0,0,0,0,0,0],[0,0,1,0,0,0,0,0,0],[0,0,0,0,0,0,0,0,1]]9494	8904,8138,9495,1997,9496,2006,7373,9497,9498,5390,1099,9499,9500,9501,9502,9503,9504,9505,9506,9507,9508,3842,9509,9510	[[0,0,0,1,0,0,0,0,0],[0,0,0,1,0,0,0,0,0],[0,0,0,1,0,0,0,0,0],[0,0,0,1,0,0,0,0,0],[0,0,0,1,0,0,0,0,0],[0,0,0,1,0,0,0,0,0],[0,0,0,1,0,0,0,0,0],[0,0,0,1,0,0,0,0,0],[0,0,0,1,0,0,0,0,0],[0,0,0,1,0,0,0,0,0],[0,0,0,1,0,0,0,0,0],[0,0,0,1,0,0,0,0,0],[0,0,0,1,0,0,0,0,0],[0,0,0,1,0,0,0,0,0],[0,0,0,1,0,0,0,0,0],[0,0,0,1,0,0,0,0,0],[0,0,0,1,0,0,0,0,0],[0,0,0,1,0,0,0,0,0],[0,0,0,1,0,0,0,0,0],[0,0,0,1,0,0,0,0,0],[0,0,0,1,0,0,0,0,0],[0,0,0,1,0,0,0,0,0],[0,0,0,1,0,0,0,0,0],[0,0,0,1,0,0,0,0,0]]9511	9512,9513	[[0,0,0,0,1,0,0,0,0],[0,0,0,0,1,0,0,0,0]]9514	9515,9516	[[0,0,0,0,1,0,0,0,0],[0,0,0,0,1,0,0,0,0]]9517	9518,9519,9520,9521,9522,9523,9524,9525,9526,9527,9528,9529,9530,9531,9532,9533,9534,9535,9536,9537,9538,9539,9540,9541,9542,9543,9544,9545,9546,3205,9547,9548,9549,9550,7309,9551,9552,9553,9187,9554,5002,9555,9556	[[0,0,1,0,0,0,0,0,0],[0,0,1,0,0,0,0,0,0],[1,0,0,1,0,0,0,0,0],[0,0,0,0,1,0,0,0,0],[0,0,1,0,0,0,0,0,0],[0,0,1,0,0,0,0,0,0],[0,1,1,0,0,0,1,0,0],[0,1,1,1,1,0,1,0,0],[0,1,1,0,0,0,1,0,0],[0,0,1,0,0,0,0,0,0],[0,0,1,0,0,0,0,0,0],[1,1,1,1,1,0,1,0,0],[0,0,1,0,0,0,0,0,0],[1,0,0,1,0,0,0,0,0],[0,0,1,0,0,0,0,0,0],[0,1,0,0,1,0,1,0,0],[0,0,1,0,0,0,0,0,0],[1,0,1,0,0,0,1,0,0],[1,1,1,1,1,0,1,0,0],[0,0,1,0,0,0,0,0,0],[1,0,0,1,0,0,0,0,0],[0,1,1,0,0,0,0,0,0],[0,0,1,0,0,0,0,0,0],[0,0,1,0,0,0,0,0,0],[0,0,1,0,0,0,0,0,0],[0,0,1,0,1,0,0,0,0],[0,0,0,0,0,0,1,0,0],[0,0,1,0,0,0,0,0,0],[0,0,1,0,0,0,0,0,0],[1,0,0,0,0,0,0,0,0],[1,0,0,1,0,0,0,0,0],[1,1,1,1,0,0,1,0,0],[1,1,0,0,0,0,0,0,0],[1,0,0,0,0,0,0,0,0],[1,0,0,0,0,0,0,0,0],[0,0,1,0,0,0,0,0,0],[0,0,1,0,0,0,0,0,0],[0,0,1,0,0,0,0,0,0],[0,0,1,0,0,0,0,0,0],[0,0,1,0,0,0,0,0,0],[0,0,1,0,0,0,0,0,0],[1,0,0,0,0,0,0,0,0],[0,0,1,0,0,0,0,0,0]]9557	8927,9558,5924,1070,1044,9510,7379,8915,8917,9559,2006,9499,9560,3715,1099,9561,9562,9563,9564,9565,1978,9566,4629,7373,9567,5390,4793,4675,9568,2032,9503,9504,2064,9498,9569,1091,1048,9505,9570,9571,4033,422,8298,8296,8324,2652,9572,9573,9338,8904,1058,9502,1997,1067,1977,9574,8299,9575,9506,9576,8787,9497,9577,9507,9495,8138,8290,1072,9496,9500,9501,9578,8294,6018,1996,2055,8790,9508,9509,3842,9579	[[1,0,0,0,0,0,0,0,0],[0,0,1,0,0,0,1,1,0],[0,0,1,0,0,0,1,1,0],[0,0,1,0,0,0,0,0,0],[1,0,1,0,0,0,1,1,1],[0,0,1,1,0,0,0,0,0],[0,0,0,0,0,0,1,0,0],[1,0,0,0,0,0,0,0,0],[1,0,0,0,0,0,0,0,0],[1,0,0,0,0,0,0,0,0],[1,0,1,1,1,0,0,1,0],[1,0,1,1,0,0,0,0,0],[1,0,1,0,0,0,1,1,0],[1,0,1,0,0,0,0,1,0],[1,0,1,1,0,0,0,1,1],[0,0,1,0,0,0,0,0,0],[0,0,1,0,0,0,0,0,0],[0,0,1,0,0,0,1,0,0],[0,0,0,0,1,0,0,0,0],[0,0,1,0,0,0,0,0,0],[0,0,1,0,0,0,0,0,0],[0,0,0,0,0,0,1,0,0],[0,0,0,0,0,0,0,1,0],[0,0,0,1,0,0,0,0,0],[0,0,0,0,0,0,0,0,1],[0,0,0,1,0,0,0,0,0],[1,0,0,0,0,0,0,1,0],[1,0,0,0,0,0,0,1,0],[1,0,1,0,0,0,0,0,0],[0,0,1,0,0,0,0,1,1],[0,0,1,1,0,0,0,1,0],[0,0,0,1,0,0,0,0,0],[0,0,1,0,0,0,0,1,1],[0,0,0,1,0,0,0,0,0],[0,0,1,0,0,0,0,0,0],[1,0,1,0,0,0,1,0,1],[1,0,0,0,0,0,0,0,1],[0,0,0,1,0,0,0,0,0],[1,0,1,0,0,0,0,0,0],[1,0,0,0,0,0,1,0,0],[1,0,1,0,0,0,0,1,0],[1,0,0,0,0,0,0,1,0],[0,0,1,0,0,0,0,0,0],[0,0,1,0,0,0,0,0,0],[1,0,0,0,0,0,0,0,0],[1,0,0,0,0,0,0,0,0],[1,0,0,0,0,0,0,0,0],[1,0,0,0,0,0,0,0,0],[1,0,0,0,0,0,0,0,0],[0,0,1,1,0,0,0,1,1],[0,0,1,0,0,0,0,1,0],[0,0,1,1,0,0,0,1,1],[0,0,1,1,0,0,0,0,0],[0,0,1,0,0,0,0,1,0],[0,0,1,0,0,0,0,0,0],[0,0,0,0,1,0,0,0,0],[0,0,1,0,0,0,0,0,0],[0,0,0,0,0,0,1,0,0],[0,0,0,1,0,0,0,0,0],[0,0,1,0,0,0,0,0,0],[0,0,0,0,0,0,0,0,1],[0,0,0,1,0,0,0,0,0],[0,0,1,0,0,0,0,0,0],[0,0,0,1,0,0,0,0,0],[0,0,0,1,0,0,0,0,0],[0,0,0,1,0,0,0,0,0],[0,0,1,0,0,0,0,0,0],[0,0,1,0,0,0,0,0,0],[0,0,0,1,0,0,0,0,0],[0,0,0,1,0,0,0,0,0],[0,0,0,1,0,0,0,0,0],[0,0,1,0,0,0,0,0,0],[0,0,1,0,0,0,0,0,0],[0,0,1,0,0,0,0,0,0],[0,0,0,0,1,0,0,0,0],[0,0,1,0,0,0,0,0,0],[0,0,0,0,0,0,0,0,1],[0,0,0,1,0,0,0,0,0],[0,0,0,1,0,0,0,0,0],[0,0,0,1,0,0,0,0,0],[0,0,1,0,0,0,0,0,0]]9580	9581	[[0,0,0,0,1,0,0,0,0]]9582	9583	[[0,0,0,0,1,0,0,0,0]]9584	9585	[[0,0,0,0,1,0,0,0,0]]9586	9587	[[0,0,0,0,1,0,0,0,0]]9588	9589	[[0,0,0,0,1,0,0,0,0]]9590	9591	[[0,0,0,0,1,0,0,0,0]]9592	9593,9594,9595,9596,9597,9598,9599,9600,9601,9602,9603,9604,9174,9605,9606,9607,9608,4178,9609,9610,4679,9611,9612,9613,3281,9614,2104,9615,7640	[[0,1,0,0,0,0,1,0,0],[0,0,0,0,0,0,0,1,0],[0,1,0,0,0,0,0,0,0],[0,1,0,0,0,0,0,1,0],[0,1,0,0,0,0,0,1,0],[0,1,0,0,0,0,1,0,0],[0,1,0,0,0,0,1,0,0],[0,1,0,0,0,0,1,1,0],[0,1,0,0,0,0,1,0,0],[0,0,0,0,0,0,0,1,0],[0,1,0,0,0,0,0,0,0],[0,0,0,0,0,0,0,1,0],[0,1,0,0,0,0,1,0,0],[0,0,0,0,0,0,0,1,0],[0,1,0,0,0,0,1,0,0],[0,0,0,0,0,0,0,1,0],[0,0,0,0,0,0,0,1,0],[0,1,0,0,0,0,1,1,0],[0,0,0,0,0,0,0,1,0],[0,1,0,0,0,0,0,0,0],[0,1,0,0,0,0,1,1,0],[0,1,0,0,0,0,1,1,0],[0,1,0,0,0,0,0,0,0],[0,0,0,0,0,0,0,1,0],[0,1,0,0,0,0,1,1,0],[0,0,0,0,0,0,0,1,0],[0,0,0,0,0,0,0,1,0],[0,1,0,0,0,0,1,0,0],[0,0,0,0,0,0,0,1,0]]9616	9617,955	[[0,0,0,0,1,0,0,0,0],[0,0,0,0,1,0,0,0,0]]9618	9619	[[0,0,0,0,1,0,0,0,0]]9620	9621	[[0,0,0,0,1,0,0,0,0]]9622	9623	[[0,0,0,0,1,0,0,0,0]]9624	9625,9626,9627	[[0,0,0,0,1,0,0,0,0],[0,0,0,0,1,0,0,0,0],[0,0,0,0,1,0,0,0,0]]9628	9629	[[0,0,0,0,1,0,0,0,0]]9630	4013,6615	[[0,0,0,1,0,0,0,0,0],[0,0,0,1,0,0,0,0,0]]9631	9632,9633,3561	[[0,0,0,0,1,0,0,0,0],[0,0,0,0,1,0,0,0,0],[0,0,0,0,1,0,0,0,0]]9634	9635,9636,3801	[[0,0,0,1,0,0,0,0,0],[0,0,0,1,0,0,0,0,0],[0,0,0,1,0,0,0,0,0]]9637	9638,9639,9640,9641,9642,2118,9643,9644,9645	[[1,0,0,0,0,0,0,0,0],[1,0,0,0,0,0,0,0,0],[1,0,0,0,0,0,0,0,0],[0,0,0,0,1,0,0,0,0],[1,0,0,0,0,0,0,0,0],[1,0,0,0,0,0,0,0,0],[1,0,0,0,0,0,0,0,0],[1,0,0,0,0,0,0,0,0],[1,0,0,0,0,0,0,0,0]]9646	7928,7939,9647,7903,7933,7773,1786,7901,7914,8529,7932,9648,9649,7113,7922,7900,7917,7915,9650,7929,4555,9651,7937,9652,4326,9653,3417,7941,7908,7898,2423,9654	[[0,0,1,0,0,0,0,1,0],[0,1,1,0,0,0,0,0,0],[0,0,1,0,0,0,0,0,0],[0,0,1,0,0,0,0,0,0],[0,0,0,0,0,0,0,1,0],[0,1,1,0,1,0,1,1,0],[0,1,1,1,0,0,1,1,0],[0,1,1,0,0,0,1,0,0],[0,1,1,0,0,0,0,1,0],[0,0,1,1,0,0,0,0,0],[0,1,1,0,0,0,1,1,0],[0,0,1,0,0,0,0,0,0],[0,1,0,0,1,0,0,0,0],[0,0,1,0,0,0,0,0,0],[0,1,1,1,0,0,1,1,0],[0,0,1,0,0,0,0,0,0],[0,1,1,0,0,0,0,0,0],[0,0,1,0,0,0,0,1,0],[0,0,0,0,1,0,0,0,0],[0,0,1,0,0,0,0,0,0],[0,1,1,1,1,0,1,1,0],[0,0,1,0,0,0,0,0,0],[0,1,1,0,1,0,0,0,0],[0,0,0,0,1,0,0,1,0],[0,0,1,0,0,0,0,0,0],[0,0,0,0,1,0,0,0,0],[0,0,1,0,0,0,0,0,0],[0,0,1,0,0,0,0,0,0],[0,0,1,0,0,0,0,0,0],[0,0,1,0,0,0,0,0,0],[0,0,1,0,0,0,0,0,0],[0,0,1,0,0,0,0,0,0]]9655	9656,9657,9658,5464,5476,9659,9660,7192,9661,9662,9663,6753,7155	[[0,0,0,0,1,0,0,0,0],[0,0,0,0,1,0,0,0,0],[0,0,0,0,1,0,0,0,0],[0,0,0,0,1,0,0,0,0],[0,0,0,0,1,0,0,0,0],[0,0,0,0,1,0,0,0,0],[0,0,0,0,1,0,0,0,0],[0,0,0,0,1,0,0,0,0],[0,0,0,0,1,0,0,0,0],[0,0,0,0,1,0,0,0,0],[0,0,0,0,1,0,0,0,0],[0,0,0,0,1,0,0,0,0],[0,0,0,0,1,0,0,0,0]]9664	9665,9666,9667,7535,9668,9669,9670,9671,9672,9673,9674,9675,9676,1723,9677,9678,9679,9680,9681,9682,5619,9683,9684,9685,9686,9687	[[0,0,1,1,0,0,0,0,0],[0,0,1,0,0,0,0,0,0],[0,0,1,0,0,0,0,0,0],[0,0,1,0,0,0,0,0,0],[0,0,1,0,0,0,0,0,0],[0,0,1,0,0,0,0,0,0],[0,0,1,0,0,0,0,0,0],[0,0,1,0,0,0,0,0,0],[0,0,1,0,0,0,0,0,0],[0,0,1,0,0,0,0,0,0],[0,0,1,0,0,0,0,0,0],[0,0,1,0,0,0,0,0,0],[0,0,1,0,0,0,0,0,0],[0,0,1,0,0,0,0,0,0],[0,0,1,0,0,0,0,0,0],[0,0,1,0,0,0,0,0,0],[0,0,1,0,0,0,0,0,0],[0,0,1,0,0,0,0,0,0],[0,0,1,0,0,0,0,0,0],[0,0,1,0,0,0,0,0,0],[0,0,1,0,0,0,0,0,0],[0,0,1,0,0,0,0,0,0],[0,0,1,0,0,0,0,0,0],[0,0,1,0,0,0,0,0,0],[0,0,1,1,0,0,0,0,0],[0,0,1,0,0,0,0,0,0]]9688	9689,9690,9691,9692,4013,6615,9693,9694,9695,9696	[[0,0,0,0,1,0,0,0,0],[0,0,0,0,1,0,0,0,0],[0,0,0,0,1,0,0,0,0],[0,0,0,0,1,0,0,0,0],[0,0,0,1,0,0,0,0,0],[0,0,0,1,0,0,0,0,0],[0,0,0,0,1,0,0,0,0],[0,0,0,0,1,0,0,0,0],[0,0,0,0,1,0,0,0,0],[0,0,0,0,1,0,0,0,0]]9697	559,9698,2366,3196,7924	[[0,0,1,0,0,0,0,0,0],[0,0,1,0,0,0,0,0,0],[0,0,1,0,0,0,0,0,0],[0,0,1,0,0,0,0,0,0],[0,0,1,0,0,0,0,0,0]]9699	9700,6786,2239,6761,6760	[[0,0,0,0,1,0,0,0,0],[0,0,0,1,0,0,0,0,0],[0,0,0,0,1,0,0,0,0],[0,0,0,1,0,0,0,0,0],[0,0,0,1,0,0,0,0,0]]9701	9702,9703,9704,9705,9706,9707,9708,9709,9710	[[0,1,0,0,0,0,0,0,0],[0,1,0,0,0,0,1,0,0],[0,1,0,0,0,0,0,0,0],[0,1,0,0,0,0,1,0,0],[0,1,0,0,0,0,1,0,0],[0,1,0,0,0,0,0,0,0],[0,1,0,0,0,0,1,0,0],[0,1,0,0,0,0,1,0,0],[0,1,0,0,0,0,0,0,0]]9711	9712,9713,9714,9715,5500,4878,9716,9717,9718,896	[[0,0,0,1,0,0,0,0,0],[0,0,0,1,0,0,0,0,0],[0,0,0,1,0,0,0,0,0],[0,0,0,1,0,0,0,0,0],[0,0,0,1,0,0,0,0,0],[0,0,0,1,0,0,0,0,0],[0,0,0,1,0,0,0,0,0],[0,0,0,1,0,0,0,0,0],[0,0,0,1,0,0,0,0,0],[0,0,0,1,0,0,0,0,0]]9719	9720	[[0,0,0,1,0,0,0,0,0]]9721	9076,4232,8717,9244,9071,9722,765,9723,9724,6861,4209,9725,5017,2480,2264,4219,9726,7061,9241,9085,9727,9728,9054,9729,9242,9730,7105,9247,2896,9731,9732,9246,3416,2137,2878,333,614,9733,9053,9734,9735,9736,9737,78,8965,3185,9738,9248,6294,9065,8184,9061,3729,9049,9067,9739,9249,4237,9064,9740,9741,9742,9062,4239,9743,8960,9046,9250,8665,8963,1601,9744,9745,9746,9075,1304,9747,7092,5722,479,9748,5609,9749,9750,9068,2581,26,9751,7927,9752,8157,9753,3145,9641,7171,7129,2530,6087,3932,8973,6441,9754,4610,9755,9756,9757,4440,9758,7004	[[0,0,1,0,0,0,0,0,0],[0,0,1,0,0,0,0,0,0],[0,1,1,0,0,0,1,0,0],[0,0,1,0,0,0,0,0,0],[0,1,1,0,0,0,0,0,0],[0,0,1,0,0,0,0,0,0],[0,0,0,0,1,0,0,0,0],[0,0,1,0,0,0,0,0,0],[0,0,1,0,0,0,0,0,0],[0,0,1,0,0,0,0,0,0],[0,0,1,0,0,0,0,0,0],[0,0,1,0,0,0,0,0,0],[0,0,1,0,0,0,0,0,0],[0,0,1,0,0,0,0,0,0],[0,0,1,0,0,0,0,0,0],[0,0,1,0,0,0,0,0,0],[0,0,0,0,1,0,0,0,0],[0,0,0,1,0,0,0,0,0],[0,1,1,0,1,0,1,0,0],[0,0,1,0,0,0,0,0,0],[0,0,0,0,1,0,0,0,0],[0,1,1,0,0,0,1,0,0],[0,0,1,0,0,0,0,0,0],[0,1,1,1,1,0,1,0,0],[0,0,1,1,0,0,0,0,0],[0,1,1,0,0,0,0,0,0],[0,1,0,0,1,0,1,0,0],[0,0,1,0,0,0,0,0,0],[0,0,1,0,0,0,0,0,0],[0,0,1,0,0,0,0,0,0],[0,0,1,0,0,0,0,0,0],[0,0,1,0,0,0,0,0,0],[0,0,1,0,0,0,0,0,0],[0,0,1,0,0,0,0,0,0],[0,0,1,0,1,0,0,0,0],[0,0,1,0,0,0,0,0,0],[0,1,1,0,0,0,1,0,0],[0,0,1,0,0,0,0,0,0],[0,1,1,0,0,0,0,0,0],[0,0,1,0,0,0,0,0,0],[0,1,0,0,0,0,1,0,0],[0,0,1,0,0,0,0,0,0],[0,0,1,0,0,0,0,0,0],[0,0,1,0,0,0,0,0,0],[0,1,1,0,0,0,0,0,0],[0,0,1,0,0,0,0,0,0],[0,0,1,1,0,0,0,0,0],[0,0,0,0,1,0,0,0,0],[0,0,1,0,0,0,0,0,0],[0,0,0,0,1,0,0,0,0],[0,0,0,0,1,0,0,0,0],[0,0,1,0,0,0,0,0,0],[0,1,1,0,0,0,1,0,0],[0,1,1,0,0,0,0,0,0],[0,1,1,0,0,0,1,0,0],[0,0,1,0,0,0,0,0,0],[0,0,1,0,0,0,0,0,0],[0,0,1,0,0,0,0,0,0],[0,1,0,0,1,0,0,0,0],[0,0,1,0,0,0,0,0,0],[0,0,1,0,0,0,0,0,0],[0,0,0,0,1,0,0,0,0],[0,0,1,0,0,0,0,0,0],[0,0,1,0,0,0,0,0,0],[0,1,0,0,0,0,1,0,0],[0,1,1,0,0,0,1,0,0],[0,1,1,0,0,0,1,0,0],[0,0,1,0,0,0,0,0,0],[0,1,1,0,0,0,0,0,0],[0,1,1,0,0,0,0,0,0],[0,1,0,0,0,0,0,0,0],[0,1,0,0,0,0,0,0,0],[0,1,0,0,0,0,0,0,0],[0,1,0,0,0,0,1,0,0],[0,1,1,0,0,0,1,0,0],[0,1,0,0,0,0,0,0,0],[0,1,0,0,0,0,0,0,0],[0,0,1,0,0,0,0,0,0],[0,1,0,0,0,0,0,0,0],[0,1,0,0,0,0,0,0,0],[0,1,0,0,0,0,0,0,0],[0,1,0,0,0,0,0,0,0],[0,1,0,0,0,0,0,0,0],[0,1,0,0,0,0,0,0,0],[0,1,1,0,0,0,1,0,0],[0,1,1,0,0,0,0,0,0],[0,1,0,0,0,0,0,0,0],[0,0,1,0,0,0,0,0,0],[0,0,1,0,0,0,0,0,0],[0,0,0,0,1,0,0,0,0],[0,0,0,1,0,0,0,0,0],[0,0,1,0,0,0,0,0,0],[0,0,1,0,0,0,0,0,0],[0,1,0,0,0,0,0,0,0],[0,0,1,0,0,0,0,0,0],[0,0,0,0,1,0,0,0,0],[0,0,1,0,0,0,0,0,0],[0,0,1,0,0,0,0,0,0],[0,0,1,0,0,0,0,0,0],[0,0,1,0,0,0,0,0,0],[0,0,1,0,0,0,0,0,0],[0,0,1,0,0,0,0,0,0],[0,0,1,0,0,0,0,0,0],[0,0,1,0,0,0,0,0,0],[0,0,1,0,0,0,0,0,0],[0,0,1,0,0,0,0,0,0],[0,0,1,0,0,0,0,0,0],[0,0,1,0,0,0,0,0,0],[0,0,1,0,0,0,0,0,0]]9759	7345,9760,7330,7336,7322,7340	[[0,0,0,0,1,0,0,0,0],[0,0,0,0,1,0,0,0,0],[0,0,0,0,1,0,0,0,0],[0,0,0,0,1,0,0,0,0],[0,0,0,0,1,0,0,0,0],[0,0,0,0,1,0,0,0,0]]9761	9762	[[0,0,0,0,1,0,0,0,0]]9763	9764	[[0,0,0,0,1,0,0,0,0]]9765	9766,9767,9768	[[0,0,0,0,1,0,0,0,0],[0,0,0,0,1,0,0,0,0],[0,0,0,0,1,0,0,0,0]]9769	9770,9771,9772,9773,9774,9775,9776	[[0,0,0,0,1,0,0,0,0],[0,0,0,0,1,0,0,0,0],[0,0,0,0,1,0,0,0,0],[0,0,0,0,1,0,0,0,0],[0,0,0,0,1,0,0,0,0],[0,0,0,0,1,0,0,0,0],[0,0,0,0,1,0,0,0,0]]9777	9778,9779	[[0,0,0,1,0,0,0,0,0],[0,0,0,1,0,0,0,0,0]]9780	9781,9782,9783,9784,9785,9786,8296,9787,9788,8472,9789,9790,9791,9792,9793,9794,9795,3578,9796,3834,9797,9798,9799,9800,9801,9802,9803,9804,9805,9806	[[0,1,1,0,0,0,0,0,0],[0,0,1,0,0,0,0,0,0],[0,0,1,0,0,0,0,0,0],[0,0,1,0,0,0,0,0,0],[0,0,1,0,0,0,0,0,0],[0,0,1,0,0,0,0,0,0],[0,1,0,0,0,0,0,0,0],[0,1,1,0,0,0,0,0,0],[0,1,1,0,0,0,0,0,0],[0,1,0,0,0,0,0,0,0],[0,0,1,0,0,0,0,0,0],[0,1,1,0,0,0,0,0,0],[0,1,1,0,0,0,0,0,0],[0,1,1,0,0,0,0,0,0],[0,1,0,0,0,0,0,0,0],[0,0,1,0,0,0,0,0,0],[0,0,1,0,0,0,0,0,0],[0,1,1,1,0,0,0,0,0],[0,0,1,0,0,0,0,0,0],[0,0,1,0,0,0,0,0,0],[0,1,1,0,0,0,0,0,0],[0,0,0,0,1,0,0,0,0],[0,0,1,1,0,0,0,0,0],[0,0,1,0,0,0,0,0,0],[0,0,1,0,0,0,0,0,0],[0,0,1,0,0,0,0,0,0],[0,0,1,1,0,0,0,0,0],[0,0,1,0,0,0,0,0,0],[0,0,1,0,0,0,0,0,0],[0,0,1,0,0,0,0,0,0]]9807	9808,9809,9810,9811,9812,9813,9814,9815,9816,9817,9818,9819,9820,9821,9822,9823,9824,9825,9826,9827,9828,9829,9830,9831,9832,9833,9834,9835,9836,9837,9838,7620,9839,9840,9841,9842,9843,9844,9845,9846	[[0,1,1,1,0,0,1,1,0],[0,1,1,1,0,0,1,1,0],[0,0,0,1,0,0,0,1,0],[0,0,0,1,0,0,0,0,0],[0,1,1,1,0,0,1,1,0],[0,0,1,0,0,0,0,0,0],[0,1,1,1,0,0,1,1,0],[0,0,1,1,0,0,0,1,0],[0,0,1,1,0,0,0,1,0],[0,0,1,0,0,0,0,0,0],[0,1,1,1,0,0,0,0,0],[0,0,1,1,0,0,0,1,0],[0,0,1,0,0,0,0,0,0],[0,1,1,1,0,0,1,1,0],[0,0,1,0,0,0,0,0,0],[0,0,1,0,1,0,0,0,0],[0,1,1,1,0,0,1,0,0],[0,0,1,1,0,0,0,0,0],[0,0,0,1,0,0,0,0,0],[0,0,0,0,0,0,0,1,0],[0,1,1,1,0,0,0,0,0],[0,0,1,0,0,0,0,0,0],[0,0,1,0,0,0,0,0,0],[0,0,1,0,0,0,0,0,0],[0,0,1,1,0,0,0,1,0],[0,0,1,0,0,0,0,0,0],[0,0,1,0,0,0,0,0,0],[0,0,1,0,0,0,0,0,0],[0,0,1,0,0,0,0,0,0],[0,0,1,0,0,0,0,0,0],[0,0,0,1,0,0,0,0,0],[0,0,1,0,0,0,0,0,0],[0,0,1,0,0,0,0,0,0],[0,0,1,0,0,0,0,0,0],[0,0,1,0,0,0,0,0,0],[0,1,0,0,0,0,0,0,0],[0,1,0,0,0,0,0,0,0],[0,0,1,0,0,0,0,0,0],[0,0,1,0,0,0,0,0,0],[0,0,1,0,0,0,0,0,0]]9847	9848	[[0,0,0,0,1,0,0,0,0]]9849	9850	[[0,0,0,0,1,0,0,0,0]]9851	9852,9319,9853,8196	[[0,0,0,0,1,0,0,0,0],[0,0,0,0,1,0,0,0,0],[0,0,0,0,1,0,0,0,0],[0,0,0,0,1,0,0,0,0]]9854	9855,9856,9857	[[0,0,0,0,1,0,0,0,0],[0,0,0,0,1,0,0,0,0],[0,0,0,0,1,0,0,0,0]]9858	9859,9860,9861,9862,2668,9863,9864,9865,9866,9867	[[0,0,0,1,0,0,0,0,0],[0,0,0,1,0,0,0,0,0],[0,0,0,1,0,0,0,0,0],[0,0,0,1,0,0,0,0,0],[0,0,0,1,0,0,0,0,0],[0,0,0,1,0,0,0,0,0],[0,0,0,1,0,0,0,0,0],[0,0,0,1,0,0,0,0,0],[0,0,0,1,0,0,0,0,0],[0,0,0,1,0,0,0,0,0]]9868	9869,9870,9871,9872	[[0,0,0,0,1,0,0,0,0],[0,0,0,0,1,0,0,0,0],[0,0,0,0,1,0,0,0,0],[0,0,0,0,1,0,0,0,0]]9873	6422,9874,2434,6434,9875,9595	[[0,1,0,0,0,0,0,0,0],[0,0,0,1,0,0,0,0,0],[0,1,0,0,0,0,0,0,0],[0,1,0,0,0,0,0,0,0],[0,1,0,0,0,0,0,0,0],[0,1,0,1,0,0,0,0,0]]9876	9877,4035,5377,2991,9878,9879,440,9880,9881,9882,9883,9884,9885,9886,9887,9888,5383,9889,9890,9891,5176,9892,9362,4653,5404,9893,9566,9894,9895,2982,9896,5402,9897,9142,9898,5385,5396,1235,3446,9899,7621,9900,5405,8479,9901,9902,9903,5401,5400,9904,4687,9905,4529,8422,1814,5391	[[0,0,0,1,0,0,0,0,0],[0,0,0,1,0,0,0,0,0],[0,0,0,1,0,0,0,0,0],[0,0,0,1,0,0,0,0,0],[0,0,0,1,0,0,0,0,0],[0,0,0,1,0,0,0,0,0],[0,0,0,1,0,0,0,0,0],[0,0,0,1,0,0,0,0,0],[0,0,0,1,0,0,0,0,0],[0,0,0,1,0,0,0,0,0],[0,0,0,1,0,0,0,0,0],[0,0,0,1,0,0,0,0,0],[0,0,0,1,0,0,0,0,0],[0,0,0,1,0,0,0,0,0],[0,0,0,1,0,0,0,0,0],[0,0,0,1,0,0,0,0,0],[0,0,0,1,0,0,0,0,0],[0,0,0,1,0,0,0,0,0],[0,0,0,1,0,0,0,0,0],[0,0,0,1,0,0,0,0,0],[0,0,0,1,0,0,0,0,0],[0,0,0,1,0,0,0,0,0],[0,0,0,1,0,0,0,0,0],[0,0,0,1,0,0,0,0,0],[0,0,0,1,0,0,0,0,0],[0,0,0,1,0,0,0,0,0],[0,0,0,1,0,0,0,0,0],[0,0,0,1,0,0,0,0,0],[0,0,0,1,0,0,0,0,0],[0,0,0,1,0,0,0,0,0],[0,0,0,1,0,0,0,0,0],[0,0,0,1,0,0,0,0,0],[0,0,0,1,0,0,0,0,0],[0,0,0,1,0,0,0,0,0],[0,0,0,1,0,0,0,0,0],[0,0,0,1,0,0,0,0,0],[0,0,0,1,0,0,0,0,0],[0,0,0,1,0,0,0,0,0],[0,0,0,1,0,0,0,0,0],[0,0,0,1,0,0,0,0,0],[0,0,0,1,0,0,0,0,0],[0,0,0,1,0,0,0,0,0],[0,0,0,1,0,0,0,0,0],[0,0,0,1,0,0,0,0,0],[0,0,0,1,0,0,0,0,0],[0,0,0,1,0,0,0,0,0],[0,0,0,1,0,0,0,0,0],[0,0,0,1,0,0,0,0,0],[0,0,0,1,0,0,0,0,0],[0,0,0,1,0,0,0,0,0],[0,0,0,1,0,0,0,0,0],[0,0,0,1,0,0,0,0,0],[0,0,0,1,0,0,0,0,0],[0,0,0,1,0,0,0,0,0],[0,0,0,1,0,0,0,0,0],[0,0,0,1,0,0,0,0,0]]9906	9825,9818,9838,9808,9812,9809,9814,9819,9826,9824,9811,9832,9815,9828,9821,9816,9810	[[0,0,0,1,0,0,0,0,0],[0,0,0,1,0,0,0,0,0],[0,0,0,1,0,0,0,0,0],[0,0,0,1,0,0,0,0,0],[0,0,0,1,0,0,0,0,0],[0,0,0,1,0,0,0,0,0],[0,0,0,1,0,0,0,0,0],[0,0,0,1,0,0,0,0,0],[0,0,0,1,0,0,0,0,0],[0,0,0,1,0,0,0,0,0],[0,0,0,1,0,0,0,0,0],[0,0,0,1,0,0,0,0,0],[0,0,0,1,0,0,0,0,0],[0,0,0,1,0,0,0,0,0],[0,0,0,1,0,0,0,0,0],[0,0,0,1,0,0,0,0,0],[0,0,0,1,0,0,0,0,0]]9907	9799,9803,3578	[[0,0,0,1,0,0,0,0,0],[0,0,0,1,0,0,0,0,0],[0,0,0,1,0,0,0,0,0]]9908	9253,9909,9910,9911,9912,9913,9914,9915,4141,9916,9536,9534,9917,9918,3537,9919,9920,9519,9921,9922,9923,9924,9925,4614,9926,4564,9526,9927,9928	[[0,0,1,0,1,0,0,0,0],[0,0,1,0,1,0,0,0,0],[0,0,0,0,1,0,0,0,0],[0,0,1,0,1,0,0,0,0],[0,0,1,0,0,0,0,0,0],[0,0,1,0,0,0,0,0,0],[0,0,0,0,1,0,0,0,0],[0,0,1,0,0,0,0,0,0],[0,0,1,0,0,0,0,0,0],[0,0,1,0,0,0,0,0,0],[0,0,1,0,0,0,0,0,0],[0,0,1,0,0,0,0,0,0],[0,0,1,0,0,0,0,0,0],[0,0,1,1,0,0,0,0,0],[0,0,1,0,0,0,0,0,0],[0,0,1,1,0,0,0,0,0],[0,0,1,0,0,0,0,0,0],[0,0,1,0,0,0,0,0,0],[0,0,1,0,0,0,0,0,0],[0,0,1,0,0,0,0,0,0],[0,0,1,1,0,0,0,0,0],[0,0,1,0,0,0,0,0,0],[0,0,1,0,0,0,0,0,0],[0,0,1,0,0,0,0,0,0],[0,0,1,0,0,0,0,0,0],[0,0,1,0,0,0,0,0,0],[0,0,1,0,0,0,0,0,0],[0,0,0,0,1,0,0,0,0],[0,0,1,0,0,0,0,0,0]]9929	9930,9931,9932,5663,9933,8974,4976,6057,5355,9934,5701,9935,5707,5348,5145,5710,9936,1543,7906,9937,9938,9939,9940,7048,9941,9942,9943,7965,9944,9945,9946,7968,6926,5346,9947,5349,6064,5363,9948,5706,9949,6075,5702,9950,3890,9951,5714,6058	[[1,0,0,0,0,0,0,0,0],[1,0,0,0,1,0,0,0,0],[0,0,0,0,1,0,0,0,0],[0,0,1,0,0,0,0,0,0],[1,1,1,1,0,0,1,0,0],[1,0,0,0,0,0,0,0,0],[1,1,1,0,1,0,1,0,0],[1,1,1,1,1,0,1,0,0],[1,1,0,0,0,0,0,0,0],[1,0,0,0,0,0,0,0,0],[1,0,1,0,0,0,0,0,0],[1,0,0,0,1,0,0,0,0],[1,0,1,0,0,0,0,0,0],[1,0,0,0,0,0,0,0,0],[1,0,0,0,1,0,0,0,0],[1,0,1,0,0,0,0,0,0],[0,0,1,0,0,0,0,0,0],[0,0,1,0,0,0,0,0,0],[0,1,1,0,0,0,1,0,0],[0,1,1,0,0,0,1,0,0],[0,1,1,1,0,0,1,0,0],[0,0,1,0,0,0,0,0,0],[0,0,1,0,0,0,0,0,0],[0,0,0,0,1,0,0,0,0],[0,0,0,0,1,0,0,0,0],[0,0,1,0,0,0,0,0,0],[0,1,0,0,0,0,1,0,0],[0,0,1,0,0,0,0,0,0],[0,0,1,0,0,0,0,0,0],[0,0,1,0,0,0,0,0,0],[0,0,1,0,0,0,0,0,0],[0,0,1,0,0,0,0,0,0],[0,0,1,0,0,0,0,0,0],[0,1,1,1,1,0,1,0,0],[0,1,0,0,0,0,0,0,0],[0,0,0,0,1,0,0,0,0],[0,1,1,0,0,0,1,0,0],[0,1,1,0,0,0,0,0,0],[0,0,1,0,0,0,0,0,0],[0,0,1,0,0,0,0,0,0],[0,0,1,0,0,0,0,0,0],[0,0,1,0,0,0,0,0,0],[0,0,1,0,0,0,0,0,0],[0,1,0,0,0,0,0,0,0],[0,0,1,0,0,0,0,0,0],[1,0,0,0,0,0,0,0,0],[0,0,1,0,0,0,0,0,0],[0,0,1,0,0,0,0,0,0]]9952	9953,9954,9955,9956,2032,9957,9958,9567,1060,9959,9960,4634,9961,9962,9963,9352,9964,9965,8722,1975,9966,9967,9968,9969,9970,8787,9971,9896,9972,4629,9973,1044,4635,9974,9975,9976,9977,9978,1099,9979,9980,9981,1067,2055,8790,1997,9982,9983,9984,1048,9985,9986,550,9987	[[0,1,1,0,0,0,0,0,0],[0,1,1,0,0,0,0,1,0],[0,1,1,0,0,0,0,0,0],[0,1,0,0,0,0,0,0,0],[0,1,1,1,0,0,0,1,0],[0,1,1,0,0,0,0,1,0],[0,1,1,0,0,0,0,1,0],[0,1,1,1,0,0,0,1,0],[0,0,1,0,0,0,0,1,0],[0,0,1,0,0,0,0,0,0],[0,1,1,0,0,0,0,0,0],[0,0,1,0,0,0,0,0,0],[0,0,1,0,0,0,0,0,0],[0,1,1,0,0,0,0,0,0],[0,0,1,0,0,0,0,0,0],[0,0,1,0,0,0,0,0,0],[0,1,0,0,0,0,0,1,0],[0,1,1,0,0,0,0,0,0],[0,0,1,0,0,0,0,0,0],[0,0,1,0,0,0,0,0,0],[0,1,1,0,0,0,0,1,0],[0,0,1,0,0,0,0,0,0],[0,0,1,0,0,0,0,0,0],[0,1,0,0,0,0,0,0,0],[0,0,1,0,0,0,0,0,0],[0,0,0,0,0,0,0,1,0],[0,0,1,0,0,0,0,0,0],[0,0,1,0,0,0,0,0,0],[0,0,1,0,0,0,0,0,0],[0,0,1,0,0,0,0,0,0],[0,0,0,1,0,0,0,1,0],[0,0,0,0,0,0,0,1,0],[0,0,1,0,0,0,0,0,0],[0,0,1,0,0,0,0,0,0],[0,0,1,0,0,0,0,0,0],[0,0,1,0,0,0,0,0,0],[0,0,1,0,0,0,0,0,0],[0,0,1,0,0,0,0,0,0],[0,0,1,0,0,0,0,0,0],[0,0,1,0,0,0,0,0,0],[0,0,1,0,0,0,0,0,0],[0,0,1,0,0,0,0,0,0],[0,0,1,0,0,0,0,0,0],[0,0,0,0,0,0,0,1,0],[0,0,1,0,0,0,0,1,0],[0,0,1,0,0,0,0,0,0],[0,0,0,0,0,0,0,1,0],[0,0,1,0,0,0,0,0,0],[0,0,0,0,0,0,0,1,0],[0,0,1,0,0,0,0,0,0],[0,0,1,0,0,0,0,0,0],[0,0,0,0,0,0,0,1,0],[0,0,1,0,0,0,0,0,0],[0,0,1,0,0,0,0,0,0]]9988	1218,1064,1109,1063,1217,9989,1062,4680,1105,9990,1644,1060	[[0,0,0,1,0,0,0,0,0],[0,0,0,1,0,0,0,0,0],[0,0,0,1,0,0,0,0,0],[0,0,0,1,0,0,0,0,0],[0,0,0,1,0,0,0,0,0],[0,0,0,1,0,0,0,0,0],[0,0,0,1,0,0,0,0,0],[0,0,0,1,0,0,0,0,0],[0,0,0,1,0,0,0,0,0],[0,0,0,1,0,0,0,0,0],[0,0,0,1,0,0,0,0,0],[0,0,0,1,0,0,0,0,0]]9991	9992,4502,9993,9994	[[0,0,0,1,0,0,0,0,0],[0,0,0,1,0,0,0,0,0],[0,0,0,1,0,0,0,0,0],[0,0,0,1,0,0,0,0,0]]9995	9996	[[0,0,0,0,1,0,0,0,0]]9997	9998	[[0,0,0,0,1,0,0,0,0]]9999	9064,9065,2878,9248,5730,9245	[[0,0,0,0,1,0,0,0,0],[0,0,0,0,1,0,0,0,0],[0,0,0,0,1,0,0,0,0],[0,0,0,0,1,0,0,0,0],[0,0,0,0,1,0,0,0,0],[0,0,0,0,1,0,0,0,0]]10000	10001,10002,10003,10004,10005,10006,10007	[[0,0,0,0,1,0,0,0,0],[0,0,0,0,1,0,0,0,0],[0,0,0,0,1,0,0,0,0],[0,0,0,0,1,0,0,0,0],[0,0,0,0,1,0,0,0,0],[0,0,0,0,1,0,0,0,0],[0,0,0,0,1,0,0,0,0]]10008	10009	[[0,0,0,0,1,0,0,0,0]]10010	8699,5167,5168,10011,10012,7801,9421,10013,3139,8942,10014,10015,860,1543,8941,4208,5173,5366,10016,4215,1601,5720,10017,10018,6277,4214,10019,10020,10021,8003,10022,6930,10023,10024,10025,2495,6142,10026,6622,10027,3940,4189,10028,895,10029,10030,10031,1769,10032,10033,10034,7981,750,10035,3047,5123,10036,10037,6948,9066,10038,10039,10040,5156,2515,3652,4226,9412,3120,6928,10041,10042,10043,10044,5485,3050,10045,10046,7980,5153,2435,2178,1640,7996,10047,5012,8602,10048,10049,3111,3609,10050,7323,5743,2353,1604	[[0,0,1,1,1,1,0,0,1],[0,0,0,1,0,0,0,0,0],[0,0,1,1,0,0,0,1,0],[0,0,1,0,0,0,0,0,0],[0,0,1,1,1,1,0,0,1],[1,0,1,0,0,0,1,0,0],[0,0,1,0,0,0,0,0,0],[0,0,0,1,0,0,0,1,0],[0,0,0,1,0,0,0,1,0],[0,0,1,1,0,0,0,1,0],[0,0,1,0,0,0,0,0,0],[0,0,1,0,0,0,0,0,0],[0,0,1,0,0,0,0,1,0],[0,0,1,1,0,0,0,0,0],[0,1,1,1,1,1,1,1,1],[0,0,0,1,0,0,0,1,0],[0,0,1,0,0,0,0,0,0],[0,0,1,1,0,1,0,0,1],[0,0,0,1,0,0,0,1,0],[0,0,0,1,0,0,0,0,0],[0,0,1,1,0,0,0,0,1],[0,0,0,1,0,0,0,0,1],[0,0,1,0,0,0,0,0,0],[0,1,1,1,0,0,0,1,0],[0,0,1,0,0,0,0,0,0],[0,0,0,1,0,0,0,0,0],[0,0,0,1,0,0,0,0,0],[0,0,1,0,0,0,0,0,0],[0,0,0,0,1,0,0,0,0],[0,0,1,1,0,1,0,0,1],[0,0,1,0,0,0,0,0,0],[0,1,1,1,0,0,0,1,0],[0,0,0,1,0,0,0,0,1],[0,0,1,0,0,0,0,0,0],[0,0,0,1,0,0,0,1,0],[0,0,0,0,0,0,0,0,1],[0,0,0,1,0,0,0,0,0],[0,1,1,1,0,0,1,1,0],[0,0,0,1,0,0,0,0,0],[0,1,0,0,0,0,1,0,0],[0,0,1,0,0,0,0,0,0],[0,0,0,1,0,0,0,0,0],[0,0,0,0,1,0,0,0,0],[1,1,1,1,0,0,1,1,0],[0,0,1,0,0,0,0,0,0],[0,0,1,0,0,0,0,0,0],[0,0,0,1,0,0,0,0,0],[0,0,0,1,0,0,0,0,0],[0,0,0,0,0,0,0,0,1],[0,0,1,1,0,0,0,0,0],[0,0,1,0,0,0,0,0,0],[1,1,1,0,0,0,0,1,0],[0,0,0,1,0,0,0,1,0],[0,0,0,1,0,0,0,0,0],[0,1,1,1,0,0,0,1,0],[0,0,0,0,0,0,0,0,1],[0,0,0,0,0,0,1,0,0],[0,1,1,1,0,1,0,1,1],[0,0,0,0,0,0,0,1,0],[1,0,1,1,0,0,0,1,0],[0,0,0,1,0,1,0,1,0],[0,0,1,0,0,0,0,0,0],[0,0,0,1,0,0,0,0,1],[0,0,1,0,0,0,0,0,0],[0,0,1,0,0,0,0,0,0],[0,0,1,0,0,0,0,0,0],[0,0,1,1,0,0,0,1,0],[0,0,0,1,0,0,0,0,0],[0,0,1,1,0,0,0,1,0],[0,1,1,1,1,0,1,0,0],[0,1,1,1,0,1,0,1,1],[0,1,1,0,0,0,0,0,0],[0,0,1,1,0,0,0,0,0],[0,0,1,0,0,0,0,0,0],[1,0,1,1,1,1,0,0,0],[0,1,1,1,0,0,1,1,0],[1,0,1,0,0,0,0,0,0],[0,0,0,1,0,0,0,1,0],[0,1,0,1,0,0,1,0,0],[0,1,1,1,0,0,1,1,0],[0,1,1,1,0,0,0,1,0],[1,1,1,1,1,0,1,1,0],[1,1,0,0,0,0,1,0,0],[1,0,0,0,0,0,0,0,0],[1,0,0,0,0,0,0,0,0],[0,0,0,1,0,0,0,1,0],[0,0,1,1,1,1,0,1,1],[0,0,1,1,0,1,0,0,1],[0,0,0,0,0,0,0,1,0],[0,0,1,0,0,0,0,0,0],[0,0,1,0,0,0,0,0,0],[0,0,0,1,0,0,0,0,0],[0,0,0,1,0,0,0,1,0],[0,0,1,0,0,0,0,0,0],[0,0,0,1,0,0,0,1,0],[0,0,1,1,0,0,0,0,0]]10051	10052	[[0,0,0,0,1,0,0,0,0]]10053	10054	[[0,0,0,0,1,0,0,0,0]]10055	10056	[[0,0,0,0,1,0,0,0,0]]10057	10058,10059,10060,10061,10062,9993,9416,9994,9937,10063,10064,10065,10066,10067,6071,9943,6075,4502,10068,10069,6057,10070,10071,10072,10073,10074,10075,10076,6092,6054,10077,10078,1716,10079,10080,10081,10082,10083,10084,10085,4455,10086,10087,10088,10089,10090,10091,9992,10092,4755,10093,10094,10095	[[0,1,0,0,0,0,0,0,0],[0,1,1,0,0,0,0,0,0],[0,0,1,0,0,0,0,1,0],[0,1,1,0,0,0,1,0,0],[0,0,1,0,0,0,0,0,0],[0,1,1,1,1,0,1,0,0],[0,1,1,0,0,0,1,1,0],[0,1,1,1,1,0,1,1,0],[0,1,0,0,0,0,1,0,0],[0,1,1,0,1,0,0,1,0],[0,1,0,0,0,0,1,0,0],[0,1,1,0,0,0,1,1,0],[0,1,0,0,0,0,1,1,0],[0,1,0,0,0,0,1,1,0],[0,1,1,0,1,0,1,0,0],[0,1,0,0,0,0,0,0,0],[0,1,1,0,0,0,1,1,0],[0,1,1,1,0,0,1,1,0],[0,1,0,0,0,0,1,0,0],[0,1,0,0,0,0,1,1,0],[0,1,1,0,0,0,1,1,0],[0,1,0,0,0,0,0,0,0],[0,1,0,0,0,0,1,0,0],[0,1,0,0,0,0,0,0,0],[0,1,0,0,1,0,0,0,0],[0,1,0,0,0,0,0,0,0],[0,1,1,0,0,0,1,1,0],[0,1,1,0,0,0,1,0,0],[0,1,0,0,0,0,1,0,0],[0,1,0,0,0,0,1,1,0],[0,1,1,0,1,0,1,1,0],[0,1,1,0,0,0,1,0,0],[0,0,1,0,0,0,0,0,0],[0,0,0,0,0,0,0,1,0],[0,0,1,0,0,0,0,0,0],[0,0,1,0,0,0,0,0,0],[0,0,1,0,0,0,0,0,0],[0,0,1,0,0,0,0,0,0],[0,0,1,0,0,0,0,0,0],[0,0,1,0,0,0,0,1,0],[0,0,1,0,0,0,0,0,0],[0,0,1,0,0,0,0,0,0],[0,0,0,0,1,0,1,0,0],[0,0,1,0,0,0,0,0,0],[0,0,1,0,0,0,0,0,0],[0,0,0,0,0,0,1,0,0],[0,0,1,0,0,0,0,0,0],[0,0,1,1,0,0,0,1,0],[0,0,1,0,0,0,0,0,0],[0,0,0,0,1,0,0,0,0],[0,0,1,0,0,0,0,0,0],[0,0,1,0,0,0,0,0,0],[0,0,0,0,0,0,0,1,0]]10096	1059,10097,10098,1106,9989,1069,1218,1644,10099,10100,9990,1109,3667,1215,1224,9126,1213,1093,10101,2051,1105,1220,10102,4717,10103,2035,10104,4622,10105,7901,1046,4387,1102,4680,1053,1060,9192,1221,4696,10106,10107,10108,1217,1399,4702,10109,1049,10110,10111,10112,1064,1062,1063,10113,1061,1045,10114,1057,4629,10115,4981,1222,10116,10117,4679,1210,1214,5317,10118,10119,1344	[[0,1,1,0,1,0,0,1,0],[0,1,1,0,0,0,0,0,0],[0,0,0,0,1,0,0,0,0],[0,1,0,0,0,0,0,0,0],[0,1,1,1,0,0,0,1,0],[0,0,1,0,0,0,0,0,0],[0,1,1,1,0,0,0,1,0],[0,0,0,1,0,0,0,0,0],[0,0,0,0,1,0,1,0,0],[0,0,1,0,0,0,0,0,0],[0,1,1,1,0,0,1,0,0],[0,1,0,1,0,0,0,0,0],[0,0,0,0,0,0,0,1,0],[0,0,1,0,0,0,0,0,0],[0,1,0,0,0,0,1,0,0],[0,0,1,0,0,0,0,0,0],[0,0,1,0,0,0,0,0,0],[0,0,0,0,0,0,0,1,0],[0,1,0,0,0,0,1,0,0],[0,1,0,0,0,0,1,0,0],[0,1,0,1,0,0,1,0,0],[0,1,0,0,0,0,1,0,0],[0,1,1,0,0,0,0,1,0],[0,0,1,0,0,0,0,0,0],[0,1,0,0,0,0,0,0,0],[0,1,0,0,0,0,1,0,0],[0,1,0,0,0,0,0,0,0],[0,1,1,0,0,0,1,1,0],[0,1,0,0,0,0,0,0,0],[0,1,0,0,0,0,0,0,0],[0,1,1,0,1,0,1,0,0],[0,1,0,0,0,0,1,0,0],[0,1,0,0,0,0,1,0,0],[0,1,1,1,0,0,1,1,0],[0,1,1,0,0,0,0,0,0],[0,1,0,1,0,0,1,0,0],[0,0,0,0,1,0,0,0,0],[0,1,0,0,1,0,0,1,0],[0,1,1,0,0,0,0,1,0],[0,0,1,0,0,0,0,0,0],[0,0,1,0,0,0,0,0,0],[0,0,0,0,1,0,0,0,0],[0,1,1,1,0,0,1,1,0],[0,1,1,0,0,0,1,0,0],[0,1,1,0,0,0,0,1,0],[0,1,1,0,0,0,0,1,0],[0,1,0,0,0,0,1,1,0],[0,0,1,0,0,0,0,1,0],[0,0,1,0,0,0,0,0,0],[0,0,0,0,1,0,0,0,0],[0,1,1,1,1,0,1,0,0],[0,1,0,1,0,0,1,1,0],[0,1,1,1,0,0,1,0,0],[0,0,1,0,0,0,0,0,0],[0,0,1,0,0,0,0,0,0],[0,0,1,0,0,0,0,0,0],[0,0,1,0,0,0,0,0,0],[0,0,0,0,0,0,0,1,0],[0,1,1,0,0,0,1,0,0],[0,1,0,0,0,0,0,0,0],[0,0,0,0,0,0,0,1,0],[0,0,1,0,0,0,0,0,0],[0,0,1,0,0,0,0,0,0],[0,0,0,0,1,0,0,0,0],[0,0,0,0,0,0,0,1,0],[0,0,1,0,0,0,0,0,0],[0,0,1,0,0,0,0,0,0],[0,0,1,0,0,0,0,0,0],[0,0,0,0,1,0,0,0,0],[0,0,1,0,0,0,0,0,0],[0,0,0,0,0,0,0,1,0]]10120	10121,10122,10123,10124	[[0,0,0,0,1,0,0,0,0],[0,0,0,0,1,0,0,0,0],[0,0,0,0,1,0,0,0,0],[0,0,0,0,1,0,0,0,0]]10125	4184	[[0,0,0,0,1,0,0,0,0]]10126	10127,7943,10128,10129,10063,10059,6071,10130,7209,7317,6075,10131,10132,10133,10134,6291,10071,10087,10135,6057,6098,10136,7305,6064,6054,10137,6300,6069,10078,10138,9481,10139,10140,7288,10141,10142,10143,6295,10144,10145,10146,10147,5369,10094,10148,10149,6311,10150,10151,10152,5367,10153,10154,10155,10156,10157,10158,10159,6316,7966,10160,10161,9487,6926,10162,9946,10163,10164,10165,10166,10167,10168	[[0,1,1,1,0,0,1,0,0],[0,0,0,1,0,0,0,0,0],[0,0,1,0,0,0,0,0,0],[0,0,0,0,1,0,0,0,0],[1,1,1,1,0,0,0,0,1],[0,0,0,0,1,0,0,0,0],[0,1,0,1,0,0,1,0,1],[0,0,0,0,1,0,0,0,0],[0,0,0,0,0,0,0,0,1],[0,0,1,0,0,0,0,0,0],[1,1,0,1,1,0,1,0,0],[1,1,1,1,1,0,1,0,1],[0,0,1,0,0,0,0,0,0],[0,0,1,0,0,0,0,0,0],[0,0,1,0,0,0,0,0,0],[0,1,0,1,0,0,0,0,0],[1,1,0,1,0,0,0,0,0],[0,0,0,0,0,0,1,0,0],[0,0,0,1,0,0,0,0,0],[1,1,1,1,0,0,1,0,1],[1,1,0,1,0,0,1,0,0],[1,1,1,1,0,0,1,0,0],[1,1,0,1,0,0,0,0,0],[1,1,0,1,0,0,1,0,0],[1,1,0,0,0,0,0,0,0],[1,0,1,0,0,0,0,0,1],[1,0,0,1,0,0,0,0,0],[1,0,0,0,0,0,0,0,0],[1,0,0,0,0,0,0,0,0],[1,0,0,0,0,0,0,0,0],[0,0,1,0,0,0,0,0,0],[0,0,0,1,0,0,0,0,1],[0,0,0,0,1,0,0,0,0],[0,0,1,0,0,0,0,0,0],[0,0,0,0,1,0,0,0,0],[0,0,0,1,0,0,0,0,0],[0,1,0,0,0,0,0,0,0],[0,0,0,1,0,0,0,0,0],[0,0,1,1,1,0,0,0,0],[0,0,1,0,0,0,0,0,0],[0,0,1,0,0,0,0,0,0],[0,1,0,1,1,0,1,0,0],[0,0,0,1,0,0,0,0,0],[0,0,1,0,0,0,0,0,0],[0,1,0,0,0,0,0,0,0],[0,1,0,0,0,0,0,0,0],[0,1,0,0,0,0,0,0,0],[0,1,0,0,0,0,0,0,0],[0,1,1,0,0,0,0,0,0],[0,1,1,1,0,0,1,0,0],[0,0,0,1,0,0,0,0,0],[0,0,1,0,0,0,0,0,0],[0,0,1,0,0,0,0,0,0],[0,1,0,0,0,0,0,0,0],[0,0,1,1,0,0,0,0,1],[0,0,1,0,1,0,0,0,0],[0,0,1,0,0,0,0,0,0],[0,0,1,0,0,0,0,0,0],[0,0,0,1,0,0,0,0,0],[0,0,0,1,0,0,0,0,0],[0,0,1,0,0,0,0,0,0],[0,0,1,0,1,0,0,0,0],[0,0,1,0,0,0,0,0,0],[0,0,1,0,0,0,0,0,0],[0,0,1,1,0,0,0,0,0],[0,0,1,0,0,0,0,0,0],[0,0,1,0,0,0,0,0,0],[0,0,1,0,0,0,0,0,0],[0,0,0,1,0,0,0,0,0],[0,0,1,0,0,0,0,0,0],[0,0,1,0,0,0,0,0,0],[0,0,0,1,0,0,0,0,0]]10169	10170,6181,10171,10172	[[0,0,0,0,1,0,0,0,0],[0,0,0,0,1,0,0,0,0],[0,0,0,0,1,0,0,0,0],[0,0,0,0,1,0,0,0,0]]10173	10174,10175,10176	[[0,0,0,0,1,0,0,0,0],[0,0,0,0,1,0,0,0,0],[0,0,0,0,1,0,0,0,0]]10177	7314,10178,7297,7313,10179,10180,10181,3196,10182	[[0,1,0,0,0,0,0,0,0],[0,1,0,0,0,0,0,0,0],[0,1,0,0,0,0,0,0,0],[0,1,0,0,0,0,0,0,0],[0,1,0,0,0,0,0,0,0],[0,1,0,0,0,0,0,0,0],[0,1,0,0,0,0,0,0,0],[0,0,0,0,1,0,0,0,0],[0,1,0,0,0,0,0,0,0]]10183	10184,10185,10186,10187,10188,10189,10190,10191,10192,2472,10193,10194,10195	[[0,0,0,1,0,0,0,0,0],[0,0,0,1,0,0,0,0,0],[0,0,0,1,0,0,0,0,0],[0,0,0,1,0,0,0,0,0],[0,0,0,1,1,0,0,0,0],[0,1,0,1,0,0,0,0,0],[0,1,0,1,0,0,0,0,0],[0,0,0,1,0,0,0,0,0],[0,0,0,1,0,0,0,0,0],[0,0,0,1,0,0,0,0,0],[0,0,0,1,0,0,0,0,0],[0,0,0,1,0,0,0,0,0],[0,0,0,1,0,0,0,0,0]]10196	10197,2412,10198,10199,10200,7209,10201,10202,2299,10203,10204,2213,10205,5087,10206	[[0,0,1,1,0,0,0,0,0],[0,0,1,0,0,0,0,0,0],[0,0,1,0,1,0,0,0,0],[0,0,1,0,0,0,0,0,0],[0,0,1,0,0,0,0,0,0],[0,0,0,0,1,0,0,0,0],[0,0,0,0,1,0,0,0,0],[0,0,1,0,0,0,0,0,0],[0,0,0,0,1,0,0,0,0],[0,0,1,1,0,0,0,0,0],[0,0,1,1,0,0,0,0,0],[0,0,1,0,0,0,0,0,0],[0,0,0,0,1,0,0,0,0],[0,0,1,0,0,0,0,0,0],[0,0,0,0,1,0,0,0,0]]10207	10208,10209,10210,8419,10211,10212,64,1062,10213,10214,4696,7517,1067,9965,1045,10215,10216,1061,10217,4702,9367,10218,10219,8790,8458,4687,1058,10220,8927,2038,10221,1048,10222,1696,10223,9985,4701,6629,10224,8474,8907,8889,10225,10226,10227,1976,10228,10229,9170,10230,10231,10114,6537,2400,5510,2006,10232,10233,10234,1603,4615,1046,8794,1050,10235,10236,4286,5130,10237,10238,7545,10239,550,10240,4302,10241,1072,10242,10243,10244,10245,9990,2057,7547,10246,10247,10248,10249,10250,79,10251,10252,10253,4688,10254,10255,10256,10257,90,1099,9352,10258,10259,10260,10261,10262,10263,10264,10265,10266,10267,6906,7526,9371,1660,10268,7523,10269,7529,8755,10270,9348,1197,10271,5400,9191,1814,2051,1217,8784,422,1396,4672,10272,10273,10274,10275,7572,2043,10276,10277,10278,10279,9953,5611,10280,4680,2053,4618,10281,10282,10283,10284,1982,4635,8903,1069,1097,10285,1092,1095,7535,2026,1060,10286,4225,10287,10288,9366,8888,10289,1053,10290,2032,10291,2033,10292,8776,10293,4642,10294,4707,8465,9703,4682,1644,2017,10295,1218,8678,1102,42,2016,1109,6277,9126,4529,1093,4664,8788	[[0,0,1,0,0,0,0,0,0],[0,0,1,0,0,0,0,0,0],[0,0,1,0,0,0,0,1,0],[0,0,1,0,1,0,0,0,1],[0,0,1,0,0,0,0,0,0],[0,0,1,0,0,0,0,0,0],[0,0,1,0,0,0,0,0,0],[0,1,1,0,0,0,1,1,0],[0,1,0,0,0,0,0,0,0],[0,1,1,0,0,0,1,0,0],[0,0,1,0,0,1,0,0,0],[0,0,1,0,0,0,0,0,0],[0,1,1,1,0,0,0,1,0],[0,0,1,0,0,0,0,0,0],[0,0,1,0,0,0,0,0,0],[0,0,1,0,0,0,0,0,0],[0,1,1,0,0,0,0,0,0],[0,0,1,0,0,0,0,0,0],[0,0,0,0,0,0,0,1,0],[0,0,1,0,0,0,0,0,0],[0,1,1,1,0,0,0,1,0],[0,0,0,0,0,0,0,1,0],[0,1,1,0,0,0,1,0,0],[0,1,1,1,0,1,0,1,1],[0,1,1,1,0,0,0,1,1],[0,1,1,0,0,0,1,0,0],[0,1,1,0,0,0,0,1,0],[0,0,1,0,0,0,0,0,0],[0,0,1,0,0,0,0,0,0],[0,0,1,0,0,0,0,0,0],[0,0,1,0,0,0,0,0,0],[0,0,0,0,0,0,0,1,0],[0,0,1,0,0,0,0,0,0],[0,0,1,0,0,0,0,0,0],[0,0,1,0,0,0,0,0,0],[0,0,1,0,0,0,0,0,0],[0,1,1,0,0,0,0,1,0],[0,0,1,0,0,0,0,1,0],[0,0,1,0,0,0,0,0,0],[0,0,1,0,0,0,0,0,1],[0,0,1,0,0,0,0,0,0],[0,0,1,0,0,0,0,0,0],[0,0,1,0,0,0,0,0,0],[0,0,1,0,0,0,0,0,0],[0,0,1,0,0,0,0,0,0],[0,0,1,0,0,0,0,0,0],[0,1,1,0,0,0,0,1,0],[0,0,1,0,0,0,0,0,0],[0,0,1,0,0,0,0,0,0],[0,0,1,0,0,0,0,0,0],[0,0,1,0,0,0,0,0,0],[0,0,1,0,0,0,0,0,0],[0,0,0,0,1,0,0,1,0],[0,0,1,0,0,0,0,0,0],[0,1,1,0,0,0,1,0,0],[0,1,1,0,0,0,1,1,0],[0,1,1,0,0,0,0,1,0],[0,0,1,0,0,0,0,0,0],[0,0,1,0,0,0,0,0,0],[0,0,1,0,0,0,0,0,0],[0,1,1,0,0,0,0,0,0],[0,0,1,0,0,0,0,0,0],[0,0,1,0,0,0,0,0,0],[0,0,1,0,0,0,0,0,0],[0,0,1,0,0,0,0,0,0],[0,0,1,0,0,0,0,0,0],[0,0,1,0,0,0,0,0,0],[0,0,1,0,0,0,0,0,0],[0,1,0,0,0,0,0,0,0],[0,1,1,0,0,0,1,1,0],[0,1,0,0,0,0,1,0,0],[0,0,1,0,0,0,0,0,0],[0,1,1,1,0,0,0,0,0],[0,0,1,0,0,0,0,0,0],[0,0,0,0,0,0,0,0,1],[0,0,1,0,0,0,0,0,0],[0,1,0,0,0,0,0,0,0],[0,1,1,0,0,0,1,1,0],[0,1,0,0,0,0,0,1,0],[0,0,1,0,0,0,0,0,0],[0,0,1,0,0,0,0,0,0],[0,0,1,0,0,0,0,0,0],[0,0,1,0,0,0,0,0,0],[0,0,1,0,0,0,0,0,0],[0,0,1,0,0,0,0,0,0],[0,0,1,0,0,0,0,0,0],[0,0,1,0,0,0,0,0,0],[0,0,1,0,0,0,0,0,0],[0,0,1,0,0,0,0,0,0],[0,0,0,0,0,0,0,1,0],[0,0,1,0,0,0,0,0,0],[0,0,1,0,0,0,0,0,0],[0,0,1,0,0,0,0,0,0],[0,0,0,0,0,0,0,1,0],[0,0,1,0,0,0,0,0,0],[0,0,1,0,0,0,0,0,0],[0,0,1,0,0,0,0,0,0],[0,0,1,0,0,0,0,0,0],[0,0,1,0,0,0,0,0,0],[0,1,1,0,1,1,1,1,1],[0,1,1,0,0,0,0,0,0],[0,0,1,0,0,0,0,0,0],[0,0,1,0,0,0,0,0,0],[0,0,1,0,0,0,0,0,0],[0,0,1,0,0,0,0,0,0],[0,0,1,0,0,0,0,0,0],[0,0,1,0,0,0,0,1,1],[0,0,1,0,0,0,0,0,0],[0,0,1,0,0,0,0,0,0],[0,0,1,0,0,0,0,0,0],[0,1,0,0,1,0,0,1,0],[0,1,0,0,0,0,0,0,0],[0,1,1,0,1,0,1,1,0],[0,1,1,0,0,0,0,0,0],[0,0,1,0,0,0,0,0,0],[0,0,1,0,0,0,0,0,0],[0,0,1,0,0,0,0,0,0],[0,0,0,0,0,0,0,1,0],[0,0,1,0,0,0,0,1,0],[0,0,1,0,0,0,0,0,0],[0,0,1,0,0,0,0,0,0],[0,1,0,0,0,0,0,0,0],[0,1,1,0,0,0,0,1,0],[0,1,0,0,0,0,0,1,0],[0,1,0,0,0,0,0,1,0],[0,1,0,0,0,0,0,0,0],[0,0,1,0,0,0,0,1,0],[0,1,1,1,0,1,1,0,1],[0,1,1,1,1,1,1,1,1],[0,1,1,0,0,0,1,0,0],[0,0,0,0,0,0,0,1,0],[0,0,1,0,0,0,0,0,0],[0,1,1,0,0,0,1,0,0],[0,0,1,0,0,0,0,0,0],[0,0,1,0,0,0,0,0,0],[0,0,0,1,0,0,0,1,0],[0,0,1,0,0,0,0,0,0],[0,0,0,0,0,0,0,1,0],[0,0,1,0,0,0,0,1,0],[0,0,1,0,0,0,0,0,0],[0,0,1,0,0,0,0,0,0],[0,0,1,0,0,0,0,1,0],[0,1,1,0,0,0,1,1,0],[0,0,1,0,0,0,0,0,0],[0,0,1,1,0,1,0,1,1],[0,0,1,0,0,0,0,0,0],[0,0,1,0,0,0,0,0,0],[0,0,1,0,0,1,0,0,0],[0,1,1,0,0,0,1,0,0],[0,1,1,1,0,0,0,1,0],[0,0,1,0,0,0,0,0,0],[0,0,1,0,0,0,0,0,0],[0,1,0,0,0,0,0,0,0],[0,0,1,0,0,0,0,0,0],[0,1,1,0,0,1,1,1,1],[0,0,1,0,0,0,0,0,0],[0,0,1,0,0,0,0,1,0],[0,0,1,0,0,0,0,0,0],[0,1,1,0,0,0,1,0,0],[0,0,1,0,0,0,0,0,0],[0,0,1,0,0,0,0,0,0],[0,0,1,0,0,0,0,0,0],[0,1,1,0,0,0,0,1,0],[0,1,1,0,1,1,1,0,0],[0,1,1,0,0,1,1,1,0],[0,0,1,0,0,0,0,0,0],[0,0,0,0,0,0,0,1,0],[0,0,1,0,0,0,0,0,0],[0,0,1,0,0,0,0,1,0],[0,0,1,0,0,0,0,0,0],[0,0,1,0,0,0,0,1,0],[0,0,1,0,0,0,0,0,0],[0,0,1,0,0,0,0,0,0],[0,0,1,1,0,0,0,1,0],[0,0,1,0,0,0,0,0,0],[0,0,1,0,0,1,0,0,0],[0,0,1,0,0,0,0,0,0],[0,0,0,1,0,0,0,1,0],[0,0,1,0,0,0,0,1,0],[0,0,1,0,0,0,0,0,0],[0,0,1,0,0,1,0,0,0],[0,0,1,0,0,0,0,0,0],[0,0,1,0,0,0,0,0,0],[0,0,1,0,0,0,0,0,0],[0,0,1,0,0,0,0,1,0],[0,0,0,0,1,0,0,0,0],[0,1,0,0,0,0,1,0,1],[0,0,1,0,0,0,0,0,0],[0,0,1,0,0,0,0,0,0],[0,0,0,0,0,0,0,1,0],[0,0,1,0,0,0,0,0,0],[0,0,1,0,0,0,0,0,0],[0,0,1,0,0,0,0,0,0],[0,0,1,0,0,0,0,0,0],[0,0,0,0,1,0,0,0,0],[0,0,1,1,0,1,0,1,1],[0,0,0,0,0,1,0,0,0],[0,0,1,0,0,0,0,0,0],[0,0,1,0,0,0,0,0,0],[0,0,1,0,0,0,0,0,0]]10296	10297,10298,10299	[[0,0,0,1,0,0,0,0,0],[0,0,0,1,0,0,0,0,0],[0,0,0,1,0,0,0,0,0]]10300	10301,10302,10303,10304	[[0,0,0,0,1,0,0,0,0],[0,0,0,0,1,0,0,0,0],[0,0,0,0,1,0,0,0,0],[0,0,0,0,1,0,0,0,0]]10305	10306	[[0,0,0,0,1,0,0,0,0]]10307	10308,10309,10310	[[0,0,0,0,1,0,0,0,0],[0,0,0,0,1,0,0,0,0],[0,0,0,0,1,0,0,0,0]]10311	10312,10313	[[0,0,0,0,1,0,0,0,0],[0,0,0,0,1,0,0,0,0]]10314	10315,10316	[[0,0,0,0,1,0,0,0,0],[0,0,0,0,1,0,0,0,0]]10317	636,2803,635,10318,10319,4014,5311,8283,5992,634,10320,9610,660,1961,9607,10321,656,4400,10322,10323,633,10324,10325,9605,125,640,10298,10326,664,10327,10299,10328,661,642,10329,10330,10331,4160,10332,4069,10333,4401,10334,4395,4146,9141,649,10335,10336,10337,4154,4156,10338,4141,4171,668,10339,4191,646,10340,647,4023,10341,10342,655,1402,10343,10344,10345,4188,9614,10297,632,650,10346,10347,10348,10349	[[0,0,1,0,0,0,0,0,0],[0,0,1,0,0,0,0,0,0],[0,0,1,0,0,0,0,0,0],[0,0,1,0,0,0,0,0,0],[0,0,1,0,0,0,0,0,0],[0,0,1,0,0,0,0,0,0],[0,0,1,0,0,0,0,0,0],[0,0,1,0,0,0,0,0,0],[0,0,1,0,0,0,0,0,0],[0,0,1,0,0,0,0,0,0],[0,1,0,0,0,0,0,0,0],[0,1,0,0,0,0,0,0,0],[0,0,1,0,0,0,0,0,0],[0,0,1,0,0,0,0,0,0],[0,1,1,0,0,0,0,0,0],[0,0,1,0,0,0,0,0,0],[0,1,1,0,0,0,0,0,0],[0,0,0,0,1,0,0,0,0],[0,0,1,0,0,0,0,0,0],[0,0,1,0,0,0,0,0,0],[0,0,1,0,0,0,0,0,0],[0,0,1,0,0,0,0,0,0],[0,0,1,0,0,0,0,0,0],[0,0,1,0,0,0,0,0,0],[0,0,1,0,0,0,0,0,0],[0,0,1,0,0,0,0,0,0],[0,0,1,1,0,0,0,0,0],[0,0,1,0,0,0,0,0,0],[0,0,1,0,0,0,0,0,0],[0,1,1,0,0,0,0,0,0],[0,0,1,1,0,0,0,0,0],[0,0,1,0,0,0,0,0,0],[0,1,1,0,0,0,0,0,0],[0,0,1,0,0,0,0,0,0],[0,0,1,0,0,0,0,0,0],[0,0,1,0,0,0,0,0,0],[0,0,1,0,0,0,0,0,0],[0,0,1,0,0,0,0,0,0],[0,0,1,0,0,0,0,0,0],[0,0,1,0,0,0,0,0,0],[0,0,1,0,0,0,0,0,0],[0,0,0,0,1,0,0,0,0],[0,1,0,0,0,0,0,0,0],[0,0,0,0,1,0,0,0,0],[0,0,1,0,0,0,0,0,0],[0,0,1,0,0,0,0,0,0],[0,0,1,0,0,0,0,0,0],[0,0,1,0,0,0,0,0,0],[0,0,1,0,0,0,0,0,0],[0,0,1,0,0,0,0,0,0],[0,0,1,0,0,0,0,0,0],[0,0,1,0,0,0,0,0,0],[0,0,1,0,0,0,0,0,0],[0,0,1,0,0,0,0,0,0],[0,1,1,0,0,0,0,0,0],[0,0,1,0,0,0,0,0,0],[0,0,1,0,0,0,0,0,0],[0,0,1,0,0,0,0,0,0],[0,1,1,0,0,0,0,0,0],[0,1,1,0,0,0,0,0,0],[0,0,1,0,0,0,0,0,0],[0,0,0,0,1,0,0,0,0],[0,0,1,0,0,0,0,0,0],[0,0,1,0,0,0,0,0,0],[0,1,1,0,0,0,0,0,0],[0,0,1,0,0,0,0,0,0],[0,1,0,0,0,0,0,0,0],[0,1,0,0,0,0,0,0,0],[0,0,1,0,0,0,0,0,0],[0,0,1,0,0,0,0,0,0],[0,1,1,0,0,0,0,0,0],[0,0,1,1,0,0,0,0,0],[0,0,1,0,0,0,0,0,0],[0,0,1,0,0,0,0,0,0],[0,0,1,0,0,0,0,0,0],[0,0,1,0,0,0,0,0,0],[0,0,1,0,0,0,0,0,0],[0,0,1,0,0,0,0,0,0]]10350	10351,5633,10352,5621,10353,5628,10354,4794,10355,5623,10356,4801,4795,10357,4796,3996,3734,10358,10359,4798,1539,10360,10361,4797,10362,10363,5638,7958,3664,4792,2343,3722,10364,10365,3753,10366,7701,10367,5643,10368,10369,10370,8668,10371,10372,905,10373,6112,10374,10375,8108,10376,10377,10378,10379,6351,10380,7680,10381,10382,5657	[[0,0,0,1,0,0,0,0,1],[0,1,1,0,0,0,0,0,1],[0,0,0,0,0,0,0,0,1],[0,1,1,0,0,0,0,0,1],[0,1,1,0,0,0,0,1,1],[0,0,0,1,1,0,0,0,0],[0,0,1,1,0,0,0,0,0],[0,1,1,0,1,0,1,1,1],[0,0,1,1,1,0,0,0,1],[0,0,0,1,0,0,0,0,0],[0,0,0,0,0,0,0,0,1],[0,1,1,1,0,0,1,0,1],[0,1,1,1,0,0,1,1,1],[0,0,0,0,0,0,0,0,1],[0,1,1,0,0,0,1,1,1],[0,0,0,0,0,0,0,0,1],[0,1,0,0,0,0,0,0,0],[0,1,1,0,0,0,0,0,0],[0,1,1,0,0,0,0,0,0],[0,1,1,0,0,0,0,1,1],[0,1,0,0,0,0,0,0,0],[0,1,1,0,0,0,1,0,1],[0,1,1,0,0,0,1,0,1],[0,1,1,0,0,0,1,1,1],[0,1,1,0,0,0,0,0,0],[0,1,0,0,0,0,0,0,0],[0,1,1,0,1,0,0,0,0],[0,0,0,1,0,0,0,1,1],[0,1,1,0,0,0,0,1,1],[0,1,1,0,0,0,0,1,1],[0,1,1,0,0,0,1,0,1],[0,1,0,0,0,0,0,0,0],[0,1,1,1,0,0,1,0,1],[0,1,0,0,0,0,0,0,0],[0,1,1,0,0,0,1,0,0],[0,1,1,0,0,0,0,0,0],[0,0,0,0,0,0,0,0,1],[0,0,0,0,0,0,0,0,1],[0,0,0,0,1,0,0,0,0],[0,0,1,0,0,0,0,0,0],[0,0,1,0,0,0,1,0,0],[0,0,1,0,0,0,0,0,0],[0,0,0,0,0,0,0,0,1],[0,0,1,0,0,0,0,0,0],[0,0,0,0,0,0,0,1,1],[0,0,0,0,0,0,0,0,1],[0,0,1,0,0,0,0,0,0],[0,0,1,0,0,0,0,0,0],[0,0,0,0,0,0,0,0,1],[0,0,0,0,0,0,0,0,1],[0,0,0,0,1,0,0,0,0],[0,0,1,0,0,0,0,0,0],[0,0,0,0,0,0,0,0,1],[0,0,1,0,0,0,0,1,1],[0,0,0,0,0,0,0,0,1],[0,0,0,0,0,0,0,0,1],[0,0,1,0,0,0,0,0,0],[0,0,1,0,0,0,0,1,0],[0,0,0,0,0,0,0,0,1],[0,0,1,0,0,0,0,0,0],[0,0,1,0,0,0,0,0,0]]10383	10384,4756,6304	[[0,0,0,1,1,0,0,0,0],[0,1,0,1,1,0,0,0,0],[0,1,0,1,0,0,0,0,0]]10385	10386,10387	[[0,0,0,0,1,0,0,0,0],[0,0,0,0,1,0,0,0,0]]10388	10389,10390,10391,10392,10393,10394,2428,314,4261,7345,9430,7338,10395,7339,10396,10397,10398,7340,7330,10399,10400,7336,10401,2588,309,8006,7951,10402,10403	[[0,0,1,0,0,0,0,1,0],[0,0,1,1,1,0,0,1,0],[0,1,1,0,0,0,0,0,0],[0,0,1,0,0,0,0,1,0],[0,0,1,0,0,0,0,0,0],[0,0,0,0,1,0,0,0,0],[0,0,0,0,0,0,0,1,0],[0,1,1,1,0,0,0,1,0],[0,0,1,0,0,0,0,0,0],[0,1,1,0,0,0,0,0,0],[0,1,1,0,0,0,0,1,0],[0,1,1,0,0,0,0,0,0],[0,0,1,0,0,0,0,0,0],[0,1,1,0,0,0,0,0,0],[0,1,1,1,1,0,0,1,0],[0,0,0,0,0,0,0,1,0],[0,1,0,0,0,0,0,0,0],[0,1,1,0,0,0,0,1,0],[0,1,1,0,0,0,0,0,0],[0,0,1,0,0,0,0,0,0],[0,1,1,0,0,0,0,1,0],[0,1,1,1,0,0,0,1,0],[0,1,0,0,0,0,0,0,0],[0,1,0,0,0,0,0,0,0],[0,1,1,0,0,0,0,1,0],[0,0,0,0,0,0,0,1,0],[0,0,1,0,0,0,0,0,0],[0,0,1,0,0,0,0,0,0],[0,0,1,0,1,0,0,1,0]]10404	10405,10406,10407,10408,3630,1589,10409,7582,5852,10410,10411,10412,10413,8327,10414	[[0,1,0,0,0,0,0,0,0],[0,1,0,0,0,0,0,0,0],[0,1,0,0,0,0,0,0,0],[0,1,0,0,0,0,0,0,0],[0,1,0,0,0,0,0,0,0],[0,1,0,0,1,0,0,0,0],[0,1,0,0,1,0,0,0,0],[0,1,0,0,0,0,0,0,0],[0,1,0,0,0,0,0,0,0],[0,1,0,0,0,0,0,0,0],[0,0,0,0,1,0,0,0,0],[0,1,0,0,0,0,0,0,0],[0,1,0,0,0,0,0,0,0],[0,1,0,0,0,0,0,0,0],[0,1,0,0,0,0,0,0,0]]10415	10416,9743,10417,3748,2941,10418,9754,7130,10419,10420,8939,3741,3782,5003,10421,10422,7025,10423,7057,8929,3676,10424,7124,7926,3417,3132,10425,2137,5164,1576,7061,1802,10426,10427,10428,3654,3778,10429,5163,7120,6241,10430,3774,10431,3121,4867,6622,3142,3682	[[1,0,0,0,0,0,0,0,0],[1,0,1,0,0,0,0,0,0],[0,1,0,0,0,0,0,1,0],[1,0,1,0,0,0,0,0,0],[0,0,1,0,0,0,0,0,0],[1,0,0,0,0,0,0,0,0],[1,0,0,0,0,0,0,0,0],[0,0,1,0,0,0,0,0,0],[1,0,0,0,0,0,0,0,0],[1,0,0,0,1,0,0,0,0],[1,0,0,0,0,0,0,0,0],[1,0,0,0,0,0,0,0,0],[1,0,0,0,0,0,0,0,0],[1,0,0,0,0,0,0,0,0],[0,0,0,0,0,0,0,1,0],[0,0,1,0,0,0,0,0,0],[0,0,1,0,0,0,0,0,0],[1,1,0,1,1,0,0,1,0],[1,0,0,0,0,0,0,1,0],[1,1,0,0,1,0,0,1,0],[0,1,0,0,0,0,0,1,0],[1,1,0,1,0,0,0,1,0],[0,0,0,0,0,0,0,1,0],[0,0,0,0,0,0,0,1,0],[0,0,0,0,0,0,0,1,0],[0,0,0,0,0,0,0,1,0],[0,0,1,0,0,0,0,0,0],[0,0,1,0,0,0,0,0,0],[0,0,1,1,0,0,0,1,0],[1,0,0,0,0,0,0,1,0],[0,0,0,0,0,0,0,1,0],[1,0,0,0,1,0,0,0,0],[0,0,0,0,0,0,0,1,0],[1,0,0,0,0,0,0,0,0],[1,0,0,0,0,0,0,1,0],[1,0,0,0,0,0,0,0,0],[0,0,0,0,1,0,0,0,0],[1,1,0,0,0,0,0,0,0],[0,0,1,0,0,0,0,0,0],[0,1,0,0,0,0,0,0,0],[0,1,0,0,0,0,0,0,0],[1,0,0,0,0,0,0,0,0],[0,0,0,0,1,0,0,0,0],[0,0,0,0,1,0,0,0,0],[1,0,0,0,0,0,0,0,0],[1,0,0,0,0,0,0,0,0],[1,0,0,0,0,0,0,0,0],[1,0,0,0,0,0,0,0,0],[1,0,0,0,0,0,0,0,0]]10432	10433,10434,10435,10436,10437,10438,10439,10440,10441,10442,10443	[[0,0,1,0,0,0,0,0,0],[0,0,1,1,1,0,0,0,1],[0,0,1,0,0,0,0,0,1],[0,0,0,0,1,0,0,0,1],[0,0,1,1,1,0,0,0,1],[0,0,0,0,1,0,0,0,0],[0,0,1,0,1,0,0,0,0],[0,0,0,1,0,0,0,0,0],[0,0,1,0,0,0,0,0,1],[0,0,0,0,1,0,0,0,0],[0,0,0,0,0,0,0,0,1]]10444	10445,10289,10446,10447,10448,3620,10449,10450,3534,10451,10452,3873,10453	[[0,0,0,1,0,0,0,0,0],[0,0,0,1,0,0,0,0,0],[0,0,0,1,0,0,0,0,0],[0,0,0,1,0,0,0,0,0],[0,0,0,1,0,0,0,0,0],[0,0,0,1,0,0,0,0,0],[0,0,0,1,0,0,0,0,0],[0,0,0,1,0,0,0,0,0],[0,0,0,1,0,0,0,0,0],[0,0,0,1,0,0,0,0,0],[0,0,0,1,0,0,0,0,0],[0,0,0,1,0,0,0,0,0],[0,0,0,1,0,0,0,0,0]]10454	10455,3561,10456,10457,10458,8400,3563,6239,4636,10459,425,10460,4490,10461,9635,3562,10462,3575,3568,10463,10464,3560,10465,3558,10466,231,9636,9572,10467,10468,10469,10470,4493,10471,10472,10473,10474,1354,10475,4627,863,10476,10477,4177,10478,10479,5256,1585,4614,4172,10480,10481,3566,10482,10483,10484,1491,10485,10486,3569,3567,2687,10487,10488,4703,1485,5243,3576,3573,3559,10489,5289,10490,10491,10492,1962,10493,4700,4699,10494,4365,10495,2053,3578,10496,10497,9334,10498,4680,10499,10500,10501,10502,10503	[[0,1,1,1,0,0,1,1,1],[0,1,1,0,0,0,1,0,1],[0,0,1,0,0,0,0,0,0],[0,0,0,0,0,0,0,1,0],[0,0,1,0,0,0,0,0,0],[0,1,1,0,0,0,0,0,0],[0,0,1,0,0,0,0,0,0],[0,0,1,0,0,0,0,0,0],[0,0,1,0,0,0,0,0,0],[0,0,1,0,0,0,0,0,0],[0,0,1,0,0,0,0,0,0],[0,0,1,0,0,0,0,0,0],[0,0,1,0,0,0,0,0,0],[0,0,1,0,0,0,0,0,0],[0,0,1,0,0,0,0,0,0],[0,0,1,1,0,0,0,1,1],[0,0,1,0,0,0,0,0,1],[0,0,1,1,0,0,0,0,1],[0,1,1,1,0,0,1,1,1],[0,1,0,0,0,0,0,0,0],[0,1,0,0,0,0,0,0,0],[0,1,1,0,0,0,1,1,1],[0,1,1,0,0,0,0,0,0],[0,1,1,0,0,0,0,0,0],[0,1,0,0,0,0,0,0,0],[0,1,0,0,0,0,0,0,0],[0,1,0,0,1,0,1,1,0],[0,1,0,0,0,0,0,0,0],[0,0,1,0,0,0,0,0,0],[0,0,1,0,0,0,0,0,0],[0,0,1,0,0,0,0,0,0],[0,0,1,0,0,0,0,1,0],[0,1,1,0,0,0,1,0,0],[0,0,0,0,0,0,0,0,1],[0,1,1,1,0,0,1,1,1],[0,1,0,0,1,0,1,1,0],[0,0,1,0,0,0,0,0,0],[0,0,1,0,0,0,0,0,0],[0,0,1,0,0,0,0,0,0],[0,1,1,0,0,0,1,1,1],[0,0,0,0,0,0,0,0,1],[0,0,1,0,0,0,0,0,0],[0,0,1,0,0,0,0,0,0],[0,0,1,0,0,0,0,0,0],[0,0,0,1,0,0,0,0,0],[0,1,0,0,0,0,1,0,1],[0,0,1,1,0,0,0,1,0],[0,1,1,0,0,0,1,0,1],[0,0,0,0,0,0,0,1,0],[0,0,1,0,0,0,0,0,0],[0,1,0,0,0,0,1,0,0],[0,0,1,0,0,0,0,0,0],[0,0,1,0,0,0,0,0,1],[0,0,1,0,0,0,0,0,0],[0,0,0,0,0,0,0,0,1],[0,0,0,1,0,0,0,0,1],[0,0,0,0,0,0,0,1,0],[0,0,1,0,0,0,0,1,0],[0,0,1,0,0,0,0,0,0],[0,0,1,0,0,0,0,0,1],[0,1,1,0,0,0,0,1,1],[0,0,1,0,0,0,0,0,0],[0,1,0,0,1,0,1,1,0],[0,0,0,0,1,0,0,0,0],[0,1,0,0,0,0,1,0,0],[0,0,1,0,0,0,0,0,0],[0,0,1,0,0,0,0,0,0],[0,0,1,0,0,0,0,0,1],[0,0,1,0,0,0,0,0,1],[0,0,1,0,0,0,0,0,0],[0,0,0,0,0,0,0,1,0],[0,0,1,0,0,0,0,0,0],[0,0,1,0,0,0,1,0,0],[0,0,1,0,0,0,0,0,0],[0,0,1,0,0,0,0,0,0],[0,0,0,0,0,0,0,1,0],[0,1,1,0,0,0,1,0,0],[0,1,0,0,0,0,0,1,0],[0,0,0,0,0,0,0,1,0],[0,0,1,0,0,0,0,0,0],[0,1,0,0,0,0,0,0,0],[0,1,0,0,0,0,0,0,0],[0,0,0,0,0,0,0,1,0],[0,0,1,0,0,0,0,0,0],[0,0,1,0,0,0,0,0,0],[0,0,1,0,0,0,0,0,0],[0,0,1,1,0,0,0,0,0],[0,0,1,0,0,0,0,0,0],[0,0,1,0,0,0,0,0,0],[0,0,0,0,0,0,0,1,0],[0,0,1,0,0,0,0,0,0],[0,0,1,0,0,0,0,0,0],[0,0,1,0,0,0,0,0,0],[0,0,1,0,0,0,0,0,0]]10504	10505	[[0,0,0,0,1,0,0,0,0]]10506	10507,10508,10509,10510,10511,10512,10513,10514,10515,1813,1798,4247,10516,10517,202,10518,10519,546,10520,4831,1799,10521,10522,10523,1851,10524,10525,10526,1796,10527,10528,10529,6433,1841,4215,10530,1837,1816,10531,10532,10533,10534,10535,10536	[[0,0,1,0,1,0,0,0,0],[0,0,1,0,0,0,0,0,0],[0,0,0,0,1,0,0,0,0],[0,0,1,0,0,0,0,0,0],[0,0,0,0,1,0,0,0,0],[0,0,1,0,1,0,0,0,0],[0,0,1,0,1,0,0,0,0],[0,0,1,0,0,0,0,0,0],[0,0,1,0,1,0,0,0,0],[0,0,1,0,0,0,0,0,0],[0,0,1,0,0,0,0,0,0],[0,0,0,1,0,0,0,0,0],[0,0,1,0,1,0,0,0,0],[0,0,1,0,0,0,0,0,0],[0,0,1,0,1,0,0,0,0],[0,0,1,0,0,0,0,0,0],[0,0,1,0,1,0,0,0,0],[0,0,1,0,0,0,0,0,0],[0,0,0,1,0,0,0,0,0],[0,0,1,0,0,0,0,0,0],[0,0,1,0,0,0,0,0,0],[0,0,1,0,0,0,0,0,0],[0,0,0,0,1,0,0,0,0],[0,0,1,0,0,0,0,0,0],[0,0,1,0,0,0,0,0,0],[0,0,1,0,1,0,0,0,0],[0,0,1,0,1,0,0,0,0],[0,0,0,0,1,0,0,0,0],[0,0,1,0,0,0,0,0,0],[0,0,1,1,0,0,0,0,0],[0,0,1,0,1,0,0,0,0],[0,0,1,0,0,0,0,0,0],[0,0,1,0,1,0,0,0,0],[0,0,1,0,0,0,0,0,0],[0,0,0,1,0,0,0,0,0],[0,0,0,0,1,0,0,0,0],[0,0,1,0,0,0,0,0,0],[0,0,1,0,0,0,0,0,0],[0,0,1,0,1,0,0,0,0],[0,0,0,1,0,0,0,0,0],[0,0,1,0,0,0,0,0,0],[0,0,0,0,1,0,0,0,0],[0,0,1,0,0,0,0,0,0],[0,0,1,0,0,0,0,0,0]]10537	6014,1067,8282,10538,8300,8223,10539,429,42,8288,10540,6533,10541,10542,9568,1649,1044,8306,10543,8291,1676,5982,9571,6527,10544,10292,1091,10545,1653,1053,8304,4069,8222,10546,1072,1977,9570,2037,10547,3797,6462,10548,10549,438,426,8292,10550,10551,2753,8297,10552,10553,1710,1408,1069,10554,10555,1699,1070,435,434,3582,8299,8289,3870,8287,1086,8285,8302,8281,8290,640,2253,10556,1650,1682,10557,1099,1683,10558,8296,1945,1647,8294,10559,1665,1646,437,9499,6497,1060,10560,8298,10561,8293,10562,10563,1689,658,10564,10565	[[0,1,1,0,0,0,0,1,0],[0,0,1,0,0,0,0,0,0],[0,1,1,0,0,0,0,0,0],[0,0,1,0,0,0,0,0,0],[0,0,1,0,0,0,0,0,1],[0,1,1,0,0,0,0,1,1],[0,0,1,0,1,0,0,0,0],[0,0,1,0,0,0,0,1,0],[0,0,0,0,0,0,0,0,1],[0,1,1,0,0,0,0,1,1],[0,0,1,0,0,0,0,0,0],[0,0,1,0,0,0,0,1,0],[0,0,0,0,0,0,0,1,0],[0,0,0,0,0,0,0,1,0],[0,0,1,0,0,0,0,0,0],[0,0,1,0,0,0,0,1,1],[0,0,1,0,0,0,0,0,0],[0,0,1,0,0,0,0,0,0],[0,0,0,0,0,0,0,0,1],[0,1,1,0,0,1,0,1,1],[0,1,0,0,0,0,0,1,0],[0,1,0,0,0,0,0,1,1],[0,0,1,0,0,0,0,0,0],[0,0,1,0,0,0,0,0,0],[0,0,1,0,0,0,0,0,0],[0,0,0,0,0,0,0,0,1],[0,0,1,0,0,0,0,0,0],[0,0,0,0,1,0,0,0,0],[0,0,0,0,0,0,0,0,1],[0,0,0,0,0,0,0,0,1],[0,1,1,1,1,1,0,1,1],[0,0,1,1,0,0,0,0,0],[0,1,1,0,0,1,0,1,1],[0,1,1,0,0,0,0,1,1],[0,0,1,0,0,0,0,0,0],[0,0,1,0,0,0,0,0,0],[0,0,1,0,0,0,0,0,0],[0,0,1,0,0,0,0,0,0],[0,0,0,0,1,0,0,0,1],[0,0,1,0,0,0,0,0,0],[0,0,1,0,0,1,0,0,1],[0,0,1,0,0,0,0,0,0],[0,0,1,0,0,0,0,0,0],[0,0,0,1,0,0,0,1,1],[0,0,1,0,0,0,0,1,0],[0,1,1,0,0,1,0,1,1],[0,0,1,0,1,0,0,1,0],[0,0,1,0,0,0,0,0,0],[0,0,1,0,0,0,0,0,0],[0,1,1,1,0,1,0,1,1],[0,1,1,0,0,0,0,0,0],[0,1,1,0,0,0,0,0,0],[0,0,1,0,0,0,0,1,0],[0,0,0,0,0,0,0,0,1],[0,0,1,0,0,0,0,0,0],[0,0,1,0,0,0,0,0,0],[0,0,0,0,0,1,0,0,1],[0,0,1,0,0,0,0,0,0],[0,0,1,0,0,0,0,0,0],[0,1,0,0,0,0,0,1,0],[0,0,1,0,0,0,0,0,0],[0,0,1,0,0,1,0,0,1],[0,0,1,0,0,0,0,0,0],[0,1,1,0,0,0,0,0,0],[0,1,1,0,0,0,0,1,0],[0,1,1,0,0,0,0,0,1],[0,1,0,0,0,0,0,1,0],[0,1,0,0,0,0,0,1,0],[0,1,1,0,0,0,0,0,0],[0,1,1,0,0,1,0,1,1],[0,1,0,0,0,0,0,0,0],[0,1,1,0,0,1,0,1,1],[0,1,1,0,0,1,0,0,1],[0,0,1,0,0,0,0,0,0],[0,1,1,0,0,1,0,1,1],[0,1,1,0,0,0,0,1,1],[0,0,1,0,0,0,0,0,0],[0,0,1,0,0,0,0,0,0],[0,0,0,0,0,0,0,0,1],[0,0,0,0,0,0,0,0,1],[0,0,1,0,0,0,0,0,0],[0,0,0,0,0,0,0,1,0],[0,0,1,0,0,0,0,0,0],[0,1,1,0,0,0,0,0,0],[0,0,1,0,0,0,0,0,0],[0,0,1,0,0,0,0,0,0],[0,1,1,0,0,0,0,0,0],[0,0,1,0,0,0,0,0,0],[0,0,1,0,0,0,0,0,0],[0,0,1,0,0,0,0,0,0],[0,0,0,0,0,0,0,0,1],[0,1,0,0,0,0,0,0,0],[0,1,1,0,0,0,0,0,1],[0,0,1,0,1,0,0,1,0],[0,1,0,0,0,0,0,0,0],[0,0,1,0,0,0,0,0,0],[0,0,1,0,0,0,0,0,0],[0,0,1,0,0,0,0,1,0],[0,0,1,0,0,0,0,0,0],[0,0,1,0,0,0,0,0,0],[0,0,0,0,0,0,0,0,1]]10566	10567	[[0,0,0,0,1,0,0,0,0]]10568	10569,10570,10571,64,10572,7994,5533,1397,10573,8292,9199,10574,10575,5536,10576,1886,10577,2253,10578,10579,10580,10581,10582,10583,10584,10585,1069,10586,10587,10588,6002,1749	[[0,0,0,0,1,0,0,0,0],[0,0,1,0,0,0,0,0,0],[0,0,1,0,0,0,0,0,0],[0,0,0,0,1,0,0,0,0],[0,0,1,0,0,0,0,0,0],[0,0,1,0,0,0,0,0,0],[0,0,1,0,1,0,0,0,0],[0,0,1,0,0,0,0,0,0],[0,0,1,0,1,0,0,0,0],[0,0,1,0,0,0,0,0,0],[0,0,1,0,0,0,0,0,0],[0,0,0,0,1,0,0,0,0],[0,0,0,0,1,0,0,0,0],[0,0,1,0,0,0,0,0,0],[0,0,1,0,0,0,0,0,0],[0,0,1,0,0,0,0,0,0],[0,0,1,0,0,0,0,0,0],[0,0,1,0,0,0,0,0,0],[0,0,1,0,0,0,0,0,0],[0,0,1,0,0,0,0,0,0],[0,0,0,0,1,0,0,0,0],[0,0,1,0,0,0,0,0,0],[0,0,1,0,0,0,0,0,0],[0,0,1,0,0,0,0,0,0],[0,0,1,0,0,0,0,0,0],[0,0,1,0,0,0,0,0,0],[0,0,1,0,0,0,0,0,0],[0,0,0,0,1,0,0,0,0],[0,0,1,0,0,0,0,0,0],[0,0,1,0,0,0,0,0,0],[0,0,1,0,0,0,0,0,0],[0,0,1,0,0,0,0,0,0]]10589	10590	[[0,0,0,0,1,0,0,0,0]]10591	10446,10592,10449,10448,9989,10451,10593,10594,10595,10596,4471,3534,3620,10447,10452,10289,10445,10597,10453,10598,3873,10450	[[0,1,1,1,0,0,0,1,0],[0,0,1,0,0,0,0,0,0],[0,1,1,1,0,0,0,1,0],[0,1,0,1,0,0,0,0,0],[0,1,1,0,0,0,0,1,0],[0,1,1,1,0,0,0,1,0],[0,1,1,0,0,0,0,1,0],[0,1,0,0,0,0,0,0,0],[0,1,0,0,1,0,0,0,0],[0,1,1,0,0,0,0,0,0],[0,1,0,0,0,0,0,1,0],[0,1,1,1,0,0,0,1,0],[0,1,0,1,0,0,0,0,0],[0,0,0,1,0,0,0,0,0],[0,0,0,1,0,0,0,1,0],[0,1,1,1,0,0,0,0,0],[0,0,1,1,0,0,0,1,0],[0,0,1,0,0,0,0,0,0],[0,0,0,1,0,0,0,1,0],[0,1,1,0,0,0,0,0,0],[0,1,0,1,0,0,0,0,0],[0,0,1,1,0,0,0,1,0]]10599	5177,10600,10601,10602,2948,8814,7337	[[0,0,0,0,0,0,1,0,0],[0,0,0,0,0,0,1,0,0],[0,0,0,0,0,0,1,0,0],[0,0,0,0,0,0,1,0,0],[0,0,0,0,0,0,1,0,0],[0,0,0,0,0,0,1,0,0],[0,0,0,0,0,0,1,0,0]]10603	10604,10605,10606,10420,9743,10607,10416,10430	[[0,1,1,0,0,0,0,0,0],[0,0,1,0,0,0,0,0,0],[0,0,1,0,1,0,0,0,0],[0,1,0,0,0,0,0,0,0],[0,1,0,0,0,0,0,0,0],[0,0,0,0,1,0,0,0,0],[0,1,0,0,0,0,0,0,0],[0,0,0,0,1,0,0,0,0]]10608	10609	[[0,0,0,0,1,0,0,0,0]]10610	10611	[[0,0,0,0,1,0,0,0,0]]10612	2118,10613,1437,10614,4156,10615,10616,10617,10618,10619,4160,10620,10621,1436,10622,10623,3429,10624,10625,5541,3121,10626,10627,10628,10629,10630,10631,10632,10633,10634,10635,10322,1626,10636,10637,2119,1718,10638,10639,4154,10640,1556,1625,4141,10641,10642,10643,1461,10644,1432,5973,10645,10646	[[0,0,1,1,0,0,0,0,0],[0,0,1,1,0,0,0,1,0],[0,0,0,1,0,0,0,0,0],[0,0,0,1,0,0,0,1,0],[0,0,1,0,0,0,0,0,0],[0,0,0,0,1,0,0,1,0],[0,0,1,0,0,0,0,0,0],[0,0,0,0,0,0,0,1,0],[0,0,1,1,1,0,0,1,0],[0,0,1,1,0,0,0,1,0],[0,0,1,0,0,0,0,0,0],[0,0,1,0,0,0,0,0,0],[0,0,1,1,0,0,0,0,0],[0,0,1,1,0,0,0,1,0],[0,0,1,0,0,0,0,0,0],[0,0,1,0,0,0,0,0,0],[0,0,0,0,0,0,0,1,0],[0,0,1,0,1,0,0,0,0],[0,0,1,0,0,0,0,0,0],[0,0,1,0,0,0,0,0,0],[0,0,0,1,0,0,0,0,0],[0,0,1,0,0,0,0,0,0],[0,0,0,0,0,0,0,1,0],[0,0,1,1,0,0,0,1,0],[0,0,1,1,0,0,0,0,0],[0,0,0,0,0,0,0,1,0],[0,0,0,1,0,0,0,0,0],[0,0,1,0,0,0,0,0,0],[0,0,1,1,0,0,0,1,0],[0,0,1,0,0,0,0,0,0],[0,0,1,0,0,0,0,0,0],[0,0,1,0,0,0,0,0,0],[0,0,1,0,0,0,0,0,0],[0,0,1,0,0,0,0,0,0],[0,0,0,0,0,0,0,1,0],[0,0,1,1,0,0,0,1,0],[0,0,1,0,0,0,0,0,0],[0,0,0,0,0,0,0,1,0],[0,0,1,1,0,0,0,0,0],[0,0,0,0,0,0,0,1,0],[0,0,1,0,0,0,0,1,0],[0,0,0,0,0,0,0,1,0],[0,0,1,0,0,0,0,0,0],[0,0,1,0,0,0,0,0,0],[0,0,1,1,0,0,0,1,0],[0,0,0,0,0,0,0,1,0],[0,0,1,0,0,0,0,0,0],[0,0,1,0,1,0,0,1,0],[0,0,1,0,0,0,0,0,0],[0,0,1,0,0,0,0,0,0],[0,0,1,0,0,0,0,0,0],[0,0,1,1,0,0,0,1,0],[0,0,0,0,1,0,0,0,0]]10647	10648,10649,10650,10651,10652,10653,10654,10655,10656,2810,10657,10658	[[0,0,0,0,1,0,0,0,0],[0,0,0,0,1,0,0,0,0],[0,0,0,0,1,0,0,0,0],[0,0,0,0,1,0,0,0,0],[0,0,0,0,1,0,0,0,0],[0,0,0,0,1,0,0,0,0],[0,0,0,0,1,0,0,0,0],[0,0,0,0,1,0,0,0,0],[0,0,0,0,1,0,0,0,0],[0,0,0,0,1,0,0,0,0],[0,0,0,0,1,0,0,0,0],[0,0,0,0,1,0,0,0,0]]10659	10660	[[0,0,0,0,1,0,0,0,0]]10661	10662	[[0,0,0,0,1,0,0,0,0]]10663	8161,5037,1611,10664,2912,10665,10384,10666,2521,6304,9728,810,10667,6443,10668,10669,10670,10671,10672,10673,4567,8754,4756,10674,10675,10676,10677,10678,6180,10679,10680,10681,10682,1170,10683,10684,10685,10686,10687,10688,10689,10690,10691,10692,10693,10694,10695	[[0,1,1,0,0,0,0,0,0],[0,0,1,0,0,0,0,0,0],[0,1,0,1,0,0,0,0,0],[0,1,1,1,0,0,0,0,0],[0,1,1,0,0,0,0,0,0],[0,1,1,1,1,0,0,0,0],[0,1,1,0,0,0,0,0,0],[0,0,1,0,0,0,0,0,0],[0,0,1,0,0,0,0,0,0],[0,0,1,0,0,0,0,0,0],[0,0,1,0,0,0,0,0,0],[0,0,1,0,0,0,0,0,0],[0,1,1,0,1,0,0,0,0],[0,1,1,1,0,0,0,0,0],[0,1,0,0,0,0,0,0,0],[0,0,1,0,0,0,0,0,0],[0,1,1,0,0,0,0,0,0],[0,0,1,0,0,0,0,0,0],[0,0,1,0,0,0,0,0,0],[0,0,1,0,0,0,0,0,0],[0,0,1,0,0,0,0,0,0],[0,0,1,0,0,0,0,0,0],[0,0,1,0,0,0,0,0,0],[0,0,1,0,0,0,0,0,0],[0,0,1,0,0,0,0,0,0],[0,0,0,0,1,0,0,0,0],[0,0,1,0,0,0,0,0,0],[0,0,0,1,0,0,0,0,0],[0,1,1,0,0,0,0,0,0],[0,0,1,1,0,0,0,0,0],[0,0,0,0,1,0,0,0,0],[0,0,1,0,0,0,0,0,0],[0,0,1,0,0,0,0,0,0],[0,0,1,0,0,0,0,0,0],[0,0,1,0,0,0,0,0,0],[0,0,1,0,0,0,0,0,0],[0,0,1,0,0,0,0,0,0],[0,0,1,0,0,0,0,0,0],[0,0,1,0,1,0,0,0,0],[0,0,1,0,0,0,0,0,0],[0,0,1,0,0,0,0,0,0],[0,0,1,0,0,0,0,0,0],[0,0,1,0,0,0,0,0,0],[0,0,1,0,0,0,0,0,0],[0,0,1,0,0,0,0,0,0],[0,0,1,0,0,0,0,0,0],[0,0,1,0,0,0,0,0,0]]10696	10697	[[0,0,0,0,1,0,0,0,0]]10698	10699,10700	[[0,0,0,0,1,0,0,0,0],[0,0,0,0,1,0,0,0,0]]10701	10702,10703,10704	[[0,0,0,0,1,0,0,0,0],[0,0,0,0,1,0,0,0,0],[0,0,0,0,1,0,0,0,0]]10705	10706,10707,10708,10709,4530,10710,4519,48,10711,10712,10713,4524,4518,10714,40,10715,10716,4520,4517,4522,10717,10718,4523	[[0,1,0,1,1,0,1,0,0],[0,1,0,0,1,0,1,0,0],[0,1,0,1,0,0,1,0,0],[0,1,0,1,0,0,1,0,0],[0,1,0,0,0,0,1,0,0],[0,1,0,0,0,0,1,0,0],[0,1,0,0,0,0,1,0,0],[0,1,0,0,0,0,1,0,0],[0,1,0,0,0,0,0,0,0],[0,1,0,0,0,0,0,0,0],[0,1,0,0,0,0,0,0,0],[0,1,0,0,0,0,1,0,0],[0,1,0,0,0,0,1,0,0],[0,0,0,0,1,0,0,0,0],[0,1,0,0,0,0,1,0,0],[0,1,0,0,0,0,0,0,0],[0,1,0,0,1,0,1,0,0],[0,1,0,0,0,0,1,0,0],[0,1,0,0,0,0,1,0,0],[0,1,0,0,0,0,1,0,0],[0,1,0,0,0,0,1,0,0],[0,0,0,0,1,0,0,0,0],[0,1,0,0,0,0,1,0,0]]10719	10720,10721,3580,10722,3582,10723,10724	[[0,0,1,0,0,0,0,0,0],[0,0,1,0,0,0,0,0,0],[0,0,1,0,0,0,0,0,0],[0,0,1,0,0,0,0,0,0],[0,0,1,0,0,0,0,0,0],[0,0,1,0,0,0,0,0,0],[0,0,1,0,0,0,0,0,0]]10725	10726,10727	[[0,0,0,0,1,0,0,0,0],[0,0,0,0,1,0,0,0,0]]10728	10729	[[0,0,0,0,1,0,0,0,0]]10730	10731,10732,2114	[[0,0,0,0,1,0,0,0,0],[0,0,0,0,1,0,0,0,0],[0,0,0,0,1,0,0,0,0]]10733	10734,8826,10735,8816,10736,10737,10738,10739,8808,10740,7158,10741,10742,3118,7174,10743,10744,7117,6842,6868	[[0,0,0,0,1,0,0,0,0],[0,1,0,0,0,0,0,0,0],[0,1,0,0,0,0,0,0,0],[0,1,1,1,0,0,0,0,0],[0,1,1,0,0,0,0,0,0],[0,1,0,0,0,0,0,0,0],[0,1,0,0,0,0,0,0,0],[0,0,1,0,0,0,0,0,0],[0,1,1,1,0,0,0,0,0],[0,0,1,0,0,0,0,0,0],[0,1,1,1,0,0,0,0,0],[0,0,1,0,0,0,0,0,0],[0,0,0,0,1,0,0,0,0],[0,1,0,0,0,0,0,0,0],[0,1,1,0,0,0,0,0,0],[0,1,0,0,1,0,0,0,0],[0,0,1,0,0,0,0,0,0],[0,0,0,1,0,0,0,0,0],[0,1,0,0,0,0,0,0,0],[0,0,1,0,0,0,0,0,0]]10745	863,10022,10746,3609,1564,8908,7553,10747,1330,10748,8458,10749,10750	[[0,1,1,0,1,0,0,0,0],[0,1,1,1,1,0,0,0,0],[0,1,1,0,0,0,0,0,0],[0,1,1,1,0,0,0,0,0],[0,1,1,0,0,0,0,0,0],[0,1,1,0,0,0,0,0,0],[0,1,1,0,0,0,0,0,0],[0,1,0,0,0,0,0,0,0],[0,0,1,1,0,0,0,0,0],[0,0,1,0,0,0,0,0,0],[0,0,1,0,0,0,0,0,0],[0,0,1,0,0,0,0,0,0],[0,0,1,0,0,0,0,0,0]]10751	10752,10753	[[0,0,0,0,1,0,0,0,0],[0,0,0,0,1,0,0,0,0]]10754	3258,3255,10755,3236,10756,5130,3294,3256,3272,3261,3259,10757,3269,10758,3262,3282,3286,3284,3237,10759,3285,3185,10760	[[0,1,1,1,0,0,0,0,0],[0,1,1,1,0,0,0,0,0],[0,1,0,0,0,0,0,0,0],[0,1,0,0,0,0,0,0,0],[0,1,0,0,0,0,0,0,0],[0,0,1,1,1,0,0,0,0],[0,0,1,1,0,0,0,0,0],[0,0,1,1,0,0,0,0,0],[0,0,1,0,0,0,0,0,0],[0,1,1,1,0,0,0,0,0],[0,0,1,0,1,0,0,0,0],[0,0,0,1,0,0,0,0,0],[0,0,1,0,0,0,0,0,0],[0,0,1,1,0,0,0,0,0],[0,0,1,1,0,0,0,0,0],[0,0,1,0,0,0,0,0,0],[0,0,1,0,1,0,0,0,0],[0,0,0,0,1,0,0,0,0],[0,0,0,1,0,0,0,0,0],[0,0,1,0,0,0,0,0,0],[0,0,1,0,0,0,0,0,0],[0,0,1,0,0,0,0,0,0],[0,0,1,0,0,0,0,0,0]]10761	10762,10688,10763,10764,10765,10766	[[0,0,0,0,1,0,0,0,0],[0,0,0,0,1,0,0,0,0],[0,0,0,0,1,0,0,0,0],[0,0,0,0,1,0,0,0,0],[0,0,0,0,1,0,0,0,0],[0,0,0,0,1,0,0,0,0]]10767	10768	[[0,0,0,0,1,0,0,0,0]]10769	10770,10771,3259,3266,10772,2133,10773,10774,3261,10775,10776,3185,3256,10777,10778,10779,5130,4680,3279,10780,10781,3283,10782,10783,8577,10784,3237,10785,3273,10786,10787	[[0,0,1,0,0,0,0,0,0],[0,0,1,0,0,0,0,0,0],[1,1,0,0,0,0,1,0,0],[0,1,1,0,0,0,1,0,0],[0,0,1,0,0,0,0,0,0],[0,0,1,0,0,0,0,0,0],[0,0,1,0,0,0,0,0,0],[0,0,0,0,1,0,0,0,0],[0,1,1,0,0,0,1,0,0],[0,0,1,0,0,0,0,0,0],[0,0,1,0,0,0,0,0,0],[1,0,0,0,1,0,0,0,0],[0,1,1,0,0,0,1,0,0],[0,0,1,0,0,0,0,0,0],[0,0,1,0,0,0,0,0,0],[0,0,0,0,1,0,0,0,0],[1,1,1,0,0,0,1,0,0],[0,0,1,0,0,0,0,0,0],[0,0,1,0,0,0,0,0,0],[1,1,0,0,0,0,1,0,0],[0,0,1,0,0,0,0,0,0],[0,1,0,0,0,0,1,0,0],[0,0,1,0,0,0,0,0,0],[1,0,0,0,0,0,0,0,0],[1,0,0,0,0,0,0,0,0],[1,0,0,0,0,0,0,0,0],[1,0,0,0,0,0,0,0,0],[1,0,0,0,0,0,0,0,0],[1,0,0,0,0,0,0,0,0],[0,0,0,0,1,0,0,0,0],[0,0,1,0,0,0,0,0,0]]10788	10789	[[0,0,0,0,1,0,0,0,0]]10790	10791	[[0,0,0,0,1,0,0,0,0]]10792	5347,10793,10794	[[0,0,0,0,1,0,0,0,0],[0,0,0,0,1,0,0,0,0],[0,0,0,0,1,0,0,0,0]]10795	2786,1798,3010,3006,10796,10797,10798,10799,10800,3009,3031,2999,10801,2722,3008,2490,3014,10802,10803,10804,10805,3007,10806,290,3028,10807,10808,3932,10809,10810,10811,10812,10813,3690,10814,10815,2950,10816,10817,3017,3981,10818,10819,10820,10821,3003,2998,1826,3715,10822,2924,10823,10824,3021,10825,10826,10827,10828,3672,10829,3987,5889,3026	[[0,1,1,0,0,0,1,0,1],[0,1,1,1,1,1,1,1,1],[0,1,1,1,0,0,1,1,0],[0,1,1,1,0,0,0,0,0],[0,0,0,0,0,0,0,1,0],[0,0,1,0,0,0,0,0,0],[0,0,1,0,0,0,0,0,0],[0,0,1,0,0,0,0,0,0],[0,0,1,0,0,0,0,0,0],[0,0,0,1,0,0,0,0,0],[0,0,1,0,0,0,0,0,0],[0,0,0,1,0,1,0,0,1],[0,0,0,1,0,0,0,1,1],[0,0,0,1,0,0,0,1,1],[0,0,1,0,0,0,0,0,0],[0,0,0,0,0,0,0,0,1],[0,0,1,0,0,0,0,0,0],[0,1,1,1,0,1,1,1,1],[0,0,0,0,0,0,0,1,0],[0,0,0,0,0,0,1,0,0],[0,0,0,0,0,0,0,1,0],[0,1,1,0,1,0,0,1,1],[0,0,1,0,0,0,0,0,0],[0,0,1,0,0,0,0,0,0],[0,0,0,1,0,1,0,0,1],[0,0,1,0,0,0,0,0,0],[0,1,1,1,0,0,0,0,0],[0,0,1,0,0,0,0,0,0],[0,0,1,0,0,0,0,0,0],[0,0,0,0,1,0,0,0,0],[0,0,1,0,0,0,0,0,0],[0,0,1,0,0,0,0,0,0],[0,0,1,0,0,0,0,0,0],[0,0,0,0,0,0,0,1,0],[0,0,0,1,0,0,0,0,0],[0,0,0,1,0,0,0,1,0],[0,0,1,0,0,0,0,0,0],[0,0,1,0,0,0,0,0,0],[0,0,1,1,0,0,0,0,0],[0,0,1,0,0,0,0,0,0],[0,0,0,1,0,0,0,0,1],[0,0,1,0,0,0,0,0,0],[0,0,1,0,0,0,0,0,0],[0,0,1,1,0,0,0,1,1],[0,1,1,1,0,0,1,1,0],[0,1,1,1,0,1,0,1,1],[0,1,1,1,1,1,1,1,1],[0,1,1,1,1,1,1,1,1],[0,0,0,0,0,0,0,1,0],[0,0,0,1,0,0,0,1,0],[0,0,0,0,0,0,0,1,0],[0,0,0,0,0,0,0,0,1],[0,0,1,0,0,0,0,0,0],[0,0,1,0,0,0,0,0,0],[0,1,1,1,1,0,1,1,0],[0,0,1,0,0,0,0,0,0],[0,1,0,1,0,0,1,0,0],[0,0,0,1,0,0,0,0,0],[0,0,0,0,0,0,0,1,0],[0,0,1,0,0,0,0,0,0],[0,0,0,0,0,1,0,0,0],[0,0,1,0,0,0,0,0,0],[0,0,1,0,0,0,0,0,0]]10830	10831,10832,10833,10834,10835,10836	[[0,0,0,0,1,0,0,0,0],[0,0,0,0,1,0,0,0,0],[0,0,0,0,1,0,0,0,0],[0,0,0,0,1,0,0,0,0],[0,0,0,0,1,0,0,0,0],[0,0,0,0,1,0,0,0,0]]10837	10838,10839,7683,10840,10841,7704,10842,10843,10844,10845,10846,5908,10847,10848,10849,10850,10851,10852,10853,10854,10855,10856,10857,7697,10858,2490,7319,2709,10859,10860,10861,8175,10862,10863,10864,10865,10866,10867,10868,10869,10870,2393,10871,10872,2537,10873,10874,10875,10876,10877,10878,10879,3979,9743,10880,3970,10881,10882,10883,10884,10885,9639,10886,10887,10888	[[0,0,1,0,0,0,0,0,0],[0,1,1,0,0,0,0,0,0],[0,0,1,0,0,0,0,0,0],[0,0,1,0,0,0,0,0,0],[0,0,1,0,0,0,0,0,0],[0,0,1,0,0,0,0,0,0],[0,0,1,0,0,0,0,0,0],[0,0,1,0,0,0,0,0,0],[0,0,1,0,0,0,0,0,0],[0,0,1,0,0,0,0,0,0],[0,0,1,0,0,0,0,0,0],[0,0,1,0,0,0,0,0,0],[0,0,1,0,0,0,0,0,0],[0,0,1,0,0,0,0,0,0],[0,0,1,0,0,0,0,0,0],[0,1,0,0,0,0,1,0,0],[0,0,1,0,0,0,0,0,0],[0,0,1,0,0,0,0,0,0],[0,0,1,0,0,0,0,0,0],[0,1,1,0,0,0,0,0,0],[0,0,1,0,0,0,0,0,0],[0,1,0,0,0,0,0,0,0],[0,0,1,0,0,0,0,0,0],[0,0,1,0,0,0,0,0,0],[0,0,1,0,0,0,0,0,0],[0,0,1,0,0,0,0,0,0],[0,1,1,0,0,0,0,0,0],[0,1,1,0,0,0,0,0,0],[0,0,0,0,0,0,0,0,1],[0,1,1,1,1,0,1,0,1],[0,0,1,0,0,0,0,0,0],[0,1,0,0,0,0,0,0,0],[0,0,1,0,0,0,0,0,0],[0,0,1,0,0,0,0,0,0],[0,1,0,0,0,0,0,0,0],[0,0,1,0,0,0,0,0,0],[0,1,0,0,1,0,0,0,0],[0,1,1,0,0,0,1,0,1],[0,1,0,0,0,0,0,0,0],[0,1,0,0,0,0,0,0,0],[0,1,0,0,0,0,0,0,0],[0,1,0,0,0,0,0,0,0],[0,0,1,0,0,0,0,0,0],[1,1,0,0,0,0,0,0,0],[0,1,1,0,0,0,0,0,0],[0,0,1,0,0,0,0,0,0],[0,0,1,0,0,0,0,0,0],[0,1,1,0,0,0,1,0,1],[0,1,0,1,0,0,1,0,0],[0,0,1,0,0,0,0,0,0],[1,1,0,0,0,0,0,0,0],[0,1,0,0,0,0,0,0,0],[0,0,1,0,0,0,0,0,0],[0,0,1,0,0,0,0,0,0],[0,0,1,0,0,0,0,0,0],[0,0,1,0,0,0,0,0,0],[0,0,1,0,0,0,0,0,0],[0,0,1,0,0,0,0,0,0],[0,0,1,0,0,0,0,0,0],[0,0,1,0,0,0,0,0,0],[1,0,0,0,0,0,0,0,0],[1,0,0,0,0,0,0,0,0],[1,0,0,0,0,0,0,0,0],[0,0,1,0,0,0,0,0,0],[0,0,1,0,0,0,0,0,0]]10889	10890,10891,10738,10892,10893,10894,10895,10896,10743,10897,10898,10899,10900,10901,10902,494,10903,8180,10904,10905,10906,10907,10908,10909,10910,10737,10911,10912,10913,10914,10915,10916,6842,2241,10917	[[0,0,1,1,0,0,0,0,0],[0,0,1,0,0,0,0,0,0],[0,0,1,1,0,0,0,0,0],[0,0,1,0,0,0,0,0,0],[0,0,1,0,0,0,0,0,0],[0,0,0,1,0,0,0,0,0],[0,0,1,0,0,0,0,0,0],[0,0,1,0,0,0,0,0,0],[0,0,1,0,0,0,0,0,0],[0,0,1,0,0,0,0,0,0],[0,0,1,0,0,0,0,0,0],[0,0,1,0,0,0,0,0,0],[0,0,1,0,0,0,0,0,0],[0,0,1,0,0,0,0,0,0],[0,0,0,1,0,0,0,0,0],[0,0,1,0,0,0,0,0,0],[0,0,1,0,0,0,0,0,0],[0,0,1,0,1,0,0,0,0],[0,0,1,0,0,0,0,0,0],[0,0,1,1,0,0,0,0,0],[0,0,1,0,0,0,0,0,0],[0,0,0,0,1,0,0,0,0],[0,0,1,0,0,0,0,0,0],[0,0,1,0,0,0,0,0,0],[0,0,1,0,0,0,0,0,0],[0,0,1,0,0,0,0,0,0],[0,0,0,1,0,0,0,0,0],[0,0,1,0,0,0,0,0,0],[0,0,1,0,0,0,0,0,0],[0,0,1,1,0,0,0,0,0],[0,0,1,0,1,0,0,0,0],[0,0,1,0,0,0,0,0,0],[0,0,1,0,0,0,0,0,0],[0,0,1,0,0,0,0,0,0],[0,0,1,0,0,0,0,0,0]]10918	10919	[[0,0,0,0,1,0,0,0,0]]10920	10921,10922	[[0,0,0,0,1,0,0,0,0],[0,0,0,0,1,0,0,0,0]]10923	10924	[[0,0,0,0,1,0,0,0,0]]10925	10926	[[0,0,0,0,1,0,0,0,0]]10927	2022,6007,3268,10928,10929,10930,10931,2052,10932,10933,10934,10935,10936,1315,10937,10938,10939,8174,8177,10940,10941,10942,10943,10944,10945,2647,10946,10947,5974,10948,10949,10950,10951,2232,10952,10953,10954,10955,10956,3242,10957,10958,10959,10960,10961,10962,10337,10963	[[0,1,1,1,1,0,0,0,0],[0,1,1,1,0,0,0,0,0],[0,1,0,0,0,0,0,0,0],[0,1,1,1,0,0,0,0,0],[0,1,1,0,0,0,0,0,0],[0,0,1,0,0,0,0,0,0],[0,1,0,0,0,0,0,0,0],[0,1,1,1,1,0,0,0,0],[0,1,0,0,0,0,0,0,0],[0,0,0,1,0,0,0,0,0],[0,0,0,1,0,0,0,0,0],[0,1,0,1,0,0,0,0,0],[0,0,1,1,0,0,0,0,0],[0,0,0,1,0,0,0,0,0],[0,0,0,1,1,0,0,0,0],[0,0,1,0,0,0,0,0,0],[0,0,1,1,0,0,0,0,0],[0,1,0,0,0,0,0,0,0],[0,1,1,1,0,0,0,0,0],[0,0,1,0,0,0,0,0,0],[0,1,1,1,0,0,0,0,0],[0,0,1,0,0,0,0,0,0],[0,0,1,1,0,0,0,0,0],[0,0,0,1,0,0,0,0,0],[0,0,1,0,0,0,0,0,0],[0,0,1,0,0,0,0,0,0],[0,0,0,1,0,0,0,0,0],[0,0,1,0,0,0,0,0,0],[0,0,1,1,0,0,0,0,0],[0,0,0,1,0,0,0,0,0],[0,0,1,1,0,0,0,0,0],[0,0,1,0,0,0,0,0,0],[0,1,1,1,0,0,0,0,0],[0,1,0,0,0,0,0,0,0],[0,0,1,0,0,0,0,0,0],[0,0,0,1,0,0,0,0,0],[0,0,1,0,0,0,0,0,0],[0,0,1,1,0,0,0,0,0],[0,0,0,1,0,0,0,0,0],[0,0,0,1,0,0,0,0,0],[0,0,1,0,0,0,0,0,0],[0,0,1,0,0,0,0,0,0],[0,0,1,0,0,0,0,0,0],[0,0,1,0,0,0,0,0,0],[0,0,1,0,0,0,0,0,0],[0,0,1,1,0,0,0,0,0],[0,0,1,0,0,0,0,0,0],[0,0,1,0,0,0,0,0,0]]10964	10965	[[0,0,0,0,1,0,0,0,0]]10966	10967,10968,2710,3656,5626,10969,5619,8014,10970,5641,3724,2842,5615,10971	[[0,1,0,0,0,0,0,0,0],[0,1,0,1,0,0,0,0,0],[0,1,0,0,0,0,0,0,0],[0,1,0,1,0,0,0,0,0],[0,1,0,0,0,0,0,0,0],[0,1,0,0,0,0,0,0,0],[0,1,0,0,0,0,0,0,0],[0,0,0,1,0,0,0,0,0],[0,1,0,0,0,0,0,0,0],[0,1,0,0,0,0,0,0,0],[0,0,0,1,0,0,0,0,0],[0,1,0,0,0,0,0,0,0],[0,1,0,0,0,0,0,0,0],[0,1,0,0,0,0,0,0,0]]10972	10973,1908,10974,10975,10976,1904,10977,10978,5762,5163,10979,10980,10981,10982,10983,10984,10985,10986,3672,2354,10987,10988,559,10989,10990,10991,10604,10992,4274,10993,3984,10994,10995,10996,2705,10997,1905,10998,5694,10999,11000,7704,11001,11002,11003,11004,11005,11006	[[0,1,1,0,0,0,0,0,0],[0,0,0,0,0,0,1,0,0],[0,1,0,0,0,0,1,0,0],[0,1,0,0,0,0,0,0,0],[0,1,1,1,0,0,1,0,0],[0,1,1,1,0,0,1,0,0],[0,0,1,0,0,0,0,0,0],[0,0,1,0,0,0,0,0,0],[0,1,1,1,0,0,1,0,0],[0,0,0,1,0,0,0,0,0],[0,0,0,0,1,0,0,0,0],[0,1,0,0,0,0,0,0,0],[0,0,0,0,0,0,1,0,0],[0,0,0,0,1,0,0,0,0],[0,0,0,0,1,0,0,0,0],[0,0,1,0,0,0,0,0,0],[0,1,0,1,0,0,0,0,0],[0,1,1,0,0,0,0,0,0],[0,0,0,0,1,0,0,0,0],[0,0,0,1,0,0,0,0,0],[0,1,1,1,0,0,0,0,0],[0,1,0,0,0,0,0,0,0],[0,1,0,0,0,0,0,0,0],[0,1,0,0,0,0,0,0,0],[0,1,0,0,0,0,0,0,0],[0,0,1,0,0,0,0,0,0],[0,0,0,1,0,0,0,0,0],[0,0,1,0,0,0,0,0,0],[0,0,1,0,0,0,0,0,0],[0,0,0,0,1,0,0,0,0],[0,0,0,0,1,0,0,0,0],[0,0,0,0,1,0,0,0,0],[0,1,0,0,0,0,0,0,0],[0,0,0,0,1,0,0,0,0],[0,0,0,0,1,0,0,0,0],[0,0,0,1,0,0,0,0,0],[0,0,0,0,0,0,1,0,0],[0,0,1,0,0,0,0,0,0],[0,0,0,1,0,0,0,0,0],[0,0,1,0,0,0,0,0,0],[0,0,0,0,1,0,0,0,0],[0,0,0,0,1,0,0,0,0],[0,0,1,0,0,0,0,0,0],[0,0,1,0,0,0,0,0,0],[0,0,0,0,1,0,0,0,0],[0,0,0,1,0,0,0,0,0],[0,0,1,0,0,0,0,0,0],[0,0,1,0,0,0,0,0,0]]11007	11008	[[0,0,0,0,1,0,0,0,0]]11009	5922,10483,3568	[[0,0,0,1,0,0,0,0,0],[0,0,0,1,0,0,0,0,0],[0,0,0,1,0,0,0,0,0]]11010	11011,1397,1496,2747,7758,1520,2748,11012,11013,11014,11015,11016,11017,2746,9187,11018,1515,1261,11019,11020,11021,11022,11023,11024,7754,2310,11025,11026,1516,7752,5157,11027,8564,11028,1499,1526,11029,7765,7751,11030	[[0,0,1,0,0,0,0,0,0],[0,0,1,0,0,0,0,0,0],[0,0,1,0,0,0,0,0,0],[0,0,1,0,0,0,0,0,0],[0,0,1,0,0,0,0,0,0],[0,0,1,0,0,0,0,0,0],[0,0,1,0,0,0,0,0,0],[0,0,1,0,0,0,0,0,0],[0,0,1,0,0,0,0,0,0],[0,0,1,0,0,0,0,0,0],[0,0,1,0,0,0,0,0,0],[0,0,1,0,0,0,0,0,0],[0,0,1,0,0,0,0,0,0],[0,0,1,0,0,0,0,0,0],[0,0,1,0,0,0,0,0,0],[0,0,1,0,0,0,0,0,0],[0,0,1,0,0,0,0,0,0],[0,0,1,0,0,0,0,0,0],[0,0,1,0,0,0,0,0,0],[0,0,1,0,0,0,0,0,0],[0,0,1,0,0,0,0,0,0],[0,0,1,0,0,0,0,0,0],[0,0,1,0,0,0,0,0,0],[0,0,1,0,0,0,0,0,0],[0,0,1,0,0,0,0,0,0],[0,0,1,0,0,0,0,0,0],[0,0,1,0,0,0,0,0,0],[0,0,1,0,0,0,0,0,0],[0,0,1,0,0,0,0,0,0],[0,0,1,0,0,0,0,0,0],[0,0,1,0,0,0,0,0,0],[0,0,1,0,0,0,0,0,0],[0,0,1,0,0,0,0,0,0],[0,0,1,0,0,0,0,0,0],[0,0,1,0,0,0,0,0,0],[0,0,1,0,0,0,0,0,0],[0,0,1,0,0,0,0,0,0],[0,0,1,0,0,0,0,0,0],[0,0,1,0,0,0,0,0,0],[0,0,1,0,0,0,0,0,0]]11031	11032,11033	[[0,0,0,0,1,0,0,0,0],[0,0,0,0,1,0,0,0,0]]11034	11035	[[0,0,0,0,1,0,0,0,0]]11036	11037	[[0,0,0,0,1,0,0,0,0]]11038	11039,11040,11041	[[0,0,0,1,0,0,0,0,0],[0,0,0,1,0,0,0,0,0],[0,0,0,1,0,0,0,0,0]]11042	7983,6386,6383,1197,7982,3094,8693,11043,2248,2242,3892,6360,6377,2285,2333,2331,5170,7990,9057,11044,2315,7944,2265,11045,2434,1397,11046,11047,9079,11048,7986,7985,11049,6552,11050,5169,9052,11051,11052,11053,8700,11054,2353,11055,5178,2342,11056,5157,895,11057,5167,2702,7994,1795,11058,11059,11060,8100,16,11061,5158,11062,3047,2178,1261,11063,5136,2435,11064,9417,3903,11065,2310,11066,10039,11067,5173,7438,11068	[[1,1,0,1,0,0,1,1,1],[1,1,1,1,0,0,1,1,1],[1,0,1,1,0,0,0,1,0],[1,1,1,1,0,0,1,1,1],[1,0,0,0,0,0,0,0,0],[1,0,0,1,0,0,0,1,0],[1,0,0,0,0,0,0,1,0],[1,0,0,0,0,0,0,0,0],[1,0,0,0,1,0,0,1,0],[1,0,0,1,0,0,0,1,0],[1,0,0,0,0,0,0,1,0],[1,1,1,1,1,0,1,1,1],[1,1,1,1,0,0,1,1,1],[1,0,0,0,0,0,0,0,0],[1,0,0,0,0,0,0,0,0],[1,0,0,0,0,0,0,0,0],[0,1,1,1,0,0,0,1,0],[1,0,1,0,0,0,0,1,1],[1,0,0,0,0,0,0,1,0],[0,1,1,1,0,0,1,1,1],[0,1,1,1,0,0,0,1,0],[0,1,1,1,0,0,1,1,0],[1,0,0,1,0,0,0,1,0],[1,0,0,0,0,0,0,1,0],[1,0,0,1,0,0,0,0,1],[0,1,0,1,1,0,0,1,0],[0,0,0,1,0,0,0,1,0],[0,0,0,1,0,0,0,1,0],[1,0,0,0,0,0,0,0,0],[0,0,0,1,0,0,0,1,0],[1,0,1,1,0,0,0,1,0],[1,0,1,1,0,0,0,0,0],[1,0,0,0,0,0,0,1,0],[0,1,0,0,0,0,1,0,0],[0,0,0,1,0,0,0,1,0],[0,1,0,1,0,0,1,0,0],[1,0,0,0,1,0,0,0,0],[0,0,0,1,0,0,0,0,0],[0,0,0,1,0,0,0,1,1],[0,0,1,1,0,0,0,1,0],[0,0,1,0,0,0,0,0,0],[0,1,0,0,0,0,0,0,0],[0,1,1,1,1,0,1,1,0],[0,1,1,1,0,0,1,1,0],[0,1,1,1,0,0,0,1,0],[0,1,0,0,0,0,0,0,0],[0,0,1,0,0,0,0,0,0],[0,0,1,1,0,0,0,1,0],[0,0,0,1,0,0,0,0,0],[0,0,0,0,0,0,0,1,0],[0,0,1,1,0,0,0,1,0],[0,0,1,0,0,0,0,0,0],[0,0,0,1,0,0,0,0,0],[0,0,0,1,0,0,0,0,0],[0,0,1,0,0,0,0,0,0],[0,0,0,1,0,0,0,1,0],[0,0,1,0,0,0,0,0,0],[0,0,0,1,0,0,0,0,0],[0,0,0,0,0,0,0,1,0],[0,0,1,1,0,0,0,0,0],[0,0,0,1,0,0,0,1,0],[0,0,1,1,1,0,0,1,0],[0,0,0,1,0,0,0,1,0],[0,0,0,1,1,0,0,0,0],[0,0,0,1,0,0,0,1,0],[0,0,0,1,0,0,0,1,0],[0,0,0,1,0,0,0,0,0],[0,0,1,1,0,0,0,1,1],[0,0,0,1,0,0,0,1,0],[0,0,0,1,0,0,0,0,0],[0,0,1,1,0,0,0,1,0],[0,0,1,0,1,0,0,0,0],[0,0,0,0,0,0,0,1,0],[0,0,0,0,0,0,0,1,0],[0,0,0,1,0,0,0,1,0],[0,0,0,1,0,0,0,1,0],[0,0,0,1,0,0,0,0,0],[0,0,0,1,0,0,0,1,0],[0,0,1,0,0,0,0,0,0]]11069	2372,5086,11070,11071,5087,5096,5081,11072,1570,11073,4878,2387,650,5095,3435,11074,11075,11076	[[0,1,0,0,0,0,0,0,0],[0,0,1,1,1,0,0,0,0],[0,1,0,0,0,0,0,0,0],[0,0,1,0,0,0,0,0,0],[0,0,1,0,0,0,0,0,0],[0,1,1,0,0,0,0,0,0],[0,1,1,0,0,0,0,0,0],[0,1,0,0,0,0,0,0,0],[0,1,0,0,0,0,0,0,0],[0,1,0,0,0,0,0,0,0],[0,1,0,0,0,0,0,0,0],[0,1,0,0,0,0,0,0,0],[0,0,1,0,0,0,0,0,0],[0,1,1,1,1,0,0,0,0],[0,0,1,0,0,0,0,0,0],[0,0,1,0,0,0,0,0,0],[0,1,0,0,0,0,0,0,0],[0,1,0,0,0,0,0,0,0]]11077	11078	[[0,0,0,0,1,0,0,0,0]]11079	9626	[[0,0,0,0,1,0,0,0,0]]11080	11081,8195,5126,11082,11083,2864,11084,2141,5004,11085,11086,5084,11087,8074,792,2865,8000,8002,11088,7440,3906,11089,11090,11091,8651,11092,8042,8039,11093,11094,8032,11095,11096,5155,11097,4226,11098,11099,5135,11100,11101,11102,9422,11103,11104,2862,3887	[[0,1,1,1,0,0,1,0,0],[0,1,1,1,0,0,1,1,0],[0,0,1,0,0,0,0,0,0],[0,1,0,1,1,0,1,1,1],[0,0,1,0,0,0,0,0,0],[0,1,1,0,0,0,1,0,0],[0,0,1,1,0,0,0,0,0],[0,0,1,0,0,0,0,0,1],[0,1,1,0,0,0,0,0,0],[0,0,1,0,0,0,0,0,0],[0,1,0,1,0,0,0,0,0],[0,1,0,1,1,0,1,1,0],[0,0,1,0,0,0,0,0,0],[0,0,0,1,0,0,0,1,0],[0,1,1,0,0,0,1,0,0],[0,1,0,0,0,0,0,0,0],[0,0,0,0,1,0,0,1,0],[0,0,1,0,0,0,0,0,0],[0,0,1,0,0,0,0,0,0],[0,0,1,0,0,0,0,0,0],[0,1,1,0,0,0,1,0,1],[0,0,1,0,0,0,0,0,0],[0,0,1,0,0,0,0,0,0],[0,0,0,1,0,0,0,1,0],[0,0,1,0,0,0,0,0,0],[0,0,0,0,0,0,0,1,0],[0,0,1,0,0,0,0,0,1],[0,0,1,0,0,0,0,0,0],[0,1,0,0,0,0,0,0,0],[0,0,0,1,0,0,0,0,0],[0,0,1,0,0,0,0,0,0],[0,0,0,0,1,0,0,0,1],[0,0,1,1,0,0,0,0,0],[0,0,1,0,0,0,0,0,0],[0,0,0,1,0,0,0,0,1],[0,0,0,0,0,0,0,1,0],[0,0,1,0,0,0,0,0,0],[0,1,0,0,0,0,1,1,0],[0,0,1,0,0,0,0,0,1],[0,0,1,1,0,0,0,0,0],[0,1,0,1,0,0,0,1,0],[0,1,1,1,0,0,0,1,0],[0,1,0,0,0,0,0,0,0],[0,0,1,0,0,0,0,0,0],[0,0,0,0,0,0,0,1,0],[0,1,1,0,1,0,1,0,0],[0,1,1,1,0,0,1,1,0]]11105	5573,11106,4473,11107,7675,11108,849,11109,7690,10160,10139,11110,11111,11112,11113,11114,4796,2919,11115,11116,10164,11117,11118,11119,4857,11120,11121,7681,5568,2160,11122,10353,4852,11123,7084,11124,7647,11125,10369,10168,11126,11127,11128,4795,2343,4798,11129,7719,10988,11130,11131,11132,8220,11133,7680,11134,7662,4801,4799,7657,11135,7712,11136,11137,4790,3665,3196,7669,10379,11138,7709,3672,11139,11140,4850,4855,4853,11141,5569,10378,11142,11143,11144,11145,10372,11146,7673,2911,11147,11148,2170,11149	[[0,0,0,0,0,1,0,0,0],[0,0,0,0,0,0,1,0,0],[0,1,1,1,1,1,1,0,0],[0,1,1,0,1,1,1,0,0],[0,1,1,0,0,0,0,0,0],[0,1,1,1,0,1,1,0,0],[0,0,1,0,0,0,0,0,0],[0,0,1,0,0,0,0,0,0],[0,0,1,0,0,0,0,0,0],[0,0,1,0,0,1,0,0,0],[0,1,1,0,0,0,1,0,0],[0,0,1,0,0,0,0,0,0],[0,1,1,1,0,0,1,0,0],[0,1,1,0,0,0,1,0,0],[0,1,1,0,1,0,1,0,0],[0,0,1,0,0,0,0,0,0],[0,0,0,0,0,0,1,0,0],[0,0,1,0,0,0,0,0,0],[0,1,1,1,0,0,1,0,0],[0,1,1,0,1,1,1,0,0],[0,0,1,0,0,0,0,0,0],[0,1,1,0,0,0,1,0,0],[0,1,0,0,0,0,1,0,0],[0,1,0,0,0,0,1,0,0],[0,1,0,0,0,0,1,0,0],[0,0,0,0,0,1,0,0,0],[0,0,1,0,0,0,0,0,0],[0,1,0,1,0,0,0,0,0],[0,1,0,0,0,1,1,0,0],[0,1,0,0,0,0,0,0,0],[0,0,1,0,1,0,0,0,0],[1,0,1,0,0,0,0,0,0],[0,1,0,0,0,0,1,0,0],[0,1,1,1,1,0,1,0,0],[0,0,0,1,0,0,1,0,0],[0,1,1,0,0,0,0,0,0],[0,1,0,0,0,0,0,0,0],[0,1,1,0,0,0,0,0,0],[0,0,1,0,0,0,0,0,0],[0,0,1,0,0,1,0,0,0],[0,1,0,0,0,0,1,0,0],[0,0,1,0,0,0,0,0,0],[0,1,0,0,0,0,1,0,0],[1,1,1,1,0,0,1,0,0],[0,1,0,1,0,0,1,0,0],[1,0,1,1,0,0,0,0,0],[0,0,1,0,0,0,0,0,0],[0,0,1,0,0,0,0,0,0],[0,0,1,0,0,0,0,0,0],[0,1,0,0,1,0,0,0,0],[0,0,1,0,0,0,0,0,0],[0,0,0,0,1,0,0,0,0],[0,1,1,0,0,0,1,0,0],[0,1,0,0,0,0,0,0,0],[1,1,1,0,0,0,0,0,0],[0,0,1,0,0,0,0,0,0],[0,1,0,1,0,0,0,0,0],[1,1,0,0,0,0,1,0,0],[0,1,1,0,0,0,0,0,0],[0,1,0,0,0,0,0,0,0],[0,1,1,1,0,1,1,0,0],[0,0,1,0,0,0,0,0,0],[0,0,1,0,0,0,0,0,0],[0,0,1,0,0,0,0,0,0],[1,0,1,0,1,0,1,0,0],[0,0,1,0,0,0,0,0,0],[0,0,1,0,0,0,0,0,0],[0,1,1,0,0,0,0,0,0],[0,0,0,1,0,0,0,0,0],[0,0,0,0,0,1,0,0,0],[0,1,0,1,0,0,0,0,0],[0,0,1,0,0,0,0,0,0],[0,1,1,0,0,0,1,0,0],[0,0,1,0,0,0,0,0,0],[0,1,0,0,0,0,1,0,0],[0,1,0,0,0,0,1,0,0],[0,1,0,0,0,0,0,0,0],[0,1,0,0,0,0,1,0,0],[0,0,1,0,0,0,0,0,0],[0,1,0,0,0,0,0,0,0],[0,0,0,0,0,1,0,0,0],[0,0,1,0,0,0,0,0,0],[0,1,0,0,0,0,0,0,0],[1,0,0,0,0,0,0,0,0],[1,1,1,0,0,1,0,0,0],[0,1,0,0,0,0,0,0,0],[0,1,0,0,0,0,0,0,0],[0,0,1,0,0,0,0,0,0],[0,0,1,0,0,0,0,0,0],[1,0,0,0,0,0,0,0,0],[0,0,1,0,0,0,0,0,0],[0,0,1,0,0,0,0,0,0]]11150	11151	[[0,0,0,0,1,0,0,0,0]]11152	11153,1094,2820,3534,11154	[[0,0,0,1,0,0,0,0,0],[0,0,0,1,0,0,0,0,0],[0,0,0,1,0,0,0,0,0],[0,0,0,1,0,0,0,0,0],[0,0,0,1,0,0,0,0,0]]11155	11156,11157	[[0,0,1,0,0,0,0,0,0],[0,0,1,0,0,0,0,0,0]]11158	11159	[[0,0,0,0,1,0,0,0,0]]11160	11161,11162,11163,11164,11165,11166,11167,11168,11169	[[0,0,0,0,1,0,0,0,0],[0,0,0,0,1,0,0,0,0],[0,0,0,0,1,0,0,0,0],[0,0,0,0,1,0,0,0,0],[0,0,0,0,1,0,0,0,0],[0,0,0,0,1,0,0,0,0],[0,0,0,0,1,0,0,0,0],[0,0,0,0,1,0,0,0,0],[0,0,0,0,1,0,0,0,0]]11170	11171	[[0,0,0,0,1,0,0,0,0]]11172	11173,11174	[[0,0,0,0,1,0,0,0,0],[0,0,0,0,1,0,0,0,0]]11175	11176,11177,11178	[[0,0,0,1,0,0,0,0,0],[0,0,0,1,0,0,0,0,0],[0,0,0,1,0,0,0,0,0]]11179	2820,11153,3534,11154,11180,1094,11181	[[0,0,0,1,0,0,0,0,0],[0,0,0,1,0,0,0,0,0],[0,0,0,1,0,0,0,0,0],[0,0,0,1,0,0,0,0,0],[0,0,0,0,1,0,0,0,0],[0,0,0,1,0,0,0,0,0],[0,0,0,0,1,0,0,0,0]]11182	11183	[[0,0,0,0,1,0,0,0,0]]11184	4575	[[0,0,0,0,1,0,0,0,0]]11185	11186,11187,7486,10494,11188,11189,11190,11191,11192,11193,3560,11194,3569,10485,11195,11196,11197,2053,3568,11198,10496,11199,11200,11201,4172,10461,11202,11203,11204,1982,11205,11206,8720,10503,10465	[[0,0,1,0,0,0,0,0,0],[0,0,1,0,0,0,0,0,0],[0,0,1,0,0,0,0,0,0],[0,0,1,0,0,0,0,0,0],[0,0,1,0,0,0,0,0,0],[0,0,1,0,0,0,0,0,0],[0,0,1,0,0,0,0,0,0],[0,0,1,0,0,0,0,0,0],[0,0,1,0,0,0,0,0,0],[0,0,1,0,0,0,0,0,0],[0,0,1,0,0,0,0,0,0],[0,0,1,0,0,0,0,0,0],[0,0,1,0,0,0,0,0,0],[0,0,1,0,0,0,0,0,0],[0,0,1,0,0,0,0,0,0],[0,0,1,0,0,0,0,0,0],[0,0,1,0,0,0,0,0,0],[0,0,1,0,0,0,0,0,0],[0,0,1,0,0,0,0,0,0],[0,0,1,0,0,0,0,0,0],[0,0,1,0,0,0,0,0,0],[0,0,1,0,0,0,0,0,0],[0,0,1,0,0,0,0,0,0],[0,0,1,0,0,0,0,0,0],[0,0,1,0,0,0,0,0,0],[0,0,1,0,0,0,0,0,0],[0,0,1,0,0,0,0,0,0],[0,0,1,0,0,0,0,0,0],[0,0,1,0,0,0,0,0,0],[0,0,1,0,0,0,0,0,0],[0,0,1,0,0,0,0,0,0],[0,0,1,0,0,0,0,0,0],[0,0,1,0,0,0,0,0,0],[0,0,1,0,0,0,0,0,0],[0,0,1,0,0,0,0,0,0]]11207	6420	[[0,0,0,0,1,0,0,0,0]]11208	6305,11209,11210,1733,11211,4929,4930,10794,11212,11213,11214,11215,3166,11216,11217	[[0,0,1,1,0,0,0,0,0],[0,0,1,0,0,0,0,0,0],[0,0,1,0,0,0,0,0,0],[0,0,0,1,0,0,0,0,0],[0,0,0,0,1,0,0,0,0],[0,0,1,1,0,0,0,0,0],[0,0,0,1,0,0,0,0,0],[0,0,1,1,1,0,0,0,0],[0,0,0,1,0,0,0,0,0],[0,0,1,1,0,0,0,0,0],[0,0,1,0,0,0,0,0,0],[0,0,0,1,0,0,0,0,0],[0,0,0,1,0,0,0,0,0],[0,0,0,1,0,0,0,0,0],[0,0,0,1,0,0,0,0,0]]11218	11219	[[0,0,0,0,1,0,0,0,0]]11220	10280,2852,11221	[[0,0,0,0,1,0,0,0,0],[0,0,0,0,1,0,0,0,0],[0,0,0,0,1,0,0,0,0]]11222	10456,3569,6227,10465,6644,6242,6239,11223,11224,6246,11225,6243,11226,11227,3867,11228,6252,11229,11230,11231,10476,6248,11232,463,6221,6925,4454,2053,336,10461,11233,11234,2673,10494,11235,11236,11188,2051,1671,11237,5289,11238,11239,6651,11240,6226,3873,11241,11242,769,2665,11243,11244	[[0,1,1,0,0,0,0,1,0],[0,0,1,0,0,0,0,0,0],[0,0,1,0,0,0,0,1,0],[0,0,1,0,0,0,0,0,0],[0,0,0,0,0,0,0,1,0],[0,1,1,1,0,0,1,0,0],[0,0,1,0,0,0,0,0,0],[0,0,0,0,0,1,0,0,0],[0,0,1,0,0,0,0,0,0],[0,1,1,0,0,0,0,0,0],[0,0,0,0,0,1,0,0,0],[0,1,1,1,0,1,1,1,0],[0,0,1,0,0,0,0,0,0],[0,0,1,0,0,0,0,1,0],[0,0,1,0,0,0,0,0,0],[0,0,1,0,0,0,0,0,0],[0,1,1,0,0,0,0,0,0],[0,0,1,0,0,0,0,1,0],[0,0,0,0,0,1,0,0,0],[0,0,1,0,0,0,0,0,0],[0,1,1,0,0,0,0,0,0],[0,1,0,0,0,0,0,0,0],[0,1,0,0,0,0,0,0,0],[0,1,0,0,0,0,0,0,0],[0,1,1,0,0,0,0,1,0],[0,0,1,0,0,0,1,1,0],[0,1,0,0,0,0,0,0,0],[0,1,0,0,0,0,0,0,0],[0,1,0,0,0,0,1,1,0],[0,0,1,0,0,0,0,0,0],[0,1,1,1,0,1,1,1,0],[0,0,0,0,0,0,0,1,0],[0,1,1,0,0,0,0,0,0],[0,0,1,0,0,0,0,1,0],[0,0,1,0,0,0,0,1,0],[0,0,1,0,0,0,0,0,0],[0,0,1,0,0,0,0,0,0],[0,0,0,0,0,0,0,1,0],[0,0,0,0,0,0,0,1,0],[0,0,1,0,0,0,0,0,0],[0,0,1,0,0,0,0,0,0],[0,0,1,0,0,0,0,0,0],[0,0,1,0,0,0,0,0,0],[0,0,0,0,0,0,1,0,0],[0,0,0,0,0,0,1,0,0],[0,0,1,0,0,0,1,1,0],[0,0,1,0,0,0,0,0,0],[0,0,1,0,0,0,0,0,0],[0,0,1,0,0,0,0,1,0],[0,0,1,0,0,0,1,0,0],[0,0,1,0,0,0,0,0,0],[0,0,0,0,0,0,1,0,0],[0,0,0,0,0,0,0,1,0]]11245	11246,11247,11248,1754,11249,11250,11251,11252,1703,4317,9002,11253,11254,11255,11256,11257,11258,11259,11260,11261,10374,11262,11263	[[0,0,1,0,0,0,0,0,0],[0,0,0,1,0,0,0,0,0],[0,0,0,1,0,0,0,0,0],[0,1,0,1,0,0,0,0,0],[0,0,0,1,0,0,0,0,0],[0,0,0,1,0,0,0,0,0],[0,0,0,1,0,0,0,0,0],[0,0,0,1,0,0,0,0,0],[0,0,0,0,1,0,0,0,0],[0,0,0,1,0,0,0,0,0],[0,0,1,0,0,0,0,0,0],[0,0,1,0,0,0,0,0,0],[0,0,1,0,0,0,0,0,0],[0,0,1,0,0,0,0,0,0],[0,0,0,1,0,0,0,0,0],[0,1,0,0,0,0,0,0,0],[0,1,0,0,0,0,0,0,0],[0,0,1,0,0,0,0,0,0],[0,1,0,0,0,0,0,0,0],[0,1,0,0,1,0,0,0,0],[0,1,0,0,0,0,0,0,0],[0,0,0,1,0,0,0,0,0],[0,0,1,0,0,0,0,0,0]]11264	11265	[[0,0,0,0,1,0,0,0,0]]11266	11267,11268,11269	[[0,0,0,0,1,0,0,0,0],[0,0,0,0,1,0,0,0,0],[0,0,0,0,1,0,0,0,0]]11270	11271,11272,10944,11273,3534,11274	[[0,0,0,1,0,0,0,0,0],[0,0,0,1,0,0,0,0,0],[0,0,0,1,0,0,0,0,0],[0,0,0,1,0,0,0,0,0],[0,0,0,1,0,0,0,0,0],[0,0,0,1,0,0,0,0,0]]11275	11276,6622,11277,5348,9044,9065,11278,10794,11279,8607,11280,3216,11281,11282,11283,5354,11284,2530,11285,11286,11287,11288,11289,11290,11291,10664,11292,580,11293	[[0,0,1,0,0,0,0,0,0],[0,0,0,1,0,0,0,0,0],[0,0,1,0,1,0,0,0,0],[0,0,1,1,0,0,0,0,0],[0,0,0,1,0,0,0,0,0],[0,0,0,1,0,0,0,0,0],[0,0,1,1,1,0,0,0,0],[0,0,0,1,0,0,0,0,0],[0,0,0,1,0,0,0,0,0],[0,0,0,1,0,0,0,0,0],[0,0,1,0,0,0,0,0,0],[0,0,1,0,0,0,0,0,0],[0,0,1,1,0,0,0,0,0],[0,0,0,1,0,0,0,0,0],[0,0,1,1,0,0,0,0,0],[0,0,1,1,0,0,0,0,0],[0,0,0,1,0,0,0,0,0],[0,0,0,1,0,0,0,0,0],[0,0,1,0,0,0,0,0,0],[0,0,0,1,0,0,0,0,0],[0,0,0,1,0,0,0,0,0],[0,0,1,0,0,0,0,0,0],[0,0,0,1,0,0,0,0,0],[0,0,0,1,0,0,0,0,0],[0,0,0,1,0,0,0,0,0],[0,0,0,1,0,0,0,0,0],[0,0,0,1,0,0,0,0,0],[0,0,0,1,0,0,0,0,0],[0,0,1,0,0,0,0,0,0]]11294	11295,11296	[[0,0,0,0,1,0,0,0,0],[0,0,0,0,1,0,0,0,0]]11297	404,3894,11298,370,11299,7282,11300,10308,5473,11301,1251,5023,11302,11225,11303,6763,11304,11305,3019,11306,11307,11308,11309,3443,11310,11311,5438,11312,5814,11313,5818,11314,11315,6786,11316,1259,11317,5934,11318,11319,11320,1674,11321,11322,2999,5472,11323,11324,11325,11326,1040,11230,11327,11328,5847,11329,5828,11330,11331,3002,5838,11332,11333,11334,11335,10399,11336,11337,11338,11339,7329,5445,6784,415,5830,11340,3013,6772,5439,11341,1570,11342,1143,11343,3729,11344,8595,11345,10806,3016,6761,6760,5821,8610,6864,11346,2225,6740,8841,1338,11347,8662,6796,3742,11348,1258,10199,5836,8612,7295,11349,5797,3005,6781,8181,9689,1449,11350,6764,5431,5794,11351,11352,10981,3015,5432,11353,11354,3004,10814,4424,11355	[[0,1,1,0,0,0,1,0,0],[0,0,0,1,0,0,0,0,0],[0,0,1,0,0,0,0,0,0],[0,0,0,1,0,0,0,0,1],[0,0,0,0,1,0,0,0,0],[0,1,0,0,0,0,1,0,0],[0,1,1,0,0,0,0,0,0],[0,0,0,1,0,0,0,0,0],[0,0,0,1,0,0,0,0,0],[0,0,1,0,0,0,0,0,0],[0,0,1,0,0,0,0,0,0],[0,1,1,0,0,0,1,0,0],[1,1,1,1,0,1,1,0,1],[0,1,1,0,0,0,0,0,0],[0,0,1,0,0,0,0,0,0],[1,0,1,1,0,0,0,0,1],[0,1,0,0,0,0,0,0,0],[0,0,0,1,0,0,0,0,0],[0,0,0,1,0,0,0,0,0],[1,1,0,1,0,1,1,0,0],[0,0,1,0,0,0,0,0,0],[0,1,1,0,0,0,0,0,0],[0,1,1,0,0,0,0,0,0],[0,1,0,0,0,1,0,0,0],[0,0,0,1,0,0,0,0,0],[1,1,0,0,0,0,1,0,0],[0,0,1,1,0,0,0,0,1],[0,1,1,0,0,0,0,0,0],[0,1,1,1,0,1,0,0,1],[0,1,0,0,0,0,0,0,0],[1,1,1,1,1,0,0,0,1],[0,0,1,0,0,0,0,0,0],[0,1,0,0,0,0,0,0,0],[0,0,1,0,0,1,0,0,1],[0,0,1,0,0,0,0,0,0],[0,0,0,1,0,0,0,0,1],[0,0,1,0,0,0,0,0,0],[0,0,1,0,0,0,0,0,0],[0,1,1,0,0,0,0,0,0],[0,1,0,0,0,0,0,0,0],[0,1,0,0,0,0,0,0,0],[0,1,0,0,0,0,0,0,0],[0,1,0,0,0,0,0,0,0],[0,0,0,0,0,1,0,0,0],[0,1,0,1,0,0,1,0,0],[0,1,0,0,0,0,0,0,0],[0,1,0,0,0,0,1,0,0],[0,1,0,0,0,0,0,0,0],[0,1,0,0,0,0,0,0,0],[0,1,0,0,0,0,0,0,0],[0,1,0,0,0,0,1,0,0],[0,1,1,0,0,0,0,0,0],[0,1,0,0,0,0,0,0,0],[0,1,0,0,0,0,1,0,0],[0,1,0,0,0,0,1,0,0],[0,1,0,0,0,0,0,0,0],[0,1,0,0,0,0,0,0,0],[0,1,1,0,0,0,0,0,0],[0,1,1,0,0,0,0,0,0],[0,1,0,1,0,0,1,0,0],[0,1,0,0,0,0,1,0,0],[0,1,0,0,0,0,0,0,0],[0,0,1,0,0,0,0,0,0],[0,1,0,0,0,0,0,0,0],[0,1,0,0,0,0,0,0,0],[1,0,1,1,0,0,0,0,0],[0,1,0,0,0,0,0,0,0],[0,1,0,0,0,0,0,0,0],[0,1,0,0,0,0,0,0,0],[1,0,0,0,0,0,0,0,0],[1,0,0,0,0,0,0,0,0],[0,0,0,1,0,0,0,0,0],[0,0,1,1,0,1,0,0,0],[0,1,0,0,0,0,1,0,0],[0,0,0,1,0,0,0,0,0],[0,0,0,1,0,0,0,0,0],[0,0,1,1,0,0,0,0,0],[1,0,0,0,0,0,0,0,0],[0,0,1,0,0,0,0,0,0],[0,0,0,1,0,0,0,0,1],[0,0,0,0,1,0,0,0,0],[0,0,0,1,0,0,0,0,0],[0,0,0,1,0,0,0,0,0],[0,0,0,1,0,0,0,0,0],[0,0,1,0,0,0,0,0,0],[0,0,1,0,0,0,0,0,0],[0,0,0,1,0,0,0,0,0],[0,0,0,0,0,0,1,0,0],[0,0,0,1,0,0,0,0,0],[0,0,0,1,0,0,0,0,0],[0,0,1,1,0,0,0,0,0],[0,0,1,0,0,0,0,0,0],[0,0,0,1,0,0,0,0,0],[0,0,0,1,0,0,0,0,1],[0,0,1,1,0,0,0,0,0],[0,0,1,0,0,0,0,0,0],[0,0,0,1,0,0,0,0,1],[0,0,1,1,0,0,0,0,0],[0,0,0,1,0,0,0,0,0],[0,0,0,0,1,0,0,0,0],[0,0,0,1,0,0,0,0,0],[0,0,1,1,0,0,0,0,1],[0,0,0,0,0,0,1,0,0],[0,0,1,0,0,0,0,0,0],[0,0,0,1,0,0,0,0,0],[0,0,1,1,0,0,0,0,1],[0,0,1,0,0,0,0,0,0],[0,0,1,0,0,0,0,0,0],[0,0,0,1,0,0,0,0,1],[0,0,1,0,0,0,0,0,0],[0,0,0,1,0,0,0,0,0],[0,0,1,0,0,0,0,0,0],[0,0,1,1,0,0,1,0,0],[0,0,1,1,0,0,0,0,1],[0,0,0,1,0,0,0,0,0],[0,0,0,0,1,0,0,0,0],[0,0,0,0,1,0,0,0,0],[0,0,1,0,0,0,0,0,0],[0,0,1,0,0,0,0,0,0],[0,0,0,0,1,0,0,0,0],[0,0,0,1,0,0,0,0,0],[0,0,0,1,0,0,0,0,0],[0,0,1,1,0,0,0,0,0],[0,0,0,0,1,0,0,0,0],[0,0,0,1,0,0,0,0,0],[0,0,1,0,0,0,0,0,0],[0,0,0,1,0,0,0,0,0],[0,0,1,0,0,1,0,0,1],[0,0,1,1,0,0,0,0,0],[0,0,0,0,0,0,1,0,0],[0,0,0,0,0,0,1,0,0],[0,0,0,1,0,0,0,0,0]]11356	11233,6243,6242	[[0,0,0,1,0,0,0,0,0],[0,0,0,1,0,0,0,0,0],[0,0,0,1,0,0,0,0,0]]11357	2808,10655,11358	[[0,0,0,1,0,0,0,0,0],[0,0,0,1,0,0,0,0,0],[0,0,0,1,0,0,0,0,0]]11359	9105,8727,3275,11360,11361,11362,11363,8337,11364,9573,1432,11365,8324,11366,11367,1276,231,11355,11368,11369,8732,7583,11370,3356,4136,8733,11371,5147,2861,8729,8728,2634,2652,11372,11373,2662,11374,8342,11375,7579,8330,2642,4140,8735,138,11376,8328,11377,10495,11378,1720,11379	[[0,1,0,0,0,0,1,0,0],[0,0,1,1,1,0,0,1,0],[0,0,1,0,0,0,0,1,0],[0,0,1,0,0,0,0,0,0],[0,0,0,0,1,0,0,0,0],[0,0,1,0,0,0,0,0,0],[0,0,1,0,0,0,0,0,0],[0,0,0,0,0,0,0,1,0],[0,1,0,1,0,0,1,1,0],[0,0,1,0,1,0,0,0,0],[0,0,1,0,0,0,0,1,0],[0,1,1,0,0,0,0,0,0],[0,1,1,0,0,0,0,0,0],[0,0,1,0,0,0,0,0,0],[0,0,1,0,0,0,0,0,0],[0,0,0,1,0,0,0,1,0],[0,1,0,0,0,0,0,0,0],[0,1,0,1,0,0,0,1,0],[0,1,0,0,0,0,0,1,0],[0,1,1,1,0,0,0,0,0],[0,1,0,0,0,0,1,0,0],[0,1,1,1,1,0,1,1,0],[0,0,0,0,0,0,0,1,0],[0,0,0,0,0,0,0,1,0],[0,0,0,1,0,0,0,1,0],[0,1,1,0,0,0,0,0,0],[0,1,1,0,0,0,1,1,0],[0,1,1,0,0,0,0,0,0],[0,1,1,0,0,0,0,1,0],[0,1,1,0,0,0,1,0,0],[0,1,1,0,0,0,1,0,0],[0,1,1,0,0,0,0,1,0],[0,1,1,0,0,0,1,0,0],[0,0,0,0,0,0,0,1,0],[0,1,1,0,0,0,1,1,0],[0,1,0,0,0,0,1,0,0],[0,1,0,0,0,0,1,1,0],[0,1,1,0,0,0,0,0,0],[0,0,0,0,0,0,0,1,0],[0,0,0,1,0,0,0,0,0],[0,0,0,0,0,0,0,1,0],[0,1,0,0,0,0,1,0,0],[0,0,1,0,0,0,0,0,0],[0,0,1,0,0,0,0,0,0],[0,0,1,0,0,0,0,1,0],[0,1,0,0,0,0,1,1,0],[0,0,1,1,0,0,0,0,0],[0,1,0,0,0,0,1,0,0],[0,0,1,0,0,0,0,0,0],[0,0,1,0,0,0,0,0,0],[0,0,0,0,0,0,0,1,0],[0,0,1,0,0,0,0,0,0]]11380	11381,11382,11383,11384,11385,11386,6837,11387,11388,7134,5437,11389,11390,11391,11392,11393,10426,11394,11395,11396,11397,11398,11399,11400,11401,11402,11403,5452	[[1,1,1,0,0,0,0,1,0],[0,0,1,0,0,0,0,1,0],[1,0,1,1,0,0,0,1,0],[1,0,1,0,0,0,0,0,0],[0,0,1,0,0,0,0,0,0],[0,0,1,0,0,0,0,0,0],[1,1,1,0,0,0,0,1,0],[1,1,1,0,0,0,0,1,0],[1,1,1,0,0,0,0,1,0],[0,0,0,0,0,0,0,1,0],[1,0,1,0,0,0,0,0,0],[1,0,1,0,0,0,0,1,0],[1,1,0,0,0,0,0,1,0],[1,1,0,0,0,0,0,1,0],[1,1,0,0,0,0,0,1,0],[1,0,0,0,0,0,0,0,0],[0,1,1,1,0,0,0,0,0],[1,1,1,0,0,0,0,0,0],[0,0,0,0,0,0,0,1,0],[0,0,1,0,0,0,0,0,0],[0,0,1,0,0,0,0,0,0],[0,0,1,0,0,0,0,0,0],[0,0,1,0,0,0,0,1,0],[0,0,1,0,1,0,0,0,0],[0,0,1,0,0,0,0,0,0],[0,0,1,0,0,0,0,0,0],[0,0,1,0,0,0,0,0,0],[0,0,1,1,0,0,0,1,0]]11404	11405,11406	[[0,0,0,0,1,0,0,0,0],[0,0,0,0,1,0,0,0,0]]11407	11408,11409	[[0,0,0,0,1,0,0,0,0],[0,0,0,0,1,0,0,0,0]]11410	11411,11412	[[0,0,0,0,1,0,0,0,0],[0,0,0,0,1,0,0,0,0]]11413	7025,3133,2355,11414,11415,11416,3614,822,11417,11418,2342,9109,11419,11420,11421,2353,9400,11422,11423,6165,2332,11424,2059,7438,11425,2434,5178,1163,4818,11426,11427,11428,3126,11429,1623,4569,11430,7983,11431,11432,11433,11434,11435,5170,2435,9382,1197,11436,10664,5154,11437,1552,7927,6386,11438,11439,7935,11440,11047,11441,5175,11442,11443,11444,11061,7355,11445,4910,7895,11446,11447,11448,5167,11449,11450,11451,11452,7367,11453,2915,11068,11454,771,11455,6549,11456,11457,2315,4921,2310,11458,6360,5941,11459,11460,5718,11461,3664,11462,11463,11464,5169,11465	[[1,0,0,0,0,0,0,1,0],[0,1,0,0,0,0,0,1,0],[1,1,1,1,0,0,0,1,0],[0,0,1,0,0,0,0,0,0],[1,0,1,0,0,0,0,0,0],[0,0,1,0,0,0,0,0,0],[1,0,0,0,0,0,0,1,0],[1,0,0,0,0,0,0,1,0],[1,0,0,0,0,0,0,0,0],[1,0,0,0,0,0,0,0,0],[0,0,0,1,0,0,1,0,0],[1,0,0,0,0,0,0,1,0],[0,0,1,0,0,0,0,0,0],[1,0,0,0,0,0,0,0,0],[0,0,1,0,0,0,0,0,0],[1,0,0,0,0,0,0,1,0],[0,0,1,0,0,0,0,0,0],[0,1,1,0,0,0,1,1,0],[0,0,0,1,0,0,0,0,0],[1,0,0,0,0,0,0,0,0],[0,0,0,0,1,0,0,0,0],[0,0,1,0,0,0,0,0,0],[0,0,1,0,0,0,0,0,0],[0,0,1,0,0,0,0,0,0],[0,0,1,0,0,0,0,0,0],[0,0,1,0,0,0,0,0,0],[0,0,1,0,0,0,0,0,0],[0,0,1,0,0,0,0,0,0],[1,0,1,1,0,0,0,0,0],[0,0,1,0,0,0,0,0,0],[0,0,1,0,0,0,0,1,0],[0,0,1,0,0,0,0,0,0],[0,1,0,0,0,0,1,1,0],[0,0,1,0,0,0,0,0,0],[0,0,1,0,0,0,0,0,0],[1,0,0,0,0,0,0,0,0],[1,0,0,0,0,0,0,0,0],[0,1,1,0,0,0,1,1,0],[0,0,1,0,0,0,0,0,0],[0,0,1,0,0,0,0,0,0],[0,0,1,0,0,0,0,1,0],[1,1,1,1,0,0,0,1,0],[0,1,0,0,0,0,0,1,0],[0,0,1,0,0,0,0,0,0],[0,0,1,0,0,0,0,0,0],[0,0,0,0,1,0,0,1,0],[0,1,0,0,0,0,1,1,0],[0,0,1,0,0,0,0,0,0],[0,0,1,0,1,0,0,0,0],[1,0,1,1,0,0,0,0,0],[1,0,1,1,0,0,0,1,0],[1,0,0,0,0,0,0,0,0],[0,0,0,0,1,0,0,0,0],[0,0,1,0,0,0,0,0,0],[0,0,1,0,0,0,0,0,0],[1,0,1,1,0,0,0,1,0],[0,1,1,1,0,0,1,1,0],[0,0,1,0,0,0,0,0,0],[0,0,0,1,0,0,0,0,0],[1,0,1,0,0,0,0,0,0],[0,0,1,0,0,0,0,0,0],[1,0,0,0,0,0,0,0,0],[0,0,1,0,0,0,0,0,0],[0,0,1,0,0,0,0,0,0],[1,1,0,0,0,0,0,0,0],[1,1,1,1,0,0,0,1,0],[1,0,0,0,0,0,0,0,0],[1,1,1,0,1,0,1,1,0],[0,1,0,0,0,0,1,0,0],[0,1,0,0,0,0,1,0,0],[0,1,0,0,0,0,0,0,0],[0,1,1,0,0,0,0,0,0],[1,0,0,1,0,0,0,1,0],[0,1,0,0,0,0,0,0,0],[1,1,0,0,0,0,0,0,0],[1,0,0,0,0,0,0,0,0],[1,0,1,0,0,0,0,0,0],[1,0,0,0,0,0,0,1,0],[1,0,0,0,0,0,0,0,0],[1,0,0,0,0,0,0,0,0],[0,0,0,0,1,0,0,0,0],[0,0,0,0,0,0,0,1,0],[0,0,1,1,0,0,0,1,0],[0,0,1,0,0,0,0,0,0],[0,0,1,0,0,0,0,0,0],[0,0,1,0,0,0,0,0,0],[0,0,1,1,0,0,0,0,0],[0,0,1,0,0,0,0,1,0],[0,0,1,0,0,0,0,0,0],[0,0,0,0,0,0,0,1,0],[0,0,1,0,0,0,0,0,0],[0,0,1,0,0,0,0,0,0],[0,0,1,0,0,0,0,0,0],[0,0,1,0,0,0,0,0,0],[0,0,1,0,0,0,0,0,0],[0,0,0,0,0,0,0,1,0],[0,0,1,0,0,0,0,0,0],[0,0,1,0,0,0,0,0,0],[0,0,1,0,0,0,0,0,0],[0,0,1,0,0,0,0,0,0],[0,0,1,0,0,0,0,0,0],[0,0,1,0,0,0,0,0,0],[0,0,0,0,0,0,0,1,0]]11466	5311,11467,11468,11469,11470,11471,11472,11473,11474,4972,1489,11475,4861,11476,4479,9344,2810,9370,4499,10458,1485,1490,4146	[[0,1,0,0,0,0,0,1,0],[0,1,0,0,0,0,0,0,0],[0,1,0,0,0,0,0,0,0],[0,1,0,0,0,0,0,0,0],[0,1,0,0,0,0,0,0,0],[0,1,0,0,0,0,0,0,0],[0,1,0,0,0,0,0,0,0],[0,1,0,0,0,0,0,0,0],[0,1,0,0,0,0,0,0,0],[0,1,0,0,0,0,0,0,0],[0,1,0,0,1,0,0,0,0],[0,1,0,0,0,0,0,0,0],[0,0,0,0,0,0,0,1,0],[0,0,0,0,0,0,0,1,0],[0,0,0,0,0,0,0,1,0],[0,0,0,0,0,0,0,1,0],[0,0,0,0,0,0,0,1,0],[0,0,0,0,0,0,0,1,0],[0,0,0,0,0,0,0,1,0],[0,1,0,0,0,0,0,1,0],[0,0,0,0,0,0,0,1,0],[0,1,0,0,0,0,0,0,0],[0,0,0,0,0,0,0,1,0]]11477	3443,11177,1126,11176,11478,11178,11479,11480,11481,11482,11483,11484,11485	[[0,0,0,1,0,0,0,0,0],[0,0,0,1,0,0,0,0,0],[0,0,0,1,0,0,0,0,0],[0,0,0,1,0,0,0,0,0],[0,0,0,1,0,0,0,0,0],[0,0,0,1,0,0,0,0,0],[0,0,0,1,0,0,0,0,0],[0,0,0,1,0,0,0,0,0],[0,0,0,1,0,0,0,0,0],[0,0,0,1,0,0,0,0,0],[0,0,0,1,0,0,0,0,0],[0,0,0,1,0,0,0,0,0],[0,0,0,1,0,0,0,0,0]]11486	11487	[[0,0,0,0,1,0,0,0,0]]11488	10162,11489,6759,6762,11490,11491,11492,11493,11494,11495,3968,1297,1312,824,11496,6772,11497,11498,1300,2244,11499,9853,11500,11501,11502,11503,8842,6758,1313,11504,11505	[[0,0,1,0,0,0,0,0,0],[0,0,1,0,0,0,0,0,0],[0,0,1,0,1,0,0,0,0],[0,0,1,0,0,0,0,0,0],[0,0,0,0,1,0,0,0,0],[0,0,1,0,0,0,0,0,0],[0,0,1,0,0,0,0,0,0],[0,0,0,0,1,0,0,0,0],[0,0,1,0,0,0,0,0,0],[0,0,1,1,0,0,0,0,0],[0,0,1,0,0,0,0,0,0],[0,0,1,0,0,0,0,0,0],[0,0,1,0,0,0,0,0,0],[0,0,1,0,0,0,0,0,0],[0,0,1,0,0,0,0,0,0],[0,0,1,0,0,0,0,0,0],[0,0,1,0,0,0,0,0,0],[0,0,1,0,0,0,0,0,0],[0,0,1,0,0,0,0,0,0],[0,0,1,1,0,0,0,0,0],[0,0,1,0,0,0,0,0,0],[0,0,1,0,0,0,0,0,0],[0,0,1,0,0,0,0,0,0],[0,0,1,1,1,0,0,0,0],[0,0,1,0,0,0,0,0,0],[0,0,1,1,0,0,0,0,0],[0,0,1,0,0,0,0,0,0],[0,0,1,0,0,0,0,0,0],[0,0,1,0,0,0,0,0,0],[0,0,1,0,0,0,0,0,0],[0,0,1,0,0,0,0,0,0]]11506	11507,11508,11509,9689	[[0,0,0,0,1,0,0,0,0],[0,0,0,0,1,0,0,0,0],[0,0,0,0,1,0,0,0,0],[0,0,0,0,1,0,0,0,0]]11510	11511,11512,11513,11514,11515,11516,11517,11518,11519,7747,11520,11521,11522,11523,6742,11524,11525,11526,6772,11527,11528,11529,11530,11531,11532,11533,11534,11535,11536,11537,11538	[[0,0,1,0,0,0,0,0,0],[0,0,1,0,0,0,0,0,0],[0,0,1,0,0,0,0,0,0],[0,0,1,0,0,0,0,0,0],[0,0,1,0,0,0,0,0,0],[0,0,1,0,0,0,0,0,0],[0,0,1,0,0,0,0,0,0],[0,0,1,0,0,0,0,0,0],[0,0,1,0,0,0,0,0,0],[0,0,1,0,0,0,0,0,0],[0,0,1,0,0,0,0,0,0],[0,0,1,0,0,0,0,0,0],[0,0,1,0,0,0,0,0,0],[0,0,1,0,0,0,0,0,0],[0,0,1,0,0,0,0,0,0],[0,0,1,0,0,0,0,0,0],[0,0,1,0,0,0,0,0,0],[0,0,1,0,0,0,0,0,0],[0,0,1,0,0,0,0,0,0],[0,0,1,0,0,0,0,0,0],[0,0,1,0,0,0,0,0,0],[0,0,1,0,0,0,0,0,0],[0,0,1,0,0,0,0,0,0],[0,0,1,0,0,0,0,0,0],[0,0,1,0,0,0,0,0,0],[0,0,1,0,0,0,0,0,0],[0,0,1,0,0,0,0,0,0],[0,0,1,0,0,0,0,0,0],[0,0,1,0,0,0,0,0,0],[0,0,0,0,1,0,0,0,0],[0,0,0,0,1,0,0,0,0]]11539	2657	[[0,0,0,0,1,0,0,0,0]]11540	10465,11188,2053	[[0,0,0,1,0,0,0,0,0],[0,0,0,1,0,0,0,0,0],[0,0,0,1,0,0,0,0,0]]11541	11542,11543	[[0,0,0,0,1,0,0,0,0],[0,0,0,0,1,0,0,0,0]]11544	11545	[[0,0,0,0,1,0,0,0,0]]11546	11547,695,724,718,711,11548,707,2486,723,762,709,11549,11550	[[0,0,1,0,0,0,0,0,0],[0,0,1,0,0,0,0,0,1],[0,0,1,0,0,0,0,0,0],[0,0,1,0,0,0,0,0,1],[0,0,1,0,1,0,0,0,1],[0,0,1,0,0,0,0,0,1],[0,0,1,0,0,0,0,0,0],[0,0,1,0,0,0,0,0,0],[0,0,1,0,1,0,0,0,1],[0,0,1,0,0,0,0,0,0],[0,0,1,0,0,0,0,0,1],[0,0,0,0,1,0,0,0,0],[0,0,0,0,1,0,0,0,0]]11551	4828,4805,1925,11552,2520,11553,11554,7782,4985,7747,11555,7778,11556,11557,8323,5485,4989,26,11558,1319,3931,4845,11559,2244,11560,3782,11561,11562,11563,4817,11564,11565,1920,4882,4905,11566,7185	[[0,1,0,0,1,0,1,0,0],[0,1,0,0,0,0,1,0,0],[0,1,0,1,0,0,1,0,0],[0,1,0,0,0,0,1,0,0],[0,1,0,0,0,0,1,0,0],[0,1,0,0,1,0,1,0,0],[0,1,0,1,1,0,1,0,0],[0,1,0,0,1,0,0,0,0],[0,1,0,0,0,0,1,0,0],[0,1,0,1,0,0,0,0,0],[0,0,0,1,0,0,0,0,0],[0,1,0,0,0,0,1,0,0],[0,1,0,0,0,0,1,0,0],[0,1,0,0,0,0,1,0,0],[0,0,0,0,0,0,1,0,0],[0,1,0,0,1,0,1,0,0],[0,1,0,0,0,0,1,0,0],[0,1,0,0,0,0,1,0,0],[0,0,0,0,0,0,1,0,0],[0,1,0,1,0,0,1,0,0],[0,1,0,0,0,0,1,0,0],[0,1,0,0,0,0,1,0,0],[0,0,0,1,0,0,1,0,0],[0,0,0,0,0,0,1,0,0],[0,0,0,1,0,0,0,0,0],[0,0,0,1,0,0,0,0,0],[0,0,0,1,1,0,0,0,0],[0,0,0,1,0,0,1,0,0],[0,1,0,0,0,0,1,0,0],[0,1,0,0,0,0,0,0,0],[0,1,0,0,0,0,0,0,0],[0,1,0,0,0,0,0,0,0],[0,1,0,0,0,0,0,0,0],[0,1,0,0,0,0,0,0,0],[0,1,0,0,0,0,0,0,0],[0,1,0,0,0,0,0,0,0],[0,1,0,0,0,0,0,0,0]]11567	11568	[[0,0,0,0,1,0,0,0,0]]11569	11570,11571,9110	[[0,0,0,0,1,0,0,0,0],[0,0,0,0,1,0,0,0,0],[0,0,1,0,1,0,0,0,0]]11572	11573	[[0,0,0,0,1,0,0,0,0]]11574	4825,11575,11576,1370	[[0,0,0,1,0,0,0,0,0],[0,0,0,1,0,0,0,0,0],[0,0,0,1,0,0,0,0,0],[0,0,0,1,0,0,0,0,0]]11577	11578,11579	[[0,0,0,1,0,0,0,0,0],[0,0,0,1,0,0,0,0,0]]11580	11581,282,2932,7821,7811,3453,7814,4224,3661,3726,11582,8952,4840,3773,11583,356,5433,4235,3007,11584,11585,11586,3732,7830,3723,11587,3749,11588,8859,8846,4523,7844,11589,11590,11591,11592,2565,7808,2922,7807,11593,9229,11594,7557,11595,7558,7561,4524,11596,11597,8845,11598,11599,2909,11600,11601,11602,11603,11604,11605,5944,2148,11606,11607,11608,11609,11610,2915,11611,288,5070,11612,4520,11613	[[0,0,0,1,0,0,0,0,0],[0,0,1,0,0,0,0,0,0],[0,1,0,1,0,0,1,0,0],[0,1,0,0,0,0,1,0,0],[0,0,0,1,0,0,0,0,0],[0,1,1,0,0,0,1,0,0],[0,1,0,0,0,0,1,0,0],[0,1,0,1,1,0,1,0,0],[0,1,1,1,0,0,1,0,0],[0,1,0,0,0,0,1,0,0],[0,0,1,0,0,0,0,0,0],[0,0,1,0,0,0,0,0,0],[0,0,0,1,0,0,0,0,0],[0,0,0,1,0,0,0,0,0],[0,0,0,1,0,0,0,0,0],[0,0,0,1,0,0,0,0,0],[0,0,0,1,0,0,0,0,0],[0,1,1,1,0,0,1,0,0],[0,0,1,1,0,0,0,0,0],[0,0,1,0,0,0,0,0,0],[0,0,1,1,0,0,0,0,0],[0,1,1,0,0,0,0,0,0],[0,1,0,1,0,0,1,0,0],[0,1,0,0,0,0,1,0,0],[0,1,1,0,1,0,1,0,0],[0,0,0,1,0,0,0,0,0],[0,1,0,0,0,0,1,0,0],[0,0,0,1,0,0,0,0,0],[0,1,1,0,0,0,0,0,0],[0,0,1,0,0,0,0,0,0],[0,1,1,0,0,0,0,0,0],[0,1,0,1,0,0,1,0,0],[0,1,1,0,0,0,1,0,0],[0,0,0,1,0,0,0,0,0],[0,0,1,1,0,0,0,0,0],[0,0,1,0,0,0,0,0,0],[0,0,1,0,0,0,0,0,0],[0,0,1,0,0,0,0,0,0],[0,0,0,1,0,0,0,0,0],[0,0,0,1,0,0,0,0,0],[0,1,1,1,0,0,0,0,0],[0,0,0,1,0,0,0,0,0],[0,1,0,0,0,0,0,0,0],[0,1,1,0,0,0,0,0,0],[0,1,1,0,0,0,0,0,0],[0,1,0,0,0,0,0,0,0],[0,1,0,0,0,0,0,0,0],[0,1,0,0,0,0,0,0,0],[0,1,0,0,0,0,0,0,0],[0,1,0,0,0,0,0,0,0],[0,1,1,0,0,0,0,0,0],[0,0,0,1,0,0,0,0,0],[0,0,0,1,0,0,0,0,0],[0,0,0,1,0,0,0,0,0],[0,0,0,1,0,0,0,0,0],[0,0,0,1,0,0,0,0,0],[0,0,1,1,0,0,0,0,0],[0,0,1,0,0,0,0,0,0],[0,0,0,1,0,0,0,0,0],[0,0,1,0,0,0,0,0,0],[0,1,1,1,0,0,0,0,0],[0,0,0,1,0,0,0,0,0],[0,0,0,1,0,0,0,0,0],[0,0,1,1,0,0,0,0,0],[0,0,0,1,0,0,0,0,0],[0,0,0,1,0,0,0,0,0],[0,0,0,1,0,0,0,0,0],[0,0,0,1,0,0,0,0,0],[0,0,0,1,0,0,0,0,0],[0,1,0,0,0,0,1,0,0],[0,0,0,0,0,0,1,0,0],[0,0,1,1,0,0,0,0,0],[0,0,1,0,0,0,0,0,0],[0,1,0,0,0,0,0,0,0]]11614	11615,11616	[[0,0,0,0,1,0,0,0,0],[0,0,0,0,1,0,0,0,0]]11617	6993,11618,11619,11620	[[0,0,0,0,1,0,0,0,0],[0,0,0,0,1,0,0,0,0],[0,0,0,0,1,0,0,0,0],[0,0,0,0,1,0,0,0,0]]11621	11622,1585,8473,10289,11623,11624,11625,2027,11626,3506,11627,4083,5317,9126,11628,11629,10100,11630,11631,11632,11633,11634,11635,11636,11637,5974,11638,11639,6000	[[0,0,1,0,0,0,0,0,0],[0,0,1,0,0,0,0,0,0],[0,0,1,0,0,0,0,0,0],[0,0,1,0,0,0,0,0,0],[0,0,1,0,0,0,0,0,0],[0,0,1,0,0,0,0,0,0],[0,0,1,0,0,0,0,0,0],[0,0,1,0,0,0,0,0,0],[0,0,1,0,0,0,0,0,0],[0,0,1,0,0,0,0,0,0],[0,0,1,0,0,0,0,0,0],[0,0,1,0,0,0,0,0,0],[0,0,1,0,0,0,0,0,0],[0,0,1,0,0,0,0,0,0],[0,0,1,0,0,0,0,0,0],[0,0,1,0,0,0,0,0,0],[0,0,1,0,0,0,0,0,0],[0,0,1,0,0,0,0,0,0],[0,0,1,0,0,0,0,0,0],[0,0,1,0,0,0,0,0,0],[0,0,1,0,1,0,0,0,0],[0,0,1,0,0,0,0,0,0],[0,0,1,0,0,0,0,0,0],[0,0,1,0,0,0,0,0,0],[0,0,1,0,0,0,0,0,0],[0,0,1,0,0,0,0,0,0],[0,0,1,0,0,0,0,0,0],[0,0,1,0,0,0,0,0,0],[0,0,1,0,0,0,0,0,0]]11640	11641,11642,11643,11644	[[0,0,0,1,0,0,0,0,0],[0,0,0,1,0,0,0,0,0],[0,0,0,0,1,0,0,0,0],[0,0,0,1,0,0,0,0,0]]11645	11646,11647,11648,11649,11650,11651,11652,11653,11654,3240,11655,9266,11656,11657,11658,11659,11660,11661,11662,11663,11664	[[1,0,1,0,0,0,0,0,0],[1,0,1,0,1,0,0,0,0],[1,0,0,0,0,0,0,0,0],[1,0,0,0,0,0,0,0,0],[1,0,1,0,1,0,0,0,0],[1,0,0,0,0,0,0,0,0],[1,0,1,0,0,0,0,0,0],[0,0,1,0,0,0,0,0,0],[0,0,1,0,0,0,0,0,0],[1,0,1,0,0,0,0,0,0],[1,0,1,0,0,0,0,0,0],[0,0,1,0,0,0,0,0,0],[1,0,1,0,0,0,0,0,0],[1,0,0,0,0,0,0,0,0],[1,0,1,0,0,0,0,0,0],[1,0,1,0,0,0,0,0,0],[1,0,0,0,0,0,0,0,0],[1,0,1,0,0,0,0,0,0],[1,0,0,0,0,0,0,0,0],[1,0,0,0,0,0,0,0,0],[1,0,0,0,0,0,0,0,0]]11665	11666,11667,474,11668,11669,11670,473,11671,11672,11673,11674,7441,8163,8167,11675,11676,11677,11678,11679,11680,5168,11681,11682,11683	[[0,0,1,0,0,0,0,0,0],[0,1,0,0,0,0,0,0,0],[0,1,1,0,0,0,0,1,0],[0,1,0,0,0,0,0,0,0],[0,1,0,0,0,0,0,0,0],[0,1,0,0,0,0,0,1,0],[0,1,1,1,1,1,0,1,0],[0,0,0,1,0,0,0,1,0],[0,0,0,0,0,0,0,1,0],[0,0,1,0,0,0,0,1,0],[0,0,0,0,0,0,0,1,0],[0,0,0,0,0,1,0,1,0],[0,0,0,0,0,0,0,1,0],[0,0,0,0,0,1,0,0,0],[0,1,1,0,0,0,0,1,0],[0,0,1,0,0,1,0,0,0],[0,0,0,0,0,0,0,1,0],[0,0,0,0,0,1,0,1,0],[0,0,0,0,0,1,0,1,0],[0,0,0,0,0,0,0,1,0],[0,0,0,0,0,0,0,1,0],[0,1,1,0,0,0,0,0,0],[0,0,0,0,0,0,0,1,0],[0,0,0,0,0,1,0,0,0]]11684	5278,11685,5289,11686	[[0,0,1,0,0,0,0,0,0],[0,0,1,0,0,0,0,0,0],[0,0,1,0,0,0,0,0,0],[0,0,1,0,0,0,0,0,0]]11687	3925,11558,11688,11689,3753,3931,7135,4892,5432,1319,2239,1305,4913,11690,11691,7784,11692,6603,4845,7113,132,11693,11694,11695,3686,7014,11696,2891,11697,3946,11698,7189,3929,11699,11700,11562,11701,11702,7747,11703,10685,8323,11704,4756	[[0,1,0,0,0,0,1,0,0],[0,0,0,0,0,0,0,1,0],[0,0,1,0,0,0,0,0,0],[0,1,0,0,0,0,1,0,0],[0,1,0,0,0,0,1,0,0],[0,1,1,0,0,0,1,0,0],[0,1,0,0,0,0,0,0,0],[0,1,1,0,1,0,1,1,0],[0,0,0,0,1,0,0,0,0],[0,1,1,0,0,0,1,0,0],[0,1,0,0,1,0,1,1,0],[0,1,0,0,0,0,1,0,0],[0,1,1,0,0,0,1,1,0],[0,1,0,0,1,0,1,1,0],[0,1,0,0,0,0,0,0,0],[0,1,1,0,0,0,0,0,0],[0,0,0,0,0,0,0,1,0],[0,1,1,0,0,0,1,0,0],[0,1,0,0,0,0,1,0,0],[0,1,0,0,0,0,1,0,0],[0,1,0,0,0,0,1,0,0],[0,1,0,0,0,0,1,0,0],[0,1,0,0,0,0,0,0,0],[0,1,0,0,0,0,1,0,0],[0,1,0,0,0,0,1,0,0],[0,0,1,0,0,0,0,0,0],[0,0,0,1,0,0,0,0,0],[0,1,0,0,0,0,0,0,0],[0,0,0,0,0,0,1,0,0],[0,1,0,0,0,0,1,0,0],[0,0,0,0,0,0,0,1,0],[0,0,1,1,0,0,0,1,0],[0,1,1,0,0,0,1,0,0],[0,0,1,0,0,0,0,0,0],[0,0,0,1,0,0,0,0,0],[0,1,1,1,0,0,1,1,0],[0,0,0,1,0,0,0,1,0],[0,0,1,0,1,0,0,0,0],[0,0,1,0,0,0,0,0,0],[0,0,1,1,0,0,0,1,0],[0,0,0,0,1,0,0,0,0],[0,0,1,1,1,0,0,1,0],[0,0,0,0,1,0,0,0,0],[0,0,0,1,0,0,0,0,0]]11705	11706,11707,7962	[[0,0,0,1,0,0,0,0,0],[0,0,0,1,0,0,0,0,0],[0,0,0,1,0,0,0,0,0]]11708	10103,1240	[[0,0,0,0,1,0,0,0,0],[0,0,0,0,1,0,0,0,0]]11709	10279,9332,1058,1072,1067,4032,9889,1814,550,9367,4635,9170,4682,8915,8927,9102,11710,10286,8896,554,11711,5398,11712,9327,8790,637,10225,11713,1099,9366,7483,11714,435,9960,9571,644,1197,4659,5519,4629,8917,9191,11715,11716,10288,5400,11717,9566,9371,5406,8891,10291,2983,11718,11719,1060,11720,11721,8882,8908,6906,9977,1048,10227,10221,11722,5510,10233,11723,11724,9192,8889,11725,8907,8888,11726,11727,1
[truncated: 1,200,000 more chars]
