# Supplementary material for: Quantifying the Impact and Extent of Undocumented Biomedical Synonymy
Source: PLoS Comput Biol. 2014 Sep 25;10(9):e1003799. doi: 10.1371/journal.pcbi.1003799 (PMC4177665; doi:10.1371/journal.pcbi.1003799)
Supplement: Dataset S2 — The Diseases and Syndromes synonym dataset. The format of this file is identical to that of Dataset S1. See Supporting Information Text S1 for the processing procedures that resulted in this dataset. (ZIP) [file pcbi.1003799.s002.zip › SupportingDataset_S2.rtf]

Vocab Key: SNOMEDCT,MSH,COSTAR,OMIM,ICD9CM,PDQ,MTH,CHV,CST,CSP,DXP,NCI,NDFRT,MTHICD9Concept ID#	Variant List	AnnotationsC0220597	adult hodgkin lymphoma,adult hodgkins disease,adult hd,adult hodgkins lymphoma lymphoma	[[0,1,0,0,0,1,0,1,0,0,0,1,0,0],[0,0,0,0,0,1,0,1,0,0,0,1,0,0],[0,0,0,0,0,1,0,0,0,0,0,1,0,0],[0,0,0,0,0,1,0,0,0,0,0,0,0,0]]C0520719	spinopontine atrophy,spinopontine degeneration	[[1,1,0,0,0,0,0,0,0,0,0,0,0,0],[1,0,0,0,0,0,0,0,0,0,0,0,0,0]]C0520714	pallidonigroluysian degeneration	[[1,0,0,0,0,0,0,0,0,0,0,0,0,0]]C0520715	pallidonigrospinal degeneration	[[1,0,0,0,0,0,0,0,0,0,0,0,0,0]]C0520716	pallidopontonigral degeneration,ppnd	[[1,0,0,1,0,0,0,0,0,0,0,0,0,0],[0,0,0,1,0,0,0,0,0,0,0,0,0,0]]C0520717	primary progressive cerebellar degeneration,holmes disease	[[1,0,0,0,0,0,0,1,0,0,0,0,0,0],[1,0,0,0,0,0,0,1,0,0,0,0,0,0]]C0520711	opticocochleodentate degeneration	[[1,0,0,1,0,0,0,0,0,0,0,0,0,0]]C3151201	multisystemic smooth muscle dysfunction syndrome,and vasculopathy thoracic aortic aneurysm with patent ductus arteriosus congenital mydriasis	[[0,0,0,1,0,0,0,0,0,0,0,0,0,0],[0,0,0,1,0,0,0,0,0,0,0,0,0,0]]C0877557	periarthritis scapulohumeralis	[[0,0,0,0,0,0,1,0,0,0,0,0,0,0]]C3151203	quantitative trait locus 5 serum level of adiponectin,adipqtl5	[[0,0,0,1,0,0,0,0,0,0,0,0,0,0],[0,0,0,1,0,0,0,0,0,0,0,0,0,0]]C1838604	1 childhood absence epilepsy,eca1,1 susceptibility to childhood absence epilepsy	[[0,0,0,0,0,0,1,0,0,0,0,0,0,0],[0,0,0,1,0,0,0,0,0,0,0,0,0,0],[0,0,0,1,0,0,0,0,0,0,0,0,0,0]]C0730505	distance exotropia	[[1,0,0,0,0,0,0,0,0,0,0,0,0,0]]C1838606	atrioventricular septal defect with blepharophimosis and anal and radial defects	[[0,0,0,1,0,0,0,0,0,0,0,0,0,0]]C0334469	sclerosing liposarcoma	[[1,0,0,0,0,0,0,0,0,0,0,1,0,0]]C2931409	flavimonas oryzihabitans bacteremia	[[0,1,0,0,0,0,0,0,0,0,0,0,0,0]]C1838603	retinitis pigmentosa 14,rp14,tulp1 related juvenile retinitis pigmentosa	[[0,0,0,1,0,0,0,0,0,0,0,0,0,0],[0,0,0,1,0,0,0,0,0,0,0,0,0,0],[0,0,0,1,0,0,0,0,0,0,0,0,0,0]]C0334465	myxomatous neoplasm	[[1,0,0,0,0,0,0,0,0,0,0,0,0,0]]C0334464	pigmented dermatofibrosarcoma protuberans,bednars tumor,bednar tumour,m pigmented dermatofibrosarcoma protuberans,pigmented dermatofibrosarcoma,pigmented storiform neurofibroma,pigmented dermatofibrosarcoma protuberans of skin	[[0,1,0,0,0,0,0,0,0,0,0,1,0,0],[1,1,0,0,0,0,0,0,0,0,0,1,0,0],[1,0,0,0,0,0,0,0,0,0,0,0,0,0],[1,0,0,0,0,0,0,0,0,0,0,0,0,0],[1,0,0,0,0,0,0,0,0,0,0,0,0,0],[1,0,0,0,0,0,0,0,0,0,0,0,0,0],[1,0,0,0,0,0,0,0,0,0,0,0,0,0]]C0334467	fibrolipoma	[[1,0,0,0,0,0,0,1,0,0,0,0,0,0]]C1710177	thyroid gland squamous cell carcinoma	[[0,0,0,0,0,0,0,0,0,0,0,1,0,0]]C2931400	familial lipomyelomeningocele	[[0,1,0,0,0,0,0,0,0,0,0,0,0,0]]C0334460	elastofibroma	[[0,0,0,0,0,0,0,1,0,0,0,1,0,0]]C0334463	malignant fibrous histiocytoma,malignant fibroxanthoma,fibroxanthosarcoma,mfh,malignant fibrous histiocytoma of soft tissue and bone,malignant fibrohistiocytic tumors,malignant fibrous cytoma	[[1,1,0,0,0,0,0,1,0,0,0,0,1,0],[1,0,0,0,0,0,0,0,0,0,0,1,0,0],[0,0,0,0,0,0,0,0,0,0,0,1,0,0],[0,0,0,0,0,0,0,0,0,0,0,1,0,0],[0,0,0,0,0,0,0,0,0,0,0,1,0,0],[0,1,0,0,0,0,0,0,0,0,0,0,1,0],[0,0,0,0,0,0,0,0,0,0,0,1,0,0]]C2931403	minimal pigment type albinism,minimal pigment type oculocutaneous albinism	[[0,1,0,0,0,0,0,0,0,0,0,0,0,0],[0,1,0,0,0,0,0,0,0,0,0,0,0,0]]C2675066	lymphedema distichiasis syndrome with renal disease and diabetes mellitus	[[0,0,0,1,0,0,0,0,0,0,0,0,0,0]]C1709553	pituitary gland neoplasm	[[0,0,0,0,0,0,0,0,0,0,0,1,0,0]]C0346448	neoplasm of upper limb,tumor of upper limb,tumour of upper limb	[[1,0,0,0,0,0,0,0,0,0,0,0,0,0],[1,0,0,0,0,0,0,0,0,0,0,0,0,0],[1,0,0,0,0,0,0,0,0,0,0,0,0,0]]C1334385	leiomyosarcoma of the central nervous system,cns leiomyosarcoma	[[0,0,0,0,0,0,0,0,0,0,0,1,0,0],[0,0,0,0,0,0,0,0,0,0,0,1,0,0]]C0346444	benign neoplasm of abdomen,benign tumor of abdomen,benign tumour of abdomen	[[1,0,0,0,0,0,0,0,0,0,0,0,0,0],[1,0,0,0,0,0,0,0,0,0,0,0,0,0],[1,0,0,0,0,0,0,0,0,0,0,0,0,0]]C1334386	leptomeningeal melanoma,meningeal melanoma	[[0,0,0,1,0,0,0,0,0,0,0,1,0,0],[0,0,0,0,0,0,0,0,0,0,0,1,0,0]]C1334380	lateral ventricle meningioma	[[0,0,0,0,0,0,0,0,0,0,0,1,0,0]]C0346440	benign neoplasm of thorax,benign tumor of thorax,benign tumour of thorax,benign thoracic neoplasm,benign thoracic tumor	[[1,0,0,0,0,0,0,0,0,0,0,1,0,0],[1,0,0,0,0,0,0,0,0,0,0,1,0,0],[1,0,0,0,0,0,0,0,0,0,0,0,0,0],[0,0,0,0,0,0,0,0,0,0,0,1,0,1],[0,0,0,0,0,0,0,0,0,0,0,1,0,0]]C1334382	leiomyoma of the central nervous system,leiomyoma of the cns	[[0,0,0,0,0,0,0,0,0,0,0,1,0,0],[0,0,0,0,0,0,0,0,0,0,0,1,0,0]]C0271051	macular retinal edema,macular retinal oedema,macular edema,macular oedema	[[1,0,0,0,0,0,0,0,0,0,0,0,0,1],[1,0,0,0,0,0,0,0,0,0,0,0,0,0],[1,1,0,0,0,0,1,1,0,1,0,1,0,0],[1,0,0,0,0,0,0,1,0,0,0,0,0,0]]C0271055	rhegmatogenous retinal detachment,retinal detachment with break	[[0,0,0,0,0,0,0,1,0,0,0,0,0,1],[1,0,0,0,0,0,0,0,0,0,0,0,0,0]]C0271056	recent total retinal detachment	[[1,0,0,0,0,0,0,0,0,0,0,0,0,0]]C0271057	recent subtotal retinal detachment	[[1,0,0,0,0,0,0,0,0,0,0,0,0,0]]C0271058	old total retinal detachment	[[1,0,0,0,0,0,0,0,0,0,0,0,0,0]]C0271059	old subtotal retinal detachment	[[1,0,0,0,0,0,0,0,0,0,0,0,0,0]]C1881801	metastatic signet ring cell carcinoma	[[1,0,0,0,0,0,0,0,0,0,0,1,0,0]]C1845902	fg syndrome 2,fgs2	[[0,1,0,1,0,0,0,0,0,0,0,0,0,0],[0,0,0,1,0,0,0,0,0,0,0,0,0,0]]C0342562	gastrointestinal hormone secreting endocrine tumor,gastrointestinal hormone secreting endocrine tumour	[[1,0,0,0,0,0,0,0,0,0,0,0,0,0],[1,0,0,0,0,0,0,0,0,0,0,0,0,0]]C0472342	myelopathy due to hematomyelia,myelopathy due to haematomyelia	[[1,0,0,0,0,0,0,0,0,0,0,0,0,0],[1,0,0,0,0,0,0,0,0,0,0,0,0,0]]C0342569	carcinoid crisis	[[1,0,0,0,0,0,0,0,0,0,0,0,0,0]]C0472341	myelopathy due to edema of spinal cord,myelopathy due to oedema of spinal cord	[[1,0,0,0,0,0,0,0,0,0,0,0,0,0],[1,0,0,0,0,0,0,0,0,0,0,0,0,0]]C1709361	outer hair sheath and infundibulum neoplasm,outer hair sheath and infundibulum tumor	[[0,0,0,0,0,0,0,0,0,0,0,1,0,0],[0,0,0,0,0,0,0,0,0,0,0,1,0,0]]C0472340	postoperative hematoma formation,postoperative haematoma formation	[[1,0,0,0,0,0,0,0,0,0,0,0,0,0],[1,0,0,0,0,0,0,0,0,0,0,0,0,0]]C1963763	failed back surgery syndrome	[[0,0,0,0,0,0,0,0,0,0,0,0,1,0]]C2674081	7 variation in skin hair eye pigmentation,dark light skin skin hair eye pigmentation 7,blond brown hair skin hair eye pigmentation 7,shep7	[[0,0,0,1,0,0,0,0,0,0,0,0,0,0],[0,0,0,1,0,0,0,0,0,0,0,0,0,0],[0,0,0,1,0,0,0,0,0,0,0,0,0,0],[0,0,0,1,0,0,0,0,0,0,0,0,0,0]]C1959600	obscure african cardiomyopathy,endomyocardial fibrosis,obscure cardiomyopathy of africa,african endomyocardial fibrosis,beckers disease,idiopathic mural endomyocardial disease	[[1,0,0,0,0,0,0,0,0,0,0,0,0,0],[0,0,0,0,0,0,0,1,0,0,0,0,0,0],[0,0,0,0,1,0,0,1,0,0,0,0,0,0],[1,0,0,0,0,0,0,0,0,0,0,0,0,0],[0,0,0,0,0,0,0,1,0,0,0,0,0,0],[0,0,0,0,0,0,0,0,0,0,0,0,0,1]]C3164319	papilloma of lip	[[1,0,0,0,0,0,0,0,0,0,0,0,0,0]]C1333372	eccrine sweat gland hamartoma	[[0,0,0,0,0,0,0,0,0,0,0,1,0,0]]C1847319	paraganglioma and gastric stromal sarcoma,gist,carney stratakis syndrome,paraganglioma and gastrointestinal stromal tumor,carney stratakis dyad,carney dyad	[[0,0,0,1,0,0,0,0,0,0,0,0,0,0],[0,0,0,1,0,0,0,0,0,0,0,0,0,0],[0,0,0,1,0,0,0,0,0,0,0,1,0,0],[0,0,0,1,0,0,0,0,0,0,0,0,0,0],[0,0,0,0,0,0,0,0,0,0,0,1,0,0],[0,0,0,0,0,0,0,0,0,0,0,1,0,0]]C0472345	myxedema encephalopathy,myxoedema encephalopathy	[[1,0,0,0,0,0,0,0,0,0,0,0,0,0],[1,0,0,0,0,0,0,0,0,0,0,0,0,0]]C0031190	persistent fetal circulation syndrome,persistent fetal circulation,persistent pulmonary hypertension of newborn,pfc persistent fetal circulation,pphn persistent pulmonary hypertension in newborn,of the newborn familial persistent pulmonary hypertension,acdmpv,alveolar capillary dysplasia with misalignment of pulmonary veins	[[1,1,0,0,0,0,0,0,0,0,0,0,0,0],[0,0,0,0,1,0,0,0,0,0,1,0,1,0],[1,0,0,0,0,0,0,0,0,1,0,0,1,0],[1,0,0,0,0,0,0,0,0,0,0,0,0,0],[1,0,0,0,0,0,0,0,0,0,0,0,0,0],[0,0,0,1,0,0,0,0,0,0,0,0,1,0],[0,0,0,1,0,0,0,0,0,0,0,0,0,0],[0,0,0,1,0,0,0,0,0,0,0,0,0,0]]C2676023	autosomal dominant hypercarotenemia and vitamin a deficiency	[[0,0,0,1,0,0,0,0,0,0,0,0,0,0]]C1306781	congenital disease of spinal cord	[[0,0,0,0,0,0,0,0,0,0,0,0,0,1]]C1306783	pterygoarthromyodysplasia syndrome	[[0,0,0,0,0,0,1,0,0,0,0,0,0,0]]C0678202	regional enteritis	[[0,0,1,0,1,0,0,0,0,0,1,1,0,1]]C0678200	medulla	[[0,0,0,0,0,0,0,0,1,0,0,0,0,0]]C0678201	terminal ileitis,ileitis	[[1,0,0,0,0,0,0,1,0,0,0,0,0,1],[0,0,0,0,0,0,0,0,0,1,0,0,0,0]]C0564706	inner quadrant carcinoma of breast upper	[[1,0,0,0,0,0,0,0,0,0,0,0,0,0]]C0030824	allergy to penicillin,pcn allergy	[[1,0,1,0,0,0,0,1,0,0,0,1,0,0],[0,0,0,0,0,0,0,1,0,0,0,0,0,0]]C0375311	phlebitis and thrombophlebitis of superficial veins of upper extremities	[[0,0,0,0,1,0,0,0,0,0,0,0,0,0]]C0375312	phlebitis and thrombophlebitis of deep veins of upper extremities	[[0,0,0,0,1,0,0,0,0,0,0,0,0,0]]C0375314	iatrogenic hypotension	[[0,0,0,0,1,0,0,0,0,0,0,0,0,0]]C0865245	progressive anemia	[[0,0,0,0,0,0,0,1,0,0,0,0,0,1]]C1845604	recurrent bacterial meningitis	[[0,0,0,1,0,0,0,0,0,0,0,0,0,0]]C0686063	secondary malignant neoplasm of upper third of esophagus,secondary malignant neoplasm of upper third of oesophagus,metastatic malignant neoplasm to upper third of esophagus,metastatic malignant neoplasm to upper third of oesophagus	[[1,0,0,0,0,0,0,0,0,0,0,0,0,0],[1,0,0,0,0,0,0,0,0,0,0,0,0,0],[1,0,0,0,0,0,0,0,0,0,0,0,0,0],[1,0,0,0,0,0,0,0,0,0,0,0,0,0]]C0865243	not due to blood loss normocytic anemia	[[0,0,0,0,0,0,0,0,0,0,0,0,0,1]]C1444838	chronic arthritis of juvenile onset	[[1,0,0,0,0,0,0,0,0,0,0,0,0,0]]C0268148	muscular form glycogen storage disease	[[1,0,0,0,0,0,0,0,0,0,0,0,0,0]]C0268149	glycogen storage disease type x,muscle phosphoglycerate mutase deficiency,glycogenosis due to inactive phosphorylase and kinase,gsd x,pgamm deficiency,myopathy due to phosphoglycerate mutase deficiency,gsd10,glycogen storage disease x,human muscle phosphoglycerate mutase deficiency,dimauro disease	[[1,0,0,0,0,0,0,0,0,0,0,0,0,0],[1,0,0,1,0,0,0,0,0,0,0,0,0,0],[1,0,0,0,0,0,0,0,0,0,0,0,0,0],[0,0,0,1,0,0,0,0,0,0,0,0,0,0],[0,1,0,1,0,0,0,0,0,0,0,0,0,0],[0,1,0,1,0,0,0,0,0,0,0,0,0,0],[0,0,0,1,0,0,0,0,0,0,0,0,0,0],[0,0,0,1,0,0,0,0,0,0,0,0,0,0],[0,1,0,0,0,0,0,0,0,0,0,0,0,0],[0,1,0,0,0,0,0,0,0,0,0,0,0,0]]C0341141	nonspecific esophageal motility disorder,nonspecific oesophageal motility disorder	[[1,0,0,0,0,0,0,0,0,0,0,0,0,0],[1,0,0,0,0,0,0,0,0,0,0,0,0,0]]C0341146	post vagotomy dysphagia	[[1,0,0,0,0,0,0,0,0,0,0,0,0,0]]C3150859	forsythe wakeling syndrome,fws,microcephaly and growth retardation with childhood onset nephrotic syndrome and thrombocytopenia	[[0,0,0,1,0,0,0,0,0,0,0,0,0,0],[0,0,0,1,0,0,0,0,0,0,0,0,0,0],[0,0,0,1,0,0,0,0,0,0,0,0,0,0]]C0341144	cricopharyngeal bar	[[1,0,0,0,0,0,0,0,0,0,0,0,0,0]]C0341145	palatoesophageal incoordination,palato esophageal incoordination,palato oesophageal incoordination	[[1,0,0,0,0,0,0,0,0,0,0,0,0,0],[1,0,0,0,0,0,0,0,0,0,0,0,0,0],[1,0,0,0,0,0,0,0,0,0,0,0,0,0]]C0268140	group f xeroderma pigmentosum,complementation group f xeroderma pigmentosum,xp f,xpf,xeroderma pigmentosum vi,group f xp,xp6	[[1,0,0,0,0,0,0,0,0,0,0,0,0,0],[0,0,0,1,0,0,0,0,0,0,0,1,0,0],[0,0,0,0,0,0,0,0,0,0,0,1,0,0],[0,0,0,1,0,0,0,0,0,0,0,0,0,0],[0,0,0,1,0,0,0,0,0,0,0,0,0,0],[0,0,0,1,0,0,0,0,0,0,0,0,0,0],[0,0,0,1,0,0,0,0,0,0,0,0,0,0]]C0268141	group g xeroderma pigmentosum,complementation group g xeroderma pigmentosum,xp g,xpg,xpgc,xp7,xeroderma pigmentosum vii,group g xp	[[1,0,0,0,0,0,0,0,0,0,0,0,0,0],[0,0,0,1,0,0,0,0,0,0,0,1,0,0],[0,0,0,0,0,0,0,0,0,0,0,1,0,0],[0,0,0,1,0,0,0,0,0,0,0,0,0,0],[0,0,0,1,0,0,0,0,0,0,0,0,0,0],[0,0,0,1,0,0,0,0,0,0,0,0,0,0],[0,0,0,1,0,0,0,0,0,0,0,0,0,0],[0,0,0,1,0,0,0,0,0,0,0,0,0,0]]C0341148	mid esophageal diverticulum,mid oesophageal diverticulum	[[1,0,0,0,0,0,0,0,0,0,0,0,0,0],[1,0,0,0,0,0,0,0,0,0,0,0,0,0]]C0268143	disorder of central nervous system due to xeroderma pigmentosum,neurologic xeroderma pigmentosum	[[1,0,0,0,0,0,0,0,0,0,0,0,0,0],[1,0,0,0,0,0,0,0,0,0,0,0,0,0]]C0268144	non neurologic xeroderma pigmentosum	[[1,0,0,0,0,0,0,0,0,0,0,0,0,0]]C0268145	hepatic form glycogen storage disease	[[1,0,0,0,0,0,0,0,0,0,0,0,0,0]]C0268146	glucose 6 phosphate transport defect,glycogen storage disease ib,glycogen storage disease type ib,gsd1b	[[1,0,0,1,0,0,0,0,0,0,0,0,0,0],[0,0,0,1,0,0,0,0,0,0,0,0,0,0],[1,0,0,0,0,0,0,0,0,0,0,0,0,0],[0,0,0,1,0,0,0,0,0,0,0,0,0,0]]C0268147	type ix glycogen storage disease,gsd ix,glycogen phosphorylase kinase deficiency,hepatic phosphorylase kinase deficiency,phosphorylase kinase deficiency of liver,glycogen storage disease viii,glycogenosis viiia,hepatic glycogen phosphorylase kinase deficiency,phk hepatic phosphorylase kinase deficiency	[[1,0,0,0,0,0,0,0,0,0,0,0,0,0],[1,0,0,0,0,0,0,0,0,0,0,0,0,0],[1,0,0,0,0,0,0,0,0,0,0,0,0,0],[1,0,0,0,0,0,0,0,0,1,0,0,0,0],[1,0,0,0,0,0,0,0,0,0,0,0,0,0],[1,0,0,0,0,0,0,0,0,0,0,0,0,0],[1,0,0,0,0,0,0,0,0,0,0,0,0,0],[1,0,0,0,0,0,0,0,0,0,0,0,0,0],[1,0,0,0,0,0,0,0,0,0,0,0,0,0]]C1690443	chronic iridocyclitis due to another disorder	[[1,0,0,0,0,0,0,0,0,0,0,0,0,0]]C1690442	acute myocarditis associated with another disorder	[[1,0,0,0,0,0,0,0,0,0,0,0,0,0]]C1856241	hair defect with photosensitivity and mental retardation,and nonprogressive mental retardation broken eyebrows and eyelashes photosensitivity kinky hair,calderon gonzalez cantu syndrome	[[0,1,0,1,0,0,0,0,0,0,0,0,0,0],[0,1,0,0,0,0,0,0,0,0,0,0,0,0],[0,1,0,0,0,0,0,0,0,0,0,0,0,0]]C2585960	heterozygous protein c deficiency	[[1,0,0,0,0,0,0,0,0,0,0,0,0,0]]C2931404	albrights hereditary osteodystrophy,aho albright hereditary osteodystrophy	[[0,1,0,0,0,0,0,0,0,1,1,0,0,0],[1,0,0,0,0,0,0,0,0,0,0,0,0,0]]C0684718	neoplasm of uncertain behavior of soft tissues of axilla,neoplasm of uncertain behaviour of soft tissues of axilla	[[1,0,0,0,0,0,0,0,0,0,0,0,0,0],[1,0,0,0,0,0,0,0,0,0,0,0,0,0]]C2931405	aloi tomasini isaia syndrome,abnormal bone mineralization anodontia basal cell nevus	[[0,1,0,0,0,0,0,0,0,0,0,0,0,0],[0,1,0,0,0,0,0,0,0,0,0,0,0,0]]C0865249	fibrin stabilizing deficiency	[[0,0,0,0,0,0,0,0,0,0,0,0,0,1]]C2931406	alopecia hypogonadism extrapyramidal disorder,progressive extrapyramidal disorder with primary hypogonadism and alopecia	[[0,1,0,0,0,0,0,0,0,0,0,0,0,0],[0,1,0,0,0,0,0,0,0,0,0,0,0,0]]C1300256	type 1 thanatophoric dysplasia	[[1,0,0,0,0,0,0,0,0,0,0,0,0,0]]C1300257	type 2 thanatophoric dysplasia,cloverleaf skull with thanatophoric dwarfism,thanatophoric dysplasia with straight femurs and cloverleaf skull,thanatophoric dysplasia with kleeblattschaedel,type ii thanatophoric dysplasia,td2	[[1,0,0,0,0,0,0,0,0,0,0,0,0,0],[0,1,0,1,0,0,0,0,0,0,0,0,0,0],[0,0,0,1,0,0,0,0,0,0,0,0,0,0],[0,1,0,1,0,0,0,0,0,0,0,0,0,0],[0,0,0,1,0,0,0,0,0,0,0,0,0,0],[0,0,0,1,0,0,0,0,0,0,0,0,0,0]]C1274690	hypertrichosis in hypothyroidism	[[1,0,0,0,0,0,0,0,0,0,0,0,0,0]]C0265676	lumbosacral prespondylolisthesis,prespondylolisthesis	[[1,0,0,0,0,0,0,0,0,0,0,0,0,1],[0,0,0,0,0,0,0,0,0,0,0,0,0,1]]C0265671	congenital discoid meniscus	[[1,0,0,0,0,0,0,0,0,0,0,0,0,1]]C0265670	rudimentary patella	[[0,0,0,0,0,0,0,0,0,0,0,0,0,1]]C0265672	straight back syndrome	[[1,0,1,0,0,0,0,0,0,0,0,0,0,0]]C2677576	recombination rate quantitative trait locus 1,rrqtl1	[[0,0,0,1,0,0,0,0,0,0,0,0,0,0],[0,0,0,1,0,0,0,0,0,0,0,0,0,0]]C1306162	kidney replacement disorder,kidney replacement	[[0,0,0,0,0,0,1,0,0,0,0,0,0,0],[1,0,0,0,0,0,0,1,0,0,0,0,0,0]]C1335514	prostate myeloid sarcoma,prostatic chloroma,prostate extramedullary myeloid neoplasm,prostate extramedullary myeloid tumor	[[0,0,0,0,0,0,0,0,0,0,0,1,0,0],[0,0,0,0,0,0,0,0,0,0,0,1,0,0],[0,0,0,0,0,0,0,0,0,0,0,1,0,0],[0,0,0,0,0,0,0,0,0,0,0,1,0,0]]C0700045	middle ear deafness,deafness middle ear type	[[0,0,0,0,0,0,1,0,0,0,1,0,0,0],[0,0,0,0,0,0,0,0,1,0,0,0,0,0]]C0263143	cellulitis of neck	[[1,0,0,0,0,0,0,1,0,0,0,0,0,0]]C1333095	colon mantle cell lymphoma	[[0,0,0,0,0,0,0,0,0,0,0,1,0,0]]C1336839	type 1 papillary renal cell carcinoma	[[0,0,0,0,0,0,0,0,0,0,0,1,0,0]]C0263142	cellulitis of temple region	[[1,0,0,0,0,0,0,0,0,0,0,0,0,0]]C1291264	glyceraldehyde 3 phosphate dehydrogenase deficiency,deficiency of triosephosphate dehydrogenase	[[1,1,0,0,0,0,0,0,0,0,0,0,0,0],[1,0,0,0,0,0,0,0,0,0,0,0,0,0]]C3151293	1hh dilated cardiomyopathy,cmd1hh	[[0,0,0,1,0,0,0,0,0,0,0,0,0,0],[0,0,0,1,0,0,0,0,0,0,0,0,0,0]]C1854416	macrocephaly autism syndrome	[[0,0,0,1,0,0,0,0,0,0,0,0,0,0]]C1854414	autosomal recessive 10 deafness,dfnb10	[[0,0,0,1,0,0,0,0,0,0,0,0,0,0],[0,0,0,1,0,0,0,0,0,0,0,0,0,0]]C0744943	hip plate	[[0,0,0,0,0,0,0,1,0,0,0,0,0,0]]C0744945	hip prosthesis infection	[[0,0,0,0,0,0,0,1,0,0,0,0,0,0]]C0744944	hip problem congenital	[[0,0,0,0,0,0,0,1,0,0,0,0,0,0]]C1370740	adrenal carcinoma	[[0,0,1,1,0,0,0,0,1,0,0,0,0,0]]C0686614	secondary malignant neoplasm of lingual tonsil,metastatic malignant neoplasm to lingual tonsil	[[1,0,0,0,0,0,0,0,0,0,0,0,0,0],[1,0,0,0,0,0,0,0,0,0,0,0,0,0]]C0263149	cellulitis of groin	[[1,0,0,0,0,0,0,0,0,0,0,0,0,0]]C1519410	solid mammary carcinoma of mouse	[[0,0,0,0,0,0,0,0,0,0,0,1,0,0]]C0686617	secondary malignant neoplasm of thymus,secondary malignant tumor of thymus,secondary malignant tumour of thymus,metastasis to thymus,metastatic malignant neoplasm to thymus,metastatic malignant tumor to the thymus,metastatic neoplasm to the thymus,metastatic tumor to the thymus	[[1,0,0,0,0,0,0,0,0,0,0,1,0,0],[1,0,0,0,0,0,0,0,0,0,0,1,0,0],[1,0,0,0,0,0,0,0,0,0,0,0,0,0],[1,0,0,0,0,0,0,0,0,0,0,1,0,0],[1,0,0,0,0,0,0,0,0,0,0,1,0,0],[0,0,0,0,0,0,0,0,0,0,0,1,0,0],[0,0,0,0,0,0,0,0,0,0,0,1,0,0],[0,0,0,0,0,0,0,0,0,0,0,1,0,0]]C0686610	neoplasm of uncertain behavior of adenoid,neoplasm of uncertain behaviour of adenoid	[[1,0,0,0,0,0,0,0,0,0,0,0,0,0],[1,0,0,0,0,0,0,0,0,0,0,0,0,0]]C0686611	carcinoma in situ of adenoid	[[1,0,0,0,0,0,0,0,0,0,0,0,0,0]]C0686612	secondary malignant neoplasm of adenoid,metastatic malignant neoplasm to adenoid	[[1,0,0,0,0,0,0,0,0,0,0,0,0,0],[1,0,0,0,0,0,0,0,0,0,0,0,0,0]]C0686613	neoplasm of uncertain behavior of lingual tonsil,neoplasm of uncertain behaviour of lingual tonsil	[[1,0,0,0,0,0,0,0,0,0,0,0,0,0],[1,0,0,0,0,0,0,0,0,0,0,0,0,0]]C0030312	pancytopenia,bone marrow failure	[[0,1,1,1,0,0,0,1,0,0,0,0,0,0],[0,0,0,1,0,0,0,1,0,0,0,1,0,0]]C0686619	secondary malignant neoplasm of lymph node,metastases to lymph nodes,lymph metastasis nodes,secondary lymph node cancer,metastatic malignant neoplasm to lymph node,metastatic neoplasm to the lymph node,metastatic tumor to lymph node	[[1,0,0,0,0,0,0,0,0,0,0,0,0,0],[0,0,0,0,0,0,0,1,0,0,0,1,0,0],[0,0,0,0,0,0,0,1,0,0,0,1,0,0],[1,0,0,0,0,0,0,1,0,0,0,0,0,0],[1,0,0,0,0,0,0,0,0,0,0,1,0,0],[0,0,0,0,0,0,0,0,0,0,0,1,0,0],[0,0,0,0,0,0,0,0,0,0,0,1,0,0]]C0340231	tracheobronchomalacia,williams campbell syndrome,chondromalacia of trachea and bronchi	[[0,1,0,1,0,0,0,0,0,0,0,0,0,0],[1,0,0,1,0,0,0,0,0,0,0,0,0,0],[0,0,0,0,0,0,0,0,0,0,0,0,1,0]]C0340237	tuberculous bronchopleural fistula	[[1,0,0,0,0,0,0,0,0,0,0,0,0,0]]C0340236	bronchocutaneous fistula	[[1,0,0,0,0,0,0,0,0,0,0,0,0,1]]C0024449	mycetoma,mycetomata,maduromycosis,madura foot,fungal mycetoma,maduramycosis	[[0,1,0,0,0,0,0,1,0,0,1,0,0,0],[1,0,0,0,0,0,0,0,0,0,0,0,0,0],[0,0,0,0,0,0,0,1,0,0,0,0,0,0],[0,0,0,0,0,0,0,1,0,0,0,0,0,0],[0,0,0,0,0,0,0,1,0,0,0,0,0,0],[0,0,0,0,0,0,0,1,0,0,0,0,0,0]]C1565886	neonatal indirect hyperbilirubinemia	[[0,1,0,0,0,0,0,0,0,0,0,0,0,0]]C0024445	familial benign cervical lipomatosis,multiple symmetrical lipomatosis,madelung neck,madelung disease,cervical symmetrical lipomatosis,disease madelung s	[[0,1,0,1,0,0,0,0,0,0,0,0,0,0],[0,0,0,0,0,0,0,0,0,0,0,1,0,0],[1,1,0,0,0,0,0,0,0,0,0,0,0,0],[0,1,0,0,0,0,0,1,0,0,0,0,0,0],[0,0,0,0,0,0,0,0,0,0,0,1,0,0],[0,0,0,0,0,0,0,1,0,0,0,0,0,0]]C0340238	infectious disorder of bronchus,bronchial sepsis,bronchial infection,infection of bronchus	[[1,0,0,0,0,0,0,0,0,0,0,0,0,0],[1,0,0,0,0,0,0,1,0,0,0,0,0,0],[1,0,0,0,0,0,0,1,0,0,0,0,0,0],[1,0,0,0,0,0,0,0,0,0,0,0,0,0]]C0024441	macular holes	[[1,1,0,0,0,0,0,1,0,0,0,0,0,0]]C0024440	cystoid macular edema,cystoid macular oedema,cystoid central retinal edema,cme cystoid macular edema,cmo cystoid macular oedema,macular edema,cystoid macular dystrophy,cymd,dominant cystoid macular dystrophy,mddc	[[0,1,0,1,0,0,0,1,0,0,0,1,0,0],[1,0,0,0,0,0,0,1,0,0,0,0,0,0],[0,1,0,0,0,0,0,0,0,0,0,0,0,0],[1,0,0,0,0,0,0,0,0,0,0,0,0,0],[1,0,0,0,0,0,0,0,0,0,0,0,0,0],[0,0,0,0,0,0,0,0,0,0,0,0,1,0],[0,0,0,1,0,0,0,0,0,0,0,0,0,0],[0,0,0,1,0,0,0,0,0,0,0,0,0,0],[0,0,0,1,0,0,0,0,0,0,0,0,0,0],[0,0,0,1,0,0,0,0,0,0,0,0,0,0]]C0155949	exfoliation of teeth due to systemic causes	[[0,0,0,0,1,0,0,0,0,0,0,0,0,0]]C0866462	carbuncle and furuncle of ear,carbuncle and furuncle of any part of ear	[[0,0,0,0,0,0,0,0,0,0,0,0,0,1],[0,0,0,0,0,0,0,0,0,0,0,0,0,1]]C0866467	carbuncle and furuncle of temple,carbuncle and furuncle of temple region	[[0,0,0,0,0,0,0,0,0,0,0,0,0,1],[0,0,0,0,0,0,0,0,0,0,0,0,0,1]]C0866466	carbuncle and furuncle of septum of nose	[[0,0,0,0,0,0,0,0,0,0,0,0,0,1]]C0866465	carbuncle and furuncle of nose	[[0,0,0,0,0,0,0,0,0,0,0,0,0,1]]C0155943	arthralgia of temporomandibular joint,temporomandibular joint pain	[[0,0,0,0,1,0,0,0,0,0,0,0,0,0],[0,0,0,0,0,0,0,1,0,0,0,0,0,0]]C0155942	adhesions and ankylosis,bony or fibrous adhesions and ankylosis	[[0,0,0,0,1,0,0,0,0,0,0,0,0,1],[0,0,0,0,0,0,0,0,0,0,0,0,0,1]]C1532560	plasmacytoma category	[[1,0,0,0,0,0,0,0,0,0,0,0,0,0]]C2959462	aortic orifice right side by side with respect to pulmonary orifice	[[1,0,0,0,0,0,0,0,0,0,0,0,0,0]]C1397800	nail fragility	[[0,0,0,1,0,0,0,0,0,0,0,0,0,0]]C0266327	accessory ureter	[[0,0,0,0,0,0,0,0,0,0,0,0,0,1]]C2183395	dieulafoy lesion hemorrhagic of intestine	[[0,0,0,0,1,0,0,0,0,0,0,0,0,0]]C1261359	fibromatous neoplasm,soft tissue tumor of fibrous differentiation,soft tissue tumour of fibrous differentiation	[[1,0,0,0,0,0,0,0,0,0,0,0,0,0],[1,0,0,0,0,0,0,0,0,0,0,0,0,0],[1,0,0,0,0,0,0,0,0,0,0,0,0,0]]C0277548	epidemic disease	[[1,0,0,0,0,0,0,1,0,0,0,0,0,0]]C0277549	epizootic disease	[[1,0,0,0,0,0,0,0,0,0,0,0,0,0]]C0277540	disease suspected	[[1,0,0,0,0,0,0,0,0,0,0,0,0,0]]C0277542	organic disease present	[[1,0,0,0,0,0,0,0,0,0,0,0,0,0]]C0277543	functional disease present,related to functional disturbance clinical disease and or syndrome present,functional disorder present	[[1,0,0,0,0,0,0,0,0,0,0,0,0,0],[1,0,0,0,0,0,0,0,0,0,0,0,0,0],[1,0,0,0,0,0,0,0,0,0,0,0,0,0]]C0277544	subclinical disease and or syndrome	[[1,0,0,0,0,0,0,0,0,0,0,0,0,0]]C0041296	tuberculosis,tuberculoses,infection tbc,infection tuberculosis,infection due to mycobacterium tuberculosis,tb tuberculosis,tb,tuberculous,tbc,kochs disease	[[0,0,1,0,0,0,0,1,0,0,0,0,0,0],[0,1,0,0,0,0,0,0,0,0,0,0,0,0],[0,0,0,0,0,0,0,0,1,0,0,0,0,0],[0,0,0,0,0,0,0,1,1,0,0,0,0,0],[1,0,0,0,0,0,0,0,0,0,0,0,0,0],[1,0,0,0,0,0,0,0,0,0,0,0,0,0],[0,0,0,0,0,0,0,0,0,0,0,1,0,0],[1,0,0,0,0,0,0,1,0,0,0,0,0,0],[0,0,0,0,0,0,0,1,0,0,0,0,0,0],[0,1,0,0,0,0,0,0,0,0,0,0,1,0]]C0041295	tuberculoma	[[0,1,0,0,0,0,0,1,0,0,0,0,1,0]]C0277547	disease type and or category not assigned	[[1,0,0,0,0,0,0,0,0,0,0,0,0,0]]C1333092	colon leiomyoma	[[0,0,0,0,0,0,0,0,0,0,0,1,0,0]]C2711266	infection by anisakis larva,anisakiasis	[[1,0,0,0,0,0,0,0,0,0,0,0,0,0],[1,0,0,0,0,0,0,0,0,0,0,0,0,0]]C0155351	episcleritis periodica fugax	[[0,0,0,0,1,0,0,0,0,0,0,0,0,0]]C0854165	papillary conjunctivitis	[[1,0,0,0,0,0,0,1,0,0,0,1,0,0]]C0263318	dermal type erythema multiforme	[[1,0,0,0,0,0,0,0,0,0,0,0,0,0]]C0263319	mixed dermal epidermal type erythema multiforme	[[1,0,0,0,0,0,0,0,0,0,0,0,0,0]]C0263314	pemphigus and fogo selvagem,amendolas syndrome,brazilian pemphigus foliaceus,fogo selvagem,wildfire pemphigus,brazilian pemphigus,wild fire,south american pemphigus,endemic pemphigus foliaceus	[[0,1,0,0,0,0,0,0,0,0,0,0,0,0],[1,0,0,0,0,0,0,0,0,0,0,0,0,0],[0,1,0,0,0,0,0,0,0,0,0,0,0,0],[1,0,0,0,0,0,0,1,0,0,0,0,0,0],[1,0,0,0,0,0,0,0,0,0,0,0,0,0],[0,1,0,0,0,0,0,0,0,0,0,0,0,0],[1,0,0,0,0,0,0,1,0,0,0,0,0,0],[0,1,0,0,0,0,0,0,0,0,0,0,0,0],[0,1,0,0,0,0,0,0,0,0,0,0,0,0]]C0263315	malignant pemphigus	[[1,0,0,0,0,0,0,0,0,0,0,0,0,1]]C0263316	pemphigus vegetans	[[1,0,0,0,0,0,0,1,0,0,0,0,0,1]]C0263310	hydroa herpetiformis	[[0,0,0,0,0,0,0,0,0,0,0,0,0,1]]C0263311	senile dermatitis herpetiformis	[[0,0,0,0,0,0,0,0,0,0,0,0,0,1]]C0263312	pemphigus erythematosus,senear usher syndrome	[[1,0,0,0,0,0,0,0,0,0,0,0,0,1],[1,0,0,0,0,0,0,0,0,0,0,0,0,0]]C0263313	pemphigus foliaceus,pemphigus foliaceous,pf pemphigus foliaceous	[[1,1,0,0,0,0,0,1,0,0,0,0,0,1],[1,0,0,0,0,0,0,1,0,0,0,0,0,0],[1,0,0,0,0,0,0,0,0,0,0,0,0,0]]C0411096	premature weight 1000g 2499g or gestation of 28 37weeks	[[1,0,0,0,0,0,0,0,0,0,0,0,0,0]]C0411090	very large baby weight greater than 4500gm	[[1,0,0,0,0,0,0,0,0,0,0,0,0,0]]C0155356	brawny scleritis,diffuse scleritis	[[0,0,0,0,1,0,0,0,0,0,0,0,0,0],[1,0,0,0,0,0,0,0,0,0,0,0,0,0]]C1960445	tachycardia induced cardiomyopathy	[[1,0,0,0,0,0,0,0,0,0,0,0,0,0]]C1960447	hypergonadotropic amenorrhea,hypergonadotropic amenorrhoea	[[1,0,0,0,0,0,0,0,0,0,0,0,0,0],[1,0,0,0,0,0,0,0,0,0,0,0,0,0]]C1960446	pneumothorax ex vacuo	[[1,0,0,0,0,0,0,0,0,0,0,0,0,0]]C1960443	vasculitic neuropathy	[[1,0,0,0,0,0,0,0,0,0,0,0,0,0]]C1960449	fat overload syndrome	[[1,0,0,0,0,0,0,0,0,0,0,0,0,0]]C0852667	metastatic vulva cancer,metastatic vulvar cancer,stage ivb vulval cancer,stage ivb vulvar cancer,stage ivb vulva carcinoma,stage ivb vulval carcinoma,stage ivb vulvar carcinoma,metastatic vulvar carcinoma,stage ivb vulvar cancer ajcc v7,stage ivb vulvar cancer ajcc v6	[[0,0,0,0,0,1,0,0,0,0,0,1,0,0],[0,0,0,0,0,1,0,0,0,0,0,1,0,0],[0,0,0,0,0,0,0,0,0,0,0,1,0,0],[0,0,0,0,0,0,0,0,0,0,0,1,0,0],[0,0,0,0,0,0,0,0,0,0,0,1,0,0],[0,0,0,0,0,0,0,0,0,0,0,1,0,0],[0,0,0,0,0,0,0,0,0,0,0,1,0,0],[0,0,0,0,0,0,0,0,0,0,0,1,0,0],[0,0,0,0,0,0,0,0,0,0,0,1,0,0],[0,0,0,0,0,0,0,0,0,0,0,1,0,0]]C0398649	metabolic thrombocytopenic purpura	[[1,0,0,0,0,0,0,0,0,0,0,0,0,0]]C2751843	without megalencephaly cystic leukoencephalopathy	[[0,0,0,1,0,0,0,0,0,0,0,0,0,0]]C0856563	osteochondrosis of head of femur	[[0,0,0,0,0,0,0,0,0,0,0,0,0,1]]C1513671	mouse pancreatic intraepithelial neoplasia 1a	[[0,0,0,0,0,0,0,0,0,0,0,1,0,0]]C0398643	mediterranean thrombocytopenia	[[1,0,0,0,0,0,0,0,0,0,0,0,0,0]]C0398642	montreal platelet syndrome,montreal syndrome	[[1,0,0,0,0,0,0,0,0,0,0,0,0,0],[1,0,0,0,0,0,0,0,0,0,0,0,0,0]]C0398641	epsteins macrothrombocytopenia syndrome,epstein syndrome,alport syndrome with macrothrombocytopenia,and deafness nephritis macrothrombocytopenia	[[1,0,0,0,0,0,0,0,0,0,0,0,0,0],[1,0,0,1,0,0,0,1,0,1,0,0,0,0],[0,1,0,0,0,0,0,0,0,0,0,0,0,0],[0,0,0,1,0,0,0,0,0,0,0,0,0,0]]C0398640	megakaryocytic thrombocytopenia	[[1,0,0,0,0,0,0,0,0,0,0,0,0,0]]C1513672	mouse pathologic diagnoses	[[0,0,0,0,0,0,0,0,0,0,0,1,0,0]]C0398645	benign primary hypergammaglobulinemic purpura,benign primary hypergammaglobulinaemic purpura	[[0,0,0,0,0,0,0,0,0,0,0,0,0,1],[1,0,0,0,0,0,0,0,0,0,0,0,0,0]]C0398644	hypergammaglobulinemic purpura of waldenstrom,waldenstroms hypergammaglobulinaemic purpura	[[1,1,0,0,0,0,0,0,0,0,0,0,0,1],[1,0,0,0,0,0,0,0,0,0,0,0,0,0]]C0027404	narcolepsy,gelineau syndrome,gelineaus syndrome,paroxysmal sleep,narcoleptic syndrome,narcolepsy disorder,excessive uncontrollable daytime sleepiness	[[0,0,1,0,0,0,0,1,0,0,0,0,0,1],[0,1,0,0,0,0,0,1,0,0,1,0,1,0],[0,1,0,0,0,0,0,1,0,1,0,0,1,0],[1,1,0,0,0,0,0,1,0,0,1,0,1,0],[1,1,0,0,0,0,0,1,0,0,0,0,1,0],[0,0,0,0,0,0,0,1,0,0,0,0,0,0],[0,0,0,0,0,0,0,1,0,0,0,0,0,0]]C2012072	glassy cell carcinoma of the cervix,glassy cell adenocarcinoma of the uterine cervix	[[0,1,0,0,0,0,0,0,0,0,0,0,0,0],[0,1,0,0,0,0,0,0,0,0,0,0,0,0]]C1844137	hemophilia a with vascular abnormality	[[0,0,0,1,0,0,0,0,0,0,0,0,0,0]]C1532230	disease due to parapoxvirus	[[1,0,0,0,0,0,0,0,0,0,0,0,0,0]]C2712941	disorder of stomach function and feeding problems in newborn	[[0,0,0,0,1,0,0,0,0,0,0,0,0,0]]C1335719	recurrent peripheral primitive neuroectodermal tumor	[[0,0,0,0,0,0,0,0,0,0,0,1,0,0]]C1335718	recurrent oral cavity cancer,relapsed oral cavity cancer,recurrent oral cavity carcinoma,relapsed oral cavity carcinoma,recurrent mouth carcinoma,relapsed mouth carcinoma	[[0,0,0,0,0,0,0,0,0,0,0,1,0,0],[0,0,0,0,0,0,0,0,0,0,0,1,0,0],[0,0,0,0,0,0,0,0,0,0,0,1,0,0],[0,0,0,0,0,0,0,0,0,0,0,1,0,0],[0,0,0,0,0,0,0,0,0,0,0,1,0,0],[0,0,0,0,0,0,0,0,0,0,0,1,0,0]]C0347278	benign neoplasm of biliary tract,benign tumor of biliary tract,benign tumour of biliary tract	[[1,0,0,0,0,0,0,0,0,0,0,0,0,0],[1,0,0,0,0,0,0,0,0,0,0,0,0,0],[1,0,0,0,0,0,0,0,0,0,0,0,0,0]]C0347279	benign neoplasm of sphincter of oddi	[[0,0,0,0,0,0,0,0,0,0,0,0,0,1]]C1299930	medial coronoid process disease	[[1,0,0,0,0,0,0,0,0,0,0,0,0,0]]C0032319	pneumopericardium	[[0,1,0,0,0,0,0,1,0,0,0,0,1,0]]C0836973	acute myelomonocytic leukemia in remission	[[0,0,0,0,0,0,0,0,0,0,0,1,0,0]]C0347272	benign neoplasm of large intestine,benign tumor of large intestine,benign tumour of large intestine,benign neoplasm of the large bowel,benign tumor of the large bowel,benign colorectal neoplasm,benign colorectal tumors	[[1,0,0,0,0,0,0,0,0,0,0,1,0,1],[1,0,0,0,0,0,0,0,0,0,0,1,0,0],[1,0,0,0,0,0,0,0,0,0,0,0,0,0],[0,0,0,0,0,0,0,0,0,0,0,1,0,0],[0,0,0,0,0,0,0,0,0,0,0,1,0,0],[0,0,0,0,0,0,0,0,0,0,0,1,0,0],[0,0,0,0,0,0,0,0,0,0,0,1,0,0]]C0347273	benign neoplasm of ileocecal valve,benign neoplasm of ileocaecal valve	[[0,0,0,0,0,0,0,0,0,0,0,0,0,1],[1,0,0,0,0,0,0,0,0,0,0,0,0,0]]C1335713	recurrent meningioma,relapsed meningioma	[[0,0,0,0,0,0,0,0,0,0,0,1,0,0],[0,0,0,0,0,0,0,0,0,0,0,1,0,0]]C0347271	jejunal polyp	[[1,0,0,0,0,0,0,0,0,0,0,0,0,0]]C0032310	viral pneumonia,abacterial pneumonia,viral pneumonitis	[[0,1,0,0,0,0,0,1,0,0,1,0,1,0],[0,0,0,0,0,0,0,0,0,0,1,0,0,0],[0,0,1,0,0,0,0,1,0,0,0,0,0,0]]C0347277	benign neoplasm of liver and or biliary ducts,benign neoplasm of liver and biliary passages	[[1,0,0,0,0,0,0,0,0,0,0,0,0,0],[0,0,0,0,1,0,0,0,0,0,0,0,0,0]]C1335717	recurrent non cutaneous melanoma	[[0,0,0,0,0,0,0,0,0,0,0,1,0,0]]C0347275	benign papilloma rectum	[[1,0,0,0,0,0,0,0,0,0,0,0,0,0]]C1532233	disorder of cellular component of blood in newborn	[[1,0,0,0,0,0,0,0,0,0,0,0,0,0]]C0684507	malignant melanoma of skin of umbilicus	[[1,0,0,0,0,0,0,0,0,0,0,0,0,0]]C0684504	malignant melanoma of skin of buttock	[[1,0,0,0,0,0,0,0,0,0,0,0,0,0]]C0399400	tooth surface loss,tooth substance loss	[[1,0,0,0,0,0,0,0,0,0,0,0,0,0],[1,0,0,0,0,0,0,0,0,0,0,0,0,0]]C0684502	malignant melanoma of skin of chest	[[1,0,0,0,0,0,0,0,0,0,0,0,0,0]]C0684503	malignant melanoma of skin of breast	[[1,0,0,0,0,0,0,0,0,0,0,0,0,0]]C0684500	malignant melanoma of skin of abdomen	[[1,0,0,0,0,0,0,0,0,0,0,0,0,0]]C0684501	malignant melanoma of skin of back	[[1,0,0,0,0,0,0,0,0,0,0,0,0,0]]C0017086	gangrene,gangrenous disorder	[[1,1,1,0,0,0,0,1,0,1,0,0,0,0],[1,0,0,0,0,0,0,0,0,0,0,0,0,0]]C0017083	gangliosidoses,gangliosidosis,ganglioside accumulation in nervous tissue lysosomes,ganglioside storage diseases,ganglioside storage disorders,gangliosidosis disease	[[0,0,0,0,0,0,0,1,0,0,0,0,1,0],[0,0,0,0,0,0,0,1,0,0,0,0,1,0],[1,0,0,0,0,0,0,0,0,0,0,0,0,0],[1,1,0,0,0,0,0,1,0,0,0,0,1,0],[0,1,0,0,0,0,0,0,0,0,0,0,1,0],[0,0,0,0,0,0,0,0,0,0,0,0,0,1]]C0684509	malignant melanoma of skin of elbow	[[1,0,0,0,0,0,0,0,0,0,0,0,0,0]]C0747556	pharyngitis recurrent	[[0,0,0,0,0,0,0,1,0,0,0,0,0,0]]C0153453	malignant tumor of extrahepatic bile duct,malignant tumour of extrahepatic bile duct,malignant extrahepatic bile duct neoplasm,extrahepatic bile duct cancer,ca extrahepatic bile ducts	[[1,0,0,0,0,0,0,0,0,0,0,1,0,0],[1,0,0,0,0,0,0,0,0,0,0,0,0,0],[0,0,0,0,1,0,0,0,0,0,0,1,0,0],[0,0,0,0,0,1,0,0,0,0,0,0,0,0],[1,0,0,0,0,0,0,0,0,0,0,0,0,0]]C0153452	malignant neoplasm of gallbladder,cancer of gallbladder,malignant tumor of gallbladder,malignant tumour of gallbladder,gallbladder ca,gall bladder cancer	[[0,0,0,0,1,0,0,1,0,0,0,1,0,0],[0,1,0,0,0,1,0,1,0,0,0,0,0,0],[1,0,0,0,0,0,0,0,0,0,0,1,0,0],[1,0,0,0,0,0,0,0,0,0,0,0,0,0],[1,0,0,0,0,0,0,1,0,0,0,0,0,0],[0,1,0,0,0,0,0,0,0,0,0,0,0,0]]C1519954	vascular fibrous neoplasms of the mouse pharynx	[[0,0,0,0,0,0,0,0,0,0,0,1,0,0]]C0153454	malignant neoplasm of ampulla of vater,malignant tumor of ampulla of vater,malignant tumour of ampulla of vater	[[0,0,0,0,1,0,0,0,0,0,0,1,0,0],[1,0,0,0,0,0,0,0,0,0,0,1,0,0],[1,0,0,0,0,0,0,0,0,0,0,0,0,0]]C0153459	malignant neoplasm of body of pancreas,malignant tumor of body of pancreas,malignant tumour of body of pancreas,ca body of pancreas	[[1,0,0,0,0,0,0,0,0,0,0,0,0,0],[1,0,0,0,0,0,0,0,0,0,0,0,0,0],[1,0,0,0,0,0,0,0,0,0,0,0,0,0],[1,0,0,0,0,0,0,0,0,0,0,0,0,0]]C0153458	malignant neoplasm of head of pancreas,malignant tumor of head of pancreas,malignant tumour of head of pancreas,ca head of pancreas	[[0,0,0,0,1,0,1,0,0,0,0,0,0,0],[1,0,0,0,0,0,0,0,0,0,0,0,0,0],[1,0,0,0,0,0,0,0,0,0,0,0,0,0],[1,0,0,0,0,0,0,0,0,0,0,0,0,0]]C1332970	childhood hematopoietic neoplasm	[[0,0,0,0,0,0,0,0,0,0,0,1,0,0]]C1332971	childhood hepatic neoplasm,childhood liver neoplasm	[[0,0,0,0,0,0,0,0,0,0,0,1,0,0],[0,0,0,0,0,0,0,0,0,0,0,1,0,0]]C1827311	neuroinvasive western equine encephalitis virus infection	[[1,0,0,0,0,0,0,0,0,0,0,0,0,0]]C1332972	childhood infratentorial ependymoblastoma,pediatric infratentorial ependymoblastoma	[[0,0,0,0,0,0,0,0,0,0,0,1,0,0],[0,0,0,0,0,0,0,0,0,0,0,1,0,0]]C0157514	with delivery antepartum deep phlebothrombosis	[[0,0,0,0,1,0,0,0,0,0,0,0,0,0]]C0157517	with delivery postpartum deep phlebothrombosis	[[0,0,0,0,1,0,0,0,0,0,0,0,0,0]]C1332974	childhood conventional osteosarcoma,childhood intracortical osteosarcoma	[[0,0,0,0,0,0,0,0,0,0,0,1,0,0],[0,0,0,0,0,0,0,0,0,0,0,1,0,0]]C1706980	bone leiomyoma	[[0,0,0,0,0,0,0,0,0,0,0,1,0,0]]C0398592	cr3 receptor deficiency	[[1,0,0,0,0,0,0,0,0,0,0,0,0,0]]C1864861	familial normophosphatemic tumoral calcinosis,with normophosphatemia tumoral calcinosis,nftc	[[0,0,0,1,0,0,0,0,0,0,0,0,0,0],[0,0,0,1,0,0,0,0,0,0,0,0,0,0],[0,0,0,1,0,0,0,0,0,0,0,0,0,0]]C1516069	astler coller c1 colorectal carcinoma	[[0,0,0,0,0,0,0,0,0,0,0,1,0,0]]C1332976	childhood leptomeningeal melanoma,pediatric leptomeningeal melanoma	[[0,0,0,0,0,0,0,0,0,0,0,1,0,0],[0,0,0,0,0,0,0,0,0,0,0,1,0,0]]C0206683	papillary and follicular adenocarcinoma,follicular papillary carcinoma,follicular variant papillary adenocarcinoma,follicular variant papillary carcinoma,thyroid gland papillary and follicular carcinoma,follicular variant thyroid gland papillary carcinoma	[[1,0,0,0,0,0,0,0,0,0,0,1,0,0],[1,0,0,0,0,0,0,0,0,0,0,1,1,0],[1,0,0,0,0,0,0,0,0,0,0,1,0,0],[1,0,0,0,0,0,0,0,0,0,0,1,0,0],[0,0,0,0,0,0,0,0,0,0,0,1,0,0],[0,0,0,0,0,0,0,0,0,0,0,1,0,0]]C0206682	follicular thyroid carcinoma,follicular carcinoma,follicular adenocarcinoma,adenocarcinoma thyroid,follicular carcinoma thyroid cancer,follicular thyroid cancer,well differentiated follicular adenocarcinoma,well differentiated follicular carcinoma,ftc follicular thyroid carcinoma,follicular cancer of the thyroid gland,thyroid gland follicular carcinoma,ftc	[[1,0,0,1,0,0,0,1,0,0,1,1,0,0],[1,0,0,0,0,0,0,1,0,0,0,1,0,0],[1,1,0,0,0,0,0,0,0,0,0,0,1,0],[0,0,0,0,0,0,0,1,0,0,0,0,0,0],[0,0,0,0,0,0,0,0,0,0,1,0,0,0],[0,0,0,0,0,1,0,1,0,0,0,1,0,0],[1,0,0,0,0,0,0,0,0,0,0,1,0,0],[1,0,0,0,0,0,0,0,0,0,0,1,0,0],[1,0,0,0,0,0,0,0,0,0,0,0,0,0],[0,0,0,0,0,0,0,0,0,0,0,1,0,0],[0,0,0,0,0,0,0,0,0,0,0,1,0,0],[0,0,0,1,0,0,0,0,0,0,0,0,0,0]]C0206681	clear cell adenocarcinoma,clear cell carcinoma,mesonephroid clear cell adenocarcinoma,mesonephroid clear cell carcinoma,malignant glandular clear cell	[[1,1,0,0,0,0,0,1,0,0,0,1,1,0],[1,0,0,0,0,0,0,1,0,0,0,1,0,0],[1,0,0,0,0,0,0,0,0,0,0,1,0,0],[0,0,0,0,0,0,0,0,0,0,0,1,0,0],[0,0,0,0,0,0,0,0,0,0,0,1,0,0]]C0206680	cystic mesothelioma,m cystic mesothelioma,intermediate mesothelioma,multicystic mesothelioma	[[1,1,0,0,0,0,0,0,0,0,0,0,1,0],[1,0,0,0,0,0,0,0,0,0,0,0,0,0],[0,0,0,0,0,0,0,0,0,0,0,1,0,0],[0,0,0,0,0,0,0,0,0,0,0,1,0,0]]C0206687	endometrioid carcinoma,endometrioid adenocarcinoma,female reproductive endometrioid carcinoma,endometrioid carcinoma of the female reproductive system	[[1,1,0,0,0,0,0,1,0,0,0,0,0,0],[0,0,0,0,0,0,0,1,0,0,0,1,0,0],[0,0,0,0,0,0,0,0,0,0,0,1,0,0],[0,0,0,0,0,0,0,0,0,0,0,1,0,0]]C0206686	adrenocortical carcinoma,adrenal cortical adenocarcinoma,adrenal cortical carcinoma,adrenal cortex cancer,adrenocortical cancer,carcinoma adrenal cortex cancer,adrenal cortex carcinoma,adrenal carcinoma,adrenal adenocarcinoma,adrenal cortex adenocarcinoma	[[0,1,0,1,0,1,0,1,0,0,0,0,1,0],[1,0,0,0,0,0,0,0,0,0,0,1,0,0],[1,1,0,0,0,0,0,1,0,0,0,0,1,0],[0,0,0,0,0,0,0,0,0,0,0,1,0,0],[0,0,0,0,0,0,0,0,0,0,0,1,0,0],[0,0,0,0,0,0,0,0,0,0,1,0,0,0],[0,0,0,0,0,0,0,1,0,0,1,1,0,0],[0,0,0,0,0,0,0,1,0,0,0,0,0,0],[0,0,0,0,0,0,0,1,0,0,0,0,0,0],[0,0,0,0,0,0,0,0,0,0,0,1,0,0]]C0206685	acinar cell carcinoma,acinic cell adenocarcinoma,acinar adenocarcinoma,acinar carcinoma,acinic cell tumor,acinar cell adenocarcinoma,acinic cell carcinoma	[[1,1,0,0,0,0,0,1,0,0,0,0,1,0],[1,1,0,0,0,0,0,0,0,0,0,0,1,0],[1,0,0,0,0,0,0,1,0,0,0,1,0,0],[1,1,0,0,0,0,0,1,0,0,0,0,1,0],[0,1,0,0,0,0,0,0,0,0,0,0,1,0],[0,1,0,0,0,0,0,0,0,0,0,0,1,0],[0,1,0,0,0,0,0,0,0,0,0,0,1,0]]C0206684	sebaceous adenocarcinoma,sebaceous carcinoma,carcinoma of sebaceous gland,m sebaceous adenocarcinoma	[[1,1,0,0,0,0,0,1,0,0,0,0,1,0],[0,0,0,1,0,0,0,1,0,0,0,1,0,0],[1,0,0,0,0,0,0,1,0,0,0,1,0,0],[1,0,0,0,0,0,0,0,0,0,0,0,0,0]]C0855036	metastatic alveolar soft part sarcoma	[[0,0,0,0,0,0,0,0,0,0,0,1,0,0]]C0855037	primary alveolar soft part sarcoma,alveolar soft part sarcoma nonmetastatic,non metastatic alveolar soft part sarcoma	[[0,0,0,0,0,0,0,0,0,0,0,1,0,0],[0,0,0,0,0,0,0,0,0,0,0,1,0,0],[0,0,0,0,0,0,0,0,0,0,0,1,0,0]]C0571888	class ii antiarrhythmic allergy	[[1,0,0,0,0,0,0,0,0,0,0,0,0,0]]C0571889	bretylium allergy	[[1,0,0,0,0,0,0,0,0,0,0,0,0,0]]C0855032	stage iii superficial spreading melanoma	[[0,0,0,0,0,0,0,0,0,0,0,1,0,0]]C0855033	stage iv superficial spreading melanoma	[[0,0,0,0,0,0,0,0,0,0,0,1,0,0]]C0855030	stage i superficial spreading melanoma	[[0,0,0,0,0,0,0,0,0,0,0,1,0,0]]C0400952	bacterial portal cirrhosis	[[1,0,0,0,0,0,0,0,0,0,0,0,0,0]]C0571882	flecainide allergy	[[1,0,0,0,0,0,0,0,0,0,0,0,0,0]]C0571883	mexiletine allergy	[[1,0,0,0,0,0,0,0,0,0,0,0,0,0]]C0571880	disopyramide allergy	[[1,0,0,0,0,0,0,0,0,0,0,0,0,0]]C0571881	quinidine allergy	[[1,0,0,0,0,0,0,0,0,0,0,0,0,0]]C0571886	propafenone allergy	[[1,0,0,0,0,0,0,0,0,0,0,0,0,0]]C0571887	tocainide allergy	[[1,0,0,0,0,0,0,0,0,0,0,0,0,0]]C0571884	moracizine allergy	[[1,0,0,0,0,0,0,0,0,0,0,0,0,0]]C0571885	procainamide allergy	[[1,0,0,0,0,0,0,0,0,0,0,0,0,0]]C2112532	postmenopausal endometrium,senile endometrium	[[1,0,0,0,0,0,0,0,0,0,0,0,0,0],[1,0,0,0,0,0,0,0,0,0,0,0,0,0]]C1862472	oculomelic amyoplasia,arthrogryposis with oculomotor limitation and electroretinal abnormalities,type 5 distal arthrogryposis,da5,type iib distal arthrogryposis,daiib	[[0,1,0,1,0,0,0,0,0,0,0,0,0,0],[0,1,0,1,0,0,0,0,0,0,0,0,0,0],[0,0,0,1,0,0,0,0,0,0,0,0,0,0],[0,0,0,1,0,0,0,0,0,0,0,0,0,0],[0,0,0,1,0,0,0,0,0,0,0,0,0,0],[0,0,0,1,0,0,0,0,0,0,0,0,0,0]]C0887801	intracranial tubercular abscess	[[0,1,0,0,0,0,0,0,0,0,0,0,0,0]]C0423676	lumbosacral neuritis	[[1,0,0,0,0,0,0,1,0,0,0,0,0,0]]C0410054	wrist impingement syndrome	[[1,0,0,0,0,0,0,0,0,0,0,0,0,0]]C1532237	disorder of immune function	[[1,0,0,0,0,0,0,0,0,0,0,0,0,0]]C1096037	venous angioma of brain	[[0,0,0,0,0,0,0,1,0,0,0,0,0,0]]C1336040	sphenoorbital meningioma	[[0,0,0,0,0,0,0,0,0,0,0,1,0,0]]C1859384	brachioskeletogenital syndrome,bsg syndrome	[[0,1,0,1,0,0,0,0,0,0,0,0,0,0],[0,0,0,1,0,0,0,0,0,0,0,0,0,0]]C1444087	disease due to neisseria	[[1,0,0,0,0,0,0,0,0,0,0,0,0,0]]C1532236	disorder of hematopoietic system in newborn,disorder of haematopoietic system in newborn	[[1,0,0,0,0,0,0,0,0,0,0,0,0,0],[1,0,0,0,0,0,0,0,0,0,0,0,0,0]]C0154157	postoperative hypothyroidism,postsurgical hypothyroidism	[[1,0,0,0,0,0,0,0,0,0,0,0,0,0],[0,0,0,0,1,0,0,0,0,0,0,0,0,0]]C0154159	iodine hypothyroidism	[[0,0,0,0,1,0,0,1,0,0,0,0,0,0]]C0026431	monkey diseases	[[0,1,0,0,0,0,0,1,0,0,0,0,0,0]]C0558722	blind left eye,loss of vision of left eye	[[1,0,0,0,0,0,0,0,0,0,0,0,0,0],[1,0,0,0,0,0,0,0,0,0,0,0,0,0]]C0558723	blind right eye,loss of vision of right eye	[[1,0,0,0,0,0,0,0,0,0,0,0,0,0],[1,0,0,0,0,0,0,0,0,0,0,0,0,0]]C1848934	spondylocarpotarsal synostosis syndrome,sct,spondylocarpotarsal syndrome,with unilateral unsegmented bar congenital scoliosis,congenital synspondylism,vertebral fusion with carpal coalition,spondylocarpotarsal synostosis	[[0,1,0,1,0,0,0,0,0,0,0,0,0,0],[0,0,0,1,0,0,0,0,0,0,0,0,0,0],[0,0,0,1,0,0,0,0,0,0,0,0,0,0],[0,1,0,1,0,0,0,0,0,0,0,0,0,0],[0,1,0,1,0,0,0,0,0,0,0,0,0,0],[0,1,0,1,0,0,0,0,0,0,0,0,0,0],[0,1,0,0,0,0,0,0,0,0,0,0,0,0]]C0730049	retained nuclear material in vitreous	[[1,0,0,0,0,0,0,0,0,0,0,0,0,0]]C0730048	retained lens matter in vitreous	[[1,0,0,0,0,0,0,0,0,0,0,0,0,0]]C1848931	tatsumi factor deficiency	[[0,0,0,1,0,0,0,0,0,0,0,0,0,0]]C1848932	tapetoretinal degeneration with ataxia	[[0,0,0,1,0,0,0,0,0,0,0,0,0,0]]C1291459	deficiency of hydroxymethylglutaryl coa hydrolase	[[1,0,0,0,0,0,0,0,0,0,0,0,0,0]]C1291457	deficiency of succinyl coa hydrolase,deficiency of succinyl coa acylase	[[1,0,0,0,0,0,0,0,0,0,0,0,0,0],[1,0,0,0,0,0,0,0,0,0,0,0,0,0]]C2732889	virilization of female due to sry gene translocation,virilisation of female due to sry gene translocation	[[1,0,0,0,0,0,0,0,0,0,0,0,0,0],[1,0,0,0,0,0,0,0,0,0,0,0,0,0]]C1291455	deficiency of retinyl palmitate esterase	[[1,0,0,0,0,0,0,0,0,0,0,0,0,0]]C1291454	deficiency of tannase	[[1,0,0,0,0,0,0,0,0,0,0,0,0,0]]C1291453	deficiency of uronolactonase	[[1,0,0,0,0,0,0,0,0,0,0,0,0,0]]C1291452	deficiency of chlorophyllase	[[1,0,0,0,0,0,0,0,0,0,0,0,0,0]]C1291451	deficiency of cholesterol esterase	[[1,0,0,0,0,0,0,0,0,0,0,0,0,0]]C1291450	deficiency of tropinesterase	[[1,0,0,0,0,0,0,0,0,0,0,0,0,0]]C0016024	fibroadenosis,adenofibrosis	[[1,0,0,0,0,0,0,0,0,0,0,0,0,0],[1,0,0,0,0,0,0,0,0,0,0,0,0,0]]C1336043	spinal cord hamartoma	[[0,0,0,0,0,0,0,0,0,0,0,1,0,0]]C1843884	spinocerebellar ataxia 18,smna,sca18,autosomal dominant sensorimotor neuropathy with ataxia	[[0,1,0,1,0,0,0,0,0,0,0,0,0,0],[0,0,0,1,0,0,0,0,0,0,0,0,0,0],[0,0,0,1,0,0,0,0,0,0,0,0,0,0],[0,1,0,1,0,0,0,0,0,0,0,0,0,0]]C1444192	polyarthritis due to erysipelothrix rhusiopathiae	[[1,0,0,0,0,0,0,0,0,0,0,0,0,0]]C1444193	post catheterization femoral pseudoaneurysm,post catheterisation femoral pseudoaneurysm,femoral pseudoaneurysm after arterial catheterization,femoral pseudoaneurysm after arterial catheterisation,post catheterization femoral false aneurysm,post catheterisation femoral false aneurysm	[[1,0,0,0,0,0,0,0,0,0,0,0,0,0],[1,0,0,0,0,0,0,0,0,0,0,0,0,0],[1,0,0,0,0,0,0,0,0,0,0,0,0,0],[1,0,0,0,0,0,0,0,0,0,0,0,0,0],[1,0,0,0,0,0,0,0,0,0,0,0,0,0],[1,0,0,0,0,0,0,0,0,0,0,0,0,0]]C0745157	hypokinesis global	[[0,0,0,0,0,0,0,1,0,0,0,0,0,0]]C0865178	female adrenal pseudohermaphroditism	[[0,0,0,0,0,0,0,0,0,0,0,0,0,1]]C0745150	acute hypoglycemia	[[0,0,0,1,0,0,0,1,0,0,0,0,0,0]]C0745153	hypoglycemic episodes	[[0,0,0,1,0,0,0,0,0,0,0,0,0,0]]C0393779	mucosal anosmia	[[1,0,0,0,0,0,0,0,0,0,0,0,0,0]]C0393776	sleep related painful erections	[[1,1,0,0,0,0,0,0,0,0,0,0,0,0]]C0393777	nocturnal paroxysmal dystonia,sleep related dystonia,hypnogenic paroxysmal dystonia	[[0,1,0,0,0,0,0,1,0,0,0,0,1,0],[1,1,0,0,0,0,0,0,0,0,0,0,1,0],[0,1,0,0,0,0,0,0,0,0,0,0,1,0]]C0393774	sleep related bruxism,teeth grinding while asleep,teeth grinding at night,teeth grinding whilst asleep	[[1,1,0,0,1,0,0,0,0,0,0,0,0,0],[1,0,0,0,0,0,0,0,0,0,0,0,0,0],[1,0,0,0,0,0,0,0,0,0,0,0,0,0],[1,0,0,0,0,0,0,0,0,0,0,0,0,0]]C0865177	whether acquired or associated with congenital adrenal hyperplasia consequent on inborn enzyme defects in hormone synthesis virilizing or feminizing adrenogenital syndromes	[[0,0,0,0,0,0,0,0,0,0,0,0,0,1]]C0865170	hyperplasia of pancreatic alpha cells with gastrin excess	[[0,0,0,0,0,0,0,0,0,0,0,0,0,1]]C0393773	work shift change	[[1,0,0,0,0,0,0,0,0,0,0,0,0,0]]C0393771	irregular sleep wake pattern	[[1,0,0,0,0,0,0,0,0,0,0,0,0,0]]C1862839	anterior segment mesenchymal dysgenesis,asmd,anterior segment ocular dysgenesis,asod	[[0,1,0,1,0,0,0,0,0,0,0,0,0,0],[0,0,0,1,0,0,0,0,0,0,0,0,0,0],[0,1,0,1,0,0,0,0,0,0,0,0,0,0],[0,0,0,1,0,0,0,0,0,0,0,0,0,0]]C1720470	widely invasive follicular carcinoma	[[1,0,0,0,0,0,0,0,0,0,0,0,0,0]]C1720471	reticulosarcoma associated with aids	[[1,0,0,0,0,0,0,0,0,0,0,0,0,0]]C2677099	crouzon syndrome with acanthosis nigricans,crouzonodermoskeletal syndrome	[[0,0,0,1,0,0,1,0,0,0,0,0,0,0],[0,0,0,1,0,0,0,0,0,0,0,0,0,0]]C1707390	chondroid hamartoma	[[0,0,0,0,0,0,0,0,0,0,0,1,0,0]]C1707395	ciliary body fuchs pseudoadenoma	[[0,0,0,0,0,0,0,0,0,0,0,1,0,0]]C0751356	idiopathic inflammatory myopathies	[[0,1,0,0,0,0,0,1,0,0,0,0,0,0]]C0751357	focal myositis,focal myositides	[[0,1,0,0,0,0,0,0,0,0,0,0,0,0],[0,1,0,0,0,0,0,0,0,0,0,0,0,0]]C0403353	urinary complications of care	[[1,0,0,0,0,0,0,0,0,0,0,0,0,0]]C0403358	adhesions of kidney	[[1,0,0,0,0,0,0,0,0,0,0,0,0,0]]C0854077	diabetic retinal edema	[[0,0,0,0,0,0,0,0,0,0,0,0,0,1]]C0854076	distal ileal obstruction syndrome,dios	[[0,0,0,0,0,0,0,1,0,0,0,0,0,0],[0,0,0,0,0,0,0,1,0,0,0,0,0,0]]C0404498	uterine cornual polyp	[[1,0,0,0,0,0,0,0,0,0,0,0,0,0]]C0598341	torsion neurosis	[[0,0,0,0,0,0,0,0,0,1,0,0,0,0]]C0155206	sensory disorder of eyelid	[[1,0,0,0,1,0,0,0,0,0,0,0,0,0]]C1402315	vascular lesions	[[0,0,0,1,0,0,0,0,0,0,0,0,0,0]]C1275175	pagetoid bowens disease	[[1,0,0,0,0,0,0,0,0,0,0,0,0,0]]C0155209	degenerative disorder of eyelid	[[1,0,0,0,0,0,0,0,0,0,0,0,0,0]]C3164256	sepsis due to acinetobacter	[[1,0,0,0,0,0,0,0,0,0,0,0,0,0]]C0865586	mitral and aortic valve incompetence	[[0,0,0,0,0,0,0,0,0,0,0,0,0,1]]C1321135	paraneoplastic hyponatremia,paraneoplastic hyponatraemia	[[1,0,0,0,0,0,0,0,0,0,0,0,0,0],[1,0,0,0,0,0,0,0,0,0,0,0,0,0]]C1321136	paraneoplastic hypokalemia,paraneoplastic hypokalaemia	[[1,0,0,0,0,0,0,0,0,0,0,0,0,0],[1,0,0,0,0,0,0,0,0,0,0,0,0,0]]C2677091	inflammatory bowel disease 17,ibd17	[[0,0,0,1,0,0,0,0,0,0,0,0,0,0],[0,0,0,1,0,0,0,0,0,0,0,0,0,0]]C0020179	huntington disease,chronic progressive hereditary chorea,huntington chorea,chronic progressive chorea,hc huntington chorea,hd huntington chorea,hd,huntington chronic progressive hereditary chorea	[[0,1,0,0,0,0,0,1,0,1,0,0,0,0],[1,0,0,0,0,0,0,0,0,0,1,0,1,0],[1,1,1,0,0,0,0,1,0,0,1,0,1,0],[1,0,0,0,0,0,0,0,0,0,0,0,0,0],[1,0,0,0,0,0,0,0,0,0,0,0,0,0],[1,0,0,0,0,0,0,0,0,0,0,0,0,0],[0,0,0,0,0,0,0,0,0,1,0,0,0,0],[0,0,0,0,0,0,0,0,0,0,0,0,1,0]]C2677092	myd88 deficiency,myd88d,due to myd88 deficiency recurrent pyogenic bacterial infections	[[0,0,0,1,0,0,1,0,0,0,0,0,0,0],[0,0,0,1,0,0,0,0,0,0,0,0,0,0],[0,0,0,1,0,0,0,0,0,0,0,0,0,0]]C0333540	symmetrical gangrene	[[1,0,0,0,0,0,0,0,0,0,0,0,0,0]]C0406200	polyvinyl pyrrolidone disease	[[1,0,0,0,0,0,0,0,0,0,0,0,0,0]]C1868074	endosteal and exosteal osteomas	[[0,0,0,1,0,0,0,0,0,0,0,0,0,0]]C0456039	newborn drug reaction	[[1,0,0,0,0,0,0,0,0,0,0,0,0,0]]C0456038	neutropenia of the small for gestational age baby,mcintosh syndrome	[[1,0,0,0,0,0,0,0,0,0,0,0,0,0],[1,0,0,0,0,0,0,0,0,0,0,0,0,0]]C0406209	photo onycholysis,photoonycholysis,photo onycholyses	[[1,1,0,0,0,0,0,1,0,0,0,0,0,0],[1,0,0,0,0,0,0,1,1,0,0,0,0,0],[0,1,0,0,0,0,0,0,0,0,0,0,0,0]]C0456036	chronic partial asphyxia of newborn	[[1,0,0,0,0,0,0,0,0,0,0,0,0,0]]C1291599	deficiency of carbon halide lyase	[[1,0,0,0,0,0,0,0,0,0,0,0,0,0]]C0456032	perinatal disorder of electrolytes	[[1,0,0,0,0,0,0,0,0,0,0,0,0,0]]C0456031	intrapartum fetal acidosis	[[1,0,0,0,0,0,0,0,0,0,0,0,0,0]]C0456030	antepartum fetal acidosis	[[1,0,0,0,0,0,0,0,0,0,0,0,0,0]]C1291596	deficiency of carbon nitrogen lyase	[[1,0,0,0,0,0,0,0,0,0,0,0,0,0]]C0741611	bowel ischemic	[[0,0,0,0,0,0,0,1,0,0,0,0,0,0]]C1291597	deficiency of ammonia lyase	[[1,0,0,0,0,0,0,0,0,0,0,0,0,0]]C2711706	angiodysplasia of duodenum	[[1,0,0,0,0,0,0,0,0,0,0,0,0,0]]C1291594	deficiency of carbon oxygen lyase	[[1,0,0,0,0,0,0,0,0,0,0,0,0,0]]C2316850	abscess of perivesicular tissue of urinary bladder	[[1,0,0,0,0,0,0,0,0,0,0,0,0,0]]C0570708	fludarabine allergy	[[1,0,0,0,0,0,0,0,0,0,0,0,0,0]]C0570709	aminoglutethimide allergy	[[1,0,0,0,0,0,0,0,0,0,0,0,0,0]]C0570702	cisplatin allergy	[[1,0,0,0,0,0,0,0,0,0,0,0,0,0]]C0570703	hydroxycarbamide allergy,hydroxyurea allergy	[[1,0,0,0,0,0,0,0,0,0,0,0,0,0],[1,0,0,0,0,0,0,0,0,0,0,0,0,0]]C0570700	amsacrine allergy	[[1,0,0,0,0,0,0,0,0,0,0,0,0,0]]C0570701	carboplatin allergy	[[1,0,0,0,0,0,0,0,0,0,0,0,0,0]]C0570706	crisantaspase allergy	[[1,0,0,0,0,0,0,0,0,0,0,0,0,0]]C0570707	paclitaxel allergy	[[1,0,0,0,0,0,0,0,0,0,0,0,0,0]]C0570704	procarbazine allergy	[[1,0,0,0,0,0,0,0,0,0,0,0,0,0]]C0570705	razoxane allergy	[[1,0,0,0,0,0,0,0,0,0,0,0,0,0]]C1319188	platelet fibrin retinal embolus,platelet retinal embolus,platelet thrombin embolus	[[1,0,0,0,0,0,0,0,0,0,0,0,0,0],[1,0,0,0,0,0,0,0,0,0,0,0,0,0],[1,0,0,0,0,0,0,0,0,0,0,0,0,0]]C1291590	deficiency of carbon carbon lyase	[[1,0,0,0,0,0,0,0,0,0,0,0,0,0]]C1319183	corticosteroid induced cataract,steroid induced cataract	[[1,0,0,0,0,0,0,0,0,0,0,0,0,0],[1,0,0,0,0,0,0,0,0,0,0,0,0,0]]C1291591	deficiency of carboxy lyase,deficiency of decarboxylase	[[1,0,0,0,0,0,0,1,0,0,0,0,0,0],[1,0,0,0,0,0,0,1,0,0,0,0,0,0]]C1306848	mumps without complication	[[1,0,0,0,0,0,0,1,0,0,0,0,0,0]]C1319184	lens particle glaucoma	[[1,0,0,0,0,0,0,0,0,0,0,0,0,0]]C1319185	chiasmal glioma,optic chiasmal glioma	[[1,0,0,0,0,0,0,0,0,0,0,0,0,0],[1,0,0,0,0,0,0,0,0,0,0,0,0,0]]C0580008	mild to moderate birth asphyxia apgar score 4 7 at 1 minute,birth asphyxia with 1 minute apgar score 4 7	[[1,0,0,0,0,0,0,0,0,0,0,0,0,0],[1,0,0,0,0,0,0,0,0,0,0,0,0,0]]C0008591	cutaneous chromomycosis	[[1,0,0,0,0,0,0,0,0,0,0,0,0,0]]C0580002	abscess of tendon	[[1,0,0,0,0,0,0,0,0,0,0,0,0,0]]C0686688	benign neoplasm of intrapelvic lymph nodes	[[1,0,0,0,0,0,0,0,0,0,0,0,0,0]]C0853892	catabolic state	[[0,0,0,0,0,0,0,1,0,0,0,0,0,0]]C1697280	tracheal inflammation	[[0,0,0,0,0,0,0,0,0,0,0,1,0,0]]C0853897	diabetic cardiomyopathies	[[0,1,0,0,0,0,0,0,0,1,0,0,1,0]]C0865828	interlobar sterile pleurisy	[[0,0,0,0,0,0,0,0,0,0,0,0,0,1]]C0865826	fibrinous sterile pleurisy	[[0,0,0,0,0,0,0,0,0,0,0,0,0,1]]C0865827	interlobar acute pleurisy	[[0,0,0,0,0,0,0,0,0,0,0,0,0,1]]C0865824	sterile diaphragmatic pleurisy	[[0,0,0,0,0,0,0,0,0,0,0,0,0,1]]C0865822	lung or pleura adhesion	[[0,0,0,0,0,0,0,0,0,0,0,0,0,1]]C0865823	acute diaphragmatic pleurisy	[[0,0,0,0,0,0,0,0,0,0,0,0,0,1]]C0865821	pleura abscess	[[0,0,0,0,0,0,0,0,0,0,0,0,0,1]]C0155000	transient refractive change	[[0,0,0,0,1,0,0,0,0,0,0,0,0,0]]C1969562	autosomal dominant 1 mental retardation,mrd1	[[0,0,0,1,0,0,0,0,0,0,0,0,0,0],[0,0,0,1,0,0,0,0,0,0,0,0,0,0]]C2613439	extramedullary hematopoiesis,extramedullary haematopoiesis	[[1,0,0,0,0,0,1,0,0,0,0,1,0,0],[1,0,0,0,0,0,0,0,0,0,0,0,0,0]]C3151236	alpha b crystallin related fatal infantile hypertonic myofibrillar myopathy,alpha b crystallin related fatal infantile hypertonic mfm	[[0,0,0,1,0,0,0,0,0,0,0,0,0,0],[0,0,0,1,0,0,0,0,0,0,0,0,0,0]]C0392169	encephalopathy neonatal acute	[[0,0,0,0,0,0,0,0,1,0,0,0,0,0]]C0278620	refractory plasma cell neoplasm,refractory plasma cell myeloma,refractory plasma cell tumor	[[0,0,0,0,0,1,0,0,0,0,0,1,0,0],[0,0,0,0,0,0,0,0,0,0,0,1,0,0],[0,0,0,0,0,0,0,0,0,0,0,1,0,0]]C0221542	dumping	[[0,0,1,0,0,0,0,1,0,0,0,0,0,0]]C0221541	disc rupture,ruptured disk	[[0,0,1,0,0,0,0,1,0,0,0,0,0,0],[0,0,0,0,0,0,0,1,0,0,0,0,0,0]]C0392163	corneal erosion,superficial ulcer of cornea	[[1,0,0,1,0,0,0,1,1,0,0,0,0,0],[1,0,0,0,0,0,0,0,0,0,0,0,0,0]]C0278628	low grade childhood cerebral astrocytoma,low grade childhood cerebral glioma	[[0,0,0,0,0,1,0,0,0,0,0,0,0,0],[0,0,0,0,0,1,0,0,0,0,0,0,0,0]]C0392165	cystitis due to pseudomonas,pseudomonal cystitis	[[1,0,0,0,0,0,0,0,0,0,0,0,0,0],[1,0,0,0,0,0,0,0,1,0,0,0,0,0]]C2064236	malignant neoplasm of anal margin	[[0,0,0,0,0,0,1,0,0,0,0,0,0,0]]C1858211	giant congenital aortic aneurysm	[[0,0,0,1,0,0,0,0,0,0,0,0,0,0]]C1997182	nutritional deficiency in pregnancy	[[1,0,0,0,0,0,0,0,0,0,0,0,0,0]]C3151237	and autonomic dysfunction cardiac defects hirschsprung disease	[[0,0,0,1,0,0,0,0,0,0,0,0,0,0]]C1332230	aleukemic acute lymphoblastic leukemia,aleukemic lymphoblastic leukemia,aleukemic precursor lymphoblastic leukemia	[[0,0,0,0,0,0,0,0,0,0,0,1,0,0],[0,0,0,0,0,0,0,0,0,0,0,1,0,0],[0,0,0,0,0,0,0,0,0,0,0,1,0,0]]C1290027	disorder of skin and or subcutaneous tissue of neck	[[1,0,0,0,0,0,0,0,0,0,0,0,0,0]]C1853249	spinocerebellar ataxia 28,sca28	[[0,1,0,1,0,0,0,0,0,0,0,0,0,0],[0,0,0,1,0,0,0,0,0,0,0,0,0,0]]C1867717	saposin d,sapd	[[0,0,0,1,0,0,1,0,0,0,0,0,0,0],[0,0,0,1,0,0,0,0,0,0,0,0,0,0]]C1854990	complementation group c molybdenum cofactor deficiency	[[0,0,0,1,0,0,0,0,0,0,0,0,0,0]]C0018614	hashish abuse	[[0,1,0,0,0,0,0,0,0,0,0,0,0,0]]C0339535	congenital stationary night blindness,csnb congenital stationary night blindness,x linked complete csnb,csnb1a,with myopia congenital stationary night blindness,hemeralopia myopia,type 1a congenital stationary night blindness,nbm1,myopia night blindness,x linked congenital stationary night blindness,type 1 congenital stationary night blindness	[[1,1,0,0,0,0,0,0,0,0,0,0,0,0],[1,0,0,0,0,0,0,0,0,0,0,0,0,0],[0,0,0,1,0,0,0,0,0,0,0,0,0,0],[0,0,0,1,0,0,0,0,0,0,0,0,0,0],[0,1,0,1,0,0,0,0,0,0,0,0,0,0],[0,1,0,1,0,0,0,0,0,0,0,0,0,0],[0,0,0,1,0,0,0,0,0,0,0,0,0,0],[0,0,0,1,0,0,0,0,0,0,0,0,0,0],[0,1,0,1,0,0,0,0,0,0,0,0,0,0],[0,1,0,0,0,0,0,0,0,0,0,0,0,0],[0,1,0,0,0,0,0,0,0,0,0,0,0,0]]C0339537	cone monochromatism,blue cone monochromatism,bcm,blue mono cone monochromatic type colorblindness,cbbm,color blindness blue mono cone monochromatic type,blue cone monochromacy	[[1,0,0,0,0,0,0,0,0,0,0,0,0,0],[1,0,0,1,0,0,0,0,0,0,0,0,0,0],[0,0,0,1,0,0,0,0,0,0,0,0,0,0],[0,0,0,1,0,0,0,0,0,0,0,0,0,0],[0,0,0,1,0,0,0,0,0,0,0,0,0,0],[0,1,0,0,0,0,0,0,0,0,0,0,0,0],[0,0,0,1,0,0,0,0,0,0,0,0,0,0]]C0339530	progressive cone rod dystrophy,cone rod retinal dystrophy,progressive cone dystrophy	[[1,0,0,0,0,0,0,0,0,0,0,0,0,0],[1,0,0,0,0,0,0,0,0,0,0,0,0,0],[0,0,0,0,0,0,0,0,0,0,0,0,0,1]]C0152268	nodular sclerosis hodgkins disease,hodgkins nodular sclerosis,nodular sclerosis,nodular sclerosis hodgkin lymphoma,nodular sclerosis classical hodgkin lymphoma,nshl,nshd,nschl	[[1,0,0,0,1,0,0,0,0,0,0,1,0,1],[0,0,0,0,0,0,0,0,0,0,0,1,0,0],[0,0,0,0,0,0,0,1,0,0,0,0,0,0],[1,0,0,0,0,0,0,0,0,0,0,1,0,0],[1,0,0,0,0,0,0,0,0,0,0,1,0,0],[0,0,0,0,0,0,0,0,0,0,0,1,0,0],[0,0,0,0,0,0,0,0,0,0,0,1,0,0],[0,0,0,0,0,0,0,0,0,0,0,1,0,0]]C1709884	regional adrenal gland pheochromocytoma,regional adrenal gland chromaffin neoplasm,regional adrenal gland chromaffin paraganglioma,regional adrenal gland chromaffin tumor,regional adrenal gland chromaffinoma	[[0,0,0,0,0,0,0,0,0,0,0,1,0,0],[0,0,0,0,0,0,0,0,0,0,0,1,0,0],[0,0,0,0,0,0,0,0,0,0,0,1,0,0],[0,0,0,0,0,0,0,0,0,0,0,1,0,0],[0,0,0,0,0,0,0,0,0,0,0,1,0,0]]C0152264	familial erythrocytosis,familial polycythemia,familial polycythaemia,benign familial polycythemia,primary familial erythrocytosis,hereditary pure erythrocytosis,familiar polycythemia	[[1,0,0,0,0,0,0,0,0,0,0,0,0,0],[0,0,0,0,1,0,0,0,0,0,0,0,0,0],[1,0,0,0,0,0,0,0,0,0,0,0,0,0],[0,0,0,0,0,0,0,0,0,0,1,0,0,1],[0,0,0,0,0,0,0,0,0,0,1,0,0,0],[1,0,0,0,0,0,0,0,0,0,0,0,0,0],[0,0,0,0,0,0,0,0,0,0,0,1,0,0]]C0152267	lymphocyte depletion hodgkin lymphoma,lymphocytic depletion hodgkins disease,lymphocyte depletion classical hodgkin lymphoma,ldhl,ldchl	[[1,1,0,0,0,0,0,0,0,0,0,1,0,0],[1,0,0,0,0,0,0,0,0,0,0,1,0,1],[1,0,0,0,0,0,0,0,0,0,0,1,0,0],[0,0,0,0,0,0,0,0,0,0,0,1,0,0],[0,0,0,0,0,0,0,0,0,0,0,1,0,0]]C0152266	mixed cellularity hodgkin lymphoma,mixed cellularity hodgkins disease,mixed cellularity m hodgkins disease,mixed cellularity classical hodgkin lymphoma,mchl,mcchl	[[1,0,0,0,0,0,0,0,0,0,0,1,0,0],[1,0,0,0,0,0,0,0,0,0,0,1,0,0],[1,0,0,0,0,0,0,0,0,0,0,0,0,0],[1,0,0,0,0,0,0,0,0,0,0,1,0,0],[0,0,0,0,0,0,0,0,0,0,0,1,0,0],[0,0,0,0,0,0,0,0,0,0,0,1,0,0]]C0152263	osteitis condensans,sclerosing osteitis,condensing osteitis,focal sclerosing osteomyelitis	[[0,0,0,0,1,0,0,1,0,0,0,0,0,0],[1,0,0,0,0,0,0,1,0,0,0,0,0,0],[1,0,0,0,0,0,0,1,0,0,0,0,0,0],[1,0,0,0,0,0,0,0,0,0,0,0,0,0]]C0152262	cystitis cystica	[[0,0,0,0,1,0,0,0,0,0,0,0,0,0]]C1456502	pneumococcal pericarditis	[[0,0,0,0,0,0,0,0,0,0,0,0,0,1]]C1456503	staphylococcal pericarditis	[[0,0,0,0,0,0,0,0,0,0,0,0,0,1]]C0571079	drug groups and agents primarily acting on the skin allergy	[[1,0,0,0,0,0,0,0,0,0,0,0,0,0]]C0571078	allergy to inhaled corticosteroids	[[1,0,0,0,0,0,0,0,0,0,0,0,0,0]]C0005741	blepharitis,blepharitides,eyelid inflammation,tarsitis,inflammation of lid margin	[[0,1,1,0,0,0,0,0,0,1,0,0,0,0],[0,1,0,0,0,0,0,0,0,0,0,0,0,0],[1,0,0,0,0,0,0,1,0,0,1,0,0,0],[0,0,0,0,0,0,0,1,0,0,1,0,0,0],[1,0,0,0,0,0,0,0,0,0,0,0,0,0]]C0024121	lung neoplasms,pulmonary neoplasms,tumor of lung,tumour of lung	[[1,1,1,0,0,0,0,1,0,1,0,1,1,0],[0,1,0,0,0,0,0,1,0,0,0,0,1,0],[1,0,0,0,0,0,0,1,0,0,1,1,0,0],[1,0,0,0,0,0,0,1,0,0,0,0,0,0]]C0005743	blepharoconjunctivitis	[[0,0,1,0,0,0,0,1,0,0,0,0,0,0]]C0005742	blepharochalasis,dermatolysis palpebrarum,fuchs syndrome ii	[[0,0,0,1,0,0,0,1,0,0,0,0,0,0],[1,0,0,0,0,0,0,0,0,0,0,0,0,0],[1,0,0,0,0,0,0,0,0,0,0,0,0,0]]C0571077	bronchodilator preparations allergy	[[1,0,0,0,0,0,0,0,0,0,0,0,0,0]]C0005744	blepharophimosis,blepharophimoses	[[0,0,0,1,0,0,0,1,0,0,0,0,0,0],[0,1,0,0,0,0,0,0,0,0,0,0,0,0]]C0571075	choline theophyllinate allergy	[[1,0,0,0,0,0,0,0,0,0,0,0,0,0]]C0571074	aminophylline allergy	[[1,0,0,0,0,0,0,0,0,0,0,0,0,0]]C0434720	temporomandibular subluxation,subluxation of temporomandibular joint,tmj subluxation of temporomandibular joint	[[1,0,0,0,0,0,0,0,0,0,0,0,0,0],[1,0,0,0,0,0,0,0,0,0,0,0,0,0],[1,0,0,0,0,0,0,0,0,0,0,0,0,0]]C2750850	glioma susceptibility 1,glm1	[[0,0,0,1,0,0,0,0,0,0,0,0,0,0],[0,0,0,1,0,0,0,0,0,0,0,0,0,0]]C3164026	melanocytic nevus of skin of chest,melanocytic naevus of skin of chest	[[1,0,0,0,0,0,0,0,0,0,0,0,0,0],[1,0,0,0,0,0,0,0,0,0,0,0,0,0]]C1853247	autosomal dominant spastic paraplegia 31,spg31	[[0,0,0,1,0,0,0,0,0,0,0,0,0,0],[0,0,0,1,0,0,0,0,0,0,0,0,0,0]]C0241870	schistosomal dysentery	[[0,0,0,0,0,0,0,0,0,0,1,0,0,0]]C0241873	impetiginous eczema	[[0,0,0,0,0,0,0,0,0,0,1,0,0,0]]C0241872	constitutional eczema,endogenous eczema	[[1,0,0,0,0,0,0,1,0,0,1,0,0,0],[1,0,0,0,0,0,0,1,0,0,0,0,0,0]]C0241875	stress edema	[[0,0,0,0,0,0,0,0,0,0,1,0,0,0]]C0241877	pulmonary bullous emphysema	[[0,0,0,0,0,0,0,0,0,0,1,0,0,0]]C0241876	obstructive emphysema,obstructive lung or pulmonary emphysema	[[1,0,0,0,0,0,0,1,0,0,1,0,0,1],[0,0,0,0,0,0,0,0,0,0,0,0,0,1]]C0086457	industrial dermatosis,industrial dermatoses	[[0,1,0,0,0,0,0,0,0,0,0,0,0,0],[0,1,0,0,0,0,0,0,0,0,0,0,0,0]]C1559553	eye infection adverse event documented clinically or microbiologically with grade 3 or 4 neutrophils,eye infection documented clinically or microbiologically with grade 3 or 4 neutrophils anc 1 0 x 10e9 l	[[0,0,0,0,0,0,0,0,0,0,0,1,0,0],[0,0,0,0,0,0,0,0,0,0,0,1,0,0]]C0587254	esophageal varices in alcoholic cirrhosis of the liver,oesophageal varices in alcoholic cirrhosis of the liver	[[1,0,0,0,0,0,0,0,0,0,0,0,0,0],[1,0,0,0,0,0,0,0,0,0,0,0,0,0]]C1263835	infectious disorder of the fetus,infectious disorder of the foetus	[[1,0,0,0,0,0,0,0,0,0,0,0,0,0],[1,0,0,0,0,0,0,0,0,0,0,0,0,0]]C0348593	subsequent myocardial infarction,reinfarction of myocardium	[[1,0,0,0,0,0,0,0,0,0,0,0,0,0],[1,0,0,0,0,0,0,0,0,0,0,0,0,0]]C0334669	lymphosarcoma cell leukemia	[[0,0,0,0,0,0,0,0,0,0,0,0,0,1]]C0334667	aleukemic lymphoid leukemia,aleukaemic lymphoid leukaemia,aleukemic lymphatic leukemia,aleukemic lymphocytic leukemia,aleukemic lymphogenous leukemia	[[1,0,0,0,0,0,0,0,0,0,0,1,0,1],[1,0,0,0,0,0,0,0,0,0,0,0,0,0],[0,0,0,0,0,0,0,0,0,0,0,1,0,1],[0,0,0,0,0,0,0,0,0,0,0,1,0,1],[0,0,0,0,0,0,0,0,0,0,0,1,0,0]]C0334664	mast cell neoplasm,mastocytosis,mast cell tumor,mast cell tumour,mastocytoma,mast cell proliferative disease	[[1,0,0,0,0,0,0,0,0,1,0,1,0,0],[0,0,0,0,0,0,0,0,0,0,0,1,0,0],[1,0,0,0,0,0,0,1,0,0,0,1,0,1],[1,0,0,0,0,0,0,0,0,0,0,0,0,0],[0,0,0,0,0,0,0,1,0,0,0,0,0,0],[0,0,0,0,0,0,0,0,0,0,0,1,0,0]]C0334663	histiocytic sarcoma,histiocytic lymphoma,true histiocytic lymphoma,m true histiocytic lymphoma,true malignant histiocytosis,true malignant histiocytoses	[[1,1,0,0,0,0,0,1,0,0,0,0,1,0],[0,0,0,0,0,0,0,0,0,0,0,1,0,0],[1,1,0,0,0,0,0,0,0,0,0,0,1,0],[1,0,0,0,0,0,0,0,0,0,0,0,0,0],[0,1,0,0,0,0,0,0,0,0,0,0,1,0],[0,1,0,0,0,0,0,0,0,0,0,0,0,0]]C0334660	angioendotheliomatosis,malignant angioendotheliomatosis,intravascular lymphomatosis,angiotropic lymphoma,intravascular large b cell lymphoma,intravascular b cell lymphoma,angiotropic large cell lymphoma	[[1,0,0,0,0,0,0,1,0,0,0,0,0,0],[1,0,0,0,0,0,0,0,0,0,0,1,0,0],[1,0,0,0,0,0,0,1,0,0,0,0,0,0],[1,0,0,0,0,0,0,0,0,0,0,1,0,0],[1,0,0,0,0,0,0,0,0,0,0,1,0,0],[1,0,0,0,0,0,0,0,0,0,0,1,0,0],[0,0,0,0,0,0,0,0,0,0,0,1,0,0]]C0275778	lyme carditis	[[1,0,0,0,0,0,0,1,0,0,0,0,0,0]]C0008049	chickenpox,varicella,varicella infection,chicken pox	[[0,0,1,0,0,0,0,1,0,0,1,0,0,1],[0,0,0,0,0,0,0,1,0,0,1,0,1,1],[1,0,0,0,0,0,0,1,0,0,0,0,0,0],[1,0,0,0,0,0,0,1,0,0,1,0,1,0]]C0275771	infection by borrelia turicatae	[[1,0,0,0,0,0,0,0,0,0,0,0,0,0]]C0275770	relapsing fever of central and or south africa,infection by borrelia duttonii	[[1,0,0,0,0,0,0,0,0,0,0,0,0,0],[1,0,0,0,0,0,0,0,0,0,0,0,0,0]]C0275773	relapsing fever of the caucasus,infection by borrelia caucasica	[[1,0,0,0,0,0,0,0,0,0,0,0,0,0],[1,0,0,0,0,0,0,0,0,0,0,0,0,0]]C0275772	relapsing fever of central and or south america,infection by borrelia venezuelensis	[[1,0,0,0,0,0,0,0,0,0,0,0,0,0],[1,0,0,0,0,0,0,0,0,0,0,0,0,0]]C0275775	relapsing fever of western united states,infection by borrelia parkeri	[[1,0,0,0,0,0,0,0,0,0,0,0,0,0],[1,0,0,0,0,0,0,0,0,0,0,0,0,0]]C0275774	relapsing fever of north africa	[[1,0,0,0,0,0,0,0,0,0,0,0,0,0]]C0008043	chiari frommel syndrome,frommels disease,pregnancy related a g syndrome,lactation with atrophy of uterus,persistent postpartum amenorrhea galactorrhea syndrome,persistent postpartum amenorrhoea galactorrhoea syndrome	[[1,1,0,0,0,0,0,1,0,0,1,0,1,0],[1,1,0,0,0,0,0,1,0,0,0,0,1,0],[1,0,0,0,0,0,0,0,0,0,0,0,0,0],[0,0,0,0,0,0,0,0,0,0,1,0,0,0],[1,0,0,0,0,0,0,0,0,0,0,0,0,0],[1,0,0,0,0,0,0,0,0,0,0,0,0,0]]C0275776	relapsing fever of iran and or central asia,infection by borrelia latyschewii	[[1,0,0,0,0,0,0,0,0,0,0,0,0,0],[1,0,0,0,0,0,0,0,0,0,0,0,0,0]]C1290191	abscess of nasal orbit complex	[[1,0,0,0,0,0,0,0,0,0,0,0,0,0]]C1290190	abscess of temporal bone	[[1,0,0,0,0,0,0,0,0,0,0,0,0,0]]C1290193	disorder with defective osteoid mineralization,disorder with defective osteoid mineralisation	[[1,0,0,0,0,0,0,0,0,0,0,0,0,0],[1,0,0,0,0,0,0,0,0,0,0,0,0,0]]C1290192	abscess of bone of skull	[[1,0,0,0,0,0,0,0,0,0,0,0,0,0]]C1260879	autoimmune progesterone dermatitis,autoimmune progesterone urticaria,progesterone dermatitis,autoimmune progesterone dermatitis urticaria	[[1,0,0,0,0,0,0,0,0,0,0,0,0,0],[1,0,0,0,0,0,0,0,0,0,0,0,0,0],[1,0,0,0,0,0,0,1,0,0,0,0,0,0],[1,0,0,0,0,0,0,0,0,0,0,0,0,0]]C1290194	osteoradionecrosis of bone of skull	[[1,0,0,0,0,0,0,0,0,0,0,0,0,0]]C1290197	osteoradionecrosis of the frontal bone	[[1,0,0,0,0,0,0,0,0,0,0,0,0,0]]C0745095	hyperglycemia hyperosmolar	[[0,0,0,0,0,0,0,1,0,0,0,0,0,0]]C1290199	osteoradionecrosis of the sphenoid bone	[[1,0,0,0,0,0,0,0,0,0,0,0,0,0]]C1260874	infective dermatitis	[[0,0,0,0,0,0,1,1,0,0,0,0,0,0]]C1260871	acetonemia,acetonaemia	[[0,0,0,0,0,0,0,0,1,0,0,0,0,0],[1,0,0,0,0,0,0,0,0,0,0,0,0,0]]C1260873	aortic valve disorder,aortic valve disease,aortic valve defect,avd aortic valve disease,aortic valvular disease	[[1,0,0,0,1,0,0,1,0,0,0,1,0,0],[0,0,1,0,1,0,0,1,0,0,0,1,0,0],[0,0,0,0,0,0,0,1,0,0,1,0,0,0],[1,0,0,0,0,0,0,0,0,0,0,0,0,0],[0,0,0,0,0,0,0,1,0,0,0,0,0,0]]C1297997	secondary malignant neoplasm of right ovary	[[1,0,0,0,0,0,0,0,0,0,0,0,0,0]]C1515295	testicular seminoma with syncytiotrophoblastic cells	[[0,0,0,0,0,0,0,0,0,0,0,1,0,0]]C1297990	secondary malignant neoplasm of left ovary	[[1,0,0,0,0,0,0,0,0,0,0,0,0,0]]C1515296	lipid rich variant testicular sertoli cell tumor	[[0,0,0,0,0,0,0,0,0,0,0,1,0,0]]C0745091	hypereosinophilia	[[0,0,0,1,0,0,0,1,0,0,0,0,0,0]]C1264009	chronic cold agglutinin disease associated with b cell neoplasm	[[1,0,0,0,0,0,0,0,0,0,0,0,0,0]]C0730070	disorders of prostheses and implants of the eye	[[1,0,0,0,0,0,0,0,0,0,0,0,0,0]]C1297992	malignant tumor involving left ovary by direct extension from fallopian tube,malignant tumour involving left ovary by direct extension from fallopian tube	[[1,0,0,0,0,0,0,0,0,0,0,0,0,0],[1,0,0,0,0,0,0,0,0,0,0,0,0,0]]C0477633	cervical disc disorder,cervical disc disease,cervical spine disc disease	[[1,0,0,0,0,0,0,0,0,0,0,0,0,0],[0,0,1,0,0,0,0,1,0,0,0,0,0,0],[0,0,0,0,0,0,0,1,0,0,0,0,0,0]]C1515290	testicular monophasic choriocarcinoma	[[0,0,0,0,0,0,0,0,0,0,0,1,0,0]]C1297993	malignant tumor involving left ovary by direct extension from right ovary,malignant tumour involving left ovary by direct extension from right ovary	[[1,0,0,0,0,0,0,0,0,0,0,0,0,0],[1,0,0,0,0,0,0,0,0,0,0,0,0,0]]C1290074	chronic mucositis	[[1,0,0,0,0,0,0,0,0,0,0,0,0,0]]C1515291	testicular sclerosing sertoli cell tumor	[[0,0,0,0,0,0,0,0,0,0,0,1,0,0]]C0342763	glycerol intolerance	[[1,0,0,0,0,0,0,0,0,0,0,0,0,0]]C0342762	disorder of glycerol metabolism	[[1,0,0,0,0,0,0,1,0,0,0,0,0,0]]C0272279	amegakaryocytic thrombocytopenia with congenital malformation	[[1,0,0,0,0,0,0,0,0,0,0,0,0,0]]C0272278	congenital thrombocytopenia,congenital and hereditary thrombocytopenia	[[0,0,0,1,0,0,0,0,0,0,0,0,0,0],[0,0,0,0,0,0,0,0,0,0,0,0,0,1]]C0342765	d glyceric aciduria	[[1,0,0,1,0,0,0,0,0,0,0,0,0,0]]C0342764	disorder of glycerate metabolism	[[1,0,0,0,0,0,0,0,0,0,0,0,0,0]]C0272275	heparin induced thrombocytopenia with thrombosis,white clot syndrome,heparin associated thrombotic thrombocytopenia	[[1,0,0,0,0,0,0,0,0,0,0,0,0,0],[1,0,0,0,0,0,0,0,0,0,0,0,0,0],[1,0,0,0,0,0,0,0,0,0,0,0,0,0]]C0272274	familial hemorrhagic diathesis,familial haemorrhagic diathesis	[[0,0,0,0,0,0,0,0,0,0,0,0,0,1],[1,0,0,0,0,0,0,0,0,0,0,0,0,0]]C0272277	thrombocytopenia due to defective platelet production	[[1,0,0,0,0,0,0,0,0,0,0,0,0,0]]C0272276	thrombocytopenia due to diminished platelet production	[[1,0,0,0,0,0,0,0,0,0,0,0,0,0]]C0272271	blood coagulation disorder due to liver disease	[[1,0,0,0,0,0,0,0,0,0,0,0,0,0]]C1304069	constitutional factors as co factor in hand eczema	[[1,0,0,0,0,0,0,0,0,0,0,0,0,0]]C0272273	hemorrhagic disorder due to hyperheparinemia,haemorrhagic disorder due to hyperheparinaemia	[[1,0,0,0,0,0,0,0,0,0,0,0,0,0],[1,0,0,0,0,0,0,0,0,0,0,0,0,0]]C0272272	systemic fibrinogenolysis	[[1,0,0,0,0,0,0,0,0,0,0,0,0,0]]C3179508	thumb absent or hypoplastic	[[0,1,0,0,0,0,0,0,0,0,0,0,0,0]]C3179509	keratoactinomycosis	[[0,1,0,0,0,0,0,0,0,0,0,0,0,0]]C1304068	chronic relapsing vesiculosquamous hand eczema	[[1,0,0,0,0,0,0,0,0,0,0,0,0,0]]C0157646	antepartum engorgement of breast associated with childbirth	[[0,0,0,0,1,0,0,0,0,0,0,0,0,0]]C1865870	autosomal recessive 18 deafness,dfnb18	[[0,0,0,1,0,0,0,0,0,0,0,0,0,0],[0,0,0,1,0,0,0,0,0,0,0,0,0,0]]C1297998	malignant tumor involving right ovary by direct extension from endometrium,malignant tumour involving right ovary by direct extension from endometrium	[[1,0,0,0,0,0,0,0,0,0,0,0,0,0],[1,0,0,0,0,0,0,0,0,0,0,0,0,0]]C1865872	nephronophthisis 2,nph2,nphp2,infantile nephronophthisis	[[0,0,0,1,0,0,0,0,0,0,0,0,0,0],[0,0,0,1,0,0,0,0,0,0,0,0,0,0],[0,0,0,1,0,0,0,0,0,0,0,0,0,0],[1,0,0,0,0,0,0,0,0,0,0,0,0,0]]C1290071	disorder of oral mucous membrane,disease of oral mucosa	[[1,0,0,0,0,0,0,0,0,0,0,0,0,0],[1,0,0,0,0,0,0,0,0,0,0,0,0,0]]C1297999	malignant tumor involving right ovary by direct extension from fallopian tube,malignant tumour involving right ovary by direct extension from fallopian tube	[[1,0,0,0,0,0,0,0,0,0,0,0,0,0],[1,0,0,0,0,0,0,0,0,0,0,0,0,0]]C2930748	combat stress disorders	[[0,1,0,0,0,0,0,0,0,0,0,0,0,0]]C1260216	autonomic central nervous system diseases	[[0,1,0,0,0,0,0,0,0,0,0,0,0,0]]C0856009	stage ii supradiaphragmatic nodular sclerosis classical hodgkin lymphoma,stage ii supradiaphragmatic nodular sclerosis hodgkins disease,stage ii nodular sclerosis hodgkins disease above the diaphragm,stage ii nodular sclerosis hodgkins lymphoma above the diaphragm,stage ii supradiaphragmatic nodular sclerosis hodgkin lymphoma	[[0,0,0,0,0,0,0,0,0,0,0,1,0,0],[0,0,0,0,0,0,0,0,0,0,0,1,0,0],[0,0,0,0,0,0,0,0,0,0,0,1,0,0],[0,0,0,0,0,0,0,0,0,0,0,1,0,0],[0,0,0,0,0,0,0,0,0,0,0,1,0,0]]C0341142	cricopharyngeal disorder	[[1,0,0,0,0,0,0,0,0,0,0,0,0,0]]C0341143	cricopharyngeal incoordination	[[1,0,0,0,0,0,0,0,0,0,0,0,0,0]]C2118439	antibiotic associated staphylococcal colitis	[[0,0,0,0,0,0,1,0,0,0,0,0,0,0]]C0489970	cortical and zonular cataract,congenital cortical and zonular cataract	[[1,0,0,0,0,0,0,0,0,0,0,0,0,0],[0,0,0,0,1,0,0,0,0,0,0,0,0,0]]C2675750	autosomal dominant 3a deafness,dfna3a	[[0,0,0,1,0,0,1,0,0,0,0,0,0,0],[0,0,0,1,0,0,0,0,0,0,0,0,0,0]]C1263999	sickle cell hemoglobin lepore disease,sickle cell haemoglobin lepore disease,sickle cell hb lepore disease	[[1,0,0,0,0,0,0,0,0,0,0,0,0,0],[1,0,0,0,0,0,0,0,0,0,0,0,0,0],[1,0,0,0,0,0,0,0,0,0,0,0,0,0]]C1263998	sickle cell trait with coexistent alpha thalassemia,sickle cell trait with coexistent alpha thalassaemia	[[1,0,0,0,0,0,0,0,0,0,0,0,0,0],[1,0,0,0,0,0,0,0,0,0,0,0,0,0]]C1855114	methylmalonic aciduria due to methylmalonyl coa mutase deficiency,mut type methylmalonic aciduria	[[0,0,0,1,0,0,0,0,0,0,0,0,0,0],[0,0,0,1,0,0,0,0,0,0,0,0,0,0]]C1263992	toxic methemoglobinemia with cyanosis,toxic methaemoglobinaemia with cyanosis	[[1,0,0,0,0,0,0,0,0,0,0,0,0,0],[1,0,0,0,0,0,0,0,0,0,0,0,0,0]]C1263991	cytochrome b 3 deficiency,cytochrome b sub 3 sub deficiency,cytochrome b3 deficiency	[[1,0,0,0,0,0,0,0,0,0,0,0,0,0],[1,0,0,0,0,0,0,0,0,0,0,0,0,0],[1,0,0,0,0,0,0,0,0,0,0,0,0,0]]C1263990	enzymatic type hereditary methemoglobinemia,enzymatic type hereditary methaemoglobinaemia	[[1,0,0,0,0,0,0,0,0,0,0,0,0,0],[1,0,0,0,0,0,0,0,0,0,0,0,0,0]]C1263997	sickle cell anemia with coexistent alpha thalassemia,sickle cell anaemia with coexistent alpha thalassaemia	[[1,0,0,0,0,0,0,0,0,0,0,0,0,0],[1,0,0,0,0,0,0,0,0,0,0,0,0,0]]C1263996	sickle cell beta 0 thalassemia,sickle cell beta 0 thalassaemia,sickle cell beta sup 0 sup thalassemia,sickle cell beta sup 0 sup thalassaemia,sickle cell beta0 thalassemia,sickle cell beta0 thalassaemia	[[1,0,0,0,0,0,0,0,0,0,0,0,0,0],[1,0,0,0,0,0,0,0,0,0,0,0,0,0],[1,0,0,0,0,0,0,0,0,0,0,0,0,0],[1,0,0,0,0,0,0,0,0,0,0,0,0,0],[1,0,0,0,0,0,0,0,0,0,0,0,0,0],[1,0,0,0,0,0,0,0,0,0,0,0,0,0]]C1263995	acquired hemoglobinopathy,acquired haemoglobinopathy	[[1,0,0,0,0,0,0,0,0,0,0,0,0,0],[1,0,0,0,0,0,0,0,0,0,0,0,0,0]]C0085437	bacterial meningitis,bacterial meningitides,bm bacterial meningitis	[[0,0,0,0,1,0,0,1,0,0,1,0,1,1],[0,0,0,0,0,0,0,1,0,0,0,0,1,0],[1,0,0,0,0,0,0,0,0,0,0,0,0,0]]C0085436	cryptococcal meningitis,cryptococcal meningitides,meningitis torula,meningitis due to cryptococcus,crypto meningitis	[[0,0,0,0,1,0,0,1,1,0,0,0,1,0],[0,1,0,0,0,0,0,0,0,0,0,0,0,0],[0,0,0,0,0,0,0,0,1,0,0,0,0,0],[1,0,0,0,0,0,0,0,0,0,0,0,0,0],[0,0,0,0,0,0,0,1,0,0,0,0,0,0]]C0085435	reactive arthritis,reactive arthritides,postinfectious arthritis,postinfectious arthritides,arthritis occurring after infection,post infective arthritis,post infective arthropathy,post bacterial arthropathy,post infectious arthritis,post infectious arthritides	[[0,1,0,0,0,0,0,1,0,0,0,0,1,1],[0,1,0,0,0,0,0,0,0,0,0,0,0,0],[0,0,0,0,0,0,0,0,0,0,0,0,1,0],[0,1,0,0,0,0,0,0,0,0,0,0,0,0],[1,0,0,0,0,0,0,0,0,0,0,0,0,0],[1,0,0,0,0,0,0,0,0,0,0,0,0,0],[1,0,0,0,0,0,0,0,0,0,0,0,0,0],[1,0,0,0,0,0,0,0,0,0,0,0,0,0],[0,1,0,0,0,0,0,0,0,0,0,0,1,0],[0,1,0,0,0,0,0,0,0,0,0,0,0,0]]C0085434	bacillary angiomatosis,bacillary angiomatoses,bacillary epithelioid angiomatosis,bacillary epithelioid angiomatoses,epithelioid angiomatosis,epithelioid angiomatoses	[[1,0,0,0,0,0,0,1,0,0,0,0,1,0],[0,1,0,0,0,0,0,0,0,0,0,0,0,0],[0,1,0,0,0,0,0,0,0,0,0,0,1,0],[0,1,0,0,0,0,0,0,0,0,0,0,0,0],[0,1,0,0,0,0,0,0,0,0,0,0,1,0],[0,1,0,0,0,0,0,0,0,0,0,0,0,0]]C0598716	panmural cystitis	[[1,0,0,0,0,0,0,0,0,1,0,0,0,0]]C0085438	fungal meningitis,fungal meningitides	[[1,0,0,0,0,0,0,1,0,0,0,0,1,0],[0,1,0,0,0,0,0,0,0,0,0,0,0,0]]C1562859	chronic progressive coccidioidal pneumonia	[[1,0,0,0,0,0,0,0,0,0,0,0,0,0]]C2931407	alopecia macular degeneration growth retardation	[[0,1,0,0,0,0,0,0,0,0,0,0,0,0]]C0268788	hereditary tubulointerstitial disorder	[[1,0,0,0,0,0,0,0,0,0,0,0,0,0]]C0268789	familial interstitial nephritis	[[1,0,0,0,0,0,0,0,0,0,0,0,0,0]]C1275433	facial seborrheic dermatitis,facial seborrhoeic dermatitis,facial seborrheic eczema,facial seborrhoeic eczema	[[1,0,0,0,0,0,0,0,0,0,0,0,0,0],[1,0,0,0,0,0,0,0,0,0,0,0,0,0],[1,0,0,0,0,0,0,0,0,0,0,0,0,0],[1,0,0,0,0,0,0,0,0,0,0,0,0,0]]C1290073	acute mucositis	[[1,0,0,0,0,0,0,0,0,0,0,0,0,0]]C0268783	light chain nephropathy	[[1,0,0,0,0,0,0,0,0,0,0,0,0,0]]C0268780	idiopathic acute interstitial nephritis	[[1,0,0,0,0,0,0,0,0,0,0,0,0,0]]C0268781	idiopathic granulomatous interstitial nephropathy	[[1,0,0,0,0,0,0,0,0,0,0,0,0,0]]C0268786	salt wasting syndrome of infancy	[[1,0,0,0,0,0,0,0,0,0,0,0,0,0]]C0524799	hyperlucent lung,hyperlucent thorax	[[0,1,0,0,0,0,0,1,0,0,0,0,1,0],[0,1,0,0,0,0,0,0,0,0,0,0,1,0]]C0268784	myeloma kidney,myeloma cast nephropathy	[[1,0,0,0,0,0,0,1,0,0,0,0,0,0],[1,0,0,0,0,0,0,1,0,0,0,0,0,0]]C0268785	salt losing nephropathy,pseudo addisons disease,salt losing nephritis,thorns syndrome	[[1,0,0,0,0,0,0,1,0,0,0,0,0,0],[1,0,0,0,0,0,0,0,0,0,0,0,0,0],[1,0,0,0,0,0,0,1,0,0,0,0,0,0],[1,0,0,0,0,0,0,1,0,0,0,0,0,0]]C0451872	post surgical malabsorption osteoporosis	[[1,0,0,0,0,0,0,0,0,0,0,0,0,0]]C0079765	follicular small cleaved cell lymphoma,poorly differentiated nodular lymphocytic lymphoma,follicular small lymphoid lymphoma,nodular poorly differentiated lymphocytic malignant lymphoma,small follicular center cell lymphoma,follicular malignant lymphoma small cleaved cell	[[0,1,0,0,0,0,0,0,0,0,0,0,0,0],[0,1,0,0,0,0,0,0,0,0,0,0,0,0],[0,1,0,0,0,0,0,0,0,0,0,0,0,0],[1,0,0,0,0,0,0,0,0,0,0,0,0,0],[0,1,0,0,0,0,0,0,0,0,0,0,0,0],[1,0,0,0,0,0,0,0,0,0,0,0,0,0]]C0341149	mid esophageal pulsion diverticulum,mid oesophageal pulsion diverticulum	[[1,0,0,0,0,0,0,0,0,0,0,0,0,0],[1,0,0,0,0,0,0,0,0,0,0,0,0,0]]C0451876	adult osteomalacia due to malnutrition	[[1,0,0,0,0,0,0,0,0,0,0,0,0,0]]C0451874	senile osteomalacia	[[1,0,0,0,0,0,0,0,0,0,0,0,0,0]]C0451875	adult osteomalacia due to malabsorption	[[1,0,0,0,0,0,0,0,0,0,0,0,0,0]]C1883411	tumor replacement	[[0,0,0,0,0,0,0,0,0,0,0,1,0,0]]C1275432	perianal seborrheic dermatitis,perianal seborrhoeic dermatitis,perianal seborrheic eczema,perianal seborrhoeic eczema	[[1,0,0,0,0,0,0,0,0,0,0,0,0,0],[1,0,0,0,0,0,0,0,0,0,0,0,0,0],[1,0,0,0,0,0,0,0,0,0,0,0,0,0],[1,0,0,0,0,0,0,0,0,0,0,0,0,0]]C1536085	geographic atrophy,dry macular degeneration	[[0,1,0,0,0,0,0,0,0,0,0,0,1,0],[0,1,0,0,0,0,0,0,0,0,0,0,1,0]]C1851120	with or without gingival hyperplasia generalized hypertrichosis terminalis,with hypertrichosis gingival fibromatosis,microdeletion 17q24 2 q24 3 syndrome,chromosome 17q24 2 q24 3 deletion syndrome	[[0,0,0,1,0,0,0,0,0,0,0,0,0,0],[0,0,0,1,0,0,0,0,0,0,0,0,0,0],[0,0,0,1,0,0,0,0,0,0,0,0,0,0],[0,0,0,1,0,0,0,0,0,0,0,0,0,0]]C1851124	hereditary desmoid disease,fif,familial infiltrative fibromatosis	[[0,1,0,1,0,0,0,0,0,0,0,0,0,0],[0,0,0,1,0,0,0,0,0,0,0,0,0,0],[0,1,0,1,0,0,0,0,0,0,0,0,0,0]]C3164179	dermatofibroma with monster cells	[[1,0,0,0,0,0,0,0,0,0,0,0,0,0]]C0235573	hemoglobinemia,haemoglobinaemia	[[1,0,0,0,0,0,0,1,1,0,0,0,0,0],[1,0,0,0,0,0,0,0,0,0,0,0,0,0]]C0235571	anemia primaquine sensitivity,anemia primaquine sensitivity type	[[0,0,0,0,0,0,1,0,0,0,0,0,0,0],[0,0,0,0,0,0,0,0,1,0,0,0,0,0]]C2677772	14 hereditary prostate cancer,hpc14	[[0,0,0,1,0,0,0,0,0,0,0,0,0,0],[0,0,0,1,0,0,0,0,0,0,0,0,0,0]]C2677774	age related macular degeneration type 11,armd11,11 age related macular degeneration	[[0,0,0,0,0,0,1,0,0,0,0,0,0,0],[0,0,0,1,0,0,0,0,0,0,0,0,0,0],[0,0,0,1,0,0,0,0,0,0,0,0,0,0]]C0235575	hemolytic reaction,haemolytic reaction	[[0,0,0,0,0,0,0,1,1,0,0,0,0,0],[0,0,0,0,0,0,0,1,0,0,0,0,0,0]]C0235574	intravascular hemolysis,intravascular haemolysis	[[1,0,0,0,0,0,0,1,1,0,0,0,0,0],[1,0,0,0,0,0,0,1,0,0,0,0,0,0]]C0375259	mitral and aortic valve disease	[[0,0,0,0,1,0,1,0,0,0,0,0,0,0]]C1275431	pityriasiform seborrheic dermatitis,pityriasiform seborrhoeic dermatitis,pityriasiform seborrheic eczema,pityriasiform seborrhoeic eczema	[[1,0,0,0,0,0,0,0,0,0,0,0,0,0],[1,0,0,0,0,0,0,0,0,0,0,0,0,0],[1,0,0,0,0,0,0,0,0,0,0,0,0,0],[1,0,0,0,0,0,0,0,0,0,0,0,0,0]]C1262117	fungal keratitis,fungal infection of cornea,mycotic keratitis,keratomycosis	[[1,0,0,0,0,0,0,1,0,0,0,0,0,0],[1,0,0,0,0,0,0,0,0,0,0,0,0,0],[1,0,0,0,0,0,0,1,0,0,0,0,0,0],[1,0,0,0,0,0,0,1,0,0,0,0,0,0]]C1262113	lipohypertrophy	[[0,0,0,0,0,0,0,0,0,0,0,1,0,0]]C0270659	endophlebitis of lateral venous sinus	[[1,0,0,0,0,0,0,0,0,0,0,0,0,0]]C0270658	endophlebitis of cavernous venous sinus	[[1,0,0,0,0,0,0,0,0,0,0,0,0,0]]C0342536	drug induced feminization,drug induced feminisation	[[1,0,0,0,0,0,0,0,0,0,0,0,0,0],[1,0,0,0,0,0,0,0,0,0,0,0,0,0]]C1720861	type 3 familial partial lipodystrophy,fpld3,associated with pparg mutations familial partial lipodystrophy	[[0,1,0,1,0,0,0,0,0,0,0,0,0,0],[0,0,0,1,0,0,0,0,0,0,0,0,0,0],[0,0,0,1,0,0,0,0,0,0,0,0,0,0]]C0270650	phlebitis of intracranial venous sinus	[[1,0,0,0,0,0,0,0,0,0,0,0,0,0]]C0270653	phlebitis of superior sagittal sinus	[[1,0,0,0,0,0,0,0,0,0,0,0,0,0]]C0270652	phlebitis of lateral venous sinus	[[1,0,0,0,0,0,0,0,0,0,0,0,0,0]]C0270655	phlebitis of basilar sinus	[[1,0,0,0,0,0,0,0,0,0,0,0,0,0]]C0270654	phlebitis of inferior sagittal sinus	[[1,0,0,0,0,0,0,0,0,0,0,0,0,0]]C0270657	endophlebitis of intracranial venous sinus	[[1,0,0,0,0,0,0,0,0,0,0,0,0,0]]C0270656	phlebitis of torcular herophili	[[1,0,0,0,0,0,0,0,0,0,0,0,0,0]]C0269785	abo isoimmunization affecting pregnancy,abo isoimmunisation affecting pregnancy,abo isoimmunization in pregnancy,abo isoimmunisation in pregnancy,abo isoimmunization affecting management of mother	[[1,0,0,0,0,0,0,0,0,0,0,0,0,0],[1,0,0,0,0,0,0,0,0,0,0,0,0,0],[1,0,0,0,0,0,0,0,0,0,0,0,0,0],[1,0,0,0,0,0,0,0,0,0,0,0,0,0],[0,0,0,0,0,0,0,0,0,0,0,0,0,1]]C0864705	pustular smallpox	[[0,0,0,0,0,0,0,0,0,0,0,0,0,1]]C0864700	non arthropod borne acute inclusion body encephalitis	[[0,0,0,0,0,0,0,0,0,0,0,0,0,1]]C0864701	non arthropod borne acute necrotizing encephalitis	[[0,0,0,0,0,0,0,0,0,0,0,0,0,1]]C0864702	non arthropod borne epidemic encephalitis	[[0,0,0,0,0,0,0,0,0,0,0,0,0,1]]C0269782	fetal and or placental disorder affecting management of mother,feto placental disorder affecting management of mother,foeto placental disorder affecting management of mother	[[1,0,0,0,0,0,0,0,0,0,0,0,0,0],[1,0,0,0,0,0,0,0,0,0,0,0,0,0],[1,0,0,0,0,0,0,0,0,0,0,0,0,0]]C1517580	invasive small cell carcinoma of the mouse prostate gland	[[0,0,0,0,0,0,0,0,0,0,0,1,0,0]]C0269789	abnormal acid base balance affecting management of mother	[[0,0,0,0,0,0,0,0,0,0,0,0,0,1]]C1857492	craniosynostosis with fibular aplasia	[[0,0,0,1,0,0,0,0,0,0,0,0,0,0]]C0795932	fontaine syndrome	[[0,0,0,0,0,0,0,1,0,0,0,0,0,0]]C0795933	edinburgh malformation syndrome	[[0,0,0,1,0,0,0,0,0,0,0,0,0,0]]C0795936	faciocardiorenal syndrome,eastman bixler syndrome	[[0,1,0,1,0,0,0,0,0,0,0,0,0,0],[0,1,0,1,0,0,0,0,0,0,0,0,0,0]]C0795934	digitorenocerebral syndrome,eronen syndrome,brachydactyly due to absence of distal phalanges,drc syndrome	[[0,0,0,1,0,0,0,0,0,0,0,0,0,0],[0,0,0,1,0,0,0,0,0,0,0,0,0,0],[0,0,0,1,0,0,0,0,0,0,0,0,0,0],[0,0,0,1,0,0,0,0,0,0,0,0,0,0]]C1302820	disorder of skin of head	[[1,0,0,0,0,0,0,0,0,0,0,0,0,0]]C1561617	class iv complete edentulism	[[0,0,0,0,1,0,0,0,0,0,0,0,0,0]]C0795939	aminopterin syndrome sine aminopterin,pseudoaminopterin syndrome,assa	[[0,1,0,1,0,0,0,0,0,0,0,0,0,0],[0,1,0,1,0,0,0,0,0,0,0,0,0,0],[0,0,0,1,0,0,0,0,0,0,0,0,0,0]]C0153354	malignant tumor of anterior two thirds of tongue,malignant tumour of anterior two thirds of tongue,malignant neoplasm of mobile part of tongue	[[1,0,0,0,0,0,0,0,0,0,0,0,0,0],[1,0,0,0,0,0,0,0,0,0,0,0,0,0],[0,0,0,0,0,0,0,0,0,0,0,0,0,1]]C0855226	enema abuse	[[0,0,0,0,0,0,0,1,0,0,0,0,0,0]]C0686457	secondary malignant neoplasm of conjunctiva,metastatic malignant neoplasm to conjunctiva	[[1,0,0,0,0,0,0,0,0,0,0,0,0,0],[1,0,0,0,0,0,0,0,0,0,0,0,0,0]]C0686455	neoplasm of uncertain behavior of conjunctiva,neoplasm of uncertain behaviour of conjunctiva	[[1,0,0,0,0,0,0,0,0,0,0,0,0,0],[1,0,0,0,0,0,0,0,0,0,0,0,0,0]]C0686452	primary malignant neoplasm of hypoglossal nerve	[[1,0,0,0,0,0,0,0,0,0,0,0,0,0]]C0686453	secondary malignant neoplasm of hypoglossal nerve,metastatic malignant neoplasm to hypoglossal nerve	[[1,0,0,0,0,0,0,0,0,0,0,0,0,0],[1,0,0,0,0,0,0,0,0,0,0,0,0,0]]C0348868	listerial endocarditis	[[1,0,0,0,0,0,0,0,0,0,0,0,0,0]]C0348869	non rheumatic tricuspid valve stenosis with insufficiency	[[1,0,0,0,0,0,0,0,0,0,0,0,0,0]]C0348866	atrial septal defect as current complication following acute myocardial infarction	[[1,0,0,0,0,0,0,0,0,0,0,0,0,0]]C0348867	rupture of cardiac wall without hemopericardium as current complication following acute myocardial infarction,rupture of cardiac wall without haemopericardium as current complication following acute myocardial infarction	[[1,0,0,0,0,0,0,0,0,0,0,0,0,0],[1,0,0,0,0,0,0,0,0,0,0,0,0,0]]C0405079	hyperemesis gravidarum with metabolic disturbance not delivered	[[1,0,0,0,0,0,0,0,0,0,0,0,0,0]]C0348862	subsequent myocardial infarction of anterior wall	[[1,0,0,0,0,0,0,0,0,0,0,0,0,0]]C0348863	subsequent myocardial infarction of inferior wall	[[1,0,0,0,0,0,0,0,0,0,0,0,0,0]]C0348860	hypertensive renal disease with renal failure	[[1,0,0,0,0,0,0,0,0,0,0,0,0,0]]C0686459	carcinoma in situ of cornea	[[1,0,0,0,0,0,0,0,0,0,0,0,0,0]]C0270905	median nerve neuritis	[[0,0,0,0,0,0,0,0,0,0,0,0,0,1]]C1302827	benign neoplasm with apocrine differentiation	[[1,0,0,0,0,0,0,0,0,0,0,0,0,0]]C1855809	with intracerebral calcifications renal hypophosphatemia	[[0,0,0,1,0,0,0,0,0,0,0,0,0,0]]C0576801	infected tooth socket	[[1,0,0,0,0,0,0,0,0,0,0,0,0,0]]C0153351	malignant neoplasm of dorsal surface of tongue,malignant neoplasm of dorsal tongue	[[0,0,0,0,1,0,0,0,0,0,0,0,0,0],[0,0,0,0,0,0,0,0,0,0,0,0,0,1]]C1275436	primary polygenic combined hyperlipidemia,primary polygenic combined hyperlipidaemia,primary polygenic type iib combined hyperlipidemia,primary polygenic type iib combined hyperlipidaemia,primary polygenic type iib hyperlipidemia,primary polygenic type iib hyperlipidaemia	[[1,0,0,0,0,0,0,0,0,0,0,0,0,0],[1,0,0,0,0,0,0,0,0,0,0,0,0,0],[1,0,0,0,0,0,0,0,0,0,0,0,0,0],[1,0,0,0,0,0,0,0,0,0,0,0,0,0],[1,0,0,0,0,0,0,0,0,0,0,0,0,0],[1,0,0,0,0,0,0,0,0,0,0,0,0,0]]C0270907	acute radial nerve palsy	[[0,0,0,0,0,0,0,0,0,0,0,0,0,1]]C1855800	with central diabetes insipidus congenital hypopituitarism	[[0,0,0,1,0,0,0,0,0,0,0,0,0,0]]C0007659	cementoma,benign cementoblastoma,periapical cemental dysplasia,periapical cemento osseous dysplasia,cementoblastoma,true cementoma,periradicular cemental dysplasia	[[0,1,0,0,0,0,0,1,0,0,0,1,0,0],[1,0,0,0,0,0,0,0,0,0,0,1,0,0],[1,0,0,0,0,0,0,1,0,0,0,0,0,0],[1,0,0,0,0,0,0,0,0,0,0,0,0,0],[0,0,0,0,0,0,0,1,0,0,0,1,0,0],[1,0,0,0,0,0,0,0,0,0,0,0,0,0],[1,0,0,0,0,0,0,0,0,0,0,0,0,0]]C1882738	rat malignant craniopharyngioma,malignant craniopharyngioma	[[0,0,0,0,0,0,0,0,0,0,0,1,0,0],[0,0,0,0,0,0,0,0,0,0,0,1,0,0]]C0270906	tardy ulnar nerve palsy	[[0,0,0,0,0,0,0,0,0,0,0,0,0,1]]C1859318	myopathic carnitine deficiency	[[0,1,0,1,0,0,0,0,0,0,0,0,0,0]]C1302828	uncertain whether benign or malignant blood vessel neoplasm	[[1,0,0,0,0,0,0,0,0,0,0,0,0,0]]C1859316	cataract alopecia sclerodactyly syndrome,cass,sclerodactyly alopecia cataract,wallis type ppk ca,autosomal recessive palmoplantar keratoderma and congenital alopecia	[[0,1,0,1,0,0,0,0,0,0,0,0,0,0],[0,0,0,1,0,0,0,0,0,0,0,0,0,0],[0,1,0,0,0,0,0,0,0,0,0,0,0,0],[0,0,0,1,0,0,0,0,0,0,0,0,0,0],[0,0,0,1,0,0,0,0,0,0,0,0,0,0]]C1859317	cataract and cardiomyopathy,sengers syndrome	[[0,1,0,1,0,0,0,0,0,0,0,0,0,0],[0,1,0,1,0,0,0,0,0,0,0,0,0,0]]C0751839	frontal gait apraxia	[[0,1,0,0,0,0,0,0,0,0,0,0,0,0]]C0751838	bruns apraxia of gait	[[1,1,0,0,0,0,1,0,0,0,0,0,0,0]]C1859312	camfak syndrome,kyphoscoliosis syndrome failure to thrive microcephaly cataract,cataract microcephaly failure to thrive kyphoscoliosis	[[0,1,0,1,0,0,0,0,0,0,0,0,0,0],[0,0,0,1,0,0,0,0,0,0,0,0,0,0],[0,1,0,0,0,0,0,0,0,0,0,0,0,0]]C0007657	cementitis	[[1,0,0,0,0,0,0,0,0,0,0,0,0,0]]C0023364	leptospirosis,leptospiroses,fort bragg fever,spirochetal jaundice,spirochaetal jaundice,swamp fever,weil disease,infection due to leptospira,red water fever,cane cutters fever,rice field workers disease,harvest fever,mud fever,queensland fever,japanese autumnal fever	[[0,0,0,0,0,0,0,0,0,1,1,0,0,0],[0,1,0,0,0,0,0,1,0,0,0,0,0,0],[0,0,0,0,0,0,0,1,0,0,0,0,0,0],[1,0,0,0,0,0,0,0,0,0,0,0,0,0],[1,0,0,0,0,0,0,1,0,0,0,0,0,0],[1,0,0,0,0,0,0,1,0,0,0,0,0,0],[0,0,0,0,0,0,0,0,0,0,0,1,0,0],[1,0,0,0,0,0,0,0,0,0,0,0,0,0],[1,0,0,0,0,0,0,1,0,0,0,0,0,0],[1,0,0,0,0,0,0,0,0,0,0,0,0,0],[1,0,0,0,0,0,0,0,0,0,0,0,0,0],[1,0,0,0,0,0,0,0,0,0,0,0,0,0],[0,0,0,0,0,0,0,1,0,0,0,0,0,0],[1,0,0,0,0,0,0,1,0,0,0,0,0,0],[1,0,0,0,0,0,0,0,0,0,0,0,0,0]]C0851366	reproductive system and breast disorder class,reproductive system and breast disorders	[[0,0,0,0,0,0,0,0,0,0,0,1,0,0],[0,0,0,0,0,0,0,0,0,0,0,1,0,0]]C0156188	angiodysplasia of intestine with hemorrhage	[[0,0,0,0,1,0,0,0,0,0,0,0,0,0]]C2960633	deep keratitis	[[1,0,0,0,0,0,0,0,0,0,0,0,0,0]]C0851362	general disorders and administration site conditions class,general disorders and administration site conditions	[[0,0,0,0,0,0,0,0,0,0,0,1,0,0],[0,0,0,0,0,0,0,0,0,0,0,1,0,0]]C0851363	puerperium and perinatal conditions pregnancy,puerperium and perinatal condition class pregnancy	[[0,0,0,0,0,0,1,0,0,0,0,1,0,0],[0,0,0,0,0,0,0,0,0,0,0,1,0,0]]C0333027	microlithiasis,microlith	[[1,0,0,0,0,0,0,1,0,0,0,0,0,0],[1,0,0,0,0,0,0,1,0,0,0,0,0,0]]C0599193	fetal macrosomatia	[[0,0,0,0,0,0,0,0,0,1,0,0,0,0]]C1290388	heart valve stenosis and regurgitation,heart valve stenosis and incompetence	[[1,0,0,0,0,0,0,0,0,0,0,0,0,0],[1,0,0,0,0,0,0,0,0,0,0,0,0,0]]C1290389	mitral valve stenosis and regurgitation,mitral valve stenosis and incompetence	[[1,0,0,0,0,0,0,0,0,0,0,0,0,0],[1,0,0,0,0,0,0,0,0,0,0,0,0,0]]C2930959	unilateral loss of facial flushing and sweating with contralateral anhidrosis	[[0,1,0,0,0,0,0,0,0,0,0,0,0,0]]C1691228	cystic kidney diseases,cystic renal diseases	[[1,1,0,0,0,0,0,0,0,0,0,0,1,0],[0,1,0,1,0,0,0,0,0,0,0,0,1,0]]C1270923	functional amblyopia,reversible amblyopia	[[1,0,0,0,0,0,0,0,0,0,0,0,0,0],[1,0,0,0,0,0,0,0,0,0,0,0,0,0]]C2930958	cod	[[0,1,0,0,0,0,0,0,0,0,0,0,0,0]]C0270909	lateral popliteal nerve lesion,common peroneal nerve lesion	[[1,0,0,0,1,0,0,0,0,0,0,0,0,0],[1,0,0,0,0,0,0,0,0,0,0,0,0,1]]C0267435	dietetic sigmoiditis	[[1,0,0,0,0,0,0,0,0,0,0,0,0,0]]C0267434	allergic sigmoiditis	[[1,0,0,0,0,0,0,0,0,0,0,0,0,0]]C0267437	allergic diarrhea,allergic diarrhoea	[[1,0,0,0,0,0,0,0,0,0,0,0,0,0],[1,0,0,0,0,0,0,0,0,0,0,0,0,0]]C0267436	non infective diarrhea,non infective diarrhoea,presumed non infectious diarrhea,presumed non infectious diarrhoea	[[1,0,0,0,0,0,0,0,0,0,0,0,0,0],[1,0,0,0,0,0,0,0,0,0,0,0,0,0],[1,0,0,0,0,0,0,0,0,0,0,0,0,0],[1,0,0,0,0,0,0,0,0,0,0,0,0,0]]C1304229	oral mucosal involvement by linear iga disease	[[1,0,0,0,0,0,0,0,0,0,0,0,0,0]]C0267430	noninfectious colitis,non infective colitis	[[1,0,0,0,0,0,0,0,0,0,0,0,0,0],[1,0,0,0,0,0,0,0,0,0,0,0,0,0]]C0267433	noninfectious sigmoiditis	[[1,0,0,0,0,0,0,0,0,0,0,0,0,0]]C0267432	dietetic colitis	[[1,0,0,0,0,0,0,0,0,0,0,0,0,0]]C0277382	infestation by sarcophaga,sarcophagosis	[[1,0,0,0,0,0,0,0,0,0,0,0,0,0],[1,0,0,0,0,0,0,0,0,0,0,0,0,0]]C0277383	infestation by wohlfahrtia,wohlfahrtiosis	[[1,0,0,0,0,0,0,0,0,0,0,0,0,0],[1,0,0,0,0,0,0,0,0,0,0,0,0,0]]C0277380	infestation by cochliomyia,infestation by callitroga,callitrogosis,cochliomyiosis	[[1,0,0,0,0,0,0,0,0,0,0,0,0,0],[1,0,0,0,0,0,0,0,0,0,0,0,0,0],[1,0,0,0,0,0,0,0,0,0,0,0,0,0],[1,0,0,0,0,0,0,0,0,0,0,0,0,0]]C0277381	infestation by sarcophagidae,infestation by flesh flies,sarcophagidosis	[[1,0,0,0,0,0,0,0,0,0,0,0,0,0],[1,0,0,0,0,0,0,0,0,0,0,0,0,0],[1,0,0,0,0,0,0,0,0,0,0,0,0,0]]C0277386	infestation by hypoderma bovis,ox warble	[[1,0,0,0,0,0,0,0,0,0,0,0,0,0],[1,0,0,0,0,0,0,0,0,0,0,0,0,0]]C0277387	infestation by hypoderma lineatum	[[1,0,0,0,0,0,0,0,0,0,0,0,0,0]]C0277384	infestation by hypodermatinae,hypodermatidosis	[[1,0,0,0,0,0,0,0,0,0,0,0,0,0],[1,0,0,0,0,0,0,0,0,0,0,0,0,0]]C0277385	infestation by hypoderma,hypodermosis	[[1,0,0,0,0,0,0,0,0,0,0,0,0,0],[1,0,0,0,0,0,0,0,0,0,0,0,0,0]]C2930890	asrar facharzt haque syndrome	[[0,1,0,0,0,0,0,0,0,0,0,0,0,0]]C0040038	thromboembolism,thromboembolus,thromboembolism lesion,thromboembolic event,thromboembolic disease,thromboembolic disorder	[[0,1,0,0,0,0,0,1,1,0,0,0,0,0],[1,0,0,0,0,0,0,1,0,0,0,0,0,0],[1,0,0,0,0,0,0,0,0,0,0,0,0,0],[0,0,0,0,0,0,0,0,0,0,0,1,0,0],[1,0,0,0,0,0,0,1,0,0,0,0,0,0],[1,0,0,0,0,0,0,1,0,0,0,0,0,0]]C1827086	undifferentiated large cell carcinomatosis	[[1,0,0,0,0,0,0,0,0,0,0,0,0,0]]C3163885	melanocytic nevus of tongue,melanocytic naevus of tongue,pigmented mole of tongue	[[1,0,0,0,0,0,0,0,0,0,0,0,0,0],[1,0,0,0,0,0,0,0,0,0,0,0,0,0],[1,0,0,0,0,0,0,0,0,0,0,0,0,0]]C1832774	osler rendu weber syndrome 3,type 3 hereditary hemorrhagic telangiectasia,hht3	[[0,1,0,0,0,0,0,0,0,0,0,0,0,0],[0,1,0,1,0,0,0,0,0,0,0,0,0,0],[0,0,0,1,0,0,0,0,0,0,0,0,0,0]]C1290382	bacterial infectious disease of heart	[[1,0,0,0,0,0,0,0,0,0,0,0,0,0]]C1279386	postoperative pneumonia	[[1,0,0,0,0,0,0,0,0,0,0,0,0,0]]C1720137	nocardiosis associated with aids,nocardia infection associated with aids	[[1,0,0,0,0,0,0,0,0,0,0,0,0,0],[1,0,0,0,0,0,0,0,0,0,0,0,0,0]]C1720135	gastrointestinal infection due to klebsiella mobilis	[[1,0,0,0,0,0,0,0,0,0,0,0,0,0]]C2930891	dominant intermediate 1 charcot marie tooth disease	[[0,1,0,0,0,0,0,0,0,0,0,0,0,0]]C1868690	familial hypoadrenocorticism	[[0,1,0,1,0,0,0,0,0,0,0,0,0,0]]C0156000	with perforation and with obstruction acute duodenal ulcer with hemorrhage,with perforation and with obstruction acute duodenal ulcer with haemorrhage	[[1,0,0,0,1,0,0,0,0,0,0,0,0,0],[1,0,0,0,0,0,0,0,0,0,0,0,0,0]]C2930955	hamanishi ueba tsuji syndrome,congenital aplasia of the extensor muscles of the fingers and thumb associated with generalized polyneuropathy	[[0,1,0,0,0,0,0,0,0,0,0,0,0,0],[0,1,0,0,0,0,0,0,0,0,0,0,0,0]]C2930954	halal syndrome,microcephaly cleft palate autosomal dominant	[[0,1,0,0,0,0,0,0,0,0,0,0,0,0],[0,1,0,0,0,0,0,0,0,0,0,0,0,0]]C2987120	intramucosal adenocarcinoma	[[0,0,0,0,0,0,0,0,0,0,0,1,0,0]]C2930957	hantavirosis,hantavirus fever	[[0,1,0,0,0,0,0,0,0,0,0,0,0,0],[0,1,0,0,0,0,0,0,0,0,0,0,0,0]]C2987128	gastrointestinal neuroendocrine carcinoma	[[0,0,0,0,0,0,0,0,0,0,0,1,0,0]]C2930956	hamano tsukamoto syndrome	[[0,1,0,0,0,0,0,0,0,0,0,0,0,0]]C1334608	malignant ovarian luteinized thecoma,malignant ovarian luteinized thecal cell neoplasm,malignant luteinized thecal cell neoplasm of the ovary,malignant ovarian luteinized thecal cell tumor,malignant luteinized thecal cell tumor of the ovary,malignant luteinized thecoma of the ovary	[[0,0,0,0,0,0,0,0,0,0,0,1,0,0],[0,0,0,0,0,0,0,0,0,0,0,1,0,0],[0,0,0,0,0,0,0,0,0,0,0,1,0,0],[0,0,0,0,0,0,0,0,0,0,0,1,0,0],[0,0,0,0,0,0,0,0,0,0,0,1,0,0],[0,0,0,0,0,0,0,0,0,0,0,1,0,0]]C0276161	subacute adenoviral encephalitis,slow virus infection	[[1,0,0,0,0,0,0,0,0,0,0,0,0,0],[1,0,0,0,0,0,0,0,0,0,0,0,0,0]]C0276160	adenoviral meningitis,meningitis due to adenovirus	[[1,0,0,0,0,0,0,0,0,0,0,0,0,0],[1,0,0,0,0,0,0,0,0,0,0,0,0,0]]C0276163	adenoviral myocarditis	[[1,0,0,0,0,0,0,0,0,0,0,0,0,0]]C0276162	adenoviral enteritis,enteritis due to adenovirus	[[1,0,0,0,0,0,0,0,0,0,0,0,0,0],[0,0,0,0,1,0,0,0,0,0,0,0,0,0]]C0276165	turkey hemorrhagic enteritis,turkey haemorrhagic enteritis	[[1,0,0,0,0,0,0,0,0,0,0,0,0,0],[1,0,0,0,0,0,0,0,0,0,0,0,0,0]]C0276164	avian inclusion body hepatitis,chicken hemorrhagic syndrome,chicken haemorrhagic syndrome	[[1,0,0,0,0,0,0,0,0,0,0,0,0,0],[1,0,0,0,0,0,0,0,0,0,0,0,0,0],[1,0,0,0,0,0,0,0,0,0,0,0,0,0]]C0276167	marble spleen disease	[[1,0,0,0,0,0,0,0,0,0,0,0,0,0]]C0276166	avian hemorrhagic enteritis,avian haemorrhagic enteritis	[[1,0,0,0,0,0,0,0,0,0,0,0,0,0],[1,0,0,0,0,0,0,0,0,0,0,0,0,0]]C0276169	quail bronchitis	[[1,0,0,0,0,0,0,0,0,0,0,0,0,0]]C0276168	egg drop syndrome	[[1,0,0,0,0,0,0,0,0,0,0,0,0,0]]C1839321	scarf syndrome,and facial abnormalities retardation ambiguous genitalia craniostenosis cutis laxa skeletal abnormalities	[[0,1,0,1,0,0,0,0,0,0,0,0,0,0],[0,0,0,1,0,0,0,0,0,0,0,0,0,0]]C1290136	chronic disease of musculoskeletal system,chronic musculoskeletal disease,chronic musculoskeletal disorder	[[1,0,0,0,0,0,0,0,0,0,0,0,0,0],[1,0,0,0,0,0,0,0,0,0,0,0,0,0],[1,0,0,0,0,0,0,0,0,0,0,0,0,0]]C3266091	stercoral colitis	[[1,0,0,0,0,0,0,0,0,0,0,0,0,0]]C3266090	eosinophilic cholangitis	[[1,0,0,0,0,0,0,0,0,0,0,0,0,0]]C2349401	de quervains syndrome	[[0,0,0,0,0,0,0,0,0,0,0,0,0,1]]C3266099	hematoma of perianal region,haematoma of perianal region	[[1,0,0,0,0,0,0,0,0,0,0,0,0,0],[1,0,0,0,0,0,0,0,0,0,0,0,0,0]]C1334609	malignant ovarian sex cord stromal tumor,malignant ovarian sex cord stromal neoplasm,malignant sex cord stromal tumor of the ovary	[[0,0,0,0,0,0,0,0,0,0,0,1,0,0],[0,0,0,0,0,0,0,0,0,0,0,1,0,0],[0,0,0,0,0,0,0,0,0,0,0,1,0,0]]C2930893	dominant intermediate 3 charcot marie tooth disease	[[0,1,0,0,0,0,0,0,0,0,0,0,0,0]]C2675495	mean platelet volume quantitative trait locus 1,mpvqtl1	[[0,0,0,1,0,0,0,0,0,0,0,0,0,0],[0,0,0,1,0,0,0,0,0,0,0,0,0,0]]C0347079	carcinoma in situ of floor of mouth,fom carcinoma in situ of floor of mouth	[[1,0,0,0,0,0,0,0,0,0,0,0,0,0],[1,0,0,0,0,0,0,0,0,0,0,0,0,0]]C0347074	carcinoma in situ of tongue	[[0,0,0,0,0,0,0,0,0,0,0,0,0,1]]C0347075	carcinoma in situ of anterior two thirds of tongue,carcinoma in situ of mobile part of tongue	[[1,0,0,0,0,0,0,0,0,0,0,0,0,0],[1,0,0,0,0,0,0,0,0,0,0,0,0,0]]C0347076	carcinoma in situ of anterior two thirds of tongue ventral surface,carcinoma in situ anterior 2 3 tongue ventrum	[[1,0,0,0,0,0,0,0,0,0,0,0,0,0],[1,0,0,0,0,0,0,0,0,0,0,0,0,0]]C0347077	carcinoma in situ of base of tongue,carcinoma in situ of fixed part of tongue,carcinoma in situ of posterior third of tongue	[[1,0,0,0,0,0,0,0,0,0,0,0,0,0],[1,0,0,0,0,0,0,0,0,0,0,0,0,0],[1,0,0,0,0,0,0,0,0,0,0,0,0,0]]C0347072	salivary glands lips carcinoma in situ of oral cavity	[[1,0,0,0,0,0,0,0,0,0,0,0,0,0]]C0347073	carcinoma in situ of mouth,carcinoma in situ of oral cavity,carcinoma in situ of any part of mouth,stage 0 oral cavity carcinoma,stage 0 mouth carcinoma	[[0,0,0,0,0,0,0,0,0,0,0,1,0,1],[1,0,0,0,0,0,0,0,0,0,0,1,0,0],[0,0,0,0,0,0,0,0,0,0,0,0,0,1],[0,0,0,0,0,0,0,0,0,0,0,1,0,0],[0,0,0,0,0,0,0,0,0,0,0,1,0,0]]C1959867	herpes simplex corneal endotheliitis,herpes simplex corneal endotheliolitis	[[1,0,0,0,0,0,0,0,0,0,0,0,0,0],[1,0,0,0,0,0,0,0,0,0,0,0,0,0]]C3150945	long qt syndrome 2 9,lqt2 9	[[0,0,0,1,0,0,1,0,0,0,0,0,0,0],[0,0,0,1,0,0,0,0,0,0,0,0,0,0]]C3150944	long qt syndrome 1 2,lqt1 2	[[0,0,0,1,0,0,1,0,0,0,0,0,0,0],[0,0,0,1,0,0,0,0,0,0,0,0,0,0]]C2930894	galactorrhea hyperprolactinemia,galactorrhoea hyperprolactinaemia	[[0,1,0,0,0,0,0,0,0,0,0,0,0,0],[0,1,0,0,0,0,0,0,0,0,0,0,0,0]]C3150946	mammary digital nail syndrome,mdns	[[0,0,0,1,0,0,0,0,0,0,0,0,0,0],[0,0,0,1,0,0,0,0,0,0,0,0,0,0]]C3150941	rubinstein taybi syndrome 2,rsts2	[[0,0,0,1,0,0,0,0,0,0,0,0,0,0],[0,0,0,1,0,0,0,0,0,0,0,0,0,0]]C1959865	hypersomnia disorder related to menstruation	[[1,0,0,0,0,0,0,0,0,0,0,0,0,0]]C3150943	long qt syndrome 2,susceptibility to acquired long qt syndrome 2,lqt2	[[0,0,0,1,0,0,0,0,0,0,0,0,0,0],[0,0,0,1,0,0,0,0,0,0,0,0,0,0],[0,0,0,1,0,0,0,0,0,0,0,0,0,0]]C3150942	autosomal recessive spondylocostal dysostosis 4,scdo4	[[0,0,0,1,0,0,0,0,0,0,0,0,0,0],[0,0,0,1,0,0,0,0,0,0,0,0,0,0]]C2931167	parvovirus antenatal infection,parvovirus b19 antenatal infection,maternofetal infection by parvovirus	[[0,1,0,0,0,0,0,0,0,0,0,0,0,0],[0,1,0,0,0,0,0,0,0,0,0,0,0,0],[0,1,0,0,0,0,0,0,0,0,0,0,0,0]]C0686020	neoplasm of uncertain behavior of vestibule of mouth,neoplasm of uncertain behaviour of vestibule of mouth	[[1,0,0,0,0,0,0,0,0,0,0,0,0,0],[1,0,0,0,0,0,0,0,0,0,0,0,0,0]]C2677571	lung cancer susceptibility 2,lncr2	[[0,0,0,1,0,0,1,0,0,0,0,0,0,0],[0,0,0,1,0,0,0,0,0,0,0,0,0,0]]C1306446	elephantiasis vulvae,vulvar elephantiasis	[[1,0,0,0,0,0,0,0,0,0,0,0,0,0],[0,0,0,0,0,0,0,0,0,0,1,0,0,0]]C0857297	vitamin depletion	[[0,0,0,0,0,0,0,1,0,0,0,0,0,0]]C1456261	acute hepatitis c with hepatic coma	[[0,0,0,0,1,0,0,0,0,0,0,0,0,0]]C2931587	gemignani syndrome,sensorineural deafness and spastic paraparesis spinocerebellar ataxia associated with localized amyotrophy of the hands	[[0,1,0,0,0,0,0,0,0,0,0,0,0,0],[0,1,0,0,0,0,0,0,0,0,0,0,0,0]]C0001163	vestibulocochlear nerve diseases,disorder of acoustic nerve,disorder of eighth nerve,disorder of the vestibulocochlear nerve,disorder of acoustovestibular nerve,eighth cranial nerve diseases,cranial nerve viii diseases,cranial nerve viii disorders,disorder of acoustic or eighth nerve	[[0,1,0,0,0,0,0,1,0,0,0,0,0,0],[1,0,0,0,1,0,0,1,0,0,0,1,0,0],[1,0,0,0,0,0,0,0,0,0,0,0,0,0],[1,0,0,0,0,0,0,0,0,0,0,1,0,0],[1,0,0,0,0,0,0,0,0,0,0,0,0,0],[0,0,0,0,0,0,0,0,0,0,0,0,1,0],[0,0,0,0,0,0,0,0,0,0,0,0,1,0],[0,0,0,0,0,0,0,0,0,0,0,0,1,0],[0,0,0,0,0,0,0,0,0,0,0,0,0,1]]C1837122	fast channel congenital myasthenic syndrome,fccms	[[0,0,0,1,0,0,0,0,0,0,0,0,0,0],[0,0,0,1,0,0,0,0,0,0,0,0,0,0]]C1518189	large cell type malignant lymphoma	[[0,0,0,0,0,0,0,0,0,0,0,1,0,0]]C0001169	acquired coagulation factor deficiency	[[0,0,0,0,1,0,0,0,0,0,0,1,0,0]]C1302507	diffuse embryoma	[[1,0,0,0,0,0,0,0,0,0,0,0,0,0]]C1518188	large noncleaved cell type malignant lymphoma follicular	[[0,0,0,0,0,0,0,0,0,0,0,1,0,0]]C1857750	autosomal recessive 66 deafness,dfnb66	[[0,0,0,1,0,0,0,0,0,0,0,0,0,0],[0,0,0,1,0,0,0,0,0,0,0,0,0,0]]C1302501	dysplasia of fallopian tube	[[1,0,0,0,0,0,0,0,0,0,0,0,0,0]]C2931161	immunoglobulin a deficiency 1,selective iga immunodeficiency,selective deficiency of iga,selective immunoglobulin a deficiency,igad1,selective deficiency of gamma a globulin	[[0,1,0,1,0,0,0,0,0,0,0,0,0,0],[0,0,0,0,1,0,0,1,0,0,0,1,0,0],[0,1,0,1,0,0,0,1,0,0,1,0,0,0],[1,1,0,1,0,0,0,0,0,0,0,0,0,0],[0,0,0,1,0,0,0,0,0,0,0,0,0,0],[0,1,0,1,0,0,0,0,0,0,0,0,0,0]]C1863551	3 autosomal dominant hypercholesterolemia,fh3,hchola3	[[0,0,0,1,0,0,0,0,0,0,0,0,0,0],[0,0,0,1,0,0,0,0,0,0,0,0,0,0],[0,0,0,1,0,0,0,0,0,0,0,0,0,0]]C2931160	cilia with defective radial spokes	[[0,1,0,0,0,0,0,0,0,0,0,0,0,0]]C0157756	pyogenic arthritis involving ankle and foot	[[0,0,0,0,1,0,0,0,0,0,0,0,0,0]]C1260405	frontal dementia	[[0,0,0,0,0,0,0,1,0,0,0,0,0,1]]C0157754	pyogenic arthritis involving pelvic region and thigh	[[0,0,0,0,1,0,0,0,0,0,0,0,0,0]]C1260400	sickle cell hb e disease without crisis,hb s hb e disease without crisis	[[0,0,0,0,0,0,0,0,0,0,0,0,0,1],[0,0,0,0,0,0,0,0,0,0,0,0,0,1]]C1836255	preeclampsia eclampsia 4,pee4	[[0,0,0,1,0,0,0,0,0,0,0,0,0,0],[0,0,0,1,0,0,0,0,0,0,0,0,0,0]]C1260402	splenic sequestration	[[0,0,0,0,1,0,0,1,0,0,0,0,0,0]]C1260403	prothrombin gene mutation	[[0,0,0,0,0,0,0,1,0,0,0,0,0,1]]C1843096	acrocapitofemoral dysplasia,acfd	[[0,0,0,1,0,0,0,0,0,0,0,0,0,0],[0,0,0,1,0,0,0,0,0,0,0,0,0,0]]C1840402	hyperparathyroidism 1,hrpt1,familial isolated primary hyperparathyroidism,fihp	[[0,0,0,1,0,0,0,0,0,0,0,0,0,0],[0,0,0,1,0,0,0,0,0,0,0,0,0,0],[0,0,0,1,0,0,0,0,0,0,0,0,0,0],[0,0,0,1,0,0,0,0,0,0,0,0,0,0]]C1332038	aids related anal carcinoma,aids related anal cancer	[[0,0,0,0,0,0,0,0,0,0,0,1,0,0],[0,0,0,0,0,0,0,0,0,0,0,1,0,0]]C1260409	myasthenia gravis without acute exacerbation	[[0,0,0,0,1,0,0,0,0,0,0,0,0,0]]C1846331	juvenile onset dystonia	[[0,1,0,1,0,0,0,0,0,0,0,0,0,0]]C0263840	hemarthrosis involving lower leg	[[0,0,0,0,1,0,0,0,0,0,0,0,0,0]]C0263841	hemarthrosis of the ankle and or foot,haemarthrosis of the ankle and or foot,hemarthrosis involving ankle and foot	[[1,0,0,0,0,0,0,0,0,0,0,0,0,0],[1,0,0,0,0,0,0,0,0,0,0,0,0,0],[0,0,0,0,1,0,0,0,0,0,0,0,0,0]]C0263843	snapping hip,perrin ferraton disease,iliotibial band syndrome,snapping hip syndrome,snapping hip disorder	[[1,0,0,0,0,0,0,1,0,0,0,0,0,0],[1,0,0,0,0,0,0,0,0,0,0,0,0,0],[0,0,0,0,0,0,0,1,0,0,0,0,0,0],[0,0,0,0,0,0,0,1,0,0,0,0,0,0],[0,0,0,0,0,0,0,1,0,0,0,0,0,0]]C1368903	cystic teratoma	[[1,0,0,0,0,0,0,0,0,0,0,1,0,0]]C0263846	fistula of joint	[[1,0,0,0,0,0,0,0,0,0,0,0,0,1]]C1562723	retinal arteriolitis	[[1,0,0,0,0,0,0,0,0,0,0,0,0,0]]C1306089	primary malignant neoplasm of carotid body	[[1,0,0,0,0,0,0,0,0,0,0,0,0,0]]C0855002	recurrent lung cancer,recurrent lung carcinoma	[[0,0,0,0,0,0,0,0,0,0,0,1,0,0],[0,0,0,0,0,0,0,0,0,0,0,1,0,0]]C2930897	ben ari shuper mimouni syndrome	[[0,1,0,0,0,0,0,0,0,0,0,0,0,0]]C1285471	hereditary disorder of the visual system	[[1,0,0,0,0,0,0,0,0,0,0,0,0,0]]C1285337	inflammatory disorder of head	[[1,0,0,0,0,0,0,0,0,0,0,0,0,0]]C1281999	rapid atrial fibrillation	[[1,0,0,0,0,0,0,1,0,0,0,0,0,0]]C0853928	chillblains exacerbated	[[0,0,0,0,0,0,1,0,0,0,0,0,0,0]]C0264315	edema of glottis,oedema of glottis,glottic edema,glottic oedema	[[0,0,0,0,0,0,0,1,1,0,0,0,0,1],[1,0,0,0,0,0,0,0,0,0,0,0,0,0],[1,0,0,0,0,0,0,1,1,0,0,0,0,0],[1,0,0,0,0,0,0,1,0,0,0,0,0,0]]C0264314	chorditis	[[0,0,0,0,0,0,0,1,0,0,0,0,0,1]]C0264311	cellulitis of vocal cords	[[0,0,0,0,0,0,0,0,0,0,0,0,0,1]]C0264310	abscess of vocal cords	[[0,0,0,0,0,0,0,0,0,0,0,0,0,1]]C0264313	leukoplakia of vocal cords,leucoplakia of vocal cords,keratotic vocal cord	[[1,0,1,0,0,0,0,1,0,0,0,1,0,1],[1,0,0,0,0,0,0,0,0,0,0,0,0,0],[1,0,0,0,0,0,0,0,0,0,0,1,0,0]]C0264312	granuloma of vocal cords	[[1,0,0,0,0,0,0,1,0,0,0,0,0,1]]C0574912	acquired calyceal diverticulum,calyceal diverticulum	[[1,0,0,0,0,0,0,0,0,0,0,0,0,0],[1,0,0,0,0,0,0,0,0,0,0,0,0,0]]C0264319	abscess of trachea,tracheal abscess	[[0,0,0,0,0,0,0,0,0,0,0,0,0,1],[1,0,0,0,0,0,0,0,0,0,0,0,0,0]]C0264318	catarrhal tracheitis	[[1,0,0,0,0,0,0,0,0,0,0,0,0,1]]C0042769	virus diseases,disease caused by virus,viral illness,viral infection,viral infectious disease,viral diseases,virus infection,viral disorder	[[1,1,0,0,0,0,0,1,0,0,0,0,0,0],[1,0,0,0,0,0,0,1,0,0,0,0,0,0],[1,0,0,0,0,0,0,0,0,0,0,0,0,0],[0,0,1,0,0,0,0,1,1,1,0,1,0,1],[1,0,1,0,0,0,0,1,0,0,0,0,0,0],[1,1,0,0,0,0,0,1,0,0,0,0,1,0],[0,0,0,0,0,0,0,0,0,1,0,0,0,0],[0,0,0,0,0,0,0,1,0,0,0,0,0,0]]C1708751	low grade myofibroblastic sarcoma,myofibrosarcoma	[[0,0,0,0,0,0,0,0,0,0,0,1,0,0],[0,0,0,0,0,0,0,0,0,0,0,1,0,0]]C1708750	low grade lung neuroendocrine neoplasm	[[0,0,0,0,0,0,0,0,0,0,0,1,0,0]]C1832284	congenital hcfp1 hereditary facial paresis,congenital hereditary facial paresis,hcfp1,unilateral or bilateral congenital facial palsy,formerly mobius syndrome 2,formerly moebius syndrome 2,formerly mbs2	[[0,0,0,0,0,0,1,0,0,0,0,0,0,0],[0,0,0,1,0,0,0,0,0,0,0,0,0,0],[0,0,0,1,0,0,0,0,0,0,0,0,0,0],[0,0,0,1,0,0,0,0,0,0,0,0,0,0],[0,0,0,1,0,0,0,0,0,0,0,0,0,0],[0,0,0,1,0,0,0,0,0,0,0,0,0,0],[0,0,0,1,0,0,0,0,0,0,0,0,0,0]]C1333411	epicardial lipoma,lipoma of the epicardium	[[0,0,0,0,0,0,0,0,0,0,0,1,0,0],[0,0,0,0,0,0,0,0,0,0,0,1,0,0]]C1837617	autosomal dominant 31 deafness,dfna31	[[0,0,0,1,0,0,1,0,0,0,0,0,0,0],[0,0,0,1,0,0,0,0,0,0,0,0,0,0]]C0232865	functional proteinuria,intermittent proteinuria,paroxysmal proteinuria,physiologic proteinuria,transient proteinuria	[[1,0,0,0,0,0,0,0,0,0,0,0,0,0],[1,0,0,0,0,0,0,1,0,0,0,0,0,0],[1,0,0,0,0,0,0,0,0,0,0,0,0,0],[1,0,0,0,0,0,0,0,0,0,0,0,0,0],[1,0,0,0,0,0,0,1,0,0,0,0,0,0]]C0232866	essential proteinuria	[[1,0,0,0,0,0,0,0,0,0,0,0,0,0]]C0232867	orthostatic proteinuria,postural proteinuria,postural albuminuria,benign postural proteinuria	[[0,0,0,0,0,0,0,1,0,0,0,0,0,1],[0,0,0,0,1,0,0,1,0,0,0,0,0,0],[1,0,0,0,0,0,0,0,0,0,0,0,0,0],[0,0,0,0,0,0,0,0,0,0,0,0,0,1]]C1837610	ichthyosis prematurity syndrome,ips,ichthyosis congenita iv	[[0,1,0,1,0,0,0,0,0,0,0,0,0,0],[0,0,0,1,0,0,0,0,0,0,0,0,0,0],[0,1,0,1,0,0,0,0,0,0,0,0,0,0]]C0043410	yersinia pseudotuberculosis infections,pseudotuberculosis,pasteurella pseudotuberculosis,pasteurella pseudotuberculoses,rodent pseudotuberculosis,infection by pasteurella pseudotuberculosis	[[1,1,0,0,0,0,0,0,0,0,0,0,1,0],[1,0,0,0,0,0,0,1,0,0,0,0,0,0],[0,1,0,0,0,0,0,0,0,0,0,0,1,0],[0,1,0,0,0,0,0,0,0,0,0,0,0,0],[1,0,0,0,0,0,0,0,0,0,0,0,0,0],[1,0,0,0,0,0,0,0,0,0,0,0,0,1]]C0232868	lordotic proteinuria,lordotic albuminuria	[[1,0,0,0,0,0,0,0,0,0,0,0,0,0],[1,0,0,0,0,0,0,0,0,0,0,0,0,0]]C1837618	3 primary ciliary dyskinesia,cild3,with or without situs inversus 3 primary ciliary dyskinesia	[[0,1,0,1,0,0,0,0,0,0,0,0,0,0],[0,0,0,1,0,0,0,0,0,0,0,0,0,0],[0,0,0,1,0,0,0,0,0,0,0,0,0,0]]C1370857	localized resectable adult primary hepatoma	[[0,0,0,0,0,1,0,0,0,0,0,0,0,0]]C1837640	autosomal dominant 28 deafness,dfna28	[[0,0,0,1,0,0,0,0,0,0,0,0,0,0],[0,0,0,1,0,0,0,0,0,0,0,0,0,0]]C1258104	diffuse scleroderma,progressive scleroderma,progressive systemic sclerosis,pss progressive systemic sclerosis,diffuse systemic sclerosis,diffuse systemic scleroses,diffuse cutaneous scleroderma,diffuse cutaneous systemic sclerosis,sudden onset scleroderma	[[1,0,0,0,0,0,0,1,0,0,1,0,1,0],[0,1,0,0,0,0,0,1,0,0,0,0,1,0],[1,1,0,0,0,0,0,1,0,0,0,0,1,0],[1,0,0,0,0,0,0,0,0,0,0,0,0,0],[1,1,0,0,0,0,0,1,0,1,0,0,1,0],[0,1,0,0,0,0,0,0,0,0,0,0,0,0],[1,0,0,0,0,0,0,0,0,0,0,0,0,0],[1,0,0,0,0,0,0,0,0,0,0,0,1,0],[0,1,0,0,0,0,0,0,0,0,0,0,1,0]]C0347927	mechanical complication of vena cava umbrella	[[1,0,0,0,0,0,0,0,0,0,0,0,0,0]]C0347924	excluding islets of langerhans benign neoplasm of pancreas,except islets of langerhans benign neoplasm of pancreas	[[1,0,0,0,0,0,0,0,0,0,0,0,0,0],[0,0,0,0,1,0,0,0,0,0,0,0,0,0]]C0347925	non syphilitic argyll robertson pupil	[[1,1,0,0,0,0,0,0,0,0,0,0,0,0]]C0347922	the puerperium or lactation cracked nipple in pregnancy	[[1,0,0,0,0,0,0,0,0,0,0,0,0,0]]C1370858	localized unresectable adult primary hepatoma	[[0,0,0,0,0,1,0,0,0,0,0,0,0,0]]C1561982	toxic anterior segment syndrome,tass toxic anterior segment syndrome	[[1,0,0,0,0,0,0,0,0,0,0,0,0,0],[1,0,0,0,0,0,0,0,0,0,0,0,0,0]]C2828100	stage iiib vulvar cancer,stage iiib vulvar carcinoma,stage iiib vulvar carcinoma ajcc v7,stage iiib vulvar cancer ajcc v7	[[0,0,0,0,0,0,0,0,0,0,0,1,0,0],[0,0,0,0,0,0,0,0,0,0,0,1,0,0],[0,0,0,0,0,0,0,0,0,0,0,1,0,0],[0,0,0,0,0,0,0,0,0,0,0,1,0,0]]C2828101	stage iiic vulvar cancer,stage iiic vulvar cancer ajcc v7,stage iiic vulvar carcinoma,stage iiic vulvar carcinoma ajcc v7	[[0,0,0,0,0,0,0,0,0,0,0,1,0,0],[0,0,0,0,0,0,0,0,0,0,0,1,0,0],[0,0,0,0,0,0,0,0,0,0,0,1,0,0],[0,0,0,0,0,0,0,0,0,0,0,1,0,0]]C0494165	secondary malignant neoplasm of liver,specified as secondary malignant neoplasm of liver,metastasis to liver,liver metastases,metastatic cancer to liver,hepatic metastasis,hepatic metastases,liver secondaries,liver secondary cancer,metastatic malignant neoplasm to liver,metastatic neoplasm to the liver,metastatic tumor to the liver	[[1,0,0,0,0,0,0,0,0,0,0,0,0,0],[0,0,0,0,1,0,0,0,0,0,0,0,0,0],[1,0,0,0,0,0,0,1,0,0,0,1,0,0],[0,0,0,0,0,0,0,1,0,0,0,1,0,0],[0,0,0,0,0,1,0,1,0,0,1,0,0,0],[1,0,0,0,0,0,0,1,0,0,0,0,0,0],[0,0,0,0,0,0,0,1,0,0,0,0,0,0],[1,0,0,0,0,0,0,1,0,0,0,0,0,0],[1,0,0,0,0,0,0,1,0,0,0,0,0,0],[1,0,0,0,0,0,0,0,0,0,0,1,0,0],[0,0,0,0,0,0,0,0,0,0,0,1,0,0],[0,0,0,0,0,0,0,0,0,0,0,1,0,0]]C0494164	secondary malignant neoplasm of small intestine,secondary malignant neoplasm of small intestine including duodenum,metastasis to small intestine,metastases to small intestine,metastatic malignant neoplasm to small intestine,secondary malignant neoplasm to the small bowel,secondary malignant tumor to the small bowel,metastasis to the small bowel,secondary malignant tumor to the small intestine,metastatic neoplasm to the small intestine	[[1,0,0,0,0,0,0,0,0,0,0,1,0,0],[0,0,0,0,1,0,0,0,0,0,0,0,0,0],[1,0,0,0,0,0,0,0,0,0,0,1,0,0],[0,0,0,0,0,0,0,0,0,0,0,1,0,0],[1,0,0,0,0,0,0,0,0,0,0,1,0,0],[0,0,0,0,0,0,0,0,0,0,0,1,0,0],[0,0,0,0,0,0,0,0,0,0,0,1,0,0],[0,0,0,0,0,0,0,0,0,0,0,1,0,0],[0,0,0,0,0,0,0,0,0,0,0,1,0,0],[0,0,0,0,0,0,0,0,0,0,0,1,0,0]]C0334348	papillary hidradenoma,hydradenomas papillary,hidradenoma papilliferum	[[1,0,0,0,0,0,0,1,0,0,0,1,0,0],[0,0,0,0,0,0,0,1,0,0,0,0,0,0],[1,0,0,0,0,0,0,1,0,0,0,1,0,0]]C2931899	familial papillary renal cell carcinoma	[[0,1,0,0,0,0,0,0,0,0,0,0,0,0]]C0035312	retinal drusen,drusen degenerative of retina,drusen body,drusen of retina,colloid bodies in retina	[[1,0,0,0,0,0,1,1,0,0,0,0,1,0],[0,0,0,0,1,0,0,0,0,0,0,0,0,1],[0,0,1,0,0,0,0,0,0,0,0,0,0,0],[0,0,0,0,0,0,0,1,0,0,0,0,0,1],[1,0,0,0,0,0,0,0,0,0,0,0,0,0]]C1847874	phace association	[[0,1,0,1,0,0,0,0,0,0,0,0,0,0]]C0334341	malignant endometrioid adenofibroma,malignant endometrioid cystadenofibroma	[[1,0,0,0,0,0,0,0,0,0,0,0,0,0],[1,0,0,0,0,0,0,0,0,0,0,0,0,0]]C0334342	skin appendage adenoma,adnexal tumor,adnexal tumour,adenoma of adnexa,adnexal adenoma	[[1,0,0,0,0,0,0,0,0,0,0,1,0,0],[1,0,0,0,0,0,0,1,0,0,0,0,0,0],[1,0,0,0,0,0,0,1,0,0,0,0,0,0],[0,0,0,0,0,0,0,0,0,0,0,1,0,0],[0,0,0,0,0,0,0,0,0,0,0,1,0,0]]C0035319	acute retinal necrosis syndrome,acute retinal necrosis,acute retinal necroses,arn acute retinal necrosis,barn bilateral acute retinal necrosis	[[0,0,0,0,0,0,0,1,0,0,0,0,1,0],[1,1,0,0,0,0,0,1,0,0,0,0,1,0],[0,1,0,0,0,0,0,0,0,0,0,0,0,0],[1,0,0,0,0,0,0,0,0,0,0,0,0,0],[1,0,0,0,0,0,0,0,0,0,0,0,0,0]]C0334345	apocrine adenoma,tubular apocrine adenoma,apocrine cystadenoma,apocrine hidrocystoma,m apocrine adenoma	[[1,0,0,0,0,0,0,1,0,0,0,1,0,0],[1,0,0,0,0,0,0,0,0,0,0,1,0,0],[0,0,0,0,0,0,0,1,0,0,0,0,0,0],[0,0,0,0,0,0,0,1,0,0,0,0,0,0],[1,0,0,0,0,0,0,0,0,0,0,0,0,0]]C0334346	apocrine adenocarcinoma	[[1,0,0,0,0,0,0,0,0,0,0,0,0,0]]C0334347	eccrine spiradenoma,spiradenoma,eccrine spiradenoma of skin,benign eccrine spiradenoma	[[1,0,0,0,0,0,0,1,0,0,0,1,0,0],[0,0,0,1,0,0,0,1,0,0,0,1,0,0],[1,0,0,0,0,0,0,0,0,0,0,0,0,0],[0,0,0,0,0,0,0,0,0,0,0,1,0,0]]C0345566	benign neoplasm of buccal mucosa,benign tumor of buccal mucosa,benign tumour of buccal mucosa,benign tumor of internal cheek,benign tumour of internal cheek,benign tumor of cheek mucosa,benign tumour of cheek mucosa,benign neoplasm of cheek mucosa	[[1,0,0,0,0,0,0,0,0,0,0,1,0,0],[1,0,0,0,0,0,0,0,0,0,0,1,0,0],[1,0,0,0,0,0,0,0,0,0,0,0,0,0],[1,0,0,0,0,0,0,0,0,0,0,0,0,0],[1,0,0,0,0,0,0,0,0,0,0,0,0,0],[1,0,0,0,0,0,0,0,0,0,0,0,0,0],[1,0,0,0,0,0,0,0,0,0,0,0,0,0],[1,0,0,0,0,0,0,0,0,0,0,0,0,0]]C0345567	neoplasm of vestibule of mouth,tumor of vestibule of mouth,tumour of vestibule of mouth	[[1,0,0,0,0,0,0,0,0,0,0,0,0,0],[1,0,0,0,0,0,0,0,0,0,0,0,0,0],[1,0,0,0,0,0,0,0,0,0,0,0,0,0]]C0345560	carcinoma of uvula	[[1,0,0,0,0,0,0,0,0,0,0,0,0,0]]C0345561	carcinoma in situ of uvula	[[1,0,0,0,0,0,0,0,0,0,0,0,0,0]]C0345562	benign neoplasm of uvula,benign tumor of uvula,benign tumour of uvula,benign uvular neoplasm,benign uvular tumor	[[1,0,0,0,0,0,0,0,0,0,0,1,0,1],[1,0,0,0,0,0,0,0,0,0,0,1,0,0],[1,0,0,0,0,0,0,0,0,0,0,1,0,0],[0,0,0,0,0,0,0,0,0,0,0,1,0,0],[0,0,0,0,0,0,0,0,0,0,0,1,0,0]]C0345563	neoplasm of buccal mucosa,tumor of buccal mucosa,tumour of buccal mucosa,tumor of cheek mucosa,tumour of cheek mucosa	[[1,0,0,0,0,0,0,0,0,0,0,1,0,0],[1,0,0,0,0,0,0,0,0,0,0,1,0,0],[1,0,0,0,0,0,0,0,0,0,0,0,0,0],[1,0,0,0,0,0,0,0,0,0,0,0,0,0],[1,0,0,0,0,0,0,0,0,0,0,0,0,0]]C0685166	secondary malignant neoplasm of blood vessel of popliteal space,metastatic malignant neoplasm to blood vessel of popliteal space	[[1,0,0,0,0,0,0,0,0,0,0,0,0,0],[1,0,0,0,0,0,0,0,0,0,0,0,0,0]]C0685165	primary malignant neoplasm of blood vessel of popliteal space	[[1,0,0,0,0,0,0,0,0,0,0,0,0,0]]C0685164	benign neoplasm of blood vessel of popliteal space	[[1,0,0,0,0,0,0,0,0,0,0,0,0,0]]C0345568	tumor of buccal sulcus,tumour of buccal sulcus	[[1,0,0,0,0,0,0,0,0,0,0,0,0,0],[1,0,0,0,0,0,0,0,0,0,0,0,0,0]]C0345569	tumor of upper buccal sulcus,tumour of upper buccal sulcus	[[1,0,0,0,0,0,0,0,0,0,0,0,0,0],[1,0,0,0,0,0,0,0,0,0,0,0,0,0]]C0685161	benign neoplasm of blood vessel of thigh	[[1,0,0,0,0,0,0,0,0,0,0,0,0,0]]C0685160	secondary malignant neoplasm of blood vessel of hip,metastatic malignant neoplasm to blood vessel of hip	[[1,0,0,0,0,0,0,0,0,0,0,0,0,0],[1,0,0,0,0,0,0,0,0,0,0,0,0,0]]C1335993	small intestinal ec cell serotonin producing tumor	[[0,0,0,0,0,0,0,0,0,0,0,1,0,0]]C1335992	small intestinal somatostatin producing tumor,small intestinal somatostatin producing neoplasm	[[0,0,0,0,0,0,0,0,0,0,0,1,0,0],[0,0,0,0,0,0,0,0,0,0,0,1,0,0]]C1335991	small intestinal burkitt lymphoma,primary small intestinal burkitts lymphoma	[[0,0,0,0,0,0,0,0,0,0,0,1,0,0],[0,0,0,0,0,0,0,0,0,0,0,1,0,0]]C1335990	small intestinal atypical burkitt burkitt like lymphoma,primary small intestinal atypical burkitts burkitts like lymphoma	[[0,0,0,0,0,0,0,0,0,0,0,1,0,0],[0,0,0,0,0,0,0,0,0,0,0,1,0,0]]C1335997	small intestinal high grade intraepithelial neoplasia,small intestinal high grade dysplasia	[[0,0,0,0,0,0,0,0,0,0,0,1,0,0],[0,0,0,0,0,0,0,0,0,0,0,1,0,0]]C1335996	gastrointestinal stromal tumor of small intestine,gastrointestinal stromal tumour of small intestine,small intestine digestive stromal neoplasm,small intestine digestive stromal tumor,small intestine gastrointestinal stromal neoplasm,small intestinal gist	[[1,0,0,0,0,0,0,0,0,0,0,1,0,0],[1,0,0,0,0,0,0,0,0,0,0,0,0,0],[0,0,0,0,0,0,0,0,0,0,0,1,0,0],[0,0,0,0,0,0,0,0,0,0,0,1,0,0],[0,0,0,0,0,0,0,0,0,0,0,1,0,0],[0,0,0,0,0,0,0,0,0,0,0,1,0,0]]C1335995	small intestinal gastrin producing tumor,small intestinal gastrin producing neoplasm	[[0,0,0,0,0,0,0,0,0,0,0,1,0,0],[0,0,0,0,0,0,0,0,0,0,0,1,0,0]]C1335994	small intestinal fibrosarcoma,small bowel fibrosarcoma	[[0,0,0,0,0,0,0,0,0,0,0,1,0,0],[0,0,0,0,0,0,0,0,0,0,0,1,0,0]]C1840475	congenital horner syndrome	[[0,0,0,1,0,0,0,0,0,0,0,0,0,0]]C1840474	beta globin related hpa i recognition polymorphism,sickle cell anemia related restriction fragment length polymorphism,hpa1	[[0,0,0,1,0,0,0,0,0,0,0,0,0,0],[0,0,0,1,0,0,0,0,0,0,0,0,0,0],[0,0,0,1,0,0,0,0,0,0,0,0,0,0]]C1335999	small intestinal intraepithelial neoplasia,small intestinal dysplasia	[[0,0,0,0,0,0,0,0,0,0,0,1,0,0],[0,0,0,0,0,0,0,0,0,0,0,1,0,0]]C1335998	small intestinal hodgkin lymphoma,small bowel hodgkins disease,small bowel hodgkins lymphoma,small intestine hodgkins disease,primary small intestinal hodgkins lymphoma	[[0,0,0,0,0,0,0,0,0,0,0,1,0,0],[0,0,0,0,0,0,0,0,0,0,0,1,0,0],[0,0,0,0,0,0,0,0,0,0,0,1,0,0],[0,0,0,0,0,0,0,0,0,0,0,1,0,0],[0,0,0,0,0,0,0,0,0,0,0,1,0,0]]C1840471	kozlowski celermajer tink syndrome,humerospinal dysostosis,omani type spondyloepiphyseal dysplasia,humero spinal dysostosis with congenital heart disease,hsd,chondrodysplasia with multiple dislocations,cdmd,spondyloepiphyseal dysplasia with congenital joint dislocations	[[0,1,0,0,0,0,0,0,0,0,0,0,0,0],[0,1,0,1,0,0,0,0,0,0,0,0,0,0],[0,0,0,1,0,0,0,0,0,0,0,0,0,0],[0,1,0,0,0,0,0,0,0,0,0,0,0,0],[0,0,0,1,0,0,0,0,0,0,0,0,0,0],[0,0,0,1,0,0,0,0,0,0,0,0,0,0],[0,0,0,1,0,0,0,0,0,0,0,0,0,0],[0,0,0,1,0,0,0,0,0,0,0,0,0,0]]C0679362	extrapulmonary tuberculosis,extra pulmonary tuberculosis,extrapulmonary tb	[[1,0,0,0,0,0,0,1,0,0,0,0,0,0],[0,0,0,0,0,0,0,1,0,0,0,0,0,0],[1,0,0,0,0,0,0,0,0,0,0,0,0,0]]C1837739	resistance to diastolic hypertension	[[0,0,0,1,0,0,0,0,0,0,0,0,0,0]]C1527320	aural vertigo	[[0,1,0,0,0,0,0,0,0,1,0,0,0,1]]C0410353	infection of metatarsal	[[1,0,0,0,0,0,0,0,0,0,0,0,0,0]]C2931219	thakker donnai syndrome,dysmorphism multiple structural anomalies,dysmorphic facial features and multiple structural abnormalities	[[0,1,0,0,0,0,0,0,0,0,0,0,0,0],[0,1,0,0,0,0,0,0,0,0,0,0,0,0],[0,1,0,0,0,0,0,0,0,0,0,0,0,0]]C2931218	tetraamelia multiple malformations,zimmer taub sova syndrome,zimmer phocomelia	[[0,1,0,0,0,0,0,0,0,0,0,0,0,0],[0,1,0,0,0,0,0,0,0,0,0,0,0,0],[0,1,0,0,0,0,0,0,0,0,0,0,0,0]]C2931215	x linked tetra amelia	[[0,1,0,0,0,0,0,0,0,0,0,0,0,0]]C1719519	unsatisfactory restoration of tooth	[[0,0,0,0,1,0,0,0,0,0,0,0,0,0]]C2931217	tetra amelia with pulmonary hypoplasia,syndrome of tetraamelia with pulmonary hypoplasia,absence of upper and lower limbs with pulmonary hypoplasia	[[0,1,0,0,0,0,0,0,0,0,0,0,0,0],[0,1,0,0,0,0,0,0,0,0,0,0,0,0],[0,1,0,0,0,0,0,0,0,0,0,0,0,0]]C2931216	tetra amelia autosomal recessive	[[0,1,0,0,0,0,0,0,0,0,0,0,0,0]]C1283594	deficiency of phospholipase d	[[1,0,0,0,0,0,0,0,0,0,0,0,0,0]]C2931213	type 2c usher syndrome,type iic usher syndrome,ush2c	[[0,1,0,0,0,0,0,0,0,0,0,0,0,0],[0,0,0,1,0,0,0,0,0,0,0,0,0,0],[0,0,0,1,0,0,0,0,0,0,0,0,0,0]]C2931212	type 2b usher syndrome	[[0,1,0,0,0,0,0,0,0,0,0,0,0,0]]C2349403	acute on chronic graft versus host disease	[[0,0,0,0,1,0,0,0,0,0,0,0,0,0]]C1334603	malignant mixed mesodermal mullerian tumor,mesodermal mixed tumor,mesodermal mixed tumour,malignant mixed mesodermal tumor,malignant mixed mesodermal tumour,malignant mixed mullerian tumor,mmmt	[[0,0,0,0,0,0,0,0,0,0,0,1,0,0],[1,1,0,0,0,0,0,1,0,0,0,0,1,0],[1,0,0,0,0,0,0,0,0,0,0,0,0,0],[1,0,0,0,0,0,0,0,0,0,0,1,0,0],[1,0,0,0,0,0,0,0,0,0,0,0,0,0],[0,0,0,0,0,0,0,0,0,0,0,1,0,0],[0,0,0,0,0,0,0,0,0,0,0,1,0,0]]C1970209	alzheimer disease 12,12 familial alzheimer disease,ad12	[[0,0,0,1,0,0,0,0,0,0,0,0,0,0],[0,0,0,1,0,0,0,0,0,0,0,0,0,0],[0,0,0,1,0,0,0,0,0,0,0,0,0,0]]C0431444	multiple anterior segment anomalies	[[1,0,0,0,0,0,0,0,0,0,0,0,0,0]]C1280465	excess skin and muscle of eyelid	[[1,0,0,0,0,0,0,0,0,0,0,0,0,0]]C0264867	chronic bacterial endocarditis,lenta chronic endocarditis	[[1,0,0,0,0,0,0,0,0,0,0,0,0,1],[0,0,0,0,0,0,0,0,0,0,0,0,0,1]]C0264866	noninfective endocarditis	[[1,0,0,0,0,0,0,0,0,0,0,0,0,0]]C0264865	valvular endocarditis	[[1,0,0,0,0,0,0,0,0,0,0,0,0,0]]C0264864	chronic endocarditis	[[1,0,0,0,0,0,0,0,0,0,0,0,0,0]]C0264863	subacute endocarditis,infective subacute endocarditis	[[1,0,0,0,0,0,0,1,0,0,0,0,0,0],[0,0,0,0,0,0,0,1,0,0,0,0,0,1]]C0410467	pagets disease scapula	[[1,0,0,0,0,0,0,0,0,0,0,0,0,0]]C0264861	myocarditis due to physical agent	[[1,0,0,0,0,0,0,0,0,0,0,0,0,0]]C0264860	myocarditis due to drug	[[1,0,0,0,0,0,0,0,0,0,0,0,0,0]]C0410468	pagets disease clavicle	[[1,0,0,0,0,0,0,0,0,0,0,0,0,0]]C0410469	pagets disease coccyx	[[1,0,0,0,0,0,0,0,0,0,0,0,0,0]]C0574914	female perineal wart	[[1,0,0,0,0,0,0,0,0,0,0,0,0,0]]C1518237	malignant ovarian transitional cell tumor,malignant ovarian transitional cell neoplasm	[[0,0,0,0,0,0,0,0,0,0,0,1,0,0],[0,0,0,0,0,0,0,0,0,0,0,1,0,0]]C0264869	vegetative endocarditis,verrucous endocarditis	[[1,0,0,0,0,0,0,0,0,0,0,0,0,1],[1,0,0,0,0,0,0,0,0,0,0,0,0,0]]C0264868	purulent endocarditis	[[1,0,0,0,0,0,0,0,0,0,0,0,0,1]]C0522627	hepatosplenic gamma delta cell lymphoma	[[1,0,0,0,0,0,0,0,0,0,0,0,0,0]]C0522624	subcutaneous panniculitic t cell lymphoma,subcutaneous panniculitis like t cell lymphoma,sptcl,alpha beta type subcutaneous panniculitis like t cell lymphoma	[[1,0,0,0,0,0,0,0,0,0,0,0,0,0],[1,0,0,0,0,0,0,0,0,0,0,1,0,0],[0,0,0,0,0,0,0,0,0,0,0,1,0,0],[0,0,0,0,0,0,0,0,0,0,0,1,0,0]]C0860471	dairy intolerance	[[0,0,0,0,0,0,0,1,0,0,0,0,0,0]]C0265198	multiple system malformation syndrome	[[1,0,0,0,0,0,0,0,0,0,0,0,0,0]]C0265199	without skeletal dysplasia small stature multiple malformation syndrome	[[1,0,0,0,0,0,0,0,0,0,0,0,0,0]]C0522621	sessile polyp	[[1,0,0,0,0,0,0,1,0,0,0,0,0,0]]C0265194	nonfilarial elephantiasis	[[1,0,0,0,0,0,0,0,0,0,0,0,0,1]]C1297917	malignant tumor involving rectum by direct extension from ovary,malignant tumour involving rectum by direct extension from ovary	[[1,0,0,0,0,0,0,0,0,0,0,0,0,0],[1,0,0,0,0,0,0,0,0,0,0,0,0,0]]C0265190	rupture of thoracic duct	[[1,0,0,0,0,0,0,0,0,0,0,0,0,0]]C0265191	chronic acquired lymphedema,chronic acquired lymphoedema,secondary lymphedema,secondary lymphoedema	[[0,0,0,0,0,0,0,0,0,0,0,0,0,1],[1,0,0,0,0,0,0,0,0,0,0,0,0,0],[1,0,0,0,0,0,0,0,0,0,0,1,0,1],[1,0,0,0,0,0,0,0,0,0,0,0,0,0]]C1518230	malignant ovarian clear cell tumor,malignant ovarian clear cell neoplasm	[[0,0,0,0,0,0,0,0,0,0,0,1,0,0],[0,0,0,0,0,0,0,0,0,0,0,1,0,0]]C0271240	blindness of one eye,blind eye,unilateral blindness	[[1,0,0,0,0,0,0,1,0,0,0,0,0,1],[1,0,0,0,0,0,0,1,0,0,0,0,0,0],[0,0,0,0,0,0,0,1,0,0,0,0,0,0]]C0399428	puberty gingivitis	[[1,0,0,0,0,0,0,0,0,0,0,0,0,0]]C0399429	pregnancy gingivitis	[[1,0,0,0,0,0,0,1,0,0,0,0,0,0]]C0399424	periapical abscess no sinus,periapical abscess without sinus tract,dental abscess without a sinus,dentoalveolar abscess without a sinus	[[1,0,0,0,1,0,1,0,0,0,0,0,0,0],[1,0,0,0,0,0,0,0,0,0,0,0,0,0],[1,0,0,0,0,0,0,0,0,0,0,0,0,0],[1,0,0,0,0,0,0,0,0,0,0,0,0,0]]C1518232	malignant ovarian mixed epithelial tumor,ovarian mixed epithelial carcinoma,mixed epithelial carcinoma of ovary,malignant ovarian mixed epithelial neoplasm	[[0,0,0,0,0,0,0,0,0,0,0,1,0,0],[0,0,0,0,0,0,0,0,0,0,0,1,0,0],[0,0,0,0,0,0,0,0,0,0,0,1,0,0],[0,0,0,0,0,0,0,0,0,0,0,1,0,0]]C0796418	visual pathway glioma,glioma of optic tract,optic pathway glioma	[[0,0,0,0,0,0,0,0,0,0,0,1,0,0],[0,0,0,0,0,0,0,0,0,0,0,1,0,0],[0,0,0,0,0,0,0,0,0,0,0,1,0,0]]C1842466	chromosome 14 paternal uniparental disomy,paternal uniparental disomy 14	[[0,1,0,1,0,0,0,0,0,0,0,0,0,0],[0,1,0,0,0,0,0,0,0,0,0,0,0,0]]C0399423	posteruptive tooth staining due to tetracycline,tetracycline staining of tooth,intrinsic staining of tooth tetracycline	[[1,0,0,0,0,0,0,0,0,0,0,0,0,0],[1,0,0,0,0,0,0,0,0,0,0,0,0,0],[1,0,0,0,0,0,0,0,0,0,0,0,0,0]]C0343673	late congenital neurovascular syphilis	[[1,0,0,0,0,0,0,0,0,0,0,0,0,0]]C2316786	chronic kidney disease stage 2,ckd stage 2	[[1,0,0,0,0,0,0,0,0,0,0,1,0,0],[1,0,0,0,0,0,0,0,0,0,0,0,0,0]]C2316787	chronic kidney disease stage 3,ckd stage 3	[[1,0,0,0,0,0,0,0,0,0,0,1,0,0],[1,0,0,0,0,0,0,0,0,0,0,0,0,0]]C1511789	desmoplastic,desmoplastic reaction	[[0,0,0,0,0,0,0,0,0,0,0,1,0,0],[0,0,0,0,0,0,0,0,0,0,0,1,0,0]]C2316780	malignant neoplasm of lower respiratory tract	[[1,0,0,0,0,0,0,0,0,0,0,0,0,0]]C2748508	narcolepsy 5,nrclp5	[[0,0,0,1,0,0,0,0,0,0,0,0,0,0],[0,0,0,1,0,0,0,0,0,0,0,0,0,0]]C0158344	nontraumatic rupture of flexor tendons of hand and wrist	[[0,0,0,0,1,0,0,0,0,0,0,0,0,0]]C0031762	photosensitivity disorders,photodermatitis,photodermatitides,photosensitivity dermatitis	[[0,1,0,0,0,0,0,0,0,0,0,0,1,0],[0,0,0,0,0,0,0,1,0,0,0,0,0,0],[0,1,0,0,0,0,0,0,0,0,0,0,0,0],[1,0,0,0,0,0,0,1,0,0,0,0,0,0]]C0031763	photosensitization	[[0,1,0,0,0,0,0,1,0,0,0,0,0,0]]C0031766	acute phototoxic dermatitis,phototoxic response	[[1,0,0,0,0,0,0,0,0,0,0,0,0,0],[0,0,0,0,0,0,0,0,0,0,0,0,0,1]]C0158343	nontraumatic rupture of extensor tendons of hand and wrist	[[0,0,0,0,1,0,0,0,0,0,0,0,0,0]]C1334644	maxillary sinus cholesteatoma	[[0,0,0,0,0,0,0,0,0,0,0,1,0,0]]C1334645	maxillary sinus inverted papilloma	[[0,0,0,0,0,0,0,0,0,0,0,1,0,0]]C0035865	ross river fever,epidemic australian polyarthritis,epidemic polyarthritis and exanthema,rr ross river fever,disease rivers ross,ross river mosquito borne viral fever	[[1,0,0,0,0,0,0,1,0,0,0,0,0,0],[1,0,0,0,0,0,0,0,0,0,0,0,0,0],[1,0,0,0,0,0,0,0,0,0,0,0,0,0],[1,0,0,0,0,0,0,0,0,0,0,0,0,0],[0,0,0,0,0,0,0,1,0,0,0,0,0,0],[0,0,0,0,0,0,0,0,0,0,0,0,0,1]]C1334647	maxillary sinus squamous cell carcinoma,squamous cell carcinoma of the maxillary antrum,maxillary antrum epidermoid carcinoma,squamous cell carcinoma of the maxillofacial sinus,maxillary sinus epidermoid carcinoma,maxillofacial sinus epidermoid carcinoma	[[0,0,0,0,0,0,0,0,0,0,0,1,0,0],[0,0,0,0,0,0,0,0,0,0,0,1,0,0],[0,0,0,0,0,0,0,0,0,0,0,1,0,0],[0,0,0,0,0,0,0,0,0,0,0,1,0,0],[0,0,0,0,0,0,0,0,0,0,0,1,0,0],[0,0,0,0,0,0,0,0,0,0,0,1,0,0]]C1334640	mature t cell and nk cell neoplasm,mature t cell neoplasm	[[0,0,0,0,0,0,0,0,0,0,0,1,0,0],[0,0,0,0,0,0,0,0,0,0,0,1,0,0]]C0339352	capsular cataract	[[1,0,0,0,0,0,0,0,0,0,0,0,0,0]]C1334642	maxillary sinus adenocarcinoma	[[0,0,0,0,0,0,0,0,0,0,0,1,0,0]]C0266099	gingival cyst of adult	[[1,0,0,0,0,0,0,0,0,0,0,0,0,0]]C1334648	meckel diverticulum carcinoid tumor,carcinoid neoplasm of the meckels diverticulum	[[0,0,0,0,0,0,0,0,0,0,0,1,0,0],[0,0,0,0,0,0,0,0,0,0,0,1,0,0]]C1334649	mediastinal angiosarcoma,angiosarcoma of the mediastinum,mediastinal hemangiosarcoma,hemangiosarcoma of the mediastinum	[[0,0,0,0,0,0,0,0,0,0,0,1,0,0],[0,0,0,0,0,0,0,0,0,0,0,1,0,0],[0,0,0,0,0,0,0,0,0,0,0,1,0,0],[0,0,0,0,0,0,0,0,0,0,0,1,0,0]]C0035869	rotavirus infections,disease due to rotavirus	[[0,1,0,0,0,0,0,1,0,0,0,0,0,0],[1,0,0,0,0,0,0,0,0,1,0,0,0,0]]C2748503	subepithelial mucinous corneal dystrophy,smcd	[[0,0,0,1,0,0,0,0,0,0,0,0,0,0],[0,0,0,1,0,0,0,0,0,0,0,0,0,0]]C1519411	solid mouse min	[[0,0,0,0,0,0,0,0,0,0,0,1,0,0]]C2748502	posterior amorphous corneal dystrophy,pacd	[[0,0,0,1,0,0,0,0,0,0,0,0,0,0],[0,0,0,1,0,0,0,0,0,0,0,0,0,0]]C0578736	inguinal lymphadenopathy,groin lymphadenopathy	[[1,0,0,0,0,0,0,1,0,0,0,0,0,0],[1,0,0,0,0,0,0,0,0,0,0,0,0,0]]C0334553	giant cell tumor of soft tissue,giant cell tumour of soft tissue,giant cell tumor of soft parts,giant cell tumour of soft parts,giant cell tumor of low malignant potential,osteoclastoma of soft tissue	[[1,0,0,0,0,0,0,0,0,0,0,1,0,0],[1,0,0,0,0,0,0,0,0,0,0,0,0,0],[1,0,0,0,0,0,0,0,0,0,0,0,0,0],[1,0,0,0,0,0,0,0,0,0,0,0,0,0],[0,0,0,0,0,0,0,0,0,0,0,1,0,0],[0,0,0,0,0,0,0,0,0,0,0,1,0,0]]C0034192	phlebitis of portal vein,pylephlebitis,portal phlebitis	[[1,0,0,0,0,0,0,0,0,0,0,0,0,0],[0,0,0,0,0,0,0,0,0,0,0,0,0,1],[1,0,0,0,0,0,0,0,0,0,0,0,0,0]]C0334552	malignant giant cell tumor of bone,malignant giant cell tumour of bone,giant cell sarcoma of bone,malignant osteoclastoma,dedifferentiated giant cell tumor	[[1,0,0,0,0,1,0,0,0,0,0,1,0,0],[1,0,0,0,0,0,0,0,0,0,0,0,0,0],[1,0,0,0,0,0,0,0,0,0,0,1,0,0],[1,0,0,0,0,0,0,0,0,0,0,0,0,0],[0,0,0,0,0,0,0,0,0,0,0,1,0,0]]C0579065	type i diabetes mellitus with ulcer,insulin dependent diabetes mellitus with ulcer,type 1 diabetes mellitus with ulcer	[[1,0,0,0,0,0,0,0,0,0,0,0,0,0],[1,0,0,0,0,0,0,0,0,0,0,0,0,0],[1,0,0,0,0,0,0,0,0,0,0,0,0,0]]C0034013	precocious puberty,pubertas praecox,accelerated sexual maturity,sexual precocity,true precocious puberty,premature puberty,maturation sex accelerated,precocious sexual development,early puberty	[[0,0,0,1,0,0,0,1,1,1,1,1,1,0],[0,0,0,0,0,0,0,1,0,0,0,0,0,0],[0,0,0,0,0,0,0,0,1,0,0,0,0,0],[1,0,0,0,0,0,0,1,0,0,1,1,0,1],[0,0,0,0,0,0,0,1,0,0,0,0,0,0],[0,0,0,1,0,0,0,1,0,0,0,0,0,0],[0,0,0,0,0,0,0,0,1,0,0,0,0,0],[1,0,0,0,0,0,0,1,0,0,0,0,0,0],[0,0,0,1,0,0,0,1,0,0,0,0,0,0]]C2748507	interleukin 1 receptor antagonist deficiency,dira	[[0,1,0,1,0,0,0,0,0,0,0,0,0,0],[0,0,0,1,0,0,0,0,0,0,0,0,0,0]]C1846452	type a3 postaxial polydactyly,papa3	[[0,0,0,1,0,0,0,0,0,0,0,0,0,0],[0,0,0,1,0,0,0,0,0,0,0,0,0,0]]C1396243	protein intolerance	[[0,0,0,1,0,0,0,0,0,0,0,0,0,0]]C1304062	acute seborrheic otitis externa,acute seborrhoeic otitis externa	[[1,0,0,0,0,0,0,0,0,0,0,0,0,0],[1,0,0,0,0,0,0,0,0,0,0,0,0,0]]C1866119	type ia autoimmune lymphoproliferative syndrome,alps1a	[[0,0,0,1,0,0,0,0,0,0,0,0,0,0],[0,0,0,1,0,0,0,0,0,0,0,0,0,0]]C1514523	prostatic urethral malignant neoplasm	[[0,0,0,0,0,0,0,0,0,0,0,1,0,0]]C0265730	pelvis oblique	[[0,0,0,0,0,0,0,1,0,0,0,0,0,0]]C1514522	prostatic urethra urothelial carcinoma	[[0,0,0,0,0,0,0,0,0,0,0,1,0,0]]C1333032	chronic adult t cell leukemia lymphoma	[[0,0,0,0,0,0,0,0,0,0,0,1,0,0]]C1297913	patchy chronic proctocolitis	[[1,0,0,0,0,0,0,0,0,0,0,0,0,0]]C1333037	chronic lymphocytic leukemia small lymphocytic lymphoma with immunoglobulin heavy chain variable region gene somatic hypermutation,postgerminal center chronic lymphocytic leukemia small lymphocytic lymphoma,cll sll with igvh shm	[[0,0,0,0,0,0,0,0,0,0,0,1,0,0],[0,0,0,0,0,0,0,0,0,0,0,1,0,0],[0,0,0,0,0,0,0,0,0,0,0,1,0,0]]C0428092	starch synovitis	[[1,0,0,0,0,0,0,0,0,0,0,0,0,0]]C1270894	deficiency of hydroxyalkyl protein kinase	[[1,0,0,0,0,0,0,0,0,0,0,0,0,0]]C1281622	deficiency of endopeptidase	[[1,0,0,0,0,0,0,0,0,0,0,0,0,0]]C0686618	neoplasm of uncertain behavior of lymph node,neoplasm of uncertain behaviour of lymph node	[[1,0,0,0,0,0,0,0,0,0,0,0,0,0],[1,0,0,0,0,0,0,0,0,0,0,0,0,0]]C1333038	chronic lymphocytic leukemia small lymphocytic lymphoma with unmutated immunoglobulin heavy chain variable region gene,pregerminal center chronic lymphocytic leukemia small lymphocytic lymphoma,cll sll with unmutated igvh	[[0,0,0,0,0,0,0,0,0,0,0,1,0,0],[0,0,0,0,0,0,0,0,0,0,0,1,0,0],[0,0,0,0,0,0,0,0,0,0,0,1,0,0]]C1333039	chronic lymphocytic leukemia with immunoglobulin heavy chain variable region gene somatic hypermutation,postgerminal center chronic lymphocytic leukemia,cll with igvh shm	[[0,0,0,0,0,0,0,0,0,0,0,1,0,0],[0,0,0,0,0,0,0,0,0,0,0,1,0,0],[0,0,0,0,0,0,0,0,0,0,0,1,0,0]]C0865074	benign neoplasm of temple	[[0,0,0,0,0,0,0,0,0,0,0,0,0,1]]C1167648	abdominal and gastrointestinal infection	[[0,0,0,0,0,0,0,0,0,0,0,1,0,0]]C1839079	y linked retinitis pigmentosa,rpy	[[0,0,0,1,0,0,0,0,0,0,0,0,0,0],[0,0,0,1,0,0,0,0,0,0,0,0,0,0]]C1839075	incomplete sertoli cell only syndrome	[[0,0,0,1,0,0,0,0,0,0,0,0,0,0]]C1839074	type ii sertoli cell only syndrome	[[0,0,0,1,0,0,0,0,0,0,0,0,0,0]]C0266427	testicular regression syndrome,vanishing testes syndrome,xy gonadal agenesis syndrome,testicular regression embryonic,trs,familial anorchia,vanishing testes,xy gonadal dysgenesis syndrome,empty scrotum	[[0,1,0,1,0,0,0,0,0,0,0,0,0,0],[1,0,0,0,0,0,0,1,0,0,0,0,0,0],[1,0,0,1,0,0,0,1,0,0,0,0,0,0],[1,0,0,1,0,0,0,0,0,0,0,0,0,0],[0,0,0,1,0,0,0,0,0,0,0,0,0,0],[0,0,0,1,0,0,0,0,0,0,0,0,0,0],[0,1,0,0,0,0,0,0,0,0,0,0,0,0],[0,1,0,0,0,0,0,0,0,0,0,0,0,0],[0,1,0,0,0,0,0,0,0,0,0,0,0,0]]C1839076	y linked pseudoautosomal hodgkin disease	[[0,0,0,1,0,0,0,0,0,0,0,0,0,0]]C1299471	infection due to mycobacterium avium intracellulare group	[[1,0,0,0,0,0,0,0,0,0,0,0,0,0]]C1839070	y linked hairy ears,y linked hypertrichosis pinnae auris	[[0,0,0,1,0,0,0,0,0,0,0,0,0,0],[0,0,0,1,0,0,0,0,0,0,0,0,0,0]]C1839073	y linked sertoli cell only syndrome,type i sertoli cell only syndrome,type 1 sertoli cell only syndrome,1 y linked spermatogenic failure,spgfy1	[[0,1,0,1,0,0,0,0,0,0,0,0,0,0],[0,0,0,1,0,0,0,0,0,0,0,0,0,0],[0,1,0,0,0,0,0,0,0,0,0,0,0,0],[0,0,0,1,0,0,0,0,0,0,0,0,0,0],[0,0,0,1,0,0,0,0,0,0,0,0,0,0]]C1299472	disseminated infection due to mycobacterium avium intracellulare group	[[1,0,0,0,0,0,0,0,0,0,0,0,0,0]]C0158969	perinatal jaundice from hereditary hemolytic anemia,perinatal jaundice from hereditary haemolytic anaemias	[[1,0,0,0,1,0,0,0,0,0,0,0,0,0],[1,0,0,0,0,0,0,0,0,0,0,0,0,0]]C1270895	deficiency of phosphorylase b kinase kinase	[[1,0,0,0,0,0,0,0,0,0,0,0,0,0]]C0267776	dialysis associated ascites	[[1,0,0,0,0,0,0,0,0,0,0,0,0,0]]C0267777	adhesion of abdominal wall	[[1,0,0,0,0,0,0,0,0,0,0,0,0,1]]C0158962	hemolytic disease of fetus or newborn due to rhd isoimmunization,haemolytic disease of foetus or newborn due to rhd isoimmunisation,hemolytic disease of fetus or newborn due to rh isoimmunization,haemolytic disease of foetus or newborn due to rh isoimmunisation,erythroblastosis fetalis due to rh isoimmunization,jaundice due to rh isoimmunization of the newborn,jaundice due to rh isoimmunisation of the newborn,rh isoimmunization of the newborn,rh isoimmunisation of the newborn,rh hemolytic disease of the newborn,rh haemolytic disease of the newborn,rh hdn rh hemolytic disease of the newborn,rh hdn rh haemolytic disease of the newborn,rhesus isoimmunization of the newborn,rhesus isoimmunisation of the newborn,anemia due to rh isoimmunization,anaemia due to rh isoimmunisation,hemolytic disease due to rh isoimmunization,haemolytic disease due to rh isoimmunisation,erythroblastosis foetalis due to rh isoimmunisation,hemolytic disease due to rhesus isoimmunization,haemolytic disease due to rhesus isoimmunisation,hemolytic disease rh,haemolytic disease rh,anemia due to rh antibodies,anemia due to rh maternal fetal incompatibility,hemolytic disease of fetus due to rh antibodies,hemolytic disease of newborn due to rh antibodies,erythroblastosis fetalis due to rh antibodies,erythroblastosis due to rh antibodies,jaundice due to rh antibodies,hemolytic disease of fetus due to rh maternal fetal incompatibility,hemolytic disease of newborn due to rh maternal fetal incompatibility,hemolytic disease of fetus due to rh isoimmunization,erythroblastosis fetalis due to rh maternal fetal incompatibility,erythroblastosis due to rh maternal fetal incompatibility,erythroblastosis due to rh isoimmunization,jaundice due to rh isoimmunization	[[1,0,0,0,0,0,0,0,0,0,0,0,0,0],[1,0,0,0,0,0,0,0,0,0,0,0,0,0],[1,0,0,0,1,0,0,0,0,0,0,0,0,0],[1,0,0,0,0,0,0,0,0,0,0,0,0,0],[1,0,0,0,0,0,0,0,0,0,0,0,0,1],[1,0,0,0,0,0,0,0,0,0,0,0,0,0],[1,0,0,0,0,0,0,0,0,0,0,0,0,0],[1,0,0,0,0,0,0,0,0,0,0,0,0,0],[1,0,0,0,0,0,0,0,0,0,0,0,0,0],[1,0,0,0,0,0,0,0,0,0,0,0,0,0],[1,0,0,0,0,0,0,0,0,0,0,0,0,0],[1,0,0,0,0,0,0,0,0,0,0,0,0,0],[1,0,0,0,0,0,0,0,0,0,0,0,0,0],[1,0,0,0,0,0,0,0,0,0,0,0,0,0],[1,0,0,0,0,0,0,0,0,0,0,0,0,0],[1,0,0,0,0,0,0,0,0,0,0,0,0,1],[1,0,0,0,0,0,0,0,0,0,0,0,0,0],[1,0,0,0,0,0,0,0,0,0,0,0,0,0],[1,0,0,0,0,0,0,0,0,0,0,0,0,0],[1,0,0,0,0,0,0,0,0,0,0,0,0,0],[1,0,0,0,0,0,0,0,0,0,0,0,0,0],[1,0,0,0,0,0,0,0,0,0,0,0,0,0],[1,0,0,0,0,0,0,0,0,0,0,0,0,1],[1,0,0,0,0,0,0,0,0,0,0,0,0,0],[0,0,0,0,0,0,0,0,0,0,0,0,0,1],[0,0,0,0,0,0,0,0,0,0,0,0,0,1],[0,0,0,0,0,0,0,0,0,0,0,0,0,1],[0,0,0,0,0,0,0,0,0,0,0,0,0,1],[0,0,0,0,0,0,0,0,0,0,0,0,0,1],[0,0,0,0,0,0,0,0,0,0,0,0,0,1],[0,0,0,0,0,0,0,0,0,0,0,0,0,1],[0,0,0,0,0,0,0,0,0,0,0,0,0,1],[0,0,0,0,0,0,0,0,0,0,0,0,0,1],[0,0,0,0,0,0,0,0,0,0,0,0,0,1],[0,0,0,0,0,0,0,0,0,0,0,0,0,1],[0,0,0,0,0,0,0,0,0,0,0,0,0,1],[0,0,0,0,0,0,0,0,0,0,0,0,0,1],[0,0,0,0,0,0,0,0,0,0,0,0,0,1]]C0267775	urine ascites of the neonate	[[1,0,0,0,0,0,0,0,0,0,0,0,0,0]]C0267772	pancreatic ascites	[[1,0,0,0,0,0,0,0,0,0,0,0,0,0]]C0267773	bile ascites	[[1,0,0,0,0,0,0,1,0,0,0,0,0,0]]C0267770	retractile mesenteritis,sclerosing mesenteritis	[[0,1,0,0,0,0,0,0,0,0,0,0,0,1],[0,1,0,0,1,0,0,0,0,0,0,0,0,0]]C0267771	retroperitoneal mass,mass of retroperitoneal structure	[[1,0,0,0,0,0,0,1,0,0,0,0,0,0],[1,0,0,0,0,0,0,0,0,0,0,0,0,0]]C2931580	bazopoulou kyrkanidou syndrome	[[0,1,0,0,0,0,0,0,0,0,0,0,0,0]]C0563210	squamous cell carcinoma of skin of cheek	[[1,0,0,0,0,0,0,0,0,0,0,0,0,0]]C1843173	type 2h charcot marie tooth disease,autosomal recessive with pyramidal features axonal charcot marie tooth neuropathy,type 2h axonal charcot marie tooth disease,cmt2h,autosomal recessive with pyramidal features axonal charcot marie tooth disease	[[0,1,0,0,0,0,0,0,0,0,0,0,0,0],[0,0,0,1,0,0,0,0,0,0,0,0,0,0],[0,1,0,1,0,0,0,0,0,0,0,0,0,0],[0,0,0,1,0,0,0,0,0,0,0,0,0,0],[0,1,0,1,0,0,0,0,0,0,0,0,0,0]]C0563211	carcinoma of anal canal,anal canal cancer,anal canal and perianal gland cancer,anal canal and perianal gland carcinoma	[[1,0,0,1,0,0,0,1,0,0,0,1,0,0],[0,0,0,0,0,0,0,0,0,0,0,1,0,0],[0,0,0,0,0,0,0,0,0,0,0,1,0,0],[0,0,0,0,0,0,0,0,0,0,0,1,0,0]]C0571210	herbal medicine allergy	[[1,0,0,0,0,0,0,1,0,0,0,0,0,0]]C0563212	ameloblastoma of jaw,adamantinoma of jaw	[[1,0,0,0,0,0,0,0,0,0,0,0,0,0],[1,0,0,0,0,0,0,0,0,0,0,0,0,0]]C0152069	echinococcus multilocularis infection,alveolar hydatid disease	[[1,0,0,0,0,0,0,0,0,0,0,0,0,0],[1,0,0,0,0,0,0,0,0,0,0,0,0,0]]C0152068	echinococcus granulosus infection,unilocular hydatid disease	[[1,0,0,0,0,0,0,0,0,0,0,0,0,0],[1,0,0,0,0,0,0,0,0,0,0,0,0,0]]C0152067	microsporosis nigra,tinea nigra,infection by cladosporium werneckii,keratomycosis nigricans palmaris,tinea palmaris nigra,pityriasis nigra,keratomycosis nigricans	[[0,0,0,0,0,0,0,0,0,0,0,0,0,1],[0,0,0,0,1,0,0,0,0,0,0,0,0,0],[1,0,0,0,0,0,0,0,0,0,0,0,0,0],[1,0,0,0,0,0,0,0,0,0,0,0,0,0],[0,0,0,0,0,0,0,0,0,0,0,0,0,1],[0,0,0,0,0,0,0,0,0,0,0,0,0,1],[0,0,0,0,0,0,0,0,0,0,0,0,0,1]]C0152066	lobomycosis,lobomycoses,keloidal blastomycosis,cheloidal blastomycosis,keloidal blastomycoses,lobos disease,infection by loboa loboii,infection by paracoccidioides loboii,cutaneous lobomycosis,lacaziosis,jorge lobo disease,lacazioses,infections by blastomyces loboi,infections by loboa loboi	[[0,0,0,0,0,0,0,1,0,0,0,0,1,0],[0,1,0,0,0,0,0,0,0,0,0,0,0,0],[0,1,0,0,0,0,0,0,0,0,0,0,1,1],[1,0,0,0,0,0,0,0,0,0,0,0,0,0],[0,1,0,0,0,0,0,0,0,0,0,0,0,0],[0,0,0,0,0,0,0,0,0,0,0,0,0,1],[1,0,0,0,0,0,0,0,0,0,0,0,0,0],[1,0,0,0,0,0,0,0,0,0,0,0,0,0],[1,0,0,0,0,0,0,0,0,0,0,0,0,0],[0,0,0,0,0,0,0,0,0,0,0,0,1,0],[0,1,0,0,0,0,0,0,0,0,0,0,1,0],[0,1,0,0,0,0,0,0,0,0,0,0,0,0],[0,0,0,0,0,0,0,0,0,0,0,0,0,1],[0,0,0,0,0,0,0,0,0,0,0,0,0,1]]C0152063	streptobacillary fever,haverhill fever,erythema arthriticum epidemicum,streptobacillary rat bite fever,epidemic arthritic erythema,rat bite fever due to streptobacillus moniliformis	[[0,0,0,0,1,0,0,0,0,0,0,0,0,0],[0,0,1,0,0,0,0,0,0,0,1,0,0,1],[1,0,0,0,0,0,0,0,0,0,1,0,0,0],[1,0,0,0,0,0,0,0,0,0,1,0,0,0],[0,0,0,0,0,0,0,0,0,0,0,0,0,1],[0,0,0,0,0,0,0,0,0,0,0,0,0,1]]C0034735	raynaud phenomenon,paroxysmal digital cyanosis,raynauds syndrome,secondary raynauds phenomenon,raynauds phen	[[0,1,1,1,0,0,0,1,0,1,1,1,0,0],[1,0,0,0,0,0,0,0,0,0,0,0,0,0],[0,0,0,0,0,0,0,0,0,0,0,1,0,0],[0,0,0,0,0,0,0,0,0,0,0,0,0,1],[0,0,0,0,0,0,0,1,0,0,0,0,0,0]]C0152061	louse borne relapsing fever,relapsing fever due to borrelia recurrentis	[[1,0,0,0,1,0,0,0,0,0,0,0,0,0],[1,0,0,0,0,0,0,0,0,0,0,0,0,0]]C1335100	occult squamous cell lung carcinoma	[[0,0,0,0,0,0,0,0,0,0,0,1,0,0]]C1960764	crohns disease in remission	[[1,0,0,0,0,0,0,0,0,0,0,0,0,0]]C1960766	disorder of immune reconstitution	[[1,0,0,0,0,0,0,0,0,0,0,0,0,0]]C1335101	occupational malignant neoplasm,occupational cancer	[[0,0,0,0,0,0,0,0,0,0,0,1,0,0],[0,0,0,0,0,0,0,0,0,0,0,1,0,0]]C1848814	familial thymoma,thymic neoplasia	[[0,0,0,1,0,0,0,0,0,0,0,0,0,0],[0,0,0,1,0,0,0,0,0,0,0,0,0,0]]C2987242	non functional pancreatic neuroendocrine tumor g1	[[0,0,0,0,0,0,0,0,0,0,0,1,0,0]]C1720580	macropsia due to organic disease	[[1,0,0,0,0,0,0,0,0,0,0,0,0,0]]C2987240	pancreatic small cell neuroendocrine carcinoma	[[0,0,0,0,0,0,0,0,0,0,0,1,0,0]]C1335102	occupational neoplasm	[[0,0,0,0,0,0,0,0,0,0,0,1,0,0]]C1142166	brugada syndrome,sudden unexplained nocturnal death syndrome,and sudden death syndrome st segment elevation right bundle branch block,sunds,ventricular arrhythmia associated with brugada syndrome,brugada syndrome 1	[[1,0,0,0,0,0,1,1,0,0,0,1,0,0],[0,0,0,1,0,0,0,0,0,0,0,0,0,0],[0,0,0,1,0,0,0,0,0,0,0,0,0,0],[0,0,0,1,0,0,0,0,0,0,0,0,0,0],[0,0,0,0,0,0,0,0,0,0,0,1,0,0],[0,0,0,1,0,0,0,0,0,0,0,0,0,0]]C1335103	ocular adnexal mucosa associated lymphoid tissue lymphoma,ocular adnexal malt lymphoma	[[0,0,0,0,0,0,0,0,0,0,0,1,0,0],[0,0,0,0,0,0,0,0,0,0,0,1,0,0]]C0571937	debrisoquine allergy	[[1,0,0,0,0,0,0,0,0,0,0,0,0,0]]C1335104	old burn scar related skin melanoma	[[0,0,0,0,0,0,0,0,0,0,0,1,0,0]]C1845181	x linked 78 mental retardation,mrx78	[[0,0,0,1,0,0,0,0,0,0,0,0,0,0],[0,0,0,1,0,0,0,0,0,0,0,0,0,0]]C0752132	posterior cerebral artery infarction,posterior cerebral artery stroke,pca infarction	[[0,0,0,0,0,0,0,0,0,0,0,0,1,0],[0,0,0,0,0,0,0,0,0,0,0,0,1,0],[0,1,0,0,0,0,0,0,0,0,0,0,1,0]]C1335105	old burn scar related neoplasm	[[0,0,0,0,0,0,0,0,0,0,0,1,0,0]]C1290339	focal pneumonia	[[1,0,0,0,0,0,0,0,0,0,0,0,0,0]]C1711373	latent celiac disease	[[0,0,0,0,0,0,0,0,0,0,0,1,0,0]]C0571935	adrenergic neurone blocking drug allergy	[[1,0,0,0,0,0,0,0,0,0,0,0,0,0]]C1519207	sebaceous breast carcinoma	[[0,0,0,0,0,0,0,0,0,0,0,1,0,0]]C1335106	old burn scar related skin squamous cell carcinoma	[[0,0,0,0,0,0,0,0,0,0,0,1,0,0]]C1298989	seizures complicating infection in the newborn	[[1,0,0,0,0,0,0,0,0,0,0,0,0,0]]C1298986	infection due to aspergillus oryzae	[[1,0,0,0,0,0,0,0,0,0,0,0,0,0]]C1298984	infection due to aspergillus notatum	[[1,0,0,0,0,0,0,0,0,0,0,0,0,0]]C1298985	infection due to aspergillus ochraceus	[[1,0,0,0,0,0,0,0,0,0,0,0,0,0]]C1298982	infection due to aspergillus niger	[[1,0,0,0,0,0,0,0,0,0,0,0,0,0]]C1298983	infection due to aspergillus niveus	[[1,0,0,0,0,0,0,0,0,0,0,0,0,0]]C1298980	infection due to aspergillus janus	[[1,0,0,0,0,0,0,0,0,0,0,0,0,0]]C1142163	intestinal mass	[[0,0,0,0,0,0,0,1,0,0,0,1,0,0]]C0340004	benign asbestos pleural effusion	[[1,0,0,0,0,0,0,0,0,0,0,0,0,0]]C0340005	drug induced pleural effusion	[[1,0,0,0,0,0,0,0,0,0,0,0,0,0]]C1332341	asymmetric motor neuropathy	[[0,0,0,0,0,0,0,0,0,0,0,1,0,0]]C0340007	catamenial pneumothorax,premenstrual pneumothorax	[[1,0,0,0,0,0,0,1,0,0,0,0,0,0],[1,0,0,0,0,0,0,0,0,0,0,0,0,0]]C0340000	acute dry pleurisy	[[1,0,0,0,0,0,0,0,0,0,0,0,0,0]]C0340001	chronic dry pleurisy	[[1,0,0,0,0,0,0,0,0,0,0,0,0,0]]C1332345	atypical adenomatous lung hyperplasia,bronchioloalveolar cell adenoma,atypical alveolar hyperplasia	[[0,0,0,0,0,0,0,0,0,0,0,1,0,0],[0,0,0,0,0,0,0,0,0,0,0,1,0,0],[0,0,0,0,0,0,0,0,0,0,0,1,0,0]]C1332344	atrial septum lipomatous hypertrophy,massive fatty deposits of the atrial septum,interatrial septum lipomatous hypertrophy	[[0,0,0,0,0,0,0,0,0,0,0,1,0,0],[0,0,0,0,0,0,0,0,0,0,0,1,0,0],[0,0,0,0,0,0,0,0,0,0,0,1,0,0]]C1332348	atypical gastric lymphoid hyperplasia,atypical gastric lymphoid hyperplasia of the stomach	[[0,0,0,0,0,0,0,0,0,0,0,1,0,0],[0,0,0,0,0,0,0,0,0,0,0,1,0,0]]C0687150	parathyroid gland adenocarcinoma,parathyroid cancer,parathyroid carcinoma,adenocarcinoma parathyroid cancer,adenocarcinoma of parathyroid,cancer of the parathyroid gland,parathyroid gland carcinoma,prtc	[[0,0,0,0,0,0,0,0,0,0,0,1,0,0],[0,1,0,0,0,1,0,0,0,0,0,1,0,0],[1,1,0,1,0,1,0,1,0,0,1,1,0,0],[0,0,0,0,0,0,0,0,0,0,1,0,0,0],[0,0,0,0,0,0,0,0,0,0,1,1,0,0],[0,0,0,0,0,0,0,0,0,0,0,1,0,0],[0,0,0,0,0,0,0,0,0,0,0,1,0,0],[0,0,0,1,0,0,0,0,0,0,0,0,0,0]]C0025500	mesothelioma	[[0,1,0,0,0,0,0,1,0,0,1,0,0,0]]C1335697	recurrent childhood central nervous system neoplasm,recurrent childhood central nervous system tumor	[[0,0,0,0,0,0,0,0,0,0,0,1,0,0],[0,0,0,0,0,0,0,0,0,0,0,1,0,0]]C0687154	acrocephalopolysyndactyly	[[1,0,0,0,0,0,0,0,0,0,0,0,0,0]]C1335691	tubulovillous adenoma of rectum,rectal tubulovillous adenoma,rectal villotubular adenoma,villotubular adenoma of the rectum	[[1,0,0,0,0,0,0,0,0,0,0,1,0,0],[0,0,0,0,0,0,0,0,0,0,0,1,0,0],[0,0,0,0,0,0,0,0,0,0,0,1,0,0],[0,0,0,0,0,0,0,0,0,0,0,1,0,0]]C0687156	infection by dirofilaria immitis	[[1,0,0,0,0,0,0,0,0,0,0,0,0,0]]C0740944	amnesia episode	[[0,0,0,0,0,0,0,1,0,0,0,0,0,0]]C2919756	anatomical narrow angle glaucoma with borderline intraocular pressure	[[1,0,0,0,0,0,0,0,0,0,0,0,0,0]]C1335698	recurrent childhood optic nerve astrocytoma,recurrent pediatric optic nerve astrocytoma	[[0,0,0,0,0,0,0,0,0,0,0,1,0,0],[0,0,0,0,0,0,0,0,0,0,0,1,0,0]]C0410644	iatrogenic cervical spinal stenosis	[[1,0,0,0,0,0,0,0,0,0,0,0,0,0]]C0748901	spinal cord degeneration	[[0,0,0,0,0,0,0,1,0,0,0,0,0,0]]C1333965	hepatic fibroma,fibroma of liver	[[0,0,0,0,0,0,0,0,0,0,0,1,0,0],[0,0,0,0,0,0,0,0,0,0,0,1,0,0]]C0748903	spinal cord involvement	[[0,0,0,0,0,0,0,1,0,0,0,0,0,0]]C0410645	degenerative cervical spinal stenosis	[[1,0,0,0,0,0,0,0,0,0,0,0,0,0]]C2919754	spondylolysis of cervical spine	[[1,0,0,0,0,0,0,0,0,0,0,0,0,0]]C1333962	hepatic angiomyolipoma	[[0,0,0,0,0,0,0,0,0,0,0,1,0,0]]C0748906	spinal cord syrinx	[[0,0,0,0,0,0,0,1,0,0,0,0,0,0]]C1112776	thyroid hyperplasia	[[0,0,0,1,0,0,0,1,0,0,0,0,0,0]]C1532710	isoniazid resistant tuberculosis	[[1,0,0,0,0,0,0,0,0,0,0,0,0,0]]C1333968	hepatic leiomyoma,liver leiomyoma	[[0,0,0,0,0,0,0,0,0,0,0,1,0,0],[0,0,0,0,0,0,0,0,0,0,0,1,0,0]]C0866469	carbuncle and furuncle of abdominal wall	[[0,0,0,0,0,0,0,0,0,0,0,0,0,1]]C1532714	juvenile xanthogranuloma of skin	[[1,0,0,0,0,0,0,0,0,0,0,0,0,0]]C1532715	kappa light chain myeloma	[[1,0,0,0,0,0,0,0,0,0,0,0,0,0]]C1838854	aminoglycoside induced deafness,streptomycin induced deafness,streptomycin ototoxicity	[[0,0,0,1,0,0,0,0,0,0,0,0,0,0],[0,0,0,1,0,0,0,0,0,0,0,0,0,0],[0,0,0,1,0,0,0,0,0,0,0,0,0,0]]C0410640	degenerative thoracic spinal stenosis	[[1,0,0,0,0,0,0,0,0,0,0,0,0,0]]C0410641	idiopathic thoracic spinal stenosis	[[1,0,0,0,0,0,0,0,0,0,0,0,0,0]]C1955708	primary central nervous system lymphoma involving lymph nodes of multiple sites	[[0,0,0,0,1,0,0,0,0,0,0,0,0,0]]C0866168	glomerulonephritis with lesion of interstitial nephritis	[[0,0,0,0,0,0,0,0,0,0,0,0,0,1]]C0866169	nephritis with lesion of exudative nephritis	[[0,0,0,0,0,0,0,0,0,0,0,0,0,1]]C0866162	nephritis with renal papillary necrosis	[[0,0,0,0,0,0,0,0,0,0,0,0,0,1]]C0866163	nephropathy with medullary necrosis	[[0,0,0,0,0,0,0,0,0,0,0,0,0,1]]C0866160	nephritis with renal medullary necrosis,nephritis with medullary necrosis	[[0,0,0,0,0,0,0,0,0,0,0,0,0,1],[0,0,0,0,0,0,0,0,0,0,0,0,0,1]]C0866161	nephritis with papillary necrosis	[[0,0,0,0,0,0,0,0,0,0,0,0,0,1]]C0866166	nephropathy with renal papillary necrosis	[[0,0,0,0,0,0,0,0,0,0,0,0,0,1]]C0866167	glomerulonephritis with lesion of exudative nephritis	[[0,0,0,0,0,0,0,0,0,0,0,0,0,1]]C1278533	disorder of neuromuscular transmission	[[1,0,0,0,0,0,0,0,0,0,0,0,0,0]]C0866165	nephropathy with papillary necrosis	[[0,0,0,0,0,0,0,0,0,0,0,0,0,1]]C0746502	menopausal bleeding	[[0,0,0,0,0,0,0,1,0,0,0,0,0,0]]C1313885	hereditary edema of legs,hereditary trophedema	[[0,0,0,0,1,0,0,0,0,0,0,0,0,0],[0,0,0,0,0,0,0,0,0,0,0,0,0,1]]C1313884	congenital elephantiasis,hereditary elaphantiasis	[[1,0,0,0,0,0,0,0,0,0,0,0,0,0],[0,0,0,0,0,0,0,0,0,0,1,0,0,0]]C1852523	costocoracoid ligament congenitally short	[[0,1,0,1,0,0,0,0,0,0,0,0,0,0]]C3163931	carcinoma of nose,malignant epithelial neoplasm of nose	[[1,0,0,0,0,0,0,0,0,0,0,0,0,0],[1,0,0,0,0,0,0,0,0,0,0,0,0,0]]C0152935	cellulocutaneous plague,cutaneous plague	[[0,0,0,0,1,0,0,0,0,0,0,0,0,0],[1,0,0,0,0,0,0,0,0,0,0,0,0,0]]C0152937	primary pneumonic plague	[[0,0,0,0,1,0,0,0,0,0,0,0,0,0]]C0152936	septicemic plague,septicaemic plague,black death,black plague	[[0,1,0,0,1,0,0,0,0,0,1,0,0,0],[1,0,0,0,0,0,0,0,0,0,0,0,0,0],[0,1,0,0,0,0,0,0,0,0,0,0,0,0],[0,1,0,0,0,0,0,0,0,0,0,0,0,0]]C0152938	secondary pneumonic plague	[[0,0,0,0,1,0,0,0,0,0,0,0,0,0]]C0600298	periodontosis,periodontoses	[[0,1,0,0,0,0,0,1,0,0,0,0,0,0],[0,1,0,0,0,0,0,0,0,0,0,0,0,0]]C0862422	malignant neoplasm of bladder stage iv	[[0,0,0,0,0,0,1,0,0,0,0,0,0,0]]C0263109	loin abscess,abscess of groin,inguinal abscess	[[1,0,0,0,0,0,0,0,0,0,0,0,0,0],[1,0,0,0,0,0,0,1,0,0,0,0,0,0],[0,0,0,0,0,0,0,1,0,0,0,0,0,0]]C0263108	abscess of flank	[[1,0,0,0,0,0,0,0,0,0,0,0,0,0]]C0263105	abscess of abdominal wall	[[1,0,0,0,0,0,0,1,0,0,0,0,0,0]]C0263107	abscess of chest wall	[[1,0,0,0,0,0,0,0,0,0,0,0,0,0]]C0263106	except buttock abscess of back	[[1,0,0,0,0,0,0,0,0,0,0,0,0,0]]C0263101	abscess of external nose	[[1,0,0,0,0,0,0,0,0,0,0,0,0,0]]C0263100	abscess of forehead	[[1,0,0,0,0,0,0,0,0,0,0,0,0,0]]C0263103	abscess of temple region	[[1,0,0,0,0,0,0,0,0,0,0,0,0,0]]C0263102	abscess of submandibular region	[[1,0,0,0,0,0,0,0,0,0,0,0,0,0]]C2733399	neoplasm of lymphoid system structure	[[1,0,0,0,0,0,0,0,0,0,0,0,0,0]]C0153084	hepatitis e with hepatic coma	[[0,0,0,0,1,0,0,0,0,0,0,0,0,0]]C0810031	liver disease alcohol related	[[0,0,0,0,0,0,0,1,0,0,0,0,0,0]]C1274801	melanotic macule of oral mucosa	[[1,0,0,0,0,0,0,0,0,0,0,0,0,0]]C0153089	viral hepatitis with hepatic coma	[[1,0,0,0,0,0,0,0,0,0,0,0,0,0]]C0018891	animal helminthiasis,animal helminthiases	[[0,1,0,0,0,0,0,0,0,0,0,0,1,0],[0,1,0,0,0,0,0,0,0,0,0,0,0,0]]C2933083	vulvar dysesthesia localized in the vestibule	[[0,1,0,0,0,0,0,0,0,0,0,0,0,0]]C1836254	holoprosencephaly 8,hpe8	[[0,0,0,1,0,0,0,0,0,0,0,0,0,0],[0,0,0,1,0,0,0,0,0,0,0,0,0,0]]C1328385	breast adenoma	[[0,0,0,0,0,0,0,0,0,0,0,1,0,0]]C0852030	pulmonary embolism and thrombosis	[[0,0,0,0,0,0,0,1,0,0,0,0,0,0]]C1518714	ovarian endometrioid stromal sarcoma	[[0,0,0,0,0,0,0,0,0,0,0,1,0,0]]C0027613	neonatal hepatitis,giant cell hepatitis	[[1,0,0,0,0,0,0,1,1,0,0,1,0,0],[0,0,0,1,0,0,0,1,0,0,0,0,0,0]]C0027612	and neonatal diseases and abnormalities hereditary congenital	[[0,0,0,0,0,0,0,0,0,0,0,0,1,0]]C0027611	neonatal dacryocystitis and conjunctivitis	[[1,0,0,0,1,0,0,0,0,0,0,0,0,0]]C1850451	1 neuronal ceroid lipofuscinosis,cln1,variable age at onset 1 neuronal ceroid lipofuscinosis	[[0,0,0,1,0,0,0,0,0,0,0,0,0,0],[0,0,0,1,0,0,0,0,0,0,0,0,0,0],[0,0,0,1,0,0,0,0,0,0,0,0,0,0]]C0032109	plasma cell mastitis	[[1,0,0,0,0,0,0,0,0,0,0,0,0,1]]C1859536	complementation group c type ii bare lymphocyte syndrome	[[0,0,0,1,0,0,0,0,0,0,0,0,0,0]]C2751764	aipl1 related cone rod dystrophy	[[0,0,0,1,0,0,1,0,0,0,0,0,0,0]]C1302839	focal acral hyperkeratosis	[[1,0,0,0,0,0,0,0,0,0,0,0,0,0]]C1335520	signet ring variant acinar prostate adenocarcinoma,signet ring cell carcinoma of the prostate	[[0,0,0,0,0,0,0,0,0,0,0,1,0,0],[0,0,0,0,0,0,0,0,0,0,0,1,0,0]]C1290602	persistent dorsal mesentery,persistent common mesentery	[[1,0,0,0,0,0,0,0,0,0,0,0,0,0],[1,0,0,0,0,0,0,0,0,0,0,0,0,0]]C1290607	cerebrofacial dysplasia	[[1,0,0,0,0,0,0,0,0,0,0,0,0,0]]C1708648	laryngeal acantholytic squamous cell carcinoma	[[0,0,0,0,0,0,0,0,0,0,0,1,0,0]]C1856111	and ventricular septal defect polysyndactyly of big toes hirschsprung disease with ulnar polydactyly	[[0,0,0,1,0,0,0,0,0,0,0,0,0,0]]C1856110	al gazali hirschsprung syndrome,and dysmorphic facial features hypoplastic nails hirschsprungs disease,and minor dysmorphic features hypoplastic nails hirschsprungs disease,al gazali donnai mueller syndrome	[[0,1,0,0,0,0,0,0,0,0,0,0,0,0],[0,1,0,1,0,0,0,0,0,0,0,0,0,0],[0,1,0,0,0,0,0,0,0,0,0,0,0,0],[0,1,0,0,0,0,0,0,0,0,0,0,0,0]]C1856113	mowat wilson syndrome,hirschsprung disease mental retardation syndrome,with or without hirschsprung disease and distinct facial features mental retardation microcephaly	[[0,1,0,1,0,0,0,0,0,0,0,1,0,0],[0,1,0,1,0,0,0,0,0,0,0,0,0,0],[0,0,0,1,0,0,0,0,0,0,0,0,0,0]]C0035459	atrophic rhinitis,atrophic rhinitides	[[0,1,0,1,0,0,0,1,1,0,1,0,1,1],[0,1,0,0,0,0,0,0,0,0,0,0,0,0]]C0035457	perennial allergic rhinitis,nonseasonal allergic rhinitis,atopic rhinitis	[[1,1,0,0,0,0,0,1,0,0,0,1,0,0],[1,0,0,0,0,0,0,0,0,0,0,0,1,0],[0,0,0,0,0,0,0,1,0,0,0,0,0,0]]C0035455	rhinitis,rhinitides,nasal catarrh	[[0,0,1,0,0,0,0,0,0,1,0,0,0,0],[0,1,0,0,0,0,0,0,0,0,0,0,0,0],[0,1,0,0,0,0,0,0,0,0,0,0,1,0]]C0948750	salivary gland carcinoma	[[0,0,0,0,0,0,1,1,0,0,0,1,0,0]]C0684717	benign neoplasm of soft tissues of axilla	[[1,0,0,0,0,0,0,0,0,0,0,0,0,0]]C0684716	secondary malignant neoplasm of soft tissues of thorax,metastatic malignant neoplasm to soft tissues of thorax	[[1,0,0,0,0,0,0,0,0,0,0,0,0,0],[1,0,0,0,0,0,0,0,0,0,0,0,0,0]]C0684715	neoplasm of uncertain behavior of soft tissues of thorax,neoplasm of uncertain behaviour of soft tissues of thorax	[[1,0,0,0,0,0,0,0,0,0,0,0,0,0],[1,0,0,0,0,0,0,0,0,0,0,0,0,0]]C0280303	lower gingival squamous cell carcinoma,squamous cell carcinoma of the lower gingiva,squamous cell carcinoma of the lower gum,scc of the lower gingiva,lower gingival scc,scc of the lower gum	[[0,0,0,0,0,0,0,0,0,0,0,1,0,0],[0,0,0,0,0,0,0,0,0,0,0,1,0,0],[0,0,0,0,0,0,0,0,0,0,0,1,0,0],[0,0,0,0,0,0,0,0,0,0,0,1,0,0],[0,0,0,0,0,0,0,0,0,0,0,1,0,0],[0,0,0,0,0,0,0,0,0,0,0,1,0,0]]C0684713	secondary malignant neoplasm of soft tissues of trunk,metastatic malignant neoplasm to soft tissues of trunk	[[1,0,0,0,0,0,0,0,0,0,0,0,0,0],[1,0,0,0,0,0,0,0,0,0,0,0,0,0]]C0684712	primary malignant neoplasm of soft tissues of trunk	[[1,0,0,0,0,0,0,0,0,0,0,0,0,0]]C0684711	neoplasm of uncertain behavior of soft tissues of trunk,neoplasm of uncertain behaviour of soft tissues of trunk	[[1,0,0,0,0,0,0,0,0,0,0,0,0,0],[1,0,0,0,0,0,0,0,0,0,0,0,0,0]]C0684710	benign neoplasm of soft tissues of trunk	[[1,0,0,0,0,0,0,0,0,0,0,0,0,0]]C0585104	left lower zone pneumonia,left basal pneumonia,lll left lower lobe pneumonia,llz left lower zone pneumonia,left lower lobe pneumonia	[[1,0,0,0,0,0,0,0,0,0,0,0,0,0],[1,0,0,0,0,0,0,1,0,0,0,0,0,0],[1,0,0,0,0,0,0,0,0,0,0,0,0,0],[1,0,0,0,0,0,0,0,0,0,0,0,0,0],[1,0,0,0,0,0,0,1,0,0,0,0,0,0]]C3149848	homozygous variant variegate porphyria	[[0,0,0,1,0,0,0,0,0,0,0,0,0,0]]C0684719	primary malignant neoplasm of soft tissues of axilla	[[1,0,0,0,0,0,0,0,0,0,0,0,0,0]]C0585105	right lower zone pneumonia,right basal pneumonia,right lower lobe pneumonia,rll right lower lobe pneumonia,rlz right lower zone pneumonia	[[1,0,0,0,0,0,0,0,0,0,0,0,0,0],[1,0,0,0,0,0,0,0,0,0,0,0,0,0],[1,0,0,0,0,0,0,1,0,0,0,0,0,0],[1,0,0,0,0,0,0,0,0,0,0,0,0,0],[1,0,0,0,0,0,0,0,0,0,0,0,0,0]]C0585106	right upper zone pneumonia,rul right upper lobe pneumonia,right upper lobe pneumonia,ruz right upper zone pneumonia	[[1,0,0,0,0,0,0,0,0,0,0,0,0,0],[1,0,0,0,0,0,0,0,0,0,0,0,0,0],[1,0,0,0,0,0,0,0,0,0,0,0,0,0],[1,0,0,0,0,0,0,0,0,0,0,0,0,0]]C1275172	verrucous bowens disease	[[1,0,0,0,0,0,0,0,0,0,0,0,0,0]]C0280308	lower gingival verrucous carcinoma,verrucous carcinoma of the lower gingiva,verrucous carcinoma of the lower gum	[[0,0,0,0,0,0,0,0,0,0,0,1,0,0],[0,0,0,0,0,0,0,0,0,0,0,1,0,0],[0,0,0,0,0,0,0,0,0,0,0,1,0,0]]C0264458	pneumonitis due to fumes and or vapors,pneumonitis due to fumes and or vapours,chemical pneumonia,chemical workers lung,chemical pneumonitis,toxic pneumonitis	[[1,0,0,0,0,0,0,0,0,0,0,0,0,0],[1,0,0,0,0,0,0,0,0,0,0,0,0,0],[1,0,0,0,0,0,0,1,0,0,0,0,0,0],[1,0,0,0,0,0,0,0,0,0,0,0,0,0],[1,0,1,0,0,0,0,1,0,0,0,0,0,0],[1,0,0,0,0,0,0,1,0,0,0,0,0,0]]C0264459	chronic diffuse emphysema due to inhalation of chemical fumes and or vapors,chronic diffuse emphysema due to inhalation of chemical fumes and or vapours	[[1,0,0,0,0,0,0,0,0,0,0,0,0,0],[1,0,0,0,0,0,0,0,0,0,0,0,0,0]]C0264456	copper fever	[[1,0,0,0,0,0,0,0,0,0,0,0,0,0]]C0264454	spelters fever,zinc chills,zinc fume fever,galvanizers poisoning,zinc poisoning tremor,galvanisers poisoning	[[1,0,0,0,0,0,0,0,0,0,0,0,0,0],[0,0,0,0,0,0,0,0,0,0,1,0,0,0],[1,0,0,0,0,0,0,0,0,0,0,0,0,0],[1,0,0,0,0,0,0,0,0,0,0,0,0,0],[1,0,0,0,0,0,0,0,0,0,0,0,0,0],[1,0,0,0,0,0,0,0,0,0,0,0,0,0]]C0264455	bronchitis due to fumes and or vapors,bronchitis due to fumes and or vapours,acute chemical bronchitis,acute bronchitis due to chemical fumes	[[1,0,0,0,0,0,0,0,0,0,0,0,0,0],[1,0,0,0,0,0,0,0,0,0,0,0,0,0],[0,0,0,0,0,0,0,0,0,0,0,0,0,1],[1,0,0,0,0,0,0,0,0,0,0,0,0,0]]C0264450	sisal workers disease	[[1,0,0,0,0,0,0,0,0,0,0,0,0,0]]C0264451	weavers cough	[[1,0,0,0,0,0,0,0,0,0,0,0,0,0]]C1410262	stress induced epilepsy	[[1,0,0,0,0,0,0,0,0,0,0,0,0,0]]C0151620	hypertensive encephalopathy	[[0,0,1,0,1,0,0,1,1,0,1,0,1,0]]C0151622	endometrial disorder	[[1,0,0,0,0,0,0,0,1,0,0,1,0,0]]C0151623	hemorrhagic enterocolitis,enterocolitis haemorrhagic	[[0,0,0,0,0,0,0,1,1,0,0,0,0,0],[0,0,0,0,0,0,0,1,0,0,0,0,0,0]]C0151624	ulcerative enterocolitis	[[1,0,0,0,0,0,0,0,1,0,0,0,0,0]]C2242796	sickle cell thalassemia disease,sickle cell thalassaemia disease,hemoglobin s f disease,haemoglobin s f disease,microdrepanocytic disease,hemoglobin s thalassemia,microdrepanocytosis,thalassemia hemoglobin s disease,thalassaemia haemoglobin s disease,sickle cell thalassemia	[[1,0,0,0,0,0,0,0,0,0,1,0,0,0],[1,0,0,0,0,0,0,0,0,0,0,0,0,0],[1,0,0,0,0,0,0,0,0,0,0,0,0,0],[1,0,0,0,0,0,0,0,0,0,0,0,0,0],[1,0,0,0,0,0,0,0,0,0,1,0,0,0],[0,0,0,0,0,0,0,0,0,0,1,0,0,0],[0,0,0,0,0,0,0,0,0,0,0,0,0,1],[1,0,0,0,0,0,0,0,0,0,0,0,0,0],[1,0,0,0,0,0,0,0,0,0,0,0,0,0],[0,0,0,0,0,0,0,0,0,0,0,1,0,1]]C0151626	epidermal necrolysis	[[0,0,0,0,0,0,0,1,1,0,0,0,0,0]]C2242794	disfigurement of neck and trunk	[[0,0,0,0,0,0,1,0,0,0,0,0,0,0]]C0017547	gigantism,giant,pituitary gigantism,genetic giant,normal giant,primordial giant,giantism,gigantism disorder	[[0,1,0,0,0,0,0,0,1,1,0,0,0,0],[1,0,0,0,0,0,0,1,0,0,0,0,0,0],[1,1,0,0,0,0,0,0,0,0,1,0,1,0],[1,0,0,0,0,0,0,1,0,0,0,0,0,0],[1,0,0,0,0,0,0,0,0,0,0,0,0,0],[1,0,0,0,0,0,0,0,0,0,0,0,0,0],[1,0,0,0,0,0,0,1,0,0,0,0,0,0],[0,0,0,0,0,0,0,1,0,0,0,0,0,0]]C1321581	bezoar disorder,bezoar	[[0,0,0,0,0,0,1,0,0,0,0,0,0,0],[0,0,0,0,0,0,0,1,0,0,1,0,0,0]]C1321580	trichobezoar disorder,hair balls,trichobezoars	[[0,0,0,0,0,0,1,0,0,0,0,0,0,0],[0,1,0,0,0,0,0,0,0,0,0,0,0,0],[0,1,0,0,0,0,0,1,0,0,0,0,0,0]]C0034089	pulmonary valve stenosis,pulmonary valve stenoses,pulmonary stenosis,pulmonic stenosis,pure stenosis pulmonary valve,pulmonic valve stenosis,ps pulmonary valve stenosis,valvular pulmonic stenosis,valvular pulmonic stenoses,pvs pulmonary valve stenosis	[[0,1,0,1,0,0,0,1,0,0,1,0,1,0],[0,0,0,0,0,0,0,0,0,0,0,0,1,0],[0,0,0,0,0,0,0,1,0,0,0,0,0,0],[0,0,0,0,0,0,0,1,0,0,0,0,0,0],[0,0,0,0,0,0,0,0,0,0,1,0,0,0],[0,0,0,1,0,0,0,1,1,0,1,0,0,0],[1,0,0,0,0,0,0,0,0,0,0,0,0,0],[0,1,0,0,0,0,0,1,0,0,0,0,1,0],[0,1,0,0,0,0,0,0,0,0,0,0,0,0],[1,0,0,0,0,0,0,0,0,0,0,0,0,0]]C2936502	familial charge syndrome	[[0,1,0,0,0,0,0,0,0,0,0,0,0,0]]C0154050	hemangioma of intracranial structure,haemangioma of intracranial structure,intracranial hemangioma,intracranial angioma,angioma of the intracranial structure	[[1,0,0,0,1,0,0,0,0,0,0,1,0,0],[1,0,0,0,0,0,0,0,0,0,0,0,0,0],[0,0,0,0,0,0,0,0,0,0,0,1,0,0],[0,0,0,0,0,0,0,0,0,0,0,1,0,0],[0,0,0,0,0,0,0,0,0,0,0,1,0,0]]C0154051	hemangioma of retina,haemangioma of retina,retinal angioma,retinal angiomata,angioma of retina,retinal hemangioma,retinal haemangioma	[[0,0,0,0,1,0,0,0,0,0,0,1,0,0],[1,0,0,0,0,0,0,0,0,0,0,0,0,0],[1,0,0,0,0,0,0,0,0,0,0,1,0,0],[0,0,0,1,0,0,0,0,0,0,0,0,0,0],[0,0,0,0,0,0,0,0,0,0,0,1,0,0],[1,0,0,0,0,0,0,0,0,0,0,1,0,0],[1,0,0,0,0,0,0,0,0,0,0,0,0,0]]C0154052	hemangioma of intra abdominal structure,haemangioma of intra abdominal structures,intra abdominal hemangioma	[[1,0,0,0,1,0,0,0,0,0,0,1,0,0],[1,0,0,0,0,0,0,0,0,0,0,0,0,0],[0,0,0,0,0,0,0,0,0,0,0,1,0,0]]C0154054	benign neoplasm of lymph nodes,benign lymph node tumor	[[1,0,0,0,1,0,1,0,0,0,0,1,0,0],[0,0,0,0,0,0,0,0,0,0,0,1,0,0]]C0028271	noma,cancrum oris,gangrenous stomatitis,gangrenous stomatitides,acute gangrenous stomatitis	[[0,1,0,0,0,0,0,1,1,0,0,1,0,0],[0,0,0,0,1,0,0,1,0,0,0,0,1,0],[1,1,0,0,0,0,0,1,0,0,0,0,1,0],[0,1,0,0,0,0,0,0,0,0,0,0,0,0],[1,0,0,0,0,0,0,0,0,0,0,0,0,0]]C0154057	carcinoma in situ of digestive organ	[[1,0,0,0,1,0,0,0,0,0,0,0,0,1]]C0154058	oral cavity and pharynx carcinoma in situ of lip	[[1,0,0,0,1,0,0,0,0,0,0,0,0,0]]C0154059	carcinoma in situ of esophagus,carcinoma in situ of oesophagus,stage 0 esophageal cancer,stage 0 esophagus cancer,severe esophageal dysplasia,severe oesophageal dysplasia,esophageal carcinoma in situ,esophageal carcinoma in situ ajcc v7,severe esophageal dysplasia ajcc v7,stage 0 esophageal carcinoma in situ,stage 0 esophageal cancer ajcc v7	[[1,0,0,0,0,0,0,0,0,0,0,0,0,0],[1,0,0,0,0,0,0,0,0,0,0,0,0,0],[0,0,0,0,0,1,0,0,0,0,0,1,0,0],[0,0,0,0,0,1,0,0,0,0,0,0,0,0],[1,0,0,0,0,0,0,0,0,0,0,1,0,0],[1,0,0,0,0,0,0,0,0,0,0,0,0,0],[0,0,0,0,0,0,0,0,0,0,0,1,0,0],[0,0,0,0,0,0,0,0,0,0,0,1,0,0],[0,0,0,0,0,0,0,0,0,0,0,1,0,0],[0,0,0,0,0,0,0,0,0,0,0,1,0,0],[0,0,0,0,0,0,0,0,0,0,0,1,0,0]]C1275170	intraepidermal squamous carcinoma of trunk,bowens disease of trunk	[[1,0,0,0,0,0,0,0,0,0,0,0,0,0],[1,0,0,0,0,0,0,0,0,0,0,0,0,0]]C1320188	escherichia coli infection of the central nervous system	[[1,0,0,0,0,0,0,0,0,0,0,0,0,0]]C1320189	gonococcal infection of the central nervous system	[[1,0,0,0,0,0,0,0,0,0,0,0,0,0]]C0854798	recurrent hepatoblastoma,relapsed hepatoblastoma	[[0,0,0,0,0,0,0,0,0,0,0,1,0,0],[0,0,0,0,0,0,0,0,0,0,0,1,0,0]]C0854794	recurrent liver cancer,recurrent liver carcinoma,recurrent hepatic cancer,relapsed hepatic cancer,relapsed liver cancer	[[0,0,0,0,0,0,0,0,0,0,0,1,0,0],[0,0,0,0,0,0,0,0,0,0,0,1,0,0],[0,0,0,0,0,0,0,0,0,0,0,1,0,0],[0,0,0,0,0,0,0,0,0,0,0,1,0,0],[0,0,0,0,0,0,0,0,0,0,0,1,0,0]]C0854795	resectable hepatic malignant neoplasm,resectable malignant neoplasm of liver	[[0,0,0,0,0,0,0,0,0,0,0,1,0,0],[0,0,0,0,0,0,0,0,0,0,0,1,0,0]]C0854796	unresectable hepatoblastoma,non resectable hepatoblastoma,nonresectable hepatoblastoma	[[0,0,0,0,0,0,0,0,0,0,0,1,0,0],[0,0,0,0,0,0,0,0,0,0,0,1,0,0],[0,0,0,0,0,0,0,0,0,0,0,1,0,0]]C0854797	resectable hepatoblastoma	[[0,0,0,0,0,0,0,0,0,0,0,1,0,0]]C1320186	enterovirus infection of the central nervous system	[[1,0,0,0,0,0,0,0,0,0,0,0,0,0]]C1320187	epstein barr virus infection of the central nervous system	[[1,0,0,0,0,0,0,0,0,0,0,0,0,0]]C0854792	resectable extrahepatic bile duct carcinoma,resectable bile duct cancer	[[0,0,0,0,0,0,0,0,0,0,0,1,0,0],[0,0,0,0,0,0,0,0,0,0,0,1,0,0]]C0854793	non resectable hepatic malignant neoplasm,non resectable malignant neoplasm of liver	[[0,0,0,0,0,0,0,0,0,0,0,1,0,0],[0,0,0,0,0,0,0,0,0,0,0,1,0,0]]C1863599	hereditary myopathy with early respiratory failure,hmerf,mprm,with early respiratory muscle involvement proximal myopathy,edstrom myopathy	[[0,0,0,1,0,0,0,0,0,0,0,0,0,0],[0,0,0,1,0,0,0,0,0,0,0,0,0,0],[0,0,0,1,0,0,0,0,0,0,0,0,0,0],[0,0,0,1,0,0,0,0,0,0,0,0,0,0],[0,0,0,1,0,0,0,0,0,0,0,0,0,0]]C0405479	disorder of nipple	[[1,0,0,0,0,0,0,1,0,0,0,0,0,0]]C0405478	lymphatic cyst of breast	[[1,0,0,0,0,0,0,0,0,0,0,0,0,0]]C1834690	autosomal dominant lower extremity spinal muscular atrophy,autosomal dominant proximal childhood spinal muscular atrophy,autosomal dominant proximal juvenile spinal muscular atrophy,autosomal dominant kugelberg welander syndrome,sma led,smaled	[[0,0,0,1,0,0,0,0,0,0,0,0,0,0],[0,0,0,1,0,0,0,0,0,0,0,0,0,0],[0,0,0,1,0,0,0,0,0,0,0,0,0,0],[0,0,0,1,0,0,0,0,0,0,0,0,0,0],[0,0,0,1,0,0,0,0,0,0,0,0,0,0],[0,0,0,1,0,0,0,0,0,0,0,0,0,0]]C1275171	atrophic bowens disease	[[1,0,0,0,0,0,0,0,0,0,0,0,0,0]]C0405471	pubertal breast hypertrophy,juvenile mammary hypertrophy,diffuse hypertrophy of breast,juvenile gigantomastia,jhb,juvenile hypertrophy of the breast	[[1,0,0,0,0,0,0,0,0,0,0,0,0,0],[1,0,0,0,0,0,0,0,0,0,0,0,0,0],[1,0,0,0,0,0,0,0,0,0,0,0,0,0],[0,1,0,1,0,0,0,0,0,0,0,0,0,0],[0,0,0,1,0,0,0,0,0,0,0,0,0,0],[0,0,0,1,0,0,0,0,0,0,0,0,0,0]]C0405470	actinomycosis of breast	[[1,0,0,0,0,0,0,0,0,0,0,0,0,0]]C0405477	hydatid cyst of breast	[[1,0,0,0,0,0,0,0,0,0,0,0,0,0]]C0405476	cutaneous papilloma of breast	[[1,0,0,0,0,0,0,0,0,0,0,0,0,0]]C1290372	neoplasm of mesothelial tissue of pleura	[[1,0,0,0,0,0,0,0,0,0,0,0,0,0]]C1857242	type 2 rhizomelic chondrodysplasia punctata,dhapat deficiency,glyceronephosphate o acyltransferase deficiency,due to dihydroxyacetonephosphate acyltransferase deficiency rhizomelic chondrodysplasia punctata,dihydroxyacetonephosphate acyltransferase deficiency,rcdp2,peroxisomal dihydroxyacetonephosphate acyltransferase deficiency,gnpat deficiency,human dihydroxyacetonephosphate acyltransferase deficiency,due to dhapat deficiency rhizomelic chondrodysplasia punctata,glyceronephosphate acyltransferase deficiency	[[0,1,0,1,0,0,0,0,0,0,0,0,0,0],[0,1,0,1,0,0,0,0,0,0,0,0,0,0],[0,0,0,1,0,0,0,0,0,0,0,0,0,0],[0,0,0,1,0,0,0,0,0,0,0,0,0,0],[0,1,0,1,0,0,0,0,0,0,0,0,0,0],[0,0,0,1,0,0,0,0,0,0,0,0,0,0],[0,1,0,1,0,0,0,0,0,0,0,0,0,0],[0,1,0,1,0,0,0,0,0,0,0,0,0,0],[0,1,0,0,0,0,0,0,0,0,0,0,0,0],[0,1,0,0,0,0,0,0,0,0,0,0,0,0],[0,1,0,0,0,0,0,0,0,0,0,0,0,0]]C1720669	platelet dysfunction associated with uremia,platelet dysfunction associated with uraemia,uremic platelet dysfunction,uraemic platelet dysfunction	[[1,0,0,0,0,0,0,0,0,0,0,0,0,0],[1,0,0,0,0,0,0,0,0,0,0,0,0,0],[1,0,0,0,0,0,0,0,0,0,0,0,0,0],[1,0,0,0,0,0,0,0,0,0,0,0,0,0]]C2607948	tuberculous abscess of brain,tuberculous intracranial abscess	[[1,0,0,0,0,0,0,0,0,0,0,0,0,0],[1,0,0,0,0,0,0,0,0,0,0,0,0,0]]C1834481	1s dilated cardiomyopathy,cmd1s	[[0,0,0,1,0,0,0,0,0,0,0,0,0,0],[0,0,0,1,0,0,0,0,0,0,0,0,0,0]]C1275176	clear cell bowens disease	[[1,0,0,0,0,0,0,0,0,0,0,0,0,0]]C0560041	focal pyelonephritis,acute focal pyelonephritis	[[1,0,0,0,0,0,0,0,0,0,0,0,0,0],[1,0,0,0,0,0,0,0,0,0,0,0,0,0]]C1297950	malignant tumor involving vasa deferentia by direct extension from prostate,malignant tumour involving vasa deferentia by direct extension from prostate	[[1,0,0,0,0,0,0,0,0,0,0,0,0,0],[1,0,0,0,0,0,0,0,0,0,0,0,0,0]]C1112360	bronchial polyps	[[0,0,0,1,0,0,0,0,0,0,0,0,0,0]]C1297952	malignant tumor involving prostate by direct extension from bladder,malignant tumour involving prostate by direct extension from bladder	[[1,0,0,0,0,0,0,0,0,0,0,0,0,0],[1,0,0,0,0,0,0,0,0,0,0,0,0,0]]C1297953	malignant tumor involving prostate by separate metastasis from bladder,malignant tumour involving prostate by separate metastasis from bladder	[[1,0,0,0,0,0,0,0,0,0,0,0,0,0],[1,0,0,0,0,0,0,0,0,0,0,0,0,0]]C0339879	fibrinous chorditis	[[0,0,0,0,0,0,0,0,0,0,0,0,0,1]]C1297955	secondary malignant neoplasm of seminal vesicle	[[1,0,0,0,0,0,0,0,0,0,0,0,0,0]]C1297956	malignant tumor involving seminal vesicle by direct extension from bladder,malignant tumour involving seminal vesicle by direct extension from bladder	[[1,0,0,0,0,0,0,0,0,0,0,0,0,0],[1,0,0,0,0,0,0,0,0,0,0,0,0,0]]C0730233	alpha 2 antitrypsin deficiency	[[1,0,0,0,0,0,0,0,0,0,0,0,0,0]]C0339875	acute subglottic laryngitis,pseudocroup	[[1,0,0,0,0,0,0,0,0,0,0,0,0,0],[1,0,0,0,0,0,0,0,0,0,0,0,0,0]]C0339874	acute membranous laryngitis,pseudomembranous croup	[[1,0,0,0,0,0,0,0,0,0,0,0,0,0],[1,0,0,0,0,0,0,0,0,0,0,0,0,0]]C0339877	acute tracheitis without obstruction	[[1,0,0,0,0,0,0,0,0,0,0,0,0,0]]C0339876	acute laryngotracheitis without obstruction	[[1,0,0,0,0,0,0,0,0,0,0,0,0,0]]C0564754	radiation respiratory disease	[[1,0,0,0,0,0,0,0,0,0,0,0,0,0]]C0564755	acute perforated appendicitis,appendicitis perforated	[[1,0,0,0,0,0,0,0,0,0,0,1,0,0],[0,0,0,0,0,0,0,0,0,0,0,1,0,0]]C0339873	vincents laryngitis	[[1,0,0,0,0,0,0,0,0,0,0,0,0,0]]C0339872	acute catarrhal laryngitis	[[1,0,0,0,0,0,0,0,0,0,0,0,0,0]]C1275177	multiple intraepidermal squamous carcinomata	[[1,0,0,0,0,0,0,0,0,0,0,0,0,0]]C0572014	deoxyribonuclease allergy	[[1,0,0,0,0,0,0,0,0,0,0,0,0,0]]C0572015	hyaluronidase allergy	[[1,0,0,0,0,0,0,0,0,0,0,0,0,0]]C0572016	streptokinase streptodornase allergy	[[1,0,0,0,0,0,0,0,0,0,0,0,0,0]]C0572017	chymotrypsin allergy	[[1,0,0,0,0,0,0,0,0,0,0,0,0,0]]C0572010	urokinase allergy	[[1,0,0,0,0,0,0,0,0,0,0,0,0,0]]C0572011	alteplase allergy	[[1,0,0,0,0,0,0,0,0,0,0,0,0,0]]C0572012	anistreplase allergy	[[1,0,0,0,0,0,0,0,0,0,0,0,0,0]]C0572013	bromelains allergy	[[1,0,0,0,0,0,0,0,0,0,0,0,0,0]]C2749509	autosomal recessive myopia 18,myp18	[[0,0,0,1,0,0,0,0,0,0,0,0,0,0],[0,0,0,1,0,0,0,0,0,0,0,0,0,0]]C1857511	craniofacial dyssynostosis,craniofacial dyssynostosis and short stature,craniosynostosis craniofacial dysostosis syndrome,bilateral lambdoid and sagittal synostosis,blss	[[0,1,0,0,0,0,0,0,0,0,0,0,0,0],[0,1,0,1,0,0,0,0,0,0,0,0,0,0],[0,1,0,0,0,0,0,0,0,0,0,0,0,0],[0,0,0,1,0,0,0,0,0,0,0,0,0,0],[0,0,0,1,0,0,0,0,0,0,0,0,0,0]]C1562309	neoplastic masquerade syndrome	[[1,0,0,0,0,0,0,0,0,0,0,0,0,0]]C1275174	pigmented bowens disease	[[1,0,0,0,0,0,0,0,0,0,0,0,0,0]]C1853899	6 atopic dermatitis,atod6	[[0,0,0,1,0,0,0,0,0,0,0,0,0,0],[0,0,0,1,0,0,0,0,0,0,0,0,0,0]]C1562300	disorder of left cardiac ventricle	[[1,0,0,0,0,0,0,0,0,0,0,0,0,0]]C1998072	disorder of intervertebral disc of thoracic spine,thoracic disc disorder,thoracic intervertebral disc disorder	[[1,0,0,0,0,0,0,0,0,0,0,0,0,0],[1,0,0,0,0,0,0,0,0,0,0,0,0,0],[1,0,0,0,0,0,0,0,0,0,0,0,0,0]]C1304066	fingertip eczema	[[1,0,0,0,0,0,0,0,0,0,0,0,0,0]]C0854135	pseudomonas aeruginosa infection	[[0,0,0,0,0,0,1,1,0,0,0,0,0,0]]C1998075	arthritis of midtarsal joint	[[1,0,0,0,0,0,0,0,0,0,0,0,0,0]]C0279706	thymic lymphoepithelioma like carcinoma,malignant lymphoepithelial thymoma,lymphoepithelial thymus cancer,lymphoepithelioma like thymus carcinoma	[[0,0,0,0,0,0,0,0,0,0,0,1,0,0],[0,0,0,0,0,0,0,0,0,0,0,1,0,0],[0,0,0,0,0,0,0,0,0,0,0,1,0,0],[0,0,0,0,0,0,0,0,0,0,0,1,0,0]]C1334461	salivary gland lymphoepithelial carcinoma	[[0,0,0,0,0,0,0,0,0,0,0,1,0,0]]C1860991	noonan syndrome 3,ns3	[[0,1,0,1,0,0,0,0,0,0,0,0,0,0],[0,0,0,1,0,0,0,0,0,0,0,0,0,0]]C0276363	disease due to vesiculovirus	[[1,0,0,0,0,0,0,0,0,0,0,0,0,0]]C0276362	pike fry rhabdovirus disease	[[1,0,0,0,0,0,0,0,0,0,0,0,0,0]]C0276361	spring viremia of carp,spring viraemia of carp	[[1,0,0,0,0,0,0,0,0,0,0,0,0,0],[1,0,0,0,0,0,0,0,0,0,0,0,0,0]]C0276360	viral hemorrhagic septicemia of trout,viral haemorrhagic septicaemia of trout	[[1,0,0,0,0,0,0,0,0,0,0,0,0,0],[1,0,0,0,0,0,0,0,0,0,0,0,0,0]]C0276367	chandipura virus disease,chandipura fever	[[1,0,0,0,0,0,0,0,0,0,0,0,0,0],[0,0,0,0,0,0,0,0,0,0,0,0,0,1]]C0276366	piry virus disease,piry fever	[[1,0,0,0,0,0,0,0,0,0,0,0,0,0],[0,0,0,0,0,0,0,0,0,0,0,0,0,1]]C0276365	vesicular stomatitis alagoas virus disease	[[1,0,0,0,0,0,0,0,0,0,0,0,0,0]]C0276364	vesicular stomatitis new jersey virus disease	[[1,0,0,0,0,0,0,0,0,0,0,0,0,0]]C0865516	disseminated choroiditis or chorioretinitis	[[0,0,0,0,0,0,0,0,0,0,0,0,0,1]]C0276369	urban rabies	[[1,0,0,0,0,0,0,0,0,0,0,0,0,0]]C0235974	pancreatic carcinoma,carcinoma of pancreas,pancreatic cancer,pancreas cancer,exocrine pancreas carcinoma,pancreatic acinar carcinoma,exocrine cancer	[[0,0,1,1,0,0,0,1,0,0,0,1,0,0],[1,0,1,0,0,0,0,1,0,0,0,1,0,0],[0,0,0,1,0,0,0,0,0,0,0,1,0,0],[0,0,0,0,0,0,0,0,0,0,0,1,0,0],[0,0,0,0,0,0,0,0,0,0,0,1,0,0],[0,0,0,1,0,0,0,0,0,0,0,0,0,0],[0,0,0,0,0,0,0,0,0,0,0,1,0,0]]C0865517	disseminated retinitis or retinochoroiditis	[[0,0,0,0,0,0,0,0,0,0,0,0,0,1]]C1956125	alagille syndrome 1,syndromatic hepatic ductular hypoplasia,algs1	[[0,1,0,1,0,0,0,0,0,0,0,0,0,0],[0,0,0,1,0,0,0,0,0,0,0,0,0,0],[0,0,0,1,0,0,0,0,0,0,0,0,0,0]]C2699776	diffuse large b cell lymphoma associated with chronic inflammation	[[0,0,0,0,0,0,0,0,0,0,0,1,0,0]]C0338588	isolated angiitis of central nervous system,primary central nervous system granulomatous vasculitis	[[1,0,0,0,0,0,0,0,0,0,0,0,0,0],[1,0,0,0,0,0,0,0,0,0,0,0,0,0]]C0338589	granulomatous angiitis of the central nervous system,granulomatous angiitis of cns,granulomatous angiitis of the nervous system	[[1,1,0,0,0,0,0,0,0,0,0,0,0,0],[0,1,0,0,0,0,0,0,0,0,0,0,0,0],[0,1,0,0,0,0,0,0,0,0,0,0,0,0]]C0338586	vertebral artery dissection,dissecting vertebral artery aneurysm	[[1,1,0,0,1,0,0,1,0,0,0,0,1,0],[0,0,0,0,0,0,0,0,0,0,0,0,1,0]]C0338587	cerebral arteritis in giant cell arteritis,cerebral arteritis in temporal arteritis	[[1,0,0,0,0,0,0,0,0,0,0,0,0,0],[1,0,0,0,0,0,0,0,0,0,0,0,0,0]]C0338585	carotid artery dissection	[[1,0,0,0,1,0,0,1,0,0,0,0,0,0]]C0338582	sporadic cerebral amyloid angiopathy	[[1,1,0,0,0,0,0,0,0,0,0,0,0,0]]C0559961	allergic reaction to insect venom	[[1,0,0,0,0,0,0,0,0,0,0,0,0,0]]C0338580	cerebral venous thrombosis of cortical vein	[[1,0,0,0,0,0,0,0,0,0,0,0,0,0]]C0338581	cerebral venous thrombosis of great cerebral vein	[[1,0,0,0,0,0,0,0,0,0,0,0,0,0]]C0155017	blue color blindness,tritan defect,tritanomaly,tritanopia	[[0,1,0,0,0,0,0,0,0,0,1,0,0,0],[0,1,0,0,1,0,0,0,0,0,0,0,0,0],[0,0,0,0,0,0,0,0,0,0,0,0,0,1],[0,0,0,0,0,0,0,0,0,0,0,0,0,1]]C0155016	red green color blindness,red green colour blindness,deutan defect,deuteranomaly,deuteranopia,reduced red green discrimination,reduced red green vision	[[1,1,0,0,0,0,0,1,0,0,0,0,0,0],[1,0,0,0,0,0,0,1,0,0,0,0,0,0],[0,1,0,0,1,0,0,1,0,0,0,0,0,0],[0,0,0,1,0,0,0,0,0,0,0,0,0,1],[0,0,0,1,0,0,0,1,0,0,0,0,0,0],[1,0,0,0,0,0,0,0,0,0,0,0,0,0],[1,0,0,0,0,0,0,0,0,0,0,0,0,0]]C0155015	red color blindness,protan defect,protanomaly,protanopia	[[0,1,0,0,0,0,0,0,0,0,1,0,0,0],[0,1,0,0,1,0,0,0,0,0,0,0,0,0],[0,0,0,1,0,0,0,0,0,0,0,0,0,1],[0,0,0,1,0,0,0,1,0,0,0,0,0,0]]C0279747	salivary gland poorly differentiated carcinoma,poorly differentiated cancer of the salivary gland	[[0,0,0,0,0,1,0,0,0,0,0,0,0,0],[0,0,0,0,0,1,0,0,0,0,0,0,0,0]]C0279740	low grade salivary gland mucoepidermoid carcinoma,grade i salivary gland mucoepidermoid carcinoma	[[0,0,0,0,0,1,0,0,0,0,0,1,0,0],[0,0,0,0,0,0,0,0,0,0,0,1,0,0]]C0155012	generalized visual field contraction or constriction	[[0,0,0,0,1,0,0,0,0,0,0,0,0,0]]C0155011	sector or arcuate visual field defects	[[0,0,0,0,1,0,0,0,0,0,0,0,0,0]]C0155010	abnormal retinal correspondence,arc abnormal retinal correspondence,anomalous retinal correspondence	[[0,0,0,0,1,0,0,0,0,0,0,0,0,0],[1,0,0,0,0,0,0,0,0,0,0,0,0,0],[1,0,0,0,0,0,0,0,0,0,0,0,0,0]]C1833218	8 insulin dependent diabetes mellitus,iddm8	[[0,0,0,1,0,0,0,0,0,0,0,0,0,0],[0,0,0,1,0,0,0,0,0,0,0,0,0,0]]C0279748	undifferentiated carcinoma of nasopharynx,nasopharyngeal lymphoepithelioma,nasopharynx lymphoepithelioma,nasopharyngeal nonkeratinizing undifferentiated carcinoma,nasopharyngeal undifferentiated carcinoma	[[1,0,0,0,0,0,0,0,0,0,0,1,0,0],[0,0,0,0,0,1,0,0,0,0,0,1,0,0],[0,0,0,0,0,1,0,0,0,0,0,1,0,0],[0,0,0,0,0,0,0,0,0,0,0,1,0,0],[0,0,0,0,0,0,0,0,0,0,0,1,0,0]]C0279749	salivary gland anaplastic carcinoma,undifferentiated cancer of the salivary gland,salivary gland undifferentiated carcinoma	[[0,0,0,0,0,1,0,0,0,0,0,1,0,0],[0,0,0,0,0,1,0,0,0,0,0,0,0,0],[0,0,0,0,0,1,0,0,0,0,0,1,0,0]]C0155019	abnormal dark adaptation curve,abnormal threshold of cones,abnormal threshold of rods,delayed adaptation of cones,delayed adaptation of rods	[[0,0,0,0,1,0,0,0,0,0,0,0,0,0],[1,0,0,0,0,0,0,0,0,0,0,0,0,0],[1,0,0,0,0,0,0,0,0,0,0,0,0,0],[1,0,0,0,0,0,0,0,0,0,0,0,0,0],[1,0,0,0,0,0,0,0,0,0,0,0,0,0]]C0155018	acquired color blindness,acquired colour blindness,acquired color vision deficiency,acquired colour vision deficiency	[[1,1,0,0,0,0,0,0,0,0,0,0,0,0],[1,0,0,0,0,0,0,0,0,0,0,0,0,0],[1,0,0,0,1,0,0,0,0,0,0,0,0,0],[1,0,0,0,0,0,0,0,0,0,0,0,0,0]]C0869147	neonatal hyperthyroidism	[[0,0,0,0,0,0,0,0,0,0,0,0,0,1]]C1843632	2 susceptibility to leprosy,lprs2	[[0,0,0,1,0,0,0,0,0,0,0,0,0,0],[0,0,0,1,0,0,0,0,0,0,0,0,0,0]]C1866340	complementation group 3 peroxisome biogenesis disorder,cg3	[[0,0,0,1,0,0,0,0,0,0,0,0,0,0],[0,0,0,1,0,0,0,0,0,0,0,0,0,0]]C0001363	acute vascular insufficiency of intestine,acute mesenteric vascular insufficiency,acute mesenteric ischemia,acute mesenteric ischaemia,acute intestinal ischemia,acute intestinal ischaemia,acute intestinal ischemic syndrome,acute intestinal ischaemic syndrome,ami acute mesenteric ischemia,ami acute mesenteric ischaemia	[[1,0,0,0,1,0,0,0,0,0,0,0,0,0],[0,0,0,0,0,0,0,0,0,0,1,0,0,0],[0,1,0,0,0,0,0,1,0,0,1,0,0,0],[1,0,0,0,0,0,0,1,0,0,0,0,0,0],[1,0,0,0,0,0,0,0,0,0,0,1,0,0],[1,0,0,0,0,0,0,1,0,0,0,0,0,0],[1,0,0,0,0,0,0,0,0,0,0,0,0,0],[1,0,0,0,0,0,0,0,0,0,0,0,0,0],[1,0,0,0,0,0,0,0,0,0,0,0,0,0],[1,0,0,0,0,0,0,0,0,0,0,0,0,0]]C1302741	neoplasm with apocrine differentiation	[[1,0,0,0,0,0,0,0,0,0,0,0,0,0]]C0001361	acute tonsillitis,infective tonsillitis	[[0,0,0,0,1,0,0,1,0,0,1,0,0,0],[1,0,0,0,0,0,0,1,0,0,0,0,0,1]]C0001360	acute thyroiditis	[[0,0,1,0,1,0,0,1,0,0,0,0,0,0]]C0751679	intracranial ganglioglioma	[[0,1,0,0,0,0,0,0,0,0,0,0,0,0]]C0751678	benign ganglioglioma	[[0,1,0,0,0,0,0,0,0,0,0,0,0,0]]C0001365	acute ill defined cerebrovascular disease	[[1,0,0,0,1,0,0,0,0,0,0,0,0,0]]C1302747	basal cell carcinoma with matrical differentiation	[[1,0,0,0,0,0,0,0,0,0,0,0,0,0]]C0751675	cerebral primitive neuroectodermal tumor,cerebral hemisphere primitive neuroectodermal neoplasm,cerebral hemisphere primitive neuroectodermal tumor,cerebral hemisphere pnet,cerebral primitive neuroectodermal neoplasm,cerebral pnet,primitive neuroectodermal neoplasm of the cerebrum,primitive neuroectodermal tumor of the cerebrum,pnet of the cerebrum	[[0,0,0,0,0,0,0,0,0,0,0,1,0,0],[0,0,0,0,0,0,0,0,0,0,0,1,0,0],[0,0,0,0,0,0,0,0,0,0,0,1,0,0],[0,0,0,0,0,0,0,0,0,0,0,1,0,0],[0,0,0,0,0,0,0,0,0,0,0,1,0,0],[0,0,0,0,0,0,0,0,0,0,0,1,0,0],[0,0,0,0,0,0,0,0,0,0,0,1,0,0],[0,0,0,0,0,0,0,0,0,0,0,1,0,0],[0,0,0,0,0,0,0,0,0,0,0,1,0,0]]C0751674	lymphangioleiomyomatosis,lymphangioleiomyomatoses,lam,lymphangiomyomatosis,lymphangiomyomatoses	[[0,0,0,1,0,0,0,1,0,0,0,0,1,0],[0,1,0,0,0,0,0,0,0,0,0,0,0,0],[0,0,0,0,0,0,0,0,0,1,0,0,0,0],[0,0,0,1,0,1,0,1,0,0,0,0,0,0],[0,1,0,0,0,0,0,0,0,0,0,0,0,0]]C0751677	ependymoastrocytoma	[[0,1,0,0,0,0,0,0,0,0,0,0,0,0]]C0751676	basal cell cancer,basal cell skin cancer	[[0,1,0,0,0,0,0,1,0,0,0,0,0,0],[0,0,0,0,0,1,0,0,0,0,0,0,0,0]]C0751671	machado joseph disease type iv	[[0,1,0,0,0,0,0,0,0,0,0,0,0,0]]C0751670	machado joseph disease type iii	[[0,1,0,0,0,0,0,0,0,0,0,0,0,0]]C1853235	sclerocornea	[[0,0,0,1,0,0,0,0,0,0,0,0,0,0]]C2931083	acetylcarnitine deficiency	[[0,1,0,0,0,0,0,0,0,0,0,0,0,0]]C1386407	meningeal adhesions	[[0,0,0,0,0,0,0,0,0,0,0,0,0,1]]C0570519	dextromoramide allergy	[[1,0,0,0,0,0,0,0,0,0,0,0,0,0]]C0570518	methadone analog allergy,methadone analogue allergy	[[1,0,0,0,0,0,0,0,0,0,0,0,0,0],[1,0,0,0,0,0,0,0,0,0,0,0,0,0]]C0271355	abducens nerve diseases,abducens nerve paralysis,sixth or abducens nerve palsy,lateral rectus muscle paralysis,abducens nerve disorder,abducens nerve palsy,abducens nerve paresis,abducens nerve weakness,sixth cranial nerve diseases,sixth cranial nerve disorders,sixth cranial nerve weakness,lateral rectus muscle innervation disorder,sixth nerve palsy,6th nerve palsy,lateral rectus palsy,vi nerve palsy,abducent nerve paralysis,sixth nerve paralysis,vith nerve paralysis,cranial nerve vi diseases,vith cranial nerve diseases,sixth cranial nerve palsy,vith nerve disorder,lateral rectus muscle denervation paresis,cranial nerve vi palsy	[[1,1,0,0,0,0,0,0,0,0,0,0,0,0],[1,0,0,0,0,0,0,1,0,0,1,0,0,0],[1,0,0,0,1,0,0,0,0,0,0,0,0,0],[0,0,0,0,0,0,0,0,0,0,1,0,0,0],[0,0,0,0,0,0,0,0,0,0,0,1,0,0],[1,1,0,0,0,0,0,1,0,0,0,0,1,0],[1,0,0,0,0,0,0,1,0,0,0,0,0,0],[1,0,0,0,0,0,0,0,0,0,0,0,0,0],[1,0,0,0,0,0,0,0,0,0,0,0,1,0],[1,0,0,0,0,0,0,0,0,0,0,0,1,0],[1,0,0,0,0,0,0,0,0,0,0,0,0,0],[1,0,0,0,0,0,0,0,0,0,0,0,0,0],[1,1,0,0,0,0,0,1,0,0,0,0,1,0],[0,1,0,0,0,0,0,1,0,0,0,0,1,0],[0,1,0,1,0,0,0,1,0,0,0,0,1,0],[1,1,0,0,0,0,0,1,0,0,0,0,1,0],[0,0,0,0,0,0,0,0,0,0,0,1,0,0],[0,0,0,0,0,0,0,1,0,0,0,1,0,0],[0,0,0,0,0,0,0,0,0,0,0,1,0,0],[0,0,0,0,0,0,0,0,0,0,0,0,1,0],[0,0,0,0,0,0,0,0,0,0,0,0,1,0],[0,0,0,0,0,0,0,1,0,0,0,0,1,0],[0,0,0,0,0,0,0,0,0,0,0,1,0,0],[1,0,0,0,0,0,0,0,0,0,0,0,0,0],[0,0,0,0,0,0,0,0,0,0,0,0,1,0]]C0570513	acetaminophen allergy,paracetamol allergy	[[1,0,0,0,0,0,0,1,0,0,0,0,0,0],[1,0,0,0,0,0,0,1,0,0,0,0,0,0]]C0570512	non opioid analgesic allergy	[[1,0,0,0,0,0,0,0,0,0,0,0,0,0]]C0004623	bacterial infections,bacterial infectious disease,disease caused by bacteria,bacterial disease,bacterial infection by site	[[0,1,0,0,0,0,0,1,1,0,0,1,1,0],[1,0,1,0,0,0,0,1,0,0,0,0,0,0],[1,0,0,0,0,0,0,1,0,0,0,0,0,0],[1,0,0,0,0,0,0,1,0,1,0,0,0,0],[1,0,0,0,0,0,0,0,0,0,0,0,0,0]]C0570510	analgesics and non steroidal anti inflammatory drug allergy	[[1,0,0,0,0,0,0,0,0,0,0,0,0,0]]C0570517	phenazocine allergy	[[1,0,0,0,0,0,0,0,0,0,0,0,0,0]]C0570516	pentazocine allergy	[[1,0,0,0,0,0,0,0,0,0,0,0,0,0]]C0570515	opioid analgesic allergy	[[1,0,0,0,0,0,0,0,0,0,0,0,0,0]]C0004626	bacterial pneumonia	[[1,1,1,0,0,0,0,1,0,0,0,0,1,0]]C1262234	gynecological infection	[[0,0,0,0,0,0,0,1,0,0,0,0,0,0]]C0238358	hypokalemic periodic paralysis,hypokalaemic periodic paralysis,familial hypokalemic periodic paralysis,familial hypokalaemic periodic paralysis,periodic paralysis i,primary hypokalemic periodic paralysis,hokpp,hokpp1,type 1 hypokalemic periodic paralysis	[[0,1,0,1,0,0,0,1,0,0,1,1,1,1],[1,0,0,0,0,0,0,1,0,0,0,0,0,0],[1,0,0,0,0,0,0,0,0,0,0,0,1,1],[1,0,0,0,0,0,0,0,0,0,0,0,0,0],[1,0,0,0,0,0,0,0,0,0,0,0,0,0],[0,0,0,0,0,0,0,0,0,0,0,0,1,0],[0,0,0,1,0,0,0,0,0,0,0,0,0,0],[0,0,0,1,0,0,0,0,0,0,0,0,0,0],[0,0,0,1,0,0,0,0,0,0,0,0,0,0]]C0238359	periodic sialadenosis,recurring salivary adenitis,periodic sialorrhea	[[0,0,0,0,0,0,0,0,0,0,1,0,0,0],[0,0,0,0,0,0,0,0,0,0,1,0,0,0],[0,0,0,0,0,0,0,0,0,0,1,0,0,0]]C0238356	perihepatitis	[[0,0,0,0,0,0,0,1,0,0,1,0,0,1]]C0238357	hyperkalemic periodic paralysis,hyperkalaemic periodic paralysis,hyperkalemic periodic paralyses,adynamia episodica hereditaria,gamstorp disease,familial hyperkalemic periodic paralysis,familial hyperkalaemic periodic paralysis,periodic paralysis ii,myotonic periodic paralysis,myotonic periodic paralyses,primary hyperkalemic periodic paralysis,hypp	[[0,1,0,1,0,0,0,1,0,0,1,0,1,1],[1,0,0,0,0,0,0,1,0,0,0,0,0,0],[0,1,0,0,0,0,0,0,0,0,0,0,0,0],[1,0,0,0,0,0,0,1,0,0,1,0,1,0],[1,0,0,0,0,0,0,0,0,0,1,0,0,0],[1,0,0,0,0,0,0,0,0,0,0,0,1,0],[1,0,0,0,0,0,0,0,0,0,0,0,0,0],[1,0,0,0,0,0,0,1,0,0,0,0,0,0],[1,1,0,0,0,0,0,0,0,0,0,0,1,0],[0,1,0,0,0,0,0,0,0,0,0,0,0,0],[0,0,0,0,0,0,0,0,0,0,0,0,1,0],[0,0,0,1,0,0,0,0,0,0,0,0,0,0]]C0238351	penile gangrene,gangrene of penis	[[1,0,0,0,0,0,0,0,0,0,0,0,0,0],[1,0,0,0,0,0,0,0,0,0,1,0,0,0]]C0238352	penile sarcoma,sarcoma penile cancer,sarcoma of penis	[[0,0,0,0,0,0,0,0,0,0,0,1,0,0],[0,0,0,0,0,0,0,0,0,0,1,0,0,0],[0,0,0,0,0,0,0,0,0,0,1,1,0,0]]C0238353	or diborane poisoning decaborane pentaborane	[[0,0,0,0,0,0,0,0,0,0,1,0,0,0]]C1302291	tail gland hyperplasia	[[1,0,0,0,0,0,0,0,0,0,0,0,0,0]]C1849472	pyruvate kinase deficiency of red cells,pk deficiency,pyruvate kinase deficiency of erythrocyte	[[0,0,0,1,0,0,0,0,0,0,0,0,0,0],[0,0,0,1,0,0,0,0,0,0,0,0,0,0],[0,0,0,1,0,0,0,0,0,0,0,0,0,0]]C1444008	adrenal cortical adenoma category	[[1,0,0,0,0,0,0,0,0,0,0,0,0,0]]C1849471	fatal neonatal radiculoneuropathy	[[0,0,0,1,0,0,0,0,0,0,0,0,0,0]]C1279141	embadomoniasis,retortamoniasis	[[1,0,0,0,0,0,0,0,0,0,0,0,0,0],[1,0,0,0,0,0,0,0,0,0,0,0,0,0]]C0752098	autosomal dominant parkinsonism	[[0,1,0,0,0,0,0,0,0,0,0,0,0,0]]C0752099	autosomal recessive juvenile parkinson disease,chromosome 6 linked autosomal recessive parkinsonism,autosomal recessive familial parkinson disease,autosomal recessive juvenile parkinsonism,autosomal recesssive juvenile parkinsonism	[[0,1,0,0,0,0,0,0,0,0,0,0,0,0],[0,1,0,0,0,0,0,0,0,0,0,0,0,0],[0,1,0,0,0,0,0,0,0,0,0,0,0,0],[0,1,0,0,0,0,0,0,0,0,0,0,0,0],[0,1,0,0,0,0,0,0,0,0,0,0,0,0]]C2919189	inflammation of pubic symphysis,osteitis of pubic symphysis	[[1,0,0,0,0,0,0,0,0,0,0,0,0,0],[1,0,0,0,0,0,0,0,0,0,0,0,0,0]]C0549189	anteroseptal accessory pathway	[[1,0,0,0,0,0,0,0,0,0,0,0,0,0]]C2750467	14 familial hypertrophic cardiomyopathy,cmh14	[[0,0,0,1,0,0,0,0,0,0,0,0,0,0],[0,0,0,1,0,0,0,0,0,0,0,0,0,0]]C2750466	1ee dilated cardiomyopathy,cmd1ee	[[0,0,0,1,0,0,0,0,0,0,0,0,0,0],[0,0,0,1,0,0,0,0,0,0,0,0,0,0]]C0345737	tumor of arytenoid,tumour of arytenoid	[[1,0,0,0,0,0,0,0,0,0,0,0,0,0],[1,0,0,0,0,0,0,0,0,0,0,0,0,0]]C0752097	autosomal dominant juvenile parkinson disease,autosomal dominant juvenile parkinsonism	[[0,1,0,0,0,0,0,0,0,0,0,0,0,0],[0,1,0,0,0,0,0,0,0,0,0,0,0,0]]C0018794	heart block,hb heart block,heart blockage	[[0,1,1,0,0,0,0,1,1,1,0,0,0,1],[1,0,0,0,0,0,0,0,0,0,0,0,0,0],[0,0,0,0,0,0,0,1,0,0,0,0,0,0]]C0403498	glycosuria during pregnancy delivered	[[1,0,0,0,0,0,0,0,0,0,0,0,0,0]]C0014236	endophthalmitis,endophthalmitides,ophthalmia,ophthalmitis,inflammatory disorder of the eye	[[0,1,0,0,0,0,0,1,0,0,0,1,0,0],[0,1,0,0,0,0,0,0,0,0,0,0,0,0],[0,1,0,0,0,0,0,1,0,0,0,0,1,0],[1,0,0,0,0,0,0,0,1,0,0,0,0,0],[1,0,0,0,0,0,0,0,0,0,0,0,0,0]]C0014234	endophlebitis	[[1,0,0,0,0,0,0,0,0,0,0,0,0,0]]C1859535	complementation group b type ii bare lymphocyte syndrome	[[0,0,0,1,0,0,0,0,0,0,0,0,0,0]]C1862170	brachydactyly with hypertension,htnb,bilginturan syndrome,brachydactyly type e with short stature and hypertension	[[0,1,0,1,0,0,0,0,0,0,0,0,0,0],[0,0,0,1,0,0,0,0,0,0,0,0,0,0],[0,1,0,1,0,0,0,0,0,0,0,0,0,0],[0,1,0,1,0,0,0,0,0,0,0,0,0,0]]C0014238	parasitic endophthalmitis	[[1,0,0,0,1,0,0,0,0,0,0,1,0,0]]C0018799	heart diseases,cardiac diseases,disorder of heart,morbus cordis,cardiac disorder,cardiopathy,syndrome heart disease,heart disease disorder	[[1,1,0,0,0,0,0,1,0,0,0,0,0,1],[0,1,0,0,0,0,0,1,0,0,0,0,0,0],[1,0,0,0,0,0,0,1,1,1,0,1,0,0],[1,0,0,0,0,0,0,1,0,0,0,0,0,1],[1,0,0,0,0,0,0,1,1,0,0,1,0,0],[1,0,0,0,0,0,0,1,0,0,0,0,0,0],[0,0,0,0,0,0,0,1,0,0,0,0,0,0],[0,0,0,0,0,0,0,1,0,0,0,0,0,0]]C1862171	and developmental delay metatarsus adductus brachmann de lange like facial changes with microcephaly	[[0,0,0,1,0,0,0,0,0,0,0,0,0,0]]C0272338	acquired factor xiii deficiency disease	[[1,0,0,0,0,0,0,0,0,0,0,0,0,0]]C0272339	prekallikrein deficiency,fletcher factor deficiency,fletcher trait,pkk deficiency	[[1,0,0,1,0,0,0,1,0,0,0,0,0,0],[1,0,0,1,0,0,0,1,0,0,0,0,0,0],[1,0,0,0,0,0,0,1,0,0,0,0,0,0],[0,0,0,1,0,0,0,0,0,0,0,0,0,0]]C0005283	beta thalassemia,beta thalassaemia,beta thalassemia syndrome,beta thalassaemia syndrome,beta sup sup thalassemia,beta sup sup thalassaemia	[[1,1,0,1,1,0,0,1,0,0,0,1,1,0],[1,0,0,0,0,0,0,1,0,0,0,0,0,0],[1,0,0,0,0,0,0,0,0,0,0,0,0,0],[1,0,0,0,0,0,0,0,0,0,0,0,0,0],[1,0,0,0,0,0,0,0,0,0,0,0,0,0],[1,0,0,0,0,0,0,0,0,0,0,0,0,0]]C0342105	metabolic and immunity disorders nutritional endocrine,and immunity disorders nutritional and metabolic diseases endocrine	[[1,0,0,0,0,0,0,0,0,0,0,0,0,0],[0,0,0,0,1,0,0,0,0,0,0,0,0,0]]C0272330	type ii factor xi deficiency	[[1,0,0,0,0,0,0,0,0,0,0,0,0,0]]C0272331	type iii factor xi deficiency	[[1,0,0,0,0,0,0,0,0,0,0,0,0,0]]C0272332	acquired factor xi deficiency disease	[[1,0,0,0,0,0,0,0,0,0,0,0,0,0]]C3164153	leiomyosarcoma of cardia of stomach	[[1,0,0,0,0,0,0,0,0,0,0,0,0,0]]C0272334	hereditary factor xii deficiency disease,hereditary hageman factor deficiency disease	[[1,0,0,0,0,0,0,0,0,0,0,0,0,0],[1,0,0,0,0,0,0,0,0,0,0,0,0,0]]C0272335	acquired factor xii deficiency disease,acquired hageman factor deficiency disease	[[1,0,0,0,0,0,0,0,0,0,0,0,0,0],[1,0,0,0,0,0,0,0,0,0,0,0,0,0]]C1861694	cervical vertebral bridge	[[0,0,0,1,0,0,0,0,0,0,0,0,0,0]]C1861695	cervical hypertrichosis with underlying kyphoscoliosis,with underlying kyphoscoliosis posterior cervical hypertrichosis	[[0,0,0,1,0,0,0,0,0,0,0,0,0,0],[0,0,0,1,0,0,0,0,0,0,0,0,0,0]]C1510479	neuralgic amyotrophy,amyotrophic neuralgia	[[0,1,0,0,1,0,0,0,0,0,0,0,0,0],[0,1,0,0,0,0,0,0,0,0,0,0,0,0]]C1861693	cervical vertebral dysplasia	[[0,0,0,1,0,0,0,0,0,0,0,0,0,0]]C1510473	stage iv hypopharyngeal cancer,metastatic hypopharyngeal cancer,stage iv hypopharynx cancer,metastatic hypopharynx cancer	[[0,0,0,0,0,1,0,0,0,0,0,0,0,0],[0,0,0,0,0,1,0,0,0,0,0,0,0,0],[0,0,0,0,0,1,0,0,0,0,0,0,0,0],[0,0,0,0,0,1,0,0,0,0,0,0,0,0]]C1510471	vitamin deficiency,hypovitaminosis,vitamin deficiency disorder	[[1,1,1,0,0,0,0,0,1,1,0,0,0,0],[1,0,0,0,0,0,0,0,1,1,0,0,0,0],[0,0,0,0,0,0,0,0,0,0,0,1,0,0]]C1997396	allergic arthritis of spine	[[1,0,0,0,0,0,0,0,0,0,0,0,0,0]]C1510475	diverticulosis,diverticular disease,enteric diverticulum,diverticula of intestine,diverticulum of intestine,dd diverticular disease,diverticular disorder	[[0,0,0,0,0,0,0,0,0,0,1,1,0,0],[0,0,1,0,0,0,0,0,0,0,0,0,0,0],[0,0,1,0,0,0,0,0,0,0,0,0,0,0],[0,0,0,0,1,0,0,0,0,0,0,0,0,0],[1,0,0,0,0,0,0,1,0,0,0,0,0,0],[1,0,0,0,0,0,0,0,0,0,0,0,0,0],[0,0,0,0,0,0,0,0,0,0,0,1,0,0]]C0871535	toxicomania	[[0,0,0,0,0,0,0,1,0,0,0,0,0,0]]C1853156	split hand foot malformation with long bone deficiency 2,shfld2	[[0,0,0,1,0,0,0,0,0,0,0,0,0,0],[0,0,0,1,0,0,0,0,0,0,0,0,0,0]]C1302782	benign neoplasm of blood vessel of lower leg	[[1,0,0,0,0,0,0,0,0,0,0,0,0,0]]C0752329	aids associated cerebral aneurysmal arteriopathy	[[0,1,0,0,0,0,0,0,0,0,0,0,0,0]]C0752327	antenatal myasthenia gravis	[[0,1,0,0,0,0,0,0,0,0,0,0,0,0]]C0752324	focal tonic seizures	[[0,1,0,0,0,0,0,0,0,0,0,0,0,0]]C0752325	hemimotor seizure disorder,hemimotor epilepsy	[[0,1,0,0,0,0,0,0,0,0,0,0,0,0],[0,1,0,0,0,0,0,0,0,0,0,0,0,0]]C0752322	sensory partial epilepsy,sensory focal seizure disorder,sensory partial seizure disorder,sensory seizure disorder	[[0,1,0,0,0,0,0,0,0,0,0,0,1,0],[0,0,0,0,0,0,0,0,0,0,0,0,1,0],[0,0,0,0,0,0,0,0,0,0,0,0,1,0],[0,1,0,0,0,0,0,0,0,0,0,0,1,0]]C0752323	focal clonic seizures	[[0,1,0,1,0,0,0,0,0,0,0,0,0,0]]C1859570	bardet biedl syndrome 12,bbs12	[[0,0,0,1,0,0,0,0,0,0,0,0,0,0],[0,0,0,1,0,0,0,0,0,0,0,0,0,0]]C0018425	gyrate atrophy,gyrate atrophy of choroid and retina,gyrate atrophy of the retina	[[1,1,0,0,0,0,0,1,0,0,0,0,0,0],[1,0,0,0,0,0,0,1,0,0,0,0,0,0],[0,0,0,0,0,0,0,1,0,0,0,0,0,0]]C0343603	germistan virus disease,germistan fever,germiston fever	[[1,0,0,0,0,0,0,0,0,0,0,0,0,0],[1,0,0,0,0,0,0,0,0,0,0,0,0,0],[1,0,0,0,0,0,0,0,0,0,0,0,0,0]]C0346234	regressed malignant testicular tumor,regressed malignant testicular tumour	[[1,0,0,0,0,0,0,0,0,0,0,0,0,0],[1,0,0,0,0,0,0,0,0,0,0,0,0,0]]C0334588	giant cell glioblastoma,monstrocellular sarcoma	[[1,1,0,0,0,0,0,0,0,1,0,1,0,0],[1,0,0,0,0,0,0,0,0,0,0,0,0,0]]C0343600	sepik fever	[[1,0,0,0,0,0,0,0,0,0,0,0,0,0]]C0343607	apeu virus disease,apeu fever	[[1,0,0,0,0,0,0,0,0,0,0,0,0,0],[1,0,0,0,0,0,0,0,0,0,0,0,0,0]]C0343606	mosquito borne serogroup c bunyavirus fever	[[1,0,0,0,0,0,0,0,0,0,0,0,0,0]]C0346233	benign tumor of corpus cavernosum,benign tumour of corpus cavernosum,benign neoplasm of corpus cavernosum	[[1,0,0,0,0,0,0,0,0,0,0,0,0,0],[1,0,0,0,0,0,0,0,0,0,0,0,0,0],[0,0,0,0,0,0,0,0,0,0,0,0,0,1]]C0343604	maguari fever	[[1,0,0,0,0,0,0,0,0,0,0,0,0,0]]C0334582	fibrillary astrocytoma,fibrous astrocytoma	[[1,1,0,0,0,0,0,1,0,0,0,1,0,0],[1,0,0,0,0,0,0,0,0,0,0,0,0,0]]C0334583	pilocytic astrocytoma,piloid astrocytoma	[[0,1,0,1,0,0,0,1,0,0,0,1,0,0],[1,0,0,0,0,0,0,0,0,0,0,0,0,0]]C0334580	protoplasmic astrocytoma	[[1,1,0,0,0,0,0,0,0,0,0,0,0,0]]C0334581	gemistocytic astrocytoma,gemistocytoma	[[1,1,0,0,0,0,0,0,0,0,0,1,0,0],[0,0,0,0,0,0,0,0,0,0,0,1,0,0]]C0334586	pleomorphic xanthoastrocytoma,m pleomorphic xanthoastrocytoma,pleomorphic xantho astrocytoma	[[1,0,0,0,0,0,0,0,0,0,0,1,0,0],[1,0,0,0,0,0,0,0,0,0,0,0,0,0],[0,0,0,0,0,0,0,0,0,0,0,1,0,0]]C0334587	astroblastoma	[[1,1,0,0,0,0,0,1,0,0,0,0,0,0]]C0334584	spongioblastoma	[[1,1,0,0,0,0,0,0,0,0,0,0,0,0]]C0345766	benign neoplasm of cervical esophagus,benign neoplasm of cervical oesophagus,benign tumor of cervical part of esophagus,benign tumour of cervical part of oesophagus	[[1,0,0,0,0,0,0,0,0,0,0,0,0,0],[1,0,0,0,0,0,0,0,0,0,0,0,0,0],[1,0,0,0,0,0,0,0,0,0,0,0,0,0],[1,0,0,0,0,0,0,0,0,0,0,0,0,0]]C0345767	neoplasm of thoracic esophagus,neoplasm of thoracic oesophagus,tumor of thoracic part of esophagus,tumour of thoracic part of oesophagus	[[1,0,0,0,0,0,0,0,0,0,0,0,0,0],[1,0,0,0,0,0,0,0,0,0,0,0,0,0],[1,0,0,0,0,0,0,0,0,0,0,0,0,0],[1,0,0,0,0,0,0,0,0,0,0,0,0,0]]C0345764	carcinoma of cervical part of esophagus,carcinoma of cervical part of oesophagus	[[1,0,0,0,0,0,0,0,0,0,0,0,0,0],[1,0,0,0,0,0,0,0,0,0,0,0,0,0]]C0345765	carcinoma in situ of cervical esophagus,carcinoma in situ of cervical oesophagus,carcinoma in situ of cervical part of esophagus,carcinoma in situ of cervical part of oesophagus	[[1,0,0,0,0,0,0,0,0,0,0,0,0,0],[1,0,0,0,0,0,0,0,0,0,0,0,0,0],[1,0,0,0,0,0,0,0,0,0,0,0,0,0],[1,0,0,0,0,0,0,0,0,0,0,0,0,0]]C0345762	neoplasm of cervical esophagus,neoplasm of cervical oesophagus,tumor of cervical part of esophagus,tumour of cervical part of oesophagus	[[1,0,0,0,0,0,0,0,0,0,0,0,0,0],[1,0,0,0,0,0,0,0,0,0,0,0,0,0],[1,0,0,0,0,0,0,0,0,0,0,0,0,0],[1,0,0,0,0,0,0,0,0,0,0,0,0,0]]C0345760	neoplasm of digestive organ,tumor of digestive organs,tumour of digestive organs	[[1,0,0,0,0,0,0,0,0,0,0,0,0,0],[1,0,0,0,0,0,0,0,0,0,0,0,0,0],[1,0,0,0,0,0,0,0,0,0,0,0,0,0]]C0345761	stomach and duodenum tumor of esophagus,stomach and duodenum tumour of oesophagus	[[1,0,0,0,0,0,0,0,0,0,0,0,0,0],[1,0,0,0,0,0,0,0,0,0,0,0,0,0]]C0521679	granulomatous chorioretinitis	[[1,0,0,0,0,0,0,0,0,0,0,0,0,0]]C0432428	whole chromosome trisomy meiotic nondisjunction	[[1,0,0,0,0,0,0,0,0,0,0,0,0,0]]C0345769	carcinoma of thoracic part of esophagus,carcinoma of thoracic part of oesophagus	[[1,0,0,0,0,0,0,0,0,0,0,0,0,0],[1,0,0,0,0,0,0,0,0,0,0,0,0,0]]C0432429	whole chromosome trisomy mitotic nondisjunction mosaicism	[[1,0,0,0,0,0,0,0,0,0,0,0,0,0]]C0001529	adiposis dolorosa,dercums disease,lipomatosis dolorosa,neurolipomatosis,dercum s disease	[[1,0,0,0,0,0,0,1,1,0,0,1,0,0],[1,1,0,0,0,0,0,1,0,0,1,1,0,0],[1,0,0,0,0,0,0,1,0,0,0,0,0,0],[1,0,0,0,0,0,0,0,0,0,0,0,0,0],[0,0,0,0,0,0,0,1,0,0,0,0,0,0]]C0002103	atopic rhinitis	[[0,0,0,0,0,0,1,0,0,0,1,1,0,0]]C2919721	disorder of articular cartilage of vertebral column	[[1,0,0,0,0,0,0,0,0,0,0,0,0,0]]C0002106	allergic pneumonia,extrinsic allergic pneumonia	[[1,0,0,0,0,0,0,0,0,0,1,0,0,1],[0,0,0,0,0,0,0,0,0,0,1,0,0,0]]C2919725	accumulation of bile in abdominal cavity,intraabdominal bile collection	[[1,0,0,0,0,0,0,0,0,0,0,0,0,0],[1,0,0,0,0,0,0,0,0,0,0,0,0,0]]C0854893	primary angiosarcoma,angiosarcoma nonmetastatic,non metastatic angiosarcoma,non metastatic hemangiosarcoma,primary hemangiosarcoma	[[0,0,0,0,0,0,0,0,0,0,0,1,0,0],[0,0,0,0,0,0,0,0,0,0,0,1,0,0],[0,0,0,0,0,0,0,0,0,0,0,1,0,0],[0,0,0,0,0,0,0,0,0,0,0,1,0,0],[0,0,0,0,0,0,0,0,0,0,0,1,0,0]]C0086649	mps iii c,acetyl coa heparan alpha d glucosaminide n acetyltransferase deficiency,heparan alpha glucosaminide acetyltransferase deficiency,mps iii c mucopolysaccharidosis,type c sanfilippo syndrome,acetyl coa alpha glucosaminide acetyltransferase deficiency,n acetyl transferase deficiency,mps iii c mucopolysaccharidosis iii c,mucopolysaccharidosis iii c,sanfilippo syndrome c,mucopolysaccharidosis type iiic,mps 3 c,mpsiiic mucopolysaccharidosis type iiic,mps3c,mps iiic,acetyl coa alpha glucosaminide n acetyltransferase deficiency,sanfilippo c,mucopolysaccharidosis type 3 c	[[0,0,0,0,0,0,0,0,0,0,0,1,0,0],[1,0,0,0,0,0,0,0,0,0,0,0,0,0],[1,0,0,0,0,0,0,0,0,0,0,0,0,0],[1,0,0,0,0,0,0,0,0,0,0,0,0,0],[1,0,0,0,0,0,0,0,0,0,0,0,0,0],[1,0,0,0,0,0,0,0,0,0,0,0,0,0],[1,0,0,0,0,0,0,0,0,0,0,0,0,0],[1,0,0,0,0,0,0,0,0,0,0,0,0,0],[1,0,0,0,0,0,0,0,0,0,0,0,0,0],[1,1,0,1,0,0,0,0,0,0,0,0,0,0],[0,0,0,1,0,0,0,0,0,0,0,1,0,0],[0,1,0,0,0,0,0,0,0,0,0,0,0,0],[1,0,0,0,0,0,0,0,0,0,0,0,0,0],[0,0,0,1,0,0,0,0,0,0,0,0,0,0],[0,0,0,1,0,0,0,0,0,0,0,0,0,0],[0,1,0,1,0,0,0,0,0,0,0,0,0,0],[0,0,0,0,0,0,0,0,0,0,0,1,0,0],[0,1,0,0,0,0,0,0,0,0,0,0,0,0]]C0086648	mps iii b,mucopolysaccharidosis type iiib,alpha n acetylglucosaminidase deficiency,mps iii b mucopolysaccharidosis,type b sanfilippo syndrome,n acetyl alpha d glucosaminidase deficiency,mps iii b mucopolysaccharidosis iii b,mucopolysaccharidosis iii b,sanfilippo syndrome b,mps 3 b,mpsiiib mucopolysaccharidosis type iiib,naglu deficiency,mps iiib,mps3b,sanfilippo b,mucopolysaccharidosis type 3 b	[[0,0,0,0,0,0,0,0,0,0,0,1,0,0],[0,0,0,1,0,0,0,0,0,0,0,1,0,0],[1,0,0,0,0,0,0,0,0,0,0,0,0,0],[1,0,0,0,0,0,0,0,0,0,0,0,0,0],[1,0,0,0,0,0,0,0,0,0,0,0,0,0],[1,1,0,1,0,0,0,0,0,0,0,0,0,0],[1,0,0,0,0,0,0,0,0,0,0,0,0,0],[1,0,0,0,0,0,0,0,0,0,0,0,0,0],[1,1,0,1,0,0,0,0,0,0,0,0,0,0],[0,1,0,0,0,0,0,0,0,0,0,0,0,0],[1,0,0,0,0,0,0,0,0,0,0,0,0,0],[0,1,0,1,0,0,0,0,0,0,0,0,0,0],[0,0,0,1,0,0,0,0,0,0,0,0,0,0],[0,0,0,1,0,0,0,0,0,0,0,0,0,0],[0,0,0,0,0,0,0,0,0,0,0,1,0,0],[0,1,0,0,0,0,0,0,0,0,0,0,0,0]]C0086647	mps iii a,mucopolysaccharidosis type iiia,mps iii a mucopolysaccharidosis,type a sanfilippo syndrome,heparan sulfate sulfatase deficiency,heparan sulphate sulphatase deficiency,n sulfoglucosamine sulfohydrolase deficiency,mps iii a mucopolysaccharidosis iii a,mucopolysaccharidosis iii a,sanfilippo syndrome a,heparan n sulfatase deficiency,heparan n sulphatase deficiency,heparan sulfamidase deficiency,heparan sulphamidase deficiency,mps 3 a,mpsiiia mucopolysaccharidosis type iiia,n sulphoglucosamine sulphohydrolase deficiency,mps3a,mps iiia,sulfamidase deficiency,mp iii,sanfilippo a,mucopolysaccharidosis type 3 a sanfilippo syndrome,mucopolysaccharidosis type 3 a	[[0,0,0,0,0,0,0,1,0,0,0,1,0,0],[1,0,0,1,0,0,0,0,0,0,0,1,0,0],[1,0,0,0,0,0,0,0,0,0,0,0,0,0],[1,0,0,0,0,0,0,1,0,0,0,0,0,0],[1,1,0,1,0,0,0,0,0,0,0,0,0,0],[1,0,0,0,0,0,0,0,0,0,0,0,0,0],[1,0,0,0,0,0,0,0,0,0,0,0,0,0],[1,0,0,0,0,0,0,0,0,0,0,0,0,0],[1,0,0,0,0,0,0,0,0,0,0,0,0,0],[1,1,0,1,0,0,0,0,0,0,0,0,0,0],[1,0,0,0,0,0,0,0,0,0,0,0,0,0],[1,0,0,0,0,0,0,0,0,0,0,0,0,0],[1,0,0,0,0,0,0,0,0,0,0,0,0,0],[1,0,0,0,0,0,0,0,0,0,0,0,0,0],[0,1,0,0,0,0,0,0,0,0,0,0,0,0],[1,0,0,0,0,0,0,0,0,0,0,0,0,0],[1,0,0,0,0,0,0,0,0,0,0,0,0,0],[0,0,0,1,0,0,0,0,0,0,0,0,0,0],[0,0,0,1,0,0,0,0,0,0,0,0,0,0],[0,0,0,1,0,0,0,0,0,0,0,0,0,0],[0,0,0,0,0,0,0,1,0,0,0,0,0,0],[0,0,0,0,0,0,0,0,0,0,0,1,0,0],[0,1,0,0,0,0,0,0,0,0,0,0,0,0],[0,1,0,0,0,0,0,0,0,0,0,0,0,0]]C1997530	hemangioendothelioma of liver,haemangioendothelioma of liver	[[1,0,0,0,0,0,0,0,0,0,0,0,0,0],[1,0,0,0,0,0,0,0,0,0,0,0,0,0]]C1641852	gelatinous droplike corneal dystrophy	[[1,0,0,0,0,0,0,0,0,0,0,0,0,0]]C0852952	optic nerve cupping	[[0,0,0,0,0,0,0,1,0,0,0,0,0,0]]C1264233	neoplasm of pararectal lymph nodes	[[1,0,0,0,0,0,0,0,0,0,0,0,0,0]]C1264232	neoplasm of paravaginal lymph nodes	[[1,0,0,0,0,0,0,0,0,0,0,0,0,0]]C1264231	neoplasm of parametrial lymph nodes	[[1,0,0,0,0,0,0,0,0,0,0,0,0,0]]C1264230	neoplasm of hypogastric lymph nodes	[[1,0,0,0,0,0,0,0,0,0,0,0,0,0]]C0003851	arteriosclerosis obliterans	[[0,1,0,0,0,0,0,1,0,0,1,0,1,0]]C0003850	arteriosclerosis,arterioscleroses,ascvd,cardiovascular arteriosclerosis,arteriosclerotic cardiovascular disease,arteriosclerotic vascular disease,vascular sclerosis,arterial sclerosis	[[0,0,1,0,0,0,0,0,0,1,0,0,0,0],[0,1,0,0,0,0,0,1,0,0,0,0,0,0],[0,0,0,0,0,0,0,1,0,0,0,0,0,1],[0,0,0,0,0,0,0,1,0,0,0,0,0,1],[0,0,0,0,0,0,0,0,0,0,0,1,0,1],[1,0,1,0,0,0,0,0,0,0,0,0,0,1],[1,0,0,0,0,0,0,0,0,0,0,1,0,0],[0,0,0,0,0,0,0,0,0,0,0,1,0,0]]C1863389	apert crouzon disease	[[0,1,0,1,0,0,0,0,0,0,0,0,0,0]]C0598642	periostitis hyperplastica	[[0,0,0,0,0,0,0,0,0,1,0,0,0,0]]C1282408	progressive locomotor ataxia	[[1,0,0,0,0,0,0,0,0,0,0,0,0,0]]C0543503	deficiency lysosomal acid phosphatase	[[0,0,0,0,0,0,0,0,0,0,1,0,0,0]]C1855305	ter haar syndrome,frank ter haar syndrome,formerly autosomal recessive melnick needles syndrome,fths,and developmental delay multiple skeletal anomalies megalocornea	[[0,1,0,1,0,0,0,0,0,0,0,0,0,0],[0,1,0,1,0,0,0,0,0,0,0,0,0,0],[0,0,0,1,0,0,0,0,0,0,0,0,0,0],[0,0,0,1,0,0,0,0,0,0,0,0,0,0],[0,1,0,0,0,0,0,0,0,0,0,0,0,0]]C1855304	autosomal recessive 1 mental retardation,mrt1	[[0,0,0,1,0,0,0,0,0,0,0,0,0,0],[0,0,0,1,0,0,0,0,0,0,0,0,0,0]]C1850155	torg winchester syndrome,winchester syndrome,mona,torg osteolysis syndrome,and arthropathy nodulosis multicentric osteolysis,osteolysis hereditary multicentric,nodulosis arthropathy osteolysis syndrome,nao syndrome,torg syndrome,al aqeel sewairi syndrome	[[0,0,0,1,0,0,0,0,0,0,0,0,0,0],[0,0,0,1,0,0,0,0,0,0,0,0,0,0],[0,0,0,1,0,0,0,0,0,0,0,0,0,0],[0,1,0,0,0,0,0,0,0,0,0,0,0,0],[0,0,0,1,0,0,0,0,0,0,0,0,0,0],[0,1,0,1,0,0,0,0,0,0,0,0,0,0],[0,0,0,1,0,0,0,0,0,0,0,0,0,0],[0,0,0,1,0,0,0,0,0,0,0,0,0,0],[0,0,0,1,0,0,0,0,0,0,0,0,0,0],[0,0,0,1,0,0,0,0,0,0,0,0,0,0]]C2349493	stage 5 retinopathy of prematurity	[[0,0,0,0,1,0,0,0,0,0,0,0,0,0]]C2349490	stage 2 retinopathy of prematurity	[[0,0,0,0,1,0,0,0,0,0,0,0,0,0]]C1517179	fibrosarcoma of the mouse intestinal tract	[[0,0,0,0,0,0,0,0,0,0,0,1,0,0]]C1517178	fibroma of the mouse skin	[[0,0,0,0,0,0,0,0,0,0,0,1,0,0]]C1517177	fibroma of the mouse intestinal tract	[[0,0,0,0,0,0,0,0,0,0,0,1,0,0]]C0274272	posttraumatic periosteoma,traumatic periosteitis ossificans	[[1,0,0,0,0,0,0,0,0,0,0,0,0,0],[1,0,0,0,0,0,0,0,0,0,0,0,0,0]]C2349491	stage 3 retinopathy of prematurity	[[0,0,0,0,1,0,0,0,0,0,0,0,0,0]]C1517173	fibroadenoma of the mouse mammary gland	[[0,0,0,0,0,0,0,0,0,0,0,1,0,0]]C0274275	late effect of open wound of extremities without tendon injury	[[1,0,0,0,0,0,0,0,0,0,0,0,0,0]]C0274274	late effect of sprain and or strain without tendon injury	[[1,0,0,0,0,0,0,0,0,0,0,0,0,0]]C2697566	head and neck fistula	[[0,0,0,0,0,0,0,0,0,0,0,1,0,0]]C1868576	with ataxia benign childhood paroxysmal tonic upgaze	[[0,0,0,1,0,0,0,0,0,0,0,0,0,0]]C1868577	patella aplasia hypoplasia,absent patella,ptlah,familial aplasia of the patella,familial absence of the patella	[[0,1,0,1,0,0,0,0,0,0,0,0,0,0],[0,1,0,0,0,0,0,0,0,0,0,0,0,0],[0,0,0,1,0,0,0,0,0,0,0,0,0,0],[0,1,0,0,0,0,0,0,0,0,0,0,0,0],[0,1,0,0,0,0,0,0,0,0,0,0,0,0]]C0268379	pseudocholinesterase deficiency,serum cholinesterase defect,suxamethonium paralysis,plasma cholinesterase deficiency,serum cholinesterase deficiency,deficiency of cholinesterase,cholinesterase 2 deficiency	[[0,1,0,1,0,0,0,1,0,0,0,0,0,0],[1,0,0,0,0,0,0,0,0,0,0,0,0,0],[1,0,0,0,0,0,0,0,0,0,0,0,0,0],[1,0,0,0,0,0,0,1,0,0,0,0,0,0],[1,0,0,0,0,0,0,0,0,0,0,0,0,0],[1,0,0,0,0,0,0,1,0,0,0,0,0,0],[0,1,0,0,0,0,0,0,0,0,0,0,0,0]]C0867359	infection and inflammatory reaction due to infusion pump	[[0,0,0,0,0,0,0,0,0,0,0,0,0,1]]C0867356	infection and inflammatory reaction due to coronary artery bypass graft	[[0,0,0,0,0,0,0,0,0,0,0,0,0,1]]C0867357	infection and inflammatory reaction due to arterial graft	[[0,0,0,0,0,0,0,0,0,0,0,0,0,1]]C0268375	autosomal dominant epidermolysis bullosa simplex,ebs 1	[[1,0,0,0,0,0,0,0,0,0,0,0,0,0],[1,0,0,0,0,0,0,0,0,0,0,0,0,0]]C0268374	adult junctional epidermolysis bullosa,non herlitz type epidermolysis bullosa junctionalis,non herlitz type junctional epidermolysis bullosa	[[1,0,0,0,0,0,0,0,0,0,0,0,0,0],[0,0,0,1,0,0,0,0,0,0,0,0,0,0],[0,0,0,1,0,0,0,0,0,0,0,0,0,0]]C0268373	congenital junctional epidermolysis bullosa pyloric atresia syndrome,epidermolysis bullosa letalis with pyloric atresia	[[1,0,0,0,0,0,0,0,0,0,0,0,0,0],[1,0,0,0,0,0,0,0,0,0,0,0,0,0]]C0867353	leads infection and inflammatory reaction due to cardiac pacemaker or defibrillator electrodes	[[0,0,0,0,0,0,0,0,0,0,0,0,0,1]]C0268371	dominant dystrophic epidermolysis bullosa with absence of skin,bart type epidermolysis bullosa dystrophica,epidermolysis bullosa with congenital localized absence of skin and deformity of nails	[[1,0,0,0,0,0,0,0,0,0,0,0,0,0],[1,0,0,1,0,0,0,0,0,0,0,0,0,0],[0,0,0,1,0,0,0,0,0,0,0,0,0,0]]C0269420	spontaneous abortion with renal tubular necrosis	[[1,0,0,0,0,0,0,0,0,0,0,0,0,0]]C0269421	spontaneous abortion with uremia,spontaneous abortion with uraemia	[[1,0,0,0,0,0,0,0,0,0,0,0,0,0],[1,0,0,0,0,0,0,0,0,0,0,0,0,0]]C0269422	spontaneous abortion with electrolyte imbalance	[[1,0,0,0,0,0,0,0,0,0,0,0,0,0]]C0269423	spontaneous abortion with postoperative shock	[[1,0,0,0,0,0,0,0,0,0,0,0,0,0]]C0269424	spontaneous abortion with septic shock	[[1,0,0,0,0,0,0,0,0,0,0,0,0,0]]C1513524	mouse cardiac rhabdomyosarcoma	[[0,0,0,0,0,0,0,0,0,0,0,1,0,0]]C0269426	spontaneous abortion with amniotic fluid embolism	[[1,0,0,0,0,0,0,0,0,0,0,0,0,0]]C0269427	spontaneous abortion with blood clot embolism	[[1,0,0,0,0,0,0,0,0,0,0,0,0,0]]C0269428	spontaneous abortion with fat embolism	[[1,0,0,0,0,0,0,0,0,0,0,0,0,0]]C0269429	spontaneous abortion with pulmonary embolism	[[1,0,0,0,0,0,0,0,0,0,0,0,0,0]]C2751842	autosomal recessive parkinson disease 14,park14,adult onset dystonia parkinsonism	[[0,0,0,1,0,0,0,0,0,0,0,0,0,0],[0,0,0,1,0,0,0,0,0,0,0,0,0,0],[0,0,0,1,0,0,0,0,0,0,0,0,0,0]]C0344497	hypoplasia of eye muscle	[[1,0,0,0,0,0,0,0,0,0,0,0,0,0]]C0432459	partial x deletion karyotype turners phenotype	[[1,0,0,0,0,0,0,0,0,0,0,0,0,0]]C0684445	carcinoma in situ of skin of forearm	[[1,0,0,0,0,0,0,0,0,0,0,0,0,0]]C0432453	balanced sex autosomal rearrangement in abnormal individual	[[1,0,0,0,0,0,0,0,0,0,0,0,0,0]]C0432452	balanced autosomal rearrangement in abnormal individual	[[1,0,0,0,0,0,0,0,0,0,0,0,0,0]]C0432450	balanced translocation and insertion in normal individual	[[1,0,0,0,0,0,0,0,0,0,0,0,0,0]]C0432456	sex chromosome abnormality female phenotype	[[1,0,0,0,0,0,0,0,0,0,0,0,0,0]]C0344499	hypoplasia of eyelid	[[1,0,0,0,0,0,0,0,0,0,0,0,0,0]]C0432454	individual with marker heterochromatin	[[1,0,0,0,0,0,0,0,0,0,0,0,0,0]]C0271738	secondary hypocortisolism,adrenal suppression,supression adrenal,secondary adrenocortical insufficiency	[[1,0,0,0,0,0,0,0,0,0,0,0,0,0],[1,0,0,0,0,0,0,1,1,0,0,0,0,0],[0,0,0,0,0,0,0,1,1,0,0,0,0,0],[1,0,0,0,0,0,0,0,0,0,0,0,0,0]]C1835980	parietal foramina 3,pfm3	[[0,0,0,1,0,0,0,0,0,0,0,0,0,0],[0,0,0,1,0,0,0,0,0,0,0,0,0,0]]C0271731	virilizing syndrome of adrenal origin,virilising syndrome of adrenal origin	[[1,0,0,0,0,0,0,0,0,0,0,0,0,0],[1,0,0,0,0,0,0,0,0,0,0,0,0,0]]C0271730	feminizing syndrome of adrenal origin,feminising syndrome of adrenal origin	[[1,0,0,0,0,0,0,0,0,0,0,0,0,0],[1,0,0,0,0,0,0,0,0,0,0,0,0,0]]C0271733	acquired benign adrenal androgenic overactivity	[[0,0,0,0,0,0,0,0,0,0,0,0,0,1]]C0271732	achard thiers syndrome,diabetic bearded woman syndrome,adenoma associated virilism of older women,diabetes in bearded women,bearded female with diabetes	[[1,0,0,0,0,0,0,0,0,0,0,0,0,0],[0,1,0,0,0,0,0,0,0,0,0,0,0,0],[0,1,0,0,0,0,0,0,0,0,0,0,0,0],[0,1,0,0,0,0,0,0,0,0,0,0,0,0],[0,1,0,0,0,0,0,0,0,0,0,0,0,0]]C0271737	addisons disease due to autoimmunity,autoimmune adrenal atrophy,adrenal atrophy,autoimmune adrenalitis,adrenalitis	[[1,0,0,0,0,0,0,1,0,0,0,0,0,0],[1,0,0,0,0,0,0,0,0,0,0,0,0,0],[0,0,0,0,0,0,0,0,0,0,0,0,0,1],[1,0,0,0,0,0,0,1,0,0,0,0,0,0],[0,0,0,0,0,0,0,1,0,0,0,0,0,0]]C2119283	uncontrolled type ii diabetes mellitus with peripheral neuropathy	[[0,0,0,0,0,0,1,0,0,0,0,0,0,0]]C0342268	protein deficient diabetes mellitus,malnutrition related diabetes mellitus protein deficient	[[1,0,0,0,0,0,0,0,0,0,0,0,0,0],[1,0,0,0,0,0,0,0,0,0,0,0,0,0]]C0342269	steroid induced diabetes,steroid induced diabetes mellitus	[[1,0,0,0,0,0,0,0,0,0,0,0,0,0],[0,0,1,0,0,0,0,0,0,0,0,0,0,0]]C1306143	primary malignant neoplasm of long bone of lower limb	[[1,0,0,0,0,0,0,0,0,0,0,0,0,0]]C0571111	anionic surfactant allergy	[[1,0,0,0,0,0,0,0,0,0,0,0,0,0]]C0456111	neonatal pyoderma	[[1,0,0,0,0,0,0,0,0,0,0,0,0,0]]C1858763	1g dilated cardiomyopathy,cmd1g	[[0,0,0,1,0,0,0,0,0,0,0,0,0,0],[0,0,0,1,0,0,0,0,0,0,0,0,0,0]]C0342266	insulin treated non insulin dependent diabetes mellitus,niddm insulin treated non insulin dependent diabetes mellitus,insulin treated type ii diabetes mellitus	[[1,0,0,0,0,0,0,0,0,0,0,0,0,0],[1,0,0,0,0,0,0,0,0,0,0,0,0,0],[1,0,0,0,0,0,0,0,0,0,0,0,0,0]]C0342267	malnutrition related diabetes mellitus fibrocalculous	[[1,0,0,0,0,0,0,0,0,0,0,0,0,0]]C0409958	osteoarthritis of proximal interphalangeal joint,degenerative joint disease of proximal interphalangeal joint	[[1,0,0,0,0,0,0,0,0,0,0,0,0,0],[1,0,0,0,0,0,0,0,0,0,0,0,0,0]]C0409959	knee osteoarthritis,knee osteoarthritides,oa osteoarthritis of knee,knee osteoarthrosis,gonarthrosis,degenerative joint disease of knee,knee djd,os6,osteoarthritis susceptibility 6	[[0,1,0,1,0,0,0,1,0,0,0,0,0,0],[0,1,0,0,0,0,0,0,0,0,0,0,0,0],[1,0,0,0,0,0,0,0,0,0,0,0,0,0],[0,0,0,0,0,0,0,1,0,0,0,0,0,0],[1,0,0,0,0,0,0,0,0,0,0,0,0,0],[1,0,0,0,0,0,0,0,0,0,0,0,0,0],[1,0,0,0,0,0,0,0,0,0,0,0,0,0],[0,0,0,1,0,0,0,0,0,0,0,0,0,0],[0,0,0,1,0,0,0,0,0,0,0,0,0,0]]C0409954	osteoarthritis of elbow,oa osteoarthritis of elbow,degenerative joint disease of elbow	[[1,0,0,0,0,0,0,0,0,0,0,0,0,0],[1,0,0,0,0,0,0,0,0,0,0,0,0,0],[1,0,0,0,0,0,0,0,0,0,0,0,0,0]]C0409955	osteoarthritis of wrist,oa osteoarthritis of wrist,degenerative joint disease of wrist	[[1,0,0,0,0,0,0,1,0,0,0,0,0,0],[1,0,0,0,0,0,0,0,0,0,0,0,0,0],[1,0,0,0,0,0,0,0,0,0,0,0,0,0]]C0409956	osteoarthrosis of the carpometacarpal joint of the thumb,osteoarthritis of first carpometacarpal joint	[[1,0,0,0,0,0,0,0,0,0,0,0,0,0],[1,0,0,0,0,0,0,0,0,0,0,0,0,0]]C0409957	osteoarthritis of distal interphalangeal joint,hoa,os2,hand osteoarthritis,degenerative joint disease of distal interphalangeal joint,oadip,dipoa,osteoarthritis susceptibility 2	[[1,0,0,1,0,0,0,0,0,0,0,0,0,0],[0,0,0,1,0,0,0,0,0,0,0,0,0,0],[0,0,0,1,0,0,0,0,0,0,0,0,0,0],[0,0,0,1,0,0,0,0,0,0,0,0,0,0],[1,0,0,0,0,0,0,0,0,0,0,0,0,0],[0,0,0,1,0,0,0,0,0,0,0,0,0,0],[0,0,0,1,0,0,0,0,0,0,0,0,0,0],[0,0,0,1,0,0,0,0,0,0,0,0,0,0]]C1336400	stage ivc nasopharyngeal keratinizing squamous cell carcinoma,stage ivc nasopharyngeal keratinizing squamous cell carcinoma ajcc v7	[[0,0,0,0,0,0,0,0,0,0,0,1,0,0],[0,0,0,0,0,0,0,0,0,0,0,1,0,0]]C1336401	stage ivc oral cavity adenoid cystic carcinoma,stage ivc mouth adenoid cystic carcinoma	[[0,0,0,0,0,0,0,0,0,0,0,1,0,0],[0,0,0,0,0,0,0,0,0,0,0,1,0,0]]C0409952	idiopathic osteoarthritis,generalized osteoarthritis,primary osteoarthritis	[[1,0,0,0,0,0,0,0,0,0,0,0,0,0],[0,0,0,0,0,0,0,1,0,0,0,0,0,0],[0,0,0,0,0,0,0,1,0,0,0,0,0,0]]C0409953	osteoarthritis of spinal facet joint,degenerative arthropathy of spinal facet joint,degenerative joint disease of spinal facet joint	[[1,0,0,0,0,0,0,0,0,0,0,0,0,0],[1,0,0,0,0,0,0,0,0,0,0,0,0,0],[1,0,0,0,0,0,0,0,0,0,0,0,0,0]]C0085605	liver failure,liver function failure,hepatic failure,liver decompensation	[[0,1,0,1,0,0,0,1,1,0,0,0,0,0],[1,0,0,0,0,0,0,1,0,0,0,0,0,0],[0,0,1,1,0,0,0,1,1,0,0,0,0,0],[1,0,0,0,0,0,0,1,0,0,0,0,0,0]]C1443283	lower eyelid tarsal ectropion	[[1,0,0,0,0,0,0,0,0,0,0,0,0,0]]C1699141	intestinal stoma prolapse	[[0,0,0,0,0,0,0,0,0,0,0,1,0,0]]C1699140	large intestinal mucositis	[[0,0,0,0,0,0,0,0,0,0,0,1,0,0]]C2959981	infection of peritoneum due to chlamydia trachomatis	[[1,0,0,0,0,0,0,0,0,0,0,0,0,0]]C0268599	methylcrotonic aciduria	[[1,0,0,0,0,0,0,0,0,0,0,0,0,0]]C0268598	type 2 autosomal recessive glutaric aciduria,ga ii b	[[1,0,0,0,0,0,0,0,0,0,0,0,0,0],[1,0,0,0,0,0,0,0,0,0,0,0,0,0]]C0341510	pericecal abscess,pericaecal abscess	[[1,0,0,0,0,0,0,0,0,0,0,0,0,0],[1,0,0,0,0,0,0,0,0,0,0,0,0,0]]C0341513	chronic mesenteric lymphadenitis	[[0,0,0,0,0,0,0,0,0,0,0,0,0,1]]C0268593	non amino organic acidemia and or aciduria,non amino organic acidaemia and or aciduria	[[1,0,0,0,0,0,0,0,0,0,0,0,0,0],[1,0,0,0,0,0,0,0,0,0,0,0,0,0]]C0268591	adenosylcobalamin and methylcobalamin synthesis defect,inherited methylmalonic acidemia and homocystinuria,inherited methylmalonic acidaemia and homocystinuria,combined methylmalonic acidemia and homocystinuria due to defects in adenosylcobalamin and methylcobalamin synthesis,combined methylmalonic acidaemia and homocystinuria due to defects in adenosylcobalamin and methylcobalamin synthesis,cobalamin locus f variant,cblf cobalamin locus f variant,cblf methylmalonic acidemia and homocystinuria,cblf methylmalonic acidaemia and homocystinuria	[[1,0,0,0,0,0,0,0,0,0,0,0,0,0],[1,0,0,0,0,0,0,0,0,0,0,0,0,0],[1,0,0,0,0,0,0,0,0,0,0,0,0,0],[1,0,0,0,0,0,0,0,0,0,0,0,0,0],[1,0,0,0,0,0,0,0,0,0,0,0,0,0],[1,0,0,0,0,0,0,0,0,0,0,0,0,0],[1,0,0,0,0,0,0,0,0,0,0,0,0,0],[1,0,0,0,0,0,0,0,0,0,0,0,0,0],[1,0,0,0,0,0,0,0,0,0,0,0,0,0]]C0268597	type 2 x linked glutaric aciduria,ga ii a	[[1,0,0,0,0,0,0,0,0,0,0,0,0,0],[1,0,0,0,0,0,0,0,0,0,0,0,0,0]]C0268596	multiple acyl coenzyme a dehydrogenase deficiency,type 2 glutaric acidemia,type 2 glutaric acidaemia,glutaric aciduria type 2,acad,ethylmalonic adipicaciduria,ema,ga ii,glutaric aciduria type ii,mad multiple acyl coa dehydrogenase deficiency,multiple acyl coa dehydrogenase deficiency,madd,glutaric aciduria ii,electron transfer flavoprotein deficiency,ethylmalonic adipic aciduria	[[0,1,0,0,0,0,0,0,0,0,0,0,0,0],[1,0,0,0,0,0,0,1,0,0,0,0,0,0],[1,0,0,0,0,0,0,0,0,0,0,0,0,0],[1,0,0,0,0,0,0,0,0,0,0,0,1,0],[1,0,0,0,0,0,0,0,0,0,0,0,0,0],[1,1,0,1,0,0,0,0,0,0,0,0,1,0],[0,0,0,1,0,0,0,1,0,0,0,0,0,0],[0,0,0,1,0,0,0,0,0,0,0,0,0,0],[1,0,0,0,0,0,0,0,0,0,0,0,1,0],[1,0,0,0,0,0,0,0,0,0,0,0,0,0],[1,1,0,1,0,0,0,0,0,0,0,1,0,0],[0,1,0,1,0,0,0,0,0,0,0,0,1,0],[0,0,0,1,0,0,0,0,0,0,0,0,1,0],[0,0,0,0,0,0,0,0,0,0,0,0,1,0],[0,1,0,0,0,0,0,0,0,0,0,0,1,0]]C0268595	type 1 glutaric aciduria,type 1 glutaric acidemia,type 1 glutaric acidaemia,ga i,glutaric aciduria type i,glutaryl coa dehydrogenase deficiency,glutaric acidemia i,glutaric aciduria i,glutaric aciduria 1,glutaric acidemia 1	[[1,0,0,0,0,0,0,1,0,0,0,0,0,0],[1,0,0,0,0,0,0,0,0,0,0,0,0,0],[1,0,0,0,0,0,0,0,0,0,0,0,0,0],[0,0,0,1,0,0,0,0,0,0,0,0,0,0],[1,0,0,0,0,0,0,1,0,0,0,0,0,0],[0,0,0,1,0,0,0,0,0,0,0,0,0,0],[0,0,0,1,0,0,0,0,0,0,0,0,0,0],[0,0,0,1,0,0,0,0,0,0,0,0,0,0],[0,1,0,0,0,0,0,0,0,0,0,0,0,0],[0,1,0,0,0,0,0,0,0,0,0,0,0,0]]C0268594	glutaric aciduria	[[1,0,0,0,0,0,0,1,0,0,0,0,0,0]]C0694550	recurrent pneumonia	[[0,0,0,1,0,0,0,1,0,0,0,0,0,0]]C1852721	colonic varices without portal hypertension	[[0,0,0,1,0,0,0,0,0,0,0,0,0,0]]C0030167	pachymeningitis,pachymeningitides	[[1,0,0,0,0,0,0,1,0,0,0,0,0,0],[0,1,0,0,0,0,0,1,0,0,0,0,0,0]]C0521752	retrobulbar optic nerve atrophy	[[1,0,0,0,0,0,0,0,0,0,0,0,0,0]]C0266624	branchial cleft sinus,branchial vestiges,branchial sinus	[[1,0,0,0,0,0,0,1,0,0,0,0,0,0],[0,0,0,0,0,0,0,1,0,0,0,0,0,0],[0,0,0,0,0,0,0,0,0,0,0,0,0,1]]C1846838	myasthenia gravis with thymus hyperplasia,myas1	[[0,0,0,1,0,0,0,0,0,0,0,0,0,0],[0,0,0,1,0,0,0,0,0,0,0,0,0,0]]C0267570	rectal cellulitis	[[0,0,0,0,0,0,0,0,0,0,0,0,0,1]]C0267571	transsphincteric anal fistula,ischiorectal fistula	[[1,0,0,0,0,0,0,0,0,0,0,0,0,0],[0,0,0,0,0,0,0,0,0,0,0,0,0,1]]C0267572	nonspecific ulcerative proctitis	[[1,0,0,0,0,0,0,0,0,0,0,0,0,0]]C0267573	anal polyp,polyp of the anus	[[1,0,1,0,0,0,0,1,0,0,0,1,0,0],[0,0,0,0,0,0,0,0,0,0,0,1,0,0]]C0267574	hypertrophied anal papilla	[[1,0,0,0,0,0,0,1,0,0,0,0,0,0]]C0267575	granuloma of rectum	[[0,0,0,0,0,0,0,0,0,0,0,0,0,1]]C0267576	incomplete rectal prolapse,partial rectal prolapse	[[1,0,0,0,0,0,0,0,0,0,0,0,0,0],[1,0,0,0,0,0,0,0,0,0,0,0,0,0]]C0267577	complete rectal prolapse with displacement of anal sphincter	[[1,0,0,0,0,0,0,0,0,0,0,0,0,0]]C0267578	complete rectal prolapse with no displacement of anal muscles	[[1,0,0,0,0,0,0,0,0,0,0,0,0,0]]C0267579	internal complete rectal prolapse with intussusception of rectosigmoid	[[1,0,0,0,0,0,0,0,0,0,0,0,0,0]]C0542367	uremic polyneuropathy,uraemic polyneuropathy	[[1,0,0,0,0,0,0,0,0,0,0,0,0,0],[1,0,0,0,0,0,0,0,0,0,0,0,0,0]]C0037199	sinusitis,sinusitides,sinus infection	[[0,0,1,0,0,0,0,0,0,1,0,0,0,1],[0,1,0,0,0,0,0,0,0,0,0,0,0,0],[0,0,0,0,0,0,0,1,0,0,0,1,0,0]]C0037198	intracranial sinus thrombosis,intracranial sinus thromboses,cranial sinus thrombosis,cranial sinus thromboses,sinus thrombosis,sinus thromboses,cranial venous sinus thrombosis	[[0,1,0,0,0,0,0,1,0,0,0,0,1,0],[0,1,0,0,0,0,0,0,0,0,0,0,0,0],[0,1,0,0,0,0,0,0,0,0,0,0,1,0],[0,1,0,0,0,0,0,0,0,0,0,0,0,0],[0,1,0,0,0,0,0,1,0,0,0,0,1,0],[0,1,0,0,0,0,0,0,0,0,0,0,0,0],[0,0,0,0,0,0,0,0,0,0,0,0,1,0]]C0549590	taste disorders	[[0,0,0,0,0,0,0,0,1,0,0,0,0,0]]C1835360	lipoprotein types lp system lp a hyperlipoproteinemia	[[0,0,0,1,0,0,0,0,0,0,0,0,0,0]]C1835362	congenital lipoprotein a deficiency,congenital lp a deficiency	[[0,0,0,1,0,0,0,0,0,0,0,0,0,0],[0,0,0,1,0,0,0,0,0,0,0,0,0,0]]C2677102	chromosome 10q23 deletion syndrome	[[0,0,0,1,0,0,0,0,0,0,0,0,0,0]]C2677103	juvenile polyposis of infancy	[[0,0,0,1,0,0,0,0,0,0,0,0,0,0]]C2677100	inflammatory bowel disease 14,ibd14	[[0,0,0,1,0,0,0,0,0,0,0,0,0,0],[0,0,0,1,0,0,0,0,0,0,0,0,0,0]]C2677101	inflammatory bowel disease 13,ibd13	[[0,0,0,1,0,0,0,0,0,0,0,0,0,0],[0,0,0,1,0,0,0,0,0,0,0,0,0,0]]C2677106	7 familial atrial fibrillation,atfb7	[[0,0,0,1,0,0,0,0,0,0,0,0,0,0],[0,0,0,1,0,0,0,0,0,0,0,0,0,0]]C1368374	primary malignant neoplasm of nervous system	[[1,0,0,0,0,0,0,0,0,0,0,0,0,0]]C2677104	infantile juvenile polyposis	[[0,0,0,1,0,0,0,0,0,0,0,0,0,0]]C2677105	inflammatory bowel disease 12,ibd12	[[0,0,0,1,0,0,0,0,0,0,0,0,0,0],[0,0,0,1,0,0,0,0,0,0,0,0,0,0]]C0521848	drug interaction potentiation	[[1,0,0,0,0,0,0,0,0,0,0,0,0,0]]C0556023	neonatal skin infection	[[1,0,0,0,0,0,0,0,0,0,0,0,0,0]]C1827983	refractory migraine variants,intractable migraine variants	[[1,0,0,0,0,0,0,0,0,0,0,0,0,0],[1,0,0,0,0,0,0,0,0,0,0,0,0,0]]C0521845	drug action decreased,drug effect decreased,drug effect deminished	[[1,0,0,0,0,0,0,0,0,0,0,0,0,0],[1,0,0,0,0,0,0,0,0,0,0,0,0,0],[0,0,0,0,0,0,0,0,1,0,0,0,0,0]]C0000774	abnormality of secretion of gastrin	[[0,0,0,0,1,0,0,0,0,0,0,0,0,0]]C0684444	neoplasm of uncertain behavior of skin of forearm,neoplasm of uncertain behaviour of skin of forearm	[[1,0,0,0,0,0,0,0,0,0,0,0,0,0],[1,0,0,0,0,0,0,0,0,0,0,0,0,0]]C1827987	lacrimal canaliculitis due to actinomyces israelii	[[1,0,0,0,0,0,0,0,0,0,0,0,0,0]]C0521841	atrophic thrush,atrophic candidiasis	[[1,0,0,0,0,0,0,0,0,0,0,0,0,0],[1,0,0,0,0,0,0,0,0,0,0,0,0,0]]C0521840	pseudomembranous thrush,pseudomembranous candidiasis	[[1,0,0,0,0,0,0,0,0,0,0,0,0,0],[0,0,0,0,0,0,0,1,0,0,0,0,0,0]]C1275464	angiomatous nevus impairing vision,angiomatous naevus impairing vision,strawberry nevus impairing vision,strawberry naevus impairing vision	[[1,0,0,0,0,0,0,0,0,0,0,0,0,0],[1,0,0,0,0,0,0,0,0,0,0,0,0,0],[1,0,0,0,0,0,0,0,0,0,0,0,0,0],[1,0,0,0,0,0,0,0,0,0,0,0,0,0]]C0266191	congenital obstruction of large intestine,congenital or infantile obstruction of large intestine	[[1,0,0,0,0,0,0,0,0,0,0,0,0,0],[0,0,0,0,0,0,0,0,0,0,0,0,0,1]]C0266190	congenital atresia of colon,colonic atresia	[[1,0,0,0,0,0,0,0,0,0,0,0,0,0],[1,0,0,1,0,0,0,0,0,0,0,0,0,1]]C0280096	adenocarcinoma cup	[[0,0,0,0,0,1,0,0,0,0,0,0,0,0]]C0280097	squamous cell cup	[[0,0,0,0,0,1,0,0,0,0,0,0,0,0]]C0280098	undifferentiated carcinoma cup	[[0,0,0,0,0,1,0,0,0,0,0,0,0,0]]C0280099	adult solid tumor,adult solid neoplasm	[[0,0,0,0,0,1,0,0,0,0,0,1,0,0],[0,0,0,0,0,0,0,0,0,0,0,1,0,0]]C0266198	dolichocolon	[[0,0,0,0,0,0,0,0,0,0,0,0,0,1]]C0277199	infection by gnathostoma nipponicum	[[1,0,0,0,0,0,0,0,0,0,0,0,0,0]]C0277198	infection by gnathostoma dolorosi	[[1,0,0,0,0,0,0,0,0,0,0,0,0,0]]C0425755	penetration without ejaculation	[[1,0,0,0,0,0,0,0,0,0,0,0,0,0]]C0277193	infection by spirocerca arctica	[[1,0,0,0,0,0,0,0,0,0,0,0,0,0]]C0277192	infection by spirocerca,spirocercosis	[[1,0,0,0,0,0,0,0,0,0,0,0,0,0],[1,0,0,0,0,0,0,0,0,0,0,0,0,0]]C0277191	infection by thelazia callipaeda	[[1,0,0,0,0,0,0,0,0,0,0,0,0,0]]C0277190	infection by thelazia californiensis	[[1,0,0,0,0,0,0,0,0,0,0,0,0,0]]C0277197	infection by simondsia,simondsiosis	[[1,0,0,0,0,0,0,0,0,0,0,0,0,0],[1,0,0,0,0,0,0,0,0,0,0,0,0,0]]C0277196	infection by oxyspirura,oxyspirurosis	[[1,0,0,0,0,0,0,0,0,0,0,0,0,0],[1,0,0,0,0,0,0,0,0,0,0,0,0,0]]C0277195	infection by spirocerca sanguinolenta	[[1,0,0,0,0,0,0,0,0,0,0,0,0,0]]C0277194	infection by spirocerca lupi	[[1,0,0,0,0,0,0,0,0,0,0,0,0,0]]C0456116	neonatal inclusion body conjunctivitis,neonatal inclusion blennorrhea,neonatal inclusion blennorrhoea	[[1,0,0,0,0,0,0,0,0,0,0,0,0,0],[1,0,0,0,0,0,0,0,0,0,0,0,0,0],[1,0,0,0,0,0,0,0,0,0,0,0,0,0]]C1849696	with skeletal anomalies female pseudohermaphroditism	[[0,0,0,1,0,0,0,0,0,0,0,0,0,0]]C1849694	pseudoinflammatory recessive form fundus dystrophy,finnish type pfd,lavia type pfd	[[0,1,0,1,0,0,0,0,0,0,0,0,0,0],[0,0,0,1,0,0,0,0,0,0,0,0,0,0],[0,0,0,1,0,0,0,0,0,0,0,0,0,0]]C1299931	branchitis	[[1,0,0,0,0,0,0,0,0,0,0,0,0,0]]C0276820	infection by hartmannella,hartmannellosis	[[1,0,0,0,0,0,0,0,0,0,0,0,0,0],[1,0,0,0,0,0,0,0,0,0,0,0,0,0]]C0276821	disease due to vahlkampfiidae	[[1,0,0,0,0,0,0,0,0,0,0,0,0,0]]C0276822	infection by naegleria,naegleriosis,naegleriasis	[[1,0,0,0,0,0,0,1,0,0,0,0,0,0],[1,0,0,0,0,0,0,0,0,0,0,0,0,0],[0,0,0,0,0,0,0,1,0,0,0,0,0,0]]C0276823	infection by naegleria gruberi	[[1,0,0,0,0,0,0,0,0,0,0,0,0,0]]C0276824	meningoencephalitis due to naegleria	[[0,0,0,0,0,0,0,0,0,0,0,0,0,1]]C0276825	infection by vahlkampfia,vahlkampfiosis	[[1,0,0,0,0,0,0,0,0,0,0,0,0,0],[1,0,0,0,0,0,0,0,0,0,0,0,0,0]]C1274397	actinomycetoma due to nocardia asteroides,mycetoma due to nocardia asteroides	[[1,0,0,0,0,0,0,0,0,0,0,0,0,0],[1,0,0,0,0,0,0,0,0,0,0,0,0,0]]C1274396	actinomycetoma due to nocardia brasiliensis,mycetoma due to nocardia brasiliensis	[[1,0,0,0,0,0,0,0,0,0,0,0,0,0],[1,0,0,0,0,0,0,0,0,0,0,0,0,0]]C0276828	avian trichomoniasis,avian canker,frounce,infection by trichomonas gallinae,roup,trichomoniasis in birds	[[1,0,0,0,0,0,0,0,0,0,0,0,0,0],[1,0,0,0,0,0,0,0,0,0,0,0,0,0],[1,0,0,0,0,0,0,0,0,0,0,0,0,0],[1,0,0,0,0,0,0,0,0,0,0,0,0,0],[1,0,0,0,0,0,0,1,0,0,0,0,0,0],[0,0,0,0,0,0,0,1,0,0,0,0,0,0]]C0276829	infection by tritrichomonas,tritrichomonosis	[[1,0,0,0,0,0,0,0,0,0,0,0,0,0],[1,0,0,0,0,0,0,0,0,0,0,0,0,0]]C0452198	sepsis of newborn due to escherichia coli	[[1,0,0,0,0,0,0,0,0,0,0,0,0,0]]C0452199	sepsis of newborn due to anaerobes	[[1,0,0,0,0,0,0,0,0,0,0,0,0,0]]C1850127	autosomal recessive 1 osteopetrosis,autosomal recessive marble bones,optb1,autosomal recessive albers schonberg disease,infantile malignant 1 osteopetrosis	[[0,0,0,1,0,0,0,0,0,0,0,0,0,0],[0,0,0,1,0,0,0,0,0,0,0,0,0,0],[0,0,0,1,0,0,0,0,0,0,0,0,0,0],[0,0,0,1,0,0,0,0,0,0,0,0,0,0],[0,0,0,1,0,0,0,0,0,0,0,0,0,0]]C0340646	type ii dissection of thoracic aorta,type 2 dissection of thoracic aorta,aortic root dissection	[[1,0,0,0,0,0,0,0,0,0,0,0,0,0],[1,0,0,0,0,0,0,0,0,0,0,0,0,0],[1,0,0,0,0,0,0,0,0,0,0,0,0,0]]C1270880	pressure urticaria,pressure angioedema urticaria,pressure angio oedema urticaria	[[1,0,0,0,0,0,0,1,0,0,0,0,0,0],[1,0,0,0,0,0,0,0,0,0,0,0,0,0],[1,0,0,0,0,0,0,0,0,0,0,0,0,0]]C0340644	dissection of thoracic aorta	[[1,0,0,0,0,0,0,1,0,0,0,0,0,0]]C0340645	type i dissection of thoracic aorta,type 1 dissection of thoracic aorta	[[1,0,0,0,0,0,0,0,0,0,0,0,0,0],[1,0,0,0,0,0,0,0,0,0,0,0,0,0]]C0340642	anterior spinal artery dissection	[[1,1,0,0,0,0,0,0,0,0,0,0,0,0]]C0340643	dissection of aorta,aortic dissection	[[1,0,0,0,0,0,0,1,0,0,0,0,0,0],[0,0,0,1,0,0,0,1,0,0,1,1,0,0]]C1879802	benign basal cell tumor of the rat skin,rat cutaneous benign basal cell tumor	[[0,0,0,0,0,0,0,0,0,0,0,1,0,0],[0,0,0,0,0,0,0,0,0,0,0,1,0,0]]C1335710	recurrent malignant peripheral nerve sheath tumor,recurrent malignant peripheral nerve sheath neoplasm,relapsed malignant peripheral nerve sheath neoplasm,relapsed malignant peripheral nerve sheath tumor,recurrent mpnst,relapsed mpnst	[[0,0,0,0,0,0,0,0,0,0,0,1,0,0],[0,0,0,0,0,0,0,0,0,0,0,1,0,0],[0,0,0,0,0,0,0,0,0,0,0,1,0,0],[0,0,0,0,0,0,0,0,0,0,0,1,0,0],[0,0,0,0,0,0,0,0,0,0,0,1,0,0],[0,0,0,0,0,0,0,0,0,0,0,1,0,0]]C0677656	carcinoma brain	[[0,0,0,0,0,0,0,1,1,0,0,0,0,0]]C1879808	benign intraocular medulloepithelioma	[[0,0,0,0,0,0,0,0,0,0,0,1,0,0]]C1879809	benign intraocular teratoid medulloepithelioma	[[0,0,0,0,0,0,0,0,0,0,0,1,0,0]]C0340648	dissection of coronary artery	[[0,0,0,0,1,0,1,1,0,0,0,0,0,0]]C0340649	iliac dissection,dissection of iliac artery	[[1,0,0,0,0,0,0,0,0,0,0,0,0,0],[0,0,0,0,1,0,0,0,0,0,0,0,0,0]]C1335419	pineal region teratoma,teratoma of the pineal area	[[0,0,0,0,0,0,0,0,0,0,0,1,0,0],[0,0,0,0,0,0,0,0,0,0,0,1,0,0]]C2986658	diffuse intrinsic pontine glioma,dipg	[[0,0,0,0,0,0,0,0,0,0,0,1,0,0],[0,0,0,0,0,0,0,0,0,0,0,1,0,0]]C1335712	recurrent medulloblastoma,relapsed medulloblastoma	[[0,0,0,0,0,0,0,0,0,0,0,1,0,0],[0,0,0,0,0,0,0,0,0,0,0,1,0,0]]C2959445	angiomyomatous hamartoma	[[1,0,0,0,0,0,0,0,0,0,0,0,0,0]]C1444672	traumatic iridodialysis	[[1,0,0,0,0,0,0,0,0,0,0,0,0,0]]C0347276	benign neoplasm of anus,benign tumor of anus,benign tumour of anus,benign anal neoplasm,benign anal tumor	[[1,0,0,0,0,0,0,0,0,0,0,1,0,1],[1,0,0,0,0,0,0,0,0,0,0,1,0,0],[1,0,0,0,0,0,0,0,0,0,0,0,0,0],[0,0,0,0,0,0,0,0,0,0,0,1,0,0],[0,0,0,0,0,0,0,0,0,0,0,1,0,0]]C2939419	secondary neoplasm,metastases,secondaries,metastatic cancer,secondary malignant deposit,metastatic malignant disease,tumor metastasis,tumour metastasis,metastatic neoplasm,metastatic tumor,metastatic tumour,secondary tumor,secondary tumour,ca secondary cancer,secondary cancer,secondary malignant neoplastic disease,metastatic malignant neoplasm	[[1,0,0,0,0,0,0,1,0,0,0,1,0,0],[1,0,0,0,0,0,0,0,0,0,0,0,0,0],[1,0,0,0,0,0,0,0,0,0,0,0,0,0],[1,0,0,0,0,0,0,0,0,0,0,1,0,0],[1,0,0,0,0,0,0,0,0,0,0,0,0,0],[1,0,0,0,0,0,0,0,0,0,0,0,0,0],[1,0,0,0,0,0,0,0,0,0,0,0,0,0],[1,0,0,0,0,0,0,0,0,0,0,0,0,0],[1,0,0,0,0,0,0,0,0,0,0,0,0,0],[1,0,0,0,0,0,0,0,0,0,0,0,0,0],[1,0,0,0,0,0,0,0,0,0,0,0,0,0],[1,0,0,0,0,0,0,0,0,0,0,1,0,0],[1,0,0,0,0,0,0,0,0,0,0,0,0,0],[1,0,0,0,0,0,0,0,0,0,0,0,0,0],[1,0,0,0,0,0,0,0,0,0,0,0,0,0],[1,0,0,0,0,0,0,0,0,0,0,0,0,0],[0,0,0,0,0,0,0,0,0,0,0,1,0,0]]C1335714	recurrent metastatic squamous cell carcinoma to the neck with occult primary,recurrent metastatic squamous cell cancer to the neck with occult primary,relapsed metastatic squamous cell neck cancer with occult primary,relapsed metastatic squamous cell carcinoma to the neck with occult primary,recurrent metastatic epidermoid carcinoma to the neck with occult primary,relapsed metastatic epidermoid carcinoma to the neck with occult primary	[[0,0,0,0,0,0,0,0,0,0,0,1,0,0],[0,0,0,0,0,0,0,0,0,0,0,1,0,0],[0,0,0,0,0,0,0,0,0,0,0,1,0,0],[0,0,0,0,0,0,0,0,0,0,0,1,0,0],[0,0,0,0,0,0,0,0,0,0,0,1,0,0],[0,0,0,0,0,0,0,0,0,0,0,1,0,0]]C1559808	trachea infection adverse event with normal absolute neutrophil count or grade 1 or 2 neutrophils,trachea infection with normal anc or grade 1 or 2 neutrophils	[[0,0,0,0,0,0,0,0,0,0,0,1,0,0],[0,0,0,0,0,0,0,0,0,0,0,1,0,0]]C0686179	secondary malignant neoplasm of paraurethral glands,metastatic malignant neoplasm to paraurethral glands	[[1,0,0,0,0,0,0,0,0,0,0,0,0,0],[1,0,0,0,0,0,0,0,0,0,0,0,0,0]]C0686178	carcinoma in situ of paraurethral glands	[[1,0,0,0,0,0,0,0,0,0,0,0,0,0]]C0452222	flatback syndrome	[[1,0,0,0,0,0,0,0,0,0,0,0,0,0]]C0452220	chronic crepitant synovitis of hand and wrist	[[1,0,0,0,0,0,0,0,0,0,0,0,0,0]]C0452221	osteomyelitis of vertebra,vertebral osteomyelitis	[[1,0,0,0,0,0,0,0,0,0,0,0,0,0],[0,0,0,0,0,0,0,1,0,0,0,0,0,0]]C0686172	carcinoma in situ of kidney	[[1,0,0,0,0,0,0,0,0,0,0,0,0,0]]C1274647	pigmentation due to exogenous pigment	[[1,0,0,0,0,0,0,0,0,0,0,0,0,0]]C1274646	ochronosis due to hydroquinone,hydroquinone induced ochronosis	[[1,0,0,0,0,0,0,0,0,0,0,0,0,0],[1,0,0,0,0,0,0,0,0,0,0,0,0,0]]C3165073	sarcoma of lower outer quadrant of female breast	[[1,0,0,0,0,0,0,0,0,0,0,0,0,0]]C1274644	hypermelanosis of the eyelids due to hyperthyroidism,jellineks sign	[[1,0,0,0,0,0,0,0,0,0,0,0,0,0],[1,0,0,0,0,0,0,0,0,0,0,0,0,0]]C1274643	hypermelanosis due to hyperthyroidism	[[1,0,0,0,0,0,0,0,0,0,0,0,0,0]]C1274642	hypermelanosis due to acromegaly	[[1,0,0,0,0,0,0,0,0,0,0,0,0,0]]C1274641	hypermelanosis due to cushing syndrome	[[1,0,0,0,0,0,0,0,0,0,0,0,0,0]]C1274640	buccal pigmentation due to addisons disease	[[1,0,0,0,0,0,0,0,0,0,0,0,0,0]]C3165079	ebsteins anomaly of right atrioventricular valve in functionally univentricular heart	[[1,0,0,0,0,0,0,0,0,0,0,0,0,0]]C2676272	3 androgenetic alopecia,aga3	[[0,0,0,1,0,0,0,0,0,0,0,0,0,0],[0,0,0,1,0,0,0,0,0,0,0,0,0,0]]C2676271	3 familial restrictive cardiomyopathy,rcm3	[[0,0,0,1,0,0,1,0,0,0,0,0,0,0],[0,0,0,1,0,0,0,0,0,0,0,0,0,0]]C1274649	trichrome vitiligo	[[1,0,0,0,0,0,0,0,0,0,0,0,0,0]]C1274648	segmental vitiligo	[[1,0,0,0,0,0,0,0,0,0,0,0,0,0]]C0276022	juxta articular yaws nodules	[[1,0,0,0,0,0,0,0,0,0,0,0,0,1]]C0001824	agranulocytosis,agranulocytoses,granulocytopenia,granulopenia,granulocytopenic disorder	[[0,0,1,0,0,0,0,0,0,1,0,0,0,0],[0,1,0,0,0,0,0,1,0,0,0,0,0,0],[0,1,1,0,0,0,0,0,1,0,0,0,0,0],[0,0,0,0,0,0,0,1,0,0,0,0,0,0],[1,0,0,0,0,0,0,0,0,0,0,0,0,0]]C0001828	agricultural workers diseases	[[0,1,0,0,0,0,0,0,0,0,0,0,1,0]]C2750424	with ocular abnormalities and congenital myasthenic syndrome congenital nephrotic syndrome	[[0,0,0,1,0,0,0,0,0,0,0,0,0,0]]C0410242	infective myositis hand	[[1,0,0,0,0,0,0,0,0,0,0,0,0,0]]C0410243	infective myositis forearm	[[1,0,0,0,0,0,0,0,0,0,0,0,0,0]]C0410240	infective myositis thigh	[[1,0,0,0,0,0,0,0,0,0,0,0,0,0]]C0410241	infective myositis pelvis	[[1,0,0,0,0,0,0,0,0,0,0,0,0,0]]C0410246	infective myositis back	[[1,0,0,0,0,0,0,0,0,0,0,0,0,0]]C0410247	infective myositis neck	[[1,0,0,0,0,0,0,0,0,0,0,0,0,0]]C0410244	infective myositis arm	[[1,0,0,0,0,0,0,0,0,0,0,0,0,0]]C0410245	infective myositis shoulder	[[1,0,0,0,0,0,0,0,0,0,0,0,0,0]]C0410248	benign acute myositis	[[1,0,0,0,0,0,0,0,0,0,0,0,0,0]]C0152203	comitant strabismus,concomitant strabismus,concomitant squint,comitant squint,constant comitant heterotropia,constant squint	[[0,1,0,0,0,0,0,0,0,0,0,0,0,0],[1,0,0,0,0,0,0,0,0,0,0,0,0,0],[1,0,0,0,0,0,0,0,0,0,0,0,0,0],[1,0,0,0,0,0,0,0,0,0,0,0,0,0],[1,0,0,0,0,0,0,0,0,0,0,0,0,0],[1,0,0,0,0,0,0,0,0,0,0,0,0,0]]C1442969	surgical ulcer	[[0,0,0,0,0,0,0,0,0,0,1,0,0,0]]C1320838	abscess of cardiac septum	[[1,0,0,0,0,0,0,0,0,0,0,0,0,0]]C0684807	neoplasm of uncertain behavior of face,neoplasm of uncertain behaviour of face	[[1,0,0,0,0,0,0,0,0,0,0,0,0,0],[1,0,0,0,0,0,0,0,0,0,0,0,0,0]]C1320839	abscess of connective tissue	[[1,0,0,0,0,0,0,0,0,0,0,0,0,0]]C0684806	benign neoplasm of face,benign tumor of face,benign tumour of face	[[1,0,0,0,0,0,0,0,0,0,0,0,0,0],[1,0,0,0,0,0,0,0,0,0,0,0,0,0],[1,0,0,0,0,0,0,0,0,0,0,0,0,0]]C0038218	status asthmaticus,asthmatic crisis,asthmatic crises,asthmatic shock,asthma with status asthmaticus,acute severe asthma	[[1,1,0,0,0,0,0,1,1,0,0,0,0,0],[0,1,0,0,0,0,0,1,0,0,0,0,1,0],[0,1,0,0,0,0,0,0,0,0,0,0,0,0],[0,1,0,0,0,0,0,0,0,0,0,0,1,0],[1,0,0,0,0,0,0,1,0,0,0,0,0,0],[1,0,0,0,0,0,0,1,0,0,0,0,0,0]]C1377605	pediatric cns embryonal cell carcinoma,childhood cns embryonal cell carcinoma,childhood central nervous system embryonal carcinoma,pediatric central nervous system embryonal carcinoma,embryonal carcinoma of the childhood cns,embryonal carcinoma of the pediatric cns	[[0,0,0,0,0,1,0,0,0,0,0,1,0,0],[0,0,0,0,0,1,0,0,0,0,0,1,0,0],[0,0,0,0,0,0,0,0,0,0,0,1,0,0],[0,0,0,0,0,0,0,0,0,0,0,1,0,0],[0,0,0,0,0,0,0,0,0,0,0,1,0,0],[0,0,0,0,0,0,0,0,0,0,0,1,0,0]]C1377604	pediatric cns choriocarcinoma,childhood cns choriocarcinoma,childhood central nervous system choriocarcinoma,pediatric central nervous system choriocarcinoma	[[0,0,0,0,0,1,0,0,0,0,0,1,0,0],[0,0,0,0,0,1,0,0,0,0,0,1,0,0],[0,0,0,0,0,0,0,0,0,0,0,1,0,0],[0,0,0,0,0,0,0,0,0,0,0,1,0,0]]C3266110	chronic otitis media with sanguineous effusion	[[1,0,0,0,0,0,0,0,0,0,0,0,0,0]]C1442968	anastomotic ulcer	[[1,0,0,0,0,0,0,0,0,0,1,0,0,0]]C1882661	rat erythroid leukemia,erythroid leukemia	[[0,0,0,0,0,0,0,0,0,0,0,1,0,0],[0,0,0,0,0,0,0,0,0,0,0,1,0,0]]C1882667	rat female genital system neoplasms	[[0,0,0,0,0,0,0,0,0,0,0,1,0,0]]C0156630	with delivery antepartum hemorrhage associated with coagulation defects	[[0,0,0,0,1,0,0,0,0,0,0,0,0,0]]C1521720	mouse gastric carcinoma	[[0,0,0,0,0,0,0,0,0,0,0,1,0,0]]C1836723	with pseudarthrosis and pectus excavatum bowing of tibia	[[0,0,0,1,0,0,0,0,0,0,0,0,0,0]]C0684809	secondary malignant neoplasm of face,metastatic malignant neoplasm to face	[[1,0,0,0,0,0,0,0,0,0,0,0,0,0],[1,0,0,0,0,0,0,0,0,0,0,0,0,0]]C0162549	tardive akathisia	[[0,1,0,0,0,0,0,0,0,0,0,0,0,0]]C2673736	dibasic amino aciduria i	[[0,0,0,1,0,0,0,0,0,0,0,0,0,0]]C3150653	complementation group o fanconi anemia,fanco	[[0,0,0,1,0,0,0,0,0,0,0,0,0,0],[0,0,0,1,0,0,0,0,0,0,0,0,0,0]]C1833030	nonepidermolytic palmoplantar keratoderma,tylosis,neppk	[[0,0,0,1,0,0,0,0,0,0,0,0,0,0],[0,0,0,1,0,0,0,0,0,0,0,0,0,0],[0,0,0,1,0,0,0,0,0,0,0,0,0,0]]C0158568	congenital macular changes	[[0,0,0,0,1,0,0,0,0,0,0,0,0,0]]C1333160	lipoma of corpus callosum	[[0,0,0,1,0,0,0,0,0,0,0,1,0,0]]C0158566	congenital chorioretinal degeneration	[[1,0,0,0,1,0,0,0,0,0,0,0,0,0]]C1838916	adult onset ataxia and polyneuropathy	[[0,0,0,1,0,0,0,0,0,0,0,0,0,0]]C1333162	cranial nodular fasciitis,cranial pseudosarcomatous fasciitis	[[0,0,0,0,0,0,0,0,0,0,0,1,0,0],[0,0,0,0,0,0,0,0,0,0,0,1,0,0]]C1838912	with villous atrophy chronic diarrhea	[[0,0,0,1,0,0,0,0,0,0,0,0,0,0]]C0263475	sweat gland cyst,sudoriferous cyst	[[1,0,0,0,0,0,0,1,0,0,0,0,0,0],[1,0,0,0,0,0,0,1,0,0,0,0,0,0]]C1275119	multiple neurofibromas in neurofibromatosis	[[1,0,0,0,0,0,0,0,0,0,0,0,0,0]]C0263477	female pattern alopecia,female pattern baldness	[[1,0,0,0,0,0,0,1,0,0,0,0,0,0],[1,0,0,0,0,0,0,1,0,0,0,0,0,0]]C1332894	central nervous system t cell non hodgkin lymphoma,central nervous system t cell and nk cell non hodgkins lymphoma,central nervous system t cell lymphoma,cns t cell lymphoma,primary central nervous system t cell non hodgkins lymphoma	[[0,0,0,0,0,0,0,0,0,0,0,1,0,0],[0,0,0,0,0,0,0,0,0,0,0,1,0,0],[0,0,0,0,0,0,0,0,0,0,0,1,0,0],[0,0,0,0,0,0,0,0,0,0,0,1,0,0],[0,0,0,0,0,0,0,0,0,0,0,1,0,0]]C0263471	granulosis rubra nasi	[[0,0,0,1,0,0,0,0,0,0,0,0,0,1]]C0263470	apocrine gland cyst,apocrine cyst,apocrine cystomatosis	[[1,0,0,0,0,0,0,1,0,0,0,0,0,0],[1,0,0,0,0,0,0,1,0,0,0,0,0,0],[1,0,0,0,0,0,0,0,0,0,0,0,0,0]]C0263473	chromhidrosis,secretion of colored sweat,secretion of coloured sweat	[[0,0,0,0,0,0,0,0,0,0,0,0,0,1],[1,0,0,0,0,0,0,0,0,0,0,0,0,0],[1,0,0,0,0,0,0,0,0,0,0,0,0,0]]C0263472	bromhidrosis,bromidrosis,osmidrosis,ozochrotia	[[0,0,0,0,0,0,0,1,0,0,0,0,0,1],[1,0,0,0,0,0,0,1,0,0,0,0,0,0],[1,0,0,0,0,0,0,1,0,0,0,0,0,0],[1,0,0,0,0,0,0,0,0,0,0,0,0,0]]C0451753	renal tubulo interstitial disorders in systemic connective tissue disorders	[[1,0,0,0,0,0,0,0,0,0,0,0,0,0]]C0451752	renal tubulo interstitial disorders in metabolic diseases	[[1,0,0,0,0,0,0,0,0,0,0,0,0,0]]C0451751	renal tubulo interstitial disorders in blood diseases and disorders involving the immune mechanism	[[1,0,0,0,0,0,0,0,0,0,0,0,0,0]]C0206019	hiv encephalopathy,aids encephalopathy,human immunodefiency virus encephalopathy,hiv encephalitis	[[0,1,0,0,0,0,0,1,0,0,0,0,0,0],[0,1,0,0,0,0,0,1,0,0,0,1,0,0],[1,0,0,0,0,0,0,0,0,0,0,0,0,0],[0,0,0,0,0,0,0,1,0,0,0,0,0,0]]C0263479	diffuse alopecia areata	[[1,0,0,0,0,0,0,0,0,0,0,0,0,0]]C0263478	ophiasis,ophiasic alopecia areata	[[1,0,0,0,0,0,0,1,0,0,0,0,0,0],[1,0,0,0,0,0,0,0,0,0,0,0,0,0]]C1275116	autosomal recessive pseudoxanthoma elasticum	[[1,0,0,0,0,0,0,0,0,0,0,0,0,0]]C1275117	cutis laxa secondary to inherited disorder of connective tissue	[[1,0,0,0,0,0,0,0,0,0,0,0,0,0]]C0275847	syphilitic ostial coronary disease	[[0,0,0,0,0,0,0,0,0,0,0,0,0,1]]C0275846	syphilitic aortic stenosis	[[1,0,0,0,0,0,0,0,0,0,0,0,0,0]]C0275844	syphilitic aneurysm of aorta,specified as syphilitic aneurysm of aorta,syphilitic dilatation of aorta,late quaternary syphilitic aortic aneurysm,syphilitic aortic aneurysm,specified as syphilitic dilatation of aorta	[[1,0,0,0,0,0,0,0,0,0,0,0,0,0],[0,0,0,0,1,0,0,0,0,0,0,0,0,0],[1,0,0,0,0,0,0,0,0,0,0,0,0,0],[1,0,0,0,0,0,0,0,0,0,0,0,0,0],[1,0,0,0,0,0,0,0,0,0,0,0,0,0],[0,0,0,0,0,0,0,0,0,0,0,0,0,1]]C0275843	with relapse after treatment negative cerebrospinal fluid positive serology early latent syphilis	[[1,0,0,0,0,0,0,0,0,0,0,0,0,0]]C0275842	less than 2 years after infection negative cerebrospinal fluid positive serology early latent syphilis,latent early syphilis	[[1,0,0,0,0,0,0,0,0,0,0,0,0,0],[1,0,0,0,1,0,0,0,0,0,0,0,0,0]]C0275841	relapse secondary syphilis,untreated secondary syphilis relapse	[[1,0,0,0,0,0,0,0,0,0,0,0,0,0],[0,0,0,0,0,0,0,0,0,0,0,0,0,1]]C0275840	relapse secondary syphilis,treated secondary syphilis relapse	[[1,0,0,0,0,0,0,0,0,0,0,0,0,0],[0,0,0,0,0,0,0,0,0,0,0,0,0,1]]C1335324	papillary epithelial neoplasm	[[0,0,0,0,0,0,0,0,0,0,0,1,0,0]]C1335325	papillary lung adenocarcinoma,lung pap ad	[[0,0,0,0,0,0,0,0,0,0,0,1,0,0],[0,0,0,0,0,0,0,0,0,0,0,1,0,0]]C1335327	thymic papillary adenocarcinoma,thymic papillary carcinoma,thymus papillary carcinoma	[[0,0,0,0,0,0,0,0,0,0,0,1,0,0],[0,0,0,0,0,0,0,0,0,0,0,1,0,0],[0,0,0,0,0,0,0,0,0,0,0,1,0,0]]C1335320	papillary breast carcinoma	[[0,0,0,0,0,0,0,0,0,0,0,1,0,0]]C1335321	papillary breast neoplasm,papillary tumor of the breast	[[0,0,0,0,0,0,0,0,0,0,0,1,0,0],[0,0,0,0,0,0,0,0,0,0,0,1,0,0]]C0275849	taboparesis,tabo paresis	[[0,0,0,0,0,0,0,0,0,0,0,0,0,1],[1,0,0,0,0,0,0,0,0,0,0,0,0,0]]C0275848	syphilitic punched out ulcer	[[1,0,0,0,0,0,0,0,0,0,0,0,0,0]]C0393945	myasthenia gravis associated with thymoma	[[1,0,0,0,0,0,0,0,0,0,0,0,0,0]]C0393947	cholinergic crisis,cholinergic paralysis	[[1,0,0,0,0,0,0,1,0,0,0,0,0,0],[1,0,0,0,0,0,0,0,0,0,0,0,0,0]]C0393946	myopathy in myasthenia gravis,myasthenic myopathy	[[1,0,0,0,0,0,0,0,0,0,0,0,0,0],[1,0,0,0,0,0,0,0,0,0,0,0,0,0]]C0393941	persistent neonatal myasthenia gravis	[[1,1,0,0,0,0,0,0,0,0,0,0,0,0]]C0393940	penicillamine induced myasthenia	[[1,0,0,0,0,0,0,0,0,0,0,0,0,0]]C0346178	ovarian gynandroblastoma,gynandroblastoma of ovary	[[0,1,0,0,0,0,0,0,0,0,0,1,0,0],[1,0,0,0,0,0,0,0,0,0,0,1,0,0]]C0346179	hilus cell tumor of ovary,hilus cell tumour of ovary,hilar cell tumor,hilar cell tumour,hilus cell tumor,hilus cell tumour,m hilar cell tumor,m hilar cell tumour,hilar cell neoplasm,hilar cell tumor of the ovary,hilus cell neoplasm,ovarian hilar cell tumor,ovarian hilus cell tumor	[[1,0,0,0,0,0,0,0,0,0,0,0,0,0],[1,0,0,0,0,0,0,0,0,0,0,0,0,0],[1,0,0,0,0,0,0,0,0,0,0,0,0,0],[1,0,0,0,0,0,0,0,0,0,0,0,0,0],[1,0,0,0,0,0,0,0,0,0,0,1,0,0],[1,0,0,0,0,0,0,0,0,0,0,0,0,0],[1,0,0,0,0,0,0,0,0,0,0,0,0,0],[1,0,0,0,0,0,0,0,0,0,0,0,0,0],[0,0,0,0,0,0,0,0,0,0,0,1,0,0],[0,0,0,0,0,0,0,0,0,0,0,1,0,0],[0,0,0,0,0,0,0,0,0,0,0,1,0,0],[0,0,0,0,0,0,0,0,0,0,0,1,0,0],[0,0,0,0,0,0,0,0,0,0,0,1,0,0]]C0346176	benign sex cord tumor of ovary,benign sex cord tumour of ovary	[[1,0,0,0,0,0,0,0,0,0,0,0,0,0],[1,0,0,0,0,0,0,0,0,0,0,0,0,0]]C0346174	malignant sex cord tumor of ovary,malignant sex cord tumour of ovary	[[1,0,0,0,0,0,0,0,0,0,0,0,0,0],[1,0,0,0,0,0,0,0,0,0,0,0,0,0]]C0346175	malignant ovarian granulosa cell tumor,malignant granulosa cell tumor of ovary,malignant granulosa cell tumour of ovary,malignant ovarian granulosa cell neoplasm,malignant granulosa cell neoplasm of the ovary	[[0,0,0,0,0,0,0,0,0,0,0,1,0,0],[1,0,0,0,0,0,0,0,0,0,0,1,0,0],[1,0,0,0,0,0,0,0,0,0,0,0,0,0],[0,0,0,0,0,0,0,0,0,0,0,1,0,0],[0,0,0,0,0,0,0,0,0,0,0,1,0,0]]C0346172	mucinous cystadenoma of ovary,benign ovarian mucinous cystadenoma,benign mucinous cystadenoma of the ovary,ovarian mucinous cystadenoma	[[1,0,0,0,0,0,0,0,0,0,0,1,0,0],[0,0,0,0,0,0,0,0,0,0,0,1,0,0],[0,0,0,0,0,0,0,0,0,0,0,1,0,0],[0,0,0,0,0,0,0,0,0,0,0,1,0,0]]C0393948	cerebrovascular and spinal vascular disorders	[[1,0,0,0,0,0,0,0,0,0,0,0,0,0]]C0346170	serous cystadenoma of ovary,benign ovarian serous cystadenoma,benign serous cystadenoma of the ovary,ovarian serous cystadenoma	[[1,0,0,0,0,0,0,0,0,0,0,1,0,0],[0,0,0,0,0,0,0,0,0,0,0,1,0,0],[0,0,0,0,0,0,0,0,0,0,0,1,0,0],[0,0,0,0,0,0,0,0,0,0,0,1,0,0]]C0346171	serous papillary cystadenoma of ovary	[[1,0,0,0,0,0,0,0,0,0,0,0,0,0]]C0684508	malignant melanoma of skin of arm	[[0,0,0,0,0,0,0,0,0,0,0,0,0,1]]C0429468	anovulatory,anovulatory cycle,anovular cycle	[[1,0,0,0,0,0,0,1,0,0,0,0,0,0],[1,0,1,0,0,0,0,1,0,0,0,0,0,0],[1,0,0,0,0,0,0,0,0,0,0,0,0,0]]C1288270	hypertension without albuminuria and without edema in the obstetric context,hypertension without albuminuria and without oedema in the obstetric context,hypertension in the obstetric context	[[1,0,0,0,0,0,0,0,0,0,0,0,0,0],[1,0,0,0,0,0,0,0,0,0,0,0,0,0],[1,0,0,0,0,0,0,0,0,0,0,0,0,0]]C1710641	vulvar angiomyofibroblastoma	[[0,0,0,0,0,0,0,0,0,0,0,1,0,0]]C1882575	rat benign hair follicle tumor,benign hair follicle tumor	[[0,0,0,0,0,0,0,0,0,0,0,1,0,0],[0,0,0,0,0,0,0,0,0,0,0,1,0,0]]C0747555	exudative pharyngitis	[[1,0,0,0,0,0,0,0,0,0,0,0,0,0]]C3164849	sarcoma of female breast	[[1,0,0,0,0,0,0,0,0,0,0,0,0,0]]C1882838	proliferative change of the rat respiratory system,rat respiratory system disorder	[[0,0,0,0,0,0,0,0,0,0,0,1,0,0],[0,0,0,0,0,0,0,0,0,0,0,1,0,0]]C1518198	malignant mouse exocervical neoplasm	[[0,0,0,0,0,0,0,0,0,0,0,1,0,0]]C0949570	wheat hypersensitivity,wheat allergy	[[0,1,0,0,0,0,0,1,0,0,0,0,1,0],[1,1,0,0,0,0,0,1,0,0,0,0,1,0]]C1840394	familial cystic parathyroid adenomatosis	[[0,0,0,1,0,0,0,0,0,0,0,0,0,0]]C3164840	double outlet ventriculoarterial connections	[[1,0,0,0,0,0,0,0,0,0,0,0,0,0]]C1828226	herpes zoster corneal endotheliolitis	[[1,0,0,0,0,0,0,0,0,0,0,0,0,0]]C1288273	deficiency of limit dextrinase,deficiency of alpha dextrin endo 1 6 alpha glucosidase	[[1,0,0,0,0,0,0,0,0,0,0,0,0,0],[1,0,0,0,0,0,0,0,0,0,0,0,0,0]]C1840392	familial progressive hyperpigmentation,hpp,melanosis universalis hereditaria,fph,muh	[[0,0,0,1,0,0,0,0,0,0,0,0,0,0],[0,0,0,1,0,0,0,0,0,0,0,0,0,0],[0,0,0,1,0,0,0,0,0,0,0,0,0,0],[0,0,0,1,0,0,0,0,0,0,0,0,0,0],[0,0,0,1,0,0,0,0,0,0,0,0,0,0]]C1840393	hyperpigmentation of fuldauer and kuijpers	[[0,0,0,1,0,0,0,0,0,0,0,0,0,0]]C2931398	kosztolanyi syndrome,and mental retardation feeding difficulties protruding eyes disturbance of cranial ossification syndrome of arachnodactyly,abnormal ossification and mental retardation arachnodactyly	[[0,1,0,0,0,0,0,0,0,0,0,0,0,0],[0,1,0,0,0,0,0,0,0,0,0,0,0,0],[0,1,0,0,0,0,0,0,0,0,0,0,0,0]]C0035127	cumulative trauma disorders,repetition strain injury,repetitive motion disorders,overuse syndrome,rsi,overuse injury,rsi repetitive strain injury,repetitive strain injury syndrome,rsis repetitive strain injury syndrome,repetitive motion injuries	[[0,1,0,0,0,0,0,1,0,0,0,0,1,0],[1,1,0,0,0,0,0,1,0,0,0,0,1,0],[0,1,0,0,0,0,0,1,0,0,0,0,1,0],[0,1,0,0,0,0,0,1,0,0,0,0,1,0],[1,0,0,0,0,0,0,1,0,0,0,0,0,0],[0,1,0,0,0,0,0,1,0,0,0,0,1,0],[1,0,0,0,0,0,0,0,0,0,0,0,0,0],[1,0,0,0,0,0,0,0,0,0,0,0,0,0],[1,0,0,0,0,0,0,0,0,0,0,0,0,0],[0,0,0,0,0,0,0,1,0,0,0,0,0,0]]C0948222	sarcoma of peritoneum	[[1,0,0,0,0,0,0,0,0,0,0,0,0,0]]C0948221	pyelocaliectasis	[[0,0,0,0,0,0,0,1,0,0,0,0,0,0]]C2931390	leichtman wood rohn syndrome,and cns anomalies and hypothalamic disorder facial anomalies cleft lip palate anophthalmia	[[0,1,0,0,0,0,0,0,0,0,0,0,0,0],[0,1,0,0,0,0,0,0,0,0,0,0,0,0]]C2931391	karandikar maria kamble syndrome,cataract mental retardation anal atresia urinary defects,congenital cataract with multiple congenital anomalies in a sibship	[[0,1,0,0,0,0,0,0,0,0,0,0,0,0],[0,1,0,0,0,0,0,0,0,0,0,0,0,0],[0,1,0,0,0,0,0,0,0,0,0,0,0,0]]C1847627	with facial myokymia familial dyskinesia	[[0,0,0,1,0,0,0,0,0,0,0,0,0,0]]C1847626	autosomal dominant 36 deafness,dfna36	[[0,0,0,1,0,0,0,0,0,0,0,0,0,0],[0,0,0,1,0,0,0,0,0,0,0,0,0,0]]C2931394	katsantoni papadakou lagoyanni syndrome,trichodermal syndrome and mental retardation	[[0,1,0,0,0,0,0,0,0,0,0,0,0,0],[0,1,0,0,0,0,0,0,0,0,0,0,0,0]]C2931395	x linked recessive bulbospinal neuronopathy,xbsn	[[0,1,0,1,0,0,0,0,0,0,0,0,0,0],[0,0,0,1,0,0,0,0,0,0,0,0,0,0]]C2931396	kennerknecht vogel syndrome,agonadism mental retardation delayed bone age	[[0,1,0,0,0,0,0,0,0,0,0,0,0,0],[0,1,0,0,0,0,0,0,0,0,0,0,0,0]]C1847622	split hand foot malformation 5,shfm5,split hand foot malformation type 5	[[0,0,0,1,0,0,1,0,0,0,0,1,0,0],[0,0,0,1,0,0,0,0,0,0,0,0,0,0],[0,0,0,0,0,0,0,0,0,0,0,1,0,0]]C1541923	infective endocarditis,infective endocarditides,ie infective endocarditis	[[1,1,0,0,0,0,0,0,0,0,0,1,0,0],[0,1,0,0,0,0,0,0,0,0,0,0,0,0],[1,0,0,0,0,0,0,0,0,0,0,0,0,0]]C2675192	type 5 spherocytosis,hs5,sph5,5 hereditary spherocytosis	[[0,0,0,1,0,0,0,0,0,0,0,0,0,0],[0,0,0,1,0,0,0,0,0,0,0,0,0,0],[0,0,0,1,0,0,0,0,0,0,0,0,0,0],[0,0,0,1,0,0,0,0,0,0,0,0,0,0]]C2675191	bilateral occipital polymicrogyria	[[0,0,0,1,0,0,0,0,0,0,0,0,0,0]]C2936701	myocardial tumors	[[0,1,0,0,0,0,0,0,0,0,0,0,0,0]]C2981274	stage ivb thyroid gland medullary carcinoma,stage ivb thyroid gland medullary carcinoma ajcc v7	[[0,0,0,0,0,0,0,0,0,0,0,1,0,0],[0,0,0,0,0,0,0,0,0,0,0,1,0,0]]C2981275	stage ivc thyroid gland medullary carcinoma,stage ivc thyroid gland medullary carcinoma ajcc v7	[[0,0,0,0,0,0,0,0,0,0,0,1,0,0],[0,0,0,0,0,0,0,0,0,0,0,1,0,0]]C2981276	stage iva thyroid gland undifferentiated anaplastic carcinoma,stage iva thyroid gland undifferentiated anaplastic carcinoma ajcc v7	[[0,0,0,0,0,0,0,0,0,0,0,1,0,0],[0,0,0,0,0,0,0,0,0,0,0,1,0,0]]C2981277	stage ivb thyroid gland undifferentiated anaplastic carcinoma,stage ivb thyroid gland undifferentiated anaplastic carcinoma ajcc v7	[[0,0,0,0,0,0,0,0,0,0,0,1,0,0],[0,0,0,0,0,0,0,0,0,0,0,1,0,0]]C2981270	stage iva differentiated thyroid gland carcinoma,stage iva differentiated thyroid gland carcinoma ajcc v7	[[0,0,0,0,0,0,0,0,0,0,0,1,0,0],[0,0,0,0,0,0,0,0,0,0,0,1,0,0]]C1864939	and early death inguinal hernias arthrogryposis multiplex with deafness	[[0,1,0,1,0,0,0,0,0,0,0,0,0,0]]C2981272	stage ivc differentiated thyroid gland carcinoma,stage ivc differentiated thyroid gland carcinoma ajcc v7	[[0,0,0,0,0,0,0,0,0,0,0,1,0,0],[0,0,0,0,0,0,0,0,0,0,0,1,0,0]]C2981273	stage iva thyroid gland medullary carcinoma,stage iva thyroid gland medullary carcinoma ajcc v7	[[0,0,0,0,0,0,0,0,0,0,0,1,0,0],[0,0,0,0,0,0,0,0,0,0,0,1,0,0]]C0220754	biotinidase deficiency,late onset multiple carboxylase deficiency,juvenile multiple carboxylase deficiency,btd deficiency,late onset biotin responsive multiple carboxylase deficiency	[[1,1,0,1,0,0,0,1,0,0,0,1,1,0],[1,1,0,1,0,0,0,0,0,0,0,0,1,0],[1,0,0,0,0,0,0,0,0,0,0,0,0,0],[0,0,0,1,0,0,0,0,0,0,0,0,0,0],[0,0,0,0,0,0,0,0,0,0,0,0,1,0]]C0220757	familial infantile type osteochondrosis deformans tibiae	[[0,1,0,0,0,0,1,0,0,0,0,0,0,0]]C0220756	type c niemann pick disease,supraoptic vertical ophthalmoplegia,npc,niemann pick disease with cholesterol esterification block,chronic neuronopathic form niemann pick disease,niemann pick disease without sphingomyelinase deficiency,neurovisceral storage disease with vertical supranuclear ophthalmoplegia	[[1,1,0,1,0,0,0,1,0,0,0,1,1,0],[1,0,0,0,0,0,0,0,0,0,0,0,0,0],[0,0,0,1,0,0,0,0,0,0,0,0,0,0],[0,1,0,1,0,0,0,0,0,0,0,0,1,0],[0,1,0,1,0,0,0,0,0,0,0,0,1,0],[0,1,0,1,0,0,0,0,0,0,0,0,1,0],[0,0,0,1,0,0,0,0,0,0,0,0,1,0]]C2981278	stage ivc thyroid gland undifferentiated anaplastic carcinoma,stage ivc thyroid gland undifferentiated anaplastic carcinoma ajcc v7	[[0,0,0,0,0,0,0,0,0,0,0,1,0,0],[0,0,0,0,0,0,0,0,0,0,0,1,0,0]]C0040156	thyrotoxicosis,thyrotoxicoses,thyrotoxicosis with or without goitre	[[0,0,1,0,0,0,0,1,1,0,0,0,0,1],[0,1,0,0,0,0,0,0,0,0,0,0,0,0],[1,0,0,0,0,0,0,0,0,0,0,0,0,0]]C0264126	tibial plateau chondromalacia	[[1,0,0,0,0,0,0,0,0,0,0,0,0,1]]C0264127	diaphysitis	[[0,0,0,1,0,0,0,0,0,0,0,0,0,0]]C0264125	localized chondromalacia,localised chondromalacia,except patella localized chondromalacia	[[1,0,0,0,0,0,0,0,0,0,0,0,0,0],[1,0,0,0,0,0,0,0,0,0,0,0,0,0],[0,0,0,0,0,0,0,0,0,0,0,0,0,1]]C0264122	disuse atrophy,disuse muscle atrophy	[[1,1,0,0,0,0,0,1,0,0,0,0,0,0],[1,0,1,0,0,0,0,1,0,0,0,0,0,0]]C0157278	antepartum prolapse of cord complicating labor and delivery	[[0,0,0,0,1,0,0,0,0,0,0,0,0,0]]C0264121	piriform sclerosis of ilium	[[0,0,0,0,0,0,0,0,0,0,0,0,0,1]]C1855762	with absence of hla determinants and beta 2 microglobulin from lymphocytes partial combined immunodeficiency	[[0,0,0,1,0,0,0,0,0,0,0,0,0,0]]C0157277	delivered prolapse of cord complicating labor and delivery	[[0,0,0,0,1,0,0,0,0,0,0,0,0,0]]C0264128	cage layer fatigue	[[1,0,0,0,0,0,0,0,0,0,0,0,0,0]]C0157272	delivered etc triplet delayed delivery of second twin	[[0,0,0,0,1,0,0,0,0,0,0,0,0,0]]C1838263	autosomal recessive 3 deafness,neurosensory nonsyndromic recessive deafness 3,dfnb3,nsrd3	[[0,0,0,1,0,0,0,0,0,0,0,0,0,0],[0,0,0,1,0,0,0,0,0,0,0,0,0,0],[0,0,0,1,0,0,0,0,0,0,0,0,0,0],[0,0,0,1,0,0,0,0,0,0,0,0,0,0]]C1838262	3 insulin dependent diabetes mellitus,iddm3	[[0,0,0,1,0,0,0,0,0,0,0,0,0,0],[0,0,0,1,0,0,0,0,0,0,0,0,0,0]]C1838261	4 insulin dependent diabetes mellitus,iddm4	[[0,0,0,1,0,0,0,0,0,0,0,0,0,0],[0,0,0,1,0,0,0,0,0,0,0,0,0,0]]C1838260	5 insulin dependent diabetes mellitus,iddm5	[[0,0,0,1,0,0,0,0,0,0,0,0,0,0],[0,0,0,1,0,0,0,0,0,0,0,0,0,0]]C2931026	moloney syndrome,choroidal atrophy alopecia,regional choroidal atrophy and alopecia	[[0,1,0,0,0,0,0,0,0,0,0,0,0,0],[0,1,0,0,0,0,0,0,0,0,0,0,0,0],[0,1,0,0,0,0,0,0,0,0,0,0,0,0]]C2217265	malignant neoplasm of oropharynx stage i	[[0,0,0,0,0,0,1,0,0,0,0,0,0,0]]C2931024	moebius axonal neuropathy hypogonadism	[[0,1,0,0,0,0,0,0,0,0,0,0,0,0]]C2931025	moebius syndrome 1	[[0,1,0,0,0,0,0,0,0,0,0,0,0,0]]C1864125	type 2 nocturnal frontal lobe epilepsy,enfl2	[[0,0,0,1,0,0,0,0,0,0,0,0,0,0],[0,0,0,1,0,0,0,0,0,0,0,0,0,0]]C1864124	schizophrenia 8,chromosome 18 related schizophrenia susceptibility locus,sczd8	[[0,0,0,1,0,0,1,0,0,0,0,0,0,0],[0,0,0,1,0,0,0,0,0,0,0,0,0,0],[0,0,0,1,0,0,0,0,0,0,0,0,0,0]]C2931020	autosomal recessive spondylocostal dysostosis	[[0,1,0,0,0,0,0,0,0,0,0,0,0,0]]C2931021	arthrogryposis and popliteal pterygium spondylohypoplasia	[[0,1,0,0,0,0,0,0,0,0,0,0,0,0]]C1519823	ureter urothelial papilloma	[[0,0,0,0,0,0,0,0,0,0,0,1,0,0]]C1519827	urethral verrucous carcinoma	[[0,0,0,0,0,0,0,0,0,0,0,1,0,0]]C1519826	urethral urothelial papilloma	[[0,0,0,0,0,0,0,0,0,0,0,1,0,0]]C1519825	urethral glandular metaplasia	[[0,0,0,0,0,0,0,0,0,0,0,1,0,0]]C1519828	urethral villous adenoma	[[0,0,0,0,0,0,0,0,0,0,0,1,0,0]]C1883278	teratoma of the rat ovary	[[0,0,0,0,0,0,0,0,0,0,0,1,0,0]]C1839792	syndromic 12 x linked mental retardation,mrxs12	[[0,0,0,1,0,0,0,0,0,0,0,0,0,0],[0,0,0,1,0,0,0,0,0,0,0,0,0,0]]C2212006	ovarian small cell carcinoma	[[0,0,0,0,0,0,0,0,0,0,0,1,0,0]]C1840531	attenuated cholesterol lowering by statins	[[0,0,0,1,0,0,0,0,0,0,0,0,0,0]]C1707525	cortisol producing adrenal cortex adenoma,cortisol producing adrenal cortical adenoma	[[0,0,0,0,0,0,0,0,0,0,0,1,0,0],[0,0,0,0,0,0,0,0,0,0,0,1,0,0]]C0409543	pyogenic arthritis of hip	[[1,0,0,0,0,0,0,0,0,0,0,0,0,0]]C0409542	knee pyogenic arthritis	[[1,0,0,0,0,0,0,0,0,0,0,0,0,0]]C0409541	pyogenic arthritis of the ankle and or foot	[[1,0,0,0,0,0,0,0,0,0,0,0,0,0]]C1112160	gastroesophageal cancer	[[0,0,0,0,0,0,0,1,0,0,0,0,0,0]]C0409546	elbow pyogenic arthritis	[[1,0,0,0,0,0,0,0,0,0,0,0,0,0]]C0409545	wrist pyogenic arthritis	[[1,0,0,0,0,0,0,0,0,0,0,0,0,0]]C0409544	pyogenic arthritis of the pelvic region and thigh	[[1,0,0,0,0,0,0,0,0,0,0,0,0,0]]C2584501	drug induced thrombotic thrombocytopenic purpura	[[1,0,0,0,0,0,0,0,0,0,0,0,0,0]]C3150596	mild mononeuropathy of the median nerve,mnmn,susceptibility to carpal tunnel syndrome	[[0,0,0,1,0,0,0,0,0,0,0,0,0,0],[0,0,0,1,0,0,0,0,0,0,0,0,0,0],[0,0,0,1,0,0,0,0,0,0,0,0,0,0]]C0571017	ethamivan allergy	[[1,0,0,0,0,0,0,0,0,0,0,0,0,0]]C0302810	uremia syndrome,uremic syndrome,syndrome uraemic	[[0,0,0,0,0,0,1,1,0,0,0,0,0,0],[0,0,0,0,0,0,0,1,1,0,0,0,0,0],[0,0,0,0,0,0,0,1,0,0,0,0,0,0]]C2677843	type 7 episodic ataxia,ea7	[[0,0,0,1,0,0,0,0,0,0,0,0,0,0],[0,0,0,1,0,0,0,0,0,0,0,0,0,0]]C0678198	postpartum endometritis	[[0,0,0,0,0,0,0,1,0,0,1,0,0,0]]C0678199	dyserythropoietic anemia,dyshematopoietic anemia	[[0,0,0,1,0,0,0,0,0,1,0,0,0,1],[0,0,0,0,0,0,0,0,0,1,0,0,0,1]]C1290273	neoplasm of tarsal bone	[[1,0,0,0,0,0,0,0,0,0,0,0,0,0]]C1297994	malignant tumor involving left ovary by direct extension from uterine cervix,malignant tumour involving left ovary by direct extension from uterine cervix	[[1,0,0,0,0,0,0,0,0,0,0,0,0,0],[1,0,0,0,0,0,0,0,0,0,0,0,0,0]]C0473472	precipitate labor,precipitate labour	[[0,0,0,0,0,0,0,1,1,0,0,0,0,0],[1,0,0,0,0,0,0,1,0,0,0,0,0,0]]C3164537	ventricular septal defect with malaligned outlet septum	[[1,0,0,0,0,0,0,0,0,0,0,0,0,0]]C0870074	inferior infarction	[[0,0,0,0,0,0,0,1,0,0,0,0,0,1]]C0571016	nikethamide allergy	[[1,0,0,0,0,0,0,0,0,0,0,0,0,0]]C1863238	late onset scid due to ada deficiency	[[0,0,0,1,0,0,0,0,0,0,0,0,0,0]]C1707296	cardiac cavernous hemangioma	[[0,0,0,0,0,0,0,0,0,0,0,1,0,0]]C1707291	carcinoma with t 15 19 q13 p13 1 translocation	[[0,0,0,0,0,0,0,0,0,0,0,1,0,0]]C1707290	carcinoma ex eccrine spiradenoma	[[0,0,0,0,0,0,0,0,0,0,0,1,0,0]]C1707293	cardiac biphasic synovial sarcoma	[[0,0,0,0,0,0,0,0,0,0,0,1,0,0]]C1297995	malignant tumor involving left ovary by direct extension from uterus,malignant tumour involving left ovary by direct extension from uterus	[[1,0,0,0,0,0,0,0,0,0,0,0,0,0],[1,0,0,0,0,0,0,0,0,0,0,0,0,0]]C1707299	cardiac inflammatory myofibroblastic tumor	[[0,0,0,0,0,0,0,0,0,0,0,1,0,0]]C1707298	cardiac hemangioma	[[0,0,0,0,0,0,0,0,0,0,0,1,0,0]]C0860559	blood clot discharge	[[0,0,0,0,0,0,0,1,0,0,0,0,0,0]]C2749477	autosomal recessive oculodentodigital dysplasia,autosomal recessive odod,autosomal recessive oculodentoosseous dysplasia,autosomal recessive oddd	[[0,0,0,1,0,0,0,0,0,0,0,0,0,0],[0,0,0,1,0,0,0,0,0,0,0,0,0,0],[0,0,0,1,0,0,0,0,0,0,0,0,0,0],[0,0,0,1,0,0,0,0,0,0,0,0,0,0]]C3164531	distal origin of brachiocephalic artery with tracheal compression	[[1,0,0,0,0,0,0,0,0,0,0,0,0,0]]C0598866	chloranemia	[[0,0,0,0,0,0,0,0,0,1,0,0,0,0]]C0031351	pharyngo conjunctival fever,adenoviral pharyngoconjunctivitis,adenoviral pharygoconjunctival fever,pharyngoconjunctival fever	[[0,1,0,0,0,0,0,1,0,0,0,0,0,0],[1,0,0,0,0,0,0,0,0,0,0,0,0,0],[1,0,0,0,0,0,0,0,0,0,0,0,0,0],[0,0,0,0,1,0,0,1,0,0,1,1,0,0]]C0031350	pharyngitis,pharyngitides,sore throat,throat infection,pharyngeal inflammation,irritation of the throat,throat inflamed,throat inflammation	[[0,0,1,0,0,0,0,1,0,0,0,0,0,0],[0,1,0,0,0,0,0,0,0,0,0,0,0,0],[0,0,0,0,0,0,0,1,0,0,0,0,0,0],[0,0,0,0,0,0,0,1,0,0,0,0,0,0],[0,0,0,0,0,0,0,0,0,0,1,0,0,0],[0,0,0,0,0,0,0,1,0,0,0,0,0,0],[0,0,0,0,0,0,0,1,0,0,0,0,0,0],[0,0,0,0,0,0,0,1,0,0,0,0,0,0]]C0684884	neoplasm of uncertain behavior of lower limb,neoplasm of uncertain behaviour of lower limb	[[1,0,0,0,0,0,0,0,0,0,0,0,0,0],[1,0,0,0,0,0,0,0,0,0,0,0,0,0]]C1299539	infection of scrotum,scrotal infection	[[1,0,0,0,0,0,0,1,0,0,0,0,0,0],[0,0,0,0,0,0,0,1,0,0,0,1,0,0]]C0578505	acute viral laryngotracheitis	[[1,0,0,0,0,0,0,0,0,0,0,0,0,0]]C0030409	paracoccidioidomycosis,paracoccidioidomycoses,paracoccidiodomycosis,south american blastomycosis,paracoccidioidal granuloma,lutz splendore almeida disease,infection by blastomyces brasiliensis,brazilian blastomycosis,infection by paracoccidioides brasiliensis,mucocutaneous lymphangitic paracoccidioidomycosis,mucocutaneous south american blastomycosis,paracoccidioidal mycosis	[[0,0,0,0,0,0,0,1,0,0,1,0,0,0],[0,1,0,0,0,0,0,0,0,0,0,0,0,0],[0,0,0,0,0,0,0,0,0,1,0,0,0,0],[0,1,0,0,0,0,0,1,0,0,1,0,1,1],[1,0,0,0,0,0,0,0,0,0,1,0,0,0],[0,0,0,0,0,0,0,0,0,0,1,0,0,1],[0,0,0,0,0,0,0,0,0,0,0,0,0,1],[0,0,0,0,0,0,0,0,0,0,0,0,0,1],[0,0,0,0,0,0,0,0,0,0,0,0,0,1],[0,0,0,0,0,0,0,0,0,0,0,0,0,1],[1,0,0,0,0,0,0,0,0,0,0,0,0,0],[0,0,0,0,0,0,0,0,0,1,0,0,0,0]]C1839130	x linked torsion dystonia 3,x linked dystonia parkinsonism,xdp,dyt3,filipino type torsion dystonia parkinsonism	[[0,0,0,1,0,0,0,0,0,0,0,0,0,0],[0,0,0,1,0,0,0,0,0,0,0,0,0,0],[0,0,0,1,0,0,0,0,0,0,0,0,0,0],[0,0,0,1,0,0,0,0,0,0,0,0,0,0],[0,0,0,1,0,0,0,0,0,0,0,0,0,0]]C0010356	cross infection	[[0,1,0,0,0,0,0,1,1,0,0,0,1,0]]C2673498	female luteinizing hormone resistance	[[0,0,0,1,0,0,1,0,0,0,0,0,0,0]]C1828221	non dystrophic myotonia	[[1,0,0,0,0,0,0,0,0,0,0,0,0,0]]C0571622	insulin allergy	[[1,0,0,0,0,0,0,1,0,0,0,0,0,0]]C0571623	soluble neutral insulin allergy	[[1,0,0,0,0,0,0,0,0,0,0,0,0,0]]C0571620	budesonide allergy	[[1,0,0,0,0,0,0,0,0,0,0,0,0,0]]C0571621	topical budesonide allergy	[[1,0,0,0,0,0,0,0,0,0,0,0,0,0]]C0571626	insulin zinc suspension amorphous allergy	[[1,0,0,0,0,0,0,0,0,0,0,0,0,0]]C0571627	insulin zinc suspension crystalline allergy	[[1,0,0,0,0,0,0,0,0,0,0,0,0,0]]C0571624	biphasic insulin allergy	[[1,0,0,0,0,0,0,0,0,0,0,0,0,0]]C0571625	insulin zinc suspension allergy	[[1,0,0,0,0,0,0,0,0,0,0,0,0,0]]C0571628	isophane insulin allergy	[[1,0,0,0,0,0,0,0,0,0,0,0,0,0]]C0571629	protamine zinc insulin allergy	[[1,0,0,0,0,0,0,0,0,0,0,0,0,0]]C1963651	renal fistula	[[0,0,0,0,0,0,1,0,0,0,0,0,0,0]]C0155658	subsequent episode of care subendocardial infarction acute myocardial infarction	[[0,0,0,0,1,0,0,0,0,0,0,0,0,0]]C0155653	initial episode of care true posterior wall infarction acute myocardial infarction	[[0,0,0,0,1,0,0,0,0,0,0,0,0,0]]C0155655	subendocardial infarction acute myocardial infarction	[[0,0,0,0,1,0,0,0,0,0,0,0,0,0]]C0155654	subsequent episode of care true posterior wall infarction acute myocardial infarction	[[0,0,0,0,1,0,0,0,0,0,0,0,0,0]]C0155657	initial episode of care subendocardial infarction acute myocardial infarction	[[0,0,0,0,1,0,0,0,0,0,0,0,0,0]]C1863235	hemolytic anemia due to elevated adenosine deaminase	[[0,0,0,1,0,0,0,0,0,0,0,0,0,0]]C0748731	viral sinusitis	[[1,0,0,0,0,0,0,0,0,0,0,0,0,0]]C2675517	quantitative trait locus 1 serum level of adiponectin,adipqtl1	[[0,0,0,1,0,0,0,0,0,0,0,0,0,0],[0,0,0,1,0,0,0,0,0,0,0,0,0,0]]C1969842	fetal hemoglobin quantitative trait locus 4,hbfqtl4	[[0,0,0,1,0,0,0,0,0,0,0,0,0,0],[0,0,0,1,0,0,0,0,0,0,0,0,0,0]]C1387797	angiostrongylus costaricensis infection	[[0,0,0,0,0,0,1,0,0,0,0,0,0,0]]C0570790	ethosuximide allergy	[[1,0,0,0,0,0,0,0,0,0,0,0,0,0]]C0861571	stage 0 oral cavity squamous cell carcinoma,stage 0 oral cavity epidermoid carcinoma,stage 0 squamous cell carcinoma of the mouth,stage 0 mouth epidermoid carcinoma,oral cavity squamous cell carcinoma in situ,oral cavity epidermoid carcinoma in situ,squamous cell carcinoma in situ of the mouth,mouth epidermoid carcinoma in situ	[[0,0,0,0,0,0,0,0,0,0,0,1,0,0],[0,0,0,0,0,0,0,0,0,0,0,1,0,0],[0,0,0,0,0,0,0,0,0,0,0,1,0,0],[0,0,0,0,0,0,0,0,0,0,0,1,0,0],[0,0,0,0,0,0,0,0,0,0,0,1,0,0],[0,0,0,0,0,0,0,0,0,0,0,1,0,0],[0,0,0,0,0,0,0,0,0,0,0,1,0,0],[0,0,0,0,0,0,0,0,0,0,0,1,0,0]]C0861572	stage 0 oral cavity verrucous carcinoma,stage 0 verrucous carcinoma of the mouth,verrucous carcinoma in situ of the oral cavity,verrucous carcinoma in situ of the mouth	[[0,0,0,0,0,0,0,0,0,0,0,1,0,0],[0,0,0,0,0,0,0,0,0,0,0,1,0,0],[0,0,0,0,0,0,0,0,0,0,0,1,0,0],[0,0,0,0,0,0,0,0,0,0,0,1,0,0]]C0342199	iodine deficiency syndrome,iodine deficiency,iodine deficiency disorder	[[1,0,0,0,0,0,0,0,0,0,0,0,0,0],[0,0,0,0,0,0,0,1,0,0,0,0,0,1],[0,0,0,0,0,0,0,1,0,0,0,0,0,0]]C0428458	uric acid bladder stone	[[1,0,0,0,0,0,0,0,0,0,0,0,0,0]]C0428459	phosphate bladder stone	[[1,0,0,0,0,0,0,0,0,0,0,0,0,0]]C1852292	with ear malformation and facial palsy conductive stapedial deafness	[[0,0,0,1,0,0,0,0,0,0,0,0,0,0]]C1863870	and mixed type hearing loss upper lid coloboma hypertelorism hypospadias	[[0,0,0,1,0,0,0,0,0,0,0,0,0,0]]C0571012	carbocisteine allergy	[[1,0,0,0,0,0,0,0,0,0,0,0,0,0]]C0393440	granulomatous meningoencephalitis,chronic granulomatous meningitis	[[1,0,0,0,0,0,0,0,0,0,0,0,0,0],[1,0,0,0,0,0,0,0,0,0,0,0,0,0]]C0393441	chronic lymphocytic meningitis	[[0,0,0,1,0,0,0,0,0,0,0,0,0,0]]C0393442	non infective meningitis	[[1,0,0,0,0,0,0,0,0,0,0,0,0,0]]C0472700	non anemic red cell disorder,non anaemic red cell disorder	[[1,0,0,0,0,0,0,0,0,0,0,0,0,0],[1,0,0,0,0,0,0,0,0,0,0,0,0,0]]C0393444	chemical meningitis	[[1,0,0,0,0,0,0,1,0,0,0,0,0,0]]C0268335	ehlers danlos syndrome type 1,ehlers danlos syndrome type i,severe classic form ehlers danlos syndrome,gravis ehlers danlos syndrome,gravis type ehlers danlos syndrome,eds i,eds1,severe classic type ehlers danlos syndrome	[[1,1,0,0,0,0,0,0,0,0,0,0,0,0],[1,0,0,1,0,0,0,0,0,0,0,0,0,0],[1,1,0,0,0,0,0,0,0,0,0,0,0,0],[1,0,0,0,0,0,0,0,0,0,0,0,0,0],[0,1,0,1,0,0,0,0,0,0,0,0,0,0],[0,0,0,1,0,0,0,0,0,0,0,0,0,0],[0,0,0,1,0,0,0,0,0,0,0,0,0,0],[0,0,0,1,0,0,0,0,0,0,0,0,0,0]]C0393446	bacterial ventriculitis,brain bacterial ventriculitis	[[1,0,0,0,0,0,0,0,0,0,0,0,0,0],[1,0,0,0,0,0,0,0,0,0,0,0,0,0]]C0393447	fungal ventriculitis,brain fungal ventriculitis	[[1,0,0,0,0,0,0,0,0,0,0,0,0,0],[1,0,0,0,0,0,0,0,0,0,0,0,0,0]]C0393448	malignant ventriculitis,brain malignant ventriculitis	[[1,0,0,0,0,0,0,0,0,0,0,0,0,0],[1,0,0,0,0,0,0,0,0,0,0,0,0,0]]C0570799	barbiturate sedative allergy	[[1,0,0,0,0,0,0,0,0,0,0,0,0,0]]C1515024	submucosal invasive colon adenocarcinoma	[[0,0,0,0,0,0,0,0,0,0,0,1,0,0]]C0570798	paraldehyde allergy	[[1,0,0,0,0,0,0,0,0,0,0,0,0,0]]C2712360	severe hypoxic ischemic encephalopathy	[[0,0,0,0,1,0,0,0,0,0,0,0,0,0]]C2827503	hfe associated hereditary hemochromatosis	[[0,0,0,0,0,0,0,0,0,0,0,1,0,0]]C0027809	neurilemmoma,neurinoma,schwannoma,neurilemoma,benign neurilemmoma,benign schwannoma,ancient schwannoma,psammomatous schwannoma	[[0,1,0,0,0,0,0,1,0,0,0,0,0,0],[0,1,0,0,0,0,0,1,0,0,0,0,1,0],[0,1,0,1,0,0,0,1,0,1,0,1,0,0],[0,1,0,0,0,0,0,1,0,0,0,0,1,0],[0,0,0,0,0,0,0,0,0,0,0,1,0,0],[0,0,0,0,0,0,0,0,0,0,0,1,0,0],[0,0,0,0,0,0,0,1,0,0,0,0,0,0],[1,0,0,0,0,0,0,0,0,0,0,0,0,0]]C0341258	perforated diverticulum of large intestine	[[1,0,0,0,0,0,0,0,0,0,0,0,0,0]]C0341259	diverticulitis of colon with perforation,perforated diverticulum of colon	[[1,0,0,0,0,0,0,0,0,0,0,0,0,0],[1,0,0,0,0,0,0,0,0,0,0,0,0,0]]C0341257	perforated diverticulum,ruptured diverticulum	[[1,0,0,0,0,0,0,1,0,0,0,0,0,0],[0,0,0,0,0,0,0,1,0,0,0,0,0,0]]C0341253	brunners gland hyperplasia,hyperplasia of brunner glands of duodenum	[[1,0,0,0,0,0,0,0,0,0,0,0,0,0],[1,0,0,0,0,0,0,0,0,0,0,0,0,0]]C0341250	duodenoenteric fistula	[[1,0,0,0,0,0,0,0,0,0,0,0,0,0]]C0341251	traumatic perforation of duodenum	[[1,0,0,0,0,0,0,0,0,0,0,0,0,0]]C0342193	hypothyroidism due to iodide organification defect	[[1,0,0,0,0,0,0,0,0,0,0,0,0,0]]C0391970	malignant carcinoid tumor,malignant carcinoid tumour,carcinoid malignant	[[1,0,0,0,0,0,1,1,0,0,0,0,0,0],[1,0,0,0,0,0,0,0,0,0,0,0,0,0],[0,0,0,0,0,0,0,1,0,0,0,0,0,0]]C2712364	failure to thrive in newborn	[[0,0,0,0,1,0,0,0,0,0,0,0,0,0]]C0400807	stress ulcer of stomach,stress ulcer,acute gastric erosion associated with severe burns,curlings ulcer of stomach,gastric stress ulcer	[[1,0,0,0,0,0,0,1,0,0,0,0,0,0],[0,0,0,0,0,0,0,1,0,0,0,0,0,0],[1,0,0,0,0,0,0,0,0,0,0,0,0,0],[1,0,0,0,0,0,0,0,0,0,0,0,0,0],[1,0,0,0,0,0,0,0,0,0,0,1,0,0]]C0743381	eclamptic seizure,eclamptic fit	[[1,0,0,0,0,0,0,0,0,0,0,0,0,0],[1,0,0,0,0,0,0,0,0,0,0,0,0,0]]C0684902	eosinophilic non allergic rhinitis,nares non allergic rhinitis with eosinophilia,non allergic rhinitis with eosinophilia,eosinophilic nonallergic rhinitis	[[1,0,0,0,0,0,0,0,0,0,0,0,0,0],[1,0,0,0,0,0,0,0,0,0,0,0,0,0],[1,0,0,0,0,0,0,0,0,0,0,0,0,0],[1,0,0,0,0,0,0,0,0,0,0,0,0,0]]C0684903	perforation of pharynx,pharyngeal perforation	[[1,0,0,0,0,0,0,0,0,0,0,0,0,0],[1,0,0,0,0,0,0,0,0,0,0,0,0,0]]C0684900	allergic rhinitis due to weed pollens	[[1,0,0,0,0,0,0,0,0,0,0,0,0,0]]C0684901	allergic rhinitis due to animals	[[1,0,0,0,0,0,0,0,0,0,0,0,0,0]]C0684906	perforation of pharyngeal diverticulum	[[1,0,0,0,0,0,0,0,0,0,0,0,0,0]]C0684907	perforation of trachea,tracheal perforation	[[1,0,0,0,0,0,0,1,0,0,0,0,0,0],[1,0,0,0,0,0,0,1,0,0,0,0,0,0]]C2347748	adult erythroleukemia	[[0,0,0,0,0,0,0,0,0,0,0,1,0,0]]C0855035	recurrent malignant fibrous histiocytoma,relapsed malignant fibrous histiocytoma	[[0,0,0,0,0,0,0,0,0,0,0,1,0,0],[0,0,0,0,0,0,0,0,0,0,0,1,0,0]]C0282526	hyperpipecolic acidemia,hyperpipecolic acidaemia,hyperpipecolatemia	[[1,1,0,1,0,0,0,0,0,0,0,0,0,0],[1,0,0,0,0,0,0,0,0,0,0,0,0,0],[0,1,0,1,0,0,0,0,0,0,0,0,0,0]]C0282527	infantile refsum disease,infantile phytanic acid storage disease,infantile form refsum disease,ird,infantile form of phytanic acid storage disease	[[1,1,0,0,0,0,1,1,0,0,0,1,1,0],[0,0,0,1,0,0,0,0,0,0,0,0,1,0],[0,0,0,1,0,0,0,0,0,0,0,0,1,0],[0,0,0,1,0,0,0,0,0,0,0,0,0,0],[0,0,0,0,0,0,0,0,0,0,0,0,1,0]]C0282525	neonatal adrenoleukodystrophy,neonatal adrenoleucodystrophy,neonatal form autosomal adrenoleukodystrophy,nald	[[0,1,0,1,0,0,0,1,0,0,0,0,0,1],[1,0,0,0,0,0,0,0,0,0,0,0,0,0],[0,1,0,1,0,0,0,0,0,0,0,0,0,0],[0,0,0,1,0,0,0,0,0,0,0,0,0,0]]C1842704	perinatal lethal gaucher disease,collodion type gaucher disease	[[0,0,0,1,0,0,0,0,0,0,0,0,0,0],[0,0,0,1,0,0,0,0,0,0,0,0,0,0]]C0282528	peroxisomal disorders,disorder of peroxisomal function	[[0,1,0,0,1,0,0,1,0,0,0,0,1,0],[1,0,0,0,0,0,0,0,0,0,0,0,0,0]]C0282529	rhizomelic chondrodysplasia punctata,rhizomelic chondrodysplasia punctata syndrome,rhizomelic type chondrodysplasia punctata,autosomal recessive type chondrodysplasia punctata,rcdp rhizomelic chondrodysplasia punctata,rhizomelic chrondrodysplasia punctata,rhizomelic form chondrodysplasia punctata	[[1,1,0,0,0,0,0,0,0,0,0,1,0,0],[1,0,0,0,0,0,0,0,0,0,0,0,0,0],[1,0,0,0,0,0,0,0,0,0,0,0,0,0],[1,0,0,0,0,0,0,0,0,0,0,0,0,0],[1,0,0,0,0,0,0,0,0,0,0,0,0,0],[0,0,0,0,0,0,0,0,0,0,0,0,0,1],[0,0,0,0,0,0,0,0,0,0,0,0,1,0]]C0586372	moderate dysplasia of rectum,moderate rectal dysplasia	[[1,0,0,0,0,0,0,0,0,0,0,1,0,0],[0,0,0,0,0,0,0,0,0,0,0,1,0,0]]C1332135	achard syndrome,dysostoses and increased ligament laxity,and joint laxity limited to the hands and feet receding lower jaw arachnodactyly	[[0,1,0,1,0,0,0,0,0,0,0,1,0,0],[0,1,0,0,0,0,0,0,0,0,0,0,0,0],[0,1,0,0,0,0,0,0,0,0,0,0,0,0]]C0586370	dysplasia of rectum,rectal dysplasia	[[1,0,0,0,0,0,0,0,0,0,0,1,0,0],[0,0,0,0,0,0,0,0,0,0,0,1,0,0]]C0586371	mild dysplasia of rectum,mild rectal dysplasia	[[1,0,0,0,0,0,0,0,0,0,0,1,0,0],[0,0,0,0,0,0,0,0,0,0,0,1,0,0]]C0021897	intrathoracic goiters	[[0,1,0,0,0,0,0,0,0,0,1,0,0,0]]C0586374	bleeding stress ulcer of stomach	[[1,0,0,0,0,0,0,0,0,0,0,0,0,0]]C1332133	abstinence syndrome	[[0,0,0,0,0,0,0,0,0,0,0,1,0,0]]C2675512	diamond blackfan anemia 7,dba7	[[0,0,0,1,0,0,0,0,0,0,0,0,0,0],[0,0,0,1,0,0,0,0,0,0,0,0,0,0]]C0855031	stage ii superficial spreading melanoma	[[0,0,0,0,0,0,0,0,0,0,0,1,0,0]]C1332139	acinar prostate adenocarcinoma	[[0,0,0,0,0,0,0,0,0,0,0,1,0,0]]C1319118	colovascular fistula,colo vascular fistula	[[1,0,0,0,0,0,0,0,0,0,0,0,0,0],[1,0,0,0,0,0,0,0,0,0,0,0,0,0]]C0338113	uterine corpus sarcoma,uterine sarcoma,sarcoma of uterus,sarcoma uterine cancer,sarcoma uterine corpus cancer,sarcoma uterus cancer,uterine body sarcoma,body of uterus sarcoma,sarcoma of the corpus uteri	[[0,0,0,0,0,0,0,0,0,0,0,1,0,0],[0,0,1,0,0,1,0,1,0,0,0,0,0,0],[1,0,0,0,0,0,0,1,0,0,0,1,0,0],[0,0,0,0,0,1,0,1,0,0,0,0,0,0],[0,0,0,0,0,1,0,0,0,0,0,0,0,0],[0,0,0,0,0,1,0,0,0,0,0,0,0,0],[0,0,0,0,0,0,0,0,0,0,0,1,0,0],[0,0,0,0,0,0,0,0,0,0,0,1,0,0],[0,0,0,0,0,0,0,0,0,0,0,1,0,0]]C1533674	borderline glaucoma	[[1,0,0,0,1,0,0,1,0,0,0,0,0,0]]C1533675	disorders of optic nerve and visual pathways	[[1,0,0,0,1,0,0,0,0,0,0,0,0,0]]C2750460	modifier of renal tsc2 angiomyolipomas	[[0,0,0,1,0,0,0,0,0,0,0,0,0,0]]C1707302	cardiac synovial sarcoma	[[0,0,0,0,0,0,0,0,0,0,0,1,0,0]]C1707301	cardiac monophasic synovial sarcoma	[[0,0,0,0,0,0,0,0,0,0,0,1,0,0]]C0279832	recurrent paranasal sinus and nasal cavity cancer	[[0,0,0,0,0,1,0,0,0,0,0,1,0,0]]C0279830	stage iii paranasal sinus and nasal cavity cancer,stage iii nasal cavity and paranasal sinus cancer ajcc v7,stage iii nasal cavity and paranasal sinus cancer ajcc v6	[[0,0,0,0,0,1,0,0,0,0,0,1,0,0],[0,0,0,0,0,0,0,0,0,0,0,1,0,0],[0,0,0,0,0,0,0,0,0,0,0,1,0,0]]C0279831	stage iv paranasal sinus and nasal cavity cancer,metastatic paranasal sinus and nasal cavity cancer,stage iv nasal cavity and paranasal sinus cancer ajcc v7	[[0,0,0,0,0,1,0,0,0,0,0,1,0,0],[0,0,0,0,0,1,0,0,0,0,0,0,0,0],[0,0,0,0,0,0,0,0,0,0,0,1,0,0]]C0279836	stage iii oropharynx carcinoma,stage iii oropharyngeal cancer,stage iii oropharynx cancer,stage iii carcinoma of oropharynx ajcc v6,stage iii ajcc v6 oropharyngeal cancer,stage iii oropharyngeal carcinoma ajcc v6	[[0,0,0,0,0,0,1,0,0,0,0,0,0,0],[0,0,0,0,0,1,0,0,0,0,0,0,0,0],[0,0,0,0,0,1,0,0,0,0,0,1,0,0],[0,0,0,0,0,0,0,0,0,0,0,1,0,0],[0,0,0,0,0,0,0,0,0,0,0,1,0,0],[0,0,0,0,0,0,0,0,0,0,0,1,0,0]]C0855038	recurrent alveolar soft part sarcoma,relapsed alveolar soft part sarcoma	[[0,0,0,0,0,0,0,0,0,0,0,1,0,0],[0,0,0,0,0,0,0,0,0,0,0,1,0,0]]C0279834	stage i oropharyngeal carcinoma,stage i oropharyngeal cancer,stage i oropharynx cancer,stage i oropharynx carcinoma,stage i oropharyngeal carcinoma ajcc v6,stage i oropharyngeal carcinoma ajcc v7	[[0,0,0,0,0,0,0,0,0,0,0,1,0,0],[0,0,0,0,0,1,0,0,0,0,0,1,0,0],[0,0,0,0,0,1,0,0,0,0,0,0,0,0],[0,0,0,0,0,0,0,0,0,0,0,1,0,0],[0,0,0,0,0,0,0,0,0,0,0,1,0,0],[0,0,0,0,0,0,0,0,0,0,0,1,0,0]]C0279835	stage ii oropharyngeal carcinoma,stage ii oropharyngeal cancer,stage ii oropharynx cancer,stage ii oropharynx carcinoma,stage ii oropharyngeal carcinoma ajcc v6,stage ii oropharyngeal carcinoma ajcc v7	[[0,0,0,0,0,0,0,0,0,0,0,1,0,0],[0,0,0,0,0,1,0,0,0,0,0,1,0,0],[0,0,0,0,0,1,0,0,0,0,0,0,0,0],[0,0,0,0,0,0,0,0,0,0,0,1,0,0],[0,0,0,0,0,0,0,0,0,0,0,1,0,0],[0,0,0,0,0,0,0,0,0,0,0,1,0,0]]C0279838	recurrent oropharyngeal cancer,recurrent oropharynx cancer,relapsed cancer of the oropharynx,relapsed oropharyngeal cancer,recurrent oropharynx carcinoma,relapsed oropharynx carcinoma,relapsed oropharyngeal carcinoma	[[0,0,0,0,0,1,0,0,0,0,0,1,0,0],[0,0,0,0,0,1,0,0,0,0,0,1,0,0],[0,0,0,0,0,0,0,0,0,0,0,1,0,0],[0,0,0,0,0,0,0,0,0,0,0,1,0,0],[0,0,0,0,0,0,0,0,0,0,0,1,0,0],[0,0,0,0,0,0,0,0,0,0,0,1,0,0],[0,0,0,0,0,0,0,0,0,0,0,1,0,0]]C0855039	metastatic epithelioid sarcoma,metastatic epithelioid cell sarcoma	[[0,0,0,0,0,0,0,0,0,0,0,1,0,0],[0,0,0,0,0,0,0,0,0,0,0,1,0,0]]C0153161	syphilitic endocarditis of aortic valve	[[0,0,0,0,1,0,0,0,0,0,0,0,0,0]]C0153160	syphilitic endocarditis of mitral valve	[[0,0,0,0,1,0,0,0,0,0,0,0,0,0]]C0153163	syphilitic endocarditis of pulmonary valve	[[0,0,0,0,1,0,0,0,0,0,0,0,0,0]]C0153162	syphilitic endocarditis of tricuspid valve	[[0,0,0,0,1,0,0,0,0,0,0,0,0,0]]C0153165	syphilitic myocarditis	[[0,0,0,0,1,0,0,0,0,0,0,0,0,0]]C0153164	syphilitic pericarditis	[[0,0,0,0,1,0,0,0,0,0,0,0,0,0]]C0153167	asymptomatic neurosyphilis	[[0,1,0,0,1,0,0,0,0,0,0,0,0,0]]C0153166	syphilitic aseptic meningitis,syphilitic meningitis,meningeal syphilis	[[0,1,0,0,0,0,0,0,0,0,0,0,0,0],[0,1,0,0,0,0,0,0,0,0,0,0,0,0],[0,1,0,0,0,0,0,0,0,0,0,0,0,0]]C0153169	syphilitic parkinsonism	[[1,0,0,0,1,0,0,0,0,0,0,0,0,0]]C0153168	syphilitic encephalitis	[[0,0,0,0,1,0,0,0,0,0,0,0,0,0]]C1518950	penile urethral malignant neoplasm	[[0,0,0,0,0,0,0,0,0,0,0,1,0,0]]C2712931	any site secondary merkel cell carcinoma	[[0,0,0,0,0,0,0,0,0,0,0,0,0,1]]C2931129	ellis yale winter syndrome	[[0,1,0,0,0,0,0,0,0,0,0,0,0,0]]C1997809	primary viral encephalitis	[[1,0,0,0,0,0,0,0,0,0,0,0,0,0]]C1859062	long qt syndrome 3,lqt3	[[0,0,0,1,0,0,0,0,0,0,0,0,0,0],[0,0,0,1,0,0,0,0,0,0,0,0,0,0]]C1969653	mungan syndrome,mgs,with barrett esophagus and cardiac abnormalities chronic idiopathic intestinal pseudoobstruction,and cardiac abnormalities barrett esophagus megaduodenum with pseudoobstruction familial visceral neuromyopathy	[[0,1,0,1,0,0,0,0,0,0,0,0,0,0],[0,0,0,1,0,0,0,0,0,0,0,0,0,0],[0,0,0,1,0,0,0,0,0,0,0,0,0,0],[0,0,0,1,0,0,0,0,0,0,0,0,0,0]]C1969652	type b2 brachydactyly,bdb2	[[0,0,0,1,0,0,1,0,0,0,0,0,0,0],[0,0,0,1,0,0,0,0,0,0,0,0,0,0]]C1969651	9 age related macular degeneration,armd9	[[0,0,0,1,0,0,0,0,0,0,0,0,0,0],[0,0,0,1,0,0,0,0,0,0,0,0,0,0]]C0262469	embolic stroke,embolic apoplexy	[[1,0,0,0,0,0,0,1,0,0,1,0,0,0],[0,0,0,0,0,0,0,0,0,0,1,0,0,0]]C1969656	4 susceptibility to juvenile myoclonic epilepsy,ejm4	[[0,0,0,1,0,0,1,0,0,0,0,0,0,0],[0,0,0,1,0,0,0,0,0,0,0,0,0,0]]C1969655	lethal congenital contractural syndrome 3,lccs3,israeli bedouin type b multiple contractural syndrome	[[0,0,0,1,0,0,0,0,0,0,0,0,0,0],[0,0,0,1,0,0,0,0,0,0,0,0,0,0],[0,0,0,1,0,0,0,0,0,0,0,0,0,0]]C1443900	inhalational botulism	[[1,0,0,0,0,0,0,0,0,0,0,0,0,0]]C1443901	intestinal botulism	[[1,0,0,0,0,0,0,0,0,0,0,0,0,0]]C1519076	physiological diffuse hyperplasia of the mouse prostate gland	[[0,0,0,0,0,0,0,0,0,0,0,1,0,0]]C1519077	physiological focal hyperplasia of the mouse prostate gland	[[0,0,0,0,0,0,0,0,0,0,0,1,0,0]]C2751535	platelet prostaglandin endoperoxide synthase 1 deficiency,pghs1 deficiency,platelet cox1 deficiency,platelet cyclooxygenase 1 deficiency	[[0,0,0,1,0,0,0,0,0,0,0,0,0,0],[0,0,0,1,0,0,0,0,0,0,0,0,0,0],[0,0,0,1,0,0,0,0,0,0,0,0,0,0],[0,0,0,1,0,0,0,0,0,0,0,0,0,0]]C2751536	app related cerebral amyloid angiopathy,flemish variant app related cerebral amyloid angiopathy,iowa variant app related cerebral amyloid angiopathy,arctic variant app related cerebral amyloid angiopathy,app related cerebroarterial amyloidosis,italian variant app related cerebral amyloid angiopathy	[[0,0,0,1,0,0,0,0,0,0,0,0,0,0],[0,0,0,1,0,0,0,0,0,0,0,0,0,0],[0,0,0,1,0,0,0,0,0,0,0,0,0,0],[0,0,0,1,0,0,0,0,0,0,0,0,0,0],[0,0,0,1,0,0,0,0,0,0,0,0,0,0],[0,0,0,1,0,0,0,0,0,0,0,0,0,0]]C1868512	autosomal dominant adult onset demyelinating leukodystrophy,adld,autosomal dominant or late onset type pelizaeus merzbacher disease,multiple sclerosis like disorder	[[0,0,0,1,0,0,0,0,0,0,0,0,0,0],[0,0,0,1,0,0,0,0,0,0,0,0,0,0],[0,0,0,1,0,0,0,0,0,0,0,0,0,0],[0,0,0,1,0,0,0,0,0,0,0,0,0,0]]C0395903	acute mastoiditis with labyrinthitis	[[1,0,0,0,0,0,0,0,0,0,0,0,0,0]]C0395902	acute mastoiditis with facial paralysis,acute mastoiditis and facial palsy	[[1,0,0,0,0,0,0,0,0,0,0,0,0,0],[1,0,0,0,0,0,0,0,0,0,0,0,0,0]]C0395901	acute mastoiditis with complication	[[1,0,0,0,0,0,0,0,0,0,0,0,0,0]]C0395904	acute mastoiditis with intracranial complication	[[1,0,0,0,0,0,0,0,0,0,0,0,0,0]]C0339247	stromal pigmentation	[[1,0,0,0,0,0,0,0,0,0,0,0,0,0]]C2931241	waldmann disease,primary intestinal lymphangiectasis	[[0,1,0,0,0,0,0,0,0,0,0,0,0,0],[0,1,0,0,0,0,0,0,0,0,0,0,0,0]]C2350476	orbital myositis,orbital myositides	[[0,1,0,0,1,0,0,1,0,0,0,0,1,0],[0,1,0,0,0,0,0,0,0,0,0,0,0,0]]C1854311	3 posterior polar cataract,ctpp3,cpp3	[[0,1,0,1,0,0,0,0,0,0,0,0,0,0],[0,0,0,1,0,0,0,0,0,0,0,0,0,0],[0,0,0,1,0,0,0,0,0,0,0,0,0,0]]C1854310	hypotrichosis simplex,hts,hhs,hereditary hypotrichosis simplex,hereditary generalized hypotrichosis simplex	[[0,1,0,0,0,0,0,0,0,0,0,0,0,0],[0,0,0,1,0,0,0,0,0,0,0,0,0,0],[0,0,0,1,0,0,0,0,0,0,0,0,0,0],[0,1,0,0,0,0,0,0,0,0,0,0,0,0],[0,0,0,1,0,0,0,0,0,0,0,0,0,0]]C0570999	ipratropium allergy	[[1,0,0,0,0,0,0,0,0,0,0,0,0,0]]C0570998	homatropine allergy	[[1,0,0,0,0,0,0,0,0,0,0,0,0,0]]C0570997	glycopyrronium allergy	[[1,0,0,0,0,0,0,0,0,0,0,0,0,0]]C0570996	cyclopentolate allergy	[[1,0,0,0,0,0,0,0,0,0,0,0,0,0]]C0013076	dourine,mal du coit,infection by trypanosoma equiperdum,mal de coit,covering disease	[[0,1,0,0,0,0,0,0,0,0,0,0,0,0],[1,0,0,0,0,0,0,0,0,0,0,0,0,0],[1,0,0,0,0,0,0,0,0,0,0,0,0,0],[0,0,0,0,0,0,1,0,0,0,0,0,0,0],[0,0,0,0,0,0,1,0,0,0,0,0,0,0]]C0570994	trihexyphenidyl allergy,benzhexol allergy	[[1,0,0,0,0,0,0,0,0,0,0,0,0,0],[1,0,0,0,0,0,0,0,0,0,0,0,0,0]]C0570993	atropine allergy	[[1,0,0,0,0,0,0,0,0,0,0,0,0,0]]C0570992	hyoscine hydrobromide allergy	[[1,0,0,0,0,0,0,0,0,0,0,0,0,0]]C0570991	hyoscine allergy	[[1,0,0,0,0,0,0,0,0,0,0,0,0,0]]C0570990	tropicamide allergy	[[1,0,0,0,0,0,0,0,0,0,0,0,0,0]]C1855675	arima syndrome,chorioretinal coloboma with cerebellar vermis aplasia,cerebro oculo hepato renal syndrome,joubert syndrome with bilateral chorioretinal coloboma,dekaban arima syndrome,cerebrooculohepatorenal syndrome	[[0,1,0,1,0,0,0,0,0,0,0,0,0,0],[0,1,0,1,0,0,0,0,0,0,0,0,0,0],[0,1,0,0,0,0,0,0,0,0,0,0,0,0],[0,1,0,1,0,0,0,0,0,0,0,0,0,0],[0,1,0,1,0,0,0,0,0,0,0,0,0,0],[0,0,0,1,0,0,0,0,0,0,0,0,0,0]]C1290838	acute heart disease	[[1,0,0,0,0,0,0,0,0,0,0,0,0,0]]C1290839	disorder of skin and or subcutaneous tissue of flank	[[1,0,0,0,0,0,0,0,0,0,0,0,0,0]]C2983009	stage ii uterine corpus cancer,stage ii uterine corpus cancer ajcc v7	[[0,0,0,0,0,0,0,0,0,0,0,1,0,0],[0,0,0,0,0,0,0,0,0,0,0,1,0,0]]C1290832	melanoma in situ by body site	[[1,0,0,0,0,0,0,0,0,0,0,0,0,0]]C1290833	neoplasm by body site	[[1,0,0,0,0,0,0,0,0,0,0,0,0,0]]C0588980	open flail chest	[[1,0,0,0,0,0,0,0,0,0,0,0,0,0]]C1290836	acute disease of bone	[[1,0,0,0,0,0,0,0,0,0,0,0,0,0]]C1290837	disorder of trunk	[[1,0,0,0,0,0,0,0,0,0,0,0,0,0]]C1290834	acute skin disorder	[[1,0,0,0,0,0,0,0,0,0,0,0,0,0]]C1290835	acute disorder of female genital organ,acute disease of female genital system,acute disorder of female genital system	[[1,0,0,0,0,0,0,0,0,0,0,0,0,0],[1,0,0,0,0,0,0,0,0,0,0,0,0,0],[1,0,0,0,0,0,0,0,0,0,0,0,0,0]]C1859161	and visual disturbance ataxia cholestasis with gallstone	[[0,0,0,1,0,0,0,0,0,0,0,0,0,0]]C0018916	hemangioma,haemangioma,angioma,any site hemangioma,benign angioma,benign hemangioma,benign haemangioma,hemangioma morphology,haemangioma morphology,no icd o subtype hemangioma	[[0,1,0,0,0,0,0,1,1,1,0,0,0,0],[1,0,0,0,0,0,0,1,0,0,0,0,0,0],[0,0,0,0,0,0,0,1,1,0,0,1,0,0],[0,0,0,0,1,0,0,0,0,0,0,0,0,0],[0,0,0,0,0,0,0,0,0,0,0,1,0,0],[1,0,0,0,0,0,0,1,0,0,0,1,0,0],[1,0,0,0,0,0,0,0,0,0,0,0,0,0],[1,0,0,0,0,0,0,0,0,0,0,0,0,0],[1,0,0,0,0,0,0,0,0,0,0,0,0,0],[1,0,0,0,0,0,0,0,0,0,0,0,0,0]]C0023138	laurence moon syndrome,laurence moon biedl syndrome,lawrence moon biedl syndrome	[[1,1,0,0,0,0,0,1,0,0,1,0,0,0],[0,1,0,0,0,0,0,1,0,1,1,0,1,0],[0,0,1,0,0,0,0,1,0,0,0,0,0,0]]C0018915	hemangioendothelioma,haemangioendothelioma,angioendothelioma,m hemangioendothelioma,m haemangioendothelioma,vascular endothelioma,hemangio endothelioma	[[0,1,0,0,0,0,0,1,0,1,0,0,1,0],[1,0,0,0,0,0,0,1,0,0,0,0,0,0],[0,0,0,0,0,0,0,0,0,0,0,1,0,0],[1,0,0,0,0,0,0,0,0,0,0,0,0,0],[1,0,0,0,0,0,0,0,0,0,0,0,0,0],[0,1,0,0,0,0,0,0,0,1,0,0,1,0],[0,1,0,0,0,0,0,0,0,0,0,0,1,0]]C0432291	mandibuloacral dysostosis,craniomandibular dermatodysostosis,familial mandibuloacral dysplasia,mandibuloacral dysplasia,mada,associated with mandibuloacral dysplasia type a lipodystrophy,mandibuloacral dysplasia with type a lipodystrophy	[[1,0,0,0,0,0,0,0,0,0,0,0,0,0],[1,0,0,1,0,0,0,0,0,0,0,0,0,0],[1,0,0,0,0,0,0,0,0,0,0,0,0,0],[1,0,0,0,0,0,0,0,0,0,0,0,0,0],[0,0,0,1,0,0,0,0,0,0,0,0,0,0],[0,1,0,1,0,0,0,0,0,0,0,0,0,0],[0,1,0,1,0,0,0,0,0,0,0,0,0,0]]C0235320	hematemesis gastric ulcer	[[0,0,0,0,0,0,0,0,1,0,0,0,0,0]]C0279160	medullary ductal breast carcinoma with lymphocytic infiltrate	[[0,0,0,0,0,1,0,0,0,0,0,0,0,0]]C0235327	small intestinal gangrene	[[1,0,0,0,0,0,0,0,1,0,0,0,0,0]]C0856457	anal excoriation	[[0,0,0,0,0,0,1,0,0,0,0,0,0,0]]C0235325	gastric hemorrhage,gastric haemorrhage,hemorrhage stomach,gastric bleeding,gastrorrhagia	[[0,0,0,0,0,0,0,1,1,0,0,1,0,0],[1,0,0,0,0,0,0,0,0,0,0,0,0,0],[0,0,0,0,0,0,0,1,1,0,0,0,0,0],[1,0,0,0,0,0,0,1,1,0,0,0,0,0],[1,0,0,0,0,0,0,1,0,0,0,0,0,0]]C0856459	elbow ankylosis	[[0,0,0,1,0,0,0,0,0,0,0,0,0,0]]C0235328	obstruction of colon,occlusion of colon	[[1,0,1,0,0,0,0,1,1,0,0,1,0,0],[1,0,0,0,0,0,0,1,0,0,0,0,0,0]]C0235329	small bowel obstruction,obstruction small intestine,sbo small bowel obstruction	[[1,0,1,0,0,0,0,1,0,0,0,0,0,0],[0,0,0,0,0,0,0,1,1,0,0,1,0,0],[1,0,0,0,0,0,0,0,0,0,0,0,0,0]]C0559044	splinter eyelids	[[1,0,0,0,0,0,0,0,0,0,0,0,0,0]]C1274322	recurrent herpes simplex infection of perianal area	[[1,0,0,0,0,0,0,0,0,0,0,0,0,0]]C3161063	including hip squamous cell carcinoma of skin of lower limb	[[0,0,0,0,1,0,0,0,0,0,0,0,0,0]]C1512328	haemangioma of the mouse nervous system	[[0,0,0,0,0,0,0,0,0,0,0,1,0,0]]C1274323	recurrent genital herpes simplex,genital herpes recurrent,recurrent herpes genitalis	[[1,0,0,0,0,0,0,0,0,0,0,0,0,0],[0,0,0,0,0,0,0,1,0,0,0,0,0,0],[1,0,0,0,0,0,0,0,0,0,0,0,0,0]]C2349289	chronic in relapse monocytic leukemia	[[0,0,0,0,1,0,0,0,0,0,0,0,0,0]]C0263739	chronic polyarticular juvenile rheumatoid arthritis	[[1,0,0,0,0,0,0,0,0,0,0,0,0,0]]C0263736	enzootic polyarthritis in goats	[[1,0,0,0,0,0,0,0,0,0,0,0,0,0]]C0263737	uveitis rheumatoid arthritis syndrome	[[1,0,0,0,0,0,0,0,0,0,0,0,0,0]]C0263734	progressive feline polyarthritis	[[1,0,0,0,0,0,0,0,0,0,0,0,0,0]]C0263735	polyarthritis in greyhounds	[[1,0,0,0,0,0,0,0,0,0,0,0,0,0]]C0263733	idiopathic polyarthritis	[[1,0,0,0,0,0,0,0,0,0,0,0,0,0]]C0263730	arthropathy associated with a hypersensitivity reaction,arthropathy due to hypersensitivity reaction	[[1,0,0,0,1,0,0,0,0,0,0,0,0,0],[1,0,0,0,0,0,0,0,0,0,0,0,0,0]]C0263731	arthropathy associated with another systemic disease	[[1,0,0,0,0,0,0,0,0,0,0,0,0,0]]C0016514	foot and mouth disease,aphthous fever,epizootic aphthae,epizootic aphtae,epizootic stomatitis,aphthosis,aphthus fever,hoof and mouth disease	[[0,1,0,0,1,0,0,1,0,0,1,0,1,0],[0,0,0,0,0,0,0,1,0,0,1,0,0,1],[0,0,0,0,0,0,0,0,0,0,1,0,0,1],[1,0,0,0,0,0,0,0,0,0,0,0,0,0],[0,0,0,0,0,0,0,0,0,0,0,0,0,1],[0,0,0,0,0,0,0,1,0,0,0,0,0,0],[1,0,0,0,0,0,0,0,0,0,0,0,0,0],[1,0,0,0,0,0,0,1,0,0,0,0,0,0]]C0016510	foot diseases,disorder of foot	[[0,1,0,0,0,0,1,1,0,0,0,0,0,0],[1,0,0,0,0,0,0,0,0,0,0,0,0,0]]C0686230	carcinoma in situ of vas deferens	[[1,0,0,0,0,0,0,0,0,0,0,0,0,0]]C0016513	foot rot,bovine foot rot,foul in the foot,infectious pododermatitis,interdigital necrobacillosis	[[1,1,0,0,0,0,0,0,0,0,0,0,0,0],[1,0,0,0,0,0,0,0,0,0,0,0,0,0],[1,0,0,0,0,0,0,0,0,0,0,0,0,0],[1,0,0,0,0,0,0,0,0,0,0,0,0,0],[1,0,0,0,0,0,0,0,0,0,0,0,0,0]]C0686231	primary malignant neoplasm of vas deferens,malignant neoplasm of vas deferens	[[1,0,0,0,0,0,0,0,0,0,0,0,0,0],[0,0,0,0,0,0,0,0,0,0,0,0,0,1]]C2349381	uncontrolled secondary diabetes mellitus with renal manifestations	[[0,0,0,0,1,0,0,0,0,0,0,0,0,0]]C2349994	tinea barbae,folliculitis barbae,not parasitic sycosis barbae,barbers itch,tinea sycosis,sycosis vulgaris,sycosis barbae,barbers rash,dermatophytosis of beard,mycotic sycosis	[[1,0,1,0,0,0,0,1,0,0,0,0,0,0],[1,0,0,0,0,0,0,1,0,0,0,0,0,0],[1,0,0,0,0,0,0,0,0,0,0,0,0,0],[1,0,0,0,0,0,0,1,0,0,0,0,0,0],[1,0,0,0,0,0,0,0,0,0,0,0,0,0],[1,0,0,0,0,0,0,1,0,0,0,0,0,0],[1,0,0,0,0,0,0,1,0,0,0,0,0,0],[1,0,0,0,0,0,0,1,0,0,0,0,0,0],[1,0,0,0,0,0,0,0,0,0,0,0,0,0],[1,0,0,0,0,0,0,0,0,0,0,0,0,1]]C0865668	chronic nonbacterial thrombotic endocarditis	[[0,0,0,0,0,0,0,0,0,0,0,0,0,1]]C1096189	type ii hypersensitivity	[[0,0,0,0,0,0,1,1,0,0,0,0,0,0]]C2675519	hypoadiponectinemia	[[0,0,0,1,0,0,0,0,0,0,0,0,0,0]]C1096184	west nile viral infection,west nile virus	[[0,0,0,0,0,0,1,1,0,0,0,0,0,0],[0,0,0,0,0,0,0,1,0,0,0,0,0,1]]C1514140	plasma cell neoplasms of the mouse oral cavity	[[0,0,0,0,0,0,0,0,0,0,0,1,0,0]]C0865660	except rheumatic tricuspid valve insufficiency of specified cause,except rheumatic tricuspid valve incompetence of specified cause	[[0,0,0,0,0,0,0,0,0,0,0,0,0,1],[0,0,0,0,0,0,0,0,0,0,0,0,0,1]]C0865661	except rheumatic tricuspid valve regurgitation of specified cause	[[0,0,0,0,0,0,0,0,0,0,0,0,0,1]]C0865662	except rheumatic tricuspid valve stenosis of specified cause	[[0,0,0,0,0,0,0,0,0,0,0,0,0,1]]C1866956	aortic root dilation	[[0,0,0,1,0,0,0,0,0,0,0,0,0,0]]C1268605	human echovirus infection	[[1,0,0,0,0,0,0,0,0,0,0,0,0,0]]C1866958	shprintzen omphalocele syndrome,and scoliosis dysmorphic facies learning disability omphalocele with hypoplasia of pharynx and larynx,pharynx and larynx hypoplasia with omphalocele,laryngeal and pharyngeal hypoplasia with omphalocele,shprintzen goldberg omphalocele syndrome	[[0,1,0,1,0,0,0,0,0,0,0,0,0,0],[0,0,0,1,0,0,0,0,0,0,0,0,0,0],[0,1,0,1,0,0,0,0,0,0,0,0,0,0],[0,1,0,0,0,0,0,0,0,0,0,0,0,0],[0,1,0,0,0,0,0,0,0,0,0,0,0,0]]C1268603	diphtheritic peripheral neuritis	[[1,0,0,0,0,0,0,0,0,0,0,0,0,0]]C0729584	infective meningitis,infectious meningitis	[[1,0,0,0,0,0,0,0,0,0,0,0,0,0],[0,0,0,0,0,0,0,1,0,0,0,1,0,0]]C0729585	infective ventriculitis,brain infective ventriculitis	[[1,0,0,0,0,0,0,0,0,0,0,0,0,0],[1,0,0,0,0,0,0,0,0,0,0,0,0,0]]C0729586	infective otitis media	[[1,0,0,0,0,0,0,0,0,0,0,0,0,0]]C0729587	infective blepharitis	[[1,0,0,0,0,0,0,0,0,0,0,0,0,0]]C0686238	secondary malignant neoplasm of body of uterus,metastatic malignant neoplasm to body of uterus,metastatic malignant neoplasm to corpus uteri,secondary malignant neoplasm of corpus uteri	[[1,0,0,0,0,0,0,0,0,0,0,0,0,0],[1,0,0,0,0,0,0,0,0,0,0,0,0,0],[1,0,0,0,0,0,0,0,0,0,0,0,0,0],[1,0,0,0,0,0,0,0,0,0,0,0,0,0]]C0729582	floating harbor syndrome,floating harbour syndrome,pelletier leisti syndrome	[[1,1,0,1,0,0,0,1,0,0,0,0,0,0],[1,0,0,0,0,0,0,1,0,0,0,0,0,0],[0,1,0,0,0,0,0,0,0,0,0,0,0,0]]C0686239	benign neoplasm of endometrium,benign endometrial neoplasm,benign endometrial tumor,benign endometrium tumor	[[0,0,0,0,0,0,0,0,0,0,0,1,0,1],[0,0,0,0,0,0,0,0,0,0,0,1,0,0],[0,0,0,0,0,0,0,0,0,0,0,1,0,0],[0,0,0,0,0,0,0,0,0,0,0,1,0,0]]C0729588	infective epididymo orchitis	[[1,0,0,0,0,0,0,0,0,0,0,0,0,0]]C0339939	acute exudative bronchiolitis	[[1,0,0,0,0,0,0,0,0,0,0,0,0,0]]C0404919	incomplete spontaneous abortion with embolism,complicated by embolism incomplete spontaneous abortion	[[1,0,0,0,0,0,0,0,0,0,0,0,0,0],[0,0,0,0,1,0,0,0,0,0,0,0,0,0]]C0023798	lipoma,lipomata,fatty tumor,lipo,fml,multiple lipomatosis,no icd o subtype lipoma,familial multiple lipomatosis	[[1,1,1,0,0,0,0,1,0,1,0,0,0,0],[0,1,0,0,0,0,0,1,0,0,0,0,0,0],[0,1,0,0,0,0,0,1,0,0,0,0,1,0],[0,0,0,1,0,0,0,0,0,0,0,0,0,0],[0,0,0,1,0,0,0,0,0,0,0,0,0,0],[0,0,0,1,0,0,0,0,0,0,0,0,0,0],[1,0,0,0,0,0,0,0,0,0,0,0,0,0],[0,0,0,1,0,0,0,0,0,0,0,0,0,0]]C0864770	lower genitourinary tract gonococcal infection specified as chronic or with duration of two months or more	[[0,0,0,0,0,0,0,0,0,0,0,0,0,1]]C0339931	acute haemophilus influenzae bronchitis	[[1,0,0,0,0,0,0,0,0,0,0,0,0,0]]C0023794	lipoidosis,lipid storage disease,lipidoses,lipidosis,inborn lipid storage disorder,lipoid storage disease	[[0,1,0,0,0,0,0,1,0,0,0,0,0,0],[1,0,0,0,0,0,0,1,0,0,0,0,0,0],[0,0,0,0,0,0,0,1,0,0,0,0,1,0],[0,0,0,0,0,0,0,1,1,0,0,0,1,0],[0,0,0,0,0,0,0,0,0,1,0,0,0,0],[0,0,0,0,0,0,0,0,0,0,0,0,0,1]]C0023795	lipoid proteinosis of urbach and wiethe,lipoidproteinosis,hyalinosis cutis et mucosae,urbach wiethe syndrome,lipoidosis cutis et mucosae,urbach wiethe disease,lipid proteinosis,lipoproteinosis,lipoid proteinosis	[[0,1,0,1,0,0,0,0,0,0,0,1,1,0],[0,0,0,0,0,0,0,0,0,0,0,0,1,0],[1,0,0,0,0,0,0,0,0,0,1,0,1,0],[0,0,0,0,0,0,0,1,0,0,1,0,1,0],[1,0,0,0,0,0,0,1,0,0,0,0,0,0],[1,1,0,1,0,0,0,0,0,0,0,0,1,0],[1,0,0,0,0,0,0,1,0,0,0,0,0,0],[0,0,0,0,0,0,0,1,0,0,0,0,1,0],[1,0,0,1,0,0,0,1,0,0,1,0,0,0]]C0339934	acute mycoplasmal bronchitis,acute bronchitis due to mycoplasma pneumoniae	[[1,0,0,0,0,0,0,0,0,0,0,0,0,0],[1,0,0,0,0,0,0,0,0,0,0,0,0,0]]C0339935	acute chlamydial bronchitis	[[1,0,0,0,0,0,0,0,0,0,0,0,0,0]]C0339937	acute viral bronchitis	[[1,0,0,0,0,0,0,0,0,0,0,0,0,0]]C0859775	w o ment of congestive heart failure or renal failure unspec hypertensive heart and renal disease	[[0,0,0,0,0,0,1,0,0,0,0,0,0,0]]C0595937	disorder of eye appendage,eye appendages	[[0,0,0,0,0,0,1,0,0,0,0,0,0,0],[0,0,0,0,0,0,0,0,1,0,0,0,0,0]]C1835166	polymorphic and lamellar cataract	[[0,0,0,1,0,0,0,0,0,0,0,0,0,0]]C2749022	chromosome xp11 23 p11 22 duplication syndrome	[[0,0,0,1,0,0,0,0,0,0,0,0,0,0]]C1835169	late onset cortical pulverulent cataract	[[0,0,0,1,0,0,0,0,0,0,0,0,0,0]]C1096458	vascular obstruction,occlusion of blood vessel,vascular occlusion	[[0,0,0,0,0,0,0,1,0,0,0,0,0,0],[0,0,0,0,0,0,0,1,0,0,0,0,0,1],[0,0,0,0,0,0,0,1,0,0,0,0,0,0]]C1384901	cystic disease of lung	[[0,0,0,1,0,0,0,0,0,0,0,0,0,0]]C0020443	hypercholesterolemia,hypercholesterolaemia,hypercholesteremia,hypercholesteraemia,high blood cholesterol level,elevated cholesterol	[[0,1,0,0,0,0,0,0,1,1,0,0,0,0],[1,0,0,0,0,0,0,1,1,0,0,0,0,0],[0,1,0,0,0,0,0,1,1,0,0,0,0,0],[0,0,0,0,0,0,0,1,0,0,0,0,0,0],[0,0,0,0,0,0,0,1,0,0,0,0,0,0],[0,0,0,0,0,0,0,1,0,0,0,0,0,0]]C0020441	hypercementosis,hypercementoses,cementum hyperplasia,cementation hyperplasia,cementosis	[[0,0,0,0,0,0,0,1,0,0,0,0,1,0],[0,1,0,0,0,0,0,0,0,0,0,0,0,0],[1,0,0,0,0,0,0,0,0,0,0,0,0,0],[0,0,0,0,0,0,0,0,0,0,0,0,0,1],[1,0,0,0,0,0,0,0,0,0,0,0,0,0]]C0021313	infection of kidney,renal infection,infectious disorder of kidney	[[1,0,0,0,0,0,1,1,0,0,1,0,0,0],[1,0,0,0,0,0,0,1,0,0,0,0,0,0],[1,0,0,0,0,0,0,0,0,0,0,0,0,0]]C0020445	familial hypercholesterolemia,familial hypercholesterolaemia,hyperbetalipoproteinemia,hyperbetalipoproteinaemia,essential hypercholesterolemia,hyperlipoproteinemia type ii,familial hyperlipoproteinemia type ii,familial hyperbetalipoproteinemia,familial hyperbetalipoproteinaemia,low density lipoprotein catabolic defect,ldl receptor disorder,ldl low density lipoprotein receptor disorder,essential familial hypercholesterolemia,essential familial hypercholesterolaemia,familial hypercholesteremia,type ii hyperlipidemia,hyper low density lipoproteinemia,hyper beta lipoproteinemias,hyperlipoproteinemia type 2s,hyperlipoproteinemia type 2	[[0,1,0,0,0,0,0,1,0,0,1,0,0,1],[1,0,0,0,0,0,0,1,0,0,0,0,0,0],[0,1,0,0,0,0,0,1,0,0,1,0,1,0],[1,0,0,0,0,0,0,0,0,0,0,0,0,0],[0,1,0,0,0,0,0,0,0,0,0,0,1,0],[0,1,0,0,0,0,0,1,0,0,1,0,1,0],[0,0,0,0,0,0,0,0,0,1,0,0,0,0],[1,0,0,0,0,0,0,0,0,1,1,0,0,0],[1,0,0,0,0,0,0,0,0,0,0,0,0,0],[1,0,0,0,0,0,0,0,0,0,0,0,0,0],[1,0,0,0,0,0,0,0,0,0,0,0,0,0],[1,0,0,0,0,0,0,0,0,0,0,0,0,0],[1,0,0,0,0,0,0,0,0,0,1,0,0,0],[1,0,0,0,0,0,0,0,0,0,0,0,0,0],[0,0,0,0,0,0,0,1,0,0,0,0,0,0],[0,0,0,0,0,0,0,1,0,0,1,1,0,0],[0,1,0,0,0,0,0,0,0,0,0,0,1,0],[0,1,0,0,0,0,0,0,0,0,0,0,1,0],[0,1,0,0,0,0,0,0,0,0,0,0,0,0],[0,1,0,0,0,0,0,0,0,0,0,0,1,0]]C0013927	amniotic fluid embolism,embolus amniotic fluid	[[0,1,0,0,1,0,0,1,0,0,1,0,1,0],[0,0,0,0,0,0,0,1,1,0,0,0,0,0]]C1298899	infection due to mycobacterium rhodesiae	[[1,0,0,0,0,0,0,0,0,0,0,0,0,0]]C1270170	disorder of chorion,abnormal chorion,abnormal chorionic villi	[[1,0,0,0,0,0,0,0,0,0,0,0,0,0],[1,0,0,0,0,0,0,0,0,0,0,0,0,0],[1,0,0,0,0,0,0,0,0,0,0,0,0,0]]C0280252	colon cancer stage	[[0,0,0,0,0,1,0,1,0,0,0,0,0,0]]C0280253	gastric cancer stage,stomach cancer stage	[[0,0,0,0,0,1,0,1,0,0,0,0,0,0],[0,0,0,0,0,1,0,1,0,0,0,0,0,0]]C0280250	small intestine cancer stage cell type,small intestine cancer stage	[[0,0,0,0,0,1,0,0,0,0,0,0,0,0],[0,0,0,0,0,1,0,0,0,0,0,0,0,0]]C0280251	chronic myelogenous leukemia stage	[[0,0,0,0,0,1,0,0,0,0,0,0,0,0]]C0280256	testicular cancer stage	[[0,0,0,0,0,1,0,1,0,0,0,0,0,0]]C0280257	esophageal cancer stage	[[0,0,0,0,0,1,0,0,0,0,0,0,0,0]]C0280254	cutaneous t cell lymphoma stage	[[0,0,0,0,0,1,0,0,0,0,0,0,0,0]]C0280255	endometrial cancer stage,stage cancer of the endometrium,stage endometrial carcinoma	[[0,0,0,0,0,1,0,1,0,0,0,0,0,0],[0,0,0,0,0,1,0,0,0,0,0,0,0,0],[0,0,0,0,0,1,0,0,0,0,0,0,0,0]]C1883486	uterine corpus cancer,endometrial cancer,cancer of the corpus uteri	[[0,0,0,0,0,0,0,0,0,0,0,1,0,0],[0,0,0,0,0,0,0,0,0,0,0,1,0,0],[0,0,0,0,0,0,0,0,0,0,0,1,0,0]]C1855345	conductive hearing loss and microtia with short stature cutaneous mastocytosis	[[0,1,0,0,0,0,0,0,0,0,0,0,0,0]]C0280258	thyroid cancer stage cell type,thyroid cancer stage	[[0,0,0,0,0,1,0,1,0,0,0,0,0,0],[0,0,0,0,0,1,0,1,0,0,0,0,0,0]]C0280259	ewings sarcoma primitive neuroepitelial tumor stage,stage ewings sarcoma	[[0,0,0,0,0,1,0,0,0,0,0,0,0,0],[0,0,0,0,0,1,0,0,0,0,0,0,0,0]]C0865088	benign neoplasm of soft parts of female breast	[[0,0,0,0,0,0,0,0,0,0,0,0,0,1]]C0023860	listeriosis,listerioses,listeria infections,circling disease,infection due to listeria monocytogenes,listerellosis	[[0,0,0,0,0,0,0,1,0,0,1,0,0,0],[0,1,0,0,0,0,0,0,0,0,0,0,0,0],[0,1,0,0,0,0,0,1,0,1,0,0,1,0],[1,0,0,0,0,0,0,0,0,0,0,0,0,0],[1,0,0,0,0,0,0,1,0,0,0,0,0,1],[1,0,0,0,0,0,0,0,0,0,0,0,0,0]]C0865080	benign neoplasm of connective tissue of breast	[[0,0,0,0,0,0,0,0,0,0,0,0,0,1]]C0865081	benign neoplasm of connective tissue of female breast	[[0,0,0,0,0,0,0,0,0,0,0,0,0,1]]C0242670	persistent vegetative state,chronic vegetative state,pvs,pvs persistent vegetative state,pvss,persistent unawareness state	[[0,1,0,0,1,0,0,1,0,0,0,0,1,0],[1,0,0,0,0,0,0,1,0,0,0,0,0,0],[0,0,0,0,0,0,0,0,0,0,0,0,1,0],[1,0,0,0,0,0,0,0,0,0,0,0,0,0],[0,1,0,0,0,0,0,0,0,0,0,0,0,0],[0,1,0,0,0,0,0,0,0,0,0,0,1,0]]C0865083	benign neoplasm of glandular tissue of breast	[[0,0,0,0,0,0,0,0,0,0,0,0,0,1]]C0242676	intrinsic positive pressure respiration,auto peep,intrinsic peep,occult peep,occult positive pressure respiration,nontherapeutic positive pressure respiration,autopeep,non therapeutic positive pressure respiration	[[0,1,0,0,0,0,0,0,0,0,0,0,1,0],[0,1,0,0,0,0,0,1,0,0,0,0,1,0],[0,1,0,0,0,0,0,1,0,0,0,0,1,0],[0,1,0,0,0,0,0,0,0,0,0,0,1,0],[0,1,0,0,0,0,0,0,0,0,0,0,1,0],[0,1,0,0,0,0,0,0,0,0,0,0,1,0],[0,0,0,0,0,0,0,1,0,0,0,0,1,0],[0,1,0,0,0,0,0,0,0,0,0,0,1,0]]C0865085	benign neoplasm of glandular tissue of female breast	[[0,0,0,0,0,0,0,0,0,0,0,0,0,1]]C0865086	benign neoplasm of soft parts of breast	[[0,0,0,0,0,0,0,0,0,0,0,0,0,1]]C0865087	benign neoplasm of soft parts of male breast	[[0,0,0,0,0,0,0,0,0,0,0,0,0,1]]C1855348	marfanoid habitus with microcephaly and glomerulonephritis	[[0,0,0,1,0,0,0,0,0,0,0,0,0,0]]C1563751	y linked genetic diseases,y chromosome linked genetic diseases	[[0,1,0,0,0,0,0,0,0,0,0,0,1,0],[0,1,0,0,0,0,0,0,0,0,0,0,1,0]]C1843815	newfoundland rod cone dystrophy,nfrcd	[[0,0,0,1,0,0,0,0,0,0,0,0,0,0],[0,0,0,1,0,0,0,0,0,0,0,0,0,0]]C1827612	diabetic autonomic neuropathy associated with type 2 diabetes mellitus	[[1,0,0,0,0,0,0,0,0,0,0,0,0,0]]C0544869	alopecia liminaris frontalis,alopecia marginalis	[[1,0,0,0,0,0,0,0,0,0,0,0,0,0],[1,0,0,0,0,0,0,0,0,0,0,0,0,0]]C1863416	autosomal dominant compelling helio ophthalmic outburst syndrome,photic sneeze reflex,achoo syndrome,peroutka sneeze,sneezing from light exposure,autosomal dominant compelling helioophthalmic outburst syndrome	[[0,1,0,0,0,0,0,0,0,0,0,0,0,0],[0,1,0,1,0,0,0,0,0,0,0,0,0,0],[0,0,0,1,0,0,0,0,0,0,0,0,0,0],[0,1,0,1,0,0,0,0,0,0,0,0,0,0],[0,0,0,1,0,0,0,0,0,0,0,0,0,0],[0,0,0,1,0,0,0,0,0,0,0,0,0,0]]C3203101	tinea unguium	[[0,1,0,0,0,0,0,0,0,0,0,0,0,0]]C1697884	esophageal necrotic lesion,esophageal necrosis	[[0,0,0,0,0,0,0,0,0,0,0,1,0,0],[0,0,0,0,0,0,0,0,0,0,0,1,0,0]]C1449721	infant gynecomastia,breast engorgement in newborn,neonatal breast engorgement,neonatal gynecomastia,neonatal gynaecomastia,newborn gynecomastia,breast engorgement of new born	[[0,1,0,0,0,0,0,0,0,0,0,0,0,0],[0,0,0,0,1,0,0,0,0,0,0,0,0,0],[0,0,1,0,0,0,0,0,0,0,0,0,0,0],[1,0,0,0,0,0,0,0,0,0,0,0,0,0],[1,0,0,0,0,0,0,0,0,0,0,0,0,0],[0,1,0,0,0,0,0,0,0,0,0,0,0,0],[1,0,0,0,0,0,0,0,0,0,0,0,0,0]]C1827618	infection due to streptococcus group g	[[1,0,0,0,0,0,0,0,0,0,0,0,0,0]]C1828432	fibrous dysplasia of orbit	[[1,0,0,0,0,0,0,0,0,0,0,0,0,0]]C1828435	anisophoria,lens induced anisophoria	[[1,0,0,0,0,0,0,0,0,0,0,0,0,0],[1,0,0,0,0,0,0,0,0,0,0,0,0,0]]C0154246	inborn urea cycle disorders,disorder of the urea cycle metabolism,urea cycle disorders,arginine and ammonia argininosuccinic acid citrulline disorders of metabolism of ornithine	[[0,0,0,0,0,0,0,0,0,1,0,1,1,0],[1,0,0,0,1,0,0,1,0,0,0,0,0,0],[1,1,0,0,0,0,0,1,0,0,0,0,1,0],[0,0,0,0,0,0,0,0,0,0,0,0,0,1]]C0154249	disorders of carbohydrate metabolism and transport	[[0,0,0,0,1,0,0,0,0,0,0,0,0,0]]C1997034	chronic recurrent sinusitis	[[1,0,0,0,0,0,0,0,0,0,0,0,0,0]]C1708498	indirect contact transmission infection,indirect infection	[[0,0,0,0,0,0,0,0,0,0,0,1,0,0],[0,0,0,0,0,0,0,0,0,0,0,1,0,0]]C1533095	pamidronic acid induced uveitis	[[1,0,0,0,0,0,0,0,0,0,0,0,0,0]]C0268923	tunica vaginalis stricture	[[1,0,0,0,0,0,0,0,0,0,0,0,0,1]]C1301398	disorder of neonatal umbilicus	[[1,0,0,0,0,0,0,0,0,0,0,0,0,0]]C0268926	carbuncle of scrotum	[[1,0,0,0,0,0,0,0,0,0,0,0,0,0]]C0268927	cellulitis of scrotum,scrotal cellulitis	[[1,0,0,0,0,0,0,1,0,0,0,0,0,0],[0,0,0,0,0,0,0,1,0,0,0,0,0,0]]C0268925	boil of scrotum	[[1,0,0,0,0,0,0,0,0,0,0,0,0,0]]C1395519	displacement of cervical intervertebral disc	[[1,0,0,0,0,0,0,0,0,0,0,0,0,0]]C0268928	abscess of spermatic cord	[[1,0,0,0,0,0,0,0,0,0,0,0,0,0]]C0268929	boil of spermatic cord	[[1,0,0,0,0,0,0,0,0,0,0,0,0,0]]C0267525	bilious vomiting following gastrointestinal surgery	[[0,0,0,0,0,0,0,0,0,0,0,0,0,1]]C0585595	anemia in ovarian carcinoma,anaemia in ovarian carcinoma	[[1,0,0,0,0,0,0,0,0,0,0,0,0,0],[1,0,0,0,0,0,0,0,0,0,0,0,0,0]]C1406891	late syphilis of central nervous system	[[0,0,0,0,0,0,0,0,0,0,0,0,0,1]]C2711529	infection of bone of ankle and or foot	[[1,0,0,0,0,0,0,0,0,0,0,0,0,0]]C0406511	thread veins	[[1,0,0,0,0,0,0,1,0,0,0,0,0,0]]C0406510	angiodyskinesia	[[1,0,0,0,0,0,0,0,0,0,0,0,0,0]]C0406513	itching purpura	[[1,0,0,0,0,0,0,0,0,0,0,0,0,0]]C0406512	idiopathic capillaritis	[[1,0,0,0,0,0,0,0,0,0,0,0,0,0]]C0406515	pigmented purpuric eruption,schamberg purpura,familial capillaritis,familial pigmented purpuric eruption,familial schambergs disease	[[0,1,0,1,0,0,0,0,0,0,0,0,0,0],[0,1,0,0,0,0,0,0,0,0,0,0,0,0],[1,0,0,0,0,0,0,0,0,0,0,0,0,0],[0,1,0,0,0,0,0,0,0,0,0,0,0,0],[0,1,0,0,0,0,0,0,0,0,0,0,0,0]]C0406514	lichen aureus	[[1,0,0,0,0,0,0,1,0,0,0,0,0,0]]C0406517	gougerot ruiter purpura,gougerot ruiter vasculitis	[[1,0,0,0,0,0,0,0,0,0,0,0,0,0],[1,0,0,0,0,0,0,0,0,0,0,0,0,0]]C0406516	primary cutaneous vasculitis	[[1,0,0,0,0,0,0,0,0,0,0,0,0,0]]C0406519	infected ulcer of skin	[[1,0,0,0,0,0,0,0,0,0,0,0,0,0]]C0406518	secondary cutaneous vasculitis	[[1,0,0,0,0,0,0,0,0,0,0,0,0,0]]C0751197	fulminant hepatic failure with cerebral edema	[[0,1,0,0,0,0,0,0,0,0,0,0,0,0]]C1333190	cystic neoplasm,cystic tumor	[[0,0,0,0,0,0,0,0,0,0,0,1,0,0],[0,0,0,0,0,0,0,0,0,0,0,1,0,0]]C1969085	intraocular pressure quantitative trait locus,iopqtl,susceptibility to glaucoma	[[0,0,0,1,0,0,0,0,0,0,0,0,0,0],[0,0,0,1,0,0,0,0,0,0,0,0,0,0],[0,0,0,1,0,0,0,0,0,0,0,0,0,0]]C1276801	erysipeloid,rosenbach erysipeloid,infection due to erysipelothrix insidiosa,infection due to erysipelothrix rhusiopathiae,infection due to e rhusiopathiae	[[0,1,0,0,0,0,0,1,0,0,1,0,1,0],[1,0,0,0,0,0,0,0,0,0,1,0,0,1],[0,0,0,0,0,0,0,0,0,0,0,0,0,1],[1,0,0,0,0,0,0,0,0,0,0,0,0,0],[0,0,0,0,0,0,0,0,0,0,0,0,0,1]]C0004186	atony of gallbladder	[[1,0,0,0,0,0,0,0,0,0,0,0,0,0]]C0004187	atopic dermatitis and related conditions	[[0,0,0,0,1,0,0,0,0,0,0,0,0,0]]C2712932	merkel cell carcinoma nodal presentation	[[0,0,0,0,0,0,0,0,0,0,0,0,0,1]]C1321878	desmoplastic infantile ganglioglioma,dig	[[1,0,0,0,0,0,0,0,0,0,0,1,0,0],[0,0,0,0,0,0,0,0,0,0,0,1,0,0]]C0005859	bloom syndrome,bloom torre machacek syndrome,congenital telangiectatic erythema syndrome,blm,bs bloom syndrome,bs,bls	[[0,1,0,1,0,0,0,1,0,1,0,0,0,0],[0,1,0,0,0,0,0,0,0,1,0,0,1,0],[1,0,0,0,0,0,0,0,0,0,0,1,0,0],[0,0,0,1,0,0,0,0,0,0,0,0,0,0],[1,0,0,0,0,0,0,0,0,0,0,0,0,0],[0,0,0,1,0,0,0,0,0,0,0,0,0,0],[0,0,0,1,0,0,0,0,0,0,0,0,0,0]]C0585945	infantile viral gastroenteritis	[[1,0,0,0,0,0,0,0,0,0,0,0,0,0]]C0585946	malignant epiglottis neoplasm,malignant epiglottic neoplasm,malignant epiglottic tumor,malignant tumor of the epiglottis	[[0,0,0,0,0,0,0,0,0,0,0,1,0,1],[0,0,0,0,0,0,0,0,0,0,0,1,0,0],[0,0,0,0,0,0,0,0,0,0,0,1,0,0],[0,0,0,0,0,0,0,0,0,0,0,1,0,0]]C0695242	neurogenic bowel	[[0,1,0,0,1,0,0,1,0,0,0,0,1,0]]C0162700	tick borne diseases,disease transmitted by tick,tick borne infectious disease	[[1,1,0,0,0,0,0,1,0,0,0,0,1,0],[1,0,0,0,0,0,0,0,0,0,0,0,0,0],[1,0,0,0,0,0,0,0,0,0,0,0,0,0]]C1720544	hypogonadotropic hypogonadism due to follicle stimulating hormone deficiency	[[1,0,0,0,0,0,0,0,0,0,0,0,0,0]]C1720545	microsporidiosis associated with aids	[[1,0,0,0,0,0,0,0,0,0,0,0,0,0]]C0585948	lipoma of back	[[1,0,0,0,0,0,0,1,0,0,0,0,0,0]]C0589616	genitourinary chlamydia infection	[[1,0,0,0,0,0,0,0,0,0,0,0,0,0]]C1720541	prion disease associated with aids	[[1,0,0,0,0,0,0,0,0,0,0,0,0,0]]C1274296	bowel bypass syndrome	[[1,0,0,0,0,0,0,0,0,0,0,0,0,0]]C2981672	stage iia ampulla of vater cancer,stage iia ampulla of vater cancer ajcc v7	[[0,0,0,0,0,0,0,0,0,0,0,1,0,0],[0,0,0,0,0,0,0,0,0,0,0,1,0,0]]C2267233	neonatal hypotonia,hypotonia	[[0,1,0,1,0,0,0,0,1,0,0,0,0,0],[0,0,0,1,0,0,0,0,0,0,0,0,0,0]]C2025996	cellulitis of left ankle	[[0,0,0,0,0,0,1,0,0,0,0,0,0,0]]C1274416	tinea capitis due to trichophyton gourvilii	[[1,0,0,0,0,0,0,0,0,0,0,0,0,0]]C1274417	tinea capitis due to microsporum equinum	[[1,0,0,0,0,0,0,0,0,0,0,0,0,0]]C0282643	type i smith lemli opitz syndrome,type 1 smith lemli opitz syndrome	[[0,1,0,0,0,0,0,0,0,0,0,0,0,0],[0,1,0,0,0,0,0,0,0,0,0,0,0,0]]C1274414	tinea capitis due to microsporum distortum,tinea capitis due to microsporum canis variant distortum	[[1,0,0,0,0,0,0,0,0,0,0,0,0,0],[1,0,0,0,0,0,0,0,0,0,0,0,0,0]]C0282644	type ii smith lemli opitz syndrome,rutledge lethal multiple congenital anomaly syndrome,lethal acrodysgenital syndrome,type 2 smith lemli opitz syndrome,rutledge friedman harrod syndrome	[[0,1,0,0,0,0,0,0,0,0,0,0,0,0],[0,1,0,1,0,0,0,0,0,0,0,0,0,0],[0,1,0,1,0,0,0,0,0,0,0,0,0,0],[0,1,0,0,0,0,0,0,0,0,0,0,0,0],[0,1,0,0,0,0,0,0,0,0,0,0,0,0]]C2981673	stage iib ampulla of vater cancer,stage iib ampulla of vater cancer ajcc v7	[[0,0,0,0,0,0,0,0,0,0,0,1,0,0],[0,0,0,0,0,0,0,0,0,0,0,1,0,0]]C1305256	infiltrating angiolipoma	[[1,0,0,0,0,0,0,0,0,0,0,1,0,0]]C1274412	chronic acquired mucocutaneous candidiasis,chronic mucocutaneous candidosis	[[1,0,0,0,0,0,0,0,0,0,0,0,0,0],[1,0,0,0,0,0,0,0,0,0,0,0,0,0]]C0920601	neonatal hypoprothrombinemia	[[0,0,0,0,0,0,0,0,0,1,0,0,0,0]]C1879536	acinar cell adenocarcinoma of the rat pancreas,rat pancreatic acinar cell adenocarcinoma	[[0,0,0,0,0,0,0,0,0,0,0,1,0,0],[0,0,0,0,0,0,0,0,0,0,0,1,0,0]]C1274413	tinea capitis due to microsporum canis,tinea capitis due to microsporum canis variant canis	[[1,0,0,0,0,0,0,0,0,0,0,0,0,0],[1,0,0,0,0,0,0,0,0,0,0,0,0,0]]C0751738	nervous system lysosomal storage diseases,nervous system lysosomal enzyme disorders	[[0,0,0,0,0,0,0,0,0,0,0,0,1,0],[0,0,0,0,0,0,0,0,0,0,0,0,1,0]]C0751739	basal ganglia cerebrovascular disease,basal ganglia vascular diseases	[[0,0,0,0,0,0,0,0,0,0,0,0,1,0],[0,0,0,0,0,0,0,0,0,0,0,0,1,0]]C1274410	nodular candidiasis of diaper area,nodular candidosis of napkin area candidosis,granuloma gluteale infantum due to candida,nodular candidiasis of napkin area	[[1,0,0,0,0,0,0,0,0,0,0,0,0,0],[1,0,0,0,0,0,0,0,0,0,0,0,0,0],[1,0,0,0,0,0,0,0,0,0,0,0,0,0],[1,0,0,0,0,0,0,0,0,0,0,0,0,0]]C0751730	anton syndrome,denial visual hallucination syndrome,psychic denial of blindness,anton babinski syndrome,reversible cortical blindness,transient antons syndrome	[[1,1,0,0,0,0,0,1,0,0,0,0,0,0],[1,0,0,0,0,0,0,0,0,0,0,0,0,0],[0,1,0,0,0,0,0,0,0,0,0,0,0,0],[0,1,0,0,0,0,0,0,0,0,0,0,0,0],[0,1,0,0,0,0,0,0,0,0,0,0,0,0],[0,1,0,0,0,0,0,0,0,0,0,0,0,0]]C0751731	essential intracranial hypotension,spontaneous intracranial hypotension	[[0,1,0,0,0,0,0,0,0,0,0,0,0,0],[0,1,0,0,0,0,0,0,0,0,0,0,0,0]]C0751732	secondary intracranial hypotension	[[0,1,0,0,0,0,0,0,0,0,0,0,0,0]]C0751733	spinal cord degenerative diseases	[[0,1,0,0,0,0,0,1,0,0,0,0,0,0]]C1274290	neonatal purpura fulminans	[[1,0,0,0,0,0,0,0,0,0,0,0,0,0]]C2981674	stage ia pancreatic cancer,stage ia pancreatic cancer ajcc v7	[[0,0,0,0,0,0,0,0,0,0,0,1,0,0],[0,0,0,0,0,0,0,0,0,0,0,1,0,0]]C1318470	phytobezoar	[[1,0,0,0,0,0,0,1,0,0,0,0,0,0]]C0349489	fetal hypoxia,fetus hypoxia,in utero hypoxia	[[1,1,0,0,0,0,0,1,0,0,0,1,0,0],[0,0,0,0,0,0,0,1,0,0,0,0,0,0],[0,0,0,0,0,0,0,0,0,0,0,1,0,0]]C0349488	early neonatal hypocalcemic tetany,early neonatal hypocalcaemic tetany	[[1,0,0,0,0,0,0,0,0,0,0,0,0,0],[1,0,0,0,0,0,0,0,0,0,0,0,0,0]]C0349480	congenital nephritis	[[1,0,0,0,0,0,0,0,0,0,0,0,0,0]]C0349487	transient hypothyrotropinemia,transient hypothyrotropinaemia	[[1,0,0,0,0,0,0,0,0,0,0,0,0,0],[1,0,0,0,0,0,0,0,0,0,0,0,0,0]]C0349486	transient hypothyroxinemia,transient hypothyroxinaemia	[[1,0,0,0,0,0,0,1,0,0,0,0,0,0],[1,0,0,0,0,0,0,1,0,0,0,0,0,0]]C1860441	deficiency of platelet receptor for collagen	[[0,0,0,1,0,0,0,0,0,0,0,0,0,0]]C0346370	papilloma of cornea	[[1,0,0,0,0,0,0,0,0,0,0,0,0,0]]C0346371	nevus of cornea,naevus of cornea	[[1,0,0,0,0,0,0,1,0,0,0,0,0,0],[1,0,0,0,0,0,0,0,0,0,0,0,0,0]]C0276668	coccidioidal granuloma	[[0,0,0,0,0,0,0,0,0,0,0,0,0,1]]C0346373	malignant melanoma of iris,iris melanoma,anterior uveal melanoma,iris intraocular melanoma	[[1,0,0,0,0,0,0,0,0,0,0,1,0,0],[1,0,0,0,0,1,0,1,0,0,0,1,0,0],[0,0,0,0,0,1,0,0,0,0,0,0,0,0],[0,0,0,0,0,1,0,0,0,0,0,0,0,0]]C0346374	benign tumor of iris,benign tumour of iris,benign neoplasm of iris	[[1,0,0,0,0,0,0,0,0,0,0,1,0,0],[1,0,0,0,0,0,0,0,0,0,0,0,0,0],[0,0,0,0,0,0,0,0,0,0,0,1,0,1]]C0346375	juvenile xanthogranuloma of iris	[[1,0,0,0,0,0,0,0,0,0,0,0,0,0]]C0346376	nevus of iris,naevus of iris,benign melanoma of iris	[[1,0,0,0,0,0,0,1,0,0,0,1,0,0],[1,0,0,0,0,0,0,0,0,0,0,0,0,0],[1,0,0,0,0,0,0,0,0,0,0,0,0,0]]C0346378	malignant medulloepithelioma of ciliary body,medulloepithelioma of ciliary body	[[1,0,0,0,0,0,0,0,0,0,0,0,0,0],[1,0,0,0,0,0,0,0,0,0,0,1,0,0]]C0346379	malignant melanoma of ciliary body,ciliary body melanoma	[[1,0,0,0,0,0,0,0,0,0,0,1,0,0],[1,0,0,0,0,0,0,0,0,0,0,1,0,0]]C0276660	equine gutturomycosis	[[1,0,0,0,0,0,0,0,0,0,0,0,0,0]]C0276661	ocular blastomycosis,north american ocular blastomycosis	[[1,0,0,0,0,0,0,0,0,0,0,0,0,0],[1,0,0,0,0,0,0,0,0,0,0,0,0,0]]C0014008	empty sella syndrome,empty sella,empty sella turcica syndrome,empty sella turcica	[[1,1,0,0,0,0,0,1,0,0,1,0,0,0],[0,0,1,0,0,0,0,1,0,0,0,0,0,0],[0,0,0,0,0,0,0,0,0,0,0,0,1,0],[0,1,0,1,0,0,0,1,0,0,1,0,1,0]]C0014009	empyema	[[0,0,1,0,0,0,0,1,0,0,0,0,0,0]]C1168308	paraproctitis	[[0,0,0,0,0,0,0,1,0,0,0,0,0,0]]C0276665	pulmonary paracoccidioidomycosis,pulmonary south american blastomycosis	[[0,0,0,0,0,0,0,0,0,0,0,0,0,1],[1,0,0,0,0,0,0,0,0,0,0,0,0,0]]C1559663	peripheral nerve infection adverse event documented clinically or microbiologically with grade 3 or 4 neutrophils,nerve peripheral infection documented clinically or microbiologically with grade 3 or 4 neutrophils anc 1 0 x 10e9 l	[[0,0,0,0,0,0,0,0,0,0,0,1,0,0],[0,0,0,0,0,0,0,0,0,0,0,1,0,0]]C1866130	rhombencephalosynapsis	[[0,0,0,1,0,0,0,0,0,0,0,0,0,0]]C1274418	tinea capitis due to microsporum ferrugineum	[[1,0,0,0,0,0,0,0,0,0,0,0,0,0]]C1444598	syphilitic retinitis	[[1,0,0,0,0,0,0,0,0,0,0,0,0,0]]C1274419	tinea capitis due to microsporum fulvum	[[1,0,0,0,0,0,0,0,0,0,0,0,0,0]]C1866138	progressive familial intrahepatic 2 cholestasis,pfic2	[[0,1,0,1,0,0,0,0,0,0,0,0,0,0],[0,0,0,1,0,0,0,0,0,0,0,0,0,0]]C1866139	vacuolar neuromyopathy,mdrv,with rimmed vacuoles autosomal dominant muscular dystrophy	[[0,0,0,1,0,0,1,0,0,0,0,0,0,0],[0,0,0,1,0,0,0,0,0,0,0,0,0,0],[0,0,0,1,0,0,0,0,0,0,0,0,0,0]]C0238494	benign polyp ureter	[[0,0,0,0,0,0,0,0,0,0,1,0,0,0]]C0342877	disorder of cholesterol metabolism	[[1,0,0,0,0,0,0,0,0,0,0,0,0,0]]C0342875	isolated dihydroxyacetone phosphate acyltransferase deficiency,isolated dhap at dihydroxyacetone phosphate acyltransferase deficiency	[[1,0,0,0,0,0,0,0,0,0,0,0,0,0],[1,0,0,0,0,0,0,0,0,0,0,0,0,0]]C1274292	dysproteinemic purpura	[[1,0,0,0,0,0,0,0,0,0,0,0,0,0]]C0342879	primary hypercholesterolemia,primary hypercholesterolaemia	[[1,0,0,0,0,0,0,1,0,0,0,0,0,0],[1,0,0,0,0,0,0,1,0,0,0,0,0,0]]C2987170	pancreatic macrocystic serous cystadenoma	[[0,0,0,0,0,0,0,0,0,0,0,1,0,0]]C0349735	infection of ovary,ovarian infection	[[1,0,0,0,0,0,0,1,0,0,0,0,0,0],[0,0,0,0,0,0,0,1,0,0,0,1,0,0]]C0349734	female pelvic cellulitis	[[1,0,0,0,0,0,0,0,0,0,0,0,0,1]]C0853968	recurrent inflammatory breast carcinoma	[[0,0,0,0,0,0,0,0,0,0,0,1,0,0]]C0349738	cutaneous disorders of yaws	[[1,0,0,0,0,0,0,0,0,0,0,0,0,0]]C3249882	infection due to mycobacterium avium	[[1,0,0,0,0,0,0,0,0,0,0,0,0,0]]C1274984	fat hypertrophy due to injected drug	[[1,0,0,0,0,0,0,0,0,0,0,0,0,0]]C0343475	cutaneous mycobacterium marinum infection,skin infection by m balnei,skin infection by mycobacterium marinum	[[1,0,0,0,0,0,0,0,0,0,0,0,0,0],[0,0,0,0,0,0,0,0,0,0,0,0,0,1],[0,0,0,0,0,0,0,0,0,0,0,0,0,1]]C1274986	fixed drug reaction affecting oral mucous membranes,drug induced fixed ulcer of oral mucous membrane	[[1,0,0,0,0,0,0,0,0,0,0,0,0,0],[1,0,0,0,0,0,0,0,0,0,0,0,0,0]]C1274987	drug induced oral ulceration	[[1,0,0,0,0,0,0,0,0,0,0,0,0,0]]C1274980	dermatosis attributable to coumarin anticoagulant	[[1,0,0,0,0,0,0,0,0,0,0,0,0,0]]C1274981	coumarin necrosis,warfarin necrosis	[[1,0,0,0,0,0,0,1,0,0,0,0,0,0],[1,0,0,0,0,0,0,1,0,0,0,0,0,0]]C0343472	cervical atypical mycobacterial lymphadenitis	[[1,0,0,0,0,0,0,0,0,0,0,0,0,0]]C0343473	skin and soft tissue atypical mycobacterial infection	[[1,0,0,0,0,0,0,0,0,0,0,0,0,0]]C1513519	mouse cardiac angiosarcoma	[[0,0,0,0,0,0,0,0,0,0,0,1,0,0]]C3150667	10 early infantile epileptic encephalopathy,eiee10,and developmental delay seizures microcephaly,mcsz	[[0,0,0,1,0,0,0,0,0,0,0,0,0,0],[0,0,0,1,0,0,0,0,0,0,0,0,0,0],[0,0,0,1,0,0,0,0,0,0,0,0,0,0],[0,0,0,1,0,0,0,0,0,0,0,0,0,0]]C0343478	cutaneous mycobacterium haemophilium infection	[[1,0,0,0,0,0,0,0,0,0,0,0,0,0]]C0343479	hyperimmune cutaneous reaction to atypical mycobacteria	[[1,0,0,0,0,0,0,0,0,0,0,0,0,0]]C1283655	deficiency of peptidase a	[[1,0,0,0,0,0,0,0,0,0,0,0,0,0]]C0238148	crocodile tears syndrome,bogorads syndrome,gustatory lacrimation syndrome,paroxysmal lacrimation	[[1,0,0,0,0,0,0,0,0,0,1,0,0,0],[1,0,0,0,0,0,0,0,0,0,1,0,0,0],[1,0,0,0,0,0,0,0,0,0,1,0,0,0],[0,0,0,0,0,0,0,0,0,0,1,0,0,0]]C0571251	hydroxyethylcellulose allergy	[[1,0,0,0,0,0,0,0,0,0,0,0,0,0]]C3249881	infection suppurative,suppuration	[[1,0,0,0,0,0,0,1,0,0,0,0,0,0],[1,0,0,0,0,0,0,0,0,0,0,0,0,0]]C0743746	eyes dry chronic	[[0,0,0,0,0,0,0,1,0,0,0,0,0,0]]C0238147	green tobacco sickness,tobacco croppers sickness	[[0,0,0,0,0,0,0,0,0,0,1,0,0,0],[0,0,0,0,0,0,0,0,0,0,1,0,0,0]]C1275052	porphyria due to hexachlorobenzene,porphyria turcica	[[1,0,0,0,0,0,0,0,0,0,0,0,0,0],[1,0,0,0,0,0,0,0,0,0,0,0,0,0]]C0238141	gingival carcinoma,carcinoma gingival cancer,carcinoma of gingiva,carcinoma of the gum	[[0,0,0,0,0,0,0,0,0,0,0,1,0,0],[0,0,0,0,0,0,0,0,0,0,1,0,0,0],[0,0,0,0,0,0,0,0,0,0,1,1,0,0],[0,0,0,0,0,0,0,0,0,0,0,1,0,0]]C1321410	keystone virus encephalitis	[[1,0,0,0,0,0,0,0,0,0,0,0,0,0]]C0238143	focal embolic glomerulonephritis,focal proliferative glomerulonephritis,local glomerulonephritis	[[0,0,0,0,0,0,0,0,0,0,1,1,0,0],[0,0,0,0,0,0,0,0,0,0,1,0,0,0],[0,0,0,0,0,0,0,0,0,0,1,0,0,0]]C1275056	arsenic induced skin malignancy	[[1,0,0,0,0,0,0,0,0,0,0,0,0,0]]C1834014	oculopharyngodistal myopathy,folp dr,opdm,faciooculolaryngopharyngeal myopathy with distal and respiratory involvement	[[0,0,0,1,0,0,0,0,0,0,0,0,0,0],[0,0,0,1,0,0,0,0,0,0,0,0,0,0],[0,0,0,1,0,0,0,0,0,0,0,0,0,0],[0,0,0,1,0,0,0,0,0,0,0,0,0,0]]C1627889	cache valley virus infection,cache valley virus disease,cv virus infection	[[1,0,0,0,0,0,0,0,0,0,0,0,0,0],[1,0,0,0,0,0,0,0,0,0,0,0,0,0],[1,0,0,0,0,0,0,0,0,0,0,0,0,0]]C2981678	stage iiia penile cancer,stage iiia penile cancer ajcc v7	[[0,0,0,0,0,0,0,0,0,0,0,1,0,0],[0,0,0,0,0,0,0,0,0,0,0,1,0,0]]C2827454	childhood renal disorder,pediatric kidney disease	[[0,0,0,0,0,0,0,0,0,0,0,1,0,0],[0,0,0,0,0,0,0,0,0,0,0,1,0,0]]C1314678	ameloblastic carcinoma	[[1,0,0,0,0,0,0,0,0,0,0,1,0,0]]C1096487	hereditary factor viii deficiency disease without inhibitor,hemophilia a without inhibitor,haemophilia a without inhibitor	[[1,0,0,0,0,0,0,0,0,0,0,0,0,0],[1,0,0,0,0,0,0,0,0,0,0,0,0,0],[1,0,0,0,0,0,0,0,0,0,0,0,0,0]]C0571250	hypromellose eye drops allergy	[[1,0,0,0,0,0,0,0,0,0,0,0,0,0]]C1275100	keratoderma with deafness	[[1,0,0,0,0,0,0,0,0,0,0,0,0,0]]C2981679	stage iiib penile cancer,stage iiib penile cancer ajcc v7	[[0,0,0,0,0,0,0,0,0,0,0,1,0,0],[0,0,0,0,0,0,0,0,0,0,0,1,0,0]]C1290712	sequestrum of the mandible	[[1,0,0,0,0,0,0,0,0,0,0,0,0,0]]C1282191	postoperative uveitis	[[1,0,0,0,0,0,0,0,0,0,0,0,0,0]]C1847399	nocturnal faciomandibular myoclonus,nocturnal facio mandibular myoclonus,sleep bruxism type parasomnia,psmnsb	[[0,1,0,1,0,0,0,0,0,0,0,0,0,0],[0,1,0,0,0,0,0,0,0,0,0,0,0,0],[0,0,0,1,0,0,0,0,0,0,0,0,0,0],[0,0,0,1,0,0,0,0,0,0,0,0,0,0]]C1335256	pretext stage 2 hepatoblastoma	[[0,0,0,0,0,0,0,0,0,0,0,1,0,0]]C1335257	pretext stage 3 hepatoblastoma	[[0,0,0,0,0,0,0,0,0,0,0,1,0,0]]C3151355	megalencephalic leukoencephalopathy with subcortical cysts 2a,mlc2a	[[0,0,0,1,0,0,0,0,0,0,0,0,0,0],[0,0,0,1,0,0,0,0,0,0,0,0,0,0]]C1408081	calcification of uterus	[[1,0,0,0,0,0,0,0,0,0,0,0,0,0]]C0231341	premature aging syndrome,physiologically old for age,premature aging	[[1,0,0,0,0,0,0,1,0,0,0,0,0,0],[1,0,0,0,0,0,0,0,0,0,0,0,0,0],[1,0,0,1,0,0,0,1,0,1,0,0,1,0]]C0281393	aids related diffuse mixed cell lymphoma,aids associated diffuse mixed cell lymphoma	[[0,0,0,0,0,1,0,0,0,0,0,1,0,0],[0,0,0,0,0,1,0,0,0,0,0,1,0,0]]C0267291	acute peptic ulcer with perforation	[[1,0,0,0,0,0,0,0,0,0,0,0,0,0]]C1862140	brachydactyly type a3,bda3,brachydactyly clinodactyly,brachymesophalangy v,brachymesophalangy 5	[[0,1,0,1,0,0,0,0,0,0,0,0,0,0],[0,0,0,1,0,0,0,0,0,0,0,0,0,0],[0,1,0,1,0,0,0,0,0,0,0,0,0,0],[0,0,0,1,0,0,0,0,0,0,0,0,0,0],[0,1,0,0,0,0,0,0,0,0,0,0,0,0]]C1389527	relaxation of pelvic floor	[[1,0,0,0,0,0,0,0,0,0,0,0,0,0]]C2673257	progressive myoclonic 3 epilepsy,epm3	[[0,0,0,1,0,0,0,0,0,0,0,0,0,0],[0,0,0,1,0,0,0,0,0,0,0,0,0,0]]C0749474	thyroid nodule solitary	[[0,0,0,0,0,0,0,1,0,0,0,0,0,0]]C0345137	balanced coronary system	[[1,0,0,0,0,0,0,0,0,0,0,0,0,0]]C0345136	left dominant coronary system	[[1,0,0,0,0,0,0,0,0,0,0,0,0,0]]C0345135	right dominant coronary system	[[1,0,0,0,0,0,0,0,0,0,0,0,0,0]]C2217549	malignant neoplasm of small intestine tnm staging primary tumor t t1	[[0,0,0,0,0,0,1,0,0,0,0,0,0,0]]C0743515	endometrial polyp benign	[[0,0,0,0,0,0,0,1,0,0,0,0,0,0]]C0345138	pericardial anomaly,defect pericardium,pericardial defect	[[1,0,0,0,0,0,0,0,0,0,0,0,0,0],[0,0,0,0,0,0,0,0,0,0,1,0,0,0],[1,0,0,0,0,0,0,0,0,0,1,0,0,0]]C0749470	thyroid cold nodule	[[0,0,0,0,0,0,1,1,0,0,0,0,0,0]]C0002312	alpha thalassemia,alpha thalassaemia,hemoglobin h disease,alpha thalassemia syndrome,alpha thalassaemia syndrome,a thalassemia	[[0,1,0,1,1,0,0,1,0,0,0,1,1,0],[1,0,0,0,0,0,0,1,0,0,0,0,0,0],[0,1,0,0,0,0,0,0,0,0,0,0,1,0],[1,0,0,0,0,0,0,0,0,0,0,0,0,0],[1,0,0,0,0,0,0,0,0,0,0,0,0,0],[0,1,0,0,0,0,0,0,0,0,0,0,1,0]]C1857808	19 insulin dependent diabetes mellitus,iddm19	[[0,0,0,1,0,0,1,0,0,0,0,0,0,0],[0,0,0,1,0,0,0,0,0,0,0,0,0,0]]C1857809	autosomal recessive 44 deafness,dfnb44	[[0,0,0,1,0,0,0,0,0,0,0,0,0,0],[0,0,0,1,0,0,0,0,0,0,0,0,0,0]]C0031118	peripheral nervous system neoplasms,peripheral nerve neoplasms,peripheral nerve tumors,tumour of peripheral nerve,neoplasm of pns,tumor of the peripheral nervous system,tumor of the pns	[[0,0,0,0,0,0,0,1,0,0,0,1,1,0],[1,1,0,0,0,0,0,1,0,0,0,1,1,0],[1,1,0,0,0,0,0,1,0,0,0,1,1,0],[1,0,0,0,0,0,0,1,0,0,0,0,0,0],[0,0,0,0,0,0,0,0,0,0,0,1,0,0],[0,0,0,0,0,0,0,0,0,0,0,1,0,0],[0,0,0,0,0,0,0,0,0,0,0,1,0,0]]C0031117	peripheral neuropathy,peripheral nerve diseases,peripheral nervous system disorders,pns diseases,peripheral nervous system diseases,peripheral nerve disorders,pns peripheral nervous system diseases	[[0,1,1,1,0,0,0,1,1,0,0,0,1,0],[1,1,0,0,0,0,1,1,0,0,0,0,0,0],[1,0,0,0,1,0,0,0,0,1,0,1,1,0],[0,1,0,0,0,0,0,0,0,0,0,0,1,0],[0,0,0,0,0,0,0,0,0,0,0,0,1,0],[0,0,0,0,0,0,0,1,0,0,0,0,0,0],[0,0,0,0,0,0,0,0,0,0,0,0,1,0]]C1857802	morm syndrome,retinal dystrophy and micropenis truncal obesity mental retardation	[[0,1,0,1,0,0,0,0,0,0,0,0,0,0],[0,1,0,1,0,0,0,0,0,0,0,0,0,0]]C1274299	diabetic wet gangrene of the foot	[[1,0,0,0,0,0,0,0,0,0,0,0,0,0]]C0865176	adrenal hyperplasia due to excess acth	[[0,0,0,0,0,0,0,0,0,0,0,0,0,1]]C0031111	periostitis,periostitides,periosteitis fibrosa	[[0,0,1,0,0,0,0,1,1,0,0,0,0,0],[0,1,0,0,0,0,0,0,0,0,0,0,0,0],[1,0,0,0,0,0,0,0,0,0,0,0,0,0]]C1444199	alkaptonuric ochronosis,alcaptonuric ochronosis,ochronosis due to alkaptonuria,pigmentation due to alkaptonuria,ochronosis due to homogentisate 1 2 dioxygenase deficiency,exogenous ochronosis,ocular ochronosis,pseudo ochronosis	[[1,0,0,0,0,0,0,0,0,0,0,0,0,0],[1,0,0,0,0,0,0,0,0,0,0,0,0,0],[1,0,0,0,0,0,0,0,0,0,0,0,0,0],[1,0,0,0,0,0,0,0,0,0,0,0,0,0],[1,0,0,0,0,0,0,0,0,0,0,0,0,0],[0,1,0,0,0,0,0,0,0,0,0,0,0,0],[0,1,0,0,0,0,0,0,0,0,0,0,0,0],[0,1,0,0,0,0,0,0,0,0,0,0,0,0]]C1274392	mycobacterium chelonae infection of skin	[[1,0,0,0,0,0,0,0,0,0,0,0,0,0]]C0034544	radiculitis,radiculitides,nerve root inflammation	[[0,0,1,0,0,0,0,1,0,0,0,0,0,0],[0,1,0,0,0,0,0,0,0,0,0,0,0,0],[0,1,0,0,0,0,0,1,0,0,0,0,0,0]]C0034543	radicular cyst,periapical cyst,apical periodontal cyst,apical cyst,residual radicular cyst,radiculodental cyst,apical radicular cyst,periradicular cyst	[[0,1,0,0,0,0,0,1,0,0,0,0,1,0],[0,1,0,0,0,0,0,1,0,0,0,0,1,1],[0,1,0,0,0,0,0,0,0,0,0,0,1,1],[1,0,0,0,0,0,0,1,0,0,0,0,0,0],[0,0,0,0,0,0,0,0,0,0,0,0,0,1],[0,0,0,0,0,0,0,0,0,0,0,0,0,1],[1,0,0,0,0,0,0,0,0,0,0,0,0,0],[1,0,0,0,0,0,0,1,0,0,0,0,0,0]]C0451940	localized skin eruption due to drugs and medicaments,localised skin eruption due to drugs and medicaments	[[1,0,0,0,0,0,0,0,0,0,0,0,0,0],[1,0,0,0,0,0,0,0,0,0,0,0,0,0]]C0865171	parathyroiditis	[[0,0,0,0,0,0,0,1,0,0,0,0,0,1]]C0040560	congenital toxoplasmosis,congenital toxoplasmoses,congenital toxoplasma gondii infection,congenital toxoplasma infections	[[0,0,0,0,0,0,0,1,0,0,1,1,1,1],[0,1,0,0,0,0,0,0,0,0,0,0,0,0],[0,0,0,0,0,0,0,0,0,0,0,0,1,0],[0,1,0,0,0,0,0,0,0,0,0,0,1,0]]C0040561	ocular toxoplasmosis,ocular toxoplasmoses	[[1,1,0,0,0,0,0,1,0,0,0,0,0,0],[0,1,0,0,0,0,0,0,0,0,0,0,0,0]]C0038437	female stress incontinence,female urinary stress incontinence	[[1,0,0,0,1,0,0,1,0,0,0,1,0,0],[1,0,0,0,0,0,0,0,0,0,0,0,0,0]]C1282218	benign neoplasm lacrimal sac	[[1,0,0,0,0,0,0,0,0,0,0,0,0,1]]C1855536	autosomal recessive larsen syndrome	[[0,0,0,1,0,0,0,0,0,0,0,0,0,0]]C1855535	lethal type larsen like syndrome,larsen like multiple joint dislocation syndrome	[[0,1,0,1,0,0,0,0,0,0,0,0,0,0],[0,1,0,0,0,0,0,0,0,0,0,0,0,0]]C1274395	mycobacterium abscessus infection of skin	[[1,0,0,0,0,0,0,0,0,0,0,0,0,0]]C3151211	type x osteogenesis imperfecta,type x oi,oi10	[[0,0,0,1,0,0,0,0,0,0,0,0,0,0],[0,0,0,1,0,0,0,0,0,0,0,0,0,0],[0,0,0,1,0,0,0,0,0,0,0,0,0,0]]C0007361	cat scratch disease,benign inoculation lymphoreticulosis,cat scratch fever,inoculation lymphoreticulosis,inoculation lymphoreticuloses,catscratch disease,benign lymphoreticulosis,csd cat scratch disease	[[1,1,0,0,1,0,0,1,0,0,1,0,0,0],[1,0,0,0,0,0,0,0,0,0,1,0,0,1],[0,0,0,0,0,0,0,1,0,0,1,0,0,1],[0,1,0,0,0,0,0,0,0,0,0,0,1,0],[0,1,0,0,0,0,0,0,0,0,0,0,0,0],[0,1,0,0,0,0,0,1,0,0,0,0,1,0],[0,0,0,0,0,0,0,0,0,0,0,0,0,1],[1,0,0,0,0,0,0,0,0,0,0,0,0,0]]C0749389	pharyngeal lesion,throat lesion	[[0,0,0,0,0,0,1,0,0,0,0,0,0,0],[0,0,0,0,0,0,0,1,0,0,0,0,0,0]]C3151219	fucosyltransferase 6 deficiency	[[0,0,0,1,0,0,0,0,0,0,0,0,0,0]]C0265541	cranioschisis,cranioschises,cranium bifidum	[[1,0,0,0,0,0,0,1,0,0,0,0,0,0],[0,1,0,0,0,0,0,0,0,0,0,0,0,0],[0,0,0,0,0,0,0,1,0,0,0,0,0,0]]C1412002	atypical pneumonia	[[1,0,0,0,0,0,0,0,0,1,1,0,0,0]]C2739810	lentigo maligna melanoma,malignant melanoma in hutchinsons melanotic freckle,lmm lentigo maligna melanoma,m malignant melanoma in hutchinsons melanotic freckle,malignant lentigo melanoma	[[1,0,0,0,0,0,0,0,0,0,0,1,0,0],[1,0,0,0,0,0,0,0,0,0,0,0,0,0],[1,0,0,0,0,0,0,0,0,0,0,0,0,0],[1,0,0,0,0,0,0,0,0,0,0,0,0,0],[0,0,0,0,0,0,0,0,0,0,0,1,0,0]]C0266012	ectopic breast tissue,heterotopic breast tissue	[[1,0,0,0,0,0,0,0,0,0,0,0,0,0],[1,0,0,0,0,0,0,0,0,0,0,0,0,0]]C1274394	mycobacterium malmoense infection of skin	[[1,0,0,0,0,0,0,0,0,0,0,0,0,0]]C0571259	tolnaftate allergy	[[1,0,0,0,0,0,0,0,0,0,0,0,0,0]]C0585552	microflutter of eye	[[1,0,0,0,0,0,0,0,0,0,0,0,0,0]]C1336960	very low risk gastrointestinal stromal tumor,vlr gist	[[0,0,0,0,0,0,0,0,0,0,0,1,0,0],[0,0,0,0,0,0,0,0,0,0,0,1,0,0]]C0269673	renal disease in pregnancy and or puerperium without hypertension,nephropathy in pregnancy and or puerperium without hypertension	[[1,0,0,0,0,0,0,0,0,0,0,0,0,0],[1,0,0,0,0,0,0,0,0,0,0,0,0,0]]C0269675	albuminuria in pregnancy without hypertension	[[1,0,0,0,0,0,0,0,0,0,0,0,0,0]]C0269677	icterus gravis of pregnancy,acute yellow atrophy of liver during pregnancy	[[0,0,0,0,0,0,0,0,0,0,0,0,0,1],[1,0,0,0,0,0,0,0,0,0,0,0,0,1]]C0269676	necrosis of liver of pregnancy	[[1,0,0,0,0,0,0,0,0,0,0,0,0,0]]C0344260	focal marginal corneal ulcer	[[1,0,0,0,0,0,0,0,0,0,0,0,0,0]]C2931376	hapnes boman skeie syndrome	[[0,1,0,0,0,0,0,0,0,0,0,0,0,0]]C1333448	esophageal granular cell tumor,esophageal granular cell myoblastoma,esophageal granular cell neoplasm,granular cell myoblastoma of the esophagus,granular cell neoplasm of the esophagus,granular cell tumor of the esophagus	[[0,0,0,0,0,0,0,0,0,0,0,1,0,0],[0,0,0,0,0,0,0,0,0,0,0,1,0,0],[0,0,0,0,0,0,0,0,0,0,0,1,0,0],[0,0,0,0,0,0,0,0,0,0,0,1,0,0],[0,0,0,0,0,0,0,0,0,0,0,1,0,0],[0,0,0,0,0,0,0,0,0,0,0,1,0,0]]C2931377	tollner horst manzke syndrome,heptacarpo octatarso dactyly combined with multiple malformation	[[0,1,0,0,0,0,0,0,0,0,0,0,0,0],[0,1,0,0,0,0,0,0,0,0,0,0,0,0]]C0267945	chronic fibrosing pancreatitis	[[1,0,0,0,0,0,0,0,0,0,0,0,0,0]]C0267947	interstitial pancreatitis	[[1,0,0,0,0,0,0,0,0,0,0,0,0,0]]C0267946	infectious pancreatitis	[[1,0,0,0,0,0,0,0,0,0,0,0,0,0]]C0267941	acute necrotizing pancreatitis,acute necrotising pancreatitis,necrotizing pancreatitis,necrotising pancreatitis	[[1,0,0,0,0,0,0,1,0,0,0,0,1,0],[1,0,0,0,0,0,0,1,0,0,0,0,0,0],[1,0,0,0,0,0,0,1,1,0,0,0,0,0],[1,0,0,0,0,0,0,1,0,0,0,0,0,0]]C0267940	acute hemorrhagic pancreatitis,acute haemorrhagic pancreatitis,hemorrhagic pancreatitis,haemorrhagic pancreatitis	[[1,0,0,0,0,0,0,1,0,0,0,0,0,0],[1,0,0,0,0,0,0,0,0,0,0,0,0,0],[1,0,0,0,0,0,0,1,1,0,0,0,0,1],[1,0,0,0,0,0,0,0,0,0,0,0,0,0]]C0007099	carcinoma in situ,intraepithelial carcinoma,carcinoma in situ morphology,cis,preinvasive carcinoma,no icd o subtype carcinoma in situ,non invasive carcinoma,stage 0 disease	[[1,1,0,0,1,0,0,1,0,0,0,1,0,1],[1,1,0,0,0,0,0,1,0,0,0,0,1,0],[1,0,0,0,0,0,0,0,0,0,0,0,0,0],[0,0,0,0,0,0,0,0,0,0,0,1,0,0],[0,1,0,0,0,0,0,0,0,0,0,0,1,0],[1,0,0,0,0,0,0,0,0,0,0,0,0,0],[0,0,0,0,0,0,0,0,0,0,0,1,0,0],[0,0,0,0,0,0,0,0,0,0,0,1,0,0]]C0267942	apoplectic pancreatitis	[[1,0,0,0,0,0,0,0,0,0,0,0,0,1]]C0007097	carcinoma,malignant epithelial tumors,malignant epithelial tumour,epithelioma,malignant epithelioma,malignant epithelial neoplasms,epithelial carcinoma,no subtype carcinoma	[[0,1,1,0,0,0,0,1,0,0,0,0,0,0],[1,1,0,0,0,0,0,0,0,0,0,0,1,0],[1,0,0,0,0,0,0,0,0,0,0,0,0,0],[0,1,0,0,0,0,0,1,0,0,0,0,1,0],[0,0,0,0,0,0,0,0,0,0,0,1,0,0],[0,1,0,0,0,0,0,0,0,0,0,0,1,0],[0,0,0,0,0,0,0,0,0,0,0,1,0,0],[1,0,0,0,0,0,0,0,0,0,0,0,0,0]]C0007095	carcinoid tumor,carcinoid tumour,carcinoid,m carcinoid tumors,m carcinoid tumours,carcinoid tumor morphology,carcinoid tumour morphology,well differentiated endocrine neoplasm,carcinoid neoplasm	[[1,1,0,1,0,0,0,1,0,0,0,0,0,1],[1,0,0,0,0,0,0,1,0,0,0,0,0,0],[0,1,1,0,0,0,0,1,0,0,0,0,1,0],[1,0,0,0,0,0,0,0,0,0,0,0,0,0],[1,0,0,0,0,0,0,0,0,0,0,0,0,0],[1,0,0,0,0,0,0,0,0,0,0,0,0,0],[1,0,0,0,0,0,0,0,0,0,0,0,0,0],[0,0,0,0,0,0,0,0,0,0,0,1,0,0],[0,0,0,0,0,0,0,0,0,0,0,1,0,0]]C2931375	temporomandibular ankylosis,ankylosis of the temporomandibular joint	[[0,1,0,0,0,0,0,0,0,0,0,0,0,0],[0,1,0,0,0,0,0,0,0,0,0,0,0,0]]C0007093	carcinoid heart disease	[[0,1,0,0,0,0,0,0,0,0,0,0,1,0]]C0267948	metabolic pancreatitis	[[1,0,0,0,0,0,0,0,0,0,0,0,0,0]]C0271948	megaloblastic anemia due to pregnancy,megaloblastic anaemia due to pregnancy	[[1,0,0,0,0,0,0,0,0,0,0,0,0,0],[1,0,0,0,0,0,0,0,0,0,0,0,0,0]]C0271949	megaloblastic anemia due to chronic hemolytic anemia,megaloblastic anaemia due to chronic haemolytic anaemia	[[1,0,0,0,0,0,0,0,0,0,0,0,0,0],[1,0,0,0,0,0,0,0,0,0,0,0,0,0]]C0271942	goats milk anemia,goats milk anaemia	[[1,0,0,0,0,0,0,0,0,0,0,0,0,1],[1,0,0,0,0,0,0,0,0,0,0,0,0,0]]C0271943	megaloblastic anemia due to impaired absorption of folate,megaloblastic anaemia due to impaired absorption of folate	[[1,0,0,0,0,0,0,0,0,0,0,0,0,0],[1,0,0,0,0,0,0,0,0,0,0,0,0,0]]C0271940	megaloblastic anemia due to hemodialysis,megaloblastic anaemia due to haemodialysis	[[1,0,0,0,0,0,0,0,0,0,0,0,0,0],[1,0,0,0,0,0,0,0,0,0,0,0,0,0]]C0271941	megaloblastic anemia of premature infant,megaloblastic anaemia of premature infant,nutritional megaloblastic anemia of infancy,nutritional megaloblastic anaemia of infancy	[[1,0,0,0,0,0,0,0,0,0,0,0,0,0],[1,0,0,0,0,0,0,0,0,0,0,0,0,0],[0,0,0,0,0,0,0,0,0,0,0,0,0,1],[1,0,0,0,0,0,0,0,0,0,0,0,0,0]]C0271946	megaloblastic anemia due to disease of small intestine,megaloblastic anaemia due to disease of small intestine,megaloblastic anemia due to ileal disease,megaloblastic anaemia due to ileal disease	[[1,0,0,0,0,0,0,0,0,0,0,0,0,0],[1,0,0,0,0,0,0,0,0,0,0,0,0,0],[1,0,0,0,0,0,0,0,0,0,0,0,0,0],[1,0,0,0,0,0,0,0,0,0,0,0,0,0]]C0271947	megaloblastic anemia due to increased requirements,megaloblastic anaemia due to increased requirements	[[1,0,0,0,0,0,0,0,0,0,0,0,0,0],[1,0,0,0,0,0,0,0,0,0,0,0,0,0]]C0271944	megaloblastic anemia due to tropical sprue,megaloblastic anaemia due to tropical sprue	[[1,0,0,0,0,0,0,0,0,0,0,0,0,0],[1,0,0,0,0,0,0,0,0,0,0,0,0,0]]C0271945	megaloblastic anemia due to nontropical sprue,megaloblastic anaemia due to nontropical sprue	[[1,0,0,0,0,0,0,0,0,0,0,0,0,0],[1,0,0,0,0,0,0,0,0,0,0,0,0,0]]C1955684	marginal zone lymphoma involving intra abdominal lymph nodes	[[0,0,0,0,1,0,0,0,0,0,0,0,0,0]]C2931536	methylmalonyl coenzyme a mutase deficiency	[[0,1,0,0,0,0,0,0,0,0,0,0,0,0]]C0585256	infected femorofemoral crossover graft	[[1,0,0,0,0,0,0,0,0,0,0,0,0,0]]C1336219	stage iiib cervical carcinoma,ajcc stage iiib cervical cancer,stage iiib cervical cancer,figo stage iiib cervical carcinoma,figo stage iiib cervix carcinoma,figo stage iiib uterine cervix carcinoma,figo stage iiib cervix uteri carcinoma,stage iiib cervical cancer ajcc v7,stage iiib cervical cancer ajcc v6	[[0,0,0,0,0,0,1,0,0,0,0,0,0,0],[0,0,0,0,0,0,0,0,0,0,0,1,0,0],[0,0,0,0,0,0,0,0,0,0,0,1,0,0],[0,0,0,0,0,0,0,0,0,0,0,1,0,0],[0,0,0,0,0,0,0,0,0,0,0,1,0,0],[0,0,0,0,0,0,0,0,0,0,0,1,0,0],[0,0,0,0,0,0,0,0,0,0,0,1,0,0],[0,0,0,0,0,0,0,0,0,0,0,1,0,0],[0,0,0,0,0,0,0,0,0,0,0,1,0,0]]C0037018	shwartzman phenomenon,schwartzman reaction,shwartzman reaction,function shwartzman reaction,schwartzman phenomenon	[[0,1,0,0,0,0,0,0,0,1,0,0,1,0],[0,1,1,0,0,0,0,0,0,0,0,0,1,0],[1,0,0,0,0,0,0,0,0,0,0,0,1,0],[1,0,0,0,0,0,0,0,0,0,0,0,0,0],[0,1,0,0,0,0,0,0,0,0,0,0,1,0]]C0037019	shy drager syndrome,orthostatic hypotension dysautonomic syndrome,multiple system atrophy,progressive autonomic failure,shy drager type idiopathic orthostatic hypotension,dysautonomia orthostatic hypotension syndrome,dysautonomic orthostatic hypotension	[[0,1,0,0,0,0,0,1,0,1,1,0,0,1],[1,0,0,0,0,0,0,0,0,0,0,0,0,0],[0,0,0,0,0,0,0,0,0,0,0,1,0,0],[0,1,0,0,0,0,0,0,0,0,0,0,1,0],[0,1,0,0,0,0,0,0,0,0,0,0,1,0],[0,1,0,0,0,0,0,0,0,0,0,0,1,0],[0,1,0,0,0,0,0,0,0,0,0,0,1,0]]C0549410	palmar plantar erythrodysesthesia syndrome,chemotherapy induced acral erythema,chemotherapy induced palmoplantar erythrodysesthesia,hand foot syndrome,palmar plantar erythodysthesia,palmar plantar erythrodysthesia,palmar plantar erythrodysesthesia	[[0,0,0,0,0,0,0,0,0,0,0,1,0,0],[0,1,0,0,0,0,0,0,0,0,0,0,1,0],[0,1,0,0,0,0,0,0,0,0,0,0,1,0],[0,1,0,0,0,0,0,0,0,0,0,1,1,0],[0,0,0,0,0,0,0,0,0,0,0,1,0,0],[0,0,0,0,0,0,0,0,0,0,0,1,0,0],[0,0,0,0,0,1,0,0,0,0,0,0,0,0]]C1336216	stage iiia squamous cell lung carcinoma,stage iiia squamous cell lung carcinoma ajcc v7	[[0,0,0,0,0,0,0,0,0,0,0,1,0,0],[0,0,0,0,0,0,0,0,0,0,0,1,0,0]]C1336211	stage iiia lung carcinoma,stage iiia lung carcinoma ajcc v7	[[0,0,0,0,0,0,0,0,0,0,0,1,0,0],[0,0,0,0,0,0,0,0,0,0,0,1,0,0]]C0549414	blast cell proliferation	[[0,0,0,0,0,0,0,0,0,0,0,1,0,0]]C1336212	stage iiia ovarian carcinoma,ajcc stage iiia ovarian cancer,figo stage iiia ovarian cancer,figo stage iiia cancer of the ovary,stage iiia ovarian cancer,figo stage iiia ovarian carcinoma,figo stage iiia carcinoma of the ovary,stage iiia ovarian cancer ajcc v6,stage iiia ovarian cancer ajcc v7	[[0,0,0,0,0,0,1,0,0,0,0,0,0,0],[0,0,0,0,0,0,0,0,0,0,0,1,0,0],[0,0,0,0,0,0,0,0,0,0,0,1,0,0],[0,0,0,0,0,0,0,0,0,0,0,1,0,0],[0,0,0,0,0,0,0,0,0,0,0,1,0,0],[0,0,0,0,0,0,0,0,0,0,0,1,0,0],[0,0,0,0,0,0,0,0,0,0,0,1,0,0],[0,0,0,0,0,0,0,0,0,0,0,1,0,0],[0,0,0,0,0,0,0,0,0,0,0,1,0,0]]C0263016	inflammatory hyperkeratotic dermatosis	[[1,0,0,0,0,0,0,0,0,0,0,0,0,0]]C0598338	myelodysplastic anemia	[[0,0,0,0,0,0,0,1,0,0,0,0,0,0]]C3164261	tripartite right ventricle	[[1,0,0,0,0,0,0,0,0,0,0,0,0,0]]C0263015	panniculitis affecting sacrum	[[1,0,0,0,0,0,0,0,0,0,0,0,0,0]]C2350171	bilateral nasal obstruction	[[0,1,0,0,0,0,0,0,0,0,0,0,0,0]]C3164264	infracardiac location of anomalous pulmonary venous connection	[[1,0,0,0,0,0,0,0,0,0,0,0,0,0]]C0341726	periureteritis	[[0,0,0,0,0,0,0,0,0,0,0,0,0,1]]C0341724	schistosomiasis of ureter,bilharzia of ureter	[[1,0,0,0,0,0,0,0,0,0,0,0,0,0],[1,0,0,0,0,0,0,0,0,0,0,0,0,0]]C0036946	sheep diseases,ovine diseases	[[0,1,0,0,0,0,0,0,0,0,0,0,0,0],[0,1,0,0,0,0,0,0,0,0,0,0,0,0]]C0686698	benign neoplasm of paravaginal lymph nodes	[[1,0,0,0,0,0,0,0,0,0,0,0,0,0]]C0686699	secondary malignant neoplasm of paravaginal lymph nodes,metastatic malignant neoplasm to paravaginal lymph nodes	[[1,0,0,0,0,0,0,0,0,0,0,0,0,0],[1,0,0,0,0,0,0,0,0,0,0,0,0,0]]C0686694	benign neoplasm of hypogastric lymph nodes	[[1,0,0,0,0,0,0,0,0,0,0,0,0,0]]C0263013	panniculitis of neck,panniculitis specified as affecting neck,panniculitis affecting neck	[[1,0,0,0,0,0,0,0,0,0,0,0,0,0],[0,0,0,0,1,0,0,0,0,0,0,0,0,0],[1,0,0,0,0,0,0,0,0,0,0,0,0,0]]C0686696	benign neoplasm of parametrial lymph nodes	[[1,0,0,0,0,0,0,0,0,0,0,0,0,0]]C0686697	secondary malignant neoplasm of parametrial lymph nodes,metastatic malignant neoplasm to parametrial lymph nodes	[[1,0,0,0,0,0,0,0,0,0,0,0,0,0],[1,0,0,0,0,0,0,0,0,0,0,0,0,0]]C0686690	benign neoplasm of iliac lymph nodes	[[1,0,0,0,0,0,0,0,0,0,0,0,0,0]]C0686691	secondary malignant neoplasm of iliac lymph nodes,metastatic malignant neoplasm to iliac lymph nodes	[[1,0,0,0,0,0,0,0,0,0,0,0,0,0],[1,0,0,0,0,0,0,0,0,0,0,0,0,0]]C0686692	benign neoplasm of obturator lymph nodes	[[1,0,0,0,0,0,0,0,0,0,0,0,0,0]]C0686693	secondary malignant neoplasm of obturator lymph nodes,metastatic malignant neoplasm to obturator lymph nodes	[[1,0,0,0,0,0,0,0,0,0,0,0,0,0],[1,0,0,0,0,0,0,0,0,0,0,0,0,0]]C0153833	leukemic reticuloendotheliosis of lymph nodes of multiple sites,leukaemic reticuloendotheliosis of lymph nodes of multiple sites,leukemic reticuloendotheliosis involving lymph nodes of multiple sites	[[1,0,0,0,0,0,0,0,0,0,0,0,0,0],[1,0,0,0,0,0,0,0,0,0,0,0,0,0],[0,0,0,0,1,0,0,0,0,0,0,0,0,0]]C0153832	hairy cell leukemia of spleen,hairy cell leukaemia of spleen,leukemic reticuloendotheliosis involving spleen,leukemic reticuloendotheliosis of spleen,leukaemic reticuloendotheliosis of spleen	[[1,0,0,0,0,0,0,0,0,0,0,0,0,0],[1,0,0,0,0,0,0,0,0,0,0,0,0,0],[0,0,0,0,1,0,0,0,0,0,0,0,0,0],[1,0,0,0,0,0,0,0,0,0,0,0,0,0],[1,0,0,0,0,0,0,0,0,0,0,0,0,0]]C0411015	pseudomonas pyocyaneus congenital infection	[[1,0,0,0,0,0,0,0,0,0,0,0,0,0]]C0411017	congenital hepatitis a infection	[[1,0,0,0,0,0,0,0,0,0,0,0,0,0]]C0153831	leukemic reticuloendotheliosis of intrapelvic lymph nodes,leukaemic reticuloendotheliosis of intrapelvic lymph nodes,leukemic reticuloendotheliosis involving intrapelvic lymph nodes	[[1,0,0,0,0,0,0,0,0,0,0,0,0,0],[1,0,0,0,0,0,0,0,0,0,0,0,0,0],[0,0,0,0,1,0,0,0,0,0,0,0,0,0]]C0411012	clostridial intra amniotic fetal infection	[[1,0,0,0,0,0,0,0,0,0,0,0,0,0]]C0411013	eschericha coli intra amniotic fetal infection	[[1,0,0,0,0,0,0,0,0,0,0,0,0,0]]C0153830	leukemic reticuloendotheliosis of lymph nodes of inguinal region and or lower limb,leukaemic reticuloendotheliosis of lymph nodes of inguinal region and or lower limb,leukemic reticuloendotheliosis involving lymph nodes of inguinal region and lower limb	[[1,0,0,0,0,0,0,0,0,0,0,0,0,0],[1,0,0,0,0,0,0,0,0,0,0,0,0,0],[0,0,0,0,1,0,0,0,0,0,0,0,0,0]]C0409910	gout secondary to drug,drug induced gout	[[1,0,0,0,0,0,0,0,0,0,0,0,0,0],[1,0,0,0,0,0,0,0,0,0,0,0,0,0]]C2315692	histoplasmosis of spinal cord,infection of spinal cord due to histoplasma	[[1,0,0,0,0,0,0,0,0,0,0,0,0,0],[1,0,0,0,0,0,0,0,0,0,0,0,0,0]]C0019359	visceral herpes simplex	[[1,0,0,0,1,0,0,0,0,0,0,0,0,0]]C0795888	chromosome xp22 deletion syndrome,autsx4,4 susceptibility to x linked autism	[[0,0,0,1,0,0,0,0,0,0,0,0,0,0],[0,0,0,1,0,0,0,0,0,0,0,0,0,0],[0,0,0,1,0,0,0,0,0,0,0,0,0,0]]C0795889	allan herndon dudley syndrome,allan herndon syndrome,ahds,x linked mental retardation with hypotonia,mental retardation and muscular atrophy,monocarboxylate transporter 8 deficiency,t3 resistance,triiodothyronine resistance,t3 resisitence	[[0,1,0,1,0,0,1,0,0,0,0,0,0,0],[0,1,0,1,0,0,0,0,0,0,0,0,0,0],[0,0,0,1,0,0,0,0,0,0,0,0,0,0],[0,1,0,1,0,0,0,0,0,0,0,0,0,0],[0,1,0,1,0,0,0,0,0,0,0,0,0,0],[0,1,0,1,0,0,0,0,0,0,0,0,0,0],[0,0,0,1,0,0,0,0,0,0,0,0,0,0],[0,1,0,1,0,0,0,0,0,0,0,0,0,0],[0,1,0,0,0,0,0,0,0,0,0,0,0,0]]C1970897	quantitative trait locus 1 age at natural menopause,menoq1	[[0,0,0,1,0,0,0,0,0,0,0,0,0,0],[0,0,0,1,0,0,0,0,0,0,0,0,0,0]]C0795887	chromosome xp21 deletion syndrome,complex glycerol kinase deficiency	[[0,0,0,1,0,0,0,0,0,0,0,0,0,0],[0,0,0,1,0,0,0,0,0,0,0,0,0,0]]C0409912	gout secondary to enzyme defect	[[1,0,0,0,0,0,0,0,0,0,0,0,0,0]]C0795880	22q deletion syndrome,22q syndrome	[[0,0,0,0,0,0,0,1,0,0,0,0,0,0],[0,0,0,0,0,0,0,1,0,0,0,0,0,0]]C2315694	bilateral sensory hearing loss	[[1,0,0,0,0,0,0,0,0,0,0,0,0,0]]C0019357	herpetic keratitis,herpetic keratitides,ocular herpes simplex,herpes simplex keratitis,herpes simplex keratitides,ocular herpes,herpes keratitis,ophthalmic herpes simplex,corneal herpes,herpes ophthalmic,eye herpes,hsv keratitis	[[1,1,0,0,0,0,0,1,0,0,0,0,1,0],[0,1,0,0,0,0,0,0,0,0,0,0,0,0],[0,0,0,0,0,0,0,1,0,0,0,0,1,0],[1,1,0,0,0,0,0,1,0,0,0,0,1,0],[0,1,0,0,0,0,0,0,0,0,0,0,0,0],[0,0,0,0,0,0,0,1,0,0,0,0,0,0],[0,0,0,0,0,0,0,1,0,0,0,0,0,0],[1,0,0,0,0,0,0,0,0,0,0,0,0,0],[0,0,0,0,0,0,0,1,0,0,0,0,0,0],[0,0,0,0,0,0,0,1,0,0,0,0,0,0],[0,0,0,0,0,0,0,1,0,0,0,0,0,0],[1,0,0,0,0,0,0,1,0,0,0,0,0,0]]C0004842	battered child syndrome	[[0,1,0,0,0,0,0,1,0,0,1,0,0,0]]C1868575	familial recurrent dislocation of patella	[[0,0,0,1,0,0,0,0,0,0,0,0,0,0]]C1336445	stage iv uveal melanoma,stage iv malignant melanoma of the uvea,stage iv uveal malignant melanoma,stage iv melanoma of the uvea,stage iv uveal melanoma ajcc v7	[[0,0,0,0,0,0,0,0,0,0,0,1,0,0],[0,0,0,0,0,0,0,0,0,0,0,1,0,0],[0,0,0,0,0,0,0,0,0,0,0,1,0,0],[0,0,0,0,0,0,0,0,0,0,0,1,0,0],[0,0,0,0,0,0,0,0,0,0,0,1,0,0]]C0153361	malignant neoplasm of sublingual gland,malignant tumor of sublingual gland,malignant tumour of sublingual gland	[[0,0,0,0,1,0,0,0,0,0,0,1,0,0],[1,0,0,0,0,0,0,0,0,0,0,1,0,0],[1,0,0,0,0,0,0,0,0,0,0,0,0,0]]C0153360	malignant neoplasm of submandibular gland,malignant tumor of submandibular gland,malignant tumour of submandibular gland,malignant neoplasm of submaxillary gland	[[1,0,0,0,0,0,0,0,0,0,0,1,0,0],[1,0,0,0,0,0,0,0,0,0,0,1,0,0],[1,0,0,0,0,0,0,0,0,0,0,0,0,0],[0,0,0,0,0,0,0,0,0,0,0,0,0,1]]C1336446	stage iv vaginal cancer,stage iv vaginal carcinoma,stage iv vaginal cancer ajcc v6,stage iv vaginal carcinoma ajcc v6,stage iv vaginal cancer ajcc v7,stage iv vaginal carcinoma ajcc v7	[[0,0,0,0,0,0,0,0,0,0,0,1,0,0],[0,0,0,0,0,0,0,0,0,0,0,1,0,0],[0,0,0,0,0,0,0,0,0,0,0,1,0,0],[0,0,0,0,0,0,0,0,0,0,0,1,0,0],[0,0,0,0,0,0,0,0,0,0,0,1,0,0],[0,0,0,0,0,0,0,0,0,0,0,1,0,0]]C0153365	malignant tumor of upper gingiva,malignant tumour of upper gingiva,malignant neoplasm of upper gum,malignant tumor of upper gum,malignant tumour of upper gum	[[1,0,0,0,0,0,0,0,0,0,0,0,0,0],[1,0,0,0,0,0,0,0,0,0,0,0,0,0],[0,0,0,0,1,0,0,0,0,0,0,0,0,0],[1,0,0,0,0,0,0,0,0,0,0,0,0,0],[1,0,0,0,0,0,0,0,0,0,0,0,0,0]]C0153364	malignant tumor of gum,malignant tumour of gum,malignant gum neoplasm,malignant tumor of alveolar mucosa,malignant tumour of alveolar mucosa,ca cancer of gum,cancer of gum,malignant tumor of gingiva,malignant tumour of gingiva,gingival cancer,malignant neoplasm of gingiva,malignant gingival neoplasm,malignant gingival tumor	[[1,0,0,0,0,0,0,0,0,0,0,1,0,0],[1,0,0,0,0,0,0,0,0,0,0,0,0,0],[0,0,0,0,1,0,0,0,0,0,0,1,0,0],[1,0,0,0,0,0,0,0,0,0,0,0,0,0],[1,0,0,0,0,0,0,0,0,0,0,0,0,0],[1,0,0,0,0,0,0,0,0,0,0,0,0,0],[1,0,0,0,0,0,0,1,0,0,0,0,0,0],[1,0,0,0,0,0,0,0,0,0,0,1,0,0],[1,0,0,0,0,0,0,0,0,0,0,0,0,0],[0,0,0,0,0,0,0,1,0,0,0,0,0,0],[0,0,0,0,0,0,0,0,0,0,0,1,0,0],[0,0,0,0,0,0,0,0,0,0,0,1,0,0],[0,0,0,0,0,0,0,0,0,0,0,1,0,0]]C1302836	lipomatous hamartoma	[[1,0,0,0,0,0,0,0,0,0,0,1,0,0]]C0153369	malignant neoplasm of anterior portion of floor of mouth,malignant tumor of anterior floor of mouth,malignant tumour of anterior floor of mouth	[[1,0,0,0,0,0,0,0,0,0,0,0,0,0],[1,0,0,0,0,0,0,0,0,0,0,0,0,0],[1,0,0,0,0,0,0,0,0,0,0,0,0,0]]C0153368	malignant neoplasm of floor of mouth,ca cancer of floor of mouth,fom cancer of floor of mouth,cancer of floor of mouth,fom malignant tumor of floor of mouth,fom malignant tumour of floor of mouth,malignant tumor of floor of mouth,malignant tumour of floor of mouth	[[0,0,0,0,1,0,0,0,0,0,0,1,0,0],[1,0,0,0,0,0,0,0,0,0,0,0,0,0],[1,0,0,0,0,0,0,0,0,0,0,0,0,0],[1,0,0,0,0,0,0,0,0,0,0,0,0,0],[1,0,0,0,0,0,0,0,0,0,0,0,0,0],[1,0,0,0,0,0,0,0,0,0,0,0,0,0],[1,0,0,0,0,0,0,0,0,0,0,1,0,0],[1,0,0,0,0,0,0,0,0,0,0,0,0,0]]C1302832	benign neuroendocrine neoplasm category	[[1,0,0,0,0,0,0,0,0,0,0,0,0,0]]C1302833	neoplasm with eccrine differentiation	[[1,0,0,0,0,0,0,0,0,0,0,0,0,0]]C1302830	localized epidermolytic epidermal nevus,localised epidermolytic epidermal naevus	[[1,0,0,0,0,0,0,0,0,0,0,0,0,0],[1,0,0,0,0,0,0,0,0,0,0,0,0,0]]C1302831	melanocytic nevus category,melanocytic naevus category	[[1,0,0,0,0,0,0,0,0,0,0,0,0,0],[1,0,0,0,0,0,0,0,0,0,0,0,0,0]]C0267057	alkaline reflux disease,alkaline reflux esophagitis,alkaline reflux oesophagitis,biliary esophagitis,biliary oesophagitis	[[1,0,0,0,0,0,0,0,0,0,0,0,0,0],[1,0,0,0,0,0,0,0,0,0,0,0,0,0],[1,0,0,0,0,0,0,0,0,0,0,0,0,0],[1,0,0,0,0,0,0,0,0,0,0,0,0,0],[1,0,0,0,0,0,0,0,0,0,0,0,0,0]]C0267056	abscess of esophagus,abscess of oesophagus	[[0,0,0,0,0,0,0,0,0,0,0,0,0,1],[1,0,0,0,0,0,0,0,0,0,0,0,0,0]]C0267055	erosive esophagitis,erosive oesophagitis	[[1,0,0,0,0,0,0,1,0,0,0,0,0,0],[1,0,0,0,0,0,0,1,0,0,0,0,0,0]]C0267054	esophagomalacia,oesophagomalacia	[[1,0,0,0,0,0,0,0,0,0,0,0,0,0],[1,0,0,0,0,0,0,0,0,0,0,0,0,0]]C0267053	leukedema of tongue,leukoedema of tongue	[[1,0,0,0,0,0,0,0,0,0,0,0,0,0],[1,0,0,0,0,0,0,0,0,0,0,0,0,0]]C0267052	focal epithelial hyperplasia of tongue	[[1,0,0,0,0,0,0,0,0,0,0,0,0,0]]C0267051	erythroplakia of tongue	[[1,0,0,0,0,0,0,0,0,0,0,0,0,0]]C0267050	bilateral paralysis of tongue	[[1,0,0,0,0,0,0,0,0,0,0,0,0,0]]C0267059	regurgitant esophagitis,regurgitant oesophagitis	[[0,0,0,0,0,0,0,0,0,0,0,0,0,1],[1,0,0,0,0,0,0,0,0,0,0,0,0,0]]C0267058	postoperative esophagitis,postoperative oesophagitis	[[0,0,0,0,0,0,0,0,0,0,0,0,0,1],[1,0,0,0,0,0,0,0,0,0,0,0,0,0]]C0686038	neoplasm of uncertain behavior of vallecula of epiglottis,neoplasm of uncertain behaviour of vallecula of epiglottis,neoplasm of uncertain behavior of vallecula,neoplasm of uncertain behaviour of vallecula	[[1,0,0,0,0,0,0,0,0,0,0,0,0,0],[1,0,0,0,0,0,0,0,0,0,0,0,0,0],[1,0,0,0,0,0,0,0,0,0,0,0,0,0],[1,0,0,0,0,0,0,0,0,0,0,0,0,0]]C0686039	carcinoma in situ of vallecula	[[1,0,0,0,0,0,0,0,0,0,0,0,0,0]]C0157843	infective arthritis of shoulder region	[[1,0,0,0,0,0,0,0,0,0,0,0,0,0]]C0395889	tympanosclerosis involving tympanic membrane and ear ossicles,tympanosclerosis of tympanic membrane and ossicles	[[1,0,0,0,1,0,0,0,0,0,0,0,0,0],[1,0,0,0,0,0,0,0,0,0,0,0,0,0]]C2930941	carrington syndrome,carringtons pulmonary eosinophilia,chronic eosinophilic pneumonia,eosinophilic idiopathic chronic pneumopathy,carringtons disease,chronic idiopathic eosinophilic pneumonia	[[0,1,0,0,0,0,0,0,0,0,0,0,0,0],[0,1,0,0,0,0,0,0,0,0,0,0,0,0],[0,1,0,0,0,0,0,0,0,0,0,0,0,0],[0,1,0,0,0,0,0,0,0,0,0,0,0,0],[0,1,0,0,0,0,0,0,0,0,0,0,0,0],[0,1,0,0,0,0,0,0,0,0,0,0,0,0]]C0686030	secondary malignant neoplasm of uvula,metastatic malignant neoplasm to uvula	[[1,0,0,0,0,0,0,0,0,0,0,0,0,0],[1,0,0,0,0,0,0,0,0,0,0,0,0,0]]C0262666	abscess of vulva,vulvar abscess,vulval abscess	[[0,0,0,0,0,0,0,1,0,0,0,0,0,1],[0,0,1,0,0,0,0,1,0,0,0,0,0,0],[0,0,0,0,0,0,0,1,0,0,0,0,0,0]]C0686032	neoplasm of uncertain behavior of tonsillar fossa,neoplasm of uncertain behaviour of tonsillar fossa	[[1,0,0,0,0,0,0,0,0,0,0,0,0,0],[1,0,0,0,0,0,0,0,0,0,0,0,0,0]]C0262664	vestibulitis of ear	[[0,0,1,0,0,0,0,0,0,0,0,0,0,0]]C0262663	ventricular irritability	[[0,0,1,0,0,0,0,0,0,0,0,0,0,0]]C0686035	neoplasm of uncertain behavior of tonsillar pillar,neoplasm of uncertain behaviour of tonsillar pillar	[[1,0,0,0,0,0,0,0,0,0,0,0,0,0],[1,0,0,0,0,0,0,0,0,0,0,0,0,0]]C0686036	carcinoma in situ of tonsillar pillar	[[1,0,0,0,0,0,0,0,0,0,0,0,0,0]]C0262660	vascular embolism	[[0,0,1,0,0,0,0,0,0,0,0,0,0,0]]C1997116	articular cartilage disorder of elbow	[[1,0,0,0,0,0,0,0,0,0,0,0,0,0]]C0347188	carcinoma in situ of adrenal gland	[[1,0,0,0,0,0,0,0,0,0,0,0,0,0]]C0156466	complete legal abortion with genital tract or pelvic infection,complicated by genital tract and pelvic infection complete legally induced abortion	[[1,0,0,0,0,0,0,0,0,0,0,0,0,0],[0,0,0,0,1,0,0,0,0,0,0,0,0,0]]C0156464	legally induced abortion complicated by genital tract and pelvic infection	[[0,0,0,0,1,0,0,0,0,0,0,0,0,0]]C1285363	hereditary disorder of musculoskeletal system	[[1,0,0,0,0,0,0,0,0,0,0,0,0,0]]C0347180	penile intraepithelial neoplasia,pin penile intraepithelial neoplasia	[[1,0,0,0,0,0,0,0,0,0,0,1,0,0],[1,0,0,0,0,0,0,0,0,0,0,0,0,0]]C0347181	carcinoma in situ of epididymis	[[1,0,0,0,0,0,0,0,0,0,0,0,0,0]]C0347182	carcinoma in situ of spermatic cord	[[1,0,0,0,0,0,0,0,0,0,0,0,0,0]]C0347184	carcinoma in situ of renal pelvis,cis carcinoma in situ of renal pelvis,stage 0is kidney pelvis carcinoma,stage 0is carcinoma of the renal pelvis,carcinoma in situ of the kidney pelvis,stage 0is renal pelvis urothelial carcinoma,stage 0is renal pelvis urothelial carcinoma ajcc v7	[[1,0,0,0,0,0,0,0,0,0,0,1,0,0],[1,0,0,0,0,0,0,0,0,0,0,0,0,0],[0,0,0,0,0,0,0,0,0,0,0,1,0,0],[0,0,0,0,0,0,0,0,0,0,0,1,0,0],[0,0,0,0,0,0,0,0,0,0,0,1,0,0],[0,0,0,0,0,0,0,0,0,0,0,1,0,0],[0,0,0,0,0,0,0,0,0,0,0,1,0,0]]C1285369	neonatal metabolic and endocrinologic disorder	[[1,0,0,0,0,0,0,0,0,0,0,0,0,0]]C0347186	carcinoma in situ of endocrine gland	[[0,0,0,0,0,0,0,0,0,0,0,0,0,1]]C0264625	vocal nodules in adults	[[1,0,0,0,0,0,0,0,0,0,0,0,0,0]]C0264624	vocal nodules in children	[[1,0,0,0,0,0,0,0,0,0,0,0,0,0]]C0264627	ventricular dysphonia,dysphonia plicae ventricularis	[[1,0,0,0,0,0,0,1,0,0,0,0,0,0],[1,0,0,0,0,0,0,1,0,0,0,0,0,0]]C0264626	contact ulcer of vocal folds	[[1,0,0,0,0,0,0,0,0,0,0,0,0,0]]C0264621	mixed nasality,rhinolalia mixta	[[1,0,0,0,0,0,0,0,0,0,0,0,0,0],[1,0,0,0,0,0,0,0,0,0,0,0,0,0]]C0264620	anterior rhinolalia clausa,cul de sac resonation	[[1,0,0,0,0,0,0,0,0,0,0,0,0,0],[1,0,0,0,0,0,0,0,0,0,0,0,0,0]]C0264623	vocal abuse in children	[[1,0,0,0,0,0,0,0,0,0,0,0,0,0]]C0264622	psychogenic voice disorder,functional voice disorder,psychogenic dysphonia,functional dysphonia	[[1,0,0,0,0,0,0,1,0,0,0,0,0,0],[1,0,0,0,0,0,0,1,0,0,0,0,0,0],[1,0,0,0,0,0,0,1,0,0,0,0,0,0],[0,0,0,0,0,0,0,1,0,0,0,0,0,0]]C0157590	with mention of postpartum complication with delivery placental polyp	[[0,0,0,0,1,0,0,0,0,0,0,0,0,0]]C0157591	postpartum placental polyp	[[0,0,0,0,1,0,0,0,0,0,0,0,0,0]]C1334253	intraductal papillary mucinous neoplasm of the biliary tract	[[0,0,0,0,0,0,0,0,0,0,0,1,0,0]]C1842852	type 2 benign adult familial myoclonic epilepsy,bafme2,benign adult familial myoclonic epilepsy 2,adcme,autosomal dominant cortical myoclonus and epilepsy,familial adult myoclonic 2 epilepsy,fame2,fcmte2,2 familial cortical myoclonic tremor with epilepsy	[[0,0,0,0,0,0,1,0,0,0,0,0,0,0],[0,0,0,1,0,0,0,0,0,0,0,0,0,0],[0,0,0,1,0,0,0,0,0,0,0,0,0,0],[0,0,0,1,0,0,0,0,0,0,0,0,0,0],[0,0,0,1,0,0,0,0,0,0,0,0,0,0],[0,0,0,1,0,0,0,0,0,0,0,0,0,0],[0,0,0,1,0,0,0,0,0,0,0,0,0,0],[0,0,0,1,0,0,0,0,0,0,0,0,0,0],[0,0,0,1,0,0,0,0,0,0,0,0,0,0]]C2748515	pakistani type spondyloepimetaphyseal dysplasia,pakistani type semd	[[0,0,0,1,0,0,0,0,0,0,0,0,0,0],[0,0,0,1,0,0,0,0,0,0,0,0,0,0]]C2748516	spondylodysplasia and premature pubarche	[[0,0,0,1,0,0,0,0,0,0,0,0,0,0]]C2827981	stage ib breast cancer,stage ib breast cancer ajcc v7	[[0,0,0,0,0,0,0,0,0,0,0,1,0,0],[0,0,0,0,0,0,0,0,0,0,0,1,0,0]]C2827980	stage ia breast cancer,stage ia breast cancer ajcc v7	[[0,0,0,0,0,0,0,0,0,0,0,1,0,0],[0,0,0,0,0,0,0,0,0,0,0,1,0,0]]C1334257	intrahepatic bile duct cystadenoma	[[0,0,0,0,0,0,0,0,0,0,0,1,0,0]]C0038395	streptococcal infections,streptococcal infectious disease,streptococcosis,streptococcus infection,strep infection,streptococcus bacteria infection	[[1,1,0,0,0,0,0,1,0,0,1,0,1,0],[1,0,0,0,0,0,0,0,0,0,0,0,0,0],[1,0,0,0,0,0,0,0,0,0,0,0,0,0],[0,0,0,0,0,0,0,1,0,1,0,0,0,0],[0,0,0,0,0,0,0,1,0,0,0,0,0,0],[0,0,0,0,0,0,0,1,0,0,0,0,0,0]]C0747625	plaque pruritic	[[0,0,0,0,0,0,0,1,0,0,0,0,0,0]]C1332442	brca 1 syndrome,early onset breast ovarian cancer syndrome	[[0,0,0,0,0,0,0,0,0,0,0,1,0,0],[0,0,0,0,0,0,0,0,0,0,0,1,0,0]]C1332443	brca 2 syndrome,site specific early onset breast cancer syndrome	[[0,0,0,0,0,0,0,0,0,0,0,1,0,0],[0,0,0,0,0,0,0,0,0,0,0,1,0,0]]C0571802	ferrous fumarate allergy	[[1,0,0,0,0,0,0,0,0,0,0,0,0,0]]C0571803	ferrous gluconate allergy	[[1,0,0,0,0,0,0,0,0,0,0,0,0,0]]C0571800	iron sorbitol allergy	[[1,0,0,0,0,0,0,0,0,0,0,0,0,0]]C0032533	polymyalgia rheumatica,forestier certonciny syndrome,peri extra articular rheumatism,myalgic syndrome of the aged,polymyalgia arteritica,senile arthritis,pmr polymyalgia rheumatica,pmr	[[0,0,1,0,0,0,0,1,0,0,0,0,0,0],[1,1,0,0,0,0,0,0,0,0,0,0,1,0],[0,1,0,0,0,0,0,0,0,0,0,0,1,0],[0,0,0,0,0,0,0,0,0,0,1,0,0,0],[0,0,0,0,0,0,0,0,0,0,1,0,0,0],[1,0,0,0,0,0,0,0,0,0,1,0,0,0],[1,0,0,0,0,0,0,0,0,0,0,0,0,0],[0,0,0,0,0,0,0,1,0,0,0,0,0,0]]C0571806	ferrous sulfate allergy,ferrous sulphate allergy	[[1,0,0,0,0,0,0,0,0,0,0,0,0,0],[1,0,0,0,0,0,0,0,0,0,0,0,0,0]]C0571807	ferrous phosphate allergy	[[1,0,0,0,0,0,0,0,0,0,0,0,0,0]]C0571804	ferrous glycine sulfate allergy,ferrous glycine sulphate allergy	[[1,0,0,0,0,0,0,0,0,0,0,0,0,0],[1,0,0,0,0,0,0,0,0,0,0,0,0,0]]C0571805	ferrous succinate allergy	[[1,0,0,0,0,0,0,0,0,0,0,0,0,0]]C0571808	intravenous nutrition allergy	[[1,0,0,0,0,0,0,0,0,0,0,0,0,0]]C0571809	supplementary preparations for parenteral nutrition allergy	[[1,0,0,0,0,0,0,0,0,0,0,0,0,0]]C1850808	miyoshi myopathy,autosomal recessive late onset distal muscular dystrophy,mmd1,miyoshi muscular dystrophy 1,miyoshi distal myopathy	[[0,1,0,1,0,0,0,0,0,0,0,0,0,0],[0,1,0,1,0,0,0,0,0,0,0,0,0,0],[0,0,0,1,0,0,0,0,0,0,0,0,0,0],[0,0,0,1,0,0,0,0,0,0,0,0,0,0],[0,1,0,0,0,0,0,0,0,0,0,0,0,0]]C1881298	keratinizing cystic epithelioma of the rat lung	[[0,0,0,0,0,0,0,0,0,0,0,1,0,0]]C1881299	keratoacanthoma of the rat external ear	[[0,0,0,0,0,0,0,0,0,0,0,1,0,0]]C0494675	pneumonitis due to inhalation of oils and essences	[[0,0,0,0,1,0,0,0,0,0,0,0,0,0]]C2732474	necrosis of skeletal and or smooth muscle due to infection by anaerobic bacteria,anaerobic myonecrosis,necrosis of muscle due to infection by anaerobic bacteria	[[1,0,0,0,0,0,0,0,0,0,0,0,0,0],[1,0,0,0,0,0,0,0,0,0,0,0,0,0],[1,0,0,0,0,0,0,0,0,0,0,0,0,0]]C1859499	3 methylcrotonyl coa carboxylase 2 deficiency,type ii methylcrotonylglycinuria,mcc2 deficiency,methylcrotonylglycinuria type 2,methylcrotonoyl coa carboxylase 2 deficiency,3 alpha methylcrotonylglycinuria 2,3 alpha methylcrotonyl coa carboxylase 2 deficiency	[[0,1,0,0,0,0,0,0,0,0,0,0,0,0],[0,0,0,1,0,0,0,0,0,0,0,0,0,0],[0,0,0,1,0,0,0,0,0,0,0,0,0,0],[0,1,0,0,0,0,0,0,0,0,0,0,0,0],[0,1,0,0,0,0,0,0,0,0,0,0,0,0],[0,1,0,0,0,0,0,0,0,0,0,0,0,0],[0,1,0,0,0,0,0,0,0,0,0,0,0,0]]C1955736	peripheral t cell lymphoma involving lymph nodes of multiple sites	[[0,0,0,0,1,0,0,0,0,0,0,0,0,0]]C1955735	peripheral t cell lymphoma involving spleen	[[0,0,0,0,1,0,0,0,0,0,0,0,0,0]]C1955734	peripheral t cell lymphoma involving intrapelvic lymph nodes	[[0,0,0,0,1,0,0,0,0,0,0,0,0,0]]C1955733	peripheral t cell lymphoma involving lymph nodes of inguinal region and lower limb	[[0,0,0,0,1,0,0,0,0,0,0,0,0,0]]C1955732	peripheral t cell lymphoma involving lymph nodes of axilla and upper limb	[[0,0,0,0,1,0,0,0,0,0,0,0,0,0]]C1955731	peripheral t cell lymphoma involving intra abdominal lymph nodes	[[0,0,0,0,1,0,0,0,0,0,0,0,0,0]]C1955730	peripheral t cell lymphoma involving intrathoracic lymph nodes	[[0,0,0,0,1,0,0,0,0,0,0,0,0,0]]C1708195	gastric soft tissue neoplasm,gastric soft tissue tumor,soft tissue neoplasm of the stomach,soft tissue tumor of the stomach	[[0,0,0,0,0,0,0,0,0,0,0,1,0,0],[0,0,0,0,0,0,0,0,0,0,0,1,0,0],[0,0,0,0,0,0,0,0,0,0,0,1,0,0],[0,0,0,0,0,0,0,0,0,0,0,1,0,0]]C1708194	gastric granular cell tumor	[[0,0,0,0,0,0,0,0,0,0,0,1,0,0]]C1708193	gastric diverticulitis	[[0,0,0,0,0,0,0,0,0,0,0,1,0,0]]C2931530	microcephaly sparse hair mental retardation seizures	[[0,1,0,0,0,0,0,0,0,0,0,0,0,0]]C0263530	longitudinal split nail,onychorrhexis	[[1,0,0,0,0,0,0,0,0,0,0,0,0,0],[0,0,0,1,0,0,0,1,0,0,0,0,0,0]]C0263532	leukonychia punctata,leuconychia punctata	[[0,0,0,0,0,0,0,0,0,0,0,0,0,1],[1,0,0,0,0,0,0,0,0,0,0,0,0,0]]C0263535	muehrckes lines	[[1,0,0,0,0,0,0,0,0,0,0,0,0,0]]C0263536	hypertrophy of nail,nail overgrowth,onychauxis,ox onychauxic	[[1,0,0,0,0,0,0,1,0,0,0,0,0,1],[1,0,0,0,0,0,0,0,0,0,0,0,0,0],[0,0,0,1,0,0,0,1,0,0,0,0,0,0],[1,0,0,0,0,0,0,0,0,0,0,0,0,0]]C0263537	onychogryposis,onychogryphosis,nail disorder,hook nail,og onychogryphosis	[[0,0,0,1,0,0,0,1,0,0,0,0,0,0],[1,0,0,0,0,0,0,1,0,0,0,0,0,0],[0,0,1,0,0,0,0,0,0,0,0,0,0,0],[1,0,0,0,0,0,0,1,0,0,0,0,0,0],[1,0,0,0,0,0,0,0,0,0,0,0,0,0]]C0263539	onychomalacia,soft nails	[[1,0,0,0,0,0,0,1,0,0,0,0,0,0],[1,0,0,0,0,0,0,1,0,0,0,0,0,0]]C0016756	fructose 1 6 diphosphatase deficiency,fructose 1 6 bisphosphatase deficiency,fructose biphosphatase deficiency,fructosediphosphatase deficiency,hereditary fructose 1 6 phosphatase deficiency	[[1,1,0,0,0,0,0,0,0,0,0,0,1,0],[1,1,0,1,0,0,0,0,0,0,0,0,1,0],[1,1,0,0,0,0,0,0,0,0,0,0,1,0],[0,1,0,0,0,0,0,0,0,0,0,0,1,0],[1,0,0,0,0,0,0,0,0,0,0,0,0,0]]C0264080	juvenile osteoporosis,feline osteogenesis imperfecta,idiopathic juvenile osteoporosis,ijo	[[1,1,0,1,0,0,0,1,0,0,0,0,0,0],[1,0,0,0,0,0,0,0,0,0,0,0,0,0],[0,1,0,1,0,0,0,0,0,0,0,0,0,0],[0,0,0,1,0,0,0,0,0,0,0,0,0,0]]C0264083	juvenile osteochondrosis of pelvis	[[1,0,0,0,0,0,0,0,0,0,0,0,0,0]]C0012236	digeorge syndrome,thymic hypoplasia syndrome,pharyngeal pouch syndrome,agenesis of the parathyroid and thymus glands,thymic parathyroid aplasia,third and fourth pharyngeal arch syndrome,digeorge sequence,third and fourth pharyngeal pouch syndrome,digeorge anomaly,branchial arch syndrome,thymic hypoplasia,thymic aplasia syndrome,familial third and fourth pharyngeal pouch syndrome,dgs,chromosome 22q11 2 deletion syndrome,22q11 2 deletion syndrome,hypoplasia of thymus and parathyroids,di george syndrome,catch22	[[1,1,0,1,0,0,0,1,0,1,0,1,0,0],[1,0,0,0,0,0,0,0,0,0,0,0,0,0],[0,1,0,0,0,0,0,1,0,0,0,0,1,1],[1,0,0,0,0,0,0,0,0,0,0,0,0,0],[1,0,0,0,0,0,0,0,0,0,0,0,0,0],[1,0,0,0,0,0,0,0,0,0,0,0,0,0],[1,0,0,0,0,0,0,1,0,0,0,0,1,0],[1,0,0,1,0,0,0,0,0,0,0,0,1,0],[1,0,0,0,0,0,0,1,0,0,0,1,0,0],[1,0,0,0,0,0,0,0,0,0,0,0,0,0],[0,0,0,0,0,0,0,1,0,0,0,0,0,0],[0,1,0,0,0,0,0,1,0,0,0,0,1,0],[0,0,0,0,0,0,0,0,0,0,0,0,1,0],[0,0,0,1,0,0,0,0,0,0,0,0,0,0],[0,0,0,1,0,0,0,0,0,0,0,0,0,0],[0,0,0,0,0,0,0,0,0,0,0,1,0,0],[0,0,0,1,0,0,0,0,0,0,0,0,1,0],[0,0,0,0,0,0,0,1,0,0,1,0,0,0],[0,0,0,1,0,0,0,0,0,0,0,0,0,0]]C0016752	inborn errors fructose metabolism	[[0,0,0,0,0,0,0,0,0,0,0,0,1,0]]C0264084	ischiopubic synchondrosis of van neck	[[0,0,0,0,0,0,0,0,0,0,0,0,0,1]]C0016751	hereditary fructose intolerance syndrome,fructose intolerance,hereditary fructose intolerance,fructosemia,fructosaemia,fructose 1 phosphate aldolase deficiency,aldb deficiency,fructose biphosphate aldolase b deficiency,aldolase b deficiency,hereditary fructosuria,fructose 1 6 bisphosphate aldolase b deficiency,aldb aldolase b deficiency,aldob deficiency,fructose aldolase b deficiency,fructose 1 6 biphosphate aldolase deficiency	[[0,0,0,0,0,0,1,0,0,0,0,0,0,0],[1,1,0,0,0,0,0,1,0,0,1,1,0,0],[0,1,0,0,1,0,0,1,0,0,1,1,0,0],[0,0,0,1,0,0,0,1,0,0,1,0,0,0],[1,0,0,0,0,0,0,0,0,0,0,0,0,0],[1,1,0,1,0,0,0,0,0,1,0,0,1,0],[1,0,0,0,0,0,0,0,0,0,0,0,0,0],[1,0,0,0,0,0,0,0,0,0,0,0,0,0],[1,0,0,1,0,0,0,0,0,0,0,0,0,0],[1,0,0,0,0,0,0,0,0,0,0,0,0,0],[0,0,0,1,0,0,0,0,0,0,0,0,0,0],[1,0,0,0,0,0,0,0,0,0,0,0,0,0],[0,0,0,1,0,0,0,0,0,0,0,0,0,0],[0,0,0,0,0,0,0,0,0,0,0,0,1,0],[0,1,0,0,0,0,0,0,0,0,0,0,1,0]]C0264088	humerus head juvenile osteochondritis,juvenile osteochondrosis of capitellum of humerus,haas disease,panners disease,juvenile osteochondrosis of head of humerus,osteochondritis of the humeral head,osteochondritis of the distal humerus,panners disease osteochondritis of capitulum of humerus,haas disease osteochondritis of head of humerus,humerus juvenile osteochondritis,juvenile osteochondrosis of capitulum of humerus,osteochondrosis of capitulum of humerus,osteochondrosis of haas,osteochondrosis of panner	[[1,0,0,0,0,0,0,0,0,0,0,0,0,0],[1,0,0,0,0,0,0,0,0,0,0,0,0,0],[1,0,0,0,0,0,0,0,0,0,0,0,0,0],[1,0,0,0,0,0,0,0,0,0,0,0,0,0],[0,0,0,0,0,0,0,0,0,0,0,0,0,1],[1,0,0,0,0,0,0,0,0,0,0,0,0,0],[1,0,0,0,0,0,0,0,0,0,0,0,0,0],[1,0,0,0,0,0,0,0,0,0,0,0,0,0],[1,0,0,0,0,0,0,0,0,0,0,0,0,0],[1,0,0,0,0,0,0,0,0,0,0,0,0,0],[0,0,0,0,0,0,0,0,0,0,0,0,0,1],[0,0,0,0,0,0,0,0,0,0,0,0,0,1],[0,0,0,0,0,0,0,0,0,0,0,0,0,1],[0,0,0,0,0,0,0,0,0,0,0,0,0,1]]C0157334	with delivery vulvar and perineal hematoma	[[0,0,0,0,1,0,0,0,0,0,0,0,0,0]]C0157335	postpartum vulvar and perineal hematoma	[[0,0,0,0,1,0,0,0,0,0,0,0,0,0]]C0456105	congenital non bacterial non viral infection	[[1,0,0,0,0,0,0,0,0,0,0,0,0,0]]C1283787	transfusion reaction due to allergens in blood,allergic transfusion reaction	[[1,0,0,0,0,0,0,0,0,0,0,0,0,0],[1,0,0,0,0,0,0,0,0,0,0,0,0,0]]C1283780	deficiency of halogenase	[[1,0,0,0,0,0,0,0,0,0,0,0,0,0]]C1532020	pyrazinamide resistant tuberculosis	[[1,0,0,0,0,0,0,0,0,0,0,0,0,0]]C2827361	myeloid neoplasms with pdgfrb rearrangement,chronic myelomonocytic leukemia with eosinophilia associated with t	[[0,0,0,0,0,0,0,0,0,0,0,1,0,0],[0,0,0,0,0,0,0,0,0,0,0,1,0,0]]C1332866	adenocarcinoma of cecum,adenocarcinoma of caecum,cecal adenocarcinoma	[[1,0,0,0,0,0,0,0,0,0,0,1,0,0],[1,0,0,0,0,0,0,0,0,0,0,0,0,0],[0,0,0,0,0,0,0,0,0,0,0,1,0,0]]C2827362	myeloid and lymphoid neoplasms with fgfr1 rearrangement,8p11 stem cell syndrome,8p11 stem cell leukemia lymphoma syndrome,8p11 myeloproliferative syndrome	[[0,0,0,0,0,0,0,0,0,0,0,1,0,0],[0,0,0,0,0,0,0,0,0,0,0,1,0,0],[0,0,0,0,0,0,0,0,0,0,0,1,0,0],[0,0,0,0,0,0,0,0,0,0,0,1,0,0]]C1283782	deficiency of phosphatase	[[1,0,0,0,0,0,0,0,0,0,0,0,0,0]]C0151859	polyserositis,bambergers disease	[[1,0,0,0,0,0,0,1,0,0,1,0,0,0],[1,0,0,0,0,0,0,0,0,0,0,0,0,0]]C0265971	acrokeratosis verruciformis of hopf,acrokeratosis verruciformis,akv acrokeratosis verruciformis,akv,hopf disease	[[1,1,0,0,0,0,0,0,0,0,0,1,0,0],[0,1,0,1,0,0,0,0,0,0,0,1,0,0],[1,0,0,0,0,0,0,0,0,0,0,0,0,0],[0,0,0,1,0,0,0,0,0,0,0,0,0,0],[0,1,0,1,0,0,0,0,0,0,0,0,0,0]]C3164839	hypoperfusion of right pulmonary artery due to preferential flow to left pulmonary artery	[[1,0,0,0,0,0,0,0,0,0,0,0,0,0]]C2931721	fuchs atrophia gyrata chorioideae et retinae,fuchs gyrate atrophy of the choroid and retina,fuchs gyrate atrophy	[[0,1,0,0,0,0,0,0,0,0,0,0,0,0],[0,1,0,0,0,0,0,0,0,0,0,0,0,0],[0,1,0,0,0,0,0,0,0,0,0,0,0,0]]C2931720	fronto facio nasal dysplasia,fronto facio nasal dysostosis	[[0,1,0,0,0,0,0,0,0,0,0,0,0,0],[0,1,0,0,0,0,0,0,0,0,0,0,0,0]]C0265978	collagen nevus of skin,collagen naevus of skin,connective tissue nevus,collagen hamartoma,collagenoma,collagen nevus,collagen naevus	[[1,0,0,0,0,0,0,0,0,0,0,0,0,0],[1,0,0,0,0,0,0,0,0,0,0,0,0,0],[0,0,0,0,0,0,0,0,0,0,0,1,0,0],[1,0,0,0,0,0,0,0,0,0,0,1,0,0],[0,0,0,1,0,0,0,1,0,0,0,1,0,0],[1,0,0,0,0,0,0,0,0,0,0,1,0,0],[1,0,0,0,0,0,0,0,0,0,0,0,0,0]]C2931722	chang davidson carlson syndrome	[[0,1,0,0,0,0,0,0,0,0,0,0,0,0]]C0206603	circoviridae infections,disease due to circoviridae	[[0,1,0,0,0,0,0,0,0,0,0,0,1,0],[1,0,0,0,0,0,0,0,0,0,0,0,0,0]]C0206602	circovirus infections	[[0,1,0,0,0,0,0,0,0,0,0,0,0,0]]C1332865	cavernous sinus meningioma	[[0,0,0,0,0,0,0,0,0,0,0,1,0,0]]C0206607	torovirus infections	[[0,1,0,0,0,0,0,0,0,0,0,0,1,0]]C0206606	birnaviridae infections,disease due to birnavirus	[[0,1,0,0,0,0,0,0,0,0,0,0,1,0],[1,0,0,0,0,0,0,0,0,0,0,0,0,0]]C2931072	epidermolysa bullosa simplex and limb girdle muscular dystrophy	[[0,1,0,0,0,0,0,0,0,0,0,0,0,0]]C0206604	arterivirus infections	[[0,1,0,0,0,0,0,0,0,0,0,0,1,0]]C0206609	flaviviridae infections,disease due to flaviviridae	[[0,1,0,0,0,0,0,0,0,0,0,0,1,0],[1,0,0,0,0,0,0,0,0,0,0,0,0,0]]C0206608	flavivirus infections,disease due to flavivirus	[[0,1,0,0,0,0,0,0,0,0,0,0,1,0],[1,0,0,0,0,0,0,0,0,0,0,0,0,0]]C0948381	semen discoloration	[[0,0,0,0,0,0,0,1,0,0,0,0,0,0]]C0456110	perinatal skin and subcutaneous infections	[[1,0,0,0,0,0,0,0,0,0,0,0,0,0]]C0948382	deep mycosis,deep fungal infection,deep mycotic infection	[[1,0,0,0,0,0,0,0,0,0,0,0,0,0],[1,0,0,0,0,0,0,1,0,0,0,0,0,0],[1,0,0,0,0,0,0,0,0,0,0,0,0,0]]C0035085	renal infarction,infarction of kidney	[[1,0,0,0,0,0,0,1,0,0,0,0,0,1],[1,0,0,0,0,0,0,1,0,0,1,0,0,0]]C0238015	autonomic dysreflexia,dysreflexia,autonomic hyperreflexia,spinal autonomic dysreflexia	[[0,1,0,0,0,0,0,1,0,0,1,0,1,0],[1,0,0,0,0,0,0,1,0,0,0,0,0,0],[0,1,0,0,0,0,0,1,0,0,1,0,1,0],[0,1,0,0,0,0,0,0,0,0,0,0,1,0]]C0395881	adhesive middle ear disease with adhesions of drum head to promontorium,adhesion of tympanic membrane to promontory	[[1,0,0,0,0,0,0,0,0,0,0,0,0,0],[1,0,0,0,0,0,0,0,0,0,0,0,0,0]]C0035086	renal osteodystrophy,pannephritic osteodystrophy,renal bone disease,rod renal osteodystrophy	[[0,1,0,0,0,0,0,1,0,0,1,0,1,0],[0,0,0,0,0,0,0,0,0,0,1,0,0,0],[1,0,0,0,0,0,0,1,0,0,0,0,0,0],[1,0,0,0,0,0,0,0,0,0,0,0,0,0]]C0741103	antibiotic allergy	[[0,0,0,0,0,0,0,1,0,0,0,0,0,0]]C0456112	neonatal breast abscess	[[1,0,0,0,0,0,0,0,0,0,0,0,0,0]]C1096618	peritoneal lesion	[[0,0,0,0,0,0,0,1,0,0,0,0,0,0]]C1847987	huntington disease like 2,hdl2	[[0,0,0,1,0,0,0,0,0,0,0,0,0,0],[0,0,0,1,0,0,0,0,0,0,0,0,0,0]]C0521748	masticatory myositis	[[1,0,0,0,0,0,0,0,0,0,0,0,0,0]]C0521749	postenucleation orbital drainage,orbital discharge after enucleation	[[1,0,0,0,0,0,0,0,0,0,0,0,0,0],[1,0,0,0,0,0,0,0,0,0,0,0,0,0]]C0456113	neonatal bacterial conjunctivitis	[[1,0,0,0,0,0,0,0,0,0,0,0,0,0]]C0456114	neonatal chlamydial dacryocystitis,neonatal dacryocystitis due to chlamydiae	[[1,0,0,0,0,0,0,0,0,0,0,0,0,0],[1,0,0,0,0,0,0,0,0,0,0,0,0,0]]C0521744	disorder of nasolacrimal duct	[[1,0,0,0,0,0,0,0,0,0,0,0,0,0]]C0456115	neonatal viral conjunctivitis,neonatal conjunctivitis due to virus,viral ophthalmia neonatorum	[[1,0,0,0,0,0,0,0,0,0,0,0,0,0],[1,0,0,0,0,0,0,0,0,0,0,0,0,0],[1,0,0,0,0,0,0,0,0,0,0,0,0,0]]C0220659	acrodysostosis,peripheral dysostosis,acrodysplasia,arkless graham syndrome,peripheral dysostosis nasal hypoplasia mental retardation pnm syndrome,nasal hypoplasia peripheral dysostosis mental retardation syndrome,maroteaux malamut syndrome	[[1,0,0,1,0,0,0,1,0,0,0,0,0,0],[1,0,0,1,0,0,0,0,0,0,0,0,0,0],[0,1,0,0,0,0,0,0,0,0,0,0,0,0],[0,1,0,0,0,0,0,0,0,0,0,0,0,0],[0,1,0,0,0,0,0,0,0,0,0,0,0,0],[0,1,0,0,0,0,0,0,0,0,0,0,0,0],[0,1,0,0,0,0,0,0,0,0,0,0,0,0]]C0345856	benign neoplasm of hepatic flexure of colon,benign tumor of hepatic flexure,benign tumour of hepatic flexure	[[1,0,0,0,0,0,0,0,0,0,0,0,0,0],[1,0,0,0,0,0,0,0,0,0,0,0,0,0],[1,0,0,0,0,0,0,0,0,0,0,0,0,0]]C0345857	neoplasm of transverse colon,tumor of transverse colon,tumour of transverse colon	[[1,0,0,0,0,0,0,1,0,0,0,0,0,0],[1,0,0,0,0,0,0,1,0,0,0,0,0,0],[1,0,0,0,0,0,0,1,0,0,0,0,0,0]]C0345851	carcinoma in situ of ascending colon	[[1,0,0,0,0,0,0,0,0,0,0,0,0,0]]C0345853	neoplasm of hepatic flexure of colon,tumor of hepatic flexure,tumour of hepatic flexure	[[1,0,0,0,0,0,0,0,0,0,0,0,0,0],[1,0,0,0,0,0,0,0,0,0,0,0,0,0],[1,0,0,0,0,0,0,0,0,0,0,0,0,0]]C0220650	metastatic malignant neoplasm to brain,metastasis to brain,brain metastases,metastatic to brain cancer,secondary cancer of brain,secondary malignant neoplasm of brain,brain secondary,metastatic tumor to the brain,adult tumors metastatic to brain,metastatic neoplasm to the brain	[[1,0,0,0,0,0,0,0,0,0,0,1,0,0],[1,0,0,0,0,0,0,1,0,0,0,1,0,0],[0,0,0,0,0,0,0,1,0,0,0,1,0,0],[0,0,0,0,0,1,0,1,0,0,0,0,0,0],[1,0,0,0,0,0,0,1,0,0,0,0,0,0],[1,0,0,0,0,0,0,0,0,0,0,0,0,0],[0,0,0,0,0,0,0,1,0,0,0,0,0,0],[0,0,0,0,0,0,0,1,0,0,0,1,0,0],[0,0,0,0,0,1,0,0,0,0,0,0,0,0],[0,0,0,0,0,0,0,0,0,0,0,1,0,0]]C0456117	neonatal bacterial dacryocystitis	[[1,0,0,0,0,0,0,0,0,0,0,0,0,0]]C0220654	meningeal carcinomatosis,meningeal carcinomatoses,carcinomatous meningitis,carcinomatous meningitides,carcinomatous leptomeningitis,leukemic meningitis,lymphomatous meningitis,meningeal carcinomatosis brain tumor,meningeal carcinomatosis cancer,meningeal carcinomatosis intracranial neoplasm,malignant meningitis,neoplastic meningitis,metastases meningeal,leptomeningeal carcinomatosis,leptomeningeal carcinomatoses	[[1,1,0,0,0,0,0,1,0,0,1,1,0,0],[0,1,0,0,0,0,0,0,0,0,0,0,0,0],[1,1,0,0,0,0,0,1,0,0,0,0,1,0],[0,1,0,0,0,0,0,0,0,0,0,0,0,0],[0,0,0,0,0,0,0,1,0,0,0,0,0,0],[0,0,0,0,0,0,0,1,0,0,0,0,0,0],[0,0,0,0,0,0,0,1,0,0,0,0,0,0],[0,0,0,0,0,0,0,0,0,0,1,0,0,0],[0,0,0,0,0,0,0,0,0,0,1,0,0,0],[0,0,0,0,0,0,0,0,0,0,1,0,0,0],[1,0,0,0,0,0,0,1,0,0,0,0,0,0],[0,0,0,0,0,0,0,1,0,0,0,0,0,0],[0,0,0,0,0,0,0,1,0,0,0,0,0,0],[0,1,0,0,0,0,0,0,0,0,0,0,1,0],[0,1,0,0,0,0,0,0,0,0,0,0,0,0]]C0220655	malignant pericardial effusion	[[1,0,0,0,0,1,0,0,0,0,0,1,0,0]]C0220656	malignant ascites	[[0,0,0,0,1,0,0,0,0,0,1,1,0,0]]C0740850	airway disease restrictive	[[0,0,0,0,0,0,0,1,0,0,0,0,0,0]]C0409731	tabetic joint	[[1,0,0,0,0,0,0,0,0,0,0,0,0,0]]C0919607	duodenal carcinoid tumor,carcinoid tumor of duodenum,duodenal carcinoid neoplasm,carcinoid neoplasm of the duodenum	[[0,0,0,0,0,0,0,0,0,0,0,1,0,0],[0,0,0,0,0,0,0,0,0,0,0,1,0,0],[0,0,0,0,0,0,0,0,0,0,0,1,0,0],[0,0,0,0,0,0,0,0,0,0,0,1,0,0]]C0031256	petechiae,petechial hemorrhage,petechial haemorrhage,petechial rash,petechiae of skin,petechial eruption	[[0,0,1,1,0,0,0,1,1,0,0,1,0,0],[1,0,0,0,0,0,0,1,0,0,0,0,0,0],[1,0,0,0,0,0,0,0,0,0,0,0,0,0],[1,0,0,0,0,0,0,1,1,0,0,0,0,0],[1,0,0,0,0,0,0,0,0,0,0,0,0,0],[1,0,0,0,0,0,0,0,0,0,0,0,0,0]]C0242473	anus prolapse,anal canal prolapse,procidentia of anus,proctoptosis,anal prolapse	[[0,1,0,0,0,0,0,0,0,0,0,0,0,0],[0,0,0,0,0,0,0,0,0,0,0,0,0,1],[1,0,0,0,0,0,0,0,0,0,0,0,0,1],[0,0,0,0,0,0,0,1,0,0,0,0,0,1],[1,0,0,0,0,0,0,1,0,0,0,0,0,0]]C0559459	sacrococcygeal teratoma	[[0,0,0,1,0,0,0,1,0,0,0,0,0,0]]C0559458	neuroblastoma of brain,cerebral neuroblastoma,neuroblastoma of the cerebral hemispheres,pnet cerebral neuroblastoma,neuroblastoma of the cerebrum	[[1,0,0,0,0,0,0,0,0,0,0,0,0,0],[0,0,0,0,0,0,0,0,0,0,0,1,0,0],[0,0,0,0,0,0,0,0,0,0,0,1,0,0],[0,0,0,0,0,0,0,0,0,0,0,1,0,0],[0,0,0,0,0,0,0,0,0,0,0,1,0,0]]C1275140	perinatal forceps injury	[[1,0,0,0,0,0,0,0,0,0,0,0,0,0]]C1864689	syndromic 6 microphthalmia,microphthalmia and pituitary anomalies,mcops6,and abnormal external genitalia digital anomalies malformed ears with micrognathia clinical anophthalmia,microphthalmia with brain and digit developmental anomalies	[[0,0,0,1,0,0,0,0,0,0,0,0,0,0],[0,0,0,1,0,0,0,0,0,0,0,0,0,0],[0,0,0,1,0,0,0,0,0,0,0,0,0,0],[0,0,0,1,0,0,0,0,0,0,0,0,0,0],[0,0,0,1,0,0,0,0,0,0,0,0,0,0]]C1336195	stage iib small cell lung carcinoma,stage iib small cell lung cancer,stage iib small cell lung carcinoma ajcc v7	[[0,0,0,0,0,0,0,0,0,0,0,1,0,0],[0,0,0,0,0,0,0,0,0,0,0,1,0,0],[0,0,0,0,0,0,0,0,0,0,0,1,0,0]]C0864849	malignant neoplasm of buccal sulcus	[[0,0,0,0,0,0,0,0,0,0,0,0,0,1]]C0864848	malignant neoplasm of contiguous or overlapping sites of floor of mouth whose point of origin cannot be determined	[[0,0,0,0,0,0,0,0,0,0,0,0,0,1]]C0864847	malignant neoplasm of anterior portion of mouth to the premolar canine junction	[[0,0,0,0,0,0,0,0,0,0,0,0,0,1]]C0864846	malignant neoplasm of contiguous or overlapping sites of gum whose point of origin cannot be determined	[[0,0,0,0,0,0,0,0,0,0,0,0,0,1]]C0864845	malignant neoplasm of contiguous or overlapping sites of salivary glands and ducts whose point of origin cannot be determined	[[0,0,0,0,0,0,0,0,0,0,0,0,0,1]]C0864844	malignant neoplasm of contiguous or overlapping sites of tongue whose point of origin cannot be determined	[[0,0,0,0,0,0,0,0,0,0,0,0,0,1]]C0864843	malignant neoplasm of border of tongue at junction of fixed and mobile parts at insertion of anterior tonsillar pillar	[[0,0,0,0,0,0,0,0,0,0,0,0,0,1]]C0864842	ventral surface malignant neoplasm of anterior two thirds of tongue	[[0,0,0,0,0,0,0,0,0,0,0,0,0,1]]C0864841	dorsal surface malignant neoplasm of anterior two thirds of tongue	[[0,0,0,0,0,0,0,0,0,0,0,0,0,1]]C0265029	portal vein obstruction,hepatic portal vein obstruction,occlusion of hepatic portal vein,portal obstruction	[[0,0,0,0,0,0,0,1,0,0,0,0,0,1],[1,0,0,0,0,0,0,0,0,0,0,0,0,0],[1,0,0,0,0,0,0,0,0,0,0,0,0,0],[0,0,0,0,0,0,0,1,0,0,0,0,0,1]]C0860549	refeeding syndrome,refeed syndrome	[[1,1,0,0,0,0,0,1,0,0,0,0,1,0],[0,0,0,0,0,0,0,1,0,0,0,0,0,0]]C0265021	ruptured sinus of valsalva into left ventricle	[[1,0,0,0,0,0,0,0,0,0,0,0,0,0]]C0265022	ruptured sinus of valsalva into right ventricle	[[1,0,0,0,0,0,0,0,0,0,0,0,0,0]]C0265023	ruptured sinus of valsalva into right atrium	[[1,0,0,0,0,0,0,0,0,0,0,0,0,0]]C0265025	capillary hyperpermeability	[[1,0,0,0,0,0,0,0,0,0,0,0,0,0]]C0265026	capillary thrombosis	[[0,0,0,0,0,0,0,0,0,0,0,0,0,1]]C0265027	non neoplastic nevus of skin,non neoplastic nevus,non neoplastic naevus	[[0,0,0,0,0,0,1,0,0,0,0,0,0,0],[1,0,0,0,1,0,0,0,0,0,0,1,0,0],[1,0,0,0,0,0,0,0,0,0,0,0,0,0]]C0221019	sickle cell beta thalassemia,sickle cell beta thalassaemia,hemoglobin s thalassemia,double heterozygous for hb s and beta thalassemia,double heterozygous for hb s and beta thalassaemia,hemoglobin s beta thalassemia,haemoglobin s beta thalassaemia,thalassemia with hemoglobin s disease,thalassaemia with haemoglobin s disease,sickle cell thalassemia,sickle cell thalassaemia,sickle beta thalassemia	[[1,0,0,0,0,0,0,0,0,0,0,1,0,0],[1,0,0,0,0,0,0,0,0,0,0,0,0,0],[0,0,0,0,0,0,0,1,0,0,0,0,0,0],[1,0,0,0,0,0,0,0,0,0,0,0,0,0],[1,0,0,0,0,0,0,0,0,0,0,0,0,0],[1,0,0,0,0,0,0,0,0,0,0,0,0,0],[1,0,0,0,0,0,0,0,0,0,0,0,0,0],[1,0,0,0,0,0,0,0,0,0,0,0,0,0],[1,0,0,0,0,0,0,0,0,0,0,0,0,0],[0,0,0,0,0,0,0,1,0,0,0,0,0,0],[0,0,0,0,0,0,0,1,0,0,0,0,0,0],[0,0,0,0,0,0,0,0,0,0,0,1,0,0]]C0221018	hereditary sideroblastic anemia,hereditary sideroblastic anaemia,hypochromic anemia,x chromosome linked sideroblastic anemia,x chromosome linked sideroblastic anaemia,erythroid 5 aminolevulinate synthetase deficiency,erythroid 5 aminolaevulinate synthetase deficiency,congenital sideroblastic anemia,congenital sideroblastic anaemia,x linked sideroblastic anemia,x linked sideroblastic anaemia,sex linked hypochromic sideroblastic anemia,xlsa,hereditary iron loading anemia,anh1	[[1,0,0,0,0,0,0,0,0,0,1,1,0,1],[1,0,0,0,0,0,0,0,0,0,0,0,0,0],[0,0,0,1,0,0,0,0,0,0,0,0,0,0],[1,0,0,0,0,0,0,0,0,0,0,0,0,0],[1,0,0,0,0,0,0,0,0,0,0,0,0,0],[1,0,0,0,0,0,0,0,0,0,0,0,0,0],[1,0,0,0,0,0,0,0,0,0,0,0,0,0],[1,0,0,0,0,0,0,0,0,0,0,0,0,1],[1,0,0,0,0,0,0,0,0,0,0,0,0,0],[1,0,0,1,0,0,0,0,0,0,0,0,0,0],[1,0,0,0,0,0,0,0,0,0,0,0,0,0],[0,0,0,0,0,0,0,0,0,0,0,0,0,1],[0,0,0,1,0,0,0,0,0,0,0,0,0,0],[0,0,0,1,0,0,0,0,0,0,0,0,0,0],[0,0,0,1,0,0,0,0,0,0,0,0,0,0]]C0570780	piracetam allergy	[[1,0,0,0,0,0,0,0,0,0,0,0,0,0]]C0570781	gabapentin allergy	[[1,0,0,0,0,0,0,0,0,0,0,0,0,0]]C0570786	primidone allergy	[[1,0,0,0,0,0,0,0,0,0,0,0,0,0]]C0570787	carbamazepine allergy	[[1,0,0,0,0,0,0,0,0,0,0,0,0,0]]C0570784	methylphenobarbitone allergy,methylphenobarbital allergy	[[1,0,0,0,0,0,0,0,0,0,0,0,0,0],[1,0,0,0,0,0,0,0,0,0,0,0,0,0]]C0570785	phenobarbitone allergy,phenobarbital allergy	[[1,0,0,0,0,0,0,0,0,0,0,0,0,0],[1,0,0,0,0,0,0,0,0,0,0,0,0,0]]C0221011	malignant atrophic papulosis,malignant atrophic papuloses,cutaneointestinal syndrome,degos disease,kohlmeier degos disease,koehlmeier degos syndrome,lethal cutaneous and gastrointestinal arteriolar thrombosis,degoss malignant atrophic papulosis,malignant atrophic papulosis of degos,erythrokeratoderma en cocardes,maladie de degos,map malignant atrophic papulosis,kohlmeier degos syndrome,degos syndrome	[[1,1,0,1,0,0,0,1,0,0,1,1,0,0],[0,1,0,0,0,0,0,0,0,0,0,0,0,0],[0,0,0,0,0,0,0,0,0,0,1,0,0,0],[1,1,0,0,0,0,0,1,0,0,1,1,0,0],[0,1,0,0,0,0,0,1,0,0,0,0,1,0],[0,0,0,0,0,0,0,0,0,0,1,0,0,0],[1,0,0,0,0,0,0,0,0,0,0,0,0,0],[0,0,0,0,0,0,0,0,0,0,0,0,1,0],[1,0,0,0,0,0,0,0,0,0,0,0,0,0],[1,0,0,0,0,0,0,0,0,0,0,0,1,0],[1,0,0,0,0,0,0,0,0,0,0,0,0,0],[1,0,0,0,0,0,0,0,0,0,0,0,0,0],[1,0,0,0,0,0,0,0,0,0,0,0,0,0],[0,1,0,0,0,0,0,0,0,0,0,0,1,0]]C0221010	osteitis of pelvic region,osteitis pubis	[[1,0,0,0,0,0,0,0,0,0,0,0,0,0],[1,0,0,0,0,0,0,1,0,0,1,0,0,0]]C0221013	systemic mastocytosis,systemic mastocytoses,systemic tissue mast cell disease,systemic mast cell disease,systemic urticaria pigmentosa,smcd systemic mast cell disease	[[1,1,0,0,0,0,0,1,0,0,1,0,1,0],[0,1,0,0,0,0,0,0,0,0,0,0,0,0],[0,0,0,0,0,0,0,0,0,0,0,1,0,1],[1,1,0,0,0,0,0,1,0,0,1,0,1,0],[0,0,0,0,0,0,0,1,0,0,0,0,0,0],[1,0,0,0,0,0,0,0,0,0,0,0,0,0]]C0221012	tropical anhidrotic asthenia,sweat retention syndrome,thermogenic anhidrosis	[[1,0,0,0,0,0,0,0,0,0,0,0,0,0],[0,0,0,0,0,0,0,0,0,0,1,0,0,0],[1,0,0,0,0,0,0,0,0,0,0,0,0,0]]C1864356	maroteaux type acromesomelic dysplasia,amdm	[[0,1,0,1,0,0,0,0,0,0,0,0,0,0],[0,0,0,1,0,0,0,0,0,0,0,0,0,0]]C0221014	secondary amyloidosis,aa amyloidosis,reactive systemic amyloidosis	[[0,0,0,0,0,0,0,1,0,0,1,1,0,1],[1,0,0,0,0,0,0,1,0,0,0,0,0,0],[1,0,0,0,0,0,0,0,0,0,0,0,0,0]]C0867369	infection and inflammatory reaction due to ventricular shunt catheter	[[0,0,0,0,0,0,0,0,0,0,0,0,0,1]]C0221016	red blood cell disorder	[[1,0,0,0,0,0,0,1,0,0,0,0,0,0]]C0154738	lumbosacral radiculopathy,lumbosacral root lesion	[[1,0,0,0,0,0,0,1,0,0,0,0,0,0],[1,0,0,0,0,0,0,1,0,0,0,0,0,0]]C0685099	canine dilated cardiomyopathy	[[1,0,0,0,0,0,0,0,0,0,0,0,0,0]]C0685098	feline dilated cardiomyopathy	[[1,0,0,0,0,0,0,0,0,0,0,0,0,0]]C0278153	clonic facial spasm	[[1,0,0,0,0,0,0,0,0,0,0,0,0,0]]C1858186	protection against dengue fever	[[0,0,0,1,0,0,0,0,0,0,0,0,0,0]]C0154731	glossopharyngeal neuralgia	[[0,1,0,0,1,0,0,1,0,0,1,0,0,0]]C0685092	neoplasm of uncertain behavior of diaphragm,neoplasm of uncertain behaviour of diaphragm	[[1,0,0,0,0,0,0,0,0,0,0,0,0,0],[1,0,0,0,0,0,0,0,0,0,0,0,0,0]]C0154733	multiple cranial nerve palsy	[[1,0,0,0,1,0,0,0,0,0,0,0,0,0]]C0685090	secondary malignant neoplasm of posterior mediastinum,metastatic malignant neoplasm to posterior mediastinum	[[1,0,0,0,0,0,0,0,0,0,0,0,0,0],[1,0,0,0,0,0,0,0,0,0,0,0,0,0]]C0154735	lumbosacral plexus lesion	[[1,0,0,0,1,0,0,0,0,0,0,0,0,0]]C0685096	feline intergrade cardiomyopathy	[[1,0,0,0,0,0,0,0,0,0,0,0,0,0]]C0154737	thoracic radiculopathy,thoracic root lesion	[[1,0,0,0,0,0,0,1,0,0,0,0,0,0],[1,0,0,0,0,0,0,1,0,0,0,0,0,0]]C0685094	calcific coronary arteriosclerosis	[[1,0,0,0,0,0,0,0,0,0,0,0,0,0]]C0406753	atypical ichthyosis vulgaris with hypogonadism	[[1,0,0,0,0,0,0,0,0,0,0,0,0,0]]C0406751	cutaneous syndrome with ichthyosis	[[1,0,0,0,0,0,0,0,0,0,0,0,0,0]]C0406750	reticulate pigmented anomaly of flexures,dowling degos syndrome	[[1,0,0,0,0,0,0,0,0,0,0,0,0,0],[1,0,0,0,0,0,0,0,0,0,0,0,0,0]]C0406757	hereditary palmoplantar keratoderma	[[1,0,0,0,0,0,0,0,0,0,0,0,0,0]]C0406756	keratolytic winter erythema,oudtshoorn disease,erythrokeratolysis hiemalis,winter erythrokeratolysis,kwe,oudtshoorn skin,erythrokeratolysis hiemalis ichthyosis	[[0,1,0,1,0,0,0,0,0,0,0,0,0,0],[1,0,0,0,0,0,0,0,0,0,0,0,0,0],[1,0,0,0,0,0,0,0,0,0,0,0,0,0],[1,0,0,0,0,0,0,0,0,0,0,0,0,0],[0,0,0,1,0,0,0,0,0,0,0,0,0,0],[0,1,0,1,0,0,0,0,0,0,0,0,0,0],[0,1,0,0,0,0,0,0,0,0,0,0,0,0]]C0406755	erythrokeratoderma progressiva of gottron,symmetrical progressive erythrokeratoderma	[[1,0,0,0,0,0,0,0,0,0,0,0,0,0],[1,0,0,0,0,0,0,0,0,0,0,0,0,0]]C0406754	keratosis pilaris with ichthyosis and deafness	[[1,0,0,0,0,0,0,0,0,0,0,0,0,0]]C0865076	benign neoplasm of skin of abdominal wall	[[0,0,0,0,0,0,0,0,0,0,0,0,0,1]]C0406759	autosomal dominant mutilating keratoderma	[[1,0,0,0,0,0,0,0,0,0,0,0,0,0]]C0406758	palmoplantar keratoderma transgrediens	[[1,0,0,0,0,0,0,0,0,0,0,0,0,0]]C1867983	familial porencephaly,porencephaly,adt1p,type 1 porencephaly,autosomal dominant type 1 porencephaly,t1p,with porencephaly infantile hemiplegia,familial porencephalic white matter disease	[[0,1,0,1,0,0,0,0,0,0,0,0,0,0],[0,0,0,1,0,0,0,0,0,0,0,0,0,0],[0,0,0,1,0,0,0,0,0,0,0,0,0,0],[0,0,0,1,0,0,0,0,0,0,0,0,0,0],[0,0,0,1,0,0,0,0,0,0,0,0,0,0],[0,0,0,1,0,0,0,0,0,0,0,0,0,0],[0,1,0,1,0,0,0,0,0,0,0,0,0,0],[0,1,0,0,0,0,0,0,0,0,0,0,0,0]]C1868476	wegener like granulomatosis	[[0,0,0,1,0,0,0,0,0,0,0,0,0,0]]C1868387	quantitative trait locus 1 plasma level of alkaline phosphatase,elevated serum alkaline phosphatase,benign familial hyperphosphatasemia,alpqtl1	[[0,0,0,1,0,0,1,0,0,0,0,0,0,0],[0,0,0,1,0,0,0,0,0,0,0,0,0,0],[0,0,0,1,0,0,0,0,0,0,0,0,0,0],[0,0,0,1,0,0,0,0,0,0,0,0,0,0]]C1096330	neoplasm of omentum	[[1,0,0,0,0,0,0,0,0,0,0,0,0,0]]C3149767	chromosome 7q11 23 triplication syndrome,wbs triplication syndrome	[[0,0,0,1,0,0,0,0,0,0,0,0,0,0],[0,0,0,1,0,0,0,0,0,0,0,0,0,0]]C0865071	benign neoplasm of external cheek	[[0,0,0,0,0,0,0,0,0,0,0,0,0,1]]C2931532	microdontia hypodontia short stature	[[0,1,0,0,0,0,0,0,0,0,0,0,0,0]]C1852510	craniofacial deafness hand syndrome,cdhs,and a sensorineural hearing loss hypoplastic nose with slitlike nares hypertelorism features of flat facial profile	[[0,1,0,1,0,0,0,0,0,0,0,0,0,0],[0,0,0,1,0,0,0,0,0,0,0,0,0,0],[0,1,0,0,0,0,0,0,0,0,0,0,0,0]]C1852513	coxoauricular syndrome	[[0,0,0,1,0,0,0,0,0,0,0,0,0,0]]C1852512	cranioacrofacial syndrome	[[0,0,0,1,0,0,0,0,0,0,0,0,0,0]]C0010495	cutis laxa,chalazodermia,dermatolysis,dermatolyses,dermatomegaly,primary elastolysis,dermatochalasis,dermatochalasia,dermatochalazia,elastolysis,generalized dermatochalasis,generalised dermatochalasis,generalized elastolysis,chalastodermia,lax skin,loose skin,skin laxity	[[0,1,0,1,0,0,0,1,0,0,0,0,0,0],[1,0,0,0,0,0,0,0,0,1,0,0,0,0],[0,0,0,0,0,0,0,1,0,0,0,0,1,0],[0,1,0,0,0,0,0,0,0,0,0,0,0,0],[0,0,0,0,0,0,0,0,0,1,0,0,1,0],[1,0,0,0,0,0,0,0,0,0,0,0,0,0],[1,0,0,0,0,0,0,1,0,0,0,0,0,0],[1,0,0,0,0,0,0,1,0,0,0,0,0,0],[0,0,0,0,0,0,0,1,0,0,0,0,0,0],[0,0,0,0,0,0,0,1,0,0,0,1,0,0],[1,0,0,0,0,0,0,0,0,0,0,0,0,0],[1,0,0,0,0,0,0,0,0,0,0,0,0,0],[0,0,0,0,0,0,0,0,0,1,0,0,0,0],[0,0,0,0,0,0,0,0,0,1,0,0,0,0],[0,0,0,0,0,0,0,1,0,0,0,0,0,0],[0,0,0,1,0,0,0,1,0,0,0,0,0,0],[0,0,0,1,0,0,0,1,0,0,0,0,0,0]]C0571789	ethamsylate allergy,etamsylate allergy	[[1,0,0,0,0,0,0,0,0,0,0,0,0,0],[1,0,0,0,0,0,0,0,0,0,0,0,0,0]]C0571788	hemostatic allergy,haemostatic allergy	[[1,0,0,0,0,0,0,0,0,0,0,0,0,0],[1,0,0,0,0,0,0,0,0,0,0,0,0,0]]C0571785	warfarin allergy	[[1,0,0,0,0,0,0,0,0,0,0,0,0,0]]C0025268	multiple endocrine neoplasia type 2a,mea ii,men ii,sipple syndrome,men 2,multiple endocrine type 2a neoplasms,men 2a,mea 2a,multiple endocrine neoplasia type ii,men iia,mea iia,men2a,ptc syndrome,type 2 multiple endocrine adenomatosis,multiple endocrine neoplasia type 2,type 2 mea,type 2 men,familial chromaffinomatosis,men 2a multiple endocrine neoplasia syndrome type 2a,multiple endocrine neoplasia syndrome type 2a,men 2a syndrome,multiple endocrine adenomatosis type ii,men type ii,mea type ii,multiple endocrine adenomatosis type 2a,men type 2a,mea type 2a,pheochromocytoma and amyloid producing medullary thyroid carcinoma,type iia multiple endocrine neoplasia,multiple endocrine neoplasia men type iia	[[0,1,0,0,0,0,0,1,0,0,0,1,1,0],[0,0,0,0,0,0,0,0,0,0,0,0,1,0],[0,0,0,0,0,0,0,0,0,1,0,0,0,0],[1,0,0,1,0,0,0,1,0,1,0,0,1,0],[0,0,0,0,0,0,0,0,0,0,0,0,1,0],[0,0,0,0,0,0,0,0,0,0,0,0,1,0],[0,0,0,0,0,0,0,0,0,0,0,1,1,0],[0,0,0,0,0,0,0,0,0,0,0,0,1,0],[0,0,0,0,0,0,0,0,0,0,1,1,0,0],[0,0,0,0,0,0,0,0,0,0,0,0,1,0],[0,0,0,0,0,0,0,0,0,0,0,0,1,0],[0,0,0,1,0,0,0,0,0,0,0,0,1,0],[1,0,0,0,0,0,0,0,0,0,1,0,0,0],[1,0,0,0,0,0,0,0,0,0,0,0,0,0],[1,0,0,0,0,0,0,0,0,0,0,0,1,0],[1,0,0,0,0,0,0,0,0,0,0,0,0,0],[1,0,0,0,0,0,0,1,0,0,0,0,0,0],[1,0,0,0,0,0,0,0,0,0,0,0,0,0],[1,0,0,0,0,0,0,0,0,0,0,0,0,0],[1,0,0,0,0,0,0,0,0,0,0,0,0,0],[1,1,0,0,0,0,0,0,0,0,0,0,1,0],[0,0,0,0,0,0,0,0,0,0,0,1,0,0],[0,0,0,0,0,0,0,1,0,0,0,1,0,0],[0,0,0,0,0,0,0,0,0,0,0,1,0,0],[0,0,0,0,0,0,0,0,0,0,0,1,0,0],[0,0,0,0,0,0,0,0,0,0,0,1,0,0],[0,0,0,0,0,0,0,0,0,0,0,1,0,0],[0,0,0,1,0,0,0,0,0,0,0,0,0,0],[0,0,0,1,0,0,0,0,0,0,0,0,0,0],[0,0,0,0,1,0,0,0,0,0,0,0,0,0]]C0571787	phenindione allergy	[[1,0,0,0,0,0,0,0,0,0,0,0,0,0]]C0571786	indanedione anticoagulant allergy	[[1,0,0,0,0,0,0,0,0,0,0,0,0,0]]C0571781	danaparoid sodium allergy	[[1,0,0,0,0,0,0,0,0,0,0,0,0,0]]C0571780	heparinoid allergy	[[1,0,0,0,0,0,0,0,0,0,0,0,0,0]]C0571783	coumarin anticoagulant allergy	[[1,0,0,0,0,0,0,0,0,0,0,0,0,0]]C0571782	indirect acting anticoagulant allergy	[[1,0,0,0,0,0,0,0,0,0,0,0,0,0]]C1846169	myotubular myopathy with abnormal genital development	[[0,0,0,1,0,0,0,0,0,0,0,0,0,0]]C1846168	syndromic 10 x linked mental retardation,camr,chorioathetosis with mental retardation and abnormal behavior,mrxs10	[[0,0,0,1,0,0,0,0,0,0,0,0,0,0],[0,0,0,1,0,0,0,0,0,0,0,0,0,0],[0,0,0,1,0,0,0,0,0,0,0,0,0,0],[0,0,0,1,0,0,0,0,0,0,0,0,0,0]]C0745730	multiple lipomata,lipoma multiple	[[1,0,0,0,0,0,0,0,0,0,0,0,0,0],[0,0,0,0,0,0,0,1,0,0,0,0,0,0]]C1876161	2 neuronal ceroid lipofuscinosis,variable age at onset 2 neuronal ceroid lipofuscinosis,cln2	[[0,0,0,1,0,0,0,0,0,0,0,0,0,0],[0,0,0,1,0,0,0,0,0,0,0,0,0,0],[0,0,0,1,0,0,0,0,0,0,0,0,0,0]]C1846164	testicular germ cell tumor 1,tgct1	[[0,0,0,1,0,0,0,0,0,0,0,0,0,0],[0,0,0,1,0,0,0,0,0,0,0,0,0,0]]C1846167	x linked pseudoautosomal hodgkin disease	[[0,1,0,1,0,0,0,0,0,0,0,0,0,0]]C0752195	perimeningeal infections,parameningeal infections	[[0,1,0,0,0,0,0,0,0,0,0,0,1,0],[0,1,0,0,0,0,0,0,0,0,0,0,1,0]]C0752194	schistosomal myelopathy	[[0,1,0,0,0,0,0,0,0,0,0,0,0,0]]C0752197	adult onset dystonias	[[0,1,0,0,0,0,0,0,0,0,0,0,0,0]]C0752196	ballismus,ballism	[[1,0,0,0,0,0,0,1,0,0,0,0,0,0],[1,0,0,0,0,0,0,0,0,0,0,0,0,0]]C0752191	neuroschistosomiasis,central nervous system schistosomiasis,neuroschistosomiases	[[0,0,0,0,0,0,0,0,0,0,0,0,1,0],[0,0,0,0,0,0,0,0,0,0,0,0,1,0],[0,1,0,0,0,0,0,0,0,0,0,0,0,0]]C0282488	interstitial cystitis,interstitial cystitides,ulcerative cystitis,ic interstitial cystitis,painful bladder syndrome	[[1,1,1,0,0,0,0,1,1,0,0,0,1,0],[0,1,0,0,0,0,0,0,0,0,0,0,0,0],[1,0,0,0,0,0,0,0,1,0,0,0,0,0],[1,0,0,0,0,0,0,0,0,0,0,0,0,0],[0,0,0,0,0,0,0,1,0,0,0,0,0,0]]C0752193	schistosomal myeloradiculopathy	[[0,1,0,0,0,0,0,0,0,0,0,0,0,0]]C0752192	schistosomal myelitis	[[0,1,0,0,0,0,0,0,0,0,0,0,0,0]]C0404597	fetus with damage due to intrauterine contraceptive device with antenatal problem	[[1,0,0,0,0,0,0,0,0,0,0,0,0,0]]C1321863	infiltrating papillary adenocarcinoma	[[1,0,0,0,0,0,0,0,0,0,0,1,0,0]]C1321861	adenocarcinoma in adenomatous polyp,adenocarcinoma in a polyp,adenocarcinoma in polypoid adenoma,carcinoma in adenomatous polyp	[[1,0,0,0,0,0,0,0,0,0,0,1,0,0],[1,0,0,0,0,0,0,0,0,0,0,0,0,0],[1,0,0,0,0,0,0,0,0,0,0,0,0,0],[1,0,0,0,0,0,0,0,0,0,0,0,0,0]]C0752199	adult onset idiopathic torsion dystonias	[[0,1,0,0,0,0,0,0,0,0,0,0,0,0]]C0752198	adult onset idiopathic focal dystonias	[[0,1,0,0,0,0,0,0,0,0,0,0,0,0]]C1321865	juvenile astrocytoma,childhood astrocytic neoplasm,childhood astrocytic tumor,childhood astrocytic tumour,pediatric astrocytic neoplasm,pediatric astrocytic tumor	[[1,0,0,0,0,0,0,0,0,0,0,0,0,0],[0,0,0,0,0,0,0,0,0,0,0,1,0,0],[0,0,0,0,0,0,0,0,0,0,0,1,0,0],[0,0,0,0,0,0,0,0,0,0,0,1,0,0],[0,0,0,0,0,0,0,0,0,0,0,1,0,0],[0,0,0,0,0,0,0,0,0,0,0,1,0,0]]C1840560	familial hidradenitis suppurativa,familial acne inversa,1 familial acne inversa	[[0,1,0,1,0,0,0,0,0,0,0,0,0,0],[0,1,0,1,0,0,0,0,0,0,0,0,0,0],[0,0,0,1,0,0,0,0,0,0,0,0,0,0]]C1332562	bladder paraganglioma,urinary bladder paraganglioma	[[0,0,0,0,0,0,0,0,0,0,0,1,0,0],[0,0,0,0,0,0,0,0,0,0,0,1,0,0]]C0862030	b lymphoblastic leukemia lymphoma,precursor b lymphoblastic leukemia lymphoma	[[0,0,0,0,0,0,0,0,0,0,0,1,0,0],[0,0,0,0,0,0,0,0,0,0,0,1,0,0]]C1628317	serosal tuberculosis of cattle,pearly disease	[[1,0,0,0,0,0,0,0,0,0,0,0,0,0],[1,0,0,0,0,0,0,0,0,0,0,0,0,0]]C0008513	chorioretinitis,chorioretinitides,choriodoretinitis,chorioretinal inflammation,choroiditis and retinitis,retinochoroiditis	[[0,0,0,0,0,0,0,0,0,1,1,0,0,0],[0,1,0,0,0,0,0,0,0,0,0,0,0,0],[0,0,0,0,0,0,0,0,1,0,0,0,0,0],[1,0,0,0,0,0,0,0,0,0,0,0,0,0],[1,0,0,0,0,0,0,0,0,0,0,0,0,0],[1,0,0,0,0,0,0,1,0,0,0,0,0,0]]C0862039	stage ii b lymphoblastic lymphoma,precursor b lymphoblastic lymphoma stage ii	[[0,0,0,0,0,0,0,0,0,0,0,1,0,0],[0,0,0,0,0,0,0,0,0,0,0,1,0,0]]C0865072	benign neoplasm of eyebrow	[[0,0,0,0,0,0,0,0,0,0,0,0,0,1]]C1332561	bladder lymphoma,urinary bladder lymphoma,primary bladder lymphoma	[[0,0,0,0,0,0,0,0,0,0,0,1,0,0],[0,0,0,0,0,0,0,0,0,0,0,1,0,0],[0,0,0,0,0,0,0,0,0,0,0,1,0,0]]C1628319	lattice corneal dystrophy type ii,lcd2	[[1,0,0,1,0,0,0,0,0,0,0,0,0,0],[0,0,0,1,0,0,0,0,0,0,0,0,0,0]]C1306871	benign myoma of prostate	[[1,0,0,0,0,0,0,0,0,0,0,0,0,0]]C0015519	factor x deficiency,stuart prower deficiency,stuart prower factor deficiency,stuart prower disease	[[0,1,0,1,0,0,0,1,0,0,1,0,1,1],[0,1,0,0,0,0,0,0,0,0,0,0,1,0],[1,1,0,0,0,0,0,0,0,0,1,0,1,0],[0,0,0,0,0,0,0,0,0,0,0,0,0,1]]C1266110	malignant steroid cell tumor,malignant steroid cell tumour	[[1,0,0,0,0,0,0,0,0,0,0,0,0,0],[1,0,0,0,0,0,0,0,0,0,0,0,0,0]]C1333454	esophageal leiomyosarcoma,leiomyosarcoma of the esophagus	[[0,0,0,0,0,0,0,0,0,0,0,1,0,0],[0,0,0,0,0,0,0,0,0,0,0,1,0,0]]C0014647	giant cell epulis,giant cell epulides,peripheral giant cell granuloma,pgcg peripheral giant cell granuloma	[[0,1,0,0,0,0,0,1,0,0,0,0,0,1],[0,1,0,0,0,0,0,0,0,0,0,0,0,0],[0,1,0,0,0,0,0,1,0,0,0,0,0,1],[1,0,0,0,0,0,0,0,0,0,0,0,0,0]]C1333456	esophageal liposarcoma,liposarcoma of the esophagus	[[0,0,0,0,0,0,0,0,0,0,0,1,0,0],[0,0,0,0,0,0,0,0,0,0,0,1,0,0]]C0152198	disorder of accommodation,abnormality of accommodation,accommodation disturbance	[[1,0,0,0,1,0,0,0,1,0,0,0,0,0],[0,0,0,0,0,0,0,0,1,0,0,0,0,0],[0,0,0,0,0,0,0,0,1,0,0,0,0,0]]C1333450	esophageal high grade intraepithelial neoplasia,esophageal high grade dysplasia	[[0,0,0,0,0,0,0,0,0,0,0,1,0,0],[0,0,0,0,0,0,0,0,0,0,0,1,0,0]]C0521805	hereditary hypoplasminogenemia,hereditary hypoplasminogenaemia	[[1,0,0,0,0,0,0,0,0,0,0,0,0,0],[1,0,0,0,0,0,0,0,0,0,0,0,0,0]]C1333452	esophageal hodgkin lymphoma,esophageal hodgkins disease,hodgkins disease of the esophagus,hodgkins lymphoma of the esophagus,primary esophageal hodgkins lymphoma	[[0,0,0,0,0,0,0,0,0,0,0,1,0,0],[0,0,0,0,0,0,0,0,0,0,0,1,0,0],[0,0,0,0,0,0,0,0,0,0,0,1,0,0],[0,0,0,0,0,0,0,0,0,0,0,1,0,0],[0,0,0,0,0,0,0,0,0,0,0,1,0,0]]C1333453	esophageal kaposi sarcoma,esophagus kaposis sarcoma	[[0,0,0,0,0,0,0,0,0,0,0,1,0,0],[0,0,0,0,0,0,0,0,0,0,0,1,0,0]]C0152193	regular astigmatism	[[0,0,0,0,1,0,0,0,0,0,0,0,0,0]]C0570801	butobarbitone allergy,butobarbital allergy	[[1,0,0,0,0,0,0,0,0,0,0,0,0,0],[1,0,0,0,0,0,0,0,0,0,0,0,0,0]]C0152190	refractive amblyopia,ametropic amblyopia,meridional amblyopia	[[0,0,0,0,1,0,0,1,0,0,0,0,0,0],[1,0,0,0,0,0,0,0,0,0,0,0,0,0],[1,0,0,0,0,0,0,1,0,0,0,0,0,0]]C0152197	total internal ophthalmoplegia,complete internal ophthalmoplegia,total or complete internal ophthalmoplegia	[[1,0,0,0,0,0,0,0,0,0,0,0,0,0],[1,0,0,0,0,0,0,0,0,0,0,0,0,0],[0,0,0,0,1,0,0,0,0,0,0,0,0,0]]C0152196	spasm of accommodation,ciliary muscle spasm	[[1,0,0,0,1,0,0,1,1,0,0,0,0,0],[1,0,0,0,0,0,0,0,0,0,0,0,0,0]]C0152194	irregular astigmatism	[[0,0,0,0,1,0,0,1,0,0,0,0,0,0]]C3164835	anorectal tubulovillous adenoma,tubulovillous adenoma of anorectum	[[1,0,0,0,0,0,0,0,0,0,0,0,0,0],[1,0,0,0,0,0,0,0,0,0,0,0,0,0]]C2960605	mixed serous and mucinous cystadenocarcinoma	[[1,0,0,0,0,0,0,0,0,0,0,0,0,0]]C0570803	amylobarbitone sodium allergy,amobarbital sodium allergy	[[1,0,0,0,0,0,0,0,0,0,0,0,0,0],[1,0,0,0,0,0,0,0,0,0,0,0,0,0]]C1844908	with cataract 3 isolated microphthalmia,with microcornea and microphthalmia congenital cataract,microphthalmia and cataract 3,mcopct3	[[0,0,0,1,0,0,0,0,0,0,0,0,0,0],[0,0,0,1,0,0,0,0,0,0,0,0,0,0],[0,0,0,1,0,0,0,0,0,0,0,0,0,0],[0,0,0,1,0,0,0,0,0,0,0,0,0,0]]C1266111	malignant glomus tumor,malignant glomus tumour,glomangiosarcoma,glomoid sarcoma,malignant glomus neoplasm	[[1,0,0,0,0,0,0,0,0,0,0,1,0,0],[1,0,0,0,0,0,0,0,0,0,0,0,0,0],[0,0,0,0,0,0,0,0,0,0,0,1,0,0],[1,0,0,0,0,0,0,0,0,0,0,0,0,0],[0,0,0,0,0,0,0,0,0,0,0,1,0,0]]C2751189	darwinian notch	[[0,0,0,1,0,0,0,0,0,0,0,0,0,0]]C0019348	herpes simplex infections,herpes simplex,herpes simplex virus infection,herpes simplex disease,herpes simplex viral infection,herpesvirus hominis disease,herpes simplex complex	[[0,0,0,0,0,0,1,1,0,0,0,0,0,0],[0,0,1,0,0,0,1,1,0,0,0,0,0,0],[0,0,0,0,0,0,0,1,0,0,0,0,0,0],[0,0,0,0,0,0,0,1,0,1,0,0,0,0],[1,0,0,0,0,0,0,0,0,0,0,0,0,0],[0,0,0,0,0,0,0,0,0,1,0,0,0,0],[1,0,0,0,0,0,0,0,0,0,0,0,0,0]]C0153826	face and or neck leukemic reticuloendotheliosis of lymph nodes of head,face and or neck leukaemic reticuloendotheliosis of lymph nodes of head,and neck face leukemic reticuloendotheliosis involving lymph nodes of head	[[1,0,0,0,0,0,0,0,0,0,0,0,0,0],[1,0,0,0,0,0,0,0,0,0,0,0,0,0],[0,0,0,0,1,0,0,0,0,0,0,0,0,1]]C0153827	leukemic reticuloendotheliosis of intrathoracic lymph nodes,leukaemic reticuloendotheliosis of intrathoracic lymph nodes,leukemic reticuloendotheliosis involving intrathoracic lymph nodes	[[1,0,0,0,0,0,0,0,0,0,0,0,0,0],[1,0,0,0,0,0,0,0,0,0,0,0,0,0],[0,0,0,0,1,0,0,0,0,0,0,0,0,0]]C0272147	erythrocytosis due to hydronephrosis	[[1,0,0,0,0,0,0,0,0,0,0,0,0,0]]C0019345	herpes labialis,cold sore,fever blister,labial herpes simplex,herpes simplex labialis,fever sore,herpes febrilis,herpes on lip,lip cold sores	[[1,0,0,0,0,0,0,1,0,0,0,0,1,0],[1,1,0,0,0,0,0,1,0,1,1,0,1,0],[0,1,0,0,0,0,0,1,0,0,1,0,1,0],[0,1,0,0,0,0,0,0,0,0,0,0,1,0],[1,0,0,0,0,0,0,1,0,0,0,0,0,0],[0,0,0,0,0,0,0,1,1,0,0,0,0,0],[0,0,0,0,0,0,0,0,0,1,0,0,0,0],[0,0,0,0,0,0,0,1,0,0,0,0,0,0],[0,0,0,0,0,0,0,1,0,0,0,0,0,0]]C0272145	inappropriate secondary erythrocytosis	[[1,0,0,0,0,0,0,0,0,0,0,0,0,0]]C0019347	herpes progenitalis	[[0,0,1,0,0,0,0,0,0,0,0,0,0,1]]C0272143	erythrocytosis due to defective oxygen transport	[[1,0,0,0,0,0,0,0,0,0,0,0,0,0]]C0272142	erythrocytosis due to alveolar hypoventilation	[[1,0,0,0,0,0,0,0,0,0,0,0,0,0]]C0019342	genital herpes,herpes genitalis,genital herpes simplex,herpes genitalia,venereal herpes,genital hsv,herpes simplex virus genital infection	[[0,1,1,0,1,0,0,1,0,1,0,0,0,0],[0,0,1,0,0,0,1,1,0,0,0,0,0,0],[1,1,0,0,0,0,0,1,0,0,0,0,1,0],[0,0,0,0,0,0,1,0,0,0,0,0,0,0],[0,0,0,0,0,0,0,0,0,1,0,0,0,0],[0,0,0,0,0,0,0,1,0,0,0,0,0,0],[0,0,0,0,0,0,0,0,0,0,0,0,1,0]]C0272140	erythrocytosis due to pulmonary disease	[[1,0,0,0,0,0,0,0,0,0,0,0,0,0]]C0205646	basal cell adenoma,salivary gland basal cell adenoma	[[1,1,0,0,0,0,0,0,0,0,0,0,0,0],[0,0,0,0,0,0,0,0,0,0,0,1,0,0]]C0205647	follicular adenoma,thyroid follicular adenoma	[[1,1,0,0,0,0,0,1,0,0,0,0,0,0],[0,0,0,0,0,0,0,1,0,0,0,0,0,0]]C0205644	granular cell carcinoma,granular cell adenocarcinoma	[[1,1,0,0,0,0,0,0,0,0,0,0,0,0],[1,1,0,0,0,0,0,0,0,0,0,0,0,0]]C0205645	tubular adenocarcinoma,tubular carcinoma	[[1,1,0,0,0,0,0,1,0,0,0,0,0,0],[1,1,0,0,0,0,0,1,0,0,0,0,0,0]]C0205642	oxyphilic adenocarcinoma,oncocytic carcinoma,oncocytic adenocarcinoma,hurthle cell carcinoma,hurthle cell adenocarcinoma,oxyphilic cell follicular carcinoma	[[1,1,0,0,0,0,0,0,0,0,0,0,0,0],[1,0,0,0,0,0,0,1,0,0,0,1,0,0],[1,0,0,0,0,0,0,0,0,0,0,1,0,0],[1,0,0,0,0,0,0,1,0,0,0,1,0,0],[1,0,0,0,0,0,0,0,0,0,0,1,0,0],[1,0,0,0,0,0,0,0,0,0,0,0,0,0]]C0205643	cribriform carcinoma,cribriform type ductal carcinoma	[[1,1,0,0,0,0,0,1,0,0,0,0,0,0],[1,0,0,0,0,0,0,0,0,0,0,0,0,0]]C0857069	chronic candidiasis	[[0,0,0,1,0,0,0,1,0,0,0,1,0,0]]C0205641	basal cell adenocarcinoma,m basal cell adenocarcinoma	[[1,1,0,0,0,0,0,1,0,0,0,0,0,0],[1,0,0,0,0,0,0,0,0,0,0,0,0,0]]C0855081	stage iv mixed cellularity classical hodgkin lymphoma,mixed cellularity hodgkins disease stage iv,mixed cellularity hodgkins lymphoma stage iv	[[0,0,0,0,0,0,0,0,0,0,0,1,0,0],[0,0,0,0,0,0,0,0,0,0,0,1,0,0],[0,0,0,0,0,0,0,0,0,0,0,1,0,0]]C0205648	microcystic adenoma,m microcystic adenoma	[[1,1,0,0,0,0,0,0,0,0,0,0,0,0],[1,0,0,0,0,0,0,0,0,0,0,0,0,0]]C0205649	monomorphic adenoma,salivary gland monomorphic adenoma	[[1,1,0,0,0,0,0,1,0,0,0,0,0,0],[0,0,0,0,0,0,0,0,0,0,0,1,0,0]]C0855080	stage i mixed cellularity classical hodgkin lymphoma,stage i mixed cellularity hodgkins disease,mixed cellularity hodgkins lymphoma stage i	[[0,0,0,0,0,0,0,0,0,0,0,1,0,0],[0,0,0,0,0,0,0,0,0,0,0,1,0,0],[0,0,0,0,0,0,0,0,0,0,0,1,0,0]]C1862358	familial azotemia	[[0,0,0,1,0,0,0,0,0,0,0,0,0,0]]C0743190	diarrhea with hiv	[[0,0,0,0,0,0,0,1,0,0,0,0,0,0]]C0740345	germ cell cancer,malignant neoplasm of the germ cell,malignant germ cell tumor	[[0,1,0,0,0,0,0,1,0,0,0,0,0,0],[0,0,0,0,0,0,0,0,0,0,0,1,0,0],[0,0,0,0,0,0,0,0,0,0,0,1,0,0]]C0240164	squamous papilloma of the larynx,laryngeal papilloma,papilloma of the larynx	[[0,0,0,0,0,0,0,0,0,0,0,1,0,0],[0,0,0,0,0,0,0,1,0,0,1,1,0,0],[0,0,0,0,0,0,0,0,0,0,0,1,0,0]]C1278535	post infarct angina	[[1,0,0,0,0,0,0,0,0,0,0,0,0,0]]C1561857	hypersomnia due to medical condition classified elsewhere	[[0,0,0,0,1,0,0,0,0,0,0,0,0,0]]C0403670	urinary incontinence of non organic origin	[[1,0,0,0,0,0,0,0,0,0,0,0,0,0]]C1561852	organic disorders of initiating and maintaining sleep	[[0,0,0,0,1,0,0,0,0,0,0,0,0,0]]C1846837	familial thoracic 2 aortic aneurysm,aat2,faa2	[[0,0,0,1,0,0,0,0,0,0,0,0,0,0],[0,0,0,1,0,0,0,0,0,0,0,0,0,0],[0,0,0,1,0,0,0,0,0,0,0,0,0,0]]C0242997	arteriogenic impotence	[[0,1,0,0,0,0,0,0,0,0,0,0,0,0]]C0025210	ocular melanosis,melanosis oculi,ocular melanocytosis	[[1,0,0,0,0,0,0,0,0,0,0,0,0,0],[1,0,0,0,0,0,0,1,0,0,0,0,0,0],[1,0,0,0,0,0,0,1,0,0,0,0,0,0]]C0242994	hantavirus infections,disease due to hantaanvirus,disease due to hantanvirus,disease due to hantavirus	[[0,1,0,0,0,0,0,1,0,0,1,0,1,0],[1,0,0,0,0,0,0,0,0,0,0,0,0,0],[1,0,0,0,0,0,0,0,0,0,0,0,0,0],[1,0,0,0,0,0,0,0,0,0,0,0,0,0]]C0242993	nephropathia epidemica	[[0,1,0,0,0,0,0,0,0,0,1,0,0,0]]C0242992	multiple chemical sensitivity,multiple chemical sensitivity syndrome,idiopathic environmental intolerances	[[0,0,0,0,0,0,0,1,0,0,0,0,1,0],[0,0,0,0,0,0,0,0,0,0,0,0,1,0],[0,1,0,0,0,0,0,0,0,0,0,0,1,0]]C0025218	chloasma,melasma	[[1,1,1,0,0,0,0,1,0,0,0,0,0,0],[1,1,0,0,0,0,0,1,1,0,0,0,0,0]]C0242998	venogenic impotence,penile venous leakage	[[0,1,0,0,0,0,0,0,0,0,0,0,0,0],[0,1,0,0,0,0,0,0,0,0,0,0,0,0]]C0570818	medazepam allergy	[[1,0,0,0,0,0,0,0,0,0,0,0,0,0]]C0570819	oxazepam allergy	[[1,0,0,0,0,0,0,0,0,0,0,0,0,0]]C1522381	malignant mouse gastric neoplasm	[[0,0,0,0,0,0,0,0,0,0,0,1,0,0]]C1522383	mouse gastric adenocarcinoma	[[0,0,0,0,0,0,0,0,0,0,0,1,0,0]]C0570812	alprazolam allergy	[[1,0,0,0,0,0,0,0,0,0,0,0,0,0]]C0411159	fetus or neonate affected by maternal incompetent cervix	[[1,0,0,0,0,0,0,0,0,0,0,0,0,0]]C0570810	nitrazepam allergy	[[1,0,0,0,0,0,0,0,0,0,0,0,0,0]]C0570811	triazolam allergy	[[1,0,0,0,0,0,0,0,0,0,0,0,0,0]]C0570816	potassium clorazepate allergy,dipotassium clorazepate allergy	[[1,0,0,0,0,0,0,0,0,0,0,0,0,0],[1,0,0,0,0,0,0,0,0,0,0,0,0,0]]C0570817	ketazolam allergy	[[1,0,0,0,0,0,0,0,0,0,0,0,0,0]]C0570814	chlordiazepoxide allergy	[[1,0,0,0,0,0,0,0,0,0,0,0,0,0]]C0570815	clobazam allergy	[[1,0,0,0,0,0,0,0,0,0,0,0,0,0]]C2960274	ependymal cyst of ventricle of brain,cerebral ependymal cyst	[[1,0,0,0,0,0,0,0,0,0,0,0,0,0],[1,0,0,0,0,0,0,0,0,0,0,0,0,0]]C0853877	genitourinary fistula,urogenital fistula	[[0,0,0,0,0,0,0,0,0,0,0,1,0,0],[0,0,0,0,0,0,0,0,0,0,0,1,0,0]]C1266115	mucosal lentiginous melanoma,mucosal lentiginous malignant melanoma	[[1,0,0,0,0,0,0,0,0,0,0,1,0,0],[0,0,0,0,0,0,0,0,0,0,0,1,0,0]]C1862840	anonychia onychodystrophy,absent nails and dystrophic nails	[[0,1,0,1,0,0,0,0,0,0,0,0,0,0],[0,1,0,0,0,0,0,0,0,0,0,0,0,0]]C1858118	1b congenital muscular dystrophy,mdc1b	[[0,0,0,1,0,0,0,0,0,0,0,0,0,0],[0,0,0,1,0,0,0,0,0,0,0,0,0,0]]C1290168	osteomyelitis of frontal bone	[[1,0,0,0,0,0,0,0,0,0,0,0,0,0]]C1858114	huntington disease like 3,hdl3,autosomal recessive huntington disease like neurodegenerative disorder	[[0,0,0,1,0,0,1,0,0,0,0,0,0,0],[0,0,0,1,0,0,0,0,0,0,0,0,0,0],[0,0,0,1,0,0,0,0,0,0,0,0,0,0]]C1290169	osteomyelitis of zygomatic bone	[[1,0,0,0,0,0,0,0,0,0,0,0,0,0]]C1263913	benign neoplasm of peripheral nerves of face	[[1,0,0,0,0,0,0,0,0,0,0,0,0,0]]C0338327	untreated childhood brain stem glioma,brainstem glioma untreated childhood brain tumor,brainstem glioma untreated pediatric brain tumor,umtreated pediatric brain stem mixed glioma,childhood brainstem glioma untreated central nervous system tumor,pediatric brainstem glioma untreated central nervous system tumor,brainstem glioma untreated childhood cns tumor,childhood brainstem untreated mixed glioma,childhood brainstem untreated gliosarcoma,brainstem glioma untreated pediatric cns tumor,untreated pediatric brainstem mixed glioma,untreated pediatric brainstem glioma,untreated pediatric brainstem gliosarcoma,untreated childhood brain stem glioma brain tumor,untreated pediatric brain stem glioma brain tumor,childhood brain stem glioma untreated central nervous system tumor,pediatric brain stem glioma untreated central nervous system tumor,childhood untreated brain stem glioma cns tumor,untreated childhood brain stem mixed glioma,untreated childhood brain stem gliosarcoma,untreated pediatric brain stem glioma cns tumor,untreated pediatric brain stem mixed glioma,untreated pediatric brain stem glioma,pediatric brain stem untreated gliosarcoma,untreated childhood brainstem glioma	[[0,0,0,0,0,1,0,0,0,0,0,0,0,0],[0,0,0,0,0,1,0,0,0,0,0,0,0,0],[0,0,0,0,0,1,0,0,0,0,0,0,0,0],[0,0,0,0,0,1,0,0,0,0,0,0,0,0],[0,0,0,0,0,1,0,0,0,0,0,0,0,0],[0,0,0,0,0,1,0,0,0,0,0,0,0,0],[0,0,0,0,0,1,0,0,0,0,0,0,0,0],[0,0,0,0,0,1,0,0,0,0,0,0,0,0],[0,0,0,0,0,1,0,0,0,0,0,0,0,0],[0,0,0,0,0,1,0,0,0,0,0,0,0,0],[0,0,0,0,0,1,0,0,0,0,0,0,0,0],[0,0,0,0,0,1,0,0,0,0,0,0,0,0],[0,0,0,0,0,1,0,0,0,0,0,0,0,0],[0,0,0,0,0,1,0,0,0,0,0,0,0,0],[0,0,0,0,0,1,0,0,0,0,0,0,0,0],[0,0,0,0,0,1,0,0,0,0,0,0,0,0],[0,0,0,0,0,1,0,0,0,0,0,0,0,0],[0,0,0,0,0,1,0,0,0,0,0,0,0,0],[0,0,0,0,0,1,0,0,0,0,0,0,0,0],[0,0,0,0,0,1,0,0,0,0,0,0,0,0],[0,0,0,0,0,1,0,0,0,0,0,0,0,0],[0,0,0,0,0,1,0,0,0,0,0,0,0,0],[0,0,0,0,0,1,0,0,0,0,0,0,0,0],[0,0,0,0,0,1,0,0,0,0,0,0,0,0],[0,0,0,0,0,1,0,0,0,0,0,0,0,0]]C1263911	primary malignant neoplasm of peripheral nerves of head	[[1,0,0,0,0,0,0,0,0,0,0,0,0,0]]C1263910	benign neoplasm of peripheral nerves of head	[[1,0,0,0,0,0,0,0,0,0,0,0,0,0]]C1263917	primary malignant neoplasm of peripheral nerves of neck	[[1,0,0,0,0,0,0,0,0,0,0,0,0,0]]C1263916	benign neoplasm of peripheral nerves of neck	[[1,0,0,0,0,0,0,0,0,0,0,0,0,0]]C1263915	neoplasm of peripheral nerves of face	[[1,0,0,0,0,0,0,0,0,0,0,0,0,0]]C1263914	primary malignant neoplasm of peripheral nerves of face	[[1,0,0,0,0,0,0,0,0,0,0,0,0,0]]C1290111	neoplasm of skin of popliteal area	[[1,0,0,0,0,0,0,0,0,0,0,0,0,0]]C1290110	neoplasm of skin of thigh	[[1,0,0,0,0,0,0,0,0,0,0,0,0,0]]C1263919	benign neoplasm of peripheral nerves of upper limb	[[1,0,0,0,0,0,0,0,0,0,0,0,0,0]]C1263918	neoplasm of peripheral nerves of neck	[[1,0,0,0,0,0,0,0,0,0,0,0,0,0]]C1290115	neoplasm of skin of foot	[[1,0,0,0,0,0,0,0,0,0,0,0,0,0]]C1290114	neoplasm of skin of ankle	[[1,0,0,0,0,0,0,0,0,0,0,0,0,0]]C1290117	melanoma in situ of non skin site	[[1,0,0,0,0,0,0,0,0,0,0,0,0,0]]C0338329	recurrent childhood brain stem glioma,childhood recurrent brainstem glioma brain tumor,pediatric recurrent brainstem glioma brain tumor,recurrent pediatric brain stem glioma,recurrent brainstem glioma childhood cns tumor,recurrent childhood brainstem mixed glioma,recurrent childhood brainstem gliosarcoma,recurrent brainstem glioma pediatric cns tumor,recurrent pediatric brainstem mixed glioma,recurrent pediatric brainstem gliosarcoma,relapsed childhood glioma of the brainstem,relapsed pediatric glioma of the brainstem,childhood recurrent brain stem glioma brain tumor,pediatric recurrent brain stem glioma brain tumor,recurrent childhood brain stem glioma cns tumor,recurrent childhood brain stem mixed glioma,relapsed childhood brain stem glioma,recurrent childhood brain stem gliosarcoma,recurrent pediatric brain stem glioma cns tumor,recurrent pediatric brain stem mixed glioma,relapsed pediatric brain stem glioma,recurrent pediatric brain stem gliosarcoma,recurrent childhood brainstem glioma,recurrent pediatric brainstem glioma	[[0,0,0,0,0,1,0,0,0,0,0,1,0,0],[0,0,0,0,0,1,0,0,0,0,0,0,0,0],[0,0,0,0,0,1,0,0,0,0,0,0,0,0],[0,0,0,0,0,1,0,0,0,0,0,1,0,0],[0,0,0,0,0,1,0,0,0,0,0,0,0,0],[0,0,0,0,0,1,0,0,0,0,0,0,0,0],[0,0,0,0,0,1,0,0,0,0,0,0,0,0],[0,0,0,0,0,1,0,0,0,0,0,0,0,0],[0,0,0,0,0,1,0,0,0,0,0,0,0,0],[0,0,0,0,0,1,0,0,0,0,0,0,0,0],[0,0,0,0,0,0,0,0,0,0,0,1,0,0],[0,0,0,0,0,0,0,0,0,0,0,1,0,0],[0,0,0,0,0,1,0,0,0,0,0,0,0,0],[0,0,0,0,0,1,0,0,0,0,0,0,0,0],[0,0,0,0,0,1,0,0,0,0,0,0,0,0],[0,0,0,0,0,1,0,0,0,0,0,0,0,0],[0,0,0,0,0,0,0,0,0,0,0,1,0,0],[0,0,0,0,0,1,0,0,0,0,0,0,0,0],[0,0,0,0,0,1,0,0,0,0,0,0,0,0],[0,0,0,0,0,1,0,0,0,0,0,0,0,0],[0,0,0,0,0,0,0,0,0,0,0,1,0,0],[0,0,0,0,0,1,0,0,0,0,0,0,0,0],[0,0,0,0,0,1,0,0,0,0,0,1,0,0],[0,0,0,0,0,1,0,0,0,0,0,1,0,0]]C0274422	perfusion and or transfusion phlebitis following infusion	[[1,0,0,0,0,0,0,0,0,0,0,0,0,1]]C0274423	perfusion and or transfusion thrombophlebitis following infusion	[[1,0,0,0,0,0,0,0,0,0,0,0,0,1]]C0274421	vascular complication of medical care	[[1,0,0,0,0,0,0,0,0,0,0,0,0,0]]C1837972	myopia 5,myp5,autosomal dominant myopia 5	[[0,0,0,0,0,0,1,0,0,0,0,0,0,0],[0,0,0,1,0,0,0,0,0,0,0,0,0,0],[0,0,0,1,0,0,0,0,0,0,0,0,0,0]]C0741845	buttock tumor	[[0,0,0,0,0,0,0,1,0,0,0,0,0,0]]C0268707	uremic encephalopathy,uraemic encephalopathy	[[1,0,0,0,0,0,0,0,0,0,0,0,0,0],[1,0,0,0,0,0,0,0,0,0,0,0,0,0]]C1837974	lattice type iiia corneal dystrophy,cdl3a	[[0,0,0,1,0,0,0,0,0,0,0,0,0,0],[0,0,0,1,0,0,0,0,0,0,0,0,0,0]]C0268705	uremic acidosis,uraemic acidosis	[[1,0,0,0,0,0,0,0,0,0,0,0,0,0],[1,0,0,0,0,0,0,0,0,0,0,0,0,0]]C0268708	uremic neuropathy,uraemic neuropathy	[[1,0,0,0,0,0,0,0,0,0,0,1,0,0],[1,0,0,0,0,0,0,0,0,0,0,0,0,0]]C0268709	renal tubular defect	[[1,0,0,0,0,0,0,0,0,0,0,0,0,0]]C0278935	uterine sarcoma stage	[[0,0,0,0,0,1,0,0,0,0,0,0,0,0]]C1290162	disorder of smooth muscle,disease of smooth muscle	[[1,0,0,0,0,0,0,1,0,0,0,0,0,0],[1,0,0,0,0,0,0,1,0,0,0,0,0,0]]C1444123	vincents disease	[[1,0,0,0,0,0,0,0,0,0,0,0,0,0]]C1263929	primary malignant neoplasm of peripheral nerves of hip	[[1,0,0,0,0,0,0,0,0,0,0,0,0,0]]C1411965	clostridium bifermentans	[[0,0,0,0,0,0,1,0,0,0,0,0,0,0]]C1411964	mycobacterium ulcerans	[[0,0,0,0,0,0,1,0,0,0,0,0,0,0]]C1411966	clostridium difficile	[[0,0,0,0,0,0,1,0,0,0,0,0,0,0]]C1969024	1x dilated cardiomyopathy,cmd1x,with mild or no proximal muscle weakness dilated cardiomyopathy	[[0,0,0,1,0,0,0,0,0,0,0,0,0,0],[0,0,0,1,0,0,0,0,0,0,0,0,0,0],[0,0,0,1,0,0,0,0,0,0,0,0,0,0]]C1860849	multiple desmoplastic trichoepitheliomas	[[0,0,0,1,0,0,0,0,0,0,0,0,0,0]]C1969029	lissencephaly 3,lis3	[[0,0,0,1,0,0,0,0,0,0,0,0,0,0],[0,0,0,1,0,0,0,0,0,0,0,0,0,0]]C1274610	toxic epidermal necrolysis associated with infection	[[1,0,0,0,0,0,0,0,0,0,0,0,0,0]]C0456061	asymmetrical growth retardation	[[1,0,0,0,0,0,0,0,0,0,0,0,0,0]]C2698016	marfan syndrome type ii	[[0,0,0,0,0,0,0,0,0,0,0,1,0,0]]C1855731	benign recurrent intrahepatic 1 cholestasis,summerskill syndrome,bric1	[[0,1,0,1,0,0,0,0,0,0,0,0,0,0],[0,1,0,1,0,0,0,0,0,0,0,0,0,0],[0,0,0,1,0,0,0,0,0,0,0,0,0,0]]C1855732	intestinal pseudoobstruction with patent ductus arteriosus and natal teeth,intestinal pseudoobstruction and patent ductus natal teeth	[[0,1,0,1,0,0,0,0,0,0,0,0,0,0],[0,1,0,0,0,0,0,0,0,0,0,0,0,0]]C1855733	neuronal intestinal pseudoobstruction,deficiency of argyrophil myenteric plexus,type a neuronal intestinal dysplasia,neuronal type chronic idiopathic intestinal pseudoobstruction,intestinal pseudoobstruction due to neuronal disease,autosomal recessive familial visceral neuropathy,nid a	[[0,1,0,0,0,0,0,0,0,0,0,0,0,0],[0,1,0,1,0,0,0,0,0,0,0,0,0,0],[0,1,0,1,0,0,0,0,0,0,0,0,0,0],[0,1,0,1,0,0,0,0,0,0,0,0,0,0],[0,1,0,1,0,0,0,0,0,0,0,0,0,0],[0,0,0,1,0,0,0,0,0,0,0,0,0,0],[0,0,0,1,0,0,0,0,0,0,0,0,0,0]]C0864788	micropapular early yaws cutaneous	[[0,0,0,0,0,0,0,0,0,0,0,0,0,1]]C0864789	palmar or plantar due to yaws hyperkeratosis	[[0,0,0,0,0,0,0,0,0,0,0,0,0,1]]C1855737	immune defect due to inosine phosphorylase deficiency	[[0,0,0,1,0,0,0,0,0,0,0,0,0,0]]C1855738	indolylacroyl glycinuria with mental retardation	[[0,0,0,1,0,0,0,0,0,0,0,0,0,0]]C0864785	macular early yaws cutaneous	[[0,0,0,0,0,0,0,0,0,0,0,0,0,1]]C0864786	papular early yaws cutaneous	[[0,0,0,0,0,0,0,0,0,0,0,0,0,1]]C0864787	maculopapular early yaws cutaneous	[[0,0,0,0,0,0,0,0,0,0,0,0,0,1]]C0864783	plantar or palmar papilloma of yaws	[[0,0,0,0,0,0,0,0,0,0,0,0,0,1]]C0868952	dust diseases	[[0,0,0,0,0,0,0,1,0,0,0,0,0,0]]C1518731	ovarian sertoli leydig cell tumor with retiform elements	[[0,0,0,0,0,0,0,0,0,0,0,1,0,0]]C1518732	ovarian sertoli stromal cell tumor	[[0,0,0,0,0,0,0,0,0,0,0,1,0,0]]C1274611	toxic epidermal necrolysis erythema multiforme overlap syndrome,toxic epidermal necrolysis erythema multiforme overlap	[[1,0,0,0,0,0,0,0,0,0,0,0,0,0],[1,0,0,0,0,0,0,0,0,0,0,0,0,0]]C1518735	ovarian signet ring stromal tumor	[[0,0,0,0,0,0,0,0,0,0,0,1,0,0]]C1518736	hypercalcemic type ovarian small cell carcinoma	[[0,0,0,0,0,0,0,0,0,0,0,1,0,0]]C1518737	pulmonary type ovarian small cell carcinoma	[[0,0,0,0,0,0,0,0,0,0,0,1,0,0]]C1859648	and aspirin intolerance nasal polyps asthma,asa triad	[[0,0,0,1,0,0,0,0,0,0,0,0,0,0],[0,0,0,1,0,0,0,0,0,0,0,0,0,0]]C1518739	ovarian squamous cell tumor,ovarian squamous cell neoplasm	[[0,0,0,0,0,0,0,0,0,0,0,1,0,0],[0,0,0,0,0,0,0,0,0,0,0,1,0,0]]C1864549	with spasticity and pigmentary tapetoretinal degeneration severe mental retardation,mrst	[[0,0,0,1,0,0,0,0,0,0,0,0,0,0],[0,0,0,1,0,0,0,0,0,0,0,0,0,0]]C1522658	choriocarcinoma of the mouse nervous system	[[0,0,0,0,0,0,0,0,0,0,0,1,0,0]]C1522659	dysplastic lhermitte duclos gangliocytoma of mouse cerebellum	[[0,0,0,0,0,0,0,0,0,0,0,1,0,0]]C0235287	dysosmia,alteration smell,changes smell,altered sense of smell,dysomia,parosmia,cacosmia	[[0,1,0,0,0,0,0,1,0,0,0,0,0,0],[0,0,0,0,0,0,0,1,0,0,0,0,0,0],[0,0,0,0,0,0,0,1,0,0,0,0,0,0],[0,0,0,0,0,0,0,1,0,0,0,0,0,0],[0,0,0,0,0,0,0,1,1,0,0,0,0,0],[0,0,0,0,0,0,0,1,0,0,0,0,0,0],[0,1,0,0,0,0,0,1,0,0,0,0,0,0]]C1522656	mouse glioblastoma	[[0,0,0,0,0,0,0,0,0,0,0,1,0,0]]C1522657	diffuse melanocytosis of the mouse nervous system	[[0,0,0,0,0,0,0,0,0,0,0,1,0,0]]C1857776	dcma,with ataxia dilated cardiomyopathy,type v mga,mga5	[[0,0,0,1,0,0,0,0,0,0,0,0,0,0],[0,0,0,1,0,0,0,0,0,0,0,0,0,0],[0,0,0,1,0,0,0,0,0,0,0,0,0,0],[0,0,0,1,0,0,0,0,0,0,0,0,0,0]]C1857777	10 familial arrhythmogenic right ventricular dysplasia,arvc10,arrhythmogenic right ventricular cardiomyopathy 10,arvd10	[[0,0,0,1,0,0,0,0,0,0,0,0,0,0],[0,0,0,1,0,0,0,0,0,0,0,0,0,0],[0,0,0,1,0,0,0,0,0,0,0,0,0,0],[0,0,0,1,0,0,0,0,0,0,0,0,0,0]]C1857775	with congenital hypothyroidism neonatal diabetes mellitus,ndh syndrome	[[0,0,0,1,0,0,0,0,0,0,0,0,0,0],[0,0,0,1,0,0,0,0,0,0,0,0,0,0]]C0280199	adult acute lymphoblastic leukemia stage,adult acute lymphocytic leukemia stage	[[0,0,0,0,0,1,0,0,0,0,0,0,0,0],[0,0,0,0,0,1,0,0,0,0,0,0,0,0]]C0280198	adrenocortical carcinoma stage	[[0,0,0,0,0,1,0,0,0,0,0,0,0,0]]C1857771	juvenile onset cortical pulverulent cataracts	[[0,0,0,1,0,0,0,0,0,0,0,0,0,0]]C0280197	islet cell carcinoma stage type,islet cell carcinoma stage	[[0,0,0,0,0,1,0,0,0,0,0,0,0,0],[0,0,0,0,0,1,0,0,0,0,0,0,0,0]]C0280196	intraocular melanoma stage	[[0,0,0,0,0,1,0,0,0,0,0,0,0,0]]C0280191	recurrent adult lymphoblastic lymphoma,relapsed adult lymphoblastic lymphoma,recurrent adult precursor lymphoblastic lymphoma	[[0,0,0,0,0,1,0,0,0,0,0,1,0,0],[0,0,0,0,0,1,0,0,0,0,0,0,0,0],[0,0,0,0,0,0,0,0,0,0,0,1,0,0]]C0280190	recurrent adult immunoblastic large cell lymphoma,relapsed adult immunoblastic large cell lymphoma,recurrent adult immunoblastic lymphoma,relapsed adult immunoblastic lymphoma	[[0,0,0,0,0,1,0,0,0,0,0,1,0,0],[0,0,0,0,0,1,0,0,0,0,0,1,0,0],[0,0,0,0,0,0,0,0,0,0,0,1,0,0],[0,0,0,0,0,0,0,0,0,0,0,1,0,0]]C0280193	adult non hodgkins lymphoma grade,adult nonhodgkins lymphoma grade	[[0,0,0,0,0,1,0,0,0,0,0,1,0,0],[0,0,0,0,0,0,0,0,0,0,0,1,0,0]]C0280192	recurrent adult burkitts lymphoma,adult recurrent small noncleaved cell lymphoma,relapsed adult small noncleaved cell lymphoma,recurrent adult diffuse small noncleaved cell burkitts lymphoma	[[0,0,0,0,0,1,0,0,0,0,0,1,0,0],[0,0,0,0,0,1,0,0,0,0,0,0,0,0],[0,0,0,0,0,1,0,0,0,0,0,0,0,0],[0,0,0,0,0,1,0,0,0,0,0,0,0,0]]C0269073	chronic cystic cervicitis,nabothian cervicitis	[[1,0,0,0,0,0,0,0,0,0,0,0,0,0],[1,0,0,0,0,0,0,0,0,0,0,0,0,0]]C0269072	chronic endocervicitis with ectropion	[[1,0,0,0,0,0,0,0,0,0,0,0,0,0]]C0269071	chronic endocervicitis with erosion	[[1,0,0,0,0,0,0,0,0,0,0,0,0,0]]C0269070	endocervicitis with ectropion	[[1,0,0,0,0,0,0,0,0,0,0,0,0,0]]C0269077	chronic vaginitis	[[1,0,0,0,0,0,0,0,0,0,0,0,0,0]]C0269076	mucopurulent vaginitis	[[1,0,0,0,0,0,0,0,0,0,0,0,0,0]]C0269075	acute vaginitis	[[1,0,0,0,0,0,0,0,0,0,0,0,0,0]]C0269079	postirradiation vaginitis,post irradiation vaginitis	[[1,0,0,0,0,0,0,0,0,0,0,0,0,1],[1,0,0,0,0,0,0,0,0,0,0,0,0,0]]C0269078	granular vaginitis	[[1,0,0,0,0,0,0,0,0,0,0,0,0,0]]C0574014	axillary vein thrombosis	[[1,0,0,0,0,0,0,0,0,0,0,0,0,0]]C0574015	intracranial venous thrombosis	[[1,0,0,0,0,0,0,0,0,0,0,0,0,0]]C1518882	bladder papillary urothelial carcinoma	[[0,0,0,0,0,0,0,0,0,0,0,1,0,0]]C0574013	superficial femoral artery thrombosis	[[1,0,0,0,0,0,0,0,0,0,0,0,0,0]]C0574010	internal iliac artery thrombosis	[[1,0,0,0,0,0,0,0,0,0,0,0,0,0]]C0574011	profunda femoris artery thrombosis	[[1,0,0,0,0,0,0,0,0,0,0,0,0,0]]C0543669	hereditary neutrophilia	[[1,0,0,1,0,0,0,0,0,0,0,0,0,0]]C1321493	kobner reaction from lichen planus	[[1,0,0,0,0,0,0,0,0,0,0,0,0,0]]C0574018	duodenal cyst	[[1,0,0,0,0,0,0,0,0,0,0,0,0,0]]C0574019	duodenal web,duodenal diaphragm	[[1,0,0,0,0,0,0,0,0,0,0,0,0,0],[1,0,0,0,0,0,0,0,0,0,0,0,0,0]]C0865787	fetid recurrent bronchitis	[[0,0,0,0,0,0,0,0,0,0,0,0,0,1]]C0865785	influenzal acute respiratory infection	[[0,0,0,0,0,0,0,0,0,0,0,0,0,1]]C0865784	influenzal upper respiratory infection	[[0,0,0,0,0,0,0,0,0,0,0,0,0,1]]C0865783	influenzal respiratory infection	[[0,0,0,0,0,0,0,0,0,0,0,0,0,1]]C0865782	pneumonia due to serratia marcescens	[[0,0,0,0,0,0,0,0,0,0,0,0,0,1]]C0865781	pneumonia due to gram negative anaerobes	[[0,0,0,0,0,0,0,0,0,0,0,0,0,1]]C0865780	pneumonia due to bacteroides melaninogenicus	[[0,0,0,0,0,0,0,0,0,0,0,0,0,1]]C1321490	eczema craquele due to acute edema,eczema craquele due to acute oedema	[[1,0,0,0,0,0,0,0,0,0,0,0,0,0],[1,0,0,0,0,0,0,0,0,0,0,0,0,0]]C0865789	purulent recurrent bronchitis	[[0,0,0,0,0,0,0,0,0,0,0,0,0,1]]C0865788	mucopurulent bronchitis	[[0,0,0,0,0,0,0,0,0,0,0,0,0,1]]C0423061	intermittent convergent squint,intermittent esotropia,intermittent comitant esotropia	[[1,0,0,0,0,0,0,0,0,0,0,0,0,0],[1,1,0,0,0,0,0,0,0,0,0,0,0,1],[1,0,0,0,0,0,0,0,0,0,0,0,0,0]]C0423062	intermittent divergent squint,intermittent exotropia,intermittent comitant exotropia,x t intermittent exotropia	[[1,0,0,0
[truncated: 1,200,000 more chars]
