# Supplementary material for: Quantifying the Impact and Extent of Undocumented Biomedical Synonymy
Source: PLoS Comput Biol. 2014 Sep 25;10(9):e1003799. doi: 10.1371/journal.pcbi.1003799 (PMC4177665; doi:10.1371/journal.pcbi.1003799)
Supplement: Dataset S3 — The Pharmacological Substances synonym dataset. The format of this file is identical to that of Dataset S1. See Supporting Information Text S1 for the processing procedures that resulted in this dataset. (ZIP) [file pcbi.1003799.s003.zip › SupportingDataset_S3.rtf]

Vocab Key: SNOMEDCT,MSH,MTHSPL,LNC,PDQ,MTH,CHV,RXNORM,CSP,NCI,NDFRTConcept ID#	Variant List	AnnotationsC0220596	decynium 22	[[0,1,0,0,0,0,0,0,0,0,0]]C0220595	1 1 diethyl 2 2 cyanine	[[0,1,0,0,0,0,0,0,0,0,0]]C0056873	cysteine hydrazide,hydrazide l cysteine	[[0,1,0,0,0,0,0,0,0,0,0],[0,1,0,0,0,0,0,0,0,0,0]]C0539619	4 fpp bsn	[[0,1,0,0,0,0,0,0,0,0,0]]C0878348	nsc 104800,nsc104800	[[0,1,0,0,0,0,0,0,0,0,0],[0,1,0,0,0,0,0,0,0,0,0]]C1165386	egg yolk phosphatides	[[0,0,0,0,0,0,0,1,0,0,0]]C0056878	cysteine sulfinic acid,alanine 3 sulfinic acid,cysteine hydrogen sulfite ester	[[0,1,0,0,0,0,0,0,0,0,0],[0,1,0,0,0,0,0,0,0,0,0],[0,1,0,0,0,0,0,0,0,0,0]]C0719301	chlorafed timecelles	[[0,0,0,0,0,0,0,1,0,0,0]]C0719300	chlorafed h s timecelles	[[0,0,0,0,0,0,0,1,0,0,0]]C0002575	aminophylline,theophyllamine,theophylline ethylenediamine,compd with 1 2 ethanediamine 3 7 dihydro 1 3 dimethyl 1h purine 2 6 dione,compound with 1 2 ethanediamine 3 7 dihydro 1 3 dimethyl 1h purine 2 6 dione,ammophyllin,theophyline ethylenediamine	[[0,1,0,0,0,0,1,0,1,1,0],[0,0,0,0,0,0,1,0,0,0,1],[0,1,0,0,0,0,0,0,1,0,1],[0,0,0,0,0,0,0,0,0,0,1],[0,0,0,0,0,0,0,0,0,1,0],[0,0,0,0,0,0,0,0,0,1,0],[0,0,0,0,0,0,0,0,0,1,0]]C0720918	hycosin expectorant	[[0,0,0,0,0,0,0,1,0,0,0]]C0055789	cirsimaritin,4 5 dihydroxy 6 7 dimethoxyflavone,5 hydroxy 2 4 hydroxyphenyl 6 7 dimethoxy 4h 1 benzopyran 4 one,skrofulein,4 5 dihydroxy 6 7 dimethoxy flavone	[[0,1,0,0,0,0,0,0,0,0,0],[0,1,0,0,0,0,0,0,0,0,0],[0,1,0,0,0,0,0,0,0,0,0],[0,1,0,0,0,0,0,0,0,0,0],[0,1,0,0,0,0,0,0,0,0,0]]C0719307	chlordrine sr	[[0,0,0,0,0,0,0,1,0,0,0]]C0719306	chlordinium	[[0,0,0,0,0,0,0,1,0,0,0]]C0719309	chloresium topical	[[0,0,0,0,0,0,0,1,0,0,0]]C0719308	chloresium	[[0,0,0,0,0,0,0,1,0,0,0]]C0720916	hycophen pediatric	[[0,0,0,0,0,0,0,1,0,0,0]]C0720917	hycort	[[0,0,0,0,0,0,0,1,0,0,0]]C0720910	hycomal dh	[[0,0,0,0,0,0,0,1,0,0,0]]C0720911	hycomed	[[0,0,0,0,0,0,0,1,0,0,0]]C0720912	hycomine	[[0,0,0,0,0,0,0,1,0,0,0]]C0055783	cirkan n d	[[0,1,0,0,0,0,0,0,0,0,0]]C0130359	n 4 methylbenzylthiocarbonyl phenylalanine	[[0,1,0,0,0,0,0,0,0,0,0]]C0130358	n 4 methylbenzylthiocarbonyl l phenylalanine	[[0,1,0,0,0,0,0,0,0,0,0]]C0606387	tropinoylcholine,bromide r 2 3 hydroxy 1 oxo 2 phenylpropoxy n n n trimethylethanaminium	[[0,1,0,0,0,0,0,0,0,0,0],[0,1,0,0,0,0,0,0,0,0,0]]C0285969	omptin,ompt protease,protease a,outer membrane protein 3b,omptin outer membrane protease	[[1,1,0,0,0,0,0,0,0,0,0],[0,1,0,0,0,0,0,0,0,0,0],[1,0,0,0,0,0,0,0,0,0,0],[1,0,0,0,0,0,0,0,0,0,0],[0,1,0,0,0,0,0,0,0,0,0]]C1564310	butaliret,fatol brand of terbutaline sulfate,butalitab	[[0,1,0,0,0,0,0,0,0,0,0],[0,1,0,0,0,0,0,0,0,0,0],[0,1,0,0,0,0,0,0,0,0,0]]C0048656	4 o glucopyranosylmoranoline	[[0,1,0,0,0,0,0,0,0,0,0]]C0644551	pd 130883,2 amino 6 4 nitrophenyl 2 propynylamino methyl 4 1h quinazolinone	[[0,1,0,0,0,0,0,0,0,0,0],[0,1,0,0,0,0,0,0,0,0,0]]C1171789	6 1 hydroxyethyl 3 4 2 hydroxymethyl 1 2 3 6 tetrahydropyridin 4 yl 1 3 thiazol 2 yl thio 4 methyl 7 oxo 1 azabicyclo 3 2 0 hept 2 ene 2 carboxylic acid	[[0,1,0,0,0,0,0,0,0,0,0]]C0644555	tegin g	[[0,1,0,0,0,0,0,0,0,0,0]]C0644554	bis maleimidoethoxy propane,bismaleimidoethoxy propane,bmiep	[[0,1,0,0,0,0,0,0,0,0,0],[0,1,0,0,0,0,0,0,0,0,0],[0,1,0,0,0,0,0,0,0,0,0]]C1171785	3 4 2 5 dihydro 1h pyrrol 3 yl 1 3 thiazol 2 yl thio 6 1 hydroxyethyl 4 methyl 7 oxo 1 azabicyclo 3 2 0 hept 2 ene 2 carboxylic acid	[[0,1,0,0,0,0,0,0,0,0,0]]C1171786	sm 197436,sm197436	[[0,1,0,0,0,0,0,0,0,0,0],[0,1,0,0,0,0,0,0,0,0,0]]C1171787	6 1 hydroxyethyl 4 methyl 3 4 2 methyl 1 2 3 6 tetrahydropyridin 4 yl 1 3 thiazol 2 yl thio 7 oxo 1 azabicyclo 3 2 0 hept 2 ene 2 carboxylic acid	[[0,1,0,0,0,0,0,0,0,0,0]]C0383382	cis 5 6a 7 8 9 9a hexahydro 2 4 trifluoromethyl phenylmethyl 5 methyl cyclopent 4 5 imidazo 2 1 b purin 4 3h one	[[0,1,0,0,0,0,0,0,0,0,0]]C0383380	rhodiocyanoside b	[[0,1,0,0,0,0,0,0,0,0,0]]C0383381	sch 51866	[[0,1,0,0,0,0,0,0,0,0,0]]C0383386	tetramethyl hematoporphyrin	[[0,1,0,0,0,0,0,0,0,0,0]]C1529946	rhinoclir,febena brand of dexpanthenol,siozwo sana	[[0,1,0,0,0,0,0,0,0,0,0],[0,1,0,0,0,0,0,0,0,0,0],[0,1,0,0,0,0,0,0,0,0,0]]C1235657	frumax	[[0,0,0,0,0,0,0,1,0,0,0]]C0106364	biotetrussin	[[0,1,0,0,0,0,0,0,0,0,0]]C0601639	16 alpha methyldichlorosone,9 alpha 11 beta dichloro 17 alpha 21 dihydroxy 16 alpha methyl 1 4 pregnadiene 3 20 dione	[[0,1,0,0,0,0,0,0,0,0,0],[0,1,0,0,0,0,0,0,0,0,0]]C1630404	purine derivative diuretic	[[1,0,0,0,0,0,0,0,0,0,0]]C0601633	mcn 2891,5 4 chlorobenzoyl alpha 1 4 trimethyl 1h pyrrole 2 acetic acid	[[0,1,0,0,0,0,0,0,0,0,0],[0,1,0,0,0,0,0,0,0,0,0]]C0601631	5 p chlorobenzoyl 1 4 alpha trimethylpyrrole 2 acetic acid	[[0,1,0,0,0,0,0,0,0,0,0]]C0058206	dimethoxon,o o dimethyl s methylcarbamoyl methyl phosphorothioate,omethoate,o methoate	[[0,1,0,0,0,0,0,0,0,0,0],[0,1,0,0,0,0,0,0,0,0,0],[0,1,0,0,0,0,0,0,0,0,0],[0,1,0,0,0,0,0,0,0,0,0]]C0720091	duramorph pf	[[0,0,0,0,0,0,0,1,0,0,0]]C0058204	dimethophrine,1 4 hydroxy 3 5 dimethoxyphenyl 2 methylaminoethanol,dimetofrine,dimetrophine	[[0,1,0,0,0,0,0,0,0,0,0],[0,1,0,0,0,0,0,0,0,0,0],[0,1,0,0,0,0,0,0,0,1,0],[0,1,0,0,0,0,0,0,0,0,0]]C0058205	dimethoxane,6 acetoxy 2 4 dimethyl m dioxane,acetomethoxane	[[1,1,0,0,0,0,0,0,0,0,0],[0,1,0,0,0,0,0,0,0,0,0],[1,0,0,0,0,0,0,0,0,0,0]]C0058202	dimethisoquin,3 butyl 1 2 dimethylaminoethoxy isoquinoline,quinisocaine	[[0,1,0,0,0,0,0,0,0,1,0],[0,1,0,0,0,0,0,0,0,0,0],[0,1,0,0,0,0,0,0,0,1,0]]C3254919	biocorneum	[[0,0,0,0,0,0,0,1,0,0,0]]C3205052	hypromellose 2906	[[0,0,1,0,0,0,0,0,0,0,0]]C0058201	dimethiodal,disodium salt diiodomethanesulfonic acid,dimethiodal sodium,diiodomethanesulfonate,diiodomethanesulfonic acid	[[0,1,0,0,0,0,0,0,0,1,0],[0,1,0,0,0,0,0,0,0,0,0],[0,1,0,0,0,0,0,0,0,1,0],[0,0,0,0,0,0,0,0,0,1,0],[0,0,0,0,0,0,0,0,0,1,0]]C0528541	l 745870,l 745 870,l745 870,l745870	[[0,1,0,0,0,0,0,0,0,0,0],[0,1,0,0,0,0,0,0,0,0,0],[0,1,0,0,0,0,0,0,0,0,0],[0,1,0,0,0,0,0,0,0,0,0]]C1881807	metioxate	[[0,0,0,0,0,0,0,0,0,1,0]]C0075455	succinylacetone,4 6 dioxo heptanoic acid,4 6 dioxoheptanoic acid	[[0,1,0,1,0,0,0,0,0,0,0],[0,1,0,0,0,0,0,0,0,0,0],[0,1,0,0,0,0,0,0,0,0,0]]C0136984	phyllitous silicate	[[0,1,0,0,0,0,0,0,0,0,0]]C0591962	pentostam,glaxosmithkline brand of sodium stibogluconate	[[0,0,0,0,1,0,1,0,0,0,0],[0,1,0,0,0,0,0,0,0,0,0]]C0088858	1 4 hydroxy 3 5 ditert butylbenzoyl homopiperazine	[[0,1,0,0,0,0,0,0,0,0,0]]C1881805	methylchromone	[[0,0,0,0,0,0,0,0,0,1,0]]C0007310	caryolysine,genopharm brand of mechlorethamine hydrochloride	[[0,0,0,0,0,0,0,0,0,1,0],[0,1,0,0,0,0,0,0,0,0,0]]C2938539	sensorcaine with epinephrine 0 25 1 200000	[[0,0,0,0,0,0,0,1,0,0,0]]C0137791	polygynax	[[0,1,0,0,0,0,0,0,0,0,0]]C0720914	hycomine pediatric	[[0,0,0,0,0,0,0,1,0,0,0]]C2346745	amiperone	[[0,0,0,0,0,0,0,0,0,1,0]]C0965129	rosuvastatin,3r 5s 6e 7 4 4 fluorophenyl 6 1 methylethyl 2 ethyl methylsulfonyl amino 5 pyrimidinyl 3 5 dihydroxy 6 heptenoic acid	[[0,0,0,0,0,0,0,1,0,1,0],[0,0,0,0,0,0,0,0,0,0,1]]C0003620	appetite depressants,anorectics,appetite suppressants,anorexic agent,anorexics,anorexigenic drugs,appetite depressing drugs,anorexic drugs,appetite suppressant drugs,anorectic agents,anorexiant,anorexiant product	[[0,1,0,0,0,0,1,0,0,0,0],[0,1,0,0,0,0,1,0,0,0,0],[0,1,0,0,0,0,1,0,0,1,1],[0,0,0,0,0,0,1,0,1,1,0],[0,0,0,0,0,0,0,0,0,1,0],[0,1,0,0,0,0,0,0,1,0,0],[0,1,0,0,0,0,0,0,0,0,0],[0,1,0,1,0,0,1,0,0,0,0],[0,1,0,0,0,0,1,0,0,0,0],[0,1,0,0,0,0,1,0,0,0,0],[1,0,0,0,0,0,1,0,0,0,0],[1,0,0,0,0,0,0,0,0,0,0]]C0540267	chloroquine triphenylphosphine gold i,au pph3 cq pf6	[[0,1,0,0,0,0,0,0,0,0,0],[0,1,0,0,0,0,0,0,0,0,0]]C0540265	1 3 diamino 8 3 4 5 trimethoxyphenyl 5 6 6a 7 8 9 10 10a octahydropyrimido 4 5 c 2 7 naphthyridine,dtop naphthyridine	[[0,1,0,0,0,0,0,0,0,0,0],[0,1,0,0,0,0,0,0,0,0,0]]C0540263	1 3 diamino 8 3 4 5 trimethoxyphenyl pyrimido 4 5 c 2 7 naphthyridin 6 one,datp pno	[[0,1,0,0,0,0,0,0,0,0,0],[0,1,0,0,0,0,0,0,0,0,0]]C0540261	n 2 dimethylamino ethyl 5 methyl 4 acridinecarboxamide,daem acridinecarboxamide	[[0,1,0,0,0,0,0,0,0,0,0],[0,1,0,0,0,0,0,0,0,0,0]]C0678203	depostat,primostat,schering brand of gestonorone caproate	[[0,1,0,0,0,0,0,0,0,0,0],[0,1,0,0,0,0,0,0,0,0,0],[0,1,0,0,0,0,0,0,0,0,0]]C1881808	metipirox	[[0,0,0,0,0,0,0,0,0,1,0]]C0678207	nimotop,bayer brand of nimodipine	[[0,0,0,0,0,0,1,1,0,0,0],[0,1,0,0,0,0,0,0,0,0,0]]C0678204	diamicron,servier brand of gliclazide	[[0,1,0,0,0,0,1,0,0,0,0],[0,1,0,0,0,0,0,0,0,0,0]]C0089188	1 chloro 4 isothiocyanatobenzene,4 chlorophenyl isothiocyanate,p chlorophenyl isothiocyanate	[[0,1,0,0,0,0,0,0,0,0,0],[0,1,0,0,0,0,0,0,0,0,0],[0,1,0,0,0,0,0,0,0,0,0]]C0054435	benzoate caffeine	[[0,0,0,0,0,0,0,1,0,0,0]]C0054434	caffeic acid phenethyl ester,capeee,phenethyl caffeate,cape,cape compound,2 phenylethyl ester 3 3 4 dihydroxyphenyl 2 propenoic acid,2 phenylethyl 3 3 4 dihydroxyphenyl 2 propenoate	[[0,1,0,0,0,0,0,0,0,1,0],[0,1,0,0,0,0,0,0,0,0,0],[0,1,0,0,0,0,0,0,0,0,0],[0,0,0,0,0,0,0,0,0,1,0],[0,1,0,0,0,0,0,0,0,0,0],[0,0,0,0,0,0,0,0,0,1,0],[0,0,0,0,0,0,0,0,0,1,0]]C0054436	caffeine citrate,caffeina citrata	[[1,0,0,0,0,0,1,1,0,1,1],[0,0,0,0,0,0,0,0,0,1,0]]C0054430	cafergot,ergotamine drug combination alliance pharmaceuticals brand of caffeine,ergotamine drug combination novartis brand of caffeine	[[0,1,0,0,0,0,1,0,0,0,0],[0,1,0,0,0,0,0,0,0,0,0],[0,1,0,0,0,0,0,0,0,0,0]]C0054433	caffeic acid,3 4 dihydroxycinnamic acid,e 3 3 4 dihydroxyphenyl prop 2 enoic acid,trans 3 4 dihydroxycinnamic acid	[[0,0,0,0,0,0,1,0,0,1,0],[0,1,0,0,0,0,0,0,0,0,0],[0,0,0,0,0,0,0,0,0,1,0],[0,0,0,0,0,0,0,0,0,1,0]]C0960981	3 tricarbonylcyclopentadienylrhenium sch 23390,3 cptr sch 23390	[[0,1,0,0,0,0,0,0,0,0,0],[0,1,0,0,0,0,0,0,0,0,0]]C0960980	2 tricarbonylcyclopentadienylrhenium sch 23390,2 cptr sch 23390	[[0,1,0,0,0,0,0,0,0,0,0],[0,1,0,0,0,0,0,0,0,0,0]]C0960983	4 4 morpholinyl thiocarbonylsulfenylamino ethyl benzenesulfonamide,mts etbsa	[[0,1,0,0,0,0,0,0,0,0,0],[0,1,0,0,0,0,0,0,0,0,0]]C0960982	4 4 morpholinylthiocarbonylsulfenylamino methyl benzenesulfonamide,mts mebsa	[[0,1,0,0,0,0,0,0,0,0,0],[0,1,0,0,0,0,0,0,0,0,0]]C0960985	4 azidophenyl galactopyranoside	[[0,1,0,0,0,0,0,0,0,0,0]]C0960984	2 benzofuranylmethoxycarboyl alpha methyl 1 4 phosphoryloxymethyl benzoyl methoxycarbonyl tryptophan n 1 phenylethyl amide,phospho pd154075 prodrug	[[0,1,0,0,0,0,0,0,0,0,0],[0,1,0,0,0,0,0,0,0,0,0]]C0068978	cafedrine,norephendrinetheophylline,7 2 1 methyl 2 hydroxy 2 phenylethylamino ethyl theophylline	[[0,0,0,0,0,0,0,1,0,1,0],[0,1,0,0,0,0,0,0,0,0,0],[0,1,0,0,0,0,0,0,0,0,0]]C0068977	nordinone,17 17 dimethyl 18 norandrosta 4 13 dien 11 alpha ol 3 one	[[0,1,0,0,0,0,0,0,0,0,0],[0,1,0,0,0,0,0,0,0,0,0]]C0960988	8 methylureido 4 10 dihydro 4 oxo 4h imidazo 1 2 a indeno 1 2 e pyrazin 2 carboxylic acid,8 methylureido doiipca	[[0,1,0,0,0,0,0,0,0,0,0],[0,1,0,0,0,0,0,0,0,0,0]]C0720913	hycomine compound	[[0,0,0,0,0,0,0,1,0,0,0]]C0068972	norcocaine	[[0,1,0,0,0,0,0,0,0,0,0]]C0068971	norclozapine,n desmethylclozapine	[[0,1,0,1,0,0,0,0,0,0,0],[0,1,0,0,0,0,0,0,0,0,0]]C0698297	compound antidepressants a z	[[1,0,0,0,0,0,0,0,0,0,0]]C0031448	phentolamine,fentolamin,ptm,3 4 5 dihydro 1h imidazol 2 yl methyl 4 methylphenyl amino phenol,2 n p tolyl n m hydroxyphenyl aminomethyl imidazoline	[[0,0,0,0,0,0,0,0,1,1,0],[0,0,0,0,0,0,1,0,0,0,1],[0,0,0,0,1,0,0,0,0,0,0],[0,0,0,0,0,0,0,0,0,0,1],[0,0,0,0,1,0,0,0,0,0,0]]C3256438	stearic monoethanolamide	[[0,0,1,0,0,0,0,0,0,0,0]]C3256439	stearoxytrimethylsilane and stearyl alcohol	[[0,0,1,0,0,0,0,0,0,0,0]]C0031445	phensedyl	[[0,1,0,0,0,0,0,0,0,0,0]]C0031444	phenprocoumon,phenprocoumalol,phenprocoumarol,phenylpropylhydroxycumarinum,4 hydroxy 3 1 phenylpropyl 2h 1 benzopyran 2 one	[[0,0,0,0,0,0,1,1,0,1,0],[0,0,0,0,0,0,0,0,0,0,1],[0,0,0,0,0,0,0,0,0,0,1],[0,0,0,0,0,0,0,0,0,0,1],[0,0,0,0,0,0,0,0,0,0,1]]C0031447	phentermine,alpha alpha dimethyl benzeneethanamine,phenyl tertiary butylamine,2 amino 2 methyl 1 phenylpropane,1 1 dimethyl 2 phenylethylamine,2 phenyl tert butylamine	[[0,0,0,0,0,0,0,1,1,1,0],[0,0,0,0,0,0,0,0,0,0,1],[1,0,0,0,0,0,0,0,0,0,0],[0,0,0,0,0,0,0,0,0,1,0],[0,0,0,0,0,0,0,0,0,1,0],[0,0,0,0,0,0,0,0,0,1,0]]C1330261	ex lax ultra	[[0,0,0,0,0,0,0,1,0,0,0]]C0031441	phenoxybenzamine,n 2 chloroethyl n 1 methyl 2 phenoxyethyl benzenemethanamine	[[0,0,0,0,0,0,0,1,1,1,0],[0,0,0,0,0,0,0,0,0,1,1]]C3256433	platycladus orientalis leaf	[[0,0,1,0,0,0,0,0,0,0,0]]C0534551	gadolinium dtpa bis glucosamide,gd dtpa biga	[[0,1,0,0,0,0,0,0,0,0,0],[0,1,0,0,0,0,0,0,0,0,0]]C3256431	piper methysticum whole	[[0,0,1,0,0,0,0,0,0,0,0]]C2827695	calcium 41 chloride aqueous solution,calcium 41 41ca chloride aqueous solution	[[0,0,0,0,0,0,0,0,0,1,0],[0,0,0,0,0,0,0,0,0,1,0]]C0127449	mel w	[[0,1,0,0,0,0,0,0,0,0,0]]C0039692	tetramisole,2 3 5 6 tetrahydro 6 phenyl imidazo 2 1 b thiazole,2 3 5 6 tetrahydro 6 phenylimidazo 2 1 b thiazole,dl tetramisole,nilverm base	[[0,0,0,0,0,0,0,0,0,1,1],[0,0,0,0,0,0,0,0,0,0,1],[0,0,0,0,0,0,0,0,0,1,0],[0,0,0,0,0,0,0,0,0,1,0],[0,0,0,0,0,0,0,0,0,1,0]]C2827696	calcium 46 chloride aqueous solution,calcium 46 46ca chloride aqueous solution	[[0,0,0,0,0,0,0,0,0,1,0],[0,0,0,0,0,0,0,0,0,1,0]]C2827691	pan pi3k mtor inhibitor sf1126	[[0,0,0,0,0,0,0,0,0,1,0]]C2827690	formoterol fumarate oral	[[0,0,0,0,0,0,0,0,0,1,0]]C1688559	topical selenium	[[1,0,0,0,0,0,0,0,0,0,0]]C2827692	autologous tarp peptide pulsed dendritic cell vaccine	[[0,0,0,0,0,0,0,0,0,1,0]]C2928952	calcium gluconate glucose	[[0,0,0,0,0,0,0,1,0,0,0]]C0062180	heliomycin,geliomitsin	[[0,1,0,0,0,0,0,0,0,1,0],[0,1,0,0,0,0,0,0,0,0,0]]C2827699	nanoplatin	[[0,0,0,0,0,0,0,0,0,1,0]]C2827698	micellar nanoparticle encapsulated cisplatin nc 6004	[[0,0,0,0,0,0,0,0,0,1,0]]C0964747	vanadicene dithiocarbamate,vddtc cpd	[[0,1,0,0,0,0,0,0,0,0,0],[0,1,0,0,0,0,0,0,0,0,0]]C0964746	vanadocene acetylacetonate monotriflate,dvacac cpd	[[0,1,0,0,0,0,0,0,0,0,0],[0,1,0,0,0,0,0,0,0,0,0]]C0764729	ag 34	[[0,1,0,0,0,0,0,0,0,0,0]]C0764728	3 methoxyl 5 2 2 dicyanoethenyl catechol	[[0,1,0,0,0,0,0,0,0,0,0]]C0964740	sma 52,sma 52 cpd	[[0,1,0,0,0,0,0,0,0,0,0],[0,1,0,0,0,0,0,0,0,0,0]]C0764722	1 dioxide 3 1 2 2 trimethylproyl amine 4h pyrido 2 3 e 1 2 4 thiadiazine	[[0,1,0,0,0,0,0,0,0,0,0]]C0627370	3 cfdunu	[[0,1,0,0,0,0,0,0,0,0,0]]C0627371	3 3 2 chloroethyl 3 nitrosoureido 2 3 dideoxy 5 fluorouridine	[[0,1,0,0,0,0,0,0,0,0,0]]C0764727	ag 372	[[0,1,0,0,0,0,0,0,0,0,0]]C0717976	phenazopyridine sulfisoxazole,phenazopyridine sulphisoxazole	[[1,0,0,0,0,0,0,1,0,0,0],[1,0,0,0,0,0,0,0,0,0,0]]C0627374	antibiotic b 21085,antibiotic b21085	[[0,1,0,0,0,0,0,0,0,0,0],[0,1,0,0,0,0,0,0,0,0,0]]C0964748	3 2 3 carboxy 4 bromo 5 6 7 8 tetrahydronaphthyl ethyl 3 6 7 8 tetrahydroimidazo 4 5 d 1 3 diazepin 8 ol,3 carboxy betido	[[0,1,0,0,0,0,0,0,0,0,0],[0,1,0,0,0,0,0,0,0,0,0]]C1874451	basis	[[0,0,0,0,0,0,0,1,0,0,1]]C0717971	permethrin topical	[[0,0,0,0,0,0,1,0,0,0,0]]C2935895	rti 212,rti212	[[0,1,0,0,0,0,0,0,0,0,0],[0,1,0,0,0,0,0,0,0,0,0]]C2935894	n 1 4 3 hydroxyphenyl 3 4 dimethylpiperidin 1 yl methyl 2 methylpropyl 7 methoxy 1 2 3 4 tetrahydroisoquinoline 3 carboxamide	[[0,1,0,0,0,0,0,0,0,0,0]]C2935893	rti 230,rti230	[[0,1,0,0,0,0,0,0,0,0,0],[0,1,0,0,0,0,0,0,0,0,0]]C2935892	n 1 4 3 carbamoylphenyl 3 4 dimethylpiperidin 1 yl methyl 2 methylpropyl 7 hydroxy 1 2 3 4 tetrahydroisoquinoline 3 carboxamide	[[0,1,0,0,0,0,0,0,0,0,0]]C2935891	rti 194,rti194	[[0,1,0,0,0,0,0,0,0,0,0],[0,1,0,0,0,0,0,0,0,0,0]]C2935890	7 hydroxy n 1 4 3 hydroxyphenyl 3 4 dimethylpiperidin 1 yl methyl 2 methylbutyl 1 2 3 4 tetrahydroisoquinoline 3 carboxamide dihydrochloride	[[0,1,0,0,0,0,0,0,0,0,0]]C1975872	zinc 124 red blood cells	[[0,0,0,1,0,0,0,0,0,0,0]]C0382485	2 acetoxyamino 1 methyl 6 phenylimidazo 4 5 b pyridine,n acetoxy phip	[[0,1,0,0,0,0,0,0,0,0,0],[0,1,0,0,0,0,0,0,0,0,0]]C3178623	isoceteth 20	[[0,1,1,0,0,0,0,0,0,0,0]]C2716502	diethyl 4 4 methoxyphenyl 2 6 dimethyl 1 4 dihydropyridin 3 5 dicarboxylate,dmddd cpd	[[0,1,0,0,0,0,0,0,0,0,0],[0,1,0,0,0,0,0,0,0,0,0]]C1529940	panthenol braun,braun brand of dexpanthenol	[[0,1,0,0,0,0,0,0,0,0,0],[0,1,0,0,0,0,0,0,0,0,0]]C1874409	bacitracin hydrocortisone neomycin polymyxin b	[[0,0,0,0,0,0,0,1,0,0,1]]C0089720	1 palmitoyl 2 trans parinaroyl sn glycero 3 phosphocholine	[[0,1,0,0,0,0,0,0,0,0,0]]C2932320	2 carbamoyl 5 chloro 4 fluoro 1h indol 3 yl 3 e 2 cyano vinyl 5 methyl phenyl r phosphinic acid methyl ester	[[0,1,0,0,0,0,0,0,0,0,0]]C3180801	phorbasone a	[[0,1,0,0,0,0,0,0,0,0,0]]C0045779	2 n nitroso n methylamino propiophenone,nmap	[[0,1,0,0,0,0,0,0,0,0,0],[0,1,0,0,0,0,0,0,0,0,0]]C3180802	phorbasone b	[[0,1,0,0,0,0,0,0,0,0,0]]C0251897	atsizol,acizole,azizol	[[0,1,0,0,0,0,0,0,0,0,0],[0,1,0,0,0,0,0,0,0,0,0],[0,1,0,0,0,0,0,0,0,0,0]]C0045776	2 n methyl n 2 2 6 6 tetramethylpiperidinooxyl ethyl 4 hexyloxybenzoate,c6sl	[[0,1,0,0,0,0,0,0,0,0,0],[0,1,0,0,0,0,0,0,0,0,0]]C0653482	16 18 ethano 20 ethyl 6 oxoprostaglandin e1,16 18 eeo pge1,16 18 ethano 20 ethyl 6 oxo pge1	[[0,1,0,0,0,0,0,0,0,0,0],[0,1,0,0,0,0,0,0,0,0,0],[0,1,0,0,0,0,0,0,0,0,0]]C2920158	ceraxon	[[0,0,0,0,0,0,0,1,0,0,0]]C1657077	calcium gluconate monohydrate	[[0,0,1,0,0,0,0,0,0,0,0]]C1709543	carvisken	[[0,0,0,0,0,0,0,0,0,1,0]]C0652754	n 2 5 nitrofurfurylidene formohydrazide 4 3 5 dicyanophenyl 6 ethoxy 2 pyridylimide,nofdepi	[[0,1,0,0,0,0,0,0,0,0,0],[0,1,0,0,0,0,0,0,0,0,0]]C3194869	diabetic tussin cough	[[0,0,0,0,0,0,0,1,0,0,0]]C0652751	zinc tetraanthraporphyrazine,zn tap	[[0,1,0,0,0,0,0,0,0,0,0],[0,1,0,0,0,0,0,0,0,0,0]]C0652752	tetraanthraporphyrazine	[[0,1,0,0,0,0,0,0,0,0,0]]C0072863	quindoxin,1 4 dihydroxyquinoxaline,1 4 dioxide quinoxaline,quinoxaline di n oxide,quinoxaline 1 4 di n oxide,quinoxaline dioxide	[[0,1,0,0,0,0,0,0,0,1,0],[0,1,0,0,0,0,0,0,0,0,0],[0,1,0,0,0,0,0,0,0,0,0],[0,1,0,0,0,0,0,0,0,0,0],[0,0,0,0,0,0,0,0,0,1,0],[0,0,0,0,0,0,0,0,0,1,0]]C0047732	32 33 34 35 bacteriohopanetetrol,32 33 34 35 bhtt,bacteriohopanetetrol,tetrahydroxybacteriohopane,bacteriohopane 32 33 34 35 tetrol,bacteriohopane tetrol	[[0,1,0,0,0,0,0,0,0,0,0],[0,1,0,0,0,0,0,0,0,0,0],[0,1,0,0,0,0,0,0,0,0,0],[0,1,0,0,0,0,0,0,0,0,0],[0,1,0,0,0,0,0,0,0,0,0],[0,1,0,0,0,0,0,0,0,0,0]]C0535203	rat rss protein,rss protein,rat stomach serotonin receptor related protein	[[0,1,0,0,0,0,0,0,0,0,0],[0,1,0,0,0,0,0,0,0,0,0],[0,1,0,0,0,0,0,0,0,0,0]]C0599608	catharanthus alkaloid	[[0,0,0,0,0,0,0,0,1,0,0]]C0599609	periwinkle alkaloid	[[0,0,0,0,0,0,0,0,1,0,0]]C0030316	panhematin	[[0,0,0,0,0,0,1,1,0,0,0]]C0525779	rx 783006	[[0,1,0,0,0,0,0,0,0,0,0]]C0030314	pangamic acid,vitamin b15	[[1,0,0,0,0,0,0,1,0,0,0],[0,1,0,0,0,0,0,0,0,0,0]]C0030310	pancuronium,1 1 2beta 3alpha 5alpha 16beta 17beta 3 17 bis acetyloxy androstane 2 16 diyl bis 1 methyl piperidinium	[[0,0,0,0,0,0,1,1,0,1,0],[0,0,0,0,0,0,0,0,0,0,1]]C0030311	pancuronium bromide,dibromide 1 1 2beta 3alpha 5alpha 16beta 17beta 3 17 bis acetyloxy androstane 2 16 diyl bis 1 methyl piperidinium,2beta 16beta dipiperidino 5alpha androstane 3alpha 17beta diol diacetate dimethobromide	[[1,1,0,0,0,0,0,0,0,1,0],[0,0,0,0,0,0,0,0,0,1,0],[0,0,0,0,0,0,0,0,0,1,0]]C2704363	meta methylhippurate para methylhippurate 124 bld ser plas	[[0,0,0,1,0,0,0,0,0,0,0]]C0178741	mercapturate	[[0,0,0,0,0,0,0,0,1,0,0]]C0072864	quinestradol,estriol 3 cyclopentyl ether	[[0,0,0,0,0,0,0,1,0,1,0],[0,1,0,0,0,0,0,0,0,0,0]]C0963211	callipeltin a	[[0,1,0,0,0,0,0,0,0,0,0]]C0254895	17 beta o butyryl vecuronium bromide	[[0,1,0,0,0,0,0,0,0,0,0]]C0072865	quinfamide,1 dichloroacetyl 1 2 3 4 tetrahydro 6 quinolinyl ester 2 furancarboxylic acid	[[0,0,0,0,0,0,0,1,0,1,0],[0,1,0,0,0,0,0,0,0,0,0]]C2928953	cholecalciferol chromium picolinate	[[0,0,0,0,0,0,0,1,0,0,0]]C0536955	arachidonyl diazomethyl ketone,admk cpd	[[0,1,0,0,0,0,0,0,0,0,0],[0,1,0,0,0,0,0,0,0,0,0]]C0075259	stilbostat	[[0,1,0,0,0,0,0,0,0,0,0]]C1530051	dostein,glaxo wellcome brand of erdosteine	[[0,1,0,0,0,0,0,0,0,0,0],[0,1,0,0,0,0,0,0,0,0,0]]C1530050	eprazinone hydrochloride,eprazinone dihydrochloride	[[0,1,0,0,0,0,0,0,0,0,0],[0,1,0,0,0,0,0,0,0,0,0]]C1530057	ethquinol,ulmer brand of ethaverine	[[0,1,0,0,0,0,0,0,0,0,0],[0,1,0,0,0,0,0,0,0,0,0]]C1530056	genoral,knoll brand of estropipate	[[0,1,0,0,0,0,0,0,0,0,0],[0,1,0,0,0,0,0,0,0,0,0]]C1530055	invanoz,msd brand of ertapenem	[[0,1,0,0,0,0,0,0,0,0,0],[0,1,0,0,0,0,0,0,0,0,0]]C1530054	vectrine,pharma 2000 brand of erdosteine	[[0,1,0,0,0,0,0,0,0,0,0],[0,1,0,0,0,0,0,0,0,0,0]]C0075252	stigmastanol,3 beta 5 alpha stigmastan 3 ol,dihydrositosterol,sitostanol	[[0,1,0,0,0,0,0,0,0,1,0],[0,1,0,0,0,0,0,0,0,0,0],[0,1,0,0,0,0,0,0,0,0,0],[0,1,0,0,0,0,0,0,0,0,0]]C0075254	stigmatellin,2 4 6 dimethoxy 3 5 11 trimethyl 7 9 11 tridecatrienyl 8 hydroxy 5 7 dimethoxy 3 methyl 4h 1 benzopyran 4 one	[[0,1,0,0,0,0,0,0,0,0,0],[0,1,0,0,0,0,0,0,0,0,0]]C0045500	2 4 dihydroxypyridine n oxide	[[0,1,0,0,0,0,0,0,0,0,0]]C0075256	stilbene oxide,2 3 diphenyl oxirane	[[0,1,0,0,0,0,0,0,0,0,0],[0,1,0,0,0,0,0,0,0,0,0]]C2704366	methotrexate 124 dose	[[0,0,0,1,0,0,0,0,0,0,0]]C1171788	sm 232724,sm232724	[[0,1,0,0,0,0,0,0,0,0,0],[0,1,0,0,0,0,0,0,0,0,0]]C1098775	s24429,s 24429	[[0,1,0,0,0,0,0,0,0,0,0],[0,1,0,0,0,0,0,0,0,0,0]]C0072869	quinidine gluconate,mono d gluconate 9s 6 methoxy cinchonan 9 ol	[[0,0,1,0,0,0,0,1,0,1,0],[0,0,0,0,0,0,0,0,0,1,0]]C1098773	3 cyclopentyl 8 benzylamino 3 4 dihydro 2h 1 4 benzoxazin 5 yl phenylmethanone,s 24718,s24718	[[0,1,0,0,0,0,0,0,0,0,0],[0,1,0,0,0,0,0,0,0,0,0],[0,1,0,0,0,0,0,0,0,0,0]]C1618777	combunox	[[0,1,0,0,0,0,0,1,0,0,0]]C0000543	5 6 dihydroxytryptamine,3 2 aminoethyl indole 5 6 diol,3 2 aminoethyl 1h indole 5 6 diol	[[0,1,0,0,0,0,0,0,0,0,1],[0,0,0,0,0,0,0,0,0,0,1],[0,0,0,0,0,0,0,0,0,0,1]]C0000544	5 7 dihydroxytryptamine,3 2 aminoethyl 1h indole 5 7 diol	[[0,1,0,0,0,0,0,0,0,0,1],[0,0,0,0,0,0,0,0,0,0,1]]C0000545	eicosapentaenoic acid,5 8 11 14 17 eicosapentaenoic acid,omega 3 eicosapentaenoic acid,timnodonic acid,5 8 11 14 17 icosapentaenoic acid,eicosapentanoic acid,epa eicosapentanoic acid,epa,ipa,icosapentaenoic acid,all cis 5 8 11 14 17 eicosapentaenoic acid,icosapentaenoic acid n 3,eicosapentaenoic acid n 3,all cis icosa 5 8 11 14 17 pentaenoic acid,fatty acid 20 5,fatty acid 20 5 n 3,icosapent	[[1,0,0,0,0,0,1,0,0,1,1],[0,0,0,0,0,0,0,0,0,0,1],[0,1,0,0,0,0,0,0,0,0,1],[0,0,0,0,0,0,1,0,0,1,0],[0,0,0,0,0,0,0,0,0,0,1],[1,1,0,0,0,0,0,0,0,0,1],[1,0,0,0,0,0,0,0,0,0,0],[0,0,0,0,0,0,0,0,0,1,0],[0,0,0,0,0,0,0,0,0,1,0],[0,0,0,0,0,0,0,0,0,1,0],[0,0,0,0,0,0,0,0,0,1,0],[0,0,0,0,0,0,0,0,0,1,0],[0,0,0,0,0,0,0,0,0,1,0],[0,0,0,0,0,0,0,0,0,1,0],[0,0,0,0,0,0,0,0,0,1,0],[0,0,0,0,0,0,0,0,0,1,0],[0,0,0,0,0,0,0,0,0,1,0]]C0000546	5 8 11 14 eicosatetraynoic acid,etya	[[0,0,0,0,0,0,0,0,0,0,1],[0,0,0,0,0,0,0,0,0,0,1]]C2949015	sinex	[[0,0,0,0,0,0,0,1,0,0,0]]C0962632	1 1 2 diphenyl ethyl 4 2 3 4 dimethoxyphenyl ethyl piperazine dihydrochloride	[[0,1,0,0,0,0,0,0,0,0,0]]C2933936	pungen c,4 o 3 methoxy 4 hydroxy benzoic acid 3 o acrolactic acid 5 methoxy gallic acid	[[0,1,0,0,0,0,0,0,0,0,0],[0,1,0,0,0,0,0,0,0,0,0]]C1975877	zinc 124 urine	[[0,0,0,1,0,0,0,0,0,0,0]]C2699205	ciapilome	[[0,0,0,0,0,0,0,0,0,1,0]]C1578092	human normal immunoglobulin,hnig normal immunoglobulin	[[0,0,0,0,0,0,0,1,0,0,0],[1,0,0,0,0,0,0,0,0,0,0]]C1882164	oral sodium phenylbutyrate	[[0,0,0,0,0,0,0,0,0,1,0]]C3198869	aluminum chlorohydrex propylene glycol	[[0,0,1,0,0,0,0,0,0,0,0]]C0718827	baycol,lipobay,bayer brand of cerivastatin sodium	[[0,0,0,0,0,0,1,0,0,1,0],[0,1,0,0,0,0,1,0,0,0,0],[0,1,0,0,0,0,0,0,0,0,0]]C0718826	bayrho d	[[0,0,0,0,0,0,1,1,0,0,0]]C1259868	jpm8 compound	[[0,1,0,0,0,0,0,0,0,0,0]]C0718823	barophen	[[0,0,0,0,0,0,1,1,0,0,0]]C0718822	baridium,pfeiffer brand of phenazopyridine hydrochloride	[[0,0,0,0,0,0,0,1,0,0,0],[0,1,0,0,0,0,0,0,0,0,0]]C0718821	barbidonna	[[0,0,0,0,0,0,1,1,0,0,0]]C0718820	bar test	[[0,0,0,0,0,0,0,1,0,0,0]]C2987658	orantinib,3 2 4 dimethyl 5 2 oxo 1 2 dihydro 3h indol 3 ylidene methyl 1h pyrrol 3 yl propionic acid,orantinibum	[[0,0,0,0,0,0,0,0,0,1,0],[0,0,0,0,0,0,0,0,0,1,0],[0,0,0,0,0,0,0,0,0,1,0]]C0718829	bayer aspirin pm extra strength	[[0,0,0,0,0,0,0,1,0,0,0]]C0718828	bayer aspirin	[[0,0,0,0,0,0,1,1,0,0,0]]C1328649	carmustine copolymer	[[0,0,0,0,0,0,0,0,0,1,0]]C0250387	n aphea msuc	[[0,1,0,0,0,0,0,0,0,0,0]]C0250386	7 4 carboxybutanamido cephalosporin mustard,7 cbnceph mustard	[[0,1,0,0,0,0,0,0,0,0,0],[0,1,0,0,0,0,0,0,0,0,0]]C0047199	3 adenin 9 yl 2 hydroxypropanoic acid isobutyl ester,3 adenin 9 yl 2 hydroxypropanoic acid 2 methylpropyl ester,ahpa isobutyl ester	[[0,1,0,0,0,0,0,0,0,0,0],[0,1,0,0,0,0,0,0,0,0,0],[0,1,0,0,0,0,0,0,0,0,0]]C0298815	leo viscous eye gel	[[0,1,0,0,0,0,0,0,0,0,0]]C0047195	3 acetylpyridine	[[0,1,0,0,0,0,0,0,0,0,0]]C0250388	n n 9 fluorenylmethoxy carbonyl 3 aminoprop 1 yl n o 4 4 dimethoxytrityl 2 oxyethyl n o 3 carboxylpropionyl 2 oxyethyl amine	[[0,1,0,0,0,0,0,0,0,0,0]]C0935761	yttrium y 90 monoclonal antibody b3,y 90 monoclonal antibody b3,yttrium y 90 moab b3,90 y b3,y90 b3	[[0,0,0,0,1,0,0,0,0,1,0],[0,0,0,0,1,0,0,0,0,1,0],[0,0,0,0,1,0,0,0,0,1,0],[0,0,0,0,0,0,0,0,0,1,0],[0,0,0,0,1,0,0,0,0,0,0]]C0767987	glucolipsin a	[[0,1,0,0,0,0,0,0,0,0,0]]C1815748	eupatorium extract,eupatorium	[[0,0,0,0,0,1,0,0,0,0,0],[0,0,0,0,0,0,0,0,0,0,1]]C2987655	amuvatinib,receptor tyrosine kinase inhibitor mp470	[[0,0,0,0,0,0,0,0,0,1,0],[0,0,0,0,0,0,0,0,0,1,0]]C0763841	biotrol	[[0,1,0,0,0,0,0,0,0,0,0]]C1815742	dillweed	[[0,0,0,0,0,0,0,0,0,0,1]]C0142900	sodium oxyferriscorbone	[[0,1,0,0,0,0,0,0,0,0,0]]C1874118	maple mix tree allergenic extract	[[0,0,0,0,0,0,0,0,0,0,1]]C2987657	pegylated liposomal belotecan,stealth liposomal belotecan	[[0,0,0,0,0,0,0,0,0,1,0],[0,0,0,0,0,0,0,0,0,1,0]]C0673940	mangan desferrioxamin,mangan desferal chelate,mn dfx	[[0,1,0,0,0,0,0,0,0,0,0],[0,1,0,0,0,0,0,0,0,0,0],[0,1,0,0,0,0,0,0,0,0,0]]C0208821	bmy 20064	[[0,1,0,0,0,0,0,0,0,0,0]]C2603422	ag 014699,ag014699	[[0,1,0,0,0,0,0,0,0,0,0],[0,0,0,0,0,0,0,0,0,1,0]]C1677795	6 nitro 3 oxoquinoxaline 2 carboxylic acid,6 n 3 oq ca	[[0,1,0,0,0,0,0,0,0,0,0],[0,1,0,0,0,0,0,0,0,0,0]]C1434631	quercetin 3 o beta d glucopyranoside	[[0,1,0,0,0,0,0,0,0,0,0]]C0607787	3 aminodesoxyequilenin,3 amino estra 1 3 5 7 9 pentaen 17 one,d 3 aminodesoxyequilenin	[[0,1,0,0,0,0,0,0,0,0,0],[0,1,0,0,0,0,0,0,0,0,0],[0,1,0,0,0,0,0,0,0,0,0]]C2928973	dextromethorphan guaiacolsulfonic acid promethazine	[[0,0,0,0,0,0,0,1,0,0,0]]C1433579	rhazinal	[[0,1,0,0,0,0,0,0,0,0,0]]C2830197	aeb071	[[0,1,0,0,0,0,0,0,0,0,0]]C0672092	methyl lexitropsin,me lex	[[0,1,0,0,0,0,0,0,0,0,0],[0,1,0,0,0,0,0,0,0,0,0]]C1874117	hickory tree allergenic extract	[[0,0,0,0,0,0,0,0,0,0,1]]C1433570	nepetoidin b	[[0,1,0,0,0,0,0,0,0,0,0]]C1433571	nepetoidin a	[[0,1,0,0,0,0,0,0,0,0,0]]C0304408	reversible anticholinesterase	[[1,0,0,0,0,0,0,0,0,0,0]]C0765442	kar 4,3 allyl 2 4 dioxo 3 5 spiro oxazolidino 4 deacetoxy vinblastine	[[0,1,0,0,0,0,0,0,0,0,0],[0,1,0,0,0,0,0,0,0,0,0]]C1743918	calceolarioside	[[0,1,0,0,0,0,0,0,0,0,0]]C2699473	cromoglicate lisetil	[[0,0,0,0,0,0,0,0,0,1,0]]C0671542	nodulisporic acid a	[[0,1,0,0,0,0,0,0,0,0,0]]C0391196	18alpha 19betah urs 20 ene 3beta 16beta diol	[[0,1,0,0,0,0,0,0,0,0,0]]C0391197	faradiol	[[0,1,0,0,0,0,0,0,0,0,0]]C2938531	marcaine with epinephrine 0 75 1 200000	[[0,0,0,0,0,0,0,1,0,0,0]]C0671544	ph 1126,ph 1126 pheophorbide	[[0,1,0,0,0,0,0,0,0,0,0],[0,1,0,0,0,0,0,0,0,0,0]]C2698934	anamorelin	[[0,0,0,0,0,0,0,0,0,1,0]]C0765852	acetylamino propylidene diphosphonic acid,apda	[[0,1,0,0,0,0,0,0,0,0,0],[0,1,0,0,0,0,0,0,0,0,0]]C1442748	flower essence sinapis arvensis	[[0,0,0,0,0,1,0,0,0,0,0]]C0103072	ammonium fumarate	[[0,1,0,0,0,0,0,0,0,0,0]]C0767268	1 3 chloro 4 fluorophenyl carbonyl 4 fluoro 4 6 dimethylamino pyridin 2 yl methyl amino methyl piperidine,1 clfphco 4 fdpmamp	[[0,1,0,0,0,0,0,0,0,0,0],[0,1,0,0,0,0,0,0,0,0,0]]C0103076	ammonium lauryl sulfate,ammonium dodecyl sulfate,ammonium salt dodecyl sulfate	[[0,1,1,0,0,0,0,0,0,1,0],[0,1,0,0,0,0,0,0,0,0,0],[0,1,0,0,0,0,0,0,0,0,0]]C0027409	narcotic analgesics,narcotic agonists,narcotic analgesic agonists,narcotic analgesic product	[[0,1,0,0,0,0,1,0,0,0,0],[0,0,0,0,0,0,1,0,0,0,0],[0,0,0,0,0,0,1,0,0,0,0],[1,0,0,0,0,0,0,0,0,0,0]]C1579450	doxepin rph,rodleben brand of doxepin hydrochloride	[[0,1,0,0,0,0,0,0,0,0,0],[0,1,0,0,0,0,0,0,0,0,0]]C0699505	butadione	[[1,1,0,0,0,0,0,0,0,0,0]]C0699506	butapyrazole,butapirazol	[[0,1,0,0,0,0,0,0,0,0,0],[0,1,0,0,0,0,0,0,0,0,0]]C0699507	fenilbutazon	[[0,1,0,0,0,0,0,0,0,0,0]]C1579454	doxepin holsten,holsten brand of doxepin hydrochloride	[[0,1,0,0,0,0,0,0,0,0,0],[0,1,0,0,0,0,0,0,0,0,0]]C1579455	doxepin lindo,lindopharm brand of doxepin hydrochloride	[[0,1,0,0,0,0,0,0,0,0,0],[0,1,0,0,0,0,0,0,0,0,0]]C1579456	quitaxon,nepalm brand of doxepin hydrochloride	[[0,1,0,0,0,0,0,0,0,0,0],[0,1,0,0,0,0,0,0,0,0,0]]C1579457	mareen,krewel brand of doxepin hydrochloride	[[0,1,0,0,0,0,0,0,0,0,0],[0,1,0,0,0,0,0,0,0,0,0]]C1579458	espadox,esparma brand of doxepin hydrochloride	[[0,1,0,0,0,0,0,0,0,0,0],[0,1,0,0,0,0,0,0,0,0,0]]C0949215	rmi 71782	[[0,1,0,0,0,0,0,0,0,0,0]]C0949217	anhydrous amiloride hydrochloride	[[0,1,1,0,0,0,0,0,0,0,0]]C0141712	sch 33844	[[0,1,0,0,0,0,0,0,0,0,0]]C0258570	mer w8020	[[0,1,0,0,0,0,0,0,0,0,0]]C0972378	cp 424391	[[0,1,0,0,0,0,0,0,0,0,0]]C0651321	ethyl 5 cyano 1 6 dihydro 2 methyl 6 oxo 3 pyridinecarboxylate,ethyl cdmop	[[0,1,0,0,0,0,0,0,0,0,0],[0,1,0,0,0,0,0,0,0,0,0]]C0972371	drf 2725,drf2725	[[0,1,0,0,0,0,0,0,0,0,0],[0,1,0,0,0,0,0,0,0,0,0]]C1435587	ggti 2154	[[0,1,0,0,0,0,0,0,0,0,0]]C2586556	pediacare childrens allergy	[[0,0,0,0,0,0,0,1,0,0,0]]C0022154	isocarboxazid,2 phenylmethyl hydrazide 5 methyl 3 isoxazolecarboxylic acid,maoi isocarboxazid	[[0,0,1,0,0,0,0,1,0,1,0],[0,0,0,0,0,0,0,0,0,1,1],[1,0,0,0,0,0,0,0,0,0,0]]C2586553	medent pei	[[0,0,0,0,0,0,0,1,0,0,0]]C1975879	zinc 124 white blood cells	[[0,0,0,1,0,0,0,0,0,0,0]]C1981335	amphetamine 124 urine	[[0,0,0,1,0,0,0,0,0,0,0]]C1981332	amphetamine 124 bld ser plas	[[0,0,0,1,0,0,0,0,0,0,0]]C1981333	amphetamine 124 meconium	[[0,0,0,1,0,0,0,0,0,0,0]]C3170095	paper wasp polistes spp recombinant rpol d 5 124 bld ser plas	[[0,0,0,1,0,0,0,0,0,0,0]]C0304407	nicotine resin complex	[[1,0,0,0,0,0,0,0,0,0,0]]C3170091	pain relieving medication 124 patient	[[0,0,0,1,0,0,0,0,0,0,0]]C2348772	thioredoxin 1 inhibitor px 12,1 methylpropyl 2 imidazolyl disulfide	[[0,0,0,0,0,0,0,0,0,1,0],[0,0,0,0,0,0,0,0,0,1,0]]C1506079	allonorsecurinine	[[0,1,0,0,0,0,0,0,0,0,0]]C2934304	sterodin	[[0,1,0,0,0,0,0,0,0,0,0]]C2713560	semagacestat	[[0,0,0,0,0,0,0,0,0,1,0]]C3247280	neutramaxx	[[0,0,0,0,0,0,0,1,0,0,0]]C0643848	caribbazoin b,ethyl 2 1 1 3 dihydro 1 3 dioxo 2h inden 2 ylidene methyl 1 methylhydrazine,ethyl ester 2 1 3 dihydro 1 3 dioxo 2h inden 2 ylidene methyl 1 methyl hydrazinecarboxylic acid	[[0,1,0,0,0,0,0,0,0,0,0],[0,1,0,0,0,0,0,0,0,0,0],[0,1,0,0,0,0,0,0,0,0,0]]C0643842	ro 19 5248a	[[0,1,0,0,0,0,0,0,0,0,0]]C0643843	cefazolin delta 2 methyl ester	[[0,1,0,0,0,0,0,0,0,0,0]]C0643840	cefazolin delta 3 methyl ester	[[0,1,0,0,0,0,0,0,0,0,0]]C1992142	mannitol 124 urine serum or plasma	[[0,0,0,1,0,0,0,0,0,0,0]]C0643846	caribbazoin a	[[0,1,0,0,0,0,0,0,0,0,0]]C0643845	ethyl 1 acetyl 2 1 1 3 dihydro 1 3 dioxo 2h inden 2 ylidene ethyl hydrazinecarboxylate	[[0,1,0,0,0,0,0,0,0,0,0]]C1166055	ignatia	[[0,0,0,0,0,0,0,1,0,0,0]]C2928974	careless weed pollen extract redroot pigweed pollen extract	[[0,0,0,0,0,0,0,1,0,0,0]]C0392429	polyvalent reptile antivenin	[[1,0,0,0,0,0,0,0,0,0,0]]C0392428	chromic phosphate p32,chromic phosphate p 32,chromic phosphate p sup 32 sup	[[1,0,0,0,0,0,1,1,0,0,0],[1,0,0,0,0,0,1,0,0,1,0],[1,0,0,0,0,0,0,0,0,0,0]]C0953241	2r 2alpha 4aalpha 8abeta isomer 4 4 10 trimethyl trans decal 3 ol	[[0,1,0,0,0,0,0,0,0,0,0]]C0392424	pronestyl,apothecon brand of procainamide hydrochloride,bristol myers squibb brand of procainamide hydrochloride	[[0,0,0,0,0,0,1,1,0,0,0],[0,1,0,0,0,0,0,0,0,0,0],[0,1,0,0,0,0,0,0,0,0,0]]C0392426	dimethoxanate	[[0,0,0,0,0,0,0,1,0,1,0]]C0392421	bryonin	[[1,0,0,0,0,0,0,0,0,0,0]]C2699886	talotrexin ammonium,2 4s 4 carboxy 4 4 2 4 diaminopteridin 6 yl methyl amino benzoyl amino butyl carbamoyl benzoic acid monoammonium salt	[[0,0,0,0,0,0,0,0,0,1,0],[0,0,0,0,0,0,0,0,0,1,0]]C0102309	alpha bitter acid	[[0,1,0,0,0,0,0,0,0,0,0]]C0077493	tuinal	[[0,0,0,0,0,0,1,1,0,0,0]]C2745653	swerilactone c	[[0,1,0,0,0,0,0,0,0,0,0]]C0077496	tullidora toxin	[[0,1,0,0,0,0,0,0,0,0,0]]C0077497	tulobuterol,1 o chlorophenyl 2 tert butylaminoethanol,2 chloro alpha 1 1 dimethylethyl amino methyl benzenemethanol,alpha tert butylamino methyl o chlorobenzyl alcohol	[[1,0,0,0,0,0,0,1,0,0,0],[0,1,0,0,0,0,0,0,0,0,0],[0,1,0,0,0,0,0,0,0,0,0],[0,1,0,0,0,0,0,0,0,0,0]]C0971886	uropolinum polfa	[[0,1,0,0,0,0,0,0,0,0,0]]C0971884	fisioquens	[[0,1,0,0,0,0,0,0,0,0,0]]C0971883	3 diethylamino 2 2 dimethylpropyl 5 p nitrophenyl 2 furoate hydrochloride	[[0,1,0,0,0,0,0,0,0,0,0]]C0111637	cyflee	[[0,1,0,0,0,0,0,0,0,0,0]]C1099021	hmr 3787	[[0,1,0,0,0,0,0,0,0,0,0]]C0111635	cyclotropium bromide,ciclotropium bromide	[[0,1,0,0,0,0,0,0,0,0,0],[0,1,0,0,0,0,0,0,0,1,0]]C0982017	amphetamine adipate	[[0,0,0,0,0,0,0,0,0,1,0]]C0982016	amodiaquine hydrochloride	[[1,1,0,0,0,0,0,0,0,1,0]]C0982014	ammonium phosphate monobasic	[[0,0,0,0,0,0,0,0,0,0,1]]C1099029	vanadocene iv acetylacetonate complex,v iv acc	[[0,1,0,0,0,0,0,0,0,0,0],[0,1,0,0,0,0,0,0,0,0,0]]C0982018	amphetamine dextroamphetamine resin complex	[[0,0,0,0,0,0,0,0,0,0,1]]C2975098	hanabiratakelide b	[[0,1,0,0,0,0,0,0,0,0,0]]C0100790	a21a	[[0,1,0,0,0,0,0,0,0,0,0]]C2936593	5 lipoxygenase activating protein inhibitors,flap inhibitors	[[0,1,0,0,0,0,0,0,0,0,0],[0,1,0,0,0,0,0,0,0,0,0]]C0887802	demeclocycline monohydrochloride	[[0,1,0,0,0,0,0,0,0,0,0]]C1974255	testosterone 124 saliva	[[0,0,0,1,0,0,0,0,0,0,0]]C2974606	pf 04928473,pf04928473	[[0,1,0,0,0,0,0,0,0,0,0],[0,1,0,0,0,0,0,0,0,0,0]]C2974601	zinc sulfate drug combination vitamin a boric acid,boric acid vitamin a zinc sulfate	[[0,1,0,0,0,0,0,0,0,0,0],[0,1,0,0,0,0,0,0,0,0,0]]C2974600	parabens drug combination methylene blue isopropyl alcohol citrate,citrate isopropyl alcohol methylene blue parabens	[[0,1,0,0,0,0,0,0,0,0,0],[0,1,0,0,0,0,0,0,0,0,0]]C2974603	mesalamine drug combination inulin butyrate,butyrate inulin mesalamine	[[0,1,0,0,0,0,0,0,0,0,0],[0,1,0,0,0,0,0,0,0,0,0]]C2974602	zinc drug combination l methionine arginine,arginine l methionine zinc	[[0,1,0,0,0,0,0,0,0,0,0],[0,1,0,0,0,0,0,0,0,0,0]]C0067451	n 2 hydroxyethoxy methyl 5 methyluracil,acyclothymidine	[[0,1,0,0,0,0,0,0,0,0,0],[0,1,0,0,0,0,0,0,0,0,0]]C0067452	n 2 methylamino ethyl 5 isoquinolinesulfonamide,h 8 protein kinase inhibitor,h8 protein kinase inhibitor	[[0,1,0,0,0,0,0,0,0,0,0],[0,1,0,0,0,0,0,0,0,0,0],[0,1,0,0,0,0,0,0,0,0,0]]C0067454	n 2 acetamido iminodiacetic acid,n 2 amino 2 oxoethyl n carboxymethyl glycine	[[0,1,0,0,0,0,0,0,0,0,0],[0,1,0,0,0,0,0,0,0,0,0]]C0067456	n 2 aminoethyl 5 isoquinolinesulfonamide,aeiqs,protein kinase c inhibitor h 9,h 9	[[0,1,0,0,0,0,0,0,0,0,0],[0,1,0,0,0,0,0,0,0,0,0],[0,1,0,0,0,0,0,0,0,0,0],[0,1,0,0,0,0,0,0,0,0,0]]C0771664	myrtillus	[[0,0,0,0,0,0,1,0,0,0,0]]C1634054	nalex a 12	[[0,0,0,0,0,0,0,1,0,0,0]]C0771667	minaprine hcl	[[0,0,0,0,0,0,0,1,0,0,0]]C0771660	miristalkonium	[[0,0,0,0,0,0,0,1,0,0,0]]C0354772	xanthine bronchodilators	[[1,0,0,0,0,0,0,0,0,0,0]]C0771662	methyl diacetylcysteinate	[[0,0,0,0,0,0,0,1,0,0,0]]C0771663	naringin sodium	[[0,1,0,0,0,0,0,0,0,0,0]]C0023401	leucine,l leucine,leu,l isomer leucine,s 2 amino 4 methylpentanoic acid	[[0,0,0,0,0,0,1,0,0,1,0],[0,1,0,0,0,0,1,0,0,0,0],[0,0,0,0,0,0,1,0,0,0,0],[0,1,0,0,0,0,0,0,0,0,1],[0,0,0,0,0,0,0,0,0,1,0]]C0252523	isopropyl glycidyl ether,1 methylethoxy methyl oxirane,isopropoxymethyl oxirane	[[1,1,0,0,0,0,0,0,0,0,0],[0,1,0,0,0,0,0,0,0,0,0],[0,1,0,0,0,0,0,0,0,0,0]]C1675314	squamostolide	[[0,1,0,0,0,0,0,0,0,0,0]]C1675315	wh30 herbal preparation	[[0,1,0,0,0,0,0,0,0,0,0]]C2348709	fospropofol disodium,disodium salt dihydrogen phosphate 2 6 bis 1 methylethyl phenoxy methanol	[[1,0,0,0,0,0,0,1,0,1,0],[0,0,0,0,0,0,0,0,0,1,0]]C0082078	compound 1929	[[0,1,0,0,0,0,0,0,0,0,0]]C0386438	2 hydroxy org 7797	[[0,1,0,0,0,0,0,0,0,0,0]]C2747248	d worm combo	[[0,0,0,0,0,0,0,1,0,0,0]]C0764180	2 chloro 5 methoxy 4 5 2 piperidylmethyl 1 2 4 oxadiazol 3 yl aniline,cl mpmoa	[[0,1,0,0,0,0,0,0,0,0,0],[0,1,0,0,0,0,0,0,0,0,0]]C0131912	naphthomycin a	[[0,1,0,0,0,0,0,0,0,0,0]]C2929619	ammonium chloride ephedrine	[[0,0,0,0,0,0,0,1,0,0,0]]C0165943	1 3 4 dichlorophenyl acetyl 4 acetyl 2 1 pyrrolidinylmethyl piperazine	[[0,1,0,0,0,0,0,0,0,0,0]]C1445829	brompheniramine maleate phenylpropanolamine	[[1,0,0,0,0,0,0,0,0,0,0]]C1445828	benzocaine triclosan	[[1,0,0,0,0,0,0,1,0,0,0]]C0165944	gr 85571,gr85571	[[0,1,0,0,0,0,0,0,0,0,0],[0,1,0,0,0,0,0,0,0,0,0]]C1445824	azo sulfamethoxazole,azo sulphamethoxazole	[[1,0,0,0,0,0,0,0,0,0,0],[1,0,0,0,0,0,0,0,0,0,0]]C1445827	benzocaine phenol	[[1,0,0,0,0,0,0,1,0,0,1]]C1445826	bee pollen preparation	[[1,0,0,0,0,0,0,0,0,0,0]]C1445821	aluminum hydroxide magnesium trisilicate alginic acid sodium bicarbonate,aluminium hydroxide magnesium trisilicate alginic acid sodium bicarbonate	[[1,0,0,0,0,0,0,0,0,0,0],[1,0,0,0,0,0,0,0,0,0,0]]C1722623	zr1121,zr 1121	[[0,1,0,0,0,0,0,0,0,0,0],[0,1,0,0,0,0,0,0,0,0,0]]C1445822	aluminum hydroxide magnesium hydroxide simethicone,aluminium hydroxide magnesium hydroxide simethicone,simethicone drug combination magnesium hydroxide aluminum hydroxide	[[1,1,0,0,0,0,0,1,0,0,1],[1,0,0,0,0,0,0,0,0,0,0],[0,1,0,0,0,0,0,0,0,0,0]]C0701852	cefuroxime sodium,monosodium salt 6r 6alpha 7beta z 3 aminocarbonyl oxy methyl 7 2 furanyl methoxyimino acetyl amino 8 oxo 5 thia 1 azabicyclo 4 2 0 oct 2 ene 2 carboxylic acid,cefurox,lifurox	[[0,0,0,0,0,0,1,1,0,1,0],[0,0,0,0,0,0,0,0,0,1,0],[0,0,0,0,0,0,0,0,0,1,0],[0,0,0,0,0,0,0,0,0,1,0]]C0701853	ketocef	[[0,1,0,0,0,0,0,0,0,0,0]]C0701855	kefurox	[[0,0,0,0,0,0,0,0,0,1,0]]C0701858	antiperspirants	[[0,1,0,0,0,0,1,0,0,0,0]]C0122855	hydroxypolyethoxydodecane	[[0,1,0,0,0,0,0,0,0,0,0]]C1722624	1 hydroxy 2 deacetoxy 5 decinnamoyl taxinine j,1 hd dct j	[[0,1,0,0,0,0,0,0,0,0,0],[0,1,0,0,0,0,0,0,0,0,0]]C3181734	16 4 3 imidazol 1 yl propoxy 3 methoxybenzylidene 5 androstene 3b 17b diol	[[0,1,0,0,0,0,0,0,0,0,0]]C0611434	cis malonato diammino platinum	[[0,1,0,0,0,0,0,0,0,0,0]]C2928328	isomyrtol pholcodine	[[0,0,0,0,0,0,0,1,0,0,0]]C3181737	dpj rg 1177	[[0,1,0,0,0,0,0,0,0,0,0]]C3181731	1 4 carbamoylpyridinium 3 4 hydroxyiminomethylpyridinium 2 oxapropane	[[0,1,0,0,0,0,0,0,0,0,0]]C3181732	kr 22934,kr22934	[[0,1,0,0,0,0,0,0,0,0,0],[0,1,0,0,0,0,0,0,0,0,0]]C3181733	phoneutria nigriventer omega phonetoxin iia,phoneutria nigriventer omega ptx iia	[[0,1,0,0,0,0,0,0,0,0,0],[0,1,0,0,0,0,0,0,0,0,0]]C0636765	acetamidotetramethylrhodamine,atmr	[[0,1,0,0,0,0,0,0,0,0,0],[0,1,0,0,0,0,0,0,0,0,0]]C2947886	catalase cysteine lysine methionine superoxide dismutase	[[0,0,0,0,0,0,0,1,0,0,0]]C0076720	tiquinamide,3 methyl 5 6 7 8 tetrahydroquinoline 8 thiocarboxamide	[[0,1,0,0,0,0,0,0,0,1,0],[0,1,0,0,0,0,0,0,0,0,0]]C0076723	tiropramide	[[0,0,0,0,0,0,0,1,0,0,0]]C1563848	pharken,elanco brand of pergolide mesylate	[[0,1,0,0,0,0,0,0,0,0,0],[0,1,0,0,0,0,0,0,0,0,0]]C0076724	tisocromide,4 dimethylamino 4 methylpentyl 2 carbamoyl 6 7 dimethoxy 1 thioisochroman 1 1 dioxide,n 3 dimethylamino 1 3 dimethylbutyl 3 4 dihydro 6 7 dimethoxy 2 1 benzoxathiin 3 carboxamide 1 1 dioxide,tisochromid	[[0,1,0,0,0,0,0,0,0,0,0],[0,1,0,0,0,0,0,0,0,0,0],[0,1,0,0,0,0,0,0,0,0,0],[0,1,0,0,0,0,0,0,0,0,0]]C1563844	symoron,yamanouchi brand of methadone hydrochloride	[[0,1,0,0,0,0,0,0,0,0,0],[0,1,0,0,0,0,0,0,0,0,0]]C2719854	trahist	[[0,0,0,0,0,0,0,1,0,0,0]]C1563840	biodone,biomet brand of methadone hydrochloride	[[0,1,0,0,0,0,0,0,0,0,0],[0,1,0,0,0,0,0,0,0,0,0]]C1563841	metadol,pharmascience brand of methadone hydrochloride	[[0,1,0,0,0,0,0,0,0,0,0],[0,1,0,0,0,0,0,0,0,0,0]]C0620260	pyridine 2 carboxaldehyde 2 pyridylhydrazonato cu ii dichloride	[[0,1,0,0,0,0,0,0,0,0,0]]C1563843	methaddict,addicare brand of methadone hydrochloride	[[0,1,0,0,0,0,0,0,0,0,0],[0,1,0,0,0,0,0,0,0,0,0]]C1879156	nyquil cough	[[0,0,0,0,0,0,0,1,0,0,0]]C1879153	symtan a	[[0,0,0,0,0,0,0,1,0,0,0]]C1720474	acacia starch tragacanth	[[1,0,0,0,0,0,0,0,0,0,0]]C0771080	japanese encephalitis vaccines,japanese b encephalitis vaccine,japanese encephalitis virus vaccine	[[0,1,0,0,0,0,1,0,0,0,1],[1,0,0,0,0,0,0,0,0,0,0],[1,0,0,0,0,0,0,0,0,0,0]]C2917444	vitamin d analog,vitamin d analogue	[[0,0,0,0,0,0,0,0,0,0,1],[0,0,0,0,0,0,0,0,0,0,1]]C1720478	aldioxa chloroxylenol	[[1,0,0,0,0,0,0,1,0,0,0]]C0389457	fr 129169	[[0,1,0,0,0,0,0,0,0,0,0]]C0092081	2 4 di 2 bromopropyl aminophenylazo benzoic acid	[[0,1,0,0,0,0,0,0,0,0,0]]C1879159	pediacare decongestant	[[0,0,0,0,0,0,0,1,0,0,0]]C0060154	fenazox,azoxybenzene	[[0,1,0,0,0,0,0,0,0,0,0],[1,1,0,0,0,0,0,0,0,0,0]]C0060157	fencamfamine,2 ethylamino 3 phenylnorcamphane,n ethyl 3 phenyl bicyclo 2 2 1 heptan 2 amine,n ethyl 3 phenylbicyclo 2 2 1 heptan 2 amine,2 phenyl 3 ethylaminobicyclo 2 2 1 heptane,2 ethylamino 3 phenylnorbornane,n ethyl 3 phenyl 2 norbornanamine	[[0,1,0,1,0,0,0,0,0,1,0],[0,1,0,0,0,0,0,0,0,0,0],[0,1,0,0,0,0,0,0,0,1,0],[0,1,0,0,0,0,0,0,0,0,0],[0,0,0,0,0,0,0,0,0,1,0],[0,0,0,0,0,0,0,0,0,1,0],[0,0,0,0,0,0,0,0,0,1,0]]C0060156	fenbufen,3 4 biphenylcarbonyl propionic acid,gamma oxo 1 1 biphenyl 4 butanoic acid	[[0,0,0,0,0,0,0,1,0,1,0],[0,1,0,0,0,0,0,0,0,0,0],[0,1,0,0,0,0,0,0,0,0,0]]C0060151	fenamiphos sulfoxide,fenamiphos sulphoxide,ethyl 3 methyl 4 methylsulfinyl phenyl ester 1 methylethyl phosphoramidic acid	[[0,1,0,0,0,0,0,0,0,0,0],[0,1,0,0,0,0,0,0,0,0,0],[0,1,0,0,0,0,0,0,0,0,0]]C0060150	fenamiphos sulfone,fenamiphos sulfphone,ethyl 3 methyl 4 methylsulfonyl phenyl ester 1 methylethyl phosphoramidic acid	[[0,1,0,0,0,0,0,0,0,0,0],[0,1,0,0,0,0,0,0,0,0,0],[0,1,0,0,0,0,0,0,0,0,0]]C0060153	fenazaflor,5 6 dichloro 1 phenoxycarbonyl 2 trifluoromethylbenzimidazole	[[0,1,0,0,0,0,0,0,0,0,0],[0,1,0,0,0,0,0,0,0,0,0]]C0634722	bis n n uracil 1 yl selenoxomethane,bnn selenoxomethane	[[0,1,0,0,0,0,0,0,0,0,0],[0,1,0,0,0,0,0,0,0,0,0]]C0115629	ekamet	[[0,1,0,0,0,0,0,0,0,0,0]]C0952166	r s isomer ifenprodil	[[0,1,0,0,0,0,0,0,0,0,0]]C0952167	r s r r r isomer ifenprodil tartrate 1 1	[[0,1,0,0,0,0,0,0,0,0,0]]C0952164	ifenprodil tartrate,r r r isomer ifenprodil tartrate 1 1	[[0,0,0,0,0,0,0,1,0,0,0],[0,1,0,0,0,0,0,0,0,0,0]]C0952165	r r r isomer ifenprodil tartrate 2 1	[[0,1,0,0,0,0,0,0,0,0,0]]C0299332	gea 5016,gea5016	[[0,1,0,0,0,0,0,0,0,0,0],[0,1,0,0,0,0,0,0,0,0,0]]C0299330	5 amino 3 4 chloro 3 trifluoromethyl phenyl chloride 1 2 3 4 oxatriazolum	[[0,1,0,0,0,0,0,0,0,0,0]]C0057258	deflazacort,11 beta 16 beta 21 acetyloxy 11 hydroxy 2 methyl 5 h pregna 1 4 dieno 17 16 d oxazole 3 20 dione,11 beta 21 dihydroxy 2 methyl 5 beta h pregna 1 4 dieno 17 16 d oxazole 3 20 dione 21 acetate	[[0,0,0,0,0,0,0,1,0,1,0],[0,1,0,0,0,0,0,0,0,1,0],[0,0,0,0,0,0,0,0,0,1,0]]C1096961	3 hydroxy 2 2 dimethyl n 4 5 dimethylamino 1 naphthyl sulfonyl amino phenyl propanamide	[[0,1,0,0,0,0,0,0,0,0,0]]C1096962	bay 38 4766	[[0,1,0,0,0,0,0,0,0,0,0]]C1306789	decongestant brand of pseudoephedrine	[[0,0,0,0,0,0,0,1,0,0,0]]C0057254	decyltrimethylammonium	[[0,1,0,0,0,0,0,0,0,0,0]]C0057256	defensins	[[0,0,0,0,0,0,1,0,0,0,1]]C0057257	defibrotide,defibrinotide,fraction p,prociclide	[[1,0,0,0,0,0,0,0,0,1,0],[0,1,0,0,0,0,0,0,0,1,0],[0,0,0,0,0,0,0,0,0,1,0],[0,0,0,0,0,0,0,0,0,1,0]]C1096969	2 p isothiocyanatobenzyl 1 4 7 10 13 pentaazacyclopentadecane n n n n n pentaacetic acid,bf pepa	[[0,1,0,0,0,0,0,0,0,0,0],[0,1,0,0,0,0,0,0,0,0,0]]C0134390	oxtripylline sa	[[0,1,0,0,0,0,0,0,0,0,0]]C0246584	cefozopran,czop	[[0,1,0,1,0,0,0,0,0,1,0],[0,1,0,0,0,0,0,0,0,0,0]]C0020172	humulin s,humulin,lilly brand of recombinant human insulin	[[0,0,0,0,0,0,0,1,0,0,0],[0,1,0,0,0,0,0,0,0,0,0],[0,1,0,0,0,0,0,0,0,0,0]]C0020171	humulin insulin,humulin	[[0,0,0,0,0,1,1,0,0,0,0],[0,0,0,0,0,0,1,0,0,0,0]]C0246581	celosorb	[[0,1,0,0,0,0,0,0,0,0,0]]C0754655	diamminebis ursodeoxycholate o o platinum ii,bamet r2	[[0,1,0,0,0,0,0,0,0,0,0],[0,1,0,0,0,0,0,0,0,0,0]]C0754656	fagaridine	[[0,1,0,0,0,0,0,0,0,0,0]]C0754651	n 1 n 1 dimethyl n 2 2 pyridylmethyl 5 isopropyl 3 8 dimethylazulene 1 carboxamidine	[[0,1,0,0,0,0,0,0,0,0,0]]C3254140	2 1 adamantyl methyl amino methyl 1 phenylcyclopropanecarboxylate	[[0,1,0,0,0,0,0,0,0,0,0]]C1120298	nsc 710305,nsc710305	[[0,1,0,0,0,0,0,0,0,0,0],[0,1,0,0,0,0,0,0,0,0,0]]C0953417	potassium salt anthraquinone sulfonate	[[0,1,0,0,0,0,0,0,0,0,0]]C0953416	sodium salt anthraquinone sulfonate	[[0,1,0,0,0,0,0,0,0,0,0]]C1566723	n 2 4 methoxybenzenesulfonamide phenyl 3 5 dimethyl 4 isoxazolcarboxamide	[[0,1,0,0,0,0,0,0,0,0,0]]C3252819	5 3 2 2 hydroxy 1 hydroxymethyl ethyl 5 methyl 1 2 3 4 tetrahydro 6 isoquinolinyl 1 2 4 oxadiazol 5 yl 2 1 methylethyl oxy benzonitrile,hhmtiom benzonitrile	[[0,1,0,0,0,0,0,0,0,0,0],[0,1,0,0,0,0,0,0,0,0,0]]C0092161	2 4 methyl 1 piperidinyl 5 aminophenyl 4 chlorophenyl methanone	[[0,1,0,0,0,0,0,0,0,0,0]]C3254149	6 chloro 2 methoxy n 2 methoxybenzyl acridin 9 amine,6 chloro mmbaa	[[0,1,0,0,0,0,0,0,0,0,0],[0,1,0,0,0,0,0,0,0,0,0]]C0539613	2 4 chlorobenzoyl 4 1 piperidinylacetyl 1 3 5 trimethylpyrrole	[[0,1,0,0,0,0,0,0,0,0,0]]C1742427	hiv lipo 6t	[[0,1,0,0,0,0,0,0,0,0,0]]C1566728	mm 10 cpd	[[0,1,0,0,0,0,0,0,0,0,0]]C0092166	2 4 methylpiperazin 1 yl 1 4 2 phenylethyl phenylethanone oxime	[[0,1,0,0,0,0,0,0,0,0,0]]C2827127	davasaicin	[[0,0,0,0,0,0,0,0,0,1,0]]C2827126	davalintide acetate,calcitonin 1 precursor 83 114 peptidamide acetate salt 5 l alanine sa 11 l arginine kr 18 l arginine kr 30 l asparagine gn 32 l tyrosine py calcitonin 1 oncorhynchus keta chum salmon l lysyl des 2 l serine 4 l threonine lt	[[0,0,0,0,0,0,0,0,0,1,0],[0,0,0,0,0,0,0,0,0,1,0]]C0529689	sitophilate	[[0,1,0,0,0,0,0,0,0,0,0]]C2722667	osteo poretical	[[0,0,0,0,0,0,0,1,0,0,0]]C0529688	serricornin	[[0,1,0,0,0,0,0,0,0,0,0]]C3255815	ziziphus extract,zizyphus	[[0,0,0,0,0,1,0,0,0,0,0],[0,0,1,0,0,0,0,0,0,0,0]]C3255814	zinc pidolate	[[0,0,1,0,0,0,0,0,0,0,0]]C3255817	methoxy peg 22 dodecyl glycol copolymer	[[0,0,1,0,0,0,0,0,0,0,0]]C1702615	white kidney bean extract,white bean extract,phaseolus vulgaris white extract	[[0,0,0,0,0,0,0,1,0,0,0],[1,0,0,0,0,0,0,0,0,0,0],[1,0,0,0,0,0,0,0,0,0,0]]C1702612	dehistine	[[0,0,0,0,0,0,0,1,0,0,0]]C3255810	beef sodium tallowate	[[0,0,1,0,0,0,0,0,0,0,0]]C3255812	spanish chestnut	[[0,0,1,0,0,0,0,0,0,0,0]]C0212332	hemoglobin polymer	[[0,1,0,0,0,0,0,0,0,0,0]]C0731023	vita e gels	[[0,0,0,0,0,0,0,1,0,0,0]]C0731020	virudox	[[0,0,0,0,1,0,0,0,0,0,0]]C0731021	vista methasone	[[0,0,0,0,0,0,0,1,0,0,0]]C0731026	vivotif	[[0,0,0,0,0,0,0,1,0,0,0]]C0731027	voltarol ophtha	[[0,0,0,0,0,0,0,1,0,0,0]]C0731024	vita e succinate	[[0,0,0,0,0,0,0,1,0,0,0]]C0731025	vivapryl	[[0,0,0,0,0,0,0,1,0,0,0]]C0635230	u 19052,r r s z 4 5 carboxy 1 3 4 heptylphenyl 1 propenyl 2 hydroxypentyl thio methyl 3 methoxy benzoic acid,u19052	[[0,1,0,0,0,0,0,0,0,0,0],[0,1,0,0,0,0,0,0,0,0,0],[0,1,0,0,0,0,0,0,0,0,0]]C0635232	ac pro phe his leu val tyr	[[0,1,0,0,0,0,0,0,0,0,0]]C0635233	ag 84 10	[[0,1,0,0,0,0,0,0,0,0,0]]C0635235	ac ile his pro phe his leu	[[0,1,0,0,0,0,0,0,0,0,0]]C0635236	ag 85 12	[[0,1,0,0,0,0,0,0,0,0,0]]C1122656	6 benzyloxy 9 2 fluoroethyl 9h purin 2 yl amine,9 fet bzopn	[[0,1,0,0,0,0,0,0,0,0,0],[0,1,0,0,0,0,0,0,0,0,0]]C1172784	endophenazine b	[[0,1,0,0,0,0,0,0,0,0,0]]C1122654	propane 2 sulfonic acid 2 4 iodophenyl cyclopentyl amide,2002uglycpd38	[[0,1,0,0,0,0,0,0,0,0,0],[0,1,0,0,0,0,0,0,0,0,0]]C1122655	7 8 dihydro 2 2 dimethyl 6 n phenyl 3 n phenylamino 4 n phenylimino 4h 6h 7h benzo a pyran 2 3 c phenazine,2002uglycpd39	[[0,1,0,0,0,0,0,0,0,0,0],[0,1,0,0,0,0,0,0,0,0,0]]C2946756	puracne oxygen cleansing	[[0,0,0,0,0,0,0,1,0,0,0]]C1122653	acinetoferrin imide	[[0,1,0,0,0,0,0,0,0,0,0]]C1122650	3 aminophthalimidoglutarimide,3 aminio phthalimido glutarimide	[[0,1,0,0,0,0,0,0,0,0,0],[0,1,0,0,0,0,0,0,0,0,0]]C1172782	endophenazine d	[[0,1,0,0,0,0,0,0,0,0,0]]C0634183	uniroid	[[0,1,0,0,0,0,0,0,0,0,0]]C0529686	sordidin	[[0,1,0,0,0,0,0,0,0,0,0]]C2699631	diclometide,3 5 dichlor n 2 diethylamino ethyl 2 methoxybenzamid	[[0,0,0,0,0,0,0,0,0,1,0],[0,0,0,0,0,0,0,0,0,1,0]]C1563671	1 5 bis 4 3 carbamimidoyl benzenesulfonylamino methyl phenoxy pentane	[[0,1,0,0,0,0,0,0,0,0,0]]C0125115	l1 oral chelate	[[0,1,0,0,0,0,0,0,0,0,0]]C1120294	criasiaticidine a,4 5 etheno 9 10 dihydroxy 6 phenanthridone	[[0,1,0,0,0,0,0,0,0,0,0],[0,1,0,0,0,0,0,0,0,0,0]]C0617220	prolonium iodide	[[0,1,0,0,0,0,0,0,0,1,0]]C0125111	l r resin	[[0,1,0,0,0,0,0,0,0,0,0]]C2720134	a hepatitis a virus inactivated antigen,a hepatitis a virus antigen	[[0,0,0,0,0,0,0,0,0,1,0],[0,0,0,0,0,0,0,0,0,1,0]]C2345444	clinisol 15	[[0,0,0,0,0,0,0,1,0,0,0]]C2738856	thyroxine 124 blood dot	[[0,0,0,1,0,0,0,0,0,0,0]]C2738857	thyroxine 124 dried blood spot	[[0,0,0,1,0,0,0,0,0,0,0]]C0106915	bornaprolol,1 2 exo bicyclo 2 2 1 hept 2 ylphenoxy 3 1 methylethyl amino 2 propanol,1 2 bicyclo 2 2 1 hept 2 ylphenoxy 3 1 methylethyl amino 2 propanol	[[0,1,0,0,0,0,0,0,0,1,0],[0,1,0,0,0,0,0,0,0,0,0],[0,1,0,0,0,0,0,0,0,0,0]]C0288171	irbesartan,2 n butyl 3 2 1h tetrazol 5 yl biphenyl 4 yl methyl 1 3 diazaspiro 4 4 non 1 en 4 one,2 butyl 3 2 1h tetrazol 5 yl 1 1 biphenyl 4 yl methyl 1 3 diazaspiro 4 4 non 1 en 4 one	[[0,0,0,0,0,0,0,0,1,1,0],[0,0,0,0,0,0,0,0,0,0,1],[0,0,0,0,0,0,0,0,0,0,1]]C0258967	recombinant fusion protein il 6 il 2,ch925	[[0,1,0,0,0,0,0,0,0,0,0],[0,1,0,0,0,0,0,0,0,0,0]]C0124862	l 2 amino 3 butynoic acid,s 2 amino 3 butynoic acid	[[0,1,0,0,0,0,0,0,0,0,0],[0,1,0,0,0,0,0,0,0,0,0]]C0061204	genkwadaphnin,12beta 12 benzoyloxy daphnetoxin	[[0,1,0,0,0,0,0,0,0,0,0],[0,1,0,0,0,0,0,0,0,0,0]]C1564262	cuxafenon,tad brand of propafenone hydrochloride	[[0,1,0,0,0,0,0,0,0,0,0],[0,1,0,0,0,0,0,0,0,0,0]]C0061202	genistein,genestein,4 5 7 trihydroxyisoflavone,genisteol,genisterin,prunetol,sophoricol,5 7 dihydroxy 3 4 hydroxyphenyl 4h 1 benzopyran 4 one	[[0,0,0,0,0,0,0,0,1,1,0],[0,0,0,0,1,0,1,0,0,0,0],[0,0,0,0,1,0,0,0,0,1,0],[0,0,0,0,1,0,0,0,0,1,0],[0,0,0,0,1,0,0,0,0,0,0],[0,0,0,0,1,0,0,0,0,1,0],[0,0,0,0,1,0,0,0,0,0,0],[0,0,0,0,1,0,0,0,0,0,0]]C0124867	l 2 methylthiazolidine 4 carboxylic acid	[[0,1,0,0,0,0,0,0,0,0,0]]C0061200	geniposide,jasminoidin	[[0,1,0,0,0,0,0,0,0,0,0],[0,1,0,0,0,0,0,0,0,0,0]]C0124869	2 oxothiazolidine 4 carboxylate,l 2 oxothiazolidine 4 carboxylate	[[0,1,0,0,0,0,0,0,0,0,0],[0,1,0,0,0,0,0,0,0,0,0]]C0209315	ecabet,12 sulfodehydroabietic acid	[[0,1,0,0,0,0,0,0,0,1,0],[0,1,0,0,0,0,0,0,0,0,0]]C0061208	gentamicin b,gentamycin b,o 6 amino 6 deoxy alpha d glucopyranosyl 1 4 o 3 deoxy 4 c methyl 3 methylamino beta l arabinopyranosyl 1 6 2 deoxy d streptamine,betamicin	[[0,1,0,0,0,0,0,0,0,0,0],[0,0,0,0,0,0,0,0,0,1,0],[0,1,0,0,0,0,0,0,0,0,0],[0,0,0,0,0,0,0,0,0,1,0]]C0651134	benzamido 2 nitro 5 thiazole,b2n5t,n 5 nitro 2 thiazolyl benzamide,2 benzamido 5 nitrothiazole	[[0,1,0,0,0,0,0,0,0,0,0],[0,1,0,0,0,0,0,0,0,0,0],[0,1,0,0,0,0,0,0,0,0,0],[0,1,0,0,0,0,0,0,0,0,0]]C0293150	n 4 methylbenzyl 4 o galactopyranosyl glucamine n carbodithioate,mebldtc	[[0,1,0,0,0,0,0,0,0,0,0],[0,1,0,0,0,0,0,0,0,0,0]]C0293153	telmesteine,3 ethylhydrogen 3 4 thiazolidinedicarboxylate	[[0,1,0,0,0,0,0,1,0,1,0],[0,1,0,0,0,0,0,0,0,0,0]]C2717280	2 3 diphenyl 1 4 naphthoquinone,dpnq cpd	[[0,1,0,0,0,0,0,0,0,0,0],[0,1,0,0,0,0,0,0,0,0,0]]C0723545	suphedrin	[[0,0,0,0,0,0,0,1,0,0,0]]C3170843	premarin pill 124 patient	[[0,0,0,1,0,0,0,0,0,0,0]]C3170841	pregabalin 124 urine	[[0,0,0,1,0,0,0,0,0,0,0]]C2699633	dicloralurea	[[0,0,0,0,0,0,0,0,0,1,0]]C1515690	rmva psa psma tricom,modified vaccinia ankara encoding psa psma tricom	[[0,0,0,0,0,0,0,0,0,1,0],[0,0,0,0,0,0,0,0,0,1,0]]C0658410	isf 3401,3beta 3 3 carboxy 1 oxopropoxy ursa 9 11 12 dien 28 oic acid	[[0,1,0,0,0,0,0,0,0,0,0],[0,1,0,0,0,0,0,0,0,0,0]]C0658415	xylosucrose,beta d fructofuranosyl alpha d xylopyranoside	[[0,1,0,0,0,0,0,0,0,0,0],[0,1,0,0,0,0,0,0,0,0,0]]C1515695	recombinant vaccinia psa l155 tricom vaccine,rvaccinia psa l155 tricom vaccine,recombinant vaccinia psa l155 tricom,prostvac v tricom,rvaccinia prostate specific antigen tricom vaccine	[[0,0,0,0,0,0,0,0,0,1,0],[0,0,0,0,0,0,0,0,0,1,0],[0,0,0,0,0,0,0,0,0,1,0],[0,0,0,0,0,0,0,0,0,1,0],[0,0,0,0,0,0,0,0,0,1,0]]C0658417	amsacrine dna,dna amsacrine complex	[[0,1,0,0,0,0,0,0,0,0,0],[0,1,0,0,0,0,0,0,0,0,0]]C0292209	samarium 153 particulate hydroxyapatite,153 sm phyp	[[0,1,0,0,0,0,0,0,0,0,0],[0,1,0,0,0,0,0,0,0,0,0]]C0080911	3 2 carboxyindol 3 yl propionic acid,cipa	[[0,1,0,0,0,0,0,0,0,0,0],[0,1,0,0,0,0,0,0,0,0,0]]C0080917	3 3 4 dimethoxyphenyl propenoic acid,3 4 dimethoxycinnamic acid	[[0,1,0,0,0,0,0,0,0,0,0],[0,1,0,0,0,0,0,0,0,0,0]]C0390452	4 acetamido 2 4 dideoxyglycerogalactooctopyranosyl phosphonic acid	[[0,1,0,0,0,0,0,0,0,0,0]]C0390450	4 acetamido 2 4 dideoxy d glycero beta d galacto octopyranosyl phosphonic acid,apana	[[0,1,0,0,0,0,0,0,0,0,0],[0,1,0,0,0,0,0,0,0,0,0]]C0390451	4 acetamido 2 4 dideoxy d glycero alpha d galacto octopyranosyl phosphonic acid,epana	[[0,1,0,0,0,0,0,0,0,0,0],[0,1,0,0,0,0,0,0,0,0,0]]C2699889	tameridone	[[0,0,0,0,0,0,0,0,0,1,0]]C2975094	3 5 dihydroxy 4 methoxy 6 6 dimethyl 4 5 dihydropyrano 2 3 7 8 flavone,3 5 dihydroxy mddf	[[0,1,0,0,0,0,0,0,0,0,0],[0,1,0,0,0,0,0,0,0,0,0]]C0667309	pro 3 bca phe nva ethyl ester	[[0,1,0,0,0,0,0,0,0,0,0]]C1831768	transdermal estrogen,estrodiol gel	[[0,0,0,0,1,0,0,0,0,1,0],[0,0,0,0,0,0,0,0,0,1,0]]C0532757	lipoyl 6 aminoquinoline	[[0,1,0,0,0,0,0,0,0,0,0]]C0667301	y 27632,y27632	[[0,1,0,0,0,0,0,0,0,0,0],[0,1,0,0,0,0,0,0,0,0,0]]C1602621	nitrotab	[[0,0,0,0,0,0,0,1,0,0,0]]C1456504	vga1102	[[0,1,0,0,0,0,0,0,0,0,0]]C1456505	ns3763	[[0,1,0,0,0,0,0,0,0,0,0]]C0005740	bleomycin,blm,bleo,bleomycin antibiotic	[[0,0,0,0,0,0,0,0,1,1,1],[0,0,0,0,1,0,1,0,0,0,0],[0,0,0,0,1,0,0,0,0,1,0],[0,0,0,0,0,0,0,0,0,1,0]]C2825465	nutrifolin	[[0,0,0,0,0,0,0,0,0,1,0]]C1456509	tannin	[[0,0,0,0,0,0,0,0,0,1,0]]C0699663	biocisplatinum	[[0,1,0,0,0,0,0,0,0,0,0]]C2365572	carbofed dm syrup	[[0,0,0,0,0,0,1,1,0,0,0]]C1528844	quadropril,awd pharma brand of spirapril hydrochloride	[[0,1,0,0,0,0,0,0,0,0,0],[0,1,0,0,0,0,0,0,0,0,0]]C0699665	platino	[[0,1,0,0,0,0,0,0,0,0,0]]C0960986	3 trifluoromethyldiazirin 3 yl galactopyranoside,3 3fmd galp	[[0,1,0,0,0,0,0,0,0,0,0],[0,1,0,0,0,0,0,0,0,0,0]]C3256023	cluster fig extract,cluster fig	[[0,0,0,0,0,1,0,0,0,0,0],[0,0,1,0,0,0,0,0,0,0,0]]C0699664	platidiam	[[0,1,0,0,0,0,0,0,1,0,0]]C0960989	2 hydroxybenzoic acid 3 nitrooxymethylphenyl ester,b nod cpd	[[0,1,0,0,0,0,0,0,0,0,0],[0,1,0,0,0,0,0,0,0,0,0]]C0052378	aristolochic acid ii,6 nitrophenanthro 3 4 d 3 dioxole 5 carboxylic acid	[[0,1,0,0,0,0,0,0,0,0,0],[0,1,0,0,0,0,0,0,0,0,0]]C1528848	myoson,ipg brand of pridinol mesylate	[[0,1,0,0,0,0,0,0,0,0,0],[0,1,0,0,0,0,0,0,0,0,0]]C0722634	piloptic 3	[[0,0,0,0,0,0,0,1,0,0,0]]C0052370	arildone,4 6 2 chloro 4 methoxy phenoxy hexyl 3 5 heptanedione	[[0,1,0,0,0,0,0,0,0,1,0],[0,1,0,0,0,0,0,0,0,0,0]]C0052372	aristeromycin diphosphate,1r 1alpha 2beta 3beta 4alpha mono 4 6 amino 9h purin 9 yl 2 3 dihydroxycyclopentyl methyl ester diphosphoric acid	[[0,1,0,0,0,0,0,0,0,0,0],[0,1,0,0,0,0,0,0,0,0,0]]C0124241	itf 282	[[0,1,0,0,0,0,0,0,0,0,0]]C1831762	allogeneic dendritic cell myeloma idiotype vaccine	[[0,0,0,0,1,0,0,0,0,1,0]]C1703485	freeze brand of benzocaine	[[0,0,0,0,0,0,0,1,0,0,0]]C0377503	tetraethylenepentamine pentahydrochloride	[[0,1,0,0,0,0,0,0,0,0,0]]C1186762	dakins solution,aqueous solution of sodium hypochlorite,dakins fluid	[[0,0,0,0,0,0,1,1,0,1,0],[0,0,0,0,0,0,0,0,0,1,0],[0,0,0,0,0,0,0,0,0,1,0]]C1312955	talaporfin sodium,taporfin sodium	[[0,0,0,0,0,0,0,0,0,1,0],[0,0,0,0,0,0,0,0,0,1,0]]C1186767	valrox	[[0,0,0,0,0,0,0,1,0,0,0]]C1186768	pranoxen continus	[[0,0,0,0,0,0,0,1,0,0,0]]C2741862	lf16 0687ms	[[0,1,0,0,0,0,0,0,0,0,0]]C2725944	accuretic 20 25	[[0,0,0,0,0,0,0,1,0,0,0]]C0648673	kansuiphorin a,6 2 3 dimethyl 1 oxobutoxy 1a 2 5 5a 6 9 10 10a octahydro 5 5a dihydroxy 1 1 7 9 tetramethyl 11 oxo 10a 1 oxododecyl oxy 1h 2 8a methanocyclopenta a cyclopropa e cyclodecen 4 yl methyl ester hexadecanoic acid,13 hydroxyingenol 3 2 3 dimethylbutanoate 13 dodecanoate 20 hexadecanoate	[[0,1,0,0,0,0,0,0,0,0,0],[0,1,0,0,0,0,0,0,0,0,0],[0,1,0,0,0,0,0,0,0,0,0]]C1656475	carbetapentane phenylephrine	[[0,0,0,0,0,0,0,1,0,0,0]]C0621237	indaniline,3 4 dimethylamino phenyl imino 6 oxo 1 4 cyclohexadiene 1 carboxamide	[[0,1,0,0,0,0,0,0,0,0,0],[0,1,0,0,0,0,0,0,0,0,0]]C1948856	elestrin	[[0,0,0,0,0,0,0,1,0,0,0]]C0121740	hh 10018	[[0,1,0,0,0,0,0,0,0,0,0]]C0121741	hh 197	[[0,1,0,0,0,0,0,0,0,0,0]]C0660650	5 1 3 3 trimethylindolinyl n 1 phenylethyl carbamate,tmi n pec	[[0,1,0,0,0,0,0,0,0,0,0],[0,1,0,0,0,0,0,0,0,0,0]]C1330262	excedrin quick tab peppermint	[[0,0,0,0,0,0,0,1,0,0,0]]C0701063	cerubidine	[[0,0,0,0,0,0,0,1,0,1,0]]C0701062	daunoblastin	[[0,0,0,0,0,0,0,0,0,1,0]]C0701064	eritron	[[0,1,0,0,0,0,0,0,0,0,0]]C0889564	2alpha 6alpha 11r isomer normetazocine hydrochloride	[[0,1,0,0,0,0,0,0,0,0,0]]C0889567	6r 6alpha 7beta r isomer oxacefamandole	[[0,1,0,0,0,0,0,0,0,0,0]]C1135667	canavalia lineata clsi iii protein	[[0,1,0,0,0,0,0,0,0,0,0]]C0052999	bathocuproine,2 9 dimethyl 4 7 diphenyl 1 10 phenanthroline	[[0,1,0,0,0,0,0,0,0,0,0],[0,1,0,0,0,0,0,0,0,0,0]]C0889566	2s 2alpha 6alpha 11r isomer normetazocine tartrate	[[0,1,0,0,0,0,0,0,0,0,0]]C1722427	shigyaku san,si ni san	[[0,1,0,0,0,0,0,0,0,0,0],[0,1,0,0,0,0,0,0,0,0,0]]C1135662	dornase alfa,alpha dornase,recombinant human deoxyribonuclease i,recombinant human dnase,rhdnase	[[1,0,0,0,0,0,1,1,0,1,0],[0,0,0,0,0,0,1,0,0,0,0],[0,1,0,0,0,0,0,0,0,1,0],[0,1,0,0,0,0,1,0,0,0,0],[0,1,0,0,0,0,1,0,0,0,0]]C0256052	dk ah 268,r monohydrochloride 3 1 2 3 4 dimethoxyphenyl ethyl 3 piperidinyl methyl 1 3 4 5 tetrahydro 7 8 dimethoxy 2h 3 benzazepin 2 one	[[0,1,0,0,0,0,0,0,0,0,0],[0,1,0,0,0,0,0,0,0,0,0]]C1330266	exoderm	[[0,0,0,0,0,0,0,1,0,0,0]]C0052993	basic aluminum carbonate gel,carbonato 2 tetrahydroxydialuminum,aluminum carbonate basic,aluminum hydroxycarbonate gel	[[0,0,0,0,0,0,0,1,0,0,0],[0,0,0,0,0,0,0,0,0,0,1],[0,0,0,0,0,0,0,0,0,0,1],[0,0,0,0,0,0,0,0,0,0,1]]C0256051	1 3 4 5 tetrahydro 7 8 dimethoxy 3 1 2 3 4 dimethoxyphenyl ethyl 3 piperidinyl methyl 2h 3 benzazepin 2 one hydrochloride	[[0,1,0,0,0,0,0,0,0,0,0]]C0889560	2s 2alpha 6alpha 11s isomer normetazocine hydrobromide	[[0,1,0,0,0,0,0,0,0,0,0]]C0610424	n n dimethyl alpha 2 4 tolyloxyl ethyl benzylamine hydrochloride	[[0,1,0,0,0,0,0,0,0,0,0]]C0610425	ly 125180,lilly 125180,hydrochloride n n dimethyl alpha 2 4 methylphenoxy ethyl benzenemethanamine,ly125180	[[0,1,0,0,0,0,0,0,0,0,0],[0,1,0,0,0,0,0,0,0,0,0],[0,1,0,0,0,0,0,0,0,0,0],[0,1,0,0,0,0,0,0,0,0,0]]C1513203	lantarel	[[0,0,0,0,1,0,0,0,0,0,0]]C1513202	fauldexato	[[0,0,0,0,1,0,0,0,0,0,0]]C1513205	lumexon	[[0,0,0,0,1,0,0,0,0,0,0]]C0720000	dok plus	[[0,0,0,0,0,0,0,1,0,0,0]]C1513207	metex	[[0,0,0,0,1,0,0,0,0,0,0]]C1513206	medsatrexate	[[0,0,0,0,1,0,0,0,0,0,0]]C1513209	metrotex	[[0,0,0,0,1,0,0,0,0,0,0]]C0256057	2 3 dihydro 5 methoxy 4 6 7 trimethyl 2 benzofuranyl acetic acid,2 3 dihydro 5 methoxy 4 6 7 trimethyl 2 benzofuranacetic acid	[[0,1,0,0,0,0,0,0,0,0,0],[0,1,0,0,0,0,0,0,0,0,0]]C0889562	2alpha 6alpha 11s isomer normetazocine	[[0,1,0,0,0,0,0,0,0,0,0]]C1881276	isocromil	[[0,0,0,0,0,0,0,0,0,1,0]]C0618217	dynorphin a,dyn a1 12	[[0,1,0,0,0,0,0,0,0,0,0],[0,1,0,0,0,0,0,0,0,0,0]]C1170544	prenatal h	[[0,0,0,0,0,0,1,0,0,0,0]]C0618215	5 3 4 trihydroxy 3 6 7 8 tetramethoxyflavone,2 3 4 dihydroxyphenyl 5 hydroxy 3 6 7 8 tetramethoxy 4h 1 benzopyran 4 one,thtmf	[[0,1,0,0,0,0,0,0,0,0,0],[0,1,0,0,0,0,0,0,0,0,0],[0,1,0,0,0,0,0,0,0,0,0]]C1170546	prenate gt	[[0,0,0,0,0,0,1,0,0,0,0]]C3178459	s 23906,s23906	[[0,1,0,0,0,0,0,0,0,0,0],[0,1,0,0,0,0,0,0,0,0,0]]C3178458	1 2 diacetoxy 3 14 dihydro 3 3 14 trimethyl 6 methoxy 7h benz b pyrano 3 2 d acridin 7 one	[[0,1,0,0,0,0,0,0,0,0,0]]C2827694	carbon c 14 eribulin acetate,14c eribulin acetate	[[0,0,0,0,0,0,0,0,0,1,0],[0,0,0,0,0,0,0,0,0,1,0]]C1881270	isaglidole	[[0,0,0,0,0,0,0,0,0,1,0]]C3178456	cc 0651,cc0651	[[0,1,0,0,0,0,0,0,0,0,0],[0,1,0,0,0,0,0,0,0,0,0]]C1170549	prevident dental rinse	[[0,0,0,0,0,0,0,1,0,0,0]]C1170548	prevex hc	[[0,0,0,0,0,0,1,0,0,0,0]]C2966569	topiramate 124 urine	[[0,0,0,1,0,0,0,0,0,0,0]]C1881271	isalsteine	[[0,0,0,0,0,0,0,0,0,1,0]]C1881272	isamoltan	[[0,0,0,0,0,0,0,0,0,1,0]]C0890216	5beta 11beta isomer 5 dihydrocortisol	[[0,1,0,0,0,0,0,0,0,0,0]]C0890211	isomer dexchlorpheniramine maleate 1 1	[[0,1,0,0,0,0,0,0,0,0,0]]C0890210	dexchlorpheniramine sodium maleate	[[0,1,0,0,0,0,0,0,0,0,0]]C0664828	21 acetoxy 18 hydroxy 10 phenyl 5 6 16 18 tetramethyl 11 cytochalasa 6 13 19 trien 1 one	[[0,1,0,0,0,0,0,0,0,0,0]]C0720240	epipram	[[0,0,0,0,0,0,0,1,0,0,0]]C0085432	aids vaccines	[[0,0,0,0,0,0,1,0,1,0,1]]C0720242	eppy n	[[0,0,0,0,0,0,0,1,0,0,0]]C0630119	u 72383,u 72 383	[[0,1,0,0,0,0,0,0,0,0,0],[0,1,0,0,0,0,0,0,0,0,0]]C0720244	equalize gas relief drops	[[0,0,0,0,0,0,0,1,0,0,0]]C0720245	equi cyte f	[[0,0,0,0,0,0,0,1,0,0,0]]C0720246	equi nade	[[0,0,0,0,0,0,0,1,0,0,0]]C0244960	levcromakalim,3s trans isomer cromakalim	[[0,0,0,0,0,0,0,0,0,1,0],[0,1,0,0,0,0,0,0,0,0,0]]C0889764	z isomer o diethylaminoethyl 4 chlorobenzaldoxime	[[0,1,0,0,0,0,0,0,0,0,0]]C0889763	hydrochloride o diethylaminoethyl 4 chlorobenzaldoxime	[[0,1,0,0,0,0,0,0,0,0,0]]C0889762	sodium salt mk 473	[[0,1,0,0,0,0,0,0,0,0,0]]C0889761	isomer sodium salt mk 473	[[0,1,0,0,0,0,0,0,0,0,0]]C0889760	isomer sodium salt mk 473	[[0,1,0,0,0,0,0,0,0,0,0]]C0388997	9 chloromethylanthracene	[[0,1,0,0,0,0,0,0,0,0,0]]C2348411	kx2 391	[[0,0,0,0,0,0,0,0,0,1,0]]C1438306	bombina bombina bsti protein,bombina bombina bombina skin trypsin thrombin inhibitor protein	[[0,1,0,0,0,0,0,0,0,0,0],[0,1,0,0,0,0,0,0,0,0,0]]C2945622	coricidin hbp	[[0,0,0,0,0,0,1,0,0,0,0]]C0723915	triphenicol	[[0,0,0,0,0,0,0,1,0,0,0]]C0723914	triphed	[[0,0,0,0,0,0,0,1,0,0,0]]C0087491	8 aza 8 4 chlorophenyl 1 4 dioxaspiro 4 5 decan 2 yl methyl 1 4 dihydro 2 6 dimethyl 4 3 nitrophenyl pyridine 3 5 dicarboxylate	[[0,1,0,0,0,0,0,0,0,0,0]]C0087490	8 1 3 4 dimethoxyphenyl 2 hydroxyethyl amino 3 7 dihydro 7 2 methoxyethyl 1 3 dimethyl 1h purine 2 6 dione	[[0,1,0,0,0,0,0,0,0,0,0]]C0723911	triotann s pediatric	[[0,0,0,0,0,0,0,1,0,0,0]]C0723910	triotann s	[[0,0,0,0,0,0,0,1,0,0,0]]C1137289	2 methyl 1 4 methyl 5 isoquinolinyl sulfonyl homopiperazine,dimethylfasudil	[[0,1,0,0,0,0,0,0,0,0,0],[0,1,0,0,0,0,0,0,0,0,0]]C1137288	aladan	[[0,1,0,0,0,0,0,0,0,0,0]]C2355148	dihydropyrazole 1 carboxylic acid	[[0,1,0,0,0,0,0,0,0,0,0]]C0296001	n n diallyl tyrosyl aminoisobutyryl aminoisobutyryl phenylalanyl leucine,n n diallyl tyr aib aib phe leu oh	[[0,1,0,0,0,0,0,0,0,0,0],[0,1,0,0,0,0,0,0,0,0,0]]C0296002	ici 174865,ici 174 865	[[0,1,0,0,0,0,0,0,0,0,0],[0,1,0,0,0,0,0,0,0,0,0]]C0968660	pramanicin	[[0,1,0,0,0,0,0,0,0,0,0]]C0164269	glutaryl alanyl alanyl alanyl ethylamide,glt ala 3 nhet,glt ala ala ala ethylamide,glutaryl trialanine ethylamide,glutaryl trialanyl ethylamide	[[0,1,0,0,0,0,0,0,0,0,0],[0,1,0,0,0,0,0,0,0,0,0],[0,1,0,0,0,0,0,0,0,0,0],[0,1,0,0,0,0,0,0,0,0,0],[0,1,0,0,0,0,0,0,0,0,0]]C0296006	trichokonin vi,gliodeliquescin a	[[0,1,0,0,0,0,0,0,0,0,0],[0,1,0,0,0,0,0,0,0,0,0]]C3257523	cucumber extract,cucumber	[[0,0,0,0,0,1,0,0,0,0,0],[0,0,1,0,0,0,0,0,0,0,0]]C1122115	n alpha 4 2 2 4 diaminoquinazolin 6 yl ethyl benzoyl n delta hemiphthaloylornithine,5 8 10 trideaza pt523	[[0,1,0,0,0,0,0,0,0,0,0],[0,1,0,0,0,0,0,0,0,0,0]]C3256980	nonoxynol 12	[[0,0,1,0,0,0,0,0,0,0,0]]C0646026	liriodendronine 2 o methyl ether,lir me	[[0,1,0,0,0,0,0,0,0,0,0],[0,1,0,0,0,0,0,0,0,0,0]]C0074513	silabolin	[[0,1,0,0,0,0,0,0,0,0,0]]C2948541	pediatex d 0 4 3 5	[[0,0,0,0,0,0,0,1,0,0,0]]C1134535	yttrium 90 ibritumomab tiuxetan,yttrium y 90 ibritumomab tiuxetan,90y ibritumomab tiuxetan,y90 labeled ibritumomab tiuxetan,yttrium y90 ibritumomab tiuxetan,y 90 ibritumomab tiuxetan,y 90 zevalin	[[0,1,0,0,1,0,0,0,0,0,0],[0,0,0,0,1,0,0,0,0,1,0],[0,0,0,0,1,0,0,0,0,0,0],[0,0,0,0,1,0,0,0,0,0,0],[0,0,0,0,0,0,0,0,0,1,0],[0,0,0,0,0,0,0,0,0,1,0],[0,0,0,0,0,0,0,0,0,1,0]]C3256988	opaspray	[[0,0,1,0,0,0,0,0,0,0,0]]C0724498	zostrix hp	[[0,0,0,0,0,0,1,1,0,0,0]]C0769577	tj 90	[[0,1,0,0,0,0,0,0,0,0,0]]C1122116	n alpha 4 5 2 4 diaminopteridin 6 yl pent 1 yn 4 yl benzoyl n delta hemiphthaloylornithine,10 propargyl 10 deaza pt523	[[0,1,0,0,0,0,0,0,0,0,0],[0,1,0,0,0,0,0,0,0,0,0]]C0894444	isomer isamfazone	[[0,1,0,0,0,0,0,0,0,0,0]]C0656074	2 benzothiazolesulfonamide,2 bts	[[0,1,0,0,0,0,0,0,0,0,0],[0,1,0,0,0,0,0,0,0,0,0]]C0894446	isomer isamfazone	[[0,1,0,0,0,0,0,0,0,0,0]]C0656071	demethylxestospongine b	[[0,1,0,0,0,0,0,0,0,0,0]]C0769578	qing fei tang,seihai to,seihaito,qingfeitang	[[0,1,0,0,0,0,0,0,0,0,0],[0,1,0,0,0,0,0,0,0,0,0],[0,1,0,0,0,0,0,0,0,0,0],[0,1,0,0,0,0,0,0,0,0,0]]C0656073	6 hydrogen 2 benzothiazolesulfonamide	[[0,1,0,0,0,0,0,0,0,0,0]]C0724496	zone a	[[0,0,0,0,0,0,0,1,0,0,0]]C0217087	cv 11974,cv11974	[[0,1,0,0,0,0,0,0,0,0,0],[0,1,0,0,0,0,0,0,0,0,0]]C0642248	4 1 naphthoyloxy 2 2 6 6 tetramethylpiperidine 1 oxyl,4 notmpo	[[0,1,0,0,0,0,0,0,0,0,0],[0,1,0,0,0,0,0,0,0,0,0]]C0074518	silatrane,silitranes	[[0,1,0,0,0,0,0,0,0,0,0],[0,1,0,0,0,0,0,0,0,0,0]]C1620124	4 2 4 difluorobiphenyl 4 yl 2 methylbutyric acid,deoxoflobufen	[[0,1,0,0,0,0,0,0,0,0,0],[0,1,0,0,0,0,0,0,0,0,0]]C0536470	sdz psd 958	[[0,1,0,0,0,0,0,0,0,0,0]]C0642241	6 bromomethyl 9h purine,6 bmpu	[[0,1,0,0,0,0,0,0,0,0,0],[0,1,0,0,0,0,0,0,0,0,0]]C0604990	erythro l isomer hydroxyglutamic acid,erythro l bhga	[[0,1,0,0,0,0,0,0,0,0,0],[0,1,0,0,0,0,0,0,0,0,0]]C1177017	dextran 110	[[0,0,0,0,0,0,0,1,0,0,0]]C1177014	nu k	[[0,0,0,0,0,0,0,1,0,0,0]]C1177015	dextraven 110	[[0,0,0,0,0,0,0,1,0,0,0]]C1177012	rescufolin	[[0,0,0,0,0,0,0,0,0,1,0]]C1177013	leo k	[[0,0,0,0,0,0,0,1,0,0,0]]C1177010	hepacon	[[0,0,0,0,0,0,0,1,0,0,0]]C2929085	tolnaftate triclosan	[[0,0,0,0,0,0,0,1,0,0,0]]C2929084	coumarin troxerutin	[[0,0,0,0,0,0,0,1,0,0,0]]C2929087	iodoquinol papaverine succinylsulfathiazole	[[0,0,0,0,0,0,0,1,0,0,0]]C1875241	hemorrhoidal preparations rectal	[[0,0,0,0,0,0,0,0,0,0,1]]C1988652	gramicidin d 124 isolate	[[0,0,0,1,0,0,0,0,0,0,0]]C1988653	gramicidin d 124 isolate serum	[[0,0,0,1,0,0,0,0,0,0,0]]C1177018	indoflex	[[0,0,0,0,0,0,0,1,0,0,0]]C1177019	indolar	[[0,0,0,0,0,0,0,1,0,0,0]]C0305396	balnetar	[[0,0,0,0,0,0,0,1,0,0,0]]C0310222	strongid t	[[0,0,0,0,0,0,0,1,0,0,0]]C0603907	senecion	[[0,1,0,0,0,0,0,0,0,0,0]]C1612759	clidinium bromide drug combination chlordiazepoxide hydrochloride,chlordiazepoxide hydrochloride clidinium bromide combination	[[0,1,0,0,0,0,0,0,0,0,0],[0,1,0,0,0,0,0,0,0,0,0]]C1829155	pliva brand of theophylline	[[0,0,0,0,0,0,0,1,0,0,0]]C1122118	1 methyl 4 3 4 5 trimethoxyphenyl 5 3 4 bis 2 methylamino ethyl phenyl imidazole,1 methyl tmpbpi	[[0,1,0,0,0,0,0,0,0,0,0],[0,1,0,0,0,0,0,0,0,0,0]]C0141550	saptomycin d	[[0,1,0,0,0,0,0,0,0,0,0]]C0054905	cb 10252,cb 10 252	[[0,1,0,0,0,0,0,0,0,0,0],[0,1,0,0,0,0,0,0,0,0,0]]C0764725	1 3 bis dicyanomethenyl 2 3 4 dihydroxyphenyl methenylindane	[[0,1,0,0,0,0,0,0,0,0,0]]C0054909	cb 3703,n 4 2 4 diamino 5 methyl 6 quinazolinyl methyl amino benzoyl l glutamic acid	[[0,1,0,0,0,0,0,0,0,0,0],[0,1,0,0,0,0,0,0,0,0,0]]C1875346	investigational anti viral drugs other	[[0,0,0,0,0,0,0,0,0,0,1]]C1875345	investigational anti viral drugs hiv	[[0,0,0,0,0,0,0,0,0,0,1]]C0603904	selenophene 6,monohydrochloride n 5 bromoselenophene 2 yl n n dimethyl n 2 pyridinyl 1 2 ethanediamine,n n dimethyl n alpha pyridyl n 5 bromoselenophene 2 yl ethylenediamine,sebrin	[[0,1,0,0,0,0,0,0,0,0,0],[0,1,0,0,0,0,0,0,0,0,0],[0,1,0,0,0,0,0,0,0,0,0],[0,1,0,0,0,0,0,0,0,0,0]]C1875343	investigational anti viral drugs hepatitis b	[[0,0,0,0,0,0,0,0,0,0,1]]C1875342	investigational anti tubercular drugs	[[0,0,0,0,0,0,0,0,0,0,1]]C1875341	investigational anti thrombotic drugs	[[0,0,0,0,0,0,0,0,0,0,1]]C1875340	investigational anti parasitic drugs	[[0,0,0,0,0,0,0,0,0,0,1]]C0387467	2 tetramethylene 1 3 dioxolane 4 5 dimethanamine n n hydroxyacetato o 1 o 2 platinum ii	[[0,1,0,0,0,0,0,0,0,0,0]]C0387466	ski 2032r	[[0,1,0,0,0,0,0,0,0,0,0]]C0387465	nsc d642488 p	[[0,1,0,0,0,0,0,0,0,0,0]]C0387464	nsc d642488	[[0,1,0,0,0,0,0,0,0,0,0]]C0387463	2 pentamethylene 1 3 dioxolane 4 5 dimethanamine n n hydroxyacetato o 1 o 2 platinum ii	[[0,1,0,0,0,0,0,0,0,0,0]]C0718278	advantage 24 contraceptive	[[0,0,0,0,0,0,0,1,0,0,0]]C0968080	bj46a anti hemorrhagic factor	[[0,1,0,0,0,0,0,0,0,0,0]]C0699333	makarol	[[0,0,0,0,1,0,0,0,0,0,0]]C0699598	sumetrolim	[[0,1,0,0,0,0,0,0,0,0,0]]C0387469	nsc d643687 s	[[0,1,0,0,0,0,0,0,0,0,0]]C0387468	nsc d643687	[[0,1,0,0,0,0,0,0,0,0,0]]C0699597	sulprim	[[0,1,0,0,0,0,0,0,0,0,0]]C0950935	rs 33295 198	[[0,1,0,0,0,0,0,0,0,0,0]]C0950934	opc 14597	[[0,1,0,0,0,0,0,0,0,0,0]]C0950931	redoxal	[[0,1,0,0,0,0,0,0,0,0,0]]C0699596	septrin	[[0,0,0,0,0,0,0,1,0,0,0]]C0386590	salmon egg cysteine protease inhibitor,chum salmon eci	[[0,1,0,0,0,0,0,0,0,0,0],[0,1,0,0,0,0,0,0,0,0,0]]C0699595	septra	[[0,0,0,0,0,0,1,1,0,0,0]]C0699594	oriprim	[[0,1,0,0,0,0,0,0,0,0,0]]C2737536	carisoprodol 124 xxx	[[0,0,0,1,0,0,0,0,0,0,0]]C0699593	metomide	[[0,1,0,0,0,0,0,0,0,0,0]]C0083486	mt 0703,6r 6 inner salt hydroxide 4 7 2 amino 4 thiazolyl 1 4 dihydro 1 5 dihydroxy 1 5 dihydroxy 4 oxo 2 pyridinyl carbonyl amino acetyl amino 2 carboxy 8 oxo 5 thia 1 azabicyclo 4 2 0 oct 2 en 3 yl methyl thio 1 2 hydroxyethyl pyridinium,mt0703	[[0,1,0,0,0,0,0,0,0,0,0],[0,1,0,0,0,0,0,0,0,0,0],[0,1,0,0,0,0,0,0,0,0,0]]C0083482	ms 932	[[0,1,0,0,0,0,0,0,0,0,0]]C0718273	adsorbocarpine	[[0,0,0,0,0,0,0,1,0,0,0]]C3165226	sumpweed pollen extract,iva annua pollen	[[0,0,0,0,0,1,0,0,0,0,0],[0,0,1,0,0,0,0,0,0,0,0]]C0627829	technetium tc 99m hexakis,tc 99m tmp 6	[[0,1,0,0,0,0,0,0,0,0,0],[0,1,0,0,0,0,0,0,0,0,0]]C0081437	acetyl sulfisoxazole,sulfafurazole acetyl,n 4 aminophenyl sulfonyl n 3 4 dimethyl 5 isoxazolyl acetamide,n 3 4 dimethylisoxazol 5 yl n sulphanilylacetamide	[[1,0,1,0,0,0,0,1,0,1,0],[1,0,0,0,0,0,0,0,0,0,0],[0,0,0,0,0,0,0,0,0,1,0],[0,0,0,0,0,0,0,0,0,1,0]]C0627824	ff 707	[[0,1,0,0,0,0,0,0,0,0,0]]C0718271	adrenocot	[[0,0,0,0,0,0,0,1,0,0,0]]C0627822	2 deoxy 3 5 bis o 4 methoxyphenoxy carbonyl 5 fluoro 3 4 n propoxybenzoyl uridine	[[0,1,0,0,0,0,0,0,0,0,0]]C0616214	sulfonated poly,sulfonated polyvinylidene fluoride,spvf	[[0,1,0,0,0,0,0,0,0,0,0],[0,1,0,0,0,0,0,0,0,0,0],[0,1,0,0,0,0,0,0,0,0,0]]C0627821	kwd 2066,alpha 1 1 dimethylethyl amino methyl 4 hydroxy benzenemethanol	[[0,1,0,0,0,0,0,0,0,0,0],[0,1,0,0,0,0,0,0,0,0,0]]C1270927	parenteral form dicyclomine	[[1,0,0,0,0,0,0,0,0,0,0]]C1270921	parenteral form pyridostigmine	[[1,0,0,0,0,0,0,0,0,0,0]]C1962045	premier skin care	[[0,0,0,0,0,0,0,1,0,0,0]]C1691227	mdx ctla 4,mdx ctla4	[[0,0,0,0,1,0,0,0,0,0,0],[0,0,0,0,1,0,0,0,0,0,0]]C1270929	parenteral form glycopyrrolate	[[1,0,0,0,0,0,0,0,0,0,0]]C0216350	aporphine	[[0,1,0,0,0,0,0,0,0,0,0]]C1130513	cleocin palmitate	[[0,1,0,0,0,0,0,0,0,0,0]]C0607342	4 4 biphenylbisdiazonium fluoroborate,bis tetrafluoroborate 1 1 1 biphenyl 4 4 bis diazonium	[[0,1,0,0,0,0,0,0,0,0,0],[0,1,0,0,0,0,0,0,0,0,0]]C0962040	dichlorodihydroxo glycylglycine platinate iv,h pt iv hdigly cl2 oh 2	[[0,1,0,0,0,0,0,0,0,0,0],[0,1,0,0,0,0,0,0,0,0,0]]C0649372	1 phenylcyclopentylamine,1 phenyl cyclopentanamine	[[0,1,0,0,0,0,0,0,0,0,0],[0,1,0,0,0,0,0,0,0,0,0]]C0964885	4 tert butyl 3 isopropyl 2 6 7 trioxa 1 phosphabicyclo 2 2 2 octane 1 sulfide,tbipps	[[0,1,0,0,0,0,0,0,0,0,0],[0,1,0,0,0,0,0,0,0,0,0]]C0649370	bn 52208,8 oxide inner salt hydroxide 8 hydroxy n n n trimethyl 5 methyloctadecylamino methyl 3 oxo 4 7 9 trioxa 2 aza 8 phosphaundecan 11 aminium	[[0,1,0,0,0,0,0,0,0,0,0],[0,1,0,0,0,0,0,0,0,0,0]]C0604699	6 chloro 5 3 hydroxycyclohexyl indan 1 carboxylic acid	[[0,1,0,0,0,0,0,0,0,0,0]]C3160335	benzodent	[[0,0,0,0,0,0,0,1,0,0,0]]C0074057	sarcophytol a,s z e e e 5 9 13 trimethyl 2 1 methylethyl 2 4 8 12 cyclotetradecatetraen 1 ol	[[0,1,0,0,0,0,0,0,0,0,0],[0,1,0,0,0,0,0,0,0,0,0]]C0649375	kahusu	[[0,1,0,0,0,0,0,0,0,0,0]]C0074058	sarcophytol b,6 10 14 trimethyl 3 1 methylethyl 3 5 9 13 cyclotetradecatetraene 1 2 diol	[[0,1,0,0,0,0,0,0,0,0,0],[0,1,0,0,0,0,0,0,0,0,0]]C0964889	1 4 fluorobenzyl 4 5 6 dimethoxy 1 oxoindan 2 yl methyl piperidine,4 fdp cpd	[[0,1,0,0,0,0,0,0,0,0,0],[0,1,0,0,0,0,0,0,0,0,0]]C0963358	ethylenediaminetetrakis methylphosphonic acid,edtp cpd	[[0,1,0,0,0,0,0,0,0,0,0],[0,1,0,0,0,0,0,0,0,0,0]]C0963353	sri 7614,ethyl 6 aminio 2 3 dihydro 4 phenyl 1h pyrido 4 3 b 1 4 diazepin 8 yl carbamate	[[0,1,0,0,0,0,0,0,0,0,0],[0,1,0,0,0,0,0,0,0,0,0]]C2351766	n benzylidenesulfonohydrazide	[[0,1,0,0,0,0,0,0,0,0,0]]C0963354	ethylmercury thiosalicylate	[[0,1,0,0,0,0,0,0,0,0,0]]C2933305	gyeongshingangjeehwan,ggex	[[0,1,0,0,0,0,0,0,0,0,0],[0,1,0,0,0,0,0,0,0,0,0]]C2930296	pilopine	[[0,0,0,0,0,0,0,1,0,0,0]]C2933307	memogain,gln 1062	[[0,1,0,0,0,0,0,0,0,0,0],[0,1,0,0,0,0,0,0,0,0,0]]C2933306	xiangdan	[[0,1,0,0,0,0,0,0,0,0,0]]C2933301	pro f egcg4	[[0,1,0,0,0,0,0,0,0,0,0]]C2933300	pro f egcg2	[[0,1,0,0,0,0,0,0,0,0,0]]C2933302	2s columbianetin 3 sulfate	[[0,1,0,0,0,0,0,0,0,0,0]]C0172586	perrimustine	[[0,1,0,0,0,0,0,0,0,0,0]]C0602445	e 383	[[0,1,0,0,0,0,0,0,0,0,0]]C0602444	3 chloro 2 3 diphenylpropiophenone,3 chloro 1 2 3 triphenyl 1 propanone	[[0,1,0,0,0,0,0,0,0,0,0],[0,1,0,0,0,0,0,0,0,0,0]]C0676730	alpha tocopheryloxybutyric acid,alpha tse	[[0,1,0,0,0,0,0,0,0,0,0],[0,1,0,0,0,0,0,0,0,0,0]]C2345761	normosol m	[[0,0,0,0,0,0,0,1,0,0,0]]C0602443	icr 340,dihydrochloride n 2 chloroethyl n 7 chloro 2 methoxybenzo b 1 5 naphthyridin 10 yl n ethyl 1 3 propanediamine	[[0,1,0,0,0,0,0,0,0,0,0],[0,1,0,0,0,0,0,0,0,0,0]]C1655108	gentex hc	[[0,0,0,0,0,0,0,1,0,0,0]]C0595331	lercanidipine hydrochloride,lercanidipine hcl,monohydrochloride 2 3 3 diphenylpropyl methylamino 1 1 dimethylethyl methyl ester 1 4 dihydro 2 6 dimethyl 4 3 nitrophenyl 3 5 pyridinedicarboxylic acid	[[0,0,0,0,0,0,1,1,0,1,0],[0,0,0,0,0,0,1,0,0,0,0],[0,0,0,0,0,0,0,0,0,1,0]]C1382890	autumn crocus preparation	[[0,0,0,0,0,1,0,0,0,0,0]]C2730082	drontal	[[0,0,0,0,0,0,0,1,0,0,0]]C2936485	adrenergic beta 3 receptor agonists,adrenergic beta 3 agonists	[[0,1,0,0,0,0,0,0,0,0,0],[0,1,0,0,0,0,0,0,0,0,0]]C2353893	golimumab	[[1,0,0,0,0,0,0,1,0,0,1]]C2936487	adrenergic beta 3 receptor antagonists,adrenergic beta 3 antagonists,adrenergic beta 3 receptor blockers,beta 3 adrenergic blocking agents	[[0,1,0,0,0,0,0,0,0,0,0],[0,1,0,0,0,0,0,0,0,0,0],[0,1,0,0,0,0,0,0,0,0,0],[0,1,0,0,0,0,0,0,0,0,0]]C0082160	cyclophellitol,5 hydroxymethyl 7 oxabicyclo 4 1 0 heptane 2 3 4 triol,1 2 anhydro 3 deoxy 3 hydroxymethyl d myo inositol,1 6 epi cyclophellitol,epi cpl	[[0,1,0,0,0,0,0,0,0,0,0],[0,1,0,0,0,0,0,0,0,0,0],[0,1,0,0,0,0,0,0,0,0,0],[0,1,0,0,0,0,0,0,0,0,0],[0,1,0,0,0,0,0,0,0,0,0]]C0082163	cyoctol,6 5 methoxy 1 heptyl bicyclo 3 3 0 octan 3 one	[[1,1,0,0,0,0,0,0,0,0,0],[0,1,0,0,0,0,0,0,0,0,0]]C1571131	aristoforin	[[0,1,0,0,0,0,0,0,0,0,0]]C0732624	detrunorm,schering plough brand of propiverine hydrochloride	[[0,1,0,0,0,0,0,0,0,0,0],[0,1,0,0,0,0,0,0,0,0,0]]C1991610	lidocaine 124 body fluid	[[0,0,0,1,0,0,0,0,0,0,0]]C1991611	lidocaine 124 urine	[[0,0,0,1,0,0,0,0,0,0,0]]C1991612	lidocaine 124 xxx	[[0,0,0,1,0,0,0,0,0,0,0]]C2932882	spinetoram	[[0,1,0,0,0,0,0,0,0,0,0]]C2700333	zoledronate trisodium,hydrate 5 2 trisodium hydrogen 1 hydroxy 2 imidazol 1 ylethylidene diphosphonate,trisodium salt tetrahydrate 5 2 1 hydroxy 2 1h imidazol 1 yl ethylidene bis phosphonic acid	[[0,0,0,0,0,0,0,0,0,1,0],[0,0,0,0,0,0,0,0,0,1,0],[0,0,0,0,0,0,0,0,0,1,0]]C3255975	propylene glycol dicaprylate	[[0,0,1,0,0,0,0,0,0,0,0]]C2932885	apigenin 7 o 6 o galloyl beta d glucopyranoside	[[0,1,0,0,0,0,0,0,0,0,0]]C0530865	5 10 15 20 tetrakis 1 decylpyridinium 4 yl 21h 23h porphin tetrabromide,por10 porphyrin	[[0,1,0,0,0,0,0,0,0,0,0],[0,1,0,0,0,0,0,0,0,0,0]]C0530863	1 4 6 8 tetramethyl 2h furo 2 3 h quinolin 2 one,1 4 6 8 tetramethyl fq	[[0,1,0,0,0,0,0,0,0,0,0],[0,1,0,0,0,0,0,0,0,0,0]]C0530860	wrc 0470	[[0,1,0,0,0,0,0,0,0,0,0]]C0254046	9 3 3 dimethyl 5 phosphonopentyl guanine,dmppg	[[0,1,0,0,0,0,0,0,0,0,0],[0,1,0,0,0,0,0,0,0,0,0]]C0381028	3 butyl 3 ethyl 2 3 4 5 tetrahydro 5 phenyl 1 4 benzothiazepine 1 1 dioxide	[[0,1,0,0,0,0,0,0,0,0,0]]C0254044	rb 105	[[0,1,0,0,0,0,0,0,0,0,0]]C0718778	b stress	[[0,0,0,0,0,0,1,0,0,0,0]]C0254042	rwj 37796	[[0,1,0,0,0,0,0,0,0,0,0]]C0254043	n 2 mercaptomethyl 3 phenylbutanoyl l alanine	[[0,1,0,0,0,0,0,0,0,0,0]]C0254041	indralin	[[0,1,0,0,0,0,0,0,0,0,0]]C0718772	b fedrine pd	[[0,0,0,0,0,0,0,1,0,0,0]]C0718771	b fedrine	[[0,0,0,0,0,0,0,1,0,0,0]]C0718770	b d glucose	[[0,0,0,0,0,0,0,1,0,0,0]]C0381025	1357u88	[[0,1,0,0,0,0,0,0,0,0,0]]C0254048	n butyl n methyl 13 14 bis 4 hydroxyphenyl 12 thiatetradecanamide,bmbhtd	[[0,1,0,0,0,0,0,0,0,0,0],[0,1,0,0,0,0,0,0,0,0,0]]C0381026	1370u88	[[0,1,0,0,0,0,0,0,0,0,0]]C2939857	phytolacca americana root	[[0,0,1,0,0,0,0,0,0,0,0]]C0062006	gur,palm sugar	[[0,1,0,0,0,0,1,0,0,0,0],[0,0,0,0,0,0,1,0,0,0,0]]C0958582	dimethylpropiothetin chloride	[[0,1,0,0,0,0,0,0,0,0,0]]C0958583	dimethylpropiothetin hydrochloride	[[0,1,0,0,0,0,0,0,0,0,0]]C0304213	animal oil	[[1,0,0,0,0,0,0,0,0,0,0]]C2938468	milkhouse brand sprayable chg teat dip	[[0,0,0,0,0,0,0,1,0,0,0]]C0719044	burn brand of nitrofurazone	[[0,0,0,0,0,0,0,1,0,0,0]]C0594013	herpetad,tad brand of aciclovir	[[0,1,0,0,0,0,0,0,0,0,0],[0,1,0,0,0,0,0,0,0,0,0]]C2938461	artemisia cina flower	[[0,0,1,0,0,0,0,0,0,0,0]]C2938460	agrimonia eupatoria preparation,agrimonia eupatoria	[[0,0,0,0,0,1,0,0,0,0,0],[0,0,1,0,0,0,0,0,0,0,0]]C1881108	cell cycle checkpoint dna repair antagonist ic83	[[0,0,0,0,0,0,0,0,0,1,0]]C2938464	dairyland brand sprayable chg teat dip	[[0,0,0,0,0,0,0,1,0,0,0]]C2698079	amanozine	[[0,0,0,0,0,0,0,0,0,1,0]]C1990779	insulin ultralente 124 dose	[[0,0,0,1,0,0,0,0,0,0,0]]C1880271	combotox	[[0,0,0,0,0,0,0,0,0,1,0]]C1880270	deglycosylated ricin a chain conjugated anti cd19 anti cd22 immunotoxins	[[0,0,0,0,0,0,0,0,0,1,0]]C0292001	birm 270	[[0,1,0,0,0,0,0,0,0,0,0]]C0526622	trans acpc	[[0,1,0,0,0,0,0,0,0,0,0]]C0072063	procymidone,3 5 dichlorophenyl 1 2 dimethylcyclopropane 1 2 dicarboximide,dicyclidine	[[0,1,0,0,0,0,0,0,0,0,0],[0,1,0,0,0,0,0,0,0,0,0],[0,1,0,0,0,0,0,0,0,0,0]]C2698073	altinicline	[[0,0,0,0,0,0,0,0,0,1,0]]C0072061	procyanidin,2 3 4 dihydroxyphenyl 2 2 3 4 dihydroxyphenyl 3 4 dihydro 5 7 dihydroxy 2h 1 benzopyran 3 yl oxy 3 4 dihydro 2h 1 benzopyran 3 4 5 7 tetrol	[[0,1,0,0,0,0,1,0,0,0,0],[0,1,0,0,0,0,0,0,0,0,0]]C0072066	prodolic acid,1 3 4 9 tetrahydro 1 propylpyrano 3 4 b indole 1 acetic acid	[[0,1,0,0,0,0,0,0,0,0,0],[0,1,0,0,0,0,0,0,0,0,0]]C2698074	altoqualine	[[0,0,0,0,0,0,0,0,0,1,0]]C2698077	alvespimycin hydrochloride,monohydrochloride geldanamycin 17 demethoxy 17 2 dimethylamino ethyl amino,17 dmag hcl	[[0,0,0,0,0,0,0,0,0,1,0],[0,0,0,0,0,0,0,0,0,1,0],[0,0,0,0,0,0,0,0,0,1,0]]C0072065	prodipin,1 tert isopropyl 4 4 diphenylpiperidine,1 isopropyl 4 4 diphenylpiperidine	[[0,1,0,0,0,0,0,0,0,1,0],[0,1,0,0,0,0,0,0,0,0,0],[0,0,0,0,0,0,0,0,0,1,0]]C0048197	parachlorophenol,4 chlorophenol,p chlorophenol,para monochlorophenol,4 monochlorophenol	[[0,1,0,0,0,0,0,0,0,1,0],[0,0,0,0,0,0,0,0,0,1,1],[0,0,0,0,0,0,0,0,0,1,1],[0,0,0,0,0,0,0,0,0,0,1],[0,0,0,0,0,0,0,0,0,0,1]]C0069588	oracon	[[0,1,0,0,0,0,0,0,0,0,0]]C2981001	lohist psb	[[0,0,0,0,0,0,0,1,0,0,0]]C2981006	revitaderm 40	[[0,0,0,0,0,0,0,1,0,0,0]]C0069585	nitecapone,3 3 4 dihydroxy 5 nitrobenzylidine 2 4 pentanedione	[[0,1,0,0,0,0,0,0,0,0,0],[0,1,0,0,0,0,0,0,0,0,0]]C0069584	optocillin	[[0,1,0,0,0,0,0,0,0,0,0]]C0069587	orabase	[[0,0,0,0,0,0,0,1,0,0,1]]C0069586	or486,or 486	[[0,1,0,0,0,0,0,0,0,0,0],[0,1,0,0,0,0,0,0,0,0,0]]C0306604	pork zinc insulin,porcine lente insulin,pork lente insulin	[[0,0,0,0,0,0,0,1,0,0,0],[0,1,0,0,0,0,0,0,0,0,0],[0,1,0,0,0,0,0,0,0,0,0]]C0078601	xanthobine,8 bromocaffeine,8 caffeine bromide	[[0,1,0,0,0,0,0,0,0,0,0],[0,1,0,0,0,0,0,0,0,0,0],[0,1,0,0,0,0,0,0,0,0,0]]C2929869	guaifenesin terpin hydrate tetracycline	[[0,0,0,0,0,0,0,1,0,0,0]]C2929868	docosahexaenoate eicosapentaenoate omega 3 acid ethyl esters usp pyridoxine vitamin b 12	[[0,0,0,0,0,0,0,1,0,0,0]]C0049829	7 amino 4 methylcoumarin 3 acetic acid,7 amca	[[0,1,0,0,0,0,0,0,0,0,0],[0,1,0,0,0,0,0,0,0,0,0]]C0306603	lente iletin ii	[[0,0,0,0,0,0,0,0,0,1,0]]C2929863	diptheria protein haemophilus capsular oligosaccharide	[[0,0,0,0,0,0,0,1,0,0,0]]C2929862	benzoyl peroxide colloid sulfur	[[0,0,0,0,0,0,0,1,0,0,0]]C2929861	atropine benzoate hyoscyamine methenamine methylene blue phenyl salicylate	[[0,0,0,0,0,0,0,1,0,0,0]]C2929860	kava preparation passion flower extract valerian root extract	[[0,0,0,0,0,0,0,1,0,0,0]]C2929867	monofluorophosphate potassium nitrate	[[0,0,0,0,0,0,0,1,0,0,0]]C0306609	levatol	[[0,0,0,0,0,0,0,0,0,1,0]]C2929865	alginic acid aluminum hydroxide magnesium carbonate	[[0,0,0,0,0,0,0,1,0,0,0]]C1655661	weight loss substance	[[0,0,0,0,0,0,0,1,0,0,0]]C0765139	7 epiclusianone	[[0,1,0,0,0,0,0,0,0,0,0]]C0059052	embelin,2 5 dihydroxy 3 undecyl 1 4 benzoquinone,2 5 dihydroxy 3 undecyl p benzoquinone	[[0,0,0,0,0,0,0,1,0,0,0],[0,1,0,0,0,0,0,0,0,0,0],[0,1,0,0,0,0,0,0,0,0,0]]C0059053	embramine,2 p bromo alpha methyl alpha phenylbenzyloxy n n dimethylethyl amine	[[0,1,0,0,0,0,0,0,0,1,0],[0,1,0,0,0,0,0,0,0,0,0]]C1171081	raspberry extract,raspberry	[[1,0,0,0,0,1,0,0,0,0,0],[0,0,0,0,0,0,1,0,0,0,0]]C0059059	embutramide,n 2 3 methoxyphenyl 2 ethylbutyl 1 gamma hydroxybutyramide	[[0,1,0,0,0,0,0,0,0,1,0],[0,1,0,0,0,0,0,0,0,0,0]]C0765133	jaspamide b	[[0,1,0,0,0,0,0,0,0,0,0]]C0765132	4 epifriedelin	[[0,1,0,0,0,0,0,0,0,0,0]]C0765134	jaspamide c	[[0,1,0,0,0,0,0,0,0,0,0]]C0765137	maprouneacin	[[0,1,0,0,0,0,0,0,0,0,0]]C1531420	ru 1148	[[0,1,0,0,0,0,0,0,0,0,0]]C0071744	post gamma globulin,cystatin c,gamma trace,cystatin 3,neuroendocrine basic polypeptide	[[0,1,0,0,0,0,0,0,0,1,1],[0,0,0,1,0,0,1,0,0,1,0],[0,1,0,0,0,0,0,0,0,1,1],[0,0,0,0,0,0,0,0,0,1,1],[0,1,0,0,0,0,0,0,0,0,1]]C1709080	mouse prostate specific membrane antigen plasmid dna vaccine,mouse psma dna,mouse psma dna vaccine	[[0,0,0,0,1,0,0,0,0,1,0],[0,0,0,0,1,0,0,0,0,0,0],[0,0,0,0,0,0,0,0,0,1,0]]C1709081	mouse gp100 plasmid dna vaccine	[[0,0,0,0,1,0,0,0,0,1,0]]C0032841	potassium sorbate	[[0,1,1,0,0,0,1,1,0,1,0]]C0605717	1 1 bis diazoacetyl 2 phenylethane	[[0,1,0,0,0,0,0,0,0,0,0]]C0378144	mls 266 337	[[0,1,0,0,0,0,0,0,0,0,0]]C0668537	heteromine d	[[0,1,0,0,0,0,0,0,0,0,0]]C1881946	no releasing ibuprofen,4 nitrooxy butyl ester 3 4 2 4 isobutylphenyl propionyl oxy 3 methoxyphenyl acrylic acid	[[0,0,0,0,0,0,0,0,0,1,0],[0,0,0,0,0,0,0,0,0,1,0]]C0668539	heteromine e	[[0,1,0,0,0,0,0,0,0,0,0]]C0668538	heteromine f	[[0,1,0,0,0,0,0,0,0,0,0]]C0063756	iodipine	[[0,1,0,0,0,0,0,0,0,0,0]]C0130259	n 3 3 4 acetyl 3 hydroxy 2 n propylphenoxy propoxy 4 chloro 6 methylphenyl 1h tetrazole 5 carboxamide	[[0,1,0,0,0,0,0,0,0,0,0]]C0063754	iodinol,ethenol homopolymer mixt with potassium iodide,iodopolyvinyl alcohol	[[0,1,0,0,0,0,0,0,0,0,0],[0,1,0,0,0,0,0,0,0,0,0],[0,1,0,0,0,0,0,0,0,0,0]]C0959674	2s cis isomer 3 benzylglycidol	[[0,1,0,0,0,0,0,0,0,0,0]]C0063753	iodine monochloride,iodine chloride	[[1,1,0,0,0,0,0,0,0,0,0],[0,1,0,0,0,0,0,0,0,0,0]]C0063750	iodinated glycerol,iodopropylidene glycerol	[[1,0,0,0,0,0,0,1,0,0,0],[0,0,0,0,0,0,0,0,0,0,1]]C0388500	plivasept	[[0,1,0,0,0,0,0,0,0,0,0]]C0055628	chromium oxide,chromic oxide	[[0,1,0,0,0,0,0,0,0,0,0],[0,1,1,0,0,0,0,0,0,0,0]]C0141357	antibiotic s 632 b2,s 632 b,s632 b2	[[0,1,0,0,0,0,0,0,0,0,0],[0,1,0,0,0,0,0,0,0,0,0],[0,1,0,0,0,0,0,0,0,0,0]]C0387859	sk 204	[[0,1,0,0,0,0,0,0,0,0,0]]C0387852	kb 23	[[0,1,0,0,0,0,0,0,0,0,0]]C2974378	4 desmethylepipodophyllotoxin 7 o glucopyranoside,4 dpg cpd	[[0,1,0,0,0,0,0,0,0,0,0],[0,1,0,0,0,0,0,0,0,0,0]]C0387851	2 hydroxy 5 n 2 5 dihydroxyphenyl methyl amino benzoic acid 3 phenylpropyl ester,3 phenylpropyl 2 hydroxy 5 n 2 5 dihydroxyphenyl methyl amino benzoate	[[0,1,0,0,0,0,0,0,0,0,0],[0,1,0,0,0,0,0,0,0,0,0]]C1698564	barri care	[[0,0,0,0,0,0,0,1,0,0,0]]C0301554	terpin hydrate,monohydrate cis isomer terpin	[[0,0,0,0,0,0,0,1,0,0,0],[0,1,0,0,0,0,0,0,0,0,0]]C0301556	arginine hydrochloride,arginine hcl	[[0,0,1,0,0,0,1,1,0,1,0],[0,0,0,0,1,0,1,0,0,0,0]]C0301551	alpha amylase preparation,alpha amylase agent	[[1,0,0,0,0,0,0,0,0,0,0],[1,0,0,0,0,0,0,0,0,0,0]]C0301550	merethoxylline procaine	[[1,0,0,0,0,0,0,0,0,0,0]]C2348142	sampirtine	[[0,0,0,0,0,0,0,0,0,1,0]]C1590098	oro clense	[[0,0,0,0,0,0,0,1,0,0,0]]C0301558	betahistidine	[[1,0,0,0,0,0,1,0,0,0,0]]C1634528	altafluor	[[0,0,0,0,0,0,0,1,0,0,0]]C0965372	taiwanschirin d	[[0,1,0,0,0,0,0,0,0,0,0]]C1165854	allergenic extract mite	[[0,0,0,0,0,0,0,1,0,0,0]]C3253758	hexadeca 4 7 10 13 tetraenoic acid,4 7 10 13 hexadecatetraenoic acid	[[0,1,0,0,0,0,0,0,0,0,0],[0,1,0,0,0,0,0,0,0,0,0]]C2747730	candida tropicalis extract	[[0,0,0,0,0,0,0,1,0,0,0]]C0626704	3 cyanophosphoenolpyruvate	[[0,1,0,0,0,0,0,0,0,0,0]]C1527323	rodex brand of warfarin,rodex	[[0,0,0,0,0,1,0,0,0,0,0],[0,0,0,0,1,0,0,0,0,0,0]]C1527328	ci 908	[[0,0,0,0,1,0,0,0,0,0,0]]C1098881	neodysiherbaine a	[[0,1,0,0,0,0,0,0,0,0,0]]C1881893	monoctanoin component b	[[0,0,0,0,0,0,0,0,0,1,0]]C0908617	dota tyr 3 octreotide,dota 0 tyr 3 octreotide,dota tyrosyl 3 octreotide	[[0,1,0,0,0,0,0,0,0,0,0],[0,1,0,0,0,0,0,0,0,0,0],[0,1,0,0,0,0,0,0,0,0,0]]C0672686	hexahydrocolupulone,hhc cpd	[[0,1,0,0,0,0,0,0,0,0,0],[0,1,0,0,0,0,0,0,0,0,0]]C0939839	dichapetalum cymosum preparation	[[0,0,0,0,0,1,0,0,0,0,0]]C0006246	brompheniramine,p bromdylamine,para bromdylamine,gamma 4 bromophenyl n n dimethyl 2 pyridinepropanamine,parabromdylamine	[[0,0,0,0,0,0,1,1,0,1,0],[0,1,0,0,0,0,0,0,0,0,1],[0,1,0,0,0,0,0,0,0,0,1],[0,0,0,0,0,0,0,0,0,0,1],[1,0,0,0,0,0,0,0,0,0,0]]C0939836	euphorbia cyparissias preparation	[[0,0,0,0,0,1,0,0,0,0,0]]C0939835	horseradish preparation,horseradish	[[0,0,0,0,0,1,0,0,0,0,0],[0,0,0,0,0,0,1,0,0,0,0]]C0939833	primula obconica preparation,primrose	[[0,0,0,0,0,1,0,0,0,0,0],[0,0,0,0,0,0,1,0,0,0,0]]C0390317	caffeoylmalic acid	[[0,1,0,0,0,0,0,0,0,0,0]]C0939831	beta vulgaris extract,beet root	[[0,0,0,0,0,1,0,0,0,0,0],[0,0,0,0,0,0,1,0,0,0,0]]C0939830	polygonum punctatum preparation	[[0,0,0,0,0,1,0,0,0,0,0]]C0641976	gyki 46544,gyki 46 544	[[0,1,0,0,0,0,0,0,0,0,0],[0,1,0,0,0,0,0,0,0,0,0]]C0390316	ergolide	[[0,1,0,0,0,0,0,0,0,0,0]]C1165640	allergenic extract gerbil hair gerbillus spp	[[0,0,0,0,0,0,0,1,0,0,0]]C0641971	ch 27584	[[0,1,0,0,0,0,0,0,0,0,0]]C0641973	gyki 52713	[[0,1,0,0,0,0,0,0,0,0,0]]C1165649	tall ambrosia trifida allergenic extract ragweed	[[0,0,0,0,0,0,0,1,0,0,0]]C2699894	taribavirin hydrochloride	[[0,0,0,0,0,0,0,0,0,1,0]]C1098884	4 sulfono difluoromethyl phenylalanine,4 sdpa cpd,l 4 sulfono difluoromethyl phenylalanine	[[0,1,0,0,0,0,0,0,0,0,0],[0,1,0,0,0,0,0,0,0,0,0],[0,1,0,0,0,0,0,0,0,0,0]]C0379233	collunovar	[[0,1,0,0,0,0,0,0,0,0,0]]C0379232	arsevan	[[0,1,0,0,0,0,0,0,0,0,0]]C0379237	novarsenobillon	[[0,1,0,0,0,0,0,0,0,0,0]]C0379236	novarsenobenzene	[[0,1,0,0,0,0,0,0,0,0,0]]C0379235	novarsan	[[0,1,0,0,0,0,0,0,0,0,0]]C0379234	neoarsenobenzol	[[0,1,0,0,0,0,0,0,0,0,0]]C0607100	eriofertopin,3ar 3ar 4r 6z 8s 10e 11ar 2 3 3a 4 5 8 9 11a octahydro 8 hydroxy 6 hydroxymethyl 10 methyl 3 methylene 2 oxocyclodeca b furan 4 yl ester 2 methyl 2 propenoic acid	[[0,1,0,0,0,0,0,0,0,0,0],[0,1,0,0,0,0,0,0,0,0,0]]C1121679	s 1360,s1360 cpd	[[0,1,0,0,0,0,0,0,0,0,0],[0,1,0,0,0,0,0,0,0,0,0]]C1121678	bms 806,bms806	[[0,1,0,0,0,0,0,0,0,0,0],[0,1,0,0,0,0,0,0,0,0,0]]C2930482	zinc oxide drug combination zinc peroxide titanium dioxide,titanium dioxide zinc peroxide zinc oxide	[[0,1,0,0,0,0,0,0,0,0,0],[0,1,0,0,0,0,0,0,0,0,0]]C1613074	eyepentolate	[[0,0,0,0,0,0,0,1,0,0,0]]C0009543	complement inhibitor	[[1,0,0,0,0,0,0,0,1,0,1]]C2740736	ambi 10peh 400gfn	[[0,0,0,0,0,0,0,1,0,0,0]]C1098888	stearyl nle 17 neurotensin 6 11 vip 7 28,snh peptide	[[0,1,0,0,0,0,0,0,0,0,0],[0,1,0,0,0,0,0,0,0,0,0]]C0302609	xanthine oxidase inhibitors	[[1,0,0,0,0,0,0,0,1,1,0]]C1881947	no releasing indomethacin,nitroxymethylphenyl indomethacin	[[0,0,0,0,0,0,0,0,0,1,0],[0,0,0,0,0,0,0,0,0,1,0]]C1171442	salbuvent	[[0,0,0,0,0,0,0,1,0,0,0]]C2984523	anti loxl2 monoclonal antibody ab0024	[[0,0,0,0,0,0,0,0,0,1,0]]C2984522	retroviral virus vector encoding cytosine deaminase	[[0,0,0,0,0,0,0,0,0,1,0]]C2984521	anti her2 monoclonal antibody mgah22	[[0,0,0,0,0,0,0,0,0,1,0]]C2984520	dual pi3 kinase mtor inhibitor gdc 0980	[[0,0,0,0,0,0,0,0,0,1,0]]C2984527	dlbs 1425,dlbs1425,phaleria macrocarpa extract dlbs 1425	[[0,1,0,0,0,0,0,0,0,0,0],[0,1,0,0,0,0,0,0,0,0,0],[0,0,0,0,0,0,0,0,0,1,0]]C1099664	fondaparinux sodium,decasodium salt	[[0,0,0,0,1,0,1,1,0,1,0],[0,0,0,0,1,0,0,0,0,0,0]]C2984525	technetium tc 99m glycopeptide,99mtc gp	[[0,0,0,0,0,0,0,0,0,1,0],[0,0,0,0,0,0,0,0,0,1,0]]C2984524	liposome incorporated grb2 antisense oligodeoxynucleotide,liposomal grb2 as odn	[[0,0,0,0,0,0,0,0,0,1,0],[0,0,0,0,0,0,0,0,0,1,0]]C0653365	4 oxatetradecanoic acid,3 decyloxy propanoic acid	[[0,1,0,0,0,0,0,0,0,0,0],[0,1,0,0,0,0,0,0,0,0,0]]C0653360	quinoprazine,4 4 4 ethylpiperazinyl 1 phenylamino benzo g quinoline	[[0,1,0,0,0,0,0,0,0,0,0],[0,1,0,0,0,0,0,0,0,0,0]]C0358677	amiloride thiazide diuretic	[[1,0,0,0,0,0,0,0,0,0,0]]C2975026	2 cyclopentyl n 2 4 dichloro 3 isoquinolin 5 yloxy methyl phenyl n methylacetamide	[[0,1,0,0,0,0,0,0,0,0,0]]C2975027	as 1708727,as1708727	[[0,1,0,0,0,0,0,0,0,0,0],[0,1,0,0,0,0,0,0,0,0,0]]C2975024	5 2 fluorophenyl spiro 1 azabicyclo 2 2 2 octane 3 2 3 h furo 2 3 b pyridine	[[0,1,0,0,0,0,0,0,0,0,0]]C0140405	rhenium v dimercaptosuccinic acid,re v dmsa,rhenium dmsa	[[0,1,0,0,0,0,0,0,0,0,0],[0,1,0,0,0,0,0,0,0,0,0],[0,1,0,0,0,0,0,0,0,0,0]]C1870443	1 4 6 bromobenzo 1 3 dioxol 5 yl 3a 4 5 9b tetrahydro 3h cyclopenta c quinolin 8 yl ethanone,g 1 cpd	[[0,1,0,0,0,0,0,0,0,0,0],[0,1,0,0,0,0,0,0,0,0,0]]C0598452	perfluorochemical emulsion	[[0,0,0,0,0,0,0,0,1,0,0]]C0598454	tetrahydrofolate antagonist	[[0,0,0,0,0,0,0,0,1,0,0]]C2827710	genkaxin	[[0,0,0,0,0,0,0,0,0,1,0]]C2827711	anti erbb2 anti erbb3 bispecific monoclonal antibody mm 111	[[0,0,0,0,0,0,0,0,0,1,0]]C0035064	renal agents,renal drug	[[1,1,0,0,0,0,1,0,0,0,0],[1,0,0,0,0,0,1,0,0,0,0]]C1975542	warfarin 124 urine	[[0,0,0,1,0,0,0,0,0,0,0]]C1975540	warfarin 124 bld ser plas	[[0,0,0,1,0,0,0,0,0,0,0]]C1975541	warfarin 124 gastric fluid	[[0,0,0,1,0,0,0,0,0,0,0]]C0065348	lymphosarcin	[[0,1,0,0,0,0,0,0,0,0,0]]C1531285	remitogen	[[0,0,0,0,0,0,0,0,0,1,0]]C3152977	niacinamide panthenol zinc pyrithione	[[0,0,0,0,0,0,0,1,0,0,0]]C2316254	topical form aluminum,topical form aluminium	[[1,0,0,0,0,0,0,0,0,0,0],[1,0,0,0,0,0,0,0,0,0,0]]C2000326	6 chloro n4 3 5 difluoro 4 3 methyl 1h pyrrolo 2 3 b pyridin 4 yl oxy phenyl pyrimidine 2 4 diamine,cdf mpp ppda cpd	[[0,1,0,0,0,0,0,0,0,0,0],[0,1,0,0,0,0,0,0,0,0,0]]C0145155	tenidap sodium	[[0,1,0,0,0,0,0,0,0,1,0]]C1981247	amilsulpride 124 bld ser plas	[[0,0,0,1,0,0,0,0,0,0,0]]C2701542	russian olive pollen extract,elaeagnus angustifolia pollen	[[0,0,0,0,0,0,0,1,0,0,0],[0,0,1,0,0,0,0,0,0,1,0]]C0912123	2 furaldehyde isonicotinoylhydrazone,2 furcho isonicotinoylhydrazone	[[0,1,0,0,0,0,0,0,0,0,0],[0,1,0,0,0,0,0,0,0,0,0]]C1711127	pngvl4a sig e7 detox hsp70 dna vaccine	[[0,0,0,0,1,0,0,0,0,1,0]]C1097547	cdb 4022,cdb4022	[[0,1,0,0,0,0,0,0,0,0,0],[0,1,0,0,0,0,0,0,0,0,0]]C0145158	teonicon	[[0,1,0,0,0,0,0,0,0,0,0]]C0381847	spiganthine,4 deoxy 20 hydroxyryanodine	[[0,1,0,0,0,0,0,0,0,0,0],[0,1,0,0,0,0,0,0,0,0,0]]C0058722	doxorubicin polyaspartic acid conjugate,dx paa	[[0,1,0,0,0,0,0,0,0,0,0],[0,1,0,0,0,0,0,0,0,0,0]]C0381840	1 hydroxyaleuritolic acid 3,1 hydroxyaleuritolic acid 3 p hydroxybenzoate,1 haa 3hb	[[0,1,0,0,0,0,0,0,0,0,0],[0,1,0,0,0,0,0,0,0,0,0],[0,1,0,0,0,0,0,0,0,0,0]]C0093516	2 nitrophenylhydrazonomesoxalic acid	[[0,1,0,0,0,0,0,0,0,0,0]]C0093513	2 nitroimidazole 1 acetohydroxamic acid,n hydroxy 2 nitro 1h imidazole 1 acetamide	[[0,1,0,0,0,0,0,0,0,0,0],[0,1,0,0,0,0,0,0,0,0,0]]C0093512	2 nitroimidazole benznidazole	[[0,1,0,0,0,0,0,0,0,0,0]]C0381849	mycalolide e	[[0,1,0,0,0,0,0,0,0,0,0]]C0381848	mycalolide d	[[0,1,0,0,0,0,0,0,0,0,0]]C0646618	5 methoxyethyl methoxyethoxy ethyl amino methyl thieno 2 3 b thiophene 2 sulfonamide,5 mmtts	[[0,1,0,0,0,0,0,0,0,0,0],[0,1,0,0,0,0,0,0,0,0,0]]C0646619	6 alpha d glucopyranosyl 3 azido 3 deoxy 5 thymidinyl phosphate	[[0,1,0,0,0,0,0,0,0,0,0]]C0058727	doxorubicin iron complex,iron adriamycin complex,doxorubicin iron iii complex,fe iii adriamycin complex	[[0,1,0,0,0,0,0,0,0,0,0],[0,1,0,0,0,0,0,0,0,0,0],[0,1,0,0,0,0,0,0,0,0,0],[0,1,0,0,0,0,0,0,0,0,0]]C0212685	1 pyrenemethyl 3 hydroxy 22 23 bisnor 5 cholenate,1 pmhbc	[[0,1,0,0,0,0,0,0,0,0,0],[0,1,0,0,0,0,0,0,0,0,0]]C0646611	aseanostatin p5,12 methyltetradecanoic acid,12 mta cpd	[[0,1,0,0,0,0,0,0,0,0,0],[0,1,0,0,0,0,0,0,0,0,0],[0,1,0,0,0,0,0,0,0,0,0]]C0646612	stipiamide,n 2 hydroxy 1 methylethyl 2 10 12 14 16 pentamethyl 18 phenyl 2 4 6 8 10 14 octadecahexaenamide	[[0,1,0,0,0,0,0,0,0,0,0],[0,1,0,0,0,0,0,0,0,0,0]]C0646614	3 4 dihydro 7 methoxy 2 methylene 3 oxo 2h 1 4 benzoxazine 5 carboxylic acid,degradation product ii f3	[[0,1,0,0,0,0,0,0,0,0,0],[0,1,0,0,0,0,0,0,0,0,0]]C0646616	dexamethasone 21 beta isothiocyanatoethyl thioether,dex ncs	[[0,1,0,0,0,0,0,0,0,0,0],[0,1,0,0,0,0,0,0,0,0,0]]C2701549	black cottonwood pollen extract,populus balsamifera ssp trichocarpa pollen,populus balsamifera subsp trichocarpa pollen	[[0,0,0,0,0,0,0,1,0,0,0],[0,0,1,0,0,0,0,0,0,0,0],[0,0,1,0,0,0,0,0,0,0,0]]C0970896	15beta isomer estratriene 2 3 14 15 16 17 hexaol	[[0,1,0,0,0,0,0,0,0,0,0]]C0717690	dextromethorphan phenylpropanolamine	[[0,0,0,0,0,0,0,1,0,0,0]]C0717693	dextromethorphan pseudoephedrine	[[1,0,0,0,0,0,0,1,0,0,1]]C0129826	n n bis 3 phenylmethyl amino propyl 1 7 diaminoheptane	[[0,1,0,0,0,0,0,0,0,0,0]]C0129821	n n bis 2 hydroxy 3 phenoxypropyl ethylenediamine	[[0,1,0,0,0,0,0,0,0,0,0]]C0129820	n n bis 2 hydoxybenzyl 1 4 bromoacetamidobenzyl 1 2 ethylenediamine n n diacetic acid,1 4 bromoacetamidobenzyl n n bis 2 hydroxybenzyl ethylenediamine n n diacetic acid,br phi hbed	[[0,1,0,0,0,0,0,0,0,0,0],[0,1,0,0,0,0,0,0,0,0,0],[0,1,0,0,0,0,0,0,0,0,0]]C0717697	dextromethorphan phenylephrine pyrilamine	[[0,0,0,0,0,0,0,1,0,0,0]]C0717696	dextromethorphan guaifenesin pseudoephedrine	[[1,0,0,0,0,0,0,1,0,0,1]]C0129828	n n bis 3 4 methoxyphenoxy 2 hydroxypropyl n n dimethylethylenediamine,n n bis 3 p methoxyphenoxy 2 hydroxypropyl n n dimethylethylenediamine	[[0,1,0,0,0,0,0,0,0,0,0],[0,1,0,0,0,0,0,0,0,0,0]]C0970898	sodium salt 4 sulfanilic acid	[[0,1,0,0,0,0,0,0,0,0,0]]C0970899	r r r isomer monosodium salt eritadenine	[[0,1,0,0,0,0,0,0,0,0,0]]C0971766	laherradurin	[[0,1,0,0,0,0,0,0,0,0,0]]C0070038	pantogab,gaba pantoate,pantoyl gaba	[[0,1,0,0,0,0,0,0,0,0,0],[0,1,0,0,0,0,0,0,0,0,0],[0,1,0,0,0,0,0,0,0,0,0]]C2743777	tianma xingnao	[[0,1,0,0,0,0,0,0,0,0,0]]C0070030	pansoral	[[0,1,0,0,0,0,0,0,0,0,0]]C1983212	buprenorphine 124 urine	[[0,0,0,1,0,0,0,0,0,0,0]]C0070034	pantethine,bis n pantothenylamidoethyl disulfide,d bis n pantothenyl 2 aminoethyl disulfide	[[0,0,0,0,0,0,0,1,0,1,0],[0,1,0,0,0,0,0,0,0,0,0],[0,0,0,0,0,0,0,0,0,1,0]]C2929407	ascorbic acid folic acid vitamin d	[[0,0,0,0,0,0,0,1,0,0,0]]C1832009	b raf inhibitor plx4032,rg7204,b raf inhibitor ro5185426,r05185426,braf v600e kinase inhibitor ro5185426	[[0,0,0,0,1,0,0,0,0,0,0],[0,0,0,0,0,0,0,0,0,1,0],[0,0,0,0,0,0,0,0,0,1,0],[0,1,0,0,0,0,0,0,0,0,0],[0,0,0,0,0,0,0,0,0,1,0]]C2730270	california mugwort pollen extract	[[0,0,0,0,0,0,0,1,0,0,0]]C2742830	ismigen	[[0,1,0,0,0,0,0,0,0,0,0]]C0733918	ergodesit	[[0,1,0,0,0,0,0,0,0,0,0]]C0733919	d 40ta,d40ta	[[0,1,0,0,0,0,0,0,0,0,0],[0,1,0,0,0,0,0,0,0,0,0]]C0887169	meclizine monohydrochloride	[[0,1,0,0,0,0,0,0,0,0,0]]C0887168	meclizine dihydrochloride	[[0,1,0,0,0,0,0,0,0,0,0]]C0776030	bentonite powder	[[1,0,0,0,0,0,0,0,0,0,0]]C2743776	suxiao jiuxinwan	[[0,1,0,0,0,0,0,0,0,0,0]]C0132496	nih 10647	[[0,1,0,0,0,0,0,0,0,0,0]]C0132497	nik 228,2 methyl imidazo 2 1 b benzothiazole 3 methanol	[[0,1,0,0,0,0,0,0,0,0,0],[0,1,0,0,0,0,0,0,0,0,0]]C1256232	bach flower essence agrimony	[[0,0,0,0,0,1,0,0,0,0,0]]C0132499	nik 247,nik247	[[0,1,0,0,0,0,0,0,0,0,0],[0,1,0,0,0,0,0,0,0,0,0]]C0733912	fimomed	[[0,1,0,0,0,0,0,0,0,0,0]]C0733913	kanokonlit	[[0,1,0,0,0,0,0,0,0,0,0]]C0733914	ligament fimomed	[[0,1,0,0,0,0,0,0,0,0,0]]C0733915	circanol	[[0,1,0,0,0,0,0,0,0,0,0]]C0593563	clotam,provalis brand of tolfenamic acid	[[0,1,0,0,0,0,0,0,0,0,0],[0,1,0,0,0,0,0,0,0,0,0]]C0733917	redergin	[[0,1,0,0,0,0,0,0,0,0,0]]C1986795	dipyrone 124 bld ser plas	[[0,0,0,1,0,0,0,0,0,0,0]]C1986794	dipyridamole 124 xxx	[[0,0,0,1,0,0,0,0,0,0,0]]C1986796	dipyrone 124 white blood cells	[[0,0,0,1,0,0,0,0,0,0,0]]C1986793	dipyridamole 124 bld ser plas	[[0,0,0,1,0,0,0,0,0,0,0]]C1986792	dipipanone 124 urine	[[0,0,0,1,0,0,0,0,0,0,0]]C3255965	peg 12 dilaurate	[[0,0,1,0,0,0,0,0,0,0,0]]C0949423	7r 7alpha 7aalpha 14alpha 14abeta isomer sparteine hydroiodide	[[0,1,0,0,0,0,0,0,0,0,0]]C0949422	7s 7alpha 7aalpha 14alpha 14abeta isomer sparteine hydrochloride	[[0,1,0,0,0,0,0,0,0,0,0]]C1742965	veliflapon	[[0,1,0,0,0,0,0,0,0,0,0]]C0209259	rs 26306	[[0,1,0,0,0,0,0,0,0,0,0]]C0026251	mitoguazone,methylglyoxal bis,methylglyoxal bis guanylhydrazone,mgbg,2 2 1 methyl 1 2 ethanediylidene bis hydrazinecarboximidamide,methyl g,mga,mggh,guanylhydrazone,1 1 methylethanediylidene dinitrilo diguanidine,2 2 methyl 1 2 ethanediylidene bis,pyruvaldehyde bis,methylglyoxal bisguanylhydrazone,me gag	[[0,1,0,0,0,0,0,0,1,1,0],[0,0,0,0,1,0,0,0,0,1,1],[0,0,0,0,0,0,0,0,1,0,0],[0,0,0,0,0,0,0,0,0,0,1],[0,0,0,0,0,0,0,0,0,0,1],[0,0,0,0,0,0,0,0,0,1,0],[0,0,0,0,0,0,1,0,0,0,0],[0,0,0,0,1,0,0,0,0,0,0],[0,0,0,0,0,0,0,0,1,0,0],[0,0,0,0,1,0,0,0,0,0,0],[0,0,0,0,1,0,0,0,0,0,0],[0,0,0,0,0,0,0,0,0,1,0],[0,0,0,0,0,0,0,0,0,1,0],[0,0,0,0,0,0,0,0,0,1,0]]C0026252	mitolactol,dibromodulcitol,dbd,1 6 dibromo 1 6 dideoxy galactitol,6 dibromodideoxydulcitol,6 dibromodulcitol,dibromdicil	[[0,0,0,0,0,0,0,0,0,1,0],[0,0,0,0,0,0,1,0,0,0,1],[0,0,0,0,1,0,0,0,0,0,0],[0,0,0,0,0,0,0,0,0,0,1],[0,0,0,0,1,0,0,0,0,1,0],[0,0,0,0,1,0,0,0,0,1,0],[0,0,0,0,0,0,0,0,0,1,0]]C0026254	mitomycins,mitomycin antineoplastic antibiotic	[[0,1,0,0,0,0,0,0,1,0,0],[0,0,0,0,0,0,0,0,0,1,0]]C0108454	carboxyimamidate,carbethimer,carbetimer,carboxyaminidate,polima	[[0,1,0,0,0,0,0,0,0,0,0],[0,1,0,0,0,0,0,0,0,1,0],[0,0,0,0,1,0,0,0,0,1,0],[0,0,0,0,0,0,0,0,0,1,0],[0,0,0,0,1,0,0,0,0,0,0]]C0026256	mitotane,mytotan,1 1 dichloro 2 o chlorophenyl 2 p chlorophenyl ethane,ddd,ortho para ddd,o p ddd,1 chloro 2 2 2 dichloro 1 4 chlorophenyl ethyl benzene,op ddd mitotane,2 2 bis 2 chlorophenyl 4 chlorophenyl 1 1 dichloroethane,2 2 chlorophenyl 2 4 chlorophenyl 1 1 dichloroethane,2 4 dichlorodiphenyldichloroethane	[[0,1,0,0,0,0,0,0,0,1,0],[0,0,0,0,1,0,0,0,0,0,0],[1,0,0,0,1,0,0,0,0,1,0],[0,0,0,0,1,0,0,0,0,0,0],[0,1,0,0,0,0,0,0,0,1,1],[1,0,0,0,1,0,0,0,0,1,1],[0,0,0,0,0,0,0,0,0,1,1],[1,0,0,0,0,0,0,0,0,0,0],[0,0,0,0,1,0,0,0,0,1,0],[0,0,0,0,1,0,0,0,0,0,0],[0,0,0,0,1,0,0,0,0,1,0]]C0026259	mitoxantrone,dhaq,mitozantrone,1 4 dihydroxy 5 8 bis 2 2 hydroxyethyl amino ethyl amino 9 10 anthracenedione,dihydroxyanthracenedione,mitoxantrone product,mitozantrone product,dhad,10 anthracenedione 1 3 dihydroxy 5 8 bis 2 2 hydroxyethyl amino ethyl amino 9,10 anthroquinone 1 4 dihydroxy 5 8 bis 2 2 hydroxyethyl amino ethyl amino 9	[[0,1,0,0,0,0,0,1,1,1,0],[0,0,0,0,0,0,0,0,0,1,0],[0,0,0,0,1,0,1,0,0,0,0],[0,0,0,0,0,0,0,0,0,0,1],[0,0,0,0,1,0,0,0,0,1,0],[1,0,0,0,0,0,0,0,0,0,0],[1,0,0,0,0,0,0,0,0,0,0],[0,0,0,0,1,0,0,0,0,0,0],[0,0,0,0,1,0,0,0,0,0,0],[0,0,0,0,1,0,0,0,0,0,0]]C0067353	n n diethyl 2 1 pyridyl ethylamine,e 2 p,hydrobromide bromide 1 2 diethylamino ethyl pyridinium	[[0,1,0,0,0,0,0,0,0,0,0],[0,1,0,0,0,0,0,0,0,0,0],[0,1,0,0,0,0,0,0,0,0,0]]C1530053	esteclin,lakeside brand of erdosteine	[[0,1,0,0,0,0,0,0,0,0,0],[0,1,0,0,0,0,0,0,0,0,0]]C0100200	8 chloro tetrahydroisoquinoline 7 sulfonamide,8 chloro 1 2 3 4 tetrahydro 7 isoquinolinesulfonamide	[[0,1,0,0,0,0,0,0,0,0,0],[0,1,0,0,0,0,0,0,0,0,0]]C0062182	heliosit	[[0,1,0,0,0,0,0,0,0,0,0]]C0977381	hemorrhoidal rectal suppositories	[[0,0,0,0,0,0,0,1,0,0,0]]C0631257	rs 37326	[[0,1,0,0,0,0,0,0,0,0,0]]C2344194	zoderm 8 5	[[0,0,0,0,0,0,0,1,0,0,0]]C0604861	tirpate	[[0,1,0,0,0,0,0,0,0,0,0]]C0604860	2 4 dimethyl 1 3 dithiolane 2 carboxaldehyde o methylcarbamoyl oxime	[[0,1,0,0,0,0,0,0,0,0,0]]C3244602	neosporin eczema	[[0,0,0,0,0,0,0,1,0,0,0]]C0971535	tco cf compound	[[0,1,0,0,0,0,0,0,0,0,0]]C1528958	pamoxan,uriach brand of pyrvinium pamoate	[[0,1,0,0,0,0,0,0,0,0,0],[0,1,0,0,0,0,0,0,0,0,0]]C1434511	2 2 4 5 dihydro 1h imidazol 2 yl 1 5 methyl 2 3 dihydrobenzofuran 7 yl ethyl pyridine	[[0,1,0,0,0,0,0,0,0,0,0]]C1121459	methylthiogliotoxin	[[0,1,0,0,0,0,0,0,0,0,0]]C0025503	mesterolone,1alpha 5alpha 17beta 17 hydroxy 1 methyl androstan 3 one,mesterolone preparation,1 alpha 5 alpha 17 beta 17 hydroxy 1 methyl androstan 3 one,17 beta hydroxy 1 alpha methyl 5 alpha androstan 3 one	[[0,0,0,0,0,0,0,1,0,1,0],[0,0,0,0,0,0,0,0,0,0,1],[1,0,0,0,0,0,0,0,0,0,0],[0,0,0,0,0,0,0,0,0,1,0],[0,0,0,0,0,0,0,0,0,1,0]]C0025506	mestranol,ethinyl estradiol 3 methyl ether,17alpha 3 methoxy 19 norpregna 1 3 5 10 trien 20 yn 17 ol,mestranol preparation,mestranol product	[[0,0,0,0,0,0,1,0,0,1,0],[0,1,0,0,0,0,0,0,0,0,1],[0,0,0,0,0,0,0,0,0,1,1],[1,0,0,0,0,0,0,0,0,0,0],[1,0,0,0,0,0,0,0,0,0,0]]C1589304	catulac	[[0,0,0,0,0,0,0,1,0,0,0]]C0730647	9a brand of intermediate factor ix	[[0,0,0,0,0,1,0,0,0,0,0]]C0972237	1 4 aminophenyl methyl 3 3 nitrophenyl 1 3 dihydroimidazo 4 5 b pyridine 2 one	[[0,1,0,0,0,0,0,0,0,0,0]]C0250020	nsc d640430	[[0,1,0,0,0,0,0,0,0,0,0]]C0250021	bisnafide,dimethanesulfonate r r r 2 2 1 2 ethanediylbis imino 1 methyl 2 1 ethanediyl bis 5 nitro 1h benz de isoquinoline 1 3 2h dione	[[0,1,0,0,0,0,0,0,0,1,0],[0,1,0,0,0,0,0,0,0,0,0]]C0537707	n n bis salicyl hydrazine,n n bis salicylhydrazine,n n disalicylhydrazine	[[0,1,0,0,0,0,0,0,0,0,0],[0,1,0,0,0,0,0,0,0,0,0],[0,1,0,0,0,0,0,0,0,0,0]]C0298992	violuric acid	[[0,1,0,0,0,0,0,0,0,0,0]]C0014958	etazolate,ethyl ester 1 ethyl 4 1 methylethylidene hydrazino 1h pyrazolo 3 4 b pyridine 5 carboxylic acid	[[0,0,0,0,0,0,0,0,0,1,0],[0,0,0,0,0,0,0,0,0,0,1]]C3248039	erwinaze	[[0,0,0,0,0,0,0,1,0,0,0]]C1881281	isotiquimide	[[0,0,0,0,0,0,0,0,0,1,0]]C2933981	2 n n diethylaminocarbonyloxymethyl 1 diphenylmethyl 4 3 4 5 trimethoxybenzoyl piperazine	[[0,1,0,0,0,0,0,0,0,0,0]]C0066068	methabenzthiazuron,n 2 benzothiazolyl n n dimethylurea	[[0,1,0,0,0,0,0,0,0,0,0],[0,1,0,0,0,0,0,0,0,0,0]]C0066067	metet,7 beta methylthioethyltheophylline	[[0,1,0,0,0,0,0,0,0,0,0],[0,1,0,0,0,0,0,0,0,0,0]]C0066066	metepa,tris 2 methyl 1 aziridinyl phosphine oxide	[[0,1,0,0,0,0,0,0,0,0,0],[0,1,0,0,0,0,0,0,0,0,0]]C0066064	metbufen,3 4 biphenylylcarbonyl 2 methylpropionic acid	[[0,1,0,0,0,0,0,0,0,1,0],[0,1,0,0,0,0,0,0,0,0,0]]C0066063	metazosin,2 4 alpha methoxypropionyl 1 piperazinyl 4 amino 6 7 dimethoxychinazoline	[[0,1,0,0,0,0,0,0,0,0,0],[0,1,0,0,0,0,0,0,0,0,0]]C0066062	metazocine,1 2 3 4 5 6 hexahydro 3 6 11 trimethyl 2 6 methano 3 benzazocin 8 ol	[[1,1,0,0,0,0,0,0,0,0,0],[0,1,0,0,0,0,0,0,0,0,0]]C0066061	metaupon	[[0,1,0,0,0,0,0,0,0,0,0]]C0631258	anirolac,2 3 dihydro 5 4 methoxybenzoyl 1h pyrrolizine 1 carboxylic acid	[[0,1,0,0,0,0,0,0,0,1,0],[0,1,0,0,0,0,0,0,0,0,0]]C0252887	jen sheng yang yung tang,jsyyt	[[0,1,0,0,0,0,0,0,0,0,0],[0,1,0,0,0,0,0,0,0,0,0]]C1517613	n 5 tert butyl 3 isoxazolyl n 4 4 pyridinyl oxyphenyl urea	[[0,0,0,0,0,0,0,0,0,1,0]]C0309311	veterinary brand name drug integrator	[[0,0,0,0,0,1,0,0,0,0,0]]C0631773	chromozym u,benzoyl beta alanyl glycyl arginine 4 nitroanilide acetate	[[0,1,0,0,0,0,0,0,0,0,0],[0,1,0,0,0,0,0,0,0,0,0]]C0630428	3 4 dinitrofluoranthene,3 4 dinitro fluoranthene	[[0,1,0,0,0,0,0,0,0,0,0],[0,1,0,0,0,0,0,0,0,0,0]]C2968645	heparin 10000 u ml 124 patient	[[0,0,0,1,0,0,0,0,0,0,0]]C0630427	n 2 dansylaminoethyl amphetamine,2 dns amphetamine	[[0,1,0,0,0,0,0,0,0,0,0],[0,1,0,0,0,0,0,0,0,0,0]]C2983975	atiratecan,9s 9 ethyl 10 13 dioxo 1 pentyl 9 10 13 15 tetrahydro 1h 12hpyrano 3 4 6 7 indolizino 2 1 5 6 pyrido 4 3 2 de quinazolin 9 yl glycyl n methylglycinate	[[0,0,0,0,0,0,0,0,0,1,0],[0,0,0,0,0,0,0,0,0,1,0]]C0630425	2 dansylaminoethyl n acetylgalactosamine,2 dansylaminoethyl alpha n acetylgalactosaminide,2 dnsgalnac	[[0,1,0,0,0,0,0,0,0,0,0],[0,1,0,0,0,0,0,0,0,0,0],[0,1,0,0,0,0,0,0,0,0,0]]C1429394	sacrophaga peregrina sarcotoxin iic protein	[[0,1,0,0,0,0,0,0,0,0,0]]C0667850	naproxen lysine,naproxen lys	[[0,1,0,0,0,0,0,0,0,0,0],[0,1,0,0,0,0,0,0,0,0,0]]C1429393	sarcophaga peregrina sarcotoxin iib protein	[[0,1,0,0,0,0,0,0,0,0,0]]C0063850	iremycin,7r trans 8 ethyl 7 8 9 10 tetrahydro 1 6 8 11 tetrahydroxy 7 2 3 6 trideoxy 3 dimethylamino alpha l lyxo hexopyranosyl oxy 5 12 naphthacenedione	[[0,1,0,0,0,0,0,0,0,0,0],[0,1,0,0,0,0,0,0,0,0,0]]C0063853	irganox 1076,3 5 bis 1 1 dimethylethyl 4 hydroxybenzenepropanoic acid octadecyl ester	[[0,1,0,0,0,0,0,0,0,0,0],[0,1,0,0,0,0,0,0,0,0,0]]C2983974	undecoylium chloride	[[0,0,0,0,0,0,0,0,0,1,0]]C0614745	tylophorinidine	[[0,1,0,0,0,0,0,0,0,0,0]]C2936063	4 oxo 4 5 6 7 tetrahydro 1h indole 3 carboxylic acid 4 methylaminomethylphenyl amide	[[0,1,0,0,0,0,0,0,0,0,0]]C0147173	tosylate 1 4 salt alpha beta gamma delta tetrakis 4 n trimethylaminophenyl porphine,ttmapp	[[0,1,0,0,0,0,0,0,0,0,0],[0,1,0,0,0,0,0,0,0,0,0]]C1453097	s 137 compound,s137 compound	[[0,1,0,0,0,0,0,0,0,0,0],[0,1,0,0,0,0,0,0,0,0,0]]C1517611	roaccutan	[[0,0,0,0,1,0,0,0,0,0,0]]C0063392	imidazopyrazole,1h imidazo 1 2 b pyrazole	[[0,1,0,0,0,0,0,0,0,0,0],[0,1,0,0,0,0,0,0,0,0,0]]C2356612	evovist	[[0,0,0,0,0,0,0,1,0,0,0]]C0593953	twinrix	[[0,0,0,0,0,0,1,1,0,0,0]]C1869885	n methyl 1 deoxynojirimycin 6 phosphate	[[0,1,0,0,0,0,0,0,0,0,0]]C1869887	artemether lumefantrine combination,artemether benflumetol combination	[[0,1,0,0,0,0,0,0,0,0,0],[0,1,0,0,0,0,0,0,0,0,0]]C0982199	hydrastis preparation,hydrastis	[[0,0,0,0,0,0,0,1,0,0,0],[0,0,0,0,0,0,1,0,0,0,0]]C1869889	artesunate drug combination amodiaquine,artesunate amodiaquine combination,asaq compound,amodiaquine artesunate	[[0,1,0,0,0,0,0,0,0,0,0],[0,1,0,0,0,0,0,0,0,0,0],[0,1,0,0,0,0,0,0,0,0,0],[0,1,0,0,0,0,0,0,0,0,0]]C1869888	coartem	[[0,1,0,0,0,0,0,0,0,0,0]]C2936062	1 6 fluoro 2 methyl 3 4 dihydroquinolin 1 2h yl 2 isoquinolin 5 yloxy ethanone,1 fmd 2 iqo ethanone	[[0,1,0,0,0,0,0,0,0,0,0],[0,1,0,0,0,0,0,0,0,0,0]]C0288653	isochromophilone iia	[[0,1,0,0,0,0,0,0,0,0,0]]C1564467	tersaseptic,trans canaderm brand of triclosan	[[0,1,0,0,0,0,0,0,0,0,0],[0,1,0,0,0,0,0,0,0,0,0]]C0626036	5 2 bromoethyl 2 deoxyuridine,beudr	[[0,1,0,0,0,0,0,0,0,0,0],[0,1,0,0,0,0,0,0,0,0,0]]C1564465	microshield t,johnson johnson brand of triclosan	[[0,1,0,0,0,0,0,0,0,0,0],[0,1,0,0,0,0,0,0,0,0,0]]C1564464	clearasil daily face wash,procter gamble brand of triclosan	[[0,1,0,0,0,0,0,0,0,0,0],[0,1,0,0,0,0,0,0,0,0,0]]C1564463	trilam,gerard brand of triazolam	[[0,1,0,0,0,0,0,0,0,0,0],[0,1,0,0,0,0,0,0,0,0,0]]C1564462	gen triazolam,genpharm brand of triazolam	[[0,1,0,0,0,0,0,0,0,0,0],[0,1,0,0,0,0,0,0,0,0,0]]C1564461	apo triazo,apotex brand of triazolam	[[0,1,0,0,0,0,0,0,0,0,0],[0,1,0,0,0,0,0,0,0,0,0]]C1564460	urocaudal,jorba brand of triamterene	[[0,1,0,0,0,0,0,0,0,0,0],[0,1,0,0,0,0,0,0,0,0,0]]C0246631	remifentanil,3 4 methoxycarbonyl 4 1 oxopropyl phenylamino 1 piperidine propanoic acid methyl ester,methyl ester 4 methoxy carbonyl 4 1 oxopropyl phenylamino 1 piperidinepropanoic acid,remifentanyl	[[0,0,0,0,0,0,0,1,0,1,0],[0,0,0,0,0,0,0,0,0,0,1],[0,0,0,0,0,0,0,0,0,0,1],[1,0,0,0,0,0,0,0,0,0,0]]C1564469	tft ophtiole,tramedico brand of trifluridine	[[0,1,0,0,0,0,0,0,0,0,0],[0,1,0,0,0,0,0,0,0,0,0]]C1564468	trisan,dermtek brand of triclosan	[[0,1,0,0,0,0,0,0,0,0,0],[0,1,0,0,0,0,0,0,0,0,0]]C1815281	carbetapentane diphenhydramine	[[0,0,0,0,0,0,0,1,0,0,0]]C0095175	3 aminopropylphosphinic acid,3 aminopropanephosphinic acid,3 aminopropylphosphonous acid,phosphinic acid	[[0,1,0,0,0,0,0,0,0,0,0],[0,1,0,0,0,0,0,0,0,0,0],[0,1,0,0,0,0,0,0,0,0,0],[0,1,0,0,0,0,0,0,0,0,0]]C0095174	3 aminopropyl n methylisothiourea,dihydrochloride 3 aminopropyl ester methyl carbamimidothioic acid	[[0,1,0,0,0,0,0,0,0,0,0],[0,1,0,0,0,0,0,0,0,0,0]]C0770472	protokylol hydrochloride	[[1,1,0,0,0,0,0,0,0,1,0]]C0051330	alpha benzyl n methylphenethylamine,abnmp	[[0,1,0,0,0,0,0,0,0,0,0],[0,1,0,0,0,0,0,0,0,0,0]]C0095171	3 aminopropyl hexyl phosphinic acid,3 aminopropyl n hexyl phosphinic acid,3 aphpa	[[0,1,0,0,0,0,0,0,0,0,0],[0,1,0,0,0,0,0,0,0,0,0],[0,1,0,0,0,0,0,0,0,0,0]]C0051332	alpha bromo beta 5 imidazolyl propionic acid	[[0,1,0,0,0,0,0,0,0,0,0]]C0619045	bzc jelly,mixt with benzenemethanol and ethyl 4 aminobenzoate chloride n n dimethyl n 2 2 4 1 1 3 3 tetramethylbutyl phenoxy ethoxy ethyl benzenemethanaminium	[[0,1,0,0,0,0,0,0,0,0,0],[0,1,0,0,0,0,0,0,0,0,0]]C0619044	n carbobenzoxyglycyl prolyl argininamide 9 ethylcarbazole,n phenylmethoxy carbonyl glycyl l prolyl n 9 ethyl 9h carbazol 3 yl l argininamide,benzyloxycarbonylglycyl prolyl arginine 3 amino 9 ethylcarbazole,cbz gly pro arg aec	[[0,1,0,0,0,0,0,0,0,0,0],[0,1,0,0,0,0,0,0,0,0,0],[0,1,0,0,0,0,0,0,0,0,0],[0,1,0,0,0,0,0,0,0,0,0]]C0619047	ami mixture	[[0,1,0,0,0,0,0,0,0,0,0]]C0619046	physcion diglucoside,1 6 o beta d glucopyranosyl beta d glucopyranosyl oxy 8 hydroxy 3 methoxy 6 methyl 9 10 anthracenedione	[[0,1,0,0,0,0,0,0,0,0,0],[0,1,0,0,0,0,0,0,0,0,0]]C0619041	1 phenyluracil	[[0,1,0,0,0,0,0,0,0,0,0]]C1100482	1 phenyl 4 4 bis 2 4 dichlorobenzoyloxymethyl 1 2 3 triazole,dichlotazole	[[0,1,0,0,0,0,0,0,0,0,0],[0,1,0,0,0,0,0,0,0,0,0]]C1100480	2 4 diamino 6 fluromethyl 5 2 3 5 trichlorophenyl pyrimidine	[[0,1,0,0,0,0,0,0,0,0,0]]C2975857	14 desoxo 3beta 5alpha 7beta 10 15beta o pentaacetyl 14alpha o benzoyl 10 18 dihydromyrinsol,dpbenzoyld cpd	[[0,1,0,0,0,0,0,0,0,0,0],[0,1,0,0,0,0,0,0,0,0,0]]C1100577	4 6 diamino 8h 1 hydroxyethoxymethyl 8 iminoimidazo 4 5 e 1 3 diazepine,4 6 a h ida	[[0,1,0,0,0,0,0,0,0,0,0],[0,1,0,0,0,0,0,0,0,0,0]]C1726021	ionsys	[[0,0,0,0,0,0,0,0,0,1,0]]C2975858	14 desoxo 3alpha 5alpha 7beta 10 15beta o pentaacetyl 14beta o butanoyl 10 18 dihydromyrinsol,dpbutanoyld cpd	[[0,1,0,0,0,0,0,0,0,0,0],[0,1,0,0,0,0,0,0,0,0,0]]C1454603	5 amino 5 deoxyaristeromycin	[[0,1,0,0,0,0,0,0,0,0,0]]C2983971	pirralkonium	[[0,0,0,0,0,0,0,0,0,1,0]]C2917919	maxiflu	[[0,0,0,0,0,0,0,1,0,0,0]]C1992486	methyl ethyl ketone 124 bld ser plas	[[0,0,0,1,0,0,0,0,0,0,0]]C1992487	methyl ethyl ketone 124 body fluid	[[0,0,0,1,0,0,0,0,0,0,0]]C1992488	methyl ethyl ketone 124 urine	[[0,0,0,1,0,0,0,0,0,0,0]]C1992489	methyl formamide 124 urine	[[0,0,0,1,0,0,0,0,0,0,0]]C2978393	calcium carbonate ergocalciferol soy protein isolate	[[0,0,0,0,0,0,0,1,0,0,0]]C0166511	3 2 4 4 fluorobenzoyl piperidin 1 yl ethyl 6 7 8 9 tetrahydro 2h pyrido 1 2 a 1 3 5 triazine 2 4 3h dione maleate	[[0,1,0,0,0,0,0,0,0,0,0]]C0166512	dv 7028,z 2 butenedioate 1 1 3 2 4 4 fluorobenzoyl 1 piperidinyl ethyl 6 7 8 9 tetrahydro 2h pyrido 1 2 a 1 3 5 triazine 2 4 3h dione	[[0,1,0,0,0,0,0,0,0,0,0],[0,1,0,0,0,0,0,0,0,0,0]]C2978390	black pepper preparation guggul lipids	[[0,0,0,0,0,0,0,1,0,0,0]]C0376816	perfluoropentane,dodecafluoropentane,perfluoropenthane	[[0,1,0,0,0,0,0,0,0,1,0],[0,1,0,0,0,0,0,0,0,1,0],[1,0,0,0,0,0,0,0,0,0,0]]C1948387	janumet	[[0,0,0,0,0,0,0,1,0,0,0]]C2698502	allegron	[[0,0,0,0,0,0,0,0,0,1,0]]C1948383	nitromist	[[0,0,0,0,0,0,0,1,0,0,0]]C0729224	celontin,warner lambert brand of methsuximide,pfizer brand of methsuximide	[[0,0,0,0,0,0,1,0,0,1,0],[0,1,0,0,0,0,0,0,0,0,0],[0,1,0,0,0,0,0,0,0,0,0]]C0729227	calcamine	[[0,1,0,0,0,0,0,0,0,0,0]]C0729226	enzactin	[[0,1,0,0,0,0,0,0,0,0,0]]C0538045	d2916,d 2916	[[0,1,0,0,0,0,0,0,0,0,0],[0,1,0,0,0,0,0,0,0,0,0]]C0538044	2 6 dimethylbenzamide n,soretolide	[[0,1,0,0,0,0,0,0,0,0,0],[0,1,0,0,0,0,0,0,0,0,0]]C0538047	d2624,d 2624	[[0,1,0,0,0,0,0,0,0,0,0],[0,1,0,0,0,0,0,0,0,0,0]]C0622476	bw 458c,dihydrochloride n n bis 4 4 5 dihydro 4 methyl 1h imidazol 2 yl phenyl 1 4 benzenedicarboxamide	[[0,1,0,0,0,0,0,0,0,0,0],[0,1,0,0,0,0,0,0,0,0,0]]C1445169	protamine extract	[[1,0,0,0,0,0,0,0,0,0,0]]C0622474	wander 2159	[[0,1,0,0,0,0,0,0,0,0,0]]C0622475	wander 2495	[[0,1,0,0,0,0,0,0,0,0,0]]C1445164	insulin bovine extract	[[1,0,0,0,0,0,0,0,0,0,0]]C1445165	insulin human extract	[[1,0,0,0,0,0,0,0,0,0,0]]C1445166	insulin porcine extract	[[1,0,0,0,0,0,0,0,0,0,0]]C1445167	penicilloyl g extract	[[1,0,0,0,0,0,0,0,0,0,0]]C1445160	ampicilloyl extract	[[1,0,0,0,0,0,0,0,0,0,0]]C0538048	n 2 6 dimethylphenyl 5 methyl 3 isoxazolecarboxamide	[[0,1,0,0,0,0,0,0,0,0,0]]C1445162	chymopapain extract	[[1,0,0,0,0,0,0,0,0,0,0]]C1445163	gelatin bovine extract	[[1,0,0,0,0,0,0,0,0,0,0]]C0908925	pyrisulfoxin a	[[0,1,0,0,0,0,0,0,0,0,0]]C0077359	n epsilon bromoacetyllysine trna,n 6 bromoacetyl lys trna,n epsilon bromoacetyl lys trna,n epsilon bromoacetyllysyl trna,nba lys trna	[[0,1,0,0,0,0,0,0,0,0,0],[0,1,0,0,0,0,0,0,0,0,0],[0,1,0,0,0,0,0,0,0,0,0],[0,1,0,0,0,0,0,0,0,0,0],[0,1,0,0,0,0,0,0,0,0,0]]C1831902	polyvinylpyrrolidone sodium hyaluronate gel	[[0,0,0,0,0,0,0,0,0,1,0]]C0641563	ty 10957,1alpha 1e 3r 2beta 3aalpha 7aalpha 2 2 3 3a 6 7 7a hexahydro 2 hydroxy 1 3 hydroxy 4 methyl 4 phenoxy 1 pentenyl 1h inden 5 yl ethoxy acetic acid	[[0,1,0,0,0,0,0,0,0,0,0],[0,1,0,0,0,0,0,0,0,0,0]]C0700603	betamethasone sodium phosphate,betamethasone 21 phosphate,betamethasone phosphate	[[1,0,0,0,0,0,1,1,0,1,1],[0,0,0,0,0,0,0,0,0,0,1],[0,0,0,0,0,0,0,0,0,0,1]]C0641561	3 3 oxa 4 carboxybutyl 7 exo 3 hydroxy 4 methyl 4 phenoxy 1 pentenyl 8 endo hydroxybicyclo 4 3 0 non 2 ene	[[0,1,0,0,0,0,0,0,0,0,0]]C0700606	esmolol hydrochloride,esmolol hydrochloride product,4 2 hydroxy 3 1 methylethyl amino propoxy benzenepropanoic acid methyl ester hcl,methyl p 2 hydroxy 3 isopropylamino propoxy hydrocinnamate hydrochloride	[[0,1,0,0,0,0,0,1,0,1,0],[1,0,0,0,0,0,0,0,0,0,0],[0,0,0,0,0,0,0,0,0,1,0],[0,0,0,0,0,0,0,0,0,1,0]]C0700607	digoxin antibodies fab fragments,digoxin immune fab,digoxin specific fab antibody fragments,digoxin specific antibody,antidigoxin fab fragments,digoxin specific antigen binding fragments,ovine digoxin immune fab	[[0,1,0,0,0,0,0,0,0,0,0],[1,0,0,0,0,0,0,0,0,0,1],[1,0,0,0,0,0,0,0,0,0,0],[1,0,0,0,0,0,0,0,0,0,0],[1,0,0,0,0,0,0,0,0,0,0],[1,0,0,0,0,0,0,0,0,0,0],[0,0,1,0,0,0,0,0,0,1,0]]C0700605	desogen	[[0,0,0,0,0,0,1,1,0,0,0]]C1831907	pr 104,pr104	[[0,0,0,0,1,0,0,0,0,0,0],[0,0,0,0,0,0,0,0,0,1,0]]C1120770	etimicin	[[0,1,0,0,0,0,0,0,0,0,0]]C2912247	poly iron 150 forte	[[0,0,0,0,0,0,0,1,0,0,0]]C1307716	oryctolagus cuniculus rk 1 protein	[[0,1,0,0,0,0,0,0,0,0,0]]C1100223	1 oxocryptotanshinone	[[0,1,0,0,0,0,0,0,0,0,0]]C1451387	aescuflavoside a	[[0,1,0,0,0,0,0,0,0,0,0]]C1451386	aescuflavoside	[[0,1,0,0,0,0,0,0,0,0,0]]C1451381	causticum	[[0,1,1,0,0,0,0,0,0,0,0]]C1565336	drf 2519	[[0,1,0,0,0,0,0,0,0,0,0]]C1100224	1 oxomiltirone	[[0,1,0,0,0,0,0,0,0,0,0]]C0028277	nomifensine,linamiphen,1 2 3 4 tetrahydro 2 methyl 4 phenyl 8 isoquinolinamine	[[0,0,0,0,0,0,0,0,0,1,1],[0,0,0,0,0,0,0,0,0,0,1],[0,0,0,0,0,0,0,0,0,0,1]]C0955570	beta isomer monosodium salt n lauryl iminodipropionate	[[0,1,0,0,0,0,0,0,0,0,0]]C0955571	beta isomer sodium salt n lauryl iminodipropionate	[[0,1,0,0,0,0,0,0,0,0,0]]C0955572	disodium salt n lauryl iminodipropionate	[[0,1,0,0,0,0,0,0,0,0,0]]C0028278	merital	[[0,1,0,0,0,0,1,0,0,0,0]]C0050066	8 chlorocarbochromen,8 chloro 3 beta diethylaminoethyl 4 methyl 7 ethyoxycarbonymethoxycoumarin,8 monochloro 3 beta diethylaminoethyl 4 methyl 7 ethoxycarboxylmethoxycoumarin,ethyl ester 8 chloro 3 2 diethylamino ethyl 4 methyl 2 oxo 2h 1 benzopyran 7 yl oxy acetic acid,cloricromen	[[0,1,0,0,0,0,0,0,0,0,0],[0,1,0,0,0,0,0,0,0,0,0],[0,1,0,0,0,0,0,0,0,0,0],[0,1,0,0,0,0,0,0,0,0,0],[0,1,0,0,0,0,0,0,0,1,0]]C0771783	orotic acid monohydrate,1 2 3 6 tetrahydro 2 6 dioxo 4 pyrimidinecarboxylic acid monohydrate,uracil 6 carboxylic acid monohydrate,1 2 3 4 tetrahydro 2 6 dioxopyrimidine 4 carboxylic acid monohydrate	[[0,0,0,0,0,0,0,0,0,1,0],[0,0,0,0,0,0,0,0,0,1,0],[0,0,0,0,0,0,0,0,0,1,0],[0,0,0,0,0,0,0,0,0,1,0]]C0050063	8 chloro cyclic adenosine monophosphate,camp,8 chloro camp,8 chloro cyclic amp,8 chloroadenosine 3 5 cyclic monophosphate,8 chloroadenosine 3 5 monophosphate,8 cl camp,8 chloro cyclic adnenosine monophosphate,adenazole,tocladesine,c amp	[[0,1,0,0,0,0,0,0,0,0,0],[0,0,0,0,1,0,0,0,0,0,0],[0,1,0,0,0,0,0,0,0,0,0],[0,0,0,0,1,0,0,0,0,1,0],[0,1,0,0,0,0,0,0,0,1,0],[0,1,0,0,1,0,0,0,0,0,0],[0,0,0,0,0,0,0,0,0,1,0],[0,0,0,0,1,0,0,0,0,0,0],[0,0,0,0,1,0,0,0,0,1,0],[0,0,0,0,0,0,0,0,0,1,0],[0,0,0,0,1,0,0,0,0,0,0]]C0050060	8 chloro 3 4 5 7 tetrahydroxyisoflavone,8 cthif	[[0,1,0,0,0,0,0,0,0,0,0],[0,1,0,0,0,0,0,0,0,0,0]]C0771780	magnesium iodide	[[0,1,0,0,0,0,0,0,0,0,0]]C0600412	nucleic acid vaccines	[[0,1,0,0,0,0,0,0,1,0,0]]C0600413	polynucleotide vaccines	[[0,1,0,0,0,0,0,0,1,0,0]]C0600416	hawthorn preparation,hawthorn	[[0,0,0,0,0,0,0,1,0,0,0],[0,0,0,0,0,0,1,0,0,0,0]]C0050069	8 cyclopropyltheophylline,8 cprt	[[0,1,0,0,0,0,0,0,0,0,0],[0,1,0,0,0,0,0,0,0,0,0]]C0057596	dexamethasone 21 phosphate	[[0,1,0,0,0,0,0,0,0,0,0]]C0057594	dexamethasone 21 palmitate	[[0,1,0,0,0,0,0,0,0,0,0]]C0057592	dexamethasone 21 iodoacetate,11beta 16alpha 9 fluoro 11 17 dihydroxy 21 iodoacetyl oxy 16 methyl pregna 1 4 diene 3 20 dione	[[0,1,0,0,0,0,0,0,0,0,0],[0,1,0,0,0,0,0,0,0,0,0]]C0057591	dexamethasone 21 bromoacetate,11beta 16alpha 21 bromoacetyl oxy 9 fluoro 11 17 dihydroxy 16 methyl pregna 1 4 diene 3 20 dione	[[0,1,0,0,0,0,0,0,0,0,0],[0,1,0,0,0,0,0,0,0,0,0]]C0388108	ncx 4215	[[0,1,0,0,0,0,0,0,0,0,0]]C1445788	phenobarbital belladona alkaloid	[[1,0,0,0,0,0,0,0,0,0,0]]C1445789	guaifenesin phenylephrine phenylpropanolamine	[[1,0,0,0,0,0,0,1,0,1,1]]C1995132	caduet 10 20	[[0,0,0,0,0,0,0,1,0,0,0]]C1097130	pentagalloylglucose,beta d pentagalloylglucose	[[0,1,0,0,0,0,0,0,0,0,0],[0,1,0,0,0,0,0,0,0,0,0]]C1097132	sb 334867	[[0,1,0,0,0,0,0,0,0,0,0]]C2975283	mozavaptan,physuline	[[0,1,0,0,0,0,0,0,0,1,0],[0,1,0,0,0,0,0,0,0,0,0]]C0113136	des n tetramethyltriostin a	[[0,1,0,0,0,0,0,0,0,0,0]]C1530888	n 3 acyloxy 2 benzylpropyl n 4 methylsulfonylamino benzyl thiourea,n 3 ab 4 msab t	[[0,1,0,0,0,0,0,0,0,0,0],[0,1,0,0,0,0,0,0,0,0,0]]C1445783	aspirin caffeine orphenadrine	[[1,0,0,0,0,0,0,1,0,0,1]]C0113132	des enkephalin gamma endorphin,1 de l tyrosine 2 deglycine 3 deglycine 4 de l phenylalanine 5 de l methionine 16a l leucine alpha endorphin,beta endorphin,beta lipotropin 66 77,beta lph,degammae	[[0,1,0,0,0,0,0,0,0,0,0],[0,1,0,0,0,0,0,0,0,0,0],[0,1,0,0,0,0,0,0,0,0,0],[0,1,0,0,0,0,0,0,0,0,0],[0,1,0,0,0,0,0,0,0,0,0],[0,1,0,0,0,0,0,0,0,0,0]]C1445785	aspirin oxycodone hydrochloride	[[1,0,0,0,0,0,0,0,0,0,0]]C1445786	penicillin g dextrose	[[1,0,0,0,0,0,0,0,0,0,0]]C1445787	penicillin g benzathine penicillin g procaine,penicillin g procaine benzathine	[[0,0,0,0,0,0,0,1,0,0,0],[1,0,0,0,0,0,0,0,0,0,0]]C0993226	mineral oil refined	[[0,0,0,0,0,0,0,0,0,0,1]]C0993224	medronate disodium	[[0,0,0,0,0,0,0,0,0,1,0]]C0755828	dichloro phenylacetaldehyde thiosemicarbazone palladium ii,pd pats cl2	[[0,1,0,0,0,0,0,0,0,0,0],[0,1,0,0,0,0,0,0,0,0,0]]C0993223	beef pork nph insulin,bovine porcine isophane insulin,beef pork isophane insulin	[[0,0,0,0,0,0,0,1,0,0,1],[0,0,0,0,0,0,0,0,0,0,1],[0,0,0,0,0,0,0,0,0,0,1]]C0993220	gluceptate sodium,sodium d glycero d gulo heptonate,monosodium d glycero d gulo heptonate	[[0,0,0,0,0,0,0,0,0,1,0],[0,0,0,0,0,0,0,0,0,1,0],[0,0,0,0,0,0,0,0,0,1,0]]C0755826	dichloro phenylacetaldehyde thiosemicarbazone platinum ii,pt pats cl2	[[0,1,0,0,0,0,0,0,0,0,0],[0,1,0,0,0,0,0,0,0,0,0]]C2975287	6 dehydrogingerdione	[[0,1,0,0,0,0,0,0,0,0,0]]C0623703	9 2 benzyloxyl 1 benzyloxymethyl ethoxy methyl 6 chloroguanine	[[0,1,0,0,0,0,0,0,0,0,0]]C0636488	ac nle 4 orn 5 glu 8 alpha msh 4 11 nh2,acetyl 4 norleucyl 5 ornithinyl 8 glutamic acid alpha msh 4 11 amide,acetyl 4 norleucyl 5 ornithinyl 8 glutamyl alpha msh 4 11 amide,alpha msh anog amide	[[0,1,0,0,0,0,0,0,0,0,0],[0,1,0,0,0,0,0,0,0,0,0],[0,1,0,0,0,0,0,0,0,0,0],[0,1,0,0,0,0,0,0,0,0,0]]C0390953	vp 616l	[[0,1,0,0,0,0,0,0,0,0,0]]C0390952	2 deoxy 2 fluoro scyllo inositol 1 o dodecylphosphonate	[[0,1,0,0,0,0,0,0,0,0,0]]C0623705	biolf 70,6 chloro 9 2 phenylmethoxy 1 phenylmethoxy methyl ethoxy methyl 9h purin 2 amine	[[0,1,0,0,0,0,0,0,0,0,0],[0,1,0,0,0,0,0,0,0,0,0]]C0636483	n methyl n 4 aminocytidine,nmact	[[0,1,0,0,0,0,0,0,0,0,0],[0,1,0,0,0,0,0,0,0,0,0]]C2975285	panaxatrol disuccinate	[[0,1,0,0,0,0,0,0,0,0,0]]C0163040	dinoterb,2 4 dinitro 6 tert butylphenol	[[0,1,0,0,0,0,0,0,0,0,0],[0,1,0,0,0,0,0,0,0,0,0]]C0163042	dipotassium glycyrrhizinate	[[0,1,1,0,0,0,0,0,0,1,0]]C2737524	carbamazepine 10 11 epoxide 124 blood dot,carbamazepine 10 11 epoxide 124 dried blood spot	[[0,0,0,1,0,0,0,0,0,0,0],[0,0,0,1,0,0,0,0,0,0,0]]C0243717	multideterminant antigen t g a l	[[0,1,0,0,0,0,0,0,0,0,0]]C3154026	calcium chloride icodextrin lactate magnesium chloride sodium chloride	[[0,0,0,0,0,0,0,1,0,0,0]]C0669079	1 dodecyl 2 octanamido sn 2 deoxyglycero 3 phosphocholine,1 dodgpc	[[0,1,0,0,0,0,0,0,0,0,0],[0,1,0,0,0,0,0,0,0,0,0]]C0669078	1 dodecyl 2 1 14 c octanamido sn 2 deoxyglycero 3 phosphocholine	[[0,1,0,0,0,0,0,0,0,0,0]]C0669076	technetium tc 99m monoclonal antibody 170h 82,tc 99m mab 170h 82,99mtc mab170h 82	[[0,1,0,0,0,0,0,0,0,0,0],[0,1,0,0,0,0,0,0,0,0,0],[0,1,0,0,0,0,0,0,0,0,0]]C0669075	truscint ad	[[0,1,0,0,0,0,0,0,0,0,0]]C0060050	falcarinol,1 9 heptadecadiene 4 6 diyn 3 ol	[[0,1,0,0,0,0,0,0,0,0,0],[0,1,0,0,0,0,0,0,0,0,0]]C2828283	mezlocillin sodium monohydrate	[[0,0,0,0,0,0,0,0,0,1,0]]C0060053	falintolol,o 3 tert butylamino 2 hydroxypropyl cyclopropyl methyl ketone oxime	[[0,1,0,0,0,0,0,0,0,1,0],[0,1,0,0,0,0,0,0,0,0,0]]C0060054	falirytmin,n n bis 3 2 ethoxyphenoxy 2 hydroxypropyl ethylenediamine	[[0,1,0,0,0,0,0,0,0,0,0],[0,1,0,0,0,0,0,0,0,0,0]]C0060059	fanetizole,2 phenethylamino 4 phenylthiazole	[[0,1,0,0,0,0,0,0,0,1,0],[0,1,0,0,0,0,0,0,0,0,0]]C1448404	caproamin,rottapharm brand of aminocaproic acid	[[0,1,0,0,0,0,0,0,0,0,0],[0,1,0,0,0,0,0,0,0,0,0]]C1448405	caprolest,pharmachemie brand of aminocaproic acid	[[0,1,0,0,0,0,0,0,0,0,0],[0,1,0,0,0,0,0,0,0,0,0]]C1448406	eac,berlin chemie brand of aminocaproic acid	[[0,1,0,0,0,0,0,0,0,0,0],[0,1,0,0,0,0,0,0,0,0,0]]C1448407	hemocaprol,delagrange brand of aminocaproic acid	[[0,1,0,0,0,0,0,0,0,0,0],[0,1,0,0,0,0,0,0,0,0,0]]C1448400	fibroderm,torlan brand of aminobenzoic acid potassium salt	[[0,1,0,0,0,0,0,0,0,0,0],[0,1,0,0,0,0,0,0,0,0,0]]C1448401	epitelplast,llorens brand of aminobenzoic acid sodium salt	[[0,1,0,0,0,0,0,0,0,0,0],[0,1,0,0,0,0,0,0,0,0,0]]C1448402	mielogen,made brand of molgramostim	[[0,1,0,0,0,0,0,0,0,0,0],[0,1,0,0,0,0,0,0,0,0,0]]C1448403	capralense,sanofi winthrop brand of aminocaproic acid,capramol	[[0,1,0,0,0,0,0,0,0,0,0],[0,1,0,0,0,0,0,0,0,0,0],[0,1,0,0,0,0,0,0,0,0,0]]C1448408	hexalense,leurquin brand of aminocaproic acid	[[0,1,0,0,0,0,0,0,0,0,0],[0,1,0,0,0,0,0,0,0,0,0]]C1448409	amiobeta,betapharm brand of amiodarone hydrochloride	[[0,1,0,0,0,0,0,0,0,0,0],[0,1,0,0,0,0,0,0,0,0,0]]C2828282	amoxicillin anhydrous,2s 5r 6r 6 r 2 amino 2 p hydroxyphenyl acetamido 3 3 dimethyl 7 oxo 4 thia 1 azabicyclo 3 2 0 heptane 2 carboxylic acid,6 amino 4 hydroxyphenyl acetyl amino 3 3 dimethyl 7 oxo 2s 2 alpha 5 alpha 6 beta s 4 thia 1 azabicyclo 3 2 0 heptane 2 carboxylic acid	[[0,0,0,0,0,0,0,0,0,1,0],[0,0,0,0,0,0,0,0,0,1,0],[0,0,0,0,0,0,0,0,0,1,0]]C1308077	streptomyces capreolus ard2 protein	[[0,1,0,0,0,0,0,0,0,0,0]]C0293058	tachypleus tridentatus lici 2 protein,tachypleus tridentatus limulus intracellular coagulation inhibitor 2	[[0,1,0,0,0,0,0,0,0,0,0],[0,1,0,0,0,0,0,0,0,0,0]]C0284763	shu 450,s hu 450	[[0,1,0,0,0,0,0,0,0,0,0],[0,1,0,0,0,0,0,0,0,0,0]]C0284766	3 4 dehydro exo brevicomin,7 exo ethyl 5 methyl 6 8 dioxabicyclo 3 2 1 3 octene	[[0,1,0,0,0,0,0,0,0,0,0],[0,1,0,0,0,0,0,0,0,0,0]]C0659243	respirantin	[[0,1,0,0,0,0,0,0,0,0,0]]C3253139	3 bromo 2 oxopropionate 1 propyl esster,3 brop cpd	[[0,1,0,0,0,0,0,0,0,0,0],[0,1,0,0,0,0,0,0,0,0,0]]C0659241	bu 4704	[[0,1,0,0,0,0,0,0,0,0,0]]C0659245	4 amino 2 deoxy 2 3 didehydro n acetylneuraminic acid,4 amino neu5ac2en	[[0,1,0,0,0,0,0,0,0,0,0],[0,1,0,0,0,0,0,0,0,0,0]]C2699774	difenoximide hydrochloride	[[0,0,0,0,0,0,0,0,0,1,0]]C1566689	4 5 naphthyl 3 trifluoromethyl 1h pyrazol 1 yl benzenesulfonamide,pc 407	[[0,1,0,0,0,0,0,0,0,0,0],[0,1,0,0,0,0,0,0,0,0,0]]C1566688	pro pro hghrh 1 44 gly gly cys,prolyl prolyl hghrh 1 44 glycyl glycyl cysteine	[[0,1,0,0,0,0,0,0,0,0,0],[0,1,0,0,0,0,0,0,0,0,0]]C0008947	clindamycin,7 chloro 7 deoxylincomycin,chlorlincocin,chlolincocin,2s trans methyl 7 chloro 6 7 8 trideoxy 6 1 methyl 4 propyl 2 pyrrolidinyl carbonyl amino 1 thio l threo alpha d galacto octopyranoside,clindamicin,clindamycin product	[[0,0,0,0,0,0,1,0,0,1,0],[0,1,0,0,0,0,0,0,0,1,0],[0,0,0,0,0,0,0,0,0,0,1],[0,0,0,0,0,0,0,0,0,0,1],[0,0,0,0,0,0,0,0,0,0,1],[1,0,0,0,0,0,1,0,0,0,0],[1,0,0,0,0,0,0,0,0,0,0]]C0019443	hexadimethrine,1 5 dimethyl 1 5 diazaundecamethylene polymethobromide,hexadimethrine bromide,polymer with 1 3 dibromopropane n n n n tetramethyl 1 6 hexanediamine	[[0,0,0,0,0,0,1,0,0,0,1],[0,0,0,0,0,0,0,0,0,0,1],[0,1,0,0,0,0,1,0,0,0,1],[0,0,0,0,0,0,0,0,0,0,1]]C0729163	bl 191,bl191	[[0,1,0,0,0,0,0,0,0,0,0],[0,1,0,0,0,0,0,0,0,0,0]]C0633095	6 11 hexadecadienyl diazoacetate	[[0,1,0,0,0,0,0,0,0,0,0]]C2963097	oraline	[[0,0,0,0,0,0,0,1,0,0,0]]C0063582	inositol hexasulfate,inositol hexkissulfate,myo inositol hexakis,myoinositol hexakissulfate,myoinositol hexasulfate	[[0,1,0,0,0,0,0,0,0,0,0],[0,1,0,0,0,0,0,0,0,0,0],[0,1,0,0,0,0,0,0,0,0,0],[0,1,0,0,0,0,0,0,0,0,0],[0,1,0,0,0,0,0,0,0,0,0]]C1567376	flusporan,menarini brand of flutrimazole	[[0,1,0,0,0,0,0,0,0,0,0],[0,1,0,0,0,0,0,0,0,0,0]]C1567377	funcenal,farma lepori brand of flutrimazole	[[0,1,0,0,0,0,0,0,0,0,0],[0,1,0,0,0,0,0,0,0,0,0]]C0039358	taurocholic acid,cholyltaurine,2 3alpha 5beta 7alpha 12alpha 3 7 12 trihydroxy 24 oxocholan 24 yl amino ethanesulfonic acid	[[1,0,0,0,0,0,0,0,0,0,1],[0,0,0,0,0,0,0,0,0,0,1],[0,0,0,0,0,0,0,0,0,0,1]]C1567375	cutimian,biohorm brand of flutrimazole	[[0,1,0,0,0,0,0,0,0,0,0],[0,1,0,0,0,0,0,0,0,0,0]]C1567372	novo flutamide,novopharm brand of flutamide,novoflutamide	[[0,1,0,0,0,0,0,0,0,0,0],[0,1,0,0,0,0,0,0,0,0,0],[0,1,0,0,0,0,0,0,0,0,0]]C1567370	flutandrona,ciclum brand of flutamide	[[0,1,0,0,0,0,0,0,0,0,0],[0,1,0,0,0,0,0,0,0,0,0]]C1567371	flutexin,juta brand of flutamide,q pharm brand of flutamide	[[0,1,0,0,0,0,0,0,0,0,0],[0,1,0,0,0,0,0,0,0,0,0],[0,1,0,0,0,0,0,0,0,0,0]]C1567378	micetal,uriach brand of flutrimazole	[[0,1,0,0,0,0,0,0,0,0,0],[0,1,0,0,0,0,0,0,0,0,0]]C1567379	amn107,amn 107	[[0,1,0,0,0,0,0,0,0,0,0],[0,0,0,0,1,0,0,0,0,0,0]]C1172244	cns 5161	[[0,1,0,0,0,0,0,0,0,0,0]]C1172245	n 2 chloro 5 methylmercapto phenyl n methylguanidine monohydrochloride	[[0,1,0,0,0,0,0,0,0,0,0]]C1172246	jte 607	[[0,1,0,0,0,0,0,0,0,0,0]]C0298816	lubrithal	[[0,1,0,0,0,0,0,0,0,0,0]]C1172240	2 4 dihydroxy 2 6 trimethyl delta 1 alpha cyclohexaneacetic r lactone	[[0,1,0,0,0,0,0,0,0,0,0]]C0885838	nosode of tuberculosis tuberculinum	[[0,0,0,0,0,1,0,0,0,0,0]]C0885833	tribulus terrestris homeopathic preparation	[[0,0,0,0,0,1,0,0,0,0,0]]C1172249	n methyl n benzylnitrosamine,n methyl n benzyl nitrosamine	[[0,1,0,0,0,0,0,0,0,0,0],[0,1,0,0,0,0,0,0,0,0,0]]C0885831	trachinus vipera homeopathic preparation	[[0,0,0,0,0,1,0,0,0,0,0]]C0643569	n 4 aminobenzoyloxy succinimide,para abs,n p aminobenzoyloxy succinimide	[[0,1,0,0,0,0,0,0,0,0,0],[0,1,0,0,0,0,0,0,0,0,0],[0,1,0,0,0,0,0,0,0,0,0]]C0011701	desmopressin,1 deamino 8 d arginine vasopressin,1 desamino 8 arginine vasopressin,deamino arginine vasopressin,1 3 mercaptopropanoic acid 8 d arginine vasopressin,desmopressin preparation,desmopressin product	[[0,0,0,0,0,0,0,1,0,1,0],[0,1,0,0,0,0,0,0,0,1,0],[0,1,0,0,0,0,0,0,0,0,1],[0,1,0,0,0,0,0,0,0,0,1],[0,0,0,0,0,0,0,0,0,0,1],[1,0,0,0,0,0,0,0,0,0,0],[1,0,0,0,0,0,0,0,0,0,0]]C0097146	4 imidazolyl 3 amino 2 butanone,3 amino 4 1h imidazol 4 yl 2 butanone	[[0,1,0,0,0,0,0,0,0,0,0],[0,1,0,0,0,0,0,0,0,0,0]]C0097147	4 imino 1 3 diazabicyclo 3 1 0 hexan 2 one,4 amino 1 3 diazabicyclo 3 1 0 hex 3 en 2 one	[[0,1,0,0,1,0,0,0,0,0,0],[0,1,0,0,0,0,0,0,0,0,0]]C0053747	bis phenanthrenequinonediimine bipyridyl rhodium iii,rh phi 2 bpy 3,rhpb	[[0,1,0,0,0,0,0,0,0,0,0],[0,1,0,0,0,0,0,0,0,0,0],[0,1,0,0,0,0,0,0,0,0,0]]C0053743	bis pentachlorophenyl oxalate,b pcpo,bis pentachlorophenyl ester ethanedioic acid	[[0,1,0,0,0,0,0,0,0,0,0],[0,1,0,0,0,0,0,0,0,0,0],[0,1,0,0,0,0,0,0,0,0,0]]C0053741	bis n maleimidomethyl ether,1 1 oxybis methylene bis 1h pyrrole 2 5 dione,n n oxydimethylene dimaleimide	[[0,1,0,0,0,0,0,0,0,0,0],[0,1,0,0,0,0,0,0,0,0,0],[0,1,0,0,0,0,0,0,0,0,0]]C0658729	2 tetradecanoylaminohexanol 1 phosphocholine,2 tdah pc	[[0,1,0,0,0,0,0,0,0,0,0],[0,1,0,0,0,0,0,0,0,0,0]]C1260246	tx 1123	[[0,1,0,0,0,0,0,0,0,0,0]]C2828286	oglemilast sodium,monosodium salt n 3 5 dichloro 4 pyridinyl 4 difluoromethoxy 8 methylsulfonyl amino 1 dibenzofurancarboxamide	[[0,0,0,0,0,0,0,0,0,1,0],[0,0,0,0,0,0,0,0,0,1,0]]C1260249	tas 202	[[0,1,0,0,0,0,0,0,0,0,0]]C2918858	tussi organidin dm 10 100	[[0,0,0,0,0,0,0,1,0,0,0]]C0045780	2 phenylethylhydrazono propionic acid,2 2 phenylethyl hydrazono propanoic acid	[[0,1,0,0,0,0,0,0,0,0,0],[0,1,0,0,0,0,0,0,0,0,0]]C1601672	bio tab	[[0,0,0,0,0,0,0,1,0,0,0]]C2918853	tussi organidin 10 100	[[0,0,0,0,0,0,0,1,0,0,0]]C0008362	choleretics,choleretic agent	[[0,1,0,0,0,0,1,0,0,0,0],[1,0,0,0,0,0,0,0,0,0,0]]C0218405	2 2 3 dicarboxycyclopropyl glycine,dcg iv	[[0,1,0,0,0,0,0,0,0,0,0],[0,1,0,0,0,0,0,0,0,0,0]]C0218403	histogranin	[[0,1,0,0,0,0,0,0,0,0,0]]C1828966	p tann d	[[0,0,0,0,0,0,0,1,0,0,0]]C1831903	lyso thermosensitive liposome doxorubicin,heat activated liposomal doxorubicin hydrochloride,temperature sensitive liposome encapsulated doxorubicin	[[0,0,0,0,1,0,0,0,0,1,0],[0,0,0,0,0,0,0,0,0,1,0],[0,0,0,0,0,0,0,0,0,1,0]]C0285417	octastatin	[[0,0,0,0,0,0,0,0,0,1,0]]C0890971	fosmidomycin monoammonium salt	[[0,1,0,0,0,0,0,0,0,0,0]]C0890972	fosmidomycin monosodium salt	[[0,1,0,0,0,0,0,0,0,0,0]]C0890973	fosmidomycin sodium salt	[[0,1,0,0,0,0,0,0,0,0,0]]C0664135	coptidis rhizoma extract	[[0,1,0,0,0,0,0,0,0,0,0]]C0620420	6 sulfoaminopenicillanic acid	[[0,1,0,0,0,0,0,0,0,0,0]]C0962314	4 3 methylbut 1 enyl 3 5 2 4 tetrahydroxystilbene,4 mebu4ohs	[[0,1,0,0,0,0,0,0,0,0,0],[0,1,0,0,0,0,0,0,0,0,0]]C0080897	3 6 diamino 9 4 methylsulfonyl aminophenyl aminoacridine,3 6 diamino amsa	[[0,1,0,0,0,0,0,0,0,0,0],[0,1,0,0,0,0,0,0,0,0,0]]C0664137	1 o octadecyl sn glycero 3 phospho acyclovir,odg p acv	[[0,1,0,0,0,0,0,0,0,0,0],[0,1,0,0,0,0,0,0,0,0,0]]C0140818	robercain r	[[0,1,0,0,0,0,0,0,0,0,0]]C0969472	5 6 hydroxy 2 5 7 8 tetramethyl chroman 2 yl 2 methyl pentanoic acid,alpha cmbhc	[[0,1,0,0,0,0,0,0,0,0,0],[0,1,0,0,0,0,0,0,0,0,0]]C0005285	beta thrombin	[[0,1,0,0,0,0,0,0,0,0,0]]C1136857	o o acetyldaurisoline,o o acetyl daurisoline	[[0,1,0,0,0,0,0,0,0,0,0],[0,1,0,0,0,0,0,0,0,0,0]]C0005287	beta tocopherol,beta tocopherol preparation,3 4 dihydro 2 5 8 trimethyl 2 4 8 12 trimethyltridecyl 2h 1 benzopyran 6 ol,2 5 8 trimethyl 2 4 8 12 trimethyltridecyl chroman 6 ol	[[1,1,0,0,0,0,0,0,0,1,1],[1,0,0,0,0,0,0,0,0,0,0],[0,0,0,0,0,0,0,0,0,0,1],[0,0,0,0,0,0,0,0,0,1,0]]C0145377	tetraiodothyroacetate	[[0,1,0,0,0,0,0,0,0,0,0]]C1739640	agelasidine a	[[0,1,0,0,0,0,0,0,0,0,0]]C1739646	ethyl 1 4 oxo 8 aryl 4 6 7 8 tetrahydroimidazo 2 1 c 1 2 4 triazin 3 yl formate,e o thi tf cpd	[[0,1,0,0,0,0,0,0,0,0,0],[0,1,0,0,0,0,0,0,0,0,0]]C1872707	o carbamoylthreonine	[[0,1,0,0,0,0,0,0,0,0,0]]C0120311	grinazole	[[0,1,0,0,0,0,0,0,0,0,0]]C0120312	1 1 dodecyl 2 piperidinecarboxylic acid griselimycin	[[0,1,0,0,0,0,0,0,0,0,0]]C0120313	griseolic acid 8 pivaloyloxymethyl ester,ga pom ester	[[0,1,0,0,0,0,0,0,0,0,0],[0,1,0,0,0,0,0,0,0,0,0]]C0967496	acetyl aavallpavllallap devd cho,ac aavallpavllallap devd cho	[[0,1,0,0,0,0,0,0,0,0,0],[0,1,0,0,0,0,0,0,0,0,0]]C0967497	acetyl aspartyl glutamyl valyl lysine chloromethyl ketone,ac devk cmk	[[0,1,0,0,0,0,0,0,0,0,0],[0,1,0,0,0,0,0,0,0,0,0]]C0967495	acetyl aavallpavllallap yvad cho,ac aavallpavllallap yvad cho	[[0,1,0,0,0,0,0,0,0,0,0],[0,1,0,0,0,0,0,0,0,0,0]]C0967492	methyl 4 o feruloyl 5 o caffeoylquinate	[[0,1,0,0,0,0,0,0,0,0,0]]C0967491	4 o feruloyl 5 o caffeoylquinic acid	[[0,1,0,0,0,0,0,0,0,0,0]]C0967498	acetyl tyrosyl valyl alanyl aspartic acid p nitroanilide,ac yvad pna	[[0,1,0,0,0,0,0,0,0,0,0],[0,1,0,0,0,0,0,0,0,0,0]]C0967499	acetyl aspartyl glutamyl valyl aspartic acid p nitroanilide,ac devd pna	[[0,1,0,0,0,0,0,0,0,0,0],[0,1,0,0,0,0,0,0,0,0,0]]C2609748	clearasil acne control	[[0,0,0,0,0,0,0,1,0,0,0]]C1564858	novo flurprofen,novopharm brand of flurbiprofen	[[0,1,0,0,0,0,0,0,0,0,0],[0,1,0,0,0,0,0,0,0,0,0]]C1564859	nu flurbiprofen,nu pharm brand of flurbiprofen	[[0,1,0,0,0,0,0,0,0,0,0],[0,1,0,0,0,0,0,0,0,0,0]]C1564856	dobrofen,klosterfrau brand of flurbiprofen	[[0,1,0,0,0,0,0,0,0,0,0],[0,1,0,0,0,0,0,0,0,0,0]]C1564857	neo artrol,recordati brand of flurbiprofen	[[0,1,0,0,0,0,0,0,0,0,0],[0,1,0,0,0,0,0,0,0,0,0]]C1564854	apo flurbiprofen,apotex brand of flurbiprofen	[[0,1,0,0,0,0,0,0,0,0,0],[0,1,0,0,0,0,0,0,0,0,0]]C1564855	cebutid,shire brand of flurbiprofen	[[0,1,0,0,0,0,0,0,0,0,0],[0,1,0,0,0,0,0,0,0,0,0]]C1564852	staurodorm,dolorgiet brand of flurazepam	[[0,1,0,0,0,0,0,0,0,0,0],[0,1,0,0,0,0,0,0,0,0,0]]C1564853	apo flurazepam,apotex brand of flurazepam monohydrochloride	[[0,1,0,0,0,0,0,0,0,0,0],[0,1,0,0,0,0,0,0,0,0,0]]C0609891	n aminomethylcarbamic acid tert butyl ester,1 1 dimethylethyl ester aminomethyl carbamic acid	[[0,1,0,0,0,0,0,0,0,0,0],[0,1,0,0,0,0,0,0,0,0,0]]C1564851	onkofluor,onkoworks brand of fluorouracil	[[0,1,0,0,0,0,0,0,0,0,0],[0,1,0,0,0,0,0,0,0,0,0]]C0101809	ag 66 18	[[0,1,0,0,0,0,0,0,0,0,0]]C0955968	disodium salt glucosulfone	[[0,1,0,0,0,0,0,0,0,0,0]]C0619920	cloximate hydrochloride	[[0,1,0,0,0,0,0,0,0,0,0]]C0890390	s isomer meclonazepam	[[0,1,0,0,0,0,0,0,0,0,0]]C0890391	r isomer meclonazepam	[[0,1,0,0,0,0,0,0,0,0,0]]C0304999	iodohippurate i123 sodium,iodohippurate i 123 sodium,iodohippurate i sup 123 sup sodium	[[1,0,0,0,0,0,0,0,0,0,0],[1,0,0,0,0,0,0,0,0,1,0],[1,0,0,0,0,0,0,0,0,0,0]]C0101805	ag 127,tyrphostin ag127	[[0,1,0,0,0,0,0,0,0,0,0],[0,1,0,0,0,0,0,0,0,0,0]]C0101804	afungin	[[0,1,0,0,0,0,0,0,0,0,0]]C0101807	ag 183,z 2 amino 4 3 4 5 trihydroxyphenyl 1 3 butadiene 1 1 3 tricarbonitrile,tyrphostin ag 183	[[0,1,0,0,0,0,0,0,0,0,0],[0,1,0,0,0,0,0,0,0,0,0],[0,1,0,0,0,0,0,0,0,0,0]]C1965521	totect	[[0,0,0,0,0,0,0,0,0,1,0]]C3178178	1 5 bis 2 3 dimethoxyphenyl penta 1 4 dien 3 one,1 5 bisdmppdo	[[0,1,0,0,0,0,0,0,0,0,0],[0,1,0,0,0,0,0,0,0,0,0]]C3253705	desferrioxamine e	[[0,1,0,0,0,0,0,0,0,0,0]]C1992584	mexiletine 124 urine	[[0,0,0,1,0,0,0,0,0,0,0]]C1965524	allres pd	[[0,0,0,0,0,0,0,1,0,0,0]]C1965527	allres g	[[0,0,0,0,0,0,0,1,0,0,0]]C0099324	6 methyl 7 hydroxyribolumazine	[[0,1,0,0,0,0,0,0,0,0,0]]C0099320	6 methyl 5 pyridy 4 yl 3h 6h 1 3 4 thiadiazin 2 one	[[0,1,0,0,0,0,0,0,0,0,0]]C0286190	3 acetoxymercuri 4 aminobenzenesulfonamide,acetato o 2 amino 5 aminosulfonyl phenyl mercury,3 amabs	[[0,1,0,0,0,0,0,0,0,0,0],[0,1,0,0,0,0,0,0,0,0,0],[0,1,0,0,0,0,0,0,0,0,0]]C1873068	ap 23573,ap23573	[[0,1,0,0,0,0,0,0,0,0,0],[0,0,0,0,0,0,0,0,0,1,0]]C0646105	trichlorobisdimethylsulphoxideaminoruthenium	[[0,1,0,0,0,0,0,0,0,0,0]]C1101330	sc 51 cpd,sc51 cpd	[[0,1,0,0,0,0,0,0,0,0,0],[0,1,0,0,0,0,0,0,0,0,0]]C0055359	chlorflavonin,3 chloro 2 5 dihydroxy 3 7 8 trimethoxyflavone	[[0,1,0,0,0,0,0,0,0,0,0],[0,1,0,0,0,0,0,0,0,0,0]]C0055357	chlorfenpropmethyl,methyl 2 chloro 3 4 chlorophenyl propionate	[[0,1,0,0,0,0,0,0,0,0,0],[0,1,0,0,0,0,0,0,0,0,0]]C0055356	chlorfenethol,4 4 dichloro alpha methylbenzhydrol,bis p chlorophenyl methyl carbinol,dcpc,di p chlorophenyl methyl carbinol,dimite,dmc	[[0,1,0,0,0,0,0,0,0,0,0],[0,1,0,0,0,0,0,0,0,0,0],[0,1,0,0,0,0,0,0,0,0,0],[0,1,0,0,0,0,0,0,0,0,0],[0,1,0,0,0,0,0,0,0,0,0],[0,1,0,0,0,0,0,0,0,0,0],[0,1,0,0,0,0,0,0,0,0,0]]C0055355	chlorfenethazine,2 chloro 10 beta dimethylaminoethyl phenothiazine,chlorphenethazine	[[0,0,0,0,0,0,0,1,0,0,0],[0,1,0,0,0,0,0,0,0,0,0],[0,1,0,0,0,0,0,0,0,0,0]]C0055354	chlorethylclonidine,chloroethylclonidine	[[0,1,0,0,0,0,0,0,0,0,0],[0,1,0,0,0,0,0,0,0,0,0]]C0055353	chlordesmethyldiazepam,2 chloronordiazepam,7 chloro 5 2 chlorophenyl 1 3 dihydro 2h 1 4 benzodiazepin 2 one,chlordemethyldiazepam,chlorodesmethyldiazepam,delorazepam	[[0,1,0,0,0,0,0,0,0,0,0],[0,1,0,0,0,0,0,0,0,0,0],[0,1,0,0,0,0,0,0,0,0,0],[0,1,0,0,0,0,0,0,0,0,0],[0,1,0,0,0,0,0,0,0,0,0],[0,1,0,0,0,0,0,0,0,0,0]]C0304424	oxyphencyclimine hydrochloride	[[0,1,0,0,0,0,0,1,0,1,0]]C0917774	trans isomer tetrahydrocannabinol	[[0,1,0,0,0,0,0,0,0,0,0]]C1992816	morphine 124 gastric fluid	[[0,0,0,1,0,0,0,0,0,0,0]]C0540057	n 4 chlorophenyl 2 3 bis cyclopropylmethylene cyclopentanecarboxamide	[[0,1,0,0,0,0,0,0,0,0,0]]C0540056	l 245976,l 245 976	[[0,1,0,0,0,0,0,0,0,0,0],[0,1,0,0,0,0,0,0,0,0,0]]C0388071	staphylococcus epidermidis epic protein	[[0,1,0,0,0,0,0,0,0,0,0]]C1992812	moricizine 124 bld ser plas	[[0,0,0,1,0,0,0,0,0,0,0]]C0540053	g28 5 sfv pe40	[[0,0,0,0,0,0,0,0,0,1,0]]C0165076	sl 81 0385,sl 81035	[[0,1,0,0,0,0,0,0,0,0,0],[0,1,0,0,0,0,0,0,0,0,0]]C0165077	litoxetine,4 2 naphthalenylmethoxy piperidine	[[0,1,0,0,0,0,0,0,0,1,0],[0,1,0,0,0,0,0,0,0,0,0]]C0165072	ym 638	[[0,1,0,0,0,0,0,0,0,0,0]]C0917779	mesnum	[[0,0,0,0,1,0,0,0,0,0,0]]C1992819	morphine 124 urine	[[0,0,0,1,0,0,0,0,0,0,0]]C1992818	morphine 124 meconium	[[0,0,0,1,0,0,0,0,0,0,0]]C0131051	n dimethylbutyramide 4 chlorophenoxyisobutyrate	[[0,1,0,0,0,0,0,0,0,0,0]]C0131050	n dimethyl n propyldodecanamide betaine sodium	[[0,1,0,0,0,0,0,0,0,0,0]]C0131057	n dodecyl n n beta imidopropionate	[[0,1,0,0,0,0,0,0,0,0,0]]C1590701	qual tussin	[[0,0,0,0,0,0,0,1,0,0,0]]C0208560	guatteguamerine,7 7 demethyldauricine,dauriciline,guattegaumerine	[[0,1,0,0,0,0,0,0,0,0,0],[0,1,0,0,0,0,0,0,0,0,0],[0,1,0,0,0,0,0,0,0,0,0],[0,1,0,0,0,0,0,0,0,0,0]]C2723461	ulipristal acetate	[[0,0,0,0,0,0,0,1,0,1,0]]C0081389	9 2 phosphonylmethoxy ethyl guanine,pmeg	[[0,1,0,0,0,0,0,0,0,0,0],[0,1,0,0,0,0,0,0,0,0,0]]C1590704	amerituss ad	[[0,0,0,0,0,0,0,1,0,0,0]]C1590707	ed a hist dm	[[0,0,0,0,0,0,0,1,0,0,0]]C0600873	spenco flakes	[[0,1,0,0,0,0,0,0,0,0,0]]C0139604	pz 51	[[0,1,0,0,0,0,0,0,0,0,0]]C0893282	oxophenarsine monohydrochloride	[[0,1,0,0,0,0,0,0,0,0,0]]C0968785	s 15183a,s 15183 a	[[0,1,0,0,0,0,0,0,0,0,0],[0,1,0,0,0,0,0,0,0,0,0]]C0968784	rp 1776,rp1776	[[0,1,0,0,0,0,0,0,0,0,0],[0,1,0,0,0,0,0,0,0,0,0]]C0893286	2 mercaptoethylguanidine dihydrobromide	[[0,1,0,0,0,0,0,0,0,0,0]]C0893285	2 mercaptoethylguanidine hydrobromide	[[0,1,0,0,0,0,0,0,0,0,0]]C0139603	pz 1511	[[0,1,0,0,0,0,0,0,0,0,0]]C1656834	desitin creamy	[[0,0,0,0,0,0,0,1,0,0,0]]C0893288	2 mercaptoethylguanidine monohydrobromide	[[0,1,0,0,0,0,0,0,0,0,0]]C0968789	streptocidin d	[[0,1,0,0,0,0,0,0,0,0,0]]C0968788	tmc 69 6h	[[0,1,0,0,0,0,0,0,0,0,0]]C3256285	lemon peel wax	[[0,0,1,0,0,0,0,0,0,0,0]]C3256284	carbomer 940 with hypoallergenic perfume	[[0,0,1,0,0,0,0,0,0,0,0]]C3256287	macrogol polyethylene glycol 3350	[[0,0,1,0,0,0,0,0,0,0,0]]C0527855	angelmicin b	[[0,1,0,0,0,0,0,0,0,0,0]]C1137471	isocurcumenol	[[0,1,0,0,0,0,0,0,0,0,0]]C3256280	c20 22 alcohols	[[0,0,1,0,0,0,0,0,0,0,0]]C3256283	caprylic capric diglycerol succinate	[[0,0,1,0,0,0,0,0,0,0,0]]C0527851	napec,n acetyl s 1 phenyl 2 hydroxyethyl cysteine n acetyl s 2 phenyl 2 hydroxyethyl cysteine mixture,n acetyl s 1 2 phenyl 2 hydroxyethyl cysteine	[[0,1,0,0,0,0,0,0,0,0,0],[0,1,0,0,0,0,0,0,0,0,0],[0,1,0,0,0,0,0,0,0,0,0]]C0609237	2 7 bis diethylamino acetamido fluoren 9 one,n n 9 oxo 3h fluorene 2 7 diyl bis 2 diethylamino acetamide	[[0,1,0,0,0,0,0,0,0,0,0],[0,1,0,0,0,0,0,0,0,0,0]]C1738999	fumarate n 4 4 5 dihydro 1h imidazol 2 yl phenyl methyl 2 2 4 methoxy 2 6 dimethylphenyl sulfonyl methylamino ethoxy n methylacetamide,lf22 0542	[[0,1,0,0,0,0,0,0,0,0,0],[0,1,0,0,0,0,0,0,0,0,0]]C0527858	sk f 97426 a,skf 97426 a	[[0,1,0,0,0,0,0,0,0,0,0],[0,1,0,0,0,0,0,0,0,0,0]]C0609234	bbal,5 butylnitrosoamino dihydro 2 3h furanone,4 n butylnitrosamino 4 hydroxybutyric acid lactone	[[0,1,0,0,0,0,0,0,0,0,0],[0,1,0,0,0,0,0,0,0,0,0],[0,1,0,0,0,0,0,0,0,0,0]]C0966367	1r 5s 6s 6 r 1 hydroxyethyl 2 3s 5s 5 s methyl 4 thiomorpholin ylcarbonyl pyrrolidin 3 thio l methylcarbapen 2 em 3 carboxylic acid	[[0,1,0,0,0,0,0,0,0,0,0]]C0796657	vaccinia tyrosinase vaccine,vactyros,recombinant vaccinia tyrosinase,rv tyr,rv tyr vaccine,recombinant vaccinia tyrosinase vaccine	[[0,0,0,0,1,0,0,0,0,1,0],[0,0,0,0,0,0,0,0,0,1,0],[0,0,0,0,0,0,0,0,0,1,0],[0,0,0,0,0,0,0,0,0,1,0],[0,0,0,0,0,0,0,0,0,1,0],[0,0,0,0,0,0,0,0,0,1,0]]C1170665	tol tab	[[0,0,0,0,0,0,0,1,0,0,0]]C1170668	tri luma	[[0,0,0,0,0,0,1,1,0,0,0]]C0058650	dollabella auricularia dolabellanin c protein,dolabellanin c	[[0,1,0,0,0,0,0,0,0,0,0],[0,1,0,0,0,0,0,0,0,0,0]]C0058652	doleron	[[0,1,0,0,0,0,0,0,0,0,0]]C2954929	perloxx 2 5 300	[[0,0,0,0,0,0,0,1,0,0,0]]C1542239	homeopathic preparation sulphur iodatum	[[0,0,0,0,0,1,0,0,0,0,0]]C1564636	eti puren,alpharma brand of etilefrine hydrochloride	[[0,1,0,0,0,0,0,0,0,0,0],[0,1,0,0,0,0,0,0,0,0,0]]C2983749	lactobacillus plantarum strain 299	[[0,0,0,0,0,0,0,0,0,1,0]]C0294399	2 bromo 3 4 dimethoxybenzyl 6 7 dimethoxy 1 2 3 4 tetrahydroisoquinoline	[[0,1,0,0,0,0,0,0,0,0,0]]C0628892	alpha aspartyl methotrexate	[[0,1,0,0,0,0,0,0,0,0,0]]C0383456	6 hydroxy 7 3 methyl 2 butenyloxy 5 benzofuranacrylic acid delta lactone	[[0,1,0,0,0,0,0,0,0,0,0]]C0767001	2 2 4 difluorophenyl 3 4 4 2 4 trifluoromethoxybenzyl 2h 1 2 4 triazol 3 one 4 yl phenyl piperazin 1 yl 1 1h 1 2 4 triazol 1 yl butan 2 ol	[[0,1,0,0,0,0,0,0,0,0,0]]C0767000	eberconazole,1 2 4 dichloro 10 11 dihydro 5h dibenzo a d cyclohepten 5 yl 1h imidazole	[[0,1,0,0,0,0,0,0,0,1,0],[0,1,0,0,0,0,0,0,0,0,0]]C0628897	methotrexate gamma dimethylamide,mxt gamma dma	[[0,1,0,0,0,0,0,0,0,0,0],[0,1,0,0,0,0,0,0,0,0,0]]C0628894	methotrexate alpha aspartate	[[0,1,0,0,0,0,0,0,0,0,0]]C0628895	methotrexate gamma methylamide	[[0,1,0,0,0,0,0,0,0,0,0]]C0055973	cm 57373,1 6 bromo 2 pyridinyl 4 piperidinamine	[[0,1,0,0,0,0,0,0,0,0,0],[0,1,0,0,0,0,0,0,0,0,0]]C0055972	cm 40907,1 6 2 chlorophenyl 3 pyridazinyl 4 piperidinol	[[0,1,0,0,0,0,0,0,0,0,0],[0,1,0,0,0,0,0,0,0,0,0]]C0889480	isomer carazolol	[[0,1,0,0,0,0,0,0,0,0,0]]C0889481	isomer carazolol	[[0,1,0,0,0,0,0,0,0,0,0]]C0889486	isomer cucurbitine	[[0,1,0,0,0,0,0,0,0,0,0]]C0055976	cm 6606,5 oxide 3 chloro 8 methoxy n n dimethyl 11h indolo 3 2 c quinoline 11 propanamine	[[0,1,0,0,0,0,0,0,0,0,0],[0,1,0,0,0,0,0,0,0,0,0]]C0055975	cm 57755	[[0,1,0,0,0,0,0,0,0,0,0]]C0055974	cm 57493,3 6 dihydro 4 3 trifluoromethyl phenyl 1 2h pyridinepropanenitrile	[[0,1,0,0,0,0,0,0,0,0,0],[0,1,0,0,0,0,0,0,0,0,0]]C0614795	7 oxo 15 methylprostaglandin e1 methyl ester	[[0,1,0,0,0,0,0,0,0,0,0]]C2586088	oral form lansoprazole	[[1,0,0,0,0,0,0,0,0,0,0]]C2699181	gastomax	[[0,0,0,0,0,0,0,0,0,1,0]]C0003277	anticholesteremic agents,cholesterol inhibitors,antihypercholesterolemic agent,anticholesteremics,anticholesteremic drugs,cholesterol lowering drug	[[0,1,0,0,0,0,1,0,0,0,0],[0,1,0,0,0,0,1,0,0,0,0],[0,0,0,0,0,0,0,0,1,0,0],[0,1,0,0,0,0,1,0,0,0,0],[0,1,0,0,0,0,0,0,0,0,0],[0,0,0,0,0,0,1,0,0,0,0]]C0003276	anticestodal agents	[[0,1,0,0,0,0,0,0,0,0,0]]C1577012	sandoz brand of amiodarone	[[0,0,0,0,0,0,0,1,0,0,0]]C2348596	sulfamazone	[[0,0,0,0,0,0,0,0,0,1,0]]C2348597	sulfametomidine	[[0,0,0,0,0,0,0,0,0,1,0]]C2348590	succisulfone	[[0,0,0,0,0,0,0,0,0,1,0]]C2348591	sulazepam	[[0,0,0,0,0,0,0,0,0,1,0]]C2348592	sulazuril	[[0,0,0,0,0,0,0,0,0,1,0]]C2348593	sulbentine	[[0,0,0,0,0,0,0,0,0,1,0]]C0760381	2 nitroimidazole acetamide	[[0,1,0,0,0,0,0,0,0,0,0]]C0760383	n 1 2 hydroxyethyl n 1 methyl 2 2 nitro 1h 1 imidazolyl acetamide	[[0,1,0,0,0,0,0,0,0,0,0]]C0089579	1 n ac 1 2 4 cl phe 3 trp 6 arg 10 alanh2 lhrh	[[0,1,0,0,0,0,0,0,0,0,0]]C0760385	tx 1877,tx1877	[[0,1,0,0,0,0,0,0,0,0,0],[0,1,0,0,0,0,0,0,0,0,0]]C0671540	iem 1460	[[0,1,0,0,0,0,0,0,0,0,0]]C0612313	4 chlorophenylalanine ethyl ester,ethyl 4 chlorophenylalanine,para chlorophenylalanine ethyl ester	[[0,1,0,0,0,0,0,0,0,0,0],[0,1,0,0,0,0,0,0,0,0,0],[0,1,0,0,0,0,0,0,0,0,0]]C1517799	folix	[[0,0,0,0,1,0,0,0,0,0,0]]C1517798	foliplus	[[0,0,0,0,1,0,0,0,0,0,0]]C0888048	glycinexylidide monohydrochloride	[[0,1,0,0,0,0,0,0,0,0,0]]C2698799	phthalylsulfamethizole	[[0,0,0,0,0,0,0,0,0,1,0]]C1517793	folidan	[[0,0,0,0,1,0,0,0,0,0,0]]C0296215	bibp 3226,bibp3226	[[0,1,0,0,0,0,0,0,0,0,0],[0,1,0,0,0,0,0,0,0,0,0]]C1517791	folaxin	[[0,0,0,0,1,0,0,0,0,0,0]]C1517790	folaren	[[0,0,0,0,1,0,0,0,0,0,0]]C1517797	folinvit	[[0,0,0,0,1,0,0,0,0,0,0]]C1517796	folinoral	[[0,0,0,0,1,0,0,0,0,0,0]]C1517795	folinac	[[0,0,0,0,1,0,0,0,0,0,0]]C0249587	sfn 70	[[0,1,0,0,0,0,0,0,0,0,0]]C3256861	propylene glycol monopalmitostearate	[[0,0,1,0,0,0,0,0,0,0,0]]C2584997	parenteral form calcitriol	[[1,0,0,0,0,0,0,0,0,0,0]]C3256863	prunella vulgaris extract,prunella vulgaris	[[0,0,0,0,0,1,0,0,0,0,0],[0,0,1,0,0,0,0,0,0,0,0]]C0055179	cgp 20308,2 1 1 dimethylethyl 6 isothiocyanato 5 methoxy benzothiazole	[[0,1,0,0,0,0,0,0,0,0,0],[0,1,0,0,0,0,0,0,0,0,0]]C2584992	parenteral form phytomenadione,parenteral form phytonadione	[[1,0,0,0,0,0,0,0,0,0,0],[1,0,0,0,0,0,0,0,0,0,0]]C2699188	chloroprednisone acetate	[[0,0,0,0,0,0,0,0,0,1,0]]C3256867	prunus serotina fruit	[[0,0,1,0,0,0,0,0,0,0,0]]C3256866	prunus mume fruit	[[0,0,1,0,0,0,0,0,0,0,0]]C3256869	sodium acrylate sodium acryloyldimethyltaurate copolymer	[[0,0,1,0,0,0,0,0,0,0,0]]C2702405	taxodium distichum pollen	[[0,0,1,0,0,0,0,0,0,1,0]]C2699189	chloroserpidine	[[0,0,0,0,0,0,0,0,0,1,0]]C1436119	etioven,cassenne brand of naftazone	[[0,1,0,0,0,0,0,0,0,0,0],[0,1,0,0,0,0,0,0,0,0,0]]C0656138	saz vii 22,3 4 chlorobenzoyl 7 1 methylethyl 3 7 diazabicyclo 3 3 1 nonane	[[0,1,0,0,0,0,0,0,0,0,0],[0,1,0,0,0,0,0,0,0,0,0]]C0769432	3 amino 2 hydroxy 5 methylhexanoyl prolyl prolyl alaninamide,ahmc6 pro pro ala nh2	[[0,1,0,0,0,0,0,0,0,0,0],[0,1,0,0,0,0,0,0,0,0,0]]C0617040	4 aminopyrazolopyrimidine 2 deoxyribofuranoside,4 amino 1 2 deoxypentofuranosyl 1h pyrazolo 3 4 d pyrimidine,4 aminopyrazolo 3 4 d pyrimidine 2 deoxyriboside,2 deoxy 4 aminopyrazolopyrimidineribofuranoside,pyrazolo 3 4 d pyrimidin 4 amine n 9 2 deoxyribofuranoside,8 aza 7 deazaadenosine	[[0,1,0,0,0,0,0,0,0,0,0],[0,1,0,0,0,0,0,0,0,0,0],[0,1,0,0,0,0,0,0,0,0,0],[0,1,0,0,0,0,0,0,0,0,0],[0,1,0,0,0,0,0,0,0,0,0],[0,1,0,0,0,0,0,0,0,0,0]]C0769434	2 n 3 pyridylsulfonyl amino 3 2 carbonyl 5 2 piperidin 4 yl ethyl thieno 2 3 b thiopheneyl amino propionic acid,2 pacpet tapa	[[0,1,0,0,0,0,0,0,0,0,0],[0,1,0,0,0,0,0,0,0,0,0]]C0769436	4 hydroxydesazadesferrithiocin,4 5 dihydro 2 2 4 dihydroxyphenyl 4 methylthiazole 4 carboxylic acid,4 5 dihydro 2 2 hydroxy 4 hydroxyphenyl 4 methylthiazole 4 carboxylic acid	[[0,1,0,0,0,0,0,0,0,0,0],[0,1,0,0,0,0,0,0,0,0,0],[0,1,0,0,0,0,0,0,0,0,0]]C0769438	4 5 dihydro 2 2 4 dihydroxphenyl thiazole 4 carboxylic acid,4 5 dihydro dptca	[[0,1,0,0,0,0,0,0,0,0,0],[0,1,0,0,0,0,0,0,0,0,0]]C0656136	n 1 isopropyl n 2 4 hydroxybenzoyl 3 7 diazabicyclo 3 3 1 nonane	[[0,1,0,0,0,0,0,0,0,0,0]]C2954428	safetussin dm	[[0,0,0,0,0,0,0,1,0,0,0]]C0288797	mazapertine succinate,compd with 1 3 4 2 1 methylethoxy phenyl 1 piperazinyl methyl benzoyl piperidine 1 1 butanedioic acid	[[0,1,0,0,0,0,0,0,0,0,0],[0,1,0,0,0,0,0,0,0,0,0]]C0724515	abacavir sulfate,abacavir sulphate	[[1,0,0,0,0,0,0,1,0,1,0],[1,0,0,0,0,0,0,0,0,0,0]]C0724516	adiastatic barley malt extract	[[0,0,0,0,0,0,0,1,0,0,0]]C0724517	alatrofloxacin mesylate	[[0,1,0,0,0,0,0,1,0,1,0]]C0724510	zymase,organon brand 3 of pancrelipase	[[0,0,0,0,0,0,0,1,0,0,0],[0,1,0,0,0,0,0,0,0,0,0]]C0288792	tazarotene,ethyl ester 6 3 4 dihydro 4 4 dimethyl 2h 1 benzothiopyran 6 yl ethynyl 3 pyridinecarboxylic acid,ethyl 6 2 4 4 dimethylthiochroman 6 yl ethynyl nicotinate	[[0,0,0,0,0,0,0,1,0,1,0],[0,0,0,0,0,0,0,0,0,1,1],[0,0,0,0,0,0,0,0,0,1,1]]C2926894	dairyland brand	[[0,0,0,0,0,0,0,1,0,0,0]]C0723084	rifadin iv	[[0,0,0,0,0,0,0,1,0,0,0]]C3162856	ena 28 day	[[0,0,0,0,0,1,0,0,0,0,0]]C3162854	dificid	[[0,0,0,0,0,0,0,1,0,0,0]]C2929544	acetaminophen atropine chlorpheniramine phenylephrine	[[0,0,0,0,0,0,0,1,0,0,0]]C0148293	venopyronum	[[0,1,0,0,0,0,0,0,0,0,0]]C1529439	acemetacin intermuti,intermuti brand of acemetacin	[[0,1,0,0,0,0,0,0,0,0,0],[0,1,0,0,0,0,0,0,0,0,0]]C1529438	acemetacin stada,stada brand of acemetacin	[[0,1,0,0,0,0,0,0,0,0,0],[0,1,0,0,0,0,0,0,0,0,0]]C0648708	malabaricone b,1 2 6 dihydroxyphenyl 9 4 hydroxyphenyl 1 nonanone	[[0,1,0,0,0,0,0,0,0,0,0],[0,1,0,0,0,0,0,0,0,0,0]]C0648709	dynemicin q	[[0,1,0,0,0,0,0,0,0,0,0]]C0639303	tensophoril,boric acid h3bo3 and 5 ethyl 5 3 methylbutyl 2 4 6 1h 3h 5h pyrimidinetrione mixt with 4 2 aminoethyl 1 2 benzenediol hydrochloride l ascorbic acid	[[0,1,0,0,0,0,0,0,0,0,0],[0,1,0,0,0,0,0,0,0,0,0]]C0148292	venopyridum	[[0,1,0,0,0,0,0,0,0,0,0]]C1529433	oldan,europharma brand of acemetacin	[[0,1,0,0,0,0,0,0,0,0,0],[0,1,0,0,0,0,0,0,0,0,0]]C1529432	espledol,fher brand of acemetacin	[[0,1,0,0,0,0,0,0,0,0,0],[0,1,0,0,0,0,0,0,0,0,0]]C1529431	gerbin,icn brand of aceclofenac,gerbin difucrem	[[0,1,0,0,0,0,0,0,0,0,0],[0,1,0,0,0,0,0,0,0,0,0],[0,1,0,0,0,0,0,0,0,0,0]]C0628275	patulolide b,r z 12 methyl oxacyclododec 3 ene 2 5 dione	[[0,1,0,0,0,0,0,0,0,0,0],[0,1,0,0,0,0,0,0,0,0,0]]C0648700	rhc 5901,rhc5901	[[0,1,0,0,0,0,0,0,0,0,0],[0,1,0,0,0,0,0,0,0,0,0]]C0075686	synalar n	[[0,1,0,0,0,0,0,1,0,0,0]]C1529435	acephlogont,azupharma brand of acemetacin	[[0,1,0,0,0,0,0,0,0,0,0],[0,1,0,0,0,0,0,0,0,0,0]]C0075684	symphytine,1r 1alpha e 7 2s 3s 7abeta 7 2 3 dihydroxy 2 1 methylethyl 1 oxobutoxy methyl 2 3 5 7a tetrahydro 1h pyrrolizin 1 yl ester 2 methyl 2 butenoic acid	[[0,1,0,0,0,0,0,0,0,0,0],[0,1,0,0,0,0,0,0,0,0,0]]C0106589	bl 194	[[0,1,0,0,0,0,0,0,0,0,0]]C2928498	benzocaine zirconium oxide	[[0,0,0,0,0,0,0,1,0,0,0]]C2928491	calcium phosphate cholecalciferol soybean preparation	[[0,0,0,0,0,0,0,1,0,0,0]]C2928490	dimethicone miconazole zinc oxide	[[0,0,0,0,0,0,0,1,0,0,0]]C2928493	folic acid vitamin b 12	[[0,0,0,0,0,0,0,1,0,0,0]]C2928492	acetaminophen codeine guaifenesin pseudoephedrine	[[0,0,0,0,0,0,0,1,0,0,0]]C2928495	domperidone ranitidine	[[0,0,0,0,0,0,0,1,0,0,0]]C2928494	thiamine vitamin b 12	[[0,0,0,0,0,0,0,1,0,0,0]]C0106587	bk 129	[[0,1,0,0,0,0,0,0,0,0,0]]C2928496	lidocaine polymyxin b	[[0,0,0,0,0,0,0,1,0,0,0]]C2343682	stanback headache powder	[[0,0,0,0,0,0,0,1,0,0,0]]C0721559	mapap sinus	[[0,0,0,0,0,0,0,1,0,0,0]]C0540134	ru bpy 2 deabpy pf6 2	[[0,1,0,0,0,0,0,0,0,0,0]]C0721558	mapap pm	[[0,0,0,0,0,0,0,1,0,0,0]]C0875944	phenylephrine pyrilamine	[[0,0,0,0,0,0,0,1,0,0,0]]C0875947	sodium ferric gluconate complex,sodium ferric gluconate,ferric na gluconate	[[0,0,0,0,0,0,1,1,0,1,0],[0,0,0,0,0,0,1,0,0,0,0],[0,0,0,0,0,0,1,0,0,0,1]]C1725664	ferrous asparto glycinate	[[0,0,1,0,0,0,0,0,0,0,0]]C0103075	ammonium hydrogen phosphate	[[0,1,0,0,0,0,0,0,0,0,0]]C0971581	21 3 methyl 3 hydroxybutyl 19 norvitamin d3,21 3 methyl 3 hydroxy butyl 19 nor d 3,gemini 19 nor	[[0,1,0,0,0,0,0,0,0,0,0],[0,1,0,0,0,0,0,0,0,0,0],[0,1,0,0,0,0,0,0,0,0,0]]C2929858	isothipendyl pipazethate	[[0,0,0,0,0,0,0,1,0,0,0]]C1579451	doxepin biomo,doxepinbiomo,biomo brand of doxepin hydrochloride	[[0,1,0,0,0,0,0,0,0,0,0],[0,1,0,0,0,0,0,0,0,0,0],[0,1,0,0,0,0,0,0,0,0,0]]C0602760	3 2 amino 2 carboxyethyl 3 8 xanthinyl indolenine,alpha amino 3 2 3 6 7 tetrahydro 2 6 dioxo 1h purin 8 yl 3h indole 3 propanoic acid	[[0,1,0,0,0,0,0,0,0,0,0],[0,1,0,0,0,0,0,0,0,0,0]]C2934194	bi 1744 cl	[[0,1,0,0,0,0,0,0,0,0,0]]C0602764	2 amino 2 deoxy 5 fluorouridine	[[0,1,0,0,0,0,0,0,0,0,0]]C1579452	doxepin stada,stadapharm brand of doxepin hydrochloride	[[0,1,0,0,0,0,0,0,0,0,0],[0,1,0,0,0,0,0,0,0,0,0]]C0102805	als 1249	[[0,1,0,0,0,0,0,0,0,0,0]]C0256765	palmitoylcarnitine isopropyl ester,p1pi ester	[[0,1,0,0,0,0,0,0,0,0,0],[0,1,0,0,0,0,0,0,0,0,0]]C0646758	de n methyl 8 9 anhydroerythromycin a 6 9 hemicetal	[[0,1,0,0,0,0,0,0,0,0,0]]C0256766	4 hydroxyamesergide	[[0,1,0,0,0,0,0,0,0,0,0]]C0256763	chf 2206	[[0,1,0,0,0,0,0,0,0,0,0]]C0256762	4 phenyl 3 phenylsulfonylfuroxan	[[0,1,0,0,0,0,0,0,0,0,0]]C0036579	selegiline,selegyline,l deprenyl,deprenil,r n alpha dimethyl n 2 propynyl benzeneethanamine,r isomer selegiline,phenylisopropylmethylpropynylamine	[[0,0,0,0,0,0,1,1,0,1,0],[0,0,0,0,0,0,0,0,0,0,1],[0,0,0,0,0,0,1,0,0,1,1],[0,0,0,0,0,0,0,0,0,1,0],[0,0,0,0,0,0,0,0,0,0,1],[0,0,0,0,0,0,0,0,0,0,1],[0,0,0,0,0,0,0,0,0,1,0]]C0646752	acetylhistidyl lysyl aspartyl methionyl glutaminyl leucyl glycyl arginine,ac his lys asp met gln leu gly arg oh,hlamglga	[[0,1,0,0,0,0,0,0,0,0,0],[0,1,0,0,0,0,0,0,0,0,0],[0,1,0,0,0,0,0,0,0,0,0]]C0646755	n 1 benzyl 4 piperidinyl 4 n methyl n 3 4 methylsulfonyl phenyl 2 propenoyl amino benzenesulfonamide	[[0,1,0,0,0,0,0,0,0,0,0]]C0069402	oleogels	[[0,1,0,0,0,0,0,0,0,0,0]]C0646757	me 34	[[0,1,0,0,0,0,0,0,0,0,0]]C0646756	er 3826	[[0,1,0,0,0,0,0,0,0,0,0]]C2929859	ascorbic acid polysaccharide iron complex	[[0,0,0,0,0,0,0,1,0,0,0]]C0069403	oleomorphocycline	[[0,1,0,0,0,0,0,0,0,0,0]]C0083521	n 4 hydroxy 5 fluorodeoxycytidine monophosphate,n 4 hydroxy 5 fluoro dcmp,n 4 oh 5 fdcmp	[[0,1,0,0,0,0,0,0,0,0,0],[0,1,0,0,0,0,0,0,0,0,0],[0,1,0,0,0,0,0,0,0,0,0]]C0721557	mapap junior strength	[[0,0,0,0,0,0,0,1,0,0,0]]C2756312	pennsaid	[[0,0,0,0,0,0,0,1,0,0,0]]C2240799	hypoglycemic agents other	[[0,0,0,0,0,0,0,0,0,0,1]]C2240798	calcium magnesium vitamin d zinc	[[0,0,0,0,0,0,0,0,0,0,1]]C0616394	ara 3 deazaadenine	[[0,1,0,0,0,0,0,0,0,0,0]]C1511792	enhanzyn	[[0,0,0,0,1,0,0,0,0,0,0]]C0616390	beta 1 2 dimethyl 4 phenyl 4 propionyloxy piperidine	[[0,1,0,0,0,0,0,0,0,0,0]]C1270884	theophylline m r	[[1,0,0,0,0,0,0,0,0,0,0]]C0616392	1 2 dimethyl 4 phenyl 4 propionyloxy piperidine,dmppop,propanoate 1 2 dimethyl 4 phenyl 4 piperidinol	[[0,1,0,0,0,0,0,0,0,0,0],[0,1,0,0,0,0,0,0,0,0,0],[0,1,0,0,0,0,0,0,0,0,0]]C0616393	1 beta d arabinofuranosyl 1h imidazo 4 5 c pyridine	[[0,1,0,0,0,0,0,0,0,0,0]]C1270888	parenteral form clonidine	[[1,0,0,0,0,0,0,0,0,0,0]]C0616398	bag 0177	[[0,1,0,0,0,0,0,0,0,0,0]]C0616399	disulfate bis n ethylidene vindesine disulfide	[[0,1,0,0,0,0,0,0,0,0,0]]C2927368	a perth 16 2009 h3n2 strain live attenuated influenza virus vaccine	[[0,0,0,0,0,0,0,1,0,0,0]]C2317059	interleukin 1 receptor blocking agent	[[1,0,0,0,0,0,0,0,0,0,0]]C0762526	n methylniphimycin	[[0,1,0,0,0,0,0,0,0,0,0]]C1622506	beta acetyldigoxin drug combination dilazep,dilazep beta acetyldigoxin	[[0,1,0,0,0,0,0,0,0,0,0],[0,1,0,0,0,0,0,0,0,0,0]]C0733859	otrivin,novartis brand of xylometazoline hydrochloride,otrivin mentol,otriven	[[0,1,0,0,0,0,1,0,0,0,0],[0,1,0,0,0,0,0,0,0,0,0],[0,1,0,0,0,0,0,0,0,0,0],[0,1,0,0,0,0,0,0,0,0,0]]C0733858	novorin	[[0,1,0,0,0,0,0,0,0,0,0]]C0457610	measles rubella vaccine	[[0,0,0,0,0,1,0,0,0,0,0]]C0733850	levoxine	[[0,1,0,0,0,0,1,0,0,0,0]]C0733853	visine,pfizer consumer healthcare brand of tetrahydrozoline hydrochloride,yxin,pfizer brand of tetrahydrozoline hydrochloride	[[0,0,0,0,0,0,1,0,0,1,0],[0,1,0,0,0,0,0,0,0,0,0],[0,1,0,0,0,0,0,0,0,0,0],[0,1,0,0,0,0,0,0,0,0,0]]C0000190	2 cmp	[[0,1,0,0,0,0,0,0,0,0,0]]C0733855	idulian	[[0,1,0,0,0,0,0,0,0,0,0]]C0733854	intralipid,phospholipid emulsion clintec brand of soybean oil	[[0,1,0,0,0,0,1,0,0,0,0],[0,1,0,0,0,0,0,0,0,0,0]]C2918441	theomax	[[0,0,0,0,0,0,0,1,0,0,0]]C0001822	agovirin	[[0,1,0,0,0,0,0,0,0,0,0]]C1330421	nifediac cc	[[0,0,0,0,0,0,0,1,0,0,0]]C1997871	parenteral form lornoxicam	[[1,0,0,0,0,0,0,0,0,0,0]]C2698715	vasospan	[[0,0,0,0,0,0,0,0,0,1,0]]C0531104	lumefantrine,benflumetol,z 2 7 dichloro 9 4 chlorophenyl methylene alpha dibutylamino methyl 9h fluorene 4 methanol	[[1,0,0,0,0,0,0,1,0,0,0],[1,0,0,0,0,0,1,0,0,0,0],[0,1,0,0,0,0,0,0,0,0,0]]C0676297	fx 2212a	[[0,1,0,0,0,0,0,0,0,0,0]]C0540135	4 4 diethylaminomethyl 2 2 bipyridine bis 2 2 bipyridine ruthenium ii	[[0,1,0,0,0,0,0,0,0,0,0]]C0063327	idazoxan	[[0,0,0,0,0,0,0,0,0,0,1]]C2742568	filicene	[[0,1,0,0,0,0,0,0,0,0,0]]C0676298	fx 2212	[[0,1,0,0,0,0,0,0,0,0,0]]C0063326	idarubicinol,13 dihydroidarubicin,4 demethoxy 14 hydroxydaunorubicin	[[0,1,0,0,0,0,0,0,0,0,0],[0,1,0,0,0,0,0,0,0,0,0],[0,1,0,0,0,0,0,0,0,0,0]]C1707944	femergin	[[0,0,0,0,0,0,0,0,0,1,0]]C2699262	salcaprozate sodium,snac sodium,sodium 8 2 hydroxybenzoyl amino octanoate	[[0,1,0,0,0,0,0,0,0,1,0],[0,1,0,0,0,0,0,0,0,0,0],[0,0,0,0,0,0,0,0,0,1,0]]C2699260	saccharin sodium anhydrous	[[0,0,0,0,0,0,0,0,0,1,0]]C0527030	bishofit	[[0,1,0,0,0,0,0,0,0,0,0]]C0011777	dexamethasone,hexadecadrol,methylfluorprednisolone,11beta 16alpha 9 fluoro 11 17 21 trihydroxy 16 methyl pregna 1 4 diene 3 20 dione,dexamethasone preparation,dxm,dm,11beta 16alpha 9 fluoro 11 17 21 trihydroxy 16 methylpregna 1 4 diene 3 20 dione,9alpha fluoro 11beta 17alpha 21 trihydroxy 16alpha methylpregna 1 4 diene 3 20 dione,16alpha methyl 9alpha fluoro 1 4 pregnadiene 11beta 17alpha 21 triol 3 20 dione,1 dehydro 16alpha methyl 9alpha fluorohydrocortisone,16alpha methyl 9alpha fluoro delta1 hydrocortisone,fluoro 9alpha methyl 16alpha prednisolone,9alpha fluoro 16alpha methylprednisolone,16alpha methyl 9alpha fluoroprednisolone,desamethasone,dexamethasonum	[[0,1,0,0,0,0,1,0,0,1,0],[0,0,0,0,0,0,0,0,0,1,0],[0,0,0,0,1,0,0,0,0,0,0],[0,0,0,0,0,0,0,0,0,0,1],[1,0,0,0,0,0,0,0,0,0,0],[0,0,0,0,1,0,0,0,0,0,0],[0,0,0,0,1,0,0,0,0,0,0],[0,0,0,0,1,0,0,0,0,0,0],[0,0,0,0,1,0,0,0,0,0,0],[0,0,0,0,1,0,0,0,0,0,0],[0,0,0,0,1,0,0,0,0,0,0],[0,0,0,0,1,0,0,0,0,0,0],[0,0,0,0,1,0,0,0,0,0,0],[0,0,0,0,1,0,0,0,0,0,0],[0,0,0,0,1,0,0,0,0,0,0],[0,0,0,0,0,0,0,0,0,1,0],[0,0,0,0,0,0,0,0,0,1,0]]C2979375	inzo	[[0,0,0,0,0,0,0,1,0,0,0]]C0064112	itazigrel,4 5 bis 4 methoxyphenyl 2 trifluoromethyl thiazole,4 5 bmtt	[[0,1,0,0,0,0,0,0,0,0,0],[0,1,0,0,0,0,0,0,0,0,0],[0,1,0,0,0,0,0,0,0,0,0]]C0527038	isopropyl unoprostone,isopropyl 20 ethyl 9alpha 11alpha dihydroxy 15 keto cis delta 5 prostanoate,5 s 3 5 dihydroxy 2 3 oxodecyl cyclopentyl 5 heptenoate 3 r 2 r isopropyl z 7 1 r	[[1,0,0,0,0,0,0,1,0,1,1],[0,0,0,0,0,0,0,0,0,0,1],[0,0,0,0,0,0,0,0,0,1,0]]C0630617	ac 7230,ac7230	[[0,1,0,0,0,0,0,0,0,0,0],[0,1,0,0,0,0,0,0,0,0,0]]C2316909	oropharyngeal form cocaine	[[1,0,0,0,0,0,0,0,0,0,0]]C0251234	n n diacetyl beta chitobiosyl allosamizoline	[[0,1,0,0,0,0,0,0,0,0,0]]C0251235	n n diacetylchitobiosyl allosamizoline	[[0,1,0,0,0,0,0,0,0,0,0]]C0251233	2 dimethylamino 4 hydroxy 6 hydroxymethyl 3a 5 6 6a tetrahydro 4h cyclopentoxazol 5 yl 2 acetamido 4 o 2 acetamido 2 deoxyglucopyranosyl 2 deoxyglucopyranoside	[[0,1,0,0,0,0,0,0,0,0,0]]C0307059	nostril nasal decongestant	[[0,0,0,0,0,0,0,1,0,0,0]]C0630611	carbazomycin d,3 4 6 trimethoxy 1 2 dimethyl 9h carbazole,3 4 6 trimethoxy 1 2 dimethylcarbazole	[[0,1,0,0,0,0,0,0,0,0,0],[0,1,0,0,0,0,0,0,0,0,0],[0,1,0,0,0,0,0,0,0,0,0]]C0647087	4 methoxyphenyl 5 methyl 6 2 4 morpholinyl ethyl 6h thieno 2 3 b pyrrol 4 yl phenylmethanone,4 mmmtpp	[[0,1,0,0,0,0,0,0,0,0,0],[0,1,0,0,0,0,0,0,0,0,0]]C0251238	c111 peptide,gly val tyr pro his lys,glycyl valyl tyrosyl prolyl histidyl lysine	[[0,1,0,0,0,0,0,0,0,0,0],[0,1,0,0,0,0,0,0,0,0,0],[0,1,0,0,0,0,0,0,0,0,0]]C0647085	6 deoxocyprodime,n cyclopropylmethyl 4 14 dimethoxymorphinan	[[0,1,0,0,0,0,0,0,0,0,0],[0,1,0,0,0,0,0,0,0,0,0]]C0530162	1 5 methanesulfonamidoindol 2 yl carbonyl 4 n methyl n 3 1 1 dimethylethyl amino 2 pyridinyl amino piperidine,1 msai pap	[[0,1,0,0,0,0,0,0,0,0,0],[0,1,0,0,0,0,0,0,0,0,0]]C2936726	promethazine drug combination codeine atropine aminopyrine,alkosin,aminopyrine atropine codeine promethazine	[[0,1,0,0,0,0,0,0,0,0,0],[0,1,0,0,0,0,0,0,0,0,0],[0,1,0,0,0,0,0,0,0,0,0]]C1991761	lormetazepam 124 urine	[[0,0,0,1,0,0,0,0,0,0,0]]C2936728	p aminobenzoyl n diethyl leucinol drug combination dihydroergotoxin,dihydroergotoxin p aminobenzoyl n diethyl leucinol	[[0,1,0,0,0,0,0,0,0,0,0],[0,1,0,0,0,0,0,0,0,0,0]]C2936729	reserpine drug combination methyldopa mefrusid,mefrusid methyldopa reserpine	[[0,1,0,0,0,0,0,0,0,0,0],[0,1,0,0,0,0,0,0,0,0,0]]C0256877	n alpha benzoyl dab gamma nbd ala d trp phe d pro pro nle nh2	[[0,1,0,0,0,0,0,0,0,0,0]]C2935264	4 n n dimethylcarbamoyl oxy 5 naphth 1 yl naphtho 2 3 b pyrrolo 1 2 d 1 4 oxazepine	[[0,1,0,0,0,0,0,0,0,0,0]]C2935265	pbox 16	[[0,1,0,0,0,0,0,0,0,0,0]]C0809889	dimaval,heyl brand of unithiol,dmps heyl	[[0,1,0,0,0,0,0,0,0,0,0],[0,1,0,0,0,0,0,0,0,0,0],[0,1,0,0,0,0,0,0,0,0,0]]C2935262	3 4 aminophenyl 2h chromen 2 one,case myelin compound	[[0,1,0,0,0,0,0,0,0,0,0],[0,1,0,0,0,0,0,0,0,0,0]]C0604331	lysine chloromethyl ketone,1 chloro 3 7 diaminoheptan 2 one	[[0,1,0,0,0,0,0,0,0,0,0],[0,1,0,0,0,0,0,0,0,0,0]]C0604333	alpha 6 8 dichloronaphtho 2 1 b thien 4 yl 1 piperidineethanol,alpha 6 8 dichloronaphtho 2 1 b thien 4 yl 1 piperidineethanol hydrochloride	[[0,1,0,0,0,0,0,0,0,0,0],[0,1,0,0,0,0,0,0,0,0,0]]C0604335	kc 18	[[0,1,0,0,0,0,0,0,0,0,0]]C2358709	rifabutin ethambutol 124 isolate	[[0,0,0,1,0,0,0,0,0,0,0]]C0604337	cleves acid,8 aminonaphthalene 2 sulfonic acid	[[0,1,0,0,0,0,0,0,0,0,0],[0,1,0,0,0,0,0,0,0,0,0]]C0809887	f 6066,f6066	[[0,1,0,0,0,0,0,0,0,0,0],[0,1,0,0,0,0,0,0,0,0,0]]C0671115	1 3 4 5 trimethoxybenzoyl 3 2 4 1 2 ethoxyethyl 1h benzimidazol 2 yl 1 4 diazepan 1 yl ethyl 3 phenylpyrrolidine dihydrochloride	[[0,1,0,0,0,0,0,0,0,0,0]]C0671117	mdl 108 207da	[[0,1,0,0,0,0,0,0,0,0,0]]C1831893	bms 690514	[[0,0,0,0,1,0,0,0,0,0,0]]C1831892	bms 641988,bms641988	[[0,1,0,0,1,0,0,0,0,0,0],[0,1,0,0,0,0,0,0,0,0,0]]C1831891	morab 009	[[0,0,0,0,1,0,0,0,0,0,0]]C0043339	xenon,xe xenon,xe,xe element	[[0,0,0,0,0,0,0,0,1,1,0],[1,0,0,0,0,0,1,0,0,0,0],[0,0,0,0,0,0,0,0,0,1,0],[0,0,0,0,0,0,0,0,1,0,0]]C1831896	camptothecin polymer conjugate it 101,cyclodextrin based polymer camptothecin crlx101	[[0,0,0,0,1,0,0,0,0,1,0],[0,0,0,0,0,0,0,0,0,1,0]]C2698430	nardeterol	[[0,0,0,0,0,0,0,0,0,1,0]]C1831894	arginine omega 3 fatty acids nucleotides oral supplement	[[0,0,0,0,1,0,0,0,0,1,0]]C1568504	2 4 di cl cbz val asp fmk,mx1122	[[0,1,0,0,0,0,0,0,0,0,0],[0,1,0,0,0,0,0,0,0,0,0]]C1568505	astaxanthin dilysinate tetrahydrochloride,di l lysinate lys 2 ast	[[0,1,0,0,0,0,0,0,0,0,0],[0,1,0,0,0,0,0,0,0,0,0]]C1831899	alpharadin	[[0,0,0,0,1,0,0,0,0,0,0]]C1831898	radium 223 based radiopharmaceutical,radium 223,radium chloride ra 223,radium 223 chloride	[[0,0,0,0,1,0,0,0,0,1,0],[0,0,0,0,0,0,0,0,0,1,0],[0,0,0,0,0,0,0,0,0,1,0],[0,0,0,0,0,0,0,0,0,1,0]]C0764965	cp 265298,cp 265 298	[[0,1,0,0,0,0,0,0,0,0,0],[0,1,0,0,0,0,0,0,0,0,0]]C0174156	swormlure 4	[[0,1,0,0,0,0,0,0,0,0,0]]C0764967	cp 288886	[[0,1,0,0,0,0,0,0,0,0,0]]C0291457	as 8	[[0,1,0,0,0,0,0,0,0,0,0]]C0381784	1 aminoindan 1 5 dicarboxylic acid,1 ai 1 5 dca	[[0,1,0,0,0,0,0,0,0,0,0],[0,1,0,0,0,0,0,0,0,0,0]]C0958460	l gln isomer l arg methotrexate alpha arginine hydrochloride	[[0,1,0,0,0,0,0,0,0,0,0]]C0381786	1 4 7 8 9 10 hexahydro 9 methyl 6 nitropyrido 3 4 f quinoxaline 2 3 dione,9 me 6 no2 hpq	[[0,1,0,0,0,0,0,0,0,0,0],[0,1,0,0,0,0,0,0,0,0,0]]C0056092	colabomycin a,1s 1alpha 3 2e 4e 6z 8e 5beta 5 1e 3e 5e 7e 6alpha n 5 hydroxy 5 9 2 hydroxy 5 oxo 1 cyclopenten 1 yl amino 9 oxo 1 3 5 7 nonatetraenyl 2 oxo 7 oxabicyclo 4 1 0 hept 3 en 3 yl 2 4 6 8 decatetraenamide	[[0,1,0,0,0,0,0,0,0,0,0],[0,1,0,0,0,0,0,0,0,0,0]]C0958465	fast violet b tetrachlorozincate	[[0,1,0,0,0,0,0,0,0,0,0]]C0958467	fast violet b chloride	[[0,1,0,0,0,0,0,0,0,0,0]]C0958466	fast violet b trichlorozincate	[[0,1,0,0,0,0,0,0,0,0,0]]C0958469	6r 3 r 6alpha 7beta isomer cefotiam hexetil	[[0,1,0,0,0,0,0,0,0,0,0]]C0958468	6r 6alpha 7beta isomer cefotiam hexetil	[[0,1,0,0,0,0,0,0,0,0,0]]C0670869	1 phenyl 2 hexadecanoylamino 3 pyrrolidino 1 propanol,pppp cpd	[[0,1,0,0,0,0,0,0,0,0,0],[0,1,0,0,0,0,0,0,0,0,0]]C0381789	l 163958,l 163 958	[[0,1,0,0,0,0,0,0,0,0,0],[0,1,0,0,0,0,0,0,0,0,0]]C0605688	1 2 benzoylethyl 2 cinnamylpiperazinyl 1 methyl benzimidazole dimaleate	[[0,1,0,0,0,0,0,0,0,0,0]]C1099470	9 methoxystrobilurin k	[[0,1,0,0,0,0,0,0,0,0,0]]C1099477	sitamaquine,8 6 diethylamino hexyl amino 6 methoxy 4 methylquinoline,n n diethyl n 6 methoxy 4 methyl 8 quinolinyl 1 6 hexanediamine	[[0,1,0,0,0,0,0,0,0,1,0],[0,1,0,0,0,0,0,0,0,0,0],[0,1,0,0,0,0,0,0,0,0,0]]C0653267	chebulagic acid	[[0,1,0,0,0,0,0,0,0,0,0]]C2827287	nicodicodine	[[0,0,0,0,0,0,0,0,0,1,0]]C2827284	nemorubicin hydrochloride,8s 10s hydrochloride 7 8 9 10 tetrahydro 6 8 11 trihydroxy 8 hydroxyacetyl 1 methoxy 10 2 3 6 trideoxy 3 2s 2 methoxy 4 morpholinyl alpha l lyxo hexopyranosyl oxy 5 12 naphthacenedione	[[0,0,0,0,0,0,0,0,0,1,0],[0,0,0,0,0,0,0,0,0,1,0]]C2827285	nepaprazole	[[0,0,0,0,0,0,0,0,0,1,0]]C2827282	nemazoline hydrochloride,monohydrochloride 2 6 dichloro 4 4 5 dihydro 1h imidazol 2 yl methyl benzenamine,2 4 amino 3 5 dichlorobenzyl 2 imidazoline monohydrochloride	[[0,0,0,0,0,0,0,0,0,1,0],[0,0,0,0,0,0,0,0,0,1,0],[0,0,0,0,0,0,0,0,0,1,0]]C2827283	nemorubicin	[[0,0,0,0,0,0,0,0,0,1,0]]C2827280	neflumozide hydrochloride,monohydrochloride 1 1 3 6 fluoro 1 2 benzisoxazol 3 yl propyl 4 piperidinyl 1 3 dihydro 2h benzimidazol 2 one,1 1 3 6 fluoro 1 2 benzisoxazol 3 yl propyl 4 piperidyl 2 benzimidazolinone monohydrochloride	[[0,0,0,0,0,0,0,0,0,1,0],[0,0,0,0,0,0,0,0,0,1,0],[0,0,0,0,0,0,0,0,0,1,0]]C2827281	nemazoline	[[0,0,0,0,0,0,0,0,0,1,0]]C0637341	quinophthalone,2 2 quinolinyl 1h indene 1 3 2h dione	[[0,1,0,0,0,0,0,0,0,0,0],[0,1,0,0,0,0,0,0,0,0,0]]C0717561	carbinoxamine pseudoephedrine	[[0,0,0,0,0,0,0,1,0,0,0]]C2827288	nictiazem	[[0,0,0,0,0,0,0,0,0,1,0]]C2827289	nifuradene	[[0,0,0,0,0,0,0,0,0,1,0]]C1564163	spiractin,alphapharm brand of spironolactone	[[0,1,0,0,0,0,0,0,0,0,0],[0,1,0,0,0,0,0,0,0,0,0]]C3180891	methyl 6 nitroarachidonate,6 aametno2,6 methylnitroarachidonate	[[0,1,0,0,0,0,0,0,0,0,0],[0,1,0,0,0,0,0,0,0,0,0],[0,1,0,0,0,0,0,0,0,0,0]]C2946219	dephyze	[[0,0,0,0,0,0,0,1,0,0,0]]C1981334	amphetamine 124 stool	[[0,0,0,1,0,0,0,0,0,0,0]]C3180897	lirigramoside b	[[0,1,0,0,0,0,0,0,0,0,0]]C0069624	org 7268	[[0,1,0,0,0,0,0,0,0,0,0]]C0069625	org 7617,bromide 1 2beta 3alpha 5alpha 16beta 17beta 3 acetyloxy 17 1 oxobutoxy 2 1 piperidinyl androstan 16 yl 1 2 propenyl piperidinium	[[0,1,0,0,0,0,0,0,0,0,0],[0,1,0,0,0,0,0,0,0,0,0]]C0069626	org 7797,z 2 butenedioate 1 1 salt 16alpha 17beta 17 methylamino estra 1 3 5 10 triene 3 16 diol	[[0,1,0,0,0,0,0,0,0,0,0],[0,1,0,0,0,0,0,0,0,0,0]]C0069621	org 6368	[[0,1,0,0,0,0,0,0,0,0,0]]C0069622	org 6582	[[0,1,0,0,0,0,0,0,0,0,0]]C0625356	7 n benzyloxycarbonylglycyl glycyl leucyl amino 4 methylcoumarin,7 z gly gly leu me	[[0,1,0,0,0,0,0,0,0,0,0],[0,1,0,0,0,0,0,0,0,0,0]]C0543463	tevacor	[[0,1,0,0,0,0,0,0,0,0,0]]C0069628	setiptiline,1 2 3 4 tetrahydro 2 methyl 9h dibenzo 3 4 6 7 cyclohepta 1 2 c pyridine,13b 4a carba mianserin	[[0,1,0,0,0,0,0,0,0,1,0],[0,1,0,0,0,0,0,0,0,0,0],[0,1,0,0,0,0,0,0,0,0,0]]C2746107	streptococcus pneumoniae serotype 19a capsular antigen diphtheria crm197 protein conjugate vaccine,streptococcus pneumoniae type 19a capsular polysaccharide diphtheria crm197 protein conjugate antigen	[[0,0,0,0,0,0,0,1,0,0,0],[0,0,1,0,0,0,0,0,0,0,0]]C0625350	lysate brain depressor i	[[0,1,0,0,0,0,0,0,0,0,0]]C1564160	jenaspiron,jenapharm brand of spironolactone	[[0,1,0,0,0,0,0,0,0,0,0],[0,1,0,0,0,0,0,0,0,0,0]]C2698425	nafcaproic acid,nafcaproate	[[0,0,0,0,0,0,0,0,0,1,0],[0,0,0,0,0,0,0,0,0,1,0]]C2946931	sundrops 77	[[0,0,0,0,0,0,0,1,0,0,0]]C2746103	streptococcus pneumoniae serotype 1 capsular antigen diphtheria crm197 protein conjugate vaccine,streptococcus pneumoniae type 1 capsular polysaccharide diphtheria crm197 protein conjugate antigen	[[0,0,0,0,0,0,0,1,0,0,0],[0,0,1,0,0,0,0,0,0,0,0]]C2954905	sparklefresh anticavity	[[0,0,0,0,0,0,0,1,0,0,0]]C0042672	vinca alkaloids,vinca alkaloid compound	[[1,1,0,0,0,0,1,0,1,0,1],[0,0,0,0,0,0,0,0,0,1,0]]C1710430	taquidil	[[0,0,0,0,0,0,0,0,0,1,0]]C1710433	focusan	[[0,0,0,0,0,0,0,0,0,1,0]]C1710432	absorbine	[[0,0,0,0,0,0,0,0,0,1,0]]C1710434	tolsanil	[[0,0,0,0,0,0,0,0,0,1,0]]C0721269	ku zyme hp	[[0,0,0,0,0,0,0,1,0,0,0]]C0670246	ad 89 eta	[[0,1,0,0,0,0,0,0,0,0,0]]C0670247	1 4 bis 2 2 2 dimethyloxazolidin 3 yl ethylamino 1 4 didehydroxypyrromycinone,1 4 bis 2 2 2 dimethyloxazolidin 3 yl ethylamino 1 4 didehydroxy eta pyrromycinone	[[0,1,0,0,0,0,0,0,0,0,0],[0,1,0,0,0,0,0,0,0,0,0]]C0721267	kronofed a jr	[[0,0,0,0,0,0,0,1,0,0,0]]C0721266	kronofed a	[[0,0,0,0,0,0,0,1,0,0,0]]C0670242	2 phenyl 3 3a dihydro 4 oxo 5 4 4 fluorophenyl piperazin 1 yl methylpyrazolo 1 5 d 1 2 4 trazine	[[0,1,0,0,0,0,0,0,0,0,0]]C0721260	kophane	[[0,0,0,0,0,0,0,1,0,0,0]]C0721263	koromex cream	[[0,0,0,0,0,0,0,1,0,0,0]]C0670241	sm3 fluor	[[0,1,0,0,0,0,0,0,0,0,0]]C0207761	schisantherin a,gomisin c	[[0,1,0,0,0,0,0,0,0,0,0],[0,1,0,0,0,0,0,0,0,0,0]]C0207760	cyclofem	[[0,1,0,0,0,0,0,0,0,0,0]]C1608551	fluoxetine olanzapine,olanzapine fluoxetine combination	[[1,0,0,0,0,0,0,1,0,0,1],[0,1,0,0,0,0,0,0,0,0,0]]C1130476	etophos	[[0,1,0,0,0,0,0,0,0,0,0]]C0044643	10 11 dehydroimipramine,5 3 dimethylamino propyl 5h dibenz b f azepine,depramine	[[0,1,0,0,0,0,0,0,0,0,0],[0,1,0,0,0,0,0,0,0,0,0],[0,1,0,0,0,0,0,0,0,1,0]]C1869570	tridentate n n n n n pentamethyldiethylenetriamine,t pmdeta cpd	[[0,1,0,0,0,0,0,0,0,0,0],[0,1,0,0,0,0,0,0,0,0,0]]C0044641	10 10 dimethyl 10 deazaaminopterin,n 4 2 2 4 diamino 6 pteridinyl 1 1 dimethylethyl benzoyl l glutamic acid,10 10 dimethyl 10 deaza aminopterin	[[0,1,0,0,0,0,0,0,0,0,0],[0,1,0,0,0,0,0,0,0,0,0],[0,1,0,0,0,0,0,0,0,0,0]]C0044647	10 11 methylenedioxy n propylnoraporphine,r 6a 7 8 9 tetrahydro 7 propyl 6h benzo de 1 3 benzodioxolo 4 5 g quinoline,mdo npa	[[0,1,0,0,0,0,0,0,0,0,0],[0,1,0,0,0,0,0,0,0,0,0],[0,1,0,0,0,0,0,0,0,0,0]]C0044644	10 11 dihydro 10 11 dihydroxy 5h dibenzazepine 5 carboxamide,10 11 dihydro 10 11 dihydroxycarbamazepine,carbazepine 10 11 diol,carbamazepine 10 11 diol,cbz diol,cbzd,10 11 dihydroxy 10 11 dihydrocarbamazepine	[[0,1,0,0,0,0,0,0,0,0,0],[0,1,0,0,0,0,0,0,0,0,0],[0,1,0,0,0,0,0,0,0,0,0],[0,1,0,0,0,0,0,0,0,0,0],[0,1,0,0,0,0,0,0,0,0,0],[0,1,0,0,0,0,0,0,0,0,0],[0,1,0,0,0,0,0,0,0,0,0]]C0149088	y 9213	[[0,1,0,0,0,0,0,0,0,0,0]]C1563419	2 ethyl 8 methyl 2 8 diazaspiro 4 5 decane 1 3 dione monofumarate	[[0,1,0,0,0,0,0,0,0,0,0]]C1563418	2 ethyl 8 methyl 2 8 diazaspiro 4 5 decane 1 3 dione fumarate 2 1	[[0,1,0,0,0,0,0,0,0,0,0]]C0149085	y 4153	[[0,1,0,0,0,0,0,0,0,0,0]]C0149084	y 1979	[[0,1,0,0,0,0,0,0,0,0,0]]C0149087	y 9179	[[0,1,0,0,0,0,0,0,0,0,0]]C0149086	y 849r	[[0,1,0,0,0,0,0,0,0,0,0]]C1563412	caroverine fumarate	[[0,1,0,0,0,0,0,0,0,0,0]]C2938727	dura ron 0 8 12	[[0,0,0,0,0,0,0,1,0,0,0]]C0310939	parvovirus vaccine erysipelothrix rhusiopathiae bacterin	[[1,0,0,0,0,0,0,0,0,0,0]]C0310938	parainfluenza 3 vaccine leptospira pomona bacterin	[[1,0,0,0,0,0,0,0,0,0,0]]C1519821	uredepa	[[0,0,0,0,0,0,0,0,0,1,0]]C2974233	1 2 3 3a 4 8b hexahydro 2 benzyl 6 n n dimethylamino 1 methylindeno 1 2 b pyrrole,hdmp cpd	[[0,1,0,0,0,0,0,0,0,0,0],[0,1,0,0,0,0,0,0,0,0,0]]C0970171	e z isomer 13 hydroxy 9 11 octadecadienoic acid	[[0,1,0,0,0,0,0,0,0,0,0]]C1122423	fumiquinazoline e	[[0,1,0,0,0,0,0,0,0,0,0]]C1122422	fumiquinazoline c	[[0,1,0,0,0,0,0,0,0,0,0]]C0310931	erysipelothrix rhusiopathiae vaccine leptospira canicola grippotyphosa hardjo icterohaemorrhagiae pomona bacterin	[[1,0,0,0,0,0,0,0,0,0,0]]C0310930	equine rhinopneumonitis influenza vaccine tetanus toxoid	[[1,0,0,0,0,0,0,0,0,0,0]]C0310933	mink distemper enteritis vaccine clostridium botulinum type c bacterin toxoid	[[1,0,0,0,0,0,0,0,0,0,0]]C0310932	mink distemper vaccine clostridium botulinum type c bacterin toxoid	[[1,0,0,0,0,0,0,0,0,0,0]]C0310935	mink enteritis vaccine clostridium botulinum type c bacterin toxoid	[[1,0,0,0,0,0,0,0,0,0,0]]C0310934	mink distemper enteritis vaccine clostridium botulinum type c pseudomonas aeruginosa bacterin toxoid	[[1,0,0,0,0,0,0,0,0,0,0]]C0310937	newcastle bronchitis vaccine mycoplasma gallisepticum bacterin	[[1,0,0,0,0,0,0,0,0,0,0]]C0310936	mink enteritis vaccine clostridium botulinum type c pseudomonas aeruginosa bacterin toxoid	[[1,0,0,0,0,0,0,0,0,0,0]]C0301459	mumps skin test antigen	[[0,0,0,0,0,0,0,1,0,0,1]]C0070074	paramylon,beta 1 3 glucan reserve polysaccharide,astasian,astazian	[[0,1,0,0,0,0,0,0,0,0,0],[0,1,0,0,0,0,0,0,0,0,0],[0,1,0,0,0,0,0,0,0,0,0],[0,1,0,0,0,0,0,0,0,0,0]]C0242937	non narcotic analgesics,nonopioid analgesics,nonopioid,nonnarcotic analgesics,non opioid analgesics	[[0,1,0,1,0,0,1,0,0,0,0],[0,1,0,0,0,0,0,0,0,0,0],[0,0,0,0,0,0,0,0,0,1,0],[0,1,0,0,0,0,1,0,0,1,0],[1,0,0,0,0,0,1,0,0,0,1]]C0669209	cl 329167,cl329167	[[0,1,0,0,0,0,0,0,0,0,0],[0,1,0,0,0,0,0,0,0,0,0]]C2975720	2 difluoromethyl 4 nitrophenyl 3 5 dideoxy d glycero alpha d galacto 2 nonulopyranosid acid	[[0,1,0,0,0,0,0,0,0,0,0]]C0064771	leptomycin b,elactocin,19 3 6 dihydro 3 methyl 6 oxo 2h pyran 2 yl 17 ethyl 6 hydroxy 3 5 7 9 11 15 hexamethyl 8 oxo 2 10 12 16 18 nonadecapentaenoic acid	[[0,1,0,0,0,0,0,0,0,0,0],[0,1,0,0,0,0,0,0,0,0,0],[0,1,0,0,0,0,0,0,0,0,0]]C0064770	leptomycin a,19 3 6 dihydro 3 methyl 6 oxo 2h pyran 2 yl 6 hydroxy 3 5 7 9 11 15 17 heptamethyl 8 oxo 2 10 12 16 18 nonadecapentaenoic acid	[[0,1,0,0,0,0,0,0,0,0,0],[0,1,0,0,0,0,0,0,0,0,0]]C0064772	leptophos oxon,o 2 5 dichloro 4 bromophenyl o methylphosphonate	[[0,1,0,0,0,0,0,0,0,0,0],[0,1,0,0,0,0,0,0,0,0,0]]C2975729	sinefungin va	[[0,1,0,0,0,0,0,0,0,0,0]]C0064774	lergotrile	[[0,1,0,0,0,0,0,0,0,1,0]]C0064777	letosteine,2 2 2 ethoxy 2 oxoethyl thio ethyl 4 thiazolidinecarboxylic acid	[[0,0,0,0,0,0,0,1,0,1,0],[0,1,0,0,0,0,0,0,0,0,0]]C2355818	chlopheniramine	[[0,0,0,0,0,0,0,1,0,0,0]]C0142501	sk f 29661,skf 29661	[[0,1,0,0,0,0,0,0,0,0,0],[0,1,0,0,0,0,0,0,0,0,0]]C0142500	skf 29044	[[0,1,0,0,0,0,0,0,0,0,0]]C0142502	sk f 34288,skf 34288	[[0,1,0,0,0,0,0,0,0,0,0],[0,1,0,0,0,0,0,0,0,0,0]]C0142504	sk f 36914,skf 36914	[[0,1,0,0,0,0,0,0,0,0,0],[0,1,0,0,0,0,0,0,0,0,0]]C0142507	sk f 40 383 a,skf 40383 a	[[0,1,0,0,0,0,0,0,0,0,0],[0,1,0,0,0,0,0,0,0,0,0]]C1097440	tak083,tak 083	[[0,1,0,0,0,0,0,0,0,0,0],[0,1,0,0,0,0,0,0,0,0,0]]C1097442	l17 compound	[[0,1,0,0,0,0,0,0,0,0,0]]C1097443	wr268961	[[0,1,0,0,0,0,0,0,0,0,0]]C0908798	cyclophane 1	[[0,1,0,0,0,0,0,0,0,0,0]]C0908799	cyclophane 2	[[0,1,0,0,0,0,0,0,0,0,0]]C0094141	3 beta acetoxy 6 ethyl 3a beta methyl 1 2 3 3a 4 5 8 9 9a 9b decahydro 7h benz e inden 7 one	[[0,1,0,0,0,0,0,0,0,0,0]]C0528122	atcc 20928 factor a	[[0,1,0,0,0,0,0,0,0,0,0]]C0528121	atcc 20928 factor c	[[0,1,0,0,0,0,0,0,0,0,0]]C2929582	calamine resorcinol	[[0,0,0,0,0,0,0,1,0,0,0]]C0908793	4 2 propenyl cholest 24 en 3 ol,4 2 propenyl 5alpha cholest 24 en 3 ol	[[0,1,0,0,0,0,0,0,0,0,0],[0,1,0,0,0,0,0,0,0,0,0]]C0094147	3 beta hydroxy 2 3 dihydrowithanolide f	[[0,1,0,0,0,0,0,0,0,0,0]]C0094146	3 beta hydroxy 13 alpha amino 13 17 seco 5 alpha androstan 17 oic 13 17 lactam 4 bis 2 chloroethyl amino phenoxyacetate	[[0,1,0,0,0,0,0,0,0,0,0]]C0060260	ferritin hydrazide	[[0,1,0,0,0,0,0,0,0,0,0]]C0673746	docosahexaenoylascorbic acid,dha as,dha ascorbic acid	[[0,1,0,0,0,0,0,0,0,0,0],[0,1,0,0,0,0,0,0,0,0,0],[0,1,0,0,0,0,0,0,0,0,0]]C0673747	polytheonamide b	[[0,1,0,0,0,0,0,0,0,0,0]]C2933888	sj 172550,sj172550	[[0,1,0,0,0,0,0,0,0,0,0],[0,1,0,0,0,0,0,0,0,0,0]]C2933886	sch 529074,sch529074	[[0,1,0,0,0,0,0,0,0,0,0],[0,1,0,0,0,0,0,0,0,0,0]]C0903679	aaedefa5	[[0,1,0,0,0,0,0,0,0,0,0]]C0730746	pork mixtard 30	[[0,0,0,0,0,0,0,1,0,0,0]]C2933882	10 13 dimethyl 17 2 6 sulfanylidene 3h purin 9 yl acetyl 1 2 6 7 8 9 11 12 14 15 16 17 dodecahydrocyclopenta a phenanthren 3 one	[[0,1,0,0,0,0,0,0,0,0,0]]C2933883	nsc 114792,nsc114792	[[0,1,0,0,0,0,0,0,0,0,0],[0,1,0,0,0,0,0,0,0,0,0]]C0730742	pneumovax ii	[[0,0,0,0,0,0,0,1,0,0,0]]C0063278	ici 118551,ici 118 551	[[0,1,0,0,0,0,0,0,0,0,0],[0,1,0,0,0,0,0,0,0,0,0]]C1121736	flavokawain b,flavokavain b	[[0,1,0,0,0,0,0,0,0,0,0],[0,1,0,0,0,0,0,0,0,0,0]]C0063275	ici 106270,ici106270	[[0,1,0,0,0,0,0,0,0,0,0],[0,1,0,0,0,0,0,0,0,0,0]]C0063274	ici 105552,sodium salt 1 3 4 dichlorophenyl methyl 1 2 dihydro 3 methyl 2 oxo 4 quinolineacetic acid	[[0,1,0,0,0,0,0,0,0,0,0],[0,1,0,0,0,0,0,0,0,0,0]]C0063277	ici 118233,n 4 1 6 dihydro 6 oxo 3 pyridazinyl phenyl n methyl urea	[[0,1,0,0,0,0,0,0,0,0,0],[0,1,0,0,0,0,0,0,0,0,0]]C0063270	ic 140,4 4 bis 2 chloroethyl amino phenoxy carbonyl amino benzoic acid	[[0,1,0,0,0,0,0,0,0,0,0],[0,1,0,0,0,0,0,0,0,0,0]]C0532542	ac arg pro asp nh isobutyl	[[0,1,0,0,0,0,0,0,0,0,0]]C0243263	beta 3 4 dihydroxyphenylethyl alcohol	[[0,1,0,0,0,0,0,0,0,0,0]]C1122283	n coumaroyltyramine	[[0,1,0,0,0,0,0,0,0,0,0]]C0243266	pirinixic acid,4 chloro 6 2 3 xylidino 2 pyrimidinylthio acetic acid,4 chloro 6 2 3 xylidinyl 2 pyrimidinylthioacetic acid,4 chloro 6 2 3 dimethylphenyl amino 2 pyrimidinyl thio acetic acid,cxpta,4 chloro 6 2 3 dimethylphenyl amino 2 pyrimidinylthioacetic acid	[[0,1,0,0,0,0,0,0,0,1,0],[0,1,0,0,0,0,0,0,0,0,0],[0,1,0,0,0,0,0,0,0,0,0],[0,1,0,0,0,0,0,0,0,0,0],[0,1,0,0,0,0,0,0,0,0,0],[0,1,0,0,0,0,0,0,0,0,0]]C0243264	hydroxytyrosol	[[0,1,0,0,0,0,0,0,0,1,0]]C0532545	pd 140798,pd140798	[[0,1,0,0,0,0,0,0,0,0,0],[0,1,0,0,0,0,0,0,0,0,0]]C0666770	nc 1800	[[0,1,0,0,0,0,0,0,0,0,0]]C1992141	mannitol 124 urine	[[0,0,0,1,0,0,0,0,0,0,0]]C1992140	mannitol 124 bld ser plas	[[0,0,0,1,0,0,0,0,0,0,0]]C0290271	e 5324,e5324	[[0,1,0,0,0,0,0,0,0,0,0],[0,1,0,0,0,0,0,0,0,0,0]]C0290270	n butyl n 2 3 5 ethyl 4 phenyl 1h imidazol 1 yl propoxy 6 methylphenyl urea,n butyl n 02 03 5 ethyl 4 phenyl 1h imidazol 1 yl propoxy 6 methylphenyl urea	[[0,1,0,0,0,0,0,0,0,0,0],[0,1,0,0,0,0,0,0,0,0,0]]C0290273	ta 7552,dimethyl ester 4 3 4 dimethoxyphenyl 1 hydroxy 5 6 7 trimethoxy 2 3 naphthalenedicarboxylic acid	[[0,1,0,0,0,0,0,0,0,0,0],[0,1,0,0,0,0,0,0,0,0,0]]C0290272	1 3 4 dimethoxyphenyl 2 3 bis methoxycarbonyl 4 hydroxy 6 7 8 trimethoxynaphthalene	[[0,1,0,0,0,0,0,0,0,0,0]]C2975189	toctino	[[0,1,0,0,0,0,0,0,0,0,0]]C0127159	mci 186	[[0,1,0,0,0,0,0,0,0,0,0]]C0299746	lu 103793	[[0,0,0,0,1,0,0,0,0,1,0]]C0299745	nsc 669356d,nsc d 669356,nsc d669356	[[0,1,0,0,0,0,0,0,0,0,0],[0,1,0,0,0,0,0,0,0,0,0],[0,1,0,0,0,0,0,0,0,0,0]]C0299748	florifenine,2 1 pyrrolidinyl ethyl n 7 trifluoromethyl 4 quinolyl anthranilate	[[0,1,0,0,0,0,0,0,0,1,0],[0,1,0,0,0,0,0,0,0,0,0]]C2073885	chlorpheniramine phenylephrine phenylpropanolamine phenyltoloxamine,chlorphenamine phenylephrine phenylpropanolamine phenyltoloxamine,phenyltoloxamine drug combination phenylpropanolamine phenylephrine chlorpheniramine,phenyltoloxamine drug combination phenylpropanolamine phenylephrine chlorphenamine	[[0,1,0,0,0,0,0,1,0,0,0],[0,1,0,0,0,0,0,0,0,0,0],[0,1,0,0,0,0,0,0,0,0,0],[0,1,0,0,0,0,0,0,0,0,0]]C1986619	diamorphine 124 bld ser plas	[[0,0,0,1,0,0,0,0,0,0,0]]C2073887	acetaminophen chlorpheniramine phenylpropanolamine phenyltoloxamine	[[0,0,0,0,0,0,0,1,0,0,0]]C0649497	3 beta phenylethyl 9beta methoxy 9 alpha m 1 cyclopentyl propionoxyphenyl 3 azabicyclo 3 3 1 nonane	[[0,1,0,0,0,0,0,0,0,0,0]]C1956451	atrop abyssomicin c	[[0,1,0,0,0,0,0,0,0,0,0]]C2917359	glucagon like peptide 1 receptor agonist,glp 1 receptor agonist	[[0,0,0,0,0,0,0,0,0,0,1],[0,0,0,0,0,0,0,0,0,0,1]]C0631099	dichlorobis tranylcypromine platinum ii,cis pt ii tranylcypromine 2cl2	[[0,1,0,0,0,0,0,0,0,0,0],[0,1,0,0,0,0,0,0,0,0,0]]C1530291	plastufer,icn brand of ferrous sulfate	[[0,1,0,0,0,0,0,0,0,0,0],[0,1,0,0,0,0,0,0,0,0,0]]C1530290	hamatopan,wolff brand of ferrous sulfate	[[0,1,0,0,0,0,0,0,0,0,0],[0,1,0,0,0,0,0,0,0,0,0]]C1530293	eisenkapseln intermuti,intermuti brand of ferroglycine sulfate	[[0,1,0,0,0,0,0,0,0,0,0],[0,1,0,0,0,0,0,0,0,0,0]]C1530292	vitaferro kapseln,hexal brand of ferrous sulfate	[[0,1,0,0,0,0,0,0,0,0,0],[0,1,0,0,0,0,0,0,0,0,0]]C1530295	glutaferro gotas,medix brand of ferroglycine sulfate	[[0,1,0,0,0,0,0,0,0,0,0],[0,1,0,0,0,0,0,0,0,0,0]]C1530294	ferro sanol,sanol brand of ferroglycin sulfate,schwarz brand of ferroglycine sulfate	[[0,1,0,0,0,0,0,0,0,0,0],[0,1,0,0,0,0,0,0,0,0,0],[0,1,0,0,0,0,0,0,0,0,0]]C1530297	squad,disodium salt aerocid brand of flavodic acid	[[0,1,0,0,0,0,0,0,0,0,0],[0,1,0,0,0,0,0,0,0,0,0]]C1530296	intercyton,disodium salt celltech brand of flavodic acid,disodium salt sanofi synthelabo brand of flavodic acid	[[0,1,0,0,0,0,0,0,0,0,0],[0,1,0,0,0,0,0,0,0,0,0],[0,1,0,0,0,0,0,0,0,0,0]]C0110177	1 cyclohexyl 3 2 4 morpholinyl ethyl carbodiimide tosylate,cmct	[[0,1,0,0,0,0,0,0,0,0,0],[0,1,0,0,0,0,0,0,0,0,0]]C0093491	2 nitratopropyl 3 nitratopropyl 2 6 dimethyl 4 3 nitrophenyl 1 4 dihydropyridine 3 5 dicarboxylate	[[0,1,0,0,0,0,0,0,0,0,0]]C0672830	sulochrin	[[0,1,0,0,0,0,0,0,0,0,0]]C0350371	pollen allergy preparations	[[1,0,0,0,0,0,0,0,0,0,0]]C0530372	ly 297802,ly297802	[[0,1,0,0,0,0,0,0,0,0,0],[0,1,0,0,0,0,0,0,0,0,0]]C0770941	antazoline sulfate,antazoline sulphate,imidamine sulfate,imidamine sulphate,phenazoline sulfate,phenazoline sulphate,n benzyl n 2 imidazolin 2 ylmethyl aniline sulfate dihydrate	[[1,0,0,0,0,0,0,0,0,1,0],[1,0,0,0,0,0,0,0,0,1,0],[1,0,0,0,0,0,0,0,0,0,0],[1,0,0,0,0,0,0,0,0,0,0],[1,0,0,0,0,0,0,0,0,0,0],[1,0,0,0,0,0,0,0,0,0,0],[0,0,0,0,0,0,0,0,0,1,0]]C0173681	omega conotoxin svib,cyclic 1 16 8 20 15 25 tris disulfide 3 l leucine 6 l glutamine 7 l serine 17 l serine 20a endo l serine omega conotoxin m viia reduced,omega ctx svib	[[0,1,0,0,0,0,0,0,0,0,0],[0,1,0,0,0,0,0,0,0,0,0],[0,1,0,0,0,0,0,0,0,0,0]]C2698844	laxabon	[[0,0,0,0,0,0,0,0,0,1,0]]C0074491	sibiromycin	[[0,1,0,0,0,0,0,0,0,0,0]]C2825617	leucinocaine	[[0,0,0,0,0,0,0,0,0,1,0]]C1330206	dimetapp 12 hour non drowsy extentabs	[[0,0,0,0,0,0,0,1,0,0,0]]C2825614	parethoxycaine hydrochloride	[[0,0,0,0,0,0,0,0,0,1,0]]C0533637	impresept espe	[[0,1,0,0,0,0,0,0,0,0,0]]C2825612	metacetamol	[[0,0,0,0,0,0,0,0,0,1,0]]C0913286	hlp 1 polypeptide	[[0,1,0,0,0,0,0,0,0,0,0]]C1968125	tussin cf cold	[[0,0,0,0,0,0,0,1,0,0,0]]C0913288	hlp 2 polypeptide	[[0,1,0,0,0,0,0,0,0,0,0]]C0071269	platinum ii pentamidine,pt pentamidine complex,pt pent	[[0,1,0,0,0,0,0,0,0,0,0],[0,1,0,0,0,0,0,0,0,0,0],[0,1,0,0,0,0,0,0,0,0,0]]C0533639	du 14	[[0,1,0,0,0,0,0,0,0,0,0]]C0533638	4 o sulfamoyl n tetradecanoyltyramine,p o sulfamoyl n tetradecanoyltetramine	[[0,1,0,0,0,0,0,0,0,0,0],[0,1,0,0,0,0,0,0,0,0,0]]C2825619	pyrrocaine hydrochloride	[[0,0,0,0,0,0,0,0,0,1,0]]C0071268	platinum ii 1 2 diaminocyclohexane malonate,sp 4 2 trans 1 2 cyclohexanediamine n n propanedioato 2 o o platinum,1 2 diaminocyclohexylplatinum malonate,1 2 diaminocylohexanemalonato platinum ii,1 2 diamminocyclohexylplatinum malonate,malonato 1 2 diaminocyclohexane platinum ii	[[0,1,0,0,0,0,0,0,0,0,0],[0,1,0,0,0,0,0,0,0,0,0],[0,1,0,0,0,0,0,0,0,0,0],[0,1,0,0,0,0,0,0,0,0,0],[0,1,0,0,0,0,0,0,0,0,0],[0,1,0,0,0,0,0,0,0,0,0]]C0049477	acexamic acid,6 acetylaminocaproic acid,6 acetamidohexanoic acid,epsilon acetylaminocaproic acid,6 acetylamino hexanoic acid,n acetylamino 6 hexanoic acid	[[0,1,0,0,0,0,0,0,0,1,0],[0,1,0,0,0,0,0,0,0,0,0],[0,1,0,0,0,0,0,0,0,0,0],[0,1,0,0,0,0,0,0,0,0,0],[0,1,0,0,0,0,0,0,0,0,0],[0,1,0,0,0,0,0,0,0,0,0]]C0061653	penicillium 8d glycoprotein a,gpa pen 8d	[[0,1,0,0,0,0,0,0,0,0,0],[0,1,0,0,0,0,0,0,0,0,0]]C0124435	kalimate	[[0,1,0,0,0,0,0,0,0,0,0]]C0593249	timoptol la	[[0,0,0,0,0,0,0,1,0,0,0]]C1366014	galanga preparation	[[0,0,0,0,0,1,0,0,0,0,0]]C1366015	galangal root	[[0,0,0,0,0,0,0,0,0,0,1]]C1366010	ligusticum preparation	[[0,0,0,0,0,1,0,0,0,0,0]]C1449546	gelopol	[[0,1,0,0,0,0,0,0,0,0,0]]C0593244	zonivent aquanasal	[[0,0,0,0,0,0,0,1,0,0,0]]C0096788	4 biphenylylacetic acid ethyl ester,bpaa et,ethyl 4 biphenylacetate,felbinac ethyl ester,ethyl 4 biphenyl acetate	[[0,1,0,0,0,0,0,0,0,0,0],[0,1,0,0,0,0,0,0,0,0,0],[0,1,0,0,0,0,0,0,0,0,0],[0,1,0,0,0,0,0,0,0,0,0],[0,1,0,0,0,0,0,0,0,0,0]]C2698272	gsk 1363089,gsk1363089,multitargeted tyrosine kinase inhibitor gsk1363089,met vegfr 2 inhibitor gsk1363089	[[0,1,0,0,0,0,0,0,0,0,0],[0,1,0,0,0,0,0,0,0,0,0],[0,0,0,0,0,0,0,0,0,1,0],[0,0,0,0,0,0,0,0,0,1,0]]C0631638	5 homocysteinyl methyl adenosine 5 beta gamma imidotriphosphate,5 hcmait	[[0,1,0,0,0,0,0,0,0,0,0],[0,1,0,0,0,0,0,0,0,0,0]]C0252928	crambescin c1,7 aminoiminomethyl amino heptyl ester 2 amino 1 4 dihydro 6 3 hydroxypropyl 4 nonyl 5 pyrimidinecarboxylic acid	[[0,1,0,0,0,0,0,0,0,0,0],[0,1,0,0,0,0,0,0,0,0,0]]C1449544	flavamed,berlin chemie brand of abmbroxol hydrochloride	[[0,1,0,0,0,0,0,0,0,0,0],[0,1,0,0,0,0,0,0,0,0,0]]C0252923	asprellic acid a,3beta e 27 e 3 27 bis 3 4 hydroxyphenyl 1 oxo 2 propenyl oxy olean 12 en 28 oic acid	[[0,1,0,0,0,0,0,0,0,0,0],[0,1,0,0,0,0,0,0,0,0,0]]C0252922	asprellic acid c	[[0,1,0,0,0,0,0,0,0,0,0]]C0252921	asprellic acid b	[[0,1,0,0,0,0,0,0,0,0,0]]C0252920	3 27 di o p coumaroyloxyolean 12 en 28 oic acid	[[0,1,0,0,0,0,0,0,0,0,0]]C0252927	crambescin b,5alpha 9beta 10beta 7 aminoiminomethyl amino heptyl ester 7 amino 9 nonyl 1 oxa 6 8 diazaspiro 4 5 dec 6 ene 10 carboxylic acid	[[0,1,0,0,0,0,0,0,0,0,0],[0,1,0,0,0,0,0,0,0,0,0]]C0252926	bruceanol f,11beta 12alpha 15beta e methyl ester 15 3 4 dimethyl 1 oxo 2 pentenyl oxy 13 20 epoxy 2 11 12 trihydroxy 1 16 dioxo picras 2 en 21 oic acid	[[0,1,0,0,0,0,0,0,0,1,0],[0,1,0,0,0,0,0,0,0,1,0]]C0252925	bruceanol e,1beta 11beta 12alpha 15beta e methyl ester 15 3 4 dimethyl 1 oxo 2 pentenyl oxy 13 20 epoxy 1 11 12 trihydroxy 2 16 dioxo picrasan 21 oic acid	[[0,1,0,0,0,0,0,0,0,1,0],[0,1,0,0,0,0,0,0,0,1,0]]C0252924	bruceanol d,1beta 11beta 12alpha 15beta e methyl ester 15 3 4 dimethyl 1 oxo 2 pentenyl oxy 13 20 epoxy 1 11 12 trihydroxy 2 16 dioxo picras 3 en 21 oic acid	[[0,1,0,0,0,0,0,0,0,1,0],[0,1,0,0,0,0,0,0,0,1,0]]C3181852	6 dodecylamino 2 3 4 methylpiperazin 1 yl propyl 1h benzo de isoquinoline 1 3 2h dione,6 dmpid cpd	[[0,1,0,0,0,0,0,0,0,0,0],[0,1,0,0,0,0,0,0,0,0,0]]C3181855	n acetyl cysteine proline cysteine amide,cb3 cpd	[[0,1,0,0,0,0,0,0,0,0,0],[0,1,0,0,0,0,0,0,0,0,0]]C0310752	newcastle disease avian encephalomyelitis vaccine	[[1,0,0,0,0,0,0,0,0,0,0]]C2717707	2 6 di t butyl 4 dimethyl 4 methoxyphenylsilyl methyloxy phenol	[[0,1,0,0,0,0,0,0,0,0,0]]C0166894	neurotrophin 4,nt 4	[[0,1,0,0,0,0,0,0,0,0,0],[0,1,0,0,0,0,0,0,0,0,0]]C0955708	2 propylamine hydrochloride	[[0,1,0,0,0,0,0,0,0,0,0]]C2917495	desquam	[[0,0,0,0,0,0,0,1,0,0,0]]C2984207	anti pgf monoclonal antibody ro5323441	[[0,0,0,0,0,0,0,0,0,1,0]]C0630325	bl p 2090,bl p2090	[[0,1,0,0,0,0,0,0,0,0,0],[0,1,0,0,0,0,0,0,0,0,0]]C0630327	whr 1582a	[[0,1,0,0,0,0,0,0,0,0,0]]C0630326	1 2 6 dimethylphenyl 3 isobutoxyamidinourea,monohydrochloride n 2 6 dimethylphenyl n imino 2 methylpropoxy amino methyl urea	[[0,1,0,0,0,0,0,0,0,0,0],[0,1,0,0,0,0,0,0,0,0,0]]C0166898	ro 09 1470	[[0,1,0,0,0,0,0,0,0,0,0]]C0630323	rhesus rotavirus based quadrivalent vaccine,rrv tetravalent vaccine,rrv tv	[[0,1,0,0,0,0,0,0,0,0,0],[0,1,0,0,0,0,0,0,0,0,0],[0,1,0,0,0,0,0,0,0,0,0]]C1514770	recombinant betacellulin,beta cellulin	[[0,0,0,0,0,0,0,0,0,1,0],[0,0,0,0,0,0,0,0,0,1,0]]C1514771	recombinant fibroblast growth factor 5,hbgf 5,fgf5	[[0,0,0,0,0,0,0,0,0,1,0],[0,0,0,0,0,0,0,0,0,1,0],[0,0,0,0,0,0,0,0,0,1,0]]C1514772	recombinant fibroblast growth factor 6,hst 2,hbgf 6,fgf6,fgf 6	[[0,0,0,0,0,0,0,0,0,1,0],[0,0,0,0,0,0,0,0,0,1,0],[0,0,0,0,0,0,0,0,0,1,0],[0,0,0,0,0,0,0,0,0,1,0],[0,0,0,0,0,0,0,0,0,1,0]]C1514776	recombinant fowlpox mgp100 vaccine,rf mgp100 vaccine	[[0,0,0,0,0,0,0,0,0,1,0],[0,0,0,0,0,0,0,0,0,1,0]]C1514778	recombinant fowlpox gp100p209,rf gp100p209	[[0,0,0,0,0,0,0,0,0,1,0],[0,0,0,0,0,0,0,0,0,1,0]]C2984202	melanoma trp2 ctl epitope vaccine scib1	[[0,0,0,0,0,0,0,0,0,1,0]]C2979987	osemozotan	[[0,1,0,0,0,0,0,0,0,1,0]]C1950859	sodium palmate	[[0,0,1,0,0,0,0,0,0,0,0]]C0049474	6 octadecylthio purine	[[0,1,0,0,0,0,0,0,0,0,0]]C0207278	l tyrosine l glutamic acid poly dl alanine poly l lysine	[[0,1,0,0,0,0,0,0,0,0,0]]C2933864	evt 201,evt201	[[0,1,0,0,0,0,0,0,0,0,0],[0,1,0,0,0,0,0,0,0,0,0]]C2984200	alpha fetoprotein peptide pulsed autologous dendritic cell vaccine	[[0,0,0,0,0,0,0,0,0,1,0]]C1882500	proxorphan tartrate	[[0,0,0,0,0,0,0,0,0,1,0]]C1997802	parenteral form calcitonin	[[1,0,0,0,0,0,0,0,0,0,0]]C0957819	isomer isamoltane	[[0,1,0,0,0,0,0,0,0,0,0]]C0957818	isomer isamoltane	[[0,1,0,0,0,0,0,0,0,0,0]]C0957815	hypaconitine hydrobromide	[[0,1,0,0,0,0,0,0,0,0,0]]C0957814	isomer 4 fluorohexahydrosiladifenidol	[[0,1,0,0,0,0,0,0,0,0,0]]C0957811	isomer duloxetine ethanedioate 1 1	[[0,1,0,0,0,0,0,0,0,0,0]]C0957810	isomer duloxetine	[[0,1,0,0,0,0,0,0,0,0,0]]C0957813	sulfate salt glutathione monoisopropyl ester	[[0,1,0,0,0,0,0,0,0,0,0]]C0957812	sulfate salt glutathione monoisopropyl ester	[[0,1,0,0,0,0,0,0,0,0,0]]C1813375	d tann hc	[[0,0,0,0,0,0,0,1,0,0,0]]C3257453	halls breezers	[[0,0,0,0,0,0,0,1,0,0,0]]C0483244	anusol	[[0,0,0,0,0,0,1,1,0,0,1]]C0537795	sdz 219 964	[[0,1,0,0,0,0,0,0,0,0,0]]C2984208	parp inhibitor e7016	[[0,0,0,0,0,0,0,0,0,1,0]]C0066382	methylglyoxal bis,mgbcp	[[0,1,0,0,0,0,0,0,0,0,0],[0,1,0,0,0,0,0,0,0,0,0]]C0066380	methylglyoxal bis,mgbb	[[0,1,0,0,0,0,0,0,0,0,0],[0,1,0,0,0,0,0,0,0,0,0]]C0066381	methylglyoxal bis,mgbc	[[0,1,0,0,0,0,0,0,0,0,0],[0,1,0,0,0,0,0,0,0,0,0]]C0066386	methylhydroxybenzimidate,methyl ester 4 hydroxy benzenecarboximidic acid	[[0,1,0,0,0,0,0,0,0,0,0],[0,1,0,0,0,0,0,0,0,0,0]]C0084717	tat 59,e dihydrogen phosphate ester 4 1 4 2 dimethylamino ethoxy phenyl 2 4 1 methylethyl phenyl 1 butenyl phenol	[[0,1,0,0,0,0,0,0,0,0,0],[0,1,0,0,0,0,0,0,0,0,0]]C0084711	talastine,4 benzyl 2 2 dimethylaminoethyl phthalazin 1 2h one	[[0,1,0,0,0,0,0,0,0,1,0],[0,1,0,0,0,0,0,0,0,0,0]]C2961248	safeguard sanitizer	[[0,0,0,0,0,0,0,1,0,0,0]]C1965895	methyl aminolevulinate hydrochloride	[[1,0,1,0,0,0,0,0,0,1,0]]C0094927	3 4 4 phenyl 1 2 3 6 tetrahydropyridyl 1 butyl indole,monohydrochloride 3 4 3 6 dihydro 4 phenyl 1 2h pyridinyl butyl 1h indole	[[0,1,0,0,0,0,0,0,0,0,0],[0,1,0,0,0,0,0,0,0,0,0]]C0094926	3 4 4 3 methylphenyl 1 piperazinyl butyl 2 4 imidazolinedione	[[0,1,0,0,0,0,0,0,0,0,0]]C1707746	flovacil	[[0,0,0,0,0,0,0,0,0,1,0]]C0094922	3 4 3 2 3 4 dimethoxyphenyl ethyl amino 2 hydroxypropoxy phenyl 2 crotonic acid nitrile	[[0,1,0,0,0,0,0,0,0,0,0]]C0094921	3 4 2 hydroxy 3 1 1 dimethylethyl amino propoxy phenyl 7 methoxy 2 methyl 1 2h isoquinolinone	[[0,1,0,0,0,0,0,0,0,0,0]]C0295382	3 bromo 2 hydroxy n 3 4 dimethyl 5 isoxazolyl 1 4 naphthoquinon 4 imine,3 brodi 1 4 ndi	[[0,1,0,0,0,0,0,0,0,0,0],[0,1,0,0,0,0,0,0,0,0,0]]C0295380	2 hydroxytacrine	[[0,1,0,0,0,0,0,0,0,0,0]]C0094928	3 4 acetyloxy 5 ethyl 3 methoxy 1 naphthalenyl 2 methyl 2 propenoic acid	[[0,1,0,0,0,0,0,0,0,0,0]]C1522429	r 12564	[[0,0,0,0,0,0,0,0,0,1,0]]C1099020	am 1121	[[0,1,0,0,0,0,0,0,0,0,0]]C1429214	histoplasma capsulatum histin protein,histin	[[0,1,0,0,0,0,0,0,0,0,0],[0,0,0,0,0,0,1,0,0,0,0]]C1992389	methamphetamine 124 milk	[[0,0,0,1,0,0,0,0,0,0,0]]C1992388	methamphetamine 124 meconium	[[0,0,0,1,0,0,0,0,0,0,0]]C1992387	methamphetamine 124 hair	[[0,0,0,1,0,0,0,0,0,0,0]]C1992386	methamphetamine 124 gastric fluid	[[0,0,0,1,0,0,0,0,0,0,0]]C1992384	methamphetamine 124 bld ser plas	[[0,0,0,1,0,0,0,0,0,0,0]]C1992383	methadone r 124 bld ser plas	[[0,0,0,1,0,0,0,0,0,0,0]]C1099022	bay43 9695,bay 43 9695	[[0,1,0,0,0,0,0,0,0,0,0],[0,1,0,0,0,0,0,0,0,0,0]]C2724467	septra 40 200	[[0,0,0,0,0,0,0,1,0,0,0]]C0637678	2 chloro 2 deoxy 3 7 dideazaadenosine	[[0,1,0,0,0,0,0,0,0,0,0]]C2357127	cefpodoxime clavulanate 124 isolate	[[0,0,0,1,0,0,0,0,0,0,0]]C0637676	methyl 1 ribofuranosyl 1 2 4 triazole 3 carboxamidate,mrtc	[[0,1,0,0,0,0,0,0,0,0,0],[0,1,0,0,0,0,0,0,0,0,0]]C0637677	2 cddda	[[0,1,0,0,0,0,0,0,0,0,0]]C0637674	ethyl 1 ribofuranosyl 1 2 4 triazole 3 carboximidate,ertc	[[0,1,0,0,0,0,0,0,0,0,0],[0,1,0,0,0,0,0,0,0,0,0]]C0637672	tetrabromorhodamine 123,tbr 123	[[0,1,0,0,0,0,0,0,0,0,0],[0,1,0,0,0,0,0,0,0,0,0]]C2723110	bepreve	[[0,0,0,0,0,0,0,0,0,1,0]]C1263073	decazate	[[0,0,0,0,0,0,0,1,0,0,0]]C0968236	technetium diethylenetriaminepentaacetic acid mannosyl dextran,99mtc dtpa mannosyl dextran,tc dtpa mannosyl dextran,99mtc diethylenetriamine pentaacetic acid mannosyl dextran	[[0,1,0,0,0,0,0,0,0,0,0],[0,1,0,0,0,0,0,0,0,1,0],[0,1,0,0,0,0,0,0,0,0,0],[0,0,0,0,0,0,0,0,0,1,0]]C0968234	auto cross linked polysaccharide,acp gel	[[0,1,0,0,0,0,0,0,0,0,0],[0,1,0,0,0,0,0,0,0,0,0]]C0209120	sdz hdc 912	[[0,1,0,0,0,0,0,0,0,0,0]]C0163948	phenylenediamine mustard	[[0,1,0,0,0,0,0,0,0,0,0]]C0111914	cytostipin	[[0,1,0,0,0,0,0,0,0,0,0]]C1722858	n n n dimethylcarboxamidomethyl cycloheximide,dm chx	[[0,1,0,0,0,0,0,0,0,0,0],[0,1,0,0,0,0,0,0,0,0,0]]C0279165	radioactive agent	[[0,0,0,0,1,0,0,0,0,0,0]]C0675048	s methyl n n diethyldithiocarbamate sulfine,meddc sulfine	[[0,1,0,0,0,0,0,0,0,0,0],[0,1,0,0,0,0,0,0,0,0,0]]C0279161	ovulatory agent	[[0,0,0,0,1,0,0,0,0,0,0]]C0243331	11 nor delta 8 tetrahydrocannabinol 9 carboxylic acid,11 nor delta 8 thc cooh	[[0,1,0,0,0,0,0,0,0,0,0],[0,1,0,0,0,0,0,0,0,0,0]]C1328222	garlipure maximum allicin	[[0,0,0,0,1,0,0,0,0,0,0]]C0951151	n hydroxyxanthine monohydrate	[[0,1,0,0,0,0,0,0,0,0,0]]C0051273	alpha bis 3 n n diethylcarbamoyl piperidino 4 xylene alpha,bdcpx,alpha alpha bis 3 n n diethylcarbamoyl piperidino p xylene,a 1 nipecotamide	[[0,1,0,0,0,0,0,0,0,0,0],[0,1,0,0,0,0,0,0,0,0,0],[0,1,0,0,0,0,0,0,0,0,0],[0,1,0,0,0,0,0,0,0,0,0]]C0051275	alpha 2 dimethyl 5h 1 benzopyrano 2 3 b pyridine 7 acetate,dbpaa	[[0,1,0,0,0,0,0,0,0,0,0],[0,1,0,0,0,0,0,0,0,0,0]]C0613704	cinnamoylhydroxamic acid	[[0,1,0,0,0,0,0,0,0,0,0]]C0051279	alpha alpha diphenylsuccinimide,3 3 diphenyl 2 5 pyrrolidinedione	[[0,1,0,0,0,0,0,0,0,0,0],[0,1,0,0,0,0,0,0,0,0,0]]C0613707	ashwagandha preparation,ashwagandha	[[0,0,0,0,0,1,0,0,0,0,0],[0,1,0,0,0,0,1,0,0,0,0]]C1815327	flulaval	[[0,1,0,0,0,0,0,0,0,0,0]]C0619366	tartaric acid dihydrazide	[[0,1,0,0,0,0,0,0,0,0,0]]C0613703	trans cinnamoylhydroxamic acid	[[0,1,0,0,0,0,0,0,0,0,0]]C1988283	gallotannin 124 gastric fluid	[[0,0,0,1,0,0,0,0,0,0,0]]C1988282	gallotannin 124 bld ser plas	[[0,0,0,1,0,0,0,0,0,0,0]]C1988284	gallotannin 124 plant	[[0,0,0,1,0,0,0,0,0,0,0]]C0112148	d phenylalanine proline arginine methyl chloride,d phe pro arg ch2cl	[[0,1,0,0,0,0,0,0,0,0,0],[0,1,0,0,0,0,0,0,0,0,0]]C2357128	ceftriaxone clavulanate 124 isolate	[[0,0,0,1,0,0,0,0,0,0,0]]C0085044	yutac	[[0,1,0,0,0,0,0,0,0,0,0]]C1992528	methylenedioxymethamphetamine 124 stool	[[0,0,0,1,0,0,0,0,0,0,0]]C0085042	ys 822a,23 3 amino 3 6 dideoxy beta d mannopyranosyl oxy 1 3 5 6 27 pentahydroxy 11 1 methylethyl 9 oxo 10 29 dioxabicyclo 23 3 1 nonacosa 15 17 19 21 tetraene 26 carboxylic acid	[[0,1,0,0,0,0,0,0,0,0,0],[0,1,0,0,0,0,0,0,0,0,0]]C0085043	yttrium chloride,ycl3	[[0,1,0,0,0,0,0,0,0,0,0],[0,1,0,0,0,0,0,0,0,0,0]]C0609135	lubrol 17a10	[[0,1,0,0,0,0,0,0,0,0,0]]C0360589	calcium regulating hormone	[[1,0,0,0,0,0,0,0,0,0,0]]C1992520	methylenedioxyamphetamine 124 vitreous fluid	[[0,0,0,1,0,0,0,0,0,0,0]]C1992527	methylenedioxymethamphetamine 124 bld ser plas	[[0,0,0,1,0,0,0,0,0,0,0]]C0065244	lumin,4 4 3 2 1 ethyl 4 1h quinolinylidene ethylidene propenylene bis 1 ethylquinolinium	[[0,0,0,0,0,0,1,0,0,0,0],[0,1,0,0,0,0,0,0,0,0,0]]C0112146	d phe pro arg chloromethyl ketone	[[0,1,0,0,0,0,0,0,0,0,0]]C0112147	d phenothrin	[[0,1,0,0,0,0,0,0,0,0,0]]C0065245	luminamicin	[[0,1,0,0,0,0,0,0,0,0,0]]C1254339	ibular	[[0,0,0,0,0,0,0,1,0,0,0]]C1702222	befetupitant	[[0,1,0,0,0,0,0,0,0,1,0]]C3180535	rks 262,rks262	[[0,1,0,0,0,0,0,0,0,0,0],[0,1,0,0,0,0,0,0,0,0,0]]C1254337	ibrufhalal	[[0,0,0,0,0,0,0,1,0,0,0]]C1702229	15 oxozoapatlin 13alpha yl 10 alpha 16 alpha dihydroxy 9 alpha methyl 20 nor kauran 19 oic acid gamma lactone 17 oate,15 odm nkl	[[0,1,0,0,0,0,0,0,0,0,0],[0,1,0,0,0,0,0,0,0,0,0]]C1602144	xpect	[[0,0,0,0,0,0,0,1,0,0,0]]C1601994	centex	[[0,0,0,0,0,0,0,1,0,0,0]]C0614284	ruthenium hexaamine trichloride,ruthenium hexaammine trichloride,ruthenium hexammine trichloride,hexammineruthenium iii chloride	[[0,1,0,0,0,0,0,0,0,0,0],[0,1,0,0,0,0,0,0,0,0,0],[0,1,0,0,0,0,0,0,0,0,0],[0,1,0,0,0,0,0,0,0,0,0]]C1601991	flex 10	[[0,0,0,0,0,0,0,1,0,0,0]]C0210399	3 hydroxy 4 methylpyridine 2 carboxaldehyde thiosemicarbazone,3 ohmpct	[[0,1,0,0,0,0,0,0,0,0,0],[0,1,0,0,0,0,0,0,0,0,0]]C0210397	4 1h tetrazol 5 yl n 4 1h tetrazol 5 yl phenyl benzamide	[[0,1,0,0,0,0,0,0,0,0,0]]C1601998	etoplac	[[0,0,0,0,0,0,0,1,0,0,0]]C1703267	matmate	[[0,0,0,0,0,0,0,1,0,0,0]]C2347170	mixed bacteria vaccine,mbv,coleys toxin	[[0,0,0,0,0,0,0,0,0,1,0],[0,0,0,0,0,0,0,0,0,1,0],[0,0,0,0,0,0,0,0,0,1,0]]C0386148	4 deacetylgriseusin a,4 deacetyl griseusin a	[[0,1,0,0,0,0,0,0,0,0,0],[0,1,0,0,0,0,0,0,0,0,0]]C2757020	potassium salt	[[0,0,0,0,0,0,0,0,0,0,1]]C0386144	rakicidin b	[[0,1,0,0,0,0,0,0,0,0,0]]C0386143	rakicidin a	[[0,1,0,0,0,0,0,0,0,0,0]]C0386142	sch 52901	[[0,1,0,0,0,0,0,0,0,0,0]]C0386141	sch 52900	[[0,1,0,0,0,0,0,0,0,0,0]]C0386140	macrosphelide b	[[0,1,0,0,0,0,0,0,0,0,0]]C2925229	levamisole 124 urine	[[0,0,0,1,0,0,0,0,0,0,0]]C1450992	rc414	[[0,1,0,0,0,0,0,0,0,0,0]]C2713011	obatoclax,2 2 3 5 dimethyl 1h pyrrol 2 yl methylene 3 methoxy 2h pyrrol 5 yl 1h indole	[[0,0,0,0,0,0,0,0,0,1,0],[0,0,0,0,0,0,0,0,0,1,0]]C0630713	hsr 740	[[0,1,0,0,0,0,0,0,0,0,0]]C2713012	berubicin hydrochloride	[[0,0,0,0,0,0,0,0,0,1,0]]C2925227	levamisole 124 body fluid	[[0,0,0,1,0,0,0,0,0,0,0]]C2925226	levamisole 124 bld ser plas	[[0,0,0,1,0,0,0,0,0,0,0]]C0138873	protein kinase stimulatory modulator	[[0,1,0,0,0,0,0,0,0,0,0]]C0090127	11 hydroxy 5 methyl 2 2 2 bioxiran 2 yl 4h anthra 1 2b pyran 4 7 12 trione	[[0,1,0,0,0,0,0,0,0,0,0]]C0090125	10 methyl 11 hydroxyaporphine,11 hmapp,11 hydroxy 10 methylaporphine	[[0,1,0,0,0,0,0,0,0,0,0],[0,1,0,0,0,0,0,0,0,0,0],[0,1,0,0,0,0,0,0,0,0,0]]C0894812	monohydrochloride 1alpha s 2beta isomer 2 1 hydroxyethyl 4 3 dimethylamino propyl 3 4 dihydro 3 phenyl 2h 1 4 benzothiazine	[[0,1,0,0,0,0,0,0,0,0,0]]C0894811	monohydrochloride 1alpha r 2beta isomer 2 1 hydroxyethyl 4 3 dimethylamino propyl 3 4 dihydro 3 phenyl 2h 1 4 benzothiazine	[[0,1,0,0,0,0,0,0,0,0,0]]C0894810	2 1 hydroxyethyl 4 3 dimethylamino propyl 3 4 dihydro 3 phenyl 2h 1 4 benzothiazine hydrochloride	[[0,1,0,0,0,0,0,0,0,0,0]]C1449658	ceplene	[[0,1,0,0,0,0,0,0,0,0,0]]C1449659	4 aminopyridine sustained release,fampridine sr	[[0,1,0,0,0,0,0,0,0,0,0],[0,1,0,0,0,0,0,0,0,0,0]]C0771669	meglumine benzoate	[[0,0,0,0,0,0,0,1,0,0,0]]C0045930	2 benzoylhydrazono 1 3 dithiolane,2 bhd,1 3 dithiolan 2 ylidenehydrazide benzoic acid	[[0,1,0,0,0,0,0,0,0,0,0],[0,1,0,0,0,0,0,0,0,0,0],[0,1,0,0,0,0,0,0,0,0,0]]C0645674	17 n n diisopropylcarbamoyl estra 1 3 5 10 triene 3 sulfonic acid,dets	[[0,1,0,0,0,0,0,0,0,0,0],[0,1,0,0,0,0,0,0,0,0,0]]C0023863	lisuride,lysuride,methylergol carbamide,n 8alpha 9 10 didehydro 6 methylergolin 8 yl n n diethyl urea	[[0,0,0,0,0,0,1,0,0,1,0],[1,0,0,0,0,0,0,0,0,0,1],[0,1,0,0,0,0,0,0,0,0,1],[0,0,0,0,0,0,0,0,0,0,1]]C2726433	advanced eye relief night time	[[0,0,0,0,0,0,0,1,0,0,0]]C1634059	phencarb gg	[[0,0,0,0,0,0,0,1,0,0,0]]C2726437	calcium pyruvate	[[0,0,0,0,0,0,0,1,0,0,0]]C1623413	4 ethyl 1 2 3 4 trimethoxybenzoyl piperazine	[[0,1,0,0,0,0,0,0,0,0,0]]C0011569	depressan,altana pharma oranienburg brand of dihydralazine sulfate	[[0,1,0,0,0,0,0,0,0,0,0],[0,1,0,0,0,0,0,0,0,0,0]]C1703178	dental resources brand of stannous fluoride gel	[[0,0,0,0,0,0,0,1,0,0,0]]C2979169	wal profen cold and sinus	[[0,0,0,0,0,0,0,1,0,0,0]]C0771661	miristalkonium chloride	[[0,0,0,0,0,0,0,1,0,0,0]]C1828434	aviptadil phentolamine	[[1,0,0,0,0,0,0,0,0,0,0]]C2938724	chemdec 0 8 12	[[0,0,0,0,0,0,0,1,0,0,0]]C1173418	ew 154,ew154	[[0,1,0,0,0,0,0,0,0,0,0],[0,1,0,0,0,0,0,0,0,0,0]]C1173416	fh 71 compound,fh71 compound	[[0,1,0,0,0,0,0,0,0,0,0],[0,1,0,0,0,0,0,0,0,0,0]]C1173417	ew65 compound,ew 65 compound	[[0,1,0,0,0,0,0,0,0,0,0],[0,1,0,0,0,0,0,0,0,0,0]]C2345332	clinimix 5 15	[[0,0,0,0,0,0,0,1,0,0,0]]C0096913	4 desacetylvinblastine hydrazide monoclonal antibody ks1 4 conjugate	[[0,1,0,0,0,0,0,0,0,0,0]]C0052709	avilamycin	[[0,1,0,1,0,0,0,0,0,0,0]]C0052704	avermectin h2b1b,ivermectin component b1b,5 o demethyl 25 de 1 methylpropyl 22 23 dihydro 25 1 methylethyl avermectin a1a,5 o demethyl 25 de 1 methylpropyl 22 23 dihydro 25 propyl avermectin a1a,22 23 dihydroavermectin b 1 b,22 23 dihydroavermectin b1,22 23 dihydroavermectin b1b	[[0,1,0,0,0,0,0,0,0,0,0],[0,1,0,0,0,0,0,0,0,0,0],[0,1,0,0,0,0,0,0,0,0,0],[0,1,0,0,0,0,0,0,0,0,0],[0,1,0,0,0,0,0,0,0,0,0],[0,1,0,0,0,0,0,0,0,0,0],[0,1,0,0,0,0,0,0,0,0,0]]C2737968	glutamine 124 blood dot,glutamine 124 dried blood spot	[[0,0,0,1,0,0,0,0,0,0,0],[0,0,0,1,0,0,0,0,0,0,0]]C0052701	avermectin b 1 a,5 o demethylavermectin a1a,avermectin b1a	[[0,1,0,0,0,0,0,0,0,0,0],[0,1,0,0,0,0,0,0,0,0,0],[0,1,0,0,0,0,0,0,0,0,0]]C0052703	avermectin b2a	[[0,1,0,0,0,0,0,0,0,0,0]]C0603165	10 2 4 dinitrophenylthio phenothiazine,10 2 4 dinitrophenyl thio 10h phenothiazine	[[0,1,0,0,0,0,0,0,0,0,0],[0,1,0,0,0,0,0,0,0,0,0]]C1994168	paramethadione 124 bld ser plas	[[0,0,0,1,0,0,0,0,0,0,0]]C0603167	n 4 dinitrosomethylaniline	[[0,1,0,0,0,0,0,0,0,0,0]]C0603166	dinitrosodimethylethylenediamine	[[0,1,0,0,0,0,0,0,0,0,0]]C0603160	5 7 dinitroindazole	[[0,1,0,0,0,0,0,0,0,0,0]]C1511000	mylosar	[[0,0,0,0,1,0,0,0,0,0,0]]C1441405	lithium serum	[[0,0,0,1,0,0,0,0,0,0,0]]C1994167	paraldehyde 124 urine	[[0,0,0,1,0,0,0,0,0,0,0]]C1994166	paraldehyde 124 bld ser plas	[[0,0,0,1,0,0,0,0,0,0,0]]C0956333	s isomer 3 4 dihydroxybutanoic acid	[[0,1,0,0,0,0,0,0,0,0,0]]C0956332	monosodium salt 3 4 dihydroxybutanoic acid	[[0,1,0,0,0,0,0,0,0,0,0]]C0148923	x 5079c	[[0,1,0,0,0,0,0,0,0,0,0]]C0956330	l arg l tyr d ala l phe d leu isomer ala 2 arg 6 enkephalin leu	[[0,1,0,0,0,0,0,0,0,0,0]]C0956337	z isomer 2 methyl 3 3 diphenyl 3 propanolamine maleate 1 1 salt	[[0,1,0,0,0,0,0,0,0,0,0]]C0956336	erythro isomer 3 deoxypentonic acid	[[0,1,0,0,0,0,0,0,0,0,0]]C0956335	d threo isomer 3 deoxypentonic acid	[[0,1,0,0,0,0,0,0,0,0,0]]C0956334	d erythro isomer 3 deoxypentonic acid	[[0,1,0,0,0,0,0,0,0,0,0]]C0758643	1 2 hydroxyethoxy methyl 5 fluorouracil,hemfu	[[0,1,0,0,0,0,0,0,0,0,0],[0,1,0,0,0,0,0,0,0,0,0]]C1589492	sone 4	[[0,0,0,0,0,0,0,1,0,0,0]]C0956339	isomer 2 methyl 3 3 diphenyl 3 propanolamine hydrochloride	[[0,1,0,0,0,0,0,0,0,0,0]]C0956338	s r r isomer 2 methyl 3 3 diphenyl 3 propanolamine tartrate 1 1 salt	[[0,1,0,0,0,0,0,0,0,0,0]]C0168853	kw 4679	[[0,1,0,0,0,0,0,0,0,0,0]]C0758645	1 1 3 dihydroxy 2 propoxy methyl 5 fluorouracil,dhpfu	[[0,1,0,0,0,0,0,0,0,0,0],[0,1,0,0,0,0,0,0,0,0,0]]C2964360	polychlorinated biphenyl aroclor 1242	[[0,0,0,1,0,0,0,0,0,0,0]]C0219250	n 3 5 4 fluorophenoxy 2 furanyl 1 methyl 2 propynyl n hydroxyurea,n hydroxy n 4 5 4 fluorophenoxy 2 furyl 3 butyn 2 yl urea	[[0,1,0,0,0,0,0,0,0,0,0],[0,1,0,0,0,0,0,0,0,0,0]]C0219251	a 78773,n 3 5 4 fluorophenoxy 2 furanyl 1 methyl 2 propynyl n hydroxy urea	[[0,1,0,0,0,0,0,0,0,0,0],[0,1,0,0,0,0,0,0,0,0,0]]C3179477	jnj 1037049,jnj1037049	[[0,1,0,0,0,0,0,0,0,0,0],[0,1,0,0,0,0,0,0,0,0,0]]C1450707	chlorphed,roberts brand of brompheniramine maleate	[[0,1,0,0,0,0,0,0,0,0,0],[0,1,0,0,0,0,0,0,0,0,0]]C1138303	gtri bb	[[0,1,0,0,0,0,0,0,0,0,0]]C1138302	halxazone,8 hydroxymethyl 1 methoxy 3h phenoxazin 3 one	[[0,1,0,0,0,0,0,0,0,0,0],[0,1,0,0,0,0,0,0,0,0,0]]C1138301	pyloricidin a	[[0,1,0,0,0,0,0,0,0,0,0]]C1138304	queenslandon	[[0,1,0,0,0,0,0,0,0,0,0]]C0639840	xylazole	[[0,1,0,0,0,0,0,0,0,0,0]]C0244731	vertigoheel	[[0,1,0,0,0,0,0,0,0,0,0]]C0621624	3 mercaptomethyl 3 4 5 6 tetrahydro 2 oxo 1h 1 benzazocine 1 acetic acid,3 mmtob	[[0,1,0,0,0,0,0,0,0,0,0],[0,1,0,0,0,0,0,0,0,0,0]]C0624098	nirurin,s 5 6 o 6 deoxy alpha l mannopyranosyl beta d glucopyranosyl oxy 2 3 dihydro 6 7 dihydroxy 2 4 hydroxyphenyl 8 3 methyl 2 butenyl 4h 1 benzopyran 4 one,5 6 7 4 tetrahydroxy 8 3 methylbut 2 enyl flavanone 5 o rutinoside	[[0,1,0,0,0,0,0,0,0,0,0],[0,1,0,0,0,0,0,0,0,0,0],[0,1,0,0,0,0,0,0,0,0,0]]C2984890	loprox lotion	[[0,0,0,0,0,0,0,0,0,1,0]]C0718123	zinc oxide topical	[[0,0,0,0,0,0,1,0,0,0,0]]C3254384	fgh 10019,fgh10019	[[0,1,0,0,0,0,0,0,0,0,0],[0,1,0,0,0,0,0,0,0,0,0]]C3254385	octaoxyethylene laurate ester,peg 8 l	[[0,1,0,0,0,0,0,0,0,0,0],[0,1,0,0,0,0,0,0,0,0,0]]C3254382	n 3 5 2 deoxyuridine 5 monophosphate prop 2 ynyl octanamide,dmpo amide	[[0,1,0,0,0,0,0,0,0,0,0],[0,1,0,0,0,0,0,0,0,0,0]]C0718127	antihistamine decongestant 12 hour	[[0,0,0,0,0,0,0,1,0,0,0]]C3254380	6 4 3 2 methylpyrrolidin 1 yl propoxy phenyl 2h pyridazin 3 one,irdabisant	[[0,1,0,0,0,0,0,0,0,0,0],[0,1,0,0,0,0,0,0,0,0,0]]C0718125	1 plus 1 f	[[0,0,0,0,0,0,0,1,0,0,0]]C0697085	eryngium aquaticum preparation	[[0,0,0,0,0,1,0,0,0,0,0]]C3254388	4 o demethylgrandisin	[[0,1,0,0,0,0,0,0,0,0,0]]C0719653	dalalone d p,dalalone dp	[[0,0,0,0,0,0,0,1,0,0,0],[0,0,0,0,0,0,0,0,0,1,0]]C0719652	dalalone	[[0,0,0,0,0,0,0,0,0,1,0]]C2825713	cetamolol hydrochloride	[[0,0,0,0,0,0,0,0,0,1,0]]C0719650	dairy ease	[[0,0,0,0,0,0,1,1,0,0,0]]C0719657	dallergy jr	[[0,0,0,0,0,0,0,1,0,0,0]]C0719656	dallergy	[[0,0,0,0,0,0,1,1,0,0,0]]C0719655	dalgan	[[0,0,0,0,0,0,1,1,0,0,0]]C0719654	dalalone l a	[[0,0,0,0,0,0,0,1,0,0,0]]C0719659	dandruff brand of pyrithione zinc,dandruff,dandruff brand	[[0,0,0,0,0,0,0,1,0,0,0],[0,0,0,0,0,0,1,0,0,0,0],[0,0,0,0,0,0,1,0,0,0,0]]C0719658	dandrex	[[0,0,0,0,0,0,0,1,0,0,0]]C0527540	aptiganel hydrochloride,n 1 naphthyl n 3 ethylphenyl n methylguanidine hcl	[[0,1,0,0,0,0,0,0,0,0,0],[0,1,0,0,0,0,0,0,0,0,0]]C0086550	tap 144,tap144	[[0,1,0,0,0,0,0,0,0,0,0],[0,1,0,0,0,0,0,0,0,0,0]]C3252101	n n bis 1 naphthaldimine o phenylenediamine	[[0,1,0,0,0,0,0,0,0,0,0]]C1879535	acetyl l carnitine hydrochloride,l acetylcarnitine hydrochloride	[[0,0,0,0,0,0,0,0,0,1,0],[0,0,0,0,0,0,0,0,0,1,0]]C2964989	clozapine 124 gastric fluid	[[0,0,0,1,0,0,0,0,0,0,0]]C2964988	clostridium difficile dna 124 xxx	[[0,0,0,1,0,0,0,0,0,0,0]]C0876129	poly histine d ped	[[0,0,0,0,0,0,0,1,0,0,0]]C0876128	plan b,womens capital brand of levonorgestrel,paladin brand of levonorgestrel	[[0,0,0,0,0,0,1,1,0,0,0],[0,1,0,0,0,0,0,0,0,0,0],[0,1,0,0,0,0,0,0,0,0,0]]C0876127	phenergan fortis	[[0,0,0,0,0,0,0,1,0,0,0]]C0876126	phazyme maximum strength	[[0,0,0,0,0,0,0,1,0,0,0]]C0876124	perimax perio rinse	[[0,0,0,0,0,0,0,1,0,0,0]]C2964987	clonazepam 124 xxx	[[0,0,0,1,0,0,0,0,0,0,0]]C2964986	clomipramine 124 xxx	[[0,0,0,1,0,0,0,0,0,0,0]]C2964985	clomipramine 124 gastric fluid	[[0,0,0,1,0,0,0,0,0,0,0]]C0876120	pedia pop palsicles	[[0,0,0,0,0,0,0,1,0,0,0]]C0634347	lk 42 82,lk 4282	[[0,1,0,0,0,0,0,0,0,0,0],[0,1,0,0,0,0,0,0,0,0,0]]C0611397	9 amino 6 chloroacridine 2 phosphate	[[0,1,0,0,0,0,0,0,0,0,0]]C0634348	2 5 dihydro 2 4 hydroxyphenyl 3h pyrazolo 4 3 c quinolin 3 one	[[0,1,0,0,0,0,0,0,0,0,0]]C1313227	mbi 226	[[0,1,0,0,0,0,0,0,0,0,0]]C1874488	benzalkonium cetylpyridinium chloroxylenol triacetin	[[0,0,0,0,0,0,0,0,0,0,1]]C0609912	11 deoxy 16 16 trimethyleneprostaglandin e1,1alpha 2beta 1e 3s 2 3 1 butylcyclobutyl 3 hydroxy 1 propenyl 5 oxo cyclopentaneheptanoic acid	[[0,1,0,0,0,0,0,0,0,0,0],[0,1,0,0,0,0,0,0,0,0,0]]C0609913	6 7 dichloro 2 3 dihydro 5 2 thienylcarbonyl benzofuran 2 carboxylic acid	[[0,1,0,0,0,0,0,0,0,0,0]]C0609911	11 deoxy 10 hydroxyprostaglandin e1 methyl ester,10alpha 13e 15s methyl ester 10 15 dihydroxy 9 oxo prost 13 en 1 oic acid	[[0,1,0,0,0,0,0,0,0,0,0],[0,1,0,0,0,0,0,0,0,0,0]]C1874482	benzalkonium chloride hydroxypropyl methylcellulose polyvinyl al	[[0,0,0,0,0,0,0,0,0,0,1]]C0609914	diethylenetriamine 7 9 dimethylhypoxanthine platinum ii ff 6 2,bis hexafluorophosphate 1 sp 4 2 n 2 aminoethyl 1 2 ethanediamine n n n 6 9 dihydro 7 9 dimethyl 6 oxo 1h puriniumato n 1 platinum 2	[[0,1,0,0,0,0,0,0,0,0,0],[0,1,0,0,0,0,0,0,0,0,0]]C1962275	chlor tan a 12	[[0,0,0,0,0,0,0,1,0,0,0]]C2341363	ricola	[[0,0,0,0,0,0,0,1,0,0,0]]C0888674	11c labeled 2 aminobicyclo 2 2 1 heptane 2 carboxylic acid	[[0,1,0,0,0,0,0,0,0,0,0]]C0888675	fumarate aminorex fumarate	[[0,1,0,0,0,0,0,0,0,0,0]]C0888672	endo isomer 2 aminobicyclo 2 2 1 heptane 2 carboxylic acid	[[0,1,0,0,0,0,0,0,0,0,0]]C0888673	exo isomer 2 aminobicyclo 2 2 1 heptane 2 carboxylic acid	[[0,1,0,0,0,0,0,0,0,0,0]]C0651559	3 hydroxy 24 dimethylaminochol 8 14 en 15 one,3 hdceo	[[0,1,0,0,0,0,0,0,0,0,0],[0,1,0,0,0,0,0,0,0,0,0]]C0888671	1r exo isomer 2 aminobicyclo 2 2 1 heptane 2 carboxylic acid	[[0,1,0,0,0,0,0,0,0,0,0]]C0043682	1 methylspiro adamantane 2 3 pyrrolidine maleate	[[0,1,0,0,0,0,0,0,0,0,0]]C0056708	cyclo alanine 1 amino 1 cyclopentane carbonyl,alaptide,cc,cyclo,cyclo 1 aminocyclopentanecarbonyl ala	[[0,1,0,0,0,0,0,0,0,0,0],[0,1,0,0,0,0,0,0,0,0,0],[0,1,0,0,0,0,0,0,0,0,0],[0,1,0,0,0,0,0,0,0,0,0],[0,1,0,0,0,0,0,0,0,0,0]]C0043680	1 hydroxyestragole,alpha ethenyl 4 methoxybenzenemethanol	[[0,1,0,0,0,0,0,0,0,0,0],[0,1,0,0,0,0,0,0,0,0,0]]C1302152	atropine sulfate hyoscyamine sulfate scopolamine hydrobromide,atropine sulphate hyoscyamine sulphate scopolamine hydrobromide	[[1,0,0,0,0,0,0,0,0,0,0],[1,0,0,0,0,0,0,0,0,0,0]]C0043686	1 1 1 1 biphenyl 4 4 diylbis 2 hydroxy 2 1 ethanediyl bis 1 4 dimethylpiperidinium,hemicholinium 3 a 5	[[0,1,0,0,0,0,0,0,0,0,0],[0,1,0,0,0,0,0,0,0,0,0]]C1302154	acetaminophen promethazine hydrochloride,paracetamol promethazine hydrochloride	[[1,0,0,0,0,0,0,0,0,0,0],[1,0,0,0,0,0,0,0,0,0,0]]C0953871	l arabinose r isomer prumycin dihydrochloride	[[0,1,0,0,0,0,0,0,0,0,0]]C0043685	1 1 3 3 3 3 hexamethylindocarbocyanine,diic1	[[0,1,0,0,0,0,0,0,0,0,0],[0,1,0,0,0,0,0,0,0,0,0]]C0092502	2 amino 5 hydroxy 4 oxopentanoic acid,2 amino 5 hydroxylevulinic acid,5 hydroxy 2 aminolevulinic acid,5 hydroxy 4 oxonorvaline,5 hydroxy 4 oxo l norvaline	[[0,1,0,0,0,0,0,0,0,0,0],[0,1,0,0,0,0,0,0,0,0,0],[0,1,0,0,0,0,0,0,0,0,0],[0,1,0,0,0,0,0,0,0,0,0],[0,1,0,0,0,0,0,0,0,0,0]]C0043689	1 1 methylethanediylidenedinitrilo bis 3 aminoguanidine,2 2 1 methyl 1 2 ethanediylidene bis carbonimidic dihydrazide,mbag	[[0,1,0,0,0,0,0,0,0,0,0],[0,1,0,0,0,0,0,0,0,0,0],[0,1,0,0,0,0,0,0,0,0,0]]C0056705	cyclo 3	[[0,1,0,0,0,0,0,0,0,0,0]]C0056704	cyclindole,2 3 4 9 tetrahydro n n dimethyl 1h carbazol 3 amine,3 dimethylamino 1 2 3 4 tetrahydrocarbazole	[[0,1,0,0,0,0,0,0,0,1,0],[0,1,0,0,0,0,0,0,0,0,0],[0,1,0,0,0,0,0,0,0,0,0]]C0056707	cyclo,cyclo aptlt	[[0,1,0,0,0,0,0,0,0,0,0],[0,1,0,0,0,0,0,0,0,0,0]]C0666493	tinopal swn	[[0,1,0,0,0,0,0,0,0,0,0]]C0950789	pd 128763	[[0,1,0,0,0,0,0,0,0,0,0]]C2742424	arnamial	[[0,1,0,0,0,0,0,0,0,0,0]]C0666497	nk 104	[[0,1,0,0,0,0,0,0,0,0,0]]C0118954	gadolinium 1 4 7 tris carboxymethyl 10 2 hydroxypropyl 1 4 7 10 tetraazacyclododecane,gadolinium hp do3a,gd hp d03a,gd hydroxypropyl d03a	[[0,1,0,0,0,0,0,0,0,0,0],[0,1,0,0,0,0,0,0,0,0,0],[0,1,0,0,0,0,0,0,0,0,0],[0,1,0,0,0,0,0,0,0,0,0]]C0950786	5 4 chlorophenyl 4 ethyl 2 4 dihydro 2 methyl 3h 1 2 4 triazol 3 one	[[0,1,0,0,0,0,0,0,0,0,0]]C0950787	aj 2615	[[0,1,0,0,0,0,0,0,0,0,0]]C1144292	saucernetilignan 8,sc 8 cpd	[[0,1,0,0,0,0,0,0,0,0,0],[0,1,0,0,0,0,0,0,0,0,0]]C0950783	1 methyl 4 1 naphthylvinyl 1 2 3 6 tetrahydropyridine hydrochloride,e hydrochloride 1 2 3 6 tetrahydro 1 methyl 4 2 1 naphthalenyl ethenyl pyridine	[[0,1,0,0,0,0,0,0,0,0,0],[0,1,0,0,0,0,0,0,0,0,0]]C0388131	n acetyl tyrosyl valyl alanyl aspartyl chloromethyl ketone,ac tyr val ala asp cmk,n acetyl tyrosinyl valinyl alanyl aspartyl chloromethyl ketone,n acetyltyrosinylvalinylalanylaspartylchloromethyl ketone,yvad cmk,yvad chloromethylketone,ac yvad cmk	[[0,1,0,0,0,0,0,0,0,0,0],[0,1,0,0,0,0,0,0,0,0,0],[0,1,0,0,0,0,0,0,0,0,0],[0,1,0,0,0,0,0,0,0,0,0],[0,1,0,0,0,0,0,0,0,0,0],[0,1,0,0,0,0,0,0,0,0,0],[0,1,0,0,0,0,0,0,0,0,0]]C0667725	kln 93	[[0,1,0,0,0,0,0,0,0,0,0]]C0130682	n acetyl s farnesylcysteine,trans farnesyl l cysteine n acetyl s trans,n afc,n acetylfarnesylcysteine	[[0,1,0,0,0,0,0,0,0,0,0],[0,1,0,0,0,0,0,0,0,0,0],[0,1,0,0,0,0,0,0,0,0,0],[0,1,0,0,0,0,0,0,0,0,0]]C2825358	amphomycin calcium	[[0,0,0,0,0,0,0,0,0,1,0]]C0138058	potesept	[[0,1,0,0,0,0,0,0,0,0,0]]C0005323	bethanechol compounds	[[0,1,0,0,0,0,0,0,0,0,1]]C2946387	cepacol sore throat plus cough	[[0,0,0,0,0,0,0,1,0,0,0]]C0005321	betazole,ametazole,1h pyrazole 3 ethanamine	[[0,0,0,0,0,0,0,0,0,1,0],[0,0,0,0,0,0,0,0,0,0,1],[0,0,0,0,0,0,0,0,0,0,1]]C0005320	betaxolol,1 4 2 cyclopropylmethoxy ethyl phenoxy 3 1 methylethyl amino 2 propanol	[[0,0,0,0,0,0,1,1,0,1,0],[0,0,0,0,0,0,0,0,0,1,1]]C0894031	sulfate of laurosept	[[0,1,0,0,0,0,0,0,0,0,0]]C0894030	chloride of laurosept	[[0,1,0,0,0,0,0,0,0,0,0]]C0005324	bethanidine,betanidine,n n dimethyl n phenylmethyl guanidine	[[0,0,0,0,0,0,0,1,0,1,0],[0,0,0,0,0,0,0,0,0,0,1],[0,0,0,0,0,0,0,0,0,0,1]]C0654451	muzigadial	[[0,1,0,0,0,0,0,0,0,0,0]]C0654450	canellal	[[0,1,0,0,0,0,0,0,0,0,0]]C3244322	donatussin syrup	[[0,0,0,0,0,0,0,1,0,0,0]]C1981962	azathioprine 124 bld ser plas	[[0,0,0,1,0,0,0,0,0,0,0]]C0625627	tylosema esculenturm protease inhibitor protein,marama bean protease inhibitor,tylosema esculenturm trypsin inhibitor	[[0,1,0,0,0,0,0,0,0,0,0],[0,1,0,0,0,0,0,0,0,0,0],[0,1,0,0,0,0,0,0,0,0,0]]C0644260	trisgalactosylglycine,n tris galactopyranosyloxymethyl glycine methylamide	[[0,1,0,0,0,0,0,0,0,0,0],[0,1,0,0,0,0,0,0,0,0,0]]C0625623	1 3 dioleoyl 2 4 pyrenylbutanoyl glycerol,dpbg	[[0,1,0,0,0,0,0,0,0,0,0],[0,1,0,0,0,0,0,0,0,0,0]]C0525944	cerebyx	[[0,0,0,0,0,0,0,0,0,1,0]]C0967759	human birc8 protein,human ilp 2 protein,human baculoviral iap repeat containing 8 protein,human iap like protein 2,human ilp2 protein	[[0,1,0,0,0,0,0,0,0,0,0],[0,1,0,0,0,0,0,0,0,0,0],[0,1,0,0,0,0,0,0,0,0,0],[0,1,0,0,0,0,0,0,0,0,0],[0,1,0,0,0,0,0,0,0,0,0]]C1981960	azatadine 124 bld ser plas	[[0,0,0,1,0,0,0,0,0,0,0]]C0625629	5 hydroxyimino 4 methoxy 2 pivaloylimino thiazolidine 3 acetamide,him pita	[[0,1,0,0,0,0,0,0,0,0,0],[0,1,0,0,0,0,0,0,0,0,0]]C2955631	amturnide	[[0,0,0,0,0,0,0,1,0,0,0]]C0032017	posterior pituitary hormones,posterior pituitary,neurohypophyseal hormones,neurohypophyseal peptides	[[1,1,0,0,0,0,1,0,0,0,1],[0,0,0,0,0,0,1,0,0,0,1],[0,0,0,0,0,0,0,0,1,0,1],[0,0,0,0,0,0,0,0,0,0,1]]C0060747	frc 8411	[[0,1,0,0,0,0,0,0,0,0,0]]C0283199	dioctahydral smectite	[[0,1,0,0,0,0,0,0,0,0,0]]C0104882	azuletil sodium	[[0,1,0,0,0,0,0,0,0,0,0]]C0104881	azulekeep	[[0,1,0,0,0,0,0,0,0,0,0]]C0104886	b 10190	[[0,1,0,0,0,0,0,0,0,0,0]]C0104887	b 10610	[[0,1,0,0,0,0,0,0,0,0,0]]C0627664	phenylmethylsulfonyl trypsin,pms trypsin	[[0,1,0,0,0,0,0,0,0,0,0],[0,1,0,0,0,0,0,0,0,0,0]]C0104888	b 11 420	[[0,1,0,0,0,0,0,0,0,0,0]]C2350808	kos 1022,kos1022	[[0,1,0,0,0,0,0,0,0,0,0],[0,1,0,0,0,0,0,0,0,0,0]]C0650204	2 chloro 5 8 9 10 tetrahydro 5 9 methanobenzocycloocten 8 amine hydrochloride	[[0,1,0,0,0,0,0,0,0,0,0]]C1170001	eprosartan hydrochlorothiazide,eprosartan mesylate hydrochlorothiazide	[[1,0,0,0,0,0,0,1,0,0,0],[1,0,0,0,0,0,0,0,0,0,0]]C0627662	technetium tc 99m tetrasulfophthalocyanine,tc 99m tspc,technetium phthalocyanine tetrasulfonate	[[0,1,0,0,0,0,0,0,0,0,0],[0,1,0,0,0,0,0,0,0,0,0],[0,1,0,0,0,0,0,0,0,0,0]]C3247211	bupivacaine liposome	[[0,0,0,0,0,0,0,1,0,0,0]]C2315166	rectal form trimethobenzamide	[[1,0,0,0,0,0,0,0,0,0,0]]C0286018	rx 821001	[[0,1,0,0,0,0,0,0,0,0,0]]C0389883	isis 5132,isis5132,isis 5132 cgp69846a	[[0,0,0,0,0,0,0,0,0,1,0],[0,1,0,0,0,0,0,0,0,0,0],[0,0,0,0,0,0,0,0,0,1,0]]C0286015	r 30490,r30490	[[0,1,0,0,0,0,0,0,0,0,0],[0,1,0,0,0,0,0,0,0,0,0]]C0959203	trans isomer cyclazosin	[[0,1,0,0,0,0,0,0,0,0,0]]C0959200	4 octylphenol barium	[[0,1,0,0,0,0,0,0,0,0,0]]C0959201	4 octylphenol potassium	[[0,1,0,0,0,0,0,0,0,0,0]]C0959206	thaliporphine hydrochloride	[[0,1,0,0,0,0,0,0,0,0,0]]C0959207	e z z z isomer 5 oxo 6 8 11 14 eicosatetraenoic acid	[[0,1,0,0,0,0,0,0,0,0,0]]C0285690	n 2 2 heptyloxyphenylcarbamoyloxy ethyl n benzylpiperdinium,n 2 2 heptyloxyphenylcarbamoyloxy ethyl n benzylpiperidinium chloride,substance h b,hpcbp	[[0,1,0,0,0,0,0,0,0,0,0],[0,1,0,0,0,0,0,0,0,0,0],[0,1,0,0,0,0,0,0,0,0,0],[0,1,0,0,0,0,0,0,0,0,0]]C1724475	gum numb gel	[[0,0,0,0,0,0,0,1,0,0,0]]C2948671	colgate proclinical	[[0,0,0,0,0,0,0,1,0,0,0]]C3181735	dpj rg 1219	[[0,1,0,0,0,0,0,0,0,0,0]]C3181736	16 4 3 imidazol 1 yl propoxy 3 methoxybenzylidene 4 androstene 3 17 dione	[[0,1,0,0,0,0,0,0,0,0,0]]C1450003	cerazet,alcala brand of levonorgestrel	[[0,1,0,0,0,0,0,0,0,0,0],[0,1,0,0,0,0,0,0,0,0,0]]C0756858	1 chloro 4 3 chloro 4 methoxybenzyl amino 6 phthalazinecarbonitrile,1 chloro 4 cmbzp	[[0,1,0,0,0,0,0,0,0,0,0],[0,1,0,0,0,0,0,0,0,0,0]]C0603981	1 2 3 4 5 6 tetrahydropyridyl 1 3 pentadiene,e e 2 3 4 5 tetrahydro 6 1 3 pentadienyl pyridine	[[0,1,0,0,0,0,0,0,0,0,0],[0,1,0,0,0,0,0,0,0,0,0]]C0081026	4 methylthiopropranolol,1 1 methylethyl amino 3 4 methylthio 1 naphthalenyl oxy 2 propanol	[[0,1,0,0,0,0,0,0,0,0,0],[0,1,0,0,0,0,0,0,0,0,0]]C0603984	1 2 9 10 tetrahydroxyaporphine	[[0,1,0,0,0,0,0,0,0,0,0]]C2974078	6 o 3 4 dihydroxy 2 methylenbutyryl 1 o trans cinnamoyl beta d glucopyranose,6 dmcg cpd	[[0,1,0,0,0,0,0,0,0,0,0],[0,1,0,0,0,0,0,0,0,0,0]]C0377892	abeorphine	[[0,1,0,0,0,0,0,0,0,0,0]]C0377896	emimycin riboside,1 beta ribofuranosyl 2 1h pyrazinone 4 oxide	[[0,1,0,0,0,0,0,0,0,0,0],[0,1,0,0,0,0,0,0,0,0,0]]C0653155	gt32 a	[[0,1,0,0,0,0,0,0,0,0,0]]C0653157	3 demethylchartreusin	[[0,1,0,0,0,0,0,0,0,0,0]]C0653156	be 14106,20 2 hexenyl 9 10 dihydroxy 7 15 dimethyl azacycloeicosa 3 5 7 11 13 15 17 heptaen 2 one	[[0,1,0,0,0,0,0,0,0,0,0],[0,1,0,0,0,0,0,0,0,0,0]]C0653153	sphingofungin d,2 acetylamino 3 4 5 14 tetrahydroxy 6 eicosenoic acid	[[0,1,0,0,0,0,0,0,0,0,0],[0,1,0,0,0,0,0,0,0,0,0]]C0289779	2 4 thiazolidinedione,thiazolidinedione	[[0,1,0,0,0,0,0,0,1,0,0],[0,0,0,0,0,0,1,0,0,0,0]]C0893565	hb 19 peptide,5 lys psi ch2n pro arg thr ala ser pro,lysyl psi ch2n prolyl arginyl 5 threonyl alanyl seryl proline,5 kpsi ch2n pr tasp	[[0,1,0,0,0,0,0,0,0,0,0],[0,1,0,0,0,0,0,0,0,0,0],[0,1,0,0,0,0,0,0,0,0,0],[0,1,0,0,0,0,0,0,0,0,0]]C0653159	adipostatin b,5 isopentadecylresorcinol	[[0,1,0,0,0,0,0,0,0,0,0],[0,1,0,0,0,0,0,0,0,0,0]]C2699139	cefetrizole	[[0,0,0,0,0,0,0,0,0,1,0]]C1698138	triaminic flu cough fever	[[0,0,0,0,0,0,0,1,0,0,0]]C0083880	org 5222	[[0,1,0,0,0,0,0,0,0,0,0]]C3256121	bitter fennel	[[0,0,1,0,0,0,0,0,0,0,0]]C0083882	org 9991	[[0,1,0,0,0,0,0,0,0,0,0]]C2699138	cefetecol	[[0,0,0,0,0,0,0,0,0,1,0]]C1330158	cold cough pd	[[0,0,0,0,0,0,0,1,0,0,0]]C0757108	2 2 phenylcyclopropyl propane	[[0,1,0,0,0,0,0,0,0,0,0]]C3256126	borate ion	[[0,0,1,0,0,0,0,0,0,0,0]]C3256127	bos taurus colostrum	[[0,0,1,0,0,0,0,0,0,0,0]]C1699844	guaphen forte	[[0,0,0,0,0,0,0,1,0,0,0]]C1450523	3 4 4 nitrophenoxy phenyl 1h pyrazole 1 carboxamide,3 no2phoph pyrazole conh2	[[0,1,0,0,0,0,0,0,0,0,0],[0,1,0,0,0,0,0,0,0,0,0]]C0757107	2 phenylcyclopropyl ethane	[[0,1,0,0,0,0,0,0,0,0,0]]C0214374	leucettamine a	[[0,1,0,0,0,0,0,0,0,0,0]]C0757100	2 azido 2 deoxyuridine	[[0,1,0,0,0,0,0,0,0,0,0]]C3192843	biotene dry mouth	[[0,0,0,0,0,0,0,1,0,0,0]]C0757102	2 azido 2 deoxyuridine 5 monophosphate bis pivaloyloxymethyl ester,n3dump bis	[[0,1,0,0,0,0,0,0,0,0,0],[0,1,0,0,0,0,0,0,0,0,0]]C0627088	e 1309,s s dioxide 4 2 5 2 diethylamino ethyl amino 4 methyl 4h 1 2 4 6 thiatriazin 3 yl amino ethyl thio methyl 2 thiazolyl guanidine	[[0,1,0,0,0,0,0,0,0,0,0],[0,1,0,0,0,0,0,0,0,0,0]]C0887333	hydrochloride 2 5 dimethoxy 4 methylamphetamine	[[0,1,0,0,0,0,0,0,0,0,0]]C0599934	peptide vaccines	[[0,1,0,0,0,0,0,0,1,1,0]]C0887337	s isomer 2 5 dimethoxy 4 methylamphetamine	[[0,1,0,0,0,0,0,0,0,0,0]]C0887334	isomer 2 5 dimethoxy 4 methylamphetamine	[[0,1,0,0,0,0,0,0,0,0,0]]C0887335	isomer 2 5 dimethoxy 4 methylamphetamine hydrochloride	[[0,1,0,0,0,0,0,0,0,0,0]]C0887338	r isomer 2 5 dimethoxy 4 methylamphetamine hydrochloride	[[0,1,0,0,0,0,0,0,0,0,0]]C0627083	bpaa me	[[0,1,0,0,0,0,0,0,0,0,0]]C0627084	4 biphenylylacetic acid methyl ester	[[0,1,0,0,0,0,0,0,0,0,0]]C1272910	radiopharmaceutical preparation kit,therapeutic radiopharmaceutical kit	[[1,0,0,0,0,0,0,0,0,0,0],[1,0,0,0,0,0,0,0,0,0,0]]C0627086	3 2 diethylaminoethylamino 5 2 2 guanidinothiazol 4 yl methylthio ethylamino 4 methyl 1 2 4 6 thiatriazine 1 1 dioxide	[[0,1,0,0,0,0,0,0,0,0,0]]C3255749	chinese cinnamon leaf oil	[[0,0,1,0,0,0,0,0,0,0,0]]C0040562	toyocamycin,toyokamycin,deazacyanoadenosine,4 amino 7 beta d ribofuranosyl 7h pyrrolo 2 3 d pyrimidine 5 carbonitrile	[[0,0,0,0,0,0,0,0,1,0,1],[0,0,0,0,0,0,0,0,0,0,1],[0,0,0,0,0,0,0,0,0,0,1],[0,0,0,0,0,0,0,0,0,0,1]]C0040563	chromomycin a3,3d o 4 o acetyl 2 6 dideoxy 3 c methyl alpha l arabino hexopyranosyl 7 methyl olivomycin d,chromomycin a 3,antibiotic b 599,aburamycin b,6 dideoxy 3 c methyl alpha l arabino hexopyranosyl 7 methyl 9ci 3b o 4 o acetyl 2 olivomycin d,3b o 4 o acetyl 2 6 dideoxy 3 c methyl alpha l arabino hexopyranosyl 7 methylolivomycin d,antibiotic from streptomyces griseus	[[0,0,0,0,1,0,0,0,0,1,1],[0,0,0,0,0,0,0,0,0,0,1],[0,0,0,0,1,0,0,0,0,1,0],[0,0,0,0,0,0,0,0,0,1,0],[0,0,0,0,0,0,0,0,0,1,0],[0,0,0,0,1,0,0,0,0,1,0],[0,0,0,0,1,0,0,0,0,0,0],[0,0,0,0,0,0,0,0,0,1,0]]C0721933	naldelate	[[0,0,0,0,0,0,0,1,0,0,0]]C0038430	streptovaricin	[[0,0,0,0,0,0,0,0,1,0,1]]C0384938	cni 1493	[[0,1,0,0,0,0,1,0,0,0,0]]C0384939	n n bis 3 5 diacetylphenyl decanediamide tetrakis amidinohydrazone tetrahydrochloride	[[0,1,0,0,0,0,0,0,0,0,0]]C0721937	nalex	[[0,0,0,0,0,0,1,1,0,0,0]]C0721936	naldelate pediatric	[[0,0,0,0,0,0,0,1,0,0,0]]C0721939	nalex jr	[[0,0,0,0,0,0,0,1,0,0,0]]C0721938	nalex expectorant	[[0,0,0,0,0,0,0,1,0,0,0]]C0136278	pge1 oligomer	[[0,1,0,0,0,0,0,0,0,0,0]]C2699131	cefaclor anhydrous	[[0,0,0,0,0,0,0,0,0,1,0]]C1652085	aczone	[[0,0,0,0,0,0,0,0,0,1,0]]C2699130	cedefingol	[[0,0,0,0,0,0,0,0,0,1,0]]C0597153	oxidoreductase inhibitor	[[0,0,0,0,0,0,0,0,1,0,0]]C2699137	cefempidone	[[0,0,0,0,0,0,0,0,0,1,0]]C1652089	ammonul	[[0,0,0,0,0,0,0,1,0,0,0]]C1564686	eto gry,gry brand of etoposide	[[0,1,0,0,0,0,0,0,0,0,0],[0,1,0,0,0,0,0,0,0,0,0]]C0720086	duragen	[[0,0,0,0,0,0,1,1,0,0,0]]C0720087	duralex	[[0,0,0,0,0,0,0,1,0,0,0]]C0720084	duraganidin	[[0,0,0,0,0,0,0,1,0,0,0]]C0720085	duraganidin nr	[[0,0,0,0,0,0,0,1,0,0,0]]C0720082	duradryl,duradryl syrup	[[0,0,0,0,0,0,1,1,0,0,0],[0,0,0,0,0,0,1,0,0,0,0]]C0720083	duragal s	[[0,0,0,0,0,0,0,1,0,0,0]]C0720080	duradal hd plus	[[0,0,0,0,0,0,0,1,0,0,0]]C0720081	duradrin	[[0,0,0,0,0,0,1,1,0,0,0]]C0287763	nsc 650893	[[0,1,0,0,0,0,0,0,0,0,0]]C1613403	n 2 chloro 5 methoxyphenyl 6 methoxy 7 1 methyl 4 piperidinyl methoxy 4 quinazolinamine	[[0,1,0,0,0,0,0,0,0,0,0]]C0287761	2 7 difluoro 4 methoxyspiro 9h fluorene 9 4 imidazolidine 2 5 dione	[[0,1,0,0,0,0,0,0,0,0,0]]C0287760	nsc 640737 m,nsc 640737m	[[0,1,0,0,0,0,0,0,0,0,0],[0,1,0,0,0,0,0,0,0,0,0]]C0287766	dtx 2,dtx2	[[0,1,0,0,0,0,0,0,0,0,0],[0,1,0,0,0,0,0,0,0,0,0]]C0720088	duralone	[[0,0,0,0,0,0,0,0,0,1,0]]C0129752	n alpha dansyl l arginine 4 ethylpiperidine amide,dansyl arg 4 ethyl piperidine amide	[[0,1,0,0,0,0,0,0,0,0,0],[0,1,0,0,0,0,0,0,0,0,0]]C0960019	2s 2alpha 5alpha 6beta isomer carbenicillin indanyl	[[0,1,0,0,0,0,0,0,0,0,0]]C0723574	t athlete	[[0,0,0,0,0,0,0,1,0,0,0]]C0723571	syprine,merck brand of trientine hydrochloride	[[0,0,0,0,0,0,0,1,0,0,0],[0,1,0,0,0,0,0,0,0,0,0]]C0694746	mr vaccine,m r	[[0,0,0,0,0,1,1,0,0,0,0],[0,0,0,0,0,0,1,0,0,0,0]]C0960013	isomer butriptyline	[[0,1,0,0,0,0,0,0,0,0,0]]C0960012	bufrolin disodium salt	[[0,1,0,0,0,0,0,0,0,0,0]]C0960011	triethylcholine iodide	[[0,1,0,0,0,0,0,0,0,0,0]]C0960010	triethylcholine bromide	[[0,1,0,0,0,0,0,0,0,0,0]]C0723579	t painol	[[0,0,0,0,0,0,0,1,0,0,0]]C0723578	t lite	[[0,0,0,0,0,0,1,0,0,0,0]]C2948529	roasted spongia officinalis skeleton	[[0,0,1,0,0,0,0,0,0,0,0]]C2947582	smart san	[[0,0,0,0,0,0,0,1,0,0,0]]C1699266	vivaglobin	[[0,0,0,0,0,0,0,1,0,0,0]]C0939352	hyosophen sr	[[0,0,0,0,0,0,0,1,0,0,0]]C1563842	metasedin,esteve brand of methadone hydrochloride	[[0,1,0,0,0,0,0,0,0,0,0],[0,1,0,0,0,0,0,0,0,0,0]]C0391168	n methyl 4 piperidyl acetate,mp4a cpd	[[0,1,0,0,0,0,0,0,0,0,0],[0,1,0,0,0,0,0,0,0,0,0]]C0066746	monodansylcadaverine,dansylcadaverine,n 5 aminopentyl 5 dimethylamino 1 naphthalenesulfonamide	[[0,1,0,0,0,0,0,0,0,0,0],[0,1,0,0,0,0,0,0,0,0,0],[0,1,0,0,0,0,0,0,0,0,0]]C0885181	cayenne pepper homeopathic preparation	[[0,0,0,0,0,1,0,0,0,0,0]]C0885180	cantharis vesicatoria preparation,spanish fly,homeopathic spanish fly	[[0,0,0,0,0,0,0,1,0,0,0],[0,0,0,0,0,0,1,0,0,0,0],[0,0,0,0,0,0,1,0,0,0,0]]C0885187	nosode of cancer carcinosin	[[0,0,0,0,0,1,0,0,0,0,0]]C0066747	monodansylthiacadaverine,mdtc	[[0,1,0,0,0,0,0,0,0,0,0],[0,1,0,0,0,0,0,0,0,0,0]]C0606852	dikar	[[0,1,0,0,0,0,0,0,0,0,0]]C2604088	naphthalecin	[[0,1,0,0,0,0,0,0,0,0,0]]C0606855	dimecoumarone,diiodide n 2 benzofuranylmethyl n ethyl n n n n tetramethyl 1 2 ethanediaminium,n ethyl n benzofurfuryl n n dimethylethylenediamine iodomethylated,dimekumaron	[[0,1,0,0,0,0,0,0,0,0,0],[0,1,0,0,0,0,0,0,0,0,0],[0,1,0,0,0,0,0,0,0,0,0],[0,1,0,0,0,0,0,0,0,0,0]]C0590685	tildiem la	[[0,0,0,0,0,0,1,1,0,0,0]]C0590684	tildiem retard	[[0,0,0,0,0,0,0,1,0,0,0]]C0590687	angeze	[[0,0,0,0,0,0,0,1,0,0,0]]C0590686	coracten	[[0,0,0,0,0,0,0,1,0,0,0]]C0590681	metazem	[[0,0,0,0,0,0,0,1,0,0,0]]C0590680	dilzem xl	[[0,0,0,0,0,0,0,1,0,0,0]]C0590683	tildiem	[[0,0,0,0,0,0,1,1,0,0,0]]C0590682	slozem	[[0,0,0,0,0,0,0,1,0,0,0]]C0381433	visoltricin,3 1 methyl 4 3 methyl 2 butenyl imidazol 5 yl 2 propenoic acid methyl ester	[[0,1,0,0,0,0,0,0,0,0,0],[0,1,0,0,0,0,0,0,0,0,0]]C0965993	bay36 7620,bay367620	[[0,1,0,0,0,0,0,0,0,0,0],[0,1,0,0,0,0,0,0,0,0,0]]C0381431	disperse red 118,red brown g	[[0,1,0,0,0,0,0,0,0,0,0],[0,1,0,0,0,0,0,0,0,0,0]]C0965995	desglucobalhimycin,desgluco balhimycin	[[0,1,0,0,0,0,0,0,0,0,0],[0,1,0,0,0,0,0,0,0,0,0]]C0590688	elantan	[[0,1,0,0,0,0,0,0,0,0,0]]C0965996	dpe28,2 4 dinitrophenyl 2 6 di tertiarybutyl phenyl ether,dpe 28	[[0,1,0,0,0,0,0,0,0,0,0],[0,1,0,0,0,0,0,0,0,0,0],[0,1,0,0,0,0,0,0,0,0,0]]C3256194	butyl monoester of polymethylvinyl ether maleic acid	[[0,0,1,0,0,0,0,0,0,0,0]]C0041214	trypanocidal agents,trypanocides,trypanosomicidal agents,trypanosomicides,trypanocidal drugs	[[0,1,0,0,0,0,0,0,0,0,0],[0,1,0,0,0,0,0,0,0,0,0],[0,1,0,0,0,0,0,0,0,0,0],[0,1,0,0,0,0,0,0,0,0,0],[0,1,0,0,0,0,0,0,0,0,0]]C0607185	plexafer f	[[0,1,0,0,0,0,0,0,0,0,0]]C1098802	5 ethyl 2 mercaptothiazole	[[0,1,0,0,0,0,0,0,0,0,0]]C0552314	aminobenzoate	[[0,0,0,0,0,0,0,1,0,0,0]]C1098807	1 phenyl 2 1 aminopropyl n n diethylcyclopropanecarboxamide,ppdc cpd	[[0,1,0,0,0,0,0,0,0,0,0],[0,1,0,0,0,0,0,0,0,0,0]]C2928514	gonadorelin thyrotropin releasing hormone	[[0,0,0,0,0,0,0,1,0,0,0]]C0173117	n hydroxy n 2 fluorenyl benzamide,n oh 2 fba	[[0,1,0,0,0,0,0,0,0,0,0],[0,1,0,0,0,0,0,0,0,0,0]]C2928516	echinacea preparation goldenseal preparation	[[0,0,0,0,0,0,0,1,0,0,0]]C2928517	hyoscyamine methenamine methylene blue phenyl salicylate	[[0,0,0,0,0,0,0,1,0,0,0]]C2928510	chlorzoxazone flufenamate	[[0,0,0,0,0,0,0,1,0,0,0]]C2928511	dehydrocholate papaverine	[[0,0,0,0,0,0,0,1,0,0,0]]C2928512	butacaine oleate	[[0,0,0,0,0,0,0,1,0,0,0]]C2928513	lanolin petrolatum zinc oxide	[[0,0,0,0,0,0,0,1,0,0,0]]C2928518	folic acid polysaccharide iron complex vitamin b 12	[[0,0,0,0,0,0,0,1,0,0,0]]C2928519	brilliant green lactate	[[0,0,0,0,0,0,0,1,0,0,0]]C0614656	disulfodisalicylidenepropane 1 1 diamine,dsspd	[[0,1,0,0,0,0,0,0,0,0,0],[0,1,0,0,0,0,0,0,0,0,0]]C0359183	intravenous nutrition,intravenous nutrition amino acids	[[1,0,0,0,0,0,0,0,0,0,0],[1,0,0,0,0,0,0,0,0,0,0]]C0722629	pilocar	[[0,0,0,0,0,0,1,1,0,0,0]]C0359181	perfluorochemical,perfluoro compound	[[0,0,0,0,0,0,1,1,0,0,1],[1,0,0,0,0,0,0,0,0,0,0]]C0359180	albumin solution,human albumin solution	[[1,0,0,0,0,0,1,0,0,0,0],[1,0,0,0,0,0,0,0,0,0,0]]C0359187	intravenous nutrition,intravenous nutrition vitamins	[[1,0,0,0,0,0,0,0,0,0,0],[1,0,0,0,0,0,0,0,0,0,0]]C0359186	intravenous nutrition,intravenous nutrition ready mixed	[[1,0,0,0,0,0,0,0,0,0,0],[1,0,0,0,0,0,0,0,0,0,0]]C0359185	intravenous nutrition,intravenous nutrition carbohydrate	[[1,0,0,0,0,0,0,0,0,0,0],[1,0,0,0,0,0,0,0,0,0,0]]C0359184	intravenous nutrition,intravenous nutrition fats	[[1,0,0,0,0,0,0,0,0,0,0],[1,0,0,0,0,0,0,0,0,0,0]]C0722620	phos flur	[[0,0,0,0,0,0,0,1,0,0,0]]C0103565	antide	[[0,1,0,0,0,0,0,0,0,0,0]]C0722622	phosphate fluoride	[[0,0,0,0,0,0,0,1,0,0,0]]C0359188	intravenous nutrition,intravenous nutrition electrolytes trace elements	[[1,0,0,0,0,0,0,0,0,0,0],[1,0,0,0,0,0,0,0,0,0,0]]C0722624	phrenilin forte	[[0,0,0,0,0,0,1,1,0,0,0]]C0722625	phyllocontin,purdue frederick brand of aminophylline,napp brand of aminophylline	[[0,0,0,0,0,0,0,1,0,0,0],[0,1,0,0,0,0,0,0,0,0,0],[0,1,0,0,0,0,0,0,0,0,0]]C0722626	physiolyte	[[0,0,0,0,0,0,0,1,0,0,0]]C0103563	anticonvertin	[[0,1,0,0,0,0,0,0,0,0,0]]C0699676	clont	[[0,1,0,0,0,0,0,0,0,0,0]]C0699677	danizol	[[0,1,0,0,0,0,0,0,0,0,0]]C0699675	metro i v	[[0,0,0,0,1,0,0,0,0,0,0]]C0699672	dalacin c	[[0,1,0,0,0,0,1,0,0,0,0]]C0699673	cleocin	[[0,0,0,0,0,0,1,1,0,0,0]]C0699670	lotrimin,schering brand of clotrimazole	[[0,0,0,0,0,0,1,1,0,0,0],[0,1,0,0,0,0,0,0,0,0,0]]C0070449	perlan blue	[[0,1,0,0,0,0,0,0,0,0,0]]C0070445	perisoxal,3 1 hydroxy 2 piperidinoethyl 5 phenylisoxazole,alpha 5 phenyl 3 isoxazolyl 1 piperidineethanol	[[0,1,0,0,0,0,0,0,0,0,0],[0,1,0,0,0,0,0,0,0,0,0],[0,1,0,0,0,0,0,0,0,0,0]]C0699678	flagyl	[[0,0,0,0,0,0,1,1,0,0,0]]C0699679	gineflavir	[[0,1,0,0,0,0,0,0,0,0,0]]C0256040	5 isothiocyanonicotine,iscn n	[[0,1,0,0,0,0,0,0,0,0,0],[0,1,0,0,0,0,0,0,0,0,0]]C0078273	vinylethylnitrosamine	[[0,1,0,0,0,0,0,0,0,0,0]]C0972049	13e 15r isomer 11 deoxyprostaglandin e1	[[0,1,0,0,0,0,0,0,0,0,0]]C0634724	9 fluoro 10 4 methyl 1 piperazinyl 7 oxospiro cyclopropane 1 3 2 h 7h pyrido 1 2 3 de 1 4 benzoxazine 6 carboxylic acid,9 fpobc	[[0,1,0,0,0,0,0,0,0,0,0],[0,1,0,0,0,0,0,0,0,0,0]]C0972047	17alpha isomer gestodene	[[0,1,0,0,0,0,0,0,0,0,0]]C0972044	meobentine sulfate	[[0,1,0,0,0,0,0,0,0,0,0]]C3257012	polyoxy 40 stearate	[[0,0,1,0,0,0,0,0,0,0,0]]C0952168	isomer ifenprodil hydrochloride	[[0,1,0,0,0,0,0,0,0,0,0]]C0004847	baxarytmon	[[0,0,0,0,0,0,0,0,0,1,0]]C0634720	s methyl n 1 isolquinolyl methylendithiocarbazate,mimdtc	[[0,1,0,0,0,0,0,0,0,0,0],[0,1,0,0,0,0,0,0,0,0,0]]C2317159	parenteral form apomorphine	[[1,0,0,0,0,0,0,0,0,0,0]]C0607728	doxivenil gel,mixt with 2 5 dihydroxybenzenesulfonic acid calcium salt potassium salt hydrogen sulfate dextran	[[0,1,0,0,0,0,0,0,0,0,0],[0,1,0,0,0,0,0,0,0,0,0]]C0952163	ifenprodil hydrochloride	[[0,1,0,0,0,0,0,0,0,0,0]]C0607722	cyanine dye 10,1 1 diethyl 2 2 tricarbocyanine iodide	[[0,1,0,0,0,0,0,0,0,0,0],[0,1,0,0,0,0,0,0,0,0,0]]C1504305	1 r 2alpha 4alpha 4 4 6 aminosulfonyl 7 chloro 1 2 3 4 tetrahydro 4 oxo 2 quinazolinyl phenoxy 1 3 mercapto 2 methyl 1 oxopropyl l proline	[[0,1,0,0,0,0,0,0,0,0,0]]C2934079	topneuter	[[0,1,0,0,0,0,0,0,0,0,0]]C0630695	3 acetoxymethyl 7 chloro 3 cephem 4 carboxylate 1 1 dioxide tert butylester,amcccdb	[[0,1,0,0,0,0,0,0,0,0,0],[0,1,0,0,0,0,0,0,0,0,0]]C3254247	dioscorolide b	[[0,1,0,0,0,0,0,0,0,0,0]]C1959878	sodium fluoride triclosan	[[1,0,0,0,0,0,0,1,0,0,0]]C0630690	2 guanidino 5 8 dimethoxy 1 2 3 4 tetrahydro 1 4 epoxynaphthalene,2 gdten	[[0,1,0,0,0,0,0,0,0,0,0],[0,1,0,0,0,0,0,0,0,0,0]]C1567463	fluvoxamin stada,stadapharm brand of fluvoxamine maleate	[[0,1,0,0,0,0,0,0,0,0,0],[0,1,0,0,0,0,0,0,0,0,0]]C1959877	ammonium bicarbonate ipecacuanha	[[1,0,0,0,0,0,0,0,0,0,0]]C0146196	toltrazuril,1 methyl 3 3 methyl 4 4 trifluoromethyl thio phenoxy phenyl 1 3 5 triazine 2 4 6 1h 3h 5h trione	[[0,0,0,0,0,0,1,0,0,1,0],[0,1,0,0,0,0,0,0,0,0,0]]C2932892	4 beta glucopyranosyl 2 furyl 5 methy 1 2 glucopyranoside phenylmethanone,gfmgp cpd	[[0,1,0,0,0,0,0,0,0,0,0],[0,1,0,0,0,0,0,0,0,0,0]]C3256197	khaya senegalensis bark	[[0,0,1,0,0,0,0,0,0,0,0]]C2932891	kaempherol 3 o beta rhamnopyranosyl 1 6 beta d glucopyranoside,krg cpd	[[0,1,0,0,0,0,0,0,0,0,0],[0,1,0,0,0,0,0,0,0,0,0]]C3255608	pueraria montana var chinensis root	[[0,0,1,0,0,0,0,0,0,0,0]]C0651964	5 methoxypodophyllotoxin,5r 5alpha 5abeta 8aalpha 9alpha 5 8 8a 9 tetrahydro 9 hydroxy 10 methoxy 5 3 4 5 trimethoxyphenyl furo 3 4 6 7 naphtho 2 3 d 1 3 dioxol 6 5ah one	[[0,1,0,0,0,0,0,0,0,0,0],[0,1,0,0,0,0,0,0,0,0,0]]C0772046	magnesium sulfate heptahydrate,epsom salt	[[0,1,1,0,0,0,1,1,0,1,0],[1,0,0,0,0,0,1,0,0,0,0]]C0651962	timorom	[[0,1,0,0,0,0,0,0,0,0,0]]C0651960	3 7 dimethyl 10 3 4 aminomethyl 5 hydroxy 6 methyl 3 pyridyl propyl 3 7 10 triazatricyclo 3 3 3 0 1 5 undecane	[[0,1,0,0,0,0,0,0,0,0,0]]C0651961	21 4 2 6 di 1 pyrrolidinyl 4 pyrimidinyl 1 piperazinyl pregna 1 4 9 11 triene 3 20 dione monomethanesulfonate	[[0,1,0,0,0,0,0,0,0,0,0]]C2933767	legumain probe 1,lp 1 aza asn epoxide	[[0,1,0,0,0,0,0,0,0,0,0],[0,1,0,0,0,0,0,0,0,0,0]]C0046777	3 4 morpholinyl 3 deaminodaunorubicin,8s cis 8 acetyl 7 8 9 10 tetrahydro 6 8 11 trihydroxy 1 methoxy 10 2 3 6 trideoxy 3 4 morpholinyl alpha l lyxo hexopyranosyl oxy 5 12 naphthacenedione,mdadr,morpholinodaunomycin	[[0,1,0,0,0,0,0,0,0,0,0],[0,1,0,0,0,0,0,0,0,0,0],[0,1,0,0,0,0,0,0,0,0,0],[0,1,0,0,0,0,0,0,0,0,0]]C0392939	recombinant human thrombopoietin,c mpl ligand,megakaryocyte growth and development factor,recombinant human tpo,rhtpo	[[0,0,0,0,1,0,0,0,0,1,0],[0,0,0,0,0,0,0,0,0,1,0],[0,0,0,0,0,0,0,0,0,1,0],[0,0,0,0,1,0,0,0,0,1,0],[0,0,0,0,1,0,0,0,0,0,0]]C0392938	zoledronate,zole,ndc zoledronate,1 hydroxy 2 1h imidazol 1 yl ethylidene bisphosphonic acid	[[1,0,0,0,0,0,1,0,0,0,0],[0,0,0,0,1,0,0,0,0,0,0],[0,0,0,0,0,0,0,0,0,1,0],[0,0,0,0,0,0,0,0,0,1,0]]C0700578	prazosin hydrochloride,prazosin hydrochloride product,prazosin hcl	[[0,1,0,0,0,0,1,0,0,1,0],[1,0,0,0,0,0,0,0,0,0,0],[0,1,0,0,0,0,1,0,0,0,0]]C0957368	l isomer benalaxyl	[[0,1,0,0,0,0,0,0,0,0,0]]C2933769	dendroaspis angusticep adtx1 peptide	[[0,1,0,0,0,0,0,0,0,0,0]]C1869996	sporminarin b	[[0,1,0,0,0,0,0,0,0,0,0]]C0111149	cs 600	[[0,1,0,0,0,0,0,0,0,0,0]]C0077143	trilobine	[[0,1,0,0,0,0,0,0,0,0,0]]C0077141	trilinolein,1 2 3 propanetriyl ester z z 9 12 octadecadienoic acid,trilinoelaidate,trilinoelaidin,trilinoleic glycerol	[[0,1,0,0,0,0,0,0,0,0,0],[0,1,0,0,0,0,0,0,0,0,0],[0,1,0,0,0,0,0,0,0,0,0],[0,1,0,0,0,0,0,0,0,0,0],[0,1,0,0,0,0,0,0,0,0,0]]C2930498	n hydroxy n 1 4 phenylmethoxy phenyl ethyl acetamide	[[0,1,0,0,0,0,0,0,0,0,0]]C0077147	trimazosin,2 hydroxy 2 methylpropyl 4 4 amino 6 7 8 trimethoxy 2 quinqzolinyl 1 piperazinecar boxylate,2 hydroxy 2 methylpropyl ester 4 4 amino 6 7 8 trimethoxy 2 quinazolinyl 1 piperazinecarboxylic acid	[[0,1,0,0,0,0,0,0,0,1,0],[0,1,0,0,0,0,0,0,0,0,0],[0,1,0,0,0,0,0,0,0,0,0]]C0077144	trilostane,4alpha 5 epoxy 17beta hydroxy 3 oxoandrostane 2 carbonitrile,4alpha 5alpha 17beta 4 5 epoxy 3 17 dihydroxyandrost 2 ene 2 carbonitrile,4 5 epoxy 17 hydroxy 3 oxoandrostane 2 carbonitrile,2alpha cyano 4alpha 5alpha epoxyandrostan 17beta ol 3 one	[[0,0,0,0,0,0,0,1,0,1,0],[0,0,0,0,0,0,0,0,0,0,1],[0,0,0,0,1,0,0,0,0,0,0],[0,0,0,0,1,0,0,0,0,0,0],[0,0,0,0,1,0,0,0,0,0,0]]C3255605	nonoxynol 20	[[0,0,1,0,0,0,0,0,0,0,0]]C0355824	penicillinase resistant penicillin	[[1,0,0,0,0,0,0,0,0,0,1]]C0077149	trimelamol,1 3 5 triazine 2 4 6 triyltris methylimino tris methanol,n 2 n 4 n 6 trihydroxymethyl n 2 n 4 n 6 trimethylmelamine,s triazine 2 4 6 triyl trimethyltrinitrilo tri methanol,trimethyloltrimethylmelamine	[[0,1,0,0,0,0,0,0,0,1,0],[0,1,0,0,0,0,0,0,0,0,0],[0,1,0,0,0,0,0,0,0,0,0],[0,0,0,0,0,0,0,0,0,1,0],[0,0,0,0,0,0,0,0,0,1,0]]C0111145	cs 370	[[0,1,0,0,0,0,0,0,0,0,0]]C0111144	cs 359	[[0,1,0,0,0,0,0,0,0,0,0]]C0111147	cs 500	[[0,1,0,0,0,0,0,0,0,0,0]]C0676311	2 4 o methoxyphenyl piperazin 1 yl methyl 1 3 dioxoperhydroimidazo 1 5 a pyridine	[[0,1,0,0,0,0,0,0,0,0,0]]C3255607	pseudopterogorgia elisabethae extract,pseudopterogorgia elisabethae	[[0,0,0,0,0,1,0,0,0,0,0],[0,0,1,0,0,0,0,0,0,0,0]]C0036050	sagatal	[[0,1,0,0,0,0,0,0,0,0,0]]C0022222	isopilocarpine	[[0,1,0,0,0,0,0,0,0,0,0]]C0081813	bis trifluoroethyl carbamodithioic acid,btca	[[0,1,0,0,0,0,0,0,0,0,0],[0,1,0,0,0,0,0,0,0,0,0]]C0081817	bismuth nitrate	[[0,0,0,0,0,0,1,0,0,0,0]]C0081816	bismuth aluminate,aluminum bismuth oxide	[[1,0,0,0,0,0,0,1,0,1,0],[0,0,0,0,0,0,0,0,0,1,0]]C3255603	nonoxynol 10	[[0,0,1,0,0,0,0,0,0,0,0]]C0067363	n n diethylpseudoisocyanine	[[0,1,0,0,0,0,0,0,0,0,0]]C0918267	liquamar	[[0,1,0,0,0,0,0,0,0,0,0]]C0918266	falithrom,hexal brand of phenprocoumon	[[0,1,0,0,0,0,0,0,0,0,0],[0,1,0,0,0,0,0,0,0,0,0]]C0918265	u 15167,u15167	[[0,1,0,0,0,0,0,0,0,0,0],[0,1,0,0,0,0,0,0,0,0,0]]C0918264	ia 887	[[0,1,0,0,0,0,0,0,0,0,0]]C1364748	diethylamine salt of chromocarb	[[1,0,0,0,0,0,0,0,0,0,0]]C0067369	n n dimethyl 1 methyldodecylamine oxide,1 methyldodecyl dimethylamine n oxide,1 methyldodecyldimethylamine oxide,2 atdno,n oxide n n dimethyl 2 tridecanamine,mddmao,n 1 methyldodecyl n n dimethylamine oxide	[[0,1,0,0,0,0,0,0,0,0,0],[0,1,0,0,0,0,0,0,0,0,0],[0,1,0,0,0,0,0,0,0,0,0],[0,1,0,0,0,0,0,0,0,0,0],[0,1,0,0,0,0,0,0,0,0,0],[0,1,0,0,0,0,0,0,0,0,0],[0,1,0,0,0,0,0,0,0,0,0]]C0067368	n n dimethyl 1 dodecanamine,n n dimethyldodecanamine,nndd,dodecyldimethylamine	[[0,1,0,0,0,0,0,0,0,0,0],[0,1,0,0,0,0,0,0,0,0,0],[0,1,0,0,0,0,0,0,0,0,0],[0,1,0,0,0,0,0,0,0,0,0]]C0615195	ethyl n succinimidoxyacetate,ethyl ester 2 5 dioxo 1 pyrrolidinyl oxy acetic acid	[[0,1,0,0,0,0,0,0,0,0,0],[0,1,0,0,0,0,0,0,0,0,0]]C1741282	artocarpanone	[[0,1,0,0,0,0,0,0,0,0,0]]C0384115	n 4 amino 1 butyl n nitrosobenzamide,n abno benzamide	[[0,1,0,0,0,0,0,0,0,0,0],[0,1,0,0,0,0,0,0,0,0,0]]C3255744	chamaemelum nobile extract,chamaemelum nobile	[[0,0,0,0,0,1,0,0,0,0,0],[0,0,1,0,0,0,0,0,0,0,0]]C1564538	betahistin al,aliud brand of betahistine mesylate	[[0,1,0,0,0,0,0,0,0,0,0],[0,1,0,0,0,0,0,0,0,0,0]]C0098566	5 methylfurtrethonium iodide,5 methylfurfuryltrimethylammonium iodide	[[0,1,0,0,0,0,0,0,0,0,0],[0,1,0,0,0,0,0,0,0,0,0]]C0211935	bk223 b	[[0,1,0,0,0,0,0,0,0,0,0]]C0211934	bk223 a	[[0,1,0,0,0,0,0,0,0,0,0]]C0211937	ng 011	[[0,1,0,0,0,0,0,0,0,0,0]]C0211936	ng 012	[[0,1,0,0,0,0,0,0,0,0,0]]C0535906	astressin,cyclo 30 33 phe 12 nle 21 38 glu 30 lys 33 r hcrf 12 41	[[0,1,0,0,0,0,0,0,0,0,0],[0,1,0,0,0,0,0,0,0,0,0]]C2740268	lotensin hct 10 12 5	[[0,0,0,0,0,0,0,1,0,0,0]]C2935380	gg 50b compound	[[0,1,0,0,0,0,0,0,0,0,0]]C0535909	sr 142948a	[[0,1,0,0,0,0,0,0,0,0,0]]C0535908	2 5 2 6 dimethoxyphenyl 1 4 n 3 dimethylaminopropyl n methylcarbamoyl 2 isopropylphenyl 1h pyrazole 3 carbonyl amino adamantane 2 carboxylic acid	[[0,1,0,0,0,0,0,0,0,0,0]]C0211939	eurystatin a,6 methyl n 7 methyl 3 2 methylpropyl 2 5 6 9 tetraoxo 1 4 8 triazacyclotridec 10 yl 2 heptenamide	[[0,1,0,0,0,0,0,0,0,0,0],[0,1,0,0,0,0,0,0,0,0,0]]C0211938	bu 4164e a	[[0,1,0,0,0,0,0,0,0,0,0]]C0671096	lipo as 013,as 013	[[0,1,0,0,0,0,0,0,0,0,0],[0,1,0,0,0,0,1,0,0,0,0]]C0671097	butyryl prostaglandin f1 butyl ester	[[0,1,0,0,0,0,0,0,0,0,0]]C1120764	24 s 25 epoxycholesterol	[[0,1,0,0,0,0,0,0,0,0,0]]C1120761	nsc 707545,nsc707545	[[0,1,0,0,0,0,0,0,0,0,0],[0,1,0,0,0,0,0,0,0,0,0]]C1120762	platinum 109,pt109	[[0,1,0,0,0,0,0,0,0,0,0],[0,1,0,0,0,0,0,0,0,0,0]]C1120763	platinum 103,pt103	[[0,1,0,0,0,0,0,0,0,0,0],[0,1,0,0,0,0,0,0,0,0,0]]C1831916	aczone gel	[[0,0,0,0,1,0,0,0,0,0,0]]C0146010	tixocortol	[[0,0,0,0,0,0,0,1,0,0,0]]C0951208	pyrrolidylmethyl n methylcyclopentylphenyl glycolate monohydrochoride salt	[[0,1,0,0,0,0,0,0,0,0,0]]C1831910	gp100 280 288 288v peptide vaccine	[[0,0,0,0,1,0,0,0,0,1,0]]C0731391	sno pilo	[[0,0,0,0,0,0,0,1,0,0,0]]C0731392	sno tears	[[0,0,0,0,0,0,0,1,0,0,0]]C2718635	acuvail	[[0,0,0,0,0,0,0,1,0,0,0]]C0641194	alpha chlorohydrin 1 phosphate	[[0,1,0,0,0,0,0,0,0,0,0]]C3252051	gamma benzyl l glutamyl n carboxy anhydride	[[0,1,0,0,0,0,0,0,0,0,0]]C3255746	chamaemelum nobile flower oil	[[0,0,1,0,0,0,0,0,0,0,0]]C2699768	dietifen	[[0,0,0,0,0,0,0,0,0,1,0]]C1981388	amylase 124 synovial fluid	[[0,0,0,1,0,0,0,0,0,0,0]]C2699762	acridorex	[[0,0,0,0,0,0,0,0,0,1,0]]C0246760	human nt 4	[[0,1,0,0,0,0,0,0,0,0,0]]C2699760	eklira	[[0,0,0,0,0,0,0,0,0,1,0]]C2699761	acoxatrine	[[0,0,0,0,0,0,0,0,0,1,0]]C2699766	diethadione	[[0,0,0,0,0,0,0,0,0,1,0]]C2699767	diethazine hydrochloride,antipar,10 2 diethylamino ethylphenothiazine hydrochloride,lodibon,aparkazin	[[0,0,0,0,0,0,0,0,0,1,0],[0,0,0,0,0,0,0,0,0,1,0],[0,0,0,0,0,0,0,0,0,1,0],[0,0,0,0,0,0,0,0,0,1,0],[0,0,0,0,0,0,0,0,0,1,0]]C0246761	rat nt 4	[[0,1,0,0,0,0,0,0,0,0,0]]C0116499	erythro 9 2 hydroxy 3 nonyl 3 deazaadenine	[[0,1,0,0,0,0,0,0,0,0,0]]C0076235	tetrachloronitrobenzene,tcnb	[[0,1,0,0,0,0,0,0,0,0,0],[0,1,0,0,0,0,0,0,0,0,0]]C0076239	tetrachloroplatinate	[[0,1,0,0,0,0,0,0,0,0,0]]C1100072	3 hydroxymethyl 3 4 di o camphanoylkhellactone,3 hydroxymethyl dck	[[0,1,0,0,0,0,0,0,0,0,0],[0,1,0,0,0,0,0,0,0,0,0]]C1100073	1 3 4 methylenedioxyphenyl sulfonylphenyl 1 1 1 n propylsulfonyl morpholin 4 yl morpholin 4 yl dioxole,dioxolecpd1	[[0,1,0,0,0,0,0,0,0,0,0],[0,1,0,0,0,0,0,0,0,0,0]]C0048358	4 hydroxymandelic acid,alpha 4 dihydroxybenzeneacetic acid,para hydroxymandelic acid,pisolithin b	[[0,1,0,0,0,0,0,0,0,0,0],[0,1,0,0,0,0,0,0,0,0,0],[0,1,0,0,0,0,0,0,0,0,0],[0,1,0,0,0,0,0,0,0,0,0]]C1100074	1 4 methoxyphenyl sulfonylphenyl 1 1 1 1 naphthylcarbonyl morpholin 4 yl morpholin 4 yl dioxole,dioxolecpd2	[[0,1,0,0,0,0,0,0,0,0,0],[0,1,0,0,0,0,0,0,0,0,0]]C1100075	1 4 methoxyphenyl sulfonylphenyl 1 1 1 4 fluoro 1 naphthylcarbonyl morpholin 4 yl morpholin 4 yl dioxole,dioxolecpd3	[[0,1,0,0,0,0,0,0,0,0,0],[0,1,0,0,0,0,0,0,0,0,0]]C0140800	ro 8 0576,ro 8 0576 12,ro 8 0576 7	[[0,1,0,0,0,0,0,0,0,0,0],[0,1,0,0,0,0,0,0,0,0,0],[0,1,0,0,0,0,0,0,0,0,0]]C0140801	ro 8 4650	[[0,1,0,0,0,0,0,0,0,0,0]]C0140807	ro 59129 001	[[0,1,0,0,0,0,0,0,0,0,0]]C0140805	ro 5 4864,ro 05 4864,ro5 4864	[[0,1,0,0,0,0,0,0,0,0,0],[0,1,0,0,0,0,0,0,0,0,0],[0,1,0,0,0,0,0,0,0,0,0]]C0208518	op 41483 alpha cd	[[0,1,0,0,0,0,0,0,0,0,0]]C1101301	5 valproylamido 1 3 4 thiadiazole 2 sulfonamide,2002uglycpd13	[[0,1,0,0,0,0,0,0,0,0,0],[0,1,0,0,0,0,0,0,0,0,0]]C0952258	gt 161 methyl sulfate	[[0,1,0,0,0,0,0,0,0,0,0]]C0952259	gt 161 iodide	[[0,1,0,0,0,0,0,0,0,0,0]]C0049408	6 6 ethylenebis oxyethyleneimino bis 17 cyclopropylmethyl 4 5 epoxymorphinan 3 14 diol,tena	[[0,1,0,0,0,0,0,0,0,0,0],[0,1,0,0,0,0,0,0,0,0,0]]C1101304	2 methyl 8 quinolyl 8 chloro 5 5 dioxoimidazo 1 2 b 1 4 2 benzodithiazine 7 carboxylate,2 methyl 8 quinolyl cdbdc	[[0,1,0,0,0,0,0,0,0,0,0],[0,1,0,0,0,0,0,0,0,0,0]]C0600842	1 4 chlorophenyl 1 phenyl 2 propynylcarbamate	[[0,1,0,0,0,0,0,0,0,0,0]]C0208513	spartanamicin a	[[0,1,0,0,0,0,0,0,0,0,0]]C0208512	antibiotic 80334 b,antibiotic sipi 80334 b	[[0,1,0,0,0,0,0,0,0,0,0],[0,1,0,0,0,0,0,0,0,0,0]]C0600847	chainin,27 lactone 2 n butyl 16 methyl 3 5 7 9 11 13 15 26 27 nonahydroxyoctacosa 16 18 20 22 24 pentaenoic acid	[[0,1,0,0,0,0,0,0,0,0,0],[0,1,0,0,0,0,0,0,0,0,0]]C0956421	disodium salt acid black s	[[0,1,0,0,0,0,0,0,0,0,0]]C1981386	amylase 124 peritoneal fluid	[[0,0,0,1,0,0,0,0,0,0,0]]C0633577	mono aldehyde gossypol	[[0,1,0,0,0,0,0,0,0,0,0]]C0968383	sp600125	[[0,1,0,0,0,0,0,0,0,0,0]]C0093904	23 24 dinor 5 cholene 3 beta 20 diol	[[0,1,0,0,0,0,0,0,0,0,0]]C0633570	methyl 2 2 hydroxyphenyl 2 thiazoline 4 carboxylate,mhptca	[[0,1,0,0,0,0,0,0,0,0,0],[0,1,0,0,0,0,0,0,0,0,0]]C0633572	2 hydroxyphenyl imido ethylether,hpiee	[[0,1,0,0,0,0,0,0,0,0,0],[0,1,0,0,0,0,0,0,0,0,0]]C0383463	tyrosyl d alanyl glycyl methylphenylalanyl n propylglycinamide,tyr d ala gly mephe gly nhc3h7	[[0,1,0,0,0,0,0,0,0,0,0],[0,1,0,0,0,0,0,0,0,0,0]]C0383461	cdri 82 205	[[0,1,0,0,0,0,0,0,0,0,0]]C1565486	isis 113715	[[0,1,0,0,0,0,0,0,0,0,0]]C0663680	125i iph	[[0,1,0,0,0,0,0,0,0,0,0]]C3194745	veneno de abeja aceite de vibora	[[0,0,0,0,0,0,0,1,0,0,0]]C0168348	sk f 96356,skf 96356	[[0,1,0,0,0,0,0,0,0,0,0],[0,1,0,0,0,0,0,0,0,0,0]]C0721548	mallotuss	[[0,0,0,0,0,0,0,1,0,0,0]]C0721549	maltsupex	[[0,0,0,0,0,0,1,1,0,0,0]]C0113786	dienochlor,decachlorobi 2 4 cyclopentadien 1 yl	[[0,1,0,0,0,0,0,0,0,0,0],[0,1,0,0,0,0,0,0,0,0,0]]C0614822	17 beta bromoacetylamino 4 androsten 3 one,2 bromo n 17beta 3 oxoandrost 4 en 17 yl acetamide,17 baa ato	[[0,1,0,0,0,0,0,0,0,0,0],[0,1,0,0,0,0,0,0,0,0,0],[0,1,0,0,0,0,0,0,0,0,0]]C1445539	ulmus americana specific extract	[[1,0,0,0,0,0,0,0,0,0,0]]C1445538	tilia cordata specific extract	[[1,0,0,0,0,0,0,0,0,0,0]]C0721540	malatal	[[0,0,0,0,0,0,0,1,0,0,0]]C1445536	sambucus nigra specific extract	[[1,0,0,0,0,0,0,0,0,0,0]]C1445535	salix caprea specific extract	[[1,0,0,0,0,0,0,0,0,0,0]]C0721543	maldroxal,maldroksal	[[0,1,0,0,0,0,0,0,0,0,0],[0,1,0,0,0,0,0,0,0,0,0]]C0721544	maldroxal plus	[[0,0,0,0,0,0,0,1,0,0,0]]C0721545	mallazine	[[0,0,0,0,0,0,0,1,0,0,0]]C1445531	populus deltoides specific extract	[[1,0,0,0,0,0,0,0,0,0,0]]C1445530	platanus acerifolia specific extract	[[1,0,0,0,0,0,0,0,0,0,0]]C1704757	therapeutic vasoactive intestinal peptide,vasoactive intestinal polypeptide,therapeutic vasoactive intestinal polypeptide,therapeutic vip	[[0,0,0,0,0,0,0,0,0,1,0],[0,0,0,0,0,0,0,0,0,1,0],[0,0,0,0,0,0,0,0,0,1,0],[0,0,0,0,0,0,0,0,0,1,0]]C1452282	1 4 bis 3 aminopropyl piperazine	[[0,1,0,0,0,0,0,0,0,0,0]]C0146896	triprolyl angiotensin ii,pro 3 aii,n 1 1 prolyl prolyl prolyl isoleucine 5 angiotensin ii,pro pro pro angiotensin ii,proline prolyl prolyl angiotensin ii,pro pro pro angiotensinii	[[0,1,0,0,0,0,0,0,0,0,0],[0,1,0,0,0,0,0,0,0,0,0],[0,1,0,0,0,0,0,0,0,0,0],[0,1,0,0,0,0,0,0,0,0,0],[0,1,0,0,0,0,0,0,0,0,0],[0,1,0,0,0,0,0,0,0,0,0]]C0063335	iem 476	[[0,1,0,0,0,0,0,0,0,0,0]]C1973154	selenium 124 tissue smears	[[0,0,0,1,0,0,0,0,0,0,0]]C0355525	morphine hydrochloride powder	[[1,0,0,0,0,0,0,0,0,0,0]]C1563598	barium 1 2 salt fructose 1 6 diphosphate	[[0,1,0,0,0,0,0,0,0,0,0]]C1563599	calcium 1 2 salt fructose 1 6 diphosphate	[[0,1,0,0,0,0,0,0,0,0,0]]C0731318	angiozem cr	[[0,0,0,0,0,0,0,1,0,0,0]]C0731319	anhydrol forte	[[0,0,0,0,0,0,0,1,0,0,0]]C0256868	al 0670,al0670	[[0,1,0,0,0,0,0,0,0,0,0],[0,1,0,0,0,0,0,0,0,0,0]]C0731314	anethaine	[[0,0,0,0,0,0,0,1,0,0,0]]C0256867	n 6 amino 3 pyridyl n bicyclo 2 2 1 hept 2 yl n cyanoguanidine	[[0,1,0,0,0,0,0,0,0,0,0]]C0027302	nadolol,5 3 1 1 dimethylethyl amino 2 hydroxypropoxy 1 2 3 4 tetrahydro 2 3 naphthalenediol	[[0,0,0,0,0,0,0,0,1,1,0],[0,0,0,0,0,0,0,0,0,0,1]]C0731311	anabact	[[0,0,0,0,0,0,0,1,0,0,0]]C0256860	kuscide,2 hydroxyethoxyethyl chloroacetate,monochloroacetate diethylene glycol	[[0,1,0,0,0,0,0,0,0,0,0],[0,1,0,0,0,0,0,0,0,0,0],[0,1,0,0,0,0,0,0,0,0,0]]C0256861	cyclopeptolide 1	[[0,1,0,0,0,0,0,0,0,0,0]]C1452317	calcipot,3m brand of calcium gluconate monohydrate	[[0,1,0,0,0,0,0,0,0,0,0],[0,1,0,0,0,0,0,0,0,0,0]]C0970032	isomer dh 990	[[0,1,0,0,0,0,0,0,0,0,0]]C0970033	alborixin monopotassium salt	[[0,1,0,0,0,0,0,0,0,0,0]]C0970035	ceftezole sodium,sodium salt ceftezol	[[0,1,0,0,0,0,0,0,0,0,0],[0,1,0,0,0,0,0,0,0,0,0]]C2825838	anti hgf monoclonal antibody tak 701,anti hgf moab tak 701	[[0,0,0,0,0,0,0,0,0,1,0],[0,0,0,0,0,0,0,0,0,1,0]]C0970037	2 7 fluorenediamine monohydrochloride	[[0,1,0,0,0,0,0,0,0,0,0]]C2946205	aspercreme max	[[0,0,0,0,0,0,0,1,0,0,0]]C1972460	ranitidine 124 xxx	[[0,0,0,1,0,0,0,0,0,0,0]]C0602632	gr 2 2132,6 pregnadien 20 one 17 acetate 3 beta benzoate 6 11 beta dichloro 3 beta 17 dihydroxy 19 nor 4	[[0,1,0,0,0,0,0,0,0,0,0],[0,1,0,0,0,0,0,0,0,0,0]]C0290594	pherofunginum	[[0,1,0,0,0,0,0,0,0,0,0]]C0290590	1 amino 3 phenylpropylphosphonic acid,1 nh2 3 phpp	[[0,1,0,0,0,0,0,0,0,0,0],[0,1,0,0,0,0,0,0,0,0,0]]C1258293	3 bromophenyl 6 acetoxymethyl 2 oxo 2h 1 benzopyran 3 carboxylate,3 brpa o bpca	[[0,1,0,0,0,0,0,0,0,0,0],[0,1,0,0,0,0,0,0,0,0,0]]C2364789	bss ophthalmic solution	[[0,0,0,0,0,0,0,1,0,0,0]]C0071208	plasma lyte 148,plasma lyte	[[0,0,0,0,0,0,0,1,0,0,0],[0,0,0,0,0,0,1,0,0,0,0]]C0253333	4 amino 1 8 naphthalimide,4 amino1 8 naphthalimide	[[0,1,0,0,0,0,0,0,0,0,0],[0,1,0,0,0,0,0,0,0,0,0]]C0253335	n cis 2 6 dimethylpiperidinocarbonyl gamma methylleucyl tryptophyl coome norleucine	[[0,1,0,0,0,0,0,0,0,0,0]]C0253334	n 2 6 dimethylpiperidinocarbonyl gamma meleu trp coome nle ona	[[0,1,0,0,0,0,0,0,0,0,0]]C0253336	bq 788	[[0,1,0,0,0,0,0,0,0,0,0]]C0632225	agelasine b	[[0,1,0,0,0,0,0,0,0,0,0]]C0632220	viridomycin a	[[0,1,0,0,0,0,0,0,0,0,0]]C0632221	n methylindisocin,3 3 acetyloxy 2 3 dihydro 1 methyl 2 oxo 1h indol 3 yl 2 chloro 2 propenenitrile	[[0,1,0,0,0,0,0,0,0,0,0],[0,1,0,0,0,0,0,0,0,0,0]]C0539349	ns 7	[[0,1,0,0,0,0,0,0,0,0,0]]C0539348	4 4 fluorophenyl 2 methyl 6 5 piperidinopentyloxy pyrimidine hydrochloride	[[0,1,0,0,0,0,0,0,0,0,0]]C2934804	n n didesmethylgrossularine 1,ddmg 1 cpd	[[0,1,0,0,0,0,0,0,0,0,0],[0,1,0,0,0,0,0,0,0,0,0]]C0606445	agronex	[[0,1,0,0,0,0,0,0,0,0,0]]C2934802	lyngbyastatin 9	[[0,1,0,0,0,0,0,0,0,0,0]]C2934800	6 bromoicosa 3 5 8 13 15 pentaene 11 19 diynoic acid,6 bromo ipda	[[0,1,0,0,0,0,0,0,0,0,0],[0,1,0,0,0,0,0,0,0,0,0]]C0539341	6 carboxytetramethylrhodamine,6 ctmr,6 carboxy tetramethylrhodamine	[[0,1,0,0,0,0,0,0,0,0,0],[0,1,0,0,0,0,0,0,0,0,0],[0,1,0,0,0,0,0,0,0,0,0]]C1452314	flopak plain,merial brand of calcium gluconate	[[0,1,0,0,0,0,0,0,0,0,0],[0,1,0,0,0,0,0,0,0,0,0]]C1735433	baltussin	[[0,0,0,0,0,0,0,1,0,0,0]]C2965034	desflurane 124 inhaled gas	[[0,0,0,1,0,0,0,0,0,0,0]]C0700899	benadryl	[[0,0,0,0,0,0,0,1,0,0,0]]C0700898	dimedrol	[[0,1,0,0,0,0,0,0,0,0,0]]C0700897	rmi 9918,rmi9918	[[0,1,0,0,0,0,0,0,0,0,0],[0,1,0,0,0,0,0,0,0,0,0]]C0700894	liquaemin	[[0,1,0,0,0,0,0,0,0,0,0]]C0700893	lipo hepin	[[0,0,0,0,0,0,0,0,0,1,0]]C0700892	hep lock	[[0,0,0,0,1,0,1,0,0,0,0]]C0700891	amo vitrax,inc brand of sodium hyaluronate advanced medical optics	[[0,1,0,0,0,0,0,0,0,0,0],[0,1,0,0,0,0,0,0,0,0,0]]C0700890	etamucine	[[0,1,0,0,0,0,0,0,0,0,0]]C0702002	mithracin	[[0,0,0,0,0,0,0,0,0,1,0]]C0702003	a 2371	[[0,0,0,0,0,0,0,0,1,0,0]]C3255816	acai oil	[[0,0,1,0,0,0,0,0,0,0,0]]C0111923	cytotoxin c	[[0,1,0,0,0,0,0,0,0,0,0]]C0702007	lysodren,bristol myers squibb brand of mitotane	[[0,0,0,0,0,0,1,1,0,0,0],[0,1,0,0,0,0,0,0,0,0,0]]C0702004	chlodithane	[[0,0,0,0,0,0,0,0,0,1,0]]C3255811	sorbitan isostearate	[[0,0,1,0,0,0,0,0,0,0,0]]C0702008	levonor	[[0,1,0,0,0,0,0,0,0,0,0]]C3180526	cdp 7851,cdp7851	[[0,1,0,0,0,0,0,0,0,0,0],[0,1,0,0,0,0,0,0,0,0,0]]C0669403	ccf 2,ccf2 compound	[[0,1,0,0,0,0,0,0,0,0,0],[0,1,0,0,0,0,0,0,0,0,0]]C1602154	bumetadine	[[0,0,0,0,0,0,0,1,0,0,0]]C3180522	triacetyl 3 4 5 6 tetrahydrouridine,triacetyl thu,tathu cpd	[[0,1,0,0,0,0,0,0,0,0,0],[0,1,0,0,0,0,0,0,0,0,0],[0,1,0,0,0,0,0,0,0,0,0]]C3180521	3 1h indol 2 yl phenyl 1h indol 2 yl methanone,ipi methanone	[[0,1,0,0,0,0,0,0,0,0,0],[0,1,0,0,0,0,0,0,0,0,0]]C0045235	2 2 2 terpyridine,2 2 2 tripyridine	[[0,1,0,0,0,0,0,0,0,0,0],[0,1,0,0,0,0,0,0,0,0,0]]C0148493	visotrast 370	[[0,1,0,0,0,0,0,0,0,0,0]]C0148492	visotrast	[[0,1,0,0,0,0,0,0,0,0,0]]C0057846	dichlorobenzyl alcohol,dichlorobenzenemethanol	[[0,0,0,0,0,0,0,1,0,1,0],[0,0,0,0,0,0,0,0,0,0,1]]C0125627	leukogel	[[0,1,0,0,0,0,0,0,0,0,0]]C2984210	iodine i 131 anti fibronectin antibody fragment l19 sip,l19 sip	[[0,0,0,0,0,0,0,0,0,1,0],[0,0,0,0,0,0,0,0,0,1,0]]C0096420	4 1 oxidothiomorpholino 8 phenethylthio s piperazinylpyrimido 5 4 d pyrimidine	[[0,1,0,0,0,0,0,0,0,0,0]]C0096426	4 2 3 bis bis carboxymethylamino propyl phenyl isothiocyanate,s n n 1 4 isothiocyanatophenyl methyl 1 2 ethanediyl bis n carboxymethyl glycine	[[0,1,0,0,0,0,0,0,0,0,0],[0,1,0,0,0,0,0,0,0,0,0]]C0096429	4 2 6 dihydroxybenzoyl 3 formyl 5 hydroxybenzoic acid,4 2 6 dihydroxybenzoyl 3 formyl 5 hydroxy benzoic acid	[[0,1,0,0,0,0,0,0,0,0,0],[0,1,0,0,0,0,0,0,0,0,0]]C3255818	methyl and propyl parabens,parabens	[[0,0,1,0,0,0,0,0,0,0,0],[0,0,1,0,0,0,0,0,0,0,0]]C3164837	metformin hydrochloride saxagliptin	[[1,0,0,0,0,0,0,0,0,0,0]]C0010146	corynanthine tartrate	[[0,1,0,0,0,0,0,0,0,0,0]]C0939410	novo metoprol	[[0,0,0,0,0,0,1,0,0,0,0]]C0939412	novolog,novo nordisk brand of insulin aspart,novorapid	[[0,0,0,0,0,0,1,1,0,1,0],[0,1,0,0,0,0,0,0,0,0,0],[0,1,0,0,0,0,1,0,0,0,0]]C0939415	nutrinate	[[0,0,0,0,0,0,1,0,0,0,0]]C0939414	nulev	[[0,0,0,0,0,0,1,1,0,0,0]]C0939416	olux	[[0,0,0,0,0,1,1,1,0,0,0]]C0939418	omnicef omni pac	[[0,0,0,0,0,0,0,1,0,0,0]]C1992248	mefenamate 124 bld ser plas	[[0,0,0,1,0,0,0,0,0,0,0]]C0285683	3ntpt	[[0,1,0,0,0,0,0,0,0,0,0]]C0285681	ly 281067,ly281067	[[0,1,0,0,0,0,0,0,0,0,0],[0,1,0,0,0,0,0,0,0,0,0]]C0285686	8 chloro 2 3 dihydrospiro pyrrolidine 3 6 5 h pyrrolo 1 2 3 de 1 4 benzoxazine 2 5 5 trione	[[0,1,0,0,0,0,0,0,0,0,0]]C0285684	polyerga	[[0,0,0,0,0,0,1,0,0,0,0]]C0285685	polyerga neu	[[0,1,0,0,0,0,0,0,0,0,0]]C0648873	n 4 azidosalicyl aminoethanol 1 1 phospho d myo inositol 3 4 5 triphosphate,asains 1 3 4 5 p4,asainsp4	[[0,1,0,0,0,0,0,0,0,0,0],[0,1,0,0,0,0,0,0,0,0,0],[0,1,0,0,0,0,0,0,0,0,0]]C0028098	ninhydrin,indantrione monohydrate,2 2 dihydroxy 1h indene 1 3 2h dione	[[0,0,0,0,0,0,0,0,0,0,1],[0,1,0,0,0,0,0,0,0,0,1],[0,0,0,0,0,0,0,0,0,0,1]]C0955799	fumarate sgb 483	[[0,1,0,0,0,0,0,0,0,0,0]]C2825488	omtriptolide	[[0,0,0,0,0,0,0,0,0,1,0]]C1172785	endophenazine c	[[0,1,0,0,0,0,0,0,0,0,0]]C0639756	aloperine	[[0,1,0,0,0,0,0,0,0,0,0]]C2965031	desflurane 124 airway	[[0,0,0,1,0,0,0,0,0,0,0]]C0954189	dl isomer 4 oxalysine	[[0,1,0,0,0,0,0,0,0,0,0]]C2946750	musclecare	[[0,0,0,0,0,0,0,1,0,0,0]]C0913329	2 mmbi	[[0,1,0,0,0,0,0,0,0,0,0]]C0128403	mhfp avt	[[0,1,0,0,0,0,0,0,0,0,0]]C0108984	cellolite	[[0,1,0,0,0,0,0,0,0,0,0]]C0638704	2 2 3 azabicyclo 3 2 2 non 3 yl ethyl 5 6 dimethoxy 1 2 benzisothiazol 3 2h one	[[0,1,0,0,0,0,0,0,0,0,0]]C1968084	zotex 12d	[[0,0,0,0,0,0,0,1,0,0,0]]C1588624	pre sed	[[0,0,0,0,0,0,0,1,0,0,0]]C1968081	visqid aa	[[0,0,0,0,0,0,0,1,0,0,0]]C1172783	endophenazine a	[[0,1,0,0,0,0,0,0,0,0,0]]C0046768	3 5 dimethylacetaminophen,3 5 dimethyl 4 hydroxyacetanilide,3 5 dimethylparacetamol	[[0,1,0,0,0,0,0,0,0,0,0],[0,1,0,0,0,0,0,0,0,0,0],[0,1,0,0,0,0,0,0,0,0,0]]C0046769	3 5 dioctanoyl 5 fluoro 2 deoxyuridine,fdurd c8,5 fluoro 2 deoxy o o dioctanoyluridine	[[0,1,0,0,0,0,0,0,0,0,0],[0,1,0,0,0,0,0,0,0,0,0],[0,1,0,0,0,0,0,0,0,0,0]]C2965032	desflurane 124 anesthesia gas machine	[[0,0,0,1,0,0,0,0,0,0,0]]C0957375	e iosmer methyl 3 methoxy 4 hydroxystyryl ketone	[[0,1,0,0,0,0,0,0,0,0,0]]C0046764	3 5 dichloromethotrexate,dichloromethotrexate,dichloroamethopterin,dcm,n 3 5 dichloro 4 2 4 diamino 6 pteridinyl methyl methylamino benzoyl glutamic acid,n 3 5 dichloro 4 2 4 diamino 6 pteridinyl methyl methylamino benzoyl l 8ci glutamic acid,n 3 5 dichloro 4 2 4 diamino 6 pteridinyl methyl methylamino benzoyl 9ci l glutamic acid,n 3 5 dichloro 4 2 4 diamino 6 pteridinylmethyl methylamino benzoyl glutamic acid,3 5 dichloro amethopterin,3 5 dichloroamethopterin,dichloro methotrexate	[[0,1,0,0,1,0,0,0,0,1,0],[0,0,0,0,1,0,0,0,0,1,0],[0,0,0,0,1,0,0,0,0,1,0],[0,0,0,0,1,0,0,0,0,0,0],[1,0,0,0,1,0,0,0,0,1,0],[0,0,0,0,1,0,0,0,0,1,0],[0,0,0,0,1,0,0,0,0,1,0],[0,0,0,0,1,0,0,0,0,1,0],[0,0,0,0,1,0,0,0,0,1,0],[0,0,0,0,1,0,0,0,0,1,0],[0,0,0,0,0,0,0,0,0,1,0]]C3181322	gsk 2324,gsk2324	[[0,1,0,0,0,0,0,0,0,0,0],[0,1,0,0,0,0,0,0,0,0,0]]C0957370	tetraammonium salt 3 azido 3 deoxythymidine 5 triphosphate	[[0,1,0,0,0,0,0,0,0,0,0]]C0028092	nilverm	[[0,1,0,0,0,0,0,0,0,0,0]]C0046761	3 4 dihydroxyflavone,3 4 dihydroxy flavone	[[0,1,0,0,0,0,0,0,0,0,0],[0,1,0,0,0,0,0,0,0,0,0]]C2966079	oxcarbazepine 124 urine	[[0,0,0,1,0,0,0,0,0,0,0]]C0610357	3 oxa 4 5 6 nor 3 7 inter 3 phenyleneprostaglandin e1,3 oxa 4 5 6 nor 3 7 inter 3 phenylene pge1,oi pge1	[[0,1,0,0,0,0,0,0,0,0,0],[0,1,0,0,0,0,0,0,0,0,0],[0,1,0,0,0,0,0,0,0,0,0]]C0610354	traceptin	[[0,1,0,0,0,0,0,0,0,0,0]]C1513757	murine mca ricin conjugate	[[0,0,0,0,0,0,0,0,0,1,0]]C0610350	11 15 bisdeoxyprostaglandin e1,13e 9 oxo prost 13 en 1 oic acid,11 15 bisdeoxy pge1	[[0,1,0,0,0,0,0,0,0,0,0],[0,1,0,0,0,0,0,0,0,0,0],[0,1,0,0,0,0,0,0,0,0,0]]C1302064	atropine sulfate edrophonium chloride,atropine sulphate edrophonium chloride	[[1,0,0,0,0,0,0,0,0,0,0],[1,0,0,0,0,0,0,0,0,0,0]]C0028094	nimodipine,2 methoxyethyl 1 methylethyl ester 1 4 dihydro 2 6 dimethyl 4 3 nitrophenyl 3 5 pyridinedicarboxylic acid,nimo	[[0,0,0,0,0,0,0,1,0,1,0],[0,0,0,0,0,0,0,0,0,0,1],[0,0,0,0,1,0,0,0,0,0,0]]C0813160	breokinase	[[0,0,0,0,0,0,0,0,0,1,0]]C3252025	ns 9283,ns9283	[[0,1,0,0,0,0,0,0,0,0,0],[0,1,0,0,0,0,0,0,0,0,0]]C1302067	diltiazem malate enalapril maleate	[[1,0,0,0,0,0,0,0,0,0,0]]C2966077	oxazepam 124 gastric fluid	[[0,0,0,1,0,0,0,0,0,0,0]]C0021918	intrinsic factor	[[1,1,0,0,0,0,1,1,1,0,1]]C0218058	cui xing ning,5 1 3 3 trimethylindoline n n dimethylcarbamate,monohydrochloride 2 3 dihydro 1 3 3 trimethyl 1h indol 5 yl ester dimethyl carbamic acid,5 1 3 3 trimethylindolinyl n n dimethylcarbamate,carbaindoline	[[0,1,0,0,0,0,0,0,0,0,0],[0,1,0,0,0,0,0,0,0,0,0],[0,1,0,0,0,0,0,0,0,0,0],[0,1,0,0,0,0,0,0,0,0,0],[0,1,0,0,0,0,0,0,0,0,0]]C1588489	sudatex	[[0,0,0,0,0,0,0,1,0,0,0]]C0115127	duoperone fumarate	[[0,1,0,0,0,0,0,0,0,1,0]]C0890529	pentamethylmelamine hydrochloride	[[0,1,0,0,0,0,0,0,0,0,0]]C2346801	daratumumab,anti cd38 monoclonal antibody	[[0,1,0,0,0,0,0,0,0,1,0],[0,0,0,0,0,0,0,0,0,1,0]]C0115128	duovent	[[0,1,0,0,0,0,0,0,0,0,0]]C0634189	et insolium	[[0,1,0,0,0,0,0,0,0,0,0]]C0093135	2 hydroxy n 3 4 dimethyl 5 isoxazolyl 1 4 naphthoquinone imine,2 hdini	[[0,1,0,0,0,0,0,0,0,0,0],[0,1,0,0,0,0,0,0,0,0,0]]C0099193	6 fluoro 7 8 dihydroxy 1 4 hydroxyphenyl 2 3 4 5 tetrahydro 1h 3 benzazepine	[[0,1,0,0,0,0,0,0,0,0,0]]C0898738	hcl of n mercaptoethylpropranolol	[[0,1,0,0,0,0,0,0,0,0,0]]C0103703	ap 159,ap159	[[0,1,0,0,0,0,0,0,0,0,0],[0,1,0,0,0,0,0,0,0,0,0]]C0003422	sodium diphenylhydantoinate	[[0,1,0,0,0,0,0,0,0,0,0]]C1522385	recombinant gonadotropin,gonadotropins,gonadotropic hormone,therapeutic gonadotropin	[[0,0,0,0,0,1,0,0,0,0,0],[0,0,0,0,0,0,0,0,0,1,0],[0,0,0,0,0,0,0,0,0,1,0],[0,0,0,0,0,0,0,0,0,1,0]]C0003420	antipyrine,phenazone,1 2 dihydro 1 5 dimethyl 2 phenyl 3h pyrazol 3 one	[[0,0,0,0,0,0,0,0,1,1,0],[0,0,0,0,0,0,1,0,0,1,0],[0,0,0,0,0,0,0,0,0,0,1]]C0003426	urinary antiseptics	[[0,1,0,0,0,0,1,0,0,0,0]]C1983645	carboxy tetrahydrocannabinol 124 bld ser plas	[[0,0,0,1,0,0,0,0,0,0,0]]C0003429	antisickling agents	[[0,1,0,0,0,0,0,0,1,0,0]]C3257445	rhus glabra bark	[[0,0,1,0,0,0,0,0,0,0,0]]C0138525	progabide acid,monosodium salt 4 4 chlorophenyl 5 fluoro 2 hydroxyphenyl methylene amino butanoic acid	[[0,1,0,0,0,0,0,0,0,0,0],[0,1,0,0,0,0,0,0,0,0,0]]C1588494	protex	[[0,0,0,0,0,0,0,1,0,0,0]]C0087760	1 3 dimethylspiro 1 3 4 5 6 6 7 12b octahydro 2h benzo b furo 2 3 a quinolizine 2 4 pyrimidin 2 one	[[0,1,0,0,0,0,0,0,0,0,0]]C0079810	mecholyl	[[0,0,0,0,0,0,0,1,0,0,0]]C0634813	18 hydroxycamptothecin,s 4 hydroxy 4 2 hydroxyethyl 1h pyrano 3 4 6 7 indolizino 1 2 b quinoline 3 14 4h 12h dione	[[0,1,0,0,0,0,0,0,0,0,0],[0,1,0,0,0,0,0,0,0,0,0]]C1588499	p tuss	[[0,0,0,0,0,0,0,1,0,0,0]]C0634810	1 methyl 5 4 dimethylaminophenylazo indazole,nme5i	[[0,1,0,0,0,0,0,0,0,0,0],[0,1,0,0,0,0,0,0,0,0,0]]C0087769	1 1 3 3 3 3 hexamethylindocarbocyanine iodide	[[0,1,0,0,0,0,0,0,0,0,0]]C0092421	2 amino 1 5 anhydro 2 deoxy d glucitol	[[0,1,0,0,0,0,0,0,0,0,0]]C0914052	ag 1295,ag1295,tyrphostin ag 1295	[[0,1,0,0,0,0,0,0,0,1,0],[0,1,0,0,0,0,0,0,0,0,0],[0,0,0,0,0,0,0,0,0,1,0]]C0614580	5 6 benzocoumarin 3 carboxylic acid ethyl ester,ethyl 5 6 benzocoumarin 3 carboxylate	[[0,1,0,0,0,0,0,0,0,0,0],[0,1,0,0,0,0,0,0,0,0,0]]C2825485	caloxetic acid	[[0,0,0,0,0,0,0,0,0,1,0]]C0592534	certoparin sodium	[[0,1,0,0,0,0,0,1,0,0,0]]C0592536	locoid crelo	[[0,0,0,0,0,0,0,1,0,0,0]]C0531929	hemibastadinol 1	[[0,1,0,0,0,0,0,0,0,0,0]]C0592533	foradil,schering brand of formoterol fumarate	[[0,0,0,0,0,0,1,0,0,1,0],[0,1,0,0,0,0,0,0,0,0,0]]C0592532	certoparin	[[1,0,0,0,0,0,0,1,0,0,0]]C2723639	dogfennel pollen extract	[[0,0,0,0,0,0,0,1,0,0,0]]C0531921	arg 1 trp 5 7 9 leu 11 substance p,arginyl 1 tryptophyl 5 7 9 leucyl 11 substance p,r3wl substance p	[[0,1,0,0,0,0,0,0,0,0,0],[0,1,0,0,0,0,0,0,0,0,0],[0,1,0,0,0,0,0,0,0,0,0]]C0957995	isomer d 595	[[0,1,0,0,0,0,0,0,0,0,0]]C0957996	5z 13e 15s isomer mr 356	[[0,1,0,0,0,0,0,0,0,0,0]]C0957997	dimethyldiazaperopyrenium dichloride	[[0,1,0,0,0,0,0,0,0,0,0]]C3257275	niacin pantothenic acid riboflavin thiamine vitamin b 12 vitamin b6	[[0,0,0,0,0,0,0,1,0,0,0]]C1312048	mixt with 2s 2alpha 5alpha 6beta 2 2 dimethyl 1 oxopropoxy 2s 2alpha 5alpha 6beta s 2 2 dimethyl 1 oxopropoxy methyl ester 6 aminophenylacetyl amino 3 3 dimethyl 7 oxo 4 thia 1 azabicyclo 3 2 0 heptane 2 carboxylic acid	[[0,1,0,0,0,0,0,0,0,0,0]]C0630607	abbeymycin,2s 2alpha 11beta 11abeta 1 2 3 10 11 11a hexahydro 2 hydroxy 11 methoxy 5h pyrrolo 2 1 c 1 4 benzodiazepin 5 one	[[0,1,0,0,0,0,0,0,0,0,0],[0,1,0,0,0,0,0,0,0,0,0]]C3178183	3 3 di 2 carboxyethyl 1 1 1 1 tetramethyl 1h disulfobenz e indocarbocyanin,3 3 dctdic	[[0,1,0,0,0,0,0,0,0,0,0],[0,1,0,0,0,0,0,0,0,0,0]]C3178181	3 methyl 6 prop 1 en 2 yl cyclohex 3 ene 1 2 diol,3 methyl peced	[[0,1,0,0,0,0,0,0,0,0,0],[0,1,0,0,0,0,0,0,0,0,0]]C1310648	neuronika,klinge brand of kawain	[[0,1,0,0,0,0,0,0,0,0,0],[0,1,0,0,0,0,0,0,0,0,0]]C3178184	3 hydroxy 11 oxopregn 17 20 ene 21 nitrile	[[0,1,0,0,0,0,0,0,0,0,0]]C1981677	aprobarbital 124 urine	[[0,0,0,1,0,0,0,0,0,0,0]]C1981676	aprobarbital 124 bld ser plas	[[0,0,0,1,0,0,0,0,0,0,0]]C0217556	technetium tc 94m teboroxime,tc94m teboroxime,technetium 94m teboroxime,tc 94m teboroxime	[[0,1,0,0,0,0,0,0,0,0,0],[0,1,0,0,0,0,0,0,0,0,0],[0,1,0,0,0,0,0,0,0,0,0],[0,1,0,0,0,0,0,0,0,0,0]]C1981673	apramycin 124 isolate	[[0,0,0,1,0,0,0,0,0,0,0]]C0217550	cucurbita maxima cmti protein	[[0,1,0,0,0,0,0,0,0,0,0]]C1981678	aprobarbital 124 xxx	[[0,0,0,1,0,0,0,0,0,0,0]]C0993617	chemipen	[[0,1,0,0,0,0,0,0,0,0,0]]C0286563	ici 207968,ici207968	[[0,1,0,0,0,0,0,0,0,0,0],[0,1,0,0,0,0,0,0,0,0,0]]C1721708	1 4 furo 2 3 b quinolin 4 ylamino phenyl ethanone,fqap ethanone	[[0,1,0,0,0,0,0,0,0,0,0],[0,1,0,0,0,0,0,0,0,0,0]]C0286567	risotilide,risitolide	[[0,1,0,0,0,0,0,0,0,0,0],[0,1,0,0,0,0,0,0,0,0,0]]C0286565	4 fluorohexbutinol,1 4 cyclohexyl 4 hydroxy 4 4 fluorophenyl 2 butynyl piperidine	[[0,1,0,0,0,0,0,0,0,0,0],[0,1,0,0,0,0,0,0,0,0,0]]C3253347	n 3 fluoro 4 5 phenylthieno 2 3 d pyrimidin 4 yloxy phenyl 1 4 fluorophenyl 2 oxo 1 2 dihydropyridine 3 carboxamide,3 fluoro pppfod carboxamide	[[0,1,0,0,0,0,0,0,0,0,0],[0,1,0,0,0,0,0,0,0,0,0]]C0615458	5 fluorocyclophosphamide,2 di 2 chloroethyl amino tetrahydro 5 fluoro 2h 1 3 2 oxazaphosphorine 2 oxide	[[0,1,0,0,0,0,0,0,0,0,0],[0,1,0,0,0,0,0,0,0,0,0]]C2927989	bioflavonoids quercetin vitis extract	[[0,0,0,0,0,0,0,1,0,0,0]]C0615452	harpagogenine	[[0,1,0,0,0,0,0,0,0,0,0]]C2825480	pibutidine	[[0,0,0,0,0,0,0,0,0,1,0]]C0615451	combiflex	[[0,0,0,0,0,0,0,1,0,0,0]]C0615456	cis 5 fluorocyclophosphamide	[[0,1,0,0,0,0,0,0,0,0,0]]C0349802	compound proprietary antacids a c	[[1,0,0,0,0,0,0,0,0,0,0]]C0700817	theopek	[[0,1,0,0,0,0,0,0,0,0,0]]C0287816	e 2020,e2020	[[0,1,0,0,0,0,0,0,0,0,0],[0,1,0,0,0,0,0,0,0,0,0]]C0387171	n dimethyl 4 fluorobenzyl silyl methyl piperidine,n dimethyl 4 fluor benzil silil methyl piperidine	[[0,1,0,0,0,0,0,0,0,0,0],[0,1,0,0,0,0,0,0,0,0,0]]C2965039	desipramine 124 xxx	[[0,0,0,1,0,0,0,0,0,0,0]]C0387172	rgh 5002	[[0,1,0,0,0,0,0,0,0,0,0]]C2929686	benzocaine capsaicin menthol methyl salicylate	[[0,0,0,0,0,0,0,1,0,0,0]]C2142852	brompheniramine codeine pseudoephedrine	[[0,0,0,0,0,0,0,1,0,0,0]]C2142855	carbinoxamine guaifenesin pseudoephedrine	[[0,0,0,0,0,0,0,1,0,0,0]]C2947810	centergy dm	[[0,0,0,0,0,0,0,1,0,0,0]]C2142857	acetaminophen chlorpheniramine codeine pseudoephedrine	[[0,0,0,0,0,0,0,1,0,0,0]]C1949898	ceprotin	[[0,0,0,0,0,0,0,1,0,0,0]]C0658374	5 7 dihydroxy 6 methoxy 2 phenylchromen 4 one,oroxylin a	[[0,1,0,0,0,0,0,0,0,0,0],[0,1,0,0,0,0,0,0,0,0,0]]C0700813	uniphylline	[[0,1,0,0,0,0,0,0,0,0,0]]C2723094	hygel	[[0,0,0,0,0,0,0,1,0,0,0]]C0700812	theovent	[[0,0,0,0,0,0,0,1,0,0,0]]C1589721	viranol	[[0,0,0,0,0,0,0,1,0,0,0]]C0091198	2 2 dithiobis	[[0,1,0,0,0,0,0,0,0,0,0]]C0146422	trans methyl retinoate	[[0,1,0,0,0,0,0,0,0,0,0]]C0146421	trans metanicotine	[[0,1,0,0,0,0,0,0,0,0,0]]C0091193	2 2 dihydroxy 3 3 5 5 tetrachlorodiphenylsulfone	[[0,1,0,0,0,0,0,0,0,0,0]]C0146425	n ethyl 7 9 dihydroxyoctahydrobenzo f quinoline,trans n ethyl 7 9 dihydroxyoctahydrobenzo f quinoline	[[0,1,0,0,0,0,0,0,0,0,0],[0,1,0,0,0,0,0,0,0,0,0]]C0982355	potassium stearate	[[0,0,1,0,0,0,0,0,0,0,0]]C0982350	polyglycerol esters of fatty acids	[[0,0,0,0,0,0,0,0,0,0,1]]C0626849	1 methyl 3 hydroxypyridine 2 one,1 methyl 3 hydroxypyrid 2 one,1 mhpo	[[0,1,0,0,0,0,0,0,0,0,0],[0,1,0,0,0,0,0,0,0,0,0],[0,1,0,0,0,0,0,0,0,0,0]]C0982353	potassium lactate	[[0,0,0,0,0,0,0,0,0,1,0]]C0626846	punctaporonin f	[[0,1,0,0,0,0,0,0,0,0,0]]C0626845	m 189122,m189122	[[0,1,0,0,0,0,0,0,0,0,0],[0,1,0,0,0,0,0,0,0,0,0]]C0626843	punctaporonin e,tri o methyl deriv 2 3 7 8 tetrahydroxy 5 methyl benzo c phenanthridinium	[[0,1,0,0,0,0,0,0,0,0,0],[0,1,0,0,0,0,0,0,0,0,0]]C0626842	m 171950,m171950	[[0,1,0,0,0,0,0,0,0,0,0],[0,1,0,0,0,0,0,0,0,0,0]]C0626840	punctaporonin d	[[0,1,0,0,0,0,0,0,0,0,0]]C0912841	bm 16 2266,6 7 dihydroxy 2 oxo 2h chromen 4 ylmethylsulfanyl acetic acid	[[0,1,0,0,0,0,0,0,0,0,0],[0,1,0,0,0,0,0,0,0,0,0]]C0051756	amphoglucamine,amphoglucomine,compd with 1 deoxy 1 methylamino d glucitol 1 1 amphotericin b,n methyl d glucamine amphotericin b	[[0,1,0,0,0,0,0,0,0,0,0],[0,1,0,0,0,0,0,0,0,0,0],[0,1,0,0,0,0,0,0,0,0,0],[0,1,0,0,0,0,0,0,0,0,0]]C0051751	amphethinile,2 amino 3 cyano 5 phenylthio indole	[[0,1,0,0,0,0,0,0,0,0,0],[0,1,0,0,0,0,0,0,0,0,0]]C0051750	amphetaminil,n alpha methylphenethyl 2 phenylglycinonitrile	[[0,0,0,1,0,0,0,1,0,0,0],[0,1,0,0,0,0,0,0,0,0,0]]C0912848	sodium 2 2 bipyridine oxodiperoxovanadate	[[0,1,0,0,0,0,0,0,0,0,0]]C0051759	amphotalide,n 5 4 aminophenoxy pentyl phthalimide,n 5 p aminophenoxy pentyl phthalimide	[[0,1,0,0,0,0,0,0,0,1,0],[0,1,0,0,0,0,0,0,0,0,0],[0,1,0,0,0,0,0,0,0,0,0]]C0051758	amphomycin,amfomycin	[[0,1,0,0,0,0,0,0,0,1,0],[1,0,0,0,0,0,0,0,0,0,0]]C0908021	trilobinine	[[0,1,0,0,0,0,0,0,0,0,0]]C0700819	theon	[[0,0,0,0,0,0,0,0,0,1,0]]C0656376	matlystatin e	[[0,1,0,0,0,0,0,0,0,0,0]]C0702085	bay vp 2674	[[0,1,0,0,0,0,0,0,0,0,0]]C0075013	spirobromin	[[0,1,0,0,0,0,0,0,0,0,0]]C3163571	brilinta	[[0,0,0,0,0,0,0,1,0,0,0]]C0075018	spirogermanium,8 8 diethyl n n dimethyl 2 aza 8 germaspiro 4 5 decane 2 propanamine,spiro 32,spg,2 3 dimethyl amino propyl 8 8 diethyl 2 aza 8 germaspiro 4 5 decane,dihydrochloride 8 diethyl n n dimethyl 8 2 aza 8 germaspiro 4 5 decane 2 propanamine,8 8 diethyl n n dimethyl 3 aza 8 germaspiro 4 5 decane 2 propanamine,spirogermanium compound	[[0,1,0,0,0,0,0,0,0,1,0],[0,1,0,0,0,0,0,0,0,0,0],[0,0,0,0,1,0,0,0,0,1,0],[0,0,0,0,1,0,0,0,0,0,0],[0,0,0,0,1,0,0,0,0,0,0],[0,0,0,0,1,0,0,0,0,0,0],[0,0,0,0,1,0,0,0,0,0,0],[0,0,0,0,0,0,0,0,0,1,0]]C0075019	spirohydantoin mustard,3 2 bis 2 chloroethyl amino ethyl 1 3 diazaspiro 4 5 decane 2 4 dione,spiromustine,1 3 diazaspiro 4 5 decane 2 4 dione 3 2 bis chloroethyl amino ethyl,shm	[[0,0,0,0,1,0,0,0,0,1,0],[0,1,0,0,0,0,0,0,0,0,0],[0,0,0,0,1,0,0,0,0,1,0],[0,0,0,0,1,0,0,0,0,0,0],[0,0,0,0,1,0,0,0,0,0,0]]C0287213	bch 189,bch189	[[0,1,0,0,0,0,0,0,0,0,0],[0,1,0,0,0,0,0,0,0,0,0]]C0064841	leukotoxin,leucotoxins	[[0,0,0,0,0,0,0,0,1,0,0],[0,1,0,0,0,0,0,0,0,0,0]]C0287219	111 indium dtpa igg complex,dtpa in igg complex	[[0,1,0,0,0,0,0,0,0,0,0],[0,1,0,0,0,0,0,0,0,0,0]]C2242260	lta 360	[[0,0,0,0,0,0,0,1,0,0,0]]C2742308	xanthepinone	[[0,1,0,0,0,0,0,0,0,0,0]]C0879370	xr 5000	[[0,1,0,0,0,0,0,0,0,0,0]]C0658994	stimuliv	[[0,1,0,0,0,0,0,0,0,0,0]]C0658996	aluminum zirconium tetrachlorohydrexglycerin,al zr tchg	[[0,1,0,0,0,0,0,0,0,0,0],[0,1,0,0,0,0,0,0,0,0,0]]C0297847	fluoroanilinoazatoxin	[[0,1,0,0,0,0,0,0,0,0,0]]C2346803	anti cd3 immunotoxin a dmdt390 bisfv,a dmdt390 bisfv	[[0,0,0,0,0,0,0,0,0,1,0],[0,0,0,0,0,0,0,0,0,1,0]]C2740652	onion allergenic extract,onion	[[0,0,0,0,0,0,0,1,0,0,0],[0,0,1,0,0,0,0,0,0,0,0]]C1703295	execof xp	[[0,0,0,0,0,0,0,1,0,0,0]]C0661035	cyclobuxine	[[0,1,0,0,0,0,0,0,0,0,0]]C0297848	nsc 662304	[[0,1,0,0,0,0,0,0,0,0,0]]C1993568	norfenfluramine 124 bld ser plas	[[0,0,0,1,0,0,0,0,0,0,0]]C1450816	2 4 morpholinoanilino 6 cyclohexylaminopurine,reversine	[[0,1,0,0,0,0,0,0,0,0,0],[0,1,0,0,0,0,0,0,0,0,0]]C0602116	3 hydroxyphenyl diethylmethylammonium,n n diethyl 3 hydroxy n methylbenzenaminium	[[0,1,0,0,0,0,0,0,0,0,0],[0,1,0,0,0,0,0,0,0,0,0]]C0602115	methoxyambenonium	[[0,1,0,0,0,0,0,0,0,0,0]]C1680588	tetrahydropyrimidinone	[[0,1,0,0,0,0,0,0,0,0,0]]C0602110	ethomoxane,dl 8 ethoxy 2 butylaminomethyl 1 4 benzodioxan	[[0,1,0,0,0,0,0,0,0,0,0],[0,1,0,0,0,0,0,0,0,0,0]]C0769862	l 739758,l 739 758	[[0,1,0,0,0,0,0,0,0,0,0],[0,1,0,0,0,0,0,0,0,0,0]]C0894992	4ar 4aalpha 6abeta 10ar 11alpha 12calpha isomer rx 336m maleate	[[0,1,0,0,0,0,0,0,0,0,0]]C3257503	cinnamomum camphora leaf	[[0,0,1,0,0,0,0,0,0,0,0]]C0894991	4ar 4aalpha 6abeta 10ar 11alpha 12calpha isomer hydrochloride rx 336m	[[0,1,0,0,0,0,0,0,0,0,0]]C3257505	hibiscus rosa sinensis leaf	[[0,0,1,0,0,0,0,0,0,0,0]]C0353706	guar preparation	[[0,0,0,0,0,1,1,0,0,0,0]]C3257506	juniperus deppeana wood oil	[[0,0,1,0,0,0,0,0,0,0,0]]C0050408	acemannan,polymannoacetate	[[1,0,0,0,0,0,0,0,0,0,1],[0,0,0,0,0,0,0,0,0,0,1]]C0050409	acemetacin,carboxymethyl ester 1 4 chlorobenzoyl 5 methoxy 2 methyl 1h indole 3 acetic acid,indomethacin carboxymethyl ester	[[1,0,0,0,0,0,0,1,0,1,0],[0,1,0,0,0,0,0,0,0,0,0],[0,1,0,0,0,0,0,0,0,0,0]]C1982512	betamethasone 124 urine	[[0,0,0,1,0,0,0,0,0,0,0]]C1982511	betamethasone 124 bld ser plas	[[0,0,0,1,0,0,0,0,0,0,0]]C2984005	narceine trihydrate	[[0,0,0,0,0,0,0,0,0,1,0]]C0050401	aceanthrylene	[[0,1,0,0,0,0,0,0,0,0,0]]C0050402	acecarbromal,1 acetyl 3 2 bromo 2 ethylbutyryl urea,acetylcarbobromal,acetylcarbromal	[[0,1,0,0,0,0,0,0,0,1,0],[0,0,0,0,0,0,0,0,0,0,1],[0,0,0,0,0,0,0,0,0,0,1],[1,0,0,0,0,0,0,0,0,0,1]]C0050403	aceclofenac,2 2 6 dichlorophenyl amino phenylacetoxyacetic acid	[[1,0,0,0,0,0,0,1,0,1,0],[0,1,0,0,0,0,0,0,0,0,0]]C0050404	acefyllin piperazinate,acephylline piperazinate,acepifylline,acepiphyllin,theophyllin 7 acetic acid piperazine salt,theophylline ethanoate of piperazine	[[0,1,0,0,0,0,0,0,0,0,0],[0,1,0,0,0,0,0,0,0,0,0],[0,1,0,0,0,0,0,0,0,0,0],[0,1,0,0,0,0,0,0,0,0,0],[0,1,0,0,0,0,0,0,0,0,0],[0,1,0,0,0,0,0,0,0,0,0]]C0050405	acefylline,7 theophyllineacetic acid,acephylline,theophylline 7 acetic acid	[[0,0,0,0,0,0,1,0,0,0,0],[0,1,0,0,0,0,0,0,0,0,0],[0,0,0,0,0,0,1,0,0,0,0],[0,1,0,0,0,0,0,0,0,0,0]]C0050406	aceglatone,2 5 di o acetyl glucaro 1 4 6 3 dilactone,diacetyl glucaro 1 4 6 3 dilactone	[[0,1,0,0,0,0,0,0,0,1,0],[0,1,0,0,0,0,0,0,0,0,0],[0,1,0,0,0,0,0,0,0,0,0]]C0050407	aceglutamide,n acetyl l glutamine,n acetylglutamine	[[0,1,0,0,0,0,0,0,0,1,0],[0,1,0,0,0,0,0,0,0,0,0],[0,1,0,0,0,0,0,0,0,0,0]]C1529046	tetrazep abz,abz brand of tetrazepam	[[0,1,0,0,0,0,0,0,0,0,0],[0,1,0,0,0,0,0,0,0,0,0]]C1529047	tetrazep von ct,ct arzneimittel brand of tetrazepam	[[0,1,0,0,0,0,0,0,0,0,0],[0,1,0,0,0,0,0,0,0,0,0]]C1529044	tetrarelax,mibe brand of tetrazepam	[[0,1,0,0,0,0,0,0,0,0,0],[0,1,0,0,0,0,0,0,0,0,0]]C1529045	tetrazep 1a pharma,1a brand of tetrazepam	[[0,1,0,0,0,0,0,0,0,0,0],[0,1,0,0,0,0,0,0,0,0,0]]C1529042	tetra saar,mip brand of tetrazepam	[[0,1,0,0,0,0,0,0,0,0,0],[0,1,0,0,0,0,0,0,0,0,0]]C1529043	tetramdura,merck dura brand of tetrazepam	[[0,1,0,0,0,0,0,0,0,0,0],[0,1,0,0,0,0,0,0,0,0,0]]C1529040	rilex,gry brand of tetrazepam	[[0,1,0,0,0,0,0,0,0,0,0],[0,1,0,0,0,0,0,0,0,0,0]]C1529041	tethexal,hexal brand of tetrazepam	[[0,1,0,0,0,0,0,0,0,0,0],[0,1,0,0,0,0,0,0,0,0,0]]C0620036	s carbobenzoxyglutathione,s cbg	[[0,1,0,0,0,0,0,0,0,0,0],[0,1,0,0,0,0,0,0,0,0,0]]C0620031	6 7 bis 1 aziridinyl 5 8 quinazolinedione,6 7 baqd	[[0,1,0,0,0,0,0,0,0,0,0],[0,1,0,0,0,0,0,0,0,0,0]]C0166138	sperabillin d,e e 3 amino n 3 amino 3 iminopropyl 2 3 4 6 7 pentadeoxy 6 1 oxo 2 4 hexadienyl amino d xylo heptonamide	[[0,1,0,0,0,0,0,0,0,0,0],[0,1,0,0,0,0,0,0,0,0,0]]C0057755	diazophenylthioether,4 4 dithiobis benzenediazonium,dapte	[[0,1,0,0,0,0,0,0,0,0,0],[0,1,0,0,0,0,0,0,0,0,0],[0,1,0,0,0,0,0,0,0,0,0]]C0166137	sperabillin c,e e 3 amino n 3 amino 3 iminopropyl 2 3 4 6 tetradeoxy 6 1 oxo 2 4 hexadienyl amino l threo hexonamide	[[0,1,0,0,0,0,0,0,0,0,0],[0,1,0,0,0,0,0,0,0,0,0]]C0166134	1 2 bis 2 hydroxyphenyl ethylenediamine dichloroplatinum ii,bhpedp	[[0,1,0,0,0,0,0,0,0,0,0],[0,1,0,0,0,0,0,0,0,0,0]]C0166135	sperabillin a,e z 3 amino n 3 amino 3 iminopropyl 2 3 4 6 tetradeoxy 6 1 oxo 2 4 hexadienyl amino l threo hexonamide	[[0,1,0,0,0,0,0,0,0,0,0],[0,1,0,0,0,0,0,0,0,0,0]]C0166132	imidazolium tetrachlorobisimidazole ruthenium,imdcr	[[0,1,0,0,0,0,0,0,0,0,0],[0,1,0,0,0,0,0,0,0,0,0]]C0057750	mebhydrolin napadisylate,diazoline,compd with 2 3 4 5 tetrahydro 2 methyl 5 phenylmethyl 1h pyrido 4 3 b indole 1 2 1 5 naphthalenedisulfonic acid,mebhydroline 1 5 naphthalenedisulfonate	[[0,1,0,0,0,0,0,0,0,0,0],[0,0,0,0,0,0,1,0,0,0,0],[0,1,0,0,0,0,0,0,0,0,0],[0,1,0,0,0,0,0,0,0,0,0]]C0057753	diazonaphthalenedisulfonic acid,diazo 7 amino 1 3 naphthalenedisulfonic acid,nds	[[0,1,0,0,0,0,0,0,0,0,0],[0,1,0,0,0,0,0,0,0,0,0],[0,1,0,0,0,0,0,0,0,0,0]]C3251900	3 4 fluorophenyl 6 7 dimethoxy 3a 4 dihydro 3h indeno 1 2 c pyrazole 2 carbothioamide,fddip carbothioamide	[[0,1,0,0,0,0,0,0,0,0,0],[0,1,0,0,0,0,0,0,0,0,0]]C0074762	sus scrofa spai 3 protein,sodium potassium atpase inhibitor 3,sus scrofa sodium potassium atpase inhibitor 3	[[0,1,0,0,0,0,0,0,0,0,0],[0,1,0,0,0,0,0,0,0,0,0],[0,1,0,0,0,0,0,0,0,0,0]]C0074761	sus scrofa spai 2 protein,sodium potassium atpase inhibitor 2,sus scrofa sodium potassium atpase inhibitor 2 protein	[[0,1,0,0,0,0,0,0,0,0,0],[0,1,0,0,0,0,0,0,0,0,0],[0,1,0,0,0,0,0,0,0,0,0]]C0074760	sus scrofa spai 1 protein,sodium potassium atpase inhibitor 1,sus scrofa sodium potassium atpase inhibitor 1 protein	[[0,1,0,0,0,0,0,0,0,0,0],[0,1,0,0,0,0,0,0,0,0,0],[0,1,0,0,0,0,0,0,0,0,0]]C0074767	sodium silicate,sodium salt silicic acid	[[1,0,1,0,0,0,0,0,0,0,1],[0,0,0,0,0,0,0,0,0,0,1]]C0074765	sodium selenate,disodium selenate,disodium salt selenic acid h2seo4	[[1,0,0,0,0,0,0,1,0,1,0],[0,0,0,0,0,0,0,0,0,0,1],[0,0,0,0,0,0,0,0,0,0,1]]C0074764	sodium pyrophosphate,tetrasodium salt diphosphoric acid,sodium diphosphate,tetrasodium pyrophosphate,sodium acid pyrophosphate	[[1,0,1,0,0,0,0,0,0,1,0],[0,0,0,0,0,0,0,0,0,0,1],[0,0,0,0,0,0,0,0,0,0,1],[1,0,0,0,0,0,0,0,0,0,1],[1,0,1,0,0,0,0,0,0,0,0]]C0771695	pralidoxime methyl sulfate,n methylpyridinium 2 aldoxime methylsulfate	[[0,1,0,0,0,0,0,0,0,0,0],[0,1,0,0,0,0,0,0,0,0,0]]C0074769	sodium sulfate,sodium sulphate,glaubers salt,bisodium sulfate	[[0,0,1,0,0,0,1,1,0,1,0],[1,0,0,0,0,0,1,0,0,0,0],[0,0,0,0,0,0,0,0,0,1,0],[0,0,0,0,0,0,0,0,0,1,0]]C3254260	18f florbetaben	[[0,1,0,0,0,0,0,0,0,0,0]]C0174340	2 dihydroxymethyl 2 hydroxy 2h pyran 3 6h one	[[0,1,0,0,0,0,0,0,0,0,0]]C0174341	cortalcerone	[[0,1,0,0,0,0,0,0,0,0,0]]C2948128	docosahexaenoate omega 3 acid ethyl esters	[[0,0,0,0,0,0,0,1,0,0,0]]C1515694	panvac v	[[0,0,0,0,1,0,0,0,0,0,0]]C0718086	tetrahydrozoline ophthalmic	[[0,0,0,0,0,0,1,0,0,0,0]]C0755403	calicheamicin theta 1 i,calicheamicin theta1	[[0,1,0,0,0,0,0,0,0,0,0],[0,1,0,0,0,0,0,0,0,0,0]]C1987573	etilefrine 124 urine	[[0,0,0,1,0,0,0,0,0,0,0]]C1101642	steganone	[[0,1,0,0,0,0,0,0,0,0,0]]C1515696	sn 1 2 didecanoylglycerol,dic10	[[0,0,0,0,0,0,0,0,0,1,0],[0,0,0,0,0,0,0,0,0,1,0]]C2828369	coagulation factor ii human,prothrombin	[[0,0,0,0,0,0,0,0,0,1,0],[0,0,0,0,0,0,0,0,0,1,0]]C2919960	berberis vulgaris fruit	[[0,0,1,0,0,0,0,0,0,0,0]]C1987571	ethylmorphine 124 urine	[[0,0,0,1,0,0,0,0,0,0,0]]C2984000	metheptazine	[[0,0,0,0,0,0,0,0,0,1,0]]C0728715	cl 395,cl395	[[0,1,0,0,0,0,0,0,0,0,0],[0,1,0,0,0,0,0,0,0,0,0]]C1566319	sk 7041	[[0,1,0,0,0,0,0,0,0,0,0]]C0635771	7 2 2 methyl 1 oxobutoxy methyl 1 cyclohexyl 3 hydroxy 5 oxoheptanoic acid,momc acid	[[0,1,0,0,0,0,0,0,0,0,0],[0,1,0,0,0,0,0,0,0,0,0]]C0603641	nicophyllin	[[0,1,0,0,0,0,0,0,0,0,0]]C0603643	nidulin,o methylustin	[[0,1,0,0,0,0,0,0,0,0,0],[0,1,0,0,0,0,0,0,0,0,0]]C0603645	nihydrazone,5 nitrofurfurylidene hydrazide acetic acid	[[0,1,0,0,0,0,0,0,0,0,0],[0,1,0,0,0,0,0,0,0,0,0]]C0603646	2 2 nitrobenzenesulfenamido benzothiazole,n 2 benzothiazolyl 2 nitro benzenesulfenamide	[[0,1,0,0,0,0,0,0,0,0,0],[0,1,0,0,0,0,0,0,0,0,0]]C0603647	2 4 nitrobenzenesulfenamido benzothiazole,n 2 benzothiazolyl 4 nitro benzenesulfenamide	[[0,1,0,0,0,0,0,0,0,0,0],[0,1,0,0,0,0,0,0,0,0,0]]C0603648	4 nitrobenzoic acid 4 amidinoanilide	[[0,1,0,0,0,0,0,0,0,0,0]]C0635778	3 methoxy 4 hydroxyclomiphene,1 4 2 diethylamino ethoxy phenyl 1 3 methoxy 4 hydroxyphenyl 2 phenyl 2 chloroethane	[[0,1,0,0,0,0,0,0,0,0,0],[0,1,0,0,0,0,0,0,0,0,0]]C1881880	mofloverine	[[0,0,0,0,0,0,0,0,0,1,0]]C2930035	acetaminophen chlorpheniramine phenylephrine phenyltoloxamine	[[0,0,0,0,0,0,0,1,0,0,0]]C0762956	3h 1 2 dihydro 2 4 methylphenylamino methy 1 pyrrolizinone	[[0,1,0,0,0,0,0,0,0,0,0]]C1509940	aloe polysaccharide	[[0,0,0,0,0,0,0,1,0,0,0]]C0762955	pyrrolizinone z 47	[[0,1,0,0,0,0,0,0,0,0,0]]C0306572	lac hydrin five	[[0,0,0,0,0,0,0,1,0,0,0]]C0625120	ephedran	[[0,1,0,0,0,0,1,0,0,0,0]]C0306571	kyolic	[[0,0,0,0,0,0,1,0,0,0,0]]C1721268	clazosentan,n 6 2 hydroxyethoxy 5 2 methoxyphenoxy 2 2 1h tetrazol 5 yl pyridin 4 yl pyrimidin 4 yl 5 methylpyridine 2 sulfonamide	[[0,1,0,0,0,0,0,0,0,1,0],[0,1,0,0,0,0,0,0,0,1,0]]C1721269	axv 034	[[0,1,0,0,0,0,0,0,0,0,0]]C0762959	emd 66203	[[0,1,0,0,0,0,0,0,0,0,0]]C0244672	fluorodopa f 18,2 fluoro 5 hydroxytyrosine,3 4 dihydroxy 6 fluorophenylalanine,6 18f fluoro l 3 4 dihydroxyphenylalanine,3 2 fluoro 18 f 4 5 dihydroxyphenyl l alanine,18 f dopa,6 fluoro dopa,18 f labeled cpd fluorodopa f 18,6 18f fluoro l dopa,6 fluorodopa,18f fdopa,fluorine f 18 fluorodopa	[[0,1,0,0,0,0,0,0,0,0,0],[0,1,0,0,0,0,0,0,0,0,0],[0,1,0,0,0,0,0,0,0,0,0],[0,1,0,0,0,0,0,0,0,0,0],[0,1,0,0,0,0,0,0,0,0,0],[0,1,0,0,0,0,0,0,0,0,0],[0,1,0,0,0,0,0,0,0,0,0],[0,1,0,0,0,0,0,0,0,0,0],[0,1,0,0,0,0,0,0,0,0,0],[0,1,0,0,0,0,0,0,0,0,0],[0,0,0,0,0,0,0,0,0,1,0],[0,0,0,0,0,0,0,0,0,1,0]]C3179591	5 4 fluorophenyl 2 hydroxypyridine,fp2hp cpd	[[0,1,0,0,0,0,0,0,0,0,0],[0,1,0,0,0,0,0,0,0,0,0]]C0304596	undecylenic acid and undecylenate salt	[[1,0,0,0,0,0,0,0,0,0,0]]C0304597	undecylenic acid and zinc undecylenate,zincundecate	[[1,0,0,0,0,0,0,1,0,0,0],[1,0,0,0,0,0,0,0,0,0,0]]C0304594	naftifine hydrochloride,n cinnamyl n methyl 1 naphthalenemethylamine hydrochloride,e n methyl n 1 naphthylmethyl 3 phenyl 2 propen 1 amine hydrochloride	[[0,1,0,0,0,0,0,1,0,1,0],[0,1,0,0,0,0,0,0,0,0,0],[0,0,0,0,0,0,0,0,0,1,0]]C0304595	undecylenate salt	[[1,0,0,0,0,0,0,0,0,0,0]]C0304592	mucous membrane antifungal agent	[[1,0,0,0,0,0,0,0,0,0,0]]C0304593	benzoic acid salicylic acid	[[1,0,0,0,0,0,0,0,0,0,0]]C0304590	mucous membrane antiviral agent	[[1,0,0,0,0,0,0,0,0,0,0]]C0304591	skin antifungal agent	[[1,0,0,0,0,0,0,0,0,0,0]]C2743630	benzylpenicillin sodium drug combination procaine benzylpenicillin benzathine benzylpenicillin	[[0,1,0,0,0,0,0,0,0,0,0]]C2743631	sodium fluoride topical preparation	[[0,1,0,0,0,0,0,0,0,0,0]]C0304599	pediculicide,pediculocide	[[0,0,0,0,0,0,1,0,0,0,1],[0,0,0,0,0,0,1,0,0,0,1]]C0650505	bis glycino iron ii	[[0,1,0,0,0,0,0,0,0,0,0]]C0650504	ferrochel	[[0,1,0,0,0,0,0,0,0,0,0]]C3179595	5 methylene 1 prop 2 enoyl 4 phenyldihydropyrrol 2 one,dhp 4 cpd	[[0,1,0,0,0,0,0,0,0,0,0],[0,1,0,0,0,0,0,0,0,0,0]]C0389187	cmdbs 25,carboxymethylbenzylamide sulfonate dextran cmdbs25,cmdbs25	[[0,1,0,0,0,0,0,0,0,0,0],[0,1,0,0,0,0,0,0,0,0,0],[0,1,0,0,0,0,0,0,0,0,0]]C0950627	rad 366	[[0,1,0,0,0,0,0,0,0,0,0]]C0950626	wf1360	[[0,1,0,0,0,0,0,0,0,0,0]]C1958530	oxathiocoraline	[[0,1,0,0,0,0,0,0,0,0,0]]C0358919	4 quinolones,4 quinolinones,4 oxoquinolines	[[1,1,0,0,0,0,0,0,0,0,1],[0,1,0,0,0,0,0,0,0,0,1],[0,1,0,0,0,0,0,0,0,0,1]]C1638383	liquid pedvaxhib	[[0,0,0,0,0,0,0,1,0,0,0]]C0959901	monohydrochloride ko 1313	[[0,1,0,0,0,0,0,0,0,0,0]]C0959900	sodium salt vanillin	[[0,1,0,0,0,0,0,0,0,0,0]]C0959902	isomer monohydrochloride ko 1313	[[0,1,0,0,0,0,0,0,0,0,0]]C3179599	mitotempo	[[0,1,0,0,0,0,0,0,0,0,0]]C0959907	azacyclonol hydrochloride	[[0,1,0,0,0,0,0,0,0,1,0]]C0959906	germine hydrochloride	[[0,1,0,0,0,0,0,0,0,0,0]]C3177209	stambomycin d	[[0,1,0,0,0,0,0,0,0,0,0]]C1564341	thiamazol hexal,hexal brand of methimazole	[[0,1,0,0,0,0,0,0,0,0,0],[0,1,0,0,0,0,0,0,0,0,0]]C3177208	stambomycin c	[[0,1,0,0,0,0,0,0,0,0,0]]C0119104	gf 109203x,gf109203x	[[0,1,0,0,0,0,0,0,0,0,0],[0,1,0,0,0,0,0,0,0,0,0]]C0119101	gestormone	[[0,1,0,0,0,0,0,0,0,0,0]]C0119102	gestranol	[[0,1,0,0,0,0,0,0,0,0,0]]C1529436	acemetadoc,docpharm brand of acemetacin	[[0,1,0,0,0,0,0,0,0,0,0],[0,1,0,0,0,0,0,0,0,0,0]]C0526518	phenylalanylphenylalanine methyl ester,phe phe ome	[[0,1,0,0,0,0,0,0,0,0,0],[0,1,0,0,0,0,0,0,0,0,0]]C1564344	thiophenicol,sanofi brand of thiamphenicol	[[0,1,0,0,0,0,0,0,0,0,0],[0,1,0,0,0,0,0,0,0,0,0]]C0162932	heparegen	[[0,0,0,0,0,0,0,0,0,1,0]]C0162933	torak,torak ec 24	[[0,1,0,0,0,0,0,0,0,0,0],[0,1,0,0,0,0,0,0,0,0,0]]C1564345	urfamycin,zambon brand of thiamphenicol	[[0,1,0,0,0,0,0,0,0,0,0],[0,1,0,0,0,0,0,0,0,0,0]]C0526510	aspirin codeine,co codaprin,codeine drug combination aspirin	[[1,1,0,0,0,0,1,1,0,0,0],[0,0,0,0,0,0,1,0,0,0,0],[0,1,0,0,0,0,0,0,0,0,0]]C0526512	synercid	[[0,1,0,0,0,0,1,0,0,0,0]]C0526513	dalfopristin quinupristin	[[1,1,0,1,0,0,0,1,0,0,1]]C0625782	n propyl 20 dihydroprednisolonamide,pdhpa	[[0,1,0,0,0,0,0,0,0,0,0],[0,1,0,0,0,0,0,0,0,0,0]]C1097068	epothilone k	[[0,1,0,0,0,0,0,0,0,0,0]]C0625786	pyruvic acid semicarbazone,2 aminocarbonyl hydrazono propanoic acid	[[0,1,0,0,0,0,0,0,0,0,0],[0,1,0,0,0,0,0,0,0,0,0]]C0625788	pyruvic acid thiosemicarbazone,2 aminothioxomethyl hydrazono propanoic acid,patsc	[[0,1,0,0,0,0,0,0,0,0,0],[0,1,0,0,0,0,0,0,0,0,0],[0,1,0,0,0,0,0,0,0,0,0]]C2965954	maprotiline 124 xxx	[[0,0,0,1,0,0,0,0,0,0,0]]C0965042	palmarumycin cp,palmarumycin cp1	[[0,1,0,0,0,0,0,0,0,0,0],[0,1,0,0,0,0,0,0,0,0,0]]C0965045	a 134974	[[0,1,0,0,0,0,0,0,0,0,0]]C0965044	jte 907	[[0,1,0,0,0,0,0,0,0,0,0]]C3245236	monistat itch relief	[[0,0,0,0,0,0,0,1,0,0,0]]C0670871	9 mono n methylnorharman,9 menh	[[0,1,0,0,0,0,0,0,0,0,0],[0,1,0,0,0,0,0,0,0,0,0]]C0117430	feiba vh,aicc complex,anti inhibitory coagulant complex	[[0,1,0,0,0,0,0,0,0,0,0],[0,1,0,0,0,0,0,0,0,0,0],[0,1,0,0,0,0,0,0,0,0,0]]C0626477	8 aminoguanosine triphosphate,8 amino guanosine 5 tetrahydrogen triphosphate	[[0,1,0,0,0,0,0,0,0,0,0],[0,1,0,0,0,0,0,0,0,0,0]]C0626475	tai 998	[[0,1,0,0,0,0,0,0,0,0,0]]C0626472	khonsuride	[[0,1,0,0,0,0,0,0,0,0,0]]C0305882	desyrel dividose	[[0,0,0,0,0,0,0,1,0,0,0]]C2609587	quadra hist d ped reformulated mar 2009	[[0,0,0,0,0,0,0,1,0,0,0]]C1529434	azeat,mibe brand of acemetacin	[[0,1,0,0,0,0,0,0,0,0,0],[0,1,0,0,0,0,0,0,0,0,0]]C2609582	sucrets original	[[0,0,0,0,0,0,0,1,0,0,0]]C0626478	homobaldrinal	[[0,1,0,0,0,0,0,0,0,0,0]]C0626479	heraclenin	[[0,1,0,0,0,0,0,0,0,0,0]]C2350102	tramex,antigen brand of tramadol hydrochloride	[[0,1,0,0,0,0,0,0,0,0,0],[0,1,0,0,0,0,0,0,0,0,0]]C1565433	cx plus	[[0,1,0,0,0,0,0,0,0,0,0]]C2350100	tramagetic,christiaens brand of tramadol hydrochloride,azupharma brand of tramadol hydrochloride	[[0,1,0,0,0,0,0,0,0,0,0],[0,1,0,0,0,0,0,0,0,0,0],[0,1,0,0,0,0,0,0,0,0,0]]C2350101	tramagit,krewel brand of tramadol hydrochloride	[[0,1,0,0,0,0,0,0,0,0,0],[0,1,0,0,0,0,0,0,0,0,0]]C2350106	zamudol,viatris brand of tramadol	[[0,1,0,0,0,0,0,0,0,0,0],[0,1,0,0,0,0,0,0,0,0,0]]C1451743	ce 108	[[0,1,0,0,0,0,0,0,0,0,0]]C2350104	trasedal,elerte brand of tramadol hydrochloride	[[0,1,0,0,0,0,0,0,0,0,0],[0,1,0,0,0,0,0,0,0,0,0]]C0084530	sesamodil,3 4 dihydro 2 5 methoxy 2 3 n methyl n 2 3 4 methylenedioxy phenoxy ethyl amino propoxy phenyl 4 methyl 3 oxo 2h 1 4 benzothiazine,semotiadil	[[0,1,0,0,0,0,0,0,0,0,0],[0,1,0,0,0,0,0,0,0,0,0],[0,1,0,0,0,0,0,0,0,1,0]]C2350108	mtw tramadol,mtwtramadol,mtw brand of tramadol hydrochloride	[[0,1,0,0,0,0,0,0,0,0,0],[0,1,0,0,0,0,0,0,0,0,0],[0,1,0,0,0,0,0,0,0,0,0]]C0052409	arprinocid n oxide,1 oxide 9 2 chloro 6 fluorophenyl methyl 9h purin 6 amine,arprinocid 1 n oxide	[[0,1,0,0,0,0,0,0,0,0,0],[0,1,0,0,0,0,0,0,0,0,0],[0,1,0,0,0,0,0,0,0,0,0]]C0664274	2 2 chloro 5 nitrostyryl 1 1 propenyl benzimidazole,2 cnspb	[[0,1,0,0,0,0,0,0,0,0,0],[0,1,0,0,0,0,0,0,0,0,0]]C1100502	n 9 oxo 9h fluoren 4 yl n pyridin 2 ylurea,n ofp urea	[[0,1,0,0,0,0,0,0,0,0,0],[0,1,0,0,0,0,0,0,0,0,0]]C0962779	n acetyl 1 d inosityl 2 amino 2 deoxy alpha d glucopyranoside deacetylase	[[0,1,0,0,0,0,0,0,0,0,0]]C0009018	clonixin,2 3 chloro 2 methylphenyl amino 3 pyridinecarboxylic acid	[[0,0,0,0,0,0,1,1,0,1,0],[0,0,0,0,0,0,0,0,0,0,1]]C0009011	clonazepam,5 2 chlorophenyl 1 3 dihydro 7 nitro 2h 1 4 benzodiazepin 2 one	[[0,1,0,0,0,0,0,1,0,1,0],[0,0,0,0,0,0,0,0,0,0,1]]C0009010	clomipramine,chlomipramine,chlorimipramine,3 chloro 10 11 dihydro n n dimethyl 5h dibenz b f azepine 5 propanamine,3 chloro 5 3 dimethylamino propyl 10 11 dihydro 5h dibenz b f azepine	[[0,0,0,0,0,0,0,1,1,1,0],[0,0,0,0,0,0,1,0,0,0,1],[0,0,0,0,0,0,1,0,0,0,1],[0,0,0,0,0,0,0,0,0,0,1],[0,0,0,0,0,0,0,0,0,1,0]]C0951572	s isomer carvone	[[0,1,0,0,0,0,0,0,0,0,0]]C0115807	endobil	[[0,1,0,0,0,0,0,0,0,0,0]]C0951574	cetiedil hydrochloride	[[0,1,0,0,0,0,0,0,0,0,0]]C0009014	clonidine,klofenil,clofenil,n 2 6 dichlorophenyl 4 5 dihydro 1h imidazol 2 amine	[[0,0,0,0,0,0,0,1,0,1,0],[0,0,0,0,0,0,0,0,0,0,1],[0,0,0,0,0,0,0,0,0,0,1],[0,0,0,0,0,0,0,0,0,1,1]]C0951576	cetiedil oxalate	[[0,1,0,0,0,0,0,0,0,0,0]]C2710446	clostridium tetani antigen	[[0,0,0,0,0,0,0,0,0,1,0]]C0885411	heracleum sphondylium preparation	[[0,0,0,0,0,1,0,0,0,0,0]]C0885415	hirudo medicinalis preparation,leech	[[0,0,0,0,0,1,0,0,0,0,0],[0,0,0,0,0,0,1,0,0,0,0]]C0885414	homeopathic preparation hippuric acid hippuricum acidum,hippuric acid	[[0,0,0,0,0,1,0,0,0,0,0],[0,0,0,0,0,0,1,0,0,0,0]]C0885419	homeria collina preparation	[[0,0,0,0,0,1,0,0,0,0,0]]C0301363	5 trimethoxyamphetamine,tma	[[1,0,0,0,0,0,1,0,0,0,0],[1,0,0,0,0,0,1,0,0,0,0]]C2981331	mexafylline,3 3 cyclohexen 1 ylmethyl 1 8 dimethylxanthine	[[0,0,0,0,0,0,0,0,0,1,0],[0,0,0,0,0,0,0,0,0,1,0]]C2585269	oral form thiothixene	[[1,0,0,0,0,0,0,0,0,0,0]]C2698395	binospirone	[[0,0,0,0,0,0,0,0,0,1,0]]C0795767	tenox bha	[[0,1,0,0,0,0,0,0,0,0,0]]C0049397	6 carboxyraffinose	[[0,1,0,0,0,0,0,0,0,0,0]]C0795765	flo 1347,flo1347	[[0,1,0,0,0,0,0,0,0,0,0],[0,1,0,0,0,0,0,0,0,0,0]]C0083928	palustran	[[0,1,0,0,0,0,0,0,0,0,0]]C0795763	fla 731,fla731	[[0,1,0,0,0,0,0,0,0,0,0],[0,1,0,0,0,0,0,0,0,0,0]]C0051017	ahr 5333	[[0,1,0,0,0,0,0,0,0,0,0]]C0049390	6 beta hydroxycortisol,6 11 beta 17 21 tetrahydroxypregn 4 ene 3 20 dione,6beta hydroxycortisol	[[1,1,0,1,0,0,0,0,0,0,0],[0,1,0,0,0,0,0,0,0,0,0],[0,1,0,0,0,0,0,0,0,0,0]]C0795760	isoprinosin,isoprinosina	[[0,0,0,0,0,0,1,0,0,1,0],[0,0,0,0,0,0,0,0,0,1,0]]C0598982	semicid	[[0,0,0,0,0,0,1,0,1,0,0]]C2927961	african pygeum extract stinging nettle extract	[[0,0,0,0,0,0,0,1,0,0,0]]C0598980	gynol ii	[[0,0,0,0,0,0,0,0,1,0,0]]C0059941	exochelins	[[0,1,0,0,0,0,0,0,0,0,0]]C2927964	ascorbic acid bioflavonoids hesperidin rutin	[[0,0,0,0,0,0,0,1,0,0,0]]C2927965	garlic preparation lecithin	[[0,0,0,0,0,0,0,1,0,0,0]]C0795769	embanox	[[0,1,0,0,0,0,0,0,0,0,0]]C0083924	padma 28	[[0,1,0,0,0,0,0,0,0,0,0]]C0383114	hi peptide	[[0,1,0,0,0,0,0,0,0,0,0]]C0796497	recombinant vaccinia df3 muc1 vaccine,rv df3 muc1	[[0,0,0,0,1,0,0,0,0,1,0],[0,0,0,0,1,0,0,0,0,0,0]]C0796491	oxi 104	[[0,0,0,0,1,0,0,0,0,1,0]]C0608444	a 748	[[0,1,0,0,0,0,0,0,0,0,0]]C0964390	10 13a 14 hydroxyantofine n oxide,10 13 hao,10 13a 14 hydroxy antofine n oxide	[[0,1,0,0,0,0,0,0,0,0,0],[0,1,0,0,0,0,0,0,0,0,0],[0,1,0,0,0,0,0,0,0,0,0]]C0964391	10beta 13aalpha 14beta hydroxyantofine n oxide	[[0,1,0,0,0,0,0,0,0,0,0]]C0964392	cis parthenolid 9 one	[[0,1,0,0,0,0,0,0,0,0,0]]C2601139	calcium 124 blood cord	[[0,0,0,1,0,0,0,0,0,0,0]]C0613873	bis 4 biphenylmethyl ether,4 4 oxybis methylene bis 1 1 biphenyl,bis bpme	[[0,1,0,0,0,0,0,0,0,0,0],[0,1,0,0,0,0,0,0,0,0,0],[0,1,0,0,0,0,0,0,0,0,0]]C1710806	kos 953	[[0,0,0,0,1,0,0,0,0,0,0]]C0058093	dihydromuscimol,5 aminomethyl 3 isoxazolidinone	[[0,1,0,0,0,0,0,0,0,0,0],[0,1,0,0,0,0,0,0,0,0,0]]C0613877	3 2 iodophenoxy 1 3 4 dimethoxyphenylethyl amino propan 2 ol,1 2 3 4 dimethoxyphenyl ethyl amino 3 2 iodophenoxy 2 propanol,ipdmpeap	[[0,1,0,0,0,0,0,0,0,0,0],[0,1,0,0,0,0,0,0,0,0,0],[0,1,0,0,0,0,0,0,0,0,0]]C0058091	dihydromevinolin,4a 5 dihydromevinolin,1s 1alpha r 3alpha 4aalpha 7beta 8beta 2s 4s 8abeta 1 2 3 4 4a 7 8 8a octahydro 3 7 dimethyl 8 2 tetrahydro 4 hydroxy 6 oxo 2h pyran 2 yl ethyl 1 naphthalenyl ester 2 methyl butanoic acid	[[0,1,0,0,0,0,0,0,0,0,0],[0,1,0,0,0,0,0,0,0,0,0],[0,1,0,0,0,0,0,0,0,0,0]]C2601131	bumetanide 124 dose	[[0,0,0,1,0,0,0,0,0,0,0]]C0528372	zosuquidar trihydrochloride,2r anti 5 3 4 10 11 difluoromethanodibenzo suber 5 yl piperazin 1 yl 2 hydroxypropoxy quinoline trihydrochloride,trihydrochloride r 4 1ar 6r 10bs 1 2 difluoro 1 1a 6 10b tetrahydrodibenzo a e cyclopropa c cycloheptan 6 yl alpha 5 quinoloyloxy methyl 1 piperazineethanol	[[0,0,0,0,0,0,0,0,0,1,0],[0,1,0,0,0,0,0,0,0,0,0],[0,0,0,0,0,0,0,0,0,1,0]]C0023929	lobeline,2r 2alpha 6alpha s 2 6 2 hydroxy 2 phenylethyl 1 methyl 2 piperidinyl 1 phenyl ethanone,2 6 beta hydroxyphenethyl 1 methyl 2 piperidyl acetophenone,inflatine	[[0,0,0,0,0,0,0,1,0,1,0],[0,0,0,0,0,0,0,0,0,0,1],[0,0,0,0,0,0,0,0,0,1,0],[0,0,0,0,0,0,0,0,0,1,0]]C1640776	eth oxydose	[[0,0,0,0,0,0,0,1,0,0,0]]C0720161	effective strength cough	[[0,0,0,0,0,0,0,1,0,0,0]]C0720160	efasin hd plus	[[0,0,0,0,0,0,0,1,0,0,0]]C0720163	effervescent pain antacid	[[0,0,0,0,0,0,0,1,0,0,0]]C0720162	effer k	[[0,0,0,0,0,0,0,1,0,0,0]]C0646113	wr 7295	[[0,1,0,0,0,0,0,0,0,0,0]]C0720167	efidac 24 chlorpheniramine	[[0,0,0,0,0,0,0,1,0,0,0]]C0720166	efidac	[[0,0,0,0,0,0,1,1,0,0,0]]C0720169	efodine	[[0,0,0,0,0,0,0,1,0,0,0]]C0720168	eflone	[[0,0,0,0,0,0,0,0,0,1,0]]C2369319	promacta	[[0,0,0,0,0,0,0,0,0,1,0]]C0771340	calteridol calcium	[[0,0,1,0,0,0,0,0,0,1,0]]C1640771	simuc hd	[[0,0,0,0,0,0,0,1,0,0,0]]C0530523	ly 314228,ly314228	[[0,1,0,0,0,0,0,0,0,0,0],[0,1,0,0,0,0,0,0,0,0,0]]C0530527	3 2 phenyl 2 carboxyethenyl 4 6 dichloro 1h indole 2 carboxylic acid	[[0,1,0,0,0,0,0,0,0,0,0]]C0530525	ly 320954,ly320954	[[0,1,0,0,0,0,0,0,0,0,0],[0,1,0,0,0,0,0,0,0,0,0]]C0308151	xerac ac	[[0,0,0,0,0,0,1,1,0,0,0]]C1448437	antithrombin iii alpha,grifols brand of antithrombin,antithrombin iiialpha	[[0,1,0,0,0,0,0,0,0,0,0],[0,1,0,0,0,0,0,0,0,0,0],[0,1,0,0,0,0,0,0,0,0,0]]C0969016	bonannione a	[[0,1,0,0,0,0,0,0,0,0,0]]C0969017	isoaurostatin	[[0,1,0,0,0,0,0,0,0,0,0]]C1873951	acetaminophen butalbital codeine	[[0,0,0,0,0,0,0,1,0,0,1]]C0071078	pinacolyl s 2 dimethylaminoethyl methylphosphonothioate	[[0,1,0,0,0,0,0,0,0,0,0]]C0071079	pinane,2 6 6 trimethyl bicyclo 3 1 1 heptane	[[0,1,0,0,0,0,0,0,0,0,0],[0,1,0,0,0,0,0,0,0,0,0]]C1518582	oncovax id il 2 vaccine,t96 0057 liposomal lymphoma igid,liposomal lymphoma idiotype ig plus interleukin 2	[[0,0,0,0,0,0,0,0,0,1,0],[0,0,0,0,0,0,0,0,0,1,0],[0,0,0,0,0,0,0,0,0,1,0]]C0071074	pinacidil,n cyano n 4 pyridinyl n 1 2 2 trimethylpropylmonohydrate guanidine	[[0,0,0,0,0,0,0,1,1,1,0],[0,0,0,0,0,0,0,0,0,0,1]]C2585880	oropharyngeal form sodium perborate	[[1,0,0,0,0,0,0,0,0,0,0]]C0071071	pimobendan,4 5 dihydro 6 2 4 methoxyphenyl 1h benzimidazole 5 yl 5 methyl 3 2h pyridazinone	[[0,1,0,0,0,0,0,1,0,1,0],[0,1,0,0,0,0,0,0,0,0,0]]C1620249	1 4 hydroxybenzyl 4 7 dimethoxy 9 10 dihydrophenanthrene 2 ol,1 4 hb ddp	[[0,1,0,0,0,0,0,0,0,0,0],[0,1,0,0,0,0,0,0,0,0,0]]C0644768	3 tert butylamino 1 3 4 dihydroxy 6 fluorophenoxy 2 propanol	[[0,1,0,0,0,0,0,0,0,0,0]]C2080580	acetaminophen chlorpheniramine guaifenesin phenylpropanolamine	[[0,0,0,0,0,0,0,1,0,0,0]]C2698391	bimethoxycaine lactate,isocaine lactate,bis b o methoxyphenyl isopropyl amine lactate,compd with lactic acid o o dimethoxy alpha alpha dimethyl diphenethylamine	[[0,0,0,0,0,0,0,0,0,1,0],[0,0,0,0,0,0,0,0,0,1,0],[0,0,0,0,0,0,0,0,0,1,0],[0,0,0,0,0,0,0,0,0,1,0]]C0120736	halysin	[[0,1,0,0,0,0,0,0,0,0,0]]C0653059	5 9 dimecde	[[0,1,0,0,0,0,0,0,0,0,0]]C0644760	ethyl 1 benzyl 3 hydroxy 2 5h oxopyrrole 4 carboxylate,1 ebpc	[[0,1,0,0,0,0,0,0,0,0,0],[0,1,0,0,0,0,0,0,0,0,0]]C0120735	haloxazolam,10 bromo 11b 2 fluorophenyl 2 3 7 11b tetrahydrooxazolo 3 2 d 1 4 benzodiazepin 6 5h one	[[0,1,0,0,0,0,0,0,0,1,0],[0,1,0,0,0,0,0,0,0,0,0]]C0377918	photosens	[[0,1,0,0,0,0,0,0,0,0,0]]C0377919	antibiotic y	[[0,1,0,0,0,0,0,0,0,0,0]]C2352993	microbisporicin	[[0,1,0,0,0,0,0,0,0,0,0]]C1589759	otomycet hc	[[0,0,0,0,0,0,0,1,0,0,0]]C1506801	1 trifluoromethyl 1 2 2 triphenylethylene,1 1 tfmte	[[0,1,0,0,0,0,0,0,0,0,0],[0,1,0,0,0,0,0,0,0,0,0]]C1506807	morphine 3 octylglucuronamide,m3goam	[[0,1,0,0,0,0,0,0,0,0,0],[0,1,0,0,0,0,0,0,0,0,0]]C3253346	3 4 hydroxyphenyl 4 2 morpholinoethoxy furan 2 5h one,hpme furanone	[[0,1,0,0,0,0,0,0,0,0,0],[0,1,0,0,0,0,0,0,0,0,0]]C2981337	histidine monohydrochloride,histidine hydrochloride,l histidine monohydrochloride,l histidine hydrochloride,histidine monohydrochloride anhydrous	[[0,0,1,0,0,0,0,0,0,1,0],[0,0,0,0,0,0,0,0,0,1,0],[0,0,0,0,0,0,0,0,0,1,0],[0,0,0,0,0,0,0,0,0,1,0],[0,0,0,0,0,0,0,0,0,1,0]]C3265711	2 carbethoxyethyl diethoxy methyl silane	[[0,0,1,0,0,0,0,0,0,0,0]]C0982346	poloxamer 185	[[0,0,0,0,0,0,0,0,0,0,1]]C0946026	dextromethorphan levorphanol	[[0,0,0,1,0,0,0,0,0,0,0]]C0673316	nte 122,nte122	[[0,1,0,0,0,0,0,0,0,0,0],[0,1,0,0,0,0,0,0,0,0,0]]C0096130	4 chlorodiazepam	[[0,1,0,0,0,0,0,0,0,0,0]]C0673313	1 4 bis 1 cyclohexyl 3 4 dimethylaminophenyl ureido methyl cyclohexane	[[0,1,0,0,0,0,0,0,0,0,0]]C0172169	u 87201	[[0,1,0,0,0,0,0,0,0,0,0]]C0982342	pine compound white	[[0,0,0,0,0,0,0,0,0,0,1]]C0108362	trans isomer 10 11 dihydro 10 11 dihydroxy 5h dibenzazepine 5 carboxamide,carbamazepine 10 11 transdiol,10 11 dihydro 10 11 transdihydroxycarbamazepine	[[0,1,0,0,0,0,0,0,0,0,0],[0,1,0,0,0,0,0,0,0,0,0],[0,1,0,0,0,0,0,0,0,0,0]]C1881493	mscv mgmt p140k vaccine	[[0,0,0,0,0,0,0,0,0,1,0]]C0731845	hemodialysis concentrate,haemodialysis concentrate	[[1,0,0,0,0,0,0,0,0,0,0],[1,0,0,0,0,0,0,0,0,0,0]]C0731846	continuous hemodialysis solution,continuous haemodialysis solution	[[1,0,0,0,0,0,0,0,0,0,0],[1,0,0,0,0,0,0,0,0,0,0]]C0731847	hemodialysis solution concentrate,haemodialysis solution concentrate,hemodialysis liquid concentrate,haemodialysis liquid concentrate	[[1,0,0,0,0,0,0,0,0,0,0],[1,0,0,0,0,0,0,0,0,0,0],[1,0,0,0,0,0,0,0,0,0,0],[1,0,0,0,0,0,0,0,0,0,0]]C0731848	hemodialysis dry concentrate,haemodialysis dry concentrate	[[1,0,0,0,0,0,0,0,0,0,0],[1,0,0,0,0,0,0,0,0,0,0]]C0731849	acid hemodialysis liquid concentrate,acid haemodialysis liquid concentrate	[[1,0,0,0,0,0,0,0,0,0,0],[1,0,0,0,0,0,0,0,0,0,0]]C0916752	r isomer cyclophosphamide	[[0,1,0,0,0,0,0,0,0,0,0]]C0552298	6 beta naltrexone	[[0,0,0,1,0,0,0,0,0,0,0]]C2947573	chionanthus virginicus bark	[[0,0,1,0,0,0,0,0,0,0,0]]C1577475	cartia	[[0,0,0,0,0,0,0,1,0,0,0]]C2701463	red oak pollen extract,quercus rubra pollen	[[0,0,0,0,0,0,0,1,0,0,0],[0,0,1,0,0,0,0,0,0,1,0]]C2932170	nsc746366	[[0,1,0,0,0,0,0,0,0,0,0]]C0090911	2 alpha carboxy 4 beta 5 carboxy 1 methyl 1 3 beta hexadienyl 3 pyrrolidineacetic acid	[[0,1,0,0,0,0,0,0,0,0,0]]C0595009	tenkicin	[[0,0,0,0,0,0,0,1,0,0,0]]C0068164	n myristoyl lysyl arginyl threonyl leucyl arginine	[[0,1,0,0,0,0,0,0,0,0,0]]C0251606	chaetomellic acid b,z z 2 7 hexadecenyl 3 methyl 2 butenedioic acid	[[0,1,0,0,0,0,0,0,0,0,0],[0,1,0,0,0,0,0,0,0,0,0]]C0024321	lynestrenol,linestrenol,lynoestrenol,ethinylestrenol,linesterol,17alpha 19 norpregn 4 en 20 yn 17 ol	[[0,0,0,0,0,0,1,1,0,1,0],[0,0,0,0,0,0,1,0,0,0,1],[0,0,0,0,0,0,0,0,0,0,1],[0,0,0,0,0,0,0,0,0,0,1],[0,0,0,0,0,0,0,0,0,0,1],[0,0,0,0,0,0,0,0,0,0,1]]C0068161	n monoacetylcystine,n acetylcystine	[[0,1,0,0,0,0,0,0,0,0,0],[0,1,0,0,0,0,1,0,0,0,0]]C0024328	lypressin,lysine vasopressin,lysyl vasopressin,8 l lysine vasopressin preparation,lypressin preparation,cyclic 1 6 disulfide cys tyr phe gln asn cys pro lys gly nh2,8 lysine vasopressin,lys vasopressin	[[0,0,0,0,0,0,0,1,0,1,0],[0,0,0,0,0,0,0,0,1,0,1],[0,1,0,0,0,0,0,0,0,0,1],[1,0,0,0,0,0,0,0,0,0,0],[1,0,0,0,0,0,0,0,0,0,0],[0,0,0,0,0,0,0,0,0,0,1],[0,1,0,0,0,0,0,0,0,0,1],[0,1,0,0,0,0,0,0,0,0,1]]C2947576	dl dimyristoylphosphatidylglycerol	[[0,0,1,0,0,0,0,0,0,0,0]]C0068168	n n propyl 3 3 hydroxyphenyl piperidine,3 3 hydroxyphenyl n n propylpiperidine,3 ppp,n n propyl 3 n hydroxyphenyl piperidine,3 1 propyl 3 piperidinyl phenol,preclamol	[[0,1,0,0,0,0,0,0,0,0,0],[0,1,0,0,0,0,0,0,0,0,0],[0,1,0,0,0,0,0,0,0,0,0],[0,1,0,0,0,0,0,0,0,0,0],[0,1,0,0,0,0,0,0,0,0,0],[0,1,0,0,0,0,0,0,0,1,0]]C0257294	mdl 102253,mdl 102 253	[[0,1,0,0,0,0,0,0,0,0,0],[0,1,0,0,0,0,0,0,0,0,0]]C2604254	yueju wan,yj compound	[[0,1,0,0,0,0,0,0,0,0,0],[0,1,0,0,0,0,0,0,0,0,0]]C2962842	evict	[[0,0,0,0,0,0,0,1,0,0,0]]C2962848	skintx	[[0,0,0,0,0,0,0,1,0,0,0]]C2955215	histex sr tablet	[[0,0,0,0,0,0,0,1,0,0,0]]C3254335	n hydroxy 4 3 3 methoxy 4 thiophen 2 ylmethyl phenyl propanamido benzamide,n hmtmppb cpd	[[0,1,0,0,0,0,0,0,0,0,0],[0,1,0,0,0,0,0,0,0,0,0]]C0593690	digenac xl	[[0,0,0,0,0,0,0,1,0,0,0]]C0593697	zemtard xl	[[0,0,0,0,0,0,0,1,0,0,0]]C0361823	fourth generation cephalosporin,cephalosporin 4th generation	[[1,0,0,0,0,0,1,0,0,0,0],[0,0,0,0,0,0,0,0,0,0,1]]C3181614	2 4 4 methylbenzyl 1 4 diazepan 1 yl n 4 5 dihydro 1 methyl 1 2 4 triazolo 4 3 a quinolin 7 yl acetamide,mddmtq acetamide	[[0,1,0,0,0,0,0,0,0,0,0],[0,1,0,0,0,0,0,0,0,0,0]]C1615721	atuss nx	[[0,0,0,0,0,0,0,1,0,0,0]]C1660030	silver sulfate	[[0,0,0,0,0,0,0,0,0,0,1]]C1691508	allertan	[[0,0,0,0,0,0,0,1,0,0,0]]C2241866	phenyl t	[[0,0,0,0,0,0,0,1,0,0,0]]C2241867	poly tussin ac	[[0,0,0,0,0,0,0,1,0,0,0]]C2241868	poly tussin dhc	[[0,0,0,0,0,0,0,1,0,0,0]]C1615728	prednisone intensol	[[0,0,0,0,0,0,0,1,0,0,0]]C0631445	6 fluorophenylephrine,6 fpe	[[0,1,0,0,0,0,0,0,0,0,0],[0,1,0,0,0,0,0,0,0,0,0]]C0631443	4 fluorophenylephrine,4 fpe	[[0,1,0,0,0,0,0,0,0,0,0],[0,1,0,0,0,0,0,0,0,0,0]]C0631441	2 fluorophenylephrine,2 fpe	[[0,1,0,0,0,0,0,0,0,0,0],[0,1,0,0,0,0,0,0,0,0,0]]C0305058	human tetanus immune globulin,human tetanus antitoxin,tetanus immune globulin,tetanus immunoglobulin,anti tetanus immunoglobulin,tig	[[0,0,0,0,0,0,0,1,0,0,0],[1,0,0,0,0,0,0,0,0,0,0],[0,0,0,0,0,0,1,0,0,0,1],[1,0,0,0,0,0,1,0,0,0,0],[1,0,0,0,0,0,0,0,0,0,0],[0,0,0,0,0,0,1,0,0,0,0]]C0305059	diphtheria and tetanus toxoid adsorbed,dt toxoid	[[1,0,0,0,0,0,0,0,0,0,0],[1,0,0,0,0,0,0,0,0,0,0]]C1874319	antipyrine hydrocortisone neomycin polymyxin b	[[0,0,0,0,0,0,0,1,0,0,0]]C0873170	clorprenaline hydrochloride,isoprophenamine hydrochloride	[[0,1,0,0,0,0,0,0,0,1,0],[0,0,0,0,0,0,0,0,0,1,0]]C0873176	oxiniacic acid	[[0,0,0,0,0,0,0,0,0,1,0]]C0041399	turisynchron	[[0,1,0,0,0,0,0,0,0,0,0]]C0305050	scorpion antivenin	[[1,0,0,0,0,0,0,0,0,0,0]]C1874312	antiparasitics other	[[0,0,0,0,0,0,0,0,0,0,1]]C0305052	gamma globulin serum,immune serum globulin,ig,isg	[[1,0,0,0,0,0,1,0,0,0,0],[0,0,0,1,0,0,1,0,0,0,0],[1,0,0,0,0,0,0,0,0,0,0],[1,0,0,0,0,0,1,0,0,0,0]]C1874310	antineoplastic radiopharmaceuticals	[[0,0,0,0,0,0,0,0,0,0,1]]C1874317	antipsychotics other	[[0,0,0,0,0,0,0,0,0,0,1]]C0305055	lymphocyte immune globulin,lymphocyte immunoglobulin	[[0,0,0,0,0,0,1,1,0,0,0],[1,0,0,0,0,0,0,0,0,0,0]]C1874315	antipsoriatics systemic	[[0,0,0,0,0,0,0,0,0,0,1]]C0305057	equine diphtheria antitoxin	[[0,0,0,0,0,0,0,1,0,0,0]]C0772360	spike lavender oil	[[0,0,1,0,0,0,0,1,0,0,0]]C0772361	white mulberry preparation,white mulberry	[[0,0,0,0,0,1,0,0,0,0,0],[0,0,1,0,0,0,0,0,0,0,0]]C0772362	halometasone monohydrate	[[0,1,0,0,0,0,0,1,0,0,0]]C0772364	loteprednol	[[0,0,0,0,0,0,1,1,0,1,0]]C0912839	4 ethyl 4 hydroxy 2 4 tolyl 4h 5 6 dihydro 1 3 selenazine,4 ethyl 4 hydroxy 2 p tolyl 4h 5 6 dihydro 1 3 selenazine,ts 2 cpd	[[0,1,0,0,0,0,0,0,0,0,0],[0,1,0,0,0,0,0,0,0,0,0],[0,1,0,0,0,0,0,0,0,0,0]]C0677768	monoclonal antibody di dga rfb4,di dga rfb4,moab di dga rfb4,di dga rfb4 immunotoxin	[[0,0,0,0,1,0,0,0,0,1,0],[0,0,0,0,1,0,0,0,0,1,0],[0,0,0,0,1,0,0,0,0,0,0],[0,0,0,0,0,0,0,0,0,1,0]]C0939713	oxeze	[[0,0,0,0,0,0,1,0,0,0,0]]C0939710	omedia	[[0,0,0,0,0,0,0,1,0,0,0]]C0939711	orapred	[[0,0,0,0,0,0,1,1,0,0,0]]C0939716	pemolert	[[0,0,0,0,0,0,0,1,0,0,0]]C0116905	ethylheptazine	[[0,1,0,0,0,0,0,0,0,0,0]]C0939714	pancrelipase sr	[[0,0,0,0,0,0,0,1,0,0,0]]C0939715	pangestyme mt 16	[[0,0,0,0,0,0,0,1,0,0,0]]C2700331	zinc sulfate anhydrous	[[0,0,0,0,0,0,0,0,0,1,0]]C0939718	poly histine d sr	[[0,0,0,0,0,0,0,1,0,0,0]]C0359665	hypromellose phenylephrine	[[1,0,0,0,0,0,0,1,0,0,0]]C0637849	3 acetylpanaxydol	[[0,1,0,0,0,0,0,0,0,0,0]]C0677765	monoclonal antibody 11d10,11d10,moab 11d10	[[0,0,0,0,1,0,0,0,0,1,0],[0,0,0,0,0,0,0,0,0,1,0],[0,0,0,0,1,0,0,0,0,1,0]]C0359660	benoxinate hcl fluorescein na,oxybuprocaine hcl fluorescein na	[[1,0,0,0,0,0,0,0,0,0,0],[1,0,0,0,0,0,0,0,0,0,0]]C2700337	zosuquidar,r 4 1ar 6r 10bs 1 2 difluoro 1 1a 6 10b tetrahydrodibenzo a e cyclopropa c cycloheptan 6 yl alpha 5 quinoloyloxy methyl 1 piperazineethanol	[[0,0,0,0,0,0,0,0,0,1,0],[0,0,0,0,0,0,0,0,0,1,0]]C1882392	piraxelate	[[0,0,0,0,0,0,0,0,0,1,0]]C1136543	125 i lin vasopressin	[[0,1,0,0,0,0,0,0,0,0,0]]C1882390	pipradimadol	[[0,0,0,0,0,0,0,0,0,1,0]]C1657867	folbee	[[0,0,0,0,0,0,0,1,0,0,0]]C1882396	piriprost potassium	[[0,0,0,0,0,0,0,0,0,1,0]]C1882397	piroxicam olamine,piroxicam monoethanolamine salt,piroxicam ethanolamine salt,compound with 2 aminoethanol 1 1 dioxide 4 hydroxy 2 methyl n 2 pyridinyl 2h 1 2 benzothiazine 3 carboxamide	[[0,0,0,0,0,0,0,0,0,1,0],[0,0,0,0,0,0,0,0,0,1,0],[0,0,0,0,0,0,0,0,0,1,0],[0,0,0,0,0,0,0,0,0,1,0]]C1882394	pirepolol	[[0,0,0,0,0,0,0,0,0,1,0]]C0950983	monohydrochloride agr 307	[[0,1,0,0,0,0,0,0,0,0,0]]C0037209	sisomicin,sisomycin,sissomicin,4 5 dehydrogentamicin,rickamicin,o 3 deoxy 4 c methyl 3 methylamino beta l arabinopyranosyl 1 6 o 2 6 diamino 2 3 4 6 tetradeoxy alpha d glycero hex 4 enopyranosyl 1 4 2 deoxy d streptamine,sizomycin	[[0,0,0,0,0,0,1,1,0,1,0],[0,0,0,0,0,0,1,0,0,0,1],[0,0,0,0,0,0,0,0,0,0,1],[0,1,0,0,0,0,0,0,0,0,1],[0,0,0,0,0,0,0,0,0,0,1],[0,0,0,0,0,0,0,0,0,0,1],[0,0,0,0,0,0,0,0,0,0,1]]C0979682	potassium nitrate granules	[[0,0,0,0,0,0,0,0,0,0,1]]C1882399	pirtenidine hydrochloride	[[0,0,0,0,0,0,0,0,0,1,0]]C0025051	medazepam,7 chloro 2 3 dihydro 1 methyl 5 phenyl 1h 1 4 benzodiazepine,medazapam	[[0,0,0,0,0,0,1,1,0,1,0],[0,0,0,0,0,0,0,0,0,0,1],[1,0,0,0,0,0,1,0,0,0,0]]C0950987	sodium salt aristolochic acid i	[[0,1,0,0,0,0,0,0,0,0,0]]C1328072	recombinant vaccinia muc 1 vaccine,rv muc 1,vaccinia muc 1 vaccine,recombinant vaccinia muc 1,rv muc 1 vaccine,recombinant vaccina v muc 1 vaccine	[[0,0,0,0,1,0,0,0,0,1,0],[0,0,0,0,1,0,0,0,0,0,0],[0,0,0,0,1,0,0,0,0,0,0],[0,0,0,0,1,0,0,0,0,0,0],[0,0,0,0,0,0,0,0,0,1,0],[0,0,0,0,1,0,0,0,0,0,0]]C0022585	keratolytic agents,desquamating agents,skin peeling agents,keratolytic	[[1,1,0,0,0,0,1,0,0,0,0],[0,1,0,0,0,0,0,0,0,0,0],[0,1,0,0,0,0,0,0,0,0,0],[1,0,0,0,0,0,1,0,0,0,0]]C1328071	methoxy polyethylene glycol epoetin beta,methoxypolyethylene glycol epoetin beta,pegzerepoetin alfa	[[1,0,0,0,1,0,0,0,0,1,0],[0,0,0,0,0,0,0,0,0,1,0],[0,0,0,0,0,0,0,0,0,1,0]]C0954330	s methylisothiopseudouronium sulfate	[[0,1,0,0,0,0,0,0,0,0,0]]C0968014	3 2 2 dimethylvaleroyl morphine,3 dmvm cpd	[[0,1,0,0,0,0,0,0,0,0,0],[0,1,0,0,0,0,0,0,0,0,0]]C3181610	feruloylacetone	[[0,1,0,0,0,0,0,0,0,0,0]]C0140309	r isomer acetorphan,retorphan	[[0,1,0,0,0,0,0,0,0,0,0],[0,1,0,0,0,0,0,0,0,0,0]]C1710029	atrochin	[[0,0,0,0,0,0,0,0,0,1,0]]C1815177	koate hp	[[0,0,0,0,0,0,0,1,0,0,0]]C0619516	carbobenzoxyvalyl glycyl arginine 4 nitroanilide,n phenylmethoxy carbonyl l valylglycyl n 4 nitrophenyl l argininamide,carbobenzoxy valyl glycyl arginine 4 nitroanilide,cbz val gly arg p na	[[0,1,0,0,0,0,0,0,0,0,0],[0,1,0,0,0,0,0,0,0,0,0],[0,1,0,0,0,0,0,0,0,0,0],[0,1,0,0,0,0,0,0,0,0,0]]C0536146	7 chloro 5 cis 3 5 dimethylpiperazine carbonyl imidazo 1 5a quinoline 3 carboxylate	[[0,1,0,0,0,0,0,0,0,0,0]]C0536147	u 101017,pnu 101017	[[0,1,0,0,0,0,0,0,0,0,0],[0,1,0,0,0,0,0,0,0,0,0]]C2364841	endal cd	[[0,0,0,0,0,0,0,1,0,0,0]]C3256470	citric acid solution	[[0,0,1,0,0,0,0,0,0,0,0]]C1622269	11b tetrahydro indeno 2 1 c chromen 10 yl ester acetic acid 3 6a 9 triacetoxy 6 6a 7	[[0,1,0,0,0,0,0,0,0,0,0]]C0648326	6 cyclohexyl 1 3 8 trichlorodibenzofuran,6 cyclohexyl 1 3 8 tricdf	[[0,1,0,0,0,0,0,0,0,0,0],[0,1,0,0,0,0,0,0,0,0,0]]C0648327	atramycin b,s 8 6 deoxy alpha l mannopyranosyl oxy 3 4 dihydro 3 methyl benz a anthracene 1 7 12 2h trione	[[0,1,0,0,0,0,0,0,0,0,0],[0,1,0,0,0,0,0,0,0,0,0]]C0648324	6 t butyl 1 3 8 trichlorodibenzofuran,6 t butyl 1 3 8 tricdf	[[0,1,0,0,0,0,0,0,0,0,0],[0,1,0,0,0,0,0,0,0,0,0]]C3256371	morus nigra fruit	[[0,0,1,0,0,0,0,0,0,0,0]]C0648322	atramycin a,s 8 6 deoxy alpha l mannopyranosyl oxy 3 4 dihydro 6 hydroxy 3 methyl benz a anthracene 1 7 12 2h trione	[[0,1,0,0,0,0,0,0,0,0,0],[0,1,0,0,0,0,0,0,0,0,0]]C0648320	naphthgeranine c,3s 3alpha 4aalpha 12balpha 3 4a 5 12b tetrahydro 3 8 10 trihydroxy 2 hydroxymethyl 5 5 dimethyl 4h benzo d naphtho 2 3 b pyran 7 12 dione	[[0,1,0,0,0,0,0,0,0,0,0],[0,1,0,0,0,0,0,0,0,0,0]]C0648321	naphthgeranine d,3r 3alpha 4beta 4aalpha 12balpha 3 4b 5 12b tetrahydro 3 4 8 10 tetrahydroxy 2 hydroxymethyl 5 5 dimethyl 4h benzo d naphtho 2 3 b pyran 7 12 dione	[[0,1,0,0,0,0,0,0,0,0,0],[0,1,0,0,0,0,0,0,0,0,0]]C0591691	konsyl	[[0,0,0,0,0,0,1,1,0,0,0]]C0630712	trans 3h 9ah 3 4 chloro benzoylquinolizidine	[[0,1,0,0,0,0,0,0,0,0,0]]C0591692	kwell	[[0,0,0,0,0,0,0,1,0,0,0]]C0630715	8 benzyl 8 azabicyclo 3 2 1 octane 3 propionanilide,8 babop	[[0,1,0,0,0,0,0,0,0,0,0],[0,1,0,0,0,0,0,0,0,0,0]]C0537899	nsc 624151	[[0,1,0,0,0,0,0,0,0,0,0]]C0630717	8 2 phenethyl 8 azabicyclo 3 2 1 octane 3 4 methylpropionanilide,8 peabomp	[[0,1,0,0,0,0,0,0,0,0,0],[0,1,0,0,0,0,0,0,0,0,0]]C0630719	8 n 2 ethylmorpholinyl 8 azabicyclo 3 2 1 octane 3 propionanilide,8 emabop	[[0,1,0,0,0,0,0,0,0,0,0],[0,1,0,0,0,0,0,0,0,0,0]]C0537895	mk 0991	[[0,1,0,0,0,0,0,0,0,0,0]]C0537894	caspofungin	[[0,0,0,0,0,0,0,1,0,1,1]]C0537893	er 35786	[[0,1,0,0,0,0,0,0,0,0,0]]C0537892	6 1 hydroxyethyl 1 methyl 2 pyrrolidin 3 yl hydroxymethyl pyrrolidin 4 ylthio 1 carbapen 2 em 3 carboxylic acid hydrochloride	[[0,1,0,0,0,0,0,0,0,0,0]]C0537891	srr sb3	[[0,1,0,0,0,0,0,0,0,0,0]]C0075007	spirapril,8s 7 r r 8r 7 2 1 ethoxycarbonyl 3 phenylpropyl amino 1 oxopropyl 1 4 dithia 7 azaspiro 4 4 nonane 8 carboxylic acid	[[0,0,0,0,0,0,0,1,0,1,0],[0,1,0,0,0,0,0,0,0,0,0]]C0254725	cgp 55398,oc 6 12 bis 3beta cholest 4 en 3 yl oxy diphenylsilanolato 29h 31h phthalocyaninato 2 n29 n30 n31 n32 germanium	[[0,1,0,0,0,0,0,0,0,0,0],[0,1,0,0,0,0,0,0,0,0,0]]C0254724	germanium iv phthalocyanine,bis diphenylcholesteryloxysiloxy germanium phthalocyanine	[[0,1,0,0,0,0,0,0,0,0,0],[0,1,0,0,0,0,0,0,0,0,0]]C3253101	4 hydroxychalcone	[[0,1,0,0,0,0,0,0,0,0,0]]C2983928	octaverine	[[0,0,0,0,0,0,0,0,0,1,0]]C2983929	stercuronium iodide	[[0,0,0,0,0,0,0,0,0,1,0]]C2983926	deutolperisone	[[0,0,0,0,0,0,0,0,0,1,0]]C2983927	nelezaprine	[[0,0,0,0,0,0,0,0,0,1,0]]C2983924	arbaclofen,r 4 amino 3 4 chlorophenyl butanoic acid,d baclofen,r baclofen	[[0,0,0,0,0,0,0,0,0,1,0],[0,0,0,0,0,0,0,0,0,1,0],[0,0,0,0,0,0,0,0,0,1,0],[0,0,0,0,0,0,0,0,0,1,0]]C2983925	baclofen s,s 4 amino 3 4 chlorophenyl butanoic acid	[[0,0,0,0,0,0,0,0,0,1,0],[0,0,0,0,0,0,0,0,0,1,0]]C2983922	r baclofen hydrochloride,4 amino 3 4 chlorophenyl butanoic acid hydrochloride r	[[0,0,0,0,0,0,0,0,0,1,0],[0,0,0,0,0,0,0,0,0,1,0]]C0032188	platelet inhibitors	[[0,1,0,0,0,0,1,0,0,0,0]]C2983920	omonasteine	[[0,0,0,0,0,0,0,0,0,1,0]]C2983921	baclofen hydrochloride,4 amino 3 4 chlorophenyl butanoic acid hydrochloride	[[0,0,0,0,0,0,0,0,0,1,0],[0,0,0,0,0,0,0,0,0,1,0]]C0719921	dimetapp allergy liquigel	[[0,0,0,0,0,0,0,1,0,0,0]]C1564178	strepto fatol,fatol brand of streptomycin sulfate	[[0,1,0,0,0,0,0,0,0,0,0],[0,1,0,0,0,0,0,0,0,0,0]]C0719923	dimetapp cold cough	[[0,0,0,0,0,0,1,1,0,0,0]]C0719922	dimetapp allergy sinus	[[0,0,0,0,0,0,0,1,0,0,0]]C0719925	dimetapp cold and cough liquigel	[[0,0,0,0,0,0,0,1,0,0,0]]C0719924	dimetapp cold and allergy	[[0,0,0,0,0,0,0,1,0,0,0]]C0719927	dimetapp cold and flu	[[0,0,0,0,0,0,0,1,0,0,0]]C0719926	dimetapp cold and fever	[[0,0,0,0,0,0,0,1,0,0,0]]C0719929	dimetapp decongestant	[[0,0,0,0,0,0,0,1,0,0,0]]C0719928	dimetapp dm	[[0,0,0,0,0,0,0,1,0,0,0]]C1564173	spirogamma,worwag brand of spironolactone	[[0,1,0,0,0,0,0,0,0,0,0],[0,1,0,0,0,0,0,0,0,0,0]]C1564175	estreptomicina cepa,cepa brand of streptomycin sulfate	[[0,1,0,0,0,0,0,0,0,0,0],[0,1,0,0,0,0,0,0,0,0,0]]C1564174	spironone,dexo brand of spironolactone	[[0,1,0,0,0,0,0,0,0,0,0],[0,1,0,0,0,0,0,0,0,0,0]]C1564177	estreptomicina normon,normon brand of streptomycin sulfate	[[0,1,0,0,0,0,0,0,0,0,0],[0,1,0,0,0,0,0,0,0,0,0]]C1564176	estreptomicina clariana,clariana brand of streptomycin sulfate	[[0,1,0,0,0,0,0,0,0,0,0],[0,1,0,0,0,0,0,0,0,0,0]]C2698338	becanthone hydrochloride,1 2 ethyl 2 hydroxy 2 methylpropyl amino ethyl amino 4 methylthioxanthen 9 one monohydrochloride	[[0,0,0,0,0,0,0,0,0,1,0],[0,0,0,0,0,0,0,0,0,1,0]]C0064871	levodopa methyl ester,l dopa methyl ester,methyl l dopa,melevodopa	[[0,1,0,0,0,0,0,0,0,0,0],[0,1,0,0,0,0,0,0,0,0,0],[0,1,0,0,0,0,0,0,0,0,0],[0,1,0,0,0,0,0,0,0,1,0]]C0048250	4 dimethylaminophenol,4 dimethylamino phenol	[[0,0,0,0,0,0,0,1,0,0,0],[0,1,0,0,0,0,0,0,0,0,0]]C0048251	4 dimethylaminophenylazophenyl 4 maleimide,dabmi	[[0,1,0,0,0,0,0,0,0,0,0],[0,1,0,0,0,0,0,0,0,0,0]]C0664087	3 carbethoxyangelicin	[[0,1,0,0,0,0,0,0,0,0,0]]C0048253	4 dimethylaminostilbene,trans 4 dimethylaminostilbene	[[0,1,0,0,0,0,0,0,0,0,0],[0,1,0,0,0,0,0,0,0,0,0]]C2698336	becampanel,7 nitro 2 3 dioxo 1 2 3 4 tetrahydroquinoxalin 5 yl methylamino methylphosphonic acid	[[0,0,0,0,0,0,0,0,0,1,0],[0,0,0,0,0,0,0,0,0,1,0]]C0048255	4 diphenylacetoxy 1 1 dimethylpiperidinium,4 damp,iodide 4 diphenylacetyl oxy 1 1 dimethyl piperidinium,1 dimethyl 4 diphenylacetoxypiperidinium	[[0,1,0,0,0,0,0,0,0,0,0],[0,1,0,0,0,0,0,0,0,0,0],[0,1,0,0,0,0,0,0,0,0,0],[0,1,0,0,0,0,0,0,0,0,0]]C0664082	8 fluoro 5 11 dihydro 11 1 methyl 4 piperidylidene benz b oxepino 4 3 b pyridine	[[0,1,0,0,0,0,0,0,0,0,0]]C0955066	4 4 diaminodibenzyl monohydrochloride	[[0,1,0,0,0,0,0,0,0,0,0]]C0955067	tetrafluoroborate 1 4 hydroxymethyl benzenediazonium ion	[[0,1,0,0,0,0,0,0,0,0,0]]C0955064	isomer isoguvacine oxide	[[0,1,0,0,0,0,0,0,0,0,0]]C0955065	tetrasodium salt phenolphthalexon	[[0,1,0,0,0,0,0,0,0,0,0]]C1449421	bisolvon nac,boehringer ingelheim brand of acetylcysteine	[[0,1,0,0,0,0,0,0,0,0,0],[0,1,0,0,0,0,0,0,0,0,0]]C1449420	actylcystine gnr,gnr pharma brand of acetylcysteine,acetylcysteine gnr	[[0,0,0,0,0,1,0,0,0,0,0],[0,1,0,0,0,0,0,0,0,0,0],[0,1,0,0,0,0,0,0,0,0,0]]C1449423	broncoclar,oberlin brand of acetylcysteine	[[0,1,0,0,0,0,0,0,0,0,0],[0,1,0,0,0,0,0,0,0,0,0]]C0955061	7s cis isomer 4 demethoxy 11 deoxyadriamycin hydrochloride	[[0,1,0,0,0,0,0,0,0,0,0]]C1992495	methyl isobutyl ketone 124 bld ser plas	[[0,0,0,1,0,0,0,0,0,0,0]]C0955068	sodium salt 1 4 carboxyphenyl 3 3 dimethyltriazene	[[0,1,0,0,0,0,0,0,0,0,0]]C0537239	glycyl valyl glutaminyl seryl leucyl lysyl arginyl arginyl arginyl cysteinyl phenylalanine,adenovirus pvic cofactor,adenovirus pvi ct protein,adenovirus gvqslkrrrcf protein	[[0,1,0,0,0,0,0,0,0,0,0],[0,1,0,0,0,0,0,0,0,0,0],[0,1,0,0,0,0,0,0,0,0,0],[0,1,0,0,0,0,0,0,0,0,0]]C1717550	alpha tocopherol beta gamma tocopherol	[[0,0,0,1,0,0,0,0,0,0,0]]C2702377	salmon allergenic extract,salmon	[[0,0,0,0,0,0,0,1,0,0,0],[0,0,1,0,0,0,0,0,0,0,0]]C0733439	chloridin	[[0,1,0,0,0,0,0,0,0,0,0]]C2702372	potato allergenic extract,potato	[[0,0,0,0,0,0,0,1,0,0,0],[0,0,1,0,0,0,0,0,0,0,0]]C2702373	poultry allergenic extract,poultry	[[0,0,0,0,0,1,0,0,0,0,0],[0,0,1,0,0,0,0,0,0,0,0]]C2702370	lobster allergenic extract,lobster	[[0,0,0,0,0,0,0,1,0,0,0],[0,0,1,0,0,0,0,0,0,0,0]]C2702371	pork allergenic extract,pork	[[0,0,0,0,0,0,0,1,0,0,0],[0,0,1,0,0,0,0,0,0,0,0]]C0733433	rs 94991 298,rs94991298	[[0,1,0,0,0,0,0,0,0,0,0],[0,1,0,0,0,0,0,0,0,0,0]]C0765273	bexarotene,4 1 3 5 5 8 8 pentamethyl 5 6 7 8 tetrahydro 2 naphthyl ethenyl benzoic acid,3 methyl ttneb,4 1 5 6 7 8 tetrahydro 3 5 5 8 8 pentamethyl 2 naphthalenyl ethenyl benzoic acid	[[0,0,0,0,0,0,0,1,0,1,0],[0,0,0,0,0,0,0,0,0,0,1],[0,0,0,0,0,0,0,0,0,1,1],[0,0,0,0,1,0,0,0,0,0,0]]C0733431	avil,aventis brand of pheniramine maleate,daneral	[[0,1,0,0,0,0,1,0,0,0,0],[0,1,0,0,0,0,0,0,0,0,0],[0,1,0,0,0,0,0,0,0,0,0]]C0733430	lon798	[[0,1,0,0,0,0,0,0,0,0,0]]C0733435	sn 307,sn307	[[0,1,0,0,0,0,0,0,0,0,0],[0,1,0,0,0,0,0,0,0,0,0]]C0112370	darodipine,diethyl ester 4 4 benzofurazanyl 1 4 dihydro 2 6 dimethyl 3 5 pyridinedicarboxylic acid	[[0,1,0,0,0,0,0,0,0,0,0],[0,1,0,0,0,0,0,0,0,0,0]]C0917838	tomosar	[[0,1,0,0,0,0,0,0,0,0,0]]C2979331	donatuss xp	[[0,0,0,0,0,0,0,1,0,0,0]]C2979336	duraxin	[[0,0,0,0,0,0,0,1,0,0,0]]C2973758	gutgard	[[0,1,0,0,0,0,0,0,0,0,0]]C1542404	homeopathic preparation leptandra virginica	[[0,0,0,0,0,1,0,0,0,0,0]]C2973756	k 2 11 compound	[[0,1,0,0,0,0,0,0,0,0,0]]C2973757	lurex 3,lurex3	[[0,1,0,0,0,0,0,0,0,0,0],[0,1,0,0,0,0,0,0,0,0,0]]C1430694	panax ginseng gpp protein	[[0,1,0,0,0,0,0,0,0,0,0]]C2973755	chlorella 11 peptide	[[0,1,0,0,0,0,0,0,0,0,0]]C2828289	rivenprost,methyl 4 2 1r 2r 3r 3 hydroxy 2 1e 3s 3 hydroxy 4 3 methoxymethyl phenyl but 1 en 1 yl 5 oxocyclopentyl ethyl sulfanyl butanoate	[[0,0,0,0,0,0,0,0,0,1,0],[0,0,0,0,0,0,0,0,0,1,0]]C2973753	cristanine b	[[0,1,0,0,0,0,0,0,0,0,0]]C0056396	cortivazol,11 beta 17 alpha 21 trihydroxy 6 16 alpha dimethyl 2 phenylpregna 2 4 6 trieno 3 2 c pyrazol 20 one 21 acetate	[[0,0,0,0,0,0,0,1,0,1,0],[0,1,0,0,0,0,0,0,0,0,0]]C1542400	homeopathic preparation thymus serpyllum	[[0,0,0,0,0,1,0,0,0,0,0]]C0533651	1 n 4 chlorobenzyl succinamoyl pyrrolidine 2 carbaldehyde	[[0,1,0,0,0,0,0,0,0,0,0]]C0163518	diperodon	[[0,0,0,0,0,0,0,1,0,1,0]]C0088272	1 3 di 2 pyrenyl propane,2py 3 2py	[[0,1,0,0,0,0,0,0,0,0,0],[0,1,0,0,0,0,0,0,0,0,0]]C2979241	legacy	[[0,0,0,0,0,0,0,1,0,0,0]]C3181618	6 2 4 difluorophenoxy 8 methyl 2 tetrahydro 2h pyran 4 ylamino pyrido 2 3 d pyrimidin 7 8h one	[[0,1,0,0,0,0,0,0,0,0,0]]C2266975	lipopeptides	[[0,0,0,0,0,0,0,0,0,0,1]]C2266974	echinocandin antifungal	[[0,0,0,0,0,0,0,0,0,0,1]]C0067668	n acetyl s n methylcarbamoyl cysteine,n amcc,snmcn acetylcysteine,s n methylcarbamoyl n acetylcysteine	[[0,1,0,0,0,0,0,0,0,0,0],[0,1,0,0,0,0,0,0,0,0,0],[0,1,0,0,0,0,0,0,0,0,0],[0,1,0,0,0,0,0,0,0,0,0]]C2266976	lipopeptide antibacterial	[[0,0,0,0,0,0,0,0,0,0,1]]C2266971	sulfonamide antimicrobial	[[0,0,0,0,0,0,0,0,0,0,1]]C2266970	sulfonamide antibacterial	[[0,0,0,0,0,0,0,0,0,0,1]]C2266973	rifamycin antimicrobial	[[0,0,0,0,0,0,0,0,0,0,1]]C2266972	tetracycline class antimicrobial	[[0,0,0,0,0,0,0,0,0,0,1]]C2266979	methylated sulfonamides	[[0,0,0,0,0,0,0,0,0,0,1]]C2266978	methylated sulfonamide antibacterial	[[0,0,0,0,0,0,0,0,0,0,1]]C0252390	3 aminopropyl cyclohexylmethylphosphinic acid	[[0,1,0,0,0,0,0,0,0,0,0]]C1589600	estro span	[[0,0,0,0,0,0,0,1,0,0,0]]C0878038	taractan	[[0,0,0,0,0,0,0,1,0,0,0]]C0758857	4 4 methyl 3 pyrrolidinyl 1h imidazole dihydrochloride	[[0,1,0,0,0,0,0,0,0,0,0]]C1589604	testradiol	[[0,0,0,0,0,0,0,1,0,0,0]]C0878033	2589 r b	[[0,1,0,0,0,0,0,0,0,0,0]]C0878032	abactal	[[0,1,0,0,0,0,0,0,0,0,0]]C0758858	sch 50971	[[0,1,0,0,0,0,0,0,0,0,0]]C0878030	am 833,am833	[[0,1,0,0,0,0,0,0,0,0,0],[0,1,0,0,0,0,0,0,0,0,0]]C0878037	taxilan	[[0,1,0,0,0,0,0,0,0,0,0]]C0878036	c i basic red 5	[[0,0,0,0,0,0,0,0,0,1,0]]C0878035	nsc 249992,nsc249992	[[0,1,0,0,0,0,0,0,0,0,0],[0,1,0,0,0,0,0,0,0,0,0]]C0878034	sn 11841,sn11841	[[0,1,0,0,0,0,0,0,0,0,0],[0,1,0,0,0,0,0,0,0,0,0]]C0250723	gyki 53405	[[0,1,0,0,1,0,0,0,0,0,0]]C0633431	tyr gly nle 28 31 4 no2 phe 33 cholecystokinin 26 33,tyr gly 28 31 nle 33 p no2 phe cholecystokinin 26 33,tyrosyl glycyl norleucyl 28 31 para no2 cholecystokinin 26 33,cck tgnno2p	[[0,1,0,0,0,0,0,0,0,0,0],[0,1,0,0,0,0,0,0,0,0,0],[0,1,0,0,0,0,0,0,0,0,0],[0,1,0,0,0,0,0,0,0,0,0]]C0952593	fenclofenac sodium salt	[[0,1,0,0,0,0,0,0,0,0,0]]C0250726	4 1 hydroxy 3 oxo 3 5 6 7 8 tetrahydro 3 hydroxy 5 5 8 8 tetramethyl 2 naphthalenyl 1 propenyl benzoic acid	[[0,1,0,0,0,0,0,0,0,0,0]]C0250725	n ethyl 1 2 4 isothiocyanothienyl cyclohexylamine,itce 1 2 4	[[0,1,0,0,0,0,0,0,0,0,0],[0,1,0,0,0,0,0,0,0,0,0]]C0952590	3 3 dipropyl 2 2 thiadicarbocyanine thiocyanate	[[0,1,0,0,0,0,0,0,0,0,0]]C2604849	rv09 compound	[[0,1,0,0,0,0,0,0,0,0,0]]C1602443	micotil	[[0,0,0,0,0,0,0,1,0,0,0]]C1701198	jln027	[[0,1,0,0,0,0,0,0,0,0,0]]C1875164	fluorometholone sulfacetamide	[[0,0,0,0,0,0,0,1,0,0,1]]C2358329	methsuximide normethsuximide 124 bld ser plas	[[0,0,0,1,0,0,0,0,0,0,0]]C1701190	bms 582664,bms582664	[[0,1,0,0,1,0,0,0,0,0,0],[0,1,0,0,0,0,0,0,0,0,0]]C2358325	methaqualone metabolite 124 urine	[[0,0,0,1,0,0,0,0,0,0,0]]C2358322	methadone metabolite 124 bld ser plas	[[0,0,0,1,0,0,0,0,0,0,0]]C2358323	methadone metabolite 124 urine	[[0,0,0,1,0,0,0,0,0,0,0]]C2358320	meta methylhippurate para methylhippurate 124 urine	[[0,0,0,1,0,0,0,0,0,0,0]]C0908556	androst 5 ene 4 17 19 trione	[[0,1,0,0,0,0,0,0,0,0,0]]C0908557	19 hydroxyandrost 5 ene 4 17 dione,19 ha5ed	[[0,1,0,0,0,0,0,0,0,0,0],[0,1,0,0,0,0,0,0,0,0,0]]C0016278	flucytosine,5 fluorocytosine,4 amino 5 fluoro 2 1h pyrimidinone,5 fc,5fc,fluorocytosine	[[0,0,0,0,0,0,0,1,1,1,0],[0,0,0,0,0,0,0,0,0,1,0],[0,0,0,0,0,0,0,0,0,1,1],[0,0,0,0,0,0,1,0,0,1,0],[0,0,0,0,1,0,0,0,0,0,0],[0,0,0,0,0,0,1,0,0,0,0]]C0016277	fluconazole,alpha 2 4 difluorophenyl alpha 1h 1 2 4 triazol 1 ylmethyl 1h 1 2 4 triazole 1 ethanol,fluconazole product,4 triazole 1 ethanol alpha 2 4 difluorophenyl alpha 1 2 4 triazol 1 ylmethyl 1 2,fcz	[[0,1,0,0,0,0,0,0,1,1,0],[0,0,0,0,0,0,0,0,0,0,1],[1,0,0,0,0,0,0,0,0,0,0],[0,0,0,0,1,0,0,0,0,0,0],[0,0,0,0,1,0,0,0,0,0,0]]C0908559	2 3 21 trihydroxycholesta 5 24 diene 3 acetate 2 21 disulfate,3hocd 3 acetate 2 21 disulfate	[[0,1,0,0,0,0,0,0,0,0,0],[0,1,0,0,0,0,0,0,0,0,0]]C0016272	fluanxol	[[0,0,0,0,0,0,1,1,0,0,0]]C0115308	e va 16	[[0,1,0,0,0,0,0,0,0,0,0]]C3181168	guttiferone a	[[0,1,0,0,0,0,0,0,0,0,0]]C2718394	fluarix 2009 2010 formula	[[0,0,0,0,0,0,0,1,0,0,0]]C0146917	tris 6 6 7 7 8 8 8 heptafluoro 2 2 dimethyl 3 5 octanedionato europium iii,eu fod 3	[[0,1,0,0,0,0,0,0,0,0,0],[0,1,0,0,0,0,0,0,0,0,0]]C3181167	xenopus stargazin protein,xenopus gamma subunit 2 protein voltage dependent calcium channel	[[0,1,0,0,0,0,0,0,0,0,0],[0,1,0,0,0,0,0,0,0,0,0]]C2718398	fluvirin 2009 2010 formula	[[0,0,0,0,0,0,0,1,0,0,0]]C3181163	2 3 chloro 4 fluorophenyl isothiazol 3 one	[[0,1,0,0,0,0,0,0,0,0,0]]C1569459	dobucor,juste brand of dobutamine hydrochloride	[[0,1,0,0,0,0,0,0,0,0,0],[0,1,0,0,0,0,0,0,0,0,0]]C0025870	metrizoate,3 acetylamino 5 acetylmethylamino 2 4 6 triiodo benzoic acid,metrizoic acid	[[0,0,0,0,0,0,0,0,0,0,1],[0,0,0,0,0,0,0,0,0,0,1],[0,0,0,0,0,0,0,0,0,1,0]]C0025872	metronidazole,2 methyl 5 nitro 1h imidazole 1 ethanol,2 methyl 5 nitroimidazole 1 ethanol,metronidazole product,metronidazole vaginal,metro	[[0,0,0,0,0,0,0,1,0,1,0],[0,0,0,0,0,0,0,0,0,0,1],[0,1,0,0,0,0,0,0,0,0,1],[1,0,0,0,0,0,0,0,0,0,0],[0,0,0,0,0,0,1,0,0,0,0],[0,0,0,0,1,0,0,0,0,0,0]]C0025876	metyrapone,methbipyranone,methopyrapone,2 methyl 1 2 di 3 pyridinyl 1 propanone	[[0,0,0,0,0,0,1,1,0,1,0],[0,0,0,0,0,0,0,0,0,0,1],[0,0,0,0,0,0,0,0,0,0,1],[0,0,0,0,0,0,0,0,0,1,0]]C2713424	viscum album qufrf,va qufrf	[[0,1,0,0,0,0,0,0,0,0,0],[0,1,0,0,0,0,0,0,0,0,0]]C1569453	antabus,altana pharma brand of disulfiram,bohm brand of disulfiram,dumex brand of disulfiram	[[0,1,0,0,0,0,0,0,0,0,0],[0,1,0,0,0,0,0,0,0,0,0],[0,1,0,0,0,0,0,0,0,0,0],[0,1,0,0,0,0,0,0,0,0,0]]C1569452	refusal,artu brand of disulfiram	[[0,1,0,0,0,0,0,0,0,0,0],[0,1,0,0,0,0,0,0,0,0,0]]C1569455	dobutamin solvay,solvay brand of dobutamine hydrochloride	[[0,1,0,0,0,0,0,0,0,0,0],[0,1,0,0,0,0,0,0,0,0,0]]C1569457	dobutamin fresenius,fresenius brand of dobutamine hydrochloride	[[0,1,0,0,0,0,0,0,0,0,0],[0,1,0,0,0,0,0,0,0,0,0]]C1569456	dobutamin hexal,hexal brand of dobutamine hydrochloride	[[0,1,0,0,0,0,0,0,0,0,0],[0,1,0,0,0,0,0,0,0,0,0]]C1569514	farmorubicina,kenfarma brand of epirubicin hydrochloride	[[0,1,0,0,0,0,0,0,0,0,0],[0,1,0,0,0,0,0,0,0,0,0]]C0956746	trans isomer ig 10	[[0,1,0,0,0,0,0,0,0,0,0]]C0956747	2alpha 6alpha 11s isomer tonazocine	[[0,1,0,0,0,0,0,0,0,0,0]]C2984006	thebacon	[[0,0,0,0,0,0,0,0,0,1,0]]C0956745	cis isomer ig 10	[[0,1,0,0,0,0,0,0,0,0,0]]C0051821	andrographolide	[[0,0,0,0,0,0,1,0,0,1,0]]C2984001	methyldesorphine	[[0,0,0,0,0,0,0,0,0,1,0]]C0956740	d threo isomer 3 4 chlorophenyl glutamic acid	[[0,1,0,0,0,0,0,0,0,0,0]]C0956741	l threo isomer 3 4 chlorophenyl glutamic acid	[[0,1,0,0,0,0,0,0,0,0,0]]C1815998	rena vite	[[0,0,0,0,0,0,0,1,0,0,0]]C2001464	alyssin	[[0,1,0,0,0,0,0,0,0,0,0]]C2001462	2 oxohexyl isothiocyanate	[[0,1,0,0,0,0,0,0,0,0,0]]C0956748	2s 2alpha 6alpha 11s isomer methanesulfonate salt tonazocine	[[0,1,0,0,0,0,0,0,0,0,0]]C0956749	2alpha 6alpha 11s isomer methanesulfonate salt tonazocine	[[0,1,0,0,0,0,0,0,0,0,0]]C2975345	1 1s 2r 1 3 5 difluorophenyl 2 hydroxy 3 methylamino propyl 7 fluoro 3 3 dimethyl 1 3 dihydro 2h indol 2 one	[[0,1,0,0,0,0,0,0,0,0,0]]C2975344	way 318068	[[0,1,0,0,0,0,0,0,0,0,0]]C2975343	2 diphenylacetamide 6r 3 azabicyclo 3 1 0 hex 6 ylmethyl 2 hydroxy n methyl 2 5s n 1r	[[0,1,0,0,0,0,0,0,0,0,0]]C2975342	ae9c90cb	[[0,1,0,0,0,0,0,0,0,0,0]]C0299184	n alpha acetyl nona d arginine amide acetate,n alpha acetylnona d arginine amide acetate	[[0,1,0,0,0,0,0,0,0,0,0],[0,1,0,0,0,0,0,0,0,0,0]]C0064312	ketocaine,1 2 2 bis 1 methylethyl amino ethoxy phenyl 1 butanone,1 2 2 bis 1 1 methylethyl amino ethoxy phenyl 1 butanone,2 2 diisopropylamino ethoxy butyrophenone	[[0,1,0,0,0,0,0,0,0,1,0],[0,1,0,0,0,0,0,0,0,0,0],[0,1,0,0,0,0,0,0,0,0,0],[0,1,0,0,0,0,0,0,0,1,0]]C0064311	ketobemidone,1 4 3 hydroxyphenyl 1 methyl 4 piperidinyl 1 propanone,1 4 m hydroxyphenyl 1 methyl 4 piperidyl 1 propanone,cetobemidon	[[0,1,0,1,0,0,0,0,0,1,0],[0,1,0,0,0,0,0,0,0,0,0],[0,1,0,0,0,0,0,0,0,0,0],[0,1,0,0,0,0,0,0,0,0,0]]C0064316	ketogin,mixt with n n dimethyl 4 4 diphenyl 3 buten 2 amine hydrochloride 1 4 3 hydroxyphenyl 1 methyl 4 piperidinyl 1 propanone,ketogan	[[0,1,0,0,0,0,1,0,0,0,0],[0,1,0,0,0,0,0,0,0,0,0],[0,1,0,0,0,0,1,0,0,0,0]]C0011134	deet,det,deta,n n diethyl m toluamide,n n diethyltoluamide,n n diethyl 3 methyl benzamide,n n diethyl 3 methylbenzamide,diethyltoluamide,deet n n diethyl meta toluamide,diethyl toluamide	[[0,0,0,0,0,0,1,1,0,0,1],[0,0,0,0,0,0,1,0,0,0,1],[0,0,0,0,0,0,1,0,0,0,1],[0,1,0,0,0,0,0,0,0,0,1],[0,1,0,0,0,0,0,0,0,0,1],[0,0,0,0,0,0,0,0,0,0,1],[0,1,0,0,0,0,0,0,0,0,1],[0,0,0,0,0,0,1,0,0,1,0],[0,0,0,0,0,0,0,0,0,0,1],[0,0,0,0,0,0,1,0,0,0,0]]C0246318	lnc 834	[[0,1,0,0,0,0,0,0,0,0,0]]C2697981	aldehyde dehydrogenase bright cells,aldhbr cells	[[0,0,0,0,0,0,0,0,0,1,0],[0,0,0,0,0,0,0,0,0,1,0]]C0020346	hydroxydione,21 hydroxy 5 alpha pregnane 3 20 dione,5beta 21 hydroxy pregnane 3 20 dione	[[1,1,0,0,0,0,0,0,0,0,0],[0,1,0,0,0,0,0,0,0,0,0],[0,1,0,0,0,0,0,0,0,0,0]]C0020347	hydroxydione sodium	[[0,1,0,0,0,0,0,0,0,0,0]]C1676051	resistoflavin methyl ether	[[0,1,0,0,0,0,0,0,0,0,0]]C0246313	phthoxazolin,z z e 3 hydroxy 2 2 4 trimethyl 10 5 oxazolyl 4 6 8 decatrienamide	[[0,1,0,0,0,0,0,0,0,0,0],[0,1,0,0,0,0,0,0,0,0,0]]C1676053	ym 231146	[[0,1,0,0,0,0,0,0,0,0,0]]C0632381	n pivaloyloxymethylchlorpromazine,cpz p	[[0,1,0,0,0,0,0,0,0,0,0],[0,1,0,0,0,0,0,0,0,0,0]]C0632383	n benzoyloxymethylchlorpromazine,cpz b	[[0,1,0,0,0,0,0,0,0,0,0],[0,1,0,0,0,0,0,0,0,0,0]]C0639657	4 pyridoxic acid 5 phosphate,4 pa 5p	[[0,1,0,0,0,0,0,0,0,0,0],[0,1,0,0,0,0,0,0,0,0,0]]C2826577	diphenylpiperidinomethyldioxolan iodide,anacolin,1 2 2 diphenyl 1 3 dioxolan 4 ylmethyl 1 methylpiperidinium iodide	[[0,0,0,0,0,0,0,0,0,1,0],[0,0,0,0,0,0,0,0,0,1,0],[0,0,0,0,0,0,0,0,0,1,0]]C0638523	2 6 dichloro 4 aminophenol iloprost,dchpa iloprost,dichlorohydroxyphenylamide iloprost,dichlorohydroxyphenylamideiloprost	[[0,1,0,0,0,0,0,0,0,0,0],[0,1,0,0,0,0,0,0,0,0,0],[0,1,0,0,0,0,0,0,0,0,0],[0,1,0,0,0,0,0,0,0,0,0]]C0622943	3 acetoxy 1 11 12 dolabell 4 8 18 trien 16 al,adta	[[0,1,0,0,0,0,0,0,0,0,0],[0,1,0,0,0,0,0,0,0,0,0]]C0622940	neoisostegane,3a 4 13 13a tetrahydro 5 6 7 10 11 pentamethoxydibenzo 4 5 6 7 cycloocta 1 2 c furan 3 1h one	[[0,1,0,0,0,0,0,0,0,0,0],[0,1,0,0,0,0,0,0,0,0,0]]C0622941	3 s ac 1 r 11 s 12 r dolabell 4 e 8 e 18 trien 16 al	[[0,1,0,0,0,0,0,0,0,0,0]]C0622946	3 hydroxy 16 acetoxy 1 11 12 dolabell 4 8 18 triene,hadt	[[0,1,0,0,0,0,0,0,0,0,0],[0,1,0,0,0,0,0,0,0,0,0]]C0622947	rollinone	[[0,1,0,0,0,0,0,0,0,0,0]]C0245653	alpha trinositol	[[0,1,0,0,0,0,0,0,0,0,0]]C2928748	riboflavin vitamin b6	[[0,0,0,0,0,0,0,1,0,0,0]]C2358192	levodopa 124 cerebral spinal fluid	[[0,0,0,1,0,0,0,0,0,0,0]]C0122044	hla drb5 antigen,hla drb5,hla drb5 chains,human histocompatibility complex derived antigens drb5	[[0,1,0,0,0,1,0,0,0,0,1],[0,0,0,1,0,0,0,0,0,0,1],[0,1,0,0,0,0,0,0,0,0,1],[0,0,0,1,0,0,0,0,0,0,0]]C2697987	pepsitox	[[0,0,0,0,0,0,0,0,0,1,0]]C0122042	hla drb3 chains,hla drb3,hla drb3 antigen,human histocompatibility complex derived antigens drb3	[[0,1,0,0,0,0,0,0,0,0,0],[0,0,0,1,0,0,0,0,0,0,1],[0,1,0,0,0,0,0,0,0,0,1],[0,0,0,1,0,0,0,0,0,0,0]]C0245658	gadolinium 1 4 7 10 tetraazacyclododecane n n n n tetrakis,gd dotp	[[0,1,0,0,0,0,0,0,0,0,0],[0,1,0,0,0,0,0,0,0,0,0]]C0122040	hla drb1 antigen,hla drb1,hla drb1 chains,human histocompatibility complex derived antigens drb1	[[0,1,0,0,0,1,0,0,0,0,1],[0,0,0,1,0,0,0,0,0,0,1],[0,1,0,0,0,0,0,0,0,0,1],[0,0,0,1,0,0,0,0,0,0,0]]C0700918	slow k	[[0,0,0,0,0,0,1,1,0,0,0]]C0700919	rum k	[[0,0,0,0,0,0,0,1,0,0,0]]C0166039	r 493	[[0,1,0,0,0,0,0,0,0,0,0]]C2697989	alitame anhydrous	[[0,0,0,0,0,0,0,0,0,1,0]]C0700912	sk f 104864 a,skf 104864 a,sk f104864a,skf104864a	[[0,1,0,0,0,0,0,0,0,0,0],[0,1,0,0,0,0,0,0,0,0,0],[0,1,0,0,0,0,0,0,0,0,0],[0,1,0,0,0,0,0,0,0,0,0]]C0700913	nsc 609699,nsc609699	[[0,1,0,0,0,0,0,0,0,0,0],[0,1,0,0,0,0,0,0,0,0,0]]C0700910	monotard insulin	[[0,1,0,0,0,0,0,0,0,0,0]]C0700911	wellcome 248u,wellcome248u	[[0,1,0,0,0,0,0,0,0,0,0],[0,1,0,0,0,0,0,0,0,0,0]]C0700916	enacard	[[0,0,0,0,1,0,0,0,0,0,0]]C0700917	tromasin	[[0,1,0,0,0,0,0,0,0,0,0]]C0166031	sk f 96231,skf 96231,sb 96231	[[0,1,0,0,0,0,0,0,0,0,0],[0,1,0,0,0,0,0,0,0,0,0],[0,1,0,0,0,0,0,0,0,0,0]]C0700915	hycamtin,smithkline beecham brand of topotecan hydrochloride	[[0,0,0,0,0,0,1,1,0,0,0],[0,1,0,0,0,0,0,0,0,0,0]]C1513201	farmitrexat	[[0,0,0,0,1,0,0,0,0,0,0]]C0282851	calcofluor white m2r	[[0,1,0,0,0,0,0,0,0,0,0]]C1137817	actara 25 wg	[[0,1,0,0,0,0,0,0,0,0,0]]C0282854	0 5 citrate cpd	[[0,1,0,0,0,0,0,0,0,0,0]]C1513200	emthexat,emthexate	[[0,0,0,0,1,0,0,0,0,0,0],[0,0,0,0,1,0,0,0,0,0,0]]C0294259	4 1 hydroxy 2 1 2 3 4 tetrahydro 1 oxo 2 naphthyl methyl amino ethyl methanesulfonanilide hydrochloride	[[0,1,0,0,0,0,0,0,0,0,0]]C0294258	9 methoxytariacuripyrone,9 mtc	[[0,1,0,0,0,0,0,0,0,0,0],[0,1,0,0,0,0,0,0,0,0,0]]C2928741	aminophylline guaifenesin phenobarbital	[[0,0,0,0,0,0,0,1,0,0,0]]C3180482	vejovine	[[0,1,0,0,0,0,0,0,0,0,0]]C1122040	1 benzylnicotinamide	[[0,1,0,0,0,0,0,0,0,0,0]]C0045373	2 3 dihydro 7 methyl 9 phenyl 1h pyrazolo 1 2 a indazolium,bromide 2 3 dihydro 7 methyl 9 phenyl 1h pyrazolo 1 2 a indazol 4 ium	[[0,1,0,0,0,0,0,0,0,0,0],[0,1,0,0,0,0,0,0,0,0,0]]C0045376	2 3 dioxo 6 nitro 7 sulfamoylbenzo f quinoxaline,nbqx,6 nitro 7 sulfamoylbenzo f quinoxaline 2 3 dione,2 3 dihydroxy 6 nitro 7 sulfamoyl benzo f quinoxaline	[[0,1,0,0,0,0,0,0,0,0,0],[0,1,0,0,0,0,0,0,0,0,0],[0,1,0,0,0,0,0,0,0,0,0],[0,1,0,0,0,0,0,0,0,0,0]]C1122048	efomycine m	[[0,1,0,0,0,0,0,0,0,0,0]]C2928742	acetaminophen pamabrom vitamin e	[[0,0,0,0,0,0,0,1,0,0,0]]C2732016	human coagulation factor viii von willebrand factor complex	[[0,0,0,0,0,0,0,0,0,1,0]]C2732017	coagulation factor viia recombinant human	[[0,0,0,0,0,0,0,0,0,1,0]]C1522596	zuclomiphene citrate,cis clomiphene citrate,cisclomiphene citrate	[[0,0,0,0,0,0,0,0,0,1,0],[0,0,0,0,0,0,0,0,0,1,0],[0,0,0,0,0,0,0,0,0,1,0]]C0219689	calcium gluconolactate carbonate	[[0,1,0,0,0,0,0,0,0,0,0]]C0219688	abbott 81282,a 81282,4 butyl 2 1h tetrazol 5 yl 1 1 biphenyl 4 yl methyl amino 5 pyrimidinecarboxylic acid	[[0,1,0,0,0,0,0,0,0,0,0],[0,1,0,0,0,0,0,0,0,0,0],[0,1,0,0,0,0,0,0,0,0,0]]C2729544	legend	[[0,0,0,0,0,0,0,1,0,0,0]]C0061098	ganglioside m1 lactone,gm1 lactone	[[0,1,0,0,0,0,0,0,0,0,0],[0,1,0,0,0,0,0,0,0,0,0]]C0020960	immune sera,immune serums,antisera,antiserum	[[0,1,0,0,0,0,1,0,0,0,1],[0,1,0,0,0,0,1,0,0,0,1],[0,0,0,0,0,0,1,0,0,0,1],[1,0,0,0,0,0,1,0,0,0,0]]C2729548	nitrozone	[[0,0,0,0,0,0,0,1,0,0,0]]C2928746	ascorbic acid ferrous gluconate folic acid	[[0,0,0,0,0,0,0,1,0,0,0]]C0219686	4 n n butyl n 2 1h tetrazol 5 yl biphenyl 4 yl methyl amino pyrimidine 5 carboxylic acid	[[0,1,0,0,0,0,0,0,0,0,0]]C1453213	isothymusin,6 7 dimethoxy 5 8 4 trihydroxyflavone	[[0,1,0,0,0,0,0,0,0,0,0],[0,1,0,0,0,0,0,0,0,0,0]]C0311006	clostridium chauvoei septicum haemolyticum novyi tetani perfringens types c d bacterin toxoid,clostridium chauvoei septicum hemolyticum novyi tetani perfringens types c d bacterin toxoid	[[1,0,0,0,0,0,0,0,0,0,0],[1,0,0,0,0,0,0,0,0,0,0]]C0311007	clostridium chauvoei septicum novyi bacterin toxoid	[[1,0,0,0,0,0,0,0,0,0,0]]C0311004	clostridium chauvoei septicum haemolyticum novyi sordellii perfringens types c d bacterin toxoid,clostridium chauvoei septicum hemolyticum novyi sordellii perfringens types c d bacterin toxoid	[[1,0,0,0,0,0,0,0,0,0,0],[1,0,0,0,0,0,0,0,0,0,0]]C0311005	clostridium chauvoei septicum haemolyticum novyi sordellii perfringens types c d leptospira pomona pasteurella haemolytica multocida bacterin toxoid,clostridium chauvoei septicum hemolyticum novyi sordellii perfringens types c d leptospira pomona pasteurella hemolytica multocida bacterin toxoid	[[1,0,0,0,0,0,0,0,0,0,0],[1,0,0,0,0,0,0,0,0,0,0]]C0311002	clostridium chauvoei septicum haemolyticum novyi sordellii leptospira pomona bacterin toxoid,clostridium chauvoei septicum hemolyticum novyi sordellii leptospira pomona bacterin toxoid	[[1,0,0,0,0,0,0,0,0,0,0],[1,0,0,0,0,0,0,0,0,0,0]]C0311003	clostridium chauvoei septicum haemolyticum novyi sordellii pasteurella haemolytica multocida bacterin toxoid,clostridium chauvoei septicum hemolyticum novyi sordellii pasteurella hemolytica multocida bacterin toxoid	[[1,0,0,0,0,0,0,0,0,0,0],[1,0,0,0,0,0,0,0,0,0,0]]C0211058	las 30538,1 2 2 6 dimethylphenoxy ethyl alpha alpha bis 4 fluorophenyl 4 piperidinemethanol	[[0,1,0,0,0,0,0,0,0,0,0],[0,1,0,0,0,0,0,0,0,0,0]]C0311001	clostridium chauvoei septicum haemolyticum novyi sordellii bacterin toxoid,clostridium chauvoei septicum hemolyticum novyi sordellii bacterin toxoid	[[1,0,0,0,0,0,0,0,0,0,0],[1,0,0,0,0,0,0,0,0,0,0]]C0211057	1 2 2 6 dimethylphenoxy ethyl alpha alpha bis p fluorophenyl 4 piperidine methanol	[[0,1,0,0,0,0,0,0,0,0,0]]C0051768	amtizol,1 2 4 thiadiazole 3 5 diamine	[[0,1,0,0,0,0,0,0,0,0,0],[0,1,0,0,0,0,0,0,0,0,0]]C0311008	clostridium chauvoei septicum novyi sordellii bacterin toxoid	[[1,0,0,0,0,0,0,0,0,0,0]]C0311009	clostridium chauvoei septicum novyi sordellii haemophilus somnus bacterin toxoid	[[1,0,0,0,0,0,0,0,0,0,0]]C0888845	isomer isometheptene	[[0,1,0,0,0,0,0,0,0,0,0]]C0888844	isometheptene monohydrochoride	[[0,1,0,0,0,0,0,0,0,0,0]]C0888847	maleate isomer isometheptene	[[0,1,0,0,0,0,0,0,0,0,0]]C0888846	monomaleate isomer isometheptene	[[0,1,0,0,0,0,0,0,0,0,0]]C0888841	maleate isometheptene	[[0,1,0,0,0,0,0,0,0,0,0]]C0888843	monotartrate isometheptene	[[0,1,0,0,0,0,0,0,0,0,0]]C0753942	bisindolylmaleimide iv	[[0,1,0,0,0,0,0,0,0,0,0]]C1601365	tuss ax	[[0,0,0,0,0,0,0,1,0,0,0]]C1990329	hydroxyalprazolam 124 stool	[[0,0,0,1,0,0,0,0,0,0,0]]C1990328	hydroxocobalamin 124 bld ser plas	[[0,0,0,1,0,0,0,0,0,0,0]]C0094527	3 4 dihydro 6 4 4 oxo 4 phenylbutyl 1 piperazinylcarbonyl 2 1h quinolinone	[[0,1,0,0,0,0,0,0,0,0,0]]C1601362	hycotuss	[[0,0,0,0,0,0,0,1,0,0,0]]C3257579	live attenuated salmonella typhi vaccine	[[0,0,0,0,0,0,0,0,0,0,1]]C1515400	utrogestan	[[0,0,0,0,0,0,0,0,0,1,0]]C1515403	testostroval pa	[[0,0,0,0,1,0,0,0,0,0,0]]C2005695	acetaminophen butabarbital	[[0,0,0,0,0,0,0,1,0,0,0]]C1515405	virosterone	[[0,0,0,0,1,0,0,0,0,0,0]]C1515404	mertestate	[[0,0,0,0,0,0,0,0,0,1,0]]C1515407	recombinant transforming growth factor beta 3,therapeutic tgf beta 3	[[0,0,0,0,0,0,0,0,0,1,0],[0,0,0,0,0,0,0,0,0,1,0]]C1515406	recombinant transforming growth factor beta 1,transforming growth factor beta 1,tgfbeta1	[[0,0,0,0,0,0,0,0,0,1,0],[0,0,0,0,0,0,0,0,0,1,0],[0,0,0,0,0,0,0,0,0,1,0]]C0379887	agrobacterium conjugation factor 1,a tumefaciens cf1,aai oohl	[[0,1,0,0,0,0,0,0,0,0,0],[0,1,0,0,0,0,0,0,0,0,0],[0,1,0,0,0,0,0,0,0,0,0]]C0950356	bis 3 diethylamino propyl fluoranthene 3 9 dicarboxylate dihyrochloride,rmi 9563 dihydrochloride	[[0,1,0,0,0,0,0,0,0,0,0],[0,1,0,0,0,0,0,0,0,0,0]]C0950353	af 1890	[[0,0,0,0,1,0,0,0,0,0,0]]C1170547	prevacid solutab	[[0,0,0,0,0,0,0,1,0,0,0]]C0950351	trichopolyns	[[0,1,0,0,0,0,0,0,0,0,0]]C3257574	live attenuated bacillus calmette guerin vaccine,live attenuated bacillus calmette guerin bcg vaccine,live attenuated bcg vaccine	[[0,0,0,0,0,0,0,0,0,0,1],[0,0,0,0,0,0,0,0,0,0,1],[0,0,0,0,0,0,0,0,0,0,1]]C3252354	ly2334737	[[0,1,0,0,0,0,0,0,0,0,0]]C2343933	temovate e	[[0,0,0,0,0,0,0,1,0,0,0]]C0390240	cgp 53153	[[0,1,0,0,0,0,0,0,0,0,0]]C0086317	phthivazide,ftivazide	[[0,1,0,0,0,0,0,0,0,0,0],[0,0,0,0,0,0,0,0,0,1,0]]C1268833	clobetasone butyrate topical preparation	[[1,0,0,0,0,0,0,0,0,0,0]]C1873794	liquicet	[[0,0,0,0,0,0,0,1,0,0,0]]C1997532	oral form hydroxyzine	[[1,0,0,0,0,0,0,0,0,0,0]]C0098944	6 4 hydroxyphenylhydrazino uracil,6 2 4 hydroxyphenyl hydrazino 2 4 1h 3h pyrimidinedione,6 p hydroxyphenylhydrazino uracil,h2 hpura,6 p hydroxyphenylazo uracil	[[0,1,0,0,0,0,0,0,0,0,0],[0,1,0,0,0,0,0,0,0,0,0],[0,1,0,0,0,0,0,0,0,0,0],[0,1,0,0,0,0,0,0,0,0,0],[0,1,0,0,0,0,0,0,0,0,0]]C1268832	clobetasol propionate topical preparation	[[1,0,0,0,0,0,0,0,0,0,0]]C0119473	gamma glutamyl l dopa,gludopa	[[0,1,0,0,0,0,0,0,0,0,0],[0,1,0,0,0,0,0,0,0,0,0]]C0098942	6 4 fluorophenyl 2 3 dihydro 5 4 pyridinyl imidazo 2 1 b thiazole	[[0,1,0,0,0,0,0,0,0,0,0]]C0595126	vesanoid	[[0,0,0,0,0,0,1,1,0,0,0]]C0130018	n 1 butyl 2 pyrrolidinyl methyl 2 methyl 5 sulfamoyl 2 3 dihydrobenzofuran 7 carboxamide	[[0,1,0,0,0,0,0,0,0,0,0]]C1602476	symax	[[0,0,0,0,0,0,0,1,0,0,0]]C0896011	r s isomer dihydrochloride bisobrin	[[0,1,0,0,0,0,0,0,0,0,0]]C0896010	dihydrochloride bisobrin	[[0,1,0,0,0,0,0,0,0,0,0]]C0896013	r r isomer monohydrochloride bisobrin	[[0,1,0,0,0,0,0,0,0,0,0]]C0896012	r s isomer monohydrochloride bisobrin	[[0,1,0,0,0,0,0,0,0,0,0]]C0005177	beta gamma thrombin	[[0,1,0,0,0,0,0,0,0,0,0]]C1170658	tev tropin	[[0,0,0,0,0,0,0,1,0,0,0]]C3257573	live attenuated bacillus anthracis vaccine	[[0,0,0,0,0,0,0,0,0,0,1]]C0060947	gal 1	[[0,1,0,0,0,0,0,0,0,0,0]]C1511871	dexacen	[[0,0,0,0,0,0,0,0,0,1,0]]C0289945	4 1 1 4 phenylbutanoyl 2 pyrrolidinyl carbonyl 2 pyrrolidinyl carbonyl thiazole,4 ppcpct	[[0,1,0,0,0,0,0,0,0,0,0],[0,1,0,0,0,0,0,0,0,0,0]]C0289947	pd 143188	[[0,1,0,0,0,0,0,0,0,0,0]]C0289946	ci 1007	[[0,1,0,0,0,0,0,0,0,0,0]]C0379881	trovafloxacin,trovafloxacin product	[[0,0,0,1,0,0,0,1,0,1,0],[1,0,0,0,0,0,0,0,0,0,0]]C0289943	2 1 1 4 phenylbutanoyl 2 pyrrolidinyl carbonyl 2 pyrrolidinyl carbonyl thiazole,2 ppcpct	[[0,1,0,0,0,0,0,0,0,0,0],[0,1,0,0,0,0,0,0,0,0,0]]C0258177	succinimidyl 6 7 nitrobenz 2 oxa 1 3 diazol 4 yl aminohexanoate,nbd suc	[[0,1,0,0,0,0,0,0,0,0,0],[0,1,0,0,0,0,0,0,0,0,0]]C0657772	ocimum oil	[[0,1,0,0,0,0,0,0,0,0,0]]C0289949	1 2 3 6 tetrahydro 4 phenyl 1 3 phenyl 3 cyclohexen 1 yl methyl pyridine,tppcmp	[[0,1,0,0,0,0,0,0,0,0,0],[0,1,0,0,0,0,0,0,0,0,0]]C0657777	clostridium botulinum m toxin,cb m toxin	[[0,1,0,0,0,0,0,0,0,0,0],[0,1,0,0,0,0,0,0,0,0,0]]C1980461	5 hydroxypropafenone 124 bld ser plas	[[0,0,0,1,0,0,0,0,0,0,0]]C0966678	3 azido 3 deoxythymidine 5 3 1 o hexadecyl 2 o methylglycerol phosphate,1 o hexadecyl 2 o methyl sn glyceryl phosphodiester azt,hmgpde azt	[[0,1,0,0,0,0,0,0,0,0,0],[0,1,0,0,0,0,0,0,0,0,0],[0,1,0,0,0,0,0,0,0,0,0]]C0293948	peonan pa	[[0,1,0,0,0,0,0,0,0,0,0]]C1980462	5 hydroxytryptophan 124 bld ser plas	[[0,0,0,1,0,0,0,0,0,0,0]]C1980464	5 hydroxytryptophan 124 urine	[[0,0,0,1,0,0,0,0,0,0,0]]C2697658	ibrolipim	[[0,0,0,0,0,0,0,0,0,1,0]]C0966670	4 5 2 4 difluorophenyl 4 5 dihydro 3 trifluoromethyl 1h pyrazol 1 yl benzenesulfonamide	[[0,1,0,0,0,0,0,0,0,0,0]]C0214105	nk 611,nk611	[[0,1,0,0,0,0,0,0,0,0,0],[0,1,0,0,0,0,0,0,0,0,0]]C3178452	ve 821,ve821 cpd	[[0,1,0,0,0,0,0,0,0,0,0],[0,1,0,0,0,0,0,0,0,0,0]]C0293947	thiopalmitic acid	[[0,1,0,0,0,0,0,0,0,0,0]]C0293944	spermine nitric oxide complex,n 4 1 3 aminopropyl 2 hydroxy 2 nitrosohydrazino butyl 1 3 propanediamine,spermine nitric oxide,sper no,spermine nonoate,noc 22	[[0,1,0,0,0,0,0,0,0,0,0],[0,1,0,0,0,0,0,0,0,0,0],[0,1,0,0,0,0,0,0,0,0,0],[0,1,0,0,0,0,0,0,0,0,0],[0,1,0,0,0,0,0,0,0,0,0],[0,1,0,0,0,0,0,0,0,0,0]]C0966676	irdye800,irdye 800	[[0,1,0,0,0,0,0,0,0,0,0],[0,1,0,0,0,0,0,0,0,0,0]]C1601585	jay phyl	[[0,0,0,0,0,0,0,1,0,0,0]]C2343930	lexiscan	[[0,0,0,0,0,0,0,1,0,0,0]]C2930435	para chlorophenol drug combination formaldehyde camphor,camphor formaldehyde para chlorophenol	[[0,1,0,0,0,0,0,0,0,0,0],[0,1,0,0,0,0,0,0,0,0,0]]C2961699	white benzocaine petrolatum	[[0,0,0,0,0,0,0,1,0,0,0]]C0958073	8 14 ene 3beta 5alpha 15beta isomer cholest 8 ene 3 15 diol	[[0,1,0,0,0,0,0,0,0,0,0]]C0245525	bunaprolast,acetate 2 butyl 4 methoxy 1 naphthalenol,1 acetoxy 2 n butyl 4 methoxynaphthalene	[[0,1,0,0,0,0,0,0,0,1,0],[0,1,0,0,0,0,0,0,0,0,0],[0,1,0,0,0,0,0,0,0,0,0]]C0287532	uk 80067	[[0,1,0,0,0,0,0,0,0,0,0]]C0287533	modipafant,4 2 chlorophenyl 6 methyl 2 4 2 methylimidazo 4 5 c pyrid 1 yl phenyl 5 2 pyridinylcarbamoyl 1 4 dihydropyridine 3 carboxylic acid ethyl ester,ethyl ester 4 2 chlorophenyl 1 4 dihydro 6 methyl 2 4 2 methyl 1h imidazo 4 5 c pyridin 1 yl phenyl 5 2 pyridinylamino carbonyl 3 pyridinecarboxylic acid	[[0,1,0,0,0,0,0,0,0,1,0],[0,1,0,0,0,0,0,0,0,0,0],[0,1,0,0,0,0,0,0,0,0,0]]C0245524	u 66 858,u 66858	[[0,1,0,0,0,0,0,0,0,0,0],[0,1,0,0,0,0,0,0,0,0,0]]C0951006	cyanine dye 1chloride	[[0,1,0,0,0,0,0,0,0,0,0]]C0951007	bumecain hydrochloride	[[0,1,0,0,0,0,0,0,0,0,0]]C0951008	coralyne sulfoacetate	[[0,1,0,0,0,0,0,0,0,0,0]]C0951009	coralyne chloride	[[0,1,0,0,0,0,0,0,0,0,0]]C0659746	sae9,sae 9	[[0,1,0,0,0,0,0,0,0,0,0],[0,1,0,0,0,0,0,0,0,0,0]]C2722028	banana allergenic extract,banana	[[0,0,0,0,0,0,0,1,0,0,0],[0,0,1,0,0,0,0,0,0,0,0]]C0659744	pd 135390	[[0,1,0,0,0,0,0,0,0,0,0]]C0659745	2 n n dimethylamino 4 6 bis 1 h imidazol 1 yl 1 3 5 triazine	[[0,1,0,0,0,0,0,0,0,0,0]]C0659742	pd 134922	[[0,1,0,0,0,0,0,0,0,0,0]]C2722029	barley allergenic extract,barley	[[0,0,0,0,0,0,0,1,0,0,0],[0,0,1,0,0,0,0,0,0,0,0]]C1588351	myophen	[[0,0,0,0,0,0,0,1,0,0,0]]C1588357	kepvance	[[0,0,0,0,0,0,0,1,0,0,0]]C0090539	16 beta ethylestradiol 17 beta,16 beta 17 beta 16 ethylestra 1 3 5 10 triene 3 17 diol	[[0,1,0,0,0,0,0,0,0,0,0],[0,1,0,0,0,0,0,0,0,0,0]]C0659749	3 5 dioctanoyl 5 bromodeoxyuridine,brdu c8	[[0,1,0,0,0,0,0,0,0,0,0],[0,1,0,0,0,0,0,0,0,0,0]]C0914797	chiron vaccine,chiron	[[0,0,0,0,0,1,0,0,0,0,0],[0,0,0,0,0,0,1,0,0,0,0]]C0914796	ethyl 4 4 hydoxyphenyl methylidene 2 methyl 5 oxoimidazoleacetate,ethyl 4 hophmmoiac	[[0,1,0,0,0,0,0,0,0,0,0],[0,1,0,0,0,0,0,0,0,0,0]]C0914794	2 5 1 tetrazolylmethyl thien 3 yl n 2 mercaptomethyl 4 phenylbutyrylglycine,2 tmt nmpbgly	[[0,1,0,0,0,0,0,0,0,0,0],[0,1,0,0,0,0,0,0,0,0,0]]C2983837	moenomycin a12	[[0,0,0,0,0,0,0,0,0,1,0]]C0914790	strobilurin p	[[0,1,0,0,0,0,0,0,0,0,0]]C0958078	s isomer pramipexol dihydrochloride	[[0,1,0,0,0,0,0,0,0,0,0]]C0914799	cd4 igg,cd4 igg2	[[0,1,0,0,0,0,0,0,0,0,0],[0,1,0,0,0,0,0,0,0,0,0]]C3256573	colloidol silicon dioxide	[[0,0,1,0,0,0,0,0,0,0,0]]C1973922	streptomycin 124 isolate serum	[[0,0,0,1,0,0,0,0,0,0,0]]C3256571	colloidial silicon dioxide	[[0,0,1,0,0,0,0,0,0,0,0]]C3256570	colloidal solicon dioxide	[[0,0,1,0,0,0,0,0,0,0,0]]C3256577	copovidone	[[0,0,1,0,0,0,0,0,0,0,0]]C0610678	2 4 dinitrophenylglycine diazoketone,1 diazo 3 2 4 dinitrophenyl amino 2 propanone	[[0,1,0,0,0,0,0,0,0,0,0],[0,1,0,0,0,0,0,0,0,0,0]]C2722039	grape allergenic extract,grape	[[0,0,0,0,0,1,0,0,0,0,0],[0,0,1,0,0,0,0,0,0,0,0]]C0720241	epivir hbv	[[0,0,0,0,0,0,1,1,0,0,0]]C3256579	corn derived proteins	[[0,0,1,0,0,0,0,0,0,0,0]]C3256578	corn starch lactose	[[0,0,1,0,0,0,0,0,0,0,0]]C1445426	chaetomium globosum extract,chaetomium globosum	[[0,0,0,0,0,0,0,1,0,0,0],[0,0,1,0,0,0,0,0,0,0,0]]C2194307	caffeine dipyrone ergotamine	[[0,0,0,0,0,0,0,1,0,0,0]]C0892804	monohydrochloride 5 fluoro alpha methyltryptamine	[[0,1,0,0,0,0,0,0,0,0,0]]C0892805	5 fluoro alpha methyltryptamine hydrochloride	[[0,1,0,0,0,0,0,0,0,0,0]]C2933297	arenamide a	[[0,1,0,0,0,0,0,0,0,0,0]]C1515377	recombinant growth factor,growth agents	[[0,0,0,0,0,0,0,0,0,1,0],[0,0,0,0,0,0,0,0,0,1,0]]C3256636	polyisobutylene	[[0,0,1,0,0,0,0,0,0,0,0]]C1515376	stimu lh	[[0,0,0,0,0,0,0,0,0,1,0]]C0627237	crisamicin b,crs b	[[0,1,0,0,0,0,0,0,0,0,0],[0,1,0,0,0,0,0,0,0,0,0]]C1515375	lutrelef	[[0,0,0,0,0,0,0,0,0,1,0]]C0627230	bis tyrosyl prolyl phenylalaninamide hydrazide	[[0,1,0,0,0,0,0,0,0,0,0]]C1515374	lutrefact	[[0,0,0,0,0,0,0,0,0,1,0]]C3256639	polymethylsilsesquioxane	[[0,0,1,0,0,0,0,0,0,0,0]]C1515373	luforan	[[0,0,0,0,0,0,0,0,0,1,0]]C1515372	cryptocur	[[0,0,0,0,0,0,0,0,0,1,0]]C3251932	mk 3281,mk3281	[[0,1,0,0,0,0,0,0,0,0,0],[0,1,0,0,0,0,0,0,0,0,0]]C2603888	globulol	[[0,1,0,0,0,0,0,0,0,0,0]]C0002904	anesthesia adjuvants,anesthetic adjuvants	[[0,1,0,0,0,0,0,0,0,0,0],[0,1,0,0,0,0,0,0,0,0,0]]C1454363	elsibucol,4 2 6 di tert butyl 4 1 3 5 di tert butyl 4 hydroxy phenylsulfanyl 1 methyl ethylsulfanyl phenoxy butyric acid	[[0,1,0,0,0,0,0,0,0,1,0],[0,1,0,0,0,0,0,0,0,0,0]]C1702916	pentetate	[[0,0,0,0,0,0,0,1,0,0,1]]C3251930	3 5 4 2 hydroxy 2 methylpropionyl piperazin 1 yl 2 trifluoromethylphenyl 4 1h indol 3 yl pyrrole 2 5 dione,hmptip dione	[[0,1,0,0,0,0,0,0,0,0,0],[0,1,0,0,0,0,0,0,0,0,0]]C0287390	cinalukast,4 3 2 4 cyclobutyl 2 thiazolyl ethenyl phenylamino 2 2 diethyl 4 oxobutanoic acid	[[0,1,0,0,0,0,0,0,0,0,0],[0,1,0,0,0,0,0,0,0,0,0]]C2603883	larotaxel	[[0,0,0,0,1,0,0,0,0,1,0]]C2973571	hh gv 678,flumatinib,4 4 methylpiperazin 1 ylmethyl n 6 methyl 5 4 pyridin 3 ylpyrimidin 2 ylamino pyridin 3 yl 3 trifluoromethylbenzamide,hh gv678	[[0,1,0,0,0,0,0,0,0,0,0],[0,1,0,0,0,0,0,0,0,0,0],[0,1,0,0,0,0,0,0,0,0,0],[0,1,0,0,0,0,0,0,0,0,0]]C2601038	bolasterone 124 dose	[[0,0,0,1,0,0,0,0,0,0,0]]C1513457	monoclonal antibody nr co 04,moab nr co 04	[[0,0,0,0,0,0,0,0,0,1,0],[0,0,0,0,0,0,0,0,0,1,0]]C0894437	r isomer thiazolidine 4 carboxylic acid	[[0,1,0,0,0,0,0,0,0,0,0]]C0639958	darutoside	[[0,1,0,0,0,0,0,0,0,0,0]]C0284843	n octyl sulfate,n octylsulfate	[[0,1,0,0,0,0,0,0,0,0,0],[0,1,0,0,0,0,0,0,0,0,0]]C3177770	6 o galloyl 1 5 anhydro d glucitol,ginnalin b	[[0,1,0,0,0,0,0,0,0,0,0],[0,1,0,0,0,0,0,0,0,0,0]]C0284841	ryoxon	[[0,1,0,0,0,0,0,0,0,0,0]]C3177772	2 o galloyl 1 5 anhydro d glucitol,ginnalin c	[[0,1,0,0,0,0,0,0,0,0,0],[0,1,0,0,0,0,0,0,0,0,0]]C3177775	1 3 dihydroxyeudesman 11 13 en 6 12 olide,1 3 dihydroxy eeo	[[0,1,0,0,0,0,0,0,0,0,0],[0,1,0,0,0,0,0,0,0,0,0]]C3177774	3 5 4 trihydroxystilbene 3 5 o diglucopyranoside,3 4 5 trihydroxy s2glc	[[0,1,0,0,0,0,0,0,0,0,0],[0,1,0,0,0,0,0,0,0,0,0]]C3169886	medication xxx 124 dose	[[0,0,0,1,0,0,0,0,0,0,0]]C3169887	medications 124 medication current	[[0,0,0,1,0,0,0,0,0,0,0]]C0719730	delta d3	[[0,0,0,0,0,0,0,1,0,0,0]]C3251935	n n di n propyl n1 methyl 2 4 nitrophenyl indol 3 yl glyoxylamide,pmni glyoxylamide	[[0,1,0,0,0,0,0,0,0,0,0],[0,1,0,0,0,0,0,0,0,0,0]]C1515378	recombinant hematopoietic growth factor	[[0,0,0,0,0,0,0,0,0,1,0]]C1871550	3 but 2 ynyl 5 methyl 2 piperazin 1 yl 3 5 dihydro 4h imidazo 4 5 d pyridazin 4 one tosylate,e3024	[[0,1,0,0,0,0,0,0,0,0,0],[0,1,0,0,0,0,0,0,0,0,0]]C1723980	rolaids softchews	[[0,0,0,0,0,0,0,1,0,0,0]]C1985147	chlormerodrin 124 urine	[[0,0,0,1,0,0,0,0,0,0,0]]C1985148	chlormezanone 124 bld ser plas	[[0,0,0,1,0,0,0,0,0,0,0]]C1871558	nxl 103,nxl103	[[0,1,0,0,0,0,0,0,0,0,0],[0,1,0,0,0,0,0,0,0,0,0]]C1999635	circulat	[[0,1,0,0,0,0,0,0,0,0,0]]C1631648	duotan pd	[[0,0,0,0,0,0,0,1,0,0,0]]C2925040	fentanyl norfentanyl 124 urine	[[0,0,0,1,0,0,0,0,0,0,0]]C2347779	clevidipine butyrate	[[1,0,0,0,0,0,0,0,0,1,0]]C0526897	s 2 thiopyridyl mercaptopropionohydrazide,3 2 pyridyldithio propionic acid hydrazide,tpmph	[[0,1,0,0,0,0,0,0,0,0,0],[0,1,0,0,0,0,0,0,0,0,0],[0,1,0,0,0,0,0,0,0,0,0]]C0526892	endo n 8 methyl 8 azabicyclo 3 2 1 oct 3 yl 2 3 dihydro 2 oxo 1h benzimidazol 1 carboxamide,n 8 methyl 8 azabicyclo 3 2 1 oct 3 yl 2 3 dihydro 2 oxo 1h benzimidazole 1 carboxamide,itasetron	[[0,1,0,0,0,0,0,0,0,0,0],[0,1,0,0,0,0,0,0,0,0,0],[0,1,0,0,0,0,0,0,0,1,0]]C1743810	cis 3 dehydrocholanoyliden l tartrate diammineplatinum ii,3 dhd l t dapt	[[0,1,0,0,0,0,0,0,0,0,0],[0,1,0,0,0,0,0,0,0,0,0]]C0613324	blankophor bkl,c i fluorescent brightener 200	[[0,1,0,0,0,0,0,0,0,0,0],[0,1,0,0,0,0,0,0,0,0,0]]C2347772	cintazone	[[0,0,0,0,0,0,0,0,0,1,0]]C0613322	n ethylacetanilide	[[0,1,0,0,0,0,0,0,0,0,0]]C0613323	n methylacetanilide	[[0,1,0,0,0,0,0,0,0,0,0]]C0613321	n guanin 8 yl n acetyl 2 aminofluorene,8 n 2 fluorenylacetoamido guanine	[[0,1,0,0,0,0,0,0,0,0,0],[0,1,0,0,0,0,0,0,0,0,0]]C0994443	iem 1556	[[0,1,0,0,0,0,0,0,0,0,0]]C0994442	12 13 dihydro 1 11 dihydroxy 5h indolo 2 3 a pyrrolo 3 4 c carbazole 5 7 6h dione	[[0,1,0,0,0,0,0,0,0,0,0]]C0994441	2 carbamoyl 4 4 fluorophenyl 5 2 4 3 fluorobenzylidene piperidine 1 yl ethyl thiazole	[[0,1,0,0,0,0,0,0,0,0,0]]C1307887	streptomyces fradiae tlrd protein	[[0,1,0,0,0,0,0,0,0,0,0]]C0608644	polyrane,polyether urethane terephthalate	[[0,1,0,0,0,0,0,0,0,0,0],[0,1,0,0,0,0,0,0,0,0,0]]C0608647	roseorubicins	[[0,1,0,0,0,0,0,0,0,0,0]]C0608640	maltosylamine,4 o alpha d glucopyranosyl d glucopyranosylamine	[[0,1,0,0,0,0,0,0,0,0,0],[0,1,0,0,0,0,0,0,0,0,0]]C1509464	polyoxyl 40 castor oil	[[0,0,1,0,0,0,0,0,0,0,0]]C3256377	macrogel	[[0,0,1,0,0,0,0,0,0,0,0]]C0665921	5 fluoro 3 1 2 1 methyl 1h pyrazol 4 yl ethyl 4 piperidinyl 1h indole	[[0,1,0,0,0,0,0,0,0,0,0]]C2929637	ethylnicotinate hexylnicotinate thurfyl salicylate	[[0,0,0,0,0,0,0,1,0,0,0]]C0665923	ly 302148	[[0,1,0,0,0,0,0,0,0,0,0]]C2929635	dicyclomine doxylamine vitamin b6	[[0,0,0,0,0,0,0,1,0,0,0]]C2929632	ascorbic acid beta carotene folic acid iodine magnesium sulfate manganese sulfate niacinamide pantothenate riboflavin thiamine vitamin a vitamin b 12 vitamin b6 vitamin d vitamin e zinc sulfate	[[0,0,0,0,0,0,0,1,0,0,0]]C0050302	a 49816	[[0,1,0,0,0,0,0,0,0,0,0]]C2929630	ergocalciferol lecithin	[[0,0,0,0,0,0,0,1,0,0,0]]C0665926	ly 306258	[[0,1,0,0,0,0,0,0,0,0,0]]C0023082	lasalocid,2r 2alpha 2s 3r 4s 5s 7r 3s 5s 5alpha 6beta 6 7 5 ethyl 5 5 ethyltetrahydro 5 hydroxy 6 methyl 2h pyran 2 yl tetrahydro 3 methyl 2 furanyl 4 hydroxy 3 5 dimethyl 6 oxononyl 2 hydroxy 3 methyl benzoic acid,2r 2alpha 2s 3r 4s 5s 7r 3s 5s 5alpha 6beta 6 7 5 ethyl 5 5 ethyltetrahydro 5 hydroxy 6 methyl 2h pyran 2 yl tetrahydro 3 methyl 2 furyl 4 hydroxy 3 5 dimethyl 6 oxononyl 3 methylsalicylic acid,ionophore x 537a	[[0,0,0,0,0,0,0,0,0,1,1],[0,0,0,0,0,0,0,0,0,0,1],[0,0,0,0,0,0,0,0,0,1,0],[0,0,0,0,0,0,0,0,0,1,0]]C0137351	pmhcr	[[0,1,0,0,0,0,0,0,0,0,0]]C2929638	dihydroxyaluminum sodium carbonate dimethicone	[[0,0,0,0,0,0,0,1,0,0,0]]C0050308	sarafloxacin	[[0,0,0,0,0,0,1,0,0,1,0]]C0723108	robinul forte	[[0,0,0,0,0,0,0,0,0,1,0]]C0380951	tetrakis 2 3 trifluoromethyl phenyl aminonicotinatodicopper ii,copper ii 2 niflumate 4	[[0,1,0,0,0,0,0,0,0,0,0],[0,1,0,0,0,0,0,0,0,0,0]]C1137286	2 2 4 trimethylhexanediisocyanate	[[0,1,0,0,0,0,0,0,0,0,0]]C0754250	4 iodine benzo b thiophene 2 carboxamidine,4 iodobenzo b thiophene 2 carboxamidine	[[0,1,0,0,0,0,0,0,0,0,0],[0,1,0,0,0,0,0,0,0,0,0]]C0723103	robaxin 750	[[0,0,0,0,0,0,1,1,0,0,0]]C0723100	robafen pe	[[0,0,0,0,0,0,0,1,0,0,0]]C0723101	robafen pediatric cough cold	[[0,0,0,0,0,0,0,1,0,0,0]]C0723106	robichem dm	[[0,0,0,0,0,0,0,1,0,0,0]]C0723107	robichem pe	[[0,0,0,0,0,0,0,1,0,0,0]]C0723104	robichem	[[0,0,0,0,0,0,0,1,0,0,0]]C0723105	robichem ac	[[0,0,0,0,0,0,0,1,0,0,0]]C2946150	dermafoam e	[[0,0,0,0,0,0,0,1,0,0,0]]C1137284	ly 329332,ly329332	[[0,1,0,0,0,0,0,0,0,0,0],[0,1,0,0,0,0,0,0,0,0,0]]C0169123	2 6 dimethylclonidine,2 6 dmc,2 6 dimethyl clonidine	[[0,1,0,0,0,0,0,0,0,0,0],[0,1,0,0,0,0,0,0,0,0,0],[0,1,0,0,0,0,0,0,0,0,0]]C1136745	4 n butyl 1 4 2 methylphenyl 4 oxo 1 butyl piperidine hydrogen chloride,ac 42,ac42	[[0,1,0,0,0,0,0,0,0,0,0],[0,1,0,0,0,0,0,0,0,0,0],[0,1,0,0,0,0,0,0,0,0,0]]C0722858	propecia,propeshia,merck sharp dohme brand 1 of finasteride,merck frosst brand 1 of finasteride,merck brand 1 of finasteride,msd brand of finasteride	[[0,0,0,0,0,0,1,1,0,0,0],[0,0,0,0,0,0,0,0,0,1,0],[0,1,0,0,0,0,0,0,0,0,0],[0,1,0,0,0,0,0,0,0,0,0],[0,1,0,0,0,0,0,0,0,0,0],[0,1,0,0,0,0,0,0,0,0,0]]C0722857	propatuss expectorant	[[0,0,0,0,0,0,0,1,0,0,0]]C0722856	propan	[[0,0,0,0,0,0,1,1,0,0,0]]C0722855	propacet 100	[[0,0,0,0,0,0,0,1,0,0,0]]C0722854	propa p h skin cleanser normal sensitive	[[0,0,0,0,0,0,0,1,0,0,0]]C0722852	propa p h foaming face wash	[[0,0,0,0,0,0,0,1,0,0,0]]C0722851	propa p h acne mask	[[0,0,0,0,0,0,0,1,0,0,0]]C0722850	propa p h	[[0,0,0,0,0,0,0,1,0,0,0]]C0623264	md 220661	[[0,1,0,0,0,0,0,0,0,0,0]]C0623266	aaptamine,8 9 dimethoxy 1h benzo de 1 6 naphthyridine	[[0,1,0,0,0,0,0,0,0,0,0],[0,1,0,0,0,0,0,0,0,0,0]]C0602099	onychofissan tincture,2 hydroxybenzoic acid compd with 8 quinolinol mixt with n n bis 2 hydroxyethyl 10 undecenamide 2 2 methylenebis 4 chlorophenol	[[0,1,0,0,0,0,0,0,0,0,0],[0,1,0,0,0,0,0,0,0,0,0]]C0623260	5 6 dimethylbenz a phenazine,7r 7alpha 8beta 10beta 5 6 dimethyl benzo a phenazine,5 6 dimethylbenzo a phenazine	[[0,1,0,0,0,0,0,0,0,0,0],[0,1,0,0,0,0,0,0,0,0,0],[0,1,0,0,0,0,0,0,0,0,0]]C0070233	penicillin n,adicillin,cephalosporin n,d 4 amino 4 carboxybutyl penicillanic acid	[[0,1,0,0,0,0,0,0,0,0,0],[0,1,0,0,0,0,0,0,0,1,0],[0,1,0,0,0,0,0,0,0,0,0],[0,1,0,0,0,0,0,0,0,0,0]]C0623263	md 220662	[[0,1,0,0,0,0,0,0,0,0,0]]C2911818	central muscle relaxant,muscle relaxant	[[0,0,0,0,0,0,0,0,0,0,1],[0,0,0,0,0,0,0,0,0,0,1]]C3253371	3 oxoolean 12 en 27 oic acid,3 oola cpd	[[0,1,0,0,0,0,0,0,0,0,0],[0,1,0,0,0,0,0,0,0,0,0]]C0070238	penicilloic acid	[[0,1,0,0,0,0,0,0,0,0,0]]C0602094	2 4 carboxystyryl 5 nitro 1 vinylimidazole	[[0,1,0,0,0,0,0,0,0,0,0]]C1170657	tetracon	[[0,0,0,0,0,0,0,1,0,0,0]]C0073766	s 26308	[[0,1,0,0,0,0,0,0,0,0,0]]C0073764	s 25930,9 fluoro 6 7 dihydro 5 8 dimethyl 1 oxo 1h 5h benzo ij quinolizine 2 carboxylic acid	[[0,1,0,0,0,0,0,0,0,0,0],[0,1,0,0,0,0,0,0,0,0,0]]C0073765	s 25932,9 fluoro 6 7 dihydro 8 1h imidazol 1 yl 5 methyl 1 oxo 1h 5h benzo ij quinolizine 2 carboxylic acid	[[0,1,0,0,0,0,0,0,0,0,0],[0,1,0,0,0,0,0,0,0,0,0]]C0073762	s 24623	[[0,1,0,0,0,0,0,0,0,0,0]]C0073761	s 2441	[[0,1,0,0,0,0,0,0,0,0,0]]C0073768	s 312,methyl ester 4 7 dihydro 6 methyl 3 2 methylpropyl 4 3 nitrophenyl thieno 2 3 b pyridine 5 carboxylic acid	[[0,1,0,0,0,0,0,0,0,0,0],[0,1,0,0,0,0,0,0,0,0,0]]C1876023	magnacet	[[0,0,0,0,0,0,0,1,0,0,0]]C0068200	n nitrosobis 2 oxobutyl amine,1 1 nitrosoimino bis 2 butanone,n bob	[[0,1,0,0,0,0,0,0,0,0,0],[0,1,0,0,0,0,0,0,0,0,0],[0,1,0,0,0,0,0,0,0,0,0]]C1257705	amylbarb sodium,protea brand of amobarbital sodium	[[0,1,0,0,0,0,0,0,0,0,0],[0,1,0,0,0,0,0,0,0,0,0]]C1257704	amsal,adams brand of amobarbital sodium,adams brand of amorbarbital	[[0,1,0,0,0,0,0,0,0,0,0],[0,1,0,0,0,0,0,0,0,0,0],[0,1,0,0,0,0,0,0,0,0,0]]C0289194	manganese poly n n bis 2 2 ethylenedioxydiethylenenitrilo n beta dihydroxypropane carbonylmethylene ethylenediamine n n diacetate,mn poly edta eoea dp	[[0,1,0,0,0,0,0,0,0,0,0],[0,1,0,0,0,0,0,0,0,0,0]]C0289195	iopiperidol	[[0,1,0,0,0,0,0,0,0,0,0]]C0289192	carboxylmethyl dextran gadopentetate dimeglumine	[[0,1,0,0,0,0,0,0,0,0,0]]C0878095	tomudex,arkomedika brand of raltitrexed,astrazeneca brand of raltitrexed,zeneca brand of raltitrexed	[[0,0,0,0,1,0,0,0,0,0,0],[0,1,0,0,0,0,0,0,0,0,0],[0,1,0,0,0,0,0,0,0,0,0],[0,1,0,0,0,0,0,0,0,0,0]]C2936050	6 hydroxy 1 2 3 4 tetrahydronaphthalene 2 carboxylic acid 1 4 3 hydroxyphenyl dimethylpiperidinylmethyl methylpropyl amide,6 hydroxy tcahda	[[0,1,0,0,0,0,0,0,0,0,0],[0,1,0,0,0,0,0,0,0,0,0]]C1257702	theophyllin eda ratiopharm,ratiopharm brand of aminophylline,theophyllin edaratiopharm	[[0,1,0,0,0,0,0,0,0,0,0],[0,1,0,0,0,0,0,0,0,0,0],[0,1,0,0,0,0,0,0,0,0,0]]C0638289	oxymorphone 6 spirohydantoin,oxymorphone 6 alpha spirohydantoin	[[0,1,0,0,0,0,0,0,0,0,0],[0,1,0,0,0,0,0,0,0,0,0]]C1257709	isoamitil sedante,hosbon brand of amorbarbital	[[0,1,0,0,0,0,0,0,0,0,0],[0,1,0,0,0,0,0,0,0,0,0]]C1257708	eunoctal,houde brand of amobarbital sodium	[[0,1,0,0,0,0,0,0,0,0,0],[0,1,0,0,0,0,0,0,0,0,0]]C2987005	nilotinib hydrochloride anhydrous	[[0,0,0,0,0,0,0,0,0,1,0]]C2987004	volinanserin,r 2 3 dimethoxyphenyl 1 2 4 fluorophenyl ethyl piperidin 4 yl methanol	[[0,0,0,0,0,0,0,0,0,1,0],[0,0,0,0,0,0,0,0,0,1,0]]C2987007	selumetinib sulfate,selumetinib sulphate,sulfate 1 1 5 4 bromo 2 chlorophenyl amino 4 fluoro n 2 hydroxyethoxy 1 methyl 1h benzimidazole 6 carboxamide,sulphate 1 1 1h benzimidazole 6 carboxamide 5 4 bromo 2 chlorophenyl amino 4 fluoro n 2 hydroxyethoxy 1 methyl,5 4 bromo 2 chlorophenyl amino 4 fluoro n 2 hydroxyethoxy 1 methyl 1h benzimidazole 6 carboxamide sulfate,5 4 bromo 2 chlorophenyl amino 4 fluoro n 2 hydroxyethoxy 1 methyl 1h benzimidazole 6 carboxamide sulphate	[[0,0,0,0,0,0,0,0,0,1,0],[0,0,0,0,0,0,0,0,0,1,0],[0,0,0,0,0,0,0,0,0,1,0],[0,0,0,0,0,0,0,0,0,1,0],[0,0,0,0,0,0,0,0,0,1,0],[0,0,0,0,0,0,0,0,0,1,0]]C2987006	saracatinib difumarate,n 5 chloro 1 3 benzodioxol 4 yl 7 2 4 methylpiperazin 1 yl ethoxy 5 tetrahydro 2h pyran 4 yloxy quinazolin 4 amine di 2e but 2 enedioate,2e 2 butenedioate 1 2 n 5 chloro 1 3 benzodioxol 4 yl 7 2 4 methyl 1 piperazinyl ethoxy 5 tetrahydro 2h pyran 4 yl oxy 4 quinazolinamine	[[0,0,0,0,0,0,0,0,0,1,0],[0,0,0,0,0,0,0,0,0,1,0],[0,0,0,0,0,0,0,0,0,1,0]]C1098438	hmr 3562,hmr3562	[[0,1,0,0,0,0,0,0,0,0,0],[0,1,0,0,0,0,0,0,0,0,0]]C2987000	ozarelix,ac d nal 2 d phe 4cl d pal 3 ser nme tyr d hcit nle arg pro d ala nh2,lhrh antagonist spi 153	[[0,1,0,0,0,0,0,0,0,1,0],[0,1,0,0,0,0,0,0,0,0,0],[0,0,0,0,0,0,0,0,0,1,0]]C2002429	rat sgf29 protein	[[0,1,0,0,0,0,0,0,0,0,0]]C0718079	tazarotene topical	[[0,0,0,0,0,0,1,0,0,0,0]]C1098434	1 methylethyl ester 1r 1alpha z 2beta 1e 3r 3alpha 5alpha 7 3 5 dihydroxy 2 3 hydroxy 4 3 trifluoromethyl phenoxy 1 butenyl cyclopentyl 5 heptenoic acid	[[0,1,0,0,0,0,0,0,0,0,0]]C1098436	al 6221,al6221	[[0,1,0,0,0,0,0,0,0,0,0],[0,1,0,0,0,0,0,0,0,0,0]]C0057654	di halo,1 bromo 3 chloro 5 5 dimethylhydantoin	[[0,1,0,0,0,0,0,0,0,0,0],[0,1,0,0,0,0,0,0,0,0,0]]C2987008	varlitinib tosylate,4 n 3 chloro 4 thiazol 2 ylmethoxy phenyl 6 n 4r 4 methyl 4 5 dihydrooxazol 2 yl quinazoline 4 6 diamine bis 4 methylbenzenesulphonate,4 methylbenzenesulphonate 1 2 n sup 4 3 chloro 4 2 thiazolylmethoxy phenyl n sup 6 4r 4 5 dihydro 4 methyl 2 oxazolyl 4 6 quinazolinediamine	[[0,0,0,0,0,0,0,0,0,1,0],[0,0,0,0,0,0,0,0,0,1,0],[0,0,0,0,0,0,0,0,0,1,0]]C2002420	thuggacin a	[[0,1,0,0,0,0,0,0,0,0,0]]C0042397	vasoconstrictor agents,vasoactive agonists,vasopressor agents,vasoconstrictors,vasoconstrictor drugs,vasopressor	[[0,1,0,0,0,0,0,0,0,0,0],[0,1,0,0,0,0,0,0,0,0,0],[0,1,0,0,0,0,0,0,0,0,0],[1,1,0,0,0,0,1,0,0,0,0],[0,1,0,0,0,0,0,0,0,0,0],[0,0,0,0,0,0,1,0,0,0,0]]C0304650	ophthalmic preparation,drug ophthalmic,drug eye	[[0,0,0,0,0,0,1,0,0,0,0],[0,0,0,0,0,0,1,0,0,0,0],[0,0,0,0,0,0,1,0,0,0,0]]C0304653	nasal dosage form product,nasal preparation	[[1,0,0,0,0,0,0,0,0,0,0],[1,0,0,0,0,0,0,0,0,0,0]]C0042394	vasoactive antagonists	[[0,1,0,0,0,0,0,0,0,0,0]]C0604007	2 2 thiobis	[[0,1,0,0,0,0,0,0,0,0,0]]C0604006	2 2 thiobis,2 2 thiobis 6 1 1 dimethylethyl 4 methyl phenol	[[0,1,0,0,0,0,0,0,0,0,0],[0,1,0,0,0,0,0,0,0,0,0]]C0304657	apraclonidine hydrochloride,p aminoclonidine hydrochloride,aplonidine hydrochloride,monohydrochloride 2 6 dichloro n sup 1 4 5 dihydro 1h imidazol 2 yl 1 4 benzenediamine	[[0,1,0,0,0,0,0,1,0,1,0],[1,0,0,0,0,0,0,0,0,1,0],[1,0,0,0,0,0,0,0,0,0,0],[0,0,0,0,0,0,0,0,0,1,0]]C0604004	nsc 146268	[[0,1,0,0,0,0,0,0,0,0,0]]C0304658	flurbiprofen sodium,2 fluoro alpha methyl 4 diphenylacetic acid sodium salt	[[0,1,1,0,0,0,0,1,0,1,0],[0,0,0,0,0,0,0,0,0,1,0]]C0671625	2 2 ethoxyethoxy ethyl furfuryl ether,eefe ether	[[0,1,0,0,0,0,0,0,0,0,0],[0,1,0,0,0,0,0,0,0,0,0]]C0717898	magnesium amino acid chelate	[[1,0,0,0,0,0,0,1,0,0,0]]C0042398	nasal decongestants,nasal decongestant preparation	[[0,1,0,0,0,0,1,0,0,0,1],[1,0,0,0,0,0,0,0,0,0,0]]C0953091	cis isomer chinoin 127	[[0,1,0,0,0,0,0,0,0,0,0]]C0953093	isomer beclobrate	[[0,1,0,0,0,0,0,0,0,0,0]]C0953092	sodium salt pyrrolidine dithiocarbamic acid	[[0,1,0,0,0,0,0,0,0,0,0]]C0953094	9 2 bromoacetamidobenzyl adenine hydrobromide	[[0,1,0,0,0,0,0,0,0,0,0]]C1615605	osi 930,osi930,tyrosine kinase inhibitor osi 930	[[0,1,0,0,0,0,0,0,0,0,0],[0,1,0,0,0,0,0,0,0,0,0],[0,0,0,0,0,0,0,0,0,1,0]]C0953099	cinepazet maleate	[[0,1,0,0,0,0,0,0,0,0,0]]C0722272	optigene 3	[[0,0,0,0,0,0,0,1,0,0,0]]C0722271	optigene,pfeiffer brand of tetrahydrozoline hydrochloride	[[0,1,0,0,0,0,0,0,0,0,0],[0,1,0,0,0,0,0,0,0,0,0]]C0722270	opticyl	[[0,0,0,0,0,0,0,0,0,1,0]]C0247605	n n 2 adamantyloxy carbonyl alpha methyltryptophyl n 2 4 chlorophenyl ethyl glycine,acycg	[[0,1,0,0,0,0,0,0,0,0,0],[0,1,0,0,0,0,0,0,0,0,0]]C0958973	1 r2 r s isomer 4 trifluoromethylphenyl 2 biphenylyl 3 hydroxypropionic acid	[[0,1,0,0,0,0,0,0,0,0,0]]C0958972	1 r2 r r isomer 4 trifluoromethylphenyl 2 biphenylyl 3 hydroxypropionic acid	[[0,1,0,0,0,0,0,0,0,0,0]]C0958971	2 r s 3 isomer 4 trifluoromethylphenyl 2 biphenylyl 3 hydroxypropionic acid	[[0,1,0,0,0,0,0,0,0,0,0]]C0958970	2 r r 3 isomer 4 trifluoromethylphenyl 2 biphenylyl 3 hydroxypropionic acid	[[0,1,0,0,0,0,0,0,0,0,0]]C0958977	1 r2 r s isomer 4 thioanisyl 2 biphenylyl 3 hydroxypropionic acid	[[0,1,0,0,0,0,0,0,0,0,0]]C0958976	1 s2 r r isomer 4 thioanisyl 2 biphenylyl 3 hydroxypropionic acid	[[0,1,0,0,0,0,0,0,0,0,0]]C0958975	1 r2 r r isomer 4 thioanisyl 2 biphenylyl 3 hydroxypropionic acid	[[0,1,0,0,0,0,0,0,0,0,0]]C0958974	2 r s 3 isomer 4 thioanisyl 2 biphenylyl 3 hydroxypropionic acid	[[0,1,0,0,0,0,0,0,0,0,0]]C0958979	hydrochloride s 20244	[[0,1,0,0,0,0,0,0,0,0,0]]C0958978	1 s2 r s isomer 4 thioanisyl 2 biphenylyl 3 hydroxypropionic acid	[[0,1,0,0,0,0,0,0,0,0,0]]C0968661	sodium salt 4 4 dinitro 2 2 stilbenedisulfonic acid	[[0,1,0,0,0,0,0,0,0,0,0]]C2962730	wal dryl d	[[0,0,0,0,0,0,0,1,0,0,0]]C0676166	cmx 13	[[0,1,0,0,0,0,0,0,0,0,0]]C0951835	mepiprazole dihydrochloride,1 3 chlorophenyl 4 2 5 methylpyrazol 3 yl ethyl piperazine dihydrochloride	[[0,1,0,0,0,0,0,0,0,0,0],[0,1,0,0,0,0,0,0,0,0,0]]C0951837	metamizil hydrochloride,hydrochloride beta diethylaminopropylbenzilic acid ester	[[0,1,0,0,0,0,0,0,0,0,0],[0,1,0,0,0,0,0,0,0,0,0]]C1872408	oenostacin	[[0,1,0,0,0,0,0,0,0,0,0]]C0617954	spiro 17 oxiranyl delta 4 androsten 3 one,soao	[[0,1,0,0,0,0,0,0,0,0,0],[0,1,0,0,0,0,0,0,0,0,0]]C0676169	huokt3gamma1	[[0,1,0,0,0,0,0,0,0,0,0]]C0617953	spiro 17 beta oxiranyl delta 4 androsten 3 one	[[0,1,0,0,0,0,0,0,0,0,0]]C2745705	2 1 hydroxyundecyl 1 4 nitrophenylamino 6 phenyl 6 7a dihydro 1h pyrrolo 3 4 b pyridine 5 7 2h 4ah dione,a12b4c3	[[0,1,0,0,0,0,0,0,0,0,0],[0,1,0,0,0,0,0,0,0,0,0]]C2709995	ivomec plus	[[0,0,0,0,0,0,0,1,0,0,0]]C0080983	3 methoxysampangine,3 methoxy 7h naphtho 1 2 3 ij 2 7 naphthyridin 7 one	[[0,1,0,0,0,0,0,0,0,0,0],[0,1,0,0,0,0,0,0,0,0,0]]C0385679	gr 203040,gr203040	[[0,1,0,0,0,0,0,0,0,0,0],[0,1,0,0,0,0,0,0,0,0,0]]C2709990	hepatitis b virus antigen	[[0,0,0,0,0,0,0,0,0,1,0]]C0385674	u 103017	[[0,1,0,0,0,0,0,0,0,0,0]]C1994502	phenmetrazine 124 milk	[[0,0,0,1,0,0,0,0,0,0,0]]C1994501	phenmetrazine 124 meconium	[[0,0,0,1,0,0,0,0,0,0,0]]C1994500	phenmetrazine 124 hair	[[0,0,0,1,0,0,0,0,0,0,0]]C1994507	phenobarbital 124 body fluid	[[0,0,0,1,0,0,0,0,0,0,0]]C1994506	phenobarbital 124 bld ser plas	[[0,0,0,1,0,0,0,0,0,0,0]]C1994505	phenmetrazine 124 vitreous fluid	[[0,0,0,1,0,0,0,0,0,0,0]]C0385673	3 3 4 cyanophenyl sulfonamido phenyl cyclopropyl methyl 4 hydroxy 2h cycloocta b pyran 2 one	[[0,1,0,0,0,0,0,0,0,0,0]]C0072497	protoporphyrin ix	[[1,1,0,0,0,0,0,0,0,0,0]]C0082338	dolastatin 10,isodolastatin 10,2s 1 1r r 2s 2r 1s 2s 3 r n n dimethyl l valyl n 2 methoxy 4 2 1 methoxy 2 methyl 3 oxo 3 2 phenyl 1 2 thiazolyl ethyl amino propyl 1 pyrrolidinyl 1 1 methylpropyl 4 oxobutyl n methyl l valinamide	[[0,0,0,0,0,0,0,0,0,1,0],[0,1,0,0,0,0,0,0,0,0,0],[0,0,0,0,1,0,0,0,0,0,0]]C0072495	protopine,4 6 7 14 tetrahydro 5 methyl bis 1 3 benzodioxolo 4 5 c 5 6 g azecin 13 5h one,fumarine	[[1,1,0,0,0,0,0,0,0,0,0],[0,1,0,0,0,0,0,0,0,0,0],[0,1,0,0,0,0,0,0,0,0,0]]C0072494	protopanaxadiol	[[0,1,0,0,0,0,0,0,0,0,0]]C0890828	mono l tartrate r r r isomer 1 4 nitrophenyl 2 aminopropane 1 3 diol	[[0,1,0,0,0,0,0,0,0,0,0]]C3162522	perox 400	[[0,0,0,0,0,0,0,1,0,0,0]]C0720792	gynodiol	[[0,0,0,0,0,0,0,1,0,0,0]]C0658481	8 methyl 2 4 morpholinyl 7 1 naphthalenylmethoxy 4h 1 benzopyran 4 one	[[0,1,0,0,0,0,0,0,0,0,0]]C0082335	dolabella auricularia dolabellanin a protein,dolabellanin a	[[0,1,0,0,0,0,0,0,0,0,0],[0,1,0,0,0,0,0,0,0,0,0]]C0082337	dolastatin 1	[[0,1,0,0,0,0,0,0,0,0,0]]C0591109	aridil	[[0,0,0,0,0,0,0,1,0,0,0]]C0591108	arelix	[[0,0,0,0,0,0,0,1,0,0,0]]C2924714	benzylpiperazine 124 bld ser plas	[[0,0,0,1,0,0,0,0,0,0,0]]C2924715	benzylpiperazine 124 urine	[[0,0,0,1,0,0,0,0,0,0,0]]C0134078	org 3770	[[0,1,0,0,0,0,0,0,0,0,0]]C0134079	org 6001	[[0,1,0,0,0,0,0,0,0,0,0]]C0591101	anusol hc	[[0,0,0,0,0,0,1,1,0,0,0]]C0591100	anugesic hc	[[0,0,0,0,0,0,1,0,0,0,0]]C0134076	org 31550	[[0,1,0,0,0,0,0,0,0,0,0]]C0591102	aprinox,knoll brand of bendroflumethiazide	[[0,0,0,0,0,0,0,1,0,0,0],[0,1,0,0,0,0,0,0,0,0,0]]C0591105	apsolol	[[0,0,0,0,0,0,0,1,0,0,0]]C0719080	caladryl for kids	[[0,0,0,0,0,0,0,1,0,0,0]]C0069158	nucleoticidin	[[0,1,0,0,0,0,0,0,0,0,0]]C0134073	org 30850,org 30850 ant	[[0,1,0,0,0,0,0,0,0,0,0],[0,1,0,0,0,0,0,0,0,0,0]]C0892077	lithium salt caprylic acid	[[0,1,0,0,0,0,0,0,0,0,0]]C0892075	e isomer cf 19415	[[0,1,0,0,0,0,0,0,0,0,0]]C0892074	z isomer cf 19415	[[0,1,0,0,0,0,0,0,0,0,0]]C2935168	rg 1678,rg1678	[[0,1,0,0,0,0,0,0,0,0,0],[0,1,0,0,0,0,0,0,0,0,0]]C1260522	becotide easi breathe	[[0,0,0,0,0,0,0,1,0,0,0]]C0074262	sedalipid,magnesium pyridoxal phosphate glutamine complex,magnesium pyridoxal 5 phosphate glutamate	[[0,1,0,0,0,0,0,0,0,0,0],[0,1,0,0,0,0,0,0,0,0,0],[0,1,0,0,0,0,0,0,0,0,0]]C0805087	amyl ether	[[0,0,0,1,0,0,0,0,0,0,0]]C0524639	urofollitropin,urofollitrophin,human urine follicle stimulating hormone,urinary human fsh	[[0,0,0,0,0,0,1,1,0,1,0],[1,0,0,0,0,0,1,0,0,0,0],[0,1,0,0,0,0,0,0,0,0,1],[0,1,0,0,0,0,0,0,0,0,1]]C0524638	org 31338,org31338	[[0,1,0,0,0,0,0,0,0,0,0],[0,1,0,0,0,0,0,0,0,0,0]]C1120337	tyrphostin ag 1024,ag 1024,ag1024	[[0,1,0,0,0,0,0,0,0,0,0],[0,1,0,0,0,0,0,0,0,0,0],[0,1,0,0,0,0,0,0,0,0,0]]C1120333	2 2 4 6 tetrachlorobiphenyl	[[0,1,0,0,0,0,0,0,0,0,0]]C1120332	7 benzoyloxy 4 hydroxy 1 methoxy 2 4 heptadiene 1 6 dione,7 benzoyloxy 4 hydroxy 1 methoxy 2e 4z heptadiene 1 6 dione,7 b h m hd	[[0,1,0,0,0,0,0,0,0,0,0],[0,1,0,0,0,0,0,0,0,0,0],[0,1,0,0,0,0,0,0,0,0,0]]C1120331	7 benzoyloxy 6 oxo 2 4 heptadiene 1 4 olide,7 benzoyloxy 6 oxo 2 4z heptadiene 1 4 olide,7 b h olide	[[0,1,0,0,0,0,0,0,0,0,0],[0,1,0,0,0,0,0,0,0,0,0],[0,1,0,0,0,0,0,0,0,0,0]]C0254254	4 bromobenzenesulfonamide	[[0,1,0,0,0,0,0,0,0,0,0]]C0038838	superoxide dismutase,erythrocuprein,hemocuprein,superoxide superoxide oxidoreductase,cytocuprein,sod,super oxide dismutase	[[0,1,0,0,0,0,1,0,0,0,0],[0,0,0,0,0,0,0,0,0,0,1],[0,0,0,0,0,0,0,0,0,0,1],[0,0,0,0,0,0,0,0,0,0,1],[0,0,0,0,0,0,0,0,1,0,0],[0,0,0,0,0,0,1,0,1,0,0],[0,0,0,0,0,0,1,0,0,0,0]]C0718544	anolor dh5	[[0,0,0,0,0,0,0,1,0,0,0]]C0718545	anoquan	[[0,0,0,0,0,0,0,1,0,0,0]]C0718546	anorex sr	[[0,0,0,0,0,0,0,1,0,0,0]]C0718547	antacid	[[0,0,0,0,0,0,0,1,0,0,0]]C0718540	anexsia	[[0,0,0,0,0,0,1,1,0,0,0]]C0718541	angioscein	[[0,0,0,0,0,0,0,1,0,0,0]]C0721779	monistat 3	[[0,0,0,0,0,0,1,1,0,0,0]]C0718543	anolor 300	[[0,0,0,0,0,0,0,1,0,0,0]]C0721777	monafed dm	[[0,0,0,0,0,0,0,1,0,0,0]]C0721776	momentum tradename,momentum	[[0,0,0,0,0,0,0,1,0,0,0],[0,0,0,0,0,0,1,0,0,0,0]]C0721775	molypen	[[0,0,0,0,0,0,1,1,0,0,0]]C0721774	mollifene,pfieffer brand of carbamide peroxide	[[0,1,0,0,0,0,0,0,0,0,0],[0,1,0,0,0,0,0,0,0,0,0]]C0718548	antacid anti gas	[[0,0,0,0,0,0,0,1,0,0,0]]C0718549	antacid ds	[[0,0,0,0,0,0,0,1,0,0,0]]C0721771	moisturel	[[0,0,0,0,0,0,0,1,0,0,0]]C0719499	cooling gel	[[0,0,0,0,0,0,1,0,0,0,0]]C2684347	timothy grass pollen extract,phleum pratense pollen	[[0,0,0,0,0,0,0,1,0,0,0],[0,0,1,0,0,0,0,0,0,1,0]]C2684344	orchard grass pollen extract,dactylis glomerata pollen	[[0,0,0,0,0,0,0,1,0,0,0],[0,0,1,0,0,0,0,0,0,1,0]]C2684345	meadow fescue grass pollen extract,festuca pratensis pollen	[[0,0,0,0,0,0,0,1,0,0,0],[0,0,0,0,0,0,0,0,0,1,0]]C0077511	tuna ai,prolyl threonyl histidyl isoleucyl lysyl trptophyl glycyl aspartic acid,pro thr his ile lys trp gly asp	[[0,1,0,0,0,0,0,0,0,0,0],[0,1,0,0,0,0,0,0,0,0,0],[0,1,0,0,0,0,0,0,0,0,0]]C2684343	bermuda grass pollen extract,cynodon dactylon pollen	[[0,0,0,0,0,0,0,1,0,0,0],[0,0,1,0,0,0,0,0,0,1,0]]C2930049	chlorpheniramine methscopolamine phenylephrine	[[0,0,0,0,0,0,0,1,0,0,0]]C2684341	redtop grass pollen extract,agrostis gigantea pollen	[[0,0,0,0,0,0,0,1,0,0,0],[0,0,1,0,0,0,0,0,0,1,0]]C0719491	contac 12 hour allergy	[[0,0,0,0,0,0,0,1,0,0,0]]C0719490	contac 12 hour	[[0,0,0,0,0,0,0,1,0,0,0]]C2930045	potassium chloride potassium gluconate	[[0,0,0,0,0,0,0,1,0,0,0]]C2930044	corticotropin zinc hydroxide	[[0,0,0,0,0,0,0,1,0,0,0]]C0719495	contac severe cold and flu non drowsy	[[0,0,0,0,0,0,0,1,0,0,0]]C0719494	contac severe cold and flu maximum stength	[[0,0,0,0,0,0,0,1,0,0,0]]C0719497	conte pak 4	[[0,0,0,0,0,0,0,1,0,0,0]]C0719496	contac sinus	[[0,0,0,0,0,0,0,1,0,0,0]]C1873969	acetaminophen chlorpheniramine phenylephrine pyrilamine	[[0,0,0,0,0,0,0,1,0,0,1]]C1873968	acetaminophen chlorpheniramine phenylephrine	[[0,0,0,0,0,0,0,1,0,0,0]]C2357325	dextromethorphan levorphanol 124 urine	[[0,0,0,1,0,0,0,0,0,0,0]]C2357324	dextromethorphan 124 gastric fluid	[[0,0,0,1,0,0,0,0,0,0,0]]C1873965	acetaminophen chlorpheniramine dextromethorphan phenylephrine	[[0,0,0,0,0,0,0,1,0,0,0]]C1873964	acetaminophen chlorpheniramine codeine phenylephrine	[[0,0,0,0,0,0,0,1,0,0,1]]C1435406	2z 4e 5 5 6 dichloro 2 indolyl 2 methoxy n 4 2 2 6 6 tetramethylpiperidinoxy 2 4 pentadienamide,5 dci mtmp pa	[[0,1,0,0,0,0,0,0,0,0,0],[0,1,0,0,0,0,0,0,0,0,0]]C0612185	nitraminoacetic acid,n nitro glycine	[[0,1,0,0,0,0,0,0,0,0,0],[0,1,0,0,0,0,0,0,0,0,0]]C1873961	acetaminophen caffeine phenylpropanolamine salicylamide	[[0,0,0,0,0,0,0,1,0,0,1]]C1873960	acetaminophen caffeine phenylpropanolamine pyrilamine	[[0,0,0,0,0,0,0,1,0,0,0]]C1873963	acetaminophen calcium carbonate magnesium carbonate magnesium ox	[[0,0,0,0,0,0,0,0,0,0,1]]C1873962	acetaminophen calcium carbonate	[[0,0,0,0,0,0,0,1,0,0,1]]C2698682	oxytetracycline anhydrous	[[0,0,0,0,0,0,0,0,0,1,0]]C2698683	padre cmv fusion peptide vaccine	[[0,0,0,0,0,0,0,0,0,1,0]]C2585188	ophthalmic form trimethoprim	[[1,0,0,0,0,0,0,0,0,0,0]]C2698681	oxysonium iodide	[[0,0,0,0,0,0,0,0,0,1,0]]C2698686	parp inhibitor mk4827	[[0,0,0,0,0,0,0,0,0,1,0]]C1975003	trypsin 124 duodenal fluid	[[0,0,0,1,0,0,0,0,0,0,0]]C0759876	tetrachloropyrocatechol,tc pyrocatechol	[[0,1,0,0,0,0,0,0,0,0,0],[0,1,0,0,0,0,0,0,0,0,0]]C2585181	oropharyngeal form fluoride	[[1,0,0,0,0,0,0,0,0,0,0]]C0910481	lv9nb,cyclo gly lys val s 3 aminododecanoyl	[[0,1,0,0,0,0,0,0,0,0,0],[0,1,0,0,0,0,0,0,0,0,0]]C0724491	zithromax iv	[[0,0,0,0,0,0,1,1,0,0,0]]C0759874	tetrachloropyrocatechol methyl ester,tcpm ester	[[0,1,0,0,0,0,0,0,0,0,0],[0,1,0,0,0,0,0,0,0,0,0]]C2979248	clear eyes cooling comfort	[[0,0,0,0,0,0,0,1,0,0,0]]C0894445	isomer isamfazone	[[0,1,0,0,0,0,0,0,0,0,0]]C0656934	js 114	[[0,1,0,0,0,0,0,0,0,1,0]]C0759872	b 90063	[[0,1,0,0,0,0,0,0,0,0,0]]C0107161	brij 30,polyoxyethylene 4 lauryl ether	[[0,1,0,0,0,0,0,0,0,0,0],[0,1,0,0,0,0,0,0,0,0,0]]C0107162	brij 35,polyoxyethylene 23 lauryl ether,c12h25 och2ch2 23oh,dodecyl tricosaoxyethylene glycol ether,polyoxyethilene 23 lauryl ether	[[0,1,0,0,0,0,0,0,0,0,0],[0,1,0,0,0,0,0,0,0,0,0],[0,1,0,0,0,0,0,0,0,0,0],[0,1,0,0,0,0,0,0,0,0,0],[0,1,0,0,0,0,0,0,0,0,0]]C0724495	zofran odt	[[0,0,0,0,0,0,1,0,0,1,0]]C0656937	cancell	[[0,0,0,0,0,0,1,0,0,1,0]]C0604607	n beta naphthylaminomethyl l alanine	[[0,1,0,0,0,0,0,0,0,0,0]]C0105997	beta hydroxy beta ethyl phenylpropionamide,beta hepp,3 hydroxy 3 ethyl 3 phenylpropionamide	[[0,1,0,0,0,0,0,0,0,0,0],[0,1,0,0,0,0,0,0,0,0,0],[0,1,0,0,0,0,0,0,0,0,0]]C0383631	2 amino 5 phosphono 3 pentenoic acid,2 appa	[[0,1,0,0,0,0,0,0,0,0,0],[0,1,0,0,0,0,0,0,0,0,0]]C0383637	cadina 4 10 15 dien 3 one	[[0,1,0,0,0,0,0,0,0,0,0]]C0383635	rhizocticin b,2 valyl arginyl amino 5 phosphono 3 pentenoic acid	[[0,1,0,0,0,0,0,0,0,0,0],[0,1,0,0,0,0,0,0,0,0,0]]C1588409	polocaine with levonordefrin	[[0,0,0,0,0,0,0,1,0,0,0]]C1628324	vandetanib	[[0,0,0,0,0,0,0,0,0,1,0]]C0721199	kg fed	[[0,0,0,0,0,0,0,1,0,0,0]]C0721198	kg dal hd plus	[[0,0,0,0,0,0,0,1,0,0,0]]C0382947	disperse red 54	[[0,1,0,0,0,0,0,0,0,0,0]]C0382946	c i dr 54	[[0,1,0,0,0,0,0,0,0,0,0]]C0721193	k y plus nonoxynol 9	[[0,0,0,0,0,0,0,1,0,0,0]]C0721192	k vescent	[[0,0,0,0,0,0,0,1,0,0,0]]C0220281	l 680833,l 680 833	[[0,1,0,0,0,0,0,0,0,0,0],[0,1,0,0,0,0,0,0,0,0,0]]C0721190	k phos no 2	[[0,0,0,0,0,0,0,1,0,0,0]]C0721197	kg dal hd	[[0,0,0,0,0,0,0,1,0,0,0]]C0721196	kg hist dm	[[0,0,0,0,0,0,0,1,0,0,0]]C0721195	kcl 40	[[0,0,0,0,0,0,0,1,0,0,0]]C0981850	california hazelnut pollen allergenic extract	[[0,0,0,0,0,0,0,0,0,0,1]]C0981856	cattle epithelia allergenic extract	[[0,0,0,0,0,0,0,0,0,0,1]]C0981857	cedar allergenic extract	[[0,0,0,0,0,0,0,0,0,0,1]]C0981855	cat epithelium allergenic extract	[[0,0,0,0,0,0,0,0,0,0,1]]C0537134	benzyloxycarbonyl glu val asp ch2oc o 2 6 dichlorobenzene,z evd ch2 dcb	[[0,1,0,0,0,0,0,0,0,0,0],[0,1,0,0,0,0,0,0,0,0,0]]C0981858	cephalosporium acrimonium allergenic extract	[[0,0,0,0,0,0,0,0,0,0,1]]C2987666	antibiotic 503 3,antibiotic sf 767b	[[0,0,0,0,0,0,0,0,0,1,0],[0,0,0,0,0,0,0,0,0,1,0]]C0720820	havrix pediatric	[[0,0,0,0,0,0,0,1,0,0,0]]C0602879	551 d 1	[[0,1,0,0,0,0,0,0,0,0,0]]C0720822	head shoulders,head shoulders brand of zinc pyrithione,procter gamble brand of zinc pyrithione	[[0,0,0,0,0,0,1,1,0,0,0],[0,1,0,0,0,0,0,0,0,0,0],[0,1,0,0,0,0,0,0,0,0,0]]C0720823	head shoulders dry scalp	[[0,0,0,0,0,0,0,1,0,0,0]]C0720824	head shoulders intensive treatment	[[0,0,0,0,0,0,0,1,0,0,0]]C1881932	mushroom extract,abmk 22,agaricus blazei murril extract 22	[[0,0,0,0,0,0,0,0,0,1,0],[0,0,0,0,0,0,0,0,0,1,0],[0,0,0,0,0,0,0,0,0,1,0]]C1881931	murocainide	[[0,0,0,0,0,0,0,0,0,1,0]]C0720827	helidac,prometheus brand of bismuth subsalicylate	[[0,1,0,0,0,0,1,0,0,0,0],[0,1,0,0,0,0,0,0,0,0,0]]C0055098	cerastase f 4	[[0,1,0,0,0,0,0,0,0,0,0]]C0720829	prep hem	[[0,0,0,0,0,0,0,1,0,0,0]]C0063144	hydroxyisonobilin	[[0,1,0,0,0,0,0,0,0,0,0]]C0063140	hydroxyguanidine,2 hydroxyguanidine	[[0,1,0,0,0,0,0,0,0,0,0],[0,1,0,0,0,0,0,0,0,0,0]]C0076647	ti 233,diacetate s 1 5 aminoiminomethyl amino 2 5 dimethylamino 1 naphthalenyl sulfonyl amino 1 oxopentyl 4 1 1 dimethylethyl piperidine	[[0,1,0,0,0,0,0,0,0,0,0],[0,1,0,0,0,0,0,0,0,0,0]]C0076643	thyrotropin daunomycin conjugate,tsh dm	[[0,1,0,0,0,0,0,0,0,0,0],[0,1,0,0,0,0,0,0,0,0,0]]C2962048	mirtazapine 1 kg in 1 kg not applicable powder	[[0,0,1,0,0,0,0,0,0,0,0]]C0308832	diquel	[[0,0,0,0,0,0,0,0,0,1,0]]C0076648	tiadenol,2 2 1 10 decanediylbis thio bis ethanol,bis 2 hydroxyethylthio 1 10 decane,bis hydroxy 2 ethylthio 1 10 decane,thiadenol,tiadenolo,2 2 decamethylenedithio diethanol	[[0,0,0,0,0,0,0,1,0,0,0],[0,1,0,0,0,0,0,0,0,0,0],[0,1,0,0,0,0,0,0,0,0,0],[0,1,0,0,0,0,0,0,0,0,0],[0,1,0,0,0,0,0,0,0,0,0],[0,1,0,0,0,0,0,0,0,0,0],[0,1,0,0,0,0,0,0,0,0,0]]C0076649	tiadilon,cpd with l arginine 2 4 thiazolidinedicarboxylic acid,arginine tidiacicate,tidiacicate arginine salt	[[0,0,0,0,0,0,0,1,0,0,0],[0,1,0,0,0,0,0,0,0,0,0],[0,1,0,0,0,0,0,0,0,0,0],[0,1,0,0,0,0,0,0,0,0,0]]C0127161	mci 9038	[[0,1,0,0,0,0,0,0,0,0,0]]C1590229	tusnel pediatric	[[0,0,0,0,0,0,0,1,0,0,0]]C0290149	angiostatins,plg,kringles 1 4 of plasminogen	[[0,0,0,0,0,0,0,0,1,1,1],[0,0,0,0,0,0,0,0,0,1,0],[0,0,0,0,0,0,0,0,0,1,0]]C0605557	nioben,1 piperidino 3 4 n octylphenyl propan 3 one	[[0,1,0,0,0,0,0,0,0,0,0],[0,1,0,0,0,0,0,0,0,0,0]]C1590223	despec dm	[[0,0,0,0,0,0,0,1,0,0,0]]C1590220	ed flex	[[0,0,0,0,0,0,0,1,0,0,0]]C0290142	idremcinal,8 9 didehydro n demethyl 9 deoxo 6 deoxy 6 9 epoxy n 1 methylethyl erythromycin	[[0,1,0,0,0,0,0,0,0,0,0],[0,1,0,0,0,0,0,0,0,0,0]]C0290140	iodoproxyfan,3 1h imidazol 4 yl propyl 4 iodophenyl methyl ether,4 3 4 iodophenyl methoxy propyl 1h imidazole	[[0,1,0,0,0,0,0,0,0,0,0],[0,1,0,0,0,0,0,0,0,0,0],[0,1,0,0,0,0,0,0,0,0,0]]C0049050	5 aminomethylsalicylic acid	[[0,1,0,0,0,0,0,0,0,0,0]]C2979190	wal tussin cold congestion capsule	[[0,0,0,0,0,0,0,1,0,0,0]]C0300168	3 5 hydroxymethyl 2 furyl 1 benzylthieno 3 2 c pyrazole	[[0,1,0,0,0,0,0,0,0,0,0]]C1161448	staphlipen	[[0,0,0,0,0,0,0,1,0,0,0]]C1161449	flower essence yerba santa	[[0,0,0,0,0,1,0,0,0,0,0]]C0645332	n 11 1 pyrene sulfonylaminoundecanoyl sphingomyelin,psa11 spm	[[0,1,0,0,0,0,0,0,0,0,0],[0,1,0,0,0,0,0,0,0,0,0]]C0058118	dihydroteleocidin b	[[0,1,0,0,0,0,0,0,0,0,0]]C0058119	dihydrotestosterone 17 bromoacetate,5 alpha 17 beta 17 bromoacetyl oxy androstan 3 one,dhtba,17 bromoacetoxydihydrotestosterone	[[0,1,0,0,0,0,0,0,0,0,0],[0,1,0,0,0,0,0,0,0,0,0],[0,1,0,0,0,0,0,0,0,0,0],[0,1,0,0,0,0,0,0,0,0,0]]C0601526	3 methyl 4 nitropyridine 1 oxide	[[0,1,0,0,0,0,0,0,0,0,0]]C0601525	iron ii 5 pyridylbenzodiazepin 2 one	[[0,1,0,0,0,0,0,0,0,0,0]]C1170146	certiva	[[0,0,0,0,0,0,0,1,0,0,0]]C2929089	diphenhydramine lidocaine	[[0,0,0,0,0,0,0,1,0,0,0]]C0601529	cyclocostunolide	[[0,1,0,0,0,0,0,0,0,0,0]]C2929088	thiamine vitamin e	[[0,0,0,0,0,0,0,1,0,0,0]]C0378976	kf 15232,4 5 dihydro 5 methyl 6 4 phenylmethyl amino 7 quinazolinyl 3 2h pyridazinone	[[0,1,0,0,0,0,0,0,0,0,0],[0,1,0,0,0,0,0,0,0,0,0]]C0378975	4 5 dihydro 6 4 benzylamino 7 quinazolinyl 3 2h pyridazinone	[[0,1,0,0,0,0,0,0,0,0,0]]C0378974	azasetron,monohydrochloride n 1 azabicyclo 2 2 2 oct 3 yl 6 chloro 3 4 dihydro 4 methyl 3 oxo 2h 1 4 benzoxazine 8 carboxamide	[[0,1,0,0,0,0,0,0,0,1,0],[0,1,0,0,0,0,0,0,0,0,0]]C0378973	9 fluorenylmethyl chloroformate hydrazine,fmoc hydrazine	[[0,1,0,0,0,0,0,0,0,0,0],[0,1,0,0,0,0,0,0,0,0,0]]C0145581	thevetoside,thevetosidum	[[0,1,0,0,0,0,0,0,0,0,0],[0,1,0,0,0,0,0,0,0,0,0]]C0378978	d ch2 5 1 d ile 2 ile 4 arg 8 ala 9 vasopressin	[[0,1,0,0,0,0,0,0,0,0,0]]C0607968	merulinic acids	[[0,1,0,0,0,0,0,0,0,0,0]]C0767567	synthetic ctuf2 protein,cyclo	[[0,1,0,0,0,0,0,0,0,0,0],[0,1,0,0,0,0,0,0,0,0,0]]C0767560	sib 1553a	[[0,1,0,0,0,0,0,0,0,0,0]]C0767563	mm 36,mm36 cpd	[[0,1,0,0,0,0,0,0,0,0,0],[0,1,0,0,0,0,0,0,0,0,0]]C0088926	1 bis chlorophenyl methyl 3 2 4 dichloro beta 2 4 dichlorobenzyloxy phenethyl imidazolinium chloride	[[0,1,0,0,0,0,0,0,0,0,0]]C0088927	1 coumaran 5 ylmethyl 4 2 thiazolyl piperazine,monohydrochloride 1 2 3 dihydro 5 benzofuranyl methyl 4 2 thiazolyl piperazine	[[0,1,0,0,0,0,0,0,0,0,0],[0,1,0,0,0,0,0,0,0,0,0]]C0889367	3beta 4alpha isomer 4 methylcholest 8 en 3 ol	[[0,1,0,0,0,0,0,0,0,0,0]]C0889366	3beta 4beta isomer 4 methylcholest 8 en 3 ol	[[0,1,0,0,0,0,0,0,0,0,0]]C2929086	chlorzoxazone flufenamic acid	[[0,0,0,0,0,0,0,1,0,0,0]]C1436328	eszopiclone,5s 6 chloropyridine 2 yl 7 oxo 6 7 dihydro 5h pyrrolo 3 4 b pyrazin 5 yl 4 methyl piperazine 1 carboxylate,5s 6 5 chloropyridin 2 yl 7 oxo 6 7 dihydro 5h pyrrolo 3 4 b pyrazin 5 yl 4 methylpiperazine 1 carboxylate,s zopiclone	[[0,0,1,0,0,0,0,1,0,0,0],[0,0,0,0,0,0,0,0,0,0,1],[0,0,0,0,0,0,0,0,0,1,0],[0,0,0,0,0,0,0,0,0,1,0]]C2073916	acetaminophen chlorphenoxamine phenylephrine	[[0,0,0,0,0,0,0,1,0,0,0]]C2929081	adrenal cortex extract rosemary oil	[[0,0,0,0,0,0,0,1,0,0,0]]C0530487	tachypleus tridentatus lici 3 protein,tachypleus tridentatus limulus intracellular coagulation inhibitor 3,tachypleus tridentatus lici type 3 protein	[[0,1,0,0,0,0,0,0,0,0,0],[0,1,0,0,0,0,0,0,0,0,0],[0,1,0,0,0,0,0,0,0,0,0]]C0967587	2 amino 2 3 3a 4 5 6 hexahydro 1h phenalene	[[0,1,0,0,0,0,0,0,0,0,0]]C2929080	anise oil thyme preparation	[[0,0,0,0,0,0,0,1,0,0,0]]C0678116	tarivid	[[0,1,0,0,0,0,1,0,0,0,0]]C0678117	sandostatin	[[0,0,0,0,0,0,1,1,1,1,0]]C0054323	buzepide metiodide,1 3 carbamoyl 3 3 diphenylpropyl hexahydro 1 methylazepinium iodide,1 4 amino 4 oxo 3 3 diphenylbutyl hexahydro 1 methyl 1h azepinium iodide	[[0,0,0,0,0,0,0,1,0,1,0],[0,1,0,0,0,0,0,0,0,0,0],[0,1,0,0,0,0,0,0,0,0,0]]C0678115	uromitexan,asta medica brand of mesna,sanfer brand of mesna	[[0,0,0,0,0,0,1,0,0,1,0],[0,1,0,0,0,0,0,0,0,0,0],[0,1,0,0,0,0,0,0,0,0,0]]C0678112	cam brand of ephedrine hydrochloride	[[0,0,0,0,0,0,0,1,0,0,0]]C0678113	actilyse,boehringer ingelheim brand of alteplase,promeco brand of alteplase,lysatec rt pa,lysatec rtpa	[[0,0,0,0,0,0,1,1,0,0,0],[0,1,0,0,0,0,0,0,0,0,0],[0,1,0,0,0,0,0,0,0,0,0],[0,1,0,0,0,0,0,0,0,0,0],[0,1,0,0,0,0,0,0,0,0,0]]C0054328	bw 12c,bw12c	[[0,1,0,0,0,0,0,0,0,0,0],[0,1,0,0,0,0,0,0,0,0,0]]C2929082	butalamine papaverine	[[0,0,0,0,0,0,0,1,0,0,0]]C0678119	pepcid	[[0,0,0,0,0,0,1,1,0,0,0]]C0913170	actinomycin hki 0155	[[0,1,0,0,0,0,0,0,0,0,0]]C0913171	pyralomicin 1c	[[0,1,0,0,0,0,0,0,0,0,0]]C2746162	pro pet antiseptic	[[0,0,0,0,0,0,0,1,0,0,0]]C2720503	casein allergenic extract,casein	[[0,0,0,0,0,0,0,1,0,0,0],[0,0,1,0,0,0,0,0,0,0,0]]C0386913	1 2 trifluoromethylphenyl imidazole,1 trim	[[0,1,0,0,0,0,0,0,0,0,0],[0,1,0,0,0,0,0,0,0,0,0]]C0386914	super malic	[[0,1,0,0,0,0,0,0,0,0,0]]C0386916	9 methyl 1 2 3 4 6 7 12 12b octahydroindolo 2 3 a quinolizine,miq compound	[[0,1,0,0,0,0,0,0,0,0,0],[0,1,0,0,0,0,0,0,0,0,0]]C0386917	pentabromophenol	[[0,1,0,0,0,0,0,0,0,0,0]]C2827589	achillea millefolium pollen	[[0,0,0,0,0,0,0,0,0,1,0]]C2827588	acer pseudoplatanus pollen	[[0,0,0,0,0,0,0,0,0,1,0]]C0069936	padimate a,amyl dimethylaminobenzoate,amyl p dimethylaminobenzoate	[[1,1,1,0,0,0,1,0,0,0,0],[0,1,0,0,0,0,0,0,0,0,0],[0,1,0,0,0,0,0,0,0,0,0]]C0069937	padimate o,2 ethylhexyl ester 4 n n dimethylamino benzoic acid,2 ethylhexyl p dimethylaminobenzoate,escalol 507,octyl dimethyl aminobenzoate	[[0,0,0,0,0,0,1,1,0,1,1],[0,0,0,0,0,0,0,0,0,0,1],[1,0,0,0,0,0,0,0,0,0,1],[0,0,0,0,0,0,0,0,0,0,1],[1,0,0,0,0,0,0,0,0,0,0]]C0069930	pachypodol,3 7 3 quercetol trimethyl ether 5 4 dihydroxy,4 5 dihydroxy 3 3 7 trimethoxyflavone,5 hydroxy 2 4 hydroxy 3 methoxyphenyl 3 7 dimethoxy 4h 1 benzopyran 4 one	[[0,1,0,0,0,0,0,0,0,0,0],[0,1,0,0,0,0,0,0,0,0,0],[0,1,0,0,0,0,0,0,0,0,0],[0,1,0,0,0,0,0,0,0,0,0]]C0046553	2 succinylamido 1 3 4 thiadiazole 5 sulfonamide,4 5 aminosulfonyl 1 3 4 thiadiazol 2 yl amino 4 oxo butanoic acid,stzsa	[[0,1,0,0,0,0,0,0,0,0,0],[0,1,0,0,0,0,0,0,0,0,0],[0,1,0,0,0,0,0,0,0,0,0]]C0046559	2 tert butyl 4 hydroxyanisole,2 bha,butylated hydroxyanisole ii,3 1 1 dimethylethyl 4 methoxy phenol	[[0,1,0,0,0,0,0,0,0,1,0],[0,1,0,0,0,0,0,0,0,0,0],[0,0,0,0,0,0,0,0,0,1,0],[0,0,0,0,0,0,0,0,0,1,0]]C0069939	paeonol,1 2 hydroxy 4 methoxyphenyl ethanone,2 hydroxy 4 methoxyacetophenone,peonol	[[0,1,0,0,0,0,0,0,0,0,0],[0,1,0,0,0,0,0,0,0,0,0],[0,1,0,0,0,0,0,0,0,0,0],[0,1,0,0,0,0,0,0,0,0,0]]C2827587	acacia decurrens pollen	[[0,0,0,0,0,0,0,0,0,1,0]]C2827586	abies procera pollen	[[0,0,0,0,0,0,0,0,0,1,0]]C0917598	tylopeptin b	[[0,1,0,0,0,0,0,0,0,0,0]]C1588401	sitrex	[[0,0,0,0,0,0,0,1,0,0,0]]C0959301	diaminomethane dihydrochloride	[[0,1,0,0,0,0,0,0,0,0,0]]C1531436	wit003 cpd	[[0,1,0,0,0,0,0,0,0,0,0]]C1531437	wit002 cpd	[[0,1,0,0,0,0,0,0,0,0,0]]C1513439	moab bombesin,monoclonal antibody bombesin itc d	[[0,0,0,0,0,0,0,0,0,1,0],[0,0,0,0,0,0,0,0,0,1,0]]C0006474	butane,n butane	[[0,0,0,0,0,0,1,0,0,1,0],[1,1,0,0,0,0,0,0,0,0,0]]C0959306	trans isomer fg 7080	[[0,1,0,0,0,0,0,0,0,0,0]]C0048001	4 amino 3 5 dichlorophenyl 4 2 3 4 trimethoxyphenyl methyl 1 piperazineethanol	[[0,1,0,0,0,0,0,0,0,0,0]]C0667022	gamma heregulin,gamma hrg	[[0,1,0,0,0,0,0,0,0,0,0],[0,1,0,0,0,0,0,0,0,0,0]]C0667023	kakkalide	[[0,1,0,0,0,0,0,0,0,0,0]]C1724010	advil pm	[[0,0,0,0,0,0,0,1,0,0,0]]C0761656	cyclopentadienyl tricarbonyl rhenium,co rhenium	[[0,1,0,0,0,0,0,0,0,0,0],[0,1,0,0,0,0,0,0,0,0,0]]C1724016	atripla,tenofovir disoproxil fumarate drug combination emtricitabine bms brand of efavirenz,tenofovir disoproxil fumarate drug combination emtricitabine gilead sciences brand of efavirenz	[[0,0,0,0,0,0,0,1,0,0,0],[0,1,0,0,0,0,0,0,0,0,0],[0,1,0,0,0,0,0,0,0,0,0]]C0717428	anhydrous calcium iodide codeine	[[0,0,0,0,0,0,0,1,0,0,0]]C0717429	anhydrous calcium iodide isoproterenol	[[0,0,0,0,0,0,0,1,0,0,0]]C3180970	n 2 1 phenyl 3 trifluoromethyl 1h pyrazol 4 yl carbonyl amino ethyl 6 2 2 2 trifluoroethoxy pyridine 3 carboxamide,ptpcae tpcnh2	[[0,1,0,0,0,0,0,0,0,0,0],[0,1,0,0,0,0,0,0,0,0,0]]C3180973	3 2 azepan 1 yl ethyl 6 propylbenzo d thiazol 2 3h one	[[0,1,0,0,0,0,0,0,0,0,0]]C0717427	anhydrous calcium iodide	[[0,0,0,0,0,0,0,1,0,0,0]]C0717424	ampicillin probenecid	[[0,0,0,0,0,0,0,1,0,0,1]]C0771989	octacaine	[[0,0,0,0,0,0,0,0,0,1,0]]C0771988	memantine hydrochloride,memantine hcl,1 amino 3 5 dimethyladamantane hydrochloride,ebixia	[[1,0,0,0,0,0,1,0,0,1,0],[0,0,0,0,0,0,1,0,0,0,0],[0,0,0,0,0,0,0,0,0,1,0],[0,0,0,0,0,0,0,0,0,1,0]]C2971527	platelet concentrate units 124 blood product unit	[[0,0,0,1,0,0,0,0,0,0,0]]C2343722	sudafed sinus nighttime plus pain relief	[[0,0,0,0,0,0,0,1,0,0,0]]C0771983	dihydroergotamine tartrate	[[0,0,0,0,0,0,0,1,0,0,0]]C0771982	morazone hcl	[[0,0,0,0,0,0,0,1,0,0,0]]C0771984	tiropramide hcl	[[0,0,0,0,0,0,0,1,0,0,0]]C0771987	nimustine hydrochloride,pimustine hydrochloride,3 4 amino 2 methyl 5 pyrimidinyl methyl 1 2 chloroethyl 1 nitrosourea hydrochloride,n 4 amino 2 methyl 5 pyrimidinyl methyl n 2 chloroethyl n nitrosourea hydrochloride	[[0,0,0,0,0,0,0,0,0,1,0],[0,0,0,0,0,0,0,0,0,1,0],[0,0,0,0,0,0,0,0,0,1,0],[0,0,0,0,0,0,0,0,0,1,0]]C3257584	live attenuated rotavirus vaccine	[[0,0,0,0,0,0,0,0,0,0,1]]C0058523	djw 94	[[0,1,0,0,0,0,0,0,0,0,0]]C1875347	investigational congestive heart failure	[[0,0,0,0,0,0,0,0,0,0,1]]C0877956	bay e 5009	[[0,1,0,0,0,0,0,0,0,0,0]]C1700359	leg 3 prodrug	[[0,1,0,0,0,0,0,0,0,0,0]]C1564024	biquinate,aventis brand of quinine bisulfate	[[0,1,0,0,0,0,0,0,0,0,0],[0,1,0,0,0,0,0,0,0,0,0]]C0058527	dl 111	[[0,1,0,0,0,0,0,0,0,0,0]]C0304586	skin antibiotic	[[1,0,0,0,0,0,1,0,0,0,0]]C0673641	naphthoxirene	[[0,1,0,0,0,0,0,0,0,0,0]]C0686726	s tricyclamol	[[1,0,0,0,0,0,0,0,0,0,0]]C0887754	albendazole monohydrochloride	[[0,1,0,0,0,0,0,0,0,0,0]]C0887755	e isomer cinnarizine	[[0,1,0,0,0,0,0,0,0,0,0]]C0887756	cinnarizine l tartrate	[[0,1,0,0,0,0,0,0,0,0,0]]C0887757	dihydrochloride cinnarizine	[[0,1,0,0,0,0,0,0,0,0,0]]C0133948	ono 1052	[[0,1,0,0,0,0,0,0,0,0,0]]C1636119	tusana d	[[0,0,0,0,0,0,0,1,0,0,0]]C0887758	cinnarizine l tartrate	[[0,1,0,0,0,0,0,0,0,0,0]]C1432313	allomyrina dichotoma coleoptericin bm protein	[[0,1,0,0,0,0,0,0,0,0,0]]C1432312	allomyrina dichotoma coleoptericin a protein	[[0,1,0,0,0,0,0,0,0,0,0]]C1974480	thyroxine 124 saliva	[[0,0,0,1,0,0,0,0,0,0,0]]C0537918	ru 39568,ru39568	[[0,1,0,0,0,0,0,0,0,0,0],[0,1,0,0,0,0,0,0,0,0,0]]C0304887	percoid liver oil preparation	[[1,0,0,0,0,0,0,0,0,0,0]]C0213960	gem91,gene expression modulator 91,gem 91,trecovirsen	[[0,1,0,0,0,0,0,0,0,0,0],[0,1,0,0,0,0,0,0,0,1,0],[0,0,0,0,0,0,0,0,0,1,0],[0,1,0,0,0,0,0,0,0,1,0]]C0209062	1 2 diamino 4 5 methylenedioxybenzene,1 2 dmb,1 2 diamino 4 5 methylenedioxy benzene,dmb cpd	[[0,1,0,0,0,0,0,0,0,0,0],[0,1,0,0,0,0,0,0,0,0,0],[0,1,0,0,0,0,0,0,0,0,0],[0,1,0,0,0,0,0,0,0,0,0]]C0255390	n 4 2 2 4 diamino 6 7 dihydro 5h cyclopenta d pyrimidin 5 yl ethyl benzoyl glutamic acid,ddcpeb glu	[[0,1,0,0,0,0,0,0,0,0,0],[0,1,0,0,0,0,0,0,0,0,0]]C0255392	7 1 azidirinyl 2 3 dihydro 1 acetoxy 5 8 dihydroxy 6 9 dimethyl 1h pyrrolo 1 2 a indole,7 adad 1h pid	[[0,1,0,0,0,0,0,0,0,0,0],[0,1,0,0,0,0,0,0,0,0,0]]C0255395	2 butyl 4 5 dihydro 4 oxo 3 2 1h tetrazol 5 yl 4 biphenylyl methyl 3h imidazo 4 5 c pyridine 5 n n dimethylacetamide	[[0,1,0,0,0,0,0,0,0,0,0]]C0255394	6 1 aziridinyl 2 3 dihydro 3 acetoxy 5 8 dihydroxy 7 methyl 1h pyrrolo 1 2 a benzimidazole,6 adad 1h pbid	[[0,1,0,0,0,0,0,0,0,0,0],[0,1,0,0,0,0,0,0,0,0,0]]C0255396	emd 66684,emd 66 684	[[0,1,0,0,0,0,0,0,0,0,0],[0,1,0,0,0,0,0,0,0,0,0]]C0063966	isofenphos,isophenphos	[[0,1,0,0,0,0,0,0,0,0,0],[0,1,0,0,0,0,0,0,0,0,0]]C0063961	isodityrosine,o 5 2 amino 2 carboxyethyl 2 hydroxyphenyl l tyrosine	[[0,1,0,0,0,0,0,0,0,0,0],[0,1,0,0,0,0,0,0,0,0,0]]C0063963	isoetam	[[0,1,0,0,0,0,0,0,0,0,0]]C0718959	bismatrol	[[0,0,0,0,0,0,0,1,0,0,0]]C0718958	bismarex	[[0,0,0,0,0,0,0,1,0,0,0]]C0718953	biotuss pe	[[0,0,0,0,0,0,0,1,0,0,0]]C0718952	biotuss dm	[[0,0,0,0,0,0,0,1,0,0,0]]C0718951	bion tears	[[0,0,0,0,0,0,1,1,0,0,0]]C0718950	biomox	[[0,0,0,0,0,0,0,1,0,0,0]]C0718957	bisco lax,schein brand of bisacodyl	[[0,1,0,0,0,0,0,1,0,0,0],[0,1,0,0,0,0,0,0,0,0,0]]C0718956	bisac evac,g w brand of bisacodyl	[[0,1,0,0,0,0,0,1,0,0,0],[0,1,0,0,0,0,0,0,0,0,0]]C0718955	bisa plex	[[0,0,0,0,0,0,0,1,0,0,0]]C0718954	biotussin dac	[[0,0,0,0,0,0,0,1,0,0,0]]C3159656	robitussin peak cold	[[0,0,0,0,0,0,0,1,0,0,0]]C0540938	u 89968e	[[0,1,0,0,0,0,0,0,0,0,0]]C0954270	2 aminobenzimidazole monobenzoate	[[0,1,0,0,0,0,0,0,0,0,0]]C0532244	camphorated phenol,phenol drug combination camphor	[[1,1,0,0,0,0,0,0,0,0,0],[0,1,0,0,0,0,0,0,0,0,0]]C1140586	flower essence mullein	[[0,0,0,0,0,1,0,0,0,0,0]]C0954277	veralipride monohydrochloride	[[0,1,0,0,0,0,0,0,0,0,0]]C0544357	human plasma	[[0,0,0,0,0,0,1,0,0,0,0]]C0142871	sodium iron iii gluconate,sodium salt ferric gluconate,sodium ferrigluconate	[[0,1,0,0,0,0,0,0,0,0,0],[0,1,0,0,0,0,0,0,0,0,0],[0,1,0,0,0,0,0,0,0,0,0]]C0142873	sodium salt 4 carboxamidopiperidine n dithiocarboxylate,sodium isonipecotamide dithiocarbamate	[[0,1,0,0,0,0,0,0,0,0,0],[0,1,0,0,0,0,0,0,0,0,0]]C0142875	sodium salt lauric acid,sodium laurate	[[0,1,0,0,0,0,0,0,0,0,0],[0,1,0,0,0,0,0,0,0,0,0]]C0142874	sodium lactate,e325 sodium lactate,2 hydroxypropanoic acid sodium salt,sodium salt lactic acid,sodium alpha hydroxypropionate,per glycerin,lacolin	[[0,1,1,0,0,0,1,0,0,1,0],[1,0,0,0,0,0,0,0,0,0,0],[0,0,0,0,0,0,0,0,0,1,0],[0,0,0,0,0,0,0,0,0,1,0],[0,0,0,0,0,0,0,0,0,1,0],[0,0,0,0,0,0,0,0,0,1,0],[0,0,0,0,0,0,0,0,0,1,0]]C0142877	sodium dodecylbenzenesulfonate,sodium laurylbenzenesulfonate,sodium dodecyl benzene sulfonate,sodium salt dodecylbenzenesulfonic acid,dodecyl benzene sulfonic acid sodium	[[0,1,1,0,0,0,0,0,0,0,0],[0,1,0,0,0,0,0,0,0,0,0],[0,1,0,0,0,0,0,0,0,0,1],[0,1,0,0,0,0,0,0,0,0,0],[0,1,0,0,0,0,0,0,0,0,0]]C0142876	sodium lauriminodipropionate	[[0,1,0,0,0,0,0,0,0,0,0]]C0142879	sodium loxoprofen	[[0,1,0,0,0,0,0,0,0,0,0]]C0142878	sodium salt lawsone,sodium lawsonate	[[0,1,0,0,0,0,0,0,0,0,0],[0,1,0,0,0,0,0,0,0,0,0]]C0619491	ro 09 0680,ro 090680	[[0,1,0,0,0,0,0,0,0,0,0],[0,1,0,0,0,0,0,0,0,0,0]]C0075098	sr 2640,sr2640	[[0,1,0,0,0,0,0,0,0,0,0],[0,1,0,0,0,0,0,0,0,0,0]]C1567703	diethyl 2 4 di 3 fluorophenyl 3 7 dimethyl 3 7 diazabicyclo 3 3 1 nonane 9 one 1 5 dicarboxylate,3flb cpd	[[0,1,0,0,0,0,0,0,0,0,0],[0,1,0,0,0,0,0,0,0,0,0]]C0950613	carbromalhydroxyzine hydrochloride	[[0,1,0,0,0,0,0,0,0,0,0]]C0544354	mixed bacterial vaccine	[[1,0,0,0,0,0,0,0,0,0,0]]C0208910	ala 10 argipressin,ala arg 8 vasopressin,alanine argipressin,ala argipressin,10 ala avp,ala 10 arginine vasopressin,alanine 10 argipressin	[[0,1,0,0,0,0,0,0,0,0,0],[0,1,0,0,0,0,0,0,0,0,0],[0,1,0,0,0,0,0,0,0,0,0],[0,1,0,0,0,0,0,0,0,0,0],[0,1,0,0,0,0,0,0,0,0,0],[0,1,0,0,0,0,0,0,0,0,0],[0,1,0,0,0,0,0,0,0,0,0]]C0360055	5 ht 3 receptor antagonist,5 ht3 receptor antagonist,5ht3 receptor antagonist,5 ht sub 3 sub receptor antagonist,serotonin subtype 3 5 ht3 receptor antagonist,5ht 3 receptor antagonist,serotonin 3 receptor antagonist,serotonin subtype 3 receptor antagonist,serotonin subtype 3 5ht 3 receptor antagonist,serotonin subtype 3 5ht3 receptor antagonist,5 hydroxytryptamine 3 receptor antagonist,type 3 serotonin receptor antagonist	[[1,0,0,0,0,0,0,0,0,0,0],[1,0,0,0,0,0,1,0,0,1,0],[0,0,0,0,0,0,0,0,0,0,1],[1,0,0,0,0,0,0,0,0,0,0],[0,0,0,0,0,0,0,0,0,0,1],[0,0,0,0,0,0,0,0,0,0,1],[0,0,0,0,0,0,0,0,0,0,1],[0,0,0,0,0,0,0,0,0,0,1],[0,0,0,0,0,0,0,0,0,0,1],[0,0,0,0,0,0,0,0,0,0,1],[0,0,0,0,0,0,0,0,0,1,0],[0,0,0,0,0,0,0,0,0,1,0]]C1518808	pr18 ras 13 cys peptide	[[0,0,0,0,0,0,0,0,0,1,0]]C1518809	pr 54 ras 13 asp peptide,ras peptide pr54	[[0,0,0,0,0,0,0,0,0,1,0],[0,0,0,0,0,0,0,0,0,1,0]]C0033798	pseudoephedrine,1s 2s 2 methylamino 1 phenyl propan 1 ol	[[0,0,0,0,0,0,1,1,0,1,0],[0,0,0,0,0,0,0,0,0,0,1]]C0950614	tno 6	[[0,1,0,0,0,0,0,0,0,0,0]]C0216687	transforming growth factor alpha pseudomonas exotoxin a 35,tgfalpha pe35,pe35 tgfalpha kdel	[[0,1,0,0,0,0,0,0,0,0,0],[0,1,0,0,0,0,0,0,0,0,0],[0,1,0,0,0,0,0,0,0,0,0]]C2724978	dent o kain	[[0,0,0,0,0,0,0,1,0,0,0]]C1254006	vertab sr	[[0,0,0,0,0,0,0,1,0,0,0]]C0389192	cinnamyl cyanocaffeate	[[0,1,0,0,0,0,0,0,0,0,0]]C1254002	libanil	[[0,0,0,0,0,0,0,1,0,0,0]]C0699218	kabivitrum	[[0,1,0,0,0,0,0,0,0,0,0]]C0640082	ac dehydro phe 1 dehydro 4 cl phe 2 dehydro trp 3 6 lhrh,acetyl dehydro 1 phenylalanyl dehydro p cl 2 phenylalanyl dehydro 3 6 tryptophyl gnrh,appt gnrh	[[0,1,0,0,0,0,0,0,0,0,0],[0,1,0,0,0,0,0,0,0,0,0],[0,1,0,0,0,0,0,0,0,0,0]]C0110658	copolymer i,synthetic peptide copolymer i,cop 1	[[0,1,0,0,0,0,0,0,1,0,0],[0,0,0,0,0,0,0,0,1,0,0],[0,1,0,0,0,0,0,0,1,0,0]]C0640080	ac d phe 1 d 4 cl phe 2 d trp 3 6 gnrh	[[0,1,0,0,0,0,0,0,0,0,0]]C0640087	n 2 hydroxyethyl 2 3 nitro 1 2 4 triazol 1 yl acetamide	[[0,1,0,0,0,0,0,0,0,0,0]]C0970444	dauricine monohydrochloride	[[0,1,0,0,0,0,0,0,0,0,0]]C0614175	1 trifluoromethyl 1 2 diphenylethylene,e 1 1 1 trifluoromethyl 1 2 ethenediyl bis benzene,1 1 tfmde	[[0,1,0,0,0,0,0,0,0,0,0],[0,1,0,0,0,0,0,0,0,0,0],[0,1,0,0,0,0,0,0,0,0,0]]C0110652	copirene	[[0,1,0,0,0,0,0,0,0,0,0]]C0699211	pontal	[[0,1,0,0,0,0,0,0,0,0,0]]C0614178	4 nitrobenzylmaleimide,1 4 nitrophenyl methyl 2 5 pyrrolidinedione,para nitrobenzylmaleimide	[[0,1,0,0,0,0,0,0,0,0,0],[0,1,0,0,0,0,0,0,0,0,0],[0,1,0,0,0,0,0,0,0,0,0]]C0699213	streptase	[[0,0,0,0,0,0,1,1,0,0,0]]C0164145	beta phenylcysteine,3 phenylcysteine,s phenylcysteine	[[0,1,0,0,0,0,0,0,0,0,0],[0,1,0,0,0,0,0,0,0,0,0],[0,1,0,0,0,0,0,0,0,0,0]]C0699215	awelysin	[[0,1,0,0,0,0,0,0,0,0,0]]C0699216	celiase	[[0,1,0,0,0,0,0,0,0,0,0]]C0699217	distreptase	[[0,1,0,0,0,0,0,0,0,0,0]]C1263567	cyclobral	[[0,0,0,0,0,0,0,1,0,0,0]]C1263563	rauwiloid	[[0,0,0,0,0,0,0,1,0,0,0]]C1263561	hypercal	[[0,0,0,0,0,0,1,1,0,0,0]]C3256063	mentha arvensis leaf oil	[[0,0,1,0,0,0,0,0,0,0,0]]C0959979	3alpha 4alpha s 4abeta 5beta 6alpha isomer actinobolin sulfate	[[0,1,0,0,0,0,0,0,0,0,0]]C0207982	2 3 dimethoxy 6 methyl 8 9 methylenedioxy 11h indeno 1 2 c isoquinoline,2 3 dmmii	[[0,1,0,0,0,0,0,0,0,0,0],[0,1,0,0,0,0,0,0,0,0,0]]C1999734	bbr3610	[[0,1,0,0,0,0,0,0,0,0,0]]C0117391	fe 169	[[0,1,0,0,0,0,0,0,0,0,0]]C0035795	rocky mountain spotted fever vaccine,fever mountain rocky spotted	[[1,0,0,0,0,1,1,0,0,0,0],[0,0,0,0,0,0,1,0,0,0,0]]C3251986	4 4 6 7 7 8 hexahydroxy 1 1 dimethoxy 6 7 dimethyl 5 6 7 8 tetrahydro 2 2 bianthracene 9 9 10 10 tetraone	[[0,1,0,0,0,0,0,0,0,0,0]]C2138494	cromolyn naphazoline	[[0,0,0,0,0,0,0,1,0,0,0]]C2138496	cromolyn xylometazoline	[[0,0,0,0,0,0,0,1,0,0,0]]C2928026	camylofine dipyrone	[[0,0,0,0,0,0,0,1,0,0,0]]C0066875	mrz 2549	[[0,1,0,0,0,0,0,0,0,0,0]]C0066876	ms 1 2,trans 2 3 dihydro n 4 2 phenoxyethyl 1 piperazinyl methyl 1 4 benzodioxin 2 carboxamide,mc 1 2	[[0,1,0,0,0,0,0,0,0,0,0],[0,1,0,0,0,0,0,0,0,0,0],[0,1,0,0,0,0,0,0,0,0,0]]C2740360	hyperrho	[[0,0,0,0,0,0,0,1,0,0,0]]C3251983	solomonsterol b	[[0,1,0,0,0,0,0,0,0,0,0]]C1981200	alprenolol 124 urine	[[0,0,0,1,0,0,0,0,0,0,0]]C3256066	peg 20 soy sterol	[[0,0,1,0,0,0,0,0,0,0,0]]C0066879	msbe	[[0,1,0,0,0,0,0,0,0,0,0]]C3251982	24 nor 5alpha cholan 2 3 24 tryl 2 3 23 sodium trisulfate	[[0,1,0,0,0,0,0,0,0,0,0]]C0533115	1 3 c ethynylribopentofuranosyl cytosine,3 c ethynyl cytidine,3 ethynylcytidine,ecyd cpd,3 c ethynylcytidine,ecyd,ecdy	[[0,1,0,0,0,0,0,0,0,0,0],[0,1,0,0,0,0,0,0,0,0,0],[0,1,0,0,0,0,0,0,0,0,0],[0,1,0,0,0,0,0,0,0,0,0],[0,0,0,0,0,0,0,0,0,1,0],[0,0,0,0,0,0,0,0,0,1,0],[0,0,0,0,0,0,0,0,0,1,0]]C0533112	1 3 c ethynyl beta d ribo pentofuranosyl cytosine	[[0,0,0,0,0,0,0,0,0,1,0]]C3251981	24 norcholan 2 3 24 tryl 2 3 23 sulfuric acid	[[0,1,0,0,0,0,0,0,0,0,0]]C0959970	2s 2alpha 5alpha 6beta s isomer phenethicillin	[[0,1,0,0,0,0,0,0,0,0,0]]C3251980	m402 heparan sulfate	[[0,1,0,0,0,0,0,0,0,0,0]]C0533119	1 3 c ethynylribopentofuranosyl uracil,1 3 c ethynyl beta d ribo pentofuranosyl uracil,3 c ethynyl uridine,3 ethynyluridine,pj 272,pj272	[[0,1,0,0,0,0,0,0,0,0,0],[0,1,0,0,0,0,0,0,0,0,0],[0,1,0,0,0,0,0,0,0,0,0],[0,1,0,0,0,0,0,0,0,0,0],[0,1,0,0,0,0,0,0,0,0,0],[0,1,0,0,0,0,0,0,0,0,0]]C0286937	beta mercapto beta beta cyclopentamethylenepropionic acid 1 o ethyl tyr 2 val 4 cit 8 vasopressin,vasopressin beta mercapto beta beta cyclopentamethylenepropionic acid 1 o ethyltyrosyl 2 valyl 4 citrulline 8,d ch2 5tyr oet 2 val 4 cit 8 vasopressin,beta mercapto beta beta cyclopentamethylenepropionic acid 2 o et tyr 4 val 8 cit vasopressin,tevcv	[[0,1,0,0,0,0,0,0,0,0,0],[0,1,0,0,0,0,0,0,0,0,0],[0,1,0,0,0,0,0,0,0,0,0],[0,1,0,0,0,0,0,0,0,0,0],[0,1,0,0,0,0,0,0,0,0,0]]C0286930	22 23 epoxy 2 aza 2 3 dihydrosqualene n oxide,22 23 eadsn	[[0,1,0,0,0,0,0,0,0,0,0],[0,1,0,0,0,0,0,0,0,0,0]]C1664701	ent 18 hydroxyisopimara 7 15 diene 3beta ol	[[0,1,0,0,0,0,0,0,0,0,0]]C1664700	6 r 6 hydroxydichotoma 3 14 diene 1 17 dial,6 hdc d d	[[0,1,0,0,0,0,0,0,0,0,0],[0,1,0,0,0,0,0,0,0,0,0]]C0937610	cinchona pubescens preparation	[[0,0,0,0,0,0,0,1,0,0,0]]C0937613	cordyceps sinensis preparation	[[0,0,0,0,0,0,0,1,0,0,0]]C0937615	delphinium staphisagria preparation	[[0,0,0,0,0,0,0,1,0,0,0]]C0937614	cuttle fish ink	[[0,0,0,0,0,0,0,1,0,0,0]]C0937617	drosera rotundifolia extract,drosera rotundifolia	[[0,0,0,0,0,0,0,1,0,0,0],[0,0,1,0,0,0,0,0,0,0,0]]C0214094	cisplatin procaine complex,dpr complex	[[0,1,0,0,0,0,0,0,0,0,0],[0,1,0,0,0,0,0,0,0,0,0]]C1948514	akurza	[[0,0,0,0,0,0,0,1,0,0,0]]C1961993	pomegranate extract	[[0,0,0,0,0,0,0,1,0,0,0]]C0546856	proguanil hydrochloride,chloroguanide hydrochloride,monohydrochloride 9ci n 4 chlorophenyl n 1 methylethyl imidodicarbonimidic diamide,1 p chlorophenyl 5 isopropylbiguanide hydrochloride,bigumalum,chloroquanil	[[1,1,0,0,0,0,0,1,0,1,0],[0,1,0,0,0,0,0,0,0,1,0],[0,0,0,0,0,0,0,0,0,1,0],[0,0,0,0,0,0,0,0,0,1,0],[0,0,0,0,0,0,0,0,0,1,0],[0,0,0,0,0,0,0,0,0,1,0]]C0620197	feudomycin d,9 methyl 10 hydroxydaunomycin	[[0,1,0,0,0,0,0,0,0,0,0],[0,1,0,0,0,0,0,0,0,0,0]]C0214099	oenothein b	[[0,1,0,0,0,0,0,0,0,0,0]]C0592162	tavegil	[[0,0,0,0,0,0,1,1,0,0,0]]C3251989	balticolid	[[0,1,0,0,0,0,0,0,0,0,0]]C0292935	8 2 2 3 dihydro 8 methoxy 1 4 benzodoxin 2 yl methylamino ethyl 8 azaspiro 4 5 decane 7 9 dione hydrochloride	[[0,1,0,0,0,0,0,0,0,0,0]]C0147962	uth 1424	[[0,1,0,0,0,0,0,0,0,0,0]]C0147963	uth wp 50 7	[[0,1,0,0,0,0,0,0,0,0,0]]C3180117	simonin a	[[0,1,0,0,0,0,0,0,0,0,0]]C3180116	kusunokinin	[[0,1,0,0,0,0,0,0,0,0,0]]C3180115	copalic acid	[[0,1,0,0,0,0,0,0,0,0,0]]C3180114	3 hydroxy 2 methoxy 9 10 methylenedioxy 8 oxoprotoberberine,3 hydroxy mmopb	[[0,1,0,0,0,0,0,0,0,0,0],[0,1,0,0,0,0,0,0,0,0,0]]C3180113	1 2 3 6 tetra o galloylallopyranose gallotannin,gt24 gallotannin	[[0,1,0,0,0,0,0,0,0,0,0],[0,1,0,0,0,0,0,0,0,0,0]]C2705113	sertraline 124 dose	[[0,0,0,1,0,0,0,0,0,0,0]]C0016595	formycins	[[0,1,0,0,0,0,0,0,1,0,0]]C1882445	prednisolone valerate acetate	[[0,0,0,0,0,0,0,0,0,1,0]]C3180119	lg 186,lg186 cpd	[[0,1,0,0,0,0,0,0,0,0,0],[0,1,0,0,0,0,0,0,0,0,0]]C0700493	rimiterol hydrobromide	[[0,0,0,0,0,0,0,1,0,1,0]]C0700492	losartan potassium,monopotassium salt 2 butyl 4 chloro 1 2 1h tetrazol 5 yl 1 1 biphenyl 4 yl methyl 1h imidazole 5 methanol,losartan monopotassium salt	[[1,1,0,0,0,0,0,0,1,1,0],[0,0,0,0,0,0,0,0,0,1,0],[0,1,0,0,0,0,0,0,0,0,1]]C0700491	serevent,glaxo wellcome brand of salmeterol xinafoate	[[0,1,0,0,0,0,1,0,0,0,0],[0,1,0,0,0,0,0,0,0,0,0]]C0700490	temopen	[[0,0,0,0,0,0,0,1,0,0,0]]C0700496	pyridoxine hydrochloride,pyridoxine hydrochloride preparation,hcl pyridoxine,pyridoxol hydrochloride,5 hydroxy 6 methyl 3 4 pyridinedimethanol hydrochloride,vitamin b6 hydrochloride	[[0,0,1,0,1,0,0,0,0,1,0],[1,0,0,0,0,0,0,0,0,0,0],[0,0,0,0,0,0,1,0,0,0,0],[0,0,0,0,0,0,0,0,0,0,1],[0,0,0,0,0,0,0,0,0,1,0],[0,0,0,0,0,0,0,0,0,1,0]]C0641352	trans dihydronarciclasine	[[0,1,0,0,0,0,0,0,0,0,0]]C0641353	dihydronarciclasine	[[0,1,0,0,0,0,0,0,0,0,0]]C0700499	thioridazine hydrochloride,thioridazine hcl	[[1,0,1,0,0,0,0,0,0,1,0],[0,0,0,0,0,0,0,0,0,0,1]]C0917621	decatromicin a	[[0,1,0,0,0,0,0,0,0,0,0]]C0773936	methyltestosterone pwdr	[[0,0,0,0,0,0,0,0,0,0,1]]C0111526	cyclo	[[0,1,0,0,0,0,0,0,0,0,0]]C0111524	cyclo	[[0,1,0,0,0,0,0,0,0,0,0]]C2920611	dok plus reformulated jul 2010	[[0,0,0,0,0,0,0,1,0,0,0]]C2365278	trovan iv	[[0,0,0,0,0,0,0,1,0,0,0]]C0616211	copoly,poly,homopolymer n l alanyl l methionine	[[0,1,0,0,0,0,0,0,0,0,0],[0,1,0,0,0,0,0,0,0,0,0],[0,1,0,0,0,0,0,0,0,0,0]]C2746955	carob allergenic extract	[[0,0,0,0,0,0,0,1,0,0,0]]C2974777	ry221b a	[[0,1,0,0,0,0,0,0,0,0,0]]C0665499	tamol	[[0,1,0,0,0,0,0,0,0,0,0]]C1707804	taxotere injection concentrate	[[0,0,0,0,0,0,0,0,0,1,0]]C0252453	2 thioxo 1 pyrrolidinethioacetamide,2 thio 1 pyrrolidine thio acetamide,thiothio	[[0,1,0,0,0,0,0,0,0,0,0],[0,1,0,0,0,0,0,0,0,0,0],[0,1,0,0,0,0,0,0,0,0,0]]C0252450	2 thioxo 1 pyrrolidineacetamide,2 thio 1 pyrrolidine acetamide,thioacet	[[0,1,0,0,0,0,0,0,0,0,0],[0,1,0,0,0,0,0,0,0,0,0],[0,1,0,0,0,0,0,0,0,0,0]]C0665495	ap aw resin	[[0,1,0,0,0,0,0,0,0,0,0]]C0665496	apacider aw	[[0,1,0,0,0,0,0,0,0,0,0]]C1873935	abolene	[[0,0,0,0,0,0,0,0,0,0,1]]C0919339	ichthyol	[[0,1,0,0,0,0,1,0,0,0,0]]C0096820	4 carbomethoxythiazoline	[[0,1,0,0,0,0,0,0,0,0,0]]C0723269	senexon	[[0,0,0,0,0,0,0,1,0,0,0]]C1529633	agrelin,roberts brand of anagrelide hydrochloride	[[0,1,0,0,0,0,0,0,0,0,0],[0,1,0,0,0,0,0,0,0,0,0]]C1529632	dolodent,gilbert brand of amylocaine hydrochloride	[[0,1,0,0,0,0,0,0,0,0,0],[0,1,0,0,0,0,0,0,0,0,0]]C0064432	kt 362,e 2 butenedioate 1 1 5 3 2 3 4 dimethoxyphenyl ethyl amino 1 oxopropyl 2 3 4 5 tetrahydro 1 5 benzothiazepine	[[0,1,0,0,0,0,0,0,0,0,0],[0,1,0,0,0,0,0,0,0,0,0]]C0064431	kt 199,antibiotic kt 199	[[0,1,0,0,0,0,0,0,0,0,0],[0,1,0,0,0,0,0,0,0,0,0]]C1529639	ushercell	[[0,1,0,0,0,0,0,0,0,0,0]]C0592032	dolipol,rastinon,aventis brand of tolbutamide,hoechst brand of tolbutamide	[[0,1,0,0,0,0,0,0,0,0,0],[0,1,0,0,0,0,0,0,0,0,0],[0,1,0,0,0,0,0,0,0,0,0],[0,1,0,0,0,0,0,0,0,0,0]]C0064434	ku 54	[[0,1,0,0,0,0,0,0,0,0,0]]C1097769	2 pyrido 1 2 e purin 4 yl amino ethanol,2 ppae	[[0,1,0,0,0,0,0,0,0,0,0],[0,1,0,0,0,0,0,0,0,0,0]]C0674739	cystothiazole a	[[0,1,0,0,0,0,0,0,0,0,0]]C1097765	6 methyl 5 oxo 5h benzo a phenothiazine	[[0,1,0,0,0,0,0,0,0,0,0]]C0538853	methyl ester alpha methyl 4 3 thienyl benzeneacetic acid,mehyl alpha methyl 4 3 thienyl benzeneacetate	[[0,1,0,0,0,0,0,0,0,0,0],[0,1,0,0,0,0,0,0,0,0,0]]C0065741	masudas compound v	[[0,1,0,0,0,0,0,0,0,0,0]]C0244404	raloxifene,keoxifene,6 hydroxy 2 4 hydroxyphenyl benzo b thien 3 yl 4 2 1 piperidinyl ethoxy phenyl methanone,ralox	[[0,0,0,0,0,0,0,1,1,1,0],[0,0,0,0,0,0,0,0,0,1,0],[0,0,0,0,1,0,0,0,0,0,1],[0,0,0,0,1,0,0,0,0,0,0]]C0538856	2 tris oleoyloxymethyl methylamino 1 ethanesulfonic acid	[[0,1,0,0,0,0,0,0,0,0,0]]C0538857	px 13	[[0,1,0,0,0,0,0,0,0,0,0]]C0538854	compound 88 765	[[0,1,0,0,0,0,0,0,0,0,0]]C0244400	isocycleanine	[[0,1,0,0,0,0,0,0,0,0,0]]C3257425	filipendula ulmaria leaf	[[0,0,1,0,0,0,0,0,0,0,0]]C0284398	piperazinium oleate	[[0,1,0,0,0,0,0,0,0,0,0]]C1981195	alprazolam 124 stool	[[0,0,0,1,0,0,0,0,0,0,0]]C0259544	2 6 dimethyl 9 methoxy 4h pyrrolo 3 2 1 ij quinolin 4 one,pq dmpqo,9 methyoxy 2 6 dimethyl 4h pyrrolo 3 2 1 ij qinolin 4 one	[[0,1,0,0,0,0,0,0,0,0,0],[0,1,0,0,0,0,0,0,0,0,0],[0,1,0,0,0,0,0,0,0,0,0]]C1985439	clomipramine 124 urine	[[0,0,0,1,0,0,0,0,0,0,0]]C1985438	clomipramine 124 milk	[[0,0,0,1,0,0,0,0,0,0,0]]C0636617	mpc 2101	[[0,1,0,0,0,0,0,0,0,0,0]]C0636616	cyclopropylmethyl 4 3 nitrophenyl 1 4 dihydro 2 6 dimethylpyridine 3 5 dicarboxylate	[[0,1,0,0,0,0,0,0,0,0,0]]C1121277	cdeu cpd,1 cyclohexyl 3 decyl urea	[[0,1,0,0,0,0,0,0,0,0,0],[0,1,0,0,0,0,0,0,0,0,0]]C1121276	cau cpd,1 adamantan 1 yl 3 cyclohexyl urea	[[0,1,0,0,0,0,0,0,0,0,0],[0,1,0,0,0,0,0,0,0,0,0]]C1121279	picovir	[[0,1,0,0,0,0,1,0,0,0,0]]C1121278	ctu cpd,1 cyclohexyl 3 tetradecyl urea	[[0,1,0,0,0,0,0,0,0,0,0],[0,1,0,0,0,0,0,0,0,0,0]]C1985433	clofazimine 124 isolate	[[0,0,0,1,0,0,0,0,0,0,0]]C1985432	clobenzorex 124 urine	[[0,0,0,1,0,0,0,0,0,0,0]]C0722796	pri cortin 50	[[0,0,0,0,0,0,0,1,0,0,0]]C1983252	butalbital 124 xxx	[[0,0,0,1,0,0,0,0,0,0,0]]C1985436	clomipramine 124 bld ser plas	[[0,0,0,1,0,0,0,0,0,0,0]]C1136665	3 6 di pyrazin 2 yl 1 2 4 5 tetrazine,dpztz cpd	[[0,1,0,0,0,0,0,0,0,0,0],[0,1,0,0,0,0,0,0,0,0,0]]C0539367	amylose tris,amylose tris s 1 phenylethylcarbamate,amylose tpec	[[0,1,0,0,0,0,0,0,0,0,0],[0,1,0,0,0,0,0,0,0,0,0],[0,1,0,0,0,0,0,0,0,0,0]]C0604093	camphidonium,trimethidinium methosulfate	[[0,1,0,0,0,0,0,0,0,0,0],[0,1,0,0,0,0,0,0,0,0,0]]C0966582	3 2 4 dichloroanilino 10 2 4 dichlorophenyl 2 10 dihydro 2 2 2 6 6 tetramethylpiperid 4 ylimino phenazine	[[0,1,0,0,0,0,0,0,0,0,0]]C0634618	thiomethoxyflurane,2 2 dichloro 1 1 difluoro 1 methylthio ethane	[[0,1,0,0,0,0,0,0,0,0,0],[0,1,0,0,0,0,0,0,0,0,0]]C3254803	inon ace tablet	[[0,0,0,0,0,0,0,1,0,0,0]]C0634611	sch 12223	[[0,1,0,0,0,0,0,0,0,0,0]]C0634612	n aminoiminomethyl n 2 6 dimethylphenyl urea	[[0,1,0,0,0,0,0,0,0,0,0]]C0634614	whr 1049	[[0,1,0,0,0,0,0,0,0,0,0]]C0015025	ethisterone,anhydrohydroxyprogesterone,pregneninolone,17 alpha ethynyltestosterone,17alpha 17 hydroxy pregn 4 en 20 yn 3 one,ethisterone preparation	[[0,0,0,0,0,0,1,1,0,1,0],[0,0,0,0,0,0,0,0,0,0,1],[0,0,0,0,0,0,0,0,0,0,1],[0,1,0,0,0,0,0,0,0,0,1],[0,0,0,0,0,0,0,0,0,0,1],[1,0,0,0,0,0,0,0,0,0,0]]C0015021	ethionamide,amidazine,ethioniamide,2 ethyl 4 pyridinecarbothioamide,2 ethylthioisonicotinamide	[[0,0,0,0,0,0,1,1,0,1,0],[0,0,0,0,0,0,0,0,0,0,1],[0,0,0,0,0,0,0,0,0,0,1],[0,0,0,0,0,0,0,0,0,1,1],[0,0,0,0,0,0,0,0,0,1,0]]C0015020	amifostine,aminopropylaminoethylthiophosphoric acid,apaetp,ethiofos,gammaphos,s n 3 aminopropyl 2 aminoethyl thiophosphoric acid,dihydrogen phosphate ester 2 3 aminopropyl amino ethanethiol,amifostine chemical,aminopropylaminoethylthiophosphoric acid trihydrate,s n 3 aminopropyl 2 aminoethyl thiophosphoric acid trihydrate,2 3 aminopropyl amino ethanethiol dihydrogen phosphate ester trihydrate,s 2 3 aminopropylamino ethylphosphorothioic acid trihydrate	[[0,1,0,0,0,0,1,0,0,1,0],[0,1,0,0,1,0,0,0,0,0,0],[0,0,0,0,1,0,0,0,0,0,0],[0,0,0,0,1,0,0,0,0,0,1],[0,0,0,0,1,0,0,0,0,1,0],[0,0,0,0,1,0,0,0,0,0,0],[0,0,0,0,0,0,0,0,0,0,1],[1,0,0,0,0,0,0,0,0,0,0],[0,0,0,0,0,0,0,0,0,1,0],[0,0,0,0,0,0,0,0,0,1,0],[0,0,0,0,0,0,0,0,0,1,0],[0,0,0,0,0,0,0,0,0,1,0]]C0015023	ethionine,s ethyl l homocysteine	[[0,0,0,0,0,0,0,0,1,0,1],[0,0,0,0,0,0,0,0,0,0,1]]C0650900	44 homooligomycin b	[[0,1,0,0,0,0,0,0,0,0,0]]C0629741	reboul cocktail	[[0,1,0,0,0,0,0,0,0,0,0]]C0629743	gadolinium 1 4 8 11 tetraazacyclotetradecane n n n n tetraacetic acid,gd teta	[[0,1,0,0,0,0,0,0,0,0,0],[0,1,0,0,0,0,0,0,0,0,0]]C0752933	lg 166 s,lg166s	[[0,1,0,0,0,0,0,0,0,0,0],[0,1,0,0,0,0,0,0,0,0,0]]C0953709	dl isomer arginine aspartate	[[0,1,0,0,0,0,0,0,0,0,0]]C0953708	c 137dihydrochloride	[[0,1,0,0,0,0,0,0,0,0,0]]C1956795	ioflupane	[[0,1,0,0,0,0,0,0,0,0,0]]C1956796	ioflupane,123i ioflupane,ioflupane i 123,methyl 8 3 fluoropropyl 3 beta p iodo sup123 i phenyl 1 alpha h 5 alpha h nortropane 2 beta carboxylate	[[1,0,0,0,0,0,0,0,0,0,0],[0,1,0,0,0,0,0,0,0,0,0],[0,0,1,0,0,0,0,0,0,1,0],[0,0,0,0,0,0,0,0,0,1,0]]C0953701	isomer 6 fluoronorepinephrine monohydrochloride	[[0,1,0,0,0,0,0,0,0,0,0]]C0953700	r isomer 3 fluoronorepinephrine	[[0,1,0,0,0,0,0,0,0,0,0]]C0953703	r isomer 6 fluoronorepinephrine	[[0,1,0,0,0,0,0,0,0,0,0]]C0953702	isomer 6 fluoronorepinephrine	[[0,1,0,0,0,0,0,0,0,0,0]]C0953705	8s cis isomer n n dimethyldoxorubicin hydrochloride,n n dimethyldoxorubicin monohydrochloride	[[0,1,0,0,0,0,0,0,0,0,0],[0,1,0,0,0,0,0,0,0,0,0]]C0614990	procion green h 4g	[[0,1,0,0,0,0,0,0,0,0,0]]C0614991	curpromeronic blue	[[0,1,0,0,0,0,0,0,0,0,0]]C1601282	canoxicabs	[[0,0,0,0,0,0,0,1,0,0,0]]C0614996	2 bromo 1 5 dihydroxy 3 pentanone 1 5 bisphosphate,1 9 dioxide 4 bromo 1 1 9 9 tetrahydroxy 2 8 dioxa 1 9 diphosphanonan 5 one,br pentanone p2	[[0,1,0,0,0,0,0,0,0,0,0],[0,1,0,0,0,0,0,0,0,0,0],[0,1,0,0,0,0,0,0,0,0,0]]C0245759	h norleucyl hexahydrotyrosyl lysine 4 nitroanilide,h nle hht lys pna,d norleucyl 3 4 hydroxycyclohexyl l alanyl l lysyl n 4 nitrophenyl l argininamide	[[0,1,0,0,0,0,0,0,0,0,0],[0,1,0,0,0,0,0,0,0,0,0],[0,1,0,0,0,0,0,0,0,0,0]]C0539721	fr 146687	[[0,1,0,0,0,0,0,0,0,0,0]]C0539720	4 1 4 1 4 isobutylphenyl butoxy benzoyl indolizin 3 yl butyric acid	[[0,1,0,0,0,0,0,0,0,0,0]]C0539727	mat 9460	[[0,1,0,0,0,0,0,0,0,0,0]]C0539726	oms 3052	[[0,1,0,0,0,0,0,0,0,0,0]]C0539725	o methoxy 2 1 1 dimethylethyl pyrimidinyl o methylethane phosphonate	[[0,1,0,0,0,0,0,0,0,0,0]]C0539724	ro 24 0409	[[0,1,0,0,0,0,0,0,0,0,0]]C0164927	26 27 diethyl 1 alpha 25 dihydroxycholecalciferol	[[0,1,0,0,0,0,0,0,0,0,0]]C0164926	26 27 ddhvd3	[[0,1,0,0,0,0,0,0,0,0,0]]C0164925	1 25 dihydroxy 26 27 diethylch
[truncated: 1,200,000 more chars]
