# Supplementary material for: Quantifying the Impact and Extent of Undocumented Biomedical Synonymy
Source: PLoS Comput Biol. 2014 Sep 25;10(9):e1003799. doi: 10.1371/journal.pcbi.1003799 (PMC4177665; doi:10.1371/journal.pcbi.1003799)
Supplement: Dataset S4 — The headwords and harvested synonym pairs obtained from the crowd-sourcing experiment. Each line in the file contains a provisional a headword, its part-of-speech, its harvested synonyms, and their associated posterior probabilities computed from the validation experiment. (ZIP) [file pcbi.1003799.s004.zip › SupportingDataset_S4.rtf]

Headword	POS	Validated Syn. Pairs	Posterior Probabilitiesmega	adjective	colossal,big,enormous,huge,epic,massive,giant,gigantic,jumbo,large,super	0.999958699698,0.999975949649,0.999565663164,0.99998633292,0.917684411399,0.999996189677,0.999987931763,0.999994067888,0.999965372071,0.999999220533,0.999959240654imaginarily	adverb	creatively,fantastically	0.999161836817,0.997650581111rebuttable	adjective	argumental,contradictive,questionable,refutable,arguable,disputable,disprovable	0.999996113108,0.996677848469,0.988425846586,0.999452438158,0.999995756847,0.997916103253,0.923618955707transitorily	adverb	fleetingly,temporarily,changeably	0.977566295961,0.999736020639,0.99945165072therewith	adverb	subsequently	0.901396312894yew	noun	shrub,balsa	0.989302781,0.96459743055raisin	noun	date,grape,fruit	0.950563550558,0.973454288687,0.998684776951phenotype	noun	trait	0.999324137008hereto	adverb	subsequently	0.958955702009cowpea	noun	legume,food	0.99977948641,0.999684008476noteworthily	adverb	prominently,significantly,notably,valuably,interestingly,uniquely,evidently,remarkably,famously,importantly	0.992847476667,0.999997211683,0.999994140876,0.999948533389,0.969570949962,0.999362885594,0.938600639119,0.993504428894,0.999994065021,0.999997962947audio	adjective	phonic,audible,sound,auditory,stereo,acoustic	0.999304976955,0.983918991802,0.999982558778,0.999997813497,0.998076407544,0.999981880213postoperative	adjective	postsurgical,postprocedure,convalescent,postsurgery	0.99996154644,0.999015527274,0.991286313917,0.999912758497telegraphese	noun	communication,shorthand,morse	0.961816000603,0.995270654902,0.990684090706pupal	adjective	immature,developmental	0.966994924876,0.967231909983metropolitanisation	noun	development,civilization	0.999713604414,0.96970982051aminophenol	noun	chemical	0.965109776586astrophysicist	noun	astronomer,stargazer,cosmologist,scientist	0.976846235117,0.999628596686,0.963695044833,0.999849481757archipelago	noun	isle,island	0.995824463974,0.996636950545rudimentarily	adverb	basically,simply,fundamentally	0.999480875539,0.999948533389,0.997812355322hereof	adverb	respectively,about,now	0.961852591925,0.99901867179,0.999400485421succinate	noun	sweetener	0.951898740976autism	noun	impairment,disability	0.974068928514,0.978744052404metastable	adjective	changeless,unchanging	0.99661627368,0.963763694927illusorily	adverb	imaginarily,deceptively	0.999987052682,0.999817030211cohesive	adjective	gummy,viscous,sticky	0.999035478455,0.98855281781,0.999990595585intramural	adjective	inside,communal,intragroup	0.98162033919,0.965800024403,0.968975597632bog	verb	stop,slow,stall	0.984755699868,0.974068928514,0.998026454922yucca	noun	plant	0.993130786602midweekly	adjective	midway	0.939182643202bubonic	adjective	diseased	0.966912044828demagnification	noun	shrink,decrease,shrinkage,reduction	0.999850809477,0.999401067279,0.99434357009,0.999843183323broadcloth	noun	dress,material,fabric	0.959905795373,0.999890659336,0.976957392419reconciliatory	adjective	forgivable,amendable,peaceable,agreeable	0.998879732647,0.983530407886,0.999202989896,0.918580258043quasistationary	adjective	static,slow	0.953053484997,0.992122096729sundress	noun	dress,toga,garment	0.999063715425,0.995815991182,0.999248072489ski	noun	activity	0.933055341192reverberatory	adjective	resonant,vibrative	0.999911009942,0.97570666689overtime	noun	extra,aftertime	0.999888935315,0.977761849939ovenware	noun	cookery,pyrex,crockery,cookware	0.989888188614,0.916032351633,0.926541569824,0.999993361514departmentalise	verb	delegate,organize,isolate,categorize,separate,segregate,section,divide	0.968548051478,0.999970278518,0.999257322639,0.996644143183,0.934708772702,0.999899889668,0.999978334179,0.996788695326upstate	adjective	northern,northerly,north,northward	0.99986411783,0.999904296166,0.9607397448,0.993271375526venography	noun	venogram,radiation	0.953709694671,0.969380533196underpants	noun	underwear,pantie,boxer,lingerie,brief,undergarment	0.999975949649,0.999943389302,0.968894006061,0.999982036845,0.998686534213,0.99998810501acrylic	adjective	plastic	0.97910751201skywriter	noun	pilot	0.984553712281electrophysiologist	noun	doctor	0.98132055237costal	adjective	expensive	0.924680816983preprepared	adjective	ready,armed,planful	0.999996563807,0.981174308224,0.999919547627sulkily	adverb	sorely,cheerlessly,sullenly,bitterly,sadly	0.99793456367,0.999462632169,0.999762377175,0.999087995333,0.995446891694collegian	noun	professor,graduate,learner,student,academic,classmate,pupil	0.99930578064,0.999752734363,0.997751527926,0.999994737203,0.99823193011,0.992977234127,0.99918795916disorientation	noun	chaoticity,fluster,delirium,dizziness,confusion	0.999027242905,0.978207976629,0.99969167849,0.999736012603,0.999979037029rudd	noun	scarlet	0.985661171978sigma	noun	glyph,symbol	0.988805125574,0.999808867007gastrula	noun	embryo	0.90475038128intermediacy	noun	middle	0.987742957959silty	adjective	turbid,sandy,earthy	0.940661993558,0.99823193011,0.967033636842facial	adjective	frontal,front	0.919514711965,0.967381527967refertilise	verb	enrich,reproduce	0.997319178183,0.999754750807parsnip	noun	vegetable	0.93667067279turbo	adjective	fast,swift,speedy,super,hyper,rapid,quick,supersonic	0.990772028636,0.999816280788,0.997370462039,0.92675518256,0.999164427062,0.999773138937,0.998475118459,0.983911381068okapi	noun	mammal,ram,herbivore,beast	0.967795571566,0.967730453976,0.969650223139,0.953228155656marmot	noun	mammal,animal	0.997251841924,0.922214769831fyke	noun	net	0.986629154069hereditarily	adverb	characteristically,intergenerationally,traditionally,transgenerationally,ancestrally,genetically,inheritably	0.99999752478,0.998717199599,0.983546095213,0.999203799266,0.99998343485,0.999992808774,0.999210438684unabated	adjective	relentless,sustained,persistent,incessant,unrelenting,continual,determined,unstoppable	0.999853034581,0.973084802979,0.999203799266,0.998338389675,0.999121929163,0.99833673424,0.989728728575,0.999869495494cucurbit	noun	gourd	0.904198558516perisarc	noun	case	0.926787373875anal	adjective	picky,fastidious,fussy,rectal	0.983113780883,0.994387800232,0.989513355878,0.999985437222homeopathic	adjective	alternative	0.984550449884alfa	noun	primary	0.973392792142hypodermis	noun	skin,stratum,covering,tissue,layer	0.999992776052,0.922267335256,0.991057009947,0.990017767154,0.99792190549antifebrile	noun	remedy	0.973392792142smoggy	adjective	hazy,smoky,foggy,mucky,pollutional,murky	0.999948981053,0.999994940878,0.999748361303,0.983636274216,0.999967389338,0.963763694927thousandfold	adverb	numerously,multiplicately,excessively,much,plentifully,abundantly	0.999544165156,0.99930578064,0.991728059308,0.999334916612,0.997016233581,0.99949536871rheometer	noun	instrument	0.945638863554comedic	adjective	witty,jovial,comic,humorous,merry,comical,amusing,hilarious,funny	0.999991323915,0.999544165156,0.995089082286,0.999576487875,0.999679273184,0.999991753554,0.999913477527,0.999989575837,0.999983349946toxigenicity	noun	poison,virulence,poisonousness	0.965197123251,0.950044630721,0.998085546592unitarily	adverb	singly,individually,indivisibly,unitedly,wholly,singularly	0.999107241786,0.930467148333,0.987893919809,0.984646504074,0.999911311243,0.999985437222stromatolite	noun	form	0.998192532087tricot	noun	fiber	0.972069078693vert	noun	green	0.999831650595ventilatory	adjective	airy	0.999987899843cholinesterase	noun	enzyme	0.996171372239moulage	noun	fake	0.953228155656inboard	adjective	inside	0.991857808627up	preposition	above	0.99998343485portliness	noun	girth,flab,chubbiness,plumpness,pudginess,stoutness,fatness,fat,obesity,largeness,heftiness	0.999737778912,0.999506343158,0.999929573204,0.999877850579,0.999992937853,0.999911009942,0.999973729037,0.999688531455,0.999975850007,0.999960183801,0.999978334179secondarily	adverb	additionally,supplementarily,subsequently,alternately,latterly,subordinately,consequentially,later,next	0.999986826108,0.934480320369,0.999996688916,0.999798202882,0.999556638341,0.999955977362,0.940039929188,0.997843386865,0.999997813497engrail	verb	indent,mark	0.998587535051,0.99996428748volcanic	adjective	eruptive,explosive,seismic	0.997397122847,0.972469788782,0.916235051305ineradicable	adjective	durable,indestructible,permanent	0.985827188435,0.999831004231,0.982076624042plumply	adverb	fully,stoutly,thickly,heavily,roundly	0.970734128277,0.999967020232,0.981133937926,0.957978085275,0.999987673444remobilize	verb	rouse,move,reanimate	0.928544623698,0.999995539278,0.999899889668ducal	adjective	princely	0.985981647535odontograph	noun	instrument	0.959652377103flatwise	adverb	flatly,horizontally,laterally,flat	0.967315046415,0.999971886736,0.993615225571,0.990147363728unguessable	adjective	unpredictable,unknowable,vague,cryptic,mysterious,inestimable	0.999895166841,0.999605271794,0.99954500105,0.999151502577,0.999960183801,0.999996346665drumstick	noun	leg,instrument	0.999689359136,0.984330722314manyfold	adverb	exponentially,multiplicately,expandingly,numerously,abundantly	0.998221323054,0.995407930845,0.966840261461,0.999982455565,0.99991617696sixtyfold	adverb	generously,abundantly,numerously	0.980074636104,0.999296674589,0.98466151751instinctual	adjective	unintentional,intuitive,impulsive,primal,natural,innate,ingrained,instinctive	0.987742957959,0.999996376558,0.991251633705,0.999979244453,0.999480875539,0.98346956762,0.952940317861,0.998012597786disconfirm	verb	refute,renege,undermine,deny,invalidate	0.940775644641,0.984579333451,0.999844774487,0.989957992849,0.999254472102headward	adverb	onward,forward,frontally,ahead	0.999555704583,0.965632537229,0.991455397451,0.998076407544pliably	adverb	compliantly,flexibly	0.936621679798,0.999075828694bisulfite	noun	chemical,crystal,acid	0.969930219838,0.924920924681,0.967795571566hysterectomize	verb	cut,sterilize,remove	0.998441677288,0.954156273902,0.998891233704distal	adjective	isolated	0.988781222149strapless	adjective	bandless,unsupported	0.999030136337,0.959905795373avant	adjective	new	0.989833657533phonocardiogram	noun	recording,record	0.971783578695,0.999178099481reutilization	noun	recycler	0.94766687586orthodoxly	adverb	conventionally,traditionally,strictly,devoutly,conformally,faithfully	0.999295342284,0.999989113257,0.999240413991,0.972532057662,0.963695044833,0.979904895554embrittlement	noun	frailness,fragility,enfeeblement	0.999996503601,0.998129578814,0.984516131096exemplarily	adverb	superbly,impressively,excellently,commendably,ideally,admirably,superiorly,faultlessly,honorably,supremely	0.99999898048,0.999689359136,0.999981044293,0.999073615708,0.996725732421,0.982116833613,0.998969523602,0.9331783303,0.972184755872,0.999996832173tidal	adjective	wavy	0.996362948904leftmost	adjective	liberal	0.999962434039viral	adjective	infected,contagious,sickly,diseased	0.999994940878,0.999786976966,0.971204121542,0.998945220334motherless	adjective	bastard	0.988620057213periglacial	adjective	glacial	0.965606746572slangily	adverb	colloquially,casually	0.973084802979,0.974715706953underexpose	verb	darken,hide	0.984327631789,0.969136381156gherkin	noun	pickle	0.992042046844whereupon	adverb	thereupon,thus,consequently	0.981956096894,0.999463435609,0.946659403234waveform	noun	wave,swell,curve	0.979562935849,0.910594231573,0.988684457894hydrobiologist	noun	scientist	0.909645430171kaiser	noun	baron,leader,bread,king,ruler,emperor	0.999180006355,0.907265570964,0.999373511192,0.999642242105,0.978083303224,0.909598598886galvanoscope	noun	tool,device,contraption,instrument	0.919393955918,0.933055341192,0.903908016719,0.997501468467enchondroma	noun	cyst,cell	0.996812610641,0.999895166841microsporangium	noun	cell	0.99937313303breezily	adverb	casually,gently,easily,happily,carelessly,effortlessly	0.967319520748,0.961059219269,0.99983416454,0.991133292144,0.986619608645,0.99977665132windowless	adjective	dark	0.900925778144pecuniarily	adverb	fiscally,financially	0.973096594486,0.999798202882sleazily	adverb	shabbily,disgustedly	0.998480125969,0.998582088199hypermnesia	noun	memorization	0.938948523909citrulline	noun	acid	0.95830230238guitar	noun	banjo,instrument,lute	0.967993794992,0.982381841942,0.967730453976deluster	verb	damage,darken,scuff,fade	0.946722612413,0.999334916612,0.990417515211,0.987283796642fragmentarily	adverb	fractionally,sectionally,partly,partially,piecemeal,segmentally,incompletely	0.999762193666,0.971551398029,0.999648796728,0.998591104273,0.945279416949,0.993578513016,0.99996428748graphitisation	noun	graphitization	0.999003747457roaster	noun	broiler,pot,cooker,fireplace,pan,oven,grill,griddle,barbeque,barbecue	0.989888188614,0.996812610641,0.999983726551,0.992114905217,0.999882467112,0.999995073366,0.999817030211,0.994214905336,0.963695044833,0.981909623425homopolar	adjective	identical,similar,consistent	0.943728009447,0.998238588546,0.95711276088footage	noun	tape,picture,movie,recording,scene,clip,film,flick	0.998727072565,0.974325933876,0.995244246055,0.989859136199,0.996182685367,0.999421061579,0.999992207924,0.999543064153desultorily	adverb	haphazardly,carelessly	0.929576398675,0.999612446018focal	adjective	central,main,principal,centered,primary	0.999744660522,0.917392788568,0.984288802538,0.999944384694,0.998304641946senatorial	adjective	political,governmental,parliamentary,congressional	0.977143238771,0.99915204774,0.999803228596,0.997251841924isodynamic	adjective	similar	0.9993087697discriminatorily	adverb	wrongly,prejudicially,selectively,unfairly	0.969039931269,0.999874548617,0.999164427062,0.980047041937monkish	adjective	churchly,monastic,religious,silent,reclusive,holy,studious	0.937877785922,0.94547475081,0.999524820655,0.989772194309,0.995835701972,0.988684457894,0.988952103849lambda	noun	symbol	0.945638863554unforgivingness	noun	mercilessness,grudge,punishment,stubbornness	0.999565663164,0.999273408987,0.959204521136,0.959085919258reoccur	verb	lapse,return,echo,reappear,repeat,relapse,recur	0.95511907494,0.999450097261,0.999639793507,0.999201536231,0.999477314417,0.999829521185,0.999648931319chlorohydrin	noun	halohydrin	0.924831079046vinaigrette	noun	vinegar,marinade,sauce,dressing,condiment	0.99035639382,0.999772866437,0.998022894631,0.919393955918,0.995634007904unprescribed	adjective	otc,unordered,unassigned	0.990501195557,0.998782659007,0.998687807299petrolatum	noun	oil,fuel,petrol,gasoline,jelly	0.985800629733,0.997926492111,0.999982558778,0.999974294707,0.999615755858absentia	noun	truancy,vacancy,nonexistence,absence,inexistence,hiatus,nonappearance,void	0.999992776052,0.999948981053,0.992553290255,0.999908305152,0.999301306038,0.999992710588,0.999735021279,0.956002200983inflammatorily	adverb	provocatively,bitterly,angrily	0.999975658621,0.999017294653,0.999990182298clingy	adjective	needy,dependant,obsessive,close,insecure,attached,sticky,tight,gooey	0.916335545542,0.999363993246,0.982788086919,0.986668241581,0.984579333451,0.99823193011,0.999993778165,0.996496287642,0.914663030882olfactorily	adverb	nasally,sensorially	0.998837293288,0.99931049963submucosa	noun	tissue	0.99793456367complimentarily	adverb	kindly,appreciatively,admirably,approvingly	0.975454601841,0.909937602097,0.915660228665,0.963315108649fir	noun	cedar,wood,pine,evergreen	0.967315046415,0.999240413991,0.916032351633,0.999975688678nimbostratus	noun	cloud,vapor,fog	0.994341555302,0.960046665118,0.972069078693dyer	noun	colored,stain	0.921312523352,0.962572492302monkhood	noun	priest,hermit	0.999304976955,0.961313856443wavily	adverb	crookedly	0.997239448286dental	adjective	oral,orthodontic,toothy	0.925995917301,0.999912142731,0.922214769831caraway	noun	fennel	0.949344501822denitrify	verb	reduce	0.959406647012stewardship	noun	duty,leadership,care	0.933600223128,0.989081330402,0.976215931041since	preposition	after	0.999863991611reemphasise	verb	indicate,stress,reiterate,accent,repeat,underscore	0.967493696922,0.999889779759,0.997008663953,0.971098140612,0.999615755858,0.97910751201contradictorily	adverb	oppositionally,opposingly,oxymoronically,inversely,reversely,oppositely,problematically,differently	0.99974304879,0.99832842674,0.952284778878,0.994431582405,0.948145451046,0.999968014188,0.909645430171,0.999773138937millet	noun	crop,grain,grass,cereal	0.965632537229,0.965594092855,0.975927417226,0.999786976966faultily	adverb	wrongly,erroneously,badly,inadequately,defectively,incorrectly	0.999295342284,0.999882724994,0.990220673369,0.999739173047,0.999959580344,0.999956255196coronary	adjective	cardiovascular,arterial,blooded,cardiac	0.994341555302,0.99998633292,0.92675518256,0.999138331292abed	adjective	relaxant	0.916235051305revolutionarily	adverb	innovatively,progressively	0.999950728691,0.997370462039riverbed	noun	bank,creek,floor	0.994809267734,0.988783839399,0.960360105122discommend	verb	disapprove,reproach,reject,disparage,belittle,disvalue,discourage,disavow,criticize,denounce,disown	0.999995077684,0.997934963096,0.99974304879,0.989741010405,0.999947405477,0.999218652433,0.999808867007,0.990346330177,0.992032327761,0.999955977362,0.989859136199lye	noun	soap,chemical	0.999078644627,0.973514857831predatorily	adverb	parasitically,cruelly,threateningly,devilishly	0.967993794992,0.998022894631,0.995422744059,0.968301756921ecospecies	noun	organism,ecotype,subspecies,type	0.996028882916,0.938988321332,0.926661627547,0.938948523909programmist	noun	operator,technician,programmer,programer,profession,implementor,organizer,coder,designer	0.989888188614,0.999794815584,0.999950819623,0.999997806068,0.975927417226,0.975963601757,0.983636664616,0.99999158657,0.999788389379misregistration	noun	mistake,falsity,error,inaccuracy	0.93677828135,0.967650782306,0.999886125068,0.998662780709unhealthily	adverb	destructively,badly,hazardously	0.999990654492,0.902820305193,0.961414386069ultravirus	noun	infection,disease,illness,contagion	0.998950593464,0.996725732421,0.948681072689,0.993634391171stomal	adjective	open	0.967315046415complementarily	adverb	admirably	0.996256470496watermelon	noun	melon,fruit,gourd	0.99996154644,0.989252010819,0.96180997624reliant	adjective	dependable,trustful,needy,dependent	0.999975685751,0.99993114071,0.910349774106,0.999781450394counterintuitive	adjective	opposite	0.949656664434expansionary	adjective	increasing,progressive,broad,widespread,increased	0.999995077684,0.999712798737,0.985548170944,0.996496287642,0.999978334179quadruply	adverb	fourfold,moreover,multiplicatively	0.999889779759,0.985661171978,0.935751292509seaborne	adjective	marine,aquatic,oceanic,nautical	0.998501905276,0.999987931763,0.9999414697,0.920242650843crouch	noun	low,duck,huddle,position,hunch,bend,squat,stoop	0.939004710769,0.999643486525,0.988199541758,0.983918991802,0.987269087749,0.992616958946,0.999957614416,0.999899649013mesogastrium	noun	intestine	0.924920924681rotameter	noun	instrument,device	0.980650073686,0.917176679443trichloroethanol	noun	drug	0.987893919809hula	noun	dance	0.975603791137feebly	adverb	weakly,timidly,inadequately,faintly	0.999898162596,0.99976210013,0.999092669153,0.978288333128vagility	noun	migration,maneuverability,mobility	0.926661627547,0.929086178698,0.972098152173endothelial	adjective	cellular	0.962743913132reflexly	adverb	habitually,spontaneously,instinctively,automatically,involuntarily	0.991022260689,0.956681892584,0.999995913795,0.999956730414,0.9998518223foreseeable	adjective	anticipative,expectable,predictable	0.985151196861,0.998902826576,0.99999391586muddily	adverb	confusedly,sloppily	0.99762731658,0.983927212859bodyweight	noun	mass,weight,bmi	0.999544165156,0.982566868301,0.943790212705foolhardily	adverb	irresponsibly,unthinkingly,recklessly,heedlessly,thoughtlessly,incautiously,naively,foolishly	0.999999234198,0.999970364117,0.999982516954,0.995870392504,0.999803228596,0.999979872748,0.940984929218,0.999684249554unaided	adjective	unguided,unsupervised,helpless,alone,independent,unsupported,unassisted	0.99857293354,0.999823988982,0.982998003723,0.998702376426,0.99998373126,0.989080822592,0.999968818697thrombosis	noun	stroke,angina,clot	0.997742474753,0.962760026434,0.999092669153supernaturalist	noun	medium,psychic,clairvoyant,spiritualist,witch,mystic	0.999833306354,0.99945102821,0.997909909717,0.999992780628,0.99937313303,0.999850265167olden	adjective	archaic,ancient,aged,old,past,medieval	0.999463435609,0.999830396338,0.961702493546,0.999369362912,0.999989507223,0.999254229499spinor	noun	backbone	0.999863433041
