# Supplementary figures and images for: Quantifying the Impact and Extent of Undocumented Biomedical Synonymy
Source: PLoS Comput Biol. 2014 Sep 25;10(9):e1003799. doi: 10.1371/journal.pcbi.1003799 (PMC4177665; doi:10.1371/journal.pcbi.1003799)

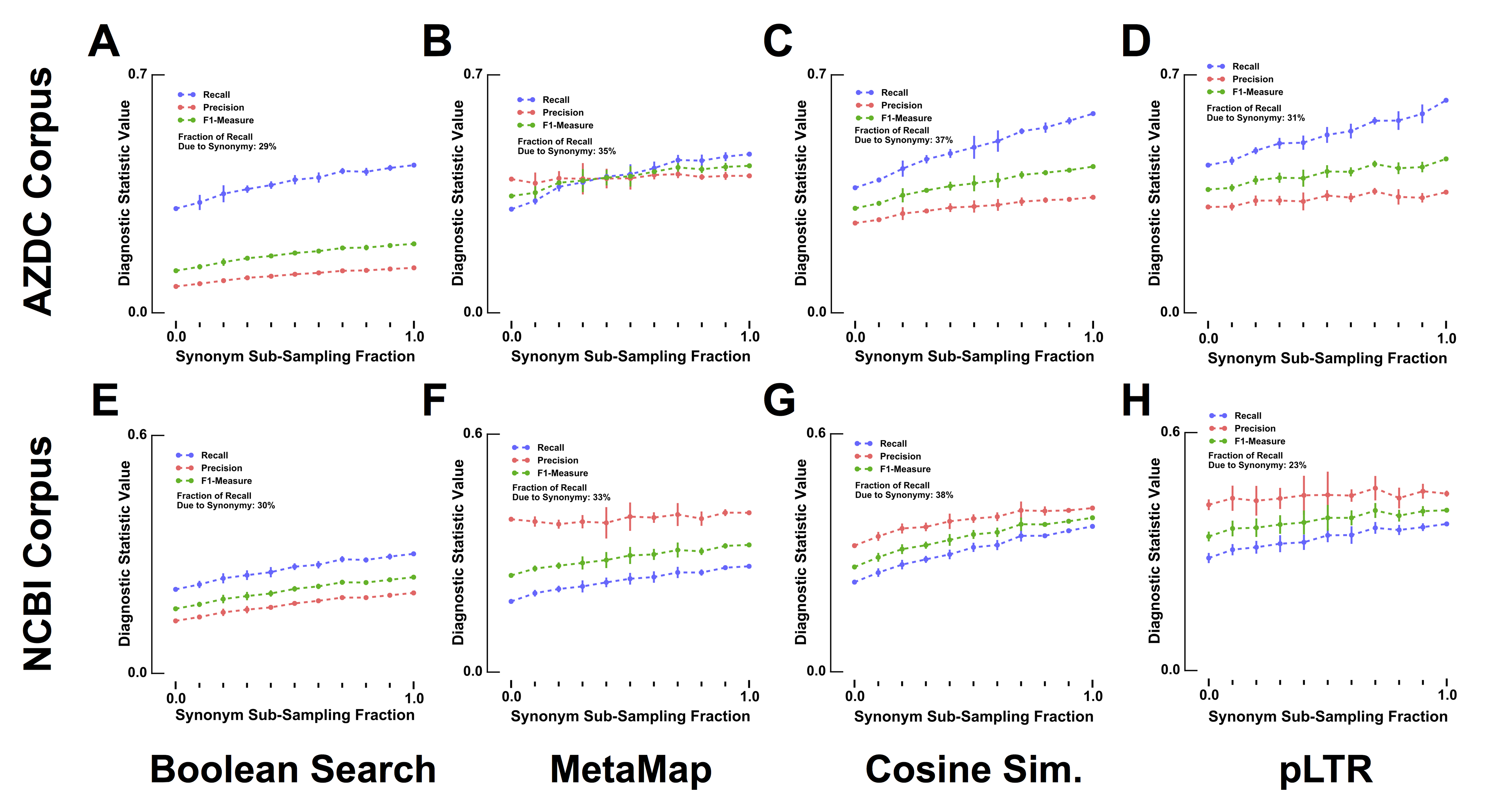

Supplement: Figure S1 — Missing synonymy negatively affects disease name normalization. To test the importance of synonymy for named entity normalization, we removed random subsets of synonyms from the Diseases and Syndromes terminology (x-axes indicate the fraction remaining) and computed recall (blue), precision (red), and their harmonic average (F1-measure, green) (y-axis) for four normalization algorithms (bottom) applied to two disease name normalization gold-standard corpora (left). Error bars represent twice the standard error of the estimates, computed from five replicates. Numerical results are presented in Table 1, and a description of the methodology is provided in the Materials and Methods and the Supporting Information Text S1. (TIF) [file pcbi.1003799.s005.tif]

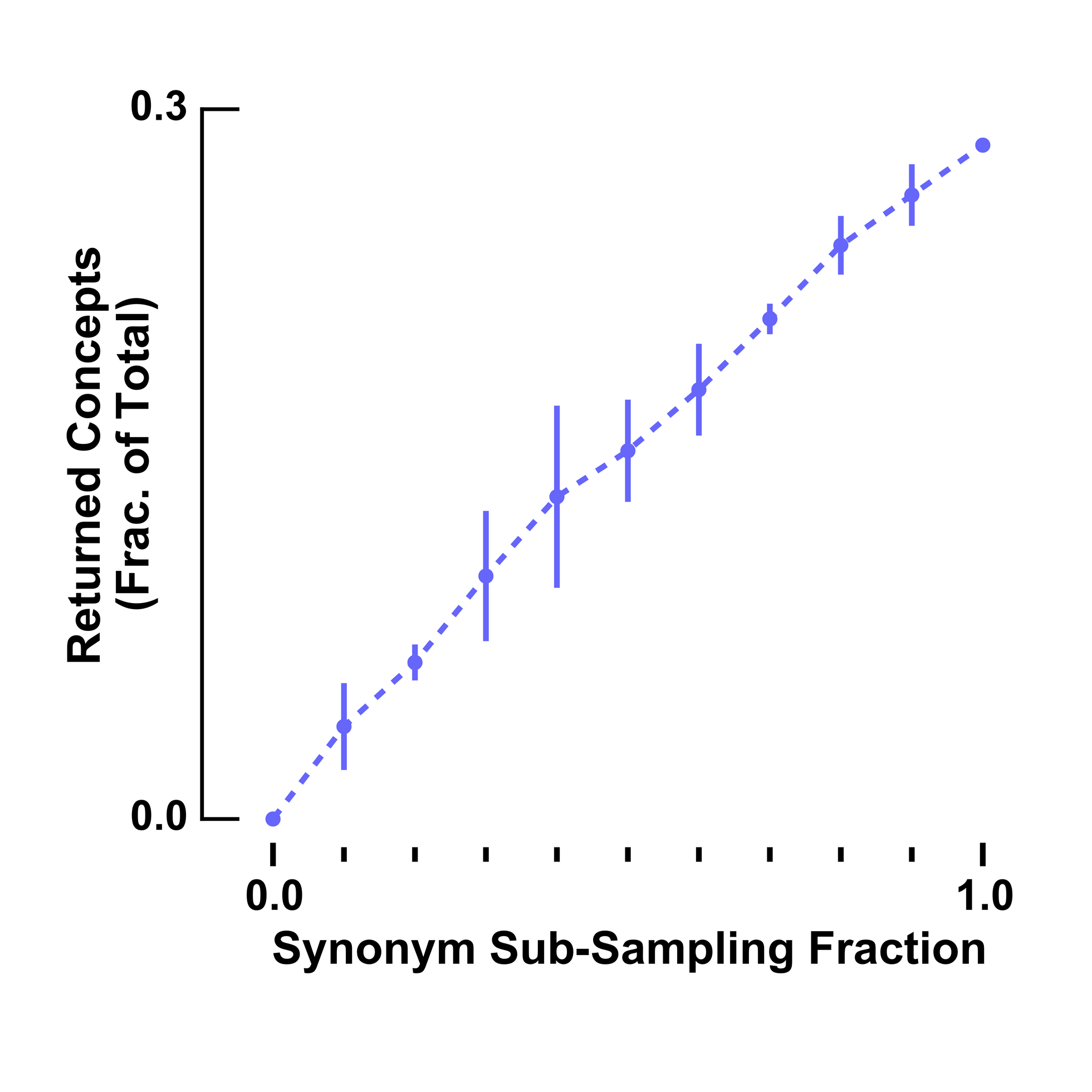

Supplement: Figure S2 — Recall of normalized Pharmacological Substances depends on synonymy. The fraction of the total number of recalled concepts returned by MetaMap (y-axis) upon removing a subset of the synonyms contained within the Pharmacological Substances terminology (x-axis indicates fraction remaining). The evaluation corpus consisted of 35,000 unique noun phrases isolated from MEDLINE (see Materials and Methods for details). (TIF) [file pcbi.1003799.s006.tif]

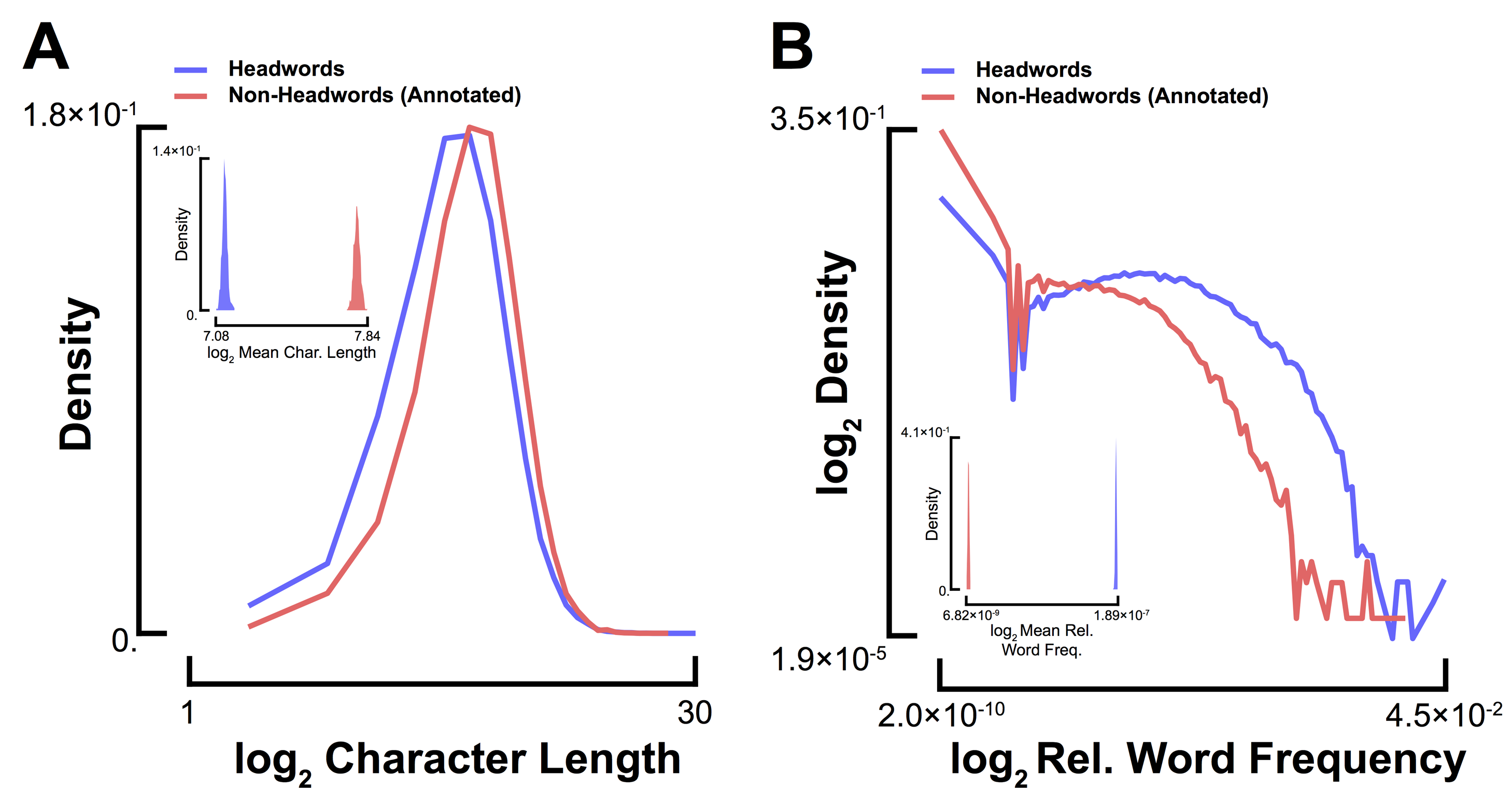

Supplement: Figure S3 — Headword selection bias in general-English thesauri. (A) The empirical distribution over stemmed word length shown for headwords (blue) and non-headwords (synonyms only, red). The inset panel depicts bootstrapped estimates (1000 re-samples) for the mean values of these two distributions. (B): Relative word frequency of headwords (blue) and non-headwords (synonyms only, red). In both cases, a Student's T-test for a difference in means produced a p-value <2.2×10^−16^. (TIF) [file pcbi.1003799.s007.tif]

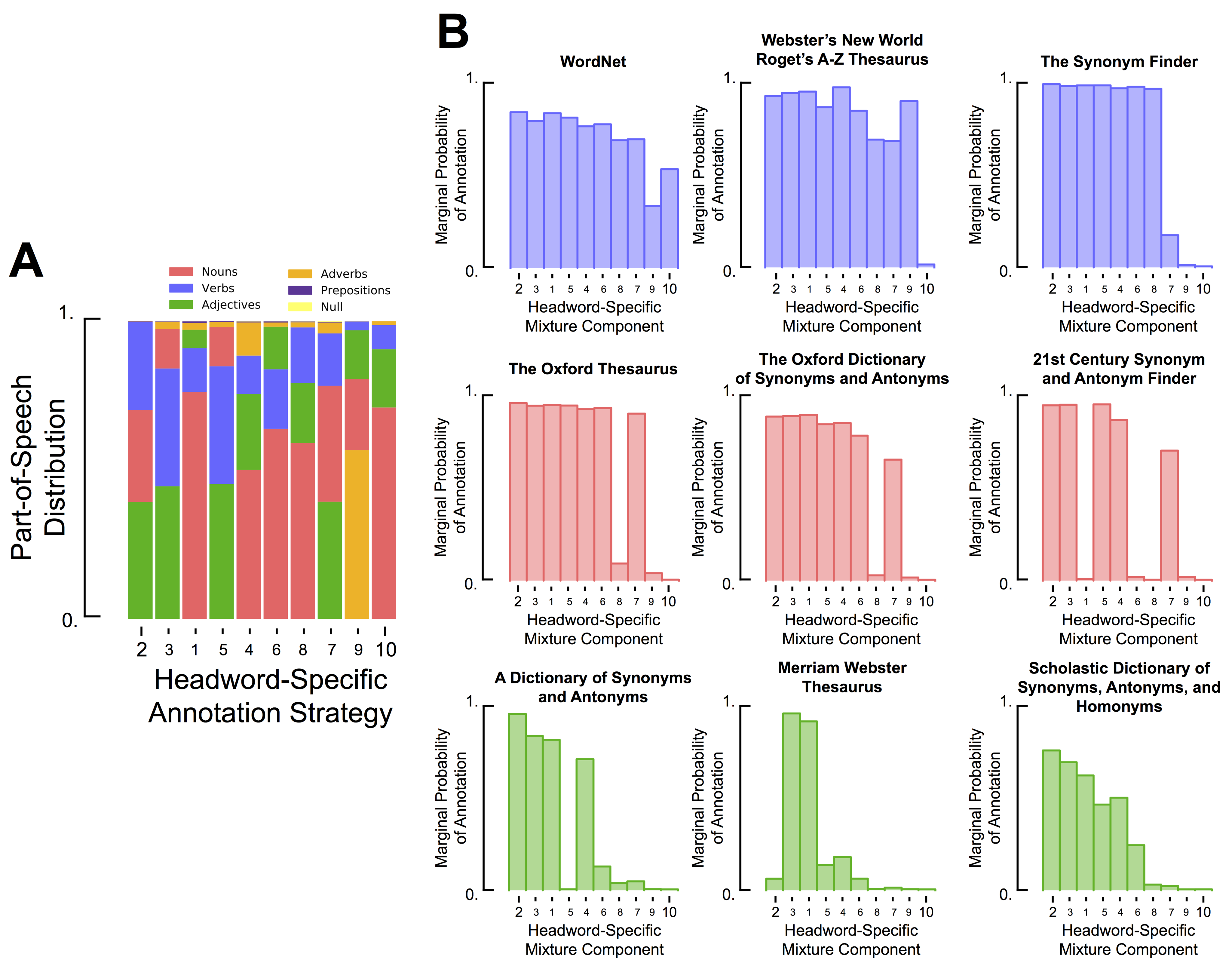

Supplement: Figure S4 — Bias and variability captured by the annotation mixture model. (A) The distributions over parts-of-speech across the ten headword components specified within the best-fitting mixture model. (B): The probability of headword annotation, marginalized over all possible numbers and classes of synonyms, for the complete set of nine, general-English thesauri. (TIF) [file pcbi.1003799.s008.tif]
