# Supplementary material for: Quantifying the Impact and Extent of Undocumented Biomedical Synonymy
Source: PLoS Comput Biol. 2014 Sep 25;10(9):e1003799. doi: 10.1371/journal.pcbi.1003799 (PMC4177665; doi:10.1371/journal.pcbi.1003799)
Supplement: Table S1 — Examples of missing synonyms annotated within the gold-standard disease name normalization corpora. The first column indicates the term mentioned in the text, while the second column provides the annotated concept. The third column indicates the corpus of origin. Algorithms considered in this study did not properly normalize any examples provided here presumably because the synonym was not provided in the complete disease name terminology. (PDF) [file pcbi.1003799.s009.pdf]

Mention in Text (Missing Synonym)	Correct Concept	Corpus of Origin
attenuated fap	attenuated adenomatous polyposis coli	AZDC
c2 deficient	complement 2 deficiency	AZDC
inherited breast ovarian cancer	hereditary breast and ovarian cancer syndrome	AZDC
multi system disorder	multisystem disorder	AZDC
cgkd	deficiency of glycerol kinase	AZDC
colorectal tumorigenesis	colorectal neoplasm	AZDC
coats telangiectasis	exudative retinopathy	AZDC
searching gaze	congenital nystagmus	AZDC
congenital forms of dm	congenital myotonic dystrophy	AZDC
neonatal ald	neonatal adrenoleukodystrophy	NCBI
breast ovary cancer family syndrome	hereditary breast and ovarian cancer syndrome	NCBI
pts1 protein import defect	zellweger syndrome	NCBI
searching gaze	congenital nystagmus	NCBI
attenuated fap	attenuated adenomatous polyposis coli	NCBI
wilms tumorigenesis	nephroblastoma	NCBI
frataxin deficiency	friedreich ataxia	NCBI
chronic neisserial infection	neisseriaceae infections	NCBI
l n syndrome	lesch nyhan syndrome	NCBI
x linked recessive thrombocytopenic disorder	x linked thrombocytopenia	NCBI
dmpk deficient	dystrophia myotonica 1	NCBI
palmoplantar hyperkeratosis	palmoplantar keratosis	NCBI
oedemic buccal mucosa	oral leukoedema	NCBI

Examples of disease name mentions that were not correctly normalized by the four algorithms considered in this study as a result of undocumented synonymy. Note, some of these errors are replicated in both corpora, indicating that they are not isolated annotations.
