# Supplementary material for: Quantifying the Impact and Extent of Undocumented Biomedical Synonymy
Source: PLoS Comput Biol. 2014 Sep 25;10(9):e1003799. doi: 10.1371/journal.pcbi.1003799 (PMC4177665; doi:10.1371/journal.pcbi.1003799)
Supplement: Table S2 — The sources for the Diseases and Syndromes dataset. Summary statistics for the thirteen thesauri used to construct the Diseases and Syndromes terminology. (PDF) [file pcbi.1003799.s010.pdf]

Table S 2. The Sources for the Diseases and Syndromes Dataset

Thesaurus	Database Code	# of Concepts	# of Terms
SNOMED Clinical Terms	SNOMEDCT	35,249	60,592
Medical Subject Headings	MSH	8,493	16,617
COSTAR	COSTAR	1,501	1,629
Online Mendelian Inheritance in Man	OMIM	8,061	18,167
International Classification of Diseases, Ninth Revision, Clinical Modification	ICD9CM	4,293	4,312
Physician Data Query	PDQ	1,439	3,705
UMLS Metathesaurus	MTH	1,931	1,949
Consumer Health Vocabulary	CHV	8,281	16,390
COSTART	CST	1,378	1,870
CRISP Thesaurus	CSP	1,430	1,891
DXplain	DXP	2,594	4,305
NCI Thesaurus	NCI	11,514	24,903
National Drug File	NDFRT	2,756	5,781
International Classification of Diseases, Ninth Revision, Clinical Modification, Metathesaurus additional entry terms, 2012	MTHICD9	5,694	7,146
Total	NA	59,265	127,431

Summary statistics for the fourteen thesauri used to construct the Diseases and Syndromes terminology.
