# Supplementary material for: Quantifying the Impact and Extent of Undocumented Biomedical Synonymy
Source: PLoS Comput Biol. 2014 Sep 25;10(9):e1003799. doi: 10.1371/journal.pcbi.1003799 (PMC4177665; doi:10.1371/journal.pcbi.1003799)
Supplement: Table S3 — The sources for the Pharmacological Substances dataset. Summary statistics for the ten thesauri used to construct the Pharmacological Substances terminology. (PDF) [file pcbi.1003799.s011.pdf]

**Table S 2. The Sources for the Pharmacological Substances Dataset**

| Thesaurus | Database Code | # of Concepts | # of Terms |
|---|---|---|---|
| SNOMED Clinical Terms | SNOMEDCT | 7,014 | 10,487 |
| Medical Subject Headings | MSH | 79,886 | 130,355 |
| Metathesaurus FDA Structured Product Labels | MTHSPL | 3,183 | 3,294 |
| LOINC | LNC | 4,479 | 4,528 |
| Physician Data Query | PDQ | 2,463 | 4,092 |
| UMLS Metathesaurus | MTH | 1,791 | 1,793 |
| Consumer Health Vocabulary | CHV | 7,738 | 10,190 |
| RxNorm Vocabulary | RXNORM | 22,370 | 22,377 |
| CRISP Thesaurus | CSP | 1,213 | 1,472 |
| NCI Thesaurus | NCI | 15,696 | 29,945 |
| National Drug File | NDFRT | 5,413 | 9,596 |
| Total | NA | 122,366 | 198,270 |

Summary statistics for the eleven thesauri used to construct the Pharmacological Substances terminology.
