# Supplementary material for: Quantifying the Impact and Extent of Undocumented Biomedical Synonymy
Source: PLoS Comput Biol. 2014 Sep 25;10(9):e1003799. doi: 10.1371/journal.pcbi.1003799 (PMC4177665; doi:10.1371/journal.pcbi.1003799)
Supplement: Table S4 — The sources for the general-English dataset. Summary statistics for nine thesauri used to construct the general-English near-synonym terminology. (PDF) [file pcbi.1003799.s012.pdf]

Table S 4. The Sources for the General-English Dataset

Thesaurus	# of Headwords	# of Synonymous Relationships
WordNet	4.34×10^4	1.32×10^5
Webster's New World Roget's A-Z Thesaurus	2.41×10^4	2.49×10^5
The Synonym Finder	1.86×10^4	6.02×10^5
The Oxford Thesaurus	1.56×10^4	2.34×10^5
The Oxford Dictionary of Synonyms and Antonyms	0.81×10^4	0.87×10^5
21st Century Synonym and Antonym Finder	0.70×10^4	1.29×10^5
A Dictionary of Synonyms and Antonyms	0.37×10^4	0.54×10^5
Merriam Webster Thesaurus	0.37×10^4	0.91×10^5
Scholastic Dictionary of Synonyms, Antonyms, and Homonyms	0.21×10^4	0.19×10^5
Full Dataset	5.33×10^4	1.04×10^6

Summary statistics for nine thesauri used to construct the general-English near-synonym terminology.
