# Supplementary material for: Quantifying the Impact and Extent of Undocumented Biomedical Synonymy
Source: PLoS Comput Biol. 2014 Sep 25;10(9):e1003799. doi: 10.1371/journal.pcbi.1003799 (PMC4177665; doi:10.1371/journal.pcbi.1003799)
Supplement: Table S5 — Estimates for the extent of undocumented synonymy for the three terminologies included in this study. This table provides the lower bound on the log-evidence for the best fitting annotation mixture models specific to each lexical domain. Moreover, it provides the fraction of headwords/concepts and synonym pairs/terms predicted to be undocumented within each dataset. Values in parenthesis indicate the 99% credible intervals for the estimates. (PDF) [file pcbi.1003799.s013.pdf]

Table S 5. Estimates for the Extent of Undocumented Synonymy for the Three Terminologies Included in this Study

Domain	(H, S)	Log Evid.	Frac. Heads/Concepts Missing	Frac. Synonyms Missing
Diseases and Syndromes	$(10, 4)$	-3.8×10^5	60.61% (60.28, 60.93)	92.26% (92.17, 92.33)
Pharmacological Substances	$(10, 4)$	-4.2×10^5	95.02% (94.98, 95.05)	99.03% (99.02, 99.04)
General-English	$(10, 4)$	-2.6×10^6	29.73% (29.30, 30.15)	93.20% (93.16, 93.23)

This table provides the lower bound on the log-evidence for the best fitting annotation mixtiure models specific to each lexical domain. Moreover, it provides the fraction of headwords/concepts and synonym pairs/terms predicted to be undocumented within each dataset. Values in parenthesis indicate the 99% credible intervals for the estimates.
