# Supplementary material for: Quantifying the Impact and Extent of Undocumented Biomedical Synonymy
Source: PLoS Comput Biol. 2014 Sep 25;10(9):e1003799. doi: 10.1371/journal.pcbi.1003799 (PMC4177665; doi:10.1371/journal.pcbi.1003799)
Supplement: Text S1 — Supplemental materials and methods. (PDF) [file pcbi.1003799.s014.pdf]

Quantifying the Impact and Extent of Undocumented Biomedical Synonymy

Supporting Information

David R. Blair[1,2], Kanix Wang[1,2], Svetlozar Nestorov[3], James A. Evans[3,4], Andrey Rzhetsky[1,2,3,5]

[1]Institute for Genomics and Systems Biology, [2]Committee on Genetics, Genomics, and Systems Biology, [3]Computation Institute, [4]Department of Sociology, [5]Departments of Medicine and Human Genetics, University of Chicago, Chicago, IL, 60637

1 SI Materials and Methods

1.1 Re-write and Suppression Rules for the Biomedical Terminologies

Consistent with previous observations [1, 2, 3], we noticed that many of the terms contained within the UMLS Metathesaurus were inappropriate for natural language-oriented analyses (ex: database-specific encodings, machine permutations, non-English language entries, etc.). Therefore, prior to generating the terminologies utilized in this study, we subjected the Metathesaurus to a thorough, rule-based filtering, which was an extension of the method outlined in [3]. Consistent with the previous study [3], our set of implemented rules can be broken into two categories: re-write and suppression rules. Below, we list each of them explicitly, providing examples when necessary.

Re-write Rule 1: *Syntactic inversion.* Given that a term contained a comma followed by a space, we split the phrase on the comma and placed the latter fragment at the beginning of the term (ex: *carcinoma, kidney* → *kidney carcinoma*).

Re-write Rule 2: *Removal of Possessives.* All apostrophes were removed from possessive nouns (ex: *Addison's diseases* → *Addisons disease*).

Re-write Rule 3: *Removal of Angular Brackets.* All tokens bracketed by “<” and “>” were stripped from the terms.

Re-write Rule 4: *Removal of Starting/Ending Parenthesis/Brackets.* If a term began or ended with a token/tokens in parentheses/brackets, the tokens surrounded by the punctuation were stripped from the term (ex: *nausea (symptom)* → *nausea*).

Re-write Rule 5: *Removal of “NOS.”* The token “NOS,” a non-specific designator, was removed from all terms.

Re-write Rule 6: *Removal of Punctuation.* All internal punctuation was removed from the terms, replaced by either whitespace or the null character, depending on context.

Re-write Rule 7: *Term Collapse.* If two (or more) terms were simple token permutations of one another (after masking stop tokens[1] and stemming [4, 5]), they were collapsed into a single term (ex: *disease of the mouth* $\equiv$ *mouth disease*).

Suppression Rule 1: *Removal of Non-English Terms.* All biomedical terms annotated with a language other than English were removed from the dataset.

Suppression Rule 2: *Removal of Terms Containing "@."* All terms containing the "@" symbol were removed from the dataset.

Suppression Rule 3: *Removal of Single Character Terms.* If a term contained a single character after masking stop tokens, it was removed from the dataset.

Suppression Rule 4: *Removal of Classification Terms.* Terms containing the tokens "NEC," "not elsewhere classified, unclassified, or "without mention" were removed from the dataset, following [3].

Suppression Rule 5: *Removal of EC Numbers.* Terms consisting of Enzyme Classification (EC) numbers were removed from the dataset.

Suppression Rule 6: *Removal of Dosage Terms.* All terms corresponding to a dosage specification were removed from the dataset. Identification of dosage terms is described in [3].

Suppression Rule 7: *Disallowed Term Types.* We removed all terms annotated with the following types: FN, PM, CA2, CA3, CCN, CCS, CSY, UCUMAB, UCUMPT, UCUMSY and AD.

These rules were applied using the CASPER software [3] and in-house python scripts.

1.2 Description of Normalization Algorithms Used in this Study

Below, we briefly describe the normalization algorithms used in this study. Except for MetaMap, all algorithms were written in Python and relied on the Whoosh search and indexing library[2].

Boolean Search: This algorithm normalized mentions by performing a simple AND-query, returning any concept annotated with a term that contained all of tokens constituting the mention of interest. Tokens were stemmed and stop words were

[1] `http://www.nlm.nih.gov/bsd/disted/pubmedtutorial/020_170.html`
[2] `https://pypi.python.org/pypi/Whoosh/`

masked. If multiple matching concepts were returned, the one(s) with the highest TF-IDF score was (were) returned.

MetaMap: This algorithm was used according to guidelines provided here[3]. Note, our analyses required the repeated construction of the databases used by MetaMap's normalization algorithm. The construction of these databases was automated using the DataFileBuilder scripts[4].

Cosine Similarity: This algorithm normalized mentions by computing the cosine similarities between a mention and all of the terms contained within the terminology, where

$$\text{Cos. Sim.} = \frac{\vec{M} \cdot \vec{T}}{\|\vec{M}\| \times \|\vec{T}\|},$$

and $\vec{M}/\vec{T}$ denote the TF-IDF vectors for the mention and term respectively. The concept(s) with the highest cosine similarity was (were) returned.

pairwise Learning-to-Rank (pLTR): This algorithm extends simple cosine similarity by adding a "token synonymy" matrix $\mathbf{W}$ to the procedure, which is learned during training through stochastic gradient descent [6]. This matrix allows tokens that are not exact matches to contribute to the similarity score, both in a positive and negative fashion. More specifically, the similarity score used to find the best concept using this method is:

$$\text{Sim. Score} = \frac{\vec{M}'\mathbf{W}\vec{T}}{\|\vec{M}\| \times \|\vec{T}\|},$$

where $\vec{M}'$ denotes the transpose of the TF-IDF vector for the mention. Our implementation of the pLTR algorithm closely followed the description in [6]. Consistent with previous results [6], it was the top performer in our analyses.

1.3 Estimating the Fraction of Redundant Concept-to-Term Relationships

It was relatively straightforward to measure the overall effects of synonymy on the named-entity normalization tasks considered in this study. We simply compared the performance of the algorithms listed in previous section before and after removing all of the synonyms from some terminology of interest (see Table 1 and Figure S1). Although this analysis provided an estimate for the total contribution of synonymy to the normalization tasks, it did not guarantee that every relationship annotated within the terminology was useful. Obviously, it was not possible to determine the utility of synonyms that were not mentioned

[3] http://metamap.nlm.nih.gov/Docs/Metamap13_Usage.shtml
[4] http://metamap.nlm.nih.gov/DataFileBuilder.shtml

in the corpora, as it is always possible that a larger, more thorough sample of natural language would in fact contain such mentions. However, we were able to ask a different but still important question: "What fraction of the synonyms used by the algorithms in the current analysis are redundant with one another?" This analysis is important because it gives us an indication of the *efficiency* of current synonym terminologies, and it also has ramifications for the utility of the undocumented synonyms inferred to exist in the latter sections of this study.

To outline our approach for estimating redundancy, consider the scenario in which the synonyms used during some normalization task were non-redundant. In other words, there was a one-to-one mapping between the concepts returned for some subset of mentions and the synonyms used by the algorithm for normalization. Let $\mathcal{C}$ denote the set of returned concepts for the subset of mentions whose normalization required synonymy, where $|\mathcal{C}| = N$ denotes the total number of such mentions. Note, these concepts can be readily identified for any normalization algorithm and corpus following the procedure outlined in the previous paragraph. Now, given the non-redundancy assumption, the successful return of each concept $C_i \in \mathcal{C}$ required only a single annotated synonym. Assuming that we randomly removed (without replacement) some fraction of the synonyms in our terminology (denoted $1-\rho$), the marginal probability that any individual concept-to-synonym mapping remained after sub-sampling is simply ρ. Therefore, the probability that the ith concept (denoted C_i) remained in $\mathcal{C}$ (denoted $C_i \in \mathcal{C}_\rho$) is:

$$P(C_i \in \mathcal{C}_\rho) = \rho.$$

By the linearity of expectation, the expected total number of concepts remaining in $\mathcal{C}$ after removing some fraction of synonyms $1 - \rho$ assuming non-redundancy is:

$$\begin{aligned}\mathbb{E}\big[|\mathcal{C}_\rho|\big] =& \sum_{i=1}^{N} P(C_i \in \mathcal{C}_\rho) \\ =& N\rho. \end{aligned} \tag{1}$$

In practice, we generally did not observe a perfectly linear decrease in concept recall as more and more synonyms were removed from a terminology, suggesting some amount of redundancy (see Figure S1 for examples). To illustrate, let K_i indicate the number of redundant synonyms used by some algorithm during the normalization of ith mention, such that a total of $K_i + 1$ synonyms could in theory be used by the algorithm to return concept C_i. Upon randomly removing some fraction of concept-to-synonym annotations, the marginal probability that at least one of required synonyms remained in the terminology enabling successful normalization is:

$$P(C_i \in \mathcal{C}_\rho | K_i) = 1 - (1-\rho) \times \prod_{i=1}^{K_i} \frac{(1-\rho)S}{S-i},$$

where S is the total number of synonymous relationships in the thesaurus. Given that S is very large (generally on the order of tens of thousands of synonyms for the terminologies considered in this study), the previously probability is well approximated by:

$$P(C_i \in \mathcal{C}_\rho | K_i) \approx 1 - (1-\rho)^{K_i+1}.$$

Now, assume that the numbers of redundant synonyms per mention were generated from some discrete probability distribution (denoted $P(K_i|\vec{\theta})$ for the ith mention) with support $[0, \infty)$. The probability that $C_i \in \mathcal{C}_\rho$ after marginalizing over all possible K_i is:

$$P(C_i \in \mathcal{C}_\rho|\vec{\theta}) \approx 1 - (1-\rho) \times \sum_{K_i=0}^{\infty} (1-\rho)^{K_i} \times P(K_i).$$

In the present study, we assumed that each K_i was sampled i.i.d from a Geometric distribution, but we also repeated our analyses using Poisson and Negative Binomial models and obtained similar results (although the Negative Binomial model had a tendency towards numerical instability). In the case of the geometric model, where $P(K_i|\gamma) = \gamma(1-\gamma)^{K_i}$, the infinite series in the previous equation can be evaluated analytically to yield:

$$\begin{aligned} P(C_i \in \mathcal{C}_\rho|\gamma) \approx & 1 - (1-\rho) \times \sum_{K_i=0}^{\infty} (1-\rho)^{K_i} \times P(K_i) \\ \approx & 1 - \gamma(1-\rho) \times \sum_{K_i=0}^{\infty} \big[(1-\rho)(1-\gamma)\big]^{K_i} \\ \approx & 1 - \frac{\gamma(1-\rho)}{1-(1-\rho)(1-\gamma)}. \end{aligned}$$

Therefore, the expected total number of concepts remaining in $\mathcal{C}$ after removing some fraction of synonyms $1-\rho$ given the previously described redundancy model is:

$$\begin{aligned} \mathbb{E}\big[|\mathcal{C}_\rho|\big] \approx & \sum_{i=1}^{N} P(C_i \in \mathcal{C}_\rho|\gamma) \\ = & N - \frac{N\gamma(1-\rho)}{1-(1-\rho)(1-\gamma)}, \end{aligned} \tag{2}$$

which is a convex (sub-linear) function of the sub-sampled fraction of synonyms.

Given that the parameter γ in 2 is known, we can directly estimate: 1) the number mentions that were normalized using redundant synonyms, and 2) the total number of redundant synonymous relationships paired to the concepts in $\mathcal{C}$. The former is obtained simply by computing the probability that a particular mention-to-concept mapping has zero redundant synonyms, and under the geometric model, this is simply:

$$P(K_i = 0|\gamma) = \gamma.$$

The latter is estimated by noting that the sum of N geometric random variables is a negative binomial distribution, and therefore, the total number of redundant synonyms paired to the concepts in $\mathcal{C}$ (denoted $\mathbb{K} = \sum_{i=1}^{N} K_i$) is:

$$P(\mathbb{K}|\gamma) = \binom{\mathbb{K} + N - 1}{\mathbb{K}} (1-\gamma)^{\mathbb{K}} \gamma^{N}.$$

In Table 1 (Column 5), we report this value after normalizing it by the total number synonyms paired to the concepts in $\mathcal{C}$, demonstrating that a considerable majority of the annotated relationships do not correspond to the redundant synonyms used by the algorithms in the present analyses.

The estimates outlined above assume that γ is known, which is not true in practice. To estimate this parameter from the recall curves in Figure S1, we assumed that the concept recall probabilities after sampling (denoted $P(C_i \in \mathcal{C}_\rho|\gamma)$) were independent of one another. Given the geometric model outlined above, this yields the following simple likelihood for the recall data returned for a particular $1-\rho$ sub-sampling experiment:

$$P(\mathcal{C}_\rho|\gamma) \propto \prod_{j=1}^{J} \left(1 - \frac{\gamma(1-\rho_j)}{1-(1-\rho_j)(1-\gamma)}\right)^{|\mathcal{C}_{\rho_j}|} \left(\frac{\gamma(1-\rho_j)}{1-(1-\rho_j)(1-\gamma)}\right)^{N-|\mathcal{C}_{\rho_j}|}, \qquad (3)$$

where j indexes a total of J independent sub-sampling experiments and N can be directly determined by removing all of the synonyms in the terminology of interest (as described previously). In practice, we estimated γ by maximizing 3 with respect to this parameter. Note, the independence assumption invoked during the specification of this likelihood is violated at many levels. For instance, we sampled synonym pairs without replacement, which should theoretically generate a small amount of negative covariance among the returned concepts. Nevertheless, we found that on simulated data with properties similar to our actual terminologies and corpora, the effect was negligible (the R^2-value between 1000 uniformly sampled and inferred γ values was 0.99 while the slope and intercept for the line-of-best-fit between these quantities was approximately 1.0 and 0.0 respectively). Perhaps more importantly, none of the algorithms considered in this study normalize concepts in an independent fashion. To some extent, they are all ranking algorithms, indicating that the concept returned for any particular mention depends on the other concepts and their annotated synonyms. That said, such correlations should only effect the recall data variance, not its expectation (due to the linearity of the latter), so we believe that our estimator should be relatively consistent in practice.

Finally, we would like to note that the pairwise Learning-to-Rank (pLTR) method generally yielded the lowest amount of redundant synonymy while MetaMap typically had the highest. This difference likely reflects the distinct approaches underlying these two algorithms. MetaMap performs automatic variant generation during the construction of its database, so it is able to generate a synonym that was previously removed during sub-sampling, creating redundancy. Alternatively, the pLTR algorithm actually learns

a matrix of weights during training that allows non-matching tokens to contribute to the similarity score for two phrases. Thus, this "token synonymy" matrix likely benefits from the inclusion of even redundant synonyms, as they potentially allow the algorithm to learn other examples of synonymy that are not currently annotated. That said, we restricted our analysis to unique mentions, and we found that it was easy to over-train the pLTR algorithm (especially on the NCBI corpus), likely due to the limited amount of information available in the training sets. Therefore, we feel that our estimates of redundant synonymy for this algorithm may be inflated.

1.4 A Corpus-Based Estimate of Semantic Similarity for General-English Words

To computationally assess the quality of the harvested headword-synonym pairings, we wanted to measure their overall semantic similarities using a large corpus of natural language and compare these measurements to those obtained for both known and random pairings. Many methods are available for estimating the semantic similarity shared between two words using large text corpora [7, 8]. In the present study, we adapted the method outlined in [9] due to its simplicity and scalability. Briefly, let $\mathcal{C}^h$ denote the set of contexts surrounding some headword w_h within a large text corpus (in our case Wikipedia), and let $\mathcal{C}^s$ denote the same for some synonym w_s. For simplicity, we defined context as the two flanking tokens occurring before and after each headword/synonym occurrence, ignoring order (the "bag-of-words" assumption). Our estimate of the semantic similarity compared the information content of the contexts shared by two words with the total information content of their contexts as follows. First, let $\mathcal{I}(C_i)$ denote the information content of the ith context, where:

$$\mathcal{I}(C_i) = -\log P(C_i|\text{Corpus}).$$

The semantic similarity shared between a headword and its synonym was defined as [9]:

$$\text{Sem. Sim.}(w_h, w_s) = \frac{2 \times \sum_{C_i \in \mathcal{C}^h \cap \mathcal{C}^s} \mathcal{I}(C_i)}{\sum_{C_i \in \mathcal{C}^h} \mathcal{I}(C_i) + \sum_{C_j \in \mathcal{C}^s} \mathcal{I}(C_j)}. \tag{4}$$

For the sake of brevity, we do not present the formal justification for this measurement of similarity and instead direct the interested reader to [9]. In practice, we found that different classes of headwords tended to have very different background semantic similarities. For example, nouns of high frequency tended to pervasively share semantic similarity with other words simply because of their low information content, making comparisons across different headwords difficult. Therefore, we constructed a null, background distribution of semantic similarity for each headword by computing 4 between it and every other word not currently paired with it in our true positive, true negative, and novel synonym pair

datasets. We then used the mean (μ_i) and variance (σ_i^2) from this background distribution to standardize the semantic similarities for the word pairs of interest:

$$\text{Sem. Sim. Score}(w_h, w_s) = \frac{\text{Sem. Sim.}(w_h, w_s) - \mu_i}{\sqrt{\sigma_i^2}}. \tag{5}$$

The output of the previous equation was reported in the main text and in Figure 3E and 3F.

1.5 A Probabilistic Model for Estimating the Extent of Undocumented Synonymy

In the main text, we outlined a statistical model for inferring the number of concepts (headwords) and terms (synonyms) that are missing from some set of thesauri. In this section, we further develop our approach by extending the model to multiple, independent terminologies, and subsequently, we increase its descriptive potential by allowing the annotation rates to vary across concepts and terms that were included within the same dictionary. We also briefly outline our Bayesian approach to inferring the extent of undocumented synonymy given the described models and the observed data, and we demonstrate how our prior distribution over the number of terms per concept (synonyms per headword) can used to estimate the total number of such relationships in the language given any possible number of concepts (headwords).

1.5.1 Extending the Model to Multiple, Independent Terminologies

Consistent with the notation used in the main text, consider a set of N' concepts that were harvested from a collection of T independent terminologies. Furthermore, let $\vec{S'} = \langle S'_1, S'_2, \ldots S'_{N'-1}, S'_{N'} \rangle$ denote the total number of annotated terms specific to these concepts. At this point, it is important to note that not all of the $\vec{S'}$ relationships were annotated by each of the T terminologies. To be included into $\vec{S'}$, each relationship only had to be annotated by at most one terminology. To encode the annotation status of each concept-to-term relationship across the T thesauri, we used the following nested list (a list of lists). Let $\mathcal{C}$ denote the complete list of concepts and terms obtained by combining multiple terminologies, such that $\mathcal{C} = \{\mathcal{R}_1, \mathcal{R}_2, \ldots, \mathcal{R}_{N'-1}, \mathcal{R}_{N'}\}$. Each $\mathcal{R}_i \in \mathcal{C}$ represents the list of terms paired to the ith concept, and to encode the annotation statuses of these relationships, $\mathcal{R}_i$ contains a total of S'_i vectors of length T, such that $\mathcal{R}_i = \{\vec{\mathbf{a}}_{i,1}, \vec{\mathbf{a}}_{i,2}, \ldots, \vec{\mathbf{a}}_{i,S'-1}, \vec{\mathbf{a}}_{i,S'}\}$. Finally, each element in $\vec{\mathbf{a}}_{i,j}$, denoted $a_{i,j,k}$, indicates whether the jth term of the ith concept was annotated within the kth terminology:

$$a_{i,j,k} = \begin{cases} 1 \text{ if } (\text{concept}_i, \text{term}_j) \in \text{terminology}_k \\ 0 \text{ otherwise.} \end{cases}$$

To specify the likelihood for the combined dataset, let $1-p^k_{\vec{\theta}}(0)$ denote probability that a concept-to-term relationship was sampled at least once by the lexicographer assigned to the kth terminology and was therefore included into the dictionary. To ease notational burden, we set $1 - p^k_{\vec{\theta}}(0) \equiv p_k$. With this simplification in place, the probability of observing $\vec{S'}$ terms annotated to a total of N' concepts, given the latent variables $\vec{S}$, ϕ, and N and the model parameters Θ, is:

$$P(\vec{S'}, N'|\vec{S}, \phi, N, \Theta) = \binom{N}{N-N'} \times \left[\binom{\phi - 1}{\phi + N' - N} \left(\prod_{k=1}^{T} [1 - p_k] \right)^{\phi} \right] \times \dots$$
$$\dots \left[\prod_{i=1}^{N'} \binom{S_i}{S_i - S'_i} \left(\prod_{j=1}^{S'_i} \prod_{k=1}^{T} p_k^{a_{i,j,k}} [1 - p_k]^{1-a_{i,j,k}} \right) \left(\prod_{k=1}^{T} [1 - p_k] \right)^{S_i - S'_i} \right], \quad (6)$$

where $\Theta = \{p_k : \forall k = 1 \dots T\}$.

1.5.2 Allowing Annotation Rates to Vary Across Concepts and Terms

As discussed in the main text, the coverage of different terminologies with respect to the same linguistic domain can vary wildly due to a multitude of factors, including their preference to annotate certain relationships at the expense of others (see Figure S3 for examples) and their potentially differing definitions of synonymy. To account for this variability, we applied an approach that has recently shown promise within the fields of ecology [10] and metagenomics [11]. Specifically, we assumed that concepts and their terms belonged to different classes, and we allowed each to be annotated at a distinct rate by the same terminology. This somewhat agnostic, mixture-modeling approach to accounting for annotation variability is an obvious oversimplification, but in practice, we found that our models well described the variation that we observed within our datasets (see main Figure 2F for example) and were capable of capturing specific examples of annotation bias (see main Figure 2G and Figure S4).

Briefly, our mixture model assumes that each concept belongs to one of H components, which in turn harbor their own set of L_h term classes. The subscript h associated with the previous variable indicates that those term classes only belong to concepts assigned to the hth component. Thus, our specific mixture model divides the space of possible synonymous relationships into $H \times L_h$ components, each with their own unique annotation rate. Let z_i denote the class assigned to the ith concept, where $z_i = h$ and $h \in \{1 \dots H\}$. Similarly, let $y_{i,j}$ denote the class assignment of the jth concept-to-term relationship annotated to the ith concept, where $y_{i,j} = l_h$ and $l_h \in \{1 \dots L_h\}$. We assume that concept and term classes were instantiated according to simple categorical models, such that:

$$P(z_i = h|\vec{\pi}) = \pi_h, \text{ where } \vec{\pi} = \langle \pi_1, \pi_2, \dots, \pi_{H-1}, \pi_H \rangle$$
$$P(y_{i,j} = l_h|z_i = h, \vec{\lambda}_h) = \lambda_{h,l_h}, \text{ where } \vec{\lambda}_h = \langle \lambda_{h,1}, \lambda_{h,2}, \dots, \lambda_{L_h-1}, \lambda_{L_h} \rangle.$$

Therefore, the joint probability for the class assignments of N' concepts and $\vec{S}'$ terms, denoted using $\mathbf{z}$ and $\vec{\mathbf{y}}$ respectively, is:

$$\begin{aligned} P(\mathbf{z}, \vec{\mathbf{y}}|\vec{\pi}, \Lambda) &= \prod_{i=1}^{N'} P(z_i = h|\vec{\pi}) \prod_{j=1}^{S'_i} P(y_{i,j} = l_h|z_i = h, \vec{\lambda}_h) \\ &\equiv \prod_{i=1}^{N} \pi_h \prod_{j=1}^{S'_i} \lambda_{h,l_h}, \end{aligned}$$

where $\Lambda = \{\vec{\lambda}_h : \forall h = 1 \dots H\}$.

To incorporate these classes into the model, let $p_{h,l_h,k}$ denote the probability of annotation for a concept-to-term relationship whose concept belongs to class h and whose term belongs class l_h. Furthermore, let $\vec{\xi}_i$ denote the number of undocumented terms that are paired to the ith concept (that is instantiated with class h) and belong to each of the L_h different synonym classes:

$$\vec{\xi}_i = \langle \xi_{i,1}, \xi_{i,2}, \dots, \xi_{i,L_h-1}, \xi_{i,L_h} \rangle,$$

where $\sum_{l_h=1}^{L_h} \xi_{i,l_h} = S - S_i$ and $\Xi = \{\vec{\xi}_i : \forall h = 1 \dots N'\}$. Similarly, let $\vec{\eta}$ denote the number of undocumented concepts that belong to each of the H classes:

$$\vec{\eta} = \langle \eta_1, \eta_2, \dots, \eta_{H-1}, \eta_H \rangle, \text{ where } \sum_{h=1}^{H} \eta_h = N - N'.$$

Each class of undocumented concepts in turn has its own set of unannotated terms, and we let $\vec{\chi}$ denote the total number of undocumented concept-to-term relationships that belong to each concept class:

$$\vec{\chi} = \langle \chi_1, \chi_2, \dots, \chi_{H-1}, \chi_H \rangle, \text{ where } \sum_{h=1}^{H} \chi_h = \phi.$$

Finally, we introduce a set of H vectors, denoted Ω, where each $\vec{\omega}_h \in \Omega$ contains the number of relationships that belong to each of the L_h synonym classes:

$$\vec{\omega}_h = \langle \omega_{h,1}, \omega_{h,2}, \dots, \omega_{h,L_h-1}, \omega_{h,L_h} \rangle, \text{ where } \sum_{l_h=1}^{L_h} \omega_{h,l_h} = \chi_h.$$

With this notation in place, the full likelihood for the observed concept-to-term relationships $\vec{S}'$, the observed number of concepts N', the various class instantiations for the

observed concepts and terms ($\mathbf{z}$ and $\vec{\mathbf{y}}$ respectively), and the four sets of latent variables described above is:

$$P(\vec{S}', N', \mathbf{z}, \vec{\mathbf{y}}, \Xi, \vec{\eta}, \vec{\chi}, \mathbf{\Omega} | N, \vec{S}, \phi, \Theta) = \left[\binom{N}{N - N'} \binom{N - N'}{\eta_1, \ldots, \eta_H} \times \ldots \right.$$

$$\ldots \left. \prod_{h=1}^{H} \pi^{\eta_h} \binom{\chi_h - 1}{\chi_h - \eta_h} \binom{\chi_h}{\omega_{h,1}, \ldots, \omega_{h,L_h}} \prod_{l_h=1}^{L_h} \left(\lambda_{h,l_h} \prod_{k=1}^{T} [1 - p_{h,l_h,k}] \right)^{\omega_{h,g_h}} \right] \times \ldots$$

$$\ldots \left[\prod_{i=1}^{S'} \pi_h \binom{S_i}{S_i - S_i'} \left(\prod_{j=1}^{S_i'} \lambda_{h,l_h} \prod_{k=1}^{T} [p_{h,l_h,k}]^{a_{i,j,k}} [1 - p_{h,l_h,k}]^{1 - a_{i,j,k}} \right) \times \right.$$

$$\left. \ldots \left(\binom{S_i - S_i'}{\xi_{i,1}, \ldots, \xi_{i,L_h}} \prod_{i=1}^{S} \left[\lambda_{h,l_h} \prod_{k=1}^{T} [1 - p_{h,l_h,k}] \right]^{\xi_{i,l_h}} \right) \right], \quad (7)$$

where $\Theta = \{\vec{p}, \vec{\pi}, \Lambda\}$ and $\vec{p} = \{p_{h,l_h,k} : \forall h = 1 \ldots H, l_h = 1 \ldots L_h, k = 1 \ldots T\}$.

1.5.3 Model Inference and Undocumented Synonymy Estimation

The joint likelihoods defined in [6] and [7] can be used to estimate the latent variables of interest (specifically $\vec{S}$, ϕ, and N) using a variety of techniques, and in current study, we took a Bayesian approach and sought the following posterior distribution:

$$P(\vec{S}, \phi, N | \vec{S}', N', \Theta, \Sigma) =$$
$$\frac{P(\vec{S}', N' | \vec{S}, \phi, N, \Theta) \times P(N, \vec{S}, \phi | \Sigma)}{\sum_{N=N'}^{\infty} \sum_{\phi=N-N'}^{\infty} \sum_{S_i=S_i',\ \forall i=1\ldots N'}^{\infty} P(\vec{S}', N' | \vec{S}, \phi, N, \Theta) \times P(N, \vec{S}, \phi | \Sigma)}.$$

where Σ denotes the set of parameters defining our prior over the variables $\vec{S}$, ϕ, and N. Although this posterior is analytically tractable (see below for details), it is important to note that the model parameters Θ are unknown, rendering it irrelevant in practice. However, by placing a prior distribution over the unknown parameters, we generate a hierarchical model for $\vec{S}'$ and N', and by integrating Θ out of this model, we can obtain a posterior distribution that does not explicitly depend on the unknown parameters:

$$P(\vec{S}, \phi, N | \vec{S}', N', \Sigma, \Psi) = \int_{\Theta} P(\vec{S}', N' | \vec{S}, \phi, N, \Theta) P(\Theta | \Psi) d\Theta,$$

where Ψ denotes the set of parameters that define the prior for Θ. Unfortunately, the previous integral is analytically intractable, necessitating an approximate inference strategy. In the present work, we invoked a mean-field variational approximation [12], which recasts the intractable integral in terms of optimizing a simple functional over joint posterior space [13].

Specifically, let $P(\vec{S'}, N'|\Sigma, \Psi)$ denote the likelihood for the observed concepts and terms, marginalized over the model parameters Θ and the latent variables $\vec{S}$, ϕ, and N:

$$P(\vec{S'},N'|\Sigma,\Psi) = \int_{\Theta} \sum_{N=N'}^{\infty} \sum_{\phi=N-N'}^{\infty} \sum_{S_i=S'_i,\ \forall i=1\ldots N'}^{\infty} P(\vec{S'}, N'|\vec{S}, \phi, N, \Theta) \times P(N, \vec{S}, \phi|\Sigma) P(\Theta|\Psi) d\Theta \quad (8)$$

Now, consider some approximate joint posterior over the model parameters and the latent variables, denoted $q(\vec{S}, \phi, N, \Theta)$. Given this approximate distribution, the previous integral can be manipulated to provide a lower bound on the model log-marginal likelihood as follows:

$$\begin{aligned}\ln P(\vec{S'}, N'|\Sigma, \Psi) &= \ln \int_{\Theta} \sum_{\mathcal{V}} q(\vec{S}, \phi, N, \Theta) \frac{P(\vec{S'}, N', \vec{S}, \phi, N, \Theta|\Sigma, \Psi)}{q(\vec{S}, \phi, N, \Theta)} d\Theta \\ &\geq \int_{\Theta} \int_{\gamma} \sum_{\mathcal{V}} q(\vec{S}, \phi, N, \Theta) \ln \frac{P(\vec{S'}, N', \vec{S}, \phi, N, \Theta|\Sigma, \Psi)}{q(\vec{S}, \phi, N, \Theta)} d\Theta, \end{aligned} \quad (9)$$

where $\mathcal{V} = \{N, \vec{S}, \phi\}$ and $\sum_{\mathcal{V}}$ abbreviates the three series specified in 8. Of course, the previous lower bound becomes exact when $q(\vec{S}, \phi, N, \Theta) \equiv P(\vec{S'}, N', \vec{S}, \phi, N, \Theta|\Sigma, \Psi)$, but we already know that $P(\vec{S'}, N', \vec{S}, \phi, N, \Theta|\Sigma, \Psi)$ cannot be expressed in closed form, necessitating the specification of an alternative, approximate posterior. In our application, we computed the lower bound on the model evidence subject to the constraint that $q(\vec{S}, \phi, N, \Theta)$ factorizes over the latent variables and the model parameters:

$$q(\vec{S}, \phi, N, \Theta) = q(\vec{S}, \phi, N) q(\Theta), \quad (10)$$

also known as the mean-field approximation. Plugging 10 into 9, taking functional derivatives with respect to each term in 10, and solving for maxima (subject to the constraint that each function integrates to 1) yields a set of interdependent equations for the probability distributions defined in 10. By cycling through the equations and updating each in turn, one obtains a coordinate-ascent algorithm for computing the functional that optimizes the lower bound in 9. Furthermore, upon convergence, standard theory indicates that $q(\vec{S}, \phi, N, \Theta)$ represents a locally optimal approximation of $P(\vec{S'}, N', \vec{S}, \phi, N, \Theta|\Sigma, \Psi)$ in that $q(\vec{S}, \phi, N, \Theta)$ minimizes the Kullback-Liebler Divergence from the analytical posterior subject to the mean-field constraint [13].

By placing independent, conjugate priors over each element of Θ, the joint approximate posterior $q(\Theta)$ is guaranteed to have a closed-form solution [12]. Therefore, in order for the lower bound defined in 9 to be computationally tractable, $q(\vec{S}, \phi, N)$ must have a closed form solution as well, which is true when the conditional posterior $P(\vec{S}, \phi, N|\vec{S'}, N', \Theta, \Sigma)$ can be specified analytically. To demonstrate that this is true, we must first specify our

prior for the latent variables $\vec{S}$, ϕ, and N. We assumed that the true number of terms paired to each concept scales geometrically, and we invoked an improper, uniform prior over the total number of concepts. According to these assumptions, the prior model, after collecting like terms and simplifying, is:

$$P(N, \vec{S}, \phi|\Sigma) = \gamma^{N}(1-\gamma)^{\phi-N+N'}\prod_{i=1}^{N'}(1-\gamma)^{S_i-1}, \tag{11}$$

where $\Sigma = \{\gamma\}$ and γ denotes the geometric scaling parameter. In practice, we inferred γ from the data by including it within the lower bound on the marginal likelihood defined in 9 and adding an additional term to the approximate posterior specified in 10.

With this prior in place, the conditional posterior distribution described at the start of this section, denoted $P(\vec{S}, \phi, N|\vec{S'}, N', \Theta, \Sigma)$, can be specified in closed form with respect to both the straightforward annotation model defined in the main text and for the mixture-model defined in the previous section. For the sake of simplicity, we perform all subsequent derivations with respect to the simple annotation model (which is equivalent to a mixture-model with only a single concept and term class), and we note that the derivation for the more general model follows a similar procedure, but with the class specific latent variables $\{\mathbf{z}, \vec{\mathbf{y}}, \Xi, \vec{\eta}, \vec{\chi}, \mathbf{\Omega}\}$ included within the joint posterior (thus becoming part of the inference problem). First, we note that the desired conditional posterior can be computed according to:

$$P(\vec{S}, \phi, N|\vec{S'}, N', \Theta, \Sigma) = \frac{P(\vec{S'}, N'|\vec{S}, \phi, N, \Theta) \times P(N, \vec{S}, \phi|\Sigma)}{\sum_{N=N'}^{\infty}\sum_{\phi=N-N'}^{\infty}\sum_{S_i=S'_i,\ \forall i=1\ldots N'}^{\infty} P(\vec{S'}, N'|\vec{S}, \phi, N, \Theta) \times P(N, \vec{S}, \phi|\Sigma)}.$$

The steps required for the computation of the normalization constant in the denominator of previous equation are somewhat tedious, so for the sake of brevity, we simply note that each summation corresponds to a negative binomial series, and by performing these summations, collecting like terms, and simplifying, we end up with the following, closed form expression for the desired conditional posterior:

$$\begin{aligned} P(\vec{S},\phi, N|\vec{S'}, N', \Theta) = &\binom{N}{N-N'}\left(1 - \frac{\gamma\prod_{k=1}^{T}[1-p_k]}{1-(1-\gamma)\prod_{k=1}^{T}[1-p_k]}\right)^{N'+1} \times \ldots \\ &\ldots\left[\left(\gamma\prod_{k=1}^{T}[1-p_k]\right)^{N-N'}\binom{\phi-1}{\phi+N'-N}\left((1-\gamma)\prod_{k=1}^{T}[1-p_k]\right)^{\phi+N'-N}\right] \times \ldots \\ &\ldots\left[\prod_{i=1}^{N'}\binom{S_i}{S_i-S'_i}\left(1-(1-\gamma)\prod_{k=1}^{T}[1-p_k]\right)^{S'_i+1}\left((1-\gamma)\prod_{k=1}^{T}[1-p_k]\right)^{S_i-S'_i}\right]. \end{aligned} \tag{12}$$

All estimates reported reported in the main text were obtained by taking the posterior expectation of the approximate density $q(\vec{S}, \phi, N)$. Table S5 provides these estimates

for each of the three datasets considered in this study along with their 99% confidence intervals, although such intervals are likely an underestimate of the true variability [11]. Note, for the biomedical terminologies, the estimates of missing synonymy provided in the main text and figures were adjusted to account for the fact that one term paired to each concept was considered the *preferred term* while the remainder were assumed to be synonyms.

Practically speaking, computing the approximate posterior defined in 10 through alternating coordinate ascent was relatively straightforward, barring a few minor issues. First, upon extending the model to multiple headword and synonym classes, the functional approximation surface became highly multimodal, and some local modes appeared very inferior when compared to others. To overcome this issue, we developed a series of algorithm initialization strategies, which relied on "seeding" the approximation algorithm using parameter values from models of lower complexity [14, 15] and performing simulated annealing on the normalization constant of $q(\vec{S}, \phi, N)$ to move the algorithm to parameter regimes with greater support [16]. We found this approach to be very effective on simulated data.

Second, although our mixture model allowed us to account for the annotation variability that was observed within our datasets, it was not immediately obvious how many headword and synonym components to include into the model. In practice, we started with the simplest model ($H = 1,\ L_h = 1$) and added components in a stepwise manner, keeping the number of synonym classes constant across the various headword components (i.e. $L_h = L_g\ \forall h, g \in \{1, \dots, H\}$). We used the lower bound on the log-marginal likelihood to select among models with differing dimensionality, and the we found that the most complex model that we tried ($H = 10,\ L_h = 4$) performed the best with respect to each dataset. Therefore, all of the results reported in the main text and in Table S5 are for a model with 10 concept (headword) components, each with 4 term (synonym) classes. The stopping criteria at ($H = 10,\ L_h = 4$) was dictated by computational limitations, as larger models quickly became unwieldy in terms of convergence times.

1.5.4 A More Liberal Estimate for the Extent of Undocumented Synonymy

In the previous section, we developed a prior model for the number of undocumented terms (or synonyms, depending on domain), denoted $P(N, \vec{S}, \phi|\Sigma)$, whose mathematical form was chosen mostly for the sake of convenience. However, one can also view this prior distribution as "generative," and after inferring its parameters from the data, this viewpoint can be invoked to provide additional insight into the inherent scale of synonymy given an arbitrary number of concepts. Assume that some fixed set of N concepts accrued terms according to the following simple scheme. Initially, each concept was paired with only a single term, and subsequently, terms were added stochastically to each concept at some constant rate $-\ln(\gamma)/\tau$ for an undetermined amount of time τ. This scheme produces the same prior distribution as defined in [11] [17], and therefore, the parame-

ter γ sets an intrinsic, geometric scale for the number of terms per concept. Assuming an arbitrary number of concepts N and a geometric scaling parameter γ, then the expected number of concept-to-term relationships in the language (denoted W) is given by a negative binomial distribution:

$$P(W|N,\gamma) = \binom{N+W-1}{W}\gamma^N(1-\gamma)^{N-W}. \tag{13}$$

In main Figure 2E, we used the posterior expectation the scaling parameter obtained from the best fitting annotation model to estimate W for a wide range of N.

References

[1] Aronson AR (2001) Effective mapping of biomedical text to the UMLS metathesaurus: the MetaMap program. Proceedings / AMIA Annual Symposium AMIA Symposium : 17–21.

[2] Xu R, Musen MA, Shah NH (2010) A comprehensive analysis of five million UMLS metathesaurus terms using eighteen million MEDLINE citations. AMIA Annual Symposium proceedings / AMIA Symposium AMIA Symposium 2010: 907–911.

[3] Hettne KM, van Mulligen EM, Schuemie MJ, Schijvenaars BJ, Kors JA (2010) Rewriting and suppressing UMLS terms for improved biomedical term identification. Journal of biomedical semantics 1: 5.

[4] Porter MF (1997) Readings in information retrieval. San Francisco, CA, USA: Morgan Kaufmann Publishers Inc. p. 313316. URL http://dl.acm.org/citation.cfm?id=275537.275705.

[5] Snowball: A language for stemming algorithms. URL http://snowball.tartarus.org/texts/introduction.html.

[6] Leaman R, Doan RI, Lu Z (2013) DNorm: disease name normalization with pairwise learning to rank. Bioinformatics 29: 2909–2917.

[7] Ferret O (2010) Testing semantic similarity measures for extracting synonyms from a corpus. In: Chair) NCC, Choukri K, Maegaard B, Mariani J, Odijk J, et al., editors, Proceedings of the Seventh International Conference on Language Resources and Evaluation (LREC'10). Valletta, Malta: European Language Resources Association (ELRA).

[8] Mihalcea R, Corley C, Strapparava C (2006) Corpus-based and knowledge-based measures of text semantic similarity. In: Proceedings of the 21st national conference on Artificial intelligence - Volume 1. Boston, Massachusetts: AAAI Press, AAAI'06, p. 775780. URL http://dl.acm.org/citation.cfm?id=1597538.1597662.

[9] Lin D (1998) An information-theoretic definition of similarity. In: In Proceedings of the 15th International Conference on Machine Learning. Morgan Kaufmann, p. 296304.

[10] Mao CX, Colwell RK (2005) Estimation of species richness: Mixture models, the role of rare species, and inferential challenges. Ecology 86: 1143–1153.

[11] Li-Thiao-T S, Jean-Jacques D, Stphane R (2012) Bayesian model averaging for estimating the number of classes: applications to the total number of species in metagenomics. Journal of Applied Statistics 39: 1489–1504.

[12] Wainwright MJ, Jordan MI (2008) Graphical models, exponential families, and variational inference. Found Trends Mach Learn 1: 1305.

[13] Attias H (2000) A variational bayesian framework for graphical models. In: In Advances in Neural Information Processing Systems 12. MIT Press, p. 209215.

[14] Thiesson B, Meek C, Chickering D, Chickering DM, Heckerman D (1997) Learning mixtures of DAG models. In: In Proc. of the Conf. on Uncertainty in AI. Morgan Kaufmann, Inc, p. 504513.

[15] Meila M, Heckerman D (1998) An experimental comparison of several clustering and initialization methods. In: Machine Learning. p. 386395.

[16] Kirkpatrick S, Gelatt C, Vecchi M (1983) Optimization by simulated annealing. Science, Number 4598, 13 May 1983 220, 4598: 671–680.

[17] Kendall DG (1949) Stochastic processes and population growth. Journal of the Royal Statistical Society Series B (Methodological) 11: 230–282.
